# Oxidative Stress Response Biomarkers of Ovarian Cancer Based on Single-Cell and Bulk RNA Sequencing

**DOI:** 10.1155/2023/1261039

**Published:** 2023-01-27

**Authors:** Mingjun Zheng, Yuexin Hu, Ouxuan Liu, Siting Li, Yuxuan Wang, Xinru Li, Juanjuan Liu, Qing Yang, Xiao Li, Bei Lin

**Affiliations:** ^1^Department of Gynecology and Obstetrics, Shengjing Hospital of China Medical University, China; ^2^Key Laboratory of Maternal-Fetal Medicine of Liaoning Province, China; ^3^Key Laboratory of Obstetrics and Gynecology of Higher Education of Liaoning Province, China; ^4^Department of Obstetrics and Gynecology, University Hospital, LMU Munich, Marchioninistraße 15, 81377 Munich, Germany

## Abstract

**Background:**

The occurrence and development of ovarian cancer (OV) are significantly influenced by increased levels of oxidative stress (OS) byproducts and the lack of an antioxidant stress repair system. Hence, it is necessary to explore the markers related to OS in OV, which can aid in predicting the prognosis and immunotherapeutic response in patients with OV.

**Methods:**

The single-cell RNA-sequencing (scRNA-seq) dataset GSE146026 was retrieved from the Gene Expression Omnibus (GEO) database, and Bulk RNA-seq data were obtained from TCGA and GTEx databases. The Seurat R package and SingleR package were used to analyze scRNA-seq and to identify OS response-related clusters based on ROS markers. The “limma” R package was used to identify the differentially expressed genes (DEGs) between normal and ovarian samples. The risk model was constructed using the least absolute shrinkage and selection operator (LASSO) regression analysis. The immune cell infiltration, genomic mutation, and drug sensitivity of the model were analyzed using the CIBERSORT algorithm, the “maftools,” and the “pRRophetic” R packages, respectively.

**Results:**

Based on scRNA-seq data, we identified 12 clusters; OS response-related genes had the strongest specificity for cluster 12. A total of 151 genes were identified from 2928 DEGs to be significantly correlated with OS response. Finally, nine prognostic genes were used to construct the risk score (RS) model. The risk score model was an independent prognostic factor for OV. The gene mutation frequency and tumor immune microenvironment in the high- and low-risk score groups were significantly different. The value of the risk score model in predicting immunotherapeutic outcomes was confirmed.

**Conclusions:**

OS response-related RS model could predict the prognosis and immune responses in patients with OV and provide new strategies for cancer treatment.

## 1. Introduction

Ovarian cancer (OV) is one of the most common cancers of the female reproductive system and accounts for the highest number of deaths among all gynecological cancers [1]. In 2020, 313959 incidences of OV and 207252 OV-related deaths were reported [2, 3]. Approximately 70% of patients with OV are diagnosed at the advanced stage due to the asymptomatic nature of the disease [4]. The 5-year survival rate of patients with OV in China was only 38.9% [5], which is a serious threat to women's health. Currently, the treatment options available for patients with OV mainly include surgery combined with chemotherapy. However, patients with OV develop chemoresistance, which is the primary cause of cancer recurrences and death. In recent years, with the increase in the use of immunotherapy for the treatment of OV, more attention has been paid to the role of the tumor microenvironment (TME) in the occurrence and development of OV.

TME is composed of stromal cells, extracellular matrix components, and exosomes and regulates the invasion and metastasis of tumor cells by establishing an autocrine-paracrine signaling pathway to transmit signals [6]. Tumor cells recruit active immune cells, which secrete molecules to create an immunosuppressive TME during the early stages of cancer development, which interferes with the occurrence and development of tumors. However, the relevant immune effector cells can enter a state of depletion or even remodeling after continuous tumor antigen stimulation and immune activation, which are unable to perform tumor-killing function and may even aid transform into a malignant phenotype leading to the formation of an immunosuppressive microenvironment [7]. Reactive oxygen species (ROS) play a vital role in creating an immunosuppressive microenvironment in solid tumors. Accumulating ROS creates oxidative stress (OS) in TME, which promotes the formation of an immunosuppressive microenvironment [8]. OS refers to the production of highly active molecules like ROS and nitrogen free radicals, by cells in response to various stress stimuli. The imbalance of free radicals and antioxidants in the body eventually damages the cells and tissues [9]. Epithelial ovarian cancers manifest a prooxidant state, which is characterized by an increase in levels of key prooxidant enzymes and a decrease in levels of antioxidant enzymes. Together, these factors play an essential role in the occurrence and development of OV [10, 11]. OS regulates various signaling pathways, like the NRF2-ARE, PI3K/AKT/mTOR, and WNT/*β*-catenin signaling pathways, and the expression of transcription factors like p53, NF-*κ*B, and HIF-1*α*. Furthermore, OS affects various immune cells like tumor-associated macrophages, neutrophils, myeloid-derived suppressor cells, and regulatory T cells in TME. Overall, these factors play a vital role in the occurrence and development of ovarian cancer [12, 13]. Therefore, the construction of a prognostic risk model based on OS-related genes for predicting the prognosis of patients with OV and the efficacy of immunotherapy would aid in the treatment of patients with OV.

In this study, we analyzed single-cell and bulk RNA-sequencing data of OV based on OS response-related genes to identify OS response-related DEGs in OV. These genes were used to construct a risk score (RS) model to predict the prognosis of patients with OV. Our results demonstrated that the RS model was stable and could efficiently predict the prognosis of patients.

## 2. Materials and Methods

### 2.1. Data Download and Preprocessing

The expression matrix and metadata-information file of the single-cell RNA-sequencing dataset GSE146026 were retrieved from the Gene Expression Omnibus (GEO; https://www.ncbi.nlm.nih.gov/geo/) database. The expression profile and survival information of patients from the GSE17260 and GSE26712 datasets were also downloaded from GEO. The data were processed as follows: (1) the samples without clinical follow-up data were removed; (2) the samples with unknown survival time, <0 days, no survival status, and the unified unit of survival time was days were removed; (3) the probe IDs were converted to gene symbol; (4) the probe corresponding to multiple genes was removed; (5) the median expression value was taken for expression values with multiple gene symbols.

The fragments per kilobase of transcripts per million mapped reads (FPKM) values of gene expression of patients with OV were retrieved from The Cancer Genome Atlas (TCGA) database using the “TCGAbiolinks” R package. The log2 (FPKM + 1) conversion was performed. The patient survival and mutation data were obtained from the publication by Liu et al. [14]. The survival information and mutation data were further analyzed after correction.

The gene sets related to OS responses were obtained from 17 ROS-related pathways published previously [15] (Table S1). A total of 467 OS-related genes were extracted and used for subsequent analysis (Table S2).

### 2.2. Analysis of Single-Cell Sequencing Data

The “Seurat” R package [16] was used to analyze the single-cell sequencing data. The analysis mainly included the following steps: constructing objects, data standardization, data dimensionality reduction clustering, and identifying marker genes. The CreateSeuratObject was used to build a Seurat object. The minimum number of cells was set to 3, the minimum number of features was 200, and the genes and cells were filtered for the first time. The following threshold was set to filter cells again: the number of features was (200, 6000), the number of counts was (300, 40000), the overexpression double_cutoff value was 0.15, the proportion of mitochondrial genes < 30%, and the percentage of red blood cell reads < 20%. The first 2000 hypervariable genes were selected as inputs for principal component analysis (PCA). The first 15 principal components (PCs) were identified as important principal components by the ElbowPlot function for subsequent analysis. Single-cell sequencing data were obtained from various datasets; hence the “harmony” R package [17] was used for batch correction to remove the batch effect interfering with downstream analysis. After debugging and referencing the clustering results in the original contribution, the FindClusters algorithm was used to identify tumor cell subsets with a resolution of 0.5. Uniform Manifold Approximation and Projection (UMAP) is a dimension reduction popular learning technology based on the theoretical framework of Riemannian geometric generation data. UMAP retains more global structure and has superior performance and better scalability when dealing with high-throughput and high-dimensional data like single-cell sequencing. The dimension of the data obtained after PCA was further reduced using UMAP. The cell types were divided into low-dimensional spaces and visualized using the DimPlot function. The FeaturePlot, DotPlot, DoHeatmap, and VlnPlot functions were used to visualize the expression, distribution of landscape, and characteristic genes of tumor cells. The DotPlot function was used to visualize marker genes related to specific OS responses in each cell subgroup. The top gene of |avg_log2FC| > 3 in the intersection gene of the cluster marker gene and OS response factor was selected for mapping display.

### 2.3. Annotations of Cell Subgroups

The “SingleR” package [18] was used to annotate the clustering results obtained using the Seurat R package. The Monaco immune data was used as a reference database for identifying the cell type. The principle of this algorithm was as follows: Spearman correlation coefficient of variable genes in a single cell and all samples in the reference dataset was calculated through multiple iterations. 80% quantile of the correlation coefficient of multiple reference samples under the same cell type was used as the score of the single-cell annotation. The reference cells with the maximum difference in score with the annotation of the reference cell type within 0.05 were retained until only two cell types were left. The known cell type with the highest correlation score was retained as the cell type annotated.

### 2.4. Identification of Active Subgroups

The intersection of marker genes was selected based on strong population specificity (adj_*p* < 0.05 & |avg_log2FoldChange| > 1.5 & pct.1 > 0.5 & pct.2 < 0.5) from each cell subgroup and factors related to OS responses. The “AUCell” R package was used to calculate the activity score of each cell based on the intersection of genes. The AUCell_exploreThresholds function was used to determine the threshold to identify the active cells from the current gene set. UMAP embedding of cell clusters was colored based on the area under the ROC curve (AUC) score of each cell to identify which subset of cells was active in which subgroup-specific to OS response-related genes. The FindAllMarkers function was used to identify the DEGs between the active and the inactive subgroups based on the default parameters. The functions were identified based on the marker genes with strong specificity (avg_log2FC > 1).

### 2.5. Enrichment Analysis

Gene set enrichment analysis (GSEA) uses predefined gene sets to sort genes based on their differential expression in two samples. Next, the analysis evaluates if a predefined gene set is enriched at the top or bottom of the sorting table. The predefined gene sets are usually obtained from functional annotation or the results of previous experiments. GSEA was performed using the “ClusterProfiler” [19] and “hallmark” pathway gene set R package to identify the characteristic genes enriched by active cell subgroups. The gene sets with *p* < 0.01 were considered significantly enriched. The Gene Ontology (GO) and Kyoto Encyclopedia of Genes and Genomes (KEGG) pathway enrichment analyses were further conducted.

### 2.6. Identification of DEGs

“Linear models for microarray data” (limma) R package was used to identify DEG between tumor and normal samples using bulk RNA-sequencing data [20]. Benjamini-Hochberg (FDR) was used to correct the *p* values. The adj. *p* value < 0.05 and |FC| > 1.5 were used as the threshold value to identify DEGs.

### 2.7. Construction of Prognostic Model and Survival Analysis

The data on DEGs obtained from tumor vs. normal samples and marker genes identified from the factors and pathways related to active OS response were intersected. The avg_log2FC > 0.585 was used as a threshold value to identify intersection genes. Univariate Cox regression analysis was used to analyze intersection genes associated with the prognosis of patients with OV. *p* < 0.05 was considered statistically significant. The least absolute shrinkage and selection operator (LASSO) regression analysis was used to identify the key genes associated with prognosis and to construct the prognostic model. LASSO regression analysis was performed using the “glmnet” R package [21]. The tumor samples were divided into high-risk and low-risk groups based on the calculated median RS as the threshold value. The Kaplan Meier (KM) survival curve was used to predict the survival of patients with OV, and the difference in survival was determined using the log-rank test. The receiver operating characteristic (ROC) curve was used to predict the score by the disturbance scoring model using the “timeROC” R package [22]. The “Ggplot2” R package [23] was used to create the scatter plot for survival time, survival state, and sample scores of the patients. The pheat map R package was used to construct the gene expression heat map of the prognostic model. The RS of the model is the sum of the expression value of each candidate gene multiplied by the weight. The formula used to calculate the RS is as follows:

(1) $$\begin{array}{c} \text{RS}=\overset{n}{\underset{i=0}{\sum }}\beta_{i}*\chi_{i}. \end{array}$$

*β*_*i*_ is the weight coefficient of each gene; *χ*_*i*_ is (log2FPKM + 1) of each gene.

To determine if a single model gene could predict the survival of OV patients, the patients with OV were divided into high- and low-expression groups based on the median expression of genes. The KM survival curve was used to predict the survival of patients with OV, and the difference in survival was determined by the log-rank test. The genes with *p* < 0.05 were considered prognostic genes that could significantly predict the prognosis of patients with OV.

### 2.8. Estimating the Proportion of Infiltrating Immune Cells and Immune Score

Cell-type Identification by Estimating Relative Subsets of RNA Transcript (CIBERSORT) and “ESTIMATE” algorithm “IBOR” R package were used to calculate the proportion of infiltrating immune cells using the TCGA-OV dataset. The CIBERSORT algorithm [24] was used to characterize the composition of cells based on the gene expression profile of complex tissues. The leukocyte characteristic gene matrix (LM22) comprises 547 genes that can differentiate between 22 immune cell types, including myeloid cell subtypes, natural killer cells, plasma cells, immature and memory B cells, and seven types of T cells. CIBERSORT combined with the LM22 characteristic matrix was used to estimate the proportion of 22 immune cell types in the samples. The sum of the proportions of all immune cell types in each sample was equal to 1.

### 2.9. Gene Mutation Analysis

The “maftools” R package was used for creating a waterfall diagram to show the distribution of genes with high somatic mutation frequency in patients with OV. The patients were categorized into high- and low-risk groups based on the differences in mutation frequency. The copy number variation (CNV) data were obtained from TCGA, and the patients classified into high- and low-risk groups based on CNV were analyzed using the “gistic2” module of the GenePattern website. The ChromPlot function in the “maftools” R package was used to visualize the output results. Simultaneously, the tumor mutation burden (TMB) of each sample was calculated to study the relationship between the RS and TMB. Further, it was used to evaluate the difference in survival of the patients classified in the high-TMB and low-TMB groups.

### 2.10. Drug Sensitivity

The sensitivity (half-maximal inhibitory concentration (IC_50_ value)) of 138 drugs from the Genomics of Drug Sensitivity in Cancer (GDSC) database was predicted using the “pRRophetic” R package [25]. The correlation between the IC_50_ value and model gene expression was calculated using Spearman's rank correlation. The IC_50_ value distribution box diagram of high-risk and low-risk groups was drawn to explore the correlation between the model and chemotherapy drugs.

### 2.11. Statistical Analyses

The Wilcoxon signed-rank test was used to compare the differences between the two groups of samples, and the Kruskal-Wallis test was used to compare the differences between more than two groups of samples. NS indicates *p* > 0.05, ^∗^ indicates *p* ≤ 0.05, ^∗∗^ indicates *p* ≤ 0.01, ^∗∗∗^ indicates *p* ≤ 0.001, and ^∗∗∗∗^ indicates *p* ≤ 0.0001.

## 3. Results

### 3.1. Identification of Cell Subgroups with Active OS Response

#### 3.1.1. Tumor Single-Cell Landscape

**Figure 1:**
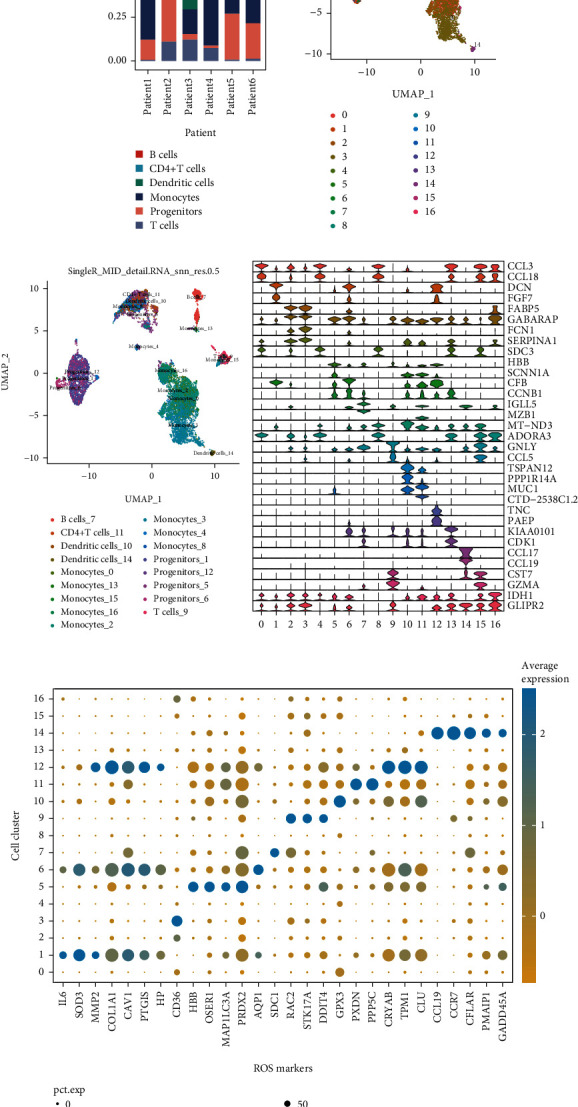
Identification of cell subgroups and expression of marker genes from single-cell RNA-sequencing database. (a) Cumulative histogram shows the distribution of cell types in patients with OV. (b) UMAP map shows the distribution of ovarian cancer (OV) cell subgroups. (c) UMAP map shows annotation results of OV cell subgroups. (d) Violin diagram shows the expression of genes specific to cell subgroups. (e) The bubble diagram shows the expression of genes related to oxidative stress (OS) responses in each cell subgroup. The darker the color blue, the higher the average expression, and the size of the dot represents the number of expressed cells.

The single-cell sequencing dataset GSE146026 retrieved from the GEO database consists of tumor tissues from six patients with OV. After integrating and filtering the original data, the remaining cells and genes were used for subgroup identification and annotation (Table S3). The distribution of cell subgroups and differences in the TME of patients with OV is shown in Figure 1(a). Based on the clustering results of the single-cell sequencing dataset and the annotation information obtained using SingleR, UMAP dimensionality reduction was used to display the expression pattern of single cells. The results revealed that the cells could be divided into 17 subgroups from 0 to 16 and were annotated as six major cell types (Figures 1(b) and 1(c)). A violin plot was drawn based on the top two genes of each cell subgroup (Table S4). The violin diagram shows the expression and distribution of specific markers of each cell subgroup (Figure 1(d)), thereby indicating that the tumor cells were heterogeneous. A total of 28 genes were selected based on the intersection of marker genes with strong specificity in each cell subgroup and genes related to OS responses (Table S5). The bubble diagram shows the expression of these genes in each cell subgroup (Figure 1(e)). The results show that genes related to OS responses had the strongest specificity for cluster 12. The cells in cluster 12 were annotated as progenitors.

#### 3.1.2. Identification of Reactive Subgroups of OS

**Figure 2:**
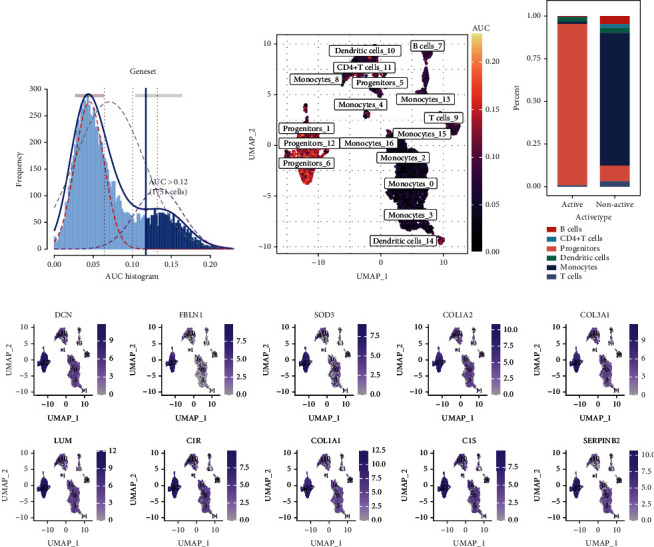
Identification of active cell subgroups. (a) AUC score of the oxidative stress marker genes, the threshold value was 0.12. (b) UMAP colorogram shows the score of cell activity. The brighter the color, the higher the activity. (c) Histogram shows the cumulative distribution of active and inactive cell population. (d) The FeaturePlot shows the active cell population.

A total of 56 genes were obtained by the intersecting specific genes related to OS responses and cell subsets. These 56 genes were defined as ROS markers (Table S6). The active cell subgroups identified based on the expression of these 56 ROS markers were used to study the expression pattern of OS response genes at the single-cell level. The optimal threshold was used to determine cell activity, and the results revealed that 1751 active cells were present in the OS response subgroups (Figure 2(a)). Figures 2(b) and 2(c) show the UMAP diagram and cumulative distribution histogram of active cells. The results revealed that progenitor cells and dendritic cells were the active cells. The expression of the top ten marker genes in the active cell subgroup is shown in Figure 2(d).

#### 3.1.3. Identifying Functions of OS Reactive Cell Subgroups

The function of the active OS cell population was determined based on the expression of marker genes with strong specificity using GSEA. The GO and KEGG pathway enrichment analysis was performed on the marker gene sets, and the top 10 significantly enriched pathways were selected to draw a bubble diagram. The results revealed that marker gene sets significantly enriched functions related to extracellular tissues and pathways associated with proteoglycans in adhesive plaque and cancer (Supplement Figure 1A–1D). GSEA was performed using a HALLMARK pathway based on the log2FC value. A total of eight pathways were significantly enriched. A close correlation was observed between interferon response factors and immune regulation in tumors (Supplement Figure 1E).

#### 3.1.4. Bulk RNA-Sequencing Analysis of OV Samples and Gene Expression Pattern

The OV data obtained from TCGA database did not contain normal samples; hence, we performed batch correction to remove the batch effect. The normal samples from Genotype-Tissue Expression and the TCGA-OV samples were integrated to obtain 352 OV samples and 88 normal samples for differential expression analysis. A total of 2928 DEGs (Table S7) were obtained. The clinical information of patients with OV is shown in Table 1. The DEGs were intersected with the marker genes of the active cell subgroup, and a total of 151 differentially active marker genes were obtained (Table S8). The expression heat map is shown in Supplement Figure 2A. GSEA was performed using the HALLMARK pathway for DEGs (Table S9), and the results showed that the DEG significantly enriched HALLMARK_EPITHELIAL_MESENCHYMAL_TRANSITION and HALLMARK_INTERFERON_GAMMA_RESPONSE (Supplement Figure 2B). The GO and KEGG pathway enrichment analysis was also performed, and the results are shown in Supplement Figure 2C–2F.

### 3.2. Construction and Verification of Prognostic Risk Model

#### 3.2.1. Identification of Prognostic Gene Signatures of OV

**Figure 3:**
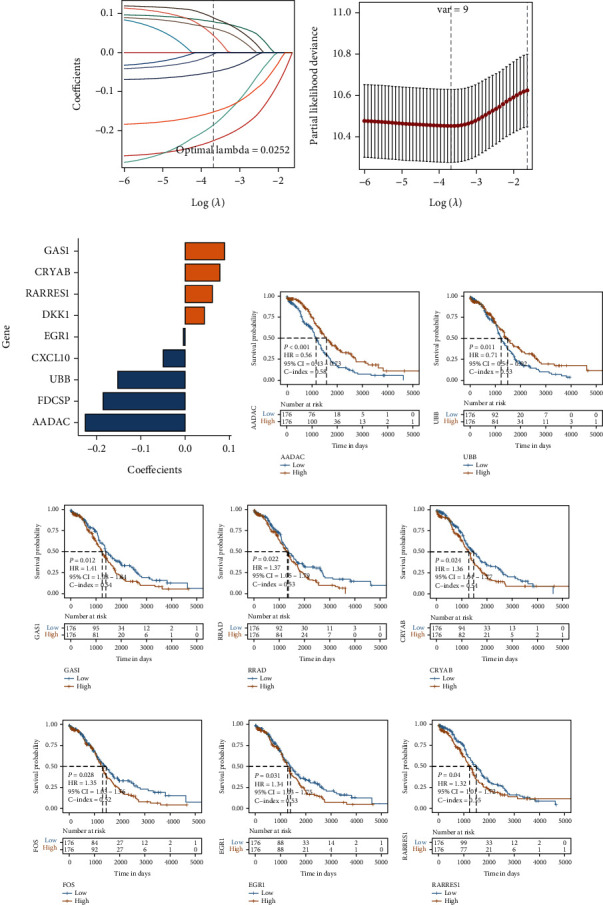
Cox and LASSO regression analysis of the TCGA-OV dataset. (a) Change trajectory of LASSO regression independent variable, the abscissa represents the logarithm of the independent variable lambda, and the ordinate represents the coefficient of the independent variable. (b) Confidence interval under each lambda in LASSO regression. (c) LASSO regression coefficient of key prognostic genes. (d–k) KM survival curve of prognostic gene signature obtained using Cox regression analysis (with significant differences), yellow represents the high-risk group, and blue represents the low-risk group.

Univariate Cox analysis was used to identify the differentially expressed marker genes in the active cell population (*p* < 0.05). A total of 12 genes related to the prognosis of OV were finally identified (Table S10). 7/10 TCGA-OV samples (*n* = 352) were selected as the training set (*n* = 246) by random sampling. LASSO regression analysis was performed on the training set to remove redundant genes, and the seed = 1110 was set. A total of nine genes related to the prognosis of patients with OV (Table S11) were identified, and the results are shown in Figures 3(a)–3(c). These nine genes were identified using Cox regression analysis, and the median expression value of each gene was used as the cutoff value to divide the patients into high- and low-risk groups. The KM survival curve was plotted for patients from TCGA-OV dataset, and the genes that could significantly predict the survival of patients were selected for representation. Of these genes, significant differences in the KM curve between the eight genes were observed. The prognosis of patients in the high-risk group was worse compared to that of patients in the low-risk group (Figures 3(d)–3(f)).

#### 3.2.2. Verification of the Model Robustness Using Internal and External Datasets

**Figure 4:**
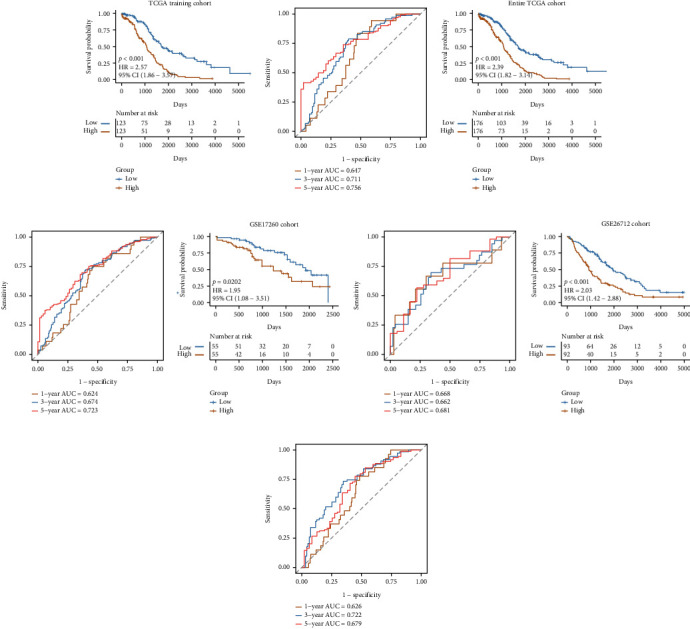
The performance of the model in different cohorts. (a, c, e, g) The survival curve of patients in high- and low-risk groups from TCGA training cohort, the entire TCGA, the GSE17260, and the GSE26712 cohorts, respectively. Yellow represents the high-risk group, and blue represents the low-risk group. (b, d, f, h) 1-, 3-, and 5-year time-dependent ROC curves of models for TCGA training, the entire TCGA, GSE17260, the GSE26712 cohorts.

To determine the robustness of the models built using the nine gene signatures, the samples were divided into high-risk and low-risk groups based on the median RS value as the threshold value. KM survival curves of patients from different groups in TCGA training cohort (Figure 4(a)), the entire TCGA cohort (Figure 4(c)), the GSE17260 cohort (Figure 4(e)), and the GSE26712 cohort (Figure 4(g)) were constructed. The results showed that the prognosis of patients in the high-risk group was significantly worse compared to patients in the low-risk groups from all cohorts. The ROC curve was used to determine the efficacy of the model in predicting the prognosis of patients (Figures 4(b)–4(h)). In TCGA training cohort, the AUC of 1-, 3-, and 5-year survival was 0.647, 0.711, and 0.756, respectively (Figure 4(b)). The ROC of the entire TCGA cohort for 1-, 3-, and 5-year survival were 0.624, 0.674, and 0.723, respectively (Figure 4(d)). In the GSE17260 cohort, the ROC of the GSE17260 cohort for 1 year was 0.668, for 3 years was 0.662, and for 5 years was 0.681 (Figure 4(f)). The AUC of the GSE26712 cohort for 1, 3, and 5 years were 0.626, 0.722, and 0.679, respectively (Figure 4(h)). Together, these results demonstrate that the performance of our prognostic model in different cohorts was good.

### 3.3. Correlation between Risk Score Model and Clinical Characteristics of OV

**Figure 5:**
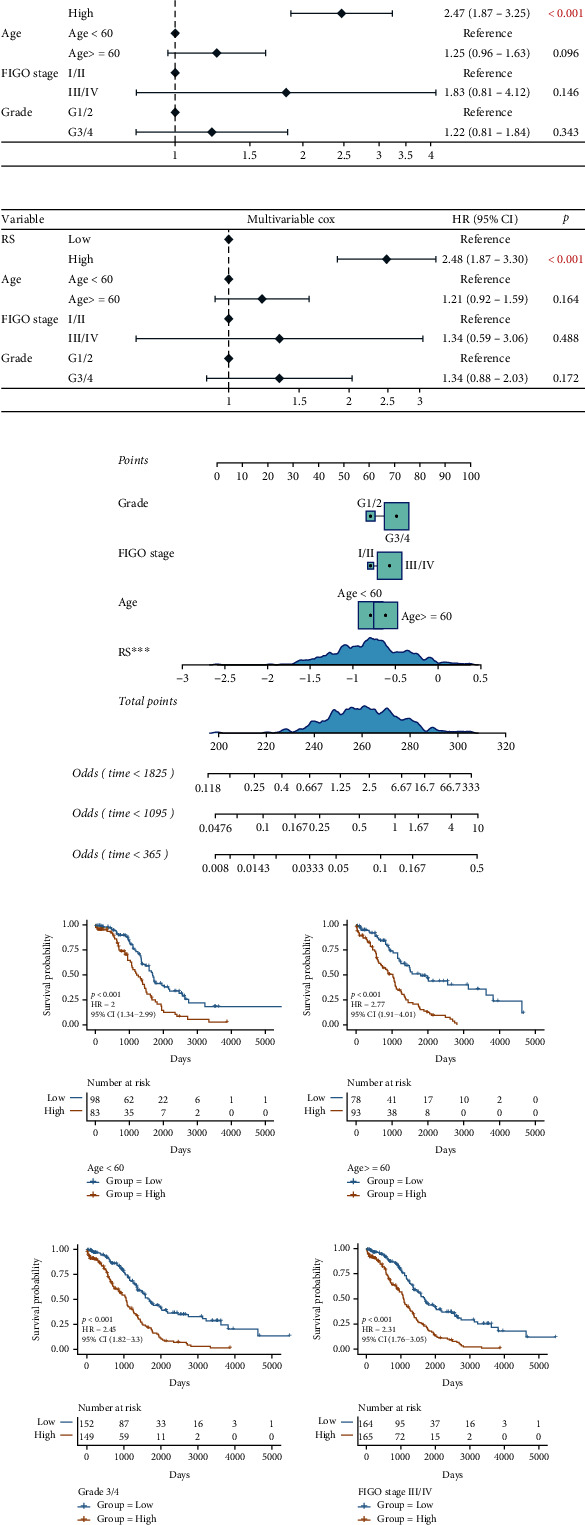
RS is an independent prognostic factor for clinical characteristics. (a, b) Forest map shows the results of Cox regression analysis performed on clinical characteristics of TCGA cohort. (c) The nomogram of the prediction model. The line segment represents the contribution of the clinical factor to the outcome events, total points represent the total score of the sum of the corresponding individual scores of the value of all variables, and the bottom three lines represent the prognosis of 1-, 3-, and 5-year survival corresponding to each value point. (d–g) The prognosis prediction was based on clinical characteristics like age < 60, age ≥ 60, grade 3/4, and FIGO stage III/IV, respectively. Yellow represents the high-risk group, and blue represents the low-risk group.

To verify whether RS can be used as an independent prognostic factor, univariate and multivariate Cox regression analysis was performed on the clinical characteristics of patients like age, FIGO stage, and grade. The results showed that RS was an independent prognostic risk factor for patients regardless of the type of Cox regression analysis used for performing statistical checks (Figures 5(a) and 5(b)). The multivariate Cox regression analysis was used to construct the nomogram, and the results indicate that RS could significantly predict clinical outcomes (Figure 5(c)). Next, we checked if the prediction model created based on the RS could predict the prognosis of patients with different characteristics. The KM survival curve showed that the prognosis of patients in the high-risk group was worse compared to that of patients in the low-risk groups with different clinical characteristics (Figures 5(d)–5(g), age < 60, *p* < 0.0001; age ≥ 60, *p* < 0.0001; stage III/IV, *p* < 0.0001; grade 3/4, *p* < 0.0001).

### 3.4. Differences in Genetic Mutations

**Figure 6:**
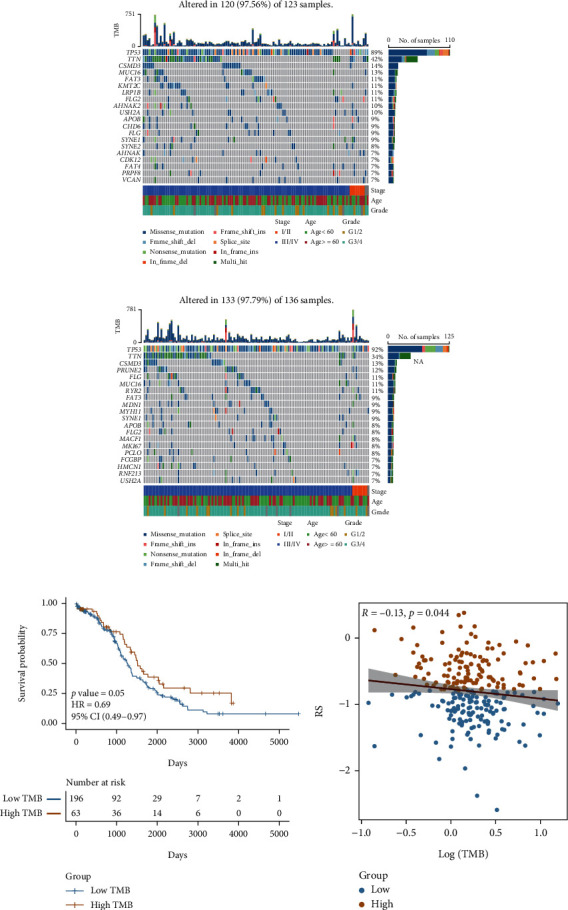
Single nucleotide variant (SNV) between model groups. (a, b) SNV waterfall of top 20 (mutation frequency) genes in the patients in the high- and low-risk groups. (c) KM survival curve in patients in high- and low-TMB groups. Yellow indicates the high-TMB group; blue indicates the low-TMB group. (d) The scatter diagram shows a correlation between RS and TMB. Red represents the high-risk group, and blue represents the low-risk group.

Genetic mutations play an important role in the occurrence and development of cancer. Therefore, enhancing our understanding of genetic mutations in OV will aid in developing targeted drugs and new tumor therapies. Therefore, the top 20 genes with the highest mutation frequency in patients from the high- and low-risk groups were analyzed, and the waterfall diagram was drawn. The results revealed differences in gene mutation frequency in patients in the high- and low-risk groups (Figures 6(a) and 6(b)). Further, TMB was calculated, and the patients were divided into high-TMB and low-TMB groups (Table S12) based on the upper quartile. The KM survival curve of the patients in the high-TMB and low-TMB groups was drawn. The results show that the prognosis of patients in the high-TMB group was good (Figure 6(c)). The scatter diagram shows a significant negative correlation between RS and TMB (Figure 6(d), *R* = −0.13, *p* < 0.05).

### 3.5. Analysis of Immune Cell Infiltration Characteristics of the Model

**Figure 7:**
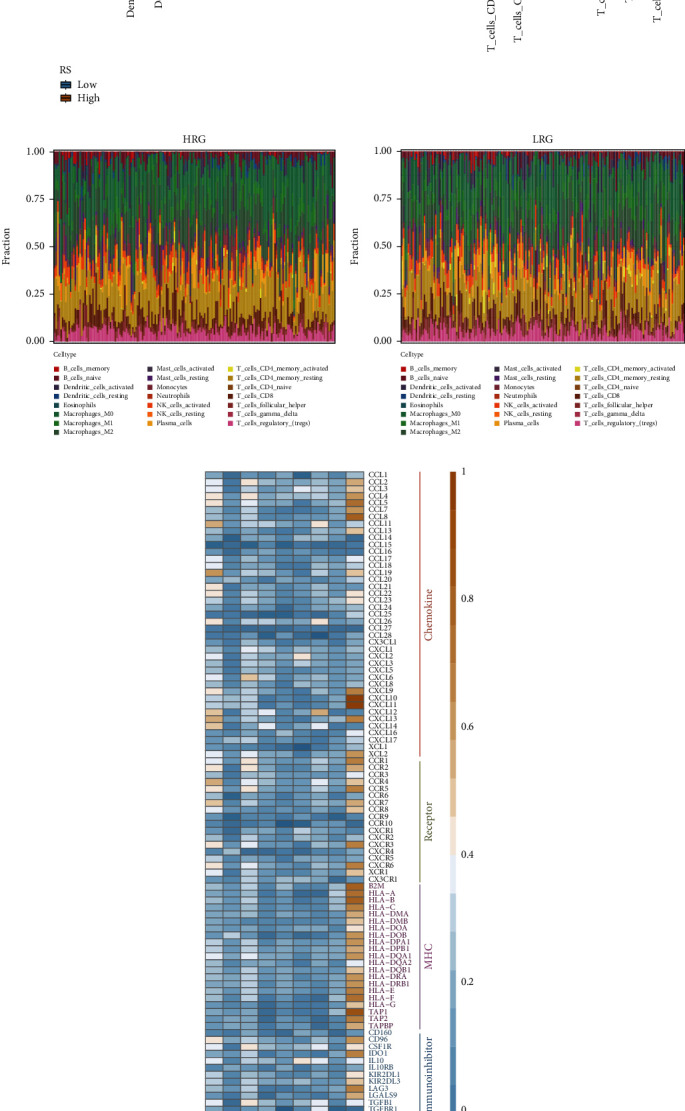
Analysis of immune cell infiltration characteristics of the model. (a) The box diagram shows the difference in the proportion of 22 infiltrating immune cells in the patients in the high- and low-risk groups. Yellow represents the high-risk group, and blue represents the low-risk group. (b, c) Cumulative histogram shows the proportion of immune cell infiltration in the high- and low-risk groups. Different colors represent different cell types. (b) The high-risk group and (c) the low-risk group. (d) The correlation heat map shows an expression of nine model genes and immune checkpoints, and the color of the dot represents the correlation.

Immune cell infiltration in the TME affects the occurrence and development of cancers. Therefore, we explored the differences in TME of the patients in the high-risk and low-risk groups using the CIBERSORT algorithm. CIBERSORT was used to estimate the proportion of 22 immune cells infiltrating in OV (Tables S13 and S14). The analysis revealed significant differences in the proportion of immune cells infiltrating the tumors in patients in the high- and low-risk groups. The results showed significant differences in the proportion of infiltrating immune cells like M1 macrophages, activated memory CD4 T cells, T follicular helper cells, and gamma delta T cells in the patients in the high-risk group/low-risk group (Figure 7(a)). The distribution of 22 immune cells in the patients in the high-risk group/low-risk group is shown in Figures 7(b) and 7(c). Immune checkpoints are molecules expressed by the immune cells, which can regulate the degree of immune activation. They play an important role in the occurrence of autoimmune diseases. Therefore, we analyzed the correlation between five categories of immunomodulators, cytokines [26], and the expression of nine model genes (Table S15). The results showed a strong correlation between *CXCL10* and immune checkpoints (Figure 7(d)).

### 3.6. Differences in Pathways Enriched in Patients from Different Risk Groups

**Figure 8:**
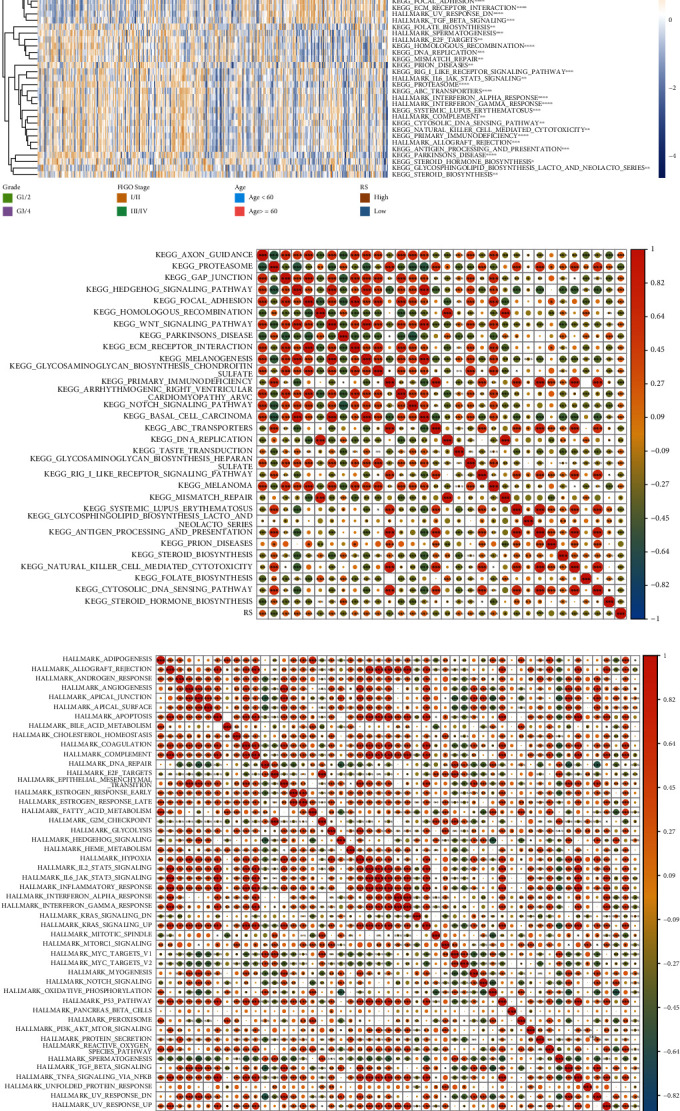
Differences in the pathway enrichment between high- and low-risk groups. (a) The heat map shows the enrichment score of differentially enriched pathways. The asterisks represent the significant differences in enrichment scores. (b) The heat map shows the correlation between the enrichment score of the differentially enriched KEGG pathway and RS. (c) The heat map shows the correlation between the enrichment score of the differentially enriched HALLMARK pathway and RS.

The HALLMARK and KEGG pathway enrichment analysis scores were calculated (Table S16) based on the gene expression pattern of patients with OV. The difference in pathways enriched in the high- and low-risk groups will aid in deciphering the underlying mechanism of carcinogenesis that may affect the prognosis of patients. The “limma” R package (adjusted *p* value < 0.01) was used to calculate the differences in scores between high- and low-risk groups based on the enrichment scores, and the heat map was drawn. The results revealed a significant difference in enrichment scores of 46 pathways between high- and low-risk groups (Figure 8(a)). Among the enriched pathways, the WNT/*β*-catenin signaling pathway plays a vital role in mediating the crosstalk between tumorigenesis and oxidative stress. The aberrant activation of the WNT/*β*-catenin signaling pathway can stimulate ROS production and chronic inflammation activation [27]. Further, the correlation between RS and enrichment score was calculated based on the pathways enriched using the HALLMARK and KEGG pathway enrichment analysis. The heat map was drawn, and the results are shown in Figures 8(b) and 8(c).

### 3.7. Predicting Drug Sensitivity and Response to Immunotherapy

**Figure 9:**
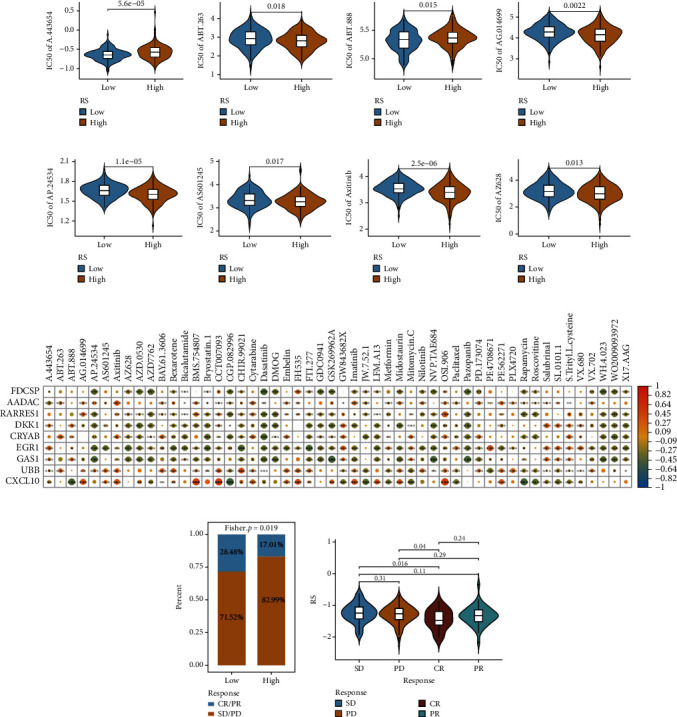
The RS model predicts the treatment outcomes of patients with OV. (a–h) The difference in the distribution of IC_50_ values of eight chemotherapeutic drugs between the high-risk and low-risk groups. Red represents the high-risk group, and blue represents the low-risk group. (i) The heat map shows the correlation between the expression of model genes and the IC_50_ values of chemotherapeutic drugs. The color of the dot represents the high and low correlation; ^∗^ represents the significance. (j) Histogram shows the cumulative distribution of immunotherapy response in patients in the high-risk and low-risk groups. (k) The violin diagram shows the distribution of RS in different immunotherapy response groups; the value is the significant difference in response between the two groups. Based on the effectiveness of immunotherapy, it is divided into complete response (CR), partial response (PR), stable disease (SD), or progressive disease (PD).

Based on the expression data of TCGA-OV samples, the IC_50_ values of 138 drugs from the GDSC database were predicted (Table S17). The violin diagram shows the differences in the distribution of IC_50_ value between high- and low-risk groups (Table S18). The results of eight chemotherapy drugs with significant differences are shown in Figures 9(a)–9(h). The correlation between the expression of the model gene and the IC_50_ value of the chemotherapeutic drug was calculated (Table S19). The results showed a strong correlation between *CXCL10* and the IC_50_ value of the chemotherapeutic drug (Figure 9(i)). In addition, to determine if the model could predict immunotherapy response, the IMvigor210 dataset was divided into high- and low-risk groups based on the median value of RS. The differences in the distribution of immune response in samples (complete response (CR) + partial response (PR)) and nonimmune response samples (stable disease (SD) + progressive disease (PD)) in high- and low-risk groups were compared using the cumulative distribution map (Figure 9(j)). The patients in low-risk groups responded to immunotherapy better compared to the patients in the high-risk group. Further, the RS of patients in the SD/PD group was significantly higher compared to that of patients in the CR group (Figure 9(k)), indicating that patients in the low-risk groups had a better response to immunotherapy.

## 4. Discussion

OV is highly heterogeneous. Owing to its heterogeneity, patients with the same type of OV can have differences in their biological characteristics, including morphology, molecular subtypes, metastasis patterns, and different responses to treatment. In fact, these differences are also observed among tumor cells of the same patient. It is difficult to predict and understand the heterogeneity of tumor cells using traditional transcriptome sequencing data (Bulk RNA-seq). With the advancement in technology, the advent of high-throughput sequencing techniques like single-cell sequencing can provide transcriptomic information at the cellular level [28]. The high resolution of single-cell sequencing allows in-depth analysis of the gene expression of a single tumor cell in a patient. This aids in identifying different subsets of cells, oncogenes, responses to treatment regimens, and possible new treatment targets [29].

In this study, we have annotated and identified different cell subgroups based on the single-cell sequencing data on patients with OV retrieved from the GEO database. The results revealed significant differences in the TME of different patients with OV. We identified a total of six cell types in OV samples using the SingleR package. Interestingly, various recent studies have demonstrated the increase in OS byproducts and the lack of an antioxidant stress system, which play a vital role in the occurrence and development of OV [12, 30, 31]. The damage caused by OS alters the balance between oxidation and antioxidation, which increase the levels of ROS and free radicals produced by the body. A high level of ROS can induce gene mutation, which promotes the activation of protooncogenes or inactivates tumor suppressor genes. The changes in gene expression patterns lead to abnormal cell proliferation and the development of tumors [32]. Therefore, in this study, we aim to explore the potential role of OS response factors and genes in predicting the prognosis of patients with OV and their response to immunotherapy. A total of 56 ROS genes were identified by intersecting the marker genes with strong specificity for each cell subgroup and OS response factor. We identified active cell subgroups and their functions based on the expression pattern of these 56 ROS genes. Studies have shown that OS enhances the metabolism of tumor cells by altering the activity of key enzymes, inducing mutations in genes associated with metabolism, and activating signal pathways. Together, this leads to malignant transformation of the cells [33]. The GO and KEGG pathway enrichment analysis showed significant enrichment of pathways associated with proteoglycan in cancer. Therefore, it is rational to speculate that OS regulates metabolic interaction between tumor cells and TME in OV.

We next used bulk RNA-sequencing data to identify the differentially expressed marker genes in patients with OV. Prognostic risk models were constructed based on the expression pattern of nine genes associated with prognosis, including *GAS1*, *CRYAB*, *RARRES1*, *DKK1*, *EGR1*, *CXCL10*, *UBB*, *FDCSP*, and *AADAC*. *CRYAB* is an antiapoptosis protein that reduces the levels of intracellular ROS and nitric oxide, as well as iron uptake by cells. Studies have shown the involvement of *CRYAB* in impairing protein oxidation, damage caused to cytoskeletal structure, and lipid peroxidation. It also prevents DNA damage caused by oxidative stress [34]. Studies have shown a correlation between *CRYAB* overexpression and cisplatin resistance in OV, which indicates that the prognosis of patients with OV overexpressing *CRYAB* is poor [35–37]. Further, a significant increase in the expression of secretory protein *DKK1* was observed in patients with malignant ascites of OV, and the prognosis of these patients is poor [38]. A study has shown that *CXCL10* can predict the prognosis of patients with advanced serous OV [39]. Increased *CXCL10* expression promotes atrial fibrosis, inflammation, and oxidative stress [40]. A study has shown that *FDCSP* promotes the invasion and migration of OV cells [41]. In colorectal cancer, *AADAC* reduces lipid peroxidation by scavenging ROS in a *SLC7A11*-dependent manner, thereby protecting metastatic CRC cells from undergoing ferroptosis [42]. Therefore, the prognosis of patients with OV overexpressing *AADAC* is good [43]. However, the role of other prognosis-related genes used to construct our prognosis model has not been reported in OV. Furthermore, growth arrest specific 1 (*GAS1*) inhibits the cell cycle progression from G0 to the S phase, which inhibits tumor progression and plays an anticancer role [44]. *GAS1* may be a negative regulator of the Hedgehog signaling pathway, in order to regulate tumor proliferation and differentiation, angiogenesis, invasion, migration, and apoptosis of cells and other functions by regulating the Hh pathway [45]. Retinoic acid receptor response 1 (*RARRES1*) is a tumor suppressor [46, 47] and regulates mitochondrial and fatty acid metabolism, stem cell differentiation, etc. Moreover, the depletion of *RARRES1* can inhibit B cell differentiation [48]. The patients were divided into high- and low-risk groups based on median RS value, and the ROC curve revealed that the performance of the model in predicting prognosis was good. The survival analysis showed that the prognosis of patients in the high-risk group was worse compared to the patients in the low-risk group. To further understand the underlying cause for the difference in prognosis in patients in the high- and low-risk groups, we analyzed genetic mutations in these patients. The results showed that the prognosis of patients in the high-TMB group was better. Further, a negative correlation was observed between RS and TMB. TMB is proportional to the number of tumor neoantigens. Studies have shown that patients with high nonsynonymous mutation burden produce more neoantigens and have better immunogenicity. This improves the patient's response to immunotherapy, thereby improving the survival rate [49, 50].

Recent studies have shown that immune checkpoints play an important role in aiding immune cells to evade immune surveillance. CTLA-4 is involved during the early stage of immune activation, specifically in regulating T cell activation [51]. PD-L1 promotes immunosuppressive TME by inhibiting T cell infiltration. We further analyzed the correlation between immune checkpoints and the expression of nine signature genes used to create the prognostic model. The results showed a strong correlation between *CXCL10* and immune checkpoints. Studies have shown that *CXCL10-secreting* macrophages mediate the antitumor immune responses in OV [52, 53]. Further, *CXCL10* promotes the immune checkpoint blockade treatment in homologous recombination-deficient tumors [54], which is consistent with our results.

However, our study has a few limitations. This is a retrospective study; hence, the accuracy of the model needs to be validated further using a larger sample size and prospective cohorts. Further, the underlying regulatory role of nine genes in the occurrence and development of OV and OS still needs further experimental validation.

## 5. Conclusions

In conclusion, we created a risk score model using single-cell and bulk RNA-sequencing data. The genes used to construct the models were based on the intersection of genes associated with OS responses and pathways and genes related to OS in OV. The nine-gene signature prognostic model can be used to predict the prognosis and immune response of patients with OV.

## Figures and Tables

**Table 1:** The clinical information of patients with OV in TCGA OV cohort.

TCGA OV cohort	Group information	Numbers of sample
Age	Age ≥ 60	171
	Age < 60	181

Stage	Stage I/II	20
	Stage III/IV	329

Grade	G1/2	41
	G3/4	301

OS	Dead	219
	Alive	133

## Data Availability

The datasets used and/or analyzed during the current study are available from the corresponding author on reasonable request.

## Supplementary Materials

Supplementary Materials Supplement Figure 1: determining the functions of reactive subgroups of OS. (A) The bubble diagram shows the KEGG pathway enrichment analysis of marker genes of the active subgroup. The size of the dot represents the number of marker genes enriched, and the color represents the significant enrichment. (B–D) The bubble diagram shows the GO enrichment analysis of marker genes of active subgroup, biological process (BP), molecular function (MF), and cellular component (CC). (E) Broken line graph shows the gene enrichment score of GSEA. Supplement Figure 2: differential expression and functional enrichment analysis of bulk RNA-sequencing tumor vs. normal samples. (A) The expression heat map shows the intersection of DEGs and marker genes of active cell subgroup, red represents high expression, and blue represents low expression. (B) Broken line graph shows the gene enrichment score of GSEA. (C–F) The bubble diagram shows the GO and KEGG pathway enrichment analysis of DEGs. The size of the point represents the number of marker genes enriched, and the color represents the significant enrichment. Table S1: gene sets related to OS response in 17 ROS pathways. Table S2: 467 OS-related genes. Table S3: subgroup identification and annotation of preprocessed and integrated cells and genes. Table S4: violin plot analysis of the top two markers from each cluster. Table S5: 28 genes obtained from the intersection of marker genes with strong cell subgroup specificity and OS response-related gene sets. Table S6: 56 intersection genes as ROS markers obtained from the intersection of OS response factors and markers specific to cell subsets. Table S7: 2928 DEGs. Table S8: intersection of DEG and marker genes of active cell populations to obtain 151 differentially expressed marker genes. Table S9: the HALLMARK pathway enrichment analysis of DEG using GSEA. Table S10: identification of differentially expressed marker genes of an active cell population using univariate Cox regression analysis to obtain the 12 prognostic genes associated with ovarian cancer. Table S11: identification of prognostic gene signatures using the LASSO Cox regression analysis. Table S12: categorizing the patients with OV into high- and low-TMB groups based on the upper quartile. Table S13: the proportion of 22 immune cells infiltrating the samples in the high-expression group estimated using the CIBERSORT algorithm. Table S14: the proportion of 22 immune cells infiltrating the samples in the low-expression group estimated using the CIBERSORT algorithm. Table S15: correlations between five classes of immunomodulators, chemokines, and the expression of nine model genes. Table S16: calculation of the HALLMARK and KEGG pathway enrichment analysis scores based on expression profiles of OV samples. Table S17: prediction of IC_50_ values of 138 drugs in the GDSC database based on TCGA-OV expression profile. Table S18: violin plot analysis shows the difference in the distribution of IC_50_ values of drugs between the high- and low-risk groups. Table S19: correlation between expression of computational model genes and IC_50_ values of chemotherapeutic drugs.
