# Supplementary material for: Oxidative Stress Response Biomarkers of Ovarian Cancer Based on Single-Cell and Bulk RNA Sequencing
Source: Oxid Med Cell Longev. 2023 Jan 27;2023:1261039. doi: 10.1155/2023/1261039 (PMC9897923; doi:10.1155/2023/1261039)
Supplement: Supplementary Materials — Supplement Figure 1: determining the functions of reactive subgroups of OS. (A) The bubble diagram shows the KEGG pathway enrichment analysis of marker genes of the active subgroup. The size of the dot represents the number of marker genes enriched, and the color represents the significant enrichment. (B–D) The bubble diagram shows the GO enrichment analysis of marker genes of active subgroup, biological process (BP), molecular function (MF), and cellular component (CC). (E) Broken line graph shows the gene enrichment score of GSEA. Supplement Figure 2: differential expression and functional enrichment analysis of bulk RNA-sequencing tumor vs. normal samples. (A) The expression heat map shows the intersection of DEGs and marker genes of active cell subgroup, red represents high expression, and blue represents low expression. (B) Broken line graph shows the gene enrichment score of GSEA. (C–F) The bubble diagram shows the GO and KEGG pathway enrichment analysis of DEGs. The size of the point represents the number of marker genes enriched, and the color represents the significant enrichment. Table S1: gene sets related to OS response in 17 ROS pathways. Table S2: 467 OS-related genes. Table S3: subgroup identification and annotation of preprocessed and integrated cells and genes. Table S4: violin plot analysis of the top two markers from each cluster. Table S5: 28 genes obtained from the intersection of marker genes with strong cell subgroup specificity and OS response-related gene sets. Table S6: 56 intersection genes as ROS markers obtained from the intersection of OS response factors and markers specific to cell subsets. Table S7: 2928 DEGs. Table S8: intersection of DEG and marker genes of active cell populations to obtain 151 differentially expressed marker genes. Table S9: the HALLMARK pathway enrichment analysis of DEG using GSEA. Table S10: identification of differentially expressed marker genes of an active cell population using univariate Cox regression anal [file 1261039.f1.zip › 1261039.f1.pdf]

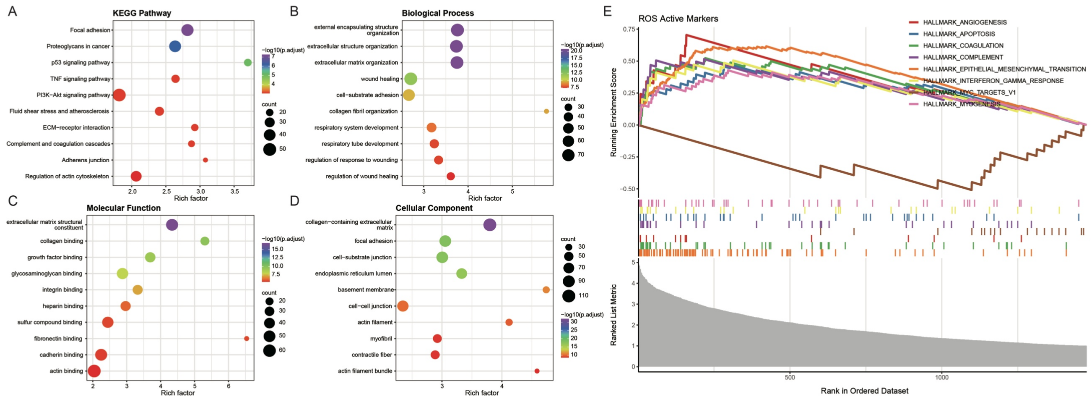

Supplement Figure 1: Functional identification of reactive subgroups of oxidative stress. A: Bubble Diagram of KEGG pathway enrichment analysis of marker gene of active subgroup. The size of the dot represents the number of marker genes enriched, and the color represents the significance of enrichment; B-D: Bubble Diagram of GO function enrichment analysis of marker gene of active subgroup, which are biological process (BP), molecular function (MF), cellular component (CC); E: Broken line graph of gene enrichment score of GSEA significant enrichment pathway.

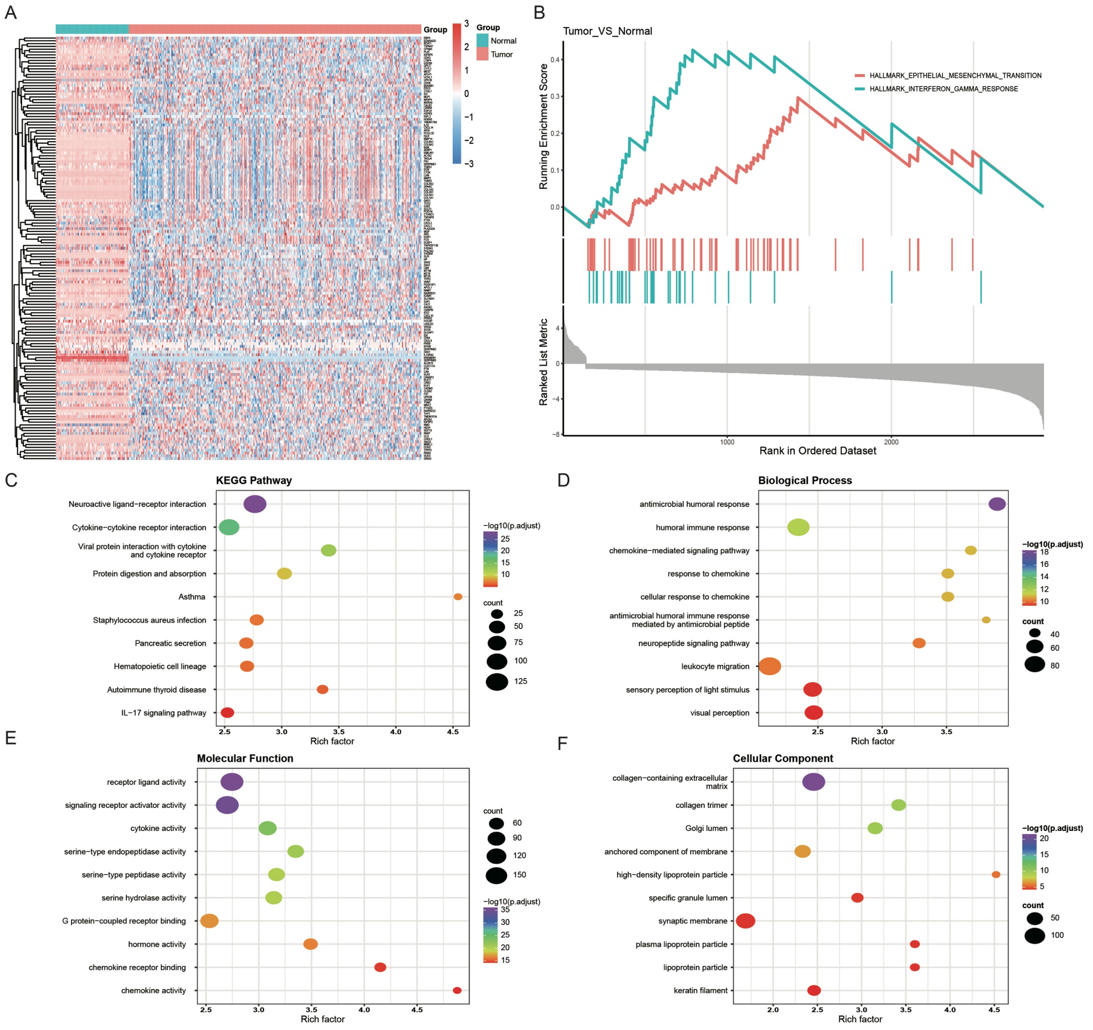

Supplement Figure 2: Differential expression and functional enrichment analysis of BulkRNA disease VS normal samples. A: The expression heat map of the intersection of DEGs and marker genes of active populations, where red represents high expression and blue represents low expression; B: Broken line graph of gene enrichment score of GSEA significant enrichment pathway; C-F: Bubble Diagram of GO and KEGG enrichment analysis of DEGs. The size of the point represents the number of marker genes enriched, and the color represents the significance of enrichment.

Table S1. Gene sets related to oxidative stress response in 17 ROS pathways

GOBP_REACTIVE_OXYGEN_SPECIES_BIOSYNTHETIC_PROCESS http://www.gsea-msigdb.org/gsea/msigdb/cards/GOBP_REACTIVE_OXYGEN_SPECIES_BIOSYNTHETIC_PROCESS MIR675 TRAP1 TLR6 KLF2 MTCO2P12 PARK7 CLCN3 CLU SIRPA TICAM1 CTNS CX3CR1 CYBA CYP1A1 CYP1A2 CYP1B1 CYB5R3 NQO1 DNM2 AGTR2 EDN1 PIKFYVE AKT1 ABCD1 PTK2B ABCD2 DDAH2 DDAH1 ALOX5 MTOR FYN MPV17L GCH1 GCHFR GLA H19 GRIN1 HBB HSP90AA1 HSP90AB1 ICAM1 IFNG IL1B IL10 INS INSR ITGB2 JAK2 ARG2 DUOXA2 MIR132 MIR181A2 MIR181B1 MIR199A1 MIR21 MIR212 MIR24-1 MIR92A1 MIR99B SMAD3 MAOB MMP8 MPO ASS1 MT-CO2 NOS1 NOS2 NOS3 ATP2B4 P2RX4 DUOX2 NOX4 ACOX1 PKD2 DUOX1 PPARA MTARC2 SLC30A10 INAVA SPHK2 PTGIS PTGS2 ADGRB1 WDR35 PTX3 RAB27A RAC1 RORA CLEC7A MTARC1 AGXT2 SLC5A3 SLC18A2 SNCA SOD1 SOD2 SPR MIR590 TSPO TLR4 TNF UCP1 ZNF205 ZC3H12A CYB5B SLC25A33 COA8 CAV1 KHSRP DYNLL1 GBF1 CFLAR CCN6 DUOXA1 RGN KLF4 CD34 ROCK2 CD36 PAGE4 CD47 NOS1AP HDAC4

GOBP_REACTIVE_OXYGEN_SPECIES_METABOLIC_PROCESS http://www.gsea-msigdb.org/gsea/msigdb/cards/GOBP_REACTIVE_OXYGEN_SPECIES_METABOLIC_PROCESS MIR675 HDAC6 TRAP1 RNF41 COQ7 CDKN1A TLR6 KLF2 PRG3 FBLN5 PRDX4 MTCO2P12 NOXA1 PRDX3 RIPK3 PARK7 TMEM106A TUSC2 CLCN3 CLU LRRK2 NOXO1 CPS1 PXDNL CRP ROMO1 SIRPA CRYAB MAPK14 TICAM1 CCN2 CTNS TAFA4 CX3CR1 CYBA CYBB CYP1A1 CYP1A2 CYP1B1 GADD45A DHFR CYB5R3 NQO1 DNM2 DRD5 AGT AGTR1 AGTR2 EDN1 EGFR PIKFYVE AKT1 F2 ABCD1 F2RL1 FANCC PTK2B ABCD2 PLA2R1 SIRT2 FOXM1 SIRT5 SIRT3 NNT DDAH2 DDAH1 FPR2

ALOX12 ALOX5 MTOR FYN IFI6G6PD MPV17L PRDX5 GCH1
GCHFR AATF NOX1 GLS2 GLA SESN1 GNAI2 GNAI3 H19
SH3PXD2B GPX1 GPX3 GRB2 GRIN1 GSTP1 IL19 HBA1 HBA2
HBM HBB HBD HBE1 HBG1 HBG2 HBQ1 HBZ HIF1A
HK2 HP BIRC2 HSP90AA1 HSP90AB1 APOA4 ICAM1 IFNG CCN1
IL1B IL10 INS INSR ITGAM ITGB2 EIF6 JAK2 NRROS ARF4
ARG2 LEP LPO DUOXA2 MIR132 MIR181A2 MIR181B1
MIR199A1 MIR21 MIR212 MIR24-1 MIR27B MIR92A1 MIR99B
SMAD3 MAOB MAPT MMP3 MMP8 MPO MPV17 PLIN5 ASS1
MT3 MT-CO2 MT-ND2 NCF2 NCF4 NDUFS1 NDUFS3
NDUFS4 NFE2L2 NQO2 NOS1 NOS2 NOS3 ATP2B4 P2RX4
DUOX2 NOX4 NOX3 PRDX1 PRKN PAX2 ACOX1 NDUFA13
CYB5R4 PDGFB PDGFRB PDK3 PDK4 PKD2 PMAIP1 ATP7A
DUOX1 PON3 DDIT4 PPARA MTARC2 PID1 RFK PRCP PARL
SLC30A10 INAVA PRKCD SELENOS SPHK2 TIGAR CD177 DHFRP1
PTGIS PTGS2 ADGRB1 WDR35 PREX1 PTX3 RAB27A RAC1
RAC2 ACE2 BCL2 RORA SFTPD CLEC7A MTARC1 AGXT2
PINK1 SLC5A3 NCF1 NCF1B NCF1C BMP7 SLC18A2 SNCA
BNIP3 SOD1 SOD2 SOD3 SPR BRCA1 BST1 SYK MIR590
PRDX2 TFAP2A TGFB1 TGFBR2 THBS1 TSPO TLR2 TLR4
TNF TP53 TPO ACOD1 TYROBP UCP1 VAV1 VDAC1 XDH
ZNF205 PXDN NOX5 ZC3H12A CYB5B EPX SESN2 BCO2
IMMP2L SLC25A33 HVCN1 COA8 CAT CAV1 KHSRP AKR1C3
DYNLL1 BECN1 GBF1 RIPK1 CFLAR CCN6 DUOXA1 RGN
STK17A KLF4 ATP5IF1 CD34 ATG5 ROCK2 CD36 PAGE4
PRDX6 CD47 SH3PXD2A NOS1AP HDAC4 CCS

GOBP_POSITIVE_REGULATION_OF_REACTIVE_OXYGEN_SPECIES_BIOSYNTHETIC_PROCESS http://www.gsea-msigdb.org/gsea/msigdb/cards/GOBP_POSITIVE_REGULATION_OF_REACTIVE_OXYGEN_SPECIES_BIOSYNTHETIC_PROCESS MIR675 TLR6 KLF2
MTCO2P12 PARK7 CLCN3 CLU TICAM1 CYBA DNM2 AGTR2
EDN1 AKT1 PTK2B DDAH2 DDAH1 MTOR H19GRIN1 HBB
HSP90AA1 HSP90AB1 ICAM1 IFNG IL1B INSR ITGB2 JAK2

DUOXA2 MIR181A2 MIR181B1 MIR24-1 MIR99B SMAD3 MMP8 ASS1 MT-CO2 P2RX4 PKD2 PTGS2 ADGRB1 PTX3 RAB27A CLEC7A AGXT2 SLC5A3 SOD2 TLR4 TNF ZNF205 DUOXA1 KLF4 CD36 NOS1AP HDAC4

GOBP_NEGATIVE_REGULATION_OF_REACTIVE_OXYGEN_SPECIES_BIOSYNTHETIC_PROCESS http://www.gsea-msigdb.org/gsea/msigdb/cards/GOBP_NEGATIVE_REGULATION_OF_REACTIVE_OXYGEN_SPECIES_BIOSYNTHETIC_PROCESS TRAP1 PARK7 SIRPA CTNS ABCD1 ABCD2 FYN MPV17L GLA IL10 INS MIR132 MIR181A2 MIR199A1 MIR21 MIR212 MIR92A1 ATP2B4 PPARA SLC30A10 PTGIS WDR35 SLC18A2 MIR590 TSPO ZC3H12A COA8 CAV1 KHSRP DYNLL1 CFLAR RGN CD34 ROCK2 PAGE4

GOBP_NEGATIVE_REGULATION_OF_REACTIVE_OXYGEN_SPECIES_METABOLIC_PROCESS http://www.gsea-msigdb.org/gsea/msigdb/cards/GOBP_NEGATIVE_REGULATION_OF_REACTIVE_OXYGEN_SPECIES_METABOLIC_PROCESS MIR675 HDAC6 TRAP1 PARK7 SIRPA CRYAB CTNS ABCD1 ABCD2 SIRT2 SIRT5 SIRT3 FYN G6PD MPV17L AATF GLA H19 HIF1A HK2 HP IL10 INS MIR132 MIR181A2 MIR199A1 MIR21 MIR212 MIR92A1 MMP3 PLIN5 MT3 ATP2B4 PRKN PAX2 PON3 PPARA SLC30A10 TIGAR PTGIS WDR35 BCL2 PINK1 SLC18A2 BNIP3 BRCA1 MIR590 TFAP2A TSPO TP53 VDAC1 ZC3H12A COA8 CAV1 KHSRP DYNLL1 BECN1 CFLAR RGN CD34 ATG5 ROCK2 PAGE4

GOBP_POSITIVE_REGULATION_OF_REACTIVE_OXYGEN_SPECIES_METABOLIC_PROCESS http://www.gsea-msigdb.org/gsea/msigdb/cards/GOBP_POSITIVE_REGULATION_OF_REACTIVE_OXYGEN_SPECIES_METABOLIC_PROCESS MIR675 RNF41 CDKN1A TLR6 KLF2 MTCO2P12 RIPK3 PARK7 TMEM106A CLCN3 CLU CRP ROMO1 MAPK14 TICAM1 CYBA GADD45A DNM2 AGT AGTR1 AGTR2 EDN1 EGFR AKT1 F2 F2RL1 PTK2B NNT

DDAH2 DDAH1 FPR2 MTOR GNAI2 GNAI3 H19 GRB2 GRIN1 GSTP1 HBB HSP90AA1 HSP90AB1 ICAM1 IFNG IL1B INSR ITGAM ITGB2 JAK2 LEP DUOXA2 MIR181A2 MIR181B1 MIR24-1 MIR99B SMAD3 MAPT MMP8 ASS1 MT-CO2 NFE2L2 NQO2 P2RX4 NOX4 PDGFB PDGFRB PKD2 PID1 PRKCD CD177 PTGS2 ADGRB1 PTX3 RAB27A ACE2 CLEC7A AGXT2 SLC5A3 SNCA SOD1 SOD2 SYK TGFB1 TGFBR2 THBS1 TSPO TLR4 TNF TP53 ACOD1 TYROBP XDH ZNF205 ZC3H12A HVCN1 AKR1C3 RIPK1 DUOXA1 KLF4 CD36 NOS1AP HDAC4

GOBP_MITOCHONDRIAL_ELECTRON_TRANSPORT_CYTOCHROME_C_TO_OXYGEN http://www.gsea-msigdb.org/gsea/msigdb/cards/GOBP_MITOCHONDRIAL_ELECTRON_TRANSPORT_CYTOCHROME_C_TO_OXYGEN MTCO2P12 COX4I1 COX5B COX6A1 COX6A2 COX6B1 COX6C COX7B COX7C COX8A COX10 COX15 AFG1L MT-CO1 MT-CO2 MT-CO3 NDUFA4 CYCS COX4I2 COX7A2L COX5A

GOBP_HYDROGEN_PEROXIDE_METABOLIC_PROCESS http://www.gsea-msigdb.org/gsea/msigdb/cards/GOBP_HYDROGEN_PEROXIDE_METABOLIC_PROCESS HDAC6 PRDX4 MTCO2P12 NOXA1 PRDX3 PARK7 NOXO1 PXDNL CTNS CYBA CYP1A1 CYP1A2 EGFR NNT FYN MPV17L PRDX5 NOX1 GPX1 GPX3 HBA1 HBA2 HBM HBB HBD HBE1 HBG1 HBG2 HBQ1 HBZ HP APOA4 LPO DUOXA2 MAOB MMP3 MPO MT-CO2 DUOX2 PRDX1 ACOX1 DUOX1 RAC1 RAC2 PINK1 SNCA SOD1 SOD2 PRDX2 TPO ZNF205 PXDN EPX CAT DUOXA1 PRDX6

GOBP_HYDROGEN_PEROXIDE_CATABOLIC_PROCESS http://www.gsea-msigdb.org/gsea/msigdb/cards/GOBP_HYDROGEN_PEROXIDE_CATABOLIC_PROCESS PRDX4 PRDX3 PXDNL NNT PRDX5 GPX1 GPX3 HBA1 HBA2 HBM HBB HBD HBE1 HBG1 HBG2 HBQ1 HBZ HP APOA4 LPO MPO DUOX2 PRDX1 DUOX1 SNCA PRDX2 TPO PXDN EPX CAT PRDX6

GOBP_PENTOSE_METABOLIC_PROCESS http://www.gsea-msigdb.org/gsea/msigdb/cards/GOBP_PENTOSE_METABOLIC_PROCESS NUDT5 RPIA G6PD DHDH OTOGL OTOG DCXR PGD FGGY RPE RBKS TALDO1 TKT RPEL1 XYLB

GOBP_RESPONSE_TO_REACTIVE_OXYGEN_SPECIES http://www.gsea-msigdb.org/gsea/msigdb/cards/GOBP_RESPONSE_TO_REACTIVE_OXYGEN_SPECIES ADA MIR675 HDAC6 PPIF TRAP1 CDK2 NET1 SIGMAR1 KLF2 RACK1 STK25 FBLN5 TXNRD2 TXNIP CAMKK2 PPARGC1A PRDX3 RIPK3 ZNF277 PDCD10 TREX1 PARK7 CHUK AGAP3 LRRK2 CCR7 PLK3 COL1A1 PPARGC1B CRK ROMO1 SIRPA CRYAB CRYGD SESN3 CYP1B1 CYP2E1 DHFR NQO1 DNM2 DPEP1 DUSP1 ECT2 EDN1 EEF2 EGFR ENDOG AKT1 ERCC6 ERN1 ETS1 EZH2 FABP1 FANCC PTK2B FER FKBP1B SETX FOXO1 KDM6B SIRT1 FOS FXN ABL1 FYN PRDX5 GCH1 SESN1 GNAO1 H19 GPR37 GPX1 GSTP1 GUCY1B1 ANXA1 HBA1 HBA2 HBB HDAC2 HGF HIF1A HMOX1 HNRNPD HP APEX1 HSF1 APOA4 HYAL1 APOD APOE IL6 AQP1 IL10 JUN KCNA5 KCNC2 ERCC6L2 ARG1 RHOB LDHA MIRLET7B MIR103A1 MIR107 MIR133A1 MIR17 MIR21 MIR34A MIR92A1 MAPT MB MAP3K5 MET MMP2 MMP3 MMP9 MPO MPV17 MT3 MT-ND5 MT-ND6 MTR MYB NFE2L2 NOS3 PJVK PRDX1 PAWR PAX2 GLRX2 PCNA ZNF580 NME8 PDE8A OSER1 PDGFRA PDGFRB PDK2 STK26 PKD2 ATP7A EGLN1 APTX ADPRS ANKZF1 PPP2CB NUDT15 PPP5C IMPACT SMPD3 PRKAA1 AXL PRKCD MAPK1 MAPK3 MAPK7 MAPK8 MAPK9 MAPK13 BAD CBX8 DHFRP1 BAK1 PTPRK PTPRN PXN PYCR1 PLEKHA1 BCL2 RELA RPS3 S100A7 CCL19 SDC1 TRA2B PINK1 NCF1 SLC8A1 BMP7 BNIP3 SOD1 SOD2 SOD3 SRC STAR BTK PRDX2 TNFAIP3 TPM1 TRAF2 TRPC6 TRPM2 TXN UBE3A UCP1 UCP2 UCP3 SCGB1A1 PCGF2 RNF112 NR4A3 PDGFD FOSL1 CAPN2 CASP3 SESN2 STK24 COA8 MAP1LC3A CAT PPP1R15B AKR1C3 BECN1 HYAL2 RIPK1

ADAM9 IL18RAP CFLAR SPHK1 CCNA2 TRPA1 GPR37L1 KLF4 CD36 CCS

GOBP_REGULATION_OF_RESPONSE_TO_REACTIVE_OXYGEN_SPECIES http://www.gsea-msigdb.org/gsea/msigdb/cards/GOBP_REGULATION_OF_RESPONSE_TO_REACTIVE_OXYGEN_SPECIES MIR675 HDAC6 TRAP1 RACK1 FBLN5 PARK7 LRRK2 SESN3 DHFR ENDOGGCH1 SESN1 H19GPR37 HGF IL10 MIRLET7B MIR133A1 MIR17 MIR21 MIR34A MIR92A1 MET NFE2L2 PAWR PDE8A STK26 DHFRP1 PYCR1 PINK1 BMP7 TXN NR4A3 SESN2 RIPK1 GPR37L1 CD36

GOBP_CELLULAR_RESPONSE_TO_REACTIVE_OXYGEN_SPECIES http://www.gsea-msigdb.org/gsea/msigdb/cards/GOBP_CELLULAR_RESPONSE_TO_REACTIVE_OXYGEN_SPECIES MIR675 HDAC6 PPIF TRAP1 CDK2 NET1 SIGMAR1 KLF2 RACK1 STK25 FBLN5 CAMKK2 PRDX3 RIPK3 ZNF277 PDCD10 TREX1 PARK7 CHUK AGAP3 LRRK2 PPARGC1B CRK ROMO1 SIRPA CRYGDCYP1B1 DHFR NQO1 DNM2 DPEP1 ECT2 EGFR ENDOGAKT1 ERN1 ETS1 EZH2 FABP1 FANCC FER SETX FOXO1 KDM6B SIRT1 FOS FXN ABL1 PRDX5 GCH1 H19GPR37 GUCY1B1 ANXA1 HDAC2 HGF HNRNPD APEX1 HSF1 APOA4 IL6 AQP1 IL10 JUN KCNC2 ERCC6L2 ARG1 RHOB MIRLET7B MIR103A1 MIR107 MIR133A1 MIR17 MIR21 MIR34A MIR92A1 MAPT MAP3K5 MET MMP2 MMP3 MMP9 MPO MPV17 MT3 MTR MYB NFE2L2 NOS3 PJVK PRDX1 PAWR PAX2 GLRX2 PCNA ZNF580 NME8 PDE8A OSER1 PDGFRA PDK2 STK26 PKD2 ATP7A ADPRS ANKZF1 PPP5C IMPACT SMPD3 PRKAA1 AXL PRKCD MAPK1 MAPK3 MAPK7 MAPK8 MAPK9 MAPK13 CBX8 DHFRP1 PTPRK PXN PYCR1 PLEKHA1 RELA RPS3 PINK1 NCF1 BMP7 BNIP3 SOD1 SOD2 SOD3 SRC BTK PRDX2 TNFAIP3 TPM1 TRAF2 TRPC6 TRPM2 TXN UCP1 PCGF2 RNF112 NR4A3 PDGFD MAP1LC3A AKR1C3 BECN1 RIPK1 IL18RAP CFLAR SPHK1 CCNA2 GPR37L1

KLF4	CD36	CCS

GOBP_REGULATION_OF_REACTIVE_OXYGEN_SPECIES_BIOSYNTHETIC_PROCESS	http://www.gsea-msigdb.org/gsea/msigdb/cards/GOBP_REGULATION_OF_REACTIVE_OXYGEN_SPECIES_BIOSYNTHETIC_PROCESS	MIR675	TRAP1	TLR6	KLF2	MTCO2P12	PARK7	CLCN3	CLU	SIRPA	TICAM1	CTNS	CX3CR1	CYBA	DNM2	AGTR2	EDN1	PIKFYVE	AKT1	ABCD1	PTK2B	ABCD2	DDAH2	DDAH1	ALOX5	MTOR	FYN	MPV17L	GLA	H19	GRIN1	HBB	HSP90AA1	HSP90AB1	ICAM1	IFNG	IL1B	IL10	INS	INSR	ITGB2	JAK2	ARG2	DUOXA2	MIR132	MIR181A2	MIR181B1	MIR199A1	MIR21	MIR212	MIR24-1	MIR92A1	MIR99B	SMAD3	MMP8	ASS1	MT-CO2	ATP2B4	P2RX4	PKD2	PPARA	SLC30A10	SPHK2	PTGIS	PTGS2	ADGRB1	WDR35	PTX3	RAB27A	RAC1	CLEC7A	AGXT2	SLC5A3	SLC18A2	SNCA	SOD2	MIR590	TSPO	TLR4	TNF	UCP1	ZNF205	ZC3H12A	SLC25A33	COA8	CAV1	KHSRP	DYNLL1	CFLAR	CCN6	DUOXA1	RGN	KLF4	CD34	ROCK2	CD36	PAGE4	CD47	NOS1AP	HDAC4

GOBP_REGULATION_OF_REACTIVE_OXYGEN_SPECIES_METABOLIC_PROCESS	http://www.gsea-msigdb.org/gsea/msigdb/cards/GOBP_REGULATION_OF_REACTIVE_OXYGEN_SPECIES_METABOLIC_PROCESS	MIR675	HDAC6	TRAP1	RNF41	COQ7	CDKN1A	TLR6	KLF2	FBLN5	MTCO2P12	NOXA1	RIPK3	PARK7	TMEM106A	TUSC2	CLCN3	CLU	NOXO1	CRP	ROMO1	SIRPA	CRYAB	MAPK14	TICAM1	CTNS	CX3CR1	CYBA	CYP1B1	GADD45A	DHFR	DNM2	AGT	AGTR1	AGTR2	EDN1	EGFR	PIKFYVE	AKT1	F2	ABCD1	F2RL1	PTK2B	ABCD2	SIRT2	FOXM1	SIRT5	SIRT3	NNT	DDAH2	DDAH1	FPR2	ALOX5	MTOR	FYN	G6PD	MPV17L	GCH1	AATF	GLA	GNAI2	GNAI3	H19	GRB2	GRIN1	GSTP1	HBB	HIF1A	HK2	HP	BIRC2	HSP90AA1	HSP90AB1	ICAM1	IFNG	IL1B	IL10	INS	INSR	ITGAM	ITGB2	EIF6	JAK2	ARF4	ARG2	LEP	DUOXA2	MIR132	MIR181A2	MIR181B1	MIR199A1	MIR21	MIR212	MIR24-1	MIR27B	MIR92A1	MIR99B

SMAD3 MAPT MMP3 MMP8 MPV17 PLIN5 ASS1 MT3 MT-CO2 NFE2L2 NQO2 ATP2B4 P2RX4 NOX4 PRKN PAX2 PDGFB PDGFRB PDK3 PKD2 PON3 PPARA PID1 PRCP PARL SLC30A10 PRKCD SELENOS SPHK2 TIGAR CD177 DHFRP1 PTGIS PTGS2 ADGRB1 WDR35 PTX3 RAB27A RAC1 RAC2 ACE2 BCL2 CLEC7A AGXT2 PINK1 SLC5A3 BMP7 SLC18A2 SNCA BNIP3 SOD1 SOD2 BRCA1 BST1 SYK MIR590 PRDX2 TFAP2A TGFB1 TGFBR2 THBS1 TSPO TLR4 TNF TP53 ACOD1 TYROBP UCP1 VDAC1 XDH ZNF205 ZC3H12A BCO2 SLC25A33 HVCN1 COA8 CAV1 KHSRP AKR1C3 DYNLL1 BECN1 RIPK1 CFLAR CCN6 DUOXA1 RGN STK17A KLF4 CD34 ATG5 ROCK2 CD36 PAGE4 CD47 NOS1AP HDAC4

GOBP_NEGATIVE_REGULATION_OF_RESPONSE_TO_REACTIVE_OXYGEN_SPECIES http://www.gsea-msigdb.org/gsea/msigdb/cards/GOBP_NEGATIVE_REGULATION_OF_RESPONSE_TO_REACTIVE_OXYGEN_SPECIES MIR675 TRAP1 RACK1 PARK7 LRRK2 H19 GPR37 HGF IL10 MIRLET7B MIR133A1 MIR17 MIR21 MIR92A1 MET NFE2L2 PDE8A PYCR1 PINK1 TXN NR4A3 GPR37L1

GOMF_SUPEROXIDE_GENERATING_NAD_P_H_OXIDASE_ACTIVITY http://www.gsea-msigdb.org/gsea/msigdb/cards/GOMF_SUPEROXIDE_GENERATING_NAD_P_H_OXIDASE_ACTIVITY CYBA CYBB NOX1 NCF2 NCF4 DUOX2 NOX4 NOX3 DUOX1 NCF1 NOX5

Table S2. 467 oxidative stress-related genes

Symbol
MIR675
TRAP1
TLR6
KLF2
MTCO2P12
PARK7
CLCN3
CLU
SIRPA
TICAM1
CTNS
CX3CR1
CYBA
CYP1A1
CYP1A2
CYP1B1
CYB5R3
NQO1
DNM2
AGTR2
EDN1
PIKFYVE
AKT1
ABCD1
PTK2B
ABCD2
DDAH2
DDAH1
ALOX5
MTOR

FYN

MPV17L

GCH1

GCHFR

GLA

H19

GRIN1

HBB

HSP90AA1

HSP90AB1

ICAM1

IFNG

IL1B

IL10

INS

INSR

ITGB2

JAK2

ARG2

DUOXA2

MIR132

MIR181A2

MIR181B1

MIR199A1

MIR21

MIR212

MIR24-1

MIR92A1

MIR99B

SMAD3

MAOB

MMP8

MPO

ASS1

MT-CO2

NOS1

NOS2

NOS3

ATP2B4

P2RX4

DUOX2

NOX4

ACOX1

PKD2

DUOX1

PPARA

MTARC2

SLC30A10

INAVA

SPHK2

PTGIS

PTGS2

ADGRB1

WDR35

PTX3

RAB27A

RAC1

RORA

CLEC7A

MTARC1

AGXT2

SLC5A3

SLC18A2

SNCA

SOD1

SOD2

SPR

MIR590

TSPO

TLR4

TNF

UCP1

ZNF205

ZC3H12A

CYB5B

SLC25A33

COA8

CAV1

KHSRP

DYNLL1

GBF1

CFLAR

CCN6

DUOXA1

RGN

KLF4

CD34

ROCK2

CD36

PAGE4

CD47

NOS1AP

HDAC4

HDAC6

RNF41

COQ7

CDKN1A

PRG3

FBLN5

PRDX4

NOXA1

PRDX3

RIPK3

TMEM106A

TUSC2

LRRK2

NOXO1

CPS1

PXDNL

CRP

ROMO1

CRYAB

MAPK14

CCN2

TAFA4

CYBB

GADD45A

DHFR

DRD5

AGT

AGTR1

EGFR

F2

F2RL1

FANCC

PLA2R1

SIRT2

FOXM1

SIRT5

SIRT3

NNT

FPR2

ALOX12

IFI6

G6PD

PRDX5

AATF

NOX1

GLS2

SESN1

GNAI2

GNAI3

SH3PXD2B

GPX1

GPX3

GRB2

GSTP1

IL19

HBA1

HBA2

HBM

HBD

HBE1

HBG1

HBG2

HBQ1

HBZ

HIF1A

HK2

HP

BIRC2

APOA4

CCN1

ITGAM

EIF6

NRROS

ARF4

LEP

LPO

MIR27B

MAPT

MMP3

MPV17

PLIN5

MT3

MT-ND2

NCF2

NCF4

NDUFS1

NDUFS3

NDUFS4

NFE2L2

NQO2

NOX3

PRDX1

PRKN

PAX2

NDUFA13

CYB5R4

PDGFB

PDGFRB

PDK3

PDK4

PMAIP1

ATP7A

PON3

DDIT4

PID1

RFK

PRCP

PARL

PRKCD

SELENOS

TIGAR

CD177

DHFRP1

PREX1

RAC2

ACE2

BCL2

SFTPD

PINK1

NCF1

NCF1B

NCF1C

BMP7

BNIP3

SOD3

BRCA1

BST1

SYK

PRDX2

TFAP2A

TGFB1

TGFBR2

THBS1

TLR2

TP53

TPO

ACOD1

TYROBP

VAV1

VDAC1

XDH

PXDN

NOX5

EPX

SESN2

BCO2

IMMP2L

HVCN1

CAT

AKR1C3

BECN1

RIPK1

STK17A

ATP5IF1

ATG5

PRDX6

SH3PXD2A

CCS

COX4I1

COX5B

COX6A1

COX6A2

COX6B1

COX6C

COX7B

COX7C

COX8A

COX10

COX15

AFG1L

MT-CO1

MT-CO3

NDUFA4

CYCS

COX4I2

COX7A2L

COX5A

NUDT5

RPIA

DHDH

OTOGL

OTOG

DCXR

PGD

FGGY

RPE

RBKS

TALDO1

TKT

RPEL1

XYLB

ADA

PPIF

CDK2

NET1

SIGMAR1

RACK1

STK25

TXNRD2

TXNIP

CAMKK2

PPARGC1A

ZNF277

PDCD10

TREX1

CHUK

AGAP3

CCR7

PLK3

COL1A1

PPARGC1B

CRK

CRYGD

SESN3

CYP2E1

DPEP1

DUSP1

ECT2

EEF2

ENDOG

ERCC6

ERN1

ETS1

EZH2

FABP1

FER

FKBP1B

SETX

FOXO1

KDM6B

SIRT1

FOS

FXN

ABL1

GNAO1

GPR37

GUCY1B1

ANXA1

HDAC2

HGF

HMOX1

HNRNPD

APEX1

HSF1

HYAL1

APOD

APOE

IL6

AQP1

JUN

KCNA5

KCNC2

ERCC6L2

ARG1

RHOB

LDHA

MIRLET7B

MIR103A1

MIR107

MIR133A1

MIR17

MIR34A

MB

MAP3K5

MET

MMP2

MMP9

MT-ND5

MT-ND6

MTR

MYB
PJVK
PAWR
GLRX2
PCNA
ZNF580
NME8
PDE8A
OSER1
PDGFRA
PDK2
STK26
EGLN1
APTX
ADPRS
ANKZF1
PPP2CB
NUDT15
PPP5C
IMPACT
SMPD3
PRKAA1
AXL
MAPK1
MAPK3
MAPK7
MAPK8
MAPK9
MAPK13
BAD
CBX8
BAK1
PTPRK

PTPRN

PXN

PYCR1

PLEKHA1

RELA

RPS3

S100A7

CCL19

SDC1

TRA2B

SLC8A1

SRC

STAR

BTK

TNFAIP3

TPM1

TRAF2

TRPC6

TRPM2

TXN

UBE3A

UCP2

UCP3

SCGB1A1

PCGF2

RNF112

NR4A3

PDGFD

FOSL1

CAPN2

CASP3

STK24

MAP1LC3A

PPP1R15B

HYAL2

ADAM9

IL18RAP

SPHK1

CCNA2

TRPA1

GPR37L1

Table S3. Subgroup identification and annotation of preprocessed and integrated cells and genes

Cell	patient	CellType	activecell	seurat_clusters	AUC	SingleR_MID_detail.RNA_snn_res.0.5
10x_3288_t1_AAACATACCTTCCG-1	Patient5	Monocytes	nonactive	8	0.07231628	Monocytes_8
10x_3288_t1_AAACATACTCCTAT-1	Patient5	Monocytes	nonactive	0	0.05305624	Monocytes_0
10x_3288_t1_AAACATTGAACTGC-1	Patient5	Monocytes	nonactive	0	0.06299537	Monocytes_0
10x_3288_t1_AAACATTGCTGACA-1	Patient5	Progenitors	active	1	0.16972608	Progenitors_1
10x_3288_t1_AAACCGTGACAGTC-1	Patient5	Progenitors	active	1	0.12819415	Progenitors_1
10x_3288_t1_AAACCGTGTACAGC-1	Patient5	Monocytes	nonactive	4	0.04525712	Monocytes_4
10x_3288_t1_AAACCGTGTATCGG-1	Patient5	Monocytes	nonactive	0	0.05717773	Monocytes_0
10x_3288_t1_AAACCGTGTTGAGC-1	Patient5	Monocytes	nonactive	8	0.03937607	Monocytes_8
10x_3288_t1_AAACGCACAGAACA-1	Patient5	Monocytes	nonactive	8	0.01797603	Monocytes_8
10x_3288_t1_AAACGCACATAAGG-1	Patient5	Progenitors	active	1	0.16189525	Progenitors_1
10x_3288_t1_AAACGCACCACACA-1	Patient5	Progenitors	active	1	0.14111344	Progenitors_1
10x_3288_t1_AAACGCACTCTGGA-1	Patient5	Monocytes	nonactive	4	0.04435356	Monocytes_4
10x_3288_t1_AAACGCTGAGTACC-1	Patient5	Progenitors	nonactive	5	0.09046668	Progenitors_5
10x_3288_t1_AAACGCTGCATTCT-1	Patient5	Monocytes	nonactive	0	0.07878384	Monocytes_0

10x_3288_t1_AAACGCTGGTCTAG-1 Patient5 Progenitors nonactive 1 0.10319574
Progenitors_1
10x_3288_t1_AAACGCTGTCCGTC-1 Patient5 Progenitors active 1 0.16983704
Progenitors_1
10x_3288_t1_AAACGCTGTGAAGA-1 Patient5 Progenitors nonactive 1 0.11733562
Progenitors_1
10x_3288_t1_AAACGGCTAAAGCA-1 Patient5 Dendritic cells nonactive 10 0.09336757
Dendritic cells_10
10x_3288_t1_AAACGGCTCGCATA-1 Patient5 Monocytes nonactive 0 0.04327563
Monocytes_0
10x_3288_t1_AAACGGCTGGAGCA-1 Patient5 Progenitors active 1 0.12180585
Progenitors_1
10x_3288_t1_AAACGGCTGGTTTG-1 Patient5 Monocytes nonactive 0 0.08149452
Monocytes_0
10x_3288_t1_AAACGGCTTTCTAC-1 Patient5 Monocytes nonactive 0 0.03054657
Monocytes_0
10x_3288_t1_AAACTTGACACACA-1 Patient5 Monocytes nonactive 0 0.03769577
Monocytes_0
10x_3288_t1_AAACTTGAGATAGA-1 Patient5 Progenitors nonactive 5 0.08395156
Progenitors_5
10x_3288_t1_AAAGACGAAGTGCT-1 Patient5 Monocytes nonactive 0 0.06718027
Monocytes_0
10x_3288_t1_AAAGACGACTATGG-1 Patient5 Progenitors active 1 0.15327183
Progenitors_1
10x_3288_t1_AAAGACGAGCTTAG-1 Patient5 Monocytes nonactive 4 0.04135756
Monocytes_4
10x_3288_t1_AAAGAGACGACGAG-1 Patient5 Monocytes nonactive 0 0.07199924
Monocytes_0
10x_3288_t1_AAAGCAGAACCGAT-1 Patient5 Monocytes nonactive 0 0.04868112
Monocytes_0
10x_3288_t1_AAAGCAGACCCACT-1 Patient5 Monocytes nonactive 8 0.03384376
Monocytes_8
10x_3288_t1_AAAGCAGAGCGTTA-1 Patient5 Monocytes nonactive 0 0.06343922

Monocytes_0
10x_3288_t1_AAAGCAGAGCTACA-1 Patient5 Monocytes nonactive 4 0.05980914 Monocytes_4
10x_3288_t1_AAAGCCTGCAGATC-1 Patient5 Monocytes nonactive 4 0.02287426 Monocytes_4
10x_3288_t1_AAAGCCTGCTGAAC-1 Patient5 Progenitors active 1 0.14001966 Progenitors_1
10x_3288_t1_AAAGCCTGGCCTTC-1 Patient5 Progenitors active 1 0.16428888 Progenitors_1
10x_3288_t1_AAAGCCTGTGCCAA-1 Patient5 Monocytes nonactive 4 0.01312536 Monocytes_4
10x_3288_t1_AAAGCCTGTGGTTG-1 Patient5 Monocytes nonactive 0 0.07491599 Monocytes_0
10x_3288_t1_AAAGGCCTTAAGGA-1 Patient5 Progenitors active 1 0.15935895 Progenitors_1
10x_3288_t1_AAAGGCCTTTCTTG-1 Patient5 Monocytes nonactive 0 0.05854099 Monocytes_0
10x_3288_t1_AAAGTTTGCTGTTT-1 Patient5 Monocytes nonactive 0 0.02132078 Monocytes_0
10x_3288_t1_AAAGTTTGGTTGGT-1 Patient5 Monocytes nonactive 13 0.02626657 Monocytes_13
10x_3288_t1_AAAGTTTGTTCCCG-1 Patient5 Monocytes nonactive 4 0.0252679 Monocytes_4
10x_3288_t1_AAATACTGACCAAC-1 Patient5 Progenitors active 1 0.17139053 Progenitors_1
10x_3288_t1_AAATACTGGAATAG-1 Patient5 Progenitors nonactive 1 0.09286031 Progenitors_1
10x_3288_t1_AAATACTGTCTTAC-1Patient5 Monocytes nonactive 0 0.03041976 Monocytes_0
10x_3288_t1_AAATCAACCATTTC-1 Patient5 Progenitors active 1 0.12664067 Progenitors_1
10x_3288_t1_AAATCAACCTGTAG-1 Patient5 Progenitors nonactive 1 0.11391161 Progenitors_1

10x_3288_t1_AAATCAACGACGGA-1 Patient5 Monocytes nonactive 0 0.02005263 Monocytes_0
10x_3288_t1_AAATCATGGCGGAA-1 Patient5 Monocytes nonactive 4 0.0337328 Monocytes_4
10x_3288_t1_AAATCATGGTTTCT-1 Patient5 Monocytes nonactive 4 0.07872044 Monocytes_4
10x_3288_t1_AAATCATGTCCTCG-1 Patient5 Monocytes nonactive 4 0.0240156 Monocytes_4
10x_3288_t1_AAATCATGTGCAGT-1 Patient5 Progenitors active 1 0.17101008 Progenitors_1
10x_3288_t1_AAATCCCTCTGACA-1 Patient5 Monocytes nonactive 4 0.03756896 Monocytes_4
10x_3288_t1_AAATCTGAACACAC-1 Patient5 Monocytes nonactive 4 0.06345508 Monocytes_4
10x_3288_t1_AAATCTGAACTCTT-1 Patient5 Monocytes nonactive 4 0.03647518 Monocytes_4
10x_3288_t1_AAATCTGAGAGAGC-1 Patient5 Monocytes nonactive 4 0.02030626 Monocytes_4
10x_3288_t1_AAATCTGATGCCCT-1Patient5 Monocytes nonactive 4 0.05342084 Monocytes_4
10x_3288_t1_AAATGGGAAAAGTG-1 Patient5 Monocytes nonactive 0 0.07155539 Monocytes_0
10x_3288_t1_AAATGGGAGTACAC-1 Patient5 Monocytes nonactive 0 0.03831399 Monocytes_0
10x_3288_t1_AAATGTTGAGCTCA-1 Patient5 Monocytes nonactive 0 0.00337645 Monocytes_0
10x_3288_t1_AAATGTTGCACTGA-1 Patient5 Monocytes nonactive 0 0.02308034 Monocytes_0
10x_3288_t1_AAATGTTGGCCATA-1 Patient5 Monocytes nonactive 0 0.06523049 Monocytes_0
10x_3288_t1_AAATGTTGGTTTGG-1 Patient5 Progenitors active 1 0.15141716 Progenitors_1
10x_3288_t1_AAATGTTGTAAGGA-1 Patient5 Progenitors active 1 0.11752584

Progenitors_1
10x_3288_t1_AAATGTTGTATCGG-1 Patient5 Monocytes nonactive 4 0.03560332 Monocytes_4
10x_3288_t1_AAATTCGAAACAGA-1 Patient5 Monocytes nonactive 0 0.04585949 Monocytes_0
10x_3288_t1_AAATTCGAGGACTT-1 Patient5 Monocytes nonactive 0 0.11386405 Monocytes_0
10x_3288_t1_AAATTGACAACCTG-1 Patient5 Monocytes nonactive 0 0.00657853 Monocytes_0
10x_3288_t1_AAATTGACCGTGTA-1 Patient5 Progenitors active 1 0.16208547 Progenitors_1
10x_3288_t1_AAATTGACGCGTTA-1 Patient5 Monocytes nonactive 4 0.04043815 Monocytes_4
10x_3288_t1_AACAAACTGGAAAT-1 Patient5 Monocytes nonactive 4 0.05494262 Monocytes_4
10x_3288_t1_AACAAACTGTCTTT-1 Patient5 Monocytes nonactive 4 0.0750428 Monocytes_4
10x_3288_t1_AACAATACAACCGT-1 Patient5 Monocytes nonactive 0 0.04224526 Monocytes_0
10x_3288_t1_AACAATACCTAGCA-1 Patient5 Monocytes nonactive 0 0.08190666 Monocytes_0
10x_3288_t1_AACAATACTGCGTA-1 Patient5 Monocytes nonactive 0 0.02843827 Monocytes_0
10x_3288_t1_AACACGTGCTATGG-1 Patient5 Monocytes nonactive 4 0.03457295 Monocytes_4
10x_3288_t1_AACACGTGCTGATG-1 Patient5 Monocytes nonactive 4 0.06548412 Monocytes_4
10x_3288_t1_AACACGTGCTTACT-1 Patient5 Progenitors nonactive 1 0.11392746 Progenitors_1
10x_3288_t1_AACACGTGTGCTAG-1 Patient5 Monocytes nonactive 0 0.04514615 Monocytes_0
10x_3288_t1_AACACTCTCACTCC-1 Patient5 Monocytes nonactive 4 0.03690318 Monocytes_4

10x_3288_t1_AACACTCTGGTCAT-1 Patient5 Progenitors active 1 0.13903684 Progenitors_1
10x_3288_t1_AACACTCTTCGACA-1 Patient5 Monocytes nonactive 0 0.05974574 Monocytes_0
10x_3288_t1_AACAGAGACTCGCT-1 Patient5 Progenitors nonactive 1 0.08696341 Progenitors_1
10x_3288_t1_AACAGAGATGTGCA-1 Patient5 Monocytes nonactive 0 0.04899816 Monocytes_0
10x_3288_t1_AACAGCACACCCAA-1 Patient5 Monocytes nonactive 4 0.04679475 Monocytes_4
10x_3288_t1_AACAGCACCACACA-1 Patient5 Progenitors active 1 0.14770782 Progenitors_1
10x_3288_t1_AACAGCACCCATGA-1 Patient5 Progenitors active 1 0.11901592 Progenitors_1
10x_3288_t1_AACAGCACCGGTAT-1 Patient5 Monocytes nonactive 4 0.05789107 Monocytes_4
10x_3288_t1_AACAGCACGCGAGA-1 Patient5 Progenitors nonactive 1 0.09644284 Progenitors_1
10x_3288_t1_AACAGCACGTTCGA-1 Patient5 Monocytes nonactive 0 0.06965316 Monocytes_0
10x_3288_t1_AACATTGATCCTGC-1 Patient5 Progenitors active 1 0.13634202 Progenitors_1
10x_3288_t1_AACCACGACTAGAC-1 Patient5 Progenitors active 6 0.15401687 Progenitors_6
10x_3288_t1_AACCACGAGTTTGG-1 Patient5 Progenitors active 1 0.20230169 Progenitors_1
10x_3288_t1_AACCACGATGAGGG-1 Patient5 Monocytes nonactive 4 0.02721768 Monocytes_4
10x_3288_t1_AACCAGTGAAGCAA-1 Patient5 Monocytes nonactive 8 0.04647771 Monocytes_8
10x_3288_t1_AACCAGTGGAAACA-1 Patient5 Progenitors nonactive 6 0.11305561 Progenitors_6
10x_3288_t1_AACCCAGATGAGCT-1 Patient5 Progenitors nonactive 1 0.11494198

Progenitors_1
10x_3288_t1_AACCCAGATTCTTG-1 Patient5 Monocytes nonactive 8 0.01258639 Monocytes_8
10x_3288_t1_AACCGATGGCGGAA-1 Patient5 Monocytes nonactive 0 0.04733371 Monocytes_0
10x_3288_t1_AACCGATGTCGTGA-1 Patient5 Progenitors active 1 0.12554689 Progenitors_1
10x_3288_t1_AACCGATGTTTGCT-1 Patient5 Monocytes nonactive 4 0.00787838 Monocytes_4
10x_3288_t1_AACCGCCTATTCTC-1 Patient5 Progenitors active 1 0.11835014 Progenitors_1
10x_3288_t1_AACCGCCTCTAGAC-1 Patient5 Progenitors nonactive 1 0.09815484 Progenitors_1
10x_3288_t1_AACCGCCTGGTGAG-1 Patient5 Progenitors nonactive 6 0.11120094 Progenitors_6
10x_3288_t1_AACCGCCTTTTGCT-1 Patient5 Monocytes nonactive 4 0.0029643 Monocytes_4
10x_3288_t1_AACCTACTCATCAG-1 Patient5 Monocytes nonactive 0 0.02877116 Monocytes_0
10x_3288_t1_AACCTACTGCTATG-1 Patient5 Progenitors active 1 0.17949084 Progenitors_1
10x_3288_t1_AACCTACTTGGATC-1 Patient5 Monocytes nonactive 0 0.08214444 Monocytes_0
10x_3288_t1_AACCTACTTGTCCC-1 Patient5 Monocytes nonactive 4 0.03901148 Monocytes_4
10x_3288_t1_AACCTTACCACAAC-1 Patient5 Monocytes nonactive 0 0.03715681 Monocytes_0
10x_3288_t1_AACCTTACGCTATG-1 Patient5 Monocytes nonactive 0 0.08293704 Monocytes_0
10x_3288_t1_AACCTTACTGGTTG-1 Patient5 Monocytes nonactive 4 0.02613975 Monocytes_4
10x_3288_t1_AACCTTTGCAATCG-1 Patient5 Monocytes nonactive 0 0.06534145 Monocytes_0

10x_3288_t1_AACCTTTGTCCTCG-1	Patient5	Monocytes	nonactive	0	0.07995688 Monocytes_0
10x_3288_t1_AACGCAACAGGCGA-1	Patient5	Progenitors	active	1	0.15078308 Progenitors_1
10x_3288_t1_AACGCATGCTTGAG-1	Patient5	Monocytes	nonactive	8	0.03568258 Monocytes_8
10x_3288_t1_AACGCATGGGTAAA-1	Patient5	Monocytes	nonactive	0	0.0058652 Monocytes_0
10x_3288_t1_AACGCATGTGTTTC-1	Patient5	Monocytes	nonactive	0	0.06916175 Monocytes_0
10x_3288_t1_AACGCCCTGTGCTA-1	Patient5	Monocytes	nonactive	4	0.02824805 Monocytes_4
10x_3288_t1_AACGCCCTTCGTTT-1	Patient5	Monocytes	nonactive	4	0.04654112 Monocytes_4
10x_3288_t1_AACGGTACAAGTAG-1	Patient5	Monocytes	nonactive	8	0.04955298 Monocytes_8
10x_3288_t1_AACGGTACCCCTCA-1	Patient5	Monocytes	nonactive	8	0.11123264 Monocytes_8
10x_3288_t1_AACGGTACCTAGAC-1	Patient5	Monocytes	nonactive	4	0.06862279 Monocytes_4
10x_3288_t1_AACGTCGATCTTCA-1	Patient5	Monocytes	nonactive	4	0.07548665 Monocytes_4
10x_3288_t1_AACGTCGATGGTCA-1	Patient5	Monocytes	nonactive	0	0.0273762 Monocytes_0
10x_3288_t1_AACGTGTGAGTTCG-1	Patient5	Monocytes	nonactive	0	0.03041976 Monocytes_0
10x_3288_t1_AACGTGTGCACACA-1	Patient5	Progenitors	active	1	0.16180014 Progenitors_1
10x_3288_t1_AACGTGTGCCAATG-1	Patient5	Progenitors	active	1	0.1277186 Progenitors_1
10x_3288_t1_AACGTGTGGTAAAG-1	Patient5	Monocytes	nonactive	4	0.05086868 Monocytes_4
10x_3288_t1_AACGTGTGTGACAC-1	Patient5	Progenitors	active	1	0.122646

Progenitors_1
10x_3288_t1_AACGTTCTCAGGAG-1 Patient5 Progenitors active 1 0.21135312
Progenitors_1
10x_3288_t1_AACGTTCTCCTCAC-1 Patient5 Progenitors active 1 0.13810158
Progenitors_1
10x_3288_t1_AACGTTCTCTGGTA-1 Patient5 Progenitors active 1 0.15626783
Progenitors_1
10x_3288_t1_AACGTTCTGAATGA-1 Patient5 Monocytes nonactive 4 0.05571936
Monocytes_4
10x_3288_t1_AACGTTCTGACAAA-1 Patient5 Progenitors active 1 0.12147296
Progenitors_1
10x_3288_t1_AACGTTCTGGAACG-1 Patient5 Monocytes nonactive 0 0.06237715
Monocytes_0
10x_3288_t1_AACGTTCTGGCAAG-1 Patient5 Monocytes nonactive 0 0.0501712
Monocytes_0
10x_3288_t1_AACGTTCTTTCCCG-1 Patient5 Monocytes nonactive 8 0.08794623
Monocytes_8
10x_3288_t1_AACTACCTGCTAAC-1 Patient5 Monocytes nonactive 4 0.00079259
Monocytes_4
10x_3288_t1_AACTCACTACCAGT-1 Patient5 Progenitors nonactive 1 0.08238222
Progenitors_1
10x_3288_t1_AACTCACTGGCAAG-1 Patient5 Monocytes nonactive 4 0.05150276
Monocytes_4
10x_3288_t1_AACTCACTTAACCG-1 Patient5 Monocytes nonactive 4 0.03127576
Monocytes_4
10x_3288_t1_AACTCACTTAAGCC-1 Patient5 Progenitors nonactive 1 0.10671486
Progenitors_1
10x_3288_t1_AACTCGGAAACGTC-1 Patient5 Monocytes nonactive 15 0.04877623
Monocytes_15
10x_3288_t1_AACTCGGACAACCA-1 Patient5 Monocytes nonactive 4 0.06481834
Monocytes_4
10x_3288_t1_AACTCGGACCTGAA-1 Patient5 Monocytes nonactive 13 0.03240124
Monocytes_13

10x_3288_t1_AACTCTTGAATCGC-1 Patient5 Progenitors active 1 0.16129288
Progenitors_1
10x_3288_t1_AACTCTTGACCTGA-1 Patient5 Monocytes nonactive 0 0.04455963
Monocytes_0
10x_3288_t1_AACTCTTGATGTCG-1 Patient5 Monocytes nonactive 4 0.02249382
Monocytes_4
10x_3288_t1_AACTCTTGCATTCT-1 Patient5 Monocytes nonactive 0 0.04468645
Monocytes_0
10x_3288_t1_AACTGTCTAGTGCT-1 Patient5 Progenitors active 1 0.15499968
Progenitors_1
10x_3288_t1_AACTGTCTATGGTC-1 Patient5 Monocytes nonactive 0 0.04392556
Monocytes_0
10x_3288_t1_AACTGTCTCTGGTA-1 Patient5 Progenitors active 1 0.17094667
Progenitors_1
10x_3288_t1_AACTGTCTTGAAGA-1 Patient5 Progenitors active 1 0.14347537
Progenitors_1
10x_3288_t1_AACTTGCTATTGGC-1 Patient5 Progenitors active 1 0.15162323
Progenitors_1
10x_3288_t1_AACTTGCTTAGAAG-1 Patient5 Monocytes nonactive 0 0.0518832
Monocytes_0
10x_3288_t1_AAGAACGAAACTGC-1 Patient5 Monocytes nonactive 0 0.05703506
Monocytes_0
10x_3288_t1_AAGAACGACGAGTT-1 Patient5 Monocytes nonactive 0 0.0800837
Monocytes_0
10x_3288_t1_AAGAACGATCTACT-1 Patient5 Progenitors nonactive 1 0.11609917
Progenitors_1
10x_3288_t1_AAGAAGACAACCAC-1 Patient5 Monocytes nonactive 0 0.03821888
Monocytes_0
10x_3288_t1_AAGAAGACCTCGCT-1 Patient5 Monocytes nonactive 8 0.08357111
Monocytes_8
10x_3288_t1_AAGAAGACTAAAGG-1 Patient5 Progenitors active 1 0.17372075
Progenitors_1
10x_3288_t1_AAGAAGACTACAGC-1 Patient5 Progenitors nonactive 1 0.10501871

Progenitors_1
10x_3288_t1_AAGAAGACTCTATC-1 Patient5 Progenitors active 1 0.15152812
Progenitors_1
10x_3288_t1_AAGAATCTACCTAG-1 Patient5 Monocytes nonactive 4 0.07035064
Monocytes_4
10x_3288_t1_AAGAATCTGAGACG-1 Patient5 Monocytes nonactive 0 0.0311965
Monocytes_0
10x_3288_t1_AAGAATCTGTTACG-1 Patient5 Monocytes nonactive 2 0.05781181
Monocytes_2
10x_3288_t1_AAGACAGAATTCGG-1 Patient5 Monocytes nonactive 4 0.06759242
Monocytes_4
10x_3288_t1_AAGACAGACACTGA-1 Patient5 Monocytes nonactive 0 0.09542832
Monocytes_0
10x_3288_t1_AAGACAGACTTGGA-1 Patient5 Monocytes nonactive 0 0.07139687
Monocytes_0
10x_3288_t1_AAGACAGAGAATGA-1 Patient5 Monocytes nonactive 0 0.04907742
Monocytes_0
10x_3288_t1_AAGACAGATTTACC-1 Patient5 Monocytes nonactive 0 0.04679475
Monocytes_0
10x_3288_t1_AAGAGATGAAGTAG-1 Patient5 Monocytes nonactive 4 0.00117304
Monocytes_4
10x_3288_t1_AAGAGATGCTAGTG-1 Patient5 Monocytes nonactive 4 0.02206582
Monocytes_4
10x_3288_t1_AAGATGGATAAGCC-1 Patient5 Progenitors nonactive 1 0.09997781
Progenitors_1
10x_3288_t1_AAGATGGATGCACA-1 Patient5 Monocytes nonactive 4 0.06803627
Monocytes_4
10x_3288_t1_AAGATGGATGTGAC-1 Patient5 Monocytes nonactive 0 0.03685562
Monocytes_0
10x_3288_t1_AAGATGGATTGCGA-1 Patient5 Monocytes nonactive 8 0.03241709
Monocytes_8
10x_3288_t1_AAGATTACCACTCC-1 Patient5 Progenitors active 1 0.15691776
Progenitors_1

10x_3288_t1_AAGATTACTGTTCT-1 Patient5 Monocytes nonactive 0 0.03731533
Monocytes_0
10x_3288_t1_AAGCAAGAAGGAGC-1 Patient5 Monocytes nonactive 0 0.04584364
Monocytes_0
10x_3288_t1_AAGCACTGACGGGA-1 Patient5 Progenitors active 1 0.16522415
Progenitors_1
10x_3288_t1_AAGCACTGATCGTG-1 Patient5 Progenitors active 1 0.14014647
Progenitors_1
10x_3288_t1_AAGCACTGCTCAAG-1 Patient5 Monocytes nonactive 4 0.03484243
Monocytes_4
10x_3288_t1_AAGCACTGTCATTC-1 Patient5 Monocytes nonactive 4 0.01704077
Monocytes_4
10x_3288_t1_AAGCACTGTGTAGC-1 Patient5 Monocytes nonactive 0 0.05453047
Monocytes_0
10x_3288_t1_AAGCCAACCGCCTT-1 Patient5 Monocytes nonactive 0 0.07673895
Monocytes_0
10x_3288_t1_AAGCCAACTGCCAA-1 Patient5 Monocytes nonactive 4 0.05373787
Monocytes_4
10x_3288_t1_AAGCCATGACACGT-1 Patient5 Progenitors active 1 0.14864308
Progenitors_1
10x_3288_t1_AAGCCATGAGCCAT-1 Patient5 Progenitors active 1 0.16021495
Progenitors_1
10x_3288_t1_AAGCCATGCTTGAG-1 Patient5 Progenitors active 5 0.13171327
Progenitors_5
10x_3288_t1_AAGCCATGGTACCA-1 Patient5 Progenitors nonactive 1 0.10272018
Progenitors_1
10x_3288_t1_AAGCCTGACTGTAG-1 Patient5 Progenitors active 1 0.13424957
Progenitors_1
10x_3288_t1_AAGCCTGAGCTAAC-1 Patient5 Monocytes nonactive 4 0.05053579
Monocytes_4
10x_3288_t1_AAGCGACTCTTTAC-1 Patient5 Monocytes nonactive 4 0.05050409
Monocytes_4
10x_3288_t1_AAGCGTACTAGACC-1 Patient5 Monocytes nonactive 4 0.0438463

Monocytes_4
10x_3288_t1_AAGGCTACAAGATG-1 Patient5 Monocytes nonactive 0 0.04587534 Monocytes_0
10x_3288_t1_AAGGCTACCAGTTG-1 Patient5 Monocytes nonactive 4 0.0404857 Monocytes_4
10x_3288_t1_AAGGCTACTAACCG-1 Patient5 Progenitors active 1 0.14767611 Progenitors_1
10x_3288_t1_AAGGCTACTCTTCA-1 Patient5 Monocytes nonactive 4 0.05690825 Monocytes_4
10x_3288_t1_AAGGCTTGGTACGT-1 Patient5 Progenitors nonactive 1 0.10053262 Progenitors_1
10x_3288_t1_AAGGTCACCGAGTT-1 Patient5 Progenitors active 1 0.16928223 Progenitors_1
10x_3288_t1_AAGGTCACGTCGAT-1 Patient5 Monocytes nonactive 4 0.05584617 Monocytes_4
10x_3288_t1_AAGGTCTGGTTGTG-1 Patient5 Progenitors active 1 0.14959419 Progenitors_1
10x_3288_t1_AAGGTCTGTATCTC-1 Patient5 Progenitors active 1 0.14520322 Progenitors_1
10x_3288_t1_AAGGTCTGTGGTCA-1 Patient5 Monocytes nonactive 8 0.00112548 Monocytes_8
10x_3288_t1_AAGGTCTGTTGCTT-1 Patient5 Monocytes nonactive 0 0.0433866 Monocytes_0
10x_3288_t1_AAGGTGCTCAGCTA-1 Patient5 Progenitors active 1 0.1576311 Progenitors_1
10x_3288_t1_AAGGTGCTTTGACG-1 Patient5 Monocytes nonactive 4 0.05655951 Monocytes_4
10x_3288_t1_AAGTAACTAACGAA-1 Patient5 Monocytes nonactive 0 0.04785683 Monocytes_0
10x_3288_t1_AAGTAACTACGGAG-1 Patient5 Monocytes nonactive 0 0.03883711 Monocytes_0
10x_3288_t1_AAGTAACTAGGGTG-1 Patient5 Progenitors nonactive 1 0.09519054 Progenitors_1

10x_3288_t1_AAGTAACTGCGTTA-1 Patient5 Progenitors active 1 0.16885423 Progenitors_1
10x_3288_t1_AAGTAACTTATCTC-1Patient5 Progenitors nonactive 1 0.11242153 Progenitors_1
10x_3288_t1_AAGTAACTTATGCG-1 Patient5 Progenitors active 1 0.14596411 Progenitors_1
10x_3288_t1_AAGTAGGAACCTAG-1 Patient5 Progenitors active 1 0.15360472 Progenitors_1
10x_3288_t1_AAGTAGGATCCTGC-1 Patient5 Progenitors active 1 0.1450447 Progenitors_1
10x_3288_t1_AAGTCCGAAGGGTG-1 Patient5 Monocytes nonactive 4 0.06477078 Monocytes_4
10x_3288_t1_AAGTCCGACTTAGG-1 Patient5 Monocytes nonactive 0 0.05506943 Monocytes_0
10x_3288_t1_AAGTCCGAGCTTCC-1 Patient5 Monocytes nonactive 0 0.07280768 Monocytes_0
10x_3288_t1_AAGTCCGATCGTTT-1 Patient5 Monocytes nonactive 0 0.019799 Monocytes_0
10x_3288_t1_AAGTCTCTGTCTAG-1 Patient5 Monocytes nonactive 0 0.05503773 Monocytes_0
10x_3288_t1_AAGTCTCTTGAACC-1 Patient5 Progenitors active 1 0.13122186 Progenitors_1
10x_3288_t1_AAGTCTCTTGCCCT-1 Patient5 Monocytes nonactive 4 0.03612643 Monocytes_4
10x_3288_t1_AAGTGCACAATGCC-1 Patient5 Progenitors active 1 0.1193171 Progenitors_1
10x_3288_t1_AAGTGCACCCAACA-1 Patient5 Monocytes nonactive 4 0.07841925 Monocytes_4
10x_3288_t1_AAGTGCACCGCCTT-1 Patient5 Monocytes nonactive 4 0.05104305 Monocytes_4
10x_3288_t1_AAGTGCACGGCATT-1 Patient5 Progenitors active 1 0.12324837 Progenitors_1
10x_3288_t1_AAGTGGCTAGCTAC-1 Patient5 Monocytes nonactive 0 0.04020037

Monocytes_0
10x_3288_t1_AAGTGGCTCGGTAT-1 Patient5 Monocytes nonactive 0 0.05015535
Monocytes_0
10x_3288_t1_AAGTGGCTGGAGGT-1 Patient5 Progenitors nonactive 1 0.07954473
Progenitors_1
10x_3288_t1_AAGTGGCTTGCCCT-1 Patient5 Progenitors nonactive 1 0.11663813
Progenitors_1
10x_3288_t1_AAGTGGCTTGTGGT-1 Patient5 Progenitors active 1 0.16325851
Progenitors_1
10x_3288_t1_AAGTTATGAACCTG-1 Patient5 Monocytes nonactive 0 0.02507767
Monocytes_0
10x_3288_t1_AAGTTATGCCGTTC-1 Patient5 Progenitors active 1 0.13800647
Progenitors_1
10x_3288_t1_AAGTTATGGCAGTT-1 Patient5 Monocytes nonactive 0 0.02799442
Monocytes_0
10x_3288_t1_AAGTTCCTATCTCT-1 Patient5 Progenitors active 1 0.14670915
Progenitors_1
10x_3288_t1_AAGTTCCTGAACTC-1 Patient5 Monocytes nonactive 4 0.05285017
Monocytes_4
10x_3288_t1_AATAACACATGCCA-1 Patient5 Progenitors active 1 0.17368905
Progenitors_1
10x_3288_t1_AATAACACATGGTC-1 Patient5 Monocytes nonactive 4 0.04581193
Monocytes_4
10x_3288_t1_AATAACACGACGGA-1 Patient5 Progenitors active 1 0.17468772
Progenitors_1
10x_3288_t1_AATAACACGTACGT-1 Patient5 Monocytes nonactive 0 0.05126498
Monocytes_0
10x_3288_t1_AATAAGCTGTCCTC-1 Patient5 Monocytes nonactive 0 0.06128337
Monocytes_0
10x_3288_t1_AATAAGCTTATCGG-1 Patient5 Monocytes nonactive 4 0.11032909
Monocytes_4
10x_3288_t1_AATACCCTAACGTC-1 Patient5 Monocytes nonactive 4 0.05201002
Monocytes_4

10x_3288_t1_AATACCCTACCTGA-1 Patient5 Monocytes nonactive 0 0.01169869
Monocytes_0
10x_3288_t1_AATACCCTCGTGAT-1 Patient5 Monocytes nonactive 0 0.04348171
Monocytes_0
10x_3288_t1_AATACCCTGACTAC-1 Patient5 Monocytes nonactive 4 0.02851753
Monocytes_4
10x_3288_t1_AATACCCTGCCTTC-1 Patient5 Monocytes nonactive 0 0.04359267
Monocytes_0
10x_3288_t1_AATACCCTGTACGT-1 Patient5 Progenitors active 1 0.13711876
Progenitors_1
10x_3288_t1_AATACTGAACGCTA-1 Patient5 Progenitors active 1 0.15981865
Progenitors_1
10x_3288_t1_AATACTGAAGTCGT-1 Patient5 Monocytes nonactive 13 0.02212922
Monocytes_13
10x_3288_t1_AATACTGACCACAA-1 Patient5 Monocytes nonactive 4 0.08859616
Monocytes_4
10x_3288_t1_AATACTGACTCAAG-1 Patient5 Progenitors nonactive 5 0.0939858
Progenitors_5
10x_3288_t1_AATACTGAGTAGCT-1 Patient5 Monocytes nonactive 8 0.04270496
Monocytes_8
10x_3288_t1_AATACTGAGTTCGA-1 Patient5 Progenitors active 1 0.15634709
Progenitors_1
10x_3288_t1_AATAGGGACCCACT-1 Patient5 Monocytes nonactive 13 0.0522795
Monocytes_13
10x_3288_t1_AATATCGATGTCGA-1 Patient5 Monocytes nonactive 0 0.05819225
Monocytes_0
10x_3288_t1_AATCAAACACTGTG-1 Patient5 Monocytes nonactive 0 0.07642191
Monocytes_0
10x_3288_t1_AATCAAACTTCTTG-1 Patient5 Progenitors active 1 0.16887008
Progenitors_1
10x_3288_t1_AATCCGGAATTTCC-1 Patient5 Monocytes nonactive 4 0.04535223
Monocytes_4
10x_3288_t1_AATCCGGACGACAT-1 Patient5 Monocytes nonactive 8 0.0923689

Monocytes_8
10x_3288_t1_AATCCGGACGGGAA-1 Patient5 Monocytes nonactive 0 0.05150276
Monocytes_0
10x_3288_t1_AATCCTACGCTACA-1 Patient5 Progenitors nonactive 1 0.11115338
Progenitors_1
10x_3288_t1_AATCCTACGGTATC-1 Patient5 Monocytes nonactive 0 0.06765582
Monocytes_0
10x_3288_t1_AATCCTACGTCTAG-1 Patient5 Progenitors active 1 0.16195866
Progenitors_1
10x_3288_t1_AATCCTACTTCTCA-1 Patient5 Monocytes nonactive 4 0.02287426
Monocytes_4
10x_3288_t1_AATCCTTGACACAC-1 Patient5 Monocytes nonactive 0 0.04468645
Monocytes_0
10x_3288_t1_AATCCTTGCCTAAG-1 Patient5 Progenitors active 1 0.14059032
Progenitors_1
10x_3288_t1_AATCCTTGCTAAGC-1 Patient5 Monocytes nonactive 0 0.04576438
Monocytes_0
10x_3288_t1_AATCCTTGGACGTT-1 Patient5 Monocytes nonactive 0 0.05329402
Monocytes_0
10x_3288_t1_AATCCTTGTAGCCA-1 Patient5 Monocytes nonactive 4 0.02094033
Monocytes_4
10x_3288_t1_AATCGGTGAAACGA-1 Patient5 Monocytes nonactive 13 0.05197831
Monocytes_13
10x_3288_t1_AATCGGTGAAGGTA-1 Patient5 Progenitors nonactive 1 0.11460909
Progenitors_1
10x_3288_t1_AATCGGTGCTATTC-1 Patient5 Dendritic cells active 10 0.12863801
Dendritic cells_10
10x_3288_t1_AATCGGTGTATTCC-1 Patient5 Monocytes nonactive 13 0.02212922
Monocytes_13
10x_3288_t1_AATCGGTGTCCAGA-1 Patient5 Monocytes nonactive 8 0.07680236
Monocytes_8
10x_3288_t1_AATCGGTGTCGACA-1 Patient5 Progenitors active 1 0.15018071
Progenitors_1

10x_3288_t1_AATCGGTGTGTCAG-1 Patient5 Monocytes nonactive 0 0.0286919 Monocytes_0
10x_3288_t1_AATCTAGAACTACG-1 Patient5 Monocytes nonactive 4 0.05971403 Monocytes_4
10x_3288_t1_AATCTAGAAGATGA-1 Patient5 Monocytes nonactive 8 0.01109632 Monocytes_8
10x_3288_t1_AATCTAGAAGGTCT-1 Patient5 Monocytes nonactive 0 0.05846173 Monocytes_0
10x_3288_t1_AATCTAGACCCTAC-1 Patient5 Monocytes nonactive 0 0.05083698 Monocytes_0
10x_3288_t1_AATCTAGATCTACT-1 Patient5 Monocytes nonactive 8 0.08406252 Monocytes_8
10x_3288_t1_AATCTCACAGTAGA-1 Patient5 Monocytes nonactive 0 0.02690064 Monocytes_0
10x_3288_t1_AATCTCACCTTGTT-1 Patient5 Progenitors active 1 0.11985606 Progenitors_1
10x_3288_t1_AATCTCACGGAGCA-1 Patient5 Monocytes nonactive 0 0.05984085 Monocytes_0
10x_3288_t1_AATCTCACTATCGG-1 Patient5 Monocytes nonactive 0 0.05307209 Monocytes_0
10x_3288_t1_AATCTCTGAGTCAC-1 Patient5 Monocytes nonactive 8 0.0497115 Monocytes_8
10x_3288_t1_AATCTCTGATTGGC-1 Patient5 Progenitors active 1 0.13640543 Progenitors_1
10x_3288_t1_AATCTCTGGTCAAC-1 Patient5 Progenitors active 1 0.14731152 Progenitors_1
10x_3288_t1_AATGAGGAAAGTAG-1 Patient5 Progenitors active 1 0.16852134 Progenitors_1
10x_3288_t1_AATGAGGAACCTAG-1 Patient5 Monocytes nonactive 0 0.02728109 Monocytes_0
10x_3288_t1_AATGAGGACTACTT-1 Patient5 Monocytes nonactive 4 0.08718534 Monocytes_4
10x_3288_t1_AATGATACCCCTTG-1 Patient5 Monocytes nonactive 4 0.04525712

Monocytes_4
10x_3288_t1_AATGATACCTCCAC-1 Patient5 Monocytes nonactive 4 0.05912751
Monocytes_4
10x_3288_t1_AATGATACCTTCCG-1 Patient5 Monocytes nonactive 4 0.05126498
Monocytes_4
10x_3288_t1_AATGATACGAAAGT-1 Patient5 Progenitors active 1 0.16630207
Progenitors_1
10x_3288_t1_AATGATACTTCGGA-1 Patient5 Monocytes nonactive 0 0.04463889
Monocytes_0
10x_3288_t1_AATGCGTGCAGTTG-1 Patient5 Monocytes nonactive 4 0.0400894
Monocytes_4
10x_3288_t1_AATGCGTGCCCGTT-1 Patient5 Monocytes nonactive 0 0.07226872
Monocytes_0
10x_3288_t1_AATGCGTGGAAGGC-1 Patient5 Monocytes nonactive 4 0.03844081
Monocytes_4
10x_3288_t1_AATGCGTGGACGTT-1 Patient5 Monocytes nonactive 0 0.06599138
Monocytes_0
10x_3288_t1_AATGCGTGGGGCAA-1 Patient5 Monocytes nonactive 4 0.04834823
Monocytes_4
10x_3288_t1_AATGGAGAAGAAGT-1 Patient5 Progenitors active 1 0.13935388
Progenitors_1
10x_3288_t1_AATGGAGAGCGTTA-1 Patient5 Monocytes nonactive 0 0.01905396
Monocytes_0
10x_3288_t1_AATGGAGATCTCTA-1 Patient5 Progenitors active 1 0.14003551
Progenitors_1
10x_3288_t1_AATGTAACACGCAT-1 Patient5 Monocytes nonactive 4 0.0463509
Monocytes_4
10x_3288_t1_AATGTAACCCTGAA-1 Patient5 Progenitors active 1 0.1210291
Progenitors_1
10x_3288_t1_AATGTAACCGTGAT-1 Patient5 Monocytes nonactive 0 0.07301376
Monocytes_0
10x_3288_t1_AATGTAACGGGAGT-1 Patient5 Progenitors nonactive 5 0.05858855
Progenitors_5

10x_3288_t1_AATGTCCTACTGGT-1 Patient5 Monocytes nonactive 4 0.0662133
Monocytes_4
10x_3288_t1_AATGTCCTGTTCTT-1 Patient5 Monocytes nonactive 0 0.03639592
Monocytes_0
10x_3288_t1_AATGTCCTTCGTGA-1 Patient5 Progenitors active 6 0.14983197
Progenitors_6
10x_3288_t1_AATGTTGAACCATG-1 Patient5 Monocytes nonactive 15 0.04950542
Monocytes_15
10x_3288_t1_AATGTTGAATCTTC-1 Patient5 Progenitors active 1 0.13169742
Progenitors_1
10x_3288_t1_AATGTTGAGCCCTT-1 Patient5 Monocytes nonactive 0 0.03598377
Monocytes_0
10x_3288_t1_AATGTTGAGTAGCT-1 Patient5 Monocytes nonactive 8 0.04205504
Monocytes_8
10x_3288_t1_AATGTTGATGATGC-1 Patient5 Monocytes nonactive 4 0.0341608
Monocytes_4
10x_3288_t1_AATTACGAACGACT-1 Patient5 Monocytes nonactive 0 0.0308002
Monocytes_0
10x_3288_t1_AATTACGACCCTTG-1 Patient5 Monocytes nonactive 4 0.00789424
Monocytes_4
10x_3288_t1_AATTACGACGTAGT-1 Patient5 Progenitors nonactive 5 0.05720944
Progenitors_5
10x_3288_t1_AATTACGAGAGGAC-1 Patient5 Monocytes nonactive 0 0.06283685
Monocytes_0
10x_3288_t1_AATTACGATCTCAT-1 Patient5 Monocytes nonactive 0 0.01103291
Monocytes_0
10x_3288_t1_AATTCCTGACCAAC-1 Patient5 Monocytes nonactive 0 0.0809714
Monocytes_0
10x_3288_t1_AATTCCTGTCGATG-1 Patient5 Progenitors active 1 0.14634456
Progenitors_1
10x_3288_t1_AATTGATGATTCTC-1 Patient5 Progenitors active 1 0.17768372
Progenitors_1
10x_3288_t1_AATTGATGGTCGAT-1 Patient5 Monocytes nonactive 0 0.06504026

Monocytes_0
10x_3288_t1_AATTGATGTCACCC-1 Patient5 Progenitors active 1 0.14206455
Progenitors_1
10x_3288_t1_AATTGATGTGCAGT-1 Patient5 Monocytes nonactive 4 0.03270243
Monocytes_4
10x_3288_t1_AATTGATGTGTGGT-1Patient5 Monocytes nonactive 8 0.07467821
Monocytes_8
10x_3288_t1_AATTGTGATAGACC-1 Patient5 Monocytes nonactive 0 0.04111978
Monocytes_0
10x_3288_t1_AATTGTGATCTGGA-1 Patient5 Progenitors active 1 0.21466616
Progenitors_1
10x_3288_t1_ACAAAGGAGGACTT-1 Patient5 Progenitors active 1 0.12324837
Progenitors_1
10x_3288_t1_ACAAAGGATTGCTT-1 Patient5 Progenitors active 1 0.13835521
Progenitors_1
10x_3288_t1_ACAAATTGCATTCT-1Patient5 Progenitors active 1 0.15536428
Progenitors_1
10x_3288_t1_ACAACCGAATTTCC-1 Patient5 Monocytes nonactive 4 0.05403906
Monocytes_4
10x_3288_t1_ACAACCGATCTAGG-1 Patient5 Monocytes nonactive 0 0.03067339
Monocytes_0
10x_3288_t1_ACAACCGATTGCGA-1 Patient5 Progenitors active 1 0.13878321
Progenitors_1
10x_3288_t1_ACAAGAGAAGAGAT-1 Patient5 Progenitors nonactive 1 0.05781181
Progenitors_1
10x_3288_t1_ACAAGAGACATACG-1 Patient5 Progenitors active 1 0.13090483
Progenitors_1
10x_3288_t1_ACAAGCACAGTCTG-1 Patient5 Monocytes nonactive 0 0.02144759
Monocytes_0
10x_3288_t1_ACAAGCACATGTGC-1 Patient5 Monocytes nonactive 0 0.07686577
Monocytes_0
10x_3288_t1_ACAAGCACGCCCTT-1 Patient5 Monocytes nonactive 0 0.06567434
Monocytes_0

10x_3288_t1_ACAATAACCCCGTT-1 Patient5 Progenitors active 1 0.14448989 Progenitors_1
10x_3288_t1_ACAATAACGGCATT-1 Patient5 Progenitors active 1 0.1336155 Progenitors_1
10x_3288_t1_ACAATAACTCCGAA-1 Patient5 Monocytes nonactive 4 0.03863103 Monocytes_4
10x_3288_t1_ACAATAACTCTCAT-1 Patient5 Monocytes nonactive 4 0.01742122 Monocytes_4
10x_3288_t1_ACAATCCTGTCGTA-1 Patient5 Progenitors nonactive 1 0.11441887 Progenitors_1
10x_3288_t1_ACAATCCTTGCCTC-1 Patient5 Progenitors active 1 0.18129795 Progenitors_1
10x_3288_t1_ACAATCCTTGGTTG-1 Patient5 Monocytes nonactive 4 0.03005516 Monocytes_4
10x_3288_t1_ACAATTGAAGCGTT-1 Patient5 Monocytes nonactive 0 0.05419758 Monocytes_0
10x_3288_t1_ACAATTGAGCCCTT-1 Patient5 Monocytes nonactive 8 0.08905586 Monocytes_8
10x_3288_t1_ACACAGACCCGTAA-1 Patient5 Progenitors active 1 0.13759432 Progenitors_1
10x_3288_t1_ACACAGACCGATAC-1 Patient5 Monocytes nonactive 4 0.03978822 Monocytes_4
10x_3288_t1_ACACAGACCTGCAA-1 Patient5 Monocytes nonactive 4 0.05369032 Monocytes_4
10x_3288_t1_ACACAGACCTTGGA-1 Patient5 Monocytes nonactive 8 0.0914495 Monocytes_8
10x_3288_t1_ACACATCTCTCGCT-1 Patient5 Progenitors active 1 0.15306575 Progenitors_1
10x_3288_t1_ACACATCTTGCATG-1 Patient5 Monocytes nonactive 0 0.0345571 Monocytes_0
10x_3288_t1_ACACCAGAAGAGGC-1 Patient5 Progenitors nonactive 1 0.10405174 Progenitors_1
10x_3288_t1_ACACCAGAAGATGA-1 Patient5 Monocytes nonactive 4 0.06572189

Monocytes_4

10x_3288_t1_ACACCAGACGGGAA-1 Patient5 Progenitors nonactive 1 0.11361042 Progenitors_1

10x_3288_t1_ACACCAGAGGTATC-1 Patient5 Progenitors active 1 0.16725319 Progenitors_1

10x_3288_t1_ACACCAGAGTGAGG-1 Patient5 Monocytes nonactive 4 0.03157695 Monocytes_4

10x_3288_t1_ACACCAGATCTCTA-1 Patient5 Monocytes nonactive 0 0.06521463 Monocytes_0

10x_3288_t1_ACACCCTGGCTATG-1 Patient5 Monocytes nonactive 0 0.08273096 Monocytes_0

10x_3288_t1_ACACCCTGTAAGCC-1 Patient5 Monocytes nonactive 4 0.03462051 Monocytes_4

10x_3288_t1_ACACCCTGTCTAGG-1 Patient5 Monocytes nonactive 4 0.04960053 Monocytes_4

10x_3288_t1_ACACCCTGTCTTTG-1 Patient5 Monocytes nonactive 4 0.01789677 Monocytes_4

10x_3288_t1_ACACGAACATGTCG-1 Patient5 Progenitors active 1 0.17275379 Progenitors_1

10x_3288_t1_ACACGAACGACACT-1 Patient5 Monocytes nonactive 4 0.07120665 Monocytes_4

10x_3288_t1_ACACGATGATGCTG-1 Patient5 Dendritic cells nonactive 10 0.0813994 Dendritic cells_10

10x_3288_t1_ACACGATGTATCTC-1 Patient5 Monocytes nonactive 4 0.05587788 Monocytes_4

10x_3288_t1_ACACGTGAATAAGG-1 Patient5 Monocytes nonactive 0 0.04557416 Monocytes_0

10x_3288_t1_ACACGTGAGTCGAT-1 Patient5 Monocytes nonactive 4 0.04050155 Monocytes_4

10x_3288_t1_ACACGTGATCTATC-1 Patient5 Monocytes nonactive 4 0.0489189 Monocytes_4

10x_3288_t1_ACAGACACCCAGTA-1 Patient5 Monocytes nonactive 0 0.05877877 Monocytes_0

10x_3288_t1_ACAGACACCCTGAA-1 Patient5 Monocytes nonactive 0 0.10685752 Monocytes_0
10x_3288_t1_ACAGACACCTCAAG-1 Patient5 Monocytes nonactive 4 0.0307368 Monocytes_4
10x_3288_t1_ACAGACACGAGGCA-1 Patient5 Progenitors nonactive 1 0.10298966 Progenitors_1
10x_3288_t1_ACAGCAACGCGTTA-1 Patient5 Progenitors active 1 0.12369222 Progenitors_1
10x_3288_t1_ACAGGTACAAGTAG-1 Patient5 Progenitors active 1 0.17078816 Progenitors_1
10x_3288_t1_ACAGGTACGTGTCA-1 Patient5 Monocytes nonactive 4 0.08502948 Monocytes_4
10x_3288_t1_ACAGGTACTACGCA-1 Patient5 Monocytes nonactive 0 0.04249889 Monocytes_0
10x_3288_t1_ACAGGTACTATGCG-1 Patient5 Monocytes nonactive 4 0.01282417 Monocytes_4
10x_3288_t1_ACAGGTACTCCTCG-1 Patient5 Monocytes nonactive 0 0.05053579 Monocytes_0
10x_3288_t1_ACAGGTACTTACTC-1 Patient5 Monocytes nonactive 4 0.02342908 Monocytes_4
10x_3288_t1_ACAGGTACTTCATC-1 Patient5 Progenitors active 1 0.15209879 Progenitors_1
10x_3288_t1_ACAGTCGACATTCT-1 Patient5 Monocytes nonactive 0 0.0556718 Monocytes_0
10x_3288_t1_ACAGTCGACCTTGC-1 Patient5 Progenitors active 1 0.15029167 Progenitors_1
10x_3288_t1_ACAGTCGAGCTTAG-1 Patient5 Monocytes nonactive 0 0.02395219 Monocytes_0
10x_3288_t1_ACAGTGACCAGTCA-1 Patient5 Progenitors active 1 0.18541944 Progenitors_1
10x_3288_t1_ACAGTGACGGGAGT-1 Patient5 Monocytes nonactive 4 0.03389132 Monocytes_4
10x_3288_t1_ACAGTGACTGTCGA-1 Patient5 Monocytes nonactive 4 0.02896138

Monocytes_4
10x_3288_t1_ACAGTGACTTCTTG-1 Patient5 Monocytes nonactive 4 0.02371441
Monocytes_4
10x_3288_t1_ACAGTGTGGGTGGA-1 Patient5 Progenitors active 6 0.13432883
Progenitors_6
10x_3288_t1_ACAGTTCTACTACG-1 Patient5 Monocytes nonactive 4 0.01990996
Monocytes_4
10x_3288_t1_ACAGTTCTATCGAC-1 Patient5 Monocytes nonactive 0 0.0518198
Monocytes_0
10x_3288_t1_ACAGTTCTTAAGGA-1 Patient5 Progenitors active 1 0.18028343
Progenitors_1
10x_3288_t1_ACAGTTCTTCCTTA-1 Patient5 Progenitors active 1 0.20719992
Progenitors_1
10x_3288_t1_ACATACCTATGTCG-1 Patient5 Monocytes nonactive 0 0.05901655
Monocytes_0
10x_3288_t1_ACATCACTCTCTTA-1 Patient5 Monocytes nonactive 0 0.01945026
Monocytes_0
10x_3288_t1_ACATCACTCTGACA-1 Patient5 Monocytes nonactive 8 0.04896646
Monocytes_8
10x_3288_t1_ACATCACTTTGAGC-1 Patient5 Monocytes nonactive 8 0.05085283
Monocytes_8
10x_3288_t1_ACATGGTGACGTGT-1 Patient5 Monocytes nonactive 0 0.03173546
Monocytes_0
10x_3288_t1_ACATGGTGAGCTCA-1 Patient5 Monocytes nonactive 4 0.02571175
Monocytes_4
10x_3288_t1_ACATGGTGATGCCA-1 Patient5 Monocytes nonactive 4 0.03295606
Monocytes_4
10x_3288_t1_ACATGGTGTGACCA-1 Patient5 Progenitors active 1 0.15374738
Progenitors_1
10x_3288_t1_ACATTCTGAGAGGC-1 Patient5 Monocytes nonactive 4 0.02003678
Monocytes_4
10x_3288_t1_ACATTCTGAGGAGC-1 Patient5 Progenitors active 1 0.13857714
Progenitors_1

10x_3288_t1_ACATTCTGGTCCTC-1 Patient5 Monocytes nonactive 0 0.05283432 Monocytes_0
10x_3288_t1_ACATTCTGTCAGGT-1 Patient5 Monocytes nonactive 4 0.03685562 Monocytes_4
10x_3288_t1_ACCAACGACCCAAA-1 Patient5 Monocytes nonactive 0 0.04283178 Monocytes_0
10x_3288_t1_ACCAACGACGTCTC-1 Patient5 Progenitors active 1 0.11752584 Progenitors_1
10x_3288_t1_ACCAACGATGTTTC-1 Patient5 Monocytes nonactive 8 0.0101135 Monocytes_8
10x_3288_t1_ACCACAGAACCCTC-1 Patient5 Progenitors nonactive 1 0.10736478 Progenitors_1
10x_3288_t1_ACCACAGAACGTTG-1 Patient5 Progenitors active 1 0.1197134 Progenitors_1
10x_3288_t1_ACCACAGACCTCAC-1 Patient5 Monocytes nonactive 0 0.03236954 Monocytes_0
10x_3288_t1_ACCACAGAGCTTCC-1 Patient5 Monocytes nonactive 0 0.06890812 Monocytes_0
10x_3288_t1_ACCACCTGGCCCTT-1 Patient5 Monocytes nonactive 4 0.03655444 Monocytes_4
10x_3288_t1_ACCACCTGTAGCCA-1 Patient5 Monocytes nonactive 0 0.02970642 Monocytes_0
10x_3288_t1_ACCACCTGTCATTC-1 Patient5 Progenitors active 1 0.17343542 Progenitors_1
10x_3288_t1_ACCACGCTAAGCCT-1 Patient5 Monocytes nonactive 0 0.05218439 Monocytes_0
10x_3288_t1_ACCACGCTACGCAT-1 Patient5 Monocytes nonactive 4 0.05261239 Monocytes_4
10x_3288_t1_ACCACGCTCATTTC-1 Patient5 Monocytes nonactive 0 0.0834443 Monocytes_0
10x_3288_t1_ACCACGCTCTGTGA-1 Patient5 Monocytes nonactive 8 0.05749477 Monocytes_8
10x_3288_t1_ACCACGCTTTCGTT-1 Patient5 Monocytes nonactive 4 0.05411832

Monocytes_4
10x_3288_t1_ACCAGCCTCCTTCG-1 Patient5 Monocytes nonactive 0 0.06191744
Monocytes_0
10x_3288_t1_ACCAGCCTTAAAGG-1 Patient5 Progenitors active 1 0.14196944
Progenitors_1
10x_3288_t1_ACCAGTGAACCTTT-1 Patient5 Monocytes nonactive 4 0.02900894
Monocytes_4
10x_3288_t1_ACCAGTGAAGTGCT-1 Patient5 Monocytes nonactive 0 0.02626657
Monocytes_0
10x_3288_t1_ACCAGTGAGCCAAT-1 Patient5 Monocytes nonactive 4 0.00603957
Monocytes_4
10x_3288_t1_ACCAGTGATCTTTG-1 Patient5 Monocytes nonactive 0 0.03216346
Monocytes_0
10x_3288_t1_ACCATTACAAAAGC-1 Patient5 Progenitors active 1 0.16089658
Progenitors_1
10x_3288_t1_ACCATTACACAGCT-1 Patient5 Monocytes nonactive 4 0.07429776
Monocytes_4
10x_3288_t1_ACCATTACACGGGA-1 Patient5 Dendritic cells nonactive 10 0.08750238
Dendritic cells_10
10x_3288_t1_ACCATTACGCCTTC-1 Patient5 Monocytes nonactive 4 0.03728362
Monocytes_4
10x_3288_t1_ACCATTACGGATCT-1 Patient5 Monocytes nonactive 4 0.03744214
Monocytes_4
10x_3288_t1_ACCATTACTGCTAG-1 Patient5 Monocytes nonactive 0 0.0594604
Monocytes_0
10x_3288_t1_ACCATTTGCGTGTA-1 Patient5 Monocytes nonactive 0 0.06087122
Monocytes_0
10x_3288_t1_ACCATTTGGTGTAC-1 Patient5 Monocytes nonactive 0 0.03687147
Monocytes_0
10x_3288_t1_ACCATTTGTCCTCG-1 Patient5 Monocytes nonactive 0 0.04663623
Monocytes_0
10x_3288_t1_ACCCAAGACGACTA-1 Patient5 Monocytes nonactive 0 0.01848329
Monocytes_0

10x_3288_t1_ACCCAAGAGAACTC-1 Patient5 Monocytes nonactive 13 0.039598
Monocytes_13
10x_3288_t1_ACCCACTGTGGAAA-1 Patient5 Progenitors active 1 0.18280388
Progenitors_1
10x_3288_t1_ACCCAGCTTATCTC-1 Patient5 Monocytes nonactive 0 0.0135058
Monocytes_0
10x_3288_t1_ACCCGTACATTTCC-1 Patient5 Monocytes nonactive 0 0.04907742
Monocytes_0
10x_3288_t1_ACCCGTACTGCGTA-1 Patient5 Monocytes nonactive 0 0.04909327
Monocytes_0
10x_3288_t1_ACCCGTACTGTCGA-1 Patient5 Dendritic cells nonactive 10 0.09368461
Dendritic cells_10
10x_3288_t1_ACCCGTACTGTCTT-1Patient5 Monocytes nonactive 4 0.07569273
Monocytes_4
10x_3288_t1_ACCCGTTGAGTCAC-1 Patient5 Monocytes nonactive 4 0.06920931
Monocytes_4
10x_3288_t1_ACCCGTTGGTAGCT-1 Patient5 Progenitors active 1 0.14434722
Progenitors_1
10x_3288_t1_ACCCGTTGTCCGTC-1 Patient5 Monocytes nonactive 0 0.03639592
Monocytes_0
10x_3288_t1_ACCCTCGAATTGGC-1 Patient5 Progenitors active 1 0.15771035
Progenitors_1
10x_3288_t1_ACCCTCGAGAAGGC-1 Patient5 Monocytes nonactive 4 0.061109
Monocytes_4
10x_3288_t1_ACCCTCGAGCGTTA-1 Patient5 Monocytes nonactive 0 0.02038552
Monocytes_0
10x_3288_t1_ACCCTCGAGGAGGT-1 Patient5 Dendritic cells nonactive 10 0.06437448
Dendritic cells_10
10x_3288_t1_ACCGAAACTCTCCG-1 Patient5 Monocytes nonactive 4 0.05787521
Monocytes_4
10x_3288_t1_ACCGCGGACGTAGT-1 Patient5 Monocytes nonactive 4 0.01507514
Monocytes_4
10x_3288_t1_ACCGCGGACTTGAG-1 Patient5 Monocytes nonactive 4 0.04533638

Monocytes_4
10x_3288_t1_ACCGCGGAGTCAAC-1 Patient5 Monocytes nonactive 4 0.03948703
Monocytes_4
10x_3288_t1_ACCGCGGATACTCT-1 Patient5 Monocytes nonactive 0 0.03828229
Monocytes_0
10x_3288_t1_ACCGTGCTAAGAAC-1 Patient5 Monocytes nonactive 8 0.02553738
Monocytes_8
10x_3288_t1_ACCGTGCTATCTTC-1 Patient5 Monocytes nonactive 0 0.03339991
Monocytes_0
10x_3288_t1_ACCGTGCTCACACA-1 Patient5 Monocytes nonactive 0 0.04560586
Monocytes_0
10x_3288_t1_ACCGTGCTCATGAC-1 Patient5 Monocytes nonactive 0 0.04061252
Monocytes_0
10x_3288_t1_ACCTATTGATGACC-1 Patient5 Progenitors active 1 0.1420804
Progenitors_1
10x_3288_t1_ACCTATTGCGCAAT-1 Patient5 Monocytes nonactive 0 0.04457549
Monocytes_0
10x_3288_t1_ACCTCCGAAGTCTG-1 Patient5 Monocytes nonactive 4 0.02499841
Monocytes_4
10x_3288_t1_ACCTCCGACACACA-1 Patient5 Monocytes nonactive 0 0.06478663
Monocytes_0
10x_3288_t1_ACCTCCGAGGATCT-1 Patient5 Monocytes nonactive 4 0.02832731
Monocytes_4
10x_3288_t1_ACCTCCGAGGTAGG-1 Patient5 Progenitors active 1 0.14987953
Progenitors_1
10x_3288_t1_ACCTCGTGACGGTT-1 Patient5 Monocytes nonactive 4 0.06142604
Monocytes_4
10x_3288_t1_ACCTCGTGCACCAA-1 Patient5 Monocytes nonactive 0 0.07634265
Monocytes_0
10x_3288_t1_ACCTCGTGCGGAGA-1 Patient5 Monocytes nonactive 4 0.05170883
Monocytes_4
10x_3288_t1_ACCTCGTGCTCCAC-1 Patient5 Monocytes nonactive 4 0.0881523
Monocytes_4

10x_3288_t1_ACCTCGTGCTCCCA-1 Patient5 Monocytes nonactive 0 0.02211337 Monocytes_0
10x_3288_t1_ACCTCGTGTCTAGG-1 Patient5 Monocytes nonactive 4 0.02609219 Monocytes_4
10x_3288_t1_ACCTGGCTAACCGT-1 Patient5 Monocytes nonactive 0 0.04094541 Monocytes_0
10x_3288_t1_ACCTGGCTGATGAA-1 Patient5 Monocytes nonactive 0 0.05717773 Monocytes_0
10x_3288_t1_ACCTGGCTTACAGC-1 Patient5 Progenitors nonactive 1 0.10604908 Progenitors_1
10x_3288_t1_ACCTTTGAGGACAG-1 Patient5 Monocytes nonactive 4 0.0273762 Monocytes_4
10x_3288_t1_ACCTTTGATCCAGA-1 Patient5 Progenitors active 1 0.16910786 Progenitors_1
10x_3288_t1_ACCTTTGATTCATC-1 Patient5 Monocytes nonactive 8 0.04630334 Monocytes_8
10x_3288_t1_ACGAACACACGGTT-1 Patient5 Progenitors active 1 0.12196436 Progenitors_1
10x_3288_t1_ACGAACACCATTGG-1 Patient5 Monocytes nonactive 0 0.07480502 Monocytes_0
10x_3288_t1_ACGAACTGACAGTC-1 Patient5 Monocytes nonactive 8 0.06123581 Monocytes_8
10x_3288_t1_ACGAACTGACCTAG-1 Patient5 Monocytes nonactive 8 0.03905903 Monocytes_8
10x_3288_t1_ACGAACTGCAACTG-1 Patient5 Monocytes nonactive 4 0.05403906 Monocytes_4
10x_3288_t1_ACGAAGCTCAAAGA-1 Patient5 Monocytes nonactive 4 0.00156934 Monocytes_4
10x_3288_t1_ACGACAACAGAAGT-1 Patient5 Monocytes nonactive 0 0.05630588 Monocytes_0
10x_3288_t1_ACGACAACGTCCTC-1 Patient5 Monocytes nonactive 0 0.08618667 Monocytes_0
10x_3288_t1_ACGACAACTTCATC-1 Patient5 Monocytes nonactive 0 0.03068924

Monocytes_0
10x_3288_t1_ACGACCCTAAGGGC-1 Patient5 Monocytes nonactive 4 0.07188828
Monocytes_4
10x_3288_t1_ACGAGGGAAAGCA-1 Patient5 Monocytes nonactive 4 0.04238793
Monocytes_4
10x_3288_t1_ACGAGGGAAAGGTA-1 Patient5 Monocytes nonactive 0 0.04205504
Monocytes_0
10x_3288_t1_ACGAGGGACGTCTC-1 Patient5 Progenitors active 1 0.15799569
Progenitors_1
10x_3288_t1_ACGAGGGAGCCAAT-1 Patient5 Monocytes nonactive 0 0.04557416
Monocytes_0
10x_3288_t1_ACGAGGGAGTTCTT-1 Patient5 Monocytes nonactive 0 0.05541817
Monocytes_0
10x_3288_t1_ACGAGGGAGTTTCT-1 Patient5 Monocytes nonactive 0 0.05717773
Monocytes_0
10x_3288_t1_ACGAGTACGGACGA-1 Patient5 Monocytes nonactive 4 0.08631349
Monocytes_4
10x_3288_t1_ACGAGTACGTACCA-1 Patient5 Monocytes nonactive 8 0.0640733
Monocytes_8
10x_3288_t1_ACGATCGAACCAAC-1 Patient5 Progenitors nonactive 1 0.10850612
Progenitors_1
10x_3288_t1_ACGATCGACCGATA-1 Patient5 Progenitors active 1 0.13854543
Progenitors_1
10x_3288_t1_ACGATCGAGTCACA-1 Patient5 Progenitors active 1 0.17250016
Progenitors_1
10x_3288_t1_ACGATCGATTACTC-1 Patient5 Progenitors active 1 0.16214888
Progenitors_1
10x_3288_t1_ACGATGACCGCTAA-1 Patient5 Monocytes nonactive 0 0.042594
Monocytes_0
10x_3288_t1_ACGATGACGTTTGG-1 Patient5 Monocytes nonactive 4 0.0438463
Monocytes_4
10x_3288_t1_ACGATTCTAGCGGA-1 Patient5 Progenitors active 1 0.14740663
Progenitors_1

10x_3288_t1_ACGATTCTCCGTAA-1 Patient5 Monocytes nonactive 4 0.03924925 Monocytes_4
10x_3288_t1_ACGATTCTCCTTGC-1 Patient5 Monocytes nonactive 4 0.06984338 Monocytes_4
10x_3288_t1_ACGATTCTCTTCCG-1 Patient5 Monocytes nonactive 0 0.04124659 Monocytes_0
10x_3288_t1_ACGCAATGCTGAAC-1 Patient5 Progenitors active 1 0.15748843 Progenitors_1
10x_3288_t1_ACGCAATGGAAACA-1 Patient5 Monocytes nonactive 0 0.0122218 Monocytes_0
10x_3288_t1_ACGCAATGGAATGA-1 Patient5 Monocytes nonactive 15 0.03956629 Monocytes_15
10x_3288_t1_ACGCACCTAATCGC-1 Patient5 Progenitors active 1 0.12602245 Progenitors_1
10x_3288_t1_ACGCACCTAGTCAC-1 Patient5 Progenitors active 1 0.12760763 Progenitors_1
10x_3288_t1_ACGCACCTTATGCG-1 Patient5 Progenitors nonactive 1 0.10371885 Progenitors_1
10x_3288_t1_ACGCACCTTTTACC-1 Patient5 Monocytes nonactive 4 0.05251728 Monocytes_4
10x_3288_t1_ACGCCACTACACGT-1 Patient5 Progenitors active 1 0.15950162 Progenitors_1
10x_3288_t1_ACGCCACTATGGTC-1 Patient5 Monocytes nonactive 4 0.03590451 Monocytes_4
10x_3288_t1_ACGCCACTGGAACG-1 Patient5 Progenitors active 6 0.14315833 Progenitors_6
10x_3288_t1_ACGCCACTTAGTCG-1 Patient5 Monocytes nonactive 0 0.04249889 Monocytes_0
10x_3288_t1_ACGCCACTTCCAGA-1 Patient5 Monocytes nonactive 0 0.02306449 Monocytes_0
10x_3288_t1_ACGCCACTTGGAAA-1 Patient5 Monocytes nonactive 4 0.06989094 Monocytes_4
10x_3288_t1_ACGCCGGACCTCCA-1 Patient5 Monocytes nonactive 0 0.0388054

Monocytes_0
10x_3288_t1_ACGCCGGACTTAGG-1 Patient5 Monocytes nonactive 4 0.07619999
Monocytes_4
10x_3288_t1_ACGCCGGAGTACGT-1 Patient5 Monocytes nonactive 8 0.02585442
Monocytes_8
10x_3288_t1_ACGCCGGAGTTACG-1 Patient5 Monocytes nonactive 0 0.02344493
Monocytes_0
10x_3288_t1_ACGCCGGATCTACT-1 Patient5 Progenitors active 1 0.14845286
Progenitors_1
10x_3288_t1_ACGCCTTGAGAAGT-1 Patient5 Monocytes nonactive 4 0.02900894
Monocytes_4
10x_3288_t1_ACGCCTTGGGACAG-1 Patient5 Monocytes nonactive 0 0.02010018
Monocytes_0
10x_3288_t1_ACGCCTTGTCGCTC-1 Patient5 Monocytes nonactive 0 0.05475239
Monocytes_0
10x_3288_t1_ACGCTCACCAGGAG-1 Patient5 Monocytes nonactive 4 0.07485258
Monocytes_4
10x_3288_t1_ACGCTCACGCAAGG-1 Patient5 Monocytes nonactive 4 0.06145774
Monocytes_4
10x_3288_t1_ACGCTCACTTTGCT-1 Patient5 Monocytes nonactive 0 0.04486082
Monocytes_0
10x_3288_t1_ACGCTGCTAGATGA-1 Patient5 Monocytes nonactive 15 0.02558493
Monocytes_15
10x_3288_t1_ACGCTGCTCTAAGC-1 Patient5 Progenitors active 1 0.18083825
Progenitors_1
10x_3288_t1_ACGCTGCTCTAGTG-1 Patient5 Progenitors active 1 0.12608585
Progenitors_1
10x_3288_t1_ACGCTGCTGGGATG-1 Patient5 Monocytes nonactive 4 0.03086361
Monocytes_4
10x_3288_t1_ACGGAACTACCATG-1 Patient5 Monocytes nonactive 0 0.05979329
Monocytes_0
10x_3288_t1_ACGGAACTACCGAT-1 Patient5 Progenitors active 1 0.15609346
Progenitors_1

10x_3288_t1_ACGGAACTACGCTA-1 Patient5 Monocytes nonactive 0 0.02877116
Monocytes_0
10x_3288_t1_ACGGAACTAGTACC-1 Patient5 Progenitors active 1 0.18096506
Progenitors_1
10x_3288_t1_ACGGAACTTACTCT-1 Patient5 Monocytes nonactive 16 0.08374548
Monocytes_16
10x_3288_t1_ACGGAGGAAGCACT-1 Patient5 Monocytes nonactive 0 0.06358189
Monocytes_0
10x_3288_t1_ACGGAGGACGTTAG-1 Patient5 Progenitors active 1 0.1404001
Progenitors_1
10x_3288_t1_ACGGAGGAGTACCA-1 Patient5 Monocytes nonactive 0 0.06697419
Monocytes_0
10x_3288_t1_ACGGAGGATGCTCC-1 Patient5 Progenitors active 1 0.12911356
Progenitors_1
10x_3288_t1_ACGGATTGAACGTC-1 Patient5 Monocytes nonactive 0 0.01150846
Monocytes_0
10x_3288_t1_ACGGATTGGAATGA-1 Patient5 Monocytes nonactive 0 0.05151861
Monocytes_0
10x_3288_t1_ACGGATTGGTGTCA-1 Patient5 Monocytes nonactive 8 0.02331812
Monocytes_8
10x_3288_t1_ACGGATTGTTGTCT-1Patient5 Progenitors active 1 0.21071904
Progenitors_1
10x_3288_t1_ACGGCGTGATAAGG-1 Patient5 Monocytes nonactive 0 0.06302708
Monocytes_0
10x_3288_t1_ACGGCGTGCATGAC-1 Patient5 Progenitors active 1 0.13735654
Progenitors_1
10x_3288_t1_ACGGCGTGCCTCAC-1 Patient5 Monocytes nonactive 13 0.03628495
Monocytes_13
10x_3288_t1_ACGGCGTGGACAAA-1 Patient5 Progenitors nonactive 1 0.11522732
Progenitors_1
10x_3288_t1_ACGGCGTGGAGACG-1 Patient5 Monocytes nonactive 4 0.07041405
Monocytes_4
10x_3288_t1_ACGGCGTGGGTATC-1 Patient5 Monocytes nonactive 0 0.02921501

Monocytes_0
10x_3288_t1_ACGGCGTGTCTAGG-1 Patient5 Monocytes nonactive 4 0.03901148 Monocytes_4
10x_3288_t1_ACGGCTCTAAGTGA-1 Patient5 Progenitors active 1 0.14095492 Progenitors_1
10x_3288_t1_ACGGCTCTACGTTG-1 Patient5 Monocytes nonactive 0 0.04535223 Monocytes_0
10x_3288_t1_ACGGCTCTCCATGA-1 Patient5 Monocytes nonactive 0 0.07581954 Monocytes_0
10x_3288_t1_ACGGCTCTCGTAAC-1 Patient5 Monocytes nonactive 4 0.03985163 Monocytes_4
10x_3288_t1_ACGGCTCTCTTCGC-1 Patient5 Progenitors active 1 0.12809904 Progenitors_1
10x_3288_t1_ACGGCTCTGTTGTG-1 Patient5 Monocytes nonactive 0 0.03924925 Monocytes_0
10x_3288_t1_ACGGCTCTTACGAC-1 Patient5 Monocytes nonactive 4 0.08751823 Monocytes_4
10x_3288_t1_ACGGCTCTTCGCTC-1 Patient5 Progenitors active 1 0.16170503 Progenitors_1
10x_3288_t1_ACGGCTCTTCTGGA-1 Patient5 Monocytes nonactive 0 0.00726016 Monocytes_0
10x_3288_t1_ACGGGAGACTACCC-1 Patient5 Monocytes nonactive 4 0.05638514 Monocytes_4
10x_3288_t1_ACGGGAGACTGAAC-1 Patient5 Progenitors nonactive 1 0.10259337 Progenitors_1
10x_3288_t1_ACGGGAGACTTATC-1 Patient5 Monocytes nonactive 4 0.05031387 Monocytes_4
10x_3288_t1_ACGGGAGATTCTAC-1 Patient5 Monocytes nonactive 4 0.0333048 Monocytes_4
10x_3288_t1_ACGGTAACCGCATA-1 Patient5 Progenitors active 1 0.13409105 Progenitors_1
10x_3288_t1_ACGGTAACTCCCGT-1 Patient5 Monocytes nonactive 8 0.081336 Monocytes_8

10x_3288_t1_ACGGTAACTCGCAA-1 Patient5 Progenitors nonactive 1 0.11598821 Progenitors_1
10x_3288_t1_ACGGTAACTCGCCT-1 Patient5 Monocytes nonactive 4 0.03273413 Monocytes_4
10x_3288_t1_ACGGTATGTGACAC-1 Patient5 Monocytes nonactive 0 0.05359521 Monocytes_0
10x_3288_t1_ACGGTCCTCAAAGA-1 Patient5 Monocytes nonactive 4 0.04890305 Monocytes_4
10x_3288_t1_ACGGTCCTTAACCG-1 Patient5 Progenitors active 1 0.16329022 Progenitors_1
10x_3288_t1_ACGGTCCTTTGGCA-1 Patient5 Monocytes nonactive 0 0.04489252 Monocytes_0
10x_3288_t1_ACGTAGACAGCAAA-1 Patient5 Monocytes nonactive 0 0.04365608 Monocytes_0
10x_3288_t1_ACGTAGACAGTTCG-1 Patient5 Monocytes nonactive 15 0.04738127 Monocytes_15
10x_3288_t1_ACGTCAGAACAGCT-1 Patient5 Monocytes nonactive 0 0.02924672 Monocytes_0
10x_3288_t1_ACGTCAGAATGACC-1 Patient5 Monocytes nonactive 4 0.02132078 Monocytes_4
10x_3288_t1_ACGTCAGACTGTTT-1 Patient5 Monocytes nonactive 8 0.02471308 Monocytes_8
10x_3288_t1_ACGTCAGAGTCGAT-1 Patient5 Monocytes nonactive 0 0.06960561 Monocytes_0
10x_3288_t1_ACGTCAGATAACCG-1 Patient5 Monocytes nonactive 0 0.07226872 Monocytes_0
10x_3288_t1_ACGTCAGATCCAGA-1 Patient5 Monocytes nonactive 4 0.03546066 Monocytes_4
10x_3288_t1_ACGTCCTGAGACTC-1 Patient5 Progenitors active 1 0.15290723 Progenitors_1
10x_3288_t1_ACGTCCTGCTAGAC-1 Patient5 Progenitors active 1 0.13317164 Progenitors_1
10x_3288_t1_ACGTCCTGGTTGGT-1 Patient5 Monocytes nonactive 0 0.03018198

Monocytes_0
10x_3288_t1_ACGTCCTGTCTCGC-1 Patient5 Monocytes nonactive 0 0.02385708
Monocytes_0
10x_3288_t1_ACGTCGCTGAGATA-1 Patient5 Monocytes nonactive 0 0.04841164
Monocytes_0
10x_3288_t1_ACGTCGCTGCTTAG-1 Patient5 Monocytes nonactive 4 0.07738888
Monocytes_4
10x_3288_t1_ACGTCGCTTTGGTG-1 Patient5 Monocytes nonactive 4 0.0607127
Monocytes_4
10x_3288_t1_ACGTGATGAACCGT-1 Patient5 Progenitors active 6 0.16211718
Progenitors_6
10x_3288_t1_ACGTGATGCGAACT-1 Patient5 Monocytes nonactive 4 0.01147676
Monocytes_4
10x_3288_t1_ACGTGATGGGTTAC-1 Patient5 Progenitors active 1 0.12470674
Progenitors_1
10x_3288_t1_ACGTGCCTAGCTCA-1 Patient5 Monocytes nonactive 4 0.0413417
Monocytes_4
10x_3288_t1_ACGTGCCTCACACA-1 Patient5 Monocytes nonactive 0 0.01035128
Monocytes_0
10x_3288_t1_ACGTGCCTGACAAA-1 Patient5 Monocytes nonactive 4 0.05105891
Monocytes_4
10x_3288_t1_ACGTGCCTGCTACA-1 Patient5 Monocytes nonactive 4 0.00551645
Monocytes_4
10x_3288_t1_ACGTGCCTGGTGAG-1 Patient5 Monocytes nonactive 0 0.04679475
Monocytes_0
10x_3288_t1_ACGTGCCTTAGACC-1 Patient5 Monocytes nonactive 0 0.02244626
Monocytes_0
10x_3288_t1_ACGTTACTTGCCCT-1Patient5 Monocytes nonactive 0 0.03038805
Monocytes_0
10x_3288_t1_ACGTTGGACTTATC-1 Patient5 Progenitors nonactive 1 0.09531735
Progenitors_1
10x_3288_t1_ACGTTGGATTGCAG-1 Patient5 Monocytes nonactive 15 0.03994674
Monocytes_15

10x_3288_t1_ACGTTTACATGGTC-1 Patient5 Monocytes active 0 0.12700526
Monocytes_0
10x_3288_t1_ACGTTTACCCTCAC-1 Patient5 Progenitors active 1 0.15842369
Progenitors_1
10x_3288_t1_ACGTTTACGAATCC-1 Patient5 Monocytes nonactive 4 0.01127069
Monocytes_4
10x_3288_t1_ACGTTTACTTCACT-1 Patient5 Monocytes nonactive 0 0.01840403
Monocytes_0
10x_3288_t1_ACTAAAACGAAACA-1 Patient5 Dendritic cells active 10 0.11744658
Dendritic cells_10
10x_3288_t1_ACTAAAACGAGAGC-1 Patient5 Progenitors active 1 0.11933295
Progenitors_1
10x_3288_t1_ACTACGGAACTGGT-1 Patient5 Monocytes nonactive 0 0.0354448
Monocytes_0
10x_3288_t1_ACTACGGACGATAC-1 Patient5 Monocytes nonactive 4 0.03064168
Monocytes_4
10x_3288_t1_ACTACGGAGCAGAG-1 Patient5 Progenitors active 6 0.16086488
Progenitors_6
10x_3288_t1_ACTACTACATCGAC-1 Patient5 Monocytes nonactive 0 0.02669457
Monocytes_0
10x_3288_t1_ACTACTACCTACGA-1 Patient5 Monocytes nonactive 0 0.05516454
Monocytes_0
10x_3288_t1_ACTACTACGGTACT-1 Patient5 Progenitors active 1 0.15417539
Progenitors_1
10x_3288_t1_ACTAGGTGGGTATC-1 Patient5 Progenitors active 1 0.12156807
Progenitors_1
10x_3288_t1_ACTAGGTGTAGAAG-1 Patient5 Monocytes nonactive 0 0.05506943
Monocytes_0
10x_3288_t1_ACTAGGTGTTTACC-1 Patient5 Monocytes nonactive 0 0.05199417
Monocytes_0
10x_3288_t1_ACTATCACACACAC-1 Patient5 Monocytes nonactive 0 0.03818718
Monocytes_0
10x_3288_t1_ACTATCACAGTTCG-1 Patient5 Progenitors active 1 0.14364974

Progenitors_1
10x_3288_t1_ACTATCACCCTCCA-1 Patient5 Monocytes nonactive 0 0.06542071
Monocytes_0
10x_3288_t1_ACTATCACTACGCA-1 Patient5 Monocytes nonactive 4 0.03901148
Monocytes_4
10x_3288_t1_ACTCAGGACTGATG-1 Patient5 Monocytes nonactive 0 0.1062393
Monocytes_0
10x_3288_t1_ACTCAGGATTCTAC-1 Patient5 Monocytes nonactive 4 0.01848329
Monocytes_4
10x_3288_t1_ACTCCCGAATGCTG-1 Patient5 Monocytes nonactive 4 0.05711432
Monocytes_4
10x_3288_t1_ACTCCCGACTTACT-1 Patient5 Monocytes nonactive 8 0.01981485
Monocytes_8
10x_3288_t1_ACTCCCGAGACGGA-1 Patient5 Monocytes nonactive 0 0.04503519
Monocytes_0
10x_3288_t1_ACTCCCGAGGCATT-1 Patient5 Progenitors active 6 0.14773952
Progenitors_6
10x_3288_t1_ACTCCCGAGGTGAG-1 Patient5 Monocytes nonactive 0 0.07788029
Monocytes_0
10x_3288_t1_ACTCCCGATAGCGT-1 Patient5 Monocytes nonactive 4 0.04578023
Monocytes_4
10x_3288_t1_ACTCCCGATTGAGC-1 Patient5 Monocytes nonactive 4 0.02204997
Monocytes_4
10x_3288_t1_ACTCCTCTAGCCAT-1 Patient5 Monocytes nonactive 4 0.06738634
Monocytes_4
10x_3288_t1_ACTCCTCTCTGACA-1 Patient5 Monocytes nonactive 4 0.03726777
Monocytes_4
10x_3288_t1_ACTCGAGAAGCATC-1 Patient5 Monocytes nonactive 15 0.05104305
Monocytes_15
10x_3288_t1_ACTCGAGAAGCCAT-1 Patient5 Monocytes nonactive 4 0.06729123
Monocytes_4
10x_3288_t1_ACTCGAGAATAAGG-1 Patient5 Monocytes nonactive 0 0.03081605
Monocytes_0

10x_3288_t1_ACTCGAGATCCAGA-1	Patient5	Monocytes	nonactive	8	0.02087693	Monocytes_8
10x_3288_t1_ACTCGCACAAGAAC-1	Patient5	Monocytes	nonactive	4	0.01320462	Monocytes_4
10x_3288_t1_ACTCTATGCCATGA-1	Patient5	Progenitors	active	1	0.12274111	Progenitors_1
10x_3288_t1_ACTCTATGCTTGCC-1	Patient5	Progenitors	nonactive	1	0.10866464	Progenitors_1
10x_3288_t1_ACTCTATGGCTACA-1	Patient5	Progenitors	active	1	0.1648437	Progenitors_1
10x_3288_t1_ACTCTATGGGTAAA-1	Patient5	Monocytes	nonactive	0	0.00927335	Monocytes_0
10x_3288_t1_ACTCTATGTTTCGT-1	Patient5	Monocytes	nonactive	4	0.10639782	Monocytes_4
10x_3288_t1_ACTCTCCTCTGTCC-1	Patient5	Progenitors	active	1	0.12576882	Progenitors_1
10x_3288_t1_ACTCTCCTGGAGGT-1	Patient5	Monocytes	nonactive	0	0.04409993	Monocytes_0
10x_3288_t1_ACTCTCCTGGTAAA-1	Patient5	Monocytes	nonactive	4	0.0139338	Monocytes_4
10x_3288_t1_ACTGAGACCGTGAT-1	Patient5	Monocytes	nonactive	0	0.05159787	Monocytes_0
10x_3288_t1_ACTGAGACTTTGCT-1	Patient5	Monocytes	nonactive	4	0.0590007	Monocytes_4
10x_3288_t1_ACTGCCACGTAAGA-1	Patient5	Monocytes	nonactive	4	0.04503519	Monocytes_4
10x_3288_t1_ACTGCCTGAAGAAC-1	Patient5	Monocytes	nonactive	0	0.06069685	Monocytes_0
10x_3288_t1_ACTGCCTGGAAGGC-1	Patient5	Monocytes	nonactive	4	0.04322808	Monocytes_4
10x_3288_t1_ACTGGCCTAGCTAC-1	Patient5	Monocytes	nonactive	4	0.06843257	Monocytes_4
10x_3288_t1_ACTGGCCTATTCTC-1	Patient5	Monocytes	nonactive	8	0.02777249	

Monocytes_8
10x_3288_t1_ACTGTGGAGCTGAT-1 Patient5 Progenitors active 1 0.1525902 Progenitors_1
10x_3288_t1_ACTGTGGATAACCG-1 Patient5 Progenitors active 1 0.13188764 Progenitors_1
10x_3288_t1_ACTGTGGATGAGAA-1 Patient5 Progenitors active 6 0.11972925 Progenitors_6
10x_3288_t1_ACTGTTACCGAACT-1 Patient5 Monocytes nonactive 4 0.05031387 Monocytes_4
10x_3288_t1_ACTGTTACGACAGG-1 Patient5 Monocytes nonactive 0 0.03607888 Monocytes_0
10x_3288_t1_ACTGTTACTAGTCG-1 Patient5 Monocytes nonactive 0 0.02590197 Monocytes_0
10x_3288_t1_ACTGTTACTTCTAC-1 Patient5 Monocytes nonactive 4 0.02734449 Monocytes_4
10x_3288_t1_ACTTAAGAACCTAG-1 Patient5 Progenitors active 1 0.13352039 Progenitors_1
10x_3288_t1_ACTTAAGAATCGAC-1 Patient5 Monocytes nonactive 4 0.02067085 Monocytes_4
10x_3288_t1_ACTTAGCTGTTGCA-1 Patient5 Monocytes nonactive 0 0.03645932 Monocytes_0
10x_3288_t1_ACTTAGCTTAGTCG-1 Patient5 Monocytes nonactive 8 0.06131507 Monocytes_8
10x_3288_t1_ACTTCAACCACCAA-1 Patient5 Monocytes nonactive 4 0.03615814 Monocytes_4
10x_3288_t1_ACTTCAACCTAGAC-1 Patient5 Monocytes nonactive 4 0.04335489 Monocytes_4
10x_3288_t1_ACTTCAACGGGCAA-1 Patient5 Monocytes nonactive 4 0.01681884 Monocytes_4
10x_3288_t1_ACTTCCCTAACGTC-1 Patient5 Monocytes nonactive 0 0.04443282 Monocytes_0
10x_3288_t1_ACTTCTGACGTGAT-1 Patient5 Monocytes nonactive 4 0.09178239 Monocytes_4

10x_3288_t1_ACTTCTGAGAATCC-1 Patient5 Progenitors active 1 0.11781117 Progenitors_1
10x_3288_t1_ACTTCTGATCCCGT-1 Patient5 Monocytes nonactive 0 0.06152115 Monocytes_0
10x_3288_t1_ACTTCTGATCCGAA-1 Patient5 Monocytes nonactive 0 0.04501934 Monocytes_0
10x_3288_t1_ACTTCTGATGACCA-1 Patient5 Monocytes nonactive 0 0.0383457 Monocytes_0
10x_3288_t1_ACTTGACTACGGTT-1 Patient5 Progenitors active 1 0.1635914 Progenitors_1
10x_3288_t1_ACTTGACTCACCAA-1 Patient5 Progenitors nonactive 1 0.09912181 Progenitors_1
10x_3288_t1_ACTTGACTTGGTGT-1 Patient5 Monocytes nonactive 4 0.01361676 Monocytes_4
10x_3288_t1_ACTTGGGAAACAGA-1 Patient5 Monocytes nonactive 0 0.07282354 Monocytes_0
10x_3288_t1_ACTTGGGAGAATCC-1 Patient5 Monocytes nonactive 0 0.042166 Monocytes_0
10x_3288_t1_ACTTGTACACCAAC-1 Patient5 Monocytes nonactive 8 0.04633505 Monocytes_8
10x_3288_t1_ACTTGTACGCGGAA-1 Patient5 Progenitors nonactive 1 0.1003107 Progenitors_1
10x_3288_t1_ACTTGTACGGTCAT-1 Patient5 Progenitors nonactive 1 0.09235305 Progenitors_1
10x_3288_t1_ACTTGTACGTACCA-1 Patient5 Monocytes nonactive 0 0.04121489 Monocytes_0
10x_3288_t1_ACTTGTACTGCTAG-1 Patient5 Monocytes nonactive 8 0.01878448 Monocytes_8
10x_3288_t1_ACTTTGTGACCACA-1 Patient5 Monocytes nonactive 0 0.04462304 Monocytes_0
10x_3288_t1_ACTTTGTGAGAACA-1 Patient5 Monocytes nonactive 0 0.02452286 Monocytes_0
10x_3288_t1_ACTTTGTGGACACT-1 Patient5 Progenitors nonactive 1 0.11292879

Progenitors_1

10x_3288_t1_ACTTTGTGGACAGG-1 Patient5 Progenitors active 1 0.18832033 Progenitors_1

10x_3288_t1_ACTTTGTGGGTGTT-1 Patient5 Monocytes nonactive 0 0.04058081 Monocytes_0

10x_3288_t1_AGAAACGACTTATC-1 Patient5 Monocytes nonactive 0 0.06973242 Monocytes_0

10x_3288_t1_AGAAACGAGCAGAG-1 Patient5 Monocytes nonactive 4 0.03525458 Monocytes_4

10x_3288_t1_AGAACAGAAGTTCG-1 Patient5 Monocytes nonactive 8 0.06163211 Monocytes_8

10x_3288_t1_AGAACAGACTGATG-1 Patient5 Progenitors active 1 0.15729821 Progenitors_1

10x_3288_t1_AGAACAGAGGAAAT-1 Patient5 Monocytes nonactive 0 0.07512206 Monocytes_0

10x_3288_t1_AGAACAGATCCGAA-1 Patient5 Progenitors active 1 0.18148817 Progenitors_1

10x_3288_t1_AGAACGCTAGTCTG-1 Patient5 Monocytes nonactive 0 0.06332826 Monocytes_0

10x_3288_t1_AGAACGCTCAGATC-1 Patient5 Progenitors active 1 0.15666413 Progenitors_1

10x_3288_t1_AGAACGCTCCCGTT-1 Patient5 Monocytes nonactive 0 0.02013189 Monocytes_0

10x_3288_t1_AGAACGCTCCTATT-1 Patient5 Monocytes nonactive 4 0.04993342 Monocytes_4

10x_3288_t1_AGAACGCTCTCTCG-1 Patient5 Monocytes nonactive 4 0.05709847 Monocytes_4

10x_3288_t1_AGAACGCTTCCGAA-1 Patient5 Monocytes nonactive 4 0.03125991 Monocytes_4

10x_3288_t1_AGAATACTAAAACG-1 Patient5 Progenitors active 1 0.15453998 Progenitors_1

10x_3288_t1_AGAATACTTCATTC-1Patient5 Monocytes nonactive 8 0.04010526 Monocytes_8

10x_3288_t1_AGAATGGAAGATGA-1 Patient5 Monocytes nonactive 0 0.06150529 Monocytes_0
10x_3288_t1_AGAATGGAGTTCAG-1 Patient5 Progenitors nonactive 5 0.05315135 Progenitors_5
10x_3288_t1_AGAATTTGACGACT-1 Patient5 Monocytes nonactive 4 0.01471054 Monocytes_4
10x_3288_t1_AGAATTTGAGCGGA-1 Patient5 Monocytes nonactive 0 0.04589119 Monocytes_0
10x_3288_t1_AGAATTTGAGCGTT-1 Patient5 Monocytes nonactive 4 0.07542325 Monocytes_4
10x_3288_t1_AGAATTTGCCCTAC-1 Patient5 Monocytes nonactive 8 0.05846173 Monocytes_8
10x_3288_t1_AGAATTTGCTCATT-1 Patient5 Monocytes nonactive 15 0.03615814 Monocytes_15
10x_3288_t1_AGAATTTGCTTCTA-1 Patient5 Progenitors active 1 0.13635787 Progenitors_1
10x_3288_t1_AGAATTTGGAGCAG-1 Patient5 Monocytes nonactive 8 0.07155539 Monocytes_8
10x_3288_t1_AGAATTTGGTGTTG-1 Patient5 Progenitors active 1 0.1437607 Progenitors_1
10x_3288_t1_AGAATTTGTCCGTC-1 Patient5 Progenitors active 1 0.15201953 Progenitors_1
10x_3288_t1_AGAATTTGTTCCCG-1 Patient5 Progenitors nonactive 1 0.11129605 Progenitors_1
10x_3288_t1_AGACACACAAGGGC-1 Patient5 Monocytes nonactive 0 0.03942363 Monocytes_0
10x_3288_t1_AGACACACCTTCGC-1 Patient5 Monocytes nonactive 0 0.03685562 Monocytes_0
10x_3288_t1_AGACACACGCAAGG-1 Patient5 Monocytes nonactive 0 0.06258322 Monocytes_0
10x_3288_t1_AGACACACGGTAAA-1 Patient5 Progenitors active 6 0.15285968 Progenitors_6
10x_3288_t1_AGACACACTTGTCT-1 Patient5 Progenitors active 1 0.16796652

Progenitors_1

10x_3288_t1_AGACACTGGTAAAG-1 Patient5 Monocytes nonactive 0 0.01374358 Monocytes_0

10x_3288_t1_AGACACTGTGTCAG-1 Patient5 Progenitors active 1 0.12174244 Progenitors_1

10x_3288_t1_AGACCTGACCGTAA-1 Patient5 Progenitors active 1 0.17094667 Progenitors_1

10x_3288_t1_AGACCTGACGATAC-1 Patient5 Progenitors nonactive 1 0.08544163 Progenitors_1

10x_3288_t1_AGACCTGACTGAAC-1 Patient5 Monocytes nonactive 0 0.03038805 Monocytes_0

10x_3288_t1_AGACCTGACTGCAA-1 Patient5 Monocytes nonactive 0 0.02522034 Monocytes_0

10x_3288_t1_AGACCTGAGCCAAT-1 Patient5 Monocytes nonactive 4 0.03788599 Monocytes_4

10x_3288_t1_AGACCTGAGTTACG-1 Patient5 Monocytes nonactive 0 0.03422421 Monocytes_0

10x_3288_t1_AGACCTGATTCCCG-1 Patient5 Monocytes nonactive 4 0.03779088 Monocytes_4

10x_3288_t1_AGACGTACTCCTCG-1 Patient5 Monocytes nonactive 4 0.01463129 Monocytes_4

10x_3288_t1_AGACGTACTGTCAG-1 Patient5 Monocytes nonactive 0 0.01627988 Monocytes_0

10x_3288_t1_AGACTCGACAGTTG-1 Patient5 Monocytes nonactive 4 0.09041912 Monocytes_4

10x_3288_t1_AGACTCGACTTAGG-1 Patient5 Monocytes nonactive 8 0.04909327 Monocytes_8

10x_3288_t1_AGACTCGATCTCAT-1Patient5 Progenitors active 6 0.1837867 Progenitors_6

10x_3288_t1_AGACTGACCATTTC-1 Patient5 Monocytes nonactive 8 0.06093463 Monocytes_8

10x_3288_t1_AGACTGACGACTAC-1 Patient5 Monocytes nonactive 8 0.03986748 Monocytes_8

10x_3288_t1_AGACTGACGTGAGG-1 Patient5 Monocytes nonactive 0 0.04205504 Monocytes_0
10x_3288_t1_AGACTTCTACTCTT-1 Patient5 Monocytes nonactive 0 0.0454949 Monocytes_0
10x_3288_t1_AGACTTCTGACTAC-1 Patient5 Monocytes nonactive 8 0.04291104 Monocytes_8
10x_3288_t1_AGACTTCTTCAGGT-1 Patient5 Monocytes nonactive 4 0.04787268 Monocytes_4
10x_3288_t1_AGACTTCTTCCTAT-1 Patient5 Monocytes nonactive 4 0.06711686 Monocytes_4
10x_3288_t1_AGAGAAACCTTTAC-1 Patient5 Monocytes nonactive 4 0.05264409 Monocytes_4
10x_3288_t1_AGAGAATGGGAAGC-1 Patient5 Monocytes nonactive 4 0.04497178 Monocytes_4
10x_3288_t1_AGAGAATGTAAGCC-1 Patient5 Monocytes nonactive 4 0.03070509 Monocytes_4
10x_3288_t1_AGAGATGAACCTAG-1 Patient5 Progenitors active 1 0.15870902 Progenitors_1
10x_3288_t1_AGAGATGACTCCAC-1 Patient5 Monocytes nonactive 4 0.06064929 Monocytes_4
10x_3288_t1_AGAGATGAGGTGTT-1 Patient5 Monocytes nonactive 4 0.06925686 Monocytes_4
10x_3288_t1_AGAGATGATGGTTG-1 Patient5 Monocytes nonactive 0 0.04207089 Monocytes_0
10x_3288_t1_AGAGCGGAAGATCC-1 Patient5 Progenitors active 1 0.15704458 Progenitors_1
10x_3288_t1_AGAGCTACGGTAGG-1 Patient5 Progenitors active 1 0.18312092 Progenitors_1
10x_3288_t1_AGAGCTACGTTCTT-1 Patient5 Monocytes nonactive 0 0.05800203 Monocytes_0
10x_3288_t1_AGAGCTACTGCATG-1 Patient5 Progenitors nonactive 1 0.11194598 Progenitors_1
10x_3288_t1_AGAGGTCTTCGCCT-1 Patient5 Monocytes nonactive 4 0.04297445

Monocytes_4
10x_3288_t1_AGAGGTCTTTCAGG-1 Patient5 Progenitors active 1 0.13176083 Progenitors_1
10x_3288_t1_AGAGTCACACTAGC-1 Patient5 Monocytes nonactive 0 0.07892651 Monocytes_0
10x_3288_t1_AGAGTCACAGAGAT-1 Patient5 Progenitors active 1 0.17476698 Progenitors_1
10x_3288_t1_AGAGTCACCTGTCC-1 Patient5 Monocytes nonactive 4 0.05869951 Monocytes_4
10x_3288_t1_AGAGTCACCTTGCC-1 Patient5 Progenitors active 1 0.13756261 Progenitors_1
10x_3288_t1_AGAGTCTGACGCAT-1 Patient5 Monocytes nonactive 4 0.00649927 Monocytes_4
10x_3288_t1_AGAGTCTGATCGTG-1 Patient5 Progenitors active 1 0.15951747 Progenitors_1
10x_3288_t1_AGAGTCTGCACTCC-1 Patient5 Monocytes nonactive 0 0.10400418 Monocytes_0
10x_3288_t1_AGAGTCTGCCGTTC-1 Patient5 Monocytes nonactive 0 0.03947118 Monocytes_0
10x_3288_t1_AGAGTGCTACCTTT-1 Patient5 Monocytes nonactive 4 0.04749223 Monocytes_4
10x_3288_t1_AGAGTGCTATCAGC-1 Patient5 Progenitors active 1 0.1433644 Progenitors_1
10x_3288_t1_AGAGTGCTTACAGC-1 Patient5 Monocytes nonactive 4 0.05245387 Monocytes_4
10x_3288_t1_AGATATACCCTATT-1 Patient5 Monocytes nonactive 4 0.03319384 Monocytes_4
10x_3288_t1_AGATATACGATGAA-1 Patient5 Progenitors active 1 0.13491535 Progenitors_1
10x_3288_t1_AGATATACTCTGGA-1 Patient5 Monocytes nonactive 0 0.03975652 Monocytes_0
10x_3288_t1_AGATATTGAACGTC-1 Patient5 Monocytes nonactive 4 0.05850929 Monocytes_4

10x_3288_t1_AGATATTGGCGAGA-1 Patient5 Monocytes nonactive 0 0.03496925 Monocytes_0
10x_3288_t1_AGATATTGTGCAAC-1 Patient5 Monocytes nonactive 4 0.04138926 Monocytes_4
10x_3288_t1_AGATCGTGATGGTC-1 Patient5 Monocytes nonactive 0 0.02450701 Monocytes_0
10x_3288_t1_AGATCGTGCCTGTC-1 Patient5 Monocytes nonactive 0 0.04853846 Monocytes_0
10x_3288_t1_AGATCGTGGTATGC-1 Patient5 Monocytes nonactive 0 0.08591719 Monocytes_0
10x_3288_t1_AGATCGTGTACGAC-1 Patient5 Monocytes nonactive 4 0.05701921 Monocytes_4
10x_3288_t1_AGATCTCTCATTTC-1 Patient5 Monocytes nonactive 0 0.0501712 Monocytes_0
10x_3288_t1_AGATCTCTTGAACC-1 Patient5 Monocytes nonactive 0 0.04682645 Monocytes_0
10x_3288_t1_AGATTAACATACCG-1 Patient5 Monocytes nonactive 4 0.06009448 Monocytes_4
10x_3288_t1_AGATTAACATGTGC-1 Patient5 Monocytes nonactive 4 0.05679729 Monocytes_4
10x_3288_t1_AGATTAACGGTGTT-1 Patient5 Monocytes nonactive 4 0.08141526 Monocytes_4
10x_3288_t1_AGATTCCTCAATCG-1 Patient5 Progenitors nonactive 1 0.10644537 Progenitors_1
10x_3288_t1_AGATTCCTCTCCAC-1 Patient5 Progenitors nonactive 1 0.1079513 Progenitors_1
10x_3288_t1_AGATTCCTGCTGAT-1 Patient5 Monocytes nonactive 4 0.02732864 Monocytes_4
10x_3288_t1_AGCAAAGAAACGTC-1 Patient5 Monocytes nonactive 0 0.07299791 Monocytes_0
10x_3288_t1_AGCAAAGAAATCGC-1 Patient5 Monocytes nonactive 0 0.08287363 Monocytes_0
10x_3288_t1_AGCAAAGAGACGTT-1 Patient5 Monocytes nonactive 4 0.07171391

Monocytes_4
10x_3288_t1_AGCAAAGAGCAAGG-1 Patient5 Monocytes nonactive 0 0.06553167
Monocytes_0
10x_3288_t1_AGCAACACCGCTAA-1 Patient5 Monocytes nonactive 4 0.03292435
Monocytes_4
10x_3288_t1_AGCAACACGGTTAC-1 Patient5 Dendritic cells nonactive 10 0.07142857
Dendritic cells_10
10x_3288_t1_AGCAAGCTACCTGA-1 Patient5 Monocytes nonactive 3 0.03894807
Monocytes_3
10x_3288_t1_AGCAAGCTCGCATA-1 Patient5 Monocytes nonactive 4 0.02476064
Monocytes_4
10x_3288_t1_AGCAAGCTTGGAAA-1 Patient5 Monocytes nonactive 8 0.07214191
Monocytes_8
10x_3288_t1_AGCACAACAGCTCA-1 Patient5 Monocytes nonactive 0 0.03717266
Monocytes_0
10x_3288_t1_AGCACAACCTCTTA-1 Patient5 Progenitors active 1 0.1656997
Progenitors_1
10x_3288_t1_AGCACTGAGGATTC-1 Patient5 Progenitors active 1 0.15450827
Progenitors_1
10x_3288_t1_AGCATGACAATCGC-1 Patient5 Monocytes nonactive 4 0.05537062
Monocytes_4
10x_3288_t1_AGCATGACACACGT-1 Patient5 Monocytes nonactive 0 0.06792531
Monocytes_0
10x_3288_t1_AGCATGACCTCCCA-1 Patient5 Monocytes nonactive 4 0.04086615
Monocytes_4
10x_3288_t1_AGCATGACCTTATC-1 Patient5 Monocytes nonactive 4 0.03943948
Monocytes_4
10x_3288_t1_AGCATGACGATACC-1 Patient5 Monocytes nonactive 0 0.09157631
Monocytes_0
10x_3288_t1_AGCATGACTAGCCA-1 Patient5 Monocytes nonactive 0 0.02609219
Monocytes_0
10x_3288_t1_AGCATTCTCCTGAA-1 Patient5 Monocytes nonactive 4 0.059825
Monocytes_4

10x_3288_t1_AGCATTCTGACACT-1 Patient5 Progenitors active 5 0.13862469 Progenitors_5
10x_3288_t1_AGCATTCTGAGGTG-1 Patient5 Progenitors active 1 0.14177922 Progenitors_1
10x_3288_t1_AGCATTCTTGCTTT-1 Patient5 Monocytes nonactive 0 0.04738127 Monocytes_0
10x_3288_t1_AGCCAATGCCTCGT-1 Patient5 Monocytes nonactive 4 0.05294528 Monocytes_4
10x_3288_t1_AGCCAATGCTCTTA-1 Patient5 Monocytes nonactive 0 0.03525458 Monocytes_0
10x_3288_t1_AGCCAATGCTGACA-1 Patient5 Progenitors active 1 0.17950669 Progenitors_1
10x_3288_t1_AGCCACCTAGCTCA-1 Patient5 Monocytes nonactive 4 0.07516962 Monocytes_4
10x_3288_t1_AGCCACCTCATGAC-1 Patient5 Monocytes nonactive 0 0.03306702 Monocytes_0
10x_3288_t1_AGCCACCTGTTCGA-1 Patient5 Monocytes nonactive 8 0.04371949 Monocytes_8
10x_3288_t1_AGCCACCTGTTGCA-1 Patient5 Monocytes nonactive 0 0.05010779 Monocytes_0
10x_3288_t1_AGCCGGACCCTCCA-1 Patient5 Progenitors active 1 0.15343035 Progenitors_1
10x_3288_t1_AGCCGGACGACGGA-1 Patient5 Progenitors active 1 0.18992137 Progenitors_1
10x_3288_t1_AGCCGGACTAACGC-1 Patient5 Monocytes nonactive 0 0.01423499 Monocytes_0
10x_3288_t1_AGCCGGACTACGAC-1 Patient5 Monocytes nonactive 0 0.03736288 Monocytes_0
10x_3288_t1_AGCCGGTGAAAACG-1 Patient5 Monocytes nonactive 4 0.0518198 Monocytes_4
10x_3288_t1_AGCCGGTGAACAGA-1 Patient5 Monocytes nonactive 0 0.04514615 Monocytes_0
10x_3288_t1_AGCCGGTGACTAGC-1 Patient5 Progenitors active 1 0.12752837

Progenitors_1

10x_3288_t1_AGCCGGTGAGTACC-1 Patient5 Progenitors active 1 0.17441824

Progenitors_1

10x_3288_t1_AGCCGTCTACGTAC-1 Patient5 Progenitors nonactive 1 0.10845856

Progenitors_1

10x_3288_t1_AGCCGTCTCGCATA-1 Patient5 Monocytes nonactive 0 0.04010526

Monocytes_0

10x_3288_t1_AGCCGTCTTCTTAC-1 Patient5 Progenitors nonactive 6 0.11202524

Progenitors_6

10x_3288_t1_AGCCGTCTTTGCTT-1Patient5 Progenitors active 1 0.2031894

Progenitors_1

10x_3288_t1_AGCCTCACATAAGG-1 Patient5 Monocytes nonactive 4 0.0455583

Monocytes_4

10x_3288_t1_AGCCTCACGGAAAT-1 Patient5 Monocytes nonactive 0 0.09747321

Monocytes_0

10x_3288_t1_AGCCTCTGCATTCT-1Patient5 Progenitors active 1 0.14799315

Progenitors_1

10x_3288_t1_AGCCTCTGCCATAG-1 Patient5 Progenitors active 1 0.16497052

Progenitors_1

10x_3288_t1_AGCCTCTGTCCAGA-1 Patient5 Progenitors active 1 0.14674085

Progenitors_1

10x_3288_t1_AGCGAACTCACAAC-1 Patient5 Progenitors active 1 0.16386088

Progenitors_1

10x_3288_t1_AGCGAACTGTGCAT-1 Patient5 Progenitors nonactive 1 0.11258005

Progenitors_1

10x_3288_t1_AGCGAACTTAGCCA-1 Patient5 Monocytes nonactive 4 0.02804198

Monocytes_4

10x_3288_t1_AGCGATACCAAGCT-1 Patient5 Progenitors active 5 0.13402765

Progenitors_5

10x_3288_t1_AGCGATACGTTACG-1 Patient5 Monocytes nonactive 13 0.0332731

Monocytes_13

10x_3288_t1_AGCGATTGGAGGCA-1 Patient5 Progenitors nonactive 1 0.11660643

Progenitors_1

10x_3288_t1_AGCGATTGGGTACT-1 Patient5 Progenitors nonactive 1 0.08016296 Progenitors_1
10x_3288_t1_AGCGCCGAGAGATA-1 Patient5 Monocytes nonactive 4 0.06790945 Monocytes_4
10x_3288_t1_AGCGCCGATGGTTG-1 Patient5 Monocytes nonactive 4 0.0307685 Monocytes_4
10x_3288_t1_AGCGCTCTAATCGC-1 Patient5 Monocytes nonactive 4 0.06656204 Monocytes_4
10x_3288_t1_AGCGCTCTCTAGTG-1 Patient5 Monocytes nonactive 4 0.02553738 Monocytes_4
10x_3288_t1_AGCGCTCTGCCAAT-1 Patient5 Monocytes nonactive 13 0.03013442 Monocytes_13
10x_3288_t1_AGCGCTCTTTCGTT-1Patient5 Dendritic cells nonactive 10 0.08284193 Dendritic cells_10
10x_3288_t1_AGCGGCACAGGTCT-1 Patient5 Progenitors active 1 0.17571809 Progenitors_1
10x_3288_t1_AGCGGCACCCTACC-1 Patient5 Monocytes nonactive 4 0.03687147 Monocytes_4
10x_3288_t1_AGCGGCACCTGTCC-1 Patient5 Progenitors active 1 0.17418046 Progenitors_1
10x_3288_t1_AGCGGCACGGTAAA-1 Patient5 Dendritic cells nonactive 10 0.09858284 Dendritic cells_10
10x_3288_t1_AGCGGCACGTCAAC-1 Patient5 Monocytes nonactive 4 0.06339167 Monocytes_4
10x_3288_t1_AGCGGCTGGTACCA-1 Patient5 Monocytes nonactive 4 0.05110646 Monocytes_4
10x_3288_t1_AGCGGCTGTGAGCT-1 Patient5 Progenitors active 1 0.17150149 Progenitors_1
10x_3288_t1_AGCGGGCTAAAAGC-1 Patient5 Progenitors active 1 0.1640194 Progenitors_1
10x_3288_t1_AGCGGGCTGCTATG-1 Patient5 Monocytes nonactive 0 0.07686577 Monocytes_0
10x_3288_t1_AGCGGGCTGGCGAA-1 Patient5 Monocytes nonactive 0 0.08418933

Monocytes_0
10x_3288_t1_AGCGGGCTTACGAC-1 Patient5 Monocytes nonactive 0 0.06867034
Monocytes_0
10x_3288_t1_AGCGTAACCACACA-1 Patient5 Monocytes nonactive 8 0.02113056
Monocytes_8
10x_3288_t1_AGCGTAACTCCAAG-1 Patient5 Monocytes nonactive 0 0.03462051
Monocytes_0
10x_3288_t1_AGCGTAACTCCGTC-1 Patient5 Progenitors active 1 0.13906854
Progenitors_1
10x_3288_t1_AGCGTAACTTCCGC-1 Patient5 Monocytes nonactive 4 0.06389893
Monocytes_4
10x_3288_t1_AGCTCGCTCCTCCA-1 Patient5 Monocytes nonactive 0 0.04693742
Monocytes_0
10x_3288_t1_AGCTCGCTGGTTTG-1 Patient5 Progenitors active 1 0.16516074
Progenitors_1
10x_3288_t1_AGCTCGCTTAGTCG-1 Patient5 Monocytes nonactive 13 0.04308541
Monocytes_13
10x_3288_t1_AGCTCGCTTGTCCC-1 Patient5 Monocytes nonactive 0 0.06362945
Monocytes_0
10x_3288_t1_AGCTCGCTTTGACG-1 Patient5 Monocytes nonactive 8 0.05083698
Monocytes_8
10x_3288_t1_AGCTGAACATGCTG-1 Patient5 Monocytes nonactive 0 0.08819986
Monocytes_0
10x_3288_t1_AGCTGAACCTAAGC-1 Patient5 Monocytes nonactive 8 0.04643016
Monocytes_8
10x_3288_t1_AGCTGAACGTCTGA-1 Patient5 Monocytes nonactive 0 0.04578023
Monocytes_0
10x_3288_t1_AGCTGAACTCGTAG-1 Patient5 Monocytes nonactive 0 0.04039059
Monocytes_0
10x_3288_t1_AGCTGCCTAACCAC-1 Patient5 Monocytes nonactive 4 0.03097457
Monocytes_4
10x_3288_t1_AGCTGCCTCCTTGC-1 Patient5 Progenitors active 1 0.1952159
Progenitors_1

10x_3288_t1_AGCTGCCTGAATCC-1 Patient5 Monocytes nonactive 0 0.05004439 Monocytes_0
10x_3288_t1_AGCTGTGAGGAGTG-1 Patient5 Monocytes nonactive 4 0.08566356 Monocytes_4
10x_3288_t1_AGCTGTGAGGTTAC-1 Patient5 Monocytes nonactive 4 0.04500349 Monocytes_4
10x_3288_t1_AGCTTACTACTACG-1 Patient5 Monocytes nonactive 0 0.01469469 Monocytes_0
10x_3288_t1_AGCTTACTAGTTCG-1 Patient5 Progenitors active 1 0.18942997 Progenitors_1
10x_3288_t1_AGCTTACTCGAACT-1 Patient5 Monocytes nonactive 4 0.02409486 Monocytes_4
10x_3288_t1_AGCTTACTCTGGTA-1 Patient5 Progenitors active 1 0.14437892 Progenitors_1
10x_3288_t1_AGCTTACTGATAAG-1 Patient5 Monocytes nonactive 4 0.07903747 Monocytes_4
10x_3288_t1_AGCTTACTGCTGTA-1 Patient5 Monocytes nonactive 0 0.03430347 Monocytes_0
10x_3288_t1_AGCTTACTGTTGCA-1 Patient5 Monocytes nonactive 0 0.05430854 Monocytes_0
10x_3288_t1_AGCTTACTTGCTAG-1 Patient5 Monocytes nonactive 0 0.04753979 Monocytes_0
10x_3288_t1_AGCTTTACATACCG-1 Patient5 Monocytes nonactive 0 0.04205504 Monocytes_0
10x_3288_t1_AGCTTTACTATTCC-1 Patient5 Progenitors active 1 0.14753345 Progenitors_1
10x_3288_t1_AGCTTTACTCCCAC-1 Patient5 Monocytes nonactive 4 0.04879209 Monocytes_4
10x_3288_t1_AGCTTTACTGACCA-1 Patient5 Monocytes nonactive 4 0.00894046 Monocytes_4
10x_3288_t1_AGGAAATGCCTGAA-1 Patient5 Progenitors nonactive 1 0.09324076 Progenitors_1
10x_3288_t1_AGGAAATGGGGTGA-1 Patient5 Monocytes nonactive 0 0.05166128

Monocytes_0

10x_3288_t1_AGGAAATGTCTACT-1 Patient5 Monocytes nonactive 0 0.04081859 Monocytes_0

10x_3288_t1_AGGAATGACGACTA-1 Patient5 Progenitors nonactive 1 0.08361867 Progenitors_1

10x_3288_t1_AGGAATGAGCAGAG-1 Patient5 Monocytes nonactive 4 0.09725128 Monocytes_4

10x_3288_t1_AGGAATGAGCTATG-1 Patient5 Progenitors nonactive 1 0.11178746 Progenitors_1

10x_3288_t1_AGGAATGAGGGTGA-1 Patient5 Progenitors active 1 0.12962082 Progenitors_1

10x_3288_t1_AGGACACTCATTCT-1 Patient5 Monocytes nonactive 0 0.04576438 Monocytes_0

10x_3288_t1_AGGACTTGGCTAAC-1 Patient5 Monocytes nonactive 0 0.02915161 Monocytes_0

10x_3288_t1_AGGACTTGTGCAAC-1 Patient5 Progenitors nonactive 1 0.09438209 Progenitors_1

10x_3288_t1_AGGACTTGTGTGCA-1 Patient5 Monocytes nonactive 0 0.02055989 Monocytes_0

10x_3288_t1_AGGACTTGTTCACT-1 Patient5 Monocytes nonactive 8 0.04511445 Monocytes_8

10x_3288_t1_AGGAGTCTCAAGCT-1 Patient5 Progenitors active 1 0.15317672 Progenitors_1

10x_3288_t1_AGGAGTCTCTAGTG-1 Patient5 Monocytes nonactive 4 0.06851183 Monocytes_4

10x_3288_t1_AGGAGTCTTGCCAA-1 Patient5 Monocytes nonactive 4 0.0792911 Monocytes_4

10x_3288_t1_AGGAGTCTTTTCAC-1 Patient5 Progenitors active 1 0.11915858 Progenitors_1

10x_3288_t1_AGGATGCTGTACAC-1 Patient5 Monocytes nonactive 4 0.01930759 Monocytes_4

10x_3288_t1_AGGATGCTGTGTAC-1 Patient5 Monocytes nonactive 0 0.03221102 Monocytes_0

10x_3288_t1_AGGATGCTTGTAGC-1 Patient5 Monocytes nonactive 4 0.02950035 Monocytes_4
10x_3288_t1_AGGCAACTCTGTCC-1 Patient5 Monocytes nonactive 4 0.06133092 Monocytes_4
10x_3288_t1_AGGCAACTTTGTCT-1 Patient5 Monocytes nonactive 4 0.02891383 Monocytes_4
10x_3288_t1_AGGCAGGACCTTAT-1 Patient5 Progenitors active 6 0.20758037 Progenitors_6
10x_3288_t1_AGGCAGGACTGAGT-1 Patient5 Monocytes nonactive 0 0.05254898 Monocytes_0
10x_3288_t1_AGGCAGGAGCTTAG-1 Patient5 Monocytes nonactive 0 0.03446199 Monocytes_0
10x_3288_t1_AGGCAGGATCAGGT-1 Patient5 Progenitors active 1 0.16116606 Progenitors_1
10x_3288_t1_AGGCAGGATTCAGG-1 Patient5 Progenitors active 1 0.17557542 Progenitors_1
10x_3288_t1_AGGCAGGATTCCGC-1 Patient5 Monocytes nonactive 0 0.03598377 Monocytes_0
10x_3288_t1_AGGCAGGATTCGCC-1 Patient5 Monocytes nonactive 0 0.08372963 Monocytes_0
10x_3288_t1_AGGCCTCTGTTTCT-1Patient5 Monocytes nonactive 0 0.05143935 Monocytes_0
10x_3288_t1_AGGCCTCTTAACGC-1 Patient5 Monocytes nonactive 0 0.08688415 Monocytes_0
10x_3288_t1_AGGCCTCTTCATTC-1 Patient5 Progenitors active 1 0.17322935 Progenitors_1
10x_3288_t1_AGGCCTCTTTCCGC-1 Patient5 Monocytes nonactive 8 0.05061505 Monocytes_8
10x_3288_t1_AGGCTAACAGCACT-1 Patient5 Progenitors active 1 0.1361518 Progenitors_1
10x_3288_t1_AGGCTAACCCTTGC-1 Patient5 Monocytes nonactive 8 0.03508021 Monocytes_8
10x_3288_t1_AGGCTAACGTCGAT-1 Patient5 Monocytes nonactive 4 0.04330734

Monocytes_4
10x_3288_t1_AGGCTAACTACGAC-1 Patient5 Monocytes nonactive 4 0.02715427
Monocytes_4
10x_3288_t1_AGGCTAACTGTTCT-1 Patient5 Progenitors active 1 0.16419377
Progenitors_1
10x_3288_t1_AGGGACGAAAGTGA-1 Patient5 Dendritic cells active 10 0.13561283
Dendritic cells_10
10x_3288_t1_AGGGACGAAGCATC-1 Patient5 Monocytes nonactive 4 0.03127576
Monocytes_4
10x_3288_t1_AGGGACGACCAGTA-1 Patient5 Progenitors active 1 0.14488618
Progenitors_1
10x_3288_t1_AGGGACGACTCCCA-1 Patient5 Progenitors active 1 0.12071207
Progenitors_1
10x_3288_t1_AGGGACGACTTGCC-1 Patient5 Monocytes nonactive 0 0.0472386
Monocytes_0
10x_3288_t1_AGGGACGAGTTGCA-1 Patient5 Progenitors nonactive 1 0.10530404
Progenitors_1
10x_3288_t1_AGGGACGATAGAGA-1 Patient5 Monocytes nonactive 0 0.04354511
Monocytes_0
10x_3288_t1_AGGGAGTGTGCGTA-1 Patient5 Monocytes nonactive 4 0.040026
Monocytes_4
10x_3288_t1_AGGGCCACCCAACA-1 Patient5 Monocytes nonactive 4 0.03818718
Monocytes_4
10x_3288_t1_AGGGCCACCCAATG-1 Patient5 Progenitors nonactive 1 0.07634265
Progenitors_1
10x_3288_t1_AGGGCCTGAGATCC-1 Patient5 Monocytes nonactive 0 0.03249635
Monocytes_0
10x_3288_t1_AGGGCCTGTTCCCG-1 Patient5 Monocytes nonactive 0 0.06718027
Monocytes_0
10x_3288_t1_AGGGCGCTAGAATG-1 Patient5 Progenitors active 1 0.11938051
Progenitors_1
10x_3288_t1_AGGGCGCTTGTCAG-1 Patient5 Monocytes nonactive 4 0.07182487
Monocytes_4

10x_3288_t1_AGGGTGGATCTTCA-1 Patient5 Monocytes nonactive 8 0.05258069
Monocytes_8
10x_3288_t1_AGGGTGGATGAACC-1 Patient5 Monocytes nonactive 4 0.01523366
Monocytes_4
10x_3288_t1_AGGGTGGATGCCCT-1 Patient5 Monocytes nonactive 4 0.03557162
Monocytes_4
10x_3288_t1_AGGGTGGATTCCCG-1 Patient5 Monocytes nonactive 3 0.0539915
Monocytes_3
10x_3288_t1_AGGGTTTGCGAACT-1 Patient5 Progenitors active 6 0.15365227
Progenitors_6
10x_3288_t1_AGGGTTTGGGCGAA-1 Patient5 Progenitors active 1 0.12061696
Progenitors_1
10x_3288_t1_AGGGTTTGTACTGG-1 Patient5 Monocytes nonactive 4 0.03316213
Monocytes_4
10x_3288_t1_AGGGTTTGTCCGTC-1 Patient5 Monocytes nonactive 4 0.07141272
Monocytes_4
10x_3288_t1_AGGTACACCTAGCA-1 Patient5 Progenitors active 1 0.14258766
Progenitors_1
10x_3288_t1_AGGTACACTCCAGA-1 Patient5 Monocytes nonactive 0 0.09268594
Monocytes_0
10x_3288_t1_AGGTACACTGCAAC-1 Patient5 Monocytes nonactive 4 0.00724431
Monocytes_4
10x_3288_t1_AGGTACTGCCCAAA-1 Patient5 Monocytes nonactive 4 0.04773001
Monocytes_4
10x_3288_t1_AGGTACTGTATCGG-1 Patient5 Progenitors nonactive 5 0.05686069
Progenitors_5
10x_3288_t1_AGGTCATGCGAACT-1 Patient5 Monocytes nonactive 4 0.0603164
Monocytes_4
10x_3288_t1_AGGTCTGACCTTAT-1Patient5 Progenitors active 1 0.13680172
Progenitors_1
10x_3288_t1_AGGTCTGAGTCTAG-1 Patient5 Monocytes nonactive 15 0.05235876
Monocytes_15
10x_3288_t1_AGGTCTGATCAGTG-1 Patient5 Progenitors active 1 0.14612263

Progenitors_1

10x_3288_t1_AGGTGGGACACTAG-1 Patient5 Monocytes nonactive 8 0.03094287 Monocytes_8

10x_3288_t1_AGGTGGGACTCCCA-1 Patient5 Monocytes nonactive 4 0.10909264 Monocytes_4

10x_3288_t1_AGGTGGGATTATCC-1 Patient5 Progenitors active 1 0.12109251 Progenitors_1

10x_3288_t1_AGGTGGGATTCGTT-1 Patient5 Monocytes nonactive 0 0.04842749 Monocytes_0

10x_3288_t1_AGGTGTTGCGATAC-1 Patient5 Monocytes nonactive 0 0.0374897 Monocytes_0

10x_3288_t1_AGGTTCGAACTACG-1 Patient5 Monocytes nonactive 0 0.01719929 Monocytes_0

10x_3288_t1_AGGTTCGAAGACTC-1 Patient5 Monocytes nonactive 0 0.10929871 Monocytes_0

10x_3288_t1_AGGTTCGAAGCAAA-1 Patient5 Monocytes nonactive 4 0.02945279 Monocytes_4

10x_3288_t1_AGGTTCGACTGTAG-1 Patient5 Progenitors active 1 0.20230169 Progenitors_1

10x_3288_t1_AGGTTCGAGTGTAC-1 Patient5 Monocytes nonactive 4 0.05218439 Monocytes_4

10x_3288_t1_AGGTTGTGACGCAT-1 Patient5 Monocytes nonactive 0 0.03378036 Monocytes_0

10x_3288_t1_AGGTTGTGGGAGGT-1 Patient5 Progenitors active 1 0.19023841 Progenitors_1

10x_3288_t1_AGGTTGTGTGTGCA-1 Patient5 Monocytes nonactive 0 0.04744468 Monocytes_0

10x_3288_t1_AGTAAGGAACACGT-1 Patient5 Monocytes nonactive 0 0.05579862 Monocytes_0

10x_3288_t1_AGTAAGGAAGCGTT-1 Patient5 Progenitors active 1 0.14892841 Progenitors_1

10x_3288_t1_AGTAAGGAGACACT-1 Patient5 Progenitors active 1 0.1424767 Progenitors_1

10x_3288_t1_AGTAAGGAGGAGGT-1 Patient5 Monocytes nonactive 0 0.03671295 Monocytes_0

10x_3288_t1_AGTAAGGAGGAGTG-1 Patient5 Progenitors active 1 0.15186101 Progenitors_1

10x_3288_t1_AGTAAGGAGGCAAG-1 Patient5 Monocytes nonactive 4 0.04370363 Monocytes_4

10x_3288_t1_AGTAAGGAGGTAAA-1 Patient5 Monocytes nonactive 4 0.0531355 Monocytes_4

10x_3288_t1_AGTAAGGAGTAAGA-1 Patient5 Monocytes nonactive 4 0.00088771 Monocytes_4

10x_3288_t1_AGTAATACAGCATC-1 Patient5 Monocytes nonactive 0 0.05372202 Monocytes_0

10x_3288_t1_AGTAATACCCAGTA-1 Patient5 Monocytes nonactive 0 0.01933929 Monocytes_0

10x_3288_t1_AGTAATTGGCGTTA-1 Patient5 Monocytes nonactive 4 0.04077104 Monocytes_4

10x_3288_t1_AGTAATTGTAACCG-1 Patient5 Progenitors nonactive 1 0.104559 Progenitors_1

10x_3288_t1_AGTACGTGCGGGAA-1 Patient5 Monocytes nonactive 13 0.01277662 Monocytes_13

10x_3288_t1_AGTACGTGCGTAGT-1 Patient5 Monocytes nonactive 4 0.02552153 Monocytes_4

10x_3288_t1_AGTACGTGCGTCTC-1 Patient5 Monocytes nonactive 8 0.03890051 Monocytes_8

10x_3288_t1_AGTACGTGCTTAGG-1 Patient5 Progenitors nonactive 1 0.11269102 Progenitors_1

10x_3288_t1_AGTACGTGTCAGGT-1 Patient5 Progenitors active 1 0.13919536 Progenitors_1

10x_3288_t1_AGTACGTGTTGTCT-1 Patient5 Monocytes nonactive 0 0.00548475 Monocytes_0

10x_3288_t1_AGTACTCTATCACG-1 Patient5 Monocytes nonactive 0 0.06960561 Monocytes_0

10x_3288_t1_AGTACTCTGCTGTA-1 Patient5 Monocytes nonactive 0 0.07987762

Monocytes_0

10x_3288_t1_AGTACTCTTGCGTA-1 Patient5 Monocytes nonactive 0 0.05178809

Monocytes_0

10x_3288_t1_AGTACTCTTGTCAG-1 Patient5 Monocytes nonactive 4 0.0333048

Monocytes_4

10x_3288_t1_AGTAGAGATAAGCC-1 Patient5 Progenitors nonactive 1 0.08612326

Progenitors_1

10x_3288_t1_AGTAGGCTATCTCT-1 Patient5 Monocytes nonactive 0 0.06129922

Monocytes_0

10x_3288_t1_AGTAGGCTCAGCTA-1 Patient5 Monocytes nonactive 0 0.0535635

Monocytes_0

10x_3288_t1_AGTATAACACGGTT-1 Patient5 Monocytes nonactive 0 0.0998827

Monocytes_0

10x_3288_t1_AGTATAACCCTTGC-1 Patient5 Monocytes nonactive 0 0.03457295

Monocytes_0

10x_3288_t1_AGTATCCTAGCACT-1 Patient5 Progenitors active 1 0.15953332

Progenitors_1

10x_3288_t1_AGTATCCTGGTGTT-1 Patient5 Progenitors active 1 0.21531609

Progenitors_1

10x_3288_t1_AGTATCCTTTCATC-1 Patient5 Monocytes nonactive 4 0.02602879

Monocytes_4

10x_3288_t1_AGTCACGAGTTGAC-1 Patient5 Progenitors active 1 0.16141969

Progenitors_1

10x_3288_t1_AGTCAGACAAGGGC-1 Patient5 Monocytes nonactive 4 0.01666033

Monocytes_4

10x_3288_t1_AGTCAGACAGATGA-1 Patient5 Monocytes nonactive 0 0.08244563

Monocytes_0

10x_3288_t1_AGTCAGACTGGTAC-1 Patient5 Monocytes nonactive 4 0.04993342

Monocytes_4

10x_3288_t1_AGTCCAGAATCAGC-1 Patient5 Monocytes nonactive 0 0.04585949

Monocytes_0

10x_3288_t1_AGTCCAGATAGCCA-1 Patient5 Progenitors active 1 0.15308161

Progenitors_1

10x_3288_t1_AGTCCAGATTCATC-1 Patient5 Monocytes nonactive 0 0.0290565 Monocytes_0
10x_3288_t1_AGTCGAACCTCGCT-1 Patient5 Monocytes nonactive 0 0.0560364 Monocytes_0
10x_3288_t1_AGTCGAACGCTAAC-1 Patient5 Progenitors active 1 0.14720056 Progenitors_1
10x_3288_t1_AGTCGCCTAAGGTA-1 Patient5 Progenitors nonactive 1 0.10021559 Progenitors_1
10x_3288_t1_AGTCGCCTCGAATC-1 Patient5 Progenitors active 1 0.14257181 Progenitors_1
10x_3288_t1_AGTCGCCTCGTCTC-1 Patient5 Monocytes nonactive 8 0.07607317 Monocytes_8
10x_3288_t1_AGTCGCCTGTTTGG-1 Patient5 Progenitors active 1 0.16224399 Progenitors_1
10x_3288_t1_AGTCGCCTTCTTAC-1 Patient5 Monocytes nonactive 4 0.03059413 Monocytes_4
10x_3288_t1_AGTCTACTATCTCT-1 Patient5 Monocytes nonactive 4 0.04562171 Monocytes_4
10x_3288_t1_AGTCTACTCTTGAG-1 Patient5 Progenitors nonactive 1 0.09771099 Progenitors_1
10x_3288_t1_AGTCTACTGTCGAT-1Patient5 Monocytes nonactive 0 0.0568924 Monocytes_0
10x_3288_t1_AGTCTACTTATGGC-1Patient5 Monocytes nonactive 4 0.02598123 Monocytes_4
10x_3288_t1_AGTCTACTTGCAGT-1 Patient5 Monocytes nonactive 0 0.04248304 Monocytes_0
10x_3288_t1_AGTCTACTTGTCAG-1 Patient5 Monocytes nonactive 0 0.0370934 Monocytes_0
10x_3288_t1_AGTCTTACATGCTG-1 Patient5 Progenitors active 1 0.13860884 Progenitors_1
10x_3288_t1_AGTCTTACCTTACT-1 Patient5 Monocytes nonactive 8 0.02794686 Monocytes_8
10x_3288_t1_AGTCTTACGCGATT-1Patient5 Progenitors active 1 0.14542515

Progenitors_1

10x_3288_t1_AGTGAAGACTTACT-1 Patient5 Monocytes nonactive 8 0.0876926 Monocytes_8

10x_3288_t1_AGTGAAGACTTCTA-1 Patient5 Monocytes nonactive 0 0.08526726 Monocytes_0

10x_3288_t1_AGTGAAGAGTTCAG-1 Patient5 Monocytes nonactive 0 0.02764568 Monocytes_0

10x_3288_t1_AGTGAAGATTCTCA-1 Patient5 Monocytes nonactive 0 0.04742883 Monocytes_0

10x_3288_t1_AGTGACACACGACT-1 Patient5 Monocytes nonactive 4 0.06619745 Monocytes_4

10x_3288_t1_AGTGACACCTATTC-1 Patient5 Monocytes nonactive 4 0.04830068 Monocytes_4

10x_3288_t1_AGTGACACCTGCTC-1 Patient5 Monocytes nonactive 4 0.04092955 Monocytes_4

10x_3288_t1_AGTGACACTGCTAG-1 Patient5 Progenitors active 1 0.14171581 Progenitors_1

10x_3288_t1_AGTGACTGCTCGAA-1 Patient5 Monocytes nonactive 4 0.02883457 Monocytes_4

10x_3288_t1_AGTGACTGCTGCTC-1 Patient5 Monocytes nonactive 0 0.03852007 Monocytes_0

10x_3288_t1_AGTGACTGGAATGA-1 Patient5 Progenitors nonactive 1 0.09672817 Progenitors_1

10x_3288_t1_AGTGCAACTTAGGC-1 Patient5 Progenitors nonactive 1 0.11403842 Progenitors_1

10x_3288_t1_AGTGTGACTAGAGA-1 Patient5 Monocytes nonactive 0 0.04107222 Monocytes_0

10x_3288_t1_AGTGTGACTCGTAG-1 Patient5 Monocytes nonactive 4 0.0463826 Monocytes_4

10x_3288_t1_AGTGTTCTCCTCGT-1Patient5 Monocytes nonactive 0 0.02063915 Monocytes_0

10x_3288_t1_AGTTAAACGCGTTA-1 Patient5 Monocytes nonactive 0 0.02214508 Monocytes_0

10x_3288_t1_AGTTAAACGTGAGG-1 Patient5 Monocytes nonactive 4 0.03189398 Monocytes_4
10x_3288_t1_AGTTATGAAAACGA-1 Patient5 Progenitors nonactive 1 0.10089722 Progenitors_1
10x_3288_t1_AGTTATGACTGTAG-1 Patient5 Dendritic cells nonactive 10 0.10016803 Dendritic cells_10
10x_3288_t1_AGTTATGATTACCT-1 Patient5 Monocytes nonactive 4 0.04194407 Monocytes_4
10x_3288_t1_AGTTCTACACGTTG-1 Patient5 Progenitors nonactive 1 0.10582715 Progenitors_1
10x_3288_t1_AGTTCTACCTACGA-1 Patient5 Monocytes nonactive 4 0.04687401 Monocytes_4
10x_3288_t1_AGTTCTACGGTGAG-1 Patient5 Progenitors nonactive 1 0.11565532 Progenitors_1
10x_3288_t1_AGTTCTTGCACTCC-1 Patient5 Monocytes nonactive 15 0.04787268 Monocytes_15
10x_3288_t1_AGTTCTTGCACTTT-1 Patient5 Monocytes nonactive 0 0.03468391 Monocytes_0
10x_3288_t1_AGTTCTTGCTGTCC-1 Patient5 Monocytes nonactive 4 0.04218185 Monocytes_4
10x_3288_t1_AGTTCTTGTGTCAG-1 Patient5 Monocytes nonactive 4 0.06659375 Monocytes_4
10x_3288_t1_AGTTGTCTCCGTAA-1 Patient5 Monocytes nonactive 0 0.01967218 Monocytes_0
10x_3288_t1_AGTTGTCTCTGAGT-1 Patient5 Progenitors nonactive 1 0.11446643 Progenitors_1
10x_3288_t1_AGTTGTCTGATAAG-1 Patient5 Progenitors active 1 0.14355463 Progenitors_1
10x_3288_t1_AGTTGTCTGGAAAT-1 Patient5 Monocytes nonactive 0 0.04696912 Monocytes_0
10x_3288_t1_AGTTGTCTTGCTTT-1 Patient5 Monocytes nonactive 0 0.09736225 Monocytes_0
10x_3288_t1_AGTTGTCTTTGCAG-1 Patient5 Monocytes nonactive 4 0.03351087

Monocytes_4
10x_3288_t1_AGTTTAGAGGGTGA-1 Patient5 Progenitors active 1 0.11955488
Progenitors_1
10x_3288_t1_AGTTTAGATACGAC-1 Patient5 Monocytes nonactive 0 0.03631666
Monocytes_0
10x_3288_t1_AGTTTCACAACTGC-1 Patient5 Monocytes nonactive 4 0.03143428
Monocytes_4
10x_3288_t1_AGTTTGCTGGGATG-1 Patient5 Progenitors active 1 0.16156236
Progenitors_1
10x_3288_t1_AGTTTGCTTGTCGA-1 Patient5 Monocytes nonactive 0 0.03936022
Monocytes_0
10x_3288_t1_ATAACAACCCTCCA-1 Patient5 Monocytes nonactive 0 0.06202841
Monocytes_0
10x_3288_t1_ATAACAACGAAGGC-1 Patient5 Progenitors active 1 0.15389005
Progenitors_1
10x_3288_t1_ATAACAACGGACAG-1 Patient5 Monocytes nonactive 0 0.0535318
Monocytes_0
10x_3288_t1_ATAACAACGGGCAA-1 Patient5 Monocytes nonactive 4 0.05410247
Monocytes_4
10x_3288_t1_ATAACAACTGTCTT-1Patient5 Monocytes nonactive 4 0.05460973
Monocytes_4
10x_3288_t1_ATAACATGCCATGA-1 Patient5 Monocytes nonactive 0 0.03043561
Monocytes_0
10x_3288_t1_ATAACATGCCCTCA-1 Patient5 Dendritic cells nonactive 10 0.10154714
Dendritic cells_10
10x_3288_t1_ATAACATGCGTTAG-1 Patient5 Monocytes nonactive 0 0.02594953
Monocytes_0
10x_3288_t1_ATAACATGCTTAGG-1 Patient5 Monocytes nonactive 0 0.01933929
Monocytes_0
10x_3288_t1_ATAACCCTAGTAGA-1 Patient5 Monocytes nonactive 0 0.04609727
Monocytes_0
10x_3288_t1_ATAACCCTAGTGTC-1 Patient5 Monocytes nonactive 4 0.04662038
Monocytes_4

10x_3288_t1_ATAACCCTATCGGT-1	Patient5	Progenitors	nonactive	1	0.10283115	Progenitors_1
10x_3288_t1_ATAACCCTCGGAGA-1	Patient5	Progenitors	active	1	0.16124532	Progenitors_1
10x_3288_t1_ATAACCCTCTCAAG-1	Patient5	Progenitors	active	1	0.14227062	Progenitors_1
10x_3288_t1_ATAACCCTGCGGAA-1	Patient5	Monocytes	nonactive	16	0.03086361	Monocytes_16
10x_3288_t1_ATAACCCTTGAAGA-1	Patient5	Progenitors	active	1	0.16143555	Progenitors_1
10x_3288_t1_ATAACCCTTTGAGC-1	Patient5	Progenitors	active	1	0.15298649	Progenitors_1
10x_3288_t1_ATAAGTACACGCAT-1	Patient5	Progenitors	active	1	0.14158899	Progenitors_1
10x_3288_t1_ATAAGTACAGTAGA-1	Patient5	Progenitors	active	1	0.15606176	Progenitors_1
10x_3288_t1_ATAAGTACCACTAG-1	Patient5	Progenitors	active	1	0.16714222	Progenitors_1
10x_3288_t1_ATAAGTACCCTTCG-1	Patient5	Monocytes	nonactive	15	0.04877623	Monocytes_15
10x_3288_t1_ATAAGTACCTGGAT-1	Patient5	Monocytes	nonactive	0	0.03492169	Monocytes_0
10x_3288_t1_ATAAGTACGAATCC-1	Patient5	Progenitors	active	1	0.13505802	Progenitors_1
10x_3288_t1_ATAAGTACGACGTT-1	Patient5	Monocytes	nonactive	0	0.06212352	Monocytes_0
10x_3288_t1_ATAAGTTGCCCTCA-1	Patient5	Progenitors	active	1	0.12667237	Progenitors_1
10x_3288_t1_ATAAGTTGGTTCGA-1	Patient5	Monocytes	nonactive	4	0.05174054	Monocytes_4
10x_3288_t1_ATAAGTTGTTGTGG-1	Patient5	Monocytes	nonactive	0	0.01415573	Monocytes_0
10x_3288_t1_ATAATCGATTGGCA-1	Patient5	Monocytes	nonactive	4	0.01737366	

Monocytes_4
10x_3288_t1_ATAATGACAGCTCA-1 Patient5 Progenitors active 1 0.14921375
Progenitors_1
10x_3288_t1_ATAATGACTCCGAA-1 Patient5 Monocytes nonactive 0 0.040026
Monocytes_0
10x_3288_t1_ATACAATGCTCCCA-1 Patient5 Monocytes nonactive 0 0.0560681
Monocytes_0
10x_3288_t1_ATACAATGGGTCAT-1 Patient5 Monocytes nonactive 4 0.04633505
Monocytes_4
10x_3288_t1_ATACACCTAATGCC-1 Patient5 Progenitors nonactive 1 0.08967409
Progenitors_1
10x_3288_t1_ATACACCTATTCTC-1 Patient5 Monocytes nonactive 8 0.04085029
Monocytes_8
10x_3288_t1_ATACACCTGCAGAG-1 Patient5 Monocytes nonactive 0 0.02919916
Monocytes_0
10x_3288_t1_ATACCACTAATGCC-1 Patient5 Monocytes nonactive 4 0.10551011
Monocytes_4
10x_3288_t1_ATACCACTGGAAAT-1 Patient5 Monocytes nonactive 0 0.02563249
Monocytes_0
10x_3288_t1_ATACCACTTACGCA-1 Patient5 Progenitors active 1 0.15395346
Progenitors_1
10x_3288_t1_ATACCGGACATCAG-1 Patient5 Monocytes nonactive 0 0.03634836
Monocytes_0
10x_3288_t1_ATACCGGAGAATGA-1 Patient5 Progenitors active 1 0.1365798
Progenitors_1
10x_3288_t1_ATACCTACGTTGAC-1 Patient5 Monocytes nonactive 0 0.043022
Monocytes_0
10x_3288_t1_ATACCTACTCTACT-1 Patient5 Monocytes nonactive 4 0.02962716
Monocytes_4
10x_3288_t1_ATACCTACTGGGAG-1 Patient5 Monocytes nonactive 0 0.04148437
Monocytes_0
10x_3288_t1_ATACCTTGCAATCG-1 Patient5 Monocytes nonactive 4 0.03246465
Monocytes_4

10x_3288_t1_ATACCTTGCAGGAG-1 Patient5 Dendritic cells active 10 0.12684674 Dendritic cells_10
10x_3288_t1_ATACCTTGCTCATT-1 Patient5 Progenitors active 1 0.16153066 Progenitors_1
10x_3288_t1_ATACCTTGCTTATC-1 Patient5 Monocytes nonactive 4 0.00355082 Monocytes_4
10x_3288_t1_ATACCTTGGAATCC-1 Patient5 Monocytes nonactive 8 0.07236383 Monocytes_8
10x_3288_t1_ATACCTTGGTAGCT-1 Patient5 Monocytes nonactive 0 0.05367447 Monocytes_0
10x_3288_t1_ATACCTTGGTCCTC-1 Patient5 Progenitors active 1 0.15534842 Progenitors_1
10x_3288_t1_ATACGGACCCGCTT-1 Patient5 Monocytes nonactive 4 0.0594287 Monocytes_4
10x_3288_t1_ATACGGACTGAACC-1 Patient5 Progenitors active 1 0.18031514 Progenitors_1
10x_3288_t1_ATACGGACTGCCTC-1 Patient5 Monocytes nonactive 13 0.07467821 Monocytes_13
10x_3288_t1_ATACGTCTATCGTG-1 Patient5 Monocytes nonactive 0 0.04836409 Monocytes_0
10x_3288_t1_ATACGTCTATTGGC-1 Patient5 Monocytes nonactive 4 0.01287173 Monocytes_4
10x_3288_t1_ATACGTCTCGTCTC-1 Patient5 Progenitors active 1 0.12656141 Progenitors_1
10x_3288_t1_ATACGTCTCTCAGA-1 Patient5 Progenitors active 1 0.15869317 Progenitors_1
10x_3288_t1_ATACGTCTGGAAGC-1 Patient5 Monocytes nonactive 0 0.05979329 Monocytes_0
10x_3288_t1_ATACGTCTTGCACA-1 Patient5 Monocytes nonactive 0 0.03733118 Monocytes_0
10x_3288_t1_ATACTCTGAAGGGC-1 Patient5 Monocytes nonactive 0 0.05527551 Monocytes_0
10x_3288_t1_ATACTCTGCGTACA-1 Patient5 Dendritic cells nonactive 10 0.08989601

Dendritic cells_10
10x_3288_t1_ATAGAACTAGAGAT-1 Patient5 Progenitors active 1 0.17969691
Progenitors_1
10x_3288_t1_ATAGAACTGCAGAG-1 Patient5 Monocytes nonactive 4 0.05713018
Monocytes_4
10x_3288_t1_ATAGAACTTAGAAG-1 Patient5 Progenitors active 1 0.16043688
Progenitors_1
10x_3288_t1_ATAGAACTTCTATC-1 Patient5 Monocytes nonactive 0 0.04099296
Monocytes_0
10x_3288_t1_ATAGATACATGCTG-1 Patient5 Monocytes nonactive 0 0.03720436
Monocytes_0
10x_3288_t1_ATAGATACGGAGCA-1 Patient5 Monocytes nonactive 0 0.06840086
Monocytes_0
10x_3288_t1_ATAGATTGGGATTC-1 Patient5 Monocytes nonactive 4 0.05570351
Monocytes_4
10x_3288_t1_ATAGATTGTCCCGT-1 Patient5 Progenitors active 6 0.12779786
Progenitors_6
10x_3288_t1_ATAGCCGAGTGCTA-1 Patient5 Monocytes nonactive 0 0.0577484
Monocytes_0
10x_3288_t1_ATAGCCGATCCTTA-1 Patient5 Monocytes nonactive 8 0.02132078
Monocytes_8
10x_3288_t1_ATAGCGTGACCAAC-1 Patient5 Dendritic cells nonactive 10 0.0721102
Dendritic cells_10
10x_3288_t1_ATAGCGTGGCGTAT-1 Patient5 Monocytes nonactive 0 0.01184135
Monocytes_0
10x_3288_t1_ATAGCGTGGGACGA-1 Patient5 Monocytes nonactive 4 0.01796018
Monocytes_4
10x_3288_t1_ATAGCGTGTCCTGC-1 Patient5 Monocytes nonactive 0 0.04001015
Monocytes_0
10x_3288_t1_ATAGCTCTAAAACG-1 Patient5 Progenitors active 1 0.1656997
Progenitors_1
10x_3288_t1_ATAGCTCTCACTGA-1 Patient5 Monocytes nonactive 4 0.04628749
Monocytes_4

10x_3288_t1_ATAGCTCTCCACAA-1 Patient5 Monocytes nonactive 4 0.05979329
Monocytes_4
10x_3288_t1_ATAGCTCTCCCTAC-1 Patient5 Monocytes nonactive 4 0.07320398
Monocytes_4
10x_3288_t1_ATAGCTCTCCTACC-1 Patient5 Monocytes nonactive 8 0.0257276
Monocytes_8
10x_3288_t1_ATAGCTCTCTGACA-1 Patient5 Monocytes nonactive 0 0.03677636
Monocytes_0
10x_3288_t1_ATAGCTCTGTGTTG-1 Patient5 Monocytes nonactive 15 0.06981168
Monocytes_15
10x_3288_t1_ATAGCTCTGTTGCA-1 Patient5 Monocytes nonactive 0 0.04142096
Monocytes_0
10x_3288_t1_ATAGCTCTTGGATC-1 Patient5 Monocytes nonactive 15 0.03774333
Monocytes_15
10x_3288_t1_ATAGCTCTTTCATC-1 Patient5 Monocytes nonactive 0 0.03443028
Monocytes_0
10x_3288_t1_ATAGGAGACCGATA-1 Patient5 Progenitors nonactive 1 0.11021812
Progenitors_1
10x_3288_t1_ATAGGAGACTCTCG-1 Patient5 Monocytes nonactive 0 0.06217107
Monocytes_0
10x_3288_t1_ATAGGAGATGCTCC-1 Patient5 Monocytes nonactive 8 0.06635597
Monocytes_8
10x_3288_t1_ATAGGCTGAAGAAC-1 Patient5 Progenitors active 1 0.17755691
Progenitors_1
10x_3288_t1_ATAGGCTGAGCGGA-1 Patient5 Progenitors active 1 0.12473844
Progenitors_1
10x_3288_t1_ATAGGCTGGCAGTT-1 Patient5 Monocytes nonactive 4 0.03230613
Monocytes_4
10x_3288_t1_ATAGGCTGTTGGTG-1 Patient5 Progenitors active 1 0.1555862
Progenitors_1
10x_3288_t1_ATAGGCTGTTTCTG-1 Patient5 Monocytes nonactive 0 0.06023714
Monocytes_0
10x_3288_t1_ATAGTCCTACACAC-1 Patient5 Monocytes nonactive 4 0.02956376

Monocytes_4

10x_3288_t1_ATAGTCCTTAGTCG-1Patient5 Monocytes nonactive 8 0.06884471 Monocytes_8

10x_3288_t1_ATAGTTGAGAGGAC-1 Patient5 Progenitors active 1 0.14106588 Progenitors_1

10x_3288_t1_ATAGTTGATTCAGG-1 Patient5 Progenitors active 1 0.13491535 Progenitors_1

10x_3288_t1_ATATACGACTGAGT-1Patient5 Progenitors nonactive 1 0.11581384 Progenitors_1

10x_3288_t1_ATATACGACTTGTT-1 Patient5 Monocytes nonactive 0 0.05901655 Monocytes_0

10x_3288_t1_ATATACGAGCAAGG-1 Patient5 Monocytes nonactive 0 0.06908249 Monocytes_0

10x_3288_t1_ATATAGTGAACTGC-1 Patient5 Monocytes nonactive 0 0.07282354 Monocytes_0

10x_3288_t1_ATATAGTGCTCATT-1 Patient5 Monocytes nonactive 4 0.05426099 Monocytes_4

10x_3288_t1_ATATAGTGTCAGAC-1 Patient5 Progenitors active 1 0.16081732 Progenitors_1

10x_3288_t1_ATATAGTGTCTAGG-1Patient5 Progenitors active 1 0.20526599 Progenitors_1

10x_3288_t1_ATATGAACGACAGG-1 Patient5 Monocytes nonactive 0 0.04620823 Monocytes_0

10x_3288_t1_ATATGAACGACGAG-1 Patient5 Monocytes nonactive 4 0.0598567 Monocytes_4

10x_3288_t1_ATATGAACGTATCG-1Patient5 Monocytes nonactive 8 0.02255723 Monocytes_8

10x_3288_t1_ATATGCCTTCAGTG-1Patient5 Progenitors active 1 0.13675417 Progenitors_1

10x_3288_t1_ATATGCCTTTCATC-1 Patient5 Monocytes nonactive 8 0.05787521 Monocytes_8

10x_3288_t1_ATCAAATGCACTGA-1 Patient5 Monocytes nonactive 0 0.03198909 Monocytes_0

10x_3288_t1_ATCAAATGCTCAAG-1 Patient5 Progenitors active 1 0.13672247 Progenitors_1
10x_3288_t1_ATCAACCTCCCACT-1 Patient5 Progenitors active 1 0.15198783 Progenitors_1
10x_3288_t1_ATCAACCTGAACCT-1 Patient5 Progenitors active 1 0.18370744 Progenitors_1
10x_3288_t1_ATCACACTAGCGTT-1 Patient5 Progenitors active 1 0.15734576 Progenitors_1
10x_3288_t1_ATCACACTCGAATC-1 Patient5 Monocytes nonactive 8 0.02667871 Monocytes_8
10x_3288_t1_ATCACACTGCTGTA-1 Patient5 Progenitors nonactive 1 0.11733562 Progenitors_1
10x_3288_t1_ATCACACTGGAAGC-1 Patient5 Progenitors nonactive 1 0.09620506 Progenitors_1
10x_3288_t1_ATCACACTGTAGCT-1 Patient5 Progenitors active 1 0.16038932 Progenitors_1
10x_3288_t1_ATCACACTGTCATG-1 Patient5 Progenitors active 1 0.14059032 Progenitors_1
10x_3288_t1_ATCACACTTGGAGG-1 Patient5 Progenitors active 1 0.1610551 Progenitors_1
10x_3288_t1_ATCACGGAACCGAT-1 Patient5 Monocytes nonactive 4 0.08509289 Monocytes_4
10x_3288_t1_ATCACGGATGACTG-1 Patient5 Monocytes nonactive 4 0.04706423 Monocytes_4
10x_3288_t1_ATCACGGATGCGTA-1 Patient5 Monocytes nonactive 4 0.03820303 Monocytes_4
10x_3288_t1_ATCACTACAAAAGC-1 Patient5 Monocytes nonactive 4 0.05302454 Monocytes_4
10x_3288_t1_ATCACTACAAACGA-1 Patient5 Monocytes nonactive 4 0.04108807 Monocytes_4
10x_3288_t1_ATCACTACATACCG-1Patient5 Monocytes nonactive 4 0.04121489 Monocytes_4
10x_3288_t1_ATCACTACCGAATC-1 Patient5 Progenitors nonactive 1 0.09059349

Progenitors_1

10x_3288_t1_ATCACTTGACTGTG-1 Patient5 Monocytes nonactive 4 0.0560998 Monocytes_4

10x_3288_t1_ATCACTTGCAATCG-1 Patient5 Progenitors active 1 0.20870585 Progenitors_1

10x_3288_t1_ATCACTTGCCGAAT-1Patient5 Monocytes nonactive 13 0.040882 Monocytes_13

10x_3288_t1_ATCACTTGGACGGA-1 Patient5 Monocytes nonactive 4 0.05592543 Monocytes_4

10x_3288_t1_ATCACTTGTGCCCT-1Patient5 Monocytes nonactive 4 0.04698497 Monocytes_4

10x_3288_t1_ATCACTTGTTGTCT-1 Patient5 Monocytes nonactive 8 0.08241392 Monocytes_8

10x_3288_t1_ATCAGGTGAGACTC-1 Patient5 Monocytes nonactive 0 0.02846998 Monocytes_0

10x_3288_t1_ATCAGGTGCTTTAC-1 Patient5 Progenitors active 1 0.16942489 Progenitors_1

10x_3288_t1_ATCAGGTGGGTGAG-1 Patient5 Monocytes nonactive 0 0.00637246 Monocytes_0

10x_3288_t1_ATCAGGTGGTAAAG-1 Patient5 Monocytes nonactive 0 0.05132839 Monocytes_0

10x_3288_t1_ATCATCTGGCATCA-1Patient5 Monocytes nonactive 0 0.03530214 Monocytes_0

10x_3288_t1_ATCATCTGTCATTC-1 Patient5 Monocytes nonactive 8 0.03127576 Monocytes_8

10x_3288_t1_ATCATCTGTGAGGG-1 Patient5 Monocytes nonactive 4 0.0674656 Monocytes_4

10x_3288_t1_ATCATCTGTTGCAG-1 Patient5 Monocytes nonactive 0 0.03888466 Monocytes_0

10x_3288_t1_ATCATGCTATCAGC-1Patient5 Progenitors active 1 0.1332509 Progenitors_1

10x_3288_t1_ATCATGCTCTCTCG-1Patient5 Monocytes nonactive 0 0.03783844 Monocytes_0

10x_3288_t1_ATCATGCTGAGATA-1 Patient5 Progenitors active 1 0.13431298 Progenitors_1
10x_3288_t1_ATCATGCTGGAAGC-1 Patient5 Monocytes nonactive 4 0.03988333 Monocytes_4
10x_3288_t1_ATCATGCTTTCGTT-1 Patient5 Monocytes nonactive 0 0.02671042 Monocytes_0
10x_3288_t1_ATCCAGGAAGTCGT-1 Patient5 Monocytes nonactive 8 0.01889544 Monocytes_8
10x_3288_t1_ATCCAGGAGTTTGG-1 Patient5 Monocytes nonactive 4 0.03021368 Monocytes_4
10x_3288_t1_ATCCAGGATAAGGA-1 Patient5 Progenitors active 1 0.14799315 Progenitors_1
10x_3288_t1_ATCCATACCAATCG-1Patient5 Progenitors active 6 0.13494705 Progenitors_6
10x_3288_t1_ATCCATACGAGGGT-1 Patient5 Progenitors active 1 0.15607761 Progenitors_1
10x_3288_t1_ATCCCGTGAAGCAA-1 Patient5 Progenitors active 1 0.14753345 Progenitors_1
10x_3288_t1_ATCCCGTGAGACTC-1 Patient5 Monocytes nonactive 0 0.0514235 Monocytes_0
10x_3288_t1_ATCCCGTGATCGGT-1Patient5 Progenitors active 1 0.1749889 Progenitors_1
10x_3288_t1_ATCCCGTGGTGCAT-1Patient5 Progenitors active 1 0.1205377 Progenitors_1
10x_3288_t1_ATCCCGTGGTGTCA-1 Patient5 Progenitors active 1 0.13172912 Progenitors_1
10x_3288_t1_ATCCGCACACAGCT-1 Patient5 Progenitors active 1 0.12744912 Progenitors_1
10x_3288_t1_ATCCGCACAGATGA-1 Patient5 Monocytes nonactive 0 0.08995942 Monocytes_0
10x_3288_t1_ATCCGCACAGTGCT-1 Patient5 Monocytes nonactive 8 0.0303405 Monocytes_8
10x_3288_t1_ATCCGCACGGATTC-1 Patient5 Monocytes nonactive 4 0.0282639

Monocytes_4

10x_3288_t1_ATCCGCACTGGTAC-1 Patient5 Progenitors nonactive 6 0.11389576 Progenitors_6

10x_3288_t1_ATCCGCACTTATCC-1 Patient5 Monocytes nonactive 0 0.01394965 Monocytes_0

10x_3288_t1_ATCCTAACCTACTT-1 Patient5 Progenitors active 1 0.16798237 Progenitors_1

10x_3288_t1_ATCCTAACCTGTAG-1 Patient5 Progenitors active 1 0.14559952 Progenitors_1

10x_3288_t1_ATCCTAACGCGATT-1 Patient5 Progenitors active 1 0.12576882 Progenitors_1

10x_3288_t1_ATCCTAACTAGAAG-1 Patient5 Monocytes nonactive 4 0.04961638 Monocytes_4

10x_3288_t1_ATCGACGACATACG-1 Patient5 Monocytes nonactive 0 0.02582271 Monocytes_0

10x_3288_t1_ATCGACGAGAGCTT-1 Patient5 Monocytes nonactive 0 0.07827658 Monocytes_0

10x_3288_t1_ATCGACGATCAGGT-1 Patient5 Monocytes nonactive 4 0.03200495 Monocytes_4

10x_3288_t1_ATCGAGTGATTCCT-1 Patient5 Progenitors active 1 0.17188194 Progenitors_1

10x_3288_t1_ATCGAGTGCTCGAA-1 Patient5 Monocytes nonactive 4 0.0581447 Monocytes_4

10x_3288_t1_ATCGAGTGCTGTGA-1 Patient5 Dendritic cells nonactive 10 0.09836092 Dendritic cells_10

10x_3288_t1_ATCGAGTGGACACT-1 Patient5 Monocytes nonactive 0 0.0476349 Monocytes_0

10x_3288_t1_ATCGAGTGGTGTTG-1 Patient5 Monocytes nonactive 4 0.0805434 Monocytes_4

10x_3288_t1_ATCGAGTGTAGCCA-1 Patient5 Progenitors active 1 0.15078308 Progenitors_1

10x_3288_t1_ATCGCAGAAAGAAC-1 Patient5 Monocytes nonactive 0 0.0682899 Monocytes_0

10x_3288_t1_ATCGCAGAGACACT-1 Patient5 Monocytes nonactive 0 0.02837487 Monocytes_0
10x_3288_t1_ATCGCAGAGGAACG-1 Patient5 Monocytes nonactive 0 0.07295035 Monocytes_0
10x_3288_t1_ATCGCAGATGCCTC-1 Patient5 Monocytes nonactive 4 0.03802866 Monocytes_4
10x_3288_t1_ATCGCCACAACCAC-1 Patient5 Monocytes nonactive 0 0.03695073 Monocytes_0
10x_3288_t1_ATCGCCACATTCCT-1 Patient5 Monocytes nonactive 8 0.08892905 Monocytes_8
10x_3288_t1_ATCGCCACGGAGGT-1 Patient5 Monocytes nonactive 4 0.00477142 Monocytes_4
10x_3288_t1_ATCGCCACGGAGTG-1 Patient5 Monocytes nonactive 0 0.07179316 Monocytes_0
10x_3288_t1_ATCGCCACTCCCGT-1 Patient5 Monocytes nonactive 4 0.05031387 Monocytes_4
10x_3288_t1_ATCGCCTGAAGATG-1 Patient5 Monocytes nonactive 0 0.07052501 Monocytes_0
10x_3288_t1_ATCGCCTGCAGGAG-1 Patient5 Monocytes nonactive 0 0.03146598 Monocytes_0
10x_3288_t1_ATCGCCTGCTGTAG-1 Patient5 Monocytes nonactive 0 0.05136009 Monocytes_0
10x_3288_t1_ATCGCGCTAAGTGA-1 Patient5 Monocytes nonactive 4 0.06537315 Monocytes_4
10x_3288_t1_ATCGCGCTCCTCGT-1Patient5 Monocytes nonactive 4 0.05598884 Monocytes_4
10x_3288_t1_ATCGCGCTGATAGA-1 Patient5 Monocytes nonactive 0 0.07515376 Monocytes_0
10x_3288_t1_ATCGCGCTGGAGGT-1 Patient5 Monocytes nonactive 0 0.01593114 Monocytes_0
10x_3288_t1_ATCGCGCTGGATTC-1 Patient5 Monocytes nonactive 4 0.05380128 Monocytes_4
10x_3288_t1_ATCGCGCTTTCACT-1Patient5 Monocytes nonactive 4 0.07342591

Monocytes_4
10x_3288_t1_ATCGGAACGGTTCA-1 Patient5 Progenitors active 1 0.12213874
Progenitors_1
10x_3288_t1_ATCGGAACTGGAAA-1 Patient5 Progenitors active 1 0.15154397
Progenitors_1
10x_3288_t1_ATCGGTGAACACAC-1 Patient5 Monocytes nonactive 4 0.08514045
Monocytes_4
10x_3288_t1_ATCGGTGACCCTCA-1 Patient5 Monocytes nonactive 0 0.03512777
Monocytes_0
10x_3288_t1_ATCGGTGAGTCGAT-1 Patient5 Monocytes nonactive 0 0.05659121
Monocytes_0
10x_3288_t1_ATCGTTTGAAGCAA-1 Patient5 Monocytes nonactive 4 0.02675797
Monocytes_4
10x_3288_t1_ATCGTTTGACACAC-1 Patient5 Monocytes nonactive 4 0.04359267
Monocytes_4
10x_3288_t1_ATCGTTTGCTGTGA-1 Patient5 Monocytes nonactive 0 0.04178556
Monocytes_0
10x_3288_t1_ATCGTTTGGAGAGC-1 Patient5 Monocytes nonactive 4 0.00415319
Monocytes_4
10x_3288_t1_ATCGTTTGTTTCGT-1 Patient5 Monocytes nonactive 8 0.05600469
Monocytes_8
10x_3288_t1_ATCTACACATCACG-1 Patient5 Monocytes nonactive 8 0.03858348
Monocytes_8
10x_3288_t1_ATCTACACCCAATG-1 Patient5 Monocytes nonactive 0 0.05067846
Monocytes_0
10x_3288_t1_ATCTACACCTGCAA-1 Patient5 Monocytes nonactive 8 0.01959292
Monocytes_8
10x_3288_t1_ATCTACACTAACCG-1 Patient5 Monocytes nonactive 8 0.05215269
Monocytes_8
10x_3288_t1_ATCTACACTGAGGG-1 Patient5 Monocytes nonactive 4 0.04744468
Monocytes_4
10x_3288_t1_ATCTACTGCTCAAG-1 Patient5 Monocytes nonactive 0 0.04035889
Monocytes_0

10x_3288_t1_ATCTACTGGACTAC-1 Patient5 Monocytes nonactive 0 0.06007863 Monocytes_0
10x_3288_t1_ATCTACTGGGTTAC-1Patient5 Progenitors active 1 0.14103418 Progenitors_1
10x_3288_t1_ATCTCAACAGACTC-1 Patient5 Monocytes nonactive 8 0.0345571 Monocytes_8
10x_3288_t1_ATCTCAACCCTTTA-1Patient5 Monocytes nonactive 4 0.02249382 Monocytes_4
10x_3288_t1_ATCTCAACTTTCAC-1 Patient5 Monocytes nonactive 0 0.04622408 Monocytes_0
10x_3288_t1_ATCTGACTAAGCCT-1 Patient5 Monocytes nonactive 0 0.04132585 Monocytes_0
10x_3288_t1_ATCTGACTAAGTGA-1 Patient5 Monocytes nonactive 0 0.0219073 Monocytes_0
10x_3288_t1_ATCTGACTGACGAG-1 Patient5 Monocytes nonactive 4 0.03395473 Monocytes_4
10x_3288_t1_ATCTGACTTGGATC-1 Patient5 Monocytes nonactive 16 0.04167459 Monocytes_16
10x_3288_t1_ATCTGACTTTGCAG-1 Patient5 Monocytes nonactive 4 0.10776108 Monocytes_4
10x_3288_t1_ATCTGGGATAGCCA-1 Patient5 Monocytes nonactive 4 0.06209181 Monocytes_4
10x_3288_t1_ATCTGTTGACGGTT-1Patient5 Progenitors active 1 0.13226809 Progenitors_1
10x_3288_t1_ATCTGTTGAGCGGA-1 Patient5 Monocytes nonactive 4 0.02778835 Monocytes_4
10x_3288_t1_ATCTGTTGGCCATA-1Patient5 Progenitors active 1 0.13989284 Progenitors_1
10x_3288_t1_ATCTTGACAAGAAC-1 Patient5 Progenitors active 1 0.1702809 Progenitors_1
10x_3288_t1_ATCTTGACATGTCG-1 Patient5 Progenitors active 1 0.16631793 Progenitors_1
10x_3288_t1_ATCTTGACGAACTC-1 Patient5 Progenitors nonactive 6 0.10172151

Progenitors_6

10x_3288_t1_ATCTTGACTTCCGC-1 Patient5 Progenitors active 1 0.11847695 Progenitors_1

10x_3288_t1_ATCTTGACTTCGTT-1 Patient5 Progenitors active 1 0.12209118 Progenitors_1

10x_3288_t1_ATCTTTCTCGACTA-1 Patient5 Monocytes nonactive 8 0.01580432 Monocytes_8

10x_3288_t1_ATCTTTCTGATACC-1 Patient5 Progenitors active 1 0.12901845 Progenitors_1

10x_3288_t1_ATGAAACTATGACC-1 Patient5 Monocytes nonactive 0 0.01101706 Monocytes_0

10x_3288_t1_ATGAAACTCCATAG-1 Patient5 Progenitors active 1 0.12440555 Progenitors_1

10x_3288_t1_ATGAAACTCCTAAG-1 Patient5 Progenitors active 1 0.13415446 Progenitors_1

10x_3288_t1_ATGAAACTGAACTC-1 Patient5 Progenitors nonactive 5 0.07594636 Progenitors_5

10x_3288_t1_ATGAAGGAAGTCAC-1 Patient5 Progenitors active 1 0.16596918 Progenitors_1

10x_3288_t1_ATGACGTGACACGT-1 Patient5 Progenitors nonactive 1 0.11598821 Progenitors_1

10x_3288_t1_ATGACGTGCCAAGT-1 Patient5 Monocytes nonactive 4 0.07789614 Monocytes_4

10x_3288_t1_ATGACGTGGGATTC-1 Patient5 Monocytes nonactive 8 0.03414495 Monocytes_8

10x_3288_t1_ATGAGAGAAAGATG-1 Patient5 Monocytes nonactive 0 0.01471054 Monocytes_0

10x_3288_t1_ATGAGAGAGAACCT-1 Patient5 Monocytes nonactive 4 0.04703253 Monocytes_4

10x_3288_t1_ATGAGAGAGGGAGT-1 Patient5 Monocytes nonactive 0 0.06290026 Monocytes_0

10x_3288_t1_ATGAGAGAGTGAGG-1 Patient5 Monocytes nonactive 4 0.05475239 Monocytes_4

10x_3288_t1_ATGAGCACCCAGTA-1 Patient5 Monocytes nonactive 0 0.03133917 Monocytes_0
10x_3288_t1_ATGATAACACACCA-1 Patient5 Monocytes nonactive 4 0.02461797 Monocytes_4
10x_3288_t1_ATGATAACGGCAAG-1 Patient5 Monocytes nonactive 4 0.02724938 Monocytes_4
10x_3288_t1_ATGATATGACGTGT-1 Patient5 Progenitors nonactive 1 0.09333587 Progenitors_1
10x_3288_t1_ATGATATGCCTTAT-1 Patient5 Monocytes nonactive 8 0.04056496 Monocytes_8
10x_3288_t1_ATGATATGTAACGC-1 Patient5 Monocytes nonactive 8 0.03974066 Monocytes_8
10x_3288_t1_ATGATATGTTGGTG-1 Patient5 Monocytes nonactive 0 0.03729947 Monocytes_0
10x_3288_t1_ATGCACGACTATGG-1 Patient5 Progenitors nonactive 1 0.08146281 Progenitors_1
10x_3288_t1_ATGCACGAGGTTTG-1 Patient5 Monocytes nonactive 4 0.03530214 Monocytes_4
10x_3288_t1_ATGCACGATTCCCG-1 Patient5 Progenitors active 6 0.13402765 Progenitors_6
10x_3288_t1_ATGCAGACATGCCA-1 Patient5 Monocytes nonactive 4 0.06096633 Monocytes_4
10x_3288_t1_ATGCAGACCAATCG-1 Patient5 Monocytes nonactive 4 0.03650688 Monocytes_4
10x_3288_t1_ATGCAGACCACACA-1 Patient5 Monocytes nonactive 4 0.03091117 Monocytes_4
10x_3288_t1_ATGCAGACGAAAGT-1 Patient5 Progenitors active 1 0.17178682 Progenitors_1
10x_3288_t1_ATGCAGACGTTACG-1 Patient5 Progenitors active 1 0.16419377 Progenitors_1
10x_3288_t1_ATGCAGACTTTCTG-1 Patient5 Progenitors nonactive 1 0.0982658 Progenitors_1
10x_3288_t1_ATGCAGTGCGCCTT-1 Patient5 Monocytes nonactive 4 0.08171644

Monocytes_4

10x_3288_t1_ATGCAGTGGGATCT-1 Patient5 Progenitors active 1 0.13258512 Progenitors_1

10x_3288_t1_ATGCAGTGTAGAAG-1 Patient5 Monocytes nonactive 4 0.03251221 Monocytes_4

10x_3288_t1_ATGCAGTGTTACCT-1Patient5 Monocytes nonactive 0 0.06147359 Monocytes_0

10x_3288_t1_ATGCAGTGTTTCAC-1 Patient5 Progenitors nonactive 1 0.11231057 Progenitors_1

10x_3288_t1_ATGCCAGAAACGAA-1 Patient5 Monocytes nonactive 4 0.03149769 Monocytes_4

10x_3288_t1_ATGCCAGAGAGGGT-1 Patient5 Monocytes nonactive 4 0.07658043 Monocytes_4

10x_3288_t1_ATGCCAGAGATAGA-1 Patient5 Monocytes nonactive 0 0.03967726 Monocytes_0

10x_3288_t1_ATGCCAGAGGTCAT-1 Patient5 Progenitors active 1 0.13691269 Progenitors_1

10x_3288_t1_ATGCCAGATGATGC-1 Patient5 Monocytes nonactive 0 0.02587027 Monocytes_0

10x_3288_t1_ATGCCGCTAATGCC-1 Patient5 Monocytes nonactive 4 0.01618477 Monocytes_4

10x_3288_t1_ATGCCGCTACAGCT-1 Patient5 Monocytes nonactive 4 0.04887135 Monocytes_4

10x_3288_t1_ATGCCGCTGTAGCT-1 Patient5 Progenitors active 1 0.17185023 Progenitors_1

10x_3288_t1_ATGCGATGATCGAC-1 Patient5 Monocytes nonactive 0 0.03520703 Monocytes_0

10x_3288_t1_ATGCGATGTATGCG-1 Patient5 Monocytes nonactive 4 0.0350168 Monocytes_4

10x_3288_t1_ATGCGCCTAAGATG-1 Patient5 Monocytes nonactive 4 0.04956883 Monocytes_4

10x_3288_t1_ATGCGCCTTAACCG-1 Patient5 Monocytes nonactive 8 0.03011857 Monocytes_8

10x_3288_t1_ATGCTTTGAATCGC-1 Patient5 Monocytes nonactive 0 0.06131507 Monocytes_0
10x_3288_t1_ATGCTTTGATGTCG-1Patient5 Monocytes nonactive 4 0.01799188 Monocytes_4
10x_3288_t1_ATGCTTTGCATTGG-1Patient5 Monocytes nonactive 4 0.04654112 Monocytes_4
10x_3288_t1_ATGGACACGGTTTG-1 Patient5 Monocytes nonactive 4 0.02002092 Monocytes_4
10x_3288_t1_ATGGGTACACGTGT-1 Patient5 Monocytes nonactive 4 0.03985163 Monocytes_4
10x_3288_t1_ATGGGTACACTGTG-1 Patient5 Monocytes nonactive 0 0.10061188 Monocytes_0
10x_3288_t1_ATGGGTACATCGTG-1 Patient5 Monocytes active 0 0.12911356 Monocytes_0
10x_3288_t1_ATGGGTACCATTCT-1 Patient5 Progenitors active 6 0.15382664 Progenitors_6
10x_3288_t1_ATGGGTACCGAATC-1 Patient5 Progenitors nonactive 1 0.09244816 Progenitors_1
10x_3288_t1_ATGGGTACGCAGAG-1 Patient5 Monocytes nonactive 8 0.06355019 Monocytes_8
10x_3288_t1_ATGGGTACTCGCAA-1 Patient5 Progenitors nonactive 12 0.11226301 Progenitors_12
10x_3288_t1_ATGGGTACTCGCTC-1 Patient5 Progenitors active 1 0.14326929 Progenitors_1
10x_3288_t1_ATGGGTACTGGAAA-1 Patient5 Progenitors active 6 0.12821001 Progenitors_6
10x_3288_t1_ATGGTGACAACCGT-1 Patient5 Monocytes nonactive 4 0.03639592 Monocytes_4
10x_3288_t1_ATGGTGACAGCCAT-1 Patient5 Monocytes nonactive 0 0.04104052 Monocytes_0
10x_3288_t1_ATGGTGACATCAGC-1 Patient5 Monocytes nonactive 0 0.0353814 Monocytes_0
10x_3288_t1_ATGGTGACGGGAGT-1 Patient5 Monocytes nonactive 0 0.02010018

Monocytes_0
10x_3288_t1_ATGGTGACGTATCG-1 Patient5 Monocytes nonactive 8 0.06752901
Monocytes_8
10x_3288_t1_ATGGTGACTCTCGC-1 Patient5 Progenitors active 1 0.15958088
Progenitors_1
10x_3288_t1_ATGTAAACAAACAG-1 Patient5 Monocytes nonactive 0 0.03460465
Monocytes_0
10x_3288_t1_ATGTAAACACTAGC-1 Patient5 Monocytes nonactive 0 0.03129161
Monocytes_0
10x_3288_t1_ATGTAAACGGATTC-1 Patient5 Monocytes nonactive 8 0.0775791
Monocytes_8
10x_3288_t1_ATGTAAACTCGTAG-1 Patient5 Monocytes nonactive 8 0.08293704
Monocytes_8
10x_3288_t1_ATGTAAACTCGTGA-1 Patient5 Progenitors active 1 0.17555957
Progenitors_1
10x_3288_t1_ATGTAAACTTCGCC-1 Patient5 Progenitors active 6 0.13199861
Progenitors_6
10x_3288_t1_ATGTACCTGCTCCT-1 Patient5 Monocytes nonactive 0 0.062393
Monocytes_0
10x_3288_t1_ATGTCACTTCCCGT-1 Patient5 Progenitors active 1 0.13225224
Progenitors_1
10x_3288_t1_ATGTCGGACACCAA-1 Patient5 Monocytes nonactive 0 0.08639275
Monocytes_0
10x_3288_t1_ATGTCGGAGCAAGG-1 Patient5 Monocytes active 0 0.12914527
Monocytes_0
10x_3288_t1_ATGTCGGAGTCACA-1 Patient5 Monocytes nonactive 8 0.04157948
Monocytes_8
10x_3288_t1_ATGTCGGATAGAGA-1 Patient5 Monocytes nonactive 0 0.02144759
Monocytes_0
10x_3288_t1_ATGTTAGAAGTGCT-1 Patient5 Monocytes nonactive 4 0.07984592
Monocytes_4
10x_3288_t1_ATGTTCACGGTGTT-1 Patient5 Progenitors active 1 0.15677509
Progenitors_1

10x_3288_t1_ATGTTCACTGTCAG-1 Patient5 Monocytes nonactive 4 0.05977744 Monocytes_4
10x_3288_t1_ATGTTCACTTGTGG-1 Patient5 Monocytes nonactive 0 0.0425623 Monocytes_0
10x_3288_t1_ATGTTGCTCCAACA-1 Patient5 Monocytes nonactive 4 0.02870775 Monocytes_4
10x_3288_t1_ATGTTGCTCCTAAG-1 Patient5 Monocytes nonactive 0 0.08507704 Monocytes_0
10x_3288_t1_ATGTTGCTTAGAGA-1 Patient5 Monocytes nonactive 0 0.00477142 Monocytes_0
10x_3288_t1_ATGTTGCTTGCATG-1Patient5 Progenitors active 1 0.14981612 Progenitors_1
10x_3288_t1_ATGTTGCTTGTAGC-1 Patient5 Monocytes nonactive 0 0.01933929 Monocytes_0
10x_3288_t1_ATTAACGACAACCA-1 Patient5 Monocytes nonactive 0 0.02931013 Monocytes_0
10x_3288_t1_ATTAACGATCGATG-1 Patient5 Progenitors active 1 0.14252425 Progenitors_1
10x_3288_t1_ATTAAGACACCAAC-1 Patient5 Monocytes nonactive 0 0.07623169 Monocytes_0
10x_3288_t1_ATTAAGACGCGGAA-1 Patient5 Monocytes nonactive 0 0.0244119 Monocytes_0
10x_3288_t1_ATTAAGACGGACTT-1 Patient5 Monocytes nonactive 4 0.00665779 Monocytes_4
10x_3288_t1_ATTAAGACGGCATT-1 Patient5 Progenitors active 1 0.13975017 Progenitors_1
10x_3288_t1_ATTAAGACTACAGC-1 Patient5 Monocytes nonactive 4 0.07220531 Monocytes_4
10x_3288_t1_ATTACCACCTCGAA-1 Patient5 Monocytes nonactive 13 0.04273667 Monocytes_13
10x_3288_t1_ATTACCACCTTACT-1 Patient5 Monocytes nonactive 0 0.07049331 Monocytes_0
10x_3288_t1_ATTACCACGAGCAG-1 Patient5 Monocytes nonactive 0 0.04505104

Monocytes_0
10x_3288_t1_ATTACCACTACGAC-1 Patient5 Monocytes nonactive 4 0.0569241
Monocytes_4
10x_3288_t1_ATTACCACTAGTCG-1 Patient5 Monocytes nonactive 0 0.0902289
Monocytes_0
10x_3288_t1_ATTACCACTTGGCA-1 Patient5 Progenitors nonactive 1 0.11353116
Progenitors_1
10x_3288_t1_ATTACCACTTTGCT-1 Patient5 Progenitors active 1 0.1399721
Progenitors_1
10x_3288_t1_ATTACCACTTTGTC-1 Patient5 Monocytes nonactive 0 0.02219263
Monocytes_0
10x_3288_t1_ATTACCTGATGCTG-1 Patient5 Progenitors nonactive 1 0.09000697
Progenitors_1
10x_3288_t1_ATTACCTGCTAGAC-1 Patient5 Monocytes nonactive 0 0.03286095
Monocytes_0
10x_3288_t1_ATTACCTGGCAAGG-1 Patient5 Monocytes nonactive 4 0.02380952
Monocytes_4
10x_3288_t1_ATTACCTGGTAAGA-1 Patient5 Monocytes nonactive 4 0.06049077
Monocytes_4
10x_3288_t1_ATTAGATGCACTAG-1 Patient5 Monocytes nonactive 4 0.02531545
Monocytes_4
10x_3288_t1_ATTAGATGCTGGTA-1 Patient5 Monocytes nonactive 0 0.06597552
Monocytes_0
10x_3288_t1_ATTAGATGGGGATG-1 Patient5 Monocytes nonactive 0 0.04280008
Monocytes_0
10x_3288_t1_ATTAGATGTACTGG-1 Patient5 Monocytes nonactive 0 0.03555577
Monocytes_0
10x_3288_t1_ATTAGATGTCACCC-1 Patient5 Monocytes nonactive 4 0.07673895
Monocytes_4
10x_3288_t1_ATTAGATGTGACCA-1 Patient5 Monocytes nonactive 0 0.02734449
Monocytes_0
10x_3288_t1_ATTAGATGTGAGGG-1 Patient5 Monocytes nonactive 0 0.03639592
Monocytes_0

10x_3288_t1_ATTAGTGAAAGGGC-1 Patient5 Monocytes nonactive 4 0.03702999 Monocytes_4
10x_3288_t1_ATTAGTGAATCAGC-1 Patient5 Progenitors active 1 0.1547619 Progenitors_1
10x_3288_t1_ATTAGTGACGTCTC-1 Patient5 Progenitors active 1 0.15333524 Progenitors_1
10x_3288_t1_ATTAGTGATTTCTG-1 Patient5 Monocytes nonactive 8 0.02751886 Monocytes_8
10x_3288_t1_ATTATGGACTTGTT-1 Patient5 Monocytes nonactive 4 0.03344747 Monocytes_4
10x_3288_t1_ATTCAGCTACACTG-1 Patient5 Monocytes nonactive 4 0.03687147 Monocytes_4
10x_3288_t1_ATTCAGCTCTATTC-1 Patient5 Progenitors active 1 0.13069875 Progenitors_1
10x_3288_t1_ATTCAGCTTTGGTG-1 Patient5 Progenitors nonactive 1 0.10777693 Progenitors_1
10x_3288_t1_ATTCCAACACCTCC-1 Patient5 Dendritic cells nonactive 10 0.07355272 Dendritic cells_10
10x_3288_t1_ATTCCAACTCCCAC-1 Patient5 Monocytes nonactive 8 0.0657853 Monocytes_8
10x_3288_t1_ATTCCAACTGACTG-1 Patient5 Monocytes nonactive 8 0.04234037 Monocytes_8
10x_3288_t1_ATTCCAACTTCATC-1 Patient5 Progenitors active 1 0.12591148 Progenitors_1
10x_3288_t1_ATTCCATGACCGAT-1 Patient5 Monocytes nonactive 4 0.09541247 Monocytes_4
10x_3288_t1_ATTCCATGAGTAGA-1 Patient5 Monocytes nonactive 4 0.04159533 Monocytes_4
10x_3288_t1_ATTCCATGAGTCTG-1 Patient5 Monocytes nonactive 4 0.02940524 Monocytes_4
10x_3288_t1_ATTCCATGATGGTC-1 Patient5 Monocytes nonactive 4 0.07076279 Monocytes_4
10x_3288_t1_ATTCCATGTGGTGT-1 Patient5 Monocytes nonactive 0 0.06770338

Monocytes_0

10x_3288_t1_ATTCGACTGGTCAT-1 Patient5 Monocytes nonactive 4 0.01415573

Monocytes_4

10x_3288_t1_ATTCGACTGTATCG-1 Patient5 Monocytes active 0 0.12022066

Monocytes_0

10x_3288_t1_ATTCGACTTGCAGT-1 Patient5 Monocytes nonactive 4 0.04798364

Monocytes_4

10x_3288_t1_ATTCGACTTTCCCG-1 Patient5 Monocytes nonactive 8 0.03807622

Monocytes_8

10x_3288_t1_ATTCGACTTTCGGA-1 Patient5 Monocytes nonactive 0 0.06459641

Monocytes_0

10x_3288_t1_ATTCGGGAAGCCAT-1 Patient5 Monocytes nonactive 4 0.04573267

Monocytes_4

10x_3288_t1_ATTCGGGACCTACC-1 Patient5 Monocytes nonactive 0 0.02736034

Monocytes_0

10x_3288_t1_ATTCTGACTCCCAC-1 Patient5 Monocytes nonactive 0 0.05557669

Monocytes_0

10x_3288_t1_ATTCTTCTACCCTC-1 Patient5 Monocytes nonactive 0 0.08748653

Monocytes_0

10x_3288_t1_ATTCTTCTAGGCGA-1 Patient5 Progenitors active 1 0.16210133

Progenitors_1

10x_3288_t1_ATTCTTCTCCGCTT-1 Patient5 Monocytes nonactive 4 0.02458627

Monocytes_4

10x_3288_t1_ATTCTTCTGACACT-1 Patient5 Progenitors nonactive 1 0.09254328

Progenitors_1

10x_3288_t1_ATTCTTCTGGAGCA-1 Patient5 Monocytes nonactive 4 0.03164035

Monocytes_4

10x_3288_t1_ATTCTTCTGTCTTT-1 Patient5 Monocytes nonactive 4 0.07157124

Monocytes_4

10x_3288_t1_ATTCTTCTTGACCA-1 Patient5 Monocytes nonactive 0 0.02976983

Monocytes_0

10x_3288_t1_ATTGAAACACTAGC-1 Patient5 Progenitors nonactive 1 0.08279437

Progenitors_1

10x_3288_t1_ATTGAAACCAGCTA-1 Patient5 Monocytes nonactive 0 0.04925179
Monocytes_0
10x_3288_t1_ATTGAAACGTGTCA-1 Patient5 Progenitors active 1 0.11750999
Progenitors_1
10x_3288_t1_ATTGAATGCATTGG-1 Patient5 Monocytes nonactive 4 0.03184643
Monocytes_4
10x_3288_t1_ATTGAATGGCTCCT-1 Patient5 Progenitors active 1 0.16165747
Progenitors_1
10x_3288_t1_ATTGAATGTCATTC-1 Patient5 Progenitors active 1 0.16004058
Progenitors_1
10x_3288_t1_ATTGATGAATTTCC-1 Patient5 Monocytes nonactive 0 0.04823727
Monocytes_0
10x_3288_t1_ATTGATGAGCGGAA-1 Patient5 Progenitors active 1 0.1357555
Progenitors_1
10x_3288_t1_ATTGCACTAACAGA-1 Patient5 Progenitors active 1 0.14442648
Progenitors_1
10x_3288_t1_ATTGCACTAAGTGA-1 Patient5 Progenitors active 1 0.16801408
Progenitors_1
10x_3288_t1_ATTGCACTCACACA-1 Patient5 Monocytes nonactive 4 0.04110392
Monocytes_4
10x_3288_t1_ATTGCACTCACTAG-1 Patient5 Progenitors active 1 0.16801408
Progenitors_1
10x_3288_t1_ATTGCACTCCCTCA-1 Patient5 Monocytes nonactive 4 0.06970072
Monocytes_4
10x_3288_t1_ATTGCACTTACGAC-1 Patient5 Monocytes nonactive 4 0.03021368
Monocytes_4
10x_3288_t1_ATTGCACTTATTCC-1 Patient5 Monocytes nonactive 0 0.05061505
Monocytes_0
10x_3288_t1_ATTGCGGACGTTAG-1 Patient5 Progenitors active 1 0.15157568
Progenitors_1
10x_3288_t1_ATTGCGGATACTTC-1 Patient5 Monocytes nonactive 0 0.03259147
Monocytes_0
10x_3288_t1_ATTGCTACAGCGTT-1 Patient5 Progenitors nonactive 1 0.11438717

Progenitors_1

10x_3288_t1_ATTGCTACCAGAGG-1 Patient5 Monocytes nonactive 8 0.05342084 Monocytes_8

10x_3288_t1_ATTGCTACGTGTAC-1Patient5 Monocytes nonactive 0 0.03265487 Monocytes_0

10x_3288_t1_ATTGCTTGCAAAGA-1 Patient5 Monocytes nonactive 8 0.07055672 Monocytes_8

10x_3288_t1_ATTGGGTGAACGTC-1 Patient5 Monocytes nonactive 4 0.04226111 Monocytes_4

10x_3288_t1_ATTGGGTGAAGGCG-1 Patient5 Monocytes nonactive 0 0.05032972 Monocytes_0

10x_3288_t1_ATTGGGTGCTCCCA-1 Patient5 Monocytes nonactive 0 0.03431932 Monocytes_0

10x_3288_t1_ATTGGGTGGTCTTT-1Patient5 Monocytes nonactive 4 0.02623486 Monocytes_4

10x_3288_t1_ATTGGTCTACCACA-1 Patient5 Monocytes nonactive 8 0.04192822 Monocytes_8

10x_3288_t1_ATTGGTCTCTGCTC-1Patient5 Progenitors active 1 0.12887578 Progenitors_1

10x_3288_t1_ATTGTAGAAGGGTG-1 Patient5 Monocytes nonactive 0 0.08019466 Monocytes_0

10x_3288_t1_ATTGTAGACCGCTT-1Patient5 Progenitors active 1 0.14913449 Progenitors_1

10x_3288_t1_ATTGTAGAGTACGT-1Patient5 Monocytes nonactive 0 0.01846744 Monocytes_0

10x_3288_t1_ATTGTAGAGTCGTA-1 Patient5 Monocytes nonactive 4 0.03825059 Monocytes_4

10x_3288_t1_ATTGTCTGACCAAC-1 Patient5 Progenitors active 1 0.14195359 Progenitors_1

10x_3288_t1_ATTGTCTGTTGGCA-1 Patient5 Monocytes nonactive 4 0.02282671 Monocytes_4

10x_3288_t1_ATTTAGGAATACCG-1 Patient5 Progenitors active 1 0.1365798 Progenitors_1

10x_3288_t1_ATTTAGGACCGATA-1 Patient5 Progenitors active 1 0.19488301 Progenitors_1
10x_3288_t1_ATTTAGGAGACTAC-1 Patient5 Monocytes nonactive 4 0.03268658 Monocytes_4
10x_3288_t1_ATTTAGGAGGTTAC-1 Patient5 Monocytes nonactive 4 0.05654366 Monocytes_4
10x_3288_t1_ATTTCCGACCACCT-1 Patient5 Monocytes nonactive 0 0.0623613 Monocytes_0
10x_3288_t1_ATTTCCGACCCTAC-1 Patient5 Monocytes nonactive 0 0.03668125 Monocytes_0
10x_3288_t1_ATTTCCGAGATAGA-1 Patient5 Progenitors active 1 0.13837106 Progenitors_1
10x_3288_t1_ATTTCGTGCCAGTA-1 Patient5 Monocytes nonactive 0 0.03533384 Monocytes_0
10x_3288_t1_ATTTCGTGCCATGA-1 Patient5 Progenitors active 1 0.15534842 Progenitors_1
10x_3288_t1_ATTTCGTGCCGAAT-1 Patient5 Monocytes nonactive 4 0 Monocytes_4
10x_3288_t1_ATTTCGTGCTGACA-1 Patient5 Monocytes nonactive 4 0.04790438 Monocytes_4
10x_3288_t1_ATTTCTCTACTGTG-1 Patient5 Monocytes nonactive 4 0.05876292 Monocytes_4
10x_3288_t1_ATTTCTCTGGAGCA-1 Patient5 Progenitors active 1 0.16636548 Progenitors_1
10x_3288_t1_ATTTCTCTTCAGAC-1 Patient5 Monocytes nonactive 0 0.06670471 Monocytes_0
10x_3288_t1_ATTTGCACACACCA-1 Patient5 Progenitors nonactive 1 0.11481517 Progenitors_1
10x_3288_t1_ATTTGCACATCTCT-1 Patient5 Monocytes nonactive 4 0.02089278 Monocytes_4
10x_3288_t1_ATTTGCACCGAACT-1 Patient5 Monocytes nonactive 4 0.03200495 Monocytes_4
10x_3288_t1_ATTTGCACTTTGCT-1 Patient5 Monocytes nonactive 0 0.06798871 Monocytes_0

10x_3288_t1_CAAACTCTCCTTAT-1 Patient5 Monocytes nonactive 0 0.03130746
Monocytes_0
10x_3288_t1_CAAACTCTCGGGAA-1 Patient5 Monocytes nonactive 0 0.04333904
Monocytes_0
10x_3288_t1_CAAACTCTCTTAGG-1 Patient5 Monocytes nonactive 15 0.03614229
Monocytes_15
10x_3288_t1_CAAACTCTGTAGGG-1 Patient5 Monocytes nonactive 0 0.05633758
Monocytes_0
10x_3288_t1_CAAACTCTTGAGAA-1 Patient5 Progenitors active 1 0.15168664
Progenitors_1
10x_3288_t1_CAAAGCACCACTCC-1 Patient5 Monocytes nonactive 15 0.0501395
Monocytes_15
10x_3288_t1_CAAAGCACTGTAGC-1 Patient5 Monocytes nonactive 0 0.03547651
Monocytes_0
10x_3288_t1_CAAAGCTGACTACG-1 Patient5 Monocytes nonactive 0 0.03832985
Monocytes_0
10x_3288_t1_CAAAGCTGCATACG-1 Patient5 Progenitors active 1 0.15438146
Progenitors_1
10x_3288_t1_CAAAGCTGCGAGTT-1 Patient5 Monocytes nonactive 8 0.0531038
Monocytes_8
10x_3288_t1_CAAAGCTGTGGAAA-1 Patient5 Monocytes nonactive 8 0.01788092
Monocytes_8
10x_3288_t1_CAAATATGCTGAGT-1 Patient5 Monocytes nonactive 4 0.07388561
Monocytes_4
10x_3288_t1_CAAATATGTCCTAT-1 Patient5 Progenitors active 1 0.15487287
Progenitors_1
10x_3288_t1_CAAATTGAATACCG-1 Patient5 Monocytes nonactive 4 0.07626339
Monocytes_4
10x_3288_t1_CAAATTGACACTAG-1 Patient5 Monocytes nonactive 4 0.01816625
Monocytes_4
10x_3288_t1_CAACAGACACCAGT-1 Patient5 Monocytes nonactive 0 0.04730201
Monocytes_0
10x_3288_t1_CAACAGACACGGAG-1 Patient5 Monocytes nonactive 13 0.0468106

Monocytes_13
10x_3288_t1_CAACAGACAGCTCA-1 Patient5 Progenitors active 1 0.12416778
Progenitors_1
10x_3288_t1_CAACCAGAAATCGC-1 Patient5 Monocytes nonactive 8 0.08325407
Monocytes_8
10x_3288_t1_CAACCAGAAGTACC-1 Patient5 Monocytes nonactive 4 0.040882
Monocytes_4
10x_3288_t1_CAACCAGAATTGGC-1 Patient5 Monocytes nonactive 4 0.0505675
Monocytes_4
10x_3288_t1_CAACCAGACGTCTC-1 Patient5 Monocytes nonactive 4 0.03476317
Monocytes_4
10x_3288_t1_CAACCGCTCGTTAG-1 Patient5 Progenitors active 6 0.13867225
Progenitors_6
10x_3288_t1_CAACCGCTTAAAGG-1 Patient5 Progenitors active 1 0.1795701
Progenitors_1
10x_3288_t1_CAACCGCTTAACGC-1 Patient5 Monocytes nonactive 0 0.0577801
Monocytes_0
10x_3288_t1_CAACGAACCGACTA-1 Patient5 Monocytes nonactive 0 0.0286285
Monocytes_0
10x_3288_t1_CAACGAACTATCGG-1 Patient5 Monocytes nonactive 4 0.03780673
Monocytes_4
10x_3288_t1_CAACGAACTTTGTC-1 Patient5 Monocytes nonactive 0 0.06264663
Monocytes_0
10x_3288_t1_CAACGATGGATAAG-1 Patient5 Monocytes nonactive 13 0.04918838
Monocytes_13
10x_3288_t1_CAACGATGGTACGT-1 Patient5 Progenitors active 1 0.12562615
Progenitors_1
10x_3288_t1_CAACGATGTACGCA-1 Patient5 Progenitors nonactive 1 0.10302137
Progenitors_1
10x_3288_t1_CAACGATGTTGGCA-1 Patient5 Monocytes nonactive 0 0.03832985
Monocytes_0
10x_3288_t1_CAACGTGACCCAAA-1 Patient5 Monocytes nonactive 15 0.05766914
Monocytes_15

10x_3288_t1_CAACGTGACGTACA-1 Patient5 Monocytes nonactive 4 0.04310126
Monocytes_4
10x_3288_t1_CAACGTGAGACGAG-1 Patient5 Monocytes nonactive 4 0.01217424
Monocytes_4
10x_3288_t1_CAACGTGAGGATTC-1 Patient5 Progenitors active 6 0.1399721
Progenitors_6
10x_3288_t1_CAACGTGATGTGAC-1 Patient5 Progenitors active 1 0.14388752
Progenitors_1
10x_3288_t1_CAACGTGATTTCTG-1 Patient5 Monocytes nonactive 0 0.06714856
Monocytes_0
10x_3288_t1_CAACTTTGAGATGA-1 Patient5 Monocytes nonactive 0 0.040882
Monocytes_0
10x_3288_t1_CAACTTTGCGTAAC-1 Patient5 Monocytes nonactive 4 0.05424513
Monocytes_4
10x_3288_t1_CAACTTTGCTGTAG-1 Patient5 Monocytes nonactive 0 0.05559254
Monocytes_0
10x_3288_t1_CAAGAAGAAAAACG-1 Patient5 Monocytes nonactive 4 0.06064929
Monocytes_4
10x_3288_t1_CAAGAAGACCGTTC-1 Patient5 Progenitors active 1 0.12061696
Progenitors_1
10x_3288_t1_CAAGAAGACTAGCA-1 Patient5 Progenitors active 1 0.15666413
Progenitors_1
10x_3288_t1_CAAGAAGATACGCA-1 Patient5 Monocytes nonactive 0 0.0387737
Monocytes_0
10x_3288_t1_CAAGAAGATAGCGT-1 Patient5 Progenitors active 1 0.15652146
Progenitors_1
10x_3288_t1_CAAGAAGATGGTAC-1 Patient5 Monocytes nonactive 4 0.05598884
Monocytes_4
10x_3288_t1_CAAGACACGAATGA-1 Patient5 Progenitors active 1 0.15324012
Progenitors_1
10x_3288_t1_CAAGACACGACAGG-1 Patient5 Progenitors active 1 0.15235242
Progenitors_1
10x_3288_t1_CAAGACACGTAAGA-1 Patient5 Progenitors nonactive 1 0.09726714

Progenitors_1
10x_3288_t1_CAAGACTGACTGGT-1 Patient5 Monocytes nonactive 4 0.03198909 Monocytes_4
10x_3288_t1_CAAGACTGGGGCAA-1 Patient5 Progenitors active 1 0.17885676 Progenitors_1
10x_3288_t1_CAAGACTGTTCCAT-1 Patient5 Monocytes nonactive 0 0.05066261 Monocytes_0
10x_3288_t1_CAAGCATGAGAGAT-1 Patient5 Progenitors active 1 0.18475366 Progenitors_1
10x_3288_t1_CAAGCATGATTTCC-1 Patient5 Progenitors active 1 0.15617272 Progenitors_1
10x_3288_t1_CAAGCATGTACGCA-1 Patient5 Monocytes nonactive 0 0.07551836 Monocytes_0
10x_3288_t1_CAAGCATGTAGAGA-1 Patient5 Progenitors active 1 0.15701287 Progenitors_1
10x_3288_t1_CAAGCATGTCTCTA-1 Patient5 Monocytes nonactive 15 0.03520703 Monocytes_15
10x_3288_t1_CAAGCCCTACTCTT-1 Patient5 Monocytes nonactive 0 0.05952381 Monocytes_0
10x_3288_t1_CAAGCCCTATCGGT-1 Patient5 Progenitors active 1 0.17743009 Progenitors_1
10x_3288_t1_CAAGCCCTGTCACA-1 Patient5 Progenitors active 1 0.1580908 Progenitors_1
10x_3288_t1_CAAGCCCTGTCTTT-1 Patient5 Progenitors nonactive 1 0.10194344 Progenitors_1
10x_3288_t1_CAAGCTGAGGTGAG-1 Patient5 Monocytes nonactive 4 0.02488745 Monocytes_4
10x_3288_t1_CAAGGACTAAGAAC-1 Patient5 Monocytes nonactive 4 0.03385962 Monocytes_4
10x_3288_t1_CAAGGACTAGGTCT-1 Patient5 Monocytes nonactive 0 0.02547397 Monocytes_0
10x_3288_t1_CAAGGACTCGAATC-1 Patient5 Monocytes nonactive 0 0.05029802 Monocytes_0

10x_3288_t1_CAAGGACTGCATCA-1 Patient5 Monocytes nonactive 0 0.04345
Monocytes_0
10x_3288_t1_CAAGGACTTTCCCG-1 Patient5 Monocytes nonactive 0 0.05866781
Monocytes_0
10x_3288_t1_CAAGGTTGCCTCAC-1 Patient5 Monocytes nonactive 8 0.0564644
Monocytes_8
10x_3288_t1_CAAGGTTGGGATCT-1 Patient5 Progenitors active 1 0.15236827
Progenitors_1
10x_3288_t1_CAAGGTTGTGACCA-1 Patient5 Progenitors active 1 0.15796398
Progenitors_1
10x_3288_t1_CAAGTCGACAGATC-1 Patient5 Monocytes nonactive 0 0.03592036
Monocytes_0
10x_3288_t1_CAAGTCGATGTCAG-1 Patient5 Progenitors active 1 0.18755944
Progenitors_1
10x_3288_t1_CAAGTTCTACTTTC-1 Patient5 Progenitors active 1 0.18365988
Progenitors_1
10x_3288_t1_CAAGTTCTCATGAC-1 Patient5 Monocytes nonactive 4 0.03574599
Monocytes_4
10x_3288_t1_CAAGTTCTCGAGAG-1 Patient5 Monocytes nonactive 0 0.05655951
Monocytes_0
10x_3288_t1_CAAGTTCTGCCATA-1 Patient5 Progenitors nonactive 5 0.06965316
Progenitors_5
10x_3288_t1_CAAGTTCTTCCCGT-1 Patient5 Monocytes nonactive 0 0.06756071
Monocytes_0
10x_3288_t1_CAATAAACAGGCGA-1 Patient5 Monocytes nonactive 4 0.04135756
Monocytes_4
10x_3288_t1_CAATAAACATTCGG-1 Patient5 Monocytes nonactive 8 0.04067592
Monocytes_8
10x_3288_t1_CAATAAACCAAAGA-1 Patient5 Progenitors nonactive 1 0.10544671
Progenitors_1
10x_3288_t1_CAATAATGGCTCCT-1 Patient5 Monocytes nonactive 0 0.05557669
Monocytes_0
10x_3288_t1_CAATAATGTTTCTG-1 Patient5 Monocytes nonactive 4 0.07361613

Monocytes_4
10x_3288_t1_CAATATGACTGTAG-1 Patient5 Monocytes nonactive 4 0.06665716
Monocytes_4
10x_3288_t1_CAATATGAGGTATC-1 Patient5 Progenitors active 1 0.14894426
Progenitors_1
10x_3288_t1_CAATATGATCAAGC-1 Patient5 Monocytes nonactive 4 0.02728109
Monocytes_4
10x_3288_t1_CAATCGGAATACCG-1 Patient5 Progenitors active 1 0.17042356
Progenitors_1
10x_3288_t1_CAATCGGACACTCC-1 Patient5 Monocytes nonactive 4 0.03064168
Monocytes_4
10x_3288_t1_CAATCGGACCGCTT-1 Patient5 Monocytes nonactive 8 0.05242217
Monocytes_8
10x_3288_t1_CAATCGGATCAGAC-1 Patient5 Monocytes nonactive 0 0.05625832
Monocytes_0
10x_3288_t1_CAATCTACACGACT-1 Patient5 Monocytes nonactive 0 0.0290565
Monocytes_0
10x_3288_t1_CAATGGACGACTAC-1 Patient5 Progenitors active 1 0.14856382
Progenitors_1
10x_3288_t1_CAATGGACGATGAA-1 Patient5 Progenitors active 1 0.14265107
Progenitors_1
10x_3288_t1_CAATGGACGGTAAA-1 Patient5 Monocytes nonactive 0 0.0493469
Monocytes_0
10x_3288_t1_CAATGGACTGCAGT-1 Patient5 Monocytes nonactive 0 0.05611566
Monocytes_0
10x_3288_t1_CAATGGACTTTGTC-1 Patient5 Progenitors active 1 0.13708706
Progenitors_1
10x_3288_t1_CAATTCACGTCATG-1 Patient5 Monocytes nonactive 8 0.01911737
Monocytes_8
10x_3288_t1_CAATTCACTAAGCC-1 Patient5 Monocytes nonactive 0 0.01345825
Monocytes_0
10x_3288_t1_CAATTCACTCACGA-1 Patient5 Progenitors active 1 0.20198466
Progenitors_1

10x_3288_t1_CAATTCTGCTTGAG-1	Patient5	Monocytes	nonactive	0	0.05250143	Monocytes_0
10x_3288_t1_CACAACGAAAAAGC-1	Patient5	Monocytes	nonactive	4	0.04814216	Monocytes_4
10x_3288_t1_CACAACGACATTGG-1	Patient5	Monocytes	nonactive	4	0.0547841	Monocytes_4
10x_3288_t1_CACAATCTGTAGGG-1	Patient5	Monocytes	nonactive	0	0.04222941	Monocytes_0
10x_3288_t1_CACACCTGCAAAGA-1	Patient5	Monocytes	nonactive	4	0.0708262	Monocytes_4
10x_3288_t1_CACACCTGCAGATC-1	Patient5	Progenitors	active	1	0.17334031	Progenitors_1
10x_3288_t1_CACACCTGGGGTGA-1	Patient5	Progenitors	active	1	0.2057257	Progenitors_1
10x_3288_t1_CACAGAACCACTTT-1	Patient5	Monocytes	nonactive	4	0.02542642	Monocytes_4
10x_3288_t1_CACAGAACGTTGAC-1	Patient5	Progenitors	active	1	0.20634392	Progenitors_1
10x_3288_t1_CACAGATGACTGTG-1	Patient5	Monocytes	nonactive	4	0.04067592	Monocytes_4
10x_3288_t1_CACAGATGCCAACA-1	Patient5	Monocytes	nonactive	4	0.05164543	Monocytes_4
10x_3288_t1_CACAGATGGGCGAA-1	Patient5	Monocytes	nonactive	0	0.06085537	Monocytes_0
10x_3288_t1_CACAGCCTCACTGA-1	Patient5	Monocytes	nonactive	13	0.03359013	Monocytes_13
10x_3288_t1_CACAGCCTCGAACT-1	Patient5	Monocytes	nonactive	4	0.01455203	Monocytes_4
10x_3288_t1_CACAGTGAAAGATG-1	Patient5	Monocytes	nonactive	0	0.03515947	Monocytes_0
10x_3288_t1_CACAGTGAACTAGC-1	Patient5	Monocytes	nonactive	4	0.00321793	Monocytes_4
10x_3288_t1_CACATACTCAGTCA-1	Patient5	Monocytes	nonactive	4	0.03596792	

Monocytes_4

10x_3288_t1_CACATACTCCCTAC-1Patient5 Monocytes nonactive 0 0.03460465

Monocytes_0

10x_3288_t1_CACATACTTATTCC-1 Patient5 Monocytes nonactive 15 0.05608395

Monocytes_15

10x_3288_t1_CACATACTTGCTAG-1 Patient5 Progenitors active 1 0.15108427

Progenitors_1

10x_3288_t1_CACATACTTGTTCT-1 Patient5 Monocytes nonactive 0 0.04421089

Monocytes_0

10x_3288_t1_CACATGGAAGTAGA-1 Patient5 Monocytes nonactive 0 0.04308541

Monocytes_0

10x_3288_t1_CACATGGATGAGGG-1 Patient5 Monocytes nonactive 4 0.05446706

Monocytes_4

10x_3288_t1_CACCACTGTGAGGG-1 Patient5 Progenitors active 1 0.12247163

Progenitors_1

10x_3288_t1_CACCCATGCACTAG-1 Patient5 Monocytes nonactive 4 0.05047239

Monocytes_4

10x_3288_t1_CACCCATGCCGTAA-1 Patient5 Monocytes nonactive 0 0.03549236

Monocytes_0

10x_3288_t1_CACCCATGGCAAGG-1 Patient5 Progenitors active 1 0.1914273

Progenitors_1

10x_3288_t1_CACCCATGTCGCTC-1 Patient5 Monocytes nonactive 0 0.07280768

Monocytes_0

10x_3288_t1_CACCCATGTGACTG-1 Patient5 Monocytes nonactive 0 0.0678619

Monocytes_0

10x_3288_t1_CACCGGGAAGAATG-1 Patient5 Monocytes nonactive 4 0.02888213

Monocytes_4

10x_3288_t1_CACCGTACTGGTTG-1 Patient5 Monocytes nonactive 4 0.0636453

Monocytes_4

10x_3288_t1_CACCGTACTTTGTC-1 Patient5 Monocytes nonactive 4 0.07661214

Monocytes_4

10x_3288_t1_CACCGTTGACGGTT-1 Patient5 Progenitors active 1 0.12846364

Progenitors_1

10x_3288_t1_CACGAAACGCTGAT-1 Patient5 Monocytes nonactive 0 0.02854924 Monocytes_0
10x_3288_t1_CACGACCTCTGCTC-1 Patient5 Progenitors active 1 0.18619618 Progenitors_1
10x_3288_t1_CACGACCTGAATCC-1 Patient5 Monocytes nonactive 0 0.04249889 Monocytes_0
10x_3288_t1_CACGACCTGTGTCA-1 Patient5 Monocytes nonactive 8 0.03051487 Monocytes_8
10x_3288_t1_CACGACCTTAGACC-1 Patient5 Monocytes nonactive 4 0.04444867 Monocytes_4
10x_3288_t1_CACGACCTTGCGTA-1 Patient5 Monocytes nonactive 4 0.02818464 Monocytes_4
10x_3288_t1_CACGACCTTGTCAG-1 Patient5 Monocytes nonactive 4 0.05823981 Monocytes_4
10x_3288_t1_CACGACCTTTAGGC-1 Patient5 Monocytes nonactive 0 0.06478663 Monocytes_0
10x_3288_t1_CACGATGAAGTACC-1 Patient5 Monocytes nonactive 4 0.04029548 Monocytes_4
10x_3288_t1_CACGATGACGCATA-1 Patient5 Monocytes nonactive 4 0.06296367 Monocytes_4
10x_3288_t1_CACGATGAGGTATC-1 Patient5 Monocytes nonactive 0 0.00559571 Monocytes_0
10x_3288_t1_CACGGGACACGCAT-1 Patient5 Monocytes nonactive 0 0.03663369 Monocytes_0
10x_3288_t1_CACGGGACACTCAG-1 Patient5 Monocytes nonactive 0 0.09043498 Monocytes_0
10x_3288_t1_CACGGGACCTATGG-1 Patient5 Dendritic cells nonactive 10 0.10693678 Dendritic cells_10
10x_3288_t1_CACGGGACTTCGTT-1 Patient5 Monocytes nonactive 0 0.03617399 Monocytes_0
10x_3288_t1_CACGGGTGAGAATG-1 Patient5 Monocytes nonactive 0 0.08567941 Monocytes_0
10x_3288_t1_CACGGGTGCCAAGT-1 Patient5 Progenitors active 1 0.19348805

Progenitors_1
10x_3288_t1_CACGGGTGCCGAAT-1 Patient5 Monocytes nonactive 0 0.05843003 Monocytes_0
10x_3288_t1_CACGGGTGCCTGAA-1 Patient5 Monocytes nonactive 0 0.05464143 Monocytes_0
10x_3288_t1_CACGGGTGGCTTCC-1 Patient5 Monocytes nonactive 0 0.05793862 Monocytes_0
10x_3288_t1_CACGGGTGTGGTCA-1 Patient5 Monocytes nonactive 0 0.0737905 Monocytes_0
10x_3288_t1_CACGGGTGTTTGGG-1 Patient5 Monocytes nonactive 0 0.09568195 Monocytes_0
10x_3288_t1_CACTAACTGGCGAA-1 Patient5 Progenitors active 1 0.2090863 Progenitors_1
10x_3288_t1_CACTAACTGTCCTC-1 Patient5 Monocytes nonactive 0 0.03926511 Monocytes_0
10x_3288_t1_CACTAACTTCGCTC-1 Patient5 Monocytes nonactive 4 0.03707755 Monocytes_4
10x_3288_t1_CACTAGGAGGTGTT-1 Patient5 Progenitors active 1 0.13145964 Progenitors_1
10x_3288_t1_CACTATACAACCTG-1 Patient5 Monocytes nonactive 0 0.02575931 Monocytes_0
10x_3288_t1_CACTATACGGAGTG-1 Patient5 Monocytes nonactive 0 0.02634582 Monocytes_0
10x_3288_t1_CACTATACTAAGCC-1 Patient5 Progenitors active 1 0.12916112 Progenitors_1
10x_3288_t1_CACTCCGACACTGA-1 Patient5 Monocytes nonactive 4 0.05319891 Monocytes_4
10x_3288_t1_CACTCCGACTTCTA-1 Patient5 Monocytes nonactive 4 0.06973242 Monocytes_4
10x_3288_t1_CACTCCGAGAGCAG-1 Patient5 Progenitors active 1 0.13333016 Progenitors_1
10x_3288_t1_CACTCCGAGCATCA-1 Patient5 Progenitors active 1 0.13038171 Progenitors_1

10x_3288_t1_CACTCCGATCTACT-1 Patient5 Monocytes nonactive 4 0.02886627
Monocytes_4
10x_3288_t1_CACTCTCTGAATGA-1 Patient5 Monocytes nonactive 0 0.08437956
Monocytes_0
10x_3288_t1_CACTGAGAACCTGA-1 Patient5 Progenitors active 1 0.14437892
Progenitors_1
10x_3288_t1_CACTGAGACTAGTG-1 Patient5 Monocytes nonactive 8 0.02872361
Monocytes_8
10x_3288_t1_CACTGAGAGGGATG-1 Patient5 Monocytes nonactive 0 0.08476
Monocytes_0
10x_3288_t1_CACTGAGAGGTCAT-1 Patient5 Monocytes nonactive 4 0.04996513
Monocytes_4
10x_3288_t1_CACTGAGATGTCGA-1 Patient5 Progenitors nonactive 1 0.07486843
Progenitors_1
10x_3288_t1_CACTGAGATTCGTT-1 Patient5 Monocytes nonactive 0 0.02043307
Monocytes_0
10x_3288_t1_CACTGCACACACAC-1 Patient5 Monocytes nonactive 8 0.03113309
Monocytes_8
10x_3288_t1_CACTGCACTCCTAT-1 Patient5 Progenitors active 1 0.13610424
Progenitors_1
10x_3288_t1_CACTGCACTGCGTA-1 Patient5 Monocytes nonactive 0 0.00919409
Monocytes_0
10x_3288_t1_CACTGCTGGGTAAA-1 Patient5 Progenitors active 1 0.14791389
Progenitors_1
10x_3288_t1_CACTGCTGGGTCAT-1 Patient5 Monocytes nonactive 0 0.05286602
Monocytes_0
10x_3288_t1_CACTGCTGTGCTTT-1 Patient5 Monocytes nonactive 0 0.06445374
Monocytes_0
10x_3288_t1_CACTTAACATGCTG-1 Patient5 Monocytes nonactive 0 0.05026631
Monocytes_0
10x_3288_t1_CACTTATGCTTACT-1 Patient5 Progenitors nonactive 1 0.10869634
Progenitors_1
10x_3288_t1_CACTTATGTATTCC-1 Patient5 Progenitors nonactive 1 0.11234227

Progenitors_1

10x_3288_t1_CACTTATGTGAGAA-1 Patient5 Progenitors active 1 0.13131697 Progenitors_1

10x_3288_t1_CACTTTGAAACCAC-1 Patient5 Monocytes nonactive 4 0.0548158 Monocytes_4

10x_3288_t1_CACTTTGACTAAGC-1 Patient5 Progenitors active 1 0.20953015 Progenitors_1

10x_3288_t1_CAGAAGCTAGGCGA-1 Patient5 Monocytes nonactive 0 0.07916429 Monocytes_0

10x_3288_t1_CAGAAGCTCTGTCC-1 Patient5 Monocytes nonactive 4 0.03214761 Monocytes_4

10x_3288_t1_CAGAAGCTGGACAG-1 Patient5 Monocytes nonactive 4 0.06209181 Monocytes_4

10x_3288_t1_CAGAAGCTGTCTGA-1 Patient5 Monocytes nonactive 4 0.09831336 Monocytes_4

10x_3288_t1_CAGACAACCTCCAC-1 Patient5 Monocytes nonactive 0 0.06275759 Monocytes_0

10x_3288_t1_CAGACAACTCCGAA-1 Patient5 Progenitors nonactive 1 0.11001205 Progenitors_1

10x_3288_t1_CAGACATGCATGCA-1 Patient5 Monocytes nonactive 4 0.06063344 Monocytes_4

10x_3288_t1_CAGACATGTGACCA-1 Patient5 Monocytes nonactive 0 0.085188 Monocytes_0

10x_3288_t1_CAGACATGTGAGGG-1 Patient5 Monocytes nonactive 0 0.03603132 Monocytes_0

10x_3288_t1_CAGACATGTTCCAT-1Patient5 Monocytes nonactive 0 0.02883457 Monocytes_0

10x_3288_t1_CAGACCCTATCGTG-1 Patient5 Progenitors nonactive 1 0.10866464 Progenitors_1

10x_3288_t1_CAGACCCTGAATAG-1 Patient5 Monocytes nonactive 0 0.06313804 Monocytes_0

10x_3288_t1_CAGACTGAAGCCTA-1 Patient5 Monocytes nonactive 0 0.05838247 Monocytes_0

10x_3288_t1_CAGACTGAATCACG-1 Patient5 Progenitors active 1 0.17689113 Progenitors_1
10x_3288_t1_CAGACTGACTATTC-1 Patient5 Monocytes nonactive 4 0.06594382 Monocytes_4
10x_3288_t1_CAGACTGACTCTCG-1 Patient5 Monocytes nonactive 4 0.0383457 Monocytes_4
10x_3288_t1_CAGACTGAGGAAAT-1 Patient5 Progenitors active 1 0.13605669 Progenitors_1
10x_3288_t1_CAGACTGAGTAAAG-1 Patient5 Progenitors active 1 0.12710037 Progenitors_1
10x_3288_t1_CAGACTGATTGTCT-1 Patient5 B cells nonactive 7 0.05559254 B cells_7
10x_3288_t1_CAGAGGGACGCATA-1 Patient5 Monocytes nonactive 0 0.03787014 Monocytes_0
10x_3288_t1_CAGAGGGAGCCTTC-1 Patient5 Monocytes nonactive 4 0.08461734 Monocytes_4
10x_3288_t1_CAGAGGGAGGACTT-1 Patient5 Monocytes nonactive 0 0.05990425 Monocytes_0
10x_3288_t1_CAGATCGAATACCG-1 Patient5 Monocytes nonactive 0 0.02166952 Monocytes_0
10x_3288_t1_CAGATCGACACTTT-1 Patient5 Monocytes nonactive 0 0.01715173 Monocytes_0
10x_3288_t1_CAGATCGATGTCGA-1 Patient5 Progenitors active 1 0.12611756 Progenitors_1
10x_3288_t1_CAGATGACCGTCTC-1 Patient5 Monocytes nonactive 13 0.06530975 Monocytes_13
10x_3288_t1_CAGATGACGACAGG-1 Patient5 Monocytes nonactive 8 0.0518198 Monocytes_8
10x_3288_t1_CAGATGACGGCGAA-1 Patient5 Progenitors active 1 0.15707628 Progenitors_1
10x_3288_t1_CAGATGACGTAAAG-1 Patient5 Monocytes nonactive 4 0.07816562 Monocytes_4
10x_3288_t1_CAGCAATGCGTGAT-1 Patient5 Monocytes nonactive 0 0.04785683

Monocytes_0

10x_3288_t1_CAGCAATGTCGTAG-1 Patient5 Progenitors active 1 0.13474098 Progenitors_1

10x_3288_t1_CAGCACCTACCATG-1 Patient5 Progenitors active 1 0.15420709 Progenitors_1

10x_3288_t1_CAGCATGAACTACG-1 Patient5 Monocytes nonactive 0 0.04658868 Monocytes_0

10x_3288_t1_CAGCATGACTCTCG-1 Patient5 Monocytes nonactive 4 0.0670059 Monocytes_4

10x_3288_t1_CAGCATGAGTTGAC-1 Patient5 Monocytes nonactive 0 0.03676051 Monocytes_0

10x_3288_t1_CAGCCTACACCAGT-1 Patient5 Monocytes nonactive 4 0.07198339 Monocytes_4

10x_3288_t1_CAGCCTTGTCGCAA-1 Patient5 Monocytes nonactive 8 0.04081859 Monocytes_8

10x_3288_t1_CAGCGGACCTTCCG-1 Patient5 Progenitors active 1 0.12572126 Progenitors_1

10x_3288_t1_CAGCGGACTGCGTA-1 Patient5 Progenitors active 1 0.15312916 Progenitors_1

10x_3288_t1_CAGCGTCTACTCTT-1Patient5 Progenitors active 1 0.12464333 Progenitors_1

10x_3288_t1_CAGCGTCTCCTTGC-1 Patient5 Monocytes nonactive 0 0.02512523 Monocytes_0

10x_3288_t1_CAGCGTCTCGTGTA-1 Patient5 Progenitors active 6 0.1593431 Progenitors_6

10x_3288_t1_CAGCGTCTCTGTTT-1Patient5 Monocytes nonactive 4 0.03030879 Monocytes_4

10x_3288_t1_CAGCGTCTTACAGC-1 Patient5 Monocytes nonactive 8 0.04936275 Monocytes_8

10x_3288_t1_CAGCGTCTTAGCCA-1 Patient5 Monocytes nonactive 0 0.01740536 Monocytes_0

10x_3288_t1_CAGCGTCTTAGCGT-1 Patient5 Monocytes nonactive 0 0.03065754 Monocytes_0

10x_3288_t1_CAGCTAGAAGTCTG-1 Patient5 Monocytes nonactive 0 0.05952381 Monocytes_0
10x_3288_t1_CAGCTAGATGCAAC-1 Patient5 Monocytes nonactive 13 0.00836979 Monocytes_13
10x_3288_t1_CAGCTCTGGGTGGA-1 Patient5 Progenitors active 1 0.15057701 Progenitors_1
10x_3288_t1_CAGGAACTGTAGCT-1 Patient5 Monocytes nonactive 4 0.04573267 Monocytes_4
10x_3288_t1_CAGGAACTTTAGGC-1 Patient5 Progenitors active 1 0.15244753 Progenitors_1
10x_3288_t1_CAGGAACTTTTCGT-1 Patient5 Monocytes nonactive 0 0.04443282 Monocytes_0
10x_3288_t1_CAGGCCGAAGCTCA-1 Patient5 Monocytes nonactive 0 0.01694566 Monocytes_0
10x_3288_t1_CAGGCCGAGAAACA-1 Patient5 Monocytes nonactive 8 0.04046985 Monocytes_8
10x_3288_t1_CAGGCCGAGGTACT-1 Patient5 Monocytes nonactive 0 0.03523873 Monocytes_0
10x_3288_t1_CAGGCCGAGTGCAT-1 Patient5 Progenitors active 1 0.17697039 Progenitors_1
10x_3288_t1_CAGGCCGAGTTAGC-1 Patient5 Progenitors active 1 0.13172912 Progenitors_1
10x_3288_t1_CAGGCCGATCGATG-1 Patient5 Progenitors active 1 0.12250333 Progenitors_1
10x_3288_t1_CAGGGCACCTATTC-1 Patient5 Monocytes nonactive 0 0.03336821 Monocytes_0
10x_3288_t1_CAGGTAACAAGGTA-1 Patient5 Monocytes nonactive 0 0.07626339 Monocytes_0
10x_3288_t1_CAGGTAACCGAGAG-1 Patient5 Monocytes nonactive 4 0.01602625 Monocytes_4
10x_3288_t1_CAGGTAACCGTGAT-1 Patient5 Monocytes nonactive 0 0.06507197 Monocytes_0
10x_3288_t1_CAGGTAACCTGTCC-1 Patient5 Monocytes nonactive 4 0.07640606

Monocytes_4
10x_3288_t1_CAGGTAACCTTCTA-1 Patient5 T cells nonactive 9 0.06098218 T cells_9
10x_3288_t1_CAGGTAACTGAAGA-1 Patient5 Progenitors active 1 0.12660897 Progenitors_1
10x_3288_t1_CAGGTATGAGTTCG-1 Patient5 Monocytes nonactive 0 0.01899055 Monocytes_0
10x_3288_t1_CAGGTATGTCGTTT-1 Patient5 Monocytes nonactive 0 0.0611407 Monocytes_0
10x_3288_t1_CAGGTTGAAATCGC-1 Patient5 Monocytes nonactive 4 0.07478917 Monocytes_4
10x_3288_t1_CAGGTTGACATGAC-1 Patient5 Monocytes nonactive 13 0.0160421 Monocytes_13
10x_3288_t1_CAGGTTGACCGTAA-1 Patient5 Monocytes nonactive 4 0.0159787 Monocytes_4
10x_3288_t1_CAGGTTGAGGATTC-1 Patient5 Monocytes nonactive 4 0.01618477 Monocytes_4
10x_3288_t1_CAGTCAGAAGCGTT-1 Patient5 Monocytes nonactive 4 0.05077357 Monocytes_4
10x_3288_t1_CAGTCAGAGAGCAG-1 Patient5 Progenitors active 1 0.18608522 Progenitors_1
10x_3288_t1_CAGTCAGATACGAC-1 Patient5 Monocytes nonactive 4 0.05754232 Monocytes_4
10x_3288_t1_CAGTCAGATGCAGT-1 Patient5 Progenitors nonactive 1 0.11524317 Progenitors_1
10x_3288_t1_CAGTGATGGACAAA-1 Patient5 Monocytes nonactive 0 0.04029548 Monocytes_0
10x_3288_t1_CAGTGATGGGAAAT-1 Patient5 Progenitors active 1 0.22151417 Progenitors_1
10x_3288_t1_CAGTGATGGGTGAG-1 Patient5 Monocytes nonactive 4 0.01215839 Monocytes_4
10x_3288_t1_CAGTGATGGTTTGG-1 Patient5 Monocytes nonactive 0 0.06556338 Monocytes_0

10x_3288_t1_CAGTGATGTAACCG-1 Patient5 Progenitors active 1 0.1492613 Progenitors_1
10x_3288_t1_CAGTGATGTACAGC-1 Patient5 Progenitors active 1 0.13152305 Progenitors_1
10x_3288_t1_CAGTGATGTCCCAC-1 Patient5 Progenitors active 1 0.18193203 Progenitors_1
10x_3288_t1_CAGTGATGTCCGAA-1 Patient5 Monocytes nonactive 4 0.03268658 Monocytes_4
10x_3288_t1_CAGTGTGACTAGCA-1 Patient5 Monocytes active 4 0.11964999 Monocytes_4
10x_3288_t1_CAGTGTGACTCAAG-1 Patient5 Monocytes nonactive 4 0.02785175 Monocytes_4
10x_3288_t1_CAGTGTGATGCGTA-1 Patient5 Progenitors active 1 0.12776615 Progenitors_1
10x_3288_t1_CAGTTACTAGTACC-1 Patient5 Monocytes nonactive 8 0.01884789 Monocytes_8
10x_3288_t1_CAGTTACTCTAAGC-1 Patient5 Monocytes nonactive 13 0.01039883 Monocytes_13
10x_3288_t1_CAGTTACTGCGAGA-1 Patient5 Progenitors nonactive 1 0.11235813 Progenitors_1
10x_3288_t1_CAGTTACTTTGGCA-1 Patient5 Monocytes nonactive 4 0.05858855 Monocytes_4
10x_3288_t1_CAGTTGGAAGACTC-1 Patient5 Dendritic cells active 10 0.12970008 Dendritic cells_10
10x_3288_t1_CAGTTGGACATGAC-1 Patient5 Progenitors active 1 0.13960751 Progenitors_1
10x_3288_t1_CAGTTGGAGCAAGG-1 Patient5 Progenitors active 1 0.17743009 Progenitors_1
10x_3288_t1_CAGTTTACAGGTTC-1 Patient5 Progenitors active 1 0.15731406 Progenitors_1
10x_3288_t1_CAGTTTACAGTGCT-1 Patient5 Progenitors active 6 0.12830512 Progenitors_6
10x_3288_t1_CAGTTTACTCTTTG-1Patient5 Monocytes nonactive 4 0.07144442

Monocytes_4

10x_3288_t1_CAGTTTACTGCAAC-1 Patient5 Monocytes nonactive 0 0.03488999

Monocytes_0

10x_3288_t1_CAGTTTACTGCCAA-1 Patient5 Progenitors active 1 0.14347537

Progenitors_1

10x_3288_t1_CATAAAACCCCACT-1 Patient5 Progenitors active 1 0.15382664

Progenitors_1

10x_3288_t1_CATAAAACGAGCTT-1 Patient5 Progenitors active 1 0.15883584

Progenitors_1

10x_3288_t1_CATAAAACTCCTTA-1 Patient5 Progenitors active 1 0.18960434

Progenitors_1

10x_3288_t1_CATAAAACTGGTTG-1 Patient5 Monocytes nonactive 0 0.0771828

Monocytes_0

10x_3288_t1_CATAAATGCACTAG-1 Patient5 Monocytes nonactive 4 0.03368525

Monocytes_4

10x_3288_t1_CATAAATGGTAGGG-1 Patient5 Monocytes nonactive 0 0.06318559

Monocytes_0

10x_3288_t1_CATAAATGGTCGAT-1 Patient5 Monocytes nonactive 0 0.04966394

Monocytes_0

10x_3288_t1_CATAAATGTTCTAC-1 Patient5 Progenitors active 1 0.13334601

Progenitors_1

10x_3288_t1_CATAACCTCTCGAA-1 Patient5 Progenitors active 1 0.14529833

Progenitors_1

10x_3288_t1_CATAACCTGGAAGC-1 Patient5 Progenitors active 1 0.12716378

Progenitors_1

10x_3288_t1_CATAACCTGTCTTT-1 Patient5 Progenitors active 1 0.14761271

Progenitors_1

10x_3288_t1_CATAACCTTGGAAA-1 Patient5 Monocytes nonactive 0 0.02653605

Monocytes_0

10x_3288_t1_CATAACCTTTCTGT-1 Patient5 Monocytes nonactive 0 0.03893222

Monocytes_0

10x_3288_t1_CATACTACAGCTCA-1 Patient5 Monocytes nonactive 0 0.02289011

Monocytes_0

10x_3288_t1_CATACTACGAATGA-1 Patient5 Monocytes nonactive 4 0.02376197
Monocytes_4
10x_3288_t1_CATACTACGATACC-1 Patient5 Monocytes nonactive 8 0.05800203
Monocytes_8
10x_3288_t1_CATAGTCTACTGTG-1 Patient5 Progenitors active 1 0.13689684
Progenitors_1
10x_3288_t1_CATAGTCTGTACGT-1 Patient5 Monocytes nonactive 8 0.03552406
Monocytes_8
10x_3288_t1_CATAGTCTTGCAGT-1 Patient5 Monocytes nonactive 0 0.04367193
Monocytes_0
10x_3288_t1_CATATAGACTCTAT-1 Patient5 Progenitors active 1 0.14870649
Progenitors_1
10x_3288_t1_CATATAGACTTAGG-1 Patient5 Progenitors nonactive 1 0.1024824
Progenitors_1
10x_3288_t1_CATATAGATCTTTG-1 Patient5 Progenitors active 1 0.16390844
Progenitors_1
10x_3288_t1_CATCAACTCGCCTT-1 Patient5 Progenitors active 1 0.16278296
Progenitors_1
10x_3288_t1_CATCAACTGAGAGC-1 Patient5 Monocytes nonactive 4 0.05448291
Monocytes_4
10x_3288_t1_CATCAGGACCTGTC-1 Patient5 Progenitors active 1 0.12020481
Progenitors_1
10x_3288_t1_CATCAGGAGAGAGC-1 Patient5 Monocytes nonactive 0 0.07502695
Monocytes_0
10x_3288_t1_CATCAGGAGGACAG-1 Patient5 Progenitors active 1 0.14022573
Progenitors_1
10x_3288_t1_CATCAGGATCGTTT-1 Patient5 Progenitors nonactive 1 0.10869634
Progenitors_1
10x_3288_t1_CATCATACCTCTAT-1 Patient5 Progenitors active 1 0.15807495
Progenitors_1
10x_3288_t1_CATCATACGGATTC-1 Patient5 Monocytes nonactive 0 0.0484592
Monocytes_0
10x_3288_t1_CATCATACTCATTC-1 Patient5 Progenitors active 1 0.16772874

Progenitors_1

10x_3288_t1_CATCCCGAACACAC-1 Patient5 Progenitors active 1 0.18709974 Progenitors_1

10x_3288_t1_CATCCCGACAGCTA-1 Patient5 Monocytes nonactive 4 0.00962209 Monocytes_4

10x_3288_t1_CATCCCGACTACCC-1 Patient5 Progenitors active 1 0.12981105 Progenitors_1

10x_3288_t1_CATCCCGAGCCAAT-1 Patient5 Monocytes nonactive 8 0.05511699 Monocytes_8

10x_3288_t1_CATCCCGAGCCTTC-1 Patient5 Progenitors active 1 0.18386596 Progenitors_1

10x_3288_t1_CATCGCTGCTTTAC-1 Patient5 Monocytes nonactive 4 0.02271574 Monocytes_4

10x_3288_t1_CATCGGCTCTCATT-1 Patient5 Progenitors active 1 0.17026504 Progenitors_1

10x_3288_t1_CATCTCCTCATTGG-1 Patient5 Progenitors active 1 0.12595904 Progenitors_1

10x_3288_t1_CATCTCCTCCTACC-1 Patient5 Monocytes nonactive 0 0.03660199 Monocytes_0

10x_3288_t1_CATCTCCTCGAGTT-1 Patient5 Monocytes nonactive 0 0.04727031 Monocytes_0

10x_3288_t1_CATCTCCTCTAGAC-1 Patient5 Monocytes nonactive 0 0.05357935 Monocytes_0

10x_3288_t1_CATCTTGACTACTT-1 Patient5 Monocytes nonactive 8 0.0308002 Monocytes_8

10x_3288_t1_CATCTTGATTCTTG-1 Patient5 Progenitors active 1 0.14412529 Progenitors_1

10x_3288_t1_CATGAGACATCGTG-1 Patient5 Monocytes nonactive 8 0.01020861 Monocytes_8

10x_3288_t1_CATGAGACCATGCA-1 Patient5 Progenitors active 1 0.13353624 Progenitors_1

10x_3288_t1_CATGAGACCTGCTC-1 Patient5 Monocytes nonactive 0 0.06692664 Monocytes_0

10x_3288_t1_CATGAGACTGCGTA-1 Patient5 Monocytes nonactive 4 0.0168347 Monocytes_4
10x_3288_t1_CATGAGACTTCCAT-1 Patient5 Monocytes nonactive 0 0.02751886 Monocytes_0
10x_3288_t1_CATGCCACATTCTC-1 Patient5 Progenitors active 6 0.14477522 Progenitors_6
10x_3288_t1_CATGCCACGTCTGA-1 Patient5 Monocytes nonactive 8 0.06973242 Monocytes_8
10x_3288_t1_CATGCCACTTCATC-1 Patient5 Progenitors nonactive 1 0.10200685 Progenitors_1
10x_3288_t1_CATGCGCTCCACCT-1 Patient5 Progenitors active 1 0.11999873 Progenitors_1
10x_3288_t1_CATGCGCTGCTATG-1 Patient5 Monocytes nonactive 4 0.03238539 Monocytes_4
10x_3288_t1_CATGGATGATCGTG-1 Patient5 Monocytes nonactive 0 0.06266248 Monocytes_0
10x_3288_t1_CATGGATGTTACTC-1 Patient5 Monocytes nonactive 8 0.02249382 Monocytes_8
10x_3288_t1_CATGGCCTAAAACG-1 Patient5 Progenitors active 1 0.17459261 Progenitors_1
10x_3288_t1_CATGGCCTAACCTG-1 Patient5 Progenitors active 1 0.14767611 Progenitors_1
10x_3288_t1_CATGGCCTGAGCAG-1 Patient5 Dendritic cells nonactive 10 0.1091719 Dendritic cells_10
10x_3288_t1_CATGTACTCACTTT-1 Patient5 Monocytes nonactive 4 0.04402067 Monocytes_4
10x_3288_t1_CATGTACTCCGTTC-1 Patient5 Progenitors active 1 0.15842369 Progenitors_1
10x_3288_t1_CATGTACTGCAAGG-1 Patient5 Monocytes nonactive 4 0.07773762 Monocytes_4
10x_3288_t1_CATGTTACCCTGTC-1 Patient5 Progenitors active 1 0.12448481 Progenitors_1
10x_3288_t1_CATGTTACGCAAGG-1 Patient5 Progenitors active 1 0.15541183

Progenitors_1

10x_3288_t1_CATGTTACTACTGG-1 Patient5 Monocytes nonactive 0 0.0531038 Monocytes_0

10x_3288_t1_CATGTTACTGCTAG-1 Patient5 Progenitors active 1 0.11979266 Progenitors_1

10x_3288_t1_CATGTTTGCTTATC-1 Patient5 Monocytes nonactive 0 0.0383457 Monocytes_0

10x_3288_t1_CATGTTTGGGTTCA-1 Patient5 Monocytes nonactive 4 0.0531038 Monocytes_4

10x_3288_t1_CATGTTTGTGTCGA-1 Patient5 Monocytes nonactive 0 0.02523619 Monocytes_0

10x_3288_t1_CATTACACAGGCGA-1 Patient5 Progenitors active 1 0.15396931 Progenitors_1

10x_3288_t1_CATTACACAGTGCT-1 Patient5 Progenitors active 1 0.14815167 Progenitors_1

10x_3288_t1_CATTACACCAGATC-1 Patient5 Monocytes nonactive 4 0.04524127 Monocytes_4

10x_3288_t1_CATTAGCTAGAAGT-1 Patient5 Progenitors nonactive 1 0.11445057 Progenitors_1

10x_3288_t1_CATTAGCTGATGAA-1 Patient5 Monocytes nonactive 0 0.07505865 Monocytes_0

10x_3288_t1_CATTAGCTGCCATA-1 Patient5 Monocytes nonactive 0 0.0358094 Monocytes_0

10x_3288_t1_CATTAGCTGTATCG-1 Patient5 Monocytes nonactive 4 0.04682645 Monocytes_4

10x_3288_t1_CATTAGCTGTGTCA-1 Patient5 Monocytes nonactive 4 0.07859362 Monocytes_4

10x_3288_t1_CATTAGCTTCTCGC-1 Patient5 Monocytes nonactive 4 0.0371251 Monocytes_4

10x_3288_t1_CATTCCCTCTCCCA-1 Patient5 Monocytes nonactive 4 0.07650117 Monocytes_4

10x_3288_t1_CATTCCCTGGTATC-1 Patient5 Monocytes nonactive 4 0.03060998 Monocytes_4

10x_3288_t1_CATTCCCTTAACGC-1 Patient5 Monocytes nonactive 4 0.06491345 Monocytes_4
10x_3288_t1_CATTGACTAATCGC-1 Patient5 Monocytes nonactive 0 0.05779595 Monocytes_0
10x_3288_t1_CATTGACTCTGTAG-1 Patient5 Monocytes nonactive 4 0.03682392 Monocytes_4
10x_3288_t1_CATTGACTTCCGTC-1 Patient5 Monocytes nonactive 0 0.06080781 Monocytes_0
10x_3288_t1_CATTGACTTGAGCT-1 Patient5 Progenitors active 1 0.13340942 Progenitors_1
10x_3288_t1_CATTGACTTTCCCG-1 Patient5 Progenitors active 1 0.13764187 Progenitors_1
10x_3288_t1_CATTGGGAGCGTTA-1 Patient5 Monocytes nonactive 8 0.05275506 Monocytes_8
10x_3288_t1_CATTGGGATAAAGG-1 Patient5 Progenitors active 1 0.1564422 Progenitors_1
10x_3288_t1_CATTGTACCCTTAT-1 Patient5 Monocytes nonactive 4 0.0412783 Monocytes_4
10x_3288_t1_CATTGTACTACGCA-1 Patient5 Progenitors active 1 0.13044512 Progenitors_1
10x_3288_t1_CATTGTACTTGGCA-1 Patient5 Monocytes nonactive 0 0.05497432 Monocytes_0
10x_3288_t1_CATTGTTGGAACCT-1 Patient5 Monocytes nonactive 4 0.01862596 Monocytes_4
10x_3288_t1_CATTGTTGTTCGTT-1 Patient5 Progenitors active 1 0.14670915 Progenitors_1
10x_3288_t1_CATTTCGACACACA-1 Patient5 Monocytes nonactive 4 0.03890051 Monocytes_4
10x_3288_t1_CATTTCGATGGGAG-1 Patient5 Progenitors active 1 0.12866971 Progenitors_1
10x_3288_t1_CATTTGACAGGGTG-1 Patient5 Monocytes nonactive 4 0.02729694 Monocytes_4
10x_3288_t1_CATTTGACTGTTTC-1 Patient5 Monocytes nonactive 0 0.08119333

Monocytes_0

10x_3288_t1_CATTTGACTTCGCC-1 Patient5 Progenitors active 1 0.1627354 Progenitors_1

10x_3288_t1_CATTTGACTTGCTT-1 Patient5 Monocytes nonactive 4 0.04736542 Monocytes_4

10x_3288_t1_CATTTGTGAACGTC-1 Patient5 Monocytes nonactive 0 0.059825 Monocytes_0

10x_3288_t1_CATTTGTGTCAGGT-1Patient5 Monocytes nonactive 4 0.02926257 Monocytes_4

10x_3288_t1_CATTTGTGTGCTGA-1 Patient5 Monocytes nonactive 8 0.02258893 Monocytes_8

10x_3288_t1_CCAAAGTGAACAGA-1 Patient5 Monocytes nonactive 0 0.04111978 Monocytes_0

10x_3288_t1_CCAAAGTGGCTTCC-1 Patient5 Monocytes nonactive 8 0.06033226 Monocytes_8

10x_3288_t1_CCAACCTGCTGTAG-1 Patient5 Dendritic cells active 10 0.13467757 Dendritic cells_10

10x_3288_t1_CCAACCTGGAGAGC-1 Patient5 Monocytes nonactive 4 0.04872868 Monocytes_4

10x_3288_t1_CCAACCTGGGGCAA-1 Patient5 Monocytes nonactive 0 0.03650688 Monocytes_0

10x_3288_t1_CCAAGAACAGACTC-1 Patient5 Monocytes nonactive 0 0.03324139 Monocytes_0

10x_3288_t1_CCAAGAACCCCTTG-1 Patient5 Monocytes nonactive 4 0.02311204 Monocytes_4

10x_3288_t1_CCAAGAACGACAAA-1 Patient5 Monocytes nonactive 4 0.05727284 Monocytes_4

10x_3288_t1_CCAAGATGCTTCGC-1 Patient5 Monocytes nonactive 0 0.04069178 Monocytes_0

10x_3288_t1_CCAAGATGGTACGT-1 Patient5 Progenitors active 1 0.14780293 Progenitors_1

10x_3288_t1_CCAAGTGAAGGCGA-1 Patient5 Progenitors nonactive 1 0.1109156 Progenitors_1

10x_3288_t1_CCAAGTGACACTCC-1 Patient5 Monocytes nonactive 0 0.0413417
Monocytes_0
10x_3288_t1_CCAAGTGACTTAGG-1 Patient5 Monocytes nonactive 4 0.0147264
Monocytes_4
10x_3288_t1_CCAATGGAATGCCA-1 Patient5 Monocytes nonactive 4 0.06469152
Monocytes_4
10x_3288_t1_CCAATGGACAGATC-1 Patient5 Progenitors nonactive 5 0.10817323
Progenitors_5
10x_3288_t1_CCAATGGACTCCAC-1 Patient5 Monocytes nonactive 4 0.01303025
Monocytes_4
10x_3288_t1_CCAATTTGACGTAC-1 Patient5 Monocytes nonactive 0 0.03558747
Monocytes_0
10x_3288_t1_CCAATTTGGCCAAT-1Patient5 Monocytes nonactive 0 0.040882
Monocytes_0
10x_3288_t1_CCAATTTGGGTAAA-1 Patient5 Monocytes nonactive 4 0.04454378
Monocytes_4
10x_3288_t1_CCACCATGAGAGGC-1 Patient5 Monocytes nonactive 0 0.00163274
Monocytes_0
10x_3288_t1_CCACCATGGCTTAG-1 Patient5 Progenitors nonactive 1 0.10828419
Progenitors_1
10x_3288_t1_CCACCATGGTGTCA-1 Patient5 Monocytes nonactive 0 0.03514362
Monocytes_0
10x_3288_t1_CCACCATGTGAGAA-1 Patient5 Progenitors nonactive 1 0.11568702
Progenitors_1
10x_3288_t1_CCACCTGAAAGGGC-1 Patient5 Monocytes nonactive 4 0.05078942
Monocytes_4
10x_3288_t1_CCACCTGACCTTAT-1Patient5 Progenitors active 1 0.14664574
Progenitors_1
10x_3288_t1_CCACCTGAGTAAGA-1 Patient5 Monocytes nonactive 4 0.07120665
Monocytes_4
10x_3288_t1_CCACGGGAAAGTAG-1 Patient5 Monocytes nonactive 0 0.04822142
Monocytes_0
10x_3288_t1_CCACTGACACCGAT-1 Patient5 Progenitors nonactive 1 0.08862786

Progenitors_1
10x_3288_t1_CCACTGACACTGGT-1 Patient5 Progenitors nonactive 1 0.11716125
Progenitors_1
10x_3288_t1_CCACTGACGTATCG-1 Patient5 Monocytes nonactive 0 0.08848519
Monocytes_0
10x_3288_t1_CCACTGTGCAATCG-1 Patient5 Monocytes nonactive 0 0.02815294
Monocytes_0
10x_3288_t1_CCACTGTGGGCGAA-1 Patient5 Monocytes nonactive 0 0.05264409
Monocytes_0
10x_3288_t1_CCACTGTGTTCCAT-1 Patient5 Monocytes nonactive 4 0.05587788
Monocytes_4
10x_3288_t1_CCACTTCTACTCAG-1 Patient5 Monocytes nonactive 4 0.03236954
Monocytes_4
10x_3288_t1_CCACTTCTTACTTC-1 Patient5 Progenitors active 1 0.12879653
Progenitors_1
10x_3288_t1_CCAGAAACAACGGG-1 Patient5 Monocytes nonactive 0 0.0564961
Monocytes_0
10x_3288_t1_CCAGAAACCAGAGG-1 Patient5 Progenitors active 1 0.15198783
Progenitors_1
10x_3288_t1_CCAGAAACCCACAA-1 Patient5 Monocytes nonactive 0 0.02238285
Monocytes_0
10x_3288_t1_CCAGACCTACGTGT-1 Patient5 Monocytes nonactive 0 0.05161372
Monocytes_0
10x_3288_t1_CCAGACCTCTTACT-1 Patient5 Progenitors active 1 0.12928793
Progenitors_1
10x_3288_t1_CCAGACCTCTTGTT-1 Patient5 Monocytes nonactive 0 0.07142857
Monocytes_0
10x_3288_t1_CCAGACCTGTAGGG-1 Patient5 Dendritic cells nonactive 10 0.09688669
Dendritic cells_10
10x_3288_t1_CCAGACCTTTAGGC-1 Patient5 Monocytes nonactive 4 0.04972735
Monocytes_4
10x_3288_t1_CCAGATGACTTCGC-1 Patient5 Progenitors active 6 0.15414368
Progenitors_6

10x_3288_t1_CCAGATGAGTGTCA-1 Patient5 Progenitors active 1 0.1400038 Progenitors_1
10x_3288_t1_CCAGCACTCAACCA-1 Patient5 Progenitors active 1 0.12641874 Progenitors_1
10x_3288_t1_CCAGCACTGAATGA-1 Patient5 Monocytes nonactive 0 0.05998351 Monocytes_0
10x_3288_t1_CCAGCACTGGTAGG-1 Patient5 Dendritic cells nonactive 10 0.08282607 Dendritic cells_10
10x_3288_t1_CCAGCACTTCGCAA-1 Patient5 Monocytes nonactive 4 0.05243802 Monocytes_4
10x_3288_t1_CCAGCACTTGCAGT-1 Patient5 Dendritic cells active 10 0.14791389 Dendritic cells_10
10x_3288_t1_CCAGCACTTGTGGT-1 Patient5 Progenitors active 1 0.16690444 Progenitors_1
10x_3288_t1_CCAGCGGAGCCTTC-1 Patient5 Monocytes nonactive 4 0.05543402 Monocytes_4
10x_3288_t1_CCAGCGGATACGAC-1 Patient5 Monocytes nonactive 13 0.0509955 Monocytes_13
10x_3288_t1_CCAGCGGATTCTTG-1 Patient5 Progenitors active 1 0.14526663 Progenitors_1
10x_3288_t1_CCAGCTACCCGAAT-1 Patient5 Progenitors active 1 0.11863547 Progenitors_1
10x_3288_t1_CCAGCTACCCGATA-1 Patient5 Monocytes nonactive 0 0.05278676 Monocytes_0
10x_3288_t1_CCAGGTCTAAGAAC-1 Patient5 Progenitors active 1 0.15698117 Progenitors_1
10x_3288_t1_CCAGGTCTCAACTG-1 Patient5 Monocytes nonactive 4 0.05437195 Monocytes_4
10x_3288_t1_CCAGGTCTGCCAAT-1 Patient5 Monocytes nonactive 8 0.01325217 Monocytes_8
10x_3288_t1_CCAGGTCTGTCACA-1 Patient5 Monocytes nonactive 8 0.06161626 Monocytes_8
10x_3288_t1_CCAGTCACGTTCAG-1 Patient5 Progenitors active 1 0.1285429

Progenitors_1

10x_3288_t1_CCAGTCTGGCGAAG-1 Patient5 Monocytes nonactive 0 0.02537886 Monocytes_0

10x_3288_t1_CCAGTGCTAACGTC-1 Patient5 Monocytes nonactive 8 0.04506689 Monocytes_8

10x_3288_t1_CCAGTGCTCGAGTT-1 Patient5 Monocytes nonactive 4 0.04895061 Monocytes_4

10x_3288_t1_CCAGTGCTCTTACT-1Patient5 Monocytes nonactive 4 0.06500856 Monocytes_4

10x_3288_t1_CCAGTGCTGAAACA-1 Patient5 Monocytes nonactive 8 0.03130746 Monocytes_8

10x_3288_t1_CCAGTGCTGATACC-1 Patient5 Monocytes nonactive 0 0.06190159 Monocytes_0

10x_3288_t1_CCAGTGCTGGCAAG-1 Patient5 Progenitors active 1 0.13126942 Progenitors_1

10x_3288_t1_CCAGTGCTTCCCGT-1 Patient5 Monocytes nonactive 4 0.03313043 Monocytes_4

10x_3288_t1_CCAGTGCTTGTAGC-1 Patient5 Monocytes nonactive 8 0.04414749 Monocytes_8

10x_3288_t1_CCATAGGAACCTCC-1 Patient5 Monocytes nonactive 0 0.03255976 Monocytes_0

10x_3288_t1_CCATAGGAAGCAAA-1 Patient5 Monocytes nonactive 4 0.10113499 Monocytes_4

10x_3288_t1_CCATAGGAATGTGC-1 Patient5 Monocytes nonactive 8 0.03214761 Monocytes_8

10x_3288_t1_CCATAGGAGTTCTT-1Patient5 Progenitors active 6 0.15408027 Progenitors_6

10x_3288_t1_CCATATACTGGTTG-1Patient5 Monocytes nonactive 4 0.05269165 Monocytes_4

10x_3288_t1_CCATCCGAAACCAC-1 Patient5 Monocytes nonactive 8 0.04267326 Monocytes_8

10x_3288_t1_CCATCCGAAGAGTA-1 Patient5 Monocytes nonactive 0 0.03118065 Monocytes_0

10x_3288_t1_CCATCCGAATGACC-1 Patient5 Progenitors active 1 0.14288885 Progenitors_1
10x_3288_t1_CCATCCGACTACTT-1 Patient5 Progenitors nonactive 1 0.08244563 Progenitors_1
10x_3288_t1_CCATCCGACTAGCA-1 Patient5 Monocytes nonactive 8 0.03622155 Monocytes_8
10x_3288_t1_CCATCCGAGAACTC-1 Patient5 Monocytes nonactive 4 0.0042166 Monocytes_4
10x_3288_t1_CCATCCGATTGTGG-1 Patient5 Monocytes nonactive 0 0.02488745 Monocytes_0
10x_3288_t1_CCATCGTGATCAGC-1 Patient5 Monocytes nonactive 4 0.02899309 Monocytes_4
10x_3288_t1_CCATCGTGTGACCA-1 Patient5 Dendritic cells active 10 0.13494705 Dendritic cells_10
10x_3288_t1_CCATGCTGAATGCC-1 Patient5 Monocytes nonactive 13 0.02636168 Monocytes_13
10x_3288_t1_CCATGCTGACTTTC-1 Patient5 Monocytes nonactive 0 0.04012111 Monocytes_0
10x_3288_t1_CCATTAACCCTTCG-1 Patient5 Monocytes nonactive 0 0.03910659 Monocytes_0
10x_3288_t1_CCATTAACCTGAAC-1 Patient5 Monocytes nonactive 4 0.07087376 Monocytes_4
10x_3288_t1_CCATTAACGTTGAC-1 Patient5 Dendritic cells nonactive 10 0.10444804 Dendritic cells_10
10x_3288_t1_CCCAAAGACGCATA-1 Patient5 Monocytes nonactive 0 0.03790185 Monocytes_0
10x_3288_t1_CCCAAAGACTGTGA-1 Patient5 Monocytes nonactive 15 0.02980153 Monocytes_15
10x_3288_t1_CCCAAAGACTTGGA-1 Patient5 Progenitors active 1 0.13375816 Progenitors_1
10x_3288_t1_CCCAAAGAGAGGCA-1 Patient5 Monocytes nonactive 0 0.03741044 Monocytes_0
10x_3288_t1_CCCAACACCACTCC-1 Patient5 Progenitors nonactive 1 0.09582461

Progenitors_1

10x_3288_t1_CCCAACACCTTGCC-1 Patient5 Progenitors nonactive 1 0.11015471 Progenitors_1

10x_3288_t1_CCCAACTGACCAAC-1 Patient5 Progenitors nonactive 1 0.1155285 Progenitors_1

10x_3288_t1_CCCAACTGACCTGA-1 Patient5 Progenitors active 1 0.1390844 Progenitors_1

10x_3288_t1_CCCAACTGACGTGT-1 Patient5 Progenitors active 1 0.1298903 Progenitors_1

10x_3288_t1_CCCAACTGTCCTTA-1 Patient5 Monocytes nonactive 4 0.03674466 Monocytes_4

10x_3288_t1_CCCAACTGTGTCCC-1 Patient5 Progenitors active 1 0.22584173 Progenitors_1

10x_3288_t1_CCCACATGAGATGA-1 Patient5 Progenitors active 1 0.12451652 Progenitors_1

10x_3288_t1_CCCACATGCATTGG-1 Patient5 Dendritic cells nonactive 10 0.0742185 Dendritic cells_10

10x_3288_t1_CCCAGACTATTGGC-1 Patient5 Progenitors active 1 0.17421216 Progenitors_1

10x_3288_t1_CCCAGACTGCCAAT-1 Patient5 Progenitors active 1 0.18380255 Progenitors_1

10x_3288_t1_CCCAGACTGTAGGG-1 Patient5 Monocytes nonactive 4 0.06752901 Monocytes_4

10x_3288_t1_CCCAGACTTCACCC-1 Patient5 Progenitors nonactive 1 0.08610741 Progenitors_1

10x_3288_t1_CCCAGACTTTCGTT-1 Patient5 Monocytes nonactive 8 0.09498447 Monocytes_8

10x_3288_t1_CCCAGTTGCAAAGA-1 Patient5 Monocytes nonactive 0 0.07505865 Monocytes_0

10x_3288_t1_CCCATCGAACCAGT-1 Patient5 Monocytes nonactive 13 0.00711749 Monocytes_13

10x_3288_t1_CCCATCGACACACA-1 Patient5 Progenitors active 1 0.12879653 Progenitors_1

10x_3288_t1_CCCATCGACTCGAA-1 Patient5 Monocytes nonactive 4 0.04669964 Monocytes_4
10x_3288_t1_CCCATCGATCCCAC-1 Patient5 Monocytes nonactive 15 0.07236383 Monocytes_15
10x_3288_t1_CCCATGTGATGCCA-1 Patient5 Progenitors active 1 0.13989284 Progenitors_1
10x_3288_t1_CCCGATTGCAACTG-1 Patient5 Monocytes nonactive 0 0.08052755 Monocytes_0
10x_3288_t1_CCCGATTGGGAACG-1 Patient5 Monocytes nonactive 4 0.02409486 Monocytes_4
10x_3288_t1_CCCGATTGTATTCC-1 Patient5 Monocytes nonactive 0 0.03974066 Monocytes_0
10x_3288_t1_CCCGGAGAACGTGT-1 Patient5 Monocytes nonactive 0 0.01529706 Monocytes_0
10x_3288_t1_CCCGGAGAAGATGA-1 Patient5 Monocytes nonactive 4 0.05816055 Monocytes_4
10x_3288_t1_CCCGGAGACTTAGG-1 Patient5 Monocytes nonactive 0 0.0328768 Monocytes_0
10x_3288_t1_CCCGGAGATGCTGA-1 Patient5 Progenitors active 1 0.14475937 Progenitors_1
10x_3288_t1_CCCGGAGATTCTAC-1 Patient5 Monocytes nonactive 0 0.01127069 Monocytes_0
10x_3288_t1_CCCTACGACTATGG-1 Patient5 Monocytes nonactive 4 0.04538393 Monocytes_4
10x_3288_t1_CCCTACGAGAGGTG-1 Patient5 Monocytes nonactive 0 0.03769577 Monocytes_0
10x_3288_t1_CCCTAGTGATTTCC-1Patient5 Monocytes nonactive 4 0.0923372 Monocytes_4
10x_3288_t1_CCCTAGTGTTACTC-1Patient5 Progenitors active 1 0.14055862 Progenitors_1
10x_3288_t1_CCCTGAACCACTCC-1 Patient5 Progenitors active 1 0.13990869 Progenitors_1
10x_3288_t1_CCCTGAACCGTGAT-1 Patient5 Monocytes nonactive 8 0.03404984

Monocytes_8
10x_3288_t1_CCCTGAACGATGAA-1 Patient5 Progenitors active 1 0.21102023
Progenitors_1
10x_3288_t1_CCCTGATGCTTCTA-1 Patient5 Progenitors active 1 0.15102086
Progenitors_1
10x_3288_t1_CCCTGATGTATTCC-1 Patient5 Progenitors active 1 0.13417031
Progenitors_1
10x_3288_t1_CCCTGATGTCTAGG-1 Patient5 Monocytes nonactive 0 0.03355843
Monocytes_0
10x_3288_t1_CCCTTACTCGGTAT-1 Patient5 Progenitors active 1 0.12825756
Progenitors_1
10x_3288_t1_CCGAAAACGCAAGG-1 Patient5 Monocytes nonactive 4 0.06014203
Monocytes_4
10x_3288_t1_CCGAAAACTTAGGC-1 Patient5 Progenitors nonactive 1 0.10796715
Progenitors_1
10x_3288_t1_CCGAAAACTTCCCG-1 Patient5 Progenitors active 1 0.15190857
Progenitors_1
10x_3288_t1_CCGACACTGTGTTG-1 Patient5 Monocytes nonactive 4 0.06748145
Monocytes_4
10x_3288_t1_CCGATAGACACTTT-1 Patient5 Progenitors active 6 0.12445311
Progenitors_6
10x_3288_t1_CCGATAGATCCAAG-1 Patient5 Progenitors active 1 0.1429047
Progenitors_1
10x_3288_t1_CCGATAGATCCGTC-1 Patient5 Monocytes nonactive 4 0.03099043
Monocytes_4
10x_3288_t1_CCGCGAGACGTTGA-1 Patient5 Monocytes nonactive 0 0.04847505
Monocytes_0
10x_3288_t1_CCGCTATGCATCAG-1 Patient5 Monocytes nonactive 0 0.03701414
Monocytes_0
10x_3288_t1_CCGCTATGGTTAGC-1 Patient5 Progenitors nonactive 1 0.08637689
Progenitors_1
10x_3288_t1_CCGCTATGTAAGGA-1 Patient5 Monocytes nonactive 4 0.09585632
Monocytes_4

10x_3288_t1_CCGCTATGTTAGGC-1 Patient5 Progenitors active 6 0.17570224 Progenitors_6
10x_3288_t1_CCGGAGACATGACC-1 Patient5 Monocytes nonactive 4 0.03945533 Monocytes_4
10x_3288_t1_CCGGAGACCATGGT-1 Patient5 Monocytes nonactive 4 0.03222687 Monocytes_4
10x_3288_t1_CCGGAGACGTGTAC-1 Patient5 Monocytes nonactive 4 0.04901401 Monocytes_4
10x_3288_t1_CCGGAGACTCGCAA-1 Patient5 Monocytes nonactive 4 0.04774586 Monocytes_4
10x_3288_t1_CCGGAGACTGACAC-1 Patient5 Progenitors nonactive 5 0.04107222 Progenitors_5
10x_3288_t1_CCGGAGACTGAGGG-1 Patient5 Progenitors active 1 0.14572633 Progenitors_1
10x_3288_t1_CCGGAGACTGTGCA-1 Patient5 Progenitors active 1 0.12859045 Progenitors_1
10x_3288_t1_CCGGAGTGAAGGGC-1 Patient5 Monocytes nonactive 0 0.0779754 Monocytes_0
10x_3288_t1_CCGGAGTGACTACG-1 Patient5 Monocytes nonactive 4 0.03295606 Monocytes_4
10x_3288_t1_CCGGAGTGAGAAGT-1 Patient5 Monocytes nonactive 15 0.05034557 Monocytes_15
10x_3288_t1_CCGGTACTAACAGA-1 Patient5 Monocytes nonactive 0 0.01992581 Monocytes_0
10x_3288_t1_CCGGTACTCACTTT-1Patient5 Progenitors active 1 0.15520576 Progenitors_1
10x_3288_t1_CCGTAAGAAGCACT-1 Patient5 Monocytes nonactive 0 0.01531292 Monocytes_0
10x_3288_t1_CCGTAAGACACTAG-1 Patient5 Progenitors nonactive 1 0.09343098 Progenitors_1
10x_3288_t1_CCGTAAGACGTACA-1 Patient5 Monocytes nonactive 4 0.06556338 Monocytes_4
10x_3288_t1_CCGTAAGATGTCTT-1Patient5 Progenitors active 1 0.22814026

Progenitors_1

10x_3288_t1_CCGTACACATACCG-1 Patient5 Progenitors active 1 0.14436307 Progenitors_1

10x_3288_t1_CCGTACACCCCACT-1 Patient5 Monocytes nonactive 4 0.04752394 Monocytes_4

10x_3288_t1_CCTAAACTAGCCAT-1Patient5 Progenitors active 1 0.14276203 Progenitors_1

10x_3288_t1_CCTAAACTATCTCT-1 Patient5 Progenitors active 1 0.19569146 Progenitors_1

10x_3288_t1_CCTAAACTCACTCC-1 Patient5 Monocytes nonactive 8 0.06063344 Monocytes_8

10x_3288_t1_CCTAAACTCTCCCA-1 Patient5 Monocytes nonactive 4 0.02902479 Monocytes_4

10x_3288_t1_CCTAAACTCTTATC-1 Patient5 Progenitors active 1 0.11866717 Progenitors_1

10x_3288_t1_CCTAAGGAACCCAA-1 Patient5 Progenitors active 1 0.12792467 Progenitors_1

10x_3288_t1_CCTAAGGACAGGAG-1 Patient5 Monocytes nonactive 4 0.03901148 Monocytes_4

10x_3288_t1_CCTACCGAACAGTC-1 Patient5 Progenitors active 1 0.16037347 Progenitors_1

10x_3288_t1_CCTACCGAAGTCTG-1 Patient5 Monocytes nonactive 8 0.02138419 Monocytes_8

10x_3288_t1_CCTACCGACCGTAA-1 Patient5 Monocytes nonactive 0 0.0480629 Monocytes_0

10x_3288_t1_CCTAGAGAACAGTC-1 Patient5 Monocytes nonactive 4 0.05741551 Monocytes_4

10x_3288_t1_CCTAGAGAACCAAC-1 Patient5 Dendritic cells active 10 0.1412561 Dendritic cells_10

10x_3288_t1_CCTAGAGACTTACT-1 Patient5 Progenitors active 1 0.14169996 Progenitors_1

10x_3288_t1_CCTAGAGAGGCAAG-1 Patient5 Monocytes nonactive 0 0.0033923 Monocytes_0

10x_3288_t1_CCTAGAGAGTTTCT-1 Patient5 Monocytes nonactive 4 0.05830321 Monocytes_4
10x_3288_t1_CCTAGAGATGTCGA-1 Patient5 Progenitors active 1 0.1754169 Progenitors_1
10x_3288_t1_CCTATAACATCGGT-1 Patient5 Monocytes nonactive 4 0.03552406 Monocytes_4
10x_3288_t1_CCTATAACGTTACG-1 Patient5 Monocytes nonactive 4 0.05061505 Monocytes_4
10x_3288_t1_CCTATAACTGAGAA-1 Patient5 Progenitors active 1 0.16864815 Progenitors_1
10x_3288_t1_CCTATAACTTGAGC-1 Patient5 Progenitors active 1 0.12592733 Progenitors_1
10x_3288_t1_CCTATTGAGAGGGT-1 Patient5 Progenitors active 1 0.18223321 Progenitors_1
10x_3288_t1_CCTATTGAGGATCT-1 Patient5 Progenitors active 1 0.14144633 Progenitors_1
10x_3288_t1_CCTCATCTCAAGCT-1 Patient5 Progenitors active 1 0.12106081 Progenitors_1
10x_3288_t1_CCTCGAACCAGTCA-1 Patient5 Monocytes nonactive 0 0.0602847 Monocytes_0
10x_3288_t1_CCTCGAACCCTCAC-1 Patient5 Monocytes nonactive 4 0.02268404 Monocytes_4
10x_3288_t1_CCTCGAACCGACTA-1 Patient5 Monocytes nonactive 4 0.02938939 Monocytes_4
10x_3288_t1_CCTCGAACGCAGAG-1 Patient5 Progenitors active 1 0.15867732 Progenitors_1
10x_3288_t1_CCTCGAACGCTACA-1 Patient5 Dendritic cells nonactive 10 0.06304293 Dendritic cells_10
10x_3288_t1_CCTCGAACGCTGAT-1 Patient5 Monocytes nonactive 4 0.04329148 Monocytes_4
10x_3288_t1_CCTCTACTAAGATG-1 Patient5 Progenitors active 1 0.1618477 Progenitors_1
10x_3288_t1_CCTCTACTAGCTCA-1 Patient5 Progenitors active 1 0.15740917

Progenitors_1

10x_3288_t1_CCTCTACTAGTTCG-1Patient5 Monocytes nonactive 4 0.04497178

Monocytes_4

10x_3288_t1_CCTCTACTCAAGCT-1 Patient5 Monocytes nonactive 4 0.06104559

Monocytes_4

10x_3288_t1_CCTCTACTCGATAC-1Patient5 Monocytes nonactive 4 0.0370617

Monocytes_4

10x_3288_t1_CCTCTACTGCCAAT-1Patient5 Progenitors active 1 0.13413861

Progenitors_1

10x_3288_t1_CCTCTACTGGTTTG-1 Patient5 Monocytes nonactive 4 0.02981739

Monocytes_4

10x_3288_t1_CCTCTACTTAAGCC-1 Patient5 Monocytes nonactive 0 0.086044

Monocytes_0

10x_3288_t1_CCTGACTGACTCAG-1 Patient5 Monocytes nonactive 0 0.04696912

Monocytes_0

10x_3288_t1_CCTGACTGGTTGGT-1 Patient5 Monocytes nonactive 4 0.04850675

Monocytes_4

10x_3288_t1_CCTGAGCTATCGTG-1 Patient5 Progenitors active 1 0.12584808

Progenitors_1

10x_3288_t1_CCTGAGCTCCCGTT-1 Patient5 Dendritic cells nonactive 10 0.0678936

Dendritic cells_10

10x_3288_t1_CCTGAGCTGAGCAG-1 Patient5 Monocytes nonactive 8 0.03522288

Monocytes_8

10x_3288_t1_CCTGCAACCTACGA-1 Patient5 Monocytes nonactive 0 0.02623486

Monocytes_0

10x_3288_t1_CCTGGACTCGATAC-1 Patient5 Progenitors active 1 0.20244436

Progenitors_1

10x_3288_t1_CCTGGACTGGAAAT-1 Patient5 Monocytes nonactive 8 0.06304293

Monocytes_8

10x_3288_t1_CCTTAATGACACTG-1 Patient5 Monocytes nonactive 4 0.07363198

Monocytes_4

10x_3288_t1_CCTTAATGCTCTAT-1 Patient5 Monocytes nonactive 16 0.09701351

Monocytes_16

10x_3288_t1_CCTTAATGGTTAGC-1Patient5 Monocytes nonactive 0 0.0454949
Monocytes_0
10x_3288_t1_CCTTAATGTTCGCC-1Patient5 Monocytes nonactive 0 0.02943694
Monocytes_0
10x_3288_t1_CCTTCACTAGACTC-1 Patient5 Monocytes nonactive 0 0.04961638
Monocytes_0
10x_3288_t1_CCTTCACTTAACGC-1 Patient5 Monocytes nonactive 0 0.06109315
Monocytes_0
10x_3288_t1_CCTTCACTTCGCCT-1Patient5 Monocytes nonactive 0 0.03633251
Monocytes_0
10x_3288_t1_CCTTTAGATTGCTT-1 Patient5 Progenitors nonactive 1 0.11407013
Progenitors_1
10x_3288_t1_CGAACATGCAACCA-1 Patient5 Monocytes nonactive 0 0.04294274
Monocytes_0
10x_3288_t1_CGAACATGCGTTGA-1 Patient5 Monocytes nonactive 0 0.07268087
Monocytes_0
10x_3288_t1_CGAAGGGACATTCT-1 Patient5 Monocytes nonactive 4 0.05919092
Monocytes_4
10x_3288_t1_CGAAGGGAGCGGAA-1 Patient5 Progenitors active 1 0.19413797
Progenitors_1
10x_3288_t1_CGAAGGGAGCTTAG-1 Patient5 Monocytes nonactive 4 0.06340752
Monocytes_4
10x_3288_t1_CGAAGGGATCTTAC-1 Patient5 Monocytes nonactive 0 0.02808953
Monocytes_0
10x_3288_t1_CGAAGGGATTCTGT-1 Patient5 Progenitors active 1 0.18578403
Progenitors_1
10x_3288_t1_CGAATCGACAAAGA-1 Patient5 Monocytes active 8 0.12559445
Monocytes_8
10x_3288_t1_CGAATCGACTAGCA-1 Patient5 Monocytes nonactive 8 0.06960561
Monocytes_8
10x_3288_t1_CGAATCGAGCCTTC-1 Patient5 Progenitors active 1 0.16650815
Progenitors_1
10x_3288_t1_CGAATCGATATCGG-1 Patient5 Monocytes nonactive 8 0.04126244

Monocytes_8
10x_3288_t1_CGAATCGATGCATG-1 Patient5 Progenitors active 1 0.11966584
Progenitors_1
10x_3288_t1_CGACAAACCGCATA-1 Patient5 Monocytes nonactive 0 0.05594128
Monocytes_0
10x_3288_t1_CGACAAACCTCCCA-1 Patient5 Monocytes nonactive 4 0.02721768
Monocytes_4
10x_3288_t1_CGACCACTAACGTC-1 Patient5 Monocytes nonactive 4 0.00881365
Monocytes_4
10x_3288_t1_CGACCACTCGTGTA-1 Patient5 Monocytes nonactive 4 0.02030626
Monocytes_4
10x_3288_t1_CGACCACTTTCATC-1 Patient5 Monocytes nonactive 0 0.01651766
Monocytes_0
10x_3288_t1_CGACCGGAACGTGT-1 Patient5 Progenitors active 1 0.14076469
Progenitors_1
10x_3288_t1_CGACCGGACGACAT-1 Patient5 Progenitors active 1 0.12454822
Progenitors_1
10x_3288_t1_CGACCGGATAAGCC-1 Patient5 Monocytes nonactive 4 0.03336821
Monocytes_4
10x_3288_t1_CGACCTACCCGTTC-1 Patient5 Progenitors active 1 0.17408535
Progenitors_1
10x_3288_t1_CGACCTACGAGGCA-1 Patient5 Progenitors active 1 0.14409359
Progenitors_1
10x_3288_t1_CGACCTACTGGTAC-1 Patient5 Progenitors active 1 0.12389829
Progenitors_1
10x_3288_t1_CGACCTTGAGAATG-1 Patient5 Monocytes nonactive 0 0.05235876
Monocytes_0
10x_3288_t1_CGACCTTGTGCTCC-1 Patient5 Monocytes nonactive 0 0.0682899
Monocytes_0
10x_3288_t1_CGACGTCTAAGTAG-1 Patient5 Progenitors nonactive 6 0.11473591
Progenitors_6
10x_3288_t1_CGACGTCTACCTTT-1Patient5 Monocytes nonactive 0 0.02897724
Monocytes_0

10x_3288_t1_CGACGTCTACTCTT-1Patient5 Monocytes nonactive 4 0.04251474
Monocytes_4
10x_3288_t1_CGACGTCTTTTGTC-1 Patient5 Monocytes nonactive 0 0.05086868
Monocytes_0
10x_3288_t1_CGACTCACAGTACC-1 Patient5 Progenitors active 1 0.16856889
Progenitors_1
10x_3288_t1_CGACTCACGATAGA-1 Patient5 Monocytes nonactive 4 0.02791516
Monocytes_4
10x_3288_t1_CGACTCTGAAGATG-1 Patient5 Progenitors active 1 0.14287299
Progenitors_1
10x_3288_t1_CGACTCTGATTTCC-1 Patient5 Monocytes nonactive 0 0.0286919
Monocytes_0
10x_3288_t1_CGACTCTGGAGACG-1 Patient5 Monocytes nonactive 0 0.01994167
Monocytes_0
10x_3288_t1_CGACTCTGTCTGGA-1 Patient5 Progenitors active 1 0.13073045
Progenitors_1
10x_3288_t1_CGACTCTGTTCGGA-1 Patient5 Monocytes nonactive 0 0.09376387
Monocytes_0
10x_3288_t1_CGACTGCTCTGTAG-1 Patient5 Progenitors nonactive 1 0.1146725
Progenitors_1
10x_3288_t1_CGACTGCTGAGGTG-1 Patient5 Monocytes nonactive 0 0.07012872
Monocytes_0
10x_3288_t1_CGACTGCTGTGAGG-1 Patient5 Monocytes nonactive 4 0.01913322
Monocytes_4
10x_3288_t1_CGACTGCTGTTAGC-1 Patient5 Monocytes nonactive 4 0.03083191
Monocytes_4
10x_3288_t1_CGACTGCTTTAGGC-1 Patient5 Progenitors active 1 0.13241075
Progenitors_1
10x_3288_t1_CGACTGCTTTCCAT-1Patient5 Monocytes nonactive 15 0.05118572
Monocytes_15
10x_3288_t1_CGAGAACTAACGTC-1 Patient5 Monocytes nonactive 0 0.0531355
Monocytes_0
10x_3288_t1_CGAGAACTACCTCC-1 Patient5 Progenitors active 1 0.14995879

Progenitors_1
10x_3288_t1_CGAGAACTACTCTT-1 Patient5 Monocytes nonactive 4 0.01580432
Monocytes_4
10x_3288_t1_CGAGAACTATGTCG-1 Patient5 Monocytes nonactive 8 0.02973813
Monocytes_8
10x_3288_t1_CGAGAACTCTTATC-1 Patient5 Monocytes nonactive 0 0.04240378
Monocytes_0
10x_3288_t1_CGAGAACTGGAAAT-1 Patient5 Monocytes nonactive 4 0.02120982
Monocytes_4
10x_3288_t1_CGAGATTGAGCATC-1 Patient5 Monocytes nonactive 4 0.04876038
Monocytes_4
10x_3288_t1_CGAGATTGCCTTGC-1 Patient5 Progenitors active 1 0.17457675
Progenitors_1
10x_3288_t1_CGAGATTGGTTGCA-1 Patient5 Progenitors active 1 0.16067466
Progenitors_1
10x_3288_t1_CGAGATTGTCCTGC-1 Patient5 Monocytes nonactive 0 0.04520956
Monocytes_0
10x_3288_t1_CGAGATTGTCCTTA-1 Patient5 Monocytes nonactive 4 0.03341576
Monocytes_4
10x_3288_t1_CGAGATTGTTCATC-1 Patient5 Monocytes nonactive 4 0.04111978
Monocytes_4
10x_3288_t1_CGAGCCGAACCTAG-1 Patient5 Monocytes nonactive 4 0.01372773
Monocytes_4
10x_3288_t1_CGAGCCGAATCACG-1 Patient5 Monocytes nonactive 15 0.04991757
Monocytes_15
10x_3288_t1_CGAGCCGACCTCGT-1 Patient5 Monocytes nonactive 0 0.03909074
Monocytes_0
10x_3288_t1_CGAGCCGAGTTGAC-1 Patient5 Monocytes nonactive 4 0.02750301
Monocytes_4
10x_3288_t1_CGAGCCGATTGGTG-1 Patient5 Progenitors active 1 0.16600089
Progenitors_1
10x_3288_t1_CGAGCGTGACGGAG-1 Patient5 Monocytes nonactive 0 0.03760066
Monocytes_0

10x_3288_t1_CGAGCGTGTCGATG-1 Patient5 Progenitors nonactive 5 0.08106651 Progenitors_5
10x_3288_t1_CGAGCGTGTCGTTT-1 Patient5 Monocytes nonactive 0 0.02285841 Monocytes_0
10x_3288_t1_CGAGCGTGTCTTCA-1 Patient5 Monocytes nonactive 4 0.05700336 Monocytes_4
10x_3288_t1_CGAGGCACACGCAT-1 Patient5 Progenitors active 1 0.12554689 Progenitors_1
10x_3288_t1_CGAGGCACAGCCAT-1 Patient5 Progenitors active 1 0.13355209 Progenitors_1
10x_3288_t1_CGAGGCACCATCAG-1 Patient5 Progenitors active 1 0.14103418 Progenitors_1
10x_3288_t1_CGAGGCACTGGAAA-1 Patient5 Monocytes nonactive 4 0.07499524 Monocytes_4
10x_3288_t1_CGAGGCACTTTCTG-1 Patient5 Monocytes nonactive 0 0.04126244 Monocytes_0
10x_3288_t1_CGAGGCTGAGGTTC-1 Patient5 Progenitors active 1 0.16936149 Progenitors_1
10x_3288_t1_CGAGGCTGCCTCAC-1 Patient5 Monocytes nonactive 0 0.05151861 Monocytes_0
10x_3288_t1_CGAGGCTGCTATGG-1 Patient5 Monocytes nonactive 8 0.03961385 Monocytes_8
10x_3288_t1_CGAGGCTGGCCTTC-1 Patient5 Monocytes nonactive 8 0.0447023 Monocytes_8
10x_3288_t1_CGAGGCTGGGATTC-1 Patient5 Monocytes nonactive 8 0.03525458 Monocytes_8
10x_3288_t1_CGAGGCTGTTCGGA-1 Patient5 Progenitors active 1 0.16222814 Progenitors_1
10x_3288_t1_CGAGGGCTACGTGT-1 Patient5 Monocytes nonactive 4 0.03780673 Monocytes_4
10x_3288_t1_CGAGGGCTGCCTTC-1 Patient5 Monocytes nonactive 4 0.01448862 Monocytes_4
10x_3288_t1_CGAGTATGAAACGA-1 Patient5 Monocytes nonactive 4 0.03590451

Monocytes_4

10x_3288_t1_CGAGTATGACACTG-1 Patient5 Progenitors active 1 0.14349122 Progenitors_1

10x_3288_t1_CGAGTATGACCTGA-1 Patient5 Dendritic cells nonactive 10 0.08810475 Dendritic cells_10

10x_3288_t1_CGAGTATGCCGATA-1 Patient5 Monocytes nonactive 0 0.09901084 Monocytes_0

10x_3288_t1_CGAGTATGGCGGAA-1 Patient5 Monocytes nonactive 0 0.09468328 Monocytes_0

10x_3288_t1_CGAGTATGTCCAAG-1 Patient5 Monocytes nonactive 0 0.03785429 Monocytes_0

10x_3288_t1_CGATACGACATGAC-1 Patient5 Monocytes nonactive 4 0.06261493 Monocytes_4

10x_3288_t1_CGATACGACCACAA-1 Patient5 Monocytes nonactive 4 0.05896899 Monocytes_4

10x_3288_t1_CGATACGATAAGGA-1 Patient5 Monocytes nonactive 4 0.04303785 Monocytes_4

10x_3288_t1_CGATACGATGCTTT-1 Patient5 Progenitors active 5 0.15089405 Progenitors_5

10x_3288_t1_CGATACGATTCCAT-1 Patient5 Progenitors nonactive 1 0.11373724 Progenitors_1

10x_3288_t1_CGATAGACAATCGC-1 Patient5 Monocytes nonactive 0 0.02536301 Monocytes_0

10x_3288_t1_CGATAGACCCCTCA-1 Patient5 Monocytes nonactive 4 0.06970072 Monocytes_4

10x_3288_t1_CGATAGACCCTGAA-1 Patient5 Progenitors active 1 0.18591085 Progenitors_1

10x_3288_t1_CGATAGACGTCCTC-1 Patient5 Monocytes nonactive 4 0.06665716 Monocytes_4

10x_3288_t1_CGATAGACTGGATC-1 Patient5 Monocytes nonactive 8 0.02192315 Monocytes_8

10x_3288_t1_CGATCAGACAGTTG-1 Patient5 Progenitors active 1 0.13591402 Progenitors_1

10x_3288_t1_CGATCAGAGTCGTA-1 Patient5 Progenitors active 1 0.1766692 Progenitors_1
10x_3288_t1_CGATCCACACCTCC-1 Patient5 Progenitors active 1 0.14177922 Progenitors_1
10x_3288_t1_CGATCCACGGTAAA-1 Patient5 Progenitors active 1 0.15972354 Progenitors_1
10x_3288_t1_CGATCCACTGACTG-1 Patient5 Progenitors active 1 0.14897597 Progenitors_1
10x_3288_t1_CGCAAATGAACCGT-1 Patient5 Monocytes nonactive 4 0.06906664 Monocytes_4
10x_3288_t1_CGCAAATGAAGCCT-1 Patient5 Monocytes nonactive 4 0.09870966 Monocytes_4
10x_3288_t1_CGCAAATGCAACTG-1 Patient5 Monocytes nonactive 4 0.03525458 Monocytes_4
10x_3288_t1_CGCAAATGCCATGA-1 Patient5 Progenitors active 1 0.17866654 Progenitors_1
10x_3288_t1_CGCAAATGCCTAAG-1 Patient5 Monocytes nonactive 0 0.01756388 Monocytes_0
10x_3288_t1_CGCAAATGTCCCGT-1 Patient5 Monocytes nonactive 0 0.07876799 Monocytes_0
10x_3288_t1_CGCAACCTTAGACC-1 Patient5 Monocytes nonactive 4 0.04522541 Monocytes_4
10x_3288_t1_CGCACGGACAACTG-1 Patient5 Monocytes nonactive 4 0.01583603 Monocytes_4
10x_3288_t1_CGCACGGACGAGTT-1 Patient5 Monocytes nonactive 4 0.02407901 Monocytes_4
10x_3288_t1_CGCACGGAGCTACA-1 Patient5 Monocytes nonactive 0 0.04923594 Monocytes_0
10x_3288_t1_CGCACGGATAGCCA-1 Patient5 Monocytes nonactive 4 0.02517279 Monocytes_4
10x_3288_t1_CGCACTACCGACAT-1 Patient5 Progenitors active 1 0.14661404 Progenitors_1
10x_3288_t1_CGCACTACCGCTAA-1 Patient5 Progenitors active 1 0.1644157

Progenitors_1

10x_3288_t1_CGCACTACTACTCT-1 Patient5 Monocytes nonactive 8 0.02445945 Monocytes_8

10x_3288_t1_CGCACTTGACCTAG-1 Patient5 Monocytes nonactive 0 0.03059413 Monocytes_0

10x_3288_t1_CGCACTTGCAACCA-1 Patient5 Monocytes nonactive 8 0.03078435 Monocytes_8

10x_3288_t1_CGCACTTGGAAAGT-1 Patient5 Progenitors active 1 0.16243421 Progenitors_1

10x_3288_t1_CGCAGGTGGGCGAA-1 Patient5 Progenitors nonactive 1 0.09867795 Progenitors_1

10x_3288_t1_CGCATAGAATTTCC-1 Patient5 Progenitors active 1 0.12486526 Progenitors_1

10x_3288_t1_CGCATAGATGGTCA-1 Patient5 Monocytes nonactive 0 0 Monocytes_0

10x_3288_t1_CGCATAGATTAGGC-1 Patient5 Monocytes nonactive 4 0.02184389 Monocytes_4

10x_3288_t1_CGCCATACAAAGTG-1 Patient5 Monocytes nonactive 4 0.03806036 Monocytes_4

10x_3288_t1_CGCCATACCCGTAA-1 Patient5 Monocytes nonactive 4 0.05855684 Monocytes_4

10x_3288_t1_CGCCATACTTGAGC-1 Patient5 Monocytes nonactive 4 0.04454378 Monocytes_4

10x_3288_t1_CGCCATTGAGTTCG-1 Patient5 Monocytes nonactive 4 0.0202587 Monocytes_4

10x_3288_t1_CGCCATTGCACTAG-1 Patient5 Progenitors active 1 0.14578974 Progenitors_1

10x_3288_t1_CGCCATTGGGGTGA-1 Patient5 Monocytes nonactive 0 0.05342084 Monocytes_0

10x_3288_t1_CGCCATTGTTGGTG-1 Patient5 Monocytes nonactive 15 0.03573014 Monocytes_15

10x_3288_t1_CGCCGAGATTTGGG-1 Patient5 Monocytes nonactive 4 0.06063344 Monocytes_4

10x_3288_t1_CGCGATCTAGCTCA-1 Patient5 Progenitors active 1 0.11828673
Progenitors_1
10x_3288_t1_CGCGATCTCACTCC-1 Patient5 Monocytes nonactive 4 0.04654112
Monocytes_4
10x_3288_t1_CGCGATCTGCTGTA-1 Patient5 Progenitors active 1 0.13978188
Progenitors_1
10x_3288_t1_CGCGATCTTACTCT-1 Patient5 Progenitors active 1 0.14694693
Progenitors_1
10x_3288_t1_CGCGATCTTAGTCG-1 Patient5 Monocytes nonactive 4 0.06405745
Monocytes_4
10x_3288_t1_CGCGGATGCAGTTG-1 Patient5 Progenitors active 1 0.12256674
Progenitors_1
10x_3288_t1_CGCTAAGACATTTC-1 Patient5 Progenitors active 1 0.14417285
Progenitors_1
10x_3288_t1_CGCTAAGACCGATA-1 Patient5 Monocytes nonactive 4 0.09106905
Monocytes_4
10x_3288_t1_CGCTAAGATCGTTT-1Patient5 Monocytes nonactive 8 0.07049331
Monocytes_8
10x_3288_t1_CGCTACACACGTTG-1 Patient5 Progenitors nonactive 1 0.10929871
Progenitors_1
10x_3288_t1_CGCTACACCATGGT-1 Patient5 Progenitors active 1 0.17381586
Progenitors_1
10x_3288_t1_CGCTACACCGTAGT-1 Patient5 Monocytes nonactive 4 0.04178556
Monocytes_4
10x_3288_t1_CGCTACACGGTTCA-1 Patient5 Progenitors active 1 0.15355716
Progenitors_1
10x_3288_t1_CGCTACACTACGCA-1 Patient5 Monocytes nonactive 0 0.03187813
Monocytes_0
10x_3288_t1_CGCTACTGTTTGGG-1 Patient5 Monocytes nonactive 0 0.0159787
Monocytes_0
10x_3288_t1_CGCTCATGAAGCCT-1 Patient5 Progenitors active 1 0.13119016
Progenitors_1
10x_3288_t1_CGCTCATGACCCAA-1 Patient5 Monocytes nonactive 15 0.01291928

Monocytes_15
10x_3288_t1_CGCTCATGCATCAG-1 Patient5 Monocytes nonactive 4 0
Monocytes_4
10x_3288_t1_CGCTCATGTGACAC-1 Patient5 Monocytes nonactive 4 0.01903811
Monocytes_4
10x_3288_t1_CGCTCATGTGGGAG-1 Patient5 Progenitors active 1 0.13523239
Progenitors_1
10x_3288_t1_CGCTCATGTTCTAC-1 Patient5 Progenitors active 1 0.13889417
Progenitors_1
10x_3288_t1_CGGAATTGCAGTTG-1 Patient5 Monocytes nonactive 0 0.02965887
Monocytes_0
10x_3288_t1_CGGAATTGCCGATA-1 Patient5 Progenitors nonactive 5 0.09408091
Progenitors_5
10x_3288_t1_CGGAATTGGGAACG-1 Patient5 Monocytes nonactive 13 0.0813994
Monocytes_13
10x_3288_t1_CGGAATTGGGGATG-1 Patient5 Monocytes nonactive 4 0.04352926
Monocytes_4
10x_3288_t1_CGGAATTGTCCCAC-1 Patient5 Progenitors active 6 0.12064866
Progenitors_6
10x_3288_t1_CGGAATTGTTCACT-1 Patient5 Progenitors active 6 0.13683343
Progenitors_6
10x_3288_t1_CGGACCGAACGACT-1 Patient5 Monocytes nonactive 8 0.03641177
Monocytes_8
10x_3288_t1_CGGACCGACTCTCG-1 Patient5 Dendritic cells nonactive 10 0.07413924
Dendritic cells_10
10x_3288_t1_CGGACCGACTTACT-1 Patient5 Monocytes nonactive 0 0.04597045
Monocytes_0
10x_3288_t1_CGGACCGAGACGAG-1 Patient5 Progenitors active 1 0.13463002
Progenitors_1
10x_3288_t1_CGGACCGAGTACAC-1 Patient5 Monocytes nonactive 0 0.03393888
Monocytes_0
10x_3288_t1_CGGACTCTCAGTTG-1 Patient5 Monocytes nonactive 15 0.05830321
Monocytes_15

10x_3288_t1_CGGACTCTGACTAC-1 Patient5 Progenitors active 1 0.14753345 Progenitors_1
10x_3288_t1_CGGACTCTTCCTTA-1 Patient5 Monocytes nonactive 8 0.0455583 Monocytes_8
10x_3288_t1_CGGACTCTTGCATG-1 Patient5 Progenitors active 1 0.18724241 Progenitors_1
10x_3288_t1_CGGACTCTTGTCTT-1Patient5 Monocytes nonactive 4 0.05884218 Monocytes_4
10x_3288_t1_CGGAGGCTATAAGG-1 Patient5 Progenitors active 1 0.12459578 Progenitors_1
10x_3288_t1_CGGAGGCTCGATAC-1 Patient5 Monocytes nonactive 4 0.00680046 Monocytes_4
10x_3288_t1_CGGAGGCTTCGATG-1 Patient5 Progenitors nonactive 1 0.11459324 Progenitors_1
10x_3288_t1_CGGATAACACACAC-1 Patient5 Monocytes nonactive 4 0.0581764 Monocytes_4
10x_3288_t1_CGGATAACCGGTAT-1Patient5 Monocytes nonactive 4 0.0750428 Monocytes_4
10x_3288_t1_CGGATAACGGTATC-1 Patient5 Monocytes nonactive 0 0.01878448 Monocytes_0
10x_3288_t1_CGGATATGAACAGA-1 Patient5 Progenitors nonactive 1 0.11563946 Progenitors_1
10x_3288_t1_CGGATATGTTGTCT-1 Patient5 Monocytes nonactive 0 0.03341576 Monocytes_0
10x_3288_t1_CGGCATCTACGCTA-1 Patient5 Progenitors active 1 0.1471213 Progenitors_1
10x_3288_t1_CGGCATCTCCTCAC-1 Patient5 Progenitors active 1 0.16154651 Progenitors_1
10x_3288_t1_CGGCATCTTCGCTC-1 Patient5 Monocytes nonactive 4 0.04131 Monocytes_4
10x_3288_t1_CGGCCAGAACTGTG-1 Patient5 Monocytes nonactive 0 0.03222687 Monocytes_0
10x_3288_t1_CGGCCAGAATGGTC-1 Patient5 Monocytes nonactive 4 0.03091117

Monocytes_4
10x_3288_t1_CGGCCAGAGCAGTT-1 Patient5 Monocytes nonactive 4 0.0273762
Monocytes_4
10x_3288_t1_CGGCCAGAGTACGT-1 Patient5 Progenitors active 1 0.15593494
Progenitors_1
10x_3288_t1_CGGCGAACCCGAAT-1 Patient5 Monocytes nonactive 8 0.04936275
Monocytes_8
10x_3288_t1_CGGCGAACGAGCAG-1 Patient5 Monocytes nonactive 0 0.05373787
Monocytes_0
10x_3288_t1_CGGCGAACGAGGAC-1 Patient5 Progenitors nonactive 1 0.09864625
Progenitors_1
10x_3288_t1_CGGCGAACGCTGAT-1 Patient5 Monocytes nonactive 4 0.03720436
Monocytes_4
10x_3288_t1_CGGCGATGACGCAT-1 Patient5 Dendritic cells nonactive 10 0.08536237
Dendritic cells_10
10x_3288_t1_CGGCGATGACGTGT-1 Patient5 Monocytes nonactive 4 0.02555323
Monocytes_4
10x_3288_t1_CGGCGATGACTCTT-1 Patient5 Monocytes nonactive 0 0.02504597
Monocytes_0
10x_3288_t1_CGGCGATGCACTTT-1 Patient5 Monocytes nonactive 0 0.03855177
Monocytes_0
10x_3288_t1_CGGCGATGCTCTAT-1 Patient5 Progenitors active 1 0.12947816
Progenitors_1
10x_3288_t1_CGGCGATGCTCTTA-1 Patient5 Progenitors active 1 0.1939319
Progenitors_1
10x_3288_t1_CGGCGATGGACGAG-1 Patient5 Monocytes nonactive 15 0.05096379
Monocytes_15
10x_3288_t1_CGGCGATGGGTACT-1 Patient5 Monocytes nonactive 0 0.03452539
Monocytes_0
10x_3288_t1_CGGCGATGTCTAGG-1 Patient5 Monocytes nonactive 4 0.05876292
Monocytes_4
10x_3288_t1_CGGCGATGTGTGAC-1 Patient5 Progenitors nonactive 1 0.10639782
Progenitors_1

10x_3288_t1_CGGGACTGACACCA-1 Patient5 Monocytes nonactive 4 0.01800774 Monocytes_4
10x_3288_t1_CGGGACTGCCTCAC-1 Patient5 Progenitors active 1 0.15893095 Progenitors_1
10x_3288_t1_CGGGACTGCTTCCG-1 Patient5 Progenitors active 1 0.13851373 Progenitors_1
10x_3288_t1_CGGGACTGCTTGAG-1 Patient5 Monocytes nonactive 8 0.03022954 Monocytes_8
10x_3288_t1_CGGGCATGAAAACG-1 Patient5 Progenitors nonactive 1 0.10761841 Progenitors_1
10x_3288_t1_CGGGCATGACCTAG-1 Patient5 Monocytes nonactive 4 0.03774333 Monocytes_4
10x_3288_t1_CGGGCATGAGATGA-1 Patient5 Monocytes nonactive 4 0.06163211 Monocytes_4
10x_3288_t1_CGGGCATGGTACGT-1 Patient5 Monocytes nonactive 8 0.06711686 Monocytes_8
10x_3288_t1_CGGGCATGTTAGGC-1 Patient5 Monocytes nonactive 8 0.04268911 Monocytes_8
10x_3288_t1_CGGTAAACACACTG-1 Patient5 Progenitors nonactive 1 0.10674656 Progenitors_1
10x_3288_t1_CGGTACCTGAGAGC-1 Patient5 Monocytes nonactive 4 0.03826644 Monocytes_4
10x_3288_t1_CGGTACCTGGAGCA-1 Patient5 Monocytes nonactive 0 0.03576184 Monocytes_0
10x_3288_t1_CGGTCACTCCATAG-1 Patient5 Progenitors nonactive 1 0.10994864 Progenitors_1
10x_3288_t1_CGGTCACTCCCGTT-1 Patient5 Progenitors active 1 0.14734322 Progenitors_1
10x_3288_t1_CGGTCACTCGTGAT-1 Patient5 Monocytes nonactive 0 0.06060174 Monocytes_0
10x_3288_t1_CGGTCACTGGAGCA-1 Patient5 Progenitors active 1 0.15122694 Progenitors_1
10x_3288_t1_CGGTCACTTAGCCA-1 Patient5 Progenitors active 1 0.13310824

Progenitors_1
10x_3288_t1_CGTAACGACTTCCG-1 Patient5 Monocytes nonactive 0 0.04727031
Monocytes_0
10x_3288_t1_CGTAACGAGAAACA-1 Patient5 Monocytes nonactive 4 0.04085029
Monocytes_4
10x_3288_t1_CGTAACGATGGCAT-1 Patient5 Monocytes nonactive 0 0.05648025
Monocytes_0
10x_3288_t1_CGTAACGATGTCCC-1 Patient5 Monocytes nonactive 0 0.04272082
Monocytes_0
10x_3288_t1_CGTAACGATTGTGG-1 Patient5 Monocytes nonactive 0 0.03723607
Monocytes_0
10x_3288_t1_CGTACAGATAACCG-1 Patient5 Progenitors active 1 0.18009321
Progenitors_1
10x_3288_t1_CGTACAGATGCAAC-1 Patient5 Progenitors active 1 0.13087312
Progenitors_1
10x_3288_t1_CGTACCACAAGAAC-1 Patient5 Progenitors nonactive 1 0.09024475
Progenitors_1
10x_3288_t1_CGTACCACTAAGGA-1 Patient5 Monocytes nonactive 0 0.02715427
Monocytes_0
10x_3288_t1_CGTACCACTTTGGG-1 Patient5 Monocytes nonactive 4 0.03303532
Monocytes_4
10x_3288_t1_CGTACCTGCTCAAG-1 Patient5 Monocytes nonactive 4 0.07076279
Monocytes_4
10x_3288_t1_CGTACCTGGTTGCA-1 Patient5 Monocytes nonactive 0 0.07114324
Monocytes_0
10x_3288_t1_CGTAGCCTCACCAA-1 Patient5 Monocytes nonactive 0 0.05006024
Monocytes_0
10x_3288_t1_CGTCAAGAACACAC-1 Patient5 Monocytes nonactive 0 0.05579862
Monocytes_0
10x_3288_t1_CGTCAAGAATGGTC-1 Patient5 Progenitors active 1 0.15853465
Progenitors_1
10x_3288_t1_CGTCAAGACATCAG-1 Patient5 Monocytes nonactive 16 0.04998098
Monocytes_16

10x_3288_t1_CGTCAAGATCTTAC-1 Patient5 Progenitors active 1 0.12941475 Progenitors_1
10x_3288_t1_CGTCCAACTTCCCG-1 Patient5 Progenitors nonactive 1 0.10628686 Progenitors_1
10x_3288_t1_CGTCCATGACACCA-1 Patient5 Monocytes nonactive 8 0.04944201 Monocytes_8
10x_3288_t1_CGTCCATGACCTCC-1 Patient5 Monocytes nonactive 0 0.05239046 Monocytes_0
10x_3288_t1_CGTCGACTCCCGTT-1 Patient5 Monocytes nonactive 0 0.04126244 Monocytes_0
10x_3288_t1_CGTGAAACAGTCTG-1 Patient5 Monocytes nonactive 4 0.02688479 Monocytes_4
10x_3288_t1_CGTGAAACCGAGAG-1 Patient5 Monocytes nonactive 0 0.05890559 Monocytes_0
10x_3288_t1_CGTGAAACCTTATC-1 Patient5 Monocytes nonactive 8 0.03325724 Monocytes_8
10x_3288_t1_CGTGAAACTCCAGA-1 Patient5 Monocytes nonactive 4 0.05077357 Monocytes_4
10x_3288_t1_CGTGAAACTTTACC-1 Patient5 Progenitors active 1 0.17372075 Progenitors_1
10x_3288_t1_CGTGAATGAGTAGA-1 Patient5 Monocytes nonactive 0 0.05717773 Monocytes_0
10x_3288_t1_CGTGAATGGAACTC-1 Patient5 Monocytes nonactive 4 0.03728362 Monocytes_4
10x_3288_t1_CGTGATGAAGAACA-1 Patient5 Progenitors nonactive 1 0.0944455 Progenitors_1
10x_3288_t1_CGTGATGAGCAAGG-1 Patient5 Monocytes nonactive 0 0.03826644 Monocytes_0
10x_3288_t1_CGTGATGATCAGTG-1 Patient5 Monocytes nonactive 0 0.04619238 Monocytes_0
10x_3288_t1_CGTGATGATGTGAC-1 Patient5 Monocytes nonactive 4 0.07071524 Monocytes_4
10x_3288_t1_CGTGCACTTTTGTC-1 Patient5 Dendritic cells nonactive 10 0.11575043

Dendritic cells_10

10x_3288_t1_CGTGTAGAGGTAAA-1 Patient5 Monocytes nonactive 4 0.03183058 Monocytes_4

10x_3288_t1_CGTGTAGATCGTAG-1 Patient5 Progenitors active 1 0.13069875 Progenitors_1

10x_3288_t1_CGTTAACTCCCTAC-1 Patient5 Monocytes nonactive 0 0.06548412 Monocytes_0

10x_3288_t1_CGTTAACTCGGTAT-1 Patient5 Progenitors active 1 0.13090483 Progenitors_1

10x_3288_t1_CGTTAACTGCTCCT-1 Patient5 Monocytes nonactive 4 0.07789614 Monocytes_4

10x_3288_t1_CGTTAACTTCTAGG-1 Patient5 Monocytes nonactive 4 0.05237461 Monocytes_4

10x_3288_t1_CGTTAACTTTGGTG-1 Patient5 Progenitors active 1 0.15113182 Progenitors_1

10x_3288_t1_CGTTAACTTTTGGG-1 Patient5 Monocytes nonactive 13 0.04449623 Monocytes_13

10x_3288_t1_CGTTAGGATTTCGT-1 Patient5 Monocytes nonactive 0 0.05992011 Monocytes_0

10x_3288_t1_CGTTATACCCGTAA-1 Patient5 Monocytes nonactive 4 0.03812377 Monocytes_4

10x_3288_t1_CGTTATACCGTAAC-1 Patient5 Monocytes nonactive 0 0.07377465 Monocytes_0

10x_3288_t1_CGTTATACCTTCGC-1 Patient5 Progenitors active 1 0.14181092 Progenitors_1

10x_3288_t1_CGTTTAACAACTGC-1 Patient5 Progenitors active 1 0.12591148 Progenitors_1

10x_3288_t1_CGTTTAACACGCAT-1 Patient5 Monocytes nonactive 0 0.02650434 Monocytes_0

10x_3288_t1_CGTTTAACGGCATT-1 Patient5 Monocytes nonactive 4 0.04251474 Monocytes_4

10x_3288_t1_CTAAACCTCATTCT-1 Patient5 Monocytes nonactive 0 0.09121172 Monocytes_0

10x_3288_t1_CTAAACCTCCTTTA-1Patient5 Monocytes nonactive 0 0.04961638 Monocytes_0
10x_3288_t1_CTAAACCTGATAGA-1 Patient5 Monocytes nonactive 4 0.07585125 Monocytes_4
10x_3288_t1_CTAACACTAGCGGA-1 Patient5 Monocytes nonactive 0 0.06727538 Monocytes_0
10x_3288_t1_CTAACACTCTCTTA-1Patient5 Monocytes nonactive 4 0.05770084 Monocytes_4
10x_3288_t1_CTAACACTTGCAAC-1 Patient5 Progenitors active 1 0.18269292 Progenitors_1
10x_3288_t1_CTAACGGAAACCGT-1 Patient5 Progenitors active 1 0.15901021 Progenitors_1
10x_3288_t1_CTAACGGAAACGAA-1 Patient5 Progenitors active 1 0.13548602 Progenitors_1
10x_3288_t1_CTAACGGAACGGAG-1 Patient5 Monocytes nonactive 4 0.06678397 Monocytes_4
10x_3288_t1_CTAACGGAAGAGTA-1 Patient5 Monocytes nonactive 8 0.10555767 Monocytes_8
10x_3288_t1_CTAACGGACACTCC-1 Patient5 Monocytes nonactive 4 0.03777503 Monocytes_4
10x_3288_t1_CTAACGGAGGTAGG-1 Patient5 Progenitors active 1 0.16550948 Progenitors_1
10x_3288_t1_CTAACGGATAGTCG-1 Patient5 Monocytes nonactive 0 0.00673705 Monocytes_0
10x_3288_t1_CTAACGGATGGAAA-1 Patient5 Progenitors active 1 0.17127956 Progenitors_1
10x_3288_t1_CTAACTACGGACTT-1 Patient5 Monocytes nonactive 8 0.06396234 Monocytes_8
10x_3288_t1_CTAACTACTCGTAG-1 Patient5 Monocytes nonactive 0 0.0682899 Monocytes_0
10x_3288_t1_CTAAGGACGCGAAG-1 Patient5 Progenitors active 1 0.16796652 Progenitors_1
10x_3288_t1_CTAAGGACGTTGAC-1 Patient5 Monocytes nonactive 4 0.0379494

Monocytes_4

10x_3288_t1_CTAAGGTGCCTCGT-1 Patient5 Progenitors active 1 0.15926384 Progenitors_1

10x_3288_t1_CTAAGGTGCTCTAT-1 Patient5 Progenitors active 1 0.1538742 Progenitors_1

10x_3288_t1_CTAAGGTGTGAGCT-1 Patient5 Monocytes nonactive 0 0.08816816 Monocytes_0

10x_3288_t1_CTAAGGTGTTGGTG-1 Patient5 Monocytes nonactive 0 0.08013125 Monocytes_0

10x_3288_t1_CTAAGGTGTTTGTC-1 Patient5 Monocytes nonactive 4 0.02124152 Monocytes_4

10x_3288_t1_CTAATAGAAGTGTC-1 Patient5 Monocytes nonactive 8 0.06630841 Monocytes_8

10x_3288_t1_CTAATAGACCCAAA-1 Patient5 Monocytes nonactive 4 0.02877116 Monocytes_4

10x_3288_t1_CTAATAGATCTTAC-1 Patient5 Monocytes nonactive 4 0.04787268 Monocytes_4

10x_3288_t1_CTAATGCTGAGGTG-1 Patient5 Monocytes nonactive 0 0.040882 Monocytes_0

10x_3288_t1_CTAATGCTGTACGT-1 Patient5 Progenitors active 1 0.14185847 Progenitors_1

10x_3288_t1_CTAATGCTTAGACC-1 Patient5 Monocytes nonactive 0 0.05746307 Monocytes_0

10x_3288_t1_CTACAACTAGATGA-1 Patient5 Monocytes nonactive 4 0.06098218 Monocytes_4

10x_3288_t1_CTACAACTCAACCA-1 Patient5 Progenitors nonactive 1 0.10745989 Progenitors_1

10x_3288_t1_CTACAACTGAATAG-1 Patient5 Progenitors nonactive 1 0.09630017 Progenitors_1

10x_3288_t1_CTACAACTTCCCAC-1 Patient5 Monocytes nonactive 0 0.03836155 Monocytes_0

10x_3288_t1_CTACCTCTCGCAAT-1 Patient5 Monocytes nonactive 0 0.01380699 Monocytes_0

10x_3288_t1_CTACGCACACGTTG-1 Patient5 Monocytes nonactive 0 0.05156617 Monocytes_0
10x_3288_t1_CTACGGCTCCTTCG-1 Patient5 Progenitors active 1 0.12250333 Progenitors_1
10x_3288_t1_CTACGGCTTTCTAC-1Patient5 Monocytes nonactive 8 0.05446706 Monocytes_8
10x_3288_t1_CTACTATGCCTATT-1 Patient5 Monocytes nonactive 8 0.06722782 Monocytes_8
10x_3288_t1_CTACTATGGCTCCT-1 Patient5 Monocytes nonactive 0 0.02116226 Monocytes_0
10x_3288_t1_CTACTATGGGTAGG-1 Patient5 Progenitors active 1 0.1189208 Progenitors_1
10x_3288_t1_CTACTATGTCGCAA-1 Patient5 Progenitors active 1 0.16370237 Progenitors_1
10x_3288_t1_CTACTCCTTCGCCT-1 Patient5 Progenitors nonactive 1 0.10427367 Progenitors_1
10x_3288_t1_CTACTCCTTGCCAA-1 Patient5 Monocytes nonactive 13 0.03969311 Monocytes_13
10x_3288_t1_CTACTCCTTGTTCT-1 Patient5 Monocytes nonactive 0 0.02452286 Monocytes_0
10x_3288_t1_CTACTCCTTTTCGT-1 Patient5 Progenitors active 1 0.14991123 Progenitors_1
10x_3288_t1_CTAGAGACCGTGTA-1 Patient5 Progenitors active 1 0.12881238 Progenitors_1
10x_3288_t1_CTAGAGACCTCTTA-1 Patient5 Monocytes nonactive 13 0.04262571 Monocytes_13
10x_3288_t1_CTAGAGACGTATCG-1 Patient5 Progenitors nonactive 1 0.11322998 Progenitors_1
10x_3288_t1_CTAGATCTGCATCA-1 Patient5 Progenitors active 1 0.13642128 Progenitors_1
10x_3288_t1_CTAGATCTTCTCTA-1 Patient5 Monocytes nonactive 4 0.05272335 Monocytes_4
10x_3288_t1_CTAGGATGAAGAAC-1 Patient5 Monocytes nonactive 4 0.03731533

Monocytes_4

10x_3288_t1_CTAGGATGCCCGTT-1 Patient5 Monocytes nonactive 4 0.03094287 Monocytes_4

10x_3288_t1_CTAGGATGTCTAGG-1 Patient5 Monocytes nonactive 0 0.05343669 Monocytes_0

10x_3288_t1_CTAGGCCTGGAGCA-1 Patient5 Monocytes nonactive 4 0.06632427 Monocytes_4

10x_3288_t1_CTAGGCCTGGCAAG-1 Patient5 Progenitors active 1 0.13724558 Progenitors_1

10x_3288_t1_CTAGGCCTTCGACA-1 Patient5 Monocytes nonactive 0 0.05445121 Monocytes_0

10x_3288_t1_CTAGGTGAGCGTTA-1 Patient5 Progenitors active 1 0.15049775 Progenitors_1

10x_3288_t1_CTAGTTACATGACC-1 Patient5 Monocytes nonactive 4 0.05427684 Monocytes_4

10x_3288_t1_CTAGTTACGGTCAT-1 Patient5 Monocytes nonactive 8 0.02904064 Monocytes_8

10x_3288_t1_CTAGTTTGCTTCCG-1 Patient5 Monocytes nonactive 0 0.0295479 Monocytes_0

10x_3288_t1_CTAGTTTGGCTATG-1 Patient5 Monocytes nonactive 4 0.05113816 Monocytes_4

10x_3288_t1_CTAGTTTGGTTGTG-1 Patient5 Monocytes nonactive 4 0.06315389 Monocytes_4

10x_3288_t1_CTAGTTTGTGTCGA-1 Patient5 Monocytes nonactive 4 0.04517786 Monocytes_4

10x_3288_t1_CTAGTTTGTGTGAC-1 Patient5 Monocytes nonactive 4 0.03051487 Monocytes_4

10x_3288_t1_CTAGTTTGTGTTTC-1 Patient5 Monocytes nonactive 0 0.0324805 Monocytes_0

10x_3288_t1_CTATAAGAATTCGG-1 Patient5 Progenitors active 1 0.13856128 Progenitors_1

10x_3288_t1_CTATAAGACCTAAG-1 Patient5 Monocytes nonactive 0 0.05950796 Monocytes_0

10x_3288_t1_CTATAAGACCTTGC-1 Patient5 Monocytes nonactive 4 0.02732864
Monocytes_4
10x_3288_t1_CTATAAGAGCTGTA-1 Patient5 Monocytes nonactive 0 0.05746307
Monocytes_0
10x_3288_t1_CTATAAGATCAAGC-1 Patient5 Monocytes nonactive 16 0.05304039
Monocytes_16
10x_3288_t1_CTATACTGAAGCCT-1 Patient5 Monocytes nonactive 0 0.04332319
Monocytes_0
10x_3288_t1_CTATACTGGGGCAA-1 Patient5 Monocytes nonactive 4 0.018943
Monocytes_4
10x_3288_t1_CTATACTGGGTTAC-1 Patient5 Progenitors active 1 0.17552787
Progenitors_1
10x_3288_t1_CTATACTGTTTGCT-1 Patient5 Progenitors active 1 0.20765963
Progenitors_1
10x_3288_t1_CTATAGCTACGTAC-1 Patient5 Monocytes nonactive 4 0.07451969
Monocytes_4
10x_3288_t1_CTATAGCTAGAGAT-1 Patient5 Monocytes nonactive 0 0.05403906
Monocytes_0
10x_3288_t1_CTATAGCTGCTAAC-1 Patient5 Progenitors nonactive 1 0.10925116
Progenitors_1
10x_3288_t1_CTATAGCTTCCAGA-1 Patient5 Monocytes nonactive 4 0.05906411
Monocytes_4
10x_3288_t1_CTATCAACGTGTCA-1 Patient5 Monocytes nonactive 4 0.02127322
Monocytes_4
10x_3288_t1_CTATCAACTCCCGT-1 Patient5 Monocytes nonactive 0 0.03381206
Monocytes_0
10x_3288_t1_CTATCAACTTCGCC-1 Patient5 Progenitors nonactive 1 0.10853782
Progenitors_1
10x_3288_t1_CTATCAACTTCGTT-1 Patient5 Monocytes nonactive 0 0.0316245
Monocytes_0
10x_3288_t1_CTATCATGAGAAGT-1 Patient5 Progenitors active 6 0.1602308
Progenitors_6
10x_3288_t1_CTATCATGCTTTAC-1 Patient5 Monocytes nonactive 0 0.05725699

Monocytes_0
10x_3288_t1_CTATCATGGACACT-1 Patient5 Monocytes nonactive 4 0.04570097
Monocytes_4
10x_3288_t1_CTATCATGGAGGGT-1 Patient5 Progenitors active 1 0.13886247
Progenitors_1
10x_3288_t1_CTATCCCTATGCCA-1 Patient5 Progenitors active 1 0.15292309
Progenitors_1
10x_3288_t1_CTATCCCTCACACA-1 Patient5 Monocytes nonactive 15 0.0455583
Monocytes_15
10x_3288_t1_CTATCCCTCGTGTA-1 Patient5 Monocytes nonactive 4 0.05242217
Monocytes_4
10x_3288_t1_CTATCCCTGCGGAA-1 Patient5 Monocytes nonactive 0 0.06519878
Monocytes_0
10x_3288_t1_CTATGACTAACCGT-1 Patient5 Progenitors active 1 0.15490457
Progenitors_1
10x_3288_t1_CTATGACTATGGTC-1 Patient5 Monocytes nonactive 4 0.04950542
Monocytes_4
10x_3288_t1_CTATGACTCATGAC-1 Patient5 Monocytes nonactive 0 0.03758481
Monocytes_0
10x_3288_t1_CTATGACTCCAGTA-1 Patient5 Monocytes nonactive 0 0.02212922
Monocytes_0
10x_3288_t1_CTATGTACCATTGG-1 Patient5 Progenitors active 1 0.2074377
Progenitors_1
10x_3288_t1_CTATGTTGCGTTGA-1 Patient5 Progenitors active 1 0.16539852
Progenitors_1
10x_3288_t1_CTATGTTGGCAAGG-1 Patient5 Progenitors active 1 0.15251094
Progenitors_1
10x_3288_t1_CTATGTTGGGAGTG-1 Patient5 Progenitors active 1 0.14615433
Progenitors_1
10x_3288_t1_CTATGTTGTGAACC-1 Patient5 Monocytes nonactive 8 0.03828229
Monocytes_8
10x_3288_t1_CTATGTTGTTCTAC-1 Patient5 Progenitors active 1 0.16929808
Progenitors_1

10x_3288_t1_CTATTGACTGGTAC-1Patient5 Monocytes nonactive 0 0.09366876
Monocytes_0
10x_3288_t1_CTATTGTGCCTACC-1 Patient5 Progenitors active 1 0.14383996
Progenitors_1
10x_3288_t1_CTATTGTGTCTCTA-1 Patient5 Progenitors nonactive 6 0.11659058
Progenitors_6
10x_3288_t1_CTCAATTGACTGTG-1 Patient5 Monocytes nonactive 8 0.02426923
Monocytes_8
10x_3288_t1_CTCAATTGCGACTA-1 Patient5 Monocytes nonactive 4 0.02379367
Monocytes_4
10x_3288_t1_CTCAATTGCGTAGT-1Patient5 Progenitors active 1 0.17513157
Progenitors_1
10x_3288_t1_CTCAATTGTGTCCC-1 Patient5 Monocytes nonactive 0 0.07789614
Monocytes_0
10x_3288_t1_CTCAGAGAAGATGA-1 Patient5 Monocytes nonactive 0 0.0577484
Monocytes_0
10x_3288_t1_CTCAGAGACTCAGA-1 Patient5 Monocytes nonactive 0 0.05670217
Monocytes_0
10x_3288_t1_CTCAGAGAGCATCA-1 Patient5 Monocytes nonactive 13 0.03474732
Monocytes_13
10x_3288_t1_CTCAGAGAGCTGAT-1 Patient5 Monocytes nonactive 0 0.080908
Monocytes_0
10x_3288_t1_CTCAGAGAGGGTGA-1 Patient5 Progenitors active 1 0.13531165
Progenitors_1
10x_3288_t1_CTCAGAGATGCAGT-1 Patient5 Progenitors active 1 0.17204045
Progenitors_1
10x_3288_t1_CTCAGAGATGTCTT-1 Patient5 Progenitors active 1 0.19653161
Progenitors_1
10x_3288_t1_CTCAGCACAACCGT-1 Patient5 Progenitors active 1 0.1521622
Progenitors_1
10x_3288_t1_CTCAGCACAATGCC-1 Patient5 Monocytes nonactive 4 0.04018452
Monocytes_4
10x_3288_t1_CTCAGCACAGTGCT-1 Patient5 Dendritic cells nonactive 10 0.04948957

Dendritic cells_10

10x_3288_t1_CTCAGCACTCAAGC-1 Patient5 Monocytes nonactive 4 0.01753218 Monocytes_4

10x_3288_t1_CTCAGCTGCTGTCC-1 Patient5 Monocytes nonactive 4 0.04979076 Monocytes_4

10x_3288_t1_CTCAGCTGTTCGCC-1 Patient5 Progenitors nonactive 5 0.10906093 Progenitors_5

10x_3288_t1_CTCAGGCTATCTTC-1 Patient5 Monocytes nonactive 4 0.06927272 Monocytes_4

10x_3288_t1_CTCAGGCTCTCGCT-1 Patient5 Monocytes nonactive 4 0.07195168 Monocytes_4

10x_3288_t1_CTCAGGCTCTGCAA-1 Patient5 Monocytes nonactive 0 0.05608395 Monocytes_0

10x_3288_t1_CTCAGGCTGCCTTC-1 Patient5 Progenitors active 1 0.12064866 Progenitors_1

10x_3288_t1_CTCATTGAATGCCA-1 Patient5 Monocytes nonactive 0 0.09542832 Monocytes_0

10x_3288_t1_CTCATTGAATTTCC-1 Patient5 Monocytes nonactive 0 0.08923023 Monocytes_0

10x_3288_t1_CTCATTGACACCAA-1 Patient5 Monocytes nonactive 0 0.05911166 Monocytes_0

10x_3288_t1_CTCATTGACCCTCA-1 Patient5 Monocytes nonactive 4 0.03790185 Monocytes_4

10x_3288_t1_CTCCACGAGATACC-1 Patient5 Monocytes nonactive 4 0.03771162 Monocytes_4

10x_3288_t1_CTCCACGAGGACGA-1 Patient5 Progenitors nonactive 1 0.10953649 Progenitors_1

10x_3288_t1_CTCCATCTACCCTC-1 Patient5 Progenitors active 1 0.14683596 Progenitors_1

10x_3288_t1_CTCCATCTAGAGGC-1 Patient5 Monocytes nonactive 0 0.01586773 Monocytes_0

10x_3288_t1_CTCCATCTGACACT-1 Patient5 Monocytes nonactive 4 0.05397565 Monocytes_4

10x_3288_t1_CTCCATCTGGTGAG-1 Patient5 Monocytes nonactive 0 0.0152178 Monocytes_0
10x_3288_t1_CTCCGAACACTCAG-1 Patient5 Monocytes nonactive 0 0.02464967 Monocytes_0
10x_3288_t1_CTCCGAACCCACAA-1 Patient5 Progenitors active 1 0.13570794 Progenitors_1
10x_3288_t1_CTCCGAACTATGCG-1 Patient5 Monocytes nonactive 4 0.03400228 Monocytes_4
10x_3288_t1_CTCCTACTGAGGTG-1 Patient5 Progenitors active 6 0.14268277 Progenitors_6
10x_3288_t1_CTCGAAGACAGAGG-1 Patient5 Monocytes nonactive 0 0.02915161 Monocytes_0
10x_3288_t1_CTCGAAGACTGGTA-1 Patient5 Monocytes nonactive 0 0.01594699 Monocytes_0
10x_3288_t1_CTCGAAGAGAGGCA-1 Patient5 Monocytes nonactive 0 0.06867034 Monocytes_0
10x_3288_t1_CTCGACACAGCCAT-1 Patient5 Monocytes nonactive 4 0.0290565 Monocytes_4
10x_3288_t1_CTCGACACCACAAC-1 Patient5 Monocytes nonactive 4 0.05605225 Monocytes_4
10x_3288_t1_CTCGACACCTCATT-1Patient5 Dendritic cells nonactive 10 0.06881301 Dendritic cells_10
10x_3288_t1_CTCGACACGAATCC-1 Patient5 Monocytes nonactive 0 0.04248304 Monocytes_0
10x_3288_t1_CTCGACACGTTCAG-1 Patient5 Monocytes nonactive 4 0.07788029 Monocytes_4
10x_3288_t1_CTCGACACTCGCTC-1 Patient5 Monocytes nonactive 4 0.06095048 Monocytes_4
10x_3288_t1_CTCGACACTGTGAC-1 Patient5 Monocytes nonactive 8 0.02038552 Monocytes_8
10x_3288_t1_CTCGACTGACGTTG-1 Patient5 Monocytes nonactive 4 0.04324393 Monocytes_4
10x_3288_t1_CTCGACTGAGCTAC-1 Patient5 Progenitors active 1 0.1429047

Progenitors_1

10x_3288_t1_CTCGACTGTTCGTT-1Patient5 Monocytes nonactive 0 0.03742629 Monocytes_0

10x_3288_t1_CTCGAGCTCTACGA-1 Patient5 Monocytes nonactive 4 0.11522732 Monocytes_4

10x_3288_t1_CTCGCATGACACTG-1 Patient5 Monocytes nonactive 4 0.03947118 Monocytes_4

10x_3288_t1_CTCGCATGCCACCT-1 Patient5 Monocytes nonactive 8 0.04814216 Monocytes_8

10x_3288_t1_CTCGCATGTGCTAG-1 Patient5 Monocytes nonactive 4 0.03955044 Monocytes_4

10x_3288_t1_CTCTAAACGAGGTG-1 Patient5 Monocytes nonactive 0 0.03904318 Monocytes_0

10x_3288_t1_CTCTAAACGGCGAA-1 Patient5 Monocytes nonactive 0 0.05904825 Monocytes_0

10x_3288_t1_CTCTAAACTCTACT-1 Patient5 Monocytes nonactive 0 0.09672817 Monocytes_0

10x_3288_t1_CTCTAATGAAGCAA-1 Patient5 Progenitors active 1 0.14739078 Progenitors_1

10x_3288_t1_CTCTAATGCCCGTT-1 Patient5 Monocytes nonactive 4 0.04698497 Monocytes_4

10x_3288_t1_CTCTAATGCGTGAT-1 Patient5 Monocytes nonactive 0 0.00982817 Monocytes_0

10x_3288_t1_CTGAACGAAAACGA-1 Patient5 Monocytes nonactive 0 0.04197578 Monocytes_0

10x_3288_t1_CTGAACGAACCATG-1 Patient5 Progenitors nonactive 1 0.10142033 Progenitors_1

10x_3288_t1_CTGAACGACAGAAA-1 Patient5 Monocytes nonactive 0 0.03091117 Monocytes_0

10x_3288_t1_CTGAAGACCATCAG-1 Patient5 Monocytes nonactive 0 0.02896138 Monocytes_0

10x_3288_t1_CTGAAGACTTTCTG-1 Patient5 Monocytes nonactive 4 0.07073109 Monocytes_4

10x_3288_t1_CTGAAGTGAAGGTA-1 Patient5 Monocytes nonactive 4 0.03474732
Monocytes_4
10x_3288_t1_CTGAAGTGGGAGGT-1 Patient5 Progenitors active 1 0.12599074
Progenitors_1
10x_3288_t1_CTGAAGTGTCGTAG-1 Patient5 Monocytes nonactive 4 0.03167206
Monocytes_4
10x_3288_t1_CTGAAGTGTTTCTG-1 Patient5 Monocytes nonactive 0 0.0370617
Monocytes_0
10x_3288_t1_CTGAATCTCATTCT-1 Patient5 Monocytes nonactive 4 0.04969564
Monocytes_4
10x_3288_t1_CTGAATCTCTCATT-1 Patient5 Monocytes nonactive 4 0.05548158
Monocytes_4
10x_3288_t1_CTGAATCTCTGGTA-1 Patient5 Progenitors active 1 0.14845286
Progenitors_1
10x_3288_t1_CTGAATCTGGAGCA-1 Patient5 Monocytes nonactive 0 0.05687655
Monocytes_0
10x_3288_t1_CTGAATCTGGTAAA-1 Patient5 Monocytes nonactive 4 0.05012364
Monocytes_4
10x_3288_t1_CTGACAGAAAGTAG-1 Patient5 Monocytes nonactive 4 0.02555323
Monocytes_4
10x_3288_t1_CTGACAGACTTTAC-1 Patient5 Monocytes nonactive 4 0.0409137
Monocytes_4
10x_3288_t1_CTGACAGATCACGA-1 Patient5 Progenitors active 1 0.14189018
Progenitors_1
10x_3288_t1_CTGAGAACACGGTT-1 Patient5 Monocytes nonactive 13 0.09002283
Monocytes_13
10x_3288_t1_CTGAGAACCCTATT-1 Patient5 Monocytes nonactive 0 0.07345761
Monocytes_0
10x_3288_t1_CTGAGCCTAGAGTA-1 Patient5 Monocytes nonactive 4 0.03462051
Monocytes_4
10x_3288_t1_CTGAGCCTTCCAAG-1 Patient5 Monocytes nonactive 4 0.0678619
Monocytes_4
10x_3288_t1_CTGAGCCTTTTGGG-1 Patient5 Progenitors active 6 0.17424387

Progenitors_6

10x_3288_t1_CTGATACTGGGTGA-1 Patient5 Monocytes nonactive 0 0.05280261 Monocytes_0

10x_3288_t1_CTGATACTGGTAAA-1 Patient5 Monocytes nonactive 0 0.04070763 Monocytes_0

10x_3288_t1_CTGATACTTCAAGC-1 Patient5 Monocytes nonactive 0 0.03549236 Monocytes_0

10x_3288_t1_CTGATACTTTCTCA-1 Patient5 Progenitors active 1 0.18280388 Progenitors_1

10x_3288_t1_CTGATTTGCCTGAA-1 Patient5 Monocytes nonactive 0 0.0240473 Monocytes_0

10x_3288_t1_CTGATTTGTGCTTT-1 Patient5 Progenitors active 1 0.18649737 Progenitors_1

10x_3288_t1_CTGATTTGTTCTAC-1 Patient5 Progenitors active 1 0.13924291 Progenitors_1

10x_3288_t1_CTGATTTGTTGGCA-1 Patient5 Monocytes nonactive 0 0.05551328 Monocytes_0

10x_3288_t1_CTGCAGCTACCAAC-1 Patient5 Monocytes nonactive 0 0.02212922 Monocytes_0

10x_3288_t1_CTGCAGCTCATTGG-1 Patient5 Monocytes nonactive 4 0.0780071 Monocytes_4

10x_3288_t1_CTGCAGCTGGGCAA-1 Patient5 Monocytes nonactive 0 0.05686069 Monocytes_0

10x_3288_t1_CTGCCAACAAGGGC-1 Patient5 Monocytes nonactive 8 0.05039313 Monocytes_8

10x_3288_t1_CTGCCAACACCGAT-1 Patient5 Monocytes nonactive 4 0.0400577 Monocytes_4

10x_3288_t1_CTGCCAACCGTAGT-1 Patient5 Monocytes nonactive 4 0.02162196 Monocytes_4

10x_3288_t1_CTGCCAACCGTGAT-1 Patient5 Progenitors active 1 0.13035001 Progenitors_1

10x_3288_t1_CTGCCAACTCCAAG-1 Patient5 Dendritic cells nonactive 10 0.08745482 Dendritic cells_10

10x_3288_t1_CTGCGACTACCAGT-1 Patient5 Progenitors active 1 0.16798237 Progenitors_1
10x_3288_t1_CTGCGACTCTGACA-1 Patient5 Progenitors active 1 0.15514235 Progenitors_1
10x_3288_t1_CTGCGACTGCTTCC-1 Patient5 Monocytes nonactive 0 0.04340245 Monocytes_0
10x_3288_t1_CTGCGACTGTATCG-1 Patient5 Progenitors nonactive 1 0.11343605 Progenitors_1
10x_3288_t1_CTGCGACTTGCGTA-1 Patient5 Monocytes nonactive 4 0.04563756 Monocytes_4
10x_3288_t1_CTGCGACTTTCGTT-1Patient5 Progenitors active 1 0.17177097 Progenitors_1
10x_3288_t1_CTGGAAACAACGGG-1 Patient5 Progenitors active 12 0.13602498 Progenitors_12
10x_3288_t1_CTGGAAACGAGATA-1 Patient5 Progenitors active 1 0.1733086 Progenitors_1
10x_3288_t1_CTGGAAACTGCAAC-1 Patient5 Monocytes nonactive 4 0.05246972 Monocytes_4
10x_3288_t1_CTGGAAACTTGACG-1 Patient5 Monocytes nonactive 4 0.0252996 Monocytes_4
10x_3288_t1_CTGGATGAAAAACG-1 Patient5 Monocytes nonactive 0 0.06494515 Monocytes_0
10x_3288_t1_CTGGATGACTCGAA-1 Patient5 Monocytes nonactive 0 0.01719929 Monocytes_0
10x_3288_t1_CTGGATGACTGGTA-1 Patient5 Monocytes nonactive 0 0.07827658 Monocytes_0
10x_3288_t1_CTGGATGAGCGGAA-1 Patient5 Monocytes nonactive 0 0.04844335 Monocytes_0
10x_3288_t1_CTGGATGATGTAGC-1 Patient5 Monocytes nonactive 4 0.01567751 Monocytes_4
10x_3288_t1_CTGGCACTTAGCGT-1 Patient5 Progenitors active 1 0.12751252 Progenitors_1
10x_3288_t1_CTGGCACTTCTCCG-1 Patient5 Monocytes nonactive 4 0.05278676

Monocytes_4

10x_3288_t1_CTGTAACTCGCCTT-1Patient5 Progenitors active 1 0.14664574

Progenitors_1

10x_3288_t1_CTGTAACTTTCCAT-1 Patient5 Monocytes nonactive 4 0.04181726

Monocytes_4

10x_3288_t1_CTGTATACACTGGT-1Patient5 Monocytes nonactive 8 0.0345571

Monocytes_8

10x_3288_t1_CTGTATACAGCAAA-1 Patient5 Monocytes nonactive 0 0.03059413

Monocytes_0

10x_3288_t1_CTGTATACCATGCA-1Patient5 Monocytes nonactive 0 0.04337074

Monocytes_0

10x_3288_t1_CTGTATACTGTCAG-1Patient5 Monocytes nonactive 0 0.04991757

Monocytes_0

10x_3288_t1_CTGTATACTTGACG-1Patient5 Monocytes nonactive 0 0.03961385

Monocytes_0

10x_3288_t1_CTTAAAGAACCATG-1 Patient5 Monocytes nonactive 0 0.02539471

Monocytes_0

10x_3288_t1_CTTAAAGAACGGGA-1 Patient5 Monocytes nonactive 0 0.03395473

Monocytes_0

10x_3288_t1_CTTAAAGACCCGTT-1 Patient5 Monocytes nonactive 0 0.04666793

Monocytes_0

10x_3288_t1_CTTAAAGACCGTAA-1 Patient5 Progenitors active 1 0.13943314

Progenitors_1

10x_3288_t1_CTTAAAGACCTACC-1 Patient5 Monocytes nonactive 0 0.02488745

Monocytes_0

10x_3288_t1_CTTAAAGATCGTAG-1 Patient5 Monocytes nonactive 4 0.04820557

Monocytes_4

10x_3288_t1_CTTAACACATGTGC-1 Patient5 Monocytes nonactive 4 0.02246211

Monocytes_4

10x_3288_t1_CTTAACACTGCGTA-1 Patient5 Monocytes nonactive 0 0.04291104

Monocytes_0

10x_3288_t1_CTTAACACTTACTC-1Patient5 Progenitors active 1 0.17013823

Progenitors_1

10x_3288_t1_CTTAAGCTACTTTC-1 Patient5 Progenitors active 1 0.1201731 Progenitors_1
10x_3288_t1_CTTAAGCTAGCGGA-1 Patient5 Monocytes nonactive 0 0.0480629 Monocytes_0
10x_3288_t1_CTTACATGGTAGGG-1 Patient5 Monocytes nonactive 0 0.07902162 Monocytes_0
10x_3288_t1_CTTACTGACATGGT-1 Patient5 Monocytes nonactive 4 0.00920994 Monocytes_4
10x_3288_t1_CTTACTGAGTTTCT-1 Patient5 Progenitors active 1 0.14856382 Progenitors_1
10x_3288_t1_CTTACTGATGAGGG-1 Patient5 Monocytes nonactive 0 0.06971657 Monocytes_0
10x_3288_t1_CTTAGACTAGGTTC-1 Patient5 Monocytes nonactive 4 0.04581193 Monocytes_4
10x_3288_t1_CTTAGACTCTGAAC-1 Patient5 Monocytes nonactive 0 0.03707755 Monocytes_0
10x_3288_t1_CTTAGGGACTGAAC-1 Patient5 Monocytes nonactive 0 0.04294274 Monocytes_0
10x_3288_t1_CTTAGGGACTTGAG-1 Patient5 Progenitors active 1 0.17294401 Progenitors_1
10x_3288_t1_CTTAGGGAGAGATA-1 Patient5 Progenitors active 1 0.13502631 Progenitors_1
10x_3288_t1_CTTAGGGAGGTGGA-1 Patient5 Monocytes nonactive 8 0.03907488 Monocytes_8
10x_3288_t1_CTTAGGGAGTTCTT-1 Patient5 Monocytes nonactive 0 0.06595967 Monocytes_0
10x_3288_t1_CTTATCGAAAGGGC-1 Patient5 Monocytes nonactive 0 0.04574853 Monocytes_0
10x_3288_t1_CTTATCGAAGACTC-1 Patient5 Progenitors nonactive 1 0.11064612 Progenitors_1
10x_3288_t1_CTTATCGAATCACG-1 Patient5 Progenitors active 1 0.1382918 Progenitors_1
10x_3288_t1_CTTATCGAGGAGCA-1 Patient5 Progenitors active 1 0.14341196

Progenitors_1
10x_3288_t1_CTTCACCTAACTGC-1 Patient5 Progenitors active 1 0.1459007
Progenitors_1
10x_3288_t1_CTTCACCTATGCCA-1 Patient5 Monocytes nonactive 4 0.00166445
Monocytes_4
10x_3288_t1_CTTCACCTATGTGC-1 Patient5 Dendritic cells nonactive 10 0.05855684
Dendritic cells_10
10x_3288_t1_CTTCACCTCTGAGT-1 Patient5 Monocytes nonactive 0 0.05806544
Monocytes_0
10x_3288_t1_CTTCACCTGACGGA-1 Patient5 Monocytes nonactive 0 0.03714095
Monocytes_0
10x_3288_t1_CTTCACCTGAGGAC-1 Patient5 Monocytes nonactive 0 0.06388308
Monocytes_0
10x_3288_t1_CTTCACCTTGCACA-1 Patient5 Monocytes nonactive 4 0.03664955
Monocytes_4
10x_3288_t1_CTTCATGAATAAGG-1 Patient5 Monocytes nonactive 0 0.09874136
Monocytes_0
10x_3288_t1_CTTCATGATCCTAT-1 Patient5 Progenitors nonactive 1 0.10765012
Progenitors_1
10x_3288_t1_CTTCATGATTCCGC-1 Patient5 Progenitors active 1 0.14716885
Progenitors_1
10x_3288_t1_CTTCTAGAACCCAA-1 Patient5 Progenitors nonactive 1 0.10836345
Progenitors_1
10x_3288_t1_CTTCTAGAATTCGG-1 Patient5 Progenitors active 1 0.13952825
Progenitors_1
10x_3288_t1_CTTCTAGACCGTTC-1 Patient5 Monocytes nonactive 0 0.0965221
Monocytes_0
10x_3288_t1_CTTCTAGACTCAAG-1 Patient5 Monocytes nonactive 0 0.04287934
Monocytes_0
10x_3288_t1_CTTCTAGATCCAGA-1 Patient5 Progenitors active 6 0.16284636
Progenitors_6
10x_3288_t1_CTTGAACTAAGGTA-1 Patient5 Progenitors active 1 0.14937227
Progenitors_1

10x_3288_t1_CTTGAACTAGAAGT-1 Patient5 Monocytes nonactive 4 0.02961131
Monocytes_4
10x_3288_t1_CTTGAACTCGGGAA-1 Patient5 Monocytes nonactive 4 0.03246465
Monocytes_4
10x_3288_t1_CTTGAACTCTGTAG-1 Patient5 Monocytes nonactive 0 0.04967979
Monocytes_0
10x_3288_t1_CTTGAGGAATGACC-1 Patient5 Progenitors active 1 0.18681441
Progenitors_1
10x_3288_t1_CTTGAGGAATGCTG-1 Patient5 Progenitors active 1 0.15000634
Progenitors_1
10x_3288_t1_CTTGAGGATTCCCG-1 Patient5 Monocytes nonactive 0 0.03905903
Monocytes_0
10x_3288_t1_CTTGAGGATTTCAC-1 Patient5 Monocytes nonactive 4 0.04776171
Monocytes_4
10x_3288_t1_CTTGAGGATTTGCT-1Patient5 Monocytes nonactive 4 0.02582271
Monocytes_4
10x_3288_t1_CTTGATTGCTTCGC-1Patient5 Progenitors active 1 0.20000317
Progenitors_1
10x_3288_t1_CTTGATTGGGAAAT-1 Patient5 Monocytes nonactive 15 0.03717266
Monocytes_15
10x_3288_t1_CTTGTATGGGAAAT-1Patient5 Progenitors active 1 0.13754676
Progenitors_1
10x_3288_t1_CTTGTATGGTCTTT-1 Patient5 Monocytes nonactive 8 0.04652527
Monocytes_8
10x_3288_t1_CTTTACGAACAGCT-1 Patient5 Monocytes nonactive 0 0.03891637
Monocytes_0
10x_3288_t1_CTTTACGAACCATG-1 Patient5 Monocytes nonactive 0 0.03463636
Monocytes_0
10x_3288_t1_CTTTACGACCTATT-1 Patient5 Monocytes nonactive 8 0.05541817
Monocytes_8
10x_3288_t1_CTTTACGAGAAACA-1 Patient5 Monocytes nonactive 4 0.01293513
Monocytes_4
10x_3288_t1_CTTTACGAGGGAGT-1 Patient5 Monocytes nonactive 0 0.04570097

Monocytes_0
10x_3288_t1_CTTTAGACCACAAC-1 Patient5 Monocytes nonactive 8 0.05969818
Monocytes_8
10x_3288_t1_CTTTAGACGCAGAG-1 Patient5 Monocytes nonactive 0 0.07014457
Monocytes_0
10x_3288_t1_CTTTAGACTGACCA-1 Patient5 Monocytes nonactive 0 0.03474732
Monocytes_0
10x_3288_t1_CTTTAGTGACTGTG-1 Patient5 Monocytes nonactive 4 0.04655697
Monocytes_4
10x_3288_t1_CTTTAGTGATAAGG-1 Patient5 Monocytes nonactive 0 0.04186482
Monocytes_0
10x_3288_t1_CTTTAGTGATTCGG-1 Patient5 Progenitors active 6 0.1593431
Progenitors_6
10x_3288_t1_CTTTAGTGGCTCCT-1 Patient5 Monocytes nonactive 4 0.07171391
Monocytes_4
10x_3288_t1_CTTTAGTGGTTCGA-1 Patient5 Monocytes nonactive 4 0.05651195
Monocytes_4
10x_3288_t1_CTTTAGTGTCACCC-1 Patient5 Monocytes nonactive 4 0.02934183
Monocytes_4
10x_3288_t1_CTTTAGTGTCATTC-1 Patient5 Monocytes nonactive 4 0.0308002
Monocytes_4
10x_3288_t1_CTTTCAGAAAAGCA-1 Patient5 Progenitors nonactive 6 0.10208611
Progenitors_6
10x_3288_t1_CTTTGATGAATCGC-1 Patient5 Monocytes nonactive 0 0.05175639
Monocytes_0
10x_3288_t1_CTTTGATGCCTCCA-1 Patient5 Monocytes nonactive 8 0.08672564
Monocytes_8
10x_3288_t1_GAAAGATGGCATCA-1 Patient5 Monocytes nonactive 0 0.0544195
Monocytes_0
10x_3288_t1_GAAAGATGTGACAC-1 Patient5 Progenitors active 1 0.15694946
Progenitors_1
10x_3288_t1_GAAAGCCTAAAGCA-1 Patient5 Monocytes nonactive 0 0.0472386
Monocytes_0

10x_3288_t1_GAAAGCCTCGCTAA-1 Patient5 Monocytes nonactive 4 0.04693742 Monocytes_4
10x_3288_t1_GAAAGCCTGTCACA-1 Patient5 Progenitors active 1 0.15728235 Progenitors_1
10x_3288_t1_GAAAGCCTGTTGCA-1 Patient5 Progenitors nonactive 1 0.11283368 Progenitors_1
10x_3288_t1_GAAAGTGACAAGCT-1 Patient5 Monocytes nonactive 4 0.03977237 Monocytes_4
10x_3288_t1_GAAAGTGACGGTAT-1 Patient5 Progenitors nonactive 1 0.10622345 Progenitors_1
10x_3288_t1_GAAAGTGATGGTAC-1 Patient5 Dendritic cells nonactive 10 0.10711115 Dendritic cells_10
10x_3288_t1_GAAAGTGATGTGCA-1 Patient5 Monocytes nonactive 0 0.05032972 Monocytes_0
10x_3288_t1_GAAAGTGATTCCCG-1 Patient5 Progenitors active 1 0.14471181 Progenitors_1
10x_3288_t1_GAAAGTGATTGCAG-1 Patient5 Progenitors active 1 0.12724304 Progenitors_1
10x_3288_t1_GAAAGTGATTTGTC-1 Patient5 Monocytes nonactive 0 0.00906728 Monocytes_0
10x_3288_t1_GAAATACTCCGATA-1 Patient5 Monocytes nonactive 0 0.05001268 Monocytes_0
10x_3288_t1_GAAATACTCTCAGA-1 Patient5 Monocytes nonactive 0 0.05749477 Monocytes_0
10x_3288_t1_GAAATACTGGAACG-1 Patient5 Progenitors active 1 0.13336187 Progenitors_1
10x_3288_t1_GAACACACAGCCTA-1 Patient5 Monocytes nonactive 4 0.05140765 Monocytes_4
10x_3288_t1_GAACACACCCAACA-1 Patient5 Monocytes nonactive 0 0.06213937 Monocytes_0
10x_3288_t1_GAACAGCTACTGGT-1 Patient5 Progenitors active 1 0.16069051 Progenitors_1
10x_3288_t1_GAACAGCTCAGTCA-1 Patient5 Progenitors active 1 0.13431298

Progenitors_1

10x_3288_t1_GAACAGCTCAGTTG-1 Patient5 Monocytes nonactive 0 0.08079703 Monocytes_0

10x_3288_t1_GAACAGCTTGCACA-1 Patient5 Monocytes nonactive 4 0.02054404 Monocytes_4

10x_3288_t1_GAACAGCTTTTGTC-1 Patient5 Dendritic cells nonactive 10 0.07237968 Dendritic cells_10

10x_3288_t1_GAACCAACCTTGGA-1 Patient5 Progenitors nonactive 1 0.11278613 Progenitors_1

10x_3288_t1_GAACCAACTGTTCT-1 Patient5 Monocytes nonactive 0 0.06039566 Monocytes_0

10x_3288_t1_GAACCTGAAAACGA-1 Patient5 Monocytes nonactive 0 0.04340245 Monocytes_0

10x_3288_t1_GAACCTGACCTGTC-1 Patient5 Monocytes nonactive 0 0.061537 Monocytes_0

10x_3288_t1_GAACCTGATCATTC-1 Patient5 Monocytes nonactive 0 0.07805466 Monocytes_0

10x_3288_t1_GAACGGGACCTTTA-1 Patient5 Monocytes nonactive 4 0.0513918 Monocytes_4

10x_3288_t1_GAACGGGATCAGAC-1 Patient5 Progenitors active 1 0.12763934 Progenitors_1

10x_3288_t1_GAACGGGATTCCGC-1 Patient5 Monocytes nonactive 8 0.04291104 Monocytes_8

10x_3288_t1_GAACGTTGAGAGAT-1 Patient5 Monocytes nonactive 0 0.05651195 Monocytes_0

10x_3288_t1_GAACGTTGGGGACA-1 Patient5 Progenitors nonactive 1 0.11600406 Progenitors_1

10x_3288_t1_GAACTGTGCAGCTA-1 Patient5 Progenitors active 1 0.1412561 Progenitors_1

10x_3288_t1_GAACTGTGGGACGA-1 Patient5 Progenitors active 1 0.12191681 Progenitors_1

10x_3288_t1_GAACTGTGTATGCG-1 Patient5 Progenitors active 1 0.19722909 Progenitors_1

10x_3288_t1_GAAGAATGAACGTC-1 Patient5 Monocytes nonactive 0 0.05630588
Monocytes_0
10x_3288_t1_GAAGAATGTGCCTC-1 Patient5 Progenitors active 1 0.16264029
Progenitors_1
10x_3288_t1_GAAGATGAAGGTCT-1 Patient5 Progenitors active 1 0.16845793
Progenitors_1
10x_3288_t1_GAAGATGAATCTTC-1 Patient5 Monocytes nonactive 0 0.06120411
Monocytes_0
10x_3288_t1_GAAGATGAGAATCC-1 Patient5 Monocytes nonactive 4 0.04303785
Monocytes_4
10x_3288_t1_GAAGATGAGTAGCT-1 Patient5 Progenitors active 1 0.15514235
Progenitors_1
10x_3288_t1_GAAGATGATGCTCC-1 Patient5 Monocytes nonactive 4 0.03027709
Monocytes_4
10x_3288_t1_GAAGCGGAATTCGG-1 Patient5 Progenitors active 1 0.14455329
Progenitors_1
10x_3288_t1_GAAGCGGATGGCAT-1 Patient5 Monocytes nonactive 0 0.04562171
Monocytes_0
10x_3288_t1_GAAGCTACGAGGCA-1 Patient5 Progenitors active 1 0.15959673
Progenitors_1
10x_3288_t1_GAAGCTTGGGTCAT-1 Patient5 Monocytes nonactive 13 0.06516708
Monocytes_13
10x_3288_t1_GAAGCTTGGTCGTA-1 Patient5 Monocytes nonactive 4 0.02417412
Monocytes_4
10x_3288_t1_GAAGCTTGTGTGGT-1 Patient5 Progenitors nonactive 1 0.08617082
Progenitors_1
10x_3288_t1_GAAGGGTGAATGCC-1 Patient5 Progenitors active 1 0.16170503
Progenitors_1
10x_3288_t1_GAAGGGTGGCAGTT-1 Patient5 Progenitors active 1 0.18239173
Progenitors_1
10x_3288_t1_GAAGGGTGGGAAAT-1 Patient5 Monocytes nonactive 8 0.03948703
Monocytes_8
10x_3288_t1_GAAGGGTGTCAGGT-1 Patient5 Monocytes nonactive 8 0.06448545

Monocytes_8
10x_3288_t1_GAAGGGTGTGAGGG-1 Patient5 Monocytes nonactive 4 0.01859426
Monocytes_4
10x_3288_t1_GAAGGGTGTGCTAG-1 Patient5 Monocytes nonactive 4 0.0708579
Monocytes_4
10x_3288_t1_GAAGGTCTGTACGT-1 Patient5 Progenitors active 1 0.13599328
Progenitors_1
10x_3288_t1_GAAGGTCTGTTGGT-1 Patient5 Monocytes nonactive 4 0.0632173
Monocytes_4
10x_3288_t1_GAAGTAGAGCAAGG-1 Patient5 Monocytes nonactive 4 0.07199924
Monocytes_4
10x_3288_t1_GAAGTAGAGTACCA-1 Patient5 Dendritic cells nonactive 10 0.08485511
Dendritic cells_10
10x_3288_t1_GAAGTCACAGCTAC-1 Patient5 Progenitors active 1 0.16043688
Progenitors_1
10x_3288_t1_GAAGTCACATGACC-1 Patient5 Monocytes nonactive 4 0.07046161
Monocytes_4
10x_3288_t1_GAAGTCACCACTAG-1 Patient5 Monocytes nonactive 4 0.08533067
Monocytes_4
10x_3288_t1_GAAGTCTGACCGAT-1 Patient5 Monocytes nonactive 4 0.06399404
Monocytes_4
10x_3288_t1_GAAGTCTGACCTCC-1 Patient5 Progenitors nonactive 1 0.11199353
Progenitors_1
10x_3288_t1_GAAGTCTGCCCTTG-1 Patient5 Progenitors nonactive 1 0.1078879
Progenitors_1
10x_3288_t1_GAAGTCTGGTTGCA-1 Patient5 Monocytes nonactive 0 0.03015028
Monocytes_0
10x_3288_t1_GAAGTCTGTATCGG-1 Patient5 Monocytes nonactive 4 0.04280008
Monocytes_4
10x_3288_t1_GAAGTGCTCCTTGC-1 Patient5 Monocytes nonactive 0 0.04007355
Monocytes_0
10x_3288_t1_GAAGTGCTCGAACT-1 Patient5 Monocytes nonactive 4 0.07016042
Monocytes_4

10x_3288_t1_GAATGCACGTCATG-1 Patient5 Monocytes nonactive 13 0.07941792 Monocytes_13
10x_3288_t1_GAATGCACGTTGAC-1 Patient5 Monocytes nonactive 0 0.04383045 Monocytes_0
10x_3288_t1_GAATGCACTATGGC-1 Patient5 Monocytes nonactive 4 0.05259654 Monocytes_4
10x_3288_t1_GAATGCACTGACTG-1 Patient5 Monocytes nonactive 0 0.07356858 Monocytes_0
10x_3288_t1_GAATGCTGACGTGT-1 Patient5 Monocytes nonactive 0 0.06760827 Monocytes_0
10x_3288_t1_GAATGGCTCTCATT-1 Patient5 Progenitors active 1 0.13677002 Progenitors_1
10x_3288_t1_GAATGGCTGCTTAG-1 Patient5 Monocytes nonactive 0 0.05847759 Monocytes_0
10x_3288_t1_GAATGGCTGTACAC-1 Patient5 Monocytes nonactive 0 0.02693234 Monocytes_0
10x_3288_t1_GAATGGCTTATTCC-1 Patient5 Monocytes nonactive 0 0.02303278 Monocytes_0
10x_3288_t1_GAATGGCTTCTAGG-1 Patient5 Monocytes nonactive 4 0.03825059 Monocytes_4
10x_3288_t1_GAATGGCTTTGCAG-1 Patient5 Monocytes nonactive 0 0.02029041 Monocytes_0
10x_3288_t1_GAATTAACGTTTCT-1 Patient5 Monocytes nonactive 0 0.06347093 Monocytes_0
10x_3288_t1_GACAACACGGGCAA-1 Patient5 Monocytes nonactive 8 0.0134741 Monocytes_8
10x_3288_t1_GACAACTGAGCCTA-1 Patient5 Progenitors active 1 0.12626022 Progenitors_1
10x_3288_t1_GACAACTGCATGGT-1 Patient5 Monocytes nonactive 0 0.05846173 Monocytes_0
10x_3288_t1_GACACTGAATCTCT-1 Patient5 Monocytes nonactive 4 0.02734449 Monocytes_4
10x_3288_t1_GACACTGAATTCGG-1 Patient5 Monocytes nonactive 0 0.05116987

Monocytes_0
10x_3288_t1_GACACTGACCTGAA-1 Patient5 Monocytes nonactive 0 0.05554499 Monocytes_0
10x_3288_t1_GACACTGAGAAAGT-1 Patient5 Progenitors nonactive 1 0.06751316 Progenitors_1
10x_3288_t1_GACACTGAGGTGTT-1 Patient5 Monocytes nonactive 0 0.05673388 Monocytes_0
10x_3288_t1_GACAGGGAAGCATC-1 Patient5 Monocytes nonactive 4 0.0442743 Monocytes_4
10x_3288_t1_GACAGGGATCCTTA-1 Patient5 Monocytes nonactive 15 0.08360282 Monocytes_15
10x_3288_t1_GACAGTACGCTGTA-1 Patient5 Progenitors active 1 0.12126688 Progenitors_1
10x_3288_t1_GACAGTTGAGCTCA-1 Patient5 Progenitors active 1 0.19941665 Progenitors_1
10x_3288_t1_GACAGTTGAGTAGA-1 Patient5 Monocytes nonactive 4 0.04682645 Monocytes_4
10x_3288_t1_GACAGTTGGAAAGT-1 Patient5 Monocytes nonactive 0 0.06320145 Monocytes_0
10x_3288_t1_GACATTCTAAGTGA-1 Patient5 Progenitors active 6 0.17265868 Progenitors_6
10x_3288_t1_GACATTCTAATGCC-1 Patient5 Progenitors active 1 0.14941982 Progenitors_1
10x_3288_t1_GACATTCTGGAGGT-1 Patient5 Progenitors active 1 0.1446167 Progenitors_1
10x_3288_t1_GACATTCTGGTTAC-1 Patient5 Monocytes nonactive 4 0.04118318 Monocytes_4
10x_3288_t1_GACATTCTTCCTTA-1 Patient5 Monocytes nonactive 0 0.06248811 Monocytes_0
10x_3288_t1_GACATTCTTGAGCT-1 Patient5 Progenitors nonactive 1 0.09910595 Progenitors_1
10x_3288_t1_GACATTCTTGGATC-1 Patient5 Monocytes nonactive 8 0.08545749 Monocytes_8

10x_3288_t1_GACATTCTTTCCCG-1 Patient5 Progenitors active 1 0.13943314 Progenitors_1
10x_3288_t1_GACCAAACCCTCCA-1 Patient5 Progenitors active 1 0.14826263 Progenitors_1
10x_3288_t1_GACCAAACTCCCAC-1 Patient5 Progenitors active 1 0.16035762 Progenitors_1
10x_3288_t1_GACCAAACTCTCTA-1 Patient5 Progenitors nonactive 1 0.09182994 Progenitors_1
10x_3288_t1_GACCATGAAAACGA-1 Patient5 Monocytes nonactive 8 0.08189081 Monocytes_8
10x_3288_t1_GACCATGAATTTCC-1 Patient5 Progenitors active 1 0.17454505 Progenitors_1
10x_3288_t1_GACCATGATATTCC-1 Patient5 Monocytes nonactive 0 0.06121996 Monocytes_0
10x_3288_t1_GACCCTACAGCATC-1 Patient5 Monocytes nonactive 0 0.07634265 Monocytes_0
10x_3288_t1_GACCCTACTCTCCG-1 Patient5 Progenitors active 1 0.19068226 Progenitors_1
10x_3288_t1_GACCCTACTTTGGG-1 Patient5 Monocytes nonactive 4 0.03904318 Monocytes_4
10x_3288_t1_GACCTAGAAGAGTA-1 Patient5 Monocytes nonactive 4 0.0164701 Monocytes_4
10x_3288_t1_GACCTAGAGTTTCT-1 Patient5 Monocytes nonactive 0 0.03010272 Monocytes_0
10x_3288_t1_GACCTAGATATCTC-1 Patient5 Monocytes nonactive 8 0.03094287 Monocytes_8
10x_3288_t1_GACCTAGATGTTCT-1 Patient5 Monocytes nonactive 0 0.02777249 Monocytes_0
10x_3288_t1_GACCTCACTAGCGT-1 Patient5 Monocytes nonactive 4 0.05443536 Monocytes_4
10x_3288_t1_GACCTCTGCTGACA-1 Patient5 Monocytes nonactive 0 0.10947308 Monocytes_0
10x_3288_t1_GACCTCTGTGCCTC-1 Patient5 Monocytes nonactive 0 0.04276837

Monocytes_0
10x_3288_t1_GACGAACTCATCAG-1 Patient5 Monocytes nonactive 4 0.0159787
Monocytes_4
10x_3288_t1_GACGAACTCGCATA-1 Patient5 Progenitors active 1 0.12835267
Progenitors_1
10x_3288_t1_GACGAACTGGTGGA-1 Patient5 Monocytes nonactive 0 0.08319067
Monocytes_0
10x_3288_t1_GACGAACTGTACGT-1 Patient5 Monocytes nonactive 0 0.02109885
Monocytes_0
10x_3288_t1_GACGAACTTGTGGT-1 Patient5 Monocytes nonactive 0 0.03105383
Monocytes_0
10x_3288_t1_GACGAGGAACTCAG-1 Patient5 Monocytes nonactive 4 0.05674973
Monocytes_4
10x_3288_t1_GACGAGGACTGAAC-1 Patient5 Monocytes nonactive 4 0.03276584
Monocytes_4
10x_3288_t1_GACGATTGCCTACC-1 Patient5 Progenitors active 1 0.14060618
Progenitors_1
10x_3288_t1_GACGATTGCTCATT-1 Patient5 Monocytes nonactive 4 0.06597552
Monocytes_4
10x_3288_t1_GACGATTGTTGCGA-1 Patient5 Monocytes nonactive 0 0.03842496
Monocytes_0
10x_3288_t1_GACGCCGACCCAAA-1 Patient5 Progenitors active 1 0.15032338
Progenitors_1
10x_3288_t1_GACGCCGACGGAGA-1 Patient5 Monocytes nonactive 0 0.01529706
Monocytes_0
10x_3288_t1_GACGCCGATCTACT-1 Patient5 Monocytes nonactive 0 0.03934437
Monocytes_0
10x_3288_t1_GACGCTCTCAGAGG-1 Patient5 Monocytes nonactive 4 0.08122503
Monocytes_4
10x_3288_t1_GACGCTCTCGGAGA-1 Patient5 Monocytes nonactive 4 0.04492423
Monocytes_4
10x_3288_t1_GACGCTCTGTTGGT-1 Patient5 Monocytes nonactive 0 0.06500856
Monocytes_0

10x_3288_t1_GACGGCACCTCTAT-1 Patient5 Monocytes nonactive 4 0.03195739 Monocytes_4
10x_3288_t1_GACGGCACGTTGCA-1 Patient5 Monocytes nonactive 4 0.03840911 Monocytes_4
10x_3288_t1_GACGGCACTCTTAC-1 Patient5 Monocytes nonactive 4 0.03878955 Monocytes_4
10x_3288_t1_GACGTAACCACTAG-1 Patient5 Monocytes nonactive 4 0.05595714 Monocytes_4
10x_3288_t1_GACGTATGACGCAT-1 Patient5 Monocytes nonactive 0 0.01849914 Monocytes_0
10x_3288_t1_GACGTATGGGTCAT-1 Patient5 Monocytes nonactive 4 0.02393634 Monocytes_4
10x_3288_t1_GACGTATGTTCGGA-1 Patient5 Progenitors active 1 0.13198275 Progenitors_1
10x_3288_t1_GACGTCCTACTCAG-1 Patient5 Monocytes nonactive 0 0.01651766 Monocytes_0
10x_3288_t1_GACGTCCTCGTGTA-1 Patient5 Monocytes nonactive 8 0.00827468 Monocytes_8
10x_3288_t1_GACTACGACGTCTC-1 Patient5 Monocytes nonactive 8 0.10658804 Monocytes_8
10x_3288_t1_GACTACGACGTGAT-1 Patient5 Monocytes nonactive 4 0.05128083 Monocytes_4
10x_3288_t1_GACTACGATGCCAA-1 Patient5 Monocytes nonactive 0 0.04795194 Monocytes_0
10x_3288_t1_GACTCCTGGCTTCC-1 Patient5 Monocytes nonactive 0 0.06649864 Monocytes_0
10x_3288_t1_GACTCCTGGTTGCA-1 Patient5 Monocytes nonactive 4 0.04761905 Monocytes_4
10x_3288_t1_GACTCCTGTTGACG-1 Patient5 Progenitors active 1 0.18625959 Progenitors_1
10x_3288_t1_GACTGAACCGCCTT-1 Patient5 Monocytes nonactive 4 0.05343669 Monocytes_4
10x_3288_t1_GACTGAACGTATGC-1 Patient5 Monocytes nonactive 4 0.02174878

Monocytes_4
10x_3288_t1_GACTGAACTCGTTT-1 Patient5 Progenitors active 1 0.15732991
Progenitors_1
10x_3288_t1_GACTGATGACTACG-1 Patient5 Progenitors active 1 0.18142477
Progenitors_1
10x_3288_t1_GACTGATGGGTGAG-1 Patient5 Progenitors active 1 0.16972608
Progenitors_1
10x_3288_t1_GACTGTGAAATGCC-1 Patient5 Progenitors active 6 0.15003804
Progenitors_6
10x_3288_t1_GACTGTGAACAGTC-1 Patient5 Monocytes nonactive 8 0.02331812
Monocytes_8
10x_3288_t1_GACTGTGAAGCCAT-1 Patient5 Monocytes nonactive 0 0.04826897
Monocytes_0
10x_3288_t1_GACTTTACGAGGTG-1 Patient5 Progenitors active 1 0.12277281
Progenitors_1
10x_3288_t1_GACTTTACTGTGAC-1 Patient5 Progenitors active 1 0.16175258
Progenitors_1
10x_3288_t1_GAGAAATGGAGATA-1 Patient5 Progenitors active 1 0.16538266
Progenitors_1
10x_3288_t1_GAGAAATGTACTGG-1 Patient5 Progenitors nonactive 1 0.11676495
Progenitors_1
10x_3288_t1_GAGAAATGTTCACT-1 Patient5 Progenitors nonactive 1 0.10598567
Progenitors_1
10x_3288_t1_GAGAAATGTTTGTC-1 Patient5 Monocytes nonactive 4 0.04490838
Monocytes_4
10x_3288_t1_GAGAGGTGCTTCGC-1 Patient5 Monocytes nonactive 0 0.0333365
Monocytes_0
10x_3288_t1_GAGAGGTGTACTCT-1 Patient5 Progenitors active 1 0.1361835
Progenitors_1
10x_3288_t1_GAGATAGACAACTG-1 Patient5 Monocytes nonactive 0 0.03633251
Monocytes_0
10x_3288_t1_GAGATAGACAGTCA-1 Patient5 Monocytes nonactive 13 0.07310887
Monocytes_13

10x_3288_t1_GAGATAGACATTTC-1 Patient5 Monocytes nonactive 0 0.03953459
Monocytes_0
10x_3288_t1_GAGATAGACCGCTT-1 Patient5 Monocytes nonactive 0 0.05898485
Monocytes_0
10x_3288_t1_GAGATAGATCGCCT-1 Patient5 Monocytes nonactive 4 0.0573204
Monocytes_4
10x_3288_t1_GAGATAGATTCCGC-1 Patient5 Monocytes nonactive 4 0.05739966
Monocytes_4
10x_3288_t1_GAGATCACGAACTC-1 Patient5 Progenitors active 1 0.14487033
Progenitors_1
10x_3288_t1_GAGATCACGCAAGG-1 Patient5 Monocytes nonactive 0 0.06267833
Monocytes_0
10x_3288_t1_GAGATGCTACGGGA-1 Patient5 Monocytes nonactive 0 0.04876038
Monocytes_0
10x_3288_t1_GAGATGCTCCGATA-1 Patient5 Monocytes nonactive 4 0.03260732
Monocytes_4
10x_3288_t1_GAGATGCTCGCATA-1 Patient5 Monocytes nonactive 0 0.04245133
Monocytes_0
10x_3288_t1_GAGATGCTTCTCCG-1 Patient5 Progenitors active 1 0.11833428
Progenitors_1
10x_3288_t1_GAGCAACTGTTTCT-1 Patient5 Progenitors active 1 0.1442204
Progenitors_1
10x_3288_t1_GAGCAGGATCGTGA-1 Patient5 Monocytes nonactive 8 0.04340245
Monocytes_8
10x_3288_t1_GAGCAGGATGAGGG-1 Patient5 Monocytes nonactive 13 0.06626086
Monocytes_13
10x_3288_t1_GAGCATACCCAAGT-1 Patient5 Monocytes nonactive 8 0.07035064
Monocytes_8
10x_3288_t1_GAGCATACGGACAG-1 Patient5 Monocytes nonactive 4 0.04584364
Monocytes_4
10x_3288_t1_GAGCGAGACCCTAC-1 Patient5 Progenitors active 1 0.12183755
Progenitors_1
10x_3288_t1_GAGCGAGACGTGTA-1 Patient5 Monocytes nonactive 16 0.10918775

Monocytes_16

10x_3288_t1_GAGCGAGAGTATCG-1 Patient5 Progenitors active 1 0.14691522 Progenitors_1

10x_3288_t1_GAGCGAGATACGAC-1 Patient5 Monocytes nonactive 4 0.03634836 Monocytes_4

10x_3288_t1_GAGCGCACACGGGA-1 Patient5 Monocytes nonactive 4 0.04327563 Monocytes_4

10x_3288_t1_GAGCGCACAGGGTG-1 Patient5 Monocytes nonactive 4 0.03924925 Monocytes_4

10x_3288_t1_GAGCGCACTGGTCA-1 Patient5 Monocytes nonactive 0 0.04245133 Monocytes_0

10x_3288_t1_GAGCGCACTTGCAG-1 Patient5 Monocytes nonactive 4 0.03282924 Monocytes_4

10x_3288_t1_GAGCGCTGGCAGTT-1 Patient5 Monocytes nonactive 8 0.03663369 Monocytes_8

10x_3288_t1_GAGCGCTGTTTGTC-1 Patient5 Monocytes nonactive 0 0.01594699 Monocytes_0

10x_3288_t1_GAGCGGCTTCCTTA-1 Patient5 Monocytes nonactive 0 0.05513284 Monocytes_0

10x_3288_t1_GAGCGGCTTTGACG-1 Patient5 Progenitors active 1 0.12587978 Progenitors_1

10x_3288_t1_GAGCTCCTAACCGT-1 Patient5 Progenitors nonactive 1 0.07348932 Progenitors_1

10x_3288_t1_GAGCTCCTAGTAGA-1 Patient5 Monocytes nonactive 4 0.0236193 Monocytes_4

10x_3288_t1_GAGCTCCTGGAACG-1 Patient5 Monocytes nonactive 0 0.06472323 Monocytes_0

10x_3288_t1_GAGCTCCTGGTAGG-1 Patient5 Monocytes nonactive 4 0.06044322 Monocytes_4

10x_3288_t1_GAGGACGACAACTG-1 Patient5 Monocytes nonactive 0 0.05641684 Monocytes_0

10x_3288_t1_GAGGACGAGGGCAA-1 Patient5 Monocytes nonactive 0 0.02591782 Monocytes_0

10x_3288_t1_GAGGATCTAGTCAC-1 Patient5 Monocytes nonactive 4 0.03230613 Monocytes_4
10x_3288_t1_GAGGATCTCAGAAA-1 Patient5 Monocytes nonactive 4 0.05202587 Monocytes_4
10x_3288_t1_GAGGATCTCTCAGA-1 Patient5 Monocytes nonactive 4 0.02816879 Monocytes_4
10x_3288_t1_GAGGATCTCTCATT-1 Patient5 Monocytes nonactive 4 0.03530214 Monocytes_4
10x_3288_t1_GAGGATCTGGATCT-1 Patient5 Monocytes nonactive 4 0.04532053 Monocytes_4
10x_3288_t1_GAGGATCTGTCGTA-1 Patient5 Monocytes nonactive 4 0.0291199 Monocytes_4
10x_3288_t1_GAGGCAGACGTTGA-1 Patient5 Monocytes nonactive 4 0.01973559 Monocytes_4
10x_3288_t1_GAGGCCACAGCCAT-1 Patient5 Monocytes nonactive 0 0.04272082 Monocytes_0
10x_3288_t1_GAGGCCACGAAGGC-1 Patient5 Monocytes nonactive 4 0.07383806 Monocytes_4
10x_3288_t1_GAGGCCACGTGAGG-1 Patient5 Monocytes nonactive 4 0.06681567 Monocytes_4
10x_3288_t1_GAGGGAACAACGGG-1 Patient5 Progenitors nonactive 1 0.11044005 Progenitors_1
10x_3288_t1_GAGGGAACTGAAGA-1 Patient5 Progenitors active 1 0.17993469 Progenitors_1
10x_3288_t1_GAGGGATGACGTGT-1 Patient5 Monocytes nonactive 4 0.04257815 Monocytes_4
10x_3288_t1_GAGGGATGAGTAGA-1 Patient5 Monocytes nonactive 4 0.05308795 Monocytes_4
10x_3288_t1_GAGGGATGGACAAA-1 Patient5 Monocytes nonactive 8 0.03734703 Monocytes_8
10x_3288_t1_GAGGGATGTCCAAG-1 Patient5 Progenitors nonactive 1 0.10894997 Progenitors_1
10x_3288_t1_GAGGGATGTCTATC-1 Patient5 Monocytes nonactive 4 0.0573204

Monocytes_4

10x_3288_t1_GAGGGCCTCAATCG-1 Patient5 Progenitors nonactive 1 0.11719295 Progenitors_1

10x_3288_t1_GAGGGCCTCCACAA-1 Patient5 Progenitors active 1 0.12434215 Progenitors_1

10x_3288_t1_GAGGGCCTGAACTC-1 Patient5 Monocytes nonactive 4 0.06277344 Monocytes_4

10x_3288_t1_GAGGGTGAAGAGAT-1 Patient5 Monocytes nonactive 0 0.03274998 Monocytes_0

10x_3288_t1_GAGGGTGACATCAG-1 Patient5 Monocytes nonactive 0 0.06399404 Monocytes_0

10x_3288_t1_GAGGGTGAGGACTT-1 Patient5 Monocytes nonactive 4 0.03471562 Monocytes_4

10x_3288_t1_GAGGTACTAACCGT-1 Patient5 Monocytes nonactive 8 0.03915414 Monocytes_8

10x_3288_t1_GAGGTACTAATCGC-1 Patient5 Progenitors active 1 0.17671676 Progenitors_1

10x_3288_t1_GAGGTACTACGCTA-1 Patient5 Progenitors active 1 0.19486716 Progenitors_1

10x_3288_t1_GAGGTACTCAGTTG-1 Patient5 Progenitors active 1 0.15377909 Progenitors_1

10x_3288_t1_GAGGTACTTTCCCG-1 Patient5 Monocytes nonactive 0 0.05719358 Monocytes_0

10x_3288_t1_GAGGTGGACCACCT-1 Patient5 Monocytes nonactive 0 0.09370046 Monocytes_0

10x_3288_t1_GAGGTTACGAGGCA-1 Patient5 Monocytes nonactive 0 0.05614736 Monocytes_0

10x_3288_t1_GAGGTTACGGACTT-1 Patient5 Monocytes nonactive 4 0.04200748 Monocytes_4

10x_3288_t1_GAGGTTTGAGGTCT-1 Patient5 Monocytes nonactive 4 0.02387293 Monocytes_4

10x_3288_t1_GAGGTTTGGGGACA-1 Patient5 Progenitors active 1 0.2044417 Progenitors_1

10x_3288_t1_GAGGTTTGTCAGTG-1 Patient5 Monocytes nonactive 0 0.0412783 Monocytes_0
10x_3288_t1_GAGGTTTGTGAGAA-1 Patient5 Monocytes nonactive 3 0.02184389 Monocytes_3
10x_3288_t1_GAGGTTTGTTGCAG-1 Patient5 Progenitors nonactive 1 0.09731469 Progenitors_1
10x_3288_t1_GAGTAAGACAACCA-1 Patient5 Monocytes nonactive 4 0.04096126 Monocytes_4
10x_3288_t1_GAGTAAGAGAGGCA-1 Patient5 Progenitors active 1 0.15110012 Progenitors_1
10x_3288_t1_GAGTAAGATCTCGC-1 Patient5 Progenitors active 1 0.16000888 Progenitors_1
10x_3288_t1_GAGTAAGATGAGAA-1 Patient5 Monocytes nonactive 0 0.05752647 Monocytes_0
10x_3288_t1_GAGTAAGATGGAGG-1 Patient5 Progenitors active 1 0.1298269 Progenitors_1
10x_3288_t1_GAGTACACCGGAGA-1 Patient5 Progenitors active 1 0.16796652 Progenitors_1
10x_3288_t1_GAGTACACGACAGG-1 Patient5 Monocytes nonactive 4 0.06435863 Monocytes_4
10x_3288_t1_GAGTACACTAGCGT-1 Patient5 Monocytes active 0 0.11871473 Monocytes_0
10x_3288_t1_GAGTACACTCCTTA-1 Patient5 Monocytes nonactive 4 0.02864435 Monocytes_4
10x_3288_t1_GAGTACACTGAGGG-1 Patient5 Monocytes nonactive 0 0.05597299 Monocytes_0
10x_3288_t1_GAGTACACTGGAAA-1 Patient5 Monocytes nonactive 4 0.03614229 Monocytes_4
10x_3288_t1_GAGTACACTTGGTG-1 Patient5 Progenitors active 1 0.14005136 Progenitors_1
10x_3288_t1_GAGTACTGCTACTT-1Patient5 Monocytes nonactive 0 0.06470737 Monocytes_0
10x_3288_t1_GAGTACTGCTCTAT-1 Patient5 Monocytes nonactive 4 0.06691079

Monocytes_4
10x_3288_t1_GAGTACTGGCGATT-1 Patient5 Progenitors active 1 0.1210291
Progenitors_1
10x_3288_t1_GAGTACTGTAGAGA-1 Patient5 Progenitors active 1 0.17107349
Progenitors_1
10x_3288_t1_GAGTACTGTGCGTA-1 Patient5 Dendritic cells active 10 0.15740917
Dendritic cells_10
10x_3288_t1_GAGTACTGTTCCGC-1 Patient5 Monocytes nonactive 0 0.04394141
Monocytes_0
10x_3288_t1_GAGTACTGTTGAGC-1 Patient5 Monocytes nonactive 0 0.06133092
Monocytes_0
10x_3288_t1_GAGTCAACACCTCC-1 Patient5 Monocytes nonactive 4 0.0362374
Monocytes_4
10x_3288_t1_GAGTCAACACTGGT-1 Patient5 Monocytes nonactive 0 0.03780673
Monocytes_0
10x_3288_t1_GAGTCAACTGACAC-1 Patient5 Monocytes nonactive 4 0.02542642
Monocytes_4
10x_3288_t1_GAGTCAACTGGGAG-1 Patient5 Monocytes nonactive 0 0.01810285
Monocytes_0
10x_3288_t1_GAGTCTGACATGCA-1 Patient5 Monocytes nonactive 4 0.09760003
Monocytes_4
10x_3288_t1_GAGTCTGACTATTC-1 Patient5 Monocytes nonactive 4 0.00817957
Monocytes_4
10x_3288_t1_GAGTCTGATCGTTT-1Patient5 Progenitors active 1 0.20090673
Progenitors_1
10x_3288_t1_GAGTGACTAAACAG-1 Patient5 Progenitors active 1 0.17264283
Progenitors_1
10x_3288_t1_GAGTGACTACGACT-1 Patient5 Progenitors nonactive 1 0.10310063
Progenitors_1
10x_3288_t1_GAGTGACTAGCGTT-1 Patient5 Progenitors active 1 0.16452666
Progenitors_1
10x_3288_t1_GAGTGGGAACCTAG-1 Patient5 Progenitors active 6 0.15520576
Progenitors_6

10x_3288_t1_GAGTGGGACCCGTT-1 Patient5 Monocytes nonactive 4 0.05464143 Monocytes_4
10x_3288_t1_GAGTGGGACCGATA-1 Patient5 Monocytes nonactive 0 0.04335489 Monocytes_0
10x_3288_t1_GAGTGGGAGAGGTG-1 Patient5 Monocytes nonactive 4 0.04417919 Monocytes_4
10x_3288_t1_GAGTGGGATATCTC-1 Patient5 Progenitors active 1 0.13990869 Progenitors_1
10x_3288_t1_GAGTGTTGAGGTCT-1 Patient5 Monocytes nonactive 0 0.02861264 Monocytes_0
10x_3288_t1_GAGTGTTGGCTACA-1 Patient5 Monocytes nonactive 0 0.02916746 Monocytes_0
10x_3288_t1_GAGTGTTGGCTATG-1 Patient5 Monocytes nonactive 0 0.04295859 Monocytes_0
10x_3288_t1_GAGTTGTGAGTCAC-1 Patient5 Progenitors active 1 0.16810919 Progenitors_1
10x_3288_t1_GAGTTGTGCAATCG-1 Patient5 Monocytes nonactive 0 0.07762666 Monocytes_0
10x_3288_t1_GAGTTGTGCAGAAA-1 Patient5 Monocytes nonactive 8 0.02671042 Monocytes_8
10x_3288_t1_GAGTTGTGCCGTAA-1 Patient5 Progenitors active 1 0.12652971 Progenitors_1
10x_3288_t1_GAGTTGTGTCTTAC-1 Patient5 Monocytes nonactive 4 0.04814216 Monocytes_4
10x_3288_t1_GAGTTGTGTGGGAG-1 Patient5 Monocytes nonactive 4 0.06225033 Monocytes_4
10x_3288_t1_GATAAGGAAGATGA-1 Patient5 Progenitors active 1 0.12484941 Progenitors_1
10x_3288_t1_GATAAGGAGCGTTA-1 Patient5 Monocytes nonactive 3 0.07049331 Monocytes_3
10x_3288_t1_GATAAGGAGGACAG-1 Patient5 Monocytes nonactive 0 0.04674719 Monocytes_0
10x_3288_t1_GATAATACAGAGAT-1Patient5 Monocytes nonactive 4 0.04547904

Monocytes_4

10x_3288_t1_GATAATACCCACCT-1 Patient5 Dendritic cells active 10 0.12613341

Dendritic cells_10

10x_3288_t1_GATAATACTCCCAC-1 Patient5 Monocytes nonactive 8 0.01895885

Monocytes_8

10x_3288_t1_GATACTCTAACGGG-1 Patient5 Monocytes nonactive 4 0.02894553

Monocytes_4

10x_3288_t1_GATACTCTGAGCTT-1 Patient5 Monocytes nonactive 13 0.06129922

Monocytes_13

10x_3288_t1_GATACTCTGTTGTG-1 Patient5 Progenitors active 1 0.13897343

Progenitors_1

10x_3288_t1_GATACTCTTCGCCT-1 Patient5 Monocytes nonactive 0 0.02709086

Monocytes_0

10x_3288_t1_GATAGAGAGGTTAC-1 Patient5 Progenitors active 1 0.1311109

Progenitors_1

10x_3288_t1_GATAGCACACACGT-1 Patient5 Progenitors active 1 0.13577135

Progenitors_1

10x_3288_t1_GATAGCACTCGCCT-1 Patient5 Monocytes nonactive 4 0.0497749

Monocytes_4

10x_3288_t1_GATATAACACACTG-1 Patient5 Monocytes nonactive 4 0.04883964

Monocytes_4

10x_3288_t1_GATATAACTGGAAA-1 Patient5 Monocytes nonactive 4 0.05143935

Monocytes_4

10x_3288_t1_GATATATGAACCGT-1 Patient5 Monocytes nonactive 0 0.04389386

Monocytes_0

10x_3288_t1_GATATATGACCCTC-1 Patient5 Progenitors active 1 0.15335109

Progenitors_1

10x_3288_t1_GATATATGAGAGAT-1 Patient5 Monocytes nonactive 4 0.00716505

Monocytes_4

10x_3288_t1_GATATATGAGAGGC-1 Patient5 Monocytes nonactive 0 0.02141589

Monocytes_0

10x_3288_t1_GATATATGCCGCTT-1 Patient5 Monocytes nonactive 0 0.02040137

Monocytes_0

10x_3288_t1_GATATCCTATGTCG-1 Patient5 Progenitors active 1 0.14016232
Progenitors_1
10x_3288_t1_GATATCCTCGCATA-1 Patient5 Monocytes nonactive 4 0.04230867
Monocytes_4
10x_3288_t1_GATATCCTTGGAAA-1 Patient5 Monocytes nonactive 8 0.02796272
Monocytes_8
10x_3288_t1_GATATTGATTGGTG-1 Patient5 Monocytes nonactive 13 0.05327817
Monocytes_13
10x_3288_t1_GATCATCTGGACAG-1 Patient5 Monocytes nonactive 0 0.04666793
Monocytes_0
10x_3288_t1_GATCATCTGTAGGG-1 Patient5 Progenitors active 1 0.1757815
Progenitors_1
10x_3288_t1_GATCCCTGCAGGAG-1 Patient5 Progenitors active 1 0.1222814
Progenitors_1
10x_3288_t1_GATCCCTGGTCTAG-1 Patient5 Progenitors nonactive 1 0.09634773
Progenitors_1
10x_3288_t1_GATCCCTGTAAGGA-1 Patient5 Monocytes nonactive 0 0.0349851
Monocytes_0
10x_3288_t1_GATCCCTGTAGAGA-1 Patient5 Progenitors active 1 0.14637626
Progenitors_1
10x_3288_t1_GATCCGCTACTAGC-1 Patient5 Monocytes nonactive 8 0.06886057
Monocytes_8
10x_3288_t1_GATCCGCTCATACG-1 Patient5 Progenitors active 6 0.15328768
Progenitors_6
10x_3288_t1_GATCCGCTGACAAA-1 Patient5 Monocytes nonactive 4 0.02287426
Monocytes_4
10x_3288_t1_GATCCGCTTTGAGC-1 Patient5 Progenitors nonactive 5 0.11728806
Progenitors_5
10x_3288_t1_GATCGAACAAAACG-1 Patient5 Progenitors active 1 0.18012491
Progenitors_1
10x_3288_t1_GATCGAACAACAGA-1 Patient5 Progenitors active 1 0.14414115
Progenitors_1
10x_3288_t1_GATCGAACAGCTCA-1 Patient5 Monocytes nonactive 4 0.0080052

Monocytes_4

10x_3288_t1_GATCGAACGGTCAT-1 Patient5 Dendritic cells nonactive 10 0.07444043 Dendritic cells_10

10x_3288_t1_GATCGATGCCCTTG-1 Patient5 Progenitors nonactive 1 0.09804388 Progenitors_1

10x_3288_t1_GATCGATGGGAAAT-1 Patient5 Monocytes nonactive 0 0.02320715 Monocytes_0

10x_3288_t1_GATCGATGTCCTAT-1 Patient5 Monocytes nonactive 0 0.0341608 Monocytes_0

10x_3288_t1_GATCGTGAGAGCTT-1 Patient5 Progenitors active 1 0.1749255 Progenitors_1

10x_3288_t1_GATCGTGAGCCAAT-1 Patient5 Monocytes nonactive 4 0.03322554 Monocytes_4

10x_3288_t1_GATCGTGAGCGAAG-1 Patient5 Monocytes nonactive 0 0.05259654 Monocytes_0

10x_3288_t1_GATCGTGAGGTGGA-1 Patient5 Monocytes nonactive 4 0.02710671 Monocytes_4

10x_3288_t1_GATCGTGAGTAGGG-1 Patient5 Monocytes nonactive 4 0.03382791 Monocytes_4

10x_3288_t1_GATCGTGATCAGGT-1 Patient5 Monocytes active 0 0.11828673 Monocytes_0

10x_3288_t1_GATCGTGATGCTCC-1 Patient5 Monocytes nonactive 0 0.05069431 Monocytes_0

10x_3288_t1_GATCGTGATTGCGA-1 Patient5 Progenitors active 1 0.16600089 Progenitors_1

10x_3288_t1_GATCTACTAAAAGC-1 Patient5 Progenitors nonactive 1 0.11147042 Progenitors_1

10x_3288_t1_GATCTACTAAAGCA-1 Patient5 Monocytes nonactive 0 0.0151861 Monocytes_0

10x_3288_t1_GATCTACTCACTGA-1 Patient5 Monocytes nonactive 4 0.09118001 Monocytes_4

10x_3288_t1_GATCTACTCCATAG-1 Patient5 Progenitors active 1 0.15986621 Progenitors_1

10x_3288_t1_GATCTACTCCTTTA-1 Patient5 Monocytes nonactive 0 0.07551836
Monocytes_0
10x_3288_t1_GATCTACTCTAGTG-1Patient5 Monocytes nonactive 4 0.06128337
Monocytes_4
10x_3288_t1_GATCTACTCTGTCC-1Patient5 Monocytes nonactive 0 0.04795194
Monocytes_0
10x_3288_t1_GATCTACTTTCGGA-1 Patient5 Monocytes nonactive 0 0.02366686
Monocytes_0
10x_3288_t1_GATCTTACAGTTCG-1 Patient5 Monocytes nonactive 8 0.06179063
Monocytes_8
10x_3288_t1_GATCTTACGAGAGC-1 Patient5 Monocytes nonactive 4 0.03046731
Monocytes_4
10x_3288_t1_GATCTTTGCTTATC-1 Patient5 Monocytes nonactive 0 0.06253567
Monocytes_0
10x_3288_t1_GATCTTTGGCTACA-1 Patient5 Monocytes nonactive 4 0.03143428
Monocytes_4
10x_3288_t1_GATCTTTGGTCTGA-1 Patient5 Progenitors nonactive 6 0.10207026
Progenitors_6
10x_3288_t1_GATCTTTGGTGAGG-1 Patient5 Monocytes nonactive 13 0.02645679
Monocytes_13
10x_3288_t1_GATCTTTGTGGGAG-1 Patient5 Progenitors active 1 0.16420963
Progenitors_1
10x_3288_t1_GATGACACTTCTGT-1 Patient5 Progenitors active 1 0.12400926
Progenitors_1
10x_3288_t1_GATGCAACAGGAGC-1 Patient5 Monocytes nonactive 0 0.00917824
Monocytes_0
10x_3288_t1_GATGCAACCTCCCA-1 Patient5 Dendritic cells nonactive 10 0.10554182
Dendritic cells_10
10x_3288_t1_GATGCATGAAGAGT-1 Patient5 Progenitors active 1 0.17462431
Progenitors_1
10x_3288_t1_GATGCATGCAGCTA-1 Patient5 Monocytes nonactive 0 0.03967726
Monocytes_0
10x_3288_t1_GATGCATGCTTGCC-1 Patient5 Monocytes nonactive 8 0.04359267

Monocytes_8
10x_3288_t1_GATGCATGGGTCTA-1 Patient5 Progenitors active 1 0.1349312
Progenitors_1
10x_3288_t1_GATGCCCTACCCTC-1 Patient5 Monocytes nonactive 4 0.04880794
Monocytes_4
10x_3288_t1_GATGCCCTGCAGAG-1 Patient5 Progenitors active 1 0.14147803
Progenitors_1
10x_3288_t1_GATGCCCTTATGCG-1 Patient5 Monocytes nonactive 0 0.07144442
Monocytes_0
10x_3288_t1_GATTACCTACCACA-1 Patient5 Monocytes nonactive 4 0.03674466
Monocytes_4
10x_3288_t1_GATTACCTCGTTAG-1Patient5 Progenitors active 1 0.15333524
Progenitors_1
10x_3288_t1_GATTACCTTATCGG-1 Patient5 Progenitors nonactive 6 0.11441887
Progenitors_6
10x_3288_t1_GATTACCTTCAAGC-1 Patient5 Progenitors active 1 0.16248177
Progenitors_1
10x_3288_t1_GATTCGGATTGTGG-1 Patient5 Monocytes nonactive 0 0.09192505
Monocytes_0
10x_3288_t1_GATTCTACAACAGA-1 Patient5 Progenitors nonactive 6 0.11525902
Progenitors_6
10x_3288_t1_GATTCTACGCGATT-1 Patient5 Monocytes nonactive 0 0.05915922
Monocytes_0
10x_3288_t1_GATTCTACTTGTGG-1 Patient5 Progenitors active 1 0.12941475
Progenitors_1
10x_3288_t1_GATTCTTGTTCTCA-1 Patient5 Monocytes nonactive 4 0.02296937
Monocytes_4
10x_3288_t1_GATTGGACCGTCTC-1 Patient5 Monocytes nonactive 8 0.06822649
Monocytes_8
10x_3288_t1_GATTGGTGACGTTG-1 Patient5 Monocytes nonactive 0 0.06602308
Monocytes_0
10x_3288_t1_GATTGGTGAGTGCT-1 Patient5 Progenitors active 1 0.13060364
Progenitors_1

10x_3288_t1_GATTGGTGCGTTAG-1 Patient5 Progenitors active 1 0.17164416
Progenitors_1
10x_3288_t1_GATTGGTGCTGTAG-1 Patient5 Monocytes nonactive 4 0.03135502
Monocytes_4
10x_3288_t1_GATTGGTGTCACGA-1 Patient5 Monocytes nonactive 8 0.06900323
Monocytes_8
10x_3288_t1_GATTTAGACTGTCC-1 Patient5 Monocytes nonactive 4 0.05554499
Monocytes_4
10x_3288_t1_GATTTAGATCAGTG-1 Patient5 Monocytes nonactive 8 0.0505992
Monocytes_8
10x_3288_t1_GATTTGCTCAGTCA-1 Patient5 Dendritic cells nonactive 10 0.10611248
Dendritic cells_10
10x_3288_t1_GCAAACTGACGGAG-1 Patient5 Monocytes nonactive 4 0.00816372
Monocytes_4
10x_3288_t1_GCAAACTGCTCCAC-1 Patient5 Monocytes nonactive 4 0.06017374
Monocytes_4
10x_3288_t1_GCAAACTGGGAAAT-1 Patient5 Progenitors active 1 0.16752267
Progenitors_1
10x_3288_t1_GCAAACTGGGAGTG-1 Patient5 Monocytes nonactive 4 0.04987001
Monocytes_4
10x_3288_t1_GCAAACTGTCCAGA-1 Patient5 Monocytes nonactive 0 0.05590958
Monocytes_0
10x_3288_t1_GCAACCCTCAGATC-1 Patient5 Progenitors active 1 0.14306322
Progenitors_1
10x_3288_t1_GCAACCCTCTCCAC-1 Patient5 Monocytes nonactive 0 0.0206233
Monocytes_0
10x_3288_t1_GCAACCCTGCTATG-1 Patient5 Monocytes nonactive 0 0.07743643
Monocytes_0
10x_3288_t1_GCAACCCTTCCCGT-1 Patient5 Monocytes nonactive 4 0.03893222
Monocytes_4
10x_3288_t1_GCAACCCTTCTCAT-1Patient5 Progenitors active 1 0.12366052
Progenitors_1
10x_3288_t1_GCAACTGAATGCCA-1 Patient5 Monocytes nonactive 4 0.04533638

Monocytes_4
10x_3288_t1_GCAACTGACCTCCA-1 Patient5 Monocytes nonactive 0 0.05508528
Monocytes_0
10x_3288_t1_GCAACTGAGGAGTG-1 Patient5 Progenitors active 1 0.17722402
Progenitors_1
10x_3288_t1_GCAACTGATACTCT-1 Patient5 Progenitors active 1 0.13266438
Progenitors_1
10x_3288_t1_GCAACTGATGGAGG-1 Patient5 Monocytes nonactive 4 0.03942363
Monocytes_4
10x_3288_t1_GCAACTGATTACTC-1 Patient5 Monocytes nonactive 4 0.03086361
Monocytes_4
10x_3288_t1_GCAAGACTATGACC-1 Patient5 Monocytes nonactive 0 0.06343922
Monocytes_0
10x_3288_t1_GCAAGACTCCGTAA-1 Patient5 Progenitors nonactive 1 0.10265678
Progenitors_1
10x_3288_t1_GCAAGACTCTACCC-1 Patient5 Progenitors active 1 0.13504217
Progenitors_1
10x_3288_t1_GCAAGACTTCCCAC-1 Patient5 Progenitors active 6 0.14499715
Progenitors_6
10x_3288_t1_GCAATCGAAACAGA-1 Patient5 Monocytes nonactive 0 0.03737873
Monocytes_0
10x_3288_t1_GCAATCGACGACAT-1 Patient5 Monocytes nonactive 0 0.04352926
Monocytes_0
10x_3288_t1_GCAATCGACTATGG-1 Patient5 Monocytes nonactive 4 0.01385454
Monocytes_4
10x_3288_t1_GCAATCGACTCAGA-1 Patient5 Monocytes nonactive 16 0.08119333
Monocytes_16
10x_3288_t1_GCAATCGAGAGGGT-1 Patient5 Monocytes nonactive 0 0.05758988
Monocytes_0
10x_3288_t1_GCAATTCTACCAGT-1 Patient5 Monocytes nonactive 0 0.02055989
Monocytes_0
10x_3288_t1_GCAATTCTATCTCT-1 Patient5 Monocytes nonactive 13 0.02355589
Monocytes_13

10x_3288_t1_GCAATTCTTATCTC-1 Patient5 Progenitors nonactive 1 0.10189588 Progenitors_1
10x_3288_t1_GCACAAACACGTTG-1 Patient5 Monocytes nonactive 4 0.03135502 Monocytes_4
10x_3288_t1_GCACAAACGGTTTG-1 Patient5 Monocytes nonactive 4 0.0454949 Monocytes_4
10x_3288_t1_GCACAAACTCTCTA-1 Patient5 Progenitors active 1 0.18643396 Progenitors_1
10x_3288_t1_GCACAATGACTGGT-1 Patient5 Monocytes nonactive 0 0.03449369 Monocytes_0
10x_3288_t1_GCACAATGTTCTGT-1 Patient5 Monocytes nonactive 0 0.03606303 Monocytes_0
10x_3288_t1_GCACACCTACCTAG-1 Patient5 Progenitors active 1 0.13995625 Progenitors_1
10x_3288_t1_GCACACCTGACGTT-1 Patient5 Monocytes nonactive 8 0.02365101 Monocytes_8
10x_3288_t1_GCACACCTTTTACC-1 Patient5 Progenitors active 1 0.12844778 Progenitors_1
10x_3288_t1_GCACCACTAACAGA-1 Patient5 Progenitors nonactive 1 0.09799632 Progenitors_1
10x_3288_t1_GCACCACTGTTACG-1 Patient5 Progenitors active 1 0.17227823 Progenitors_1
10x_3288_t1_GCACCTACATGCTG-1 Patient5 Monocytes nonactive 8 0.05150276 Monocytes_8
10x_3288_t1_GCACCTACATGTCG-1 Patient5 Progenitors active 1 0.1416207 Progenitors_1
10x_3288_t1_GCACCTACCCGCTT-1 Patient5 Progenitors active 1 0.12006214 Progenitors_1
10x_3288_t1_GCACCTACCGTACA-1 Patient5 Progenitors active 1 0.19876672 Progenitors_1
10x_3288_t1_GCACCTACGGAGGT-1 Patient5 Monocytes nonactive 4 0.02423752 Monocytes_4
10x_3288_t1_GCACCTACTCAAGC-1 Patient5 Monocytes nonactive 8 0.08580623

Monocytes_8

10x_3288_t1_GCACCTACTGCACA-1 Patient5 Progenitors active 1 0.18400862 Progenitors_1

10x_3288_t1_GCACCTTGAAACGA-1 Patient5 Monocytes nonactive 4 0.04268911 Monocytes_4

10x_3288_t1_GCACCTTGCACACA-1 Patient5 Monocytes nonactive 4 0.05952381 Monocytes_4

10x_3288_t1_GCACCTTGCTCCCA-1 Patient5 Monocytes nonactive 0 0.06488174 Monocytes_0

10x_3288_t1_GCACCTTGTTTACC-1 Patient5 Monocytes nonactive 4 0.04657282 Monocytes_4

10x_3288_t1_GCACGGACAGCGTT-1 Patient5 Monocytes nonactive 0 0.04817386 Monocytes_0

10x_3288_t1_GCACGGACAGTCAC-1 Patient5 Monocytes nonactive 0 0.04455963 Monocytes_0

10x_3288_t1_GCACGGACCAGAGG-1 Patient5 Monocytes nonactive 8 0.03958214 Monocytes_8

10x_3288_t1_GCACGGACGTACGT-1 Patient5 Monocytes nonactive 0 0.05690825 Monocytes_0

10x_3288_t1_GCACGGACTAAGCC-1 Patient5 Monocytes nonactive 0 0.06597552 Monocytes_0

10x_3288_t1_GCACGGACTGTCGA-1 Patient5 Progenitors active 1 0.12223385 Progenitors_1

10x_3288_t1_GCACGGTGAGCATC-1 Patient5 Monocytes nonactive 0 0.06932027 Monocytes_0

10x_3288_t1_GCACGGTGCCAATG-1 Patient5 Monocytes nonactive 8 0.04131 Monocytes_8

10x_3288_t1_GCACGGTGGCGATT-1 Patient5 Monocytes nonactive 8 0.06355019 Monocytes_8

10x_3288_t1_GCACGGTGGGTCAT-1 Patient5 Monocytes nonactive 4 0.04631919 Monocytes_4

10x_3288_t1_GCACGGTGTCGTAG-1 Patient5 Monocytes nonactive 4 0.02493501 Monocytes_4

10x_3288_t1_GCACGGTGTGCTTT-1 Patient5 Monocytes nonactive 8 0.060253
Monocytes_8
10x_3288_t1_GCACGGTGTGTGCA-1 Patient5 Monocytes nonactive 0 0.05340498
Monocytes_0
10x_3288_t1_GCACGTCTCACACA-1 Patient5 Monocytes nonactive 4 0.05518039
Monocytes_4
10x_3288_t1_GCACTAGACTTACT-1 Patient5 Monocytes nonactive 4 0.03866274
Monocytes_4
10x_3288_t1_GCACTAGAGGTCAT-1 Patient5 Monocytes nonactive 4 0.03114894
Monocytes_4
10x_3288_t1_GCACTAGATCAAGC-1 Patient5 Monocytes nonactive 8 0.01754803
Monocytes_8
10x_3288_t1_GCACTAGATTGAGC-1 Patient5 Monocytes nonactive 4 0.04027963
Monocytes_4
10x_3288_t1_GCACTGCTAGCCTA-1 Patient5 Progenitors active 1 0.14951493
Progenitors_1
10x_3288_t1_GCACTGCTAGTCAC-1 Patient5 Progenitors nonactive 1 0.08670978
Progenitors_1
10x_3288_t1_GCACTGCTTACTCT-1Patient5 Progenitors active 1 0.18630715
Progenitors_1
10x_3288_t1_GCACTGCTTAGCCA-1 Patient5 Progenitors nonactive 5 0.07814977
Progenitors_5
10x_3288_t1_GCACTGCTTTTCAC-1 Patient5 Progenitors active 1 0.15106842
Progenitors_1
10x_3288_t1_GCAGATACATTCGG-1 Patient5 Progenitors active 6 0.13767358
Progenitors_6
10x_3288_t1_GCAGATACCAACCA-1 Patient5 Progenitors active 1 0.13169742
Progenitors_1
10x_3288_t1_GCAGATACCAGGAG-1 Patient5 Progenitors nonactive 1 0.11489443
Progenitors_1
10x_3288_t1_GCAGATACTATCTC-1Patient5 Progenitors active 1 0.13191935
Progenitors_1
10x_3288_t1_GCAGCCGACCAGTA-1 Patient5 Monocytes nonactive 4 0.02753472

Monocytes_4
10x_3288_t1_GCAGCCGACCCTCA-1 Patient5 Monocytes nonactive 4 0.01120728
Monocytes_4
10x_3288_t1_GCAGCCGATACTTC-1 Patient5 Monocytes nonactive 0 0.09384313
Monocytes_0
10x_3288_t1_GCAGCCGATCACGA-1 Patient5 Progenitors active 1 0.16726904
Progenitors_1
10x_3288_t1_GCAGCGTGGTCACA-1 Patient5 Progenitors active 1 0.17587661
Progenitors_1
10x_3288_t1_GCAGCGTGTCCAGA-1 Patient5 Progenitors active 1 0.15278042
Progenitors_1
10x_3288_t1_GCAGGCACCAGAAA-1 Patient5 Progenitors active 1 0.17270623
Progenitors_1
10x_3288_t1_GCAGGCACCAGTCA-1 Patient5 Monocytes nonactive 4 0.07144442
Monocytes_4
10x_3288_t1_GCAGGCACGGAACG-1 Patient5 Monocytes nonactive 0 0.08158963
Monocytes_0
10x_3288_t1_GCAGGGCTAGCTCA-1 Patient5 Monocytes nonactive 0 0.03783844
Monocytes_0
10x_3288_t1_GCAGGGCTTACAGC-1 Patient5 Monocytes nonactive 0 0.04834823
Monocytes_0
10x_3288_t1_GCAGTCCTGGGACA-1 Patient5 Progenitors active 1 0.185039
Progenitors_1
10x_3288_t1_GCAGTCCTGTACCA-1 Patient5 Progenitors active 1 0.16397185
Progenitors_1
10x_3288_t1_GCAGTCCTTAAAGG-1 Patient5 Monocytes nonactive 4 0.02496671
Monocytes_4
10x_3288_t1_GCAGTCCTTGCATG-1 Patient5 Monocytes nonactive 0 0.03985163
Monocytes_0
10x_3288_t1_GCAGTTGACACTTT-1 Patient5 Monocytes nonactive 0 0.03625325
Monocytes_0
10x_3288_t1_GCAGTTGACTTGAG-1 Patient5 Monocytes nonactive 0 0.02675797
Monocytes_0

10x_3288_t1_GCAGTTGAGAGGCA-1 Patient5 Progenitors active 1 0.14184262 Progenitors_1
10x_3288_t1_GCATCAGAAGATGA-1 Patient5 Progenitors active 1 0.20380762 Progenitors_1
10x_3288_t1_GCATCAGACTTCTA-1 Patient5 Monocytes nonactive 4 0.0307368 Monocytes_4
10x_3288_t1_GCATCAGATGGATC-1 Patient5 Monocytes nonactive 0 0.04654112 Monocytes_0
10x_3288_t1_GCATGATGACAGTC-1 Patient5 Monocytes nonactive 0 0.04474986 Monocytes_0
10x_3288_t1_GCATGATGCATTGG-1 Patient5 Monocytes nonactive 0 0.04421089 Monocytes_0
10x_3288_t1_GCATGTGACAATCG-1 Patient5 Progenitors active 1 0.16103925 Progenitors_1
10x_3288_t1_GCATGTGACACTGA-1 Patient5 Progenitors nonactive 1 0.09920107 Progenitors_1
10x_3288_t1_GCATGTGACAGATC-1 Patient5 Progenitors nonactive 1 0.09986684 Progenitors_1
10x_3288_t1_GCATGTGACTGTGA-1 Patient5 Monocytes nonactive 4 0.04276837 Monocytes_4
10x_3288_t1_GCATGTGAGTTGGT-1 Patient5 Progenitors active 1 0.20149325 Progenitors_1
10x_3288_t1_GCATTGGAAACCAC-1 Patient5 Monocytes nonactive 0 0.02114641 Monocytes_0
10x_3288_t1_GCATTGGAACACAC-1 Patient5 Progenitors active 1 0.16309999 Progenitors_1
10x_3288_t1_GCATTGGAACCAAC-1 Patient5 Monocytes nonactive 0 0.01312536 Monocytes_0
10x_3288_t1_GCATTGGAGTGTTG-1 Patient5 Monocytes nonactive 0 0.0480312 Monocytes_0
10x_3288_t1_GCATTGGATTACCT-1 Patient5 Monocytes nonactive 0 0.05708262 Monocytes_0
10x_3288_t1_GCATTGGATTGCAG-1 Patient5 Progenitors active 1 0.12974764

Progenitors_1

10x_3288_t1_GCCAAAACGTTCGA-1 Patient5 Progenitors active 1 0.16386088 Progenitors_1

10x_3288_t1_GCCAAAACTGGGAG-1 Patient5 Progenitors active 1 0.19093589 Progenitors_1

10x_3288_t1_GCCAAAACTTTACC-1 Patient5 Monocytes nonactive 4 0.03904318 Monocytes_4

10x_3288_t1_GCCAAATGTGGTGT-1 Patient5 Monocytes nonactive 0 0.03481073 Monocytes_0

10x_3288_t1_GCCAACCTCGATAC-1 Patient5 Monocytes nonactive 4 0.03747384 Monocytes_4

10x_3288_t1_GCCAACCTCTATGG-1 Patient5 Progenitors active 1 0.14529833 Progenitors_1

10x_3288_t1_GCCAACCTTATCTC-1 Patient5 Monocytes nonactive 4 0.02848583 Monocytes_4

10x_3288_t1_GCCACGGACTTCTA-1 Patient5 Progenitors nonactive 1 0.1125959 Progenitors_1

10x_3288_t1_GCCACGGATAAGGA-1 Patient5 Progenitors active 1 0.18080654 Progenitors_1

10x_3288_t1_GCCACGGATCATTC-1 Patient5 Monocytes nonactive 8 0.03091117 Monocytes_8

10x_3288_t1_GCCACGGATGTTCT-1 Patient5 Progenitors active 1 0.1454727 Progenitors_1

10x_3288_t1_GCCACGGATGTTTC-1 Patient5 Progenitors nonactive 1 0.11573458 Progenitors_1

10x_3288_t1_GCCACTACCACAAC-1 Patient5 Monocytes nonactive 4 0.02785175 Monocytes_4

10x_3288_t1_GCCACTACGAGACG-1 Patient5 Progenitors active 1 0.16390844 Progenitors_1

10x_3288_t1_GCCACTACTTGGTG-1 Patient5 Progenitors active 1 0.15675924 Progenitors_1

10x_3288_t1_GCCATCACAAAGTG-1 Patient5 Monocytes nonactive 0 0.06553167 Monocytes_0

10x_3288_t1_GCCATCACCCTCGT-1 Patient5 Monocytes nonactive 4 0.06611819 Monocytes_4
10x_3288_t1_GCCATCACCGAACT-1 Patient5 Monocytes nonactive 0 0.0480946 Monocytes_0
10x_3288_t1_GCCATCACGAGGGT-1 Patient5 Progenitors active 1 0.15029167 Progenitors_1
10x_3288_t1_GCCATCACGCATCA-1 Patient5 Progenitors active 1 0.15534842 Progenitors_1
10x_3288_t1_GCCATCACTTACTC-1 Patient5 Progenitors active 1 0.14431552 Progenitors_1
10x_3288_t1_GCCATGCTTACGAC-1 Patient5 Monocytes nonactive 4 0.0560998 Monocytes_4
10x_3288_t1_GCCATGCTTATCTC-1 Patient5 Monocytes nonactive 4 0.03761651 Monocytes_4
10x_3288_t1_GCCCAACTACACAC-1 Patient5 Monocytes nonactive 8 0.04812631 Monocytes_8
10x_3288_t1_GCCCAACTGTAAGA-1 Patient5 Progenitors active 1 0.15712383 Progenitors_1
10x_3288_t1_GCCCAACTTGGTAC-1 Patient5 Monocytes nonactive 4 0.03129161 Monocytes_4
10x_3288_t1_GCCCAACTTTGGTG-1 Patient5 Monocytes nonactive 4 0.05411832 Monocytes_4
10x_3288_t1_GCCCAACTTTTCTG-1 Patient5 Monocytes nonactive 0 0.05882633 Monocytes_0
10x_3288_t1_GCCCAGGAAAGGTA-1 Patient5 Monocytes nonactive 4 0.04386215 Monocytes_4
10x_3288_t1_GCCCAGGACTACCC-1 Patient5 Monocytes nonactive 0 0.03636421 Monocytes_0
10x_3288_t1_GCCCAGGAGGACTT-1 Patient5 Monocytes nonactive 0 0.05660706 Monocytes_0
10x_3288_t1_GCCCATACCAGAAA-1 Patient5 Monocytes nonactive 0 0.07245894 Monocytes_0
10x_3288_t1_GCCCATACCGTGAT-1Patient5 Monocytes nonactive 0 0.00792594

Monocytes_0

10x_3288_t1_GCCCATACGATGAA-1 Patient5 Progenitors nonactive 1 0.09641113 Progenitors_1

10x_3288_t1_GCCCATACGTTTGG-1 Patient5 Progenitors active 1 0.12091814 Progenitors_1

10x_3288_t1_GCCGACGAAAAACG-1 Patient5 Monocytes nonactive 0 0.06418426 Monocytes_0

10x_3288_t1_GCCGACGACACTCC-1 Patient5 Progenitors active 1 0.14032084 Progenitors_1

10x_3288_t1_GCCGACGACATTGG-1 Patient5 Progenitors active 1 0.16172088 Progenitors_1

10x_3288_t1_GCCGACGACCAGTA-1 Patient5 Progenitors nonactive 1 0.10839516 Progenitors_1

10x_3288_t1_GCCGACGATTGGTG-1 Patient5 Monocytes nonactive 0 0.04799949 Monocytes_0

10x_3288_t1_GCCGAGTGAGAATG-1 Patient5 Monocytes nonactive 0 0.05340498 Monocytes_0

10x_3288_t1_GCCGAGTGAGTAGA-1 Patient5 Progenitors active 1 0.14528248 Progenitors_1

10x_3288_t1_GCCGAGTGCAGAGG-1 Patient5 Monocytes nonactive 0 0.04744468 Monocytes_0

10x_3288_t1_GCCGAGTGCTAAGC-1 Patient5 Monocytes nonactive 8 0.05207343 Monocytes_8

10x_3288_t1_GCCGAGTGTTCTAC-1 Patient5 Monocytes nonactive 4 0.01707247 Monocytes_4

10x_3288_t1_GCCGGAACGCTGTA-1 Patient5 Monocytes nonactive 0 0.04413163 Monocytes_0

10x_3288_t1_GCCGGAACGGGACA-1 Patient5 Monocytes nonactive 0 0.02192315 Monocytes_0

10x_3288_t1_GCCGGAACTTCTGT-1 Patient5 Monocytes nonactive 15 0.05487921 Monocytes_15

10x_3288_t1_GCCGTACTAACGGG-1 Patient5 Monocytes nonactive 0 0.03007102 Monocytes_0

10x_3288_t1_GCCGTACTCATTTC-1 Patient5 Monocytes nonactive 0 0.08959483
Monocytes_0
10x_3288_t1_GCCGTACTTTCTCA-1 Patient5 Monocytes nonactive 4 0.03051487
Monocytes_4
10x_3288_t1_GCCTACACAAGCCT-1 Patient5 Monocytes nonactive 0 0.01616892
Monocytes_0
10x_3288_t1_GCCTACACCCACCT-1 Patient5 Monocytes nonactive 8 0.01954537
Monocytes_8
10x_3288_t1_GCCTACACCTGAGT-1 Patient5 Monocytes nonactive 4 0.06297952
Monocytes_4
10x_3288_t1_GCCTACACTGACTG-1 Patient5 Monocytes nonactive 0 0.02992835
Monocytes_0
10x_3288_t1_GCCTAGCTCGACAT-1 Patient5 Progenitors active 1 0.16300488
Progenitors_1
10x_3288_t1_GCCTCAACTCAAGC-1 Patient5 Progenitors active 1 0.14732737
Progenitors_1
10x_3288_t1_GCCTCATGACACCA-1 Patient5 Monocytes nonactive 4 0.03359013
Monocytes_4
10x_3288_t1_GCCTCATGACCGAT-1 Patient5 Progenitors active 1 0.12684674
Progenitors_1
10x_3288_t1_GCCTGACTAGACTC-1 Patient5 Monocytes nonactive 0 0.02634582
Monocytes_0
10x_3288_t1_GCCTGACTCAACTG-1 Patient5 Progenitors nonactive 6 0.07726206
Progenitors_6
10x_3288_t1_GCCTGACTGGTTCA-1 Patient5 Progenitors active 1 0.14404603
Progenitors_1
10x_3288_t1_GCCTGACTGTCTAG-1 Patient5 Progenitors active 1 0.14566293
Progenitors_1
10x_3288_t1_GCCTGACTTGCAGT-1 Patient5 Progenitors active 1 0.16095999
Progenitors_1
10x_3288_t1_GCGAAGGAACGGGA-1 Patient5 Monocytes nonactive 4 0.06472323
Monocytes_4
10x_3288_t1_GCGAAGGATGGAGG-1 Patient5 Monocytes nonactive 4 0.08214444

Monocytes_4
10x_3288_t1_GCGACTCTCGCAAT-1 Patient5 Progenitors active 1 0.16563629 Progenitors_1
10x_3288_t1_GCGACTCTGCCATA-1 Patient5 Progenitors active 1 0.17104179 Progenitors_1
10x_3288_t1_GCGACTCTGGAGGT-1 Patient5 Monocytes nonactive 15 0.09439795 Monocytes_15
10x_3288_t1_GCGAGAGAAGAAGT-1 Patient5 Progenitors active 1 0.13161816 Progenitors_1
10x_3288_t1_GCGAGAGAGGTACT-1 Patient5 Monocytes nonactive 0 0.02647264 Monocytes_0
10x_3288_t1_GCGAGCACCTACTT-1 Patient5 Monocytes nonactive 8 0.0729028 Monocytes_8
10x_3288_t1_GCGAGCACCTCCCA-1 Patient5 Monocytes nonactive 8 0.08688415 Monocytes_8
10x_3288_t1_GCGAGCACTCCTCG-1 Patient5 Progenitors active 1 0.17164416 Progenitors_1
10x_3288_t1_GCGAGCACTTTCTG-1 Patient5 Progenitors nonactive 1 0.11578213 Progenitors_1
10x_3288_t1_GCGCACGAAGTGTC-1 Patient5 Monocytes nonactive 4 0.04444867 Monocytes_4
10x_3288_t1_GCGCACGACATGAC-1 Patient5 Monocytes nonactive 0 0.0282322 Monocytes_0
10x_3288_t1_GCGCACGACCTACC-1 Patient5 Monocytes nonactive 0 0.05001268 Monocytes_0
10x_3288_t1_GCGCACGAGAATAG-1 Patient5 Progenitors active 1 0.11941221 Progenitors_1
10x_3288_t1_GCGCACGAGGGTGA-1 Patient5 Progenitors active 1 0.16633378 Progenitors_1
10x_3288_t1_GCGCACGATGTGGT-1 Patient5 Monocytes nonactive 0 0.05741551 Monocytes_0
10x_3288_t1_GCGCGAACATTTCC-1 Patient5 Monocytes nonactive 4 0.05613151 Monocytes_4

10x_3288_t1_GCGCGAACGAGAGC-1 Patient5 Monocytes nonactive 0 0.0644696 Monocytes_0
10x_3288_t1_GCGCGAACGCTACA-1 Patient5 Progenitors nonactive 1 0.11340435 Progenitors_1
10x_3288_t1_GCGCGAACTCCTAT-1 Patient5 Progenitors nonactive 6 0.11538583 Progenitors_6
10x_3288_t1_GCGCGAACTGAAGA-1 Patient5 Monocytes nonactive 4 0.06093463 Monocytes_4
10x_3288_t1_GCGCGATGATGCCA-1 Patient5 Monocytes nonactive 4 0.04138926 Monocytes_4
10x_3288_t1_GCGCGATGCCGCTT-1 Patient5 Progenitors active 1 0.15284383 Progenitors_1
10x_3288_t1_GCGCGATGTACGCA-1 Patient5 Monocytes nonactive 4 0.04108807 Monocytes_4
10x_3288_t1_GCGCGATGTAGAGA-1 Patient5 Monocytes nonactive 8 0.02831146 Monocytes_8
10x_3288_t1_GCGCGATGTCTACT-1 Patient5 Monocytes nonactive 0 0.02181219 Monocytes_0
10x_3288_t1_GCGGACTGAAGAGT-1 Patient5 Progenitors active 1 0.13799062 Progenitors_1
10x_3288_t1_GCGGACTGGCAAGG-1 Patient5 Monocytes nonactive 0 0.03947118 Monocytes_0
10x_3288_t1_GCGGACTGGGAGGT-1 Patient5 Monocytes nonactive 4 0.04914083 Monocytes_4
10x_3288_t1_GCGGACTGTTAGGC-1 Patient5 Monocytes nonactive 16 0.02696405 Monocytes_16
10x_3288_t1_GCGGAGCTCCGATA-1 Patient5 Monocytes nonactive 4 0.05161372 Monocytes_4
10x_3288_t1_GCGGAGCTCCTCGT-1 Patient5 Progenitors active 1 0.16471689 Progenitors_1
10x_3288_t1_GCGGAGCTCTAGAC-1 Patient5 Monocytes nonactive 0 0.03127576 Monocytes_0
10x_3288_t1_GCGGAGCTTGACAC-1 Patient5 Progenitors active 1 0.14599582

Progenitors_1
10x_3288_t1_GCGGAGCTTTCTTG-1 Patient5 Monocytes nonactive 8 0.04828483 Monocytes_8
10x_3288_t1_GCGGCAACAATGCC-1 Patient5 Monocytes nonactive 8 0.02886627 Monocytes_8
10x_3288_t1_GCGGCAACCACTGA-1 Patient5 Progenitors active 1 0.17112104 Progenitors_1
10x_3288_t1_GCGGCAACCTGTGA-1 Patient5 Monocytes nonactive 0 0.06312219 Monocytes_0
10x_3288_t1_GCGGCAACTCCAAG-1 Patient5 Monocytes nonactive 0 0.0274396 Monocytes_0
10x_3288_t1_GCGGCAACTGTCAG-1 Patient5 Progenitors active 1 0.14163655 Progenitors_1
10x_3288_t1_GCGGGACTATCTCT-1 Patient5 Progenitors active 1 0.18432566 Progenitors_1
10x_3288_t1_GCGGGACTATTCTC-1 Patient5 Progenitors active 1 0.11941221 Progenitors_1
10x_3288_t1_GCGTAAACAGGGTG-1 Patient5 Monocytes nonactive 4 0.05072602 Monocytes_4
10x_3288_t1_GCGTAAACCTAAGC-1 Patient5 Progenitors active 1 0.15295479 Progenitors_1
10x_3288_t1_GCGTAAACTCCTCG-1 Patient5 Monocytes nonactive 15 0.02742375 Monocytes_15
10x_3288_t1_GCGTAAACTGCTCC-1 Patient5 Monocytes nonactive 0 0.05026631 Monocytes_0
10x_3288_t1_GCGTAATGACCTGA-1 Patient5 Monocytes nonactive 4 0.05508528 Monocytes_4
10x_3288_t1_GCGTAATGCTACGA-1 Patient5 Monocytes nonactive 4 0.03087946 Monocytes_4
10x_3288_t1_GCGTAATGGACTAC-1 Patient5 Monocytes nonactive 0 0.0838723 Monocytes_0
10x_3288_t1_GCGTAATGGCTTAG-1 Patient5 Monocytes nonactive 0 0.02476064 Monocytes_0

10x_3288_t1_GCGTAATGGGAGCA-1 Patient5 Monocytes nonactive 0 0.09257498
Monocytes_0
10x_3288_t1_GCGTAATGGTACAC-1 Patient5 Monocytes nonactive 0 0.04527297
Monocytes_0
10x_3288_t1_GCGTAATGTGAGAA-1 Patient5 Monocytes nonactive 0 0.05965062
Monocytes_0
10x_3288_t1_GCGTACCTAGTCTG-1 Patient5 Monocytes nonactive 0 0.02753472
Monocytes_0
10x_3288_t1_GCGTACCTCGATAC-1 Patient5 Monocytes nonactive 0 0.04544734
Monocytes_0
10x_3288_t1_GCGTACCTGGACAG-1 Patient5 Monocytes nonactive 0 0.03138672
Monocytes_0
10x_3288_t1_GCGTATGAAAACAG-1 Patient5 Progenitors nonactive 1 0.11465665
Progenitors_1
10x_3288_t1_GCGTATGACTTGCC-1 Patient5 Monocytes nonactive 8 0.03988333
Monocytes_8
10x_3288_t1_GCGTATGAGCTACA-1 Patient5 Progenitors active 1 0.15734576
Progenitors_1
10x_3288_t1_GCGTATGATGCAGT-1 Patient5 Progenitors active 5 0.13708706
Progenitors_5
10x_3288_t1_GCGTATGATGCCAA-1 Patient5 Monocytes nonactive 0 0.07038235
Monocytes_0
10x_3288_t1_GCGTATGATGGTAC-1 Patient5 Monocytes nonactive 0 0.06020544
Monocytes_0
10x_3288_t1_GCGTATGATGGTGT-1Patient5 Monocytes nonactive 4 0.03796525
Monocytes_4
10x_3288_t1_GCTACAGAAACAGA-1 Patient5 Monocytes nonactive 4 0.0396614
Monocytes_4
10x_3288_t1_GCTACAGAACCTTT-1 Patient5 Progenitors active 6 0.12949401
Progenitors_6
10x_3288_t1_GCTACAGATTGTCT-1Patient5 Monocytes active 8 0.12714793
Monocytes_8
10x_3288_t1_GCTACCTGAGCCTA-1 Patient5 Monocytes nonactive 4 0.03160865

Monocytes_4

10x_3288_t1_GCTACCTGATACCG-1 Patient5 Progenitors nonactive 1 0.09817069 Progenitors_1

10x_3288_t1_GCTACCTGCTTGCC-1 Patient5 Monocytes nonactive 0 0.03187813 Monocytes_0

10x_3288_t1_GCTACCTGGGTAGG-1 Patient5 Progenitors active 1 0.15273286 Progenitors_1

10x_3288_t1_GCTACCTGGTTGGT-1 Patient5 Monocytes nonactive 0 0.03636421 Monocytes_0

10x_3288_t1_GCTACGCTCGTAGT-1 Patient5 Monocytes nonactive 0 0.03599962 Monocytes_0

10x_3288_t1_GCTACGCTGAGATA-1 Patient5 Monocytes nonactive 4 0.07245894 Monocytes_4

10x_3288_t1_GCTACGCTGTAAAG-1 Patient5 Monocytes nonactive 4 0.05868366 Monocytes_4

10x_3288_t1_GCTACGCTTCCTAT-1 Patient5 Monocytes nonactive 0 0.04322808 Monocytes_0

10x_3288_t1_GCTAGAACAAGATG-1 Patient5 Monocytes nonactive 0 0.03316213 Monocytes_0

10x_3288_t1_GCTAGAACCGTAAC-1 Patient5 Progenitors active 1 0.14666159 Progenitors_1

10x_3288_t1_GCTAGAACTGCACA-1 Patient5 Monocytes nonactive 4 0.07962399 Monocytes_4

10x_3288_t1_GCTAGATGGGACTT-1 Patient5 Monocytes nonactive 0 0.06551582 Monocytes_0

10x_3288_t1_GCTAGATGGGTTTG-1 Patient5 Monocytes nonactive 8 0.01889544 Monocytes_8

10x_3288_t1_GCTAGATGTGACCA-1 Patient5 Monocytes nonactive 4 0.07315643 Monocytes_4

10x_3288_t1_GCTAGATGTGCTCC-1 Patient5 Monocytes nonactive 4 0.0485226 Monocytes_4

10x_3288_t1_GCTAGATGTGTCGA-1 Patient5 Monocytes nonactive 4 0.07302961 Monocytes_4

10x_3288_t1_GCTAGATGTGTTTC-1	Patient5 Monocytes	nonactive	0	0.03462051
Monocytes_0
10x_3288_t1_GCTATACTGAATAG-1	Patient5 Monocytes	nonactive	0	0.05136009
Monocytes_0
10x_3288_t1_GCTATACTTGACCA-1	Patient5 Monocytes	nonactive	8	0.05364276
Monocytes_8
10x_3288_t1_GCTCAAGAAAACAG-1	Patient5 Monocytes	nonactive	0	0.02774079
Monocytes_0
10x_3288_t1_GCTCAAGACCTTTA-1	Patient5 Monocytes	nonactive	0	0.106271
Monocytes_0
10x_3288_t1_GCTCAAGACTCGAA-1	Patient5 Monocytes	nonactive	0	0.06824234
Monocytes_0
10x_3288_t1_GCTCAAGATGCTTT-1	Patient5 Monocytes	nonactive	8	0.04436941
Monocytes_8
10x_3288_t1_GCTCACTGAGCATC-1	Patient5 Progenitors	active	1	0.16078562
Progenitors_1
10x_3288_t1_GCTCACTGCCCTCA-1	Patient5 Progenitors	nonactive	1	0.08331748
Progenitors_1
10x_3288_t1_GCTCACTGCCGCTT-1	Patient5 Monocytes	nonactive	0	0.0923372
Monocytes_0
10x_3288_t1_GCTCACTGCCTAAG-1	Patient5 Monocytes	nonactive	0	0.04443282
Monocytes_0
10x_3288_t1_GCTCACTGGTGCTA-1	Patient5 Progenitors	active	1	0.13558113
Progenitors_1
10x_3288_t1_GCTCACTGTGCGTA-1	Patient5 Monocytes	nonactive	4	0.07236383
Monocytes_4
10x_3288_t1_GCTCAGCTACCTAG-1	Patient5 Monocytes	nonactive	0	0.06632427
Monocytes_0
10x_3288_t1_GCTCCATGAGGTTC-1	Patient5 Progenitors	active	1	0.15276457
Progenitors_1
10x_3288_t1_GCTCCATGCTGTTT-1	Patient5 Monocytes	nonactive	0	0.04157948
Monocytes_0
10x_3288_t1_GCTCCATGTATGGC-1	Patient5 Monocytes	nonactive	8	0.01402891

Monocytes_8
10x_3288_t1_GCTCGACTACGCTA-1 Patient5 Monocytes nonactive 4 0.05538647
Monocytes_4
10x_3288_t1_GCTCGACTACGTGT-1 Patient5 Monocytes nonactive 0 0.05071016
Monocytes_0
10x_3288_t1_GCTCGACTGACAGG-1 Patient5 Progenitors active 1 0.1227094
Progenitors_1
10x_3288_t1_GCTGATGAAAGGTA-1 Patient5 Progenitors active 1 0.13895758
Progenitors_1
10x_3288_t1_GCTGATGAAGTCGT-1 Patient5 Monocytes nonactive 0 0.03939192
Monocytes_0
10x_3288_t1_GCTGATGACAGATC-1 Patient5 Monocytes nonactive 8 0.0240156
Monocytes_8
10x_3288_t1_GCTGATGACGTAAC-1 Patient5 Monocytes nonactive 4 0.07572443
Monocytes_4
10x_3288_t1_GCTGATGAGGGACA-1 Patient5 Monocytes nonactive 0 0.01537632
Monocytes_0
10x_3288_t1_GCTTAACTACACTG-1 Patient5 Progenitors active 1 0.13524824
Progenitors_1
10x_3288_t1_GCTTAACTACTCTT-1 Patient5 Dendritic cells nonactive 10 0.08173229
Dendritic cells_10
10x_3288_t1_GCTTAACTGCGAGA-1 Patient5 Monocytes nonactive 0 0.01775411
Monocytes_0
10x_3288_t1_GCTTAACTGTCACA-1 Patient5 Progenitors active 1 0.12630778
Progenitors_1
10x_3288_t1_GCTTGAGATCGCCT-1 Patient5 Progenitors nonactive 1 0.1079196
Progenitors_1
10x_3288_t1_GCTTGAGATCTCGC-1 Patient5 Progenitors active 1 0.16075392
Progenitors_1
10x_3288_t1_GCTTGAGATGTGAC-1 Patient5 Progenitors nonactive 5 0.0653573
Progenitors_5
10x_3288_t1_GGAACACTACGACT-1 Patient5 Progenitors active 1 0.22265551
Progenitors_1

10x_3288_t1_GGAACACTGTTTGG-1 Patient5 Monocytes nonactive 4 0.04972735
Monocytes_4
10x_3288_t1_GGAACACTTATGCG-1 Patient5 Monocytes nonactive 4 0.03349502
Monocytes_4
10x_3288_t1_GGAACTACTACGAC-1 Patient5 Monocytes nonactive 8 0.06897153
Monocytes_8
10x_3288_t1_GGAACTACTCTTAC-1 Patient5 Monocytes nonactive 4 0.03146598
Monocytes_4
10x_3288_t1_GGAACTTGCTGAGT-1 Patient5 Monocytes nonactive 0 0.03446199
Monocytes_0
10x_3288_t1_GGAACTTGCTGATG-1 Patient5 Monocytes nonactive 0 0.0506309
Monocytes_0
10x_3288_t1_GGAACTTGTAGAGA-1 Patient5 Progenitors active 1 0.15021242
Progenitors_1
10x_3288_t1_GGAAGGACCCTCAC-1 Patient5 Monocytes nonactive 0 0.07691332
Monocytes_0
10x_3288_t1_GGAAGGTGGTTCTT-1 Patient5 Monocytes nonactive 0 0.0497432
Monocytes_0
10x_3288_t1_GGAATCTGAAGAAC-1 Patient5 Monocytes nonactive 0 0.02485575
Monocytes_0
10x_3288_t1_GGAATCTGAAGTGA-1 Patient5 Monocytes nonactive 0 0.02086107
Monocytes_0
10x_3288_t1_GGAATCTGACCATG-1 Patient5 Monocytes nonactive 8 0.01577262
Monocytes_8
10x_3288_t1_GGAATCTGCCGATA-1 Patient5 Monocytes nonactive 4 0.05167713
Monocytes_4
10x_3288_t1_GGAATCTGCGAGAG-1 Patient5 Monocytes nonactive 0 0.04311711
Monocytes_0
10x_3288_t1_GGAATCTGGCGATT-1 Patient5 Monocytes nonactive 0 0.08152622
Monocytes_0
10x_3288_t1_GGAATGCTAGCAAA-1 Patient5 Monocytes nonactive 4 0.04812631
Monocytes_4
10x_3288_t1_GGAATGCTCCAACA-1 Patient5 Progenitors nonactive 1 0.10316404

Progenitors_1

10x_3288_t1_GGAATGCTTTGGCA-1 Patient5 Monocytes nonactive 4 0.02959546 Monocytes_4

10x_3288_t1_GGACAACTACCCTC-1 Patient5 Monocytes nonactive 8 0.02217678 Monocytes_8

10x_3288_t1_GGACAACTGTTGTG-1 Patient5 Monocytes nonactive 4 0.04563756 Monocytes_4

10x_3288_t1_GGACAACTTCCGAA-1 Patient5 Progenitors nonactive 1 0.10677826 Progenitors_1

10x_3288_t1_GGACAGGAAGTGCT-1 Patient5 Monocytes nonactive 4 0.03571429 Monocytes_4

10x_3288_t1_GGACAGGAGCCCTT-1 Patient5 Monocytes nonactive 0 0.05605225 Monocytes_0

10x_3288_t1_GGACAGGATGCCAA-1 Patient5 Monocytes nonactive 4 0.05459387 Monocytes_4

10x_3288_t1_GGACATTGAACCAC-1 Patient5 Progenitors nonactive 1 0.09287617 Progenitors_1

10x_3288_t1_GGACATTGCATGAC-1 Patient5 Progenitors active 1 0.20494896 Progenitors_1

10x_3288_t1_GGACATTGCTACCC-1 Patient5 Progenitors active 1 0.14444233 Progenitors_1

10x_3288_t1_GGACATTGGTAAGA-1 Patient5 Progenitors active 1 0.16676178 Progenitors_1

10x_3288_t1_GGACATTGTCAGAC-1 Patient5 Monocytes nonactive 0 0.04929935 Monocytes_0

10x_3288_t1_GGACATTGTTCCGC-1 Patient5 Progenitors active 1 0.13166572 Progenitors_1

10x_3288_t1_GGACATTGTTGTCT-1Patient5 Monocytes nonactive 8 0.05147105 Monocytes_8

10x_3288_t1_GGACCCGACGTAAC-1 Patient5 Monocytes nonactive 0 0.04617653 Monocytes_0

10x_3288_t1_GGACCCGAGACGTT-1 Patient5 Monocytes nonactive 4 0.02338152 Monocytes_4

10x_3288_t1_GGACCCGAGTCAAC-1 Patient5 Monocytes nonactive 8 0.01987826 Monocytes_8
10x_3288_t1_GGACCCGATCTCCG-1 Patient5 Monocytes nonactive 4 0.03831399 Monocytes_4
10x_3288_t1_GGACCGTGACCATG-1 Patient5 Monocytes nonactive 0 0.05768499 Monocytes_0
10x_3288_t1_GGACCGTGCTTCCG-1 Patient5 Monocytes nonactive 4 0.03114894 Monocytes_4
10x_3288_t1_GGACCTCTCTCGAA-1 Patient5 Progenitors nonactive 1 0.08935705 Progenitors_1
10x_3288_t1_GGACCTCTGGGAGT-1 Patient5 Monocytes nonactive 13 0.03026124 Monocytes_13
10x_3288_t1_GGACCTCTTGCGTA-1 Patient5 Monocytes nonactive 8 0.04137341 Monocytes_8
10x_3288_t1_GGACGAGAGTGAGG-1 Patient5 Monocytes nonactive 0 0.03217932 Monocytes_0
10x_3288_t1_GGACGAGAGTTGGT-1 Patient5 Progenitors active 1 0.18484877 Progenitors_1
10x_3288_t1_GGACGAGATGACAC-1 Patient5 Monocytes nonactive 8 0.03539725 Monocytes_8
10x_3288_t1_GGACGAGATTCTTG-1 Patient5 Monocytes nonactive 0 0.05827151 Monocytes_0
10x_3288_t1_GGACGAGATTTCAC-1 Patient5 Progenitors active 1 0.13729313 Progenitors_1
10x_3288_t1_GGACGCACACTGTG-1 Patient5 Monocytes nonactive 0 0.03195739 Monocytes_0
10x_3288_t1_GGACGCACCTAGTG-1 Patient5 Progenitors active 1 0.13800647 Progenitors_1
10x_3288_t1_GGACGCTGAACCGT-1 Patient5 Progenitors active 1 0.14984782 Progenitors_1
10x_3288_t1_GGACGCTGCATTGG-1 Patient5 Monocytes nonactive 4 0.02182804 Monocytes_4
10x_3288_t1_GGACGCTGCGGAGA-1 Patient5 Monocytes nonactive 4 0.05096379

Monocytes_4

10x_3288_t1_GGACGCTGCTGTAG-1 Patient5 Monocytes nonactive 4 0.06069685 Monocytes_4

10x_3288_t1_GGACGCTGGAATGA-1 Patient5 Progenitors active 1 0.15273286 Progenitors_1

10x_3288_t1_GGACGCTGGAGGCA-1 Patient5 Progenitors active 1 0.14569463 Progenitors_1

10x_3288_t1_GGACGCTGTGACTG-1 Patient5 Monocytes nonactive 0 0.05681314 Monocytes_0

10x_3288_t1_GGACGCTGTGCTTT-1 Patient5 Monocytes nonactive 4 0.0665779 Monocytes_4

10x_3288_t1_GGACGCTGTTCTCA-1 Patient5 Monocytes nonactive 0 0.04333904 Monocytes_0

10x_3288_t1_GGACTATGAAGAAC-1 Patient5 Progenitors active 1 0.15098916 Progenitors_1

10x_3288_t1_GGACTATGAGCACT-1 Patient5 Monocytes nonactive 4 0.04313297 Monocytes_4

10x_3288_t1_GGACTATGCTGGTA-1 Patient5 Monocytes nonactive 0 0.08674149 Monocytes_0

10x_3288_t1_GGACTATGGCCTTC-1 Patient5 Monocytes nonactive 0 0.0522795 Monocytes_0

10x_3288_t1_GGACTATGGGCAAG-1 Patient5 Progenitors active 1 0.13050853 Progenitors_1

10x_3288_t1_GGACTATGTTGTGG-1 Patient5 Monocytes nonactive 4 0.0370934 Monocytes_4

10x_3288_t1_GGAGACGAGAGATA-1 Patient5 Progenitors active 1 0.13586646 Progenitors_1

10x_3288_t1_GGAGAGACACGCAT-1 Patient5 Monocytes nonactive 0 0.03140257 Monocytes_0

10x_3288_t1_GGAGAGACATCTTC-1 Patient5 Progenitors nonactive 1 0.10677826 Progenitors_1

10x_3288_t1_GGAGAGACTCCAGA-1 Patient5 Monocytes nonactive 15 0.08192252 Monocytes_15

10x_3288_t1_GGAGCAGACTTGCC-1 Patient5 Monocytes nonactive 0 0.0665779
Monocytes_0
10x_3288_t1_GGAGCAGAGTTCTT-1 Patient5 Progenitors nonactive 5 0.07062013
Progenitors_5
10x_3288_t1_GGAGCAGATTCAGG-1 Patient5 Monocytes nonactive 0 0.04522541
Monocytes_0
10x_3288_t1_GGAGCCACCAGGAG-1 Patient5 Monocytes nonactive 0 0.04226111
Monocytes_0
10x_3288_t1_GGAGCCACGGAAAT-1 Patient5 Monocytes nonactive 8 0.02166952
Monocytes_8
10x_3288_t1_GGAGCCACTGAAGA-1 Patient5 Progenitors active 6 0.13922706
Progenitors_6
10x_3288_t1_GGAGCGCTAAGTGA-1 Patient5 Monocytes nonactive 8 0.02591782
Monocytes_8
10x_3288_t1_GGAGCGCTCAGTCA-1 Patient5 Progenitors active 1 0.14748589
Progenitors_1
10x_3288_t1_GGAGCGCTTCAGGT-1 Patient5 Monocytes nonactive 4 0.063249
Monocytes_4
10x_3288_t1_GGAGGATGCAAAGA-1 Patient5 Monocytes nonactive 4 0.05749477
Monocytes_4
10x_3288_t1_GGAGGATGGATACC-1 Patient5 Progenitors nonactive 1 0.10292626
Progenitors_1
10x_3288_t1_GGAGGATGGGTGTT-1 Patient5 Monocytes nonactive 0 0.04753979
Monocytes_0
10x_3288_t1_GGAGGATGTAAAGG-1 Patient5 Progenitors active 1 0.12177414
Progenitors_1
10x_3288_t1_GGAGGATGTGCAAC-1 Patient5 Progenitors active 1 0.15964428
Progenitors_1
10x_3288_t1_GGAGGCCTAGCACT-1 Patient5 Progenitors nonactive 1 0.11543339
Progenitors_1
10x_3288_t1_GGAGGCCTCCCTAC-1 Patient5 Progenitors active 1 0.15547524
Progenitors_1
10x_3288_t1_GGAGGCCTTCACCC-1 Patient5 Monocytes nonactive 4 0.03801281

Monocytes_4
10x_3288_t1_GGAGGCCTTTAGGC-1 Patient5 Progenitors active 1 0.17632046
Progenitors_1
10x_3288_t1_GGAGGCCTTTCATC-1 Patient5 Monocytes nonactive 0 0.05289772
Monocytes_0
10x_3288_t1_GGAGGTGAAGGGTG-1 Patient5 Monocytes nonactive 0 0.07608902
Monocytes_0
10x_3288_t1_GGAGGTGAAGTCGT-1 Patient5 Monocytes nonactive 0 0.03430347
Monocytes_0
10x_3288_t1_GGAGGTGAAGTGTC-1 Patient5 Monocytes nonactive 8 0.05278676
Monocytes_8
10x_3288_t1_GGAGGTGACGAGTT-1 Patient5 Monocytes nonactive 15 0.03549236
Monocytes_15
10x_3288_t1_GGAGGTGACGGAGA-1 Patient5 Progenitors active 1 0.1854987
Progenitors_1
10x_3288_t1_GGAGGTGACGTGTA-1 Patient5 Progenitors nonactive 1 0.1092036
Progenitors_1
10x_3288_t1_GGAGGTGACTTTAC-1 Patient5 Monocytes nonactive 4 0.02678968
Monocytes_4
10x_3288_t1_GGAGGTGATTGTGG-1 Patient5 Monocytes nonactive 0 0.04566927
Monocytes_0
10x_3288_t1_GGAGTTACACCAGT-1 Patient5 Dendritic cells nonactive 10 0.11543339
Dendritic cells_10
10x_3288_t1_GGAGTTACTACGAC-1 Patient5 Progenitors nonactive 5 0.06553167
Progenitors_5
10x_3288_t1_GGAGTTACTGTTTC-1 Patient5 Monocytes nonactive 0 0.03011857
Monocytes_0
10x_3288_t1_GGAGTTTGCGTGTA-1 Patient5 Progenitors active 1 0.1551582
Progenitors_1
10x_3288_t1_GGAGTTTGGGTATC-1 Patient5 Progenitors active 1 0.16978949
Progenitors_1
10x_3288_t1_GGAGTTTGTTTCAC-1 Patient5 Monocytes nonactive 0 0.03382791
Monocytes_0

10x_3288_t1_GGATACTGAAGGCG-1 Patient5 Monocytes nonactive 0 0.03783844 Monocytes_0
10x_3288_t1_GGATACTGGACGGA-1 Patient5 Progenitors active 1 0.168664 Progenitors_1
10x_3288_t1_GGATACTGGTATCG-1 Patient5 Monocytes nonactive 4 0.04455963 Monocytes_4
10x_3288_t1_GGATACTGTGACTG-1 Patient5 Monocytes nonactive 0 0.05032972 Monocytes_0
10x_3288_t1_GGATACTGTGCTGA-1 Patient5 Monocytes nonactive 0 0.04260985 Monocytes_0
10x_3288_t1_GGATAGCTAGCTAC-1 Patient5 Monocytes nonactive 4 0.0451303 Monocytes_4
10x_3288_t1_GGATAGCTATGGTC-1 Patient5 Monocytes nonactive 4 0.05326232 Monocytes_4
10x_3288_t1_GGATAGCTCTATGG-1 Patient5 Monocytes nonactive 0 0.04229282 Monocytes_0
10x_3288_t1_GGATGTACAGCGTT-1 Patient5 Monocytes nonactive 8 0.0231596 Monocytes_8
10x_3288_t1_GGATGTACCAGAGG-1 Patient5 Monocytes nonactive 8 0.05031387 Monocytes_8
10x_3288_t1_GGATGTACCCTCAC-1 Patient5 Monocytes nonactive 4 0.02515693 Monocytes_4
10x_3288_t1_GGATGTACGGTGAG-1 Patient5 Progenitors active 1 0.1517342 Progenitors_1
10x_3288_t1_GGATGTACGGTGGA-1 Patient5 Monocytes nonactive 0 0.02387293 Monocytes_0
10x_3288_t1_GGATGTACGTTACG-1 Patient5 Monocytes nonactive 4 0.04568512 Monocytes_4
10x_3288_t1_GGATGTACTCCAGA-1 Patient5 Monocytes nonactive 0 0.04286348 Monocytes_0
10x_3288_t1_GGATGTACTTTCGT-1 Patient5 Monocytes nonactive 0 0.04116733 Monocytes_0
10x_3288_t1_GGATGTTGAGCCAT-1 Patient5 Monocytes nonactive 0 0.0598884

Monocytes_0
10x_3288_t1_GGATGTTGCCATGA-1 Patient5 Progenitors active 1 0.1277186
Progenitors_1
10x_3288_t1_GGATGTTGCCGAAT-1 Patient5 Progenitors active 1 0.13707121
Progenitors_1
10x_3288_t1_GGATGTTGCTCTAT-1 Patient5 Progenitors nonactive 1 0.11308731
Progenitors_1
10x_3288_t1_GGATGTTGTAGACC-1 Patient5 Monocytes nonactive 4 0.02886627
Monocytes_4
10x_3288_t1_GGATGTTGTTACCT-1 Patient5 Monocytes nonactive 4 0.03821888
Monocytes_4
10x_3288_t1_GGATTGTGTCTTTG-1 Patient5 Monocytes nonactive 4 0.05172468
Monocytes_4
10x_3288_t1_GGATTTCTCCAAGT-1 Patient5 Monocytes nonactive 0 0.02736034
Monocytes_0
10x_3288_t1_GGATTTCTGGCGAA-1 Patient5 Monocytes nonactive 4 0.03203665
Monocytes_4
10x_3288_t1_GGATTTCTGTAGGG-1 Patient5 Progenitors active 1 0.18245514
Progenitors_1
10x_3288_t1_GGCAAGGACACAAC-1 Patient5 Monocytes nonactive 4 0.09844018
Monocytes_4
10x_3288_t1_GGCAAGGACCATGA-1 Patient5 Progenitors active 1 0.12104496
Progenitors_1
10x_3288_t1_GGCAATACGCGAAG-1 Patient5 Monocytes nonactive 15 0.01667618
Monocytes_15
10x_3288_t1_GGCACGTGCCGTAA-1 Patient5 Progenitors active 1 0.1336472
Progenitors_1
10x_3288_t1_GGCACGTGGACGAG-1 Patient5 Monocytes nonactive 0 0.01834063
Monocytes_0
10x_3288_t1_GGCACGTGTACGAC-1 Patient5 Monocytes nonactive 0 0.01945026
Monocytes_0
10x_3288_t1_GGCACGTGTCCCAC-1 Patient5 Monocytes nonactive 4 0.02314374
Monocytes_4

10x_3288_t1_GGCACTCTTCACCC-1 Patient5 Progenitors active 1 0.14989538
Progenitors_1
10x_3288_t1_GGCATATGCATGGT-1 Patient5 Monocytes nonactive 8 0.01122313
Monocytes_8
10x_3288_t1_GGCATATGCCACCT-1 Patient5 Monocytes nonactive 0 0.05040898
Monocytes_0
10x_3288_t1_GGCATATGCCTCGT-1 Patient5 Monocytes nonactive 0 0.05376958
Monocytes_0
10x_3288_t1_GGCATATGGTCTGA-1 Patient5 Monocytes nonactive 4 0.08579037
Monocytes_4
10x_3288_t1_GGCATATGTCCAAG-1 Patient5 Progenitors active 1 0.13987699
Progenitors_1
10x_3288_t1_GGCATATGTGGTGT-1 Patient5 Monocytes nonactive 0 0.02656775
Monocytes_0
10x_3288_t1_GGCCACGATTCTGT-1 Patient5 Dendritic cells nonactive 10 0.10016803
Dendritic cells_10
10x_3288_t1_GGCCAGACGGAGGT-1 Patient5 Monocytes nonactive 8 0.07228457
Monocytes_8
10x_3288_t1_GGCCAGACTGGGAG-1 Patient5 Monocytes nonactive 4 0.07778518
Monocytes_4
10x_3288_t1_GGCCAGACTTAGGC-1 Patient5 Monocytes nonactive 0 0.07534399
Monocytes_0
10x_3288_t1_GGCCCAGACAAAGA-1 Patient5 Monocytes nonactive 4 0.04200748
Monocytes_4
10x_3288_t1_GGCCCAGATTCTTG-1 Patient5 Monocytes nonactive 4 0.03883711
Monocytes_4
10x_3288_t1_GGCCGAACACTAGC-1 Patient5 Monocytes nonactive 4 0.02870775
Monocytes_4
10x_3288_t1_GGCCGAACCACTCC-1 Patient5 Monocytes nonactive 0 0.01355336
Monocytes_0
10x_3288_t1_GGCCGAACCTAGTG-1 Patient5 Monocytes nonactive 0 0.05502188
Monocytes_0
10x_3288_t1_GGCCGAACTCGCCT-1 Patient5 Monocytes nonactive 0 0.0661816

Monocytes_0
10x_3288_t1_GGCGACACCGCTAA-1 Patient5 Monocytes nonactive 0 0.03167206
Monocytes_0
10x_3288_t1_GGCGACTGACAGCT-1 Patient5 Monocytes nonactive 4 0.07217361
Monocytes_4
10x_3288_t1_GGCGACTGAGGTCT-1 Patient5 Monocytes nonactive 4 0.00944772
Monocytes_4
10x_3288_t1_GGCGACTGAGTGTC-1 Patient5 Progenitors active 1 0.17351468
Progenitors_1
10x_3288_t1_GGCGACTGATCAGC-1 Patient5 Monocytes nonactive 0 0.04714349
Monocytes_0
10x_3288_t1_GGCGACTGTACAGC-1 Patient5 Progenitors active 1 0.15303405
Progenitors_1
10x_3288_t1_GGCGCATGAGATGA-1 Patient5 Progenitors active 1 0.13768943
Progenitors_1
10x_3288_t1_GGCGCATGGGGATG-1 Patient5 Progenitors nonactive 1 0.1108839
Progenitors_1
10x_3288_t1_GGCGCATGTACTGG-1 Patient5 Progenitors active 1 0.17427557
Progenitors_1
10x_3288_t1_GGCGGACTAGCATC-1 Patient5 Progenitors active 1 0.13456661
Progenitors_1
10x_3288_t1_GGCGGACTCTACCC-1 Patient5 Monocytes nonactive 4 0.06963731
Monocytes_4
10x_3288_t1_GGCGGACTCTATTC-1 Patient5 Progenitors active 1 0.1268943
Progenitors_1
10x_3288_t1_GGCGGACTTAGACC-1 Patient5 Progenitors active 1 0.17154905
Progenitors_1
10x_3288_t1_GGCTAAACATACCG-1 Patient5 Monocytes nonactive 4 0.0535635
Monocytes_4
10x_3288_t1_GGCTAAACTACTCT-1 Patient5 Monocytes nonactive 0 0.03404984
Monocytes_0
10x_3288_t1_GGCTAATGCTGGTA-1 Patient5 Progenitors active 1 0.11990362
Progenitors_1

10x_3288_t1_GGCTACCTCACTAG-1	Patient5	Monocytes	nonactive	0	0.05781181	Monocytes_0
10x_3288_t1_GGCTCACTGAGGAC-1	Patient5	Monocytes	nonactive	0	0.02249382	Monocytes_0
10x_3288_t1_GGCTCACTGGGCAA-1	Patient5	Progenitors	active	1	0.16646059	Progenitors_1
10x_3288_t1_GGCTCACTTGCAAC-1	Patient5	Monocytes	nonactive	0	0.0337645	Monocytes_0
10x_3288_t1_GGCTCACTTGGATC-1	Patient5	Monocytes	nonactive	4	0.0421343	Monocytes_4
10x_3288_t1_GGGAACGAGGAGTG-1	Patient5	Monocytes	nonactive	0	0.09198846	Monocytes_0
10x_3288_t1_GGGAACGAGTCATG-1	Patient5	Progenitors	active	6	0.13524824	Progenitors_6
10x_3288_t1_GGGAACGATCGTGA-1	Patient5	Monocytes	nonactive	4	0.03229028	Monocytes_4
10x_3288_t1_GGGAAGACATCGGT-1	Patient5	Monocytes	nonactive	4	0.04798364	Monocytes_4
10x_3288_t1_GGGAAGACCAAGCT-1	Patient5	Monocytes	nonactive	0	0.00814787	Monocytes_0
10x_3288_t1_GGGAAGACTGCCTC-1	Patient5	Monocytes	nonactive	0	0.04915668	Monocytes_0
10x_3288_t1_GGGAAGTGATTCGG-1	Patient5	Monocytes	nonactive	0	0.08300044	Monocytes_0
10x_3288_t1_GGGAAGTGTTCACT-1	Patient5	Monocytes	nonactive	4	0.05622662	Monocytes_4
10x_3288_t1_GGGAAGTGTTGGTG-1	Patient5	Progenitors	active	1	0.14819923	Progenitors_1
10x_3288_t1_GGGACCTGTTATCC-1	Patient5	Monocytes	nonactive	4	0.02430093	Monocytes_4
10x_3288_t1_GGGATGGACCTTCG-1	Patient5	Progenitors	active	1	0.15817006	Progenitors_1
10x_3288_t1_GGGATGGACGCTAA-1	Patient5	Monocytes	nonactive	0	0.02696405	

Monocytes_0
10x_3288_t1_GGGATGGAGCATCA-1 Patient5 Progenitors nonactive 1 0.10612834
Progenitors_1
10x_3288_t1_GGGATGGAGCTACA-1 Patient5 Monocytes nonactive 0 0.03246465
Monocytes_0
10x_3288_t1_GGGATGGATGAGCT-1 Patient5 Monocytes nonactive 4 0.02006848
Monocytes_4
10x_3288_t1_GGGATGGATGCTTT-1 Patient5 Monocytes nonactive 8 0.04419504
Monocytes_8
10x_3288_t1_GGGATTACGGTGTT-1 Patient5 Monocytes nonactive 0 0.02043307
Monocytes_0
10x_3288_t1_GGGATTACGTCTTT-1 Patient5 Progenitors nonactive 1 0.11166064
Progenitors_1
10x_3288_t1_GGGCAAGAAGTACC-1 Patient5 Monocytes nonactive 4 0.03176717
Monocytes_4
10x_3288_t1_GGGCAAGAATTCCT-1 Patient5 Monocytes nonactive 0 0.07889481
Monocytes_0
10x_3288_t1_GGGCAAGAGTTACG-1 Patient5 Monocytes nonactive 0 0.02539471
Monocytes_0
10x_3288_t1_GGGCACACAGGAGC-1 Patient5 Monocytes nonactive 0 0.02929427
Monocytes_0
10x_3288_t1_GGGCAGCTGGAGCA-1 Patient5 Monocytes nonactive 13 0.06380382
Monocytes_13
10x_3288_t1_GGGCAGCTGTTAGC-1 Patient5 Monocytes nonactive 0 0.04444867
Monocytes_0
10x_3288_t1_GGGCCAACCCTATT-1 Patient5 Monocytes nonactive 4 0.03828229
Monocytes_4
10x_3288_t1_GGGCCAACCGCCTT-1 Patient5 Monocytes nonactive 0 0.06646693
Monocytes_0
10x_3288_t1_GGGCCATGCATGAC-1 Patient5 Monocytes nonactive 4 0.05611566
Monocytes_4
10x_3288_t1_GGGTAACTTGTCGA-1 Patient5 Monocytes nonactive 8 0.06729123
Monocytes_8

10x_3288_t1_GGGTTAACCGAGAG-1 Patient5 Progenitors active 1 0.18516581
Progenitors_1
10x_3288_t1_GGGTTAACCTATTC-1Patient5 Monocytes nonactive 0 0.04346586
Monocytes_0
10x_3288_t1_GGGTTAACGTAGGG-1 Patient5 Monocytes nonactive 4 0.07244309
Monocytes_4
10x_3288_t1_GGGTTATGCATTGG-1 Patient5 Progenitors nonactive 1 0.11346776
Progenitors_1
10x_3288_t1_GGTAAAGAACGTAC-1 Patient5 Monocytes nonactive 4 0.05101135
Monocytes_4
10x_3288_t1_GGTAAAGACTTTAC-1 Patient5 Monocytes nonactive 4 0.06412085
Monocytes_4
10x_3288_t1_GGTAAAGATCGTTT-1 Patient5 Progenitors active 1 0.13055608
Progenitors_1
10x_3288_t1_GGTACAACACCCAA-1 Patient5 Monocytes nonactive 0 0.06064929
Monocytes_0
10x_3288_t1_GGTACAACCTAAGC-1 Patient5 Monocytes nonactive 0 0.02355589
Monocytes_0
10x_3288_t1_GGTACATGCCTTTA-1Patient5 Monocytes nonactive 0 0.06069685
Monocytes_0
10x_3288_t1_GGTACATGGACAGG-1 Patient5 Progenitors active 1 0.15151227
Progenitors_1
10x_3288_t1_GGTACATGTGGCAT-1 Patient5 Monocytes nonactive 8 0.05519625
Monocytes_8
10x_3288_t1_GGTACTGAACGACT-1 Patient5 Monocytes nonactive 4 0.01861011
Monocytes_4
10x_3288_t1_GGTACTGACGAATC-1 Patient5 Progenitors nonactive 1 0.1167808
Progenitors_1
10x_3288_t1_GGTACTGAGGTGGA-1 Patient5 Progenitors nonactive 1 0.11594065
Progenitors_1
10x_3288_t1_GGTACTGAGGTTTG-1 Patient5 Monocytes nonactive 8 0.02182804
Monocytes_8
10x_3288_t1_GGTACTGATATCGG-1 Patient5 Progenitors active 1 0.13475683

Progenitors_1

10x_3288_t1_GGTACTGATGAGGG-1 Patient5 Monocytes nonactive 8 0.04979076 Monocytes_8

10x_3288_t1_GGTAGTACCACTAG-1 Patient5 Monocytes nonactive 15 0.06955805 Monocytes_15

10x_3288_t1_GGTAGTACCAGTTG-1 Patient5 Monocytes nonactive 0 0.03767992 Monocytes_0

10x_3288_t1_GGTAGTACCTAGAC-1 Patient5 Progenitors active 1 0.14723226 Progenitors_1

10x_3288_t1_GGTAGTACGGTATC-1 Patient5 Monocytes nonactive 0 0.0758988 Monocytes_0

10x_3288_t1_GGTATCGAACCACA-1 Patient5 Monocytes nonactive 0 0.06960561 Monocytes_0

10x_3288_t1_GGTATCGAAGACTC-1 Patient5 Monocytes nonactive 8 0.0375531 Monocytes_8

10x_3288_t1_GGTATCGAATGTGC-1 Patient5 Monocytes nonactive 4 0.05281846 Monocytes_4

10x_3288_t1_GGTATCGATTTGTC-1 Patient5 Monocytes nonactive 0 0.08526726 Monocytes_0

10x_3288_t1_GGTATGACCGTTGA-1 Patient5 Monocytes nonactive 4 0.04083444 Monocytes_4

10x_3288_t1_GGTATGACTCGTTT-1 Patient5 Monocytes nonactive 4 0.02561664 Monocytes_4

10x_3288_t1_GGTCAAACAGTGCT-1 Patient5 Progenitors active 1 0.18545114 Progenitors_1

10x_3288_t1_GGTCAAACCCTCAC-1 Patient5 Monocytes nonactive 0 0.0784034 Monocytes_0

10x_3288_t1_GGTCAAACCGGGAA-1 Patient5 Monocytes nonactive 4 0.02946864 Monocytes_4

10x_3288_t1_GGTCAAACCTTCGC-1 Patient5 Monocytes nonactive 0 0.04332319 Monocytes_0

10x_3288_t1_GGTCAAACGAAGGC-1 Patient5 Monocytes nonactive 4 0.02891383 Monocytes_4

10x_3288_t1_GGTCAAACGAGATA-1 Patient5 Monocytes nonactive 0 0.04035889 Monocytes_0
10x_3288_t1_GGTCAAACTCCAGA-1 Patient5 Progenitors nonactive 1 0.08593304 Progenitors_1
10x_3288_t1_GGTCAAACTTGCGA-1 Patient5 Monocytes nonactive 0 0.04051741 Monocytes_0
10x_3288_t1_GGTGATACCAGCTA-1 Patient5 Monocytes nonactive 0 0.03404984 Monocytes_0
10x_3288_t1_GGTGATACCATACG-1 Patient5 Monocytes nonactive 4 0.04753979 Monocytes_4
10x_3288_t1_GGTGATACTCCTGC-1 Patient5 Monocytes nonactive 4 0.01770655 Monocytes_4
10x_3288_t1_GGTGGAGACAGAAA-1 Patient5 Monocytes nonactive 0 0.00806861 Monocytes_0
10x_3288_t1_GGTGGAGAGTCTTT-1 Patient5 Monocytes nonactive 4 0.02984909 Monocytes_4
10x_3288_t1_GGTTGAACACCTGA-1 Patient5 Monocytes nonactive 0 0.05001268 Monocytes_0
10x_3288_t1_GGTTGAACCAAAGA-1 Patient5 Monocytes nonactive 0 0.063249 Monocytes_0
10x_3288_t1_GGTTGAACCGAATC-1 Patient5 Monocytes nonactive 4 0.01681884 Monocytes_4
10x_3288_t1_GGTTGAACCGTTAG-1 Patient5 Progenitors nonactive 5 0.10769767 Progenitors_5
10x_3288_t1_GGTTGAACCTAGTG-1 Patient5 Monocytes nonactive 4 0.05050409 Monocytes_4
10x_3288_t1_GGTTGAACTTTCGT-1 Patient5 Monocytes nonactive 4 0.06802042 Monocytes_4
10x_3288_t1_GGTTTACTGGTGTT-1Patient5 Monocytes nonactive 0 0.06553167 Monocytes_0
10x_3288_t1_GTAACGTGACGACT-1 Patient5 Progenitors active 1 0.12220214 Progenitors_1
10x_3288_t1_GTAACGTGCACTGA-1 Patient5 Progenitors active 1 0.12687845

Progenitors_1
10x_3288_t1_GTAACGTGGTTTCT-1 Patient5 Monocytes nonactive 8 0.09579291 Monocytes_8
10x_3288_t1_GTAACGTGTCTTAC-1 Patient5 Monocytes nonactive 0 0.03208421 Monocytes_0
10x_3288_t1_GTAAGCACGTAAGA-1 Patient5 Progenitors active 1 0.12889164 Progenitors_1
10x_3288_t1_GTAAGCACGTTACG-1 Patient5 Monocytes nonactive 4 0.06765582 Monocytes_4
10x_3288_t1_GTAAGCTGCATTCT-1Patient5 Progenitors active 1 0.14171581 Progenitors_1
10x_3288_t1_GTAAGCTGCGCAAT-1 Patient5 Progenitors active 1 0.13833936 Progenitors_1
10x_3288_t1_GTAATAACCTTGAG-1 Patient5 Monocytes nonactive 4 0.04159533 Monocytes_4
10x_3288_t1_GTAATAACGTATCG-1Patient5 Monocytes nonactive 0 0.06884471 Monocytes_0
10x_3288_t1_GTACAGTGAAGCCT-1 Patient5 Progenitors nonactive 1 0.10470167 Progenitors_1
10x_3288_t1_GTACAGTGAGAGTA-1 Patient5 Progenitors active 1 0.15745672 Progenitors_1
10x_3288_t1_GTACAGTGCATGGT-1 Patient5 Monocytes nonactive 4 0.02956376 Monocytes_4
10x_3288_t1_GTACAGTGCCCACT-1 Patient5 Monocytes nonactive 0 0.02450701 Monocytes_0
10x_3288_t1_GTACAGTGTCTCGC-1 Patient5 Monocytes nonactive 0 0.02338152 Monocytes_0
10x_3288_t1_GTACAGTGTGCTGA-1 Patient5 Monocytes nonactive 0 0.01697736 Monocytes_0
10x_3288_t1_GTACAGTGTGGTTG-1 Patient5 Monocytes nonactive 4 0.05749477 Monocytes_4
10x_3288_t1_GTACAGTGTGTGGT-1 Patient5 Monocytes nonactive 0 0.03522288 Monocytes_0

10x_3288_t1_GTACAGTGTTGCGA-1 Patient5 Progenitors active 1 0.14859552 Progenitors_1
10x_3288_t1_GTACCCTGCTTGGA-1 Patient5 Progenitors active 1 0.19141145 Progenitors_1
10x_3288_t1_GTACCCTGTCCCGT-1 Patient5 Monocytes nonactive 4 0.06697419 Monocytes_4
10x_3288_t1_GTACCCTGTTTCGT-1 Patient5 Monocytes nonactive 4 0.0152178 Monocytes_4
10x_3288_t1_GTACGAACAAGGTA-1 Patient5 Monocytes nonactive 4 0.06307463 Monocytes_4
10x_3288_t1_GTACGAACTTTGTC-1 Patient5 Monocytes nonactive 4 0.06316974 Monocytes_4
10x_3288_t1_GTACGTGACTCATT-1 Patient5 Progenitors active 1 0.17570224 Progenitors_1
10x_3288_t1_GTACTACTGGGATG-1 Patient5 Progenitors active 12 0.14552026 Progenitors_12
10x_3288_t1_GTACTACTTCTACT-1 Patient5 Progenitors nonactive 1 0.09493691 Progenitors_1
10x_3288_t1_GTACTTTGACCCAA-1 Patient5 Progenitors active 1 0.14326929 Progenitors_1
10x_3288_t1_GTACTTTGAGGGTG-1 Patient5 Monocytes nonactive 0 0.0328451 Monocytes_0
10x_3288_t1_GTACTTTGATGCCA-1 Patient5 Monocytes nonactive 0 0.0459863 Monocytes_0
10x_3288_t1_GTACTTTGCATTTC-1 Patient5 Monocytes nonactive 0 0.04421089 Monocytes_0
10x_3288_t1_GTACTTTGCTGTCC-1 Patient5 Monocytes nonactive 0 0.02659945 Monocytes_0
10x_3288_t1_GTACTTTGTCTAGG-1 Patient5 Monocytes nonactive 4 0.05457802 Monocytes_4
10x_3288_t1_GTACTTTGTTCCAT-1 Patient5 Monocytes nonactive 4 0.05687655 Monocytes_4
10x_3288_t1_GTAGACTGCCTTCG-1 Patient5 Progenitors active 1 0.11830258

Progenitors_1
10x_3288_t1_GTAGACTGGTCTTT-1 Patient5 Monocytes nonactive 4 0.03406569
Monocytes_4
10x_3288_t1_GTAGCAACCGGAGA-1 Patient5 Monocytes nonactive 8 0.05836662
Monocytes_8
10x_3288_t1_GTAGCATGAAAACG-1 Patient5 Monocytes nonactive 0 0.03896392
Monocytes_0
10x_3288_t1_GTAGCATGAACAGA-1 Patient5 Monocytes nonactive 4 0.03734703
Monocytes_4
10x_3288_t1_GTAGCATGATCGTG-1 Patient5 Monocytes nonactive 0 0.05516454
Monocytes_0
10x_3288_t1_GTAGCATGCTACTT-1 Patient5 Progenitors nonactive 1 0.10195929
Progenitors_1
10x_3288_t1_GTAGCATGCTCGCT-1 Patient5 Progenitors active 1 0.17096253
Progenitors_1
10x_3288_t1_GTAGCATGGACGAG-1 Patient5 Progenitors nonactive 1 0.11047175
Progenitors_1
10x_3288_t1_GTAGCATGGCGTTA-1 Patient5 Monocytes nonactive 4 0.0497115
Monocytes_4
10x_3288_t1_GTAGCATGTGGTGT-1 Patient5 Progenitors active 1 0.16874326
Progenitors_1
10x_3288_t1_GTAGCCCTCGACAT-1 Patient5 Progenitors active 1 0.13456661
Progenitors_1
10x_3288_t1_GTAGCCCTGGAAAT-1 Patient5 Monocytes nonactive 0 0.03219517
Monocytes_0
10x_3288_t1_GTAGCCCTGTTCAG-1 Patient5 Progenitors active 6 0.13849788
Progenitors_6
10x_3288_t1_GTAGCCCTGTTTGG-1 Patient5 Monocytes nonactive 4 0.05583032
Monocytes_4
10x_3288_t1_GTAGCTGACGTGAT-1 Patient5 Monocytes nonactive 0 0.080908
Monocytes_0
10x_3288_t1_GTAGCTGACTACGA-1 Patient5 Monocytes nonactive 4 0.07851436
Monocytes_4

10x_3288_t1_GTAGCTGAGAGGAC-1 Patient5 Monocytes nonactive 0 0.04593875
Monocytes_0
10x_3288_t1_GTAGCTGAGTAAAG-1 Patient5 Monocytes nonactive 0 0.04026378
Monocytes_0
10x_3288_t1_GTAGCTGATCAGTG-1 Patient5 Monocytes nonactive 0 0.05755818
Monocytes_0
10x_3288_t1_GTAGGTACCAATCG-1 Patient5 Monocytes nonactive 4 0.05497432
Monocytes_4
10x_3288_t1_GTAGGTACTAGCCA-1 Patient5 Progenitors active 1 0.1429364
Progenitors_1
10x_3288_t1_GTAGGTACTGGTAC-1 Patient5 Progenitors active 1 0.13512143
Progenitors_1
10x_3288_t1_GTAGTCGAACCTTT-1 Patient5 Monocytes nonactive 4 0.03493754
Monocytes_4
10x_3288_t1_GTAGTCGACAACTG-1 Patient5 Monocytes nonactive 0 0.0509955
Monocytes_0
10x_3288_t1_GTAGTCGACTTCTA-1 Patient5 Progenitors active 1 0.13721387
Progenitors_1
10x_3288_t1_GTAGTCGAGCGTAT-1 Patient5 Monocytes nonactive 4 0.02899309
Monocytes_4
10x_3288_t1_GTAGTGACAAAGTG-1 Patient5 Monocytes nonactive 4 0.03417665
Monocytes_4
10x_3288_t1_GTAGTGACCGTGAT-1 Patient5 Progenitors active 1 0.13749921
Progenitors_1
10x_3288_t1_GTAGTGACCTCTAT-1 Patient5 Progenitors active 1 0.13133283
Progenitors_1
10x_3288_t1_GTAGTGACGAGCAG-1 Patient5 Monocytes nonactive 0 0.07467821
Monocytes_0
10x_3288_t1_GTAGTGTGGACAGG-1 Patient5 Monocytes nonactive 0 0.04914083
Monocytes_0
10x_3288_t1_GTAGTGTGTTCTTG-1 Patient5 Progenitors active 1 0.12767104
Progenitors_1
10x_3288_t1_GTATCACTCTGTCC-1 Patient5 Monocytes nonactive 4 0.0476032

Monocytes_4
10x_3288_t1_GTATCACTCTGTGA-1 Patient5 Monocytes nonactive 4 0.08162133
Monocytes_4
10x_3288_t1_GTATCACTGAGCTT-1Patient5 Monocytes nonactive 4 0.03347917
Monocytes_4
10x_3288_t1_GTATCACTTCACCC-1 Patient5 Progenitors active 1 0.14723226
Progenitors_1
10x_3288_t1_GTATCTACACCACA-1 Patient5 Progenitors active 1 0.14941982
Progenitors_1
10x_3288_t1_GTATGGTGAGAAGT-1 Patient5 Progenitors nonactive 1 0.10796715
Progenitors_1
10x_3288_t1_GTATGGTGATAAGG-1 Patient5 Monocytes nonactive 0 0.05559254
Monocytes_0
10x_3288_t1_GTATGGTGGCCATA-1 Patient5 Monocytes nonactive 4 0.05510113
Monocytes_4
10x_3288_t1_GTATGGTGGGCATT-1Patient5 Monocytes nonactive 0 0.10180077
Monocytes_0
10x_3288_t1_GTATTAGAAACCGT-1 Patient5 Monocytes nonactive 0 0.04869698
Monocytes_0
10x_3288_t1_GTATTAGAACCCAA-1 Patient5 Monocytes nonactive 0 0.0527075
Monocytes_0
10x_3288_t1_GTATTAGACACACA-1 Patient5 Monocytes nonactive 0 0.04419504
Monocytes_0
10x_3288_t1_GTATTAGACAGCTA-1 Patient5 Progenitors active 1 0.13134868
Progenitors_1
10x_3288_t1_GTATTAGAGCCAAT-1Patient5 Progenitors active 1 0.14919789
Progenitors_1
10x_3288_t1_GTATTAGATGGATC-1 Patient5 Progenitors active 1 0.14987953
Progenitors_1
10x_3288_t1_GTATTCACGTTCAG-1 Patient5 Monocytes nonactive 4 0.06194915
Monocytes_4
10x_3288_t1_GTATTCACTCTTCA-1 Patient5 Monocytes nonactive 0 0.04644601
Monocytes_0

10x_3288_t1_GTATTCACTTTACC-1 Patient5 Monocytes nonactive 4 0.06573775 Monocytes_4
10x_3288_t1_GTCAACGAAACGAA-1 Patient5 Monocytes nonactive 4 0.02759812 Monocytes_4
10x_3288_t1_GTCAACGATTCTAC-1 Patient5 Progenitors active 1 0.11980851 Progenitors_1
10x_3288_t1_GTCAATCTCCGAAT-1Patient5 Progenitors active 1 0.16257688 Progenitors_1
10x_3288_t1_GTCAATCTGTTGAC-1 Patient5 Progenitors active 1 0.17414875 Progenitors_1
10x_3288_t1_GTCAATCTTGCAAC-1 Patient5 Monocytes nonactive 0 0.01803944 Monocytes_0
10x_3288_t1_GTCACAGAGCATCA-1 Patient5 Monocytes nonactive 4 0.02640923 Monocytes_4
10x_3288_t1_GTCACCTGGCTTCC-1 Patient5 Monocytes nonactive 0 0.05265995 Monocytes_0
10x_3288_t1_GTCACCTGGTAAGA-1 Patient5 Monocytes nonactive 4 0.03148183 Monocytes_4
10x_3288_t1_GTCACCTGTTACCT-1Patient5 Progenitors active 1 0.14617019 Progenitors_1
10x_3288_t1_GTCATACTAAGGTA-1 Patient5 Monocytes nonactive 0 0.03294021 Monocytes_0
10x_3288_t1_GTCATACTAGACTC-1 Patient5 Monocytes nonactive 0 0.05551328 Monocytes_0
10x_3288_t1_GTCATACTATAAGG-1Patient5 Progenitors active 1 0.1475176 Progenitors_1
10x_3288_t1_GTCATACTCCTCCA-1 Patient5 Monocytes nonactive 0 0.05039313 Monocytes_0
10x_3288_t1_GTCATACTCCTGTC-1Patient5 Monocytes nonactive 0 0.02040137 Monocytes_0
10x_3288_t1_GTCATACTCTCTCG-1Patient5 Progenitors active 1 0.17004312 Progenitors_1
10x_3288_t1_GTCATACTGAGGAC-1 Patient5 Progenitors nonactive 1 0.11231057

Progenitors_1
10x_3288_t1_GTCCAAGAAGCGTT-1 Patient5 Monocytes nonactive 0 0.02392049 Monocytes_0
10x_3288_t1_GTCCAAGAATTCGG-1 Patient5 Monocytes nonactive 0 0.02165367 Monocytes_0
10x_3288_t1_GTCCAAGACCTCGT-1 Patient5 Progenitors active 1 0.14239744 Progenitors_1
10x_3288_t1_GTCCAAGACTGTTT-1 Patient5 Monocytes nonactive 4 0.02829561 Monocytes_4
10x_3288_t1_GTCCACACCCCTTG-1 Patient5 Monocytes nonactive 8 0.0851563 Monocytes_8
10x_3288_t1_GTCCACACTCTCCG-1 Patient5 Monocytes nonactive 4 0.03141843 Monocytes_4
10x_3288_t1_GTCCACACTGTCGA-1 Patient5 Progenitors active 1 0.16078562 Progenitors_1
10x_3288_t1_GTCCACACTTGCTT-1 Patient5 Monocytes nonactive 8 0.05077357 Monocytes_8
10x_3288_t1_GTCCACTGGGTAGG-1 Patient5 Monocytes nonactive 4 0.05048824 Monocytes_4
10x_3288_t1_GTCCACTGTCTCAT-1 Patient5 Progenitors active 1 0.12938304 Progenitors_1
10x_3288_t1_GTCCACTGTTCTCA-1 Patient5 Progenitors active 1 0.14284129 Progenitors_1
10x_3288_t1_GTCCACTGTTGAGC-1 Patient5 Monocytes nonactive 0 0.05311965 Monocytes_0
10x_3288_t1_GTCCAGCTTCCTGC-1 Patient5 Progenitors active 1 0.12088644 Progenitors_1
10x_3288_t1_GTCCCATGTCGCAA-1 Patient5 Progenitors active 5 0.12003044 Progenitors_5
10x_3288_t1_GTCCCATGTCTGGA-1 Patient5 Monocytes nonactive 4 0.0366654 Monocytes_4
10x_3288_t1_GTCCCATGTGTGCA-1 Patient5 Monocytes nonactive 8 0.0956661 Monocytes_8

10x_3288_t1_GTCGAATGAAACAG-1 Patient5 Monocytes nonactive 8 0.05936529 Monocytes_8
10x_3288_t1_GTCGAATGGGTATC-1 Patient5 Monocytes nonactive 8 0.0522795 Monocytes_8
10x_3288_t1_GTCGAATGGTTAGC-1 Patient5 Progenitors active 1 0.1661277 Progenitors_1
10x_3288_t1_GTCGCACTAGTGTC-1 Patient5 Monocytes nonactive 0 0.00854416 Monocytes_0
10x_3288_t1_GTCGCACTCCTCCA-1 Patient5 Monocytes nonactive 4 0.0303722 Monocytes_4
10x_3288_t1_GTCGCACTCTGGTA-1 Patient5 Monocytes nonactive 0 0.07472576 Monocytes_0
10x_3288_t1_GTCGCACTGTGCAT-1 Patient5 Monocytes nonactive 4 0.04654112 Monocytes_4
10x_3288_t1_GTCTAACTCAGCTA-1 Patient5 Monocytes nonactive 4 0.0468106 Monocytes_4
10x_3288_t1_GTCTAACTCGAACT-1 Patient5 Monocytes nonactive 0 0.02720183 Monocytes_0
10x_3288_t1_GTCTAACTCGTACA-1 Patient5 Dendritic cells nonactive 10 0.10547841 Dendritic cells_10
10x_3288_t1_GTCTAACTGCATCA-1 Patient5 Progenitors nonactive 1 0.07737303 Progenitors_1
10x_3288_t1_GTCTAGGACCGTTC-1 Patient5 Monocytes nonactive 4 0.1058113 Monocytes_4
10x_3288_t1_GTCTAGGACTCAAG-1 Patient5 Monocytes nonactive 0 0.03866274 Monocytes_0
10x_3288_t1_GTCTAGGAGCATCA-1 Patient5 Monocytes nonactive 4 0.02371441 Monocytes_4
10x_3288_t1_GTCTAGGAGGGATG-1 Patient5 Monocytes nonactive 0 0.01816625 Monocytes_0
10x_3288_t1_GTCTAGGATAAGCC-1 Patient5 Progenitors nonactive 1 0.11395917 Progenitors_1
10x_3288_t1_GTCTGAGACTACCC-1 Patient5 Progenitors nonactive 6 0.11686006

Progenitors_6
10x_3288_t1_GTGAACACGGTTAC-1 Patient5 Monocytes nonactive 0 0.07857777 Monocytes_0
10x_3288_t1_GTGAACACGGTTCA-1 Patient5 Progenitors active 1 0.17658994 Progenitors_1
10x_3288_t1_GTGACAACAAGAAC-1 Patient5 Progenitors active 1 0.13515313 Progenitors_1
10x_3288_t1_GTGACAACAGGGTG-1 Patient5 Monocytes nonactive 4 0.06434278 Monocytes_4
10x_3288_t1_GTGACAACCCCAAA-1 Patient5 Monocytes nonactive 0 0.04275252 Monocytes_0
10x_3288_t1_GTGACAACCTACGA-1 Patient5 Monocytes nonactive 0 0.03813962 Monocytes_0
10x_3288_t1_GTGACAACTGGTGT-1 Patient5 Progenitors active 1 0.19995561 Progenitors_1
10x_3288_t1_GTGACAACTTGTCT-1 Patient5 Monocytes nonactive 4 0.05595714 Monocytes_4
10x_3288_t1_GTGACCCTATAAGG-1 Patient5 Monocytes nonactive 0 0.03203665 Monocytes_0
10x_3288_t1_GTGACCCTCCCGTT-1 Patient5 Monocytes nonactive 4 0.02384123 Monocytes_4
10x_3288_t1_GTGACCCTGAGGCA-1 Patient5 Monocytes nonactive 4 0.03030879 Monocytes_4
10x_3288_t1_GTGACCCTGGGAGT-1 Patient5 Monocytes nonactive 0 0.08566356 Monocytes_0
10x_3288_t1_GTGACCCTTTACCT-1Patient5 Monocytes nonactive 13 0.08984846 Monocytes_13
10x_3288_t1_GTGAGGGAAACGTC-1 Patient5 Monocytes nonactive 4 0.05888973 Monocytes_4
10x_3288_t1_GTGAGGGACAACTG-1 Patient5 Monocytes nonactive 0 0.08709023 Monocytes_0
10x_3288_t1_GTGAGGGATCAGTG-1 Patient5 Progenitors active 1 0.17429142 Progenitors_1

10x_3288_t1_GTGAGGGATTGGTG-1	Patient5 Monocytes	nonactive	0	0.03049902
Monocytes_0
10x_3288_t1_GTGATCGAAGCTAC-1	Patient5 Progenitors	active	1	0.12782956
Progenitors_1
10x_3288_t1_GTGATTCTCTTCTA-1 Patient5 Monocytes	nonactive	0	0.04704838
Monocytes_0
10x_3288_t1_GTGCAAACACGGTT-1	Patient5 Monocytes	nonactive	0	0.04980661
Monocytes_0
10x_3288_t1_GTGCCACTGAGCAG-1	Patient5 Monocytes	nonactive	0	0.05898485
Monocytes_0
10x_3288_t1_GTGCTAGACGATAC-1	Patient5 Progenitors	active	1	0.1504819
Progenitors_1
10x_3288_t1_GTGCTAGATATTCC-1 Patient5 Monocytes	nonactive	0	0.03522288
Monocytes_0
10x_3288_t1_GTGGAGGAATCTCT-1	Patient5 Progenitors	active	1	0.17237334
Progenitors_1
10x_3288_t1_GTGGAGGACTAGTG-1	Patient5 Monocytes	nonactive	4	0.04879209
Monocytes_4
10x_3288_t1_GTGGAGGAGCCATA-1	Patient5 Monocytes	nonactive	4	0.00781498
Monocytes_4
10x_3288_t1_GTGGAGGAGTGCAT-1	Patient5 Monocytes	nonactive	4	0.04349756
Monocytes_4
10x_3288_t1_GTGGATTGAATCGC-1	Patient5 Monocytes	nonactive	4	0.0716822
Monocytes_4
10x_3288_t1_GTGGATTGTTCCGC-1	Patient5 Progenitors	active	6	0.12744912
Progenitors_6
10x_3288_t1_GTGGATTGTTGGCA-1	Patient5 Progenitors	nonactive	6	0.11322998
Progenitors_6
10x_3288_t1_GTGGTAACAGATGA-1	Patient5 Monocytes	nonactive	0	0.04455963
Monocytes_0
10x_3288_t1_GTGGTAACCACTGA-1	Patient5 Progenitors	nonactive	6	0.10130937
Progenitors_6
10x_3288_t1_GTGGTAACCGAACT-1	Patient5 Monocytes	nonactive	4	0.04370363

Monocytes_4
10x_3288_t1_GTGGTAACGCTTAG-1 Patient5 Monocytes nonactive 0 0.02759812
Monocytes_0
10x_3288_t1_GTGTACGACGACTA-1 Patient5 Monocytes nonactive 8 0.05037727
Monocytes_8
10x_3288_t1_GTGTACGACTCTCG-1 Patient5 Monocytes nonactive 0 0.01334728
Monocytes_0
10x_3288_t1_GTGTACGAGATGAA-1 Patient5 Monocytes nonactive 0 0.03850422
Monocytes_0
10x_3288_t1_GTGTACGAGGTCTA-1 Patient5 Dendritic cells nonactive 10 0.09141779
Dendritic cells_10
10x_3288_t1_GTGTACGAGTAGGG-1 Patient5 Monocytes nonactive 0 0.06477078
Monocytes_0
10x_3288_t1_GTGTAGTGTACTTC-1 Patient5 Monocytes nonactive 15 0.07019212
Monocytes_15
10x_3288_t1_GTGTAGTGTTCTGT-1Patient5 Monocytes nonactive 0 0.06423182
Monocytes_0
10x_3288_t1_GTGTATCTACCTGA-1Patient5 Monocytes nonactive 4 0.06928857
Monocytes_4
10x_3288_t1_GTGTATCTCTCAAG-1 Patient5 Monocytes nonactive 4 0.05291358
Monocytes_4
10x_3288_t1_GTGTATCTTATTCC-1 Patient5 Progenitors nonactive 1 0.08293704
Progenitors_1
10x_3288_t1_GTGTATCTTGTCTT-1 Patient5 Monocytes nonactive 0 0.04167459
Monocytes_0
10x_3288_t1_GTGTCAGAACACAC-1 Patient5 Monocytes nonactive 0 0.03143428
Monocytes_0
10x_3288_t1_GTGTCAGAAGATCC-1 Patient5 Progenitors active 1 0.14919789
Progenitors_1
10x_3288_t1_GTGTCAGAAGGTCT-1 Patient5 Monocytes nonactive 0 0.03211591
Monocytes_0
10x_3288_t1_GTGTCAGAGCAGTT-1 Patient5 Progenitors active 1 0.13745165
Progenitors_1

10x_3288_t1_GTGTCAGAGGGACA-1 Patient5 Dendritic cells active 10 0.12488111 Dendritic cells_10
10x_3288_t1_GTGTGATGACTACG-1 Patient5 Monocytes nonactive 4 0.07055672 Monocytes_4
10x_3288_t1_GTGTGATGCATTCT-1 Patient5 Monocytes nonactive 4 0.04189652 Monocytes_4
10x_3288_t1_GTGTGATGTGTGCA-1 Patient5 Progenitors active 1 0.16478029 Progenitors_1
10x_3288_t1_GTTAAAACAGATCC-1 Patient5 Monocytes nonactive 0 0.04018452 Monocytes_0
10x_3288_t1_GTTAAAACGTAAAG-1 Patient5 Progenitors nonactive 1 0.10841101 Progenitors_1
10x_3288_t1_GTTAAATGGAATAG-1 Patient5 Progenitors active 1 0.14759685 Progenitors_1
10x_3288_t1_GTTAAATGGTGCTA-1 Patient5 Progenitors active 1 0.16011984 Progenitors_1
10x_3288_t1_GTTAACCTCTTGAG-1 Patient5 Progenitors active 1 0.17170756 Progenitors_1
10x_3288_t1_GTTACGGAGTCGAT-1 Patient5 Monocytes nonactive 0 0.03325724 Monocytes_0
10x_3288_t1_GTTACGGAGTGTAC-1 Patient5 Progenitors active 1 0.16828356 Progenitors_1
10x_3288_t1_GTTACTACCCGTAA-1 Patient5 Monocytes nonactive 8 0.03937607 Monocytes_8
10x_3288_t1_GTTACTACCTGGAT-1 Patient5 Monocytes nonactive 0 0.05487921 Monocytes_0
10x_3288_t1_GTTACTACGTTAGC-1 Patient5 Progenitors active 1 0.15536428 Progenitors_1
10x_3288_t1_GTTAGGTGACGCTA-1 Patient5 Progenitors active 1 0.16420963 Progenitors_1
10x_3288_t1_GTTAGGTGACGGTT-1 Patient5 Monocytes nonactive 4 0.05289772 Monocytes_4
10x_3288_t1_GTTAGGTGAGCCTA-1 Patient5 Progenitors active 1 0.13123771

Progenitors_1
10x_3288_t1_GTTAGGTGGGTATC-1 Patient5 Monocytes nonactive 4 0.04319637
Monocytes_4
10x_3288_t1_GTTAGGTGTGGTTG-1 Patient5 Progenitors active 1 0.17903113
Progenitors_1
10x_3288_t1_GTTAGTCTTGGTTG-1 Patient5 Monocytes nonactive 0 0.03240124
Monocytes_0
10x_3288_t1_GTTAGTCTTTGACG-1 Patient5 Progenitors active 6 0.13616765
Progenitors_6
10x_3288_t1_GTTATAGAAATCGC-1 Patient5 Progenitors nonactive 1 0.11110583
Progenitors_1
10x_3288_t1_GTTATAGATGGTGT-1 Patient5 Progenitors active 1 0.15401687
Progenitors_1
10x_3288_t1_GTTATCTGAGTAGA-1 Patient5 Monocytes nonactive 0 0.04406823
Monocytes_0
10x_3288_t1_GTTATCTGCACCAA-1 Patient5 Progenitors active 1 0.12169488
Progenitors_1
10x_3288_t1_GTTATCTGCTCCCA-1 Patient5 Monocytes nonactive 0 0.04081859
Monocytes_0
10x_3288_t1_GTTATCTGGGAAAT-1 Patient5 Monocytes nonactive 8 0.03972481
Monocytes_8
10x_3288_t1_GTTATCTGGTAGCT-1 Patient5 Monocytes nonactive 4 0.0543878
Monocytes_4
10x_3288_t1_GTTATCTGGTCCTC-1 Patient5 Monocytes nonactive 4 0.06294782
Monocytes_4
10x_3288_t1_GTTATCTGTCAAGC-1 Patient5 Monocytes nonactive 4 0.07172976
Monocytes_4
10x_3288_t1_GTTATCTGTGTCGA-1 Patient5 Monocytes nonactive 4 0.09888403
Monocytes_4
10x_3288_t1_GTTATGCTAGATGA-1 Patient5 Monocytes nonactive 4 0.0488872
Monocytes_4
10x_3288_t1_GTTATGCTCTAGAC-1 Patient5 Progenitors nonactive 1 0.07491599
Progenitors_1

10x_3288_t1_GTTATGCTGGCATT-1 Patient5 Monocytes nonactive 4 0.04701668 Monocytes_4
10x_3288_t1_GTTATGCTGGGCAA-1 Patient5 Monocytes nonactive 4 0.03550821 Monocytes_4
10x_3288_t1_GTTATGCTGTTTGG-1 Patient5 Monocytes nonactive 0 0.05201002 Monocytes_0
10x_3288_t1_GTTATGCTTCCTTA-1 Patient5 Monocytes nonactive 4 0.08339674 Monocytes_4
10x_3288_t1_GTTATGCTTTGTGG-1 Patient5 Dendritic cells nonactive 10 0.09913766 Dendritic cells_10
10x_3288_t1_GTTCAACTGCATCA-1 Patient5 Monocytes nonactive 4 0.04425845 Monocytes_4
10x_3288_t1_GTTCAGGACATACG-1 Patient5 Progenitors active 1 0.18169425 Progenitors_1
10x_3288_t1_GTTCAGGACCTTTA-1 Patient5 Monocytes nonactive 0 0.06676812 Monocytes_0
10x_3288_t1_GTTCAGGATGGTAC-1 Patient5 Monocytes nonactive 8 0.03810792 Monocytes_8
10x_3288_t1_GTTCATACAGAAGT-1 Patient5 Monocytes nonactive 4 0.05969818 Monocytes_4
10x_3288_t1_GTTCATACCGCATA-1 Patient5 Monocytes nonactive 0 0.03677636 Monocytes_0
10x_3288_t1_GTTCATACCTTGTT-1 Patient5 Monocytes nonactive 0 0.04837994 Monocytes_0
10x_3288_t1_GTTCATACTGTGCA-1 Patient5 Monocytes nonactive 4 0.04371949 Monocytes_4
10x_3288_t1_GTTGACGAACGCAT-1 Patient5 Monocytes nonactive 0 0.08625008 Monocytes_0
10x_3288_t1_GTTGACGAACGTGT-1 Patient5 Monocytes nonactive 0 0.02723353 Monocytes_0
10x_3288_t1_GTTGAGTGGCTTCC-1 Patient5 Progenitors active 1 0.15243168 Progenitors_1
10x_3288_t1_GTTGAGTGGGACTT-1 Patient5 Progenitors active 1 0.16489126

Progenitors_1
10x_3288_t1_GTTGAGTGGGATTC-1 Patient5 Monocytes nonactive 4 0.03523873
Monocytes_4
10x_3288_t1_GTTGATCTCGAGTT-1Patient5 Progenitors nonactive 5 0.07841925
Progenitors_5
10x_3288_t1_GTTGGATGCGAATC-1 Patient5 Monocytes nonactive 4 0.01846744
Monocytes_4
10x_3288_t1_GTTGGATGTCGTGA-1 Patient5 Monocytes nonactive 4 0.05953966
Monocytes_4
10x_3288_t1_GTTGTACTATTCTC-1 Patient5 Monocytes nonactive 0 0.04532053
Monocytes_0
10x_3288_t1_GTTGTACTGGCATT-1Patient5 Monocytes nonactive 4 0.02352419
Monocytes_4
10x_3288_t1_GTTTAAGACTGTGA-1 Patient5 Monocytes nonactive 0 0.05497432
Monocytes_0
10x_3288_t1_GTTTAAGAGTTGTG-1 Patient5 Monocytes nonactive 0 0.02804198
Monocytes_0
10x_3288_t1_GTTTAAGATACGCA-1 Patient5 Monocytes nonactive 0 0.01704077
Monocytes_0
10x_3288_t1_GTTTAAGATCCGAA-1 Patient5 Monocytes nonactive 4 0.06064929
Monocytes_4
10x_3288_t1_GTTTAAGATCTTAC-1Patient5 Monocytes nonactive 0 0.03465221
Monocytes_0
10x_3288_t1_GTTTAAGATGGAGG-1 Patient5 Progenitors nonactive 1 0.11261176
Progenitors_1
10x_3288_t1_GTTTAAGATGGTCA-1 Patient5 Monocytes nonactive 4 0.04343415
Monocytes_4
10x_3288_t1_TAAACAACCAGGAG-1 Patient5 Monocytes nonactive 8 0.04020037
Monocytes_8
10x_3288_t1_TAAACAACCTGAGT-1 Patient5 Monocytes nonactive 4 0.03295606
Monocytes_4
10x_3288_t1_TAAACAACGCCAAT-1 Patient5 Monocytes nonactive 0 0.02017944
Monocytes_0

10x_3288_t1_TAAACAACGCTTAG-1 Patient5 Monocytes nonactive 0 0.03863103
Monocytes_0
10x_3288_t1_TAAAGACTTTCGGA-1 Patient5 Progenitors active 1 0.17104179
Progenitors_1
10x_3288_t1_TAAAGACTTTCTCA-1 Patient5 Monocytes nonactive 0 0.04023207
Monocytes_0
10x_3288_t1_TAAAGACTTTGACG-1 Patient5 Monocytes nonactive 0 0.06085537
Monocytes_0
10x_3288_t1_TAAAGTTGATAAGG-1 Patient5 Progenitors active 6 0.11987192
Progenitors_6
10x_3288_t1_TAAAGTTGCCATGA-1 Patient5 Monocytes nonactive 8 0.05752647
Monocytes_8
10x_3288_t1_TAAAGTTGCTAGAC-1 Patient5 Progenitors active 1 0.16338533
Progenitors_1
10x_3288_t1_TAAAGTTGGGGACA-1 Patient5 Monocytes nonactive 0 0.05085283
Monocytes_0
10x_3288_t1_TAAATCGAAATGCC-1 Patient5 Progenitors active 6 0.17270623
Progenitors_6
10x_3288_t1_TAAATCGAACGACT-1 Patient5 Progenitors nonactive 6 0.08623423
Progenitors_6
10x_3288_t1_TAAATCGACAGTCA-1 Patient5 Monocytes nonactive 0 0.05787521
Monocytes_0
10x_3288_t1_TAAATCGACTTGCC-1 Patient5 Monocytes nonactive 4 0.04793609
Monocytes_4
10x_3288_t1_TAAATCGAGAGGCA-1 Patient5 Monocytes nonactive 0 0.06232959
Monocytes_0
10x_3288_t1_TAAATGTGAACCAC-1 Patient5 Monocytes nonactive 0 0.05335743
Monocytes_0
10x_3288_t1_TAAATGTGCGACAT-1 Patient5 Monocytes nonactive 4 0.0813994
Monocytes_4
10x_3288_t1_TAAATGTGGTCTTT-1 Patient5 Monocytes nonactive 4 0.04031133
Monocytes_4
10x_3288_t1_TAAATGTGTGCTCC-1 Patient5 Monocytes nonactive 0 0.02388878

Monocytes_0

10x_3288_t1_TAAATGTGTTGCTT-1 Patient5 Progenitors active 1 0.14155729 Progenitors_1

10x_3288_t1_TAACAATGACACCA-1 Patient5 Progenitors active 1 0.16614355 Progenitors_1

10x_3288_t1_TAACAATGCTTGAG-1 Patient5 Monocytes nonactive 4 0.04850675 Monocytes_4

10x_3288_t1_TAACAATGTACGCA-1 Patient5 Monocytes nonactive 0 0.03691903 Monocytes_0

10x_3288_t1_TAACAATGTGACTG-1 Patient5 Monocytes nonactive 4 0.05246972 Monocytes_4

10x_3288_t1_TAACACCTAGCAAA-1 Patient5 Progenitors active 1 0.15450827 Progenitors_1

10x_3288_t1_TAACACCTCCACCT-1 Patient5 Monocytes nonactive 4 0.01718344 Monocytes_4

10x_3288_t1_TAACACCTCCCAAA-1 Patient5 Monocytes nonactive 4 0.08246148 Monocytes_4

10x_3288_t1_TAACATGAAACAGA-1 Patient5 Monocytes nonactive 4 0.03203665 Monocytes_4

10x_3288_t1_TAACATGAAGCTAC-1 Patient5 Monocytes nonactive 0 0.0547841 Monocytes_0

10x_3288_t1_TAACATGAATGCTG-1 Patient5 Monocytes nonactive 0 0.03590451 Monocytes_0

10x_3288_t1_TAACATGAGACAGG-1 Patient5 Monocytes nonactive 8 0.03668125 Monocytes_8

10x_3288_t1_TAACCGGAACTACG-1 Patient5 Progenitors active 1 0.13711876 Progenitors_1

10x_3288_t1_TAACCGGAGATACC-1 Patient5 Monocytes nonactive 0 0.04604971 Monocytes_0

10x_3288_t1_TAACCGGAGGTTTG-1 Patient5 Monocytes nonactive 4 0.03281339 Monocytes_4

10x_3288_t1_TAACCGGATGTTTC-1 Patient5 Monocytes nonactive 4 0.06309048 Monocytes_4

10x_3288_t1_TAACGTCTACCGAT-1 Patient5 Progenitors active 1 0.15309746 Progenitors_1
10x_3288_t1_TAACGTCTACTACG-1 Patient5 Monocytes nonactive 0 0.0409137 Monocytes_0
10x_3288_t1_TAACGTCTAGGGTG-1 Patient5 Monocytes nonactive 15 0.08292118 Monocytes_15
10x_3288_t1_TAACGTCTGTGAGG-1 Patient5 Monocytes nonactive 4 0.02306449 Monocytes_4
10x_3288_t1_TAACGTCTGTTCTT-1 Patient5 Monocytes nonactive 0 0.05543402 Monocytes_0
10x_3288_t1_TAACGTCTGTTGAC-1 Patient5 Progenitors active 1 0.14284129 Progenitors_1
10x_3288_t1_TAACGTCTTGTCTT-1 Patient5 Monocytes nonactive 4 0.05693995 Monocytes_4
10x_3288_t1_TAACGTCTTTCCGC-1 Patient5 Progenitors active 1 0.14553611 Progenitors_1
10x_3288_t1_TAACTAGAAGCATC-1 Patient5 Monocytes nonactive 4 0.04492423 Monocytes_4
10x_3288_t1_TAACTAGACGTAGT-1 Patient5 Monocytes nonactive 0 0.05440365 Monocytes_0
10x_3288_t1_TAACTAGACTATGG-1 Patient5 Monocytes nonactive 4 0.03176717 Monocytes_4
10x_3288_t1_TAACTAGAGAAAGT-1 Patient5 Progenitors active 1 0.14185847 Progenitors_1
10x_3288_t1_TAACTAGAGGTATC-1 Patient5 Monocytes nonactive 0 0.06905079 Monocytes_0
10x_3288_t1_TAACTCACCCTCGT-1 Patient5 Progenitors active 1 0.12194851 Progenitors_1
10x_3288_t1_TAACTCACCCTTTA-1 Patient5 Monocytes nonactive 0 0.04311711 Monocytes_0
10x_3288_t1_TAACTCACCGTGTA-1 Patient5 Monocytes nonactive 0 0.07862532 Monocytes_0
10x_3288_t1_TAACTCACTCAGAC-1 Patient5 Progenitors nonactive 6 0.11570287

Progenitors_6
10x_3288_t1_TAACTCACTGAGGG-1 Patient5 Monocytes nonactive 0 0.08460148
Monocytes_0
10x_3288_t1_TAACTCACTTTCAC-1 Patient5 Monocytes nonactive 4 0.08070192
Monocytes_4
10x_3288_t1_TAACTCACTTTGTC-1 Patient5 Monocytes nonactive 4 0.0308002
Monocytes_4
10x_3288_t1_TAAGAACTGACGAG-1 Patient5 Monocytes nonactive 0 0.01891129
Monocytes_0
10x_3288_t1_TAAGAACTTATCGG-1 Patient5 Monocytes nonactive 4 0.04262571
Monocytes_4
10x_3288_t1_TAAGAGGAGATAGA-1 Patient5 Dendritic cells active 10 0.17744595
Dendritic cells_10
10x_3288_t1_TAAGAGGAGCCTTC-1 Patient5 Monocytes nonactive 0 0.03300361
Monocytes_0
10x_3288_t1_TAAGAGGATTGACG-1 Patient5 Monocytes nonactive 8 0.03728362
Monocytes_8
10x_3288_t1_TAAGATACGAACTC-1 Patient5 Progenitors nonactive 1 0.09855114
Progenitors_1
10x_3288_t1_TAAGATACGGAGTG-1 Patient5 Monocytes nonactive 4 0.04746053
Monocytes_4
10x_3288_t1_TAAGATACTGCATG-1 Patient5 Monocytes nonactive 4 0.06261493
Monocytes_4
10x_3288_t1_TAAGATACTGCTAG-1 Patient5 Monocytes nonactive 0 0.00848076
Monocytes_0
10x_3288_t1_TAAGATTGACCAGT-1 Patient5 Monocytes nonactive 0 0.05116987
Monocytes_0
10x_3288_t1_TAAGATTGACGTTG-1 Patient5 Monocytes nonactive 4 0.0497115
Monocytes_4
10x_3288_t1_TAAGATTGACTACG-1 Patient5 Progenitors active 1 0.22018261
Progenitors_1
10x_3288_t1_TAAGATTGATCGGT-1Patient5 Monocytes nonactive 8 0.05061505
Monocytes_8

10x_3288_t1_TAAGATTGCAGGAG-1 Patient5 Monocytes nonactive 0 0.04956883
Monocytes_0
10x_3288_t1_TAAGATTGCTTAGG-1 Patient5 Progenitors nonactive 1 0.09268594
Progenitors_1
10x_3288_t1_TAAGATTGGTTTGG-1 Patient5 Monocytes nonactive 8 0.03512777
Monocytes_8
10x_3288_t1_TAAGATTGTTCATC-1 Patient5 Monocytes nonactive 0 0.04232452
Monocytes_0
10x_3288_t1_TAAGCGTGATCGAC-1 Patient5 Progenitors nonactive 5 0.08158963
Progenitors_5
10x_3288_t1_TAAGCTCTTCATTC-1 Patient5 Monocytes nonactive 13 0.03379621
Monocytes_13
10x_3288_t1_TAAGCTCTTGTAGC-1 Patient5 Monocytes nonactive 0 0.02271574
Monocytes_0
10x_3288_t1_TAAGGCTGGAATAG-1 Patient5 Monocytes nonactive 0 0.00091941
Monocytes_0
10x_3288_t1_TAAGGCTGGGGCAA-1 Patient5 Monocytes nonactive 0 0.03975652
Monocytes_0
10x_3288_t1_TAAGGCTGGGTATC-1 Patient5 Monocytes nonactive 0 0.04901401
Monocytes_0
10x_3288_t1_TAAGGCTGTTCGGA-1 Patient5 Monocytes nonactive 8 0.05782766
Monocytes_8
10x_3288_t1_TAAGGCTGTTGTCT-1 Patient5 Progenitors active 1 0.18391351
Progenitors_1
10x_3288_t1_TAAGGGCTCAAGCT-1 Patient5 Monocytes nonactive 0 0.04625579
Monocytes_0
10x_3288_t1_TAAGGGCTGTAGCT-1 Patient5 Monocytes nonactive 4 0.03905903
Monocytes_4
10x_3288_t1_TAAGTCCTGGTTAC-1 Patient5 Progenitors active 1 0.1357555
Progenitors_1
10x_3288_t1_TAAGTCCTTAGAGA-1 Patient5 Monocytes nonactive 0 0.04371949
Monocytes_0
10x_3288_t1_TAAGTCCTTTGTGG-1 Patient5 Progenitors active 1 0.17273794

Progenitors_1
10x_3288_t1_TAATCCACCAGAAA-1 Patient5 Monocytes nonactive 0 0.02509353
Monocytes_0
10x_3288_t1_TAATCCACTGTGAC-1 Patient5 Monocytes nonactive 0 0.04715934
Monocytes_0
10x_3288_t1_TAATCGCTAATGCC-1 Patient5 Monocytes nonactive 8 0.06217107
Monocytes_8
10x_3288_t1_TAATCGCTACTCAG-1 Patient5 Progenitors active 1 0.16601674
Progenitors_1
10x_3288_t1_TAATGAACAAAACG-1 Patient5 Monocytes nonactive 0 0.05841418
Monocytes_0
10x_3288_t1_TAATGAACGGATCT-1 Patient5 Monocytes nonactive 4 0.03148183
Monocytes_4
10x_3288_t1_TAATGATGAGTCTG-1 Patient5 Monocytes nonactive 0 0.04311711
Monocytes_0
10x_3288_t1_TAATGATGGAGGTG-1 Patient5 Progenitors active 1 0.11814406
Progenitors_1
10x_3288_t1_TAATGATGTAACCG-1 Patient5 Monocytes nonactive 0 0.05491091
Monocytes_0
10x_3288_t1_TAATGATGTAGAAG-1 Patient5 Monocytes nonactive 4 0.06767168
Monocytes_4
10x_3288_t1_TAATGCCTATACCG-1 Patient5 Monocytes nonactive 4 0.08878638
Monocytes_4
10x_3288_t1_TAATGTGAAAAGTG-1 Patient5 Monocytes nonactive 0 0.04324393
Monocytes_0
10x_3288_t1_TAATGTGAGGCAAG-1 Patient5 Monocytes nonactive 0 0.03561917
Monocytes_0
10x_3288_t1_TAATGTGAGTTTCT-1 Patient5 Monocytes nonactive 0 0.08082874
Monocytes_0
10x_3288_t1_TAATGTGATGGGAG-1 Patient5 Monocytes nonactive 0 0.07607317
Monocytes_0
10x_3288_t1_TACAAATGACCTGA-1 Patient5 Monocytes nonactive 0 0.03217932
Monocytes_0

10x_3288_t1_TACAAATGCCGATA-1 Patient5 Monocytes nonactive 0 0.06318559 Monocytes_0
10x_3288_t1_TACAAATGTCGACA-1 Patient5 Monocytes nonactive 4 0.03286095 Monocytes_4
10x_3288_t1_TACAATGAGAGCTT-1 Patient5 Monocytes nonactive 13 0.01363262 Monocytes_13
10x_3288_t1_TACAATGATATCGG-1 Patient5 Monocytes nonactive 0 0.07218946 Monocytes_0
10x_3288_t1_TACAATGATCGCCT-1 Patient5 Monocytes nonactive 8 0.05064676 Monocytes_8
10x_3288_t1_TACACACTCGCCTT-1 Patient5 Monocytes nonactive 0 0.04276837 Monocytes_0
10x_3288_t1_TACACACTGTTTGG-1 Patient5 Monocytes nonactive 4 0.02888213 Monocytes_4
10x_3288_t1_TACATAGACCGAAT-1 Patient5 Monocytes nonactive 4 0.06098218 Monocytes_4
10x_3288_t1_TACATAGACTGATG-1 Patient5 Progenitors active 1 0.13859299 Progenitors_1
10x_3288_t1_TACATAGAGGAGTG-1 Patient5 Monocytes nonactive 4 0.07437702 Monocytes_4
10x_3288_t1_TACATAGAGGCAAG-1 Patient5 Monocytes nonactive 4 0.06148944 Monocytes_4
10x_3288_t1_TACCATTGCTAAGC-1 Patient5 Progenitors active 1 0.1357238 Progenitors_1
10x_3288_t1_TACCATTGGAGGTG-1 Patient5 Monocytes nonactive 8 0.0240473 Monocytes_8
10x_3288_t1_TACCATTGTGGTAC-1 Patient5 Monocytes nonactive 4 0.04765075 Monocytes_4
10x_3288_t1_TACCATTGTTCCGC-1 Patient5 Monocytes nonactive 4 0.09203602 Monocytes_4
10x_3288_t1_TACCGAGAACAGCT-1 Patient5 Progenitors active 6 0.15625198 Progenitors_6
10x_3288_t1_TACCGAGACGTGTA-1 Patient5 Monocytes nonactive 0 0.03414495

Monocytes_0
10x_3288_t1_TACCGAGATACTGG-1 Patient5 Monocytes nonactive 0 0.05961892
Monocytes_0
10x_3288_t1_TACCGAGATGGGAG-1 Patient5 Monocytes nonactive 4 0.04714349
Monocytes_4
10x_3288_t1_TACCGCTGAAGGGC-1 Patient5 Progenitors active 1 0.13347283
Progenitors_1
10x_3288_t1_TACCGCTGACAGTC-1 Patient5 Progenitors nonactive 1 0.11459324
Progenitors_1
10x_3288_t1_TACCGCTGACCTGA-1 Patient5 Monocytes nonactive 4 0.06692664
Monocytes_4
10x_3288_t1_TACCGCTGCCTTCG-1 Patient5 Progenitors nonactive 1 0.1168125
Progenitors_1
10x_3288_t1_TACCGCTGCTCAGA-1 Patient5 Monocytes nonactive 4 0.06619745
Monocytes_4
10x_3288_t1_TACCGCTGGAGAGC-1 Patient5 Monocytes nonactive 8 0.07287109
Monocytes_8
10x_3288_t1_TACCGCTGGGACAG-1 Patient5 Progenitors active 1 0.14934056
Progenitors_1
10x_3288_t1_TACCGCTGGTTACG-1 Patient5 Progenitors nonactive 1 0.09322491
Progenitors_1
10x_3288_t1_TACCGCTGGTTGTG-1 Patient5 Progenitors active 6 0.16097584
Progenitors_6
10x_3288_t1_TACCGGCTCATGAC-1 Patient5 Monocytes nonactive 0 0.01870522
Monocytes_0
10x_3288_t1_TACCGGCTCGGAGA-1 Patient5 Progenitors active 1 0.14325344
Progenitors_1
10x_3288_t1_TACGACGAACAGTC-1 Patient5 Monocytes nonactive 4 0.06632427
Monocytes_4
10x_3288_t1_TACGACGACAGATC-1 Patient5 Monocytes nonactive 0 0.03078435
Monocytes_0
10x_3288_t1_TACGACGACGGAGA-1 Patient5 Progenitors active 1 0.18579989
Progenitors_1

10x_3288_t1_TACGACGAGTTACG-1 Patient5 Monocytes nonactive 4 0.02999176 Monocytes_4
10x_3288_t1_TACGAGACAAGGGC-1 Patient5 Progenitors active 1 0.17552787 Progenitors_1
10x_3288_t1_TACGAGACCACTCC-1 Patient5 Monocytes nonactive 13 0.04462304 Monocytes_13
10x_3288_t1_TACGAGACGACGTT-1 Patient5 Progenitors active 1 0.15834443 Progenitors_1
10x_3288_t1_TACGAGACTATGCG-1 Patient5 Monocytes nonactive 4 0.0497115 Monocytes_4
10x_3288_t1_TACGAGTGAATCGC-1 Patient5 Monocytes nonactive 4 0.04123074 Monocytes_4
10x_3288_t1_TACGAGTGAGTAGA-1 Patient5 Monocytes nonactive 0 0.03939192 Monocytes_0
10x_3288_t1_TACGAGTGCGTTAG-1 Patient5 Monocytes nonactive 0 0.03278169 Monocytes_0
10x_3288_t1_TACGATCTCATGAC-1 Patient5 Monocytes nonactive 0 0.02613975 Monocytes_0
10x_3288_t1_TACGATCTCCTTCG-1Patient5 Progenitors active 1 0.13388498 Progenitors_1
10x_3288_t1_TACGATCTGAGCAG-1 Patient5 Monocytes nonactive 0 0.06708516 Monocytes_0
10x_3288_t1_TACGATCTGAGGAC-1 Patient5 Monocytes nonactive 0 0.00938431 Monocytes_0
10x_3288_t1_TACGCAGACACCAA-1 Patient5 Progenitors active 1 0.13727728 Progenitors_1
10x_3288_t1_TACGCAGATTGTCT-1Patient5 Progenitors active 1 0.14013062 Progenitors_1
10x_3288_t1_TACGCCACATTGGC-1 Patient5 Monocytes nonactive 0 0.04917253 Monocytes_0
10x_3288_t1_TACGCCACGTAAGA-1 Patient5 Progenitors active 1 0.14163655 Progenitors_1
10x_3288_t1_TACGCGCTGGACGA-1 Patient5 Monocytes nonactive 4 0.0784034

Monocytes_4
10x_3288_t1_TACGCGCTTTCCGC-1 Patient5 Monocytes nonactive 13 0.07770592 Monocytes_13
10x_3288_t1_TACGGAACACCAGT-1 Patient5 Progenitors nonactive 1 0.11690762 Progenitors_1
10x_3288_t1_TACGGAACCCTTAT-1 Patient5 Progenitors active 1 0.16582652 Progenitors_1
10x_3288_t1_TACGGAACGGTGGA-1 Patient5 Progenitors nonactive 1 0.10796715 Progenitors_1
10x_3288_t1_TACGGCCTACGTTG-1 Patient5 Progenitors nonactive 1 0.09159216 Progenitors_1
10x_3288_t1_TACGGCCTCTATGG-1 Patient5 Monocytes nonactive 0 0.0543561 Monocytes_0
10x_3288_t1_TACGGCCTGGTTCA-1 Patient5 Progenitors active 6 0.17058208 Progenitors_6
10x_3288_t1_TACGGCCTGTAAAG-1 Patient5 Monocytes nonactive 0 0.07735717 Monocytes_0
10x_3288_t1_TACGTACTCATTCT-1 Patient5 Monocytes nonactive 0 0.04135756 Monocytes_0
10x_3288_t1_TACGTACTTCCGTC-1 Patient5 Dendritic cells nonactive 10 0.11408598 Dendritic cells_10
10x_3288_t1_TACGTTACAAGCCT-1 Patient5 Progenitors active 1 0.13190349 Progenitors_1
10x_3288_t1_TACGTTACGAATAG-1 Patient5 Monocytes nonactive 0 0.07505865 Monocytes_0
10x_3288_t1_TACGTTACGTGTAC-1 Patient5 Monocytes nonactive 4 0.0201953 Monocytes_4
10x_3288_t1_TACGTTACTCCGAA-1 Patient5 Progenitors nonactive 1 0.06461226 Progenitors_1
10x_3288_t1_TACGTTACTTCAGG-1 Patient5 Monocytes nonactive 4 0.02832731 Monocytes_4
10x_3288_t1_TACTAAGAGTCAAC-1 Patient5 Progenitors active 6 0.13141209 Progenitors_6

10x_3288_t1_TACTAAGATGCTCC-1 Patient5 Monocytes nonactive 0 0.05925433 Monocytes_0
10x_3288_t1_TACTAAGATTGGTG-1 Patient5 Monocytes nonactive 0 0.09463572 Monocytes_0
10x_3288_t1_TACTACACACGTTG-1 Patient5 Monocytes nonactive 8 0.03354258 Monocytes_8
10x_3288_t1_TACTACACGACAGG-1 Patient5 Progenitors active 1 0.16622281 Progenitors_1
10x_3288_t1_TACTACACGTGTCA-1 Patient5 Progenitors active 6 0.13523239 Progenitors_6
10x_3288_t1_TACTACACTAGACC-1 Patient5 Monocytes nonactive 4 0.0467789 Monocytes_4
10x_3288_t1_TACTACTGAACCTG-1 Patient5 Progenitors nonactive 1 0.09656965 Progenitors_1
10x_3288_t1_TACTACTGTCACCC-1 Patient5 Monocytes nonactive 4 0.05540232 Monocytes_4
10x_3288_t1_TACTACTGTCTTAC-1 Patient5 Monocytes nonactive 4 0.03844081 Monocytes_4
10x_3288_t1_TACTACTGTTCGGA-1 Patient5 Progenitors active 1 0.13905269 Progenitors_1
10x_3288_t1_TACTCAACACCTAG-1 Patient5 Monocytes nonactive 0 0.02472893 Monocytes_0
10x_3288_t1_TACTCAACCTATTC-1 Patient5 Monocytes nonactive 0 0.04882379 Monocytes_0
10x_3288_t1_TACTCCCTTAAGGA-1 Patient5 Monocytes nonactive 4 0.03536554 Monocytes_4
10x_3288_t1_TACTCTGACTAGAC-1 Patient5 Progenitors active 1 0.13061949 Progenitors_1
10x_3288_t1_TACTGGGACTTGCC-1 Patient5 Monocytes nonactive 0 0.04308541 Monocytes_0
10x_3288_t1_TACTGGGATCGACA-1 Patient5 Monocytes nonactive 0 0.05300869 Monocytes_0
10x_3288_t1_TACTGTTGTAAAGG-1 Patient5 Progenitors active 1 0.14609093

Progenitors_1

10x_3288_t1_TACTGTTGTCATTC-1 Patient5 Monocytes nonactive 0 0.04625579 Monocytes_0

10x_3288_t1_TACTTGACAATGCC-1 Patient5 Progenitors active 1 0.15213049 Progenitors_1

10x_3288_t1_TACTTGACAGCACT-1 Patient5 Monocytes nonactive 0 0.0940492 Monocytes_0

10x_3288_t1_TACTTGACCTGTGA-1 Patient5 Monocytes nonactive 8 0.07068353 Monocytes_8

10x_3288_t1_TACTTGACGAAGGC-1 Patient5 Monocytes nonactive 4 0.0506309 Monocytes_4

10x_3288_t1_TACTTGACGTTGTG-1 Patient5 Progenitors active 1 0.16685689 Progenitors_1

10x_3288_t1_TACTTGACTCACGA-1 Patient5 Monocytes nonactive 4 0.01743707 Monocytes_4

10x_3288_t1_TACTTTCTAAACGA-1 Patient5 Dendritic cells active 10 0.12576882 Dendritic cells_10

10x_3288_t1_TACTTTCTGCATCA-1 Patient5 Monocytes nonactive 0 0.03450954 Monocytes_0

10x_3288_t1_TACTTTCTGTTGTG-1 Patient5 Monocytes nonactive 0 0.02786761 Monocytes_0

10x_3288_t1_TACTTTCTTTTCGT-1 Patient5 Progenitors active 1 0.12998542 Progenitors_1

10x_3288_t1_TAGAAACTTCCGAA-1 Patient5 Monocytes nonactive 0 0.01892714 Monocytes_0

10x_3288_t1_TAGAAACTTTTACC-1 Patient5 Progenitors nonactive 1 0.09297128 Progenitors_1

10x_3288_t1_TAGAATACGTTAGC-1 Patient5 Monocytes nonactive 4 0.05294528 Monocytes_4

10x_3288_t1_TAGAATTGACCTTT-1 Patient5 Monocytes nonactive 0 0.06424767 Monocytes_0

10x_3288_t1_TAGAATTGGCCTTC-1 Patient5 Monocytes nonactive 0 0.04830068 Monocytes_0

10x_3288_t1_TAGAATTGGTTGGT-1 Patient5 Monocytes nonactive 0 0.02853338 Monocytes_0
10x_3288_t1_TAGAATTGTGCCTC-1 Patient5 Monocytes nonactive 0 0.01509099 Monocytes_0
10x_3288_t1_TAGAGAGAACTCTT-1 Patient5 Progenitors active 1 0.14282544 Progenitors_1
10x_3288_t1_TAGAGAGAATACCG-1 Patient5 Monocytes nonactive 0 0.05357935 Monocytes_0
10x_3288_t1_TAGAGAGACCCTTG-1 Patient5 Monocytes nonactive 4 0.04161119 Monocytes_4
10x_3288_t1_TAGAGAGAGGGACA-1 Patient5 Monocytes nonactive 0 0.03863103 Monocytes_0
10x_3288_t1_TAGAGAGATGAAGA-1 Patient5 Monocytes nonactive 0 0.0433866 Monocytes_0
10x_3288_t1_TAGAGAGATGCTGA-1 Patient5 Progenitors active 1 0.15420709 Progenitors_1
10x_3288_t1_TAGAGCACAGAGTA-1 Patient5 Monocytes nonactive 0 0.03094287 Monocytes_0
10x_3288_t1_TAGAGCACCCTAAG-1 Patient5 Monocytes nonactive 8 0.03311458 Monocytes_8
10x_3288_t1_TAGATCCTGGTACT-1 Patient5 Progenitors active 1 0.14651893 Progenitors_1
10x_3288_t1_TAGATTGAGATGAA-1 Patient5 Monocytes nonactive 0 0.04601801 Monocytes_0
10x_3288_t1_TAGATTGAGGAAAT-1 Patient5 Progenitors active 1 0.1539059 Progenitors_1
10x_3288_t1_TAGATTGAGTGCAT-1 Patient5 Progenitors active 1 0.17105764 Progenitors_1
10x_3288_t1_TAGATTGATGAAGA-1 Patient5 Progenitors nonactive 1 0.11310316 Progenitors_1
10x_3288_t1_TAGCATCTGTTCGA-1 Patient5 Progenitors active 1 0.15837613 Progenitors_1
10x_3288_t1_TAGCATCTTCCTGC-1 Patient5 Monocytes nonactive 4 0.03924925

Monocytes_4
10x_3288_t1_TAGCATCTTGCATG-1Patient5 Progenitors active 1 0.12456407
Progenitors_1
10x_3288_t1_TAGCCCACATACCG-1 Patient5 Monocytes nonactive 0 0.05508528
Monocytes_0
10x_3288_t1_TAGCCCACGAATGA-1 Patient5 Monocytes nonactive 4 0.03149769
Monocytes_4
10x_3288_t1_TAGCCCACTCAGAC-1 Patient5 Monocytes nonactive 4 0.03785429
Monocytes_4
10x_3288_t1_TAGCCCTGAGGCGA-1 Patient5 Monocytes nonactive 0 0.06293196
Monocytes_0
10x_3288_t1_TAGCCGCTCCCACT-1 Patient5 Monocytes nonactive 0 0.04574853
Monocytes_0
10x_3288_t1_TAGCCGCTTTGAGC-1 Patient5 Monocytes nonactive 4 0.04150022
Monocytes_4
10x_3288_t1_TAGCGATGATTCGG-1 Patient5 Monocytes nonactive 0 0.05627417
Monocytes_0
10x_3288_t1_TAGCGATGTCGATG-1 Patient5 Monocytes nonactive 0 0.01954537
Monocytes_0
10x_3288_t1_TAGCTACTAACCAC-1 Patient5 Monocytes nonactive 0 0.07348932
Monocytes_0
10x_3288_t1_TAGCTACTGGTCTA-1 Patient5 Monocytes nonactive 0 0.06768753
Monocytes_0
10x_3288_t1_TAGCTACTTTCAGG-1 Patient5 Progenitors nonactive 6 0.10863293
Progenitors_6
10x_3288_t1_TAGCTACTTTGTGG-1 Patient5 Monocytes nonactive 4 0.03015028
Monocytes_4
10x_3288_t1_TAGGACTGTCTGGA-1 Patient5 Monocytes nonactive 4 0.02388878
Monocytes_4
10x_3288_t1_TAGGAGCTAATCGC-1 Patient5 Progenitors active 1 0.12922453
Progenitors_1
10x_3288_t1_TAGGAGCTACTTTC-1 Patient5 Monocytes nonactive 0 0.02135248
Monocytes_0

10x_3288_t1_TAGGAGCTTTGGCA-1 Patient5 Monocytes nonactive 4 0.02934183 Monocytes_4
10x_3288_t1_TAGGAGCTTTGTCT-1 Patient5 Monocytes nonactive 4 0.04169044 Monocytes_4
10x_3288_t1_TAGGCAACCCTTAT-1 Patient5 Monocytes nonactive 0 0.04321222 Monocytes_0
10x_3288_t1_TAGGCAACCTCCCA-1 Patient5 Monocytes nonactive 0 0.03571429 Monocytes_0
10x_3288_t1_TAGGCAACGACACT-1 Patient5 Progenitors active 1 0.17671676 Progenitors_1
10x_3288_t1_TAGGCAACGTCTAG-1 Patient5 Progenitors active 1 0.15449242 Progenitors_1
10x_3288_t1_TAGGCAACTCGTTT-1 Patient5 Monocytes nonactive 0 0.09466743 Monocytes_0
10x_3288_t1_TAGGCATGAATCGC-1 Patient5 Monocytes nonactive 0 0.03179887 Monocytes_0
10x_3288_t1_TAGGCATGACGGAG-1 Patient5 Monocytes nonactive 4 0.05662292 Monocytes_4
10x_3288_t1_TAGGCATGACTGGT-1 Patient5 Monocytes nonactive 4 0.05739966 Monocytes_4
10x_3288_t1_TAGGCATGGTCCTC-1 Patient5 Progenitors active 6 0.14133536 Progenitors_6
10x_3288_t1_TAGGCTGACGAATC-1 Patient5 Progenitors nonactive 1 0.10417856 Progenitors_1
10x_3288_t1_TAGGGACTCAGTTG-1 Patient5 Dendritic cells nonactive 10 0.0898009 Dendritic cells_10
10x_3288_t1_TAGGTCGACAGAGG-1 Patient5 Progenitors nonactive 1 0.10019973 Progenitors_1
10x_3288_t1_TAGGTCGACCCAAA-1 Patient5 Monocytes nonactive 0 0.08109822 Monocytes_0
10x_3288_t1_TAGGTCGAGTCGAT-1 Patient5 Monocytes nonactive 4 0 Monocytes_4
10x_3288_t1_TAGGTCGATCGCCT-1 Patient5 Progenitors active 6 0.19735591

Progenitors_6

10x_3288_t1_TAGGTGACCTCAAG-1 Patient5 Monocytes nonactive 0 0.03649103 Monocytes_0

10x_3288_t1_TAGGTGTGCACTAG-1 Patient5 Monocytes nonactive 4 0.01949781 Monocytes_4

10x_3288_t1_TAGGTGTGCTTTAC-1 Patient5 Monocytes nonactive 0 0.05335743 Monocytes_0

10x_3288_t1_TAGGTGTGGGACTT-1 Patient5 Monocytes nonactive 0 0.04078689 Monocytes_0

10x_3288_t1_TAGGTGTGGTTAGC-1 Patient5 Monocytes nonactive 4 0.0940492 Monocytes_4

10x_3288_t1_TAGGTTCTGATGAA-1 Patient5 Monocytes nonactive 4 0.02262063 Monocytes_4

10x_3288_t1_TAGTAAACCACAAC-1 Patient5 Monocytes nonactive 0 0.0480629 Monocytes_0

10x_3288_t1_TAGTAAACGAGCAG-1 Patient5 Progenitors active 1 0.17001141 Progenitors_1

10x_3288_t1_TAGTAAACGTGTCA-1 Patient5 Monocytes nonactive 4 0.03723607 Monocytes_4

10x_3288_t1_TAGTAATGAACGTC-1 Patient5 Progenitors active 1 0.16630207 Progenitors_1

10x_3288_t1_TAGTAATGCTCGAA-1 Patient5 Monocytes nonactive 0 0.08298459 Monocytes_0

10x_3288_t1_TAGTAATGGGAGGT-1 Patient5 Monocytes nonactive 0 0.05576691 Monocytes_0

10x_3288_t1_TAGTACCTAAACAG-1 Patient5 Monocytes nonactive 0 0.05040898 Monocytes_0

10x_3288_t1_TAGTACCTCACCAA-1 Patient5 Monocytes nonactive 0 0.04237208 Monocytes_0

10x_3288_t1_TAGTACCTGGACTT-1Patient5 Monocytes nonactive 0 0.03454125 Monocytes_0

10x_3288_t1_TAGTATGAAGATCC-1 Patient5 Progenitors active 1 0.12936719 Progenitors_1

10x_3288_t1_TAGTATGAATCACG-1 Patient5 Progenitors active 1 0.16565215 Progenitors_1

10x_3288_t1_TAGTATGACACCAA-1 Patient5 Progenitors nonactive 1 0.1146408 Progenitors_1

10x_3288_t1_TAGTATGAGCAGTT-1Patient5 Progenitors nonactive 1 0.09425528 Progenitors_1

10x_3288_t1_TAGTATGAGTTTGG-1 Patient5 Monocytes nonactive 0 0.06475493 Monocytes_0

10x_3288_t1_TAGTCACTTAACCG-1 Patient5 Monocytes nonactive 0 0.03869444 Monocytes_0

10x_3288_t1_TAGTCGGATAAAGG-1 Patient5 Monocytes nonactive 0 0.03314628 Monocytes_0

10x_3288_t1_TAGTCTTGAGTAGA-1 Patient5 Monocytes nonactive 0 0.09054594 Monocytes_0

10x_3288_t1_TAGTCTTGATCAGC-1 Patient5 Progenitors active 1 0.16050029 Progenitors_1

10x_3288_t1_TAGTCTTGATTCCT-1 Patient5 Progenitors active 1 0.14057447 Progenitors_1

10x_3288_t1_TAGTCTTGCCTTGC-1 Patient5 Monocytes nonactive 0 0.083904 Monocytes_0

10x_3288_t1_TAGTCTTGTCTCGC-1 Patient5 Monocytes nonactive 0 0.05556084 Monocytes_0

10x_3288_t1_TAGTCTTGTGTCGA-1 Patient5 Monocytes nonactive 4 0.05343669 Monocytes_4

10x_3288_t1_TAGTGGTGAGCATC-1 Patient5 Monocytes nonactive 0 0.04937861 Monocytes_0

10x_3288_t1_TAGTGGTGGGATCT-1 Patient5 Monocytes nonactive 4 0.05367447 Monocytes_4

10x_3288_t1_TAGTTAGAATGCTG-1 Patient5 Monocytes nonactive 0 0.07394902 Monocytes_0

10x_3288_t1_TAGTTAGACTTACT-1 Patient5 Monocytes nonactive 4 0.03628495 Monocytes_4

10x_3288_t1_TAGTTCACACGTTG-1 Patient5 Monocytes nonactive 0 0.04156363

Monocytes_0
10x_3288_t1_TAGTTCACCTGCTC-1 Patient5 Monocytes nonactive 0 0.0506309
Monocytes_0
10x_3288_t1_TAGTTGCTCACCAA-1 Patient5 Monocytes nonactive 4 0.05998351
Monocytes_4
10x_3288_t1_TAGTTGCTGACAGG-1 Patient5 Monocytes nonactive 0 0.03146598
Monocytes_0
10x_3288_t1_TAGTTGCTTGGCAT-1 Patient5 Progenitors nonactive 1 0.1121362
Progenitors_1
10x_3288_t1_TAGTTGCTTGTTTC-1 Patient5 Monocytes nonactive 4 0.04419504
Monocytes_4
10x_3288_t1_TATAAGACAGGAGC-1 Patient5 Monocytes nonactive 13 0.01673959
Monocytes_13
10x_3288_t1_TATAAGACGAACTC-1 Patient5 Monocytes nonactive 0 0.07634265
Monocytes_0
10x_3288_t1_TATAAGACGACTAC-1 Patient5 Progenitors active 1 0.13237905
Progenitors_1
10x_3288_t1_TATAAGACTGCGTA-1 Patient5 Progenitors active 6 0.12553104
Progenitors_6
10x_3288_t1_TATAAGTGCAGAAA-1 Patient5 Monocytes nonactive 4 0.04901401
Monocytes_4
10x_3288_t1_TATAAGTGCATGCA-1 Patient5 Progenitors active 1 0.1277186
Progenitors_1
10x_3288_t1_TATAAGTGGATAAG-1 Patient5 Monocytes nonactive 0 0.05234291
Monocytes_0
10x_3288_t1_TATAAGTGGTTAGC-1 Patient5 Monocytes nonactive 0 0.06530975
Monocytes_0
10x_3288_t1_TATAAGTGTCTACT-1 Patient5 Progenitors active 1 0.1513379
Progenitors_1
10x_3288_t1_TATACAGAAGTACC-1 Patient5 Monocytes nonactive 0 0.04538393
Monocytes_0
10x_3288_t1_TATACAGACAAGCT-1 Patient5 Progenitors active 1 0.15125864
Progenitors_1

10x_3288_t1_TATACAGACGAGTT-1 Patient5 Monocytes nonactive 4 0.02594953 Monocytes_4
10x_3288_t1_TATACAGAGGTACT-1 Patient5 Progenitors active 1 0.16818845 Progenitors_1
10x_3288_t1_TATACAGAGGTGGA-1 Patient5 Monocytes nonactive 0 0.02227189 Monocytes_0
10x_3288_t1_TATACAGATGCTAG-1 Patient5 Progenitors active 1 0.16287807 Progenitors_1
10x_3288_t1_TATACAGATGGTAC-1 Patient5 Monocytes nonactive 0 0.0564644 Monocytes_0
10x_3288_t1_TATACAGATTTCAC-1 Patient5 Progenitors active 1 0.15233657 Progenitors_1
10x_3288_t1_TATACCACCATGCA-1 Patient5 Progenitors nonactive 5 0.11660643 Progenitors_5
10x_3288_t1_TATACCACGTAAGA-1 Patient5 Monocytes nonactive 4 0.04817386 Monocytes_4
10x_3288_t1_TATACGCTAAGTGA-1 Patient5 Progenitors active 1 0.14480692 Progenitors_1
10x_3288_t1_TATAGATGACGTAC-1 Patient5 Monocytes nonactive 4 0.08406252 Monocytes_4
10x_3288_t1_TATAGATGCGATAC-1 Patient5 Progenitors active 1 0.13627861 Progenitors_1
10x_3288_t1_TATAGATGTAACCG-1 Patient5 Progenitors active 6 0.122646 Progenitors_6
10x_3288_t1_TATCAAGACTCAAG-1 Patient5 Monocytes nonactive 0 0.05835077 Monocytes_0
10x_3288_t1_TATCAAGATGTCGA-1 Patient5 Monocytes nonactive 4 0.0653256 Monocytes_4
10x_3288_t1_TATCACTGGCGATT-1 Patient5 Progenitors nonactive 6 0.11663813 Progenitors_6
10x_3288_t1_TATCACTGGCTATG-1 Patient5 Monocytes nonactive 13 0.05162957 Monocytes_13
10x_3288_t1_TATCAGCTCACAAC-1 Patient5 Monocytes nonactive 0 0.01358506

Monocytes_0
10x_3288_t1_TATCAGCTGAGCAG-1 Patient5 Monocytes nonactive 4 0.08834253 Monocytes_4
10x_3288_t1_TATCAGCTTGCGTA-1Patient5 Progenitors active 1 0.1753535 Progenitors_1
10x_3288_t1_TATCCAACAAGTAG-1 Patient5 Monocytes nonactive 4 0.03156109 Monocytes_4
10x_3288_t1_TATCCAACCACTCC-1 Patient5 Monocytes nonactive 8 0.0256959 Monocytes_8
10x_3288_t1_TATCCAACGACAGG-1 Patient5 Progenitors active 1 0.17513157 Progenitors_1
10x_3288_t1_TATCCAACGCCCTT-1 Patient5 Monocytes nonactive 0 0.03411325 Monocytes_0
10x_3288_t1_TATCCTGAGCAGAG-1 Patient5 Progenitors active 1 0.13627861 Progenitors_1
10x_3288_t1_TATCCTGAGCTCCT-1 Patient5 Progenitors nonactive 1 0.10777693 Progenitors_1
10x_3288_t1_TATCGACTACCGAT-1 Patient5 Monocytes nonactive 8 0.02070256 Monocytes_8
10x_3288_t1_TATCGACTCATACG-1 Patient5 Monocytes nonactive 0 0.06366115 Monocytes_0
10x_3288_t1_TATCGACTCTGGAT-1 Patient5 Monocytes nonactive 4 0.03993089 Monocytes_4
10x_3288_t1_TATCGACTGAGCAG-1 Patient5 Progenitors nonactive 1 0.11536998 Progenitors_1
10x_3288_t1_TATCGACTTGACTG-1 Patient5 Monocytes nonactive 0 0.11077294 Monocytes_0
10x_3288_t1_TATCGTACTGGTGT-1 Patient5 Monocytes nonactive 4 0.04484497 Monocytes_4
10x_3288_t1_TATCTCGATAGTCG-1 Patient5 Monocytes nonactive 4 0.04262571 Monocytes_4
10x_3288_t1_TATCTCGATCTCAT-1 Patient5 Monocytes nonactive 0 0.03912244 Monocytes_0

10x_3288_t1_TATCTCGATGAAGA-1 Patient5 Monocytes nonactive 8 0.03075265
Monocytes_8
10x_3288_t1_TATCTCGATTTCGT-1 Patient5 Monocytes nonactive 13 0.02731279
Monocytes_13
10x_3288_t1_TATCTGACACACTG-1 Patient5 Monocytes nonactive 0 0.10045336
Monocytes_0
10x_3288_t1_TATCTGACTGTTCT-1 Patient5 Monocytes nonactive 8 0.06164796
Monocytes_8
10x_3288_t1_TATCTTCTAGTCGT-1 Patient5 Monocytes nonactive 0 0.05781181
Monocytes_0
10x_3288_t1_TATCTTCTGGACAG-1 Patient5 Progenitors active 1 0.19730835
Progenitors_1
10x_3288_t1_TATCTTCTTCTCTA-1 Patient5 Monocytes nonactive 4 0 Monocytes_4
10x_3288_t1_TATGAATGATAAGG-1 Patient5 Progenitors active 1 0.15660072
Progenitors_1
10x_3288_t1_TATGAATGCTTGAG-1 Patient5 Monocytes nonactive 0 0.0805434
Monocytes_0
10x_3288_t1_TATGAATGTAGAGA-1 Patient5 Progenitors nonactive 1 0.08449052
Progenitors_1
10x_3288_t1_TATGCGGATCGCCT-1 Patient5 Progenitors active 1 0.15084649
Progenitors_1
10x_3288_t1_TATGGGACAGTCTG-1 Patient5 Monocytes nonactive 4 0.04169044
Monocytes_4
10x_3288_t1_TATGGGACGTCTAG-1 Patient5 Dendritic cells nonactive 10 0.09284446
Dendritic cells_10
10x_3288_t1_TATGGGTGCCGTAA-1 Patient5 Monocytes nonactive 4 0.04807875
Monocytes_4
10x_3288_t1_TATGGGTGCTGTTT-1 Patient5 Monocytes nonactive 4 0.0176907
Monocytes_4
10x_3288_t1_TATGGGTGGGTAAA-1 Patient5 Monocytes nonactive 0 0.06687908
Monocytes_0
10x_3288_t1_TATGGGTGGGTATC-1 Patient5 Monocytes nonactive 4 0.05415002
Monocytes_4

10x_3288_t1_TATGGGTGTGTGAC-1 Patient5 Monocytes nonactive 0 0.05330987
Monocytes_0
10x_3288_t1_TATGGGTGTTCTAC-1 Patient5 Monocytes nonactive 0 0.0906252
Monocytes_0
10x_3288_t1_TATGGTCTAGATCC-1 Patient5 Monocytes nonactive 4 0.05069431
Monocytes_4
10x_3288_t1_TATGGTCTGCGAAG-1 Patient5 Monocytes nonactive 0 0.04454378
Monocytes_0
10x_3288_t1_TATGGTCTTATCGG-1 Patient5 Monocytes nonactive 13 0.03991503
Monocytes_13
10x_3288_t1_TATGGTCTTCACGA-1 Patient5 Monocytes nonactive 0 0.07161879
Monocytes_0
10x_3288_t1_TATGTCACGGTCAT-1 Patient5 Monocytes nonactive 4 0.04844335
Monocytes_4
10x_3288_t1_TATGTCTGTGGCAT-1 Patient5 Monocytes nonactive 0 0.05613151
Monocytes_0
10x_3288_t1_TATGTCTGTTCGTT-1 Patient5 Monocytes nonactive 4 0.02418997
Monocytes_4
10x_3288_t1_TATGTGCTAACCGT-1 Patient5 Progenitors nonactive 1 0.10050092
Progenitors_1
10x_3288_t1_TATGTGCTATCAGC-1 Patient5 Monocytes nonactive 4 0.04492423
Monocytes_4
10x_3288_t1_TATGTGCTCTTGTT-1 Patient5 Monocytes nonactive 8 0.0552121
Monocytes_8
10x_3288_t1_TATGTGCTGTCATG-1 Patient5 Monocytes nonactive 4 0.05871536
Monocytes_4
10x_3288_t1_TATTGCTGGGCAAG-1 Patient5 Monocytes nonactive 4 0.03094287
Monocytes_4
10x_3288_t1_TATTTCCTCTCTCG-1 Patient5 Monocytes nonactive 0 0.05400736
Monocytes_0
10x_3288_t1_TATTTCCTTCTCCG-1 Patient5 Progenitors nonactive 5 0.09138609
Progenitors_5
10x_3288_t1_TCAACACTGGCGAA-1 Patient5 Monocytes nonactive 0 0.03154524

Monocytes_0

10x_3288_t1_TCAACACTTGGGAG-1 Patient5 Monocytes nonactive 4 0.05489506 Monocytes_4

10x_3288_t1_TCAAGGACCTACGA-1 Patient5 Monocytes nonactive 4 0.05958722 Monocytes_4

10x_3288_t1_TCAAGGACTAGCGT-1 Patient5 Monocytes nonactive 4 0.02685308 Monocytes_4

10x_3288_t1_TCAAGGACTGTCAG-1 Patient5 Monocytes nonactive 0 0.02458627 Monocytes_0

10x_3288_t1_TCAAGGTGCGCAAT-1 Patient5 Monocytes nonactive 0 0.01016106 Monocytes_0

10x_3288_t1_TCAAGGTGCTAGAC-1 Patient5 Monocytes nonactive 0 0.04796779 Monocytes_0

10x_3288_t1_TCAAGGTGGGTTCA-1 Patient5 Monocytes nonactive 0 0.05191491 Monocytes_0

10x_3288_t1_TCAAGGTGTAACGC-1 Patient5 Monocytes nonactive 4 0.0341291 Monocytes_4

10x_3288_t1_TCAAGGTGTCTATC-1 Patient5 Monocytes nonactive 0 0.03882125 Monocytes_0

10x_3288_t1_TCAAGTCTACTCAG-1 Patient5 Monocytes nonactive 4 0.043022 Monocytes_4

10x_3288_t1_TCAAGTCTTACTCT-1 Patient5 Progenitors active 1 0.16048443 Progenitors_1

10x_3288_t1_TCAAGTCTTTGCTT-1 Patient5 Monocytes nonactive 8 0.02542642 Monocytes_8

10x_3288_t1_TCAATAGAAAGAGT-1 Patient5 Progenitors active 1 0.1770338 Progenitors_1

10x_3288_t1_TCAATAGAACCTTT-1 Patient5 Monocytes nonactive 0 0.02239871 Monocytes_0

10x_3288_t1_TCAATAGAATTCCT-1 Patient5 Progenitors active 1 0.22378099 Progenitors_1

10x_3288_t1_TCAATAGACCGTAA-1 Patient5 Monocytes nonactive 0 0.06458056 Monocytes_0

10x_3288_t1_TCAATAGAGAGGAC-1 Patient5 Progenitors active 1 0.12299474
Progenitors_1
10x_3288_t1_TCAATAGATAGACC-1 Patient5 Monocytes nonactive 4 0.06711686
Monocytes_4
10x_3288_t1_TCAATCACACGGAG-1 Patient5 Monocytes nonactive 4 0.06867034
Monocytes_4
10x_3288_t1_TCAATCACAGTAGA-1 Patient5 Monocytes nonactive 4 0.01666033
Monocytes_4
10x_3288_t1_TCAATCACTATTCC-1 Patient5 Monocytes nonactive 0 0.03357428
Monocytes_0
10x_3288_t1_TCACAACTCCCACT-1 Patient5 Progenitors active 1 0.15820176
Progenitors_1
10x_3288_t1_TCACAACTTCGATG-1 Patient5 Monocytes nonactive 4 0.02132078
Monocytes_4
10x_3288_t1_TCACATACGCTACA-1 Patient5 Monocytes nonactive 0 0.03309873
Monocytes_0
10x_3288_t1_TCACATACTCCGAA-1 Patient5 Progenitors active 1 0.18071143
Progenitors_1
10x_3288_t1_TCACCCGAACGGAG-1 Patient5 Progenitors active 1 0.14699448
Progenitors_1
10x_3288_t1_TCACCCGAAGCAAA-1 Patient5 Monocytes nonactive 0 0.01027202
Monocytes_0
10x_3288_t1_TCACCCGACCGTTC-1 Patient5 Monocytes nonactive 0 0.02753472
Monocytes_0
10x_3288_t1_TCACCCGAGTAGCT-1 Patient5 Monocytes nonactive 0 0.05927018
Monocytes_0
10x_3288_t1_TCACCCGATGTCTT-1 Patient5 Monocytes nonactive 0 0.02938939
Monocytes_0
10x_3288_t1_TCACCCGATTTGTC-1 Patient5 Monocytes nonactive 4 0.0585727
Monocytes_4
10x_3288_t1_TCACCGTGAAGCCT-1 Patient5 Monocytes nonactive 0 0.06058589
Monocytes_0
10x_3288_t1_TCACCGTGCCGAAT-1 Patient5 Monocytes nonactive 0 0.02599708

Monocytes_0
10x_3288_t1_TCACCGTGCTCAAG-1 Patient5 Monocytes nonactive 0 0.04963224
Monocytes_0
10x_3288_t1_TCACCTCTAGCGTT-1Patient5 Progenitors nonactive 1 0.10454315
Progenitors_1
10x_3288_t1_TCACCTCTCATTCT-1 Patient5 Progenitors active 1 0.16371822
Progenitors_1
10x_3288_t1_TCACCTCTCGTAGT-1Patient5 Monocytes nonactive 0 0.06060174
Monocytes_0
10x_3288_t1_TCACCTCTTAACGC-1 Patient5 Dendritic cells nonactive 10 0.11530658
Dendritic cells_10
10x_3288_t1_TCACCTCTTGCGTA-1 Patient5 Monocytes nonactive 0 0.02510938
Monocytes_0
10x_3288_t1_TCACGAGAAGTCTG-1 Patient5 Dendritic cells nonactive 10 0.10942553
Dendritic cells_10
10x_3288_t1_TCACGAGAGAGAGC-1 Patient5 Monocytes nonactive 0 0.02460212
Monocytes_0
10x_3288_t1_TCACGAGATCGATG-1 Patient5 Monocytes nonactive 8 0.07288694
Monocytes_8
10x_3288_t1_TCACGAGATGTGGT-1 Patient5 Monocytes nonactive 4 0.04283178
Monocytes_4
10x_3288_t1_TCACGAGATTATCC-1 Patient5 Monocytes nonactive 0 0.05180394
Monocytes_0
10x_3288_t1_TCACTATGAACCTG-1 Patient5 Monocytes nonactive 0 0.03999429
Monocytes_0
10x_3288_t1_TCACTATGAGCATC-1 Patient5 Progenitors active 1 0.15232072
Progenitors_1
10x_3288_t1_TCACTATGCATCAG-1 Patient5 Monocytes nonactive 0 0.02217678
Monocytes_0
10x_3288_t1_TCACTATGCCCACT-1Patient5 Progenitors active 1 0.15701287
Progenitors_1
10x_3288_t1_TCACTATGCGAGTT-1Patient5 Progenitors active 1 0.12469089
Progenitors_1

10x_3288_t1_TCACTATGTCTACT-1 Patient5 Progenitors active 1 0.20220658 Progenitors_1
10x_3288_t1_TCAGACGACACTTT-1 Patient5 Monocytes nonactive 4 0.04281593 Monocytes_4
10x_3288_t1_TCAGACGACTCTAT-1 Patient5 Monocytes nonactive 4 0.10317989 Monocytes_4
10x_3288_t1_TCAGACGACTGCAA-1 Patient5 Monocytes nonactive 0 0.05635343 Monocytes_0
10x_3288_t1_TCAGACGAGAATGA-1 Patient5 Progenitors active 1 0.13732484 Progenitors_1
10x_3288_t1_TCAGACGATCGTTT-1 Patient5 Monocytes nonactive 4 0.01485321 Monocytes_4
10x_3288_t1_TCAGACGATGTCAG-1 Patient5 Monocytes nonactive 0 0.05961892 Monocytes_0
10x_3288_t1_TCAGAGACAACCTG-1 Patient5 Monocytes nonactive 0 0.0160104 Monocytes_0
10x_3288_t1_TCAGAGACAGATGA-1 Patient5 Monocytes nonactive 0 0.04226111 Monocytes_0
10x_3288_t1_TCAGAGACTTGTCT-1 Patient5 Monocytes nonactive 0 0.08211274 Monocytes_0
10x_3288_t1_TCAGCAGAATTCCT-1 Patient5 Monocytes nonactive 4 0.03267072 Monocytes_4
10x_3288_t1_TCAGCAGACTACGA-1 Patient5 Monocytes nonactive 0 0.02445945 Monocytes_0
10x_3288_t1_TCAGCAGACTGAGT-1 Patient5 Progenitors active 1 0.17283305 Progenitors_1
10x_3288_t1_TCAGCAGAGTATCG-1 Patient5 Monocytes nonactive 0 0.04400482 Monocytes_0
10x_3288_t1_TCAGCGCTAGTTCG-1 Patient5 Monocytes nonactive 0 0.03463636 Monocytes_0
10x_3288_t1_TCAGCGCTCGAACT-1 Patient5 Dendritic cells nonactive 10 0.07946547 Dendritic cells_10
10x_3288_t1_TCAGGATGACTTTC-1 Patient5 Progenitors active 1 0.14786634

Progenitors_1

10x_3288_t1_TCAGGATGCTGTGA-1 Patient5 Monocytes nonactive 0 0.06155285 Monocytes_0

10x_3288_t1_TCAGTACTGCTACA-1 Patient5 Monocytes nonactive 4 0.04199163 Monocytes_4

10x_3288_t1_TCAGTACTTTCCCG-1 Patient5 Progenitors active 6 0.143301 Progenitors_6

10x_3288_t1_TCAGTGGAAAGTAG-1 Patient5 Monocytes nonactive 0 0.04722275 Monocytes_0

10x_3288_t1_TCAGTGGAAGCCTA-1 Patient5 Monocytes nonactive 4 0.00732357 Monocytes_4

10x_3288_t1_TCAGTGGAGTCTTT-1 Patient5 Monocytes nonactive 4 0.06716442 Monocytes_4

10x_3288_t1_TCAGTGGAGTTCTT-1 Patient5 Monocytes nonactive 8 0.05459387 Monocytes_8

10x_3288_t1_TCAGTGGATTGGCA-1 Patient5 Monocytes nonactive 0 0.06120411 Monocytes_0

10x_3288_t1_TCAGTTACAGAGAT-1 Patient5 Monocytes nonactive 8 0.02027455 Monocytes_8

10x_3288_t1_TCAGTTACGGGTGA-1 Patient5 Monocytes nonactive 0 0.01821381 Monocytes_0

10x_3288_t1_TCAGTTACTCCTCG-1 Patient5 Progenitors nonactive 1 0.10947308 Progenitors_1

10x_3288_t1_TCATCAACTGTTTC-1Patient5 Progenitors active 1 0.15831273 Progenitors_1

10x_3288_t1_TCATCATGCGAACT-1Patient5 Monocytes nonactive 0 0.02231945 Monocytes_0

10x_3288_t1_TCATCATGCGTACA-1 Patient5 Monocytes nonactive 4 0.05519625 Monocytes_4

10x_3288_t1_TCATCATGCTGAAC-1 Patient5 Monocytes nonactive 13 0.05061505 Monocytes_13

10x_3288_t1_TCATCATGGAAAGT-1 Patient5 Monocytes nonactive 4 0.07597806 Monocytes_4

10x_3288_t1_TCATCATGGACACT-1Patient5 Monocytes nonactive 0 0.04603386
Monocytes_0
10x_3288_t1_TCATCATGGAGATA-1 Patient5 Progenitors active 1 0.12161562
Progenitors_1
10x_3288_t1_TCATCATGGTTCTT-1 Patient5 Monocytes nonactive 4 0.10222877
Monocytes_4
10x_3288_t1_TCATCATGTAGTCG-1Patient5 Progenitors active 1 0.18835204
Progenitors_1
10x_3288_t1_TCATCATGTGCCAA-1 Patient5 Progenitors active 6 0.16027836
Progenitors_6
10x_3288_t1_TCATCCCTCCCGTT-1 Patient5 Monocytes nonactive 4 0.06725953
Monocytes_4
10x_3288_t1_TCATCCCTTCCTAT-1 Patient5 Monocytes nonactive 4 0.03438273
Monocytes_4
10x_3288_t1_TCATCCCTTCGACA-1 Patient5 Monocytes nonactive 4 0.03309873
Monocytes_4
10x_3288_t1_TCATGTACAGCGTT-1Patient5 Monocytes nonactive 0 0.03145013
Monocytes_0
10x_3288_t1_TCATGTACCCAGTA-1 Patient5 Progenitors active 1 0.13141209
Progenitors_1
10x_3288_t1_TCATGTACCGTACA-1 Patient5 Progenitors active 1 0.15896265
Progenitors_1
10x_3288_t1_TCATGTACCTACTT-1 Patient5 Progenitors nonactive 1 0.09243231
Progenitors_1
10x_3288_t1_TCATTCGACCTTCG-1 Patient5 Monocytes nonactive 0 0.03038805
Monocytes_0
10x_3288_t1_TCATTGACAATCGC-1 Patient5 Monocytes nonactive 0 0.01041469
Monocytes_0
10x_3288_t1_TCCACGTGAGCATC-1 Patient5 Monocytes nonactive 4 0.04162704
Monocytes_4
10x_3288_t1_TCCACGTGCCACAA-1 Patient5 Progenitors active 1 0.12538837
Progenitors_1
10x_3288_t1_TCCACGTGCTCAAG-1 Patient5 Monocytes nonactive 13 0.03924925

Monocytes_13
10x_3288_t1_TCCACGTGGGTATC-1 Patient5 Monocytes nonactive 4 0.05128083
Monocytes_4
10x_3288_t1_TCCACTCTAAGCCT-1 Patient5 Monocytes nonactive 0 0.04026378
Monocytes_0
10x_3288_t1_TCCACTCTCGTGTA-1 Patient5 Monocytes nonactive 0 0.04448038
Monocytes_0
10x_3288_t1_TCCACTCTGGGATG-1 Patient5 Monocytes nonactive 4 0.00700653
Monocytes_4
10x_3288_t1_TCCACTCTTCTTAC-1 Patient5 Progenitors active 1 0.16194281
Progenitors_1
10x_3288_t1_TCCAGAGAAAGCCT-1 Patient5 Monocytes nonactive 4 0.06678397
Monocytes_4
10x_3288_t1_TCCAGAGATCCTAT-1 Patient5 Monocytes nonactive 4 0.02767738
Monocytes_4
10x_3288_t1_TCCAGAGATGTCAG-1 Patient5 Progenitors active 1 0.15106842
Progenitors_1
10x_3288_t1_TCCAGAGATTGCTT-1 Patient5 Monocytes nonactive 0 0.02265234
Monocytes_0
10x_3288_t1_TCCATAACCATGGT-1 Patient5 Monocytes nonactive 0 0.06072855
Monocytes_0
10x_3288_t1_TCCATAACCTAAGC-1 Patient5 Progenitors active 1 0.14116099
Progenitors_1
10x_3288_t1_TCCATAACCTATGG-1 Patient5 Monocytes nonactive 4 0.06854353
Monocytes_4
10x_3288_t1_TCCATAACGATACC-1 Patient5 Progenitors active 1 0.15208294
Progenitors_1
10x_3288_t1_TCCATAACTCGCCT-1 Patient5 Monocytes nonactive 8 0.04817386
Monocytes_8
10x_3288_t1_TCCATAACTTCCCG-1 Patient5 Progenitors nonactive 1 0.10338596
Progenitors_1
10x_3288_t1_TCCATAACTTGGTG-1 Patient5 Monocytes nonactive 4 0.0885169
Monocytes_4

10x_3288_t1_TCCATCCTATGACC-1 Patient5 Monocytes nonactive 4 0.04616067
Monocytes_4
10x_3288_t1_TCCATCCTCATACG-1 Patient5 Monocytes nonactive 4 0.06115655
Monocytes_4
10x_3288_t1_TCCCACGAACCACA-1 Patient5 Progenitors active 1 0.1741012
Progenitors_1
10x_3288_t1_TCCCACGAAGAGAT-1 Patient5 Monocytes nonactive 0 0.03496925
Monocytes_0
10x_3288_t1_TCCCACGAATTGGC-1 Patient5 Monocytes nonactive 4 0.05923848
Monocytes_4
10x_3288_t1_TCCCAGACACGACT-1 Patient5 Monocytes nonactive 4 0.03352673
Monocytes_4
10x_3288_t1_TCCCAGACCTAGTG-1 Patient5 Progenitors active 1 0.15417539
Progenitors_1
10x_3288_t1_TCCCAGACCTCGCT-1 Patient5 Progenitors active 1 0.12537252
Progenitors_1
10x_3288_t1_TCCCAGACCTGCTC-1 Patient5 Monocytes nonactive 0 0.05036142
Monocytes_0
10x_3288_t1_TCCCAGACGCTATG-1 Patient5 Monocytes active 8 0.13895758
Monocytes_8
10x_3288_t1_TCCCAGACTACTGG-1 Patient5 Progenitors active 1 0.127354
Progenitors_1
10x_3288_t1_TCCCAGACTCGTTT-1 Patient5 Monocytes nonactive 0 0.03847251
Monocytes_0
10x_3288_t1_TCCCATCTAAGGGC-1 Patient5 Progenitors nonactive 5 0.07903747
Progenitors_5
10x_3288_t1_TCCCATCTACTCTT-1 Patient5 Progenitors active 1 0.18973115
Progenitors_1
10x_3288_t1_TCCCATCTATCACG-1 Patient5 Monocytes nonactive 0 0.04543149
Monocytes_0
10x_3288_t1_TCCCATCTGCAGAG-1 Patient5 Monocytes nonactive 4 0.02312789
Monocytes_4
10x_3288_t1_TCCCATCTGCGAAG-1 Patient5 Progenitors active 1 0.17037601

Progenitors_1
10x_3288_t1_TCCCATCTTGCATG-1 Patient5 Monocytes nonactive 0 0.03430347
Monocytes_0
10x_3288_t1_TCCCGAACAGAATG-1 Patient5 Progenitors active 5 0.17443409
Progenitors_5
10x_3288_t1_TCCCGAACGAAACA-1 Patient5 Progenitors active 1 0.12483356
Progenitors_1
10x_3288_t1_TCCCGAACGACGGA-1 Patient5 Monocytes nonactive 0 0.0454949
Monocytes_0
10x_3288_t1_TCCCGATGGTACAC-1 Patient5 Progenitors active 1 0.12288377
Progenitors_1
10x_3288_t1_TCCCGATGTGTCTT-1 Patient5 Monocytes nonactive 4 0.05280261
Monocytes_4
10x_3288_t1_TCCCGATGTTCTTG-1 Patient5 Progenitors active 1 0.19028597
Progenitors_1
10x_3288_t1_TCCCTACTAAAACG-1 Patient5 Monocytes nonactive 4 0.06909835
Monocytes_4
10x_3288_t1_TCCCTACTACGCAT-1 Patient5 Monocytes nonactive 0 0.03263902
Monocytes_0
10x_3288_t1_TCCCTACTCAACTG-1 Patient5 Monocytes nonactive 0 0.04222941
Monocytes_0
10x_3288_t1_TCCCTACTCCACAA-1 Patient5 Monocytes nonactive 8 0.08531482
Monocytes_8
10x_3288_t1_TCCCTACTTCTTTG-1 Patient5 Monocytes nonactive 4 0.00781498
Monocytes_4
10x_3288_t1_TCCCTACTTGGTAC-1 Patient5 Monocytes nonactive 4 0.03490584
Monocytes_4
10x_3288_t1_TCCGAAGAACGTAC-1 Patient5 Monocytes nonactive 4 0.02412656
Monocytes_4
10x_3288_t1_TCCGAAGAGTACGT-1 Patient5 Monocytes nonactive 0 0.02428508
Monocytes_0
10x_3288_t1_TCCGAAGATTTGGG-1 Patient5 Monocytes nonactive 4 0.03912244
Monocytes_4

10x_3288_t1_TCCGAGCTGCAAGG-1 Patient5 Progenitors active 1 0.17887261 Progenitors_1
10x_3288_t1_TCCGAGCTGTTGGT-1 Patient5 Progenitors active 1 0.16218058 Progenitors_1
10x_3288_t1_TCCGAGCTTATGGC-1 Patient5 Monocytes nonactive 0 0.07642191 Monocytes_0
10x_3288_t1_TCCGAGCTTCTCAT-1 Patient5 Monocytes nonactive 0 0.03644347 Monocytes_0
10x_3288_t1_TCCGGACTGTACCA-1 Patient5 Monocytes nonactive 4 0.05974574 Monocytes_4
10x_3288_t1_TCCTAAACCGATAC-1 Patient5 Monocytes nonactive 0 0.04495593 Monocytes_0
10x_3288_t1_TCCTAAACCGTAAC-1 Patient5 Monocytes nonactive 0 0.06435863 Monocytes_0
10x_3288_t1_TCCTAAACGAGACG-1 Patient5 Monocytes nonactive 0 0.0598884 Monocytes_0
10x_3288_t1_TCCTAAACTCCAGA-1 Patient5 Monocytes nonactive 0 0.00206074 Monocytes_0
10x_3288_t1_TCCTAAACTTCTGT-1 Patient5 Progenitors active 1 0.15095745 Progenitors_1
10x_3288_t1_TCCTAATGGCCAAT-1 Patient5 Monocytes nonactive 0 0.04520956 Monocytes_0
10x_3288_t1_TCCTAATGTCCTGC-1 Patient5 Monocytes nonactive 0 0.06687908 Monocytes_0
10x_3288_t1_TCCTACCTAGAAGT-1 Patient5 Progenitors active 1 0.16996386 Progenitors_1
10x_3288_t1_TCCTACCTCATGGT-1 Patient5 Progenitors active 1 0.13453491 Progenitors_1
10x_3288_t1_TCCTACCTCTGTCC-1 Patient5 Monocytes nonactive 4 0.03078435 Monocytes_4
10x_3288_t1_TCCTACCTGGTAAA-1 Patient5 Monocytes nonactive 0 0.03400228 Monocytes_0
10x_3288_t1_TCCTACCTTCGTGA-1 Patient5 Monocytes nonactive 4 0.03068924

Monocytes_4
10x_3288_t1_TCCTACCTTTGACG-1 Patient5 Progenitors nonactive 1 0.11660643 Progenitors_1
10x_3288_t1_TCCTATGATAGTCG-1 Patient5 Progenitors active 6 0.14924545 Progenitors_6
10x_3288_t1_TCCTATGATCTACT-1 Patient5 Monocytes nonactive 4 0.02564834 Monocytes_4
10x_3288_t1_TCCTATGATTTGTC-1 Patient5 Progenitors nonactive 1 0.11284953 Progenitors_1
10x_3288_t1_TCGAATCTGGCATT-1 Patient5 Monocytes nonactive 0 0.07120665 Monocytes_0
10x_3288_t1_TCGAATCTGTGAGG-1 Patient5 Monocytes nonactive 0 0.0535635 Monocytes_0
10x_3288_t1_TCGACCTGACGGGA-1 Patient5 Monocytes nonactive 0 0.08217615 Monocytes_0
10x_3288_t1_TCGACCTGCTGACA-1 Patient5 Monocytes nonactive 0 0.06055418 Monocytes_0
10x_3288_t1_TCGACCTGCTGATG-1 Patient5 Monocytes nonactive 4 0.03804451 Monocytes_4
10x_3288_t1_TCGACCTGCTGCTC-1 Patient5 Monocytes nonactive 0 0.03864688 Monocytes_0
10x_3288_t1_TCGACCTGTGCCCT-1 Patient5 Monocytes nonactive 0 0.06080781 Monocytes_0
10x_3288_t1_TCGACGCTACGTGT-1 Patient5 Monocytes nonactive 4 0.06042737 Monocytes_4
10x_3288_t1_TCGACGCTAGGTCT-1 Patient5 Progenitors active 1 0.2035857 Progenitors_1
10x_3288_t1_TCGACGCTCCCGTT-1 Patient5 Progenitors active 1 0.12760763 Progenitors_1
10x_3288_t1_TCGAGAACAACCGT-1 Patient5 Progenitors active 1 0.15379494 Progenitors_1
10x_3288_t1_TCGAGAACAAGAAC-1 Patient5 Monocytes active 13 0.14169996 Monocytes_13

10x_3288_t1_TCGAGAACAAGAGT-1 Patient5 Monocytes nonactive 4 0.05968233 Monocytes_4
10x_3288_t1_TCGAGAACTACGAC-1 Patient5 Progenitors active 1 0.19063471 Progenitors_1
10x_3288_t1_TCGAGAACTGCGTA-1 Patient5 Progenitors active 1 0.15446072 Progenitors_1
10x_3288_t1_TCGAGCCTCTCAAG-1 Patient5 Monocytes nonactive 0 0.04119904 Monocytes_0
10x_3288_t1_TCGAGCCTCTGTAG-1 Patient5 Progenitors active 1 0.12410437 Progenitors_1
10x_3288_t1_TCGAGCCTGACGAG-1 Patient5 Progenitors active 1 0.13553357 Progenitors_1
10x_3288_t1_TCGAGCCTTCGACA-1 Patient5 Monocytes nonactive 8 0.05178809 Monocytes_8
10x_3288_t1_TCGATACTATCTCT-1 Patient5 Monocytes nonactive 0 0.01723099 Monocytes_0
10x_3288_t1_TCGATTTGTGCAAC-1 Patient5 Progenitors active 1 0.1737049 Progenitors_1
10x_3288_t1_TCGCAAGAAGCTCA-1 Patient5 Progenitors active 1 0.17048697 Progenitors_1
10x_3288_t1_TCGCAAGACAGTTG-1 Patient5 Monocytes nonactive 0 0.05835077 Monocytes_0
10x_3288_t1_TCGCAAGACGGAGA-1 Patient5 Monocytes nonactive 8 0.04264156 Monocytes_8
10x_3288_t1_TCGCAAGAGGCATT-1 Patient5 Progenitors active 1 0.11915858 Progenitors_1
10x_3288_t1_TCGCAAGAGTCTGA-1 Patient5 Monocytes nonactive 0 0.05421343 Monocytes_0
10x_3288_t1_TCGCAAGAGTTCTT-1 Patient5 Monocytes nonactive 0 0.07179316 Monocytes_0
10x_3288_t1_TCGCAAGATCCTTA-1 Patient5 Monocytes nonactive 0 0.03049902 Monocytes_0
10x_3288_t1_TCGCACACAGAGTA-1 Patient5 Monocytes nonactive 4 0.02950035

Monocytes_4
10x_3288_t1_TCGCACACGCCATA-1 Patient5 Progenitors active 1 0.15664828
Progenitors_1
10x_3288_t1_TCGCACACTGCTTT-1 Patient5 Monocytes nonactive 4 0.03764822
Monocytes_4
10x_3288_t1_TCGCACTGAGAACA-1 Patient5 Monocytes nonactive 4 0.05571936
Monocytes_4
10x_3288_t1_TCGCACTGGGGCAA-1 Patient5 Monocytes nonactive 0 0.10062773
Monocytes_0
10x_3288_t1_TCGCAGCTATCGTG-1 Patient5 Progenitors active 1 0.14366559
Progenitors_1
10x_3288_t1_TCGCAGCTGGTCTA-1 Patient5 Progenitors active 1 0.17525839
Progenitors_1
10x_3288_t1_TCGCAGCTGTCATG-1 Patient5 Monocytes nonactive 8 0.03599962
Monocytes_8
10x_3288_t1_TCGCAGCTTCGCAA-1 Patient5 Monocytes nonactive 4 0.05505358
Monocytes_4
10x_3288_t1_TCGCAGCTTCTGGA-1 Patient5 Monocytes nonactive 0 0.03904318
Monocytes_0
10x_3288_t1_TCGCAGCTTGCAAC-1 Patient5 Monocytes nonactive 4 0.02423752
Monocytes_4
10x_3288_t1_TCGCAGCTTGCCAA-1 Patient5 Progenitors nonactive 1 0.09977173
Progenitors_1
10x_3288_t1_TCGCCATGACGCTA-1 Patient5 Monocytes nonactive 0 0.03560332
Monocytes_0
10x_3288_t1_TCGCCATGAGCGTT-1 Patient5 Progenitors active 1 0.1256103
Progenitors_1
10x_3288_t1_TCGCCATGAGGGTG-1 Patient5 Progenitors nonactive 1 0.07983007
Progenitors_1
10x_3288_t1_TCGCCATGATAAGG-1 Patient5 Monocytes nonactive 4 0.03488999
Monocytes_4
10x_3288_t1_TCGCCATGCAGAAA-1 Patient5 Monocytes nonactive 0 0.05280261
Monocytes_0

10x_3288_t1_TCGCCATGTGAACC-1 Patient5 Progenitors active 1 0.17145393
Progenitors_1
10x_3288_t1_TCGGACCTCCAACA-1 Patient5 Monocytes nonactive 0 0.03395473
Monocytes_0
10x_3288_t1_TCGGACCTGAACTC-1 Patient5 Monocytes nonactive 0 0.08098726
Monocytes_0
10x_3288_t1_TCGGACCTGAATCC-1 Patient5 Progenitors active 1 0.14773952
Progenitors_1
10x_3288_t1_TCGGACCTGTTTCT-1Patient5 Monocytes nonactive 4 0.02132078
Monocytes_4
10x_3288_t1_TCGGACCTTGATGC-1 Patient5 Progenitors nonactive 1 0.09198846
Progenitors_1
10x_3288_t1_TCGGACCTTTTCTG-1 Patient5 Progenitors active 1 0.15951747
Progenitors_1
10x_3288_t1_TCGGCACTATGCTG-1 Patient5 Monocytes nonactive 4 0.05538647
Monocytes_4
10x_3288_t1_TCGTAGGACGTAAC-1 Patient5 Monocytes nonactive 8 0.02671042
Monocytes_8
10x_3288_t1_TCGTAGGAGGTATC-1 Patient5 Monocytes nonactive 4 0.01727855
Monocytes_4
10x_3288_t1_TCGTAGGAGTATGC-1 Patient5 Monocytes nonactive 4 0.02044893
Monocytes_4
10x_3288_t1_TCGTGAGAATTCCT-1 Patient5 Progenitors active 1 0.19241012
Progenitors_1
10x_3288_t1_TCGTGAGAGTTACG-1 Patient5 Monocytes nonactive 0 0.04215015
Monocytes_0
10x_3288_t1_TCGTGAGATTCACT-1 Patient5 Progenitors active 6 0.15748843
Progenitors_6
10x_3288_t1_TCGTTATGCAGTCA-1 Patient5 Progenitors nonactive 6 0.10719041
Progenitors_6
10x_3288_t1_TCGTTATGCTAGCA-1Patient5 Monocytes nonactive 0 0.09124342
Monocytes_0
10x_3288_t1_TCTAACACGAACCT-1 Patient5 Monocytes nonactive 0 0.01185721

Monocytes_0
10x_3288_t1_TCTAACACGTAAGA-1 Patient5 Progenitors active 1 0.15339864 Progenitors_1
10x_3288_t1_TCTAACACTGAGCT-1 Patient5 Progenitors active 1 0.13272779 Progenitors_1
10x_3288_t1_TCTAACTGAACGAA-1 Patient5 Monocytes nonactive 0 0.02591782 Monocytes_0
10x_3288_t1_TCTAACTGACTCTT-1 Patient5 Progenitors active 1 0.1463287 Progenitors_1
10x_3288_t1_TCTAACTGATCACG-1 Patient5 Monocytes nonactive 8 0.0433866 Monocytes_8
10x_3288_t1_TCTAACTGGTTACG-1 Patient5 Progenitors nonactive 1 0.10803056 Progenitors_1
10x_3288_t1_TCTAAGCTAGAAGT-1 Patient5 Monocytes nonactive 0 0.081764 Monocytes_0
10x_3288_t1_TCTAAGCTCACTCC-1 Patient5 Monocytes nonactive 0 0.04287934 Monocytes_0
10x_3288_t1_TCTAAGCTCTCCCA-1 Patient5 Monocytes nonactive 0 0.0244119 Monocytes_0
10x_3288_t1_TCTAAGCTGGTTAC-1 Patient5 Progenitors active 1 0.14742248 Progenitors_1
10x_3288_t1_TCTAAGCTGGTTTG-1 Patient5 Progenitors nonactive 6 0.11578213 Progenitors_6
10x_3288_t1_TCTACAACAGATCC-1 Patient5 Progenitors active 1 0.16471689 Progenitors_1
10x_3288_t1_TCTAGACTGCCCTT-1 Patient5 Monocytes nonactive 0 0.06221863 Monocytes_0
10x_3288_t1_TCTAGACTTCTACT-1 Patient5 Monocytes nonactive 0 0.06635597 Monocytes_0
10x_3288_t1_TCTAGTTGCCATAG-1 Patient5 Monocytes nonactive 15 0.04736542 Monocytes_15
10x_3288_t1_TCTAGTTGCTGCTC-1 Patient5 Monocytes nonactive 4 0.0712859 Monocytes_4

10x_3288_t1_TCTAGTTGTCTTTG-1 Patient5 Monocytes nonactive 0 0.06645108 Monocytes_0
10x_3288_t1_TCTAGTTGTTGCGA-1 Patient5 Monocytes nonactive 0 0.02433264 Monocytes_0
10x_3288_t1_TCTATGTGAAACGA-1 Patient5 Progenitors nonactive 1 0.10379811 Progenitors_1
10x_3288_t1_TCTATGTGATGTGC-1 Patient5 Monocytes nonactive 0 0.07033479 Monocytes_0
10x_3288_t1_TCTATGTGGTATCG-1 Patient5 Monocytes nonactive 0 0.06251981 Monocytes_0
10x_3288_t1_TCTATGTGGTTACG-1 Patient5 Progenitors active 1 0.14030499 Progenitors_1
10x_3288_t1_TCTATGTGTGTCGA-1 Patient5 Monocytes nonactive 0 0.04325978 Monocytes_0
10x_3288_t1_TCTCAAACATGTGC-1 Patient5 Monocytes nonactive 0 0.05906411 Monocytes_0
10x_3288_t1_TCTCAAACGACACT-1 Patient5 Progenitors active 6 0.13057194 Progenitors_6
10x_3288_t1_TCTCAAACGGAGCA-1 Patient5 Monocytes nonactive 13 0.02902479 Monocytes_13
10x_3288_t1_TCTCAAACGGTCTA-1 Patient5 Progenitors nonactive 1 0.11337265 Progenitors_1
10x_3288_t1_TCTCAAACGTACGT-1 Patient5 Monocytes nonactive 8 0.09507958 Monocytes_8
10x_3288_t1_TCTCTAGAAAGTAG-1 Patient5 Progenitors active 1 0.1804578 Progenitors_1
10x_3288_t1_TCTCTAGAAGCAAA-1 Patient5 Progenitors active 1 0.14563122 Progenitors_1
10x_3288_t1_TCTCTAGAATGGTC-1 Patient5 Progenitors active 1 0.14572633 Progenitors_1
10x_3288_t1_TCTGATACTCATTC-1 Patient5 Monocytes nonactive 4 0.042166 Monocytes_4
10x_3288_t1_TCTGATACTTCCGC-1 Patient5 Monocytes nonactive 4 0.04018452

Monocytes_4
10x_3288_t1_TCTGATACTTGCGA-1 Patient5 Monocytes nonactive 4 0.101991
Monocytes_4
10x_3288_t1_TCTTACGATTGCGA-1 Patient5 Monocytes nonactive 8 0.0767548
Monocytes_8
10x_3288_t1_TCTTCAGAAGCTAC-1 Patient5 Monocytes nonactive 0 0.03736288
Monocytes_0
10x_3288_t1_TCTTCAGAGGGTGA-1 Patient5 Monocytes nonactive 0 0.0548158
Monocytes_0
10x_3288_t1_TCTTGATGGAATAG-1 Patient5 Monocytes nonactive 0 0.02731279
Monocytes_0
10x_3288_t1_TCTTGATGGAATGA-1 Patient5 Progenitors active 1 0.13686513
Progenitors_1
10x_3288_t1_TCTTGATGGCTAAC-1 Patient5 Monocytes nonactive 4 0.05625832
Monocytes_4
10x_3288_t1_TCTTGATGGTACAC-1 Patient5 Monocytes nonactive 4 0.06104559
Monocytes_4
10x_3288_t1_TGAAATTGAGTACC-1 Patient5 Monocytes nonactive 0 0.02851753
Monocytes_0
10x_3288_t1_TGAAATTGCAATCG-1 Patient5 Monocytes nonactive 0 0.03240124
Monocytes_0
10x_3288_t1_TGAAATTGCCACAA-1 Patient5 Monocytes nonactive 0 0.06481834
Monocytes_0
10x_3288_t1_TGAAATTGTAGCGT-1 Patient5 Progenitors active 1 0.12480185
Progenitors_1
10x_3288_t1_TGAAATTGTGTTTC-1 Patient5 Monocytes nonactive 0 0.02333397
Monocytes_0
10x_3288_t1_TGAACCGACCATAG-1 Patient5 Progenitors active 1 0.18063217
Progenitors_1
10x_3288_t1_TGAACCGACCTATT-1 Patient5 Monocytes nonactive 0 0.0522478
Monocytes_0
10x_3288_t1_TGAACCGACCTTAT-1 Patient5 Monocytes nonactive 0 0.04448038
Monocytes_0

10x_3288_t1_TGAACCGAGAGAGC-1 Patient5 Monocytes nonactive 4 0.05931774 Monocytes_4
10x_3288_t1_TGAACCGATCCAAG-1 Patient5 Monocytes nonactive 4 0.03764822 Monocytes_4
10x_3288_t1_TGAACCGATGGTAC-1 Patient5 Monocytes nonactive 0 0.03222687 Monocytes_0
10x_3288_t1_TGAACCGATGTGAC-1 Patient5 Monocytes nonactive 4 0.05006024 Monocytes_4
10x_3288_t1_TGAACCGATGTGGT-1 Patient5 Monocytes nonactive 15 0.05958722 Monocytes_15
10x_3288_t1_TGAAGCACGCGATT-1 Patient5 Progenitors active 1 0.16026251 Progenitors_1
10x_3288_t1_TGAAGCACTCGCCT-1 Patient5 Monocytes nonactive 0 0.01788092 Monocytes_0
10x_3288_t1_TGAAGCACTGGTAC-1 Patient5 Monocytes nonactive 4 0.04020037 Monocytes_4
10x_3288_t1_TGAAGCTGAACCTG-1 Patient5 Monocytes nonactive 4 0.05250143 Monocytes_4
10x_3288_t1_TGAAGCTGCTGACA-1 Patient5 Monocytes nonactive 0 0.04799949 Monocytes_0
10x_3288_t1_TGAAGCTGTAAGGA-1 Patient5 Monocytes nonactive 8 0.06212352 Monocytes_8
10x_3288_t1_TGAATAACAAGATG-1 Patient5 Progenitors active 1 0.14320588 Progenitors_1
10x_3288_t1_TGACACGAGTTACG-1 Patient5 Monocytes nonactive 4 0.04757149 Monocytes_4
10x_3288_t1_TGACACGATTCCAT-1Patient5 Progenitors active 1 0.14307907 Progenitors_1
10x_3288_t1_TGACCAGAGGTGTT-1 Patient5 Progenitors active 1 0.14491789 Progenitors_1
10x_3288_t1_TGACCGCTAGAAGT-1 Patient5 Monocytes nonactive 13 0.01463129 Monocytes_13
10x_3288_t1_TGACCGCTCGACAT-1 Patient5 Progenitors nonactive 1 0.11567117

Progenitors_1

10x_3288_t1_TGACCGCTCTAGTG-1 Patient5 Monocytes nonactive 0 0.04980661 Monocytes_0

10x_3288_t1_TGACCGCTGGGAGT-1 Patient5 Monocytes nonactive 4 0.06331241 Monocytes_4

10x_3288_t1_TGACCGCTTCTACT-1Patient5 Progenitors active 1 0.13310824 Progenitors_1

10x_3288_t1_TGACCGCTTGGTGT-1 Patient5 Progenitors active 1 0.13860884 Progenitors_1

10x_3288_t1_TGACGATGGAAAGT-1 Patient5 Monocytes nonactive 0 0.07429776 Monocytes_0

10x_3288_t1_TGACGATGGTGTCA-1 Patient5 Progenitors active 1 0.12987445 Progenitors_1

10x_3288_t1_TGACGATGTCTAGG-1 Patient5 Progenitors active 1 0.18808256 Progenitors_1

10x_3288_t1_TGACGATGTGCAAC-1 Patient5 Progenitors active 1 0.12610171 Progenitors_1

10x_3288_t1_TGACGCCTAACGAA-1 Patient5 Progenitors active 1 0.14823093 Progenitors_1

10x_3288_t1_TGACGCCTCCTCAC-1 Patient5 Monocytes nonactive 8 0.0274079 Monocytes_8

10x_3288_t1_TGACGCCTCTTGGA-1 Patient5 Monocytes nonactive 0 0.05115402 Monocytes_0

10x_3288_t1_TGACGCCTGTCGTA-1 Patient5 Monocytes nonactive 0 0.05262824 Monocytes_0

10x_3288_t1_TGACTGGAAGTAGA-1 Patient5 Monocytes nonactive 4 0.05278676 Monocytes_4

10x_3288_t1_TGACTGGATCACGA-1 Patient5 Monocytes nonactive 4 0.02992835 Monocytes_4

10x_3288_t1_TGACTGGATTATCC-1Patient5 Progenitors active 1 0.12960497 Progenitors_1

10x_3288_t1_TGACTTACGCATCA-1 Patient5 Monocytes nonactive 0 0.10153129 Monocytes_0

10x_3288_t1_TGACTTACGCTTCC-1 Patient5 Monocytes nonactive 0 0.05494262 Monocytes_0
10x_3288_t1_TGACTTACGGCGAA-1 Patient5 Progenitors active 1 0.16733245 Progenitors_1
10x_3288_t1_TGACTTACTCCAGA-1 Patient5 Progenitors active 1 0.16067466 Progenitors_1
10x_3288_t1_TGACTTACTGCGTA-1 Patient5 Progenitors active 1 0.21262127 Progenitors_1
10x_3288_t1_TGACTTTGAGGTTC-1 Patient5 Monocytes nonactive 0 0.10818908 Monocytes_0
10x_3288_t1_TGACTTTGCCTCAC-1 Patient5 Monocytes nonactive 0 0.06166381 Monocytes_0
10x_3288_t1_TGACTTTGCGTTAG-1 Patient5 Progenitors active 1 0.1210291 Progenitors_1
10x_3288_t1_TGACTTTGTCGCAA-1 Patient5 Progenitors active 6 0.1209974 Progenitors_6
10x_3288_t1_TGAGACACTGGAAA-1 Patient5 Progenitors active 1 0.12286792 Progenitors_1
10x_3288_t1_TGAGCAACCTGGAT-1 Patient5 Progenitors nonactive 1 0.09912181 Progenitors_1
10x_3288_t1_TGAGCAACTACAGC-1 Patient5 Progenitors active 1 0.15583983 Progenitors_1
10x_3288_t1_TGAGCAACTTCCCG-1 Patient5 Monocytes nonactive 4 0.04516201 Monocytes_4
10x_3288_t1_TGAGCTGAAGTGCT-1 Patient5 Monocytes nonactive 0 0.07185657 Monocytes_0
10x_3288_t1_TGAGCTGACTTATC-1 Patient5 Monocytes nonactive 0 0.05537062 Monocytes_0
10x_3288_t1_TGAGCTGAGTAAGA-1 Patient5 Monocytes nonactive 0 0.09100564 Monocytes_0
10x_3288_t1_TGAGCTGATGCCAA-1 Patient5 Progenitors active 1 0.14255596 Progenitors_1
10x_3288_t1_TGAGGACTCGGGAA-1 Patient5 Progenitors active 1 0.18814596

Progenitors_1
10x_3288_t1_TGAGGACTCTTACT-1 Patient5 Progenitors active 1 0.12442141
Progenitors_1
10x_3288_t1_TGAGGACTTCCCGT-1 Patient5 Progenitors active 1 0.15541183
Progenitors_1
10x_3288_t1_TGAGGACTTGAGGG-1 Patient5 Monocytes nonactive 4 0.02837487
Monocytes_4
10x_3288_t1_TGAGGTACTGCTTT-1 Patient5 Progenitors active 1 0.16658741
Progenitors_1
10x_3288_t1_TGAGTCGAGGATCT-1 Patient5 Monocytes nonactive 4 0.06331241
Monocytes_4
10x_3288_t1_TGAGTCGAGTAGGG-1 Patient5 Monocytes nonactive 0 0.02577516
Monocytes_0
10x_3288_t1_TGAGTCGATACTCT-1Patient5 Monocytes nonactive 0 0.0383457
Monocytes_0
10x_3288_t1_TGAGTCGATTTGCT-1Patient5 Monocytes nonactive 4 0.03404984
Monocytes_4
10x_3288_t1_TGATAAACGCTACA-1 Patient5 Progenitors active 1 0.18662418
Progenitors_1
10x_3288_t1_TGATAAACGTGAGG-1 Patient5 Progenitors active 1 0.14716885
Progenitors_1
10x_3288_t1_TGATAAACTGCTTT-1 Patient5 Monocytes nonactive 0 0.05974574
Monocytes_0
10x_3288_t1_TGATACCTCACTGA-1 Patient5 Monocytes nonactive 4 0.0290565
Monocytes_4
10x_3288_t1_TGATACCTCCTTCG-1Patient5 Monocytes nonactive 0 0.04116733
Monocytes_0
10x_3288_t1_TGATACCTGCTCCT-1 Patient5 Monocytes nonactive 16 0.06565849
Monocytes_16
10x_3288_t1_TGATACCTGTCGAT-1 Patient5 Progenitors active 1 0.13843447
Progenitors_1
10x_3288_t1_TGATATGAAAAAGC-1 Patient5 Monocytes nonactive 8 0.03263902
Monocytes_8

10x_3288_t1_TGATATGAACGTAC-1 Patient5 Monocytes nonactive 0 0.03720436
Monocytes_0
10x_3288_t1_TGATATGAGCTGTA-1Patient5 Progenitors active 1 0.14052692
Progenitors_1
10x_3288_t1_TGATCACTAACCTG-1 Patient5 Monocytes nonactive 4 0.03907488
Monocytes_4
10x_3288_t1_TGATCACTCCACCT-1Patient5 Monocytes nonactive 4 0.05483165
Monocytes_4
10x_3288_t1_TGATCACTCGGAGA-1 Patient5 Progenitors active 1 0.12037918
Progenitors_1
10x_3288_t1_TGATCACTCGTTGA-1 Patient5 Dendritic cells nonactive 10 0.07161879
Dendritic cells_10
10x_3288_t1_TGATCACTGAGATA-1 Patient5 Monocytes nonactive 0 0.03763236
Monocytes_0
10x_3288_t1_TGATCACTTATGGC-1Patient5 Progenitors active 1 0.14954664
Progenitors_1
10x_3288_t1_TGATCACTTCAGAC-1 Patient5 Monocytes nonactive 0 0.05021876
Monocytes_0
10x_3288_t1_TGATCACTTCTCAT-1 Patient5 Progenitors active 1 0.15590324
Progenitors_1
10x_3288_t1_TGATCGGAGGATTC-1 Patient5 Monocytes nonactive 0 0.05511699
Monocytes_0
10x_3288_t1_TGATCGGATATTCC-1 Patient5 Monocytes nonactive 4 0.04226111
Monocytes_4
10x_3288_t1_TGATTAGAAAACAG-1 Patient5 Monocytes nonactive 0 0.02393634
Monocytes_0
10x_3288_t1_TGATTAGAAACGGG-1 Patient5 Monocytes nonactive 0 0.07975081
Monocytes_0
10x_3288_t1_TGATTAGAGACGGA-1 Patient5 Progenitors active 1 0.12870141
Progenitors_1
10x_3288_t1_TGATTAGAGTGCAT-1Patient5 Monocytes nonactive 0 0.05850929
Monocytes_0
10x_3288_t1_TGATTAGATGCCAA-1 Patient5 Monocytes nonactive 0 0.02477649

Monocytes_0
10x_3288_t1_TGATTCACACGTAC-1 Patient5 Monocytes nonactive 4 0.01738951 Monocytes_4
10x_3288_t1_TGATTCACAGGCGA-1 Patient5 Progenitors active 6 0.1483736 Progenitors_6
10x_3288_t1_TGATTCACCACTTT-1 Patient5 Monocytes nonactive 4 0.08325407 Monocytes_4
10x_3288_t1_TGATTCACGCATCA-1 Patient5 Progenitors nonactive 1 0.1142128 Progenitors_1
10x_3288_t1_TGATTCACTCCAGA-1 Patient5 Monocytes nonactive 0 0.04268911 Monocytes_0
10x_3288_t1_TGATTCACTCCCAC-1 Patient5 Progenitors nonactive 1 0.10958405 Progenitors_1
10x_3288_t1_TGATTCTGAAGCCT-1 Patient5 Monocytes nonactive 0 0.05167713 Monocytes_0
10x_3288_t1_TGATTCTGCAGAGG-1 Patient5 Progenitors nonactive 1 0.11479932 Progenitors_1
10x_3288_t1_TGATTCTGCCTAAG-1 Patient5 Monocytes nonactive 0 0.06540486 Monocytes_0
10x_3288_t1_TGATTCTGCTGGTA-1 Patient5 Monocytes nonactive 15 0.06148944 Monocytes_15
10x_3288_t1_TGATTCTGGCAGTT-1Patient5 Progenitors active 1 0.18302581 Progenitors_1
10x_3288_t1_TGATTCTGTGTGGT-1Patient5 Progenitors nonactive 1 0.11418109 Progenitors_1
10x_3288_t1_TGCAACGAAGATCC-1 Patient5 Monocytes nonactive 16 0.08255659 Monocytes_16
10x_3288_t1_TGCAACGAGTCACA-1 Patient5 Monocytes nonactive 4 0.02144759 Monocytes_4
10x_3288_t1_TGCAACGATGCGTA-1 Patient5 Monocytes nonactive 0 0.05584617 Monocytes_0
10x_3288_t1_TGCAAGACAGATGA-1 Patient5 Monocytes nonactive 0 0.04679475 Monocytes_0

10x_3288_t1_TGCAAGACCGTAGT-1 Patient5 Monocytes nonactive 0 0.05717773 Monocytes_0
10x_3288_t1_TGCAAGACTCGTTT-1 Patient5 Monocytes nonactive 0 0.03832985 Monocytes_0
10x_3288_t1_TGCAAGTGGGACGA-1 Patient5 Monocytes nonactive 0 0.03141843 Monocytes_0
10x_3288_t1_TGCAAGTGGGTAAA-1 Patient5 Monocytes nonactive 0 0.05413417 Monocytes_0
10x_3288_t1_TGCAAGTGGGTGTT-1 Patient5 Monocytes nonactive 8 0.05703506 Monocytes_8
10x_3288_t1_TGCAAGTGTGGTGT-1 Patient5 Monocytes nonactive 0 0.02996005 Monocytes_0
10x_3288_t1_TGCAATCTCCTCCA-1 Patient5 Monocytes nonactive 8 0.01707247 Monocytes_8
10x_3288_t1_TGCAATCTCGAGAG-1 Patient5 Monocytes nonactive 8 0.05281846 Monocytes_8
10x_3288_t1_TGCAATCTCGCAAT-1Patient5 Monocytes nonactive 0 0.03067339 Monocytes_0
10x_3288_t1_TGCACAGAAAGTAG-1 Patient5 Monocytes nonactive 8 0.06080781 Monocytes_8
10x_3288_t1_TGCACGCTCAGATC-1 Patient5 Monocytes nonactive 4 0.0944455 Monocytes_4
10x_3288_t1_TGCACGCTGTTGAC-1 Patient5 Monocytes nonactive 0 0.04188067 Monocytes_0
10x_3288_t1_TGCACGCTTCACCC-1 Patient5 Monocytes nonactive 0 0.06624501 Monocytes_0
10x_3288_t1_TGCACGCTTCCCGT-1 Patient5 Dendritic cells active 10 0.12381903 Dendritic cells_10
10x_3288_t1_TGCACGCTTGGGAG-1 Patient5 Monocytes nonactive 4 0.01517025 Monocytes_4
10x_3288_t1_TGCAGATGAACCTG-1 Patient5 Monocytes nonactive 4 0.06767168 Monocytes_4
10x_3288_t1_TGCAGATGGGTCAT-1 Patient5 Progenitors active 1 0.15140131

Progenitors_1

10x_3288_t1_TGCATGGAACTTTC-1 Patient5 Monocytes nonactive 0 0.07600976 Monocytes_0

10x_3288_t1_TGCATGGATGTTTC-1Patient5 Monocytes nonactive 8 0.10235559 Monocytes_8

10x_3288_t1_TGCCAAGAGTAAGA-1 Patient5 Monocytes nonactive 0 0.05025046 Monocytes_0

10x_3288_t1_TGCCACTGACTGTG-1 Patient5 Progenitors active 1 0.14334855 Progenitors_1

10x_3288_t1_TGCCACTGTGCATG-1 Patient5 Progenitors nonactive 1 0.08504534 Progenitors_1

10x_3288_t1_TGCCACTGTTGAGC-1 Patient5 Monocytes nonactive 0 0.0337645 Monocytes_0

10x_3288_t1_TGCCAGCTCAAAGA-1 Patient5 Progenitors active 1 0.12115592 Progenitors_1

10x_3288_t1_TGCCAGCTCTAGTG-1 Patient5 Monocytes nonactive 4 0.03276584 Monocytes_4

10x_3288_t1_TGCCAGCTTGAGCT-1 Patient5 Monocytes nonactive 4 0.08767675 Monocytes_4

10x_3288_t1_TGCCCAACGCTAAC-1 Patient5 Progenitors active 1 0.13721387 Progenitors_1

10x_3288_t1_TGCCCAACGTTAGC-1 Patient5 Monocytes nonactive 4 0.0547841 Monocytes_4

10x_3288_t1_TGCCGACTAGGGTG-1 Patient5 Monocytes nonactive 4 0.06635597 Monocytes_4

10x_3288_t1_TGCCGACTCTACGA-1 Patient5 Monocytes nonactive 4 0.05251728 Monocytes_4

10x_3288_t1_TGCCGACTCTCAAG-1 Patient5 Progenitors active 1 0.12020481 Progenitors_1

10x_3288_t1_TGCCGACTCTTACT-1Patient5 Monocytes nonactive 4 0.04080274 Monocytes_4

10x_3288_t1_TGCCGACTGCTGAT-1 Patient5 Monocytes nonactive 0 0.0248399 Monocytes_0

10x_3288_t1_TGCCGACTTTGTCT-1Patient5 Monocytes nonactive 4 0.06976412
Monocytes_4
10x_3288_t1_TGCCGACTTTTGTC-1 Patient5 Progenitors active 1 0.1218534
Progenitors_1
10x_3288_t1_TGCGAAACACGACT-1 Patient5 Progenitors active 1 0.15923213
Progenitors_1
10x_3288_t1_TGCGAAACGCGTTA-1 Patient5 Monocytes nonactive 0 0.06099803
Monocytes_0
10x_3288_t1_TGCGAAACGGGTGA-1 Patient5 Monocytes nonactive 0 0.01499588
Monocytes_0
10x_3288_t1_TGCGATGAAGAAGT-1 Patient5 Monocytes nonactive 0 0.01743707
Monocytes_0
10x_3288_t1_TGCGATGATCCTCG-1 Patient5 Monocytes nonactive 0 0.02980153
Monocytes_0
10x_3288_t1_TGCGCACTCCCAAA-1 Patient5 Monocytes nonactive 0 0.04988587
Monocytes_0
10x_3288_t1_TGCGCACTCCTAAG-1 Patient5 Progenitors active 1 0.16730074
Progenitors_1
10x_3288_t1_TGCGCACTGCGTTA-1 Patient5 Monocytes nonactive 0 0.03764822
Monocytes_0
10x_3288_t1_TGCGTAGAAGCCTA-1 Patient5 Progenitors active 1 0.15428635
Progenitors_1
10x_3288_t1_TGCGTAGACATTTC-1 Patient5 Monocytes nonactive 0 0.03542895
Monocytes_0
10x_3288_t1_TGCGTAGACCTTGC-1 Patient5 Monocytes nonactive 0 0.05809714
Monocytes_0
10x_3288_t1_TGCGTAGATCCCAC-1 Patient5 Monocytes nonactive 15 0.06486589
Monocytes_15
10x_3288_t1_TGCGTAGATGAAGA-1 Patient5 Monocytes nonactive 4 0.06900323
Monocytes_4
10x_3288_t1_TGCTAGGACTTTAC-1 Patient5 Monocytes nonactive 4 0.07256991
Monocytes_4
10x_3288_t1_TGCTAGGAGACACT-1 Patient5 Progenitors active 1 0.17040771

Progenitors_1

10x_3288_t1_TGCTAGGAGTTGGT-1 Patient5 Monocytes nonactive 4 0.04917253 Monocytes_4

10x_3288_t1_TGCTATACCGCAAT-1 Patient5 Monocytes nonactive 4 0.05518039 Monocytes_4

10x_3288_t1_TGCTATACCTCAAG-1 Patient5 Progenitors active 1 0.15579228 Progenitors_1

10x_3288_t1_TGCTATACCTTCGC-1 Patient5 Monocytes nonactive 0 0.03530214 Monocytes_0

10x_3288_t1_TGCTGAGAAGCAAA-1 Patient5 Progenitors active 1 0.14913449 Progenitors_1

10x_3288_t1_TGCTTAACACTACG-1 Patient5 Progenitors active 1 0.1374041 Progenitors_1

10x_3288_t1_TGCTTAACCCAAGT-1 Patient5 Monocytes nonactive 0 0.03519117 Monocytes_0

10x_3288_t1_TGCTTAACCGAATC-1 Patient5 Progenitors active 1 0.12859045 Progenitors_1

10x_3288_t1_TGCTTAACGCGAAG-1 Patient5 Monocytes nonactive 0 0.01713588 Monocytes_0

10x_3288_t1_TGCTTAACTACTTC-1 Patient5 Monocytes nonactive 0 0.09059349 Monocytes_0

10x_3288_t1_TGCTTAACTCCTGC-1 Patient5 Progenitors active 1 0.12800393 Progenitors_1

10x_3288_t1_TGGAAAGAATCGTG-1 Patient5 Monocytes nonactive 4 0.05321476 Monocytes_4

10x_3288_t1_TGGAAAGAGTATCG-1 Patient5 Monocytes nonactive 0 0.03722021 Monocytes_0

10x_3288_t1_TGGAAAGAGTCACA-1 Patient5 Monocytes nonactive 4 0.03347917 Monocytes_4

10x_3288_t1_TGGAACACAAAAGC-1 Patient5 Monocytes nonactive 0 0.05378543 Monocytes_0

10x_3288_t1_TGGAACACAGGGTG-1 Patient5 Monocytes nonactive 0 0.04590704 Monocytes_0

10x_3288_t1_TGGAACACAGGTTC-1 Patient5 Progenitors nonactive 5 0.09704521
Progenitors_5
10x_3288_t1_TGGAACACATGTCG-1 Patient5 Progenitors nonactive 1 0.09824995
Progenitors_1
10x_3288_t1_TGGAACACTGAGCT-1 Patient5 Monocytes nonactive 4 0.06049077
Monocytes_4
10x_3288_t1_TGGAACACTTCAGG-1 Patient5 Monocytes nonactive 0 0.08764504
Monocytes_0
10x_3288_t1_TGGAACTGCTGTGA-1 Patient5 Progenitors active 1 0.19786317
Progenitors_1
10x_3288_t1_TGGAACTGTCAGTG-1 Patient5 Monocytes nonactive 4 0.0299125
Monocytes_4
10x_3288_t1_TGGAACTGTGCTGA-1 Patient5 Monocytes nonactive 4 0.05687655
Monocytes_4
10x_3288_t1_TGGAAGCTCCGAAT-1 Patient5 Monocytes nonactive 0 0.03078435
Monocytes_0
10x_3288_t1_TGGAAGCTCGTGAT-1 Patient5 Monocytes nonactive 4 0.05040898
Monocytes_4
10x_3288_t1_TGGAAGCTCTAGCA-1 Patient5 Dendritic cells nonactive 10 0.0872329
Dendritic cells_10
10x_3288_t1_TGGAAGCTGGACGA-1 Patient5 Monocytes nonactive 4 0.05340498
Monocytes_4
10x_3288_t1_TGGAAGCTGTCTGA-1 Patient5 Progenitors active 1 0.12765519
Progenitors_1
10x_3288_t1_TGGAAGCTTCGTAG-1 Patient5 Progenitors active 1 0.14279374
Progenitors_1
10x_3288_t1_TGGACCCTACACAC-1 Patient5 Monocytes nonactive 0 0.04034303
Monocytes_0
10x_3288_t1_TGGACCCTTATCGG-1 Patient5 Progenitors active 1 0.14785049
Progenitors_1
10x_3288_t1_TGGACTGAACACGT-1 Patient5 Monocytes nonactive 0 0.04975905
Monocytes_0
10x_3288_t1_TGGACTGAAGCCTA-1 Patient5 Progenitors active 1 0.11977681

Progenitors_1
10x_3288_t1_TGGACTGACACAAC-1 Patient5 Progenitors nonactive 1 0.10571619
Progenitors_1
10x_3288_t1_TGGACTGAGGTGAG-1 Patient5 Monocytes nonactive 4 0.03129161
Monocytes_4
10x_3288_t1_TGGACTGAGTACCA-1 Patient5 Monocytes nonactive 0 0.01965633
Monocytes_0
10x_3288_t1_TGGACTGATTACTC-1 Patient5 Progenitors active 1 0.14845286
Progenitors_1
10x_3288_t1_TGGAGACTTTAGGC-1 Patient5 Monocytes nonactive 0 0.06759242
Monocytes_0
10x_3288_t1_TGGAGGGACTCTCG-1 Patient5 Monocytes nonactive 0 0.05342084
Monocytes_0
10x_3288_t1_TGGAGGGATGCTCC-1 Patient5 Dendritic cells nonactive 10 0.04804705
Dendritic cells_10
10x_3288_t1_TGGAGGGATTGGTG-1 Patient5 Progenitors active 1 0.17855558
Progenitors_1
10x_3288_t1_TGGATGACAACCGT-1 Patient5 Dendritic cells active 10 0.11858791
Dendritic cells_10
10x_3288_t1_TGGATGACTCCTCG-1 Patient5 Progenitors active 1 0.19380509
Progenitors_1
10x_3288_t1_TGGATGTGAGTCTG-1 Patient5 Progenitors nonactive 1 0.10793545
Progenitors_1
10x_3288_t1_TGGATTCTAAGGCG-1 Patient5 Monocytes nonactive 8 0.05220024
Monocytes_8
10x_3288_t1_TGGATTCTCTACGA-1 Patient5 Monocytes nonactive 0 0.02621901
Monocytes_0
10x_3288_t1_TGGCAATGAGCCTA-1 Patient5 Monocytes nonactive 8 0.02049648
Monocytes_8
10x_3288_t1_TGGCAATGTGCCAA-1 Patient5 Monocytes nonactive 4 0.03859933
Monocytes_4
10x_3288_t1_TGGCACCTGTCGAT-1 Patient5 Monocytes nonactive 0 0.03932851
Monocytes_0

10x_3288_t1_TGGCACCTTCAAGC-1 Patient5 Monocytes nonactive 4 0.08661467 Monocytes_4
10x_3288_t1_TGGGTATGTATCGG-1 Patient5 Monocytes nonactive 0 0.02117811 Monocytes_0
10x_3288_t1_TGGGTATGTTCGGA-1 Patient5 Progenitors active 1 0.15460339 Progenitors_1
10x_3288_t1_TGGTACGAAACGGG-1 Patient5 Monocytes nonactive 0 0.060681 Monocytes_0
10x_3288_t1_TGGTACGAACTGTG-1 Patient5 Monocytes nonactive 13 0.06775094 Monocytes_13
10x_3288_t1_TGGTACGACACAAC-1 Patient5 Progenitors nonactive 1 0.11730391 Progenitors_1
10x_3288_t1_TGGTACGAGTACAC-1 Patient5 Monocytes nonactive 0 0.02918331 Monocytes_0
10x_3288_t1_TGGTACGATCACGA-1 Patient5 Progenitors active 1 0.12925623 Progenitors_1
10x_3288_t1_TGGTACGATGTTCT-1 Patient5 Progenitors active 1 0.16265614 Progenitors_1
10x_3288_t1_TGGTAGACAGCGTT-1 Patient5 Monocytes nonactive 15 0.08255659 Monocytes_15
10x_3288_t1_TGGTAGACGCGTTA-1 Patient5 Monocytes nonactive 4 0.02632997 Monocytes_4
10x_3288_t1_TGGTAGACGTCTAG-1 Patient5 Monocytes nonactive 0 0.0666413 Monocytes_0
10x_3288_t1_TGGTAGTGCAACCA-1 Patient5 Monocytes nonactive 0 0.06737049 Monocytes_0
10x_3288_t1_TGGTAGTGGAAAGT-1 Patient5 Monocytes nonactive 4 0.07734132 Monocytes_4
10x_3288_t1_TGGTAGTGTCCAAG-1 Patient5 Progenitors active 1 0.14005136 Progenitors_1
10x_3288_t1_TGGTAGTGTGCTAG-1 Patient5 Monocytes nonactive 4 0.03682392 Monocytes_4
10x_3288_t1_TGGTATCTACCTAG-1 Patient5 Progenitors active 1 0.13835521

Progenitors_1
10x_3288_t1_TGGTATCTACTGTG-1 Patient5 Monocytes nonactive 4 0.02591782
Monocytes_4
10x_3288_t1_TGGTCAGACCCACT-1 Patient5 Monocytes nonactive 4 0.06347093
Monocytes_4
10x_3288_t1_TGGTCAGATTGTGG-1 Patient5 Monocytes nonactive 0 0.04887135
Monocytes_0
10x_3288_t1_TGGTTACTATCGTG-1 Patient5 Progenitors active 1 0.1340752
Progenitors_1
10x_3288_t1_TGTAACCTAGTCGT-1 Patient5 Monocytes nonactive 8 0.00871853
Monocytes_8
10x_3288_t1_TGTAACCTCGCAAT-1 Patient5 Monocytes nonactive 0 0.03443028
Monocytes_0
10x_3288_t1_TGTAACCTTTCTAC-1 Patient5 Monocytes nonactive 13 0.04202333
Monocytes_13
10x_3288_t1_TGTAATGACAAAGA-1 Patient5 Progenitors active 1 0.14311077
Progenitors_1
10x_3288_t1_TGTAATGACTCATT-1 Patient5 Monocytes nonactive 0 0.07065183
Monocytes_0
10x_3288_t1_TGTAATGATTGCGA-1 Patient5 Monocytes nonactive 0 0.0543878
Monocytes_0
10x_3288_t1_TGTACTTGAGGAGC-1 Patient5 Monocytes nonactive 4 0.05904825
Monocytes_4
10x_3288_t1_TGTACTTGTAGCCA-1 Patient5 Monocytes nonactive 4 0.04046985
Monocytes_4
10x_3288_t1_TGTACTTGTTATCC-1 Patient5 Progenitors active 1 0.16344874
Progenitors_1
10x_3288_t1_TGTAGGTGAGCGGA-1 Patient5 Monocytes nonactive 0 0.0256959
Monocytes_0
10x_3288_t1_TGTAGGTGCCACCT-1 Patient5 Monocytes nonactive 8 0.07071524
Monocytes_8
10x_3288_t1_TGTAGTCTAGTGTC-1 Patient5 Monocytes nonactive 0 0.05784351
Monocytes_0

10x_3288_t1_TGTATCTGAGAATG-1 Patient5 Monocytes nonactive 0 0.05315135 Monocytes_0
10x_3288_t1_TGTATCTGGAAACA-1 Patient5 Monocytes nonactive 4 0.0742185 Monocytes_4
10x_3288_t1_TGTATCTGGCTAAC-1Patient5 Progenitors active 1 0.17704965 Progenitors_1
10x_3288_t1_TGTATCTGGGGAGT-1 Patient5 Monocytes nonactive 4 0.06107729 Monocytes_4
10x_3288_t1_TGTATCTGGGTTCA-1 Patient5 Monocytes nonactive 8 0.02961131 Monocytes_8
10x_3288_t1_TGTATCTGTTACCT-1 Patient5 Monocytes nonactive 0 0.02959546 Monocytes_0
10x_3288_t1_TGTATCTGTTTGGG-1Patient5 Monocytes nonactive 0 0.04516201 Monocytes_0
10x_3288_t1_TGTATGCTCTGCAA-1 Patient5 Monocytes nonactive 0 0.02620316 Monocytes_0
10x_3288_t1_TGTATGCTGACAAA-1 Patient5 Monocytes nonactive 0 0.10305307 Monocytes_0
10x_3288_t1_TGTATGCTTATGGC-1 Patient5 Monocytes nonactive 4 0.06462811 Monocytes_4
10x_3288_t1_TGTCAGGAAACCAC-1 Patient5 Monocytes nonactive 4 0.022795 Monocytes_4
10x_3288_t1_TGTCAGGAGAATGA-1 Patient5 Monocytes nonactive 0 0.07727792 Monocytes_0
10x_3288_t1_TGTCAGGAGGAAGC-1 Patient5 Monocytes nonactive 0 0.04603386 Monocytes_0
10x_3288_t1_TGTCTAACAAGGTA-1 Patient5 Progenitors active 1 0.13989284 Progenitors_1
10x_3288_t1_TGTCTAACACCAAC-1 Patient5 Progenitors active 1 0.12950986 Progenitors_1
10x_3288_t1_TGTCTAACCAGTCA-1 Patient5 Progenitors nonactive 1 0.0982658 Progenitors_1
10x_3288_t1_TGTCTAACGCTCCT-1Patient5 Monocytes nonactive 4 0.05925433

Monocytes_4
10x_3288_t1_TGTCTAACGCTGAT-1Patient5 Monocytes nonactive 0 0.05118572
Monocytes_0
10x_3288_t1_TGTCTAACTTAGGC-1 Patient5 Monocytes nonactive 0 0.06163211
Monocytes_0
10x_3288_t1_TGTGACGAGGTGTT-1 Patient5 Progenitors active 1 0.14217551
Progenitors_1
10x_3288_t1_TGTGACGATACTCT-1Patient5 Progenitors nonactive 1 0.09869381
Progenitors_1
10x_3288_t1_TGTGAGACCGTACA-1 Patient5 Monocytes nonactive 13 0.03527043
Monocytes_13
10x_3288_t1_TGTGAGACGAGGGT-1 Patient5 Monocytes nonactive 0 0.08585378
Monocytes_0
10x_3288_t1_TGTGAGACGCTTCC-1 Patient5 Monocytes nonactive 4 0.07746814
Monocytes_4
10x_3288_t1_TGTGAGACGGAACG-1 Patient5 Monocytes nonactive 4 0.04405237
Monocytes_4
10x_3288_t1_TGTGAGACTGAGGG-1 Patient5 Monocytes nonactive 0 0.04722275
Monocytes_0
10x_3288_t1_TGTGAGTGACGCAT-1 Patient5 Monocytes nonactive 4 0.08739141
Monocytes_4
10x_3288_t1_TGTGAGTGAGGGTG-1 Patient5 Progenitors active 1 0.15877243
Progenitors_1
10x_3288_t1_TGTGAGTGCCGATA-1 Patient5 Monocytes nonactive 4 0.06784605
Monocytes_4
10x_3288_t1_TGTGAGTGCTCGAA-1 Patient5 Monocytes nonactive 8 0.0362374
Monocytes_8
10x_3288_t1_TGTGAGTGGAATGA-1 Patient5 Monocytes nonactive 0 0.06456471
Monocytes_0
10x_3288_t1_TGTGAGTGTAGACC-1 Patient5 Monocytes nonactive 4 0.0438463
Monocytes_4
10x_3288_t1_TGTGATCTCCAGTA-1 Patient5 Monocytes nonactive 4 0.08599645
Monocytes_4

10x_3288_t1_TGTGATCTCTATGG-1 Patient5 Monocytes nonactive 8 0.09281276
Monocytes_8
10x_3288_t1_TGTGATCTCTTTAC-1 Patient5 Progenitors nonactive 1 0.10934627
Progenitors_1
10x_3288_t1_TGTGATCTTGAGCT-1 Patient5 Monocytes nonactive 4 0.04752394
Monocytes_4
10x_3288_t1_TGTGGATGATGTCG-1 Patient5 Progenitors active 1 0.14653478
Progenitors_1
10x_3288_t1_TGTGGATGGCTAAC-1 Patient5 Progenitors active 1 0.17237334
Progenitors_1
10x_3288_t1_TGTGGATGTACTCT-1 Patient5 Monocytes nonactive 0 0.05161372
Monocytes_0
10x_3288_t1_TGTTAAGACCACCT-1 Patient5 Progenitors nonactive 1 0.08333333
Progenitors_1
10x_3288_t1_TGTTAAGACTACTT-1 Patient5 Monocytes nonactive 4 0.03674466
Monocytes_4
10x_3288_t1_TGTTAAGAGAGCTT-1 Patient5 Monocytes nonactive 4 0.06632427
Monocytes_4
10x_3288_t1_TGTTAAGATACAGC-1 Patient5 Monocytes nonactive 0 0.08889734
Monocytes_0
10x_3288_t1_TGTTACACAGACTC-1 Patient5 Progenitors active 1 0.11838184
Progenitors_1
10x_3288_t1_TGTTACACGTAGGG-1 Patient5 Monocytes nonactive 8 0.04516201
Monocytes_8
10x_3288_t1_TGTTACTGCCTGAA-1 Patient5 Monocytes nonactive 4 0.01383869
Monocytes_4
10x_3288_t1_TGTTACTGCGATAC-1 Patient5 Monocytes nonactive 8 0.03384376
Monocytes_8
10x_3288_t1_TTAACCACGGTACT-1 Patient5 Monocytes nonactive 4 0.02810538
Monocytes_4
10x_3288_t1_TTACACACAACCGT-1 Patient5 Monocytes nonactive 0 0.04753979
Monocytes_0
10x_3288_t1_TTACACACAACTGC-1 Patient5 Monocytes nonactive 0 0.06393063

Monocytes_0
10x_3288_t1_TTACAGCTAACGAA-1 Patient5 Monocytes nonactive 0 0.02677383
Monocytes_0
10x_3288_t1_TTACCATGCCTTCG-1 Patient5 Monocytes nonactive 0 0.07704014
Monocytes_0
10x_3288_t1_TTACCATGCTTACT-1 Patient5 Monocytes nonactive 4 0.04942616
Monocytes_4
10x_3288_t1_TTACCATGGGAACG-1 Patient5 Progenitors active 1 0.13526409
Progenitors_1
10x_3288_t1_TTACCATGTGGTAC-1 Patient5 Dendritic cells active 10 0.15045019
Dendritic cells_10
10x_3288_t1_TTACGACTGACAGG-1 Patient5 Progenitors nonactive 1 0.11337265
Progenitors_1
10x_3288_t1_TTACGACTGCTATG-1 Patient5 Monocytes nonactive 4 0.08311141
Monocytes_4
10x_3288_t1_TTACGTACAATCGC-1 Patient5 Monocytes nonactive 4 0.03596792
Monocytes_4
10x_3288_t1_TTACGTACAGAAGT-1 Patient5 Monocytes nonactive 0 0.09539661
Monocytes_0
10x_3288_t1_TTACGTACGCGTAT-1 Patient5 Monocytes nonactive 4 0.03950289
Monocytes_4
10x_3288_t1_TTACGTACGGAAGC-1 Patient5 Progenitors active 1 0.15190857
Progenitors_1
10x_3288_t1_TTACGTACGTATGC-1 Patient5 Monocytes nonactive 0 0.03186228
Monocytes_0
10x_3288_t1_TTACGTACTGGATC-1 Patient5 Dendritic cells nonactive 10 0.05055165
Dendritic cells_10
10x_3288_t1_TTACTCGAAACGTC-1 Patient5 Monocytes nonactive 4 0.0455583
Monocytes_4
10x_3288_t1_TTACTCGACCCTCA-1 Patient5 Monocytes nonactive 0 0.02789931
Monocytes_0
10x_3288_t1_TTAGAATGGCCTTC-1 Patient5 Monocytes nonactive 4 0.03176717
Monocytes_4

10x_3288_t1_TTAGAATGGCTAAC-1 Patient5 Progenitors nonactive 1 0.10554182
Progenitors_1
10x_3288_t1_TTAGAATGTCTCTA-1 Patient5 Monocytes nonactive 4 0.02663116
Monocytes_4
10x_3288_t1_TTAGACCTAGGAGC-1 Patient5 Monocytes nonactive 4 0.04574853
Monocytes_4
10x_3288_t1_TTAGACCTTCCCAC-1 Patient5 Progenitors active 1 0.12488111
Progenitors_1
10x_3288_t1_TTAGACCTTCTCGC-1 Patient5 Monocytes nonactive 0 0.03977237
Monocytes_0
10x_3288_t1_TTAGACCTTTGACG-1 Patient5 Monocytes nonactive 8 0.02675797
Monocytes_8
10x_3288_t1_TTAGCTACAAAGCA-1 Patient5 Progenitors active 1 0.16795067
Progenitors_1
10x_3288_t1_TTAGCTACCCTTTA-1 Patient5 Monocytes nonactive 0 0.0358728
Monocytes_0
10x_3288_t1_TTAGCTACCTCAGA-1 Patient5 Monocytes nonactive 4 0.01426669
Monocytes_4
10x_3288_t1_TTAGGGTGACCAGT-1 Patient5 Progenitors active 1 0.18064802
Progenitors_1
10x_3288_t1_TTAGGGTGCCTGTC-1 Patient5 Monocytes nonactive 4 0.04942616
Monocytes_4
10x_3288_t1_TTAGGGTGTTTCGT-1 Patient5 Monocytes nonactive 0 0.05107476
Monocytes_0
10x_3288_t1_TTAGGTCTGAGGTG-1 Patient5 Monocytes nonactive 4 0.00110963
Monocytes_4
10x_3288_t1_TTAGGTCTGTAAAG-1 Patient5 Monocytes nonactive 0 0.04375119
Monocytes_0
10x_3288_t1_TTAGGTCTTGGATC-1 Patient5 Monocytes nonactive 0 0.06372456
Monocytes_0
10x_3288_t1_TTAGGTCTTGGCAT-1 Patient5 Monocytes nonactive 8 0.05232706
Monocytes_8
10x_3288_t1_TTAGTCACAGTCAC-1 Patient5 Monocytes nonactive 4 0.0362691

Monocytes_4
10x_3288_t1_TTAGTCACCAGGAG-1 Patient5 Progenitors active 1 0.15095745
Progenitors_1
10x_3288_t1_TTAGTCACCGCATA-1 Patient5 Monocytes nonactive 4 0.04086615
Monocytes_4
10x_3288_t1_TTAGTCACCTACGA-1 Patient5 Monocytes nonactive 0 0.04604971
Monocytes_0
10x_3288_t1_TTAGTCTGACCTCC-1 Patient5 Progenitors active 1 0.13147549
Progenitors_1
10x_3288_t1_TTAGTCTGCCGCTT-1 Patient5 Monocytes nonactive 4 0.05708262
Monocytes_4
10x_3288_t1_TTAGTCTGCTAGTG-1 Patient5 Monocytes nonactive 0 0.08071777
Monocytes_0
10x_3288_t1_TTAGTCTGGACTAC-1 Patient5 Monocytes nonactive 0 0.07897407
Monocytes_0
10x_3288_t1_TTAGTCTGTGTCCC-1 Patient5 Progenitors nonactive 1 0.10184833
Progenitors_1
10x_3288_t1_TTAGTCTGTTGTCT-1 Patient5 Monocytes nonactive 0 0.07325154
Monocytes_0
10x_3288_t1_TTATCCGAACTCAG-1 Patient5 Monocytes nonactive 13 0.03553992
Monocytes_13
10x_3288_t1_TTATCCGAGGATCT-1 Patient5 Monocytes nonactive 0 0.02686894
Monocytes_0
10x_3288_t1_TTATCCGAGTCCTC-1 Patient5 Monocytes nonactive 8 0.04241963
Monocytes_8
10x_3288_t1_TTATCCGATTCTTG-1 Patient5 Monocytes nonactive 0 0.07329909
Monocytes_0
10x_3288_t1_TTATGAGACCCTAC-1 Patient5 Monocytes nonactive 0 0.02845412
Monocytes_0
10x_3288_t1_TTATGAGAGTAAGA-1 Patient5 Progenitors active 1 0.14827849
Progenitors_1
10x_3288_t1_TTATGAGATCTCCG-1 Patient5 Progenitors active 1 0.13149134
Progenitors_1

10x_3288_t1_TTATGAGATTCCAT-1 Patient5 Monocytes nonactive 4 0.06312219
Monocytes_4
10x_3288_t1_TTATGCACATCGAC-1 Patient5 Progenitors active 1 0.14819923
Progenitors_1
10x_3288_t1_TTATGCACCCATGA-1 Patient5 Monocytes nonactive 13 0.07700843
Monocytes_13
10x_3288_t1_TTATGCACGACGTT-1 Patient5 Monocytes nonactive 0 0.03905903
Monocytes_0
10x_3288_t1_TTATGCACGGTCAT-1 Patient5 Monocytes nonactive 0 0.0387737
Monocytes_0
10x_3288_t1_TTATGCACGTACGT-1 Patient5 Monocytes nonactive 4 0.03450954
Monocytes_4
10x_3288_t1_TTATGCACGTTAGC-1 Patient5 Monocytes nonactive 8 0.04379874
Monocytes_8
10x_3288_t1_TTATGCACTGCTAG-1 Patient5 Monocytes nonactive 4 0.02900894
Monocytes_4
10x_3288_t1_TTATGGCTCTCCCA-1 Patient5 Monocytes nonactive 0 0.02423752
Monocytes_0
10x_3288_t1_TTATGGCTGACACT-1 Patient5 Monocytes nonactive 15 0.05329402
Monocytes_15
10x_3288_t1_TTATGGCTGATAAG-1 Patient5 Monocytes nonactive 0 0.09664891
Monocytes_0
10x_3288_t1_TTATGGCTTTCTCA-1 Patient5 Monocytes nonactive 4 0.04950542
Monocytes_4
10x_3288_t1_TTATTCCTGCTATG-1 Patient5 Progenitors active 1 0.16703126
Progenitors_1
10x_3288_t1_TTCAAAGAAAGTAG-1 Patient5 Progenitors nonactive 1 0.1079513
Progenitors_1
10x_3288_t1_TTCAAAGAACCAGT-1 Patient5 Progenitors active 1 0.18209055
Progenitors_1
10x_3288_t1_TTCAAAGAATTCGG-1 Patient5 Monocytes nonactive 4 0.04031133
Monocytes_4
10x_3288_t1_TTCAAAGATCTTTG-1 Patient5 Monocytes nonactive 0 0.04314882

Monocytes_0
10x_3288_t1_TTCAACACAACAGA-1 Patient5 Monocytes nonactive 4 0.04746053
Monocytes_4
10x_3288_t1_TTCAACACAAGTGA-1 Patient5 Monocytes nonactive 0 0.04883964
Monocytes_0
10x_3288_t1_TTCAACACGGTTTG-1 Patient5 Monocytes nonactive 0 0.0455583
Monocytes_0
10x_3288_t1_TTCAAGCTGTCGTA-1 Patient5 Monocytes nonactive 0 0.02774079
Monocytes_0
10x_3288_t1_TTCAAGCTGTTGGT-1 Patient5 Monocytes nonactive 0 0.05175639
Monocytes_0
10x_3288_t1_TTCAAGCTTGCTAG-1 Patient5 Progenitors active 1 0.18007736
Progenitors_1
10x_3288_t1_TTCAAGCTTGGAGG-1 Patient5 Progenitors active 6 0.14304737
Progenitors_6
10x_3288_t1_TTCACAACAACTGC-1 Patient5 Progenitors active 6 0.12678334
Progenitors_6
10x_3288_t1_TTCACAACCCAAGT-1 Patient5 Progenitors active 1 0.15354131
Progenitors_1
10x_3288_t1_TTCACAACCTCTAT-1 Patient5 Progenitors active 1 0.11900006
Progenitors_1
10x_3288_t1_TTCACAACGTTCGA-1 Patient5 Monocytes nonactive 0 0.02374612
Monocytes_0
10x_3288_t1_TTCACCCTACCAAC-1 Patient5 Monocytes nonactive 0 0.02704331
Monocytes_0
10x_3288_t1_TTCACCCTCCTTTA-1 Patient5 Progenitors active 1 0.14350707
Progenitors_1
10x_3288_t1_TTCACCCTCTACCC-1 Patient5 Progenitors active 1 0.1517976
Progenitors_1
10x_3288_t1_TTCACCCTTATCTC-1 Patient5 Monocytes nonactive 4 0.05789107
Monocytes_4
10x_3288_t1_TTCAGACTAGATCC-1 Patient5 Monocytes nonactive 0 0.02284256
Monocytes_0

10x_3288_t1_TTCAGACTCTCAAG-1 Patient5 Monocytes nonactive 8 0.06366115 Monocytes_8
10x_3288_t1_TTCAGACTTGTCAG-1 Patient5 Monocytes nonactive 0 0.05261239 Monocytes_0
10x_3288_t1_TTCAGTACAACTGC-1 Patient5 Monocytes nonactive 4 0.05335743 Monocytes_4
10x_3288_t1_TTCAGTTGCAGAAA-1 Patient5 Monocytes nonactive 4 0.03259147 Monocytes_4
10x_3288_t1_TTCAGTTGCTTGTT-1 Patient5 Progenitors active 1 0.13684928 Progenitors_1
10x_3288_t1_TTCAGTTGTAGTCG-1 Patient5 Progenitors active 1 0.19575487 Progenitors_1
10x_3288_t1_TTCAGTTGTGTTTC-1 Patient5 Progenitors active 1 0.1876387 Progenitors_1
10x_3288_t1_TTCATCGACTTAGG-1 Patient5 Progenitors active 1 0.16489126 Progenitors_1
10x_3288_t1_TTCATGACGCAGAG-1 Patient5 Monocytes nonactive 0 0.06006277 Monocytes_0
10x_3288_t1_TTCATGACTCTCGC-1 Patient5 Progenitors active 1 0.20165177 Progenitors_1
10x_3288_t1_TTCATGTGCCGCTT-1 Patient5 Progenitors active 1 0.14097077 Progenitors_1
10x_3288_t1_TTCATGTGTTCTGT-1 Patient5 Progenitors active 1 0.16218058 Progenitors_1
10x_3288_t1_TTCATTCTGACACT-1 Patient5 Progenitors active 1 0.12748082 Progenitors_1
10x_3288_t1_TTCATTCTTGTGAC-1 Patient5 Progenitors active 1 0.14233403 Progenitors_1
10x_3288_t1_TTCCAAACCACTTT-1 Patient5 Monocytes nonactive 0 0.04753979 Monocytes_0
10x_3288_t1_TTCCATGATGTGAC-1 Patient5 Monocytes nonactive 0 0.03806036 Monocytes_0
10x_3288_t1_TTCCCACTTCTCAT-1 Patient5 Monocytes nonactive 4 0.03766407

Monocytes_4
10x_3288_t1_TTCCCACTTTCCGC-1 Patient5 Monocytes nonactive 0 0.03859933
Monocytes_0
10x_3288_t1_TTCCTAGACGCCTT-1Patient5 Dendritic cells nonactive 10 0.08758164
Dendritic cells_10
10x_3288_t1_TTCCTAGAGAGCTT-1 Patient5 Monocytes nonactive 0 0.06104559
Monocytes_0
10x_3288_t1_TTCGAGGACGTGTA-1 Patient5 Progenitors nonactive 1 0.10040581
Progenitors_1
10x_3288_t1_TTCGAGGAGTATCG-1 Patient5 Progenitors active 1 0.15932725
Progenitors_1
10x_3288_t1_TTCGAGGAGTTGAC-1 Patient5 Progenitors nonactive 1 0.09626847
Progenitors_1
10x_3288_t1_TTCGAGGATAGTCG-1 Patient5 Progenitors active 1 0.13626276
Progenitors_1
10x_3288_t1_TTCGATTGATGTGC-1Patient5 Monocytes nonactive 4 0.01637499
Monocytes_4
10x_3288_t1_TTCGATTGGGTTCA-1 Patient5 Monocytes nonactive 4 0.02767738
Monocytes_4
10x_3288_t1_TTCGGAGAACCTGA-1 Patient5 Monocytes nonactive 4 0.03281339
Monocytes_4
10x_3288_t1_TTCGGAGACGTTAG-1 Patient5 Progenitors active 1 0.19453427
Progenitors_1
10x_3288_t1_TTCGGAGACTGTCC-1 Patient5 Monocytes nonactive 15 0.05823981
Monocytes_15
10x_3288_t1_TTCGTATGATCGGT-1 Patient5 Monocytes nonactive 0 0.04072348
Monocytes_0
10x_3288_t1_TTCGTATGGCTACA-1Patient5 Monocytes nonactive 0 0.06900323
Monocytes_0
10x_3288_t1_TTCGTATGGTACAC-1Patient5 Progenitors active 1 0.17983958
Progenitors_1
10x_3288_t1_TTCTACGAACGGGA-1 Patient5 Progenitors nonactive 1 0.11508465
Progenitors_1

10x_3288_t1_TTCTACGACTGTCC-1 Patient5 Monocytes nonactive 4 0.07849851 Monocytes_4
10x_3288_t1_TTCTACGATTGGTG-1 Patient5 Monocytes nonactive 0 0.06851183 Monocytes_0
10x_3288_t1_TTCTAGTGTCGCCT-1 Patient5 Monocytes nonactive 8 0.03485828 Monocytes_8
10x_3288_t1_TTCTCAGAAGCTAC-1 Patient5 Progenitors nonactive 1 0.10568448 Progenitors_1
10x_3288_t1_TTCTCAGACCTTTA-1 Patient5 Progenitors active 1 0.1328229 Progenitors_1
10x_3288_t1_TTCTCAGACTCGCT-1 Patient5 Monocytes nonactive 0 0.09347854 Monocytes_0
10x_3288_t1_TTCTCAGAGAATGA-1 Patient5 Monocytes nonactive 13 0.06729123 Monocytes_13
10x_3288_t1_TTCTCAGAGCATCA-1 Patient5 Progenitors active 1 0.15369983 Progenitors_1
10x_3288_t1_TTCTGATGACGGAG-1 Patient5 Monocytes nonactive 0 0.04597045 Monocytes_0
10x_3288_t1_TTCTGATGCCCACT-1 Patient5 Progenitors active 1 0.14158899 Progenitors_1
10x_3288_t1_TTCTGATGGAGAGC-1 Patient5 Progenitors active 1 0.18427811 Progenitors_1
10x_3288_t1_TTCTTACTTGCAAC-1 Patient5 Monocytes nonactive 0 0.02472893 Monocytes_0
10x_3288_t1_TTGAACCTATCTCT-1 Patient5 Monocytes nonactive 0 0.07848266 Monocytes_0
10x_3288_t1_TTGAACCTCATTGG-1 Patient5 Progenitors active 1 0.12537252 Progenitors_1
10x_3288_t1_TTGAACCTCTGCTC-1 Patient5 Monocytes nonactive 4 0.05376958 Monocytes_4
10x_3288_t1_TTGAACCTCTTGAG-1 Patient5 Monocytes nonactive 0 0.03446199 Monocytes_0
10x_3288_t1_TTGAACCTCTTGGA-1 Patient5 Progenitors active 1 0.17844461

Progenitors_1

10x_3288_t1_TTGAATGAAAAGTG-1 Patient5 Monocytes nonactive 15 0.04733371 Monocytes_15

10x_3288_t1_TTGAATGAACTAGC-1 Patient5 Progenitors active 1 0.14853212 Progenitors_1

10x_3288_t1_TTGAATGAGGTGTT-1 Patient5 Monocytes nonactive 0 0.06833745 Monocytes_0

10x_3288_t1_TTGACACTAGGGTG-1 Patient5 Monocytes nonactive 4 0.03601547 Monocytes_4

10x_3288_t1_TTGACACTATGGTC-1 Patient5 Progenitors active 1 0.17754106 Progenitors_1

10x_3288_t1_TTGACACTCTCAGA-1 Patient5 Progenitors active 1 0.1340435 Progenitors_1

10x_3288_t1_TTGACACTGCTTCC-1 Patient5 Monocytes nonactive 8 0.01404477 Monocytes_8

10x_3288_t1_TTGACACTTTCAGG-1 Patient5 Monocytes nonactive 0 0.07474161 Monocytes_0

10x_3288_t1_TTGAGGACACCATG-1 Patient5 Monocytes nonactive 0 0.10883901 Monocytes_0

10x_3288_t1_TTGAGGACCTCCAC-1 Patient5 Monocytes nonactive 0 0.03855177 Monocytes_0

10x_3288_t1_TTGAGGACTAGAAG-1 Patient5 Monocytes nonactive 0 0.0489189 Monocytes_0

10x_3288_t1_TTGAGGACTTCTGT-1 Patient5 Dendritic cells nonactive 10 0.1142445 Dendritic cells_10

10x_3288_t1_TTGATCTGCTTCTA-1 Patient5 Progenitors active 1 0.17750935 Progenitors_1

10x_3288_t1_TTGATCTGGCCATA-1 Patient5 Monocytes nonactive 0 0.04650942 Monocytes_0

10x_3288_t1_TTGATCTGGGTTCA-1 Patient5 Monocytes nonactive 0 0.04631919 Monocytes_0

10x_3288_t1_TTGCATTGATTTCC-1 Patient5 Monocytes nonactive 0 0.04012111 Monocytes_0

10x_3288_t1_TTGCATTGCTGACA-1 Patient5 Progenitors active 1 0.12243992
Progenitors_1
10x_3288_t1_TTGCATTGGCAAGG-1 Patient5 Monocytes nonactive 4 0.0585727
Monocytes_4
10x_3288_t1_TTGCATTGTCGTGA-1 Patient5 Monocytes nonactive 0 0.05121742
Monocytes_0
10x_3288_t1_TTGCATTGTGTAGC-1 Patient5 Monocytes nonactive 4 0.02228774
Monocytes_4
10x_3288_t1_TTGCATTGTTGCTT-1 Patient5 Monocytes nonactive 4 0.05205757
Monocytes_4
10x_3288_t1_TTGCTAACAAGTAG-1 Patient5 Progenitors active 1 0.12361296
Progenitors_1
10x_3288_t1_TTGCTAACCCACAA-1 Patient5 Monocytes nonactive 0 0.0274079
Monocytes_0
10x_3288_t1_TTGCTATGAGAGAT-1 Patient5 Monocytes nonactive 4 0.05681314
Monocytes_4
10x_3288_t1_TTGCTATGCGCATA-1 Patient5 Progenitors active 1 0.1374041
Progenitors_1
10x_3288_t1_TTGCTATGCTTAGG-1 Patient5 Monocytes nonactive 4 0.064105
Monocytes_4
10x_3288_t1_TTGGAGACAATCGC-1 Patient5 Progenitors active 1 0.13261683
Progenitors_1
10x_3288_t1_TTGGAGACACGGAG-1 Patient5 Monocytes nonactive 0 0.07242724
Monocytes_0
10x_3288_t1_TTGGAGTGAAGCAA-1 Patient5 Monocytes nonactive 0 0.05676558
Monocytes_0
10x_3288_t1_TTGGAGTGGGAAAT-1 Patient5 Monocytes nonactive 4 0.03745799
Monocytes_4
10x_3288_t1_TTGGAGTGGGATTC-1 Patient5 Monocytes nonactive 0 0.07119079
Monocytes_0
10x_3288_t1_TTGGTACTAAAGCA-1 Patient5 Monocytes nonactive 4 0.02653605
Monocytes_4
10x_3288_t1_TTGGTACTCAAAGA-1 Patient5 Monocytes nonactive 4 0.05202587

Monocytes_4
10x_3288_t1_TTGGTACTCAAGCT-1 Patient5 Monocytes nonactive 0 0.04311711
Monocytes_0
10x_3288_t1_TTGGTACTCCTTCG-1 Patient5 Monocytes nonactive 8 0.02818464
Monocytes_8
10x_3288_t1_TTGTACACGAAACA-1 Patient5 Monocytes nonactive 0 0.05827151
Monocytes_0
10x_3288_t1_TTGTACACTCCTAT-1 Patient5 Progenitors active 1 0.16573141
Progenitors_1
10x_3288_t1_TTGTACACTCTACT-1 Patient5 Monocytes nonactive 4 0.04337074
Monocytes_4
10x_3288_t1_TTGTACACTGTCAG-1 Patient5 Progenitors active 6 0.11755754
Progenitors_6
10x_3288_t1_TTGTAGCTCCAATG-1 Patient5 Progenitors active 1 0.18922389
Progenitors_1
10x_3288_t1_TTGTAGCTCTAGCA-1 Patient5 Monocytes nonactive 4 0.04373534
Monocytes_4
10x_3288_t1_TTGTAGCTGGGAGT-1 Patient5 Monocytes nonactive 4 0.04211845
Monocytes_4
10x_3288_t1_TTGTAGCTGGTGTT-1Patient5 Monocytes nonactive 0 0.04332319
Monocytes_0
10x_3288_t1_TTGTAGCTGTGCAT-1Patient5 Monocytes nonactive 8 0.07035064
Monocytes_8
10x_3288_t1_TTGTCATGCCAACA-1 Patient5 Monocytes nonactive 0 0.02639338
Monocytes_0
10x_3288_t1_TTGTCATGTTCGCC-1Patient5 Monocytes nonactive 0 0.02932598
Monocytes_0
10x_3288_t1_TTTAGAGAAAGAGT-1 Patient5 Monocytes nonactive 0 0.0480629
Monocytes_0
10x_3288_t1_TTTAGAGACCCTTG-1 Patient5 Monocytes nonactive 13 0.05427684
Monocytes_13
10x_3288_t1_TTTAGAGACTAGCA-1 Patient5 Monocytes nonactive 0 0.05242217
Monocytes_0

10x_3288_t1_TTTAGAGATGATGC-1 Patient5 Monocytes nonactive 4 0.02498256 Monocytes_4
10x_3288_t1_TTTAGAGATGCAAC-1 Patient5 Monocytes nonactive 4 0.06586456 Monocytes_4
10x_3288_t1_TTTAGAGATTCTGT-1 Patient5 Monocytes nonactive 4 0.06757656 Monocytes_4
10x_3288_t1_TTTAGCTGGTCATG-1 Patient5 Monocytes nonactive 4 0.09539661 Monocytes_4
10x_3288_t1_TTTAGCTGTGACTG-1 Patient5 Monocytes nonactive 0 0.03181472 Monocytes_0
10x_3288_t1_TTTAGGCTACGTAC-1 Patient5 Monocytes nonactive 0 0.06104559 Monocytes_0
10x_3288_t1_TTTAGGCTTCGACA-1 Patient5 Monocytes nonactive 0 0.04999683 Monocytes_0
10x_3288_t1_TTTATCCTCCCTCA-1 Patient5 Monocytes nonactive 0 0.04265741 Monocytes_0
10x_3288_t1_TTTATCCTCGATAC-1 Patient5 Monocytes nonactive 0 0.06802042 Monocytes_0
10x_3288_t1_TTTATCCTCTGTGA-1 Patient5 Progenitors active 1 0.13608839 Progenitors_1
10x_3288_t1_TTTCACGAATGTCG-1 Patient5 Monocytes nonactive 0 0.03858348 Monocytes_0
10x_3288_t1_TTTCACGACATTTC-1Patient5 Progenitors active 1 0.15536428 Progenitors_1
10x_3288_t1_TTTCACGACTCTCG-1 Patient5 Dendritic cells nonactive 10 0.1062393 Dendritic cells_10
10x_3288_t1_TTTCAGTGCTCTCG-1 Patient5 Progenitors active 1 0.14734322 Progenitors_1
10x_3288_t1_TTTCAGTGCTGGTA-1 Patient5 Monocytes nonactive 0 0.06599138 Monocytes_0
10x_3288_t1_TTTCAGTGGGACGA-1 Patient5 Monocytes nonactive 4 0.03024539 Monocytes_4
10x_3288_t1_TTTCCAGACTTTAC-1 Patient5 Monocytes nonactive 0 0.05297698

Monocytes_0

10x_3288_t1_TTTCCAGATTGGTG-1 Patient5 Monocytes nonactive 0 0.01648596 Monocytes_0

10x_3288_t1_TTTCGAACCGTTGA-1 Patient5 Monocytes nonactive 4 0.0261556 Monocytes_4

10x_3288_t1_TTTCGAACCTTGTT-1 Patient5 Monocytes nonactive 0 0.04478156 Monocytes_0

10x_3288_t1_TTTCTACTAACCGT-1 Patient5 Progenitors active 1 0.17012238 Progenitors_1

10x_3288_t1_TTTCTACTGATGAA-1 Patient5 Monocytes nonactive 0 0.05995181 Monocytes_0

10x_3288_t1_TTTCTACTGCTAAC-1 Patient5 Monocytes nonactive 4 0.03457295 Monocytes_4

10x_3288_t1_TTTCTACTTCCGTC-1 Patient5 Progenitors nonactive 1 0.1167808 Progenitors_1

10x_3288_t1_TTTGACTGAGATCC-1 Patient5 Progenitors active 1 0.13315579 Progenitors_1

10x_3288_t1_TTTGACTGCATGAC-1 Patient5 Monocytes nonactive 4 0.05074187 Monocytes_4

10x_3288_t1_TTTGACTGCTCCAC-1 Patient5 Monocytes nonactive 4 0.01994167 Monocytes_4

10x_3288_t1_TTTGCATGGCAGTT-1Patient5 Progenitors active 1 0.13055608 Progenitors_1

10x_3288_t1_TTTGCATGTTGTCT-1 Patient5 Progenitors active 1 0.12535667 Progenitors_1

10x_3288_t1_TTTGCATGTTTCGT-1 Patient5 Monocytes nonactive 8 0.0185467 Monocytes_8

10x_3288_t2_AAACATACAAGTGA-1 Patient5 Monocytes nonactive 2 0.02282671 Monocytes_2

10x_3288_t2_AAACATACTCCTAT-1 Patient5 Monocytes nonactive 2 0.01887959 Monocytes_2

10x_3288_t2_AAACATTGACCAGT-1 Patient5 B cells nonactive 7 0.07664384 B cells_7

10x_3288_t2_AAACATTGCTGACA-1 Patient5 Monocytes nonactive 2 0.03863103 Monocytes_2
10x_3288_t2_AAACCGTGACAGTC-1 Patient5 Monocytes nonactive 3 0.03133917 Monocytes_3
10x_3288_t2_AAACCGTGTGGTGT-1 Patient5 Monocytes nonactive 2 0.03820303 Monocytes_2
10x_3288_t2_AAACGCACAGAACA-1 Patient5 Monocytes nonactive 2 0.0531355 Monocytes_2
10x_3288_t2_AAACGCACATAAGG-1 Patient5 Monocytes nonactive 2 0.04769831 Monocytes_2
10x_3288_t2_AAACGCACCACACA-1 Patient5 Monocytes nonactive 3 0.02956376 Monocytes_3
10x_3288_t2_AAACGCACCTAAGC-1 Patient5 Monocytes nonactive 3 0.03991503 Monocytes_3
10x_3288_t2_AAACGCTGAGTACC-1 Patient5 Monocytes nonactive 2 0.06851183 Monocytes_2
10x_3288_t2_AAACGCTGCAAGCT-1 Patient5 Monocytes nonactive 2 0.06454886 Monocytes_2
10x_3288_t2_AAACGCTGCATTCT-1 Patient5 Monocytes nonactive 2 0.04332319 Monocytes_2
10x_3288_t2_AAACGCTGGTCTAG-1 Patient5 Monocytes nonactive 2 0.04478156 Monocytes_2
10x_3288_t2_AAACGCTGTGAAGA-1 Patient5 Monocytes nonactive 3 0.04923594 Monocytes_3
10x_3288_t2_AAACGGCTCGCATA-1 Patient5 Monocytes nonactive 2 0.02334982 Monocytes_2
10x_3288_t2_AAACGGCTCGGTAT-1 Patient5 Monocytes nonactive 2 0.02756642 Monocytes_2
10x_3288_t2_AAACTTGAGATAGA-1 Patient5 Monocytes nonactive 2 0.04421089 Monocytes_2
10x_3288_t2_AAAGACGAAGTGCT-1 Patient5 Monocytes nonactive 2 0.05822396 Monocytes_2
10x_3288_t2_AAAGACGAGCTTAG-1 Patient5 Monocytes nonactive 2 0.03691903

Monocytes_2
10x_3288_t2_AAAGACGATTAGGC-1 Patient5 Monocytes nonactive 2 0.07069938
Monocytes_2
10x_3288_t2_AAAGAGACTCAGGT-1 Patient5 Monocytes nonactive 3 0.04857016
Monocytes_3
10x_3288_t2_AAAGATCTGCTAAC-1 Patient5 Monocytes nonactive 2 0.02217678
Monocytes_2
10x_3288_t2_AAAGCAGACCCACT-1 Patient5 Monocytes nonactive 2 0.04593875
Monocytes_2
10x_3288_t2_AAAGCAGACTGTAG-1 Patient5 Monocytes nonactive 2 0.04238793
Monocytes_2
10x_3288_t2_AAAGCAGAGCGTTA-1 Patient5 Monocytes nonactive 2 0.03490584
Monocytes_2
10x_3288_t2_AAAGCAGAGCTACA-1 Patient5 Monocytes nonactive 2 0.07781688
Monocytes_2
10x_3288_t2_AAAGCCTGCAGATC-1 Patient5 Monocytes nonactive 2 0.01245958
Monocytes_2
10x_3288_t2_AAAGCCTGCTGAAC-1 Patient5 Monocytes nonactive 2 0.04539978
Monocytes_2
10x_3288_t2_AAAGCCTGGCCTTC-1 Patient5 Monocytes nonactive 2 0.03625325
Monocytes_2
10x_3288_t2_AAAGCCTGTGGTTG-1 Patient5 Progenitors nonactive 6 0.10825249
Progenitors_6
10x_3288_t2_AAAGGCCTACACGT-1 Patient5 Monocytes nonactive 3 0.03359013
Monocytes_3
10x_3288_t2_AAAGGCCTCGGAGA-1 Patient5 Monocytes nonactive 2 0.03528628
Monocytes_2
10x_3288_t2_AAAGGCCTGGAAAT-1 Patient5 Monocytes nonactive 2 0.02006848
Monocytes_2
10x_3288_t2_AAAGGCCTTAAGGA-1 Patient5 Monocytes nonactive 2 0.02052818
Monocytes_2
10x_3288_t2_AAAGTTTGACCCTC-1 Patient5 Monocytes nonactive 2 0.02964302
Monocytes_2

10x_3288_t2_AAAGTTTGATTTCC-1 Patient5 Monocytes nonactive 2 0.03664955 Monocytes_2
10x_3288_t2_AAATACTGACCAAC-1 Patient5 Monocytes nonactive 2 0.04947372 Monocytes_2
10x_3288_t2_AAATACTGGAATAG-1 Patient5 Monocytes nonactive 3 0.04501934 Monocytes_3
10x_3288_t2_AAATACTGTACGCA-1 Patient5 Monocytes nonactive 2 0.04955298 Monocytes_2
10x_3288_t2_AAATACTGTCTTAC-1Patient5 Monocytes nonactive 2 0.07924355 Monocytes_2
10x_3288_t2_AAATCAACCATTTC-1 Patient5 Monocytes nonactive 3 0.03874199 Monocytes_3
10x_3288_t2_AAATCAACGACGGA-1 Patient5 Monocytes nonactive 3 0.03804451 Monocytes_3
10x_3288_t2_AAATCATGAGAGGC-1 Patient5 Monocytes nonactive 3 0.0590324 Monocytes_3
10x_3288_t2_AAATCATGGCGGAA-1 Patient5 Monocytes nonactive 2 0.02495086 Monocytes_2
10x_3288_t2_AAATCATGGTTTCT-1 Patient5 Monocytes nonactive 2 0.03644347 Monocytes_2
10x_3288_t2_AAATCCCTCATTTC-1 Patient5 Monocytes nonactive 2 0.04617653 Monocytes_2
10x_3288_t2_AAATCCCTCTGACA-1 Patient5 Monocytes nonactive 2 0.03251221 Monocytes_2
10x_3288_t2_AAATCTGAACACAC-1 Patient5 Monocytes nonactive 3 0.02940524 Monocytes_3
10x_3288_t2_AAATCTGAGAGAGC-1 Patient5 Progenitors nonactive 6 0.10511382 Progenitors_6
10x_3288_t2_AAATCTGATAACCG-1 Patient5 Monocytes nonactive 3 0.04129415 Monocytes_3
10x_3288_t2_AAATCTGATGCCCT-1Patient5 Monocytes nonactive 2 0.00603957 Monocytes_2
10x_3288_t2_AAATGGGAAAAGTG-1 Patient5 Monocytes nonactive 2 0.0619333

Monocytes_2
10x_3288_t2_AAATGGGAAAGGTA-1 Patient5 Monocytes nonactive 2 0.08972164
Monocytes_2
10x_3288_t2_AAATGGGAGTACAC-1 Patient5 Monocytes nonactive 2 0.03866274
Monocytes_2
10x_3288_t2_AAATGTTGAGCTCA-1 Patient5 T cells nonactive 9 0.04283178 T
cells_9
10x_3288_t2_AAATGTTGGAATCC-1 Patient5 Monocytes nonactive 2 0.03607888
Monocytes_2
10x_3288_t2_AAATGTTGGTTTGG-1 Patient5 Monocytes nonactive 3 0.06150529
Monocytes_3
10x_3288_t2_AAATGTTGTCGCCT-1 Patient5 Monocytes nonactive 3 0.05846173
Monocytes_3
10x_3288_t2_AAATTGACAACCTG-1 Patient5 Monocytes nonactive 3 0.02006848
Monocytes_3
10x_3288_t2_AAATTGACCGTGTA-1 Patient5 Progenitors active 6 0.12451652
Progenitors_6
10x_3288_t2_AAATTGACGCGTTA-1 Patient5 Monocytes nonactive 2 0.01498003
Monocytes_2
10x_3288_t2_AACAATACAACCGT-1 Patient5 Monocytes nonactive 2 0.03145013
Monocytes_2
10x_3288_t2_AACAATACCTCTTA-1 Patient5 Monocytes nonactive 3 0.05047239
Monocytes_3
10x_3288_t2_AACAATACGAGAGC-1 Patient5 Monocytes nonactive 2 0.05147105
Monocytes_2
10x_3288_t2_AACAATACTGCGTA-1 Patient5 Monocytes nonactive 2 0.00726016
Monocytes_2
10x_3288_t2_AACACGTGCACTTT-1 Patient5 Monocytes nonactive 3 0.05546573
Monocytes_3
10x_3288_t2_AACACGTGCCAAGT-1 Patient5 Monocytes nonactive 2 0.0324488
Monocytes_2
10x_3288_t2_AACACGTGCTGATG-1 Patient5 Monocytes nonactive 3 0.03463636
Monocytes_3

10x_3288_t2_AACACGTGCTTACT-1 Patient5 Monocytes nonactive 2 0.01532877 Monocytes_2
10x_3288_t2_AACACGTGTGCTAG-1 Patient5 Monocytes nonactive 2 0.01995752 Monocytes_2
10x_3288_t2_AACACTCTCACTCC-1 Patient5 Monocytes nonactive 2 0.00982817 Monocytes_2
10x_3288_t2_AACACTCTGGTCAT-1 Patient5 Monocytes nonactive 2 0.0447023 Monocytes_2
10x_3288_t2_AACAGCACCACACA-1 Patient5 Monocytes nonactive 2 0.00745038 Monocytes_2
10x_3288_t2_AACAGCACCCATGA-1 Patient5 T cells nonactive 9 0.04898231 T cells_9
10x_3288_t2_AACAGCACGCGAGA-1 Patient5 Monocytes nonactive 2 0.04359267 Monocytes_2
10x_3288_t2_AACAGCACTCCCGT-1 Patient5 Monocytes nonactive 2 0.01705662 Monocytes_2
10x_3288_t2_AACAGCACTTTCAC-1 Patient5 Monocytes nonactive 2 0.01580432 Monocytes_2
10x_3288_t2_AACATATGAGGAGC-1 Patient5 Monocytes nonactive 2 0.07654873 Monocytes_2
10x_3288_t2_AACATTGATCCTGC-1 Patient5 Monocytes nonactive 2 0.03084776 Monocytes_2
10x_3288_t2_AACCACGACCTCAC-1 Patient5 Monocytes nonactive 3 0.0573521 Monocytes_3
10x_3288_t2_AACCACGAGTTTGG-1 Patient5 T cells nonactive 9 0.08506119 T cells_9
10x_3288_t2_AACCACGATGCTTT-1 Patient5 Monocytes nonactive 2 0.01469469 Monocytes_2
10x_3288_t2_AACCAGTGGAAACA-1 Patient5 Monocytes nonactive 3 0.06047492 Monocytes_3
10x_3288_t2_AACCCAGAAAGGTA-1 Patient5 Monocytes nonactive 2 0.04890305 Monocytes_2
10x_3288_t2_AACCCAGAACGACT-1 Patient5 Monocytes nonactive 2 0.02631412

Monocytes_2
10x_3288_t2_AACCCAGATCAAGC-1 Patient5 Monocytes nonactive 3 0.05467313
Monocytes_3
10x_3288_t2_AACCCAGATTCTTG-1 Patient5 Monocytes nonactive 2 0.05012364
Monocytes_2
10x_3288_t2_AACCGATGGCGGAA-1 Patient5 Monocytes nonactive 2 0.02910405
Monocytes_2
10x_3288_t2_AACCGATGTCGTGA-1 Patient5 Monocytes nonactive 2 0.05085283
Monocytes_2
10x_3288_t2_AACCGCCTATTCTC-1 Patient5 Monocytes nonactive 2 0.0712225
Monocytes_2
10x_3288_t2_AACCGCCTCTAGAC-1 Patient5 Monocytes nonactive 2 0.00442267
Monocytes_2
10x_3288_t2_AACCGCCTGGTGAG-1 Patient5 Monocytes nonactive 2 0.0447023
Monocytes_2
10x_3288_t2_AACCTACTCATCAG-1 Patient5 Monocytes nonactive 3 0.08076533
Monocytes_3
10x_3288_t2_AACCTACTGCTATG-1 Patient5 Monocytes nonactive 2 0.01767485
Monocytes_2
10x_3288_t2_AACCTACTTGGATC-1 Patient5 Monocytes active 3 0.12174244
Monocytes_3
10x_3288_t2_AACCTTACGTATGC-1 Patient5 Monocytes nonactive 2 0.03775918
Monocytes_2
10x_3288_t2_AACCTTACTGGTTG-1 Patient5 Monocytes nonactive 2 0.04370363
Monocytes_2
10x_3288_t2_AACGCAACATCGTG-1 Patient5 Monocytes nonactive 3 0.04837994
Monocytes_3
10x_3288_t2_AACGCAACCTATGG-1 Patient5 Monocytes nonactive 2 0.03550821
Monocytes_2
10x_3288_t2_AACGCATGAGAGAT-1 Patient5 Monocytes nonactive 2 0.05508528
Monocytes_2
10x_3288_t2_AACGCCCTGTGCTA-1 Patient5 Monocytes nonactive 2 0.03652273
Monocytes_2

10x_3288_t2_AACGGTACAAGTAG-1 Patient5 Monocytes nonactive 2 0.04210259 Monocytes_2
10x_3288_t2_AACGGTACAGAACA-1 Patient5 Monocytes nonactive 2 0.03573014 Monocytes_2
10x_3288_t2_AACGGTACAGAGAT-1 Patient5 Monocytes nonactive 2 0.03676051 Monocytes_2
10x_3288_t2_AACGGTACCCCTCA-1 Patient5 Monocytes nonactive 2 0.03871029 Monocytes_2
10x_3288_t2_AACGGTTGTCTTCA-1 Patient5 Monocytes nonactive 2 0.03397058 Monocytes_2
10x_3288_t2_AACGTCGAGGGACA-1 Patient5 Monocytes nonactive 3 0.0527075 Monocytes_3
10x_3288_t2_AACGTCGATCTTCA-1 Patient5 Monocytes nonactive 2 0.0665779 Monocytes_2
10x_3288_t2_AACGTGTGAGTTCG-1 Patient5 Monocytes nonactive 2 0.03574599 Monocytes_2
10x_3288_t2_AACGTGTGCACACA-1 Patient5 Monocytes nonactive 2 0.06172722 Monocytes_2
10x_3288_t2_AACGTGTGCCAATG-1 Patient5 Monocytes nonactive 2 0.01746877 Monocytes_2
10x_3288_t2_AACGTGTGCGTGTA-1 Patient5 Monocytes nonactive 2 0.04257815 Monocytes_2
10x_3288_t2_AACGTGTGTGACAC-1 Patient5 Monocytes nonactive 2 0.07607317 Monocytes_2
10x_3288_t2_AACGTTCTCCTCAC-1 Patient5 Monocytes nonactive 3 0.01892714 Monocytes_3
10x_3288_t2_AACGTTCTCTGGTA-1 Patient5 T cells nonactive 9 0.07073109 T cells_9
10x_3288_t2_AACGTTCTGACAAA-1 Patient5 Progenitors nonactive 6 0.08287363 Progenitors_6
10x_3288_t2_AACGTTCTGGAACG-1 Patient5 Monocytes nonactive 2 0.05072602 Monocytes_2
10x_3288_t2_AACGTTCTTTCCCG-1 Patient5 Monocytes nonactive 2 0.07420265

Monocytes_2
10x_3288_t2_AACTACCTGCTAAC-1 Patient5 Monocytes nonactive 2 0.03229028 Monocytes_2
10x_3288_t2_AACTACCTGTTCAG-1 Patient5 Monocytes nonactive 3 0.02816879 Monocytes_3
10x_3288_t2_AACTACCTTGGCAT-1 Patient5 Monocytes nonactive 2 0.07244309 Monocytes_2
10x_3288_t2_AACTCACTACCAGT-1 Patient5 Monocytes nonactive 2 0.02415827 Monocytes_2
10x_3288_t2_AACTCACTCGCAAT-1 Patient5 Monocytes nonactive 3 0.06104559 Monocytes_3
10x_3288_t2_AACTCACTTAACCG-1 Patient5 Monocytes nonactive 2 0.0316245 Monocytes_2
10x_3288_t2_AACTCGGAAACGTC-1 Patient5 Monocytes nonactive 2 0.02636168 Monocytes_2
10x_3288_t2_AACTCGGACAACCA-1 Patient5 T cells nonactive 9 0.06716442 T cells_9
10x_3288_t2_AACTCGGACCTGAA-1 Patient5 Monocytes nonactive 2 0.04893475 Monocytes_2
10x_3288_t2_AACTCTTGATGTCG-1 Patient5 Monocytes nonactive 3 0.06120411 Monocytes_3
10x_3288_t2_AACTGTCTATGGTC-1 Patient5 Monocytes nonactive 2 0.03276584 Monocytes_2
10x_3288_t2_AACTGTCTTGAAGA-1 Patient5 Monocytes nonactive 2 0.01946611 Monocytes_2
10x_3288_t2_AACTTGCTACCTTT-1 Patient5 Monocytes nonactive 2 0.03341576 Monocytes_2
10x_3288_t2_AACTTGCTATTGGC-1 Patient5 Monocytes nonactive 2 0.05741551 Monocytes_2
10x_3288_t2_AAGAACGACGAGTT-1 Patient5 Monocytes nonactive 3 0.08276267 Monocytes_3
10x_3288_t2_AAGAACGAGACTAC-1 Patient5 Monocytes nonactive 2 0.02396804 Monocytes_2

10x_3288_t2_AAGAACGAGTTGGT-1	Patient5 Monocytes	nonactive	2	0.03463636	Monocytes_2
10x_3288_t2_AAGAAGACCTCGCT-1	Patient5 Monocytes	nonactive	2	0.02873946	Monocytes_2
10x_3288_t2_AAGAAGACTACAGC-1	Patient5 Monocytes	nonactive	3	0.0189747	Monocytes_3
10x_3288_t2_AAGAAGACTCTATC-1	Patient5 Monocytes	nonactive	2	0.01986241	Monocytes_2
10x_3288_t2_AAGAATCTACCTAG-1	Patient5 Monocytes	nonactive	2	0.04107222	Monocytes_2
10x_3288_t2_AAGAATCTGAGACG-1	Patient5 Monocytes	nonactive	3	0.01589944	Monocytes_3
10x_3288_t2_AAGAATCTGTTACG-1	Patient5 Monocytes	nonactive	3	0.05123328	Monocytes_3
10x_3288_t2_AAGACAGAATTCGG-1	Patient5 Monocytes	nonactive	2	0.02609219	Monocytes_2
10x_3288_t2_AAGACAGAGAATGA-1	Patient5 Monocytes	nonactive	2	0.01526536	Monocytes_2
10x_3288_t2_AAGACAGATTTACC-1	Patient5 Monocytes	nonactive	3	0.01420328	Monocytes_3
10x_3288_t2_AAGAGATGAAGTAG-1	Patient5 Monocytes	nonactive	3	0.05950796	Monocytes_3
10x_3288_t2_AAGAGATGCGGAGA-1	Patient5 Monocytes	nonactive	2	0.02564834	Monocytes_2
10x_3288_t2_AAGAGATGTATGCG-1	Patient5 Monocytes	nonactive	2	0.02282671	Monocytes_2
10x_3288_t2_AAGATGGATGCACA-1	Patient5 Monocytes	nonactive	2	0.03308287	Monocytes_2
10x_3288_t2_AAGATGGATTGCGA-1	Patient5 Monocytes	nonactive	3	0.06393063	Monocytes_3
10x_3288_t2_AAGCAAGACTCTCG-1	Patient5 Monocytes	nonactive	3	0.03649103	Monocytes_3
10x_3288_t2_AAGCACTGACGGGA-1	Patient5 Monocytes	nonactive	2	0.06348678	

Monocytes_2
10x_3288_t2_AAGCACTGATCTTC-1 Patient5 Monocytes nonactive 2 0.05619491 Monocytes_2
10x_3288_t2_AAGCACTGCTCAAG-1 Patient5 Monocytes nonactive 3 0.04831653 Monocytes_3
10x_3288_t2_AAGCACTGGAGGTG-1 Patient5 Monocytes nonactive 3 0.03425591 Monocytes_3
10x_3288_t2_AAGCACTGTCATTC-1 Patient5 Monocytes nonactive 2 0.03339991 Monocytes_2
10x_3288_t2_AAGCACTGTGTAGC-1 Patient5 Monocytes nonactive 2 0.02553738 Monocytes_2
10x_3288_t2_AAGCCAACCGCCTT-1 Patient5 Monocytes nonactive 3 0.03682392 Monocytes_3
10x_3288_t2_AAGCCATGACACGT-1 Patient5 Monocytes nonactive 2 0.06451715 Monocytes_2
10x_3288_t2_AAGCCTGACAATCG-1 Patient5 Monocytes nonactive 2 0.00917824 Monocytes_2
10x_3288_t2_AAGCGACTAACGTC-1 Patient5 Monocytes nonactive 3 0.04273667 Monocytes_3
10x_3288_t2_AAGCGTACTAGACC-1 Patient5 Monocytes nonactive 3 0.04893475 Monocytes_3
10x_3288_t2_AAGGCTACAAGATG-1 Patient5 Monocytes nonactive 2 0.05670217 Monocytes_2
10x_3288_t2_AAGGCTACAGAAGT-1 Patient5 Monocytes nonactive 2 0.02320715 Monocytes_2
10x_3288_t2_AAGGCTACAGCAAA-1 Patient5 Monocytes nonactive 2 0 Monocytes_2
10x_3288_t2_AAGGCTACCAGTTG-1 Patient5 Monocytes nonactive 3 0.04375119 Monocytes_3
10x_3288_t2_AAGGCTACTCTTCA-1 Patient5 Monocytes nonactive 2 0.04861772 Monocytes_2
10x_3288_t2_AAGGCTTGGTACGT-1 Patient5 Monocytes nonactive 2 0.05245387 Monocytes_2

10x_3288_t2_AAGGTCACCGAGTT-1 Patient5 Monocytes nonactive 2 0.06941538
Monocytes_2
10x_3288_t2_AAGGTCACGTCGAT-1 Patient5 Monocytes nonactive 3 0.05178809
Monocytes_3
10x_3288_t2_AAGGTCTGAACCAC-1 Patient5 Monocytes nonactive 3 0.04657282
Monocytes_3
10x_3288_t2_AAGGTCTGCTTGTT-1 Patient5 Monocytes nonactive 2 0.04544734
Monocytes_2
10x_3288_t2_AAGGTCTGGTAAGA-1 Patient5 Monocytes nonactive 3 0.02918331
Monocytes_3
10x_3288_t2_AAGGTCTGGTTGCA-1 Patient5 Monocytes nonactive 2 0.07412339
Monocytes_2
10x_3288_t2_AAGGTCTGGTTGTG-1 Patient5 Monocytes nonactive 3 0.05153446
Monocytes_3
10x_3288_t2_AAGGTCTGTATCTC-1 Patient5 Monocytes nonactive 2 0.01176209
Monocytes_2
10x_3288_t2_AAGGTCTGTGGTCA-1 Patient5 Monocytes nonactive 2 0.03481073
Monocytes_2
10x_3288_t2_AAGGTCTGTTGCTT-1 Patient5 Monocytes nonactive 2 0.03555577
Monocytes_2
10x_3288_t2_AAGGTGCTCACAAC-1 Patient5 Monocytes nonactive 2 0.08904001
Monocytes_2
10x_3288_t2_AAGGTGCTCAGCTA-1 Patient5 Monocytes nonactive 2 0.06830575
Monocytes_2
10x_3288_t2_AAGTAACTAACGAA-1 Patient5 Monocytes nonactive 2 0.0539598
Monocytes_2
10x_3288_t2_AAGTAACTAGGGTG-1 Patient5 Monocytes nonactive 2 0.04321222
Monocytes_2
10x_3288_t2_AAGTAACTCTCGAA-1 Patient5 Monocytes nonactive 2 0.05847759
Monocytes_2
10x_3288_t2_AAGTAACTGCGTTA-1 Patient5 Monocytes nonactive 2 0.04435356
Monocytes_2
10x_3288_t2_AAGTAACTTATCTC-1Patient5 Monocytes nonactive 2 0.02097204

Monocytes_2
10x_3288_t2_AAGTAACTTATGCG-1 Patient5 Monocytes nonactive 3 0.03314628
Monocytes_3
10x_3288_t2_AAGTAACTTTCAGG-1 Patient5 Monocytes nonactive 3 0.05440365
Monocytes_3
10x_3288_t2_AAGTAGGACTAGCA-1 Patient5 Monocytes nonactive 3 0.0257276
Monocytes_3
10x_3288_t2_AAGTAGGATCCTGC-1 Patient5 Monocytes nonactive 2 0.03271828
Monocytes_2
10x_3288_t2_AAGTATACGTCGTA-1 Patient5 Monocytes nonactive 2 0.04647771
Monocytes_2
10x_3288_t2_AAGTCCGAAGGGTG-1 Patient5 Monocytes nonactive 2 0.03054657
Monocytes_2
10x_3288_t2_AAGTCCGACCATGA-1 Patient5 Monocytes nonactive 2 0.03051487
Monocytes_2
10x_3288_t2_AAGTCCGACTTAGG-1 Patient5 Monocytes nonactive 2 0.05264409
Monocytes_2
10x_3288_t2_AAGTCCGAGCTTCC-1 Patient5 Monocytes nonactive 3 0.05608395
Monocytes_3
10x_3288_t2_AAGTCCGATCGTTT-1 Patient5 Monocytes nonactive 2 0.0316562
Monocytes_2
10x_3288_t2_AAGTCTCTCTGGTA-1 Patient5 Monocytes nonactive 2 0.05823981
Monocytes_2
10x_3288_t2_AAGTCTCTGTCACA-1 Patient5 Monocytes nonactive 2 0.04593875
Monocytes_2
10x_3288_t2_AAGTCTCTGTCTAG-1 Patient5 Monocytes nonactive 2 0.00466045
Monocytes_2
10x_3288_t2_AAGTGCACAATGCC-1 Patient5 Monocytes nonactive 3 0.02667871
Monocytes_3
10x_3288_t2_AAGTGCACCGCCTT-1 Patient5 Monocytes nonactive 2 0.03661784
Monocytes_2
10x_3288_t2_AAGTGGCTGGAGGT-1 Patient5 Monocytes nonactive 2 0.04164289
Monocytes_2

10x_3288_t2_AAGTGGCTGGTACT-1 Patient5 Monocytes nonactive 2 0.03194154 Monocytes_2
10x_3288_t2_AAGTGGCTTGTGGT-1 Patient5 Monocytes nonactive 3 0.00924165 Monocytes_3
10x_3288_t2_AAGTTATGCCGTTC-1 Patient5 T cells nonactive 9 0.07770592 T cells_9
10x_3288_t2_AAGTTATGGTGAGG-1 Patient5 Monocytes nonactive 2 0.07734132 Monocytes_2
10x_3288_t2_AAGTTCCTTGAACC-1 Patient5 Monocytes nonactive 2 0.03208421 Monocytes_2
10x_3288_t2_AATAACACCTTCCG-1 Patient5 Monocytes nonactive 3 0.06041151 Monocytes_3
10x_3288_t2_AATAACACGAATAG-1 Patient5 Monocytes nonactive 2 0.02976983 Monocytes_2
10x_3288_t2_AATAAGCTCTCATT-1 Patient5 Monocytes nonactive 3 0.0362691 Monocytes_3
10x_3288_t2_AATAAGCTTATCGG-1 Patient5 Monocytes nonactive 3 0.03777503 Monocytes_3
10x_3288_t2_AATACCCTACCTGA-1 Patient5 Monocytes nonactive 3 0.07626339 Monocytes_3
10x_3288_t2_AATACCCTCGTGAT-1 Patient5 Monocytes nonactive 2 0.05186735 Monocytes_2
10x_3288_t2_AATACCCTGCCTTC-1 Patient5 Monocytes nonactive 2 0.03722021 Monocytes_2
10x_3288_t2_AATACCCTGTACGT-1 Patient5 Monocytes nonactive 3 0.05833492 Monocytes_3
10x_3288_t2_AATACTGAACGCTA-1 Patient5 Monocytes nonactive 2 0.05980914 Monocytes_2
10x_3288_t2_AATACTGAAGTCGT-1 Patient5 Monocytes nonactive 3 0.08731216 Monocytes_3
10x_3288_t2_AATACTGACTCAAG-1 Patient5 Monocytes nonactive 2 0.05936529 Monocytes_2
10x_3288_t2_AATAGGGACTTGTT-1 Patient5 Monocytes nonactive 2 0.05424513

Monocytes_2
10x_3288_t2_AATAGGGAGACGGA-1 Patient5 Monocytes nonactive 2 0.08070192 Monocytes_2
10x_3288_t2_AATATCGATGTCGA-1 Patient5 T cells nonactive 9 0.04150022 T cells_9
10x_3288_t2_AATCAAACAAGGTA-1 Patient5 Monocytes nonactive 3 0.06867034 Monocytes_3
10x_3288_t2_AATCCGGACCTAAG-1 Patient5 Monocytes nonactive 2 0.0354131 Monocytes_2
10x_3288_t2_AATCCGGACGACAT-1 Patient5 Monocytes nonactive 2 0.02854924 Monocytes_2
10x_3288_t2_AATCCGGACGGGAA-1 Patient5 Monocytes nonactive 2 0.06840086 Monocytes_2
10x_3288_t2_AATCCTACCTCTTA-1 Patient5 Monocytes nonactive 2 0.01312536 Monocytes_2
10x_3288_t2_AATCCTACGCTACA-1 Patient5 Monocytes nonactive 3 0.03552406 Monocytes_3
10x_3288_t2_AATCCTACTACTTC-1 Patient5 Monocytes nonactive 3 0.07404413 Monocytes_3
10x_3288_t2_AATCCTACTTCTCA-1 Patient5 Monocytes nonactive 2 0.05235876 Monocytes_2
10x_3288_t2_AATCCTTGACACAC-1 Patient5 Progenitors active 6 0.13526409 Progenitors_6
10x_3288_t2_AATCCTTGACGGGA-1 Patient5 Monocytes nonactive 2 0.06037981 Monocytes_2
10x_3288_t2_AATCCTTGCTAAGC-1 Patient5 Monocytes nonactive 2 0.02449115 Monocytes_2
10x_3288_t2_AATCCTTGGACGTT-1 Patient5 Monocytes nonactive 3 0.0582081 Monocytes_3
10x_3288_t2_AATCCTTGTAGCCA-1 Patient5 Monocytes nonactive 3 0.04425845 Monocytes_3
10x_3288_t2_AATCGGTGTATTCC-1 Patient5 Monocytes nonactive 2 0.02292182 Monocytes_2

10x_3288_t2_AATCTAGAACTACG-1 Patient5 Monocytes nonactive 2 0.02774079 Monocytes_2
10x_3288_t2_AATCTAGAAGGTCT-1 Patient5 T cells nonactive 9 0.02144759 T cells_9
10x_3288_t2_AATCTAGACCCTAC-1 Patient5 Monocytes nonactive 2 0.01388625 Monocytes_2
10x_3288_t2_AATCTAGACCTGTC-1 Patient5 Monocytes nonactive 2 0.04525712 Monocytes_2
10x_3288_t2_AATCTAGAGGACAG-1 Patient5 Monocytes nonactive 2 0.08006785 Monocytes_2
10x_3288_t2_AATCTAGATCTACT-1 Patient5 Monocytes nonactive 2 0.01111217 Monocytes_2
10x_3288_t2_AATCTCACAGTAGA-1 Patient5 Monocytes nonactive 2 0.04880794 Monocytes_2
10x_3288_t2_AATCTCACGGAGCA-1 Patient5 Monocytes nonactive 2 0.03839325 Monocytes_2
10x_3288_t2_AATCTCACGGATCT-1Patient5 Monocytes nonactive 2 0.03065754 Monocytes_2
10x_3288_t2_AATCTCTGATCTCT-1 Patient5 Monocytes nonactive 2 0.04026378 Monocytes_2
10x_3288_t2_AATCTCTGCCCACT-1Patient5 Monocytes nonactive 2 0.04662038 Monocytes_2
10x_3288_t2_AATCTCTGGTCAAC-1 Patient5 Monocytes nonactive 2 0.04184896 Monocytes_2
10x_3288_t2_AATGAGGACTACTT-1 Patient5 Monocytes nonactive 3 0.05202587 Monocytes_3
10x_3288_t2_AATGAGGATCGTTT-1 Patient5 Monocytes nonactive 2 0.05256483 Monocytes_2
10x_3288_t2_AATGATACCCCTTG-1 Patient5 Monocytes nonactive 2 0.02750301 Monocytes_2
10x_3288_t2_AATGATACTTCGGA-1 Patient5 Monocytes nonactive 2 0.02160611 Monocytes_2
10x_3288_t2_AATGCGTGCCCGTT-1 Patient5 Monocytes nonactive 2 0.06776679

Monocytes_2
10x_3288_t2_AATGCGTGGAAGGC-1 Patient5 Monocytes nonactive 3 0.04782512 Monocytes_3
10x_3288_t2_AATGCGTGGACGTT-1 Patient5 Monocytes nonactive 3 0.06705345 Monocytes_3
10x_3288_t2_AATGGAGAAGAAGT-1 Patient5 Monocytes nonactive 2 0.01277662 Monocytes_2
10x_3288_t2_AATGGAGAGCGTTA-1 Patient5 Monocytes nonactive 2 0.07055672 Monocytes_2
10x_3288_t2_AATGGCTGCCTGTC-1 Patient5 Monocytes nonactive 3 0.0337328 Monocytes_3
10x_3288_t2_AATGTAACGGGAGT-1 Patient5 Monocytes nonactive 2 0.03907488 Monocytes_2
10x_3288_t2_AATGTCCTACTGGT-1Patient5 Monocytes nonactive 3 0.02352419 Monocytes_3
10x_3288_t2_AATGTTGAACCATG-1 Patient5 Dendritic cells nonactive 14 0.10161055 Dendritic cells_14
10x_3288_t2_AATGTTGAATCTTC-1Patient5 Monocytes nonactive 3 0.01355336 Monocytes_3
10x_3288_t2_AATGTTGAGCCCTT-1 Patient5 Monocytes nonactive 3 0.08059096 Monocytes_3
10x_3288_t2_AATGTTGAGTAGCT-1 Patient5 Monocytes nonactive 2 0.04494008 Monocytes_2
10x_3288_t2_AATTACGAACGACT-1 Patient5 Monocytes nonactive 2 0.02106715 Monocytes_2
10x_3288_t2_AATTACGACGTAGT-1 Patient5 Monocytes nonactive 2 0.02048063 Monocytes_2
10x_3288_t2_AATTCCTGACCAAC-1 Patient5 Monocytes nonactive 2 0.01658107 Monocytes_2
10x_3288_t2_AATTCCTGCCTTGC-1 Patient5 T cells nonactive 9 0.10909264 T cells_9
10x_3288_t2_AATTGATGCATTCT-1 Patient5 Monocytes nonactive 2 0.02678968 Monocytes_2

10x_3288_t2_AATTGATGCGTCTC-1 Patient5 Monocytes nonactive 3 0.05594128 Monocytes_3
10x_3288_t2_AATTGATGGTCGAT-1 Patient5 Monocytes nonactive 3 0.09419187 Monocytes_3
10x_3288_t2_AATTGATGTGTTTC-1 Patient5 Monocytes nonactive 2 0.04571682 Monocytes_2
10x_3288_t2_AATTGTGACACCAA-1 Patient5 Monocytes nonactive 2 0.04831653 Monocytes_2
10x_3288_t2_AATTGTGATCTGGA-1 Patient5 Monocytes nonactive 3 0.04963224 Monocytes_3
10x_3288_t2_ACAAAGGAGGACTT-1 Patient5 Monocytes nonactive 2 0.04154778 Monocytes_2
10x_3288_t2_ACAAATTGAGCAAA-1 Patient5 Monocytes nonactive 3 0.03899562 Monocytes_3
10x_3288_t2_ACAAATTGCAGCTA-1 Patient5 Monocytes nonactive 2 0.02794686 Monocytes_2
10x_3288_t2_ACAAATTGCATTCT-1 Patient5 T cells nonactive 9 0.03974066 T cells_9
10x_3288_t2_ACAAGAGAAGAGAT-1 Patient5 Dendritic cells nonactive 10 0.06148944 Dendritic cells_10
10x_3288_t2_ACAAGAGACATACG-1 Patient5 Monocytes nonactive 2 0.080908 Monocytes_2
10x_3288_t2_ACAATAACCCCGTT-1 Patient5 Monocytes nonactive 2 0.04150022 Monocytes_2
10x_3288_t2_ACAATAACGGCATT-1 Patient5 Monocytes nonactive 3 0.02691649 Monocytes_3
10x_3288_t2_ACAATCCTGGGAGT-1 Patient5 Monocytes nonactive 2 0.03160865 Monocytes_2
10x_3288_t2_ACAATCCTTCATTC-1 Patient5 Monocytes nonactive 2 0.03419251 Monocytes_2
10x_3288_t2_ACAATCCTTGCCTC-1 Patient5 Monocytes nonactive 2 0.05080528 Monocytes_2
10x_3288_t2_ACAATCCTTGGTTG-1 Patient5 Monocytes nonactive 2 0.01696151 Monocytes_2

10x_3288_t2_ACAATTGAAGCGTT-1 Patient5 Monocytes nonactive 2 0.02902479 Monocytes_2
10x_3288_t2_ACAATTGAATCGTG-1 Patient5 Monocytes nonactive 2 0.01789677 Monocytes_2
10x_3288_t2_ACAATTGAGCCCTT-1 Patient5 Monocytes nonactive 2 0.0611407 Monocytes_2
10x_3288_t2_ACACAGACCCGTAA-1 Patient5 Monocytes nonactive 3 0.03100628 Monocytes_3
10x_3288_t2_ACACAGACCTGCAA-1 Patient5 Monocytes nonactive 2 0.07577199 Monocytes_2
10x_3288_t2_ACACAGACCTTGGA-1 Patient5 Monocytes nonactive 3 0.03825059 Monocytes_3
10x_3288_t2_ACACATCTCTCGCT-1Patient5 Monocytes nonactive 3 0.04814216 Monocytes_3
10x_3288_t2_ACACATCTTGCATG-1 Patient5 T cells nonactive 9 0.06061759 T cells_9
10x_3288_t2_ACACCAGAAAGAAC-1 Patient5 Monocytes nonactive 3 0.07680236 Monocytes_3
10x_3288_t2_ACACCAGAAGAGGC-1 Patient5 Monocytes nonactive 3 0.05616321 Monocytes_3
10x_3288_t2_ACACCAGACGGGAA-1 Patient5 Monocytes nonactive 2 0.03790185 Monocytes_2
10x_3288_t2_ACACCAGAGGTATC-1 Patient5 Monocytes nonactive 2 0.06294782 Monocytes_2
10x_3288_t2_ACACCCTGAGAAGT-1 Patient5 Monocytes nonactive 3 0.06824234 Monocytes_3
10x_3288_t2_ACACCCTGTAAGCC-1 Patient5 Monocytes nonactive 3 0.04925179 Monocytes_3
10x_3288_t2_ACACCCTGTCTGGA-1 Patient5 Monocytes nonactive 3 0.04753979 Monocytes_3
10x_3288_t2_ACACCCTGTCTTTG-1 Patient5 Monocytes nonactive 2 0.05736795 Monocytes_2
10x_3288_t2_ACACGAACGACACT-1 Patient5 Monocytes nonactive 2 0.0463826

Monocytes_2
10x_3288_t2_ACACGTGAATAAGG-1 Patient5 Monocytes nonactive 2 0.02672627
Monocytes_2
10x_3288_t2_ACACGTGAGTCGAT-1 Patient5 Monocytes nonactive 2 0.042594
Monocytes_2
10x_3288_t2_ACACGTGATCTATC-1 Patient5 Monocytes nonactive 2 0.06085537
Monocytes_2
10x_3288_t2_ACAGACACCCAGTA-1 Patient5 Monocytes nonactive 3 0.01176209
Monocytes_3
10x_3288_t2_ACAGACACCCTGAA-1 Patient5 Monocytes nonactive 2 0.04782512
Monocytes_2
10x_3288_t2_ACAGACACGAGGCA-1 Patient5 Monocytes nonactive 2 0.04218185
Monocytes_2
10x_3288_t2_ACAGACACTGTTTC-1 Patient5 Monocytes nonactive 2 0.03683977
Monocytes_2
10x_3288_t2_ACAGCAACGCGTTA-1 Patient5 Monocytes nonactive 2 0.02656775
Monocytes_2
10x_3288_t2_ACAGCAACGTAAGA-1 Patient5 Monocytes nonactive 2 0.0261556
Monocytes_2
10x_3288_t2_ACAGGTACAAGTAG-1 Patient5 Monocytes nonactive 2 0.02162196
Monocytes_2
10x_3288_t2_ACAGGTACTCCTCG-1 Patient5 Monocytes nonactive 2 0.02753472
Monocytes_2
10x_3288_t2_ACAGGTACTTCATC-1 Patient5 Monocytes nonactive 2 0.03059413
Monocytes_2
10x_3288_t2_ACAGTCGACCTTGC-1 Patient5 Monocytes nonactive 2 0.04400482
Monocytes_2
10x_3288_t2_ACAGTGACCCCAAA-1 Patient5 Monocytes nonactive 2 0.03314628
Monocytes_2
10x_3288_t2_ACAGTGACGGGAGT-1 Patient5 Monocytes nonactive 3 0.03612643
Monocytes_3
10x_3288_t2_ACAGTGTGATCTCT-1 Patient5 Monocytes nonactive 2 0.05448291
Monocytes_2

10x_3288_t2_ACAGTGTGCTTATC-1	Patient5 Monocytes	nonactive	3	0.03737873
Monocytes_3
10x_3288_t2_ACAGTGTGGAACCT-1	Patient5 Monocytes	nonactive	3	0.05158202
Monocytes_3
10x_3288_t2_ACAGTTCTACTACG-1	Patient5 Monocytes	nonactive	2	0.0564644
Monocytes_2
10x_3288_t2_ACAGTTCTATCGAC-1	Patient5 Monocytes	nonactive	3	0.040454
Monocytes_3
10x_3288_t2_ACAGTTCTGAGGTG-1	Patient5 Monocytes	nonactive	3	0.06897153
Monocytes_3
10x_3288_t2_ACAGTTCTTAAGGA-1	Patient5 Monocytes	nonactive	2	0.06145774
Monocytes_2
10x_3288_t2_ACATCACTCTCTTA-1Patient5 Monocytes	nonactive	3	0.03970896
Monocytes_3
10x_3288_t2_ACATCACTCTGACA-1	Patient5 Monocytes	nonactive	2	0.0181504
Monocytes_2
10x_3288_t2_ACATCACTTTGAGC-1	Patient5 Monocytes	nonactive	3	0.06432693
Monocytes_3
10x_3288_t2_ACATGGTGAGCTCA-1	Patient5 Monocytes	nonactive	2	0.04151607
Monocytes_2
10x_3288_t2_ACATGGTGATGCCA-1	Patient5 Monocytes	nonactive	2	0.03500095
Monocytes_2
10x_3288_t2_ACATGGTGCAAGCT-1	Patient5 Monocytes	nonactive	2	0.04660453
Monocytes_2
10x_3288_t2_ACATGGTGTCAGAC-1	Patient5 Monocytes	nonactive	2	0.01889544
Monocytes_2
10x_3288_t2_ACATTCTGAGGAGC-1	Patient5 Monocytes	nonactive	2	0.03179887
Monocytes_2
10x_3288_t2_ACCAACGACCCAAA-1	Patient5 Monocytes	nonactive	3	0.05854099
Monocytes_3
10x_3288_t2_ACCACAGAACCCTC-1	Patient5 Monocytes	nonactive	2	0.03078435
Monocytes_2
10x_3288_t2_ACCACAGAACGTTG-1	Patient5 Monocytes	nonactive	2	0

Monocytes_2
10x_3288_t2_ACCACAGACCTCAC-1 Patient5 Monocytes nonactive 2 0.01998922 Monocytes_2
10x_3288_t2_ACCACAGAGCTTCC-1 Patient5 T cells nonactive 9 0.08710608 T cells_9
10x_3288_t2_ACCACCTGGCCCTT-1 Patient5 Monocytes nonactive 2 0.04080274 Monocytes_2
10x_3288_t2_ACCACCTGTAGCCA-1 Patient5 Monocytes nonactive 3 0.05086868 Monocytes_3
10x_3288_t2_ACCACCTGTCATTC-1 Patient5 Monocytes nonactive 2 0.04506689 Monocytes_2
10x_3288_t2_ACCACGCTAAGCCT-1 Patient5 Monocytes nonactive 3 0.03267072 Monocytes_3
10x_3288_t2_ACCACGCTACGCAT-1 Patient5 Monocytes nonactive 2 0.03845666 Monocytes_2
10x_3288_t2_ACCACGCTCATTTC-1 Patient5 Monocytes nonactive 3 0.03178302 Monocytes_3
10x_3288_t2_ACCACGCTCTGTGA-1 Patient5 Monocytes nonactive 2 0.02818464 Monocytes_2
10x_3288_t2_ACCAGCCTAGTCGT-1 Patient5 Monocytes nonactive 2 0.01938685 Monocytes_2
10x_3288_t2_ACCAGCCTCCTTCG-1 Patient5 Monocytes nonactive 2 0.04292689 Monocytes_2
10x_3288_t2_ACCAGCCTTAAGCC-1 Patient5 Monocytes nonactive 2 0.04330734 Monocytes_2
10x_3288_t2_ACCAGTGAAGGTTC-1 Patient5 Monocytes nonactive 2 0.04405237 Monocytes_2
10x_3288_t2_ACCAGTGAATCGAC-1 Patient5 Monocytes nonactive 3 0.0417697 Monocytes_3
10x_3288_t2_ACCAGTGAGGTGAG-1 Patient5 Monocytes nonactive 3 0.03788599 Monocytes_3
10x_3288_t2_ACCATTACAAAAGC-1 Patient5 Monocytes nonactive 2 0.043878 Monocytes_2

10x_3288_t2_ACCATTACACAGCT-1 Patient5 Monocytes nonactive 2 0.02052818 Monocytes_2
10x_3288_t2_ACCATTACGCCTTC-1 Patient5 Monocytes nonactive 2 0.04194407 Monocytes_2
10x_3288_t2_ACCATTACGGATCT-1 Patient5 Monocytes nonactive 2 0.02320715 Monocytes_2
10x_3288_t2_ACCATTACTCGCAA-1 Patient5 Monocytes nonactive 3 0.06335996 Monocytes_3
10x_3288_t2_ACCATTACTGCTAG-1 Patient5 Monocytes nonactive 3 0.0358094 Monocytes_3
10x_3288_t2_ACCATTTGGTGTAC-1 Patient5 Monocytes nonactive 3 0.05955551 Monocytes_3
10x_3288_t2_ACCCAAGACGACTA-1 Patient5 Monocytes nonactive 2 0.05909581 Monocytes_2
10x_3288_t2_ACCCAAGACGCCTT-1 Patient5 Monocytes nonactive 2 0.03817133 Monocytes_2
10x_3288_t2_ACCCAAGAGAACTC-1 Patient5 Monocytes nonactive 2 0.01868937 Monocytes_2
10x_3288_t2_ACCCACTGATCGAC-1 Patient5 Monocytes nonactive 2 0.02414241 Monocytes_2
10x_3288_t2_ACCCACTGGGAGCA-1 Patient5 Monocytes nonactive 3 0.05792277 Monocytes_3
10x_3288_t2_ACCCACTGTGGAAA-1 Patient5 Monocytes nonactive 3 0.05359521 Monocytes_3
10x_3288_t2_ACCCGTACATTTCC-1 Patient5 Monocytes nonactive 3 0.05847759 Monocytes_3
10x_3288_t2_ACCCGTACTGCGTA-1 Patient5 Monocytes nonactive 2 0.05568765 Monocytes_2
10x_3288_t2_ACCCGTTGGTAGCT-1 Patient5 Monocytes nonactive 3 0.04739712 Monocytes_3
10x_3288_t2_ACCCGTTGTGAGAA-1 Patient5 Monocytes nonactive 2 0.02257308 Monocytes_2
10x_3288_t2_ACCCTCGAGAAGGC-1 Patient5 Progenitors nonactive 6 0.09798047

Progenitors_6
10x_3288_t2_ACCCTCGAGCGTTA-1 Patient5 Monocytes nonactive 3 0.05516454 Monocytes_3
10x_3288_t2_ACCCTCGAGGAGGT-1 Patient5 Monocytes nonactive 2 0.00058652 Monocytes_2
10x_3288_t2_ACCGAAACACCATG-1 Patient5 Monocytes nonactive 2 0.04639845 Monocytes_2
10x_3288_t2_ACCGCGGACGTAGT-1 Patient5 Monocytes nonactive 3 0.0691776 Monocytes_3
10x_3288_t2_ACCGCGGACTTGAG-1 Patient5 Monocytes nonactive 2 0.04145267 Monocytes_2
10x_3288_t2_ACCGTGCTCATGAC-1 Patient5 Monocytes nonactive 3 0.05340498 Monocytes_3
10x_3288_t2_ACCGTGCTCCTAAG-1 Patient5 Monocytes nonactive 2 0.05323061 Monocytes_2
10x_3288_t2_ACCTATTGACTAGC-1 Patient5 Monocytes nonactive 2 0.06082366 Monocytes_2
10x_3288_t2_ACCTATTGAGAACA-1 Patient5 T cells nonactive 9 0.10725382 T cells_9
10x_3288_t2_ACCTATTGATGACC-1 Patient5 Monocytes nonactive 2 0.01255469 Monocytes_2
10x_3288_t2_ACCTATTGCTGATG-1 Patient5 Monocytes nonactive 2 0.04108807 Monocytes_2
10x_3288_t2_ACCTCCGAAGATCC-1 Patient5 Monocytes nonactive 2 0.01501173 Monocytes_2
10x_3288_t2_ACCTCCGACACACA-1 Patient5 Monocytes nonactive 2 0.042594 Monocytes_2
10x_3288_t2_ACCTCCGACTGCAA-1 Patient5 Monocytes nonactive 2 0.03652273 Monocytes_2
10x_3288_t2_ACCTCCGAGGATCT-1 Patient5 Monocytes nonactive 2 0.01791262 Monocytes_2
10x_3288_t2_ACCTCCGAGGTAGG-1 Patient5 Monocytes nonactive 2 0.02921501 Monocytes_2

10x_3288_t2_ACCTCCGAGTTACG-1 Patient5 Monocytes nonactive 3 0.04589119 Monocytes_3
10x_3288_t2_ACCTCGTGACGGTT-1 Patient5 Monocytes nonactive 2 0.03977237 Monocytes_2
10x_3288_t2_ACCTCGTGCACCAA-1 Patient5 Monocytes nonactive 2 0.02418997 Monocytes_2
10x_3288_t2_ACCTCGTGCTCCCA-1 Patient5 Monocytes nonactive 2 0.02472893 Monocytes_2
10x_3288_t2_ACCTCGTGGGTAAA-1 Patient5 T cells nonactive 9 0.10113499 T cells_9
10x_3288_t2_ACCTCGTGTTCATC-1 Patient5 Progenitors nonactive 6 0.07410754 Progenitors_6
10x_3288_t2_ACGAACACCATTGG-1 Patient5 Monocytes nonactive 2 0.03882125 Monocytes_2
10x_3288_t2_ACGAACACGAACCT-1 Patient5 Monocytes nonactive 3 0.04024792 Monocytes_3
10x_3288_t2_ACGAACTGACAGTC-1 Patient5 Monocytes nonactive 2 0.02119396 Monocytes_2
10x_3288_t2_ACGAACTGACCTAG-1 Patient5 Monocytes nonactive 2 0.05162957 Monocytes_2
10x_3288_t2_ACGAACTGCAACTG-1 Patient5 Monocytes nonactive 2 0.03696658 Monocytes_2
10x_3288_t2_ACGAACTGGTTGGT-1 Patient5 Monocytes nonactive 2 0.04308541 Monocytes_2
10x_3288_t2_ACGACAACTTCATC-1 Patient5 Monocytes nonactive 2 0.05933359 Monocytes_2
10x_3288_t2_ACGAGGGAAAGGTA-1 Patient5 Monocytes nonactive 2 0.04367193 Monocytes_2
10x_3288_t2_ACGAGGGAACCCTC-1 Patient5 Monocytes nonactive 2 0.06315389 Monocytes_2
10x_3288_t2_ACGAGGGACGTCTC-1 Patient5 Monocytes nonactive 3 0.04501934 Monocytes_3
10x_3288_t2_ACGAGGGAGTTTCT-1 Patient5 Monocytes nonactive 2 0.02588612

Monocytes_2
10x_3288_t2_ACGAGTACGGACGA-1 Patient5 Monocytes nonactive 2 0.03929681 Monocytes_2
10x_3288_t2_ACGAGTACGTACCA-1 Patient5 Monocytes nonactive 2 0.01998922 Monocytes_2
10x_3288_t2_ACGATCGACCGATA-1 Patient5 Monocytes nonactive 2 0.02764568 Monocytes_2
10x_3288_t2_ACGATCGACGGGAA-1 Patient5 Monocytes nonactive 2 0.0476349 Monocytes_2
10x_3288_t2_ACGATCGAGAGCTT-1 Patient5 Monocytes nonactive 2 0.064533 Monocytes_2
10x_3288_t2_ACGATCGAGTCACA-1 Patient5 Monocytes nonactive 2 0.01146091 Monocytes_2
10x_3288_t2_ACGATGACAGTTCG-1 Patient5 Monocytes nonactive 2 0.02320715 Monocytes_2
10x_3288_t2_ACGATGACCGCTAA-1 Patient5 Monocytes nonactive 2 0.0737905 Monocytes_2
10x_3288_t2_ACGATTCTCCTTGC-1 Patient5 Monocytes nonactive 2 0.06508782 Monocytes_2
10x_3288_t2_ACGATTCTCTTCCG-1 Patient5 T cells nonactive 9 0.05706677 T cells_9
10x_3288_t2_ACGCAATGGAAACA-1 Patient5 Monocytes nonactive 2 0.02214508 Monocytes_2
10x_3288_t2_ACGCAATGGAATGA-1 Patient5 Monocytes nonactive 2 0.02801027 Monocytes_2
10x_3288_t2_ACGCACCTAATCGC-1 Patient5 Monocytes nonactive 2 0.02539471 Monocytes_2
10x_3288_t2_ACGCACCTAGTCAC-1 Patient5 Monocytes nonactive 2 0.09103735 Monocytes_2
10x_3288_t2_ACGCACCTTATGCG-1 Patient5 Monocytes nonactive 2 0.06185404 Monocytes_2
10x_3288_t2_ACGCCACTACACGT-1 Patient5 Monocytes nonactive 2 0.02929427 Monocytes_2

10x_3288_t2_ACGCCACTATGGTC-1 Patient5 Monocytes nonactive 2 0.04857016 Monocytes_2
10x_3288_t2_ACGCCACTGGAACG-1 Patient5 Monocytes nonactive 2 0.01198402 Monocytes_2
10x_3288_t2_ACGCCACTGTAAGA-1 Patient5 Monocytes nonactive 2 0.04926764 Monocytes_2
10x_3288_t2_ACGCCACTTAGTCG-1 Patient5 Monocytes nonactive 2 0.01257054 Monocytes_2
10x_3288_t2_ACGCCACTTCCAGA-1 Patient5 Monocytes nonactive 2 0.02255723 Monocytes_2
10x_3288_t2_ACGCCACTTGGAAA-1 Patient5 Monocytes nonactive 2 0.00408979 Monocytes_2
10x_3288_t2_ACGCCGGACCTCCA-1 Patient5 Monocytes nonactive 3 0.06500856 Monocytes_3
10x_3288_t2_ACGCCGGAGTACGT-1 Patient5 Monocytes nonactive 2 0.02577516 Monocytes_2
10x_3288_t2_ACGCCGGAGTTACG-1 Patient5 Monocytes nonactive 2 0.0236193 Monocytes_2
10x_3288_t2_ACGCCGGATCTACT-1 Patient5 Monocytes nonactive 3 0.0227633 Monocytes_3
10x_3288_t2_ACGCCGGATTGTGG-1 Patient5 Monocytes nonactive 2 0.07412339 Monocytes_2
10x_3288_t2_ACGCCTTGAGCATC-1 Patient5 Monocytes nonactive 2 0.0353814 Monocytes_2
10x_3288_t2_ACGCCTTGCTGATG-1 Patient5 Monocytes nonactive 2 0.09707691 Monocytes_2
10x_3288_t2_ACGCGGTGATGGTC-1 Patient5 Monocytes nonactive 2 0.0413417 Monocytes_2
10x_3288_t2_ACGCTCACGCAAGG-1 Patient5 Monocytes nonactive 3 0.05532306 Monocytes_3
10x_3288_t2_ACGCTCACTTTGCT-1 Patient5 Monocytes nonactive 2 0.03259147 Monocytes_2
10x_3288_t2_ACGCTGCTAGATGA-1 Patient5 Monocytes nonactive 2 0.05744721

Monocytes_2
10x_3288_t2_ACGCTGCTCTAAGC-1 Patient5 Monocytes nonactive 2 0.02902479
Monocytes_2
10x_3288_t2_ACGCTGCTGGGATG-1 Patient5 Monocytes nonactive 3 0.02628242
Monocytes_3
10x_3288_t2_ACGCTGCTGTTGCA-1 Patient5 Monocytes nonactive 2 0.04102467
Monocytes_2
10x_3288_t2_ACGCTGCTTGTCCC-1 Patient5 Monocytes nonactive 2 0.02262063
Monocytes_2
10x_3288_t2_ACGGAACTACCATG-1 Patient5 Monocytes nonactive 3 0.04500349
Monocytes_3
10x_3288_t2_ACGGAACTACCGAT-1 Patient5 Monocytes nonactive 2 0.08542578
Monocytes_2
10x_3288_t2_ACGGAACTACGCTA-1 Patient5 Monocytes nonactive 2 0.04318052
Monocytes_2
10x_3288_t2_ACGGAACTAGTACC-1 Patient5 Monocytes nonactive 2 0.03005516
Monocytes_2
10x_3288_t2_ACGGAGGAAGTAGA-1 Patient5 Monocytes nonactive 3 0.0417063
Monocytes_3
10x_3288_t2_ACGGAGGACCGTTC-1 Patient5 Monocytes nonactive 2 0.04562171
Monocytes_2
10x_3288_t2_ACGGAGGAGTACCA-1 Patient5 Monocytes nonactive 2 0.04371949
Monocytes_2
10x_3288_t2_ACGGATTGAACGTC-1 Patient5 Monocytes nonactive 2 0.03680807
Monocytes_2
10x_3288_t2_ACGGATTGTTGTCT-1Patient5 Monocytes nonactive 2 0.03661784
Monocytes_2
10x_3288_t2_ACGGCGTGATAAGG-1 Patient5 Monocytes nonactive 3 0.08487097
Monocytes_3
10x_3288_t2_ACGGCGTGCATGAC-1 Patient5 Monocytes nonactive 2 0.02802612
Monocytes_2
10x_3288_t2_ACGGCGTGGAGACG-1 Patient5 Monocytes nonactive 3 0.06263078
Monocytes_3

10x_3288_t2_ACGGCGTGGGATTC-1 Patient5 Monocytes nonactive 2 0.03886881
Monocytes_2
10x_3288_t2_ACGGCTCTAAGTGA-1 Patient5 Monocytes nonactive 2 0.03060998
Monocytes_2
10x_3288_t2_ACGGCTCTACCCAA-1 Patient5 Monocytes nonactive 2 0.04554245
Monocytes_2
10x_3288_t2_ACGGCTCTACGTTG-1 Patient5 Monocytes nonactive 2 0.02046478
Monocytes_2
10x_3288_t2_ACGGCTCTCCATGA-1 Patient5 Monocytes nonactive 3 0.04162704
Monocytes_3
10x_3288_t2_ACGGCTCTCTTCGC-1 Patient5 Monocytes nonactive 2 0.02482404
Monocytes_2
10x_3288_t2_ACGGCTCTGGACAG-1 Patient5 Monocytes nonactive 2 0.03872614
Monocytes_2
10x_3288_t2_ACGGCTCTGTTGTG-1 Patient5 Monocytes nonactive 2 0.01531292
Monocytes_2
10x_3288_t2_ACGGCTCTTACGAC-1 Patient5 Monocytes nonactive 2 0.05040898
Monocytes_2
10x_3288_t2_ACGGCTCTTCGCTC-1 Patient5 Monocytes nonactive 2 0.06763997
Monocytes_2
10x_3288_t2_ACGGCTCTTCTGGA-1 Patient5 Monocytes nonactive 2 0.07304546
Monocytes_2
10x_3288_t2_ACGGGAGATTCTAC-1 Patient5 Monocytes nonactive 2 0.03070509
Monocytes_2
10x_3288_t2_ACGGTAACCGCATA-1 Patient5 Monocytes nonactive 2 0.03777503
Monocytes_2
10x_3288_t2_ACGGTAACTCAAGC-1 Patient5 Monocytes nonactive 2 0.04211845
Monocytes_2
10x_3288_t2_ACGGTAACTCGCAA-1 Patient5 Monocytes nonactive 3 0.03351087
Monocytes_3
10x_3288_t2_ACGGTATGTCCAAG-1 Patient5 Monocytes nonactive 2 0.03091117
Monocytes_2
10x_3288_t2_ACGGTATGTGACAC-1 Patient5 Monocytes nonactive 2 0.04254645

Monocytes_2
10x_3288_t2_ACGGTCCTCAAAGA-1 Patient5 Monocytes nonactive 3 0.04395726 Monocytes_3
10x_3288_t2_ACGGTCCTTAACCG-1 Patient5 Monocytes nonactive 2 0.06851183 Monocytes_2
10x_3288_t2_ACGGTCCTTTGGCA-1 Patient5 T cells nonactive 9 0.02675797 T cells_9
10x_3288_t2_ACGTCAGAATGACC-1 Patient5 Monocytes nonactive 3 0.06179063 Monocytes_3
10x_3288_t2_ACGTCAGAGTCGAT-1 Patient5 Progenitors active 6 0.12129859 Progenitors_6
10x_3288_t2_ACGTCAGATAACCG-1 Patient5 Monocytes nonactive 2 0.05359521 Monocytes_2
10x_3288_t2_ACGTCAGATCCAGA-1 Patient5 Monocytes nonactive 2 0.03517532 Monocytes_2
10x_3288_t2_ACGTCCTGCTAGAC-1 Patient5 Monocytes nonactive 3 0.04241963 Monocytes_3
10x_3288_t2_ACGTCCTGGTTGGT-1 Patient5 Monocytes nonactive 3 0.07249065 Monocytes_3
10x_3288_t2_ACGTCGCTTCCTGC-1 Patient5 Monocytes nonactive 2 0.05479995 Monocytes_2
10x_3288_t2_ACGTCGCTTTGGTG-1 Patient5 Monocytes nonactive 2 0.06775094 Monocytes_2
10x_3288_t2_ACGTGATGAACCGT-1 Patient5 Monocytes nonactive 2 0.03683977 Monocytes_2
10x_3288_t2_ACGTGATGGGTTAC-1 Patient5 Monocytes nonactive 2 0.04739712 Monocytes_2
10x_3288_t2_ACGTGCCTCATACG-1 Patient5 Monocytes nonactive 3 0.04568512 Monocytes_3
10x_3288_t2_ACGTGCCTGCTACA-1 Patient5 Monocytes nonactive 2 0.02916746 Monocytes_2
10x_3288_t2_ACGTGCCTGGTGAG-1 Patient5 Monocytes nonactive 2 0.07580369 Monocytes_2

10x_3288_t2_ACGTTACTATTCTC-1 Patient5 Monocytes nonactive 2 0.03642762
Monocytes_2
10x_3288_t2_ACGTTACTTGCCCT-1 Patient5 Monocytes nonactive 2 0.03958214
Monocytes_2
10x_3288_t2_ACGTTGGATTTGGG-1 Patient5 Monocytes nonactive 2 0.03731533
Monocytes_2
10x_3288_t2_ACGTTTACACCTAG-1 Patient5 Monocytes nonactive 2 0.03496925
Monocytes_2
10x_3288_t2_ACGTTTACATGGTC-1 Patient5 Monocytes nonactive 2 0.04715934
Monocytes_2
10x_3288_t2_ACGTTTACCCAGTA-1 Patient5 Monocytes nonactive 2 0.05166128
Monocytes_2
10x_3288_t2_ACGTTTACCCTCAC-1 Patient5 Monocytes nonactive 2 0.02989665
Monocytes_2
10x_3288_t2_ACGTTTACTTCACT-1 Patient5 Monocytes nonactive 3 0.03084776
Monocytes_3
10x_3288_t2_ACTAAAACGAGAGC-1 Patient5 Monocytes nonactive 3 0.04942616
Monocytes_3
10x_3288_t2_ACTACGGAACTGGT-1 Patient5 Progenitors active 6 0.12950986
Progenitors_6
10x_3288_t2_ACTACGGAGCAGAG-1 Patient5 Monocytes nonactive 2 0.02534716
Monocytes_2
10x_3288_t2_ACTACTACCCCTAC-1 Patient5 Monocytes nonactive 3 0.03221102
Monocytes_3
10x_3288_t2_ACTACTACGGTACT-1 Patient5 Monocytes nonactive 2 0.04037474
Monocytes_2
10x_3288_t2_ACTAGGTGGGTATC-1 Patient5 Monocytes nonactive 2 0.05429269
Monocytes_2
10x_3288_t2_ACTAGGTGTTTACC-1 Patient5 Monocytes nonactive 2 0.02392049
Monocytes_2
10x_3288_t2_ACTATCACAGTTCG-1 Patient5 Monocytes nonactive 2 0.06091877
Monocytes_2
10x_3288_t2_ACTATCACCCTCCA-1 Patient5 Monocytes nonactive 3 0.05793862

Monocytes_3

10x_3288_t2_ACTATCACTACGCA-1 Patient5 Monocytes nonactive 2 0.05884218 Monocytes_2

10x_3288_t2_ACTATCACTTGCAG-1 Patient5 Monocytes nonactive 2 0.05432439 Monocytes_2

10x_3288_t2_ACTCAGGACTGATG-1 Patient5 Monocytes nonactive 2 0.03961385 Monocytes_2

10x_3288_t2_ACTCAGGATCGCTC-1 Patient5 Monocytes nonactive 2 0.06207596 Monocytes_2

10x_3288_t2_ACTCCCGAAATGCC-1 Patient5 Monocytes nonactive 2 0.05709847 Monocytes_2

10x_3288_t2_ACTCCCGAATGCTG-1 Patient5 T cells nonactive 9 0.04295859 T cells_9

10x_3288_t2_ACTCCCGACTTACT-1 Patient5 Monocytes nonactive 2 0.04814216 Monocytes_2

10x_3288_t2_ACTCCCGAGACGGA-1 Patient5 Monocytes nonactive 2 0.03683977 Monocytes_2

10x_3288_t2_ACTCCCGAGGCATT-1 Patient5 Monocytes nonactive 2 0.04880794 Monocytes_2

10x_3288_t2_ACTCCCGATAGCGT-1 Patient5 Monocytes nonactive 2 0.05207343 Monocytes_2

10x_3288_t2_ACTCCCGATTGAGC-1 Patient5 Monocytes nonactive 3 0.04026378 Monocytes_3

10x_3288_t2_ACTCCTCTAAGCAA-1 Patient5 Monocytes nonactive 2 0.03867859 Monocytes_2

10x_3288_t2_ACTCCTCTCTCATT-1 Patient5 Monocytes nonactive 2 0.02547397 Monocytes_2

10x_3288_t2_ACTCCTCTCTGACA-1 Patient5 Monocytes nonactive 2 0.0417063 Monocytes_2

10x_3288_t2_ACTCCTCTGCCTTC-1 Patient5 Monocytes nonactive 2 0.0434183 Monocytes_2

10x_3288_t2_ACTCGAGAAGCATC-1 Patient5 Monocytes nonactive 3 0.02428508 Monocytes_3

10x_3288_t2_ACTCGAGAAGCCAT-1 Patient5 Monocytes nonactive 3 0.04571682 Monocytes_3
10x_3288_t2_ACTCGAGAATAAGG-1 Patient5 Monocytes nonactive 3 0.04260985 Monocytes_3
10x_3288_t2_ACTCGCACAAGAAC-1 Patient5 Monocytes nonactive 3 0.03515947 Monocytes_3
10x_3288_t2_ACTCGCACAAGAGT-1 Patient5 Monocytes nonactive 2 0.06822649 Monocytes_2
10x_3288_t2_ACTCGCACATTCGG-1 Patient5 Monocytes nonactive 2 0.02750301 Monocytes_2
10x_3288_t2_ACTCTATGCCATGA-1 Patient5 Monocytes nonactive 3 0.04318052 Monocytes_3
10x_3288_t2_ACTCTATGCTTGCC-1Patient5 Monocytes nonactive 3 0.03893222 Monocytes_3
10x_3288_t2_ACTCTATGGCTACA-1 Patient5 Monocytes nonactive 2 0.05868366 Monocytes_2
10x_3288_t2_ACTCTATGGGTAAA-1 Patient5 Monocytes nonactive 3 0.0725382 Monocytes_3
10x_3288_t2_ACTCTATGTAAGGA-1 Patient5 Monocytes nonactive 2 0.01723099 Monocytes_2
10x_3288_t2_ACTCTCCTCTGTCC-1 Patient5 Monocytes nonactive 3 0.05407076 Monocytes_3
10x_3288_t2_ACTCTCCTGGACGA-1 Patient5 Monocytes nonactive 2 0.03070509 Monocytes_2
10x_3288_t2_ACTCTCCTGGTAAA-1 Patient5 Monocytes nonactive 2 0.08290533 Monocytes_2
10x_3288_t2_ACTGAGACCGTGAT-1 Patient5 Monocytes nonactive 3 0.06843257 Monocytes_3
10x_3288_t2_ACTGAGACCTCCAC-1 Patient5 Monocytes nonactive 2 0.03083191 Monocytes_2
10x_3288_t2_ACTGAGACGTTGCA-1 Patient5 Monocytes nonactive 2 0.03848836 Monocytes_2
10x_3288_t2_ACTGAGACTTTGCT-1 Patient5 Monocytes nonactive 2 0.05527551

Monocytes_2
10x_3288_t2_ACTGCCTGAAGAAC-1 Patient5 Monocytes nonactive 2 0.04479741
Monocytes_2
10x_3288_t2_ACTGCCTGACCAGT-1 Patient5 Progenitors nonactive 6 0.10807812
Progenitors_6
10x_3288_t2_ACTGCCTGAGGAGC-1 Patient5 Monocytes nonactive 2 0.0400894
Monocytes_2
10x_3288_t2_ACTGCCTGTGGTGT-1 Patient5 Monocytes nonactive 2 0.00175956
Monocytes_2
10x_3288_t2_ACTGGCCTATTCTC-1 Patient5 Monocytes nonactive 3 0.06678397
Monocytes_3
10x_3288_t2_ACTGGCCTCAGAAA-1 Patient5 Monocytes nonactive 2 0.03241709
Monocytes_2
10x_3288_t2_ACTGTGGAAAGTAG-1 Patient5 Monocytes nonactive 2 0.06686323
Monocytes_2
10x_3288_t2_ACTGTGGATAACCG-1 Patient5 Monocytes nonactive 2 0.10186418
Monocytes_2
10x_3288_t2_ACTGTGGATGAGAA-1 Patient5 Monocytes nonactive 2 0.02390464
Monocytes_2
10x_3288_t2_ACTGTTACGACAGG-1 Patient5 Monocytes nonactive 3 0.07878384
Monocytes_3
10x_3288_t2_ACTGTTACTGTCTT-1 Patient5 Monocytes nonactive 2 0.02132078
Monocytes_2
10x_3288_t2_ACTTAAGACTTCCG-1 Patient5 Monocytes nonactive 2 0.02932598
Monocytes_2
10x_3288_t2_ACTTAAGATGATGC-1 Patient5 Monocytes nonactive 2 0.01369602
Monocytes_2
10x_3288_t2_ACTTAGCTCCGTAA-1 Patient5 Monocytes nonactive 2 0.02632997
Monocytes_2
10x_3288_t2_ACTTAGCTGTTGCA-1 Patient5 Monocytes nonactive 2 0.0375214
Monocytes_2
10x_3288_t2_ACTTCAACCACCAA-1 Patient5 Monocytes nonactive 3 0.03325724
Monocytes_3

10x_3288_t2_ACTTCAACCTAGAC-1 Patient5 Monocytes nonactive 2 0.04175385 Monocytes_2
10x_3288_t2_ACTTCAACGGGCAA-1 Patient5 Monocytes nonactive 3 0.03563503 Monocytes_3
10x_3288_t2_ACTTCCCTAACGTC-1 Patient5 Monocytes nonactive 2 0.02728109 Monocytes_2
10x_3288_t2_ACTTCTGACGTGAT-1 Patient5 Monocytes nonactive 3 0.02980153 Monocytes_3
10x_3288_t2_ACTTCTGAGAATCC-1 Patient5 Monocytes nonactive 3 0.04448038 Monocytes_3
10x_3288_t2_ACTTCTGATCCCGT-1 Patient5 Monocytes nonactive 3 0.04379874 Monocytes_3
10x_3288_t2_ACTTCTGATCCGAA-1 Patient5 Monocytes nonactive 2 0.02057574 Monocytes_2
10x_3288_t2_ACTTCTGATCGCTC-1 Patient5 Monocytes nonactive 2 0.03313043 Monocytes_2
10x_3288_t2_ACTTCTGATTAGGC-1 Patient5 Monocytes nonactive 2 0.04665208 Monocytes_2
10x_3288_t2_ACTTGACTACGGTT-1 Patient5 Monocytes nonactive 3 0.03156109 Monocytes_3
10x_3288_t2_ACTTGACTCACCAA-1 Patient5 Monocytes nonactive 2 0.02731279 Monocytes_2
10x_3288_t2_ACTTGACTGCCTTC-1 Patient5 Monocytes nonactive 3 0.03141843 Monocytes_3
10x_3288_t2_ACTTGGGAAACAGA-1 Patient5 Monocytes nonactive 2 0.04318052 Monocytes_2
10x_3288_t2_ACTTGTACACGGAG-1 Patient5 Monocytes nonactive 2 0.00280578 Monocytes_2
10x_3288_t2_ACTTGTACAGTGCT-1 Patient5 Monocytes nonactive 2 0.06313804 Monocytes_2
10x_3288_t2_ACTTTGTGAGAACA-1 Patient5 Monocytes nonactive 3 0.05933359 Monocytes_3
10x_3288_t2_ACTTTGTGGACACT-1 Patient5 Monocytes nonactive 2 0.061537

Monocytes_2
10x_3288_t2_AGAAACGACTTATC-1 Patient5 Monocytes nonactive 2 0.0429903
Monocytes_2
10x_3288_t2_AGAAACGAGTACAC-1 Patient5 Monocytes nonactive 3 0.06668886
Monocytes_3
10x_3288_t2_AGAAAGTGACTGTG-1 Patient5 Monocytes nonactive 3 0.04665208
Monocytes_3
10x_3288_t2_AGAACAGACTGATG-1 Patient5 Monocytes nonactive 2 0.05362691
Monocytes_2
10x_3288_t2_AGAACGCTAATCGC-1 Patient5 Monocytes nonactive 3 0.06725953
Monocytes_3
10x_3288_t2_AGAACGCTAGTCTG-1 Patient5 Monocytes nonactive 2 0.09496861
Monocytes_2
10x_3288_t2_AGAACGCTCCCGTT-1 Patient5 Monocytes nonactive 2 0.05326232
Monocytes_2
10x_3288_t2_AGAACGCTCCTATT-1 Patient5 Monocytes nonactive 2 0.02376197
Monocytes_2
10x_3288_t2_AGAATACTAAGAAC-1 Patient5 Monocytes nonactive 2 0.04570097
Monocytes_2
10x_3288_t2_AGAATGGAAGATGA-1 Patient5 Monocytes nonactive 2 0.02090863
Monocytes_2
10x_3288_t2_AGAATGGAGTTCAG-1 Patient5 Monocytes nonactive 2 0.021511
Monocytes_2
10x_3288_t2_AGAATTTGACGACT-1 Patient5 Monocytes nonactive 3 0.04773001
Monocytes_3
10x_3288_t2_AGAATTTGAGCGTT-1 Patient5 Monocytes nonactive 2 0.01704077
Monocytes_2
10x_3288_t2_AGAATTTGATCGAC-1 Patient5 Monocytes nonactive 2 0.05632173
Monocytes_2
10x_3288_t2_AGAATTTGCCCTAC-1 Patient5 Monocytes nonactive 2 0.03362184
Monocytes_2
10x_3288_t2_AGAATTTGGAGCAG-1 Patient5 Monocytes nonactive 3 0.05069431
Monocytes_3

10x_3288_t2_AGAATTTGGTGTTG-1	Patient5	Monocytes	nonactive	2	0.08282607	Monocytes_2
10x_3288_t2_AGAATTTGTCCGTC-1	Patient5	Monocytes	nonactive	2	0.02805783	Monocytes_2
10x_3288_t2_AGAATTTGTTCCCG-1	Patient5	Monocytes	nonactive	3	0.03631666	Monocytes_3
10x_3288_t2_AGACACACAAGGGC-1	Patient5	Monocytes	nonactive	2	0.03395473	Monocytes_2
10x_3288_t2_AGACACACACCTCC-1	Patient5	Monocytes	nonactive	3	0.04297445	Monocytes_3
10x_3288_t2_AGACACACCTTCGC-1	Patient5	Monocytes	nonactive	2	0.0349534	Monocytes_2
10x_3288_t2_AGACACACGCAAGG-1	Patient5	Monocytes	nonactive	2	0.02628242	Monocytes_2
10x_3288_t2_AGACACACGGTAAA-1	Patient5	Monocytes	nonactive	2	0.04755564	Monocytes_2
10x_3288_t2_AGACACACGTTTGG-1	Patient5	Monocytes	nonactive	3	0.08439541	Monocytes_3
10x_3288_t2_AGACACACTTGTCT-1	Patient5	Monocytes	nonactive	3	0.06542071	Monocytes_3
10x_3288_t2_AGACACTGAAGTGA-1	Patient5	Monocytes	nonactive	3	0.06622915	Monocytes_3
10x_3288_t2_AGACACTGACTCAG-1	Patient5	Monocytes	nonactive	3	0.07244309	Monocytes_3
10x_3288_t2_AGACACTGGTAAAG-1	Patient5	Monocytes	nonactive	2	0.05175639	Monocytes_2
10x_3288_t2_AGACCTGACCGTAA-1	Patient5	Monocytes	nonactive	3	0.0518515	Monocytes_3
10x_3288_t2_AGACCTGACGATAC-1	Patient5	Monocytes	nonactive	2	0.03322554	Monocytes_2
10x_3288_t2_AGACCTGAGTTACG-1	Patient5	Monocytes	nonactive	2	0.02295352	Monocytes_2
10x_3288_t2_AGACGTACCTACTT-1	Patient5	Monocytes	nonactive	2	0.00294845	

Monocytes_2

10x_3288_t2_AGACGTACGAAACA-1 Patient5 Monocytes nonactive 3 0.03817133 Monocytes_3

10x_3288_t2_AGACGTACTCCTCG-1 Patient5 Progenitors nonactive 6 0.09339928 Progenitors_6

10x_3288_t2_AGACTCGATCTACT-1 Patient5 Monocytes nonactive 3 0.06134678 Monocytes_3

10x_3288_t2_AGACTCGATCTCAT-1Patient5 Monocytes nonactive 3 0.01030372 Monocytes_3

10x_3288_t2_AGACTCGATGATGC-1 Patient5 Monocytes nonactive 3 0.03108554 Monocytes_3

10x_3288_t2_AGACTTCTGACTAC-1 Patient5 B cells nonactive 7 0.05121742 B cells_7

10x_3288_t2_AGACTTCTTCAGGT-1 Patient5 Monocytes nonactive 2 0.06041151 Monocytes_2

10x_3288_t2_AGAGAAACCTTTAC-1 Patient5 Monocytes nonactive 2 0.00887705 Monocytes_2

10x_3288_t2_AGAGAAACTCTATC-1 Patient5 Monocytes nonactive 3 0.0231913 Monocytes_3

10x_3288_t2_AGAGAATGTAAGCC-1 Patient5 Monocytes nonactive 3 0.02430093 Monocytes_3

10x_3288_t2_AGAGAATGTGACAC-1 Patient5 Monocytes nonactive 2 0.09471498 Monocytes_2

10x_3288_t2_AGAGATGACTCCAC-1 Patient5 Monocytes nonactive 2 0.02667871 Monocytes_2

10x_3288_t2_AGAGATGAGGTGTT-1 Patient5 Monocytes nonactive 2 0.02837487 Monocytes_2

10x_3288_t2_AGAGATGATGGTTG-1 Patient5 Monocytes nonactive 2 0.02821635 Monocytes_2

10x_3288_t2_AGAGCGGAAGATCC-1 Patient5 Monocytes nonactive 2 0.02268404 Monocytes_2

10x_3288_t2_AGAGCGGACCGTTC-1 Patient5 Monocytes nonactive 2 0.03181472 Monocytes_2

10x_3288_t2_AGAGCGGATATGCG-1 Patient5 Monocytes nonactive 2 0.0269482 Monocytes_2
10x_3288_t2_AGAGCTACAGGAGC-1 Patient5 Monocytes nonactive 2 0.02008433 Monocytes_2
10x_3288_t2_AGAGCTACGCGATT-1 Patient5 Monocytes nonactive 2 0.03657029 Monocytes_2
10x_3288_t2_AGAGCTACGTTCTT-1 Patient5 Monocytes nonactive 3 0.07361613 Monocytes_3
10x_3288_t2_AGAGCTACTGCATG-1 Patient5 Monocytes nonactive 3 0.03132331 Monocytes_3
10x_3288_t2_AGAGGTCTGCCATA-1 Patient5 Progenitors nonactive 6 0.10252996 Progenitors_6
10x_3288_t2_AGAGGTCTTCGCCT-1 Patient5 Monocytes nonactive 2 0.01461543 Monocytes_2
10x_3288_t2_AGAGGTCTTTCAGG-1 Patient5 Monocytes nonactive 2 0.05419758 Monocytes_2
10x_3288_t2_AGAGTCACACTAGC-1 Patient5 T cells nonactive 9 0.03638006 T cells_9
10x_3288_t2_AGAGTCACCTGTCC-1 Patient5 Monocytes active 2 0.11792214 Monocytes_2
10x_3288_t2_AGAGTCACCTTGCC-1 Patient5 Progenitors active 6 0.1471213 Progenitors_6
10x_3288_t2_AGAGTCTGACGCAT-1 Patient5 Monocytes nonactive 2 0.04067592 Monocytes_2
10x_3288_t2_AGAGTGCTCTCGCT-1 Patient5 Dendritic cells nonactive 10 0.05397565 Dendritic cells_10
10x_3288_t2_AGAGTGCTTCGTGA-1 Patient5 Monocytes nonactive 3 0.06402574 Monocytes_3
10x_3288_t2_AGATATACCCTATT-1 Patient5 Monocytes nonactive 3 0.04211845 Monocytes_3
10x_3288_t2_AGATATACCTCTTA-1 Patient5 Monocytes nonactive 3 0.04240378 Monocytes_3
10x_3288_t2_AGATATTGAACGTC-1 Patient5 Monocytes nonactive 2 0.05134424

Monocytes_2
10x_3288_t2_AGATATTGGCGAGA-1 Patient5 Monocytes nonactive 3 0.0434183 Monocytes_3
10x_3288_t2_AGATATTGGTACCA-1 Patient5 Monocytes nonactive 3 0.05754232 Monocytes_3
10x_3288_t2_AGATCGTGCCTGTC-1 Patient5 Monocytes nonactive 2 0.03347917 Monocytes_2
10x_3288_t2_AGATCGTGGTATGC-1 Patient5 Monocytes nonactive 3 0.08461734 Monocytes_3
10x_3288_t2_AGATCTCTCACTGA-1 Patient5 Monocytes nonactive 2 0.02528375 Monocytes_2
10x_3288_t2_AGATCTCTGTAAAG-1 Patient5 Monocytes nonactive 3 0.04701668 Monocytes_3
10x_3288_t2_AGATCTCTGTCCTC-1 Patient5 Monocytes nonactive 2 0.02745546 Monocytes_2
10x_3288_t2_AGATCTCTTGAACC-1 Patient5 Monocytes nonactive 3 0.02377782 Monocytes_3
10x_3288_t2_AGATTAACGGTGTT-1 Patient5 Monocytes nonactive 2 0.08442711 Monocytes_2
10x_3288_t2_AGATTCCTAAGGCG-1 Patient5 Monocytes nonactive 2 0.06356604 Monocytes_2
10x_3288_t2_AGATTCCTCAATCG-1 Patient5 Monocytes nonactive 2 0.01930759 Monocytes_2
10x_3288_t2_AGCAAAGAAATCGC-1 Patient5 Monocytes nonactive 2 0.01184135 Monocytes_2
10x_3288_t2_AGCAAAGAAGCCAT-1 Patient5 Monocytes nonactive 2 0.04566927 Monocytes_2
10x_3288_t2_AGCAACACAGCGGA-1 Patient5 Monocytes nonactive 2 0.05556084 Monocytes_2
10x_3288_t2_AGCAACACCCAATG-1 Patient5 Monocytes nonactive 2 0.03484243 Monocytes_2
10x_3288_t2_AGCAACACCGCTAA-1 Patient5 Monocytes nonactive 2 0.02913576 Monocytes_2

10x_3288_t2_AGCAACACTGAGAA-1 Patient5 Monocytes nonactive 2 0.04286348 Monocytes_2
10x_3288_t2_AGCAAGCTACCTGA-1 Patient5 T cells nonactive 9 0.03641177 T cells_9
10x_3288_t2_AGCAAGCTCGCATA-1 Patient5 Monocytes nonactive 2 0.04817386 Monocytes_2
10x_3288_t2_AGCAAGCTCGTGAT-1 Patient5 Monocytes nonactive 2 0.0762951 Monocytes_2
10x_3288_t2_AGCAAGCTTCGTAG-1 Patient5 T cells nonactive 9 0.0438463 T cells_9
10x_3288_t2_AGCACAACCTCTTA-1 Patient5 Monocytes nonactive 2 0.03357428 Monocytes_2
10x_3288_t2_AGCACTGATTGTCT-1 Patient5 Monocytes nonactive 2 0.03839325 Monocytes_2
10x_3288_t2_AGCATCGAAATCGC-1 Patient5 Monocytes nonactive 3 0.04739712 Monocytes_3
10x_3288_t2_AGCATCGACCATGA-1 Patient5 Monocytes nonactive 2 0.001712 Monocytes_2
10x_3288_t2_AGCATGACTAGCCA-1 Patient5 T cells nonactive 9 0.11659058 T cells_9
10x_3288_t2_AGCATGACTTCTTG-1 Patient5 Monocytes nonactive 2 0.04511445 Monocytes_2
10x_3288_t2_AGCATTCTATGCTG-1 Patient5 Monocytes nonactive 2 0.0362691 Monocytes_2
10x_3288_t2_AGCATTCTGACACT-1 Patient5 Monocytes nonactive 3 0.04432186 Monocytes_3
10x_3288_t2_AGCATTCTTGCTTT-1 Patient5 Monocytes nonactive 2 0.04652527 Monocytes_2
10x_3288_t2_AGCCAATGCCTCGT-1 Patient5 Monocytes nonactive 2 0.03634836 Monocytes_2
10x_3288_t2_AGCCAATGCTCTTA-1 Patient5 Monocytes nonactive 3 0.0648976 Monocytes_3
10x_3288_t2_AGCCAATGTGTCTT-1 Patient5 Monocytes nonactive 2 0.02590197

Monocytes_2
10x_3288_t2_AGCCACCTGTTCGA-1 Patient5 Monocytes nonactive 2 0.01892714 Monocytes_2
10x_3288_t2_AGCCACCTGTTGCA-1 Patient5 Monocytes nonactive 2 0.05069431 Monocytes_2
10x_3288_t2_AGCCGGACCCATGA-1 Patient5 Monocytes nonactive 2 0.05326232 Monocytes_2
10x_3288_t2_AGCCGGACCCTCCA-1 Patient5 Monocytes nonactive 2 0.0590324 Monocytes_2
10x_3288_t2_AGCCGGACTAACGC-1 Patient5 Monocytes nonactive 2 0.03316213 Monocytes_2
10x_3288_t2_AGCCGGACTACGAC-1 Patient5 Monocytes nonactive 2 0.05532306 Monocytes_2
10x_3288_t2_AGCCGGTGAAAACG-1 Patient5 Monocytes nonactive 3 0.04221356 Monocytes_3
10x_3288_t2_AGCCGGTGACTAGC-1 Patient5 Monocytes nonactive 3 0.05538647 Monocytes_3
10x_3288_t2_AGCCGTCTACGTAC-1 Patient5 Monocytes nonactive 3 0.02449115 Monocytes_3
10x_3288_t2_AGCCGTCTCGCATA-1 Patient5 Monocytes nonactive 2 0.0286285 Monocytes_2
10x_3288_t2_AGCCGTCTTTGCTT-1Patient5 Monocytes nonactive 2 0.00686386 Monocytes_2
10x_3288_t2_AGCCTCACATAAGG-1 Patient5 Monocytes nonactive 3 0.04164289 Monocytes_3
10x_3288_t2_AGCCTCACGGAAAT-1 Patient5 T cells nonactive 9 0.07017627 T cells_9
10x_3288_t2_AGCCTCTGCATTCT-1Patient5 Monocytes nonactive 2 0.0624247 Monocytes_2
10x_3288_t2_AGCCTCTGTCCAGA-1 Patient5 Monocytes nonactive 2 0.04205504 Monocytes_2
10x_3288_t2_AGCGAACTGACAAA-1 Patient5 Monocytes nonactive 2 0.02682138 Monocytes_2

10x_3288_t2_AGCGAACTGTGCAT-1 Patient5 Monocytes nonactive 2 0.08287363 Monocytes_2
10x_3288_t2_AGCGAACTTATCGG-1 Patient5 Monocytes nonactive 2 0.0350168 Monocytes_2
10x_3288_t2_AGCGATACCCTTCG-1 Patient5 T cells nonactive 9 0.04774586 T cells_9
10x_3288_t2_AGCGATACCGTGTA-1 Patient5 Monocytes nonactive 2 0.05006024 Monocytes_2
10x_3288_t2_AGCGATACGTTACG-1 Patient5 Monocytes nonactive 3 0.04603386 Monocytes_3
10x_3288_t2_AGCGATACTCACGA-1 Patient5 Monocytes nonactive 2 0.05784351 Monocytes_2
10x_3288_t2_AGCGATTGGAGGCA-1 Patient5 Monocytes nonactive 2 0.04292689 Monocytes_2
10x_3288_t2_AGCGATTGGGTACT-1 Patient5 B cells nonactive 7 0.07022383 B cells_7
10x_3288_t2_AGCGCCGAATCTTC-1 Patient5 Monocytes nonactive 3 0.05075772 Monocytes_3
10x_3288_t2_AGCGCCGAGAGATA-1 Patient5 Monocytes nonactive 2 0.05376958 Monocytes_2
10x_3288_t2_AGCGCCGAGGTTTG-1 Patient5 Monocytes nonactive 3 0.03901148 Monocytes_3
10x_3288_t2_AGCGCCGATGGTTG-1 Patient5 Monocytes nonactive 2 0.0472386 Monocytes_2
10x_3288_t2_AGCGCTCTAATCGC-1 Patient5 Monocytes nonactive 2 0.02208167 Monocytes_2
10x_3288_t2_AGCGCTCTCTAGTG-1 Patient5 Monocytes nonactive 3 0.05116987 Monocytes_3
10x_3288_t2_AGCGCTCTGAGACG-1 Patient5 Monocytes nonactive 2 0.03866274 Monocytes_2
10x_3288_t2_AGCGCTCTGCCAAT-1 Patient5 Monocytes nonactive 2 0.0522795 Monocytes_2
10x_3288_t2_AGCGCTCTTTCGTT-1Patient5 Monocytes nonactive 2 0.03452539

Monocytes_2
10x_3288_t2_AGCGGCACAGGTCT-1 Patient5 Monocytes nonactive 2 0.06358189 Monocytes_2
10x_3288_t2_AGCGGCACCCTACC-1 Patient5 Monocytes nonactive 3 0.04679475 Monocytes_3
10x_3288_t2_AGCGGCACCTGTCC-1 Patient5 Monocytes nonactive 2 0.00271067 Monocytes_2
10x_3288_t2_AGCGGCACGGTAAA-1 Patient5 Monocytes nonactive 2 0.07520132 Monocytes_2
10x_3288_t2_AGCGGCACGTCAAC-1 Patient5 Monocytes nonactive 2 0.01043054 Monocytes_2
10x_3288_t2_AGCGGCTGATCTTC-1 Patient5 Monocytes nonactive 2 0.03274998 Monocytes_2
10x_3288_t2_AGCGGCTGTGGGAG-1 Patient5 Monocytes nonactive 3 0.0354131 Monocytes_3
10x_3288_t2_AGCGGGCTCCGATA-1 Patient5 Monocytes nonactive 2 0.02736034 Monocytes_2
10x_3288_t2_AGCGGGCTCTAGCA-1 Patient5 Monocytes nonactive 3 0.03804451 Monocytes_3
10x_3288_t2_AGCGGGCTCTGTGA-1 Patient5 Monocytes nonactive 2 0.04077104 Monocytes_2
10x_3288_t2_AGCGGGCTTACGAC-1 Patient5 Monocytes nonactive 2 0.02263648 Monocytes_2
10x_3288_t2_AGCGTAACCACACA-1 Patient5 Monocytes nonactive 2 0.04172215 Monocytes_2
10x_3288_t2_AGCGTAACGCAGAG-1 Patient5 Monocytes nonactive 3 0.03863103 Monocytes_3
10x_3288_t2_AGCGTAACTCCAAG-1 Patient5 Monocytes nonactive 2 0.0577167 Monocytes_2
10x_3288_t2_AGCGTAACTCCGTC-1 Patient5 Monocytes nonactive 2 0.03431932 Monocytes_2
10x_3288_t2_AGCGTAACTTCCGC-1 Patient5 Monocytes nonactive 2 0.02961131 Monocytes_2

10x_3288_t2_AGCTCGCTCCTCCA-1 Patient5 Monocytes nonactive 3 0.06640353 Monocytes_3
10x_3288_t2_AGCTCGCTTAGTCG-1 Patient5 Monocytes nonactive 2 0.03217932 Monocytes_2
10x_3288_t2_AGCTGAACCTAAGC-1 Patient5 Monocytes nonactive 3 0.04286348 Monocytes_3
10x_3288_t2_AGCTGAACTCAGAC-1 Patient5 Monocytes nonactive 2 0.07266502 Monocytes_2
10x_3288_t2_AGCTGCCTAGGGTG-1 Patient5 Monocytes nonactive 2 0.06291611 Monocytes_2
10x_3288_t2_AGCTGCCTCCTTGC-1 Patient5 Monocytes nonactive 2 0.06901909 Monocytes_2
10x_3288_t2_AGCTGCCTGAATCC-1 Patient5 Dendritic cells nonactive 10 0.06556338 Dendritic cells_10
10x_3288_t2_AGCTGCCTGCATAC-1 Patient5 Monocytes nonactive 3 0.03590451 Monocytes_3
10x_3288_t2_AGCTGTGAGGAGTG-1 Patient5 Monocytes nonactive 2 0.05437195 Monocytes_2
10x_3288_t2_AGCTGTGATATGCG-1 Patient5 Monocytes nonactive 2 0.07499524 Monocytes_2
10x_3288_t2_AGCTGTGATGAGCT-1 Patient5 Monocytes nonactive 2 0.03038805 Monocytes_2
10x_3288_t2_AGCTTACTAGTTCG-1 Patient5 Monocytes nonactive 2 0.02789931 Monocytes_2
10x_3288_t2_AGCTTACTCGAACT-1 Patient5 Monocytes nonactive 3 0.05779595 Monocytes_3
10x_3288_t2_AGCTTACTCTGGTA-1 Patient5 Monocytes nonactive 3 0.03105383 Monocytes_3
10x_3288_t2_AGCTTACTTGCTAG-1 Patient5 Monocytes nonactive 2 0.02075011 Monocytes_2
10x_3288_t2_AGCTTACTTGTAGC-1 Patient5 Monocytes nonactive 2 0.03309873 Monocytes_2
10x_3288_t2_AGCTTTACCAAGCT-1 Patient5 Monocytes nonactive 3 0.04273667

Monocytes_3
10x_3288_t2_AGCTTTACTATTCC-1 Patient5 Monocytes nonactive 2 0.0308002 Monocytes_2
10x_3288_t2_AGGAAATGGGGTGA-1 Patient5 Monocytes nonactive 2 0.04078689 Monocytes_2
10x_3288_t2_AGGAAATGGTTACG-1 Patient5 Monocytes nonactive 2 0.02767738 Monocytes_2
10x_3288_t2_AGGAAATGTCTACT-1 Patient5 Monocytes nonactive 2 0.05977744 Monocytes_2
10x_3288_t2_AGGAATGAGCTATG-1 Patient5 Monocytes nonactive 2 0.01103291 Monocytes_2
10x_3288_t2_AGGACACTCATTCT-1 Patient5 Monocytes nonactive 3 0.06033226 Monocytes_3
10x_3288_t2_AGGACTTGGCTAAC-1 Patient5 Monocytes nonactive 3 0.02506182 Monocytes_3
10x_3288_t2_AGGACTTGGGATTC-1 Patient5 Monocytes nonactive 3 0.06009448 Monocytes_3
10x_3288_t2_AGGACTTGTTCACT-1 Patient5 Monocytes nonactive 3 0.01466299 Monocytes_3
10x_3288_t2_AGGAGTCTCAAGCT-1 Patient5 Monocytes nonactive 2 0.05327817 Monocytes_2
10x_3288_t2_AGGAGTCTCTAGTG-1 Patient5 Monocytes nonactive 3 0.04650942 Monocytes_3
10x_3288_t2_AGGAGTCTGACAAA-1 Patient5 Monocytes nonactive 3 0.05559254 Monocytes_3
10x_3288_t2_AGGAGTCTTGCCAA-1 Patient5 Monocytes nonactive 3 0.06849597 Monocytes_3
10x_3288_t2_AGGATAGAAGCGTT-1 Patient5 Monocytes nonactive 2 0.01063661 Monocytes_2
10x_3288_t2_AGGATGCTTGTAGC-1 Patient5 T cells nonactive 9 0.07201509 T cells_9
10x_3288_t2_AGGCAACTCTGTCC-1 Patient5 Monocytes nonactive 3 0.04814216 Monocytes_3

10x_3288_t2_AGGCAACTTTGTCT-1	Patient5 Monocytes	nonactive	2	0.03477902	Monocytes_2
10x_3288_t2_AGGCAGGACGTTGA-1	Patient5 Monocytes	nonactive	2	0.03401813	Monocytes_2
10x_3288_t2_AGGCAGGAGCTTAG-1	Patient5 Monocytes	nonactive	2	0.06431108	Monocytes_2
10x_3288_t2_AGGCAGGATTCAGG-1	Patient5 Monocytes	nonactive	2	0.02495086	Monocytes_2
10x_3288_t2_AGGCAGGATTCCGC-1	Patient5 Monocytes	nonactive	3	0.04251474	Monocytes_3
10x_3288_t2_AGGCCTCTTAACGC-1	Patient5 Monocytes	nonactive	2	0.01862596	Monocytes_2
10x_3288_t2_AGGCCTCTTTCCGC-1	Patient5 Monocytes	nonactive	2	0.04964809	Monocytes_2
10x_3288_t2_AGGCTAACCCTTGC-1	Patient5 Monocytes	nonactive	2	0.02044893	Monocytes_2
10x_3288_t2_AGGCTAACGAGGAC-1	Patient5 Monocytes	nonactive	2	0.04539978	Monocytes_2
10x_3288_t2_AGGCTAACTGTTCT-1	Patient5 Monocytes	nonactive	2	0.03124406	Monocytes_2
10x_3288_t2_AGGGACGAAAGTGA-1	Patient5 Monocytes	nonactive	2	0.02731279	Monocytes_2
10x_3288_t2_AGGGACGAAGCATC-1	Patient5 Monocytes	nonactive	3	0.043878	Monocytes_3
10x_3288_t2_AGGGACGACCTGTC-1	Patient5 Monocytes	nonactive	2	0.03647518	Monocytes_2
10x_3288_t2_AGGGACGACTCCCA-1	Patient5 Monocytes	nonactive	2	0.0383457	Monocytes_2
10x_3288_t2_AGGGACGACTTGCC-1	Patient5 Monocytes	nonactive	2	0.05025046	Monocytes_2
10x_3288_t2_AGGGACGAGTTGCA-1	Patient5 Monocytes	nonactive	2	0.02125737	Monocytes_2
10x_3288_t2_AGGGACGATAGAGA-1	Patient5 Monocytes	nonactive	3	0.05663877	

Monocytes_3
10x_3288_t2_AGGGCCACCCAACA-1 Patient5 Monocytes nonactive 2 0.06345508 Monocytes_2
10x_3288_t2_AGGGCCACCCAATG-1 Patient5 Monocytes nonactive 3 0.01537632 Monocytes_3
10x_3288_t2_AGGGCCTGAGATCC-1 Patient5 Monocytes nonactive 2 0.01786507 Monocytes_2
10x_3288_t2_AGGGCCTGCTTACT-1 Patient5 Monocytes nonactive 2 0.0624247 Monocytes_2
10x_3288_t2_AGGGCCTGGGTAAA-1 Patient5 T cells nonactive 9 0.08523556 T cells_9
10x_3288_t2_AGGGCCTGTTCCCG-1 Patient5 Monocytes nonactive 3 0.02536301 Monocytes_3
10x_3288_t2_AGGGCGCTTGTCAG-1 Patient5 Monocytes nonactive 2 0.06036396 Monocytes_2
10x_3288_t2_AGGGTGGAGTGTCA-1 Patient5 Monocytes nonactive 2 0.01177795 Monocytes_2
10x_3288_t2_AGGGTGGATCTTCA-1 Patient5 Monocytes nonactive 3 0.06232959 Monocytes_3
10x_3288_t2_AGGGTGGATGCCCT-1 Patient5 Monocytes nonactive 2 0.08089214 Monocytes_2
10x_3288_t2_AGGGTGGATTCCCG-1 Patient5 Monocytes nonactive 3 0.03744214 Monocytes_3
10x_3288_t2_AGGGTTTGCGAACT-1 Patient5 Monocytes nonactive 2 0.03406569 Monocytes_2
10x_3288_t2_AGGGTTTGCTACCC-1 Patient5 Monocytes nonactive 3 0.05116987 Monocytes_3
10x_3288_t2_AGGGTTTGTACTGG-1 Patient5 Monocytes nonactive 2 0.03928096 Monocytes_2
10x_3288_t2_AGGTACACCTAGCA-1 Patient5 Monocytes nonactive 2 0.02774079 Monocytes_2
10x_3288_t2_AGGTACACCTCTTA-1 Patient5 Monocytes nonactive 3 0.04633505 Monocytes_3

10x_3288_t2_AGGTACACTCCAGA-1 Patient5 Monocytes nonactive 2 0.05500602 Monocytes_2
10x_3288_t2_AGGTACTGCCCAAA-1 Patient5 Monocytes nonactive 2 0.0454949 Monocytes_2
10x_3288_t2_AGGTCTGAAGTACC-1 Patient5 Monocytes nonactive 2 0.0590324 Monocytes_2
10x_3288_t2_AGGTCTGACCTTAT-1Patient5 Monocytes nonactive 2 0.04203919 Monocytes_2
10x_3288_t2_AGGTCTGATCAGTG-1 Patient5 Monocytes nonactive 2 0.02162196 Monocytes_2
10x_3288_t2_AGGTGGGACTCCCA-1 Patient5 Monocytes nonactive 2 0.03531799 Monocytes_2
10x_3288_t2_AGGTGGGATGTCGA-1 Patient5 Monocytes nonactive 2 0.04351341 Monocytes_2
10x_3288_t2_AGGTGGGATTATCC-1 Patient5 Monocytes nonactive 3 0.03939192 Monocytes_3
10x_3288_t2_AGGTGTTGCGATAC-1 Patient5 Monocytes nonactive 2 0.04944201 Monocytes_2
10x_3288_t2_AGGTGTTGCTCCAC-1 Patient5 Monocytes nonactive 2 0.02956376 Monocytes_2
10x_3288_t2_AGGTTCGAAGACTC-1 Patient5 Monocytes nonactive 2 0.06080781 Monocytes_2
10x_3288_t2_AGGTTCGAAGAGAT-1 Patient5 Monocytes nonactive 2 0.03604718 Monocytes_2
10x_3288_t2_AGGTTCGAAGCAAA-1 Patient5 Monocytes nonactive 2 0.03100628 Monocytes_2
10x_3288_t2_AGGTTCGATGGAAA-1 Patient5 Monocytes nonactive 2 0.04362437 Monocytes_2
10x_3288_t2_AGGTTGTGCCATGA-1 Patient5 Monocytes nonactive 2 0.02035381 Monocytes_2
10x_3288_t2_AGTAAGGAACACGT-1 Patient5 Monocytes nonactive 2 0.0476666 Monocytes_2
10x_3288_t2_AGTAAGGAGGAGGT-1 Patient5 Monocytes nonactive 2 0.040454

Monocytes_2
10x_3288_t2_AGTAAGGAGGTAAA-1 Patient5 Monocytes nonactive 2 0.04609727 Monocytes_2
10x_3288_t2_AGTAAGGAGTAAGA-1 Patient5 Monocytes nonactive 2 0.01788092 Monocytes_2
10x_3288_t2_AGTAAGGATGTGCA-1 Patient5 Monocytes nonactive 3 0.05901655 Monocytes_3
10x_3288_t2_AGTAATACCCAGTA-1 Patient5 Monocytes nonactive 2 0.02488745 Monocytes_2
10x_3288_t2_AGTAATTGGCGTTA-1 Patient5 Monocytes nonactive 2 0.02786761 Monocytes_2
10x_3288_t2_AGTAATTGTAACCG-1 Patient5 Monocytes nonactive 2 0.04734957 Monocytes_2
10x_3288_t2_AGTAATTGTCGTGA-1 Patient5 Monocytes nonactive 2 0.06494515 Monocytes_2
10x_3288_t2_AGTACGTGCGGGAA-1 Patient5 Monocytes nonactive 3 0.05497432 Monocytes_3
10x_3288_t2_AGTACGTGCGTAGT-1 Patient5 Monocytes nonactive 2 0.05240632 Monocytes_2
10x_3288_t2_AGTACGTGCTTAGG-1 Patient5 Monocytes nonactive 3 0.09098979 Monocytes_3
10x_3288_t2_AGTACGTGTCAGGT-1 Patient5 Monocytes nonactive 3 0.04276837 Monocytes_3
10x_3288_t2_AGTACTCTATCACG-1 Patient5 Monocytes nonactive 2 0.02187559 Monocytes_2
10x_3288_t2_AGTACTCTGCTGTA-1 Patient5 Monocytes nonactive 3 0.05335743 Monocytes_3
10x_3288_t2_AGTACTCTTGCGTA-1 Patient5 Monocytes nonactive 3 0.03320969 Monocytes_3
10x_3288_t2_AGTACTCTTGTCAG-1 Patient5 Monocytes nonactive 2 0.06101389 Monocytes_2
10x_3288_t2_AGTAGAGATAAGCC-1 Patient5 B cells nonactive 7 0.0450986 B cells_7

10x_3288_t2_AGTAGGCTCAGCTA-1 Patient5 Monocytes nonactive 2 0.02464967 Monocytes_2
10x_3288_t2_AGTATAACACCTGA-1 Patient5 Monocytes nonactive 2 0.06251981 Monocytes_2
10x_3288_t2_AGTATAACACGGTT-1 Patient5 Monocytes nonactive 3 0.03942363 Monocytes_3
10x_3288_t2_AGTATAACCGTGTA-1 Patient5 Monocytes nonactive 2 0.06719612 Monocytes_2
10x_3288_t2_AGTATAACGATACC-1 Patient5 Monocytes nonactive 2 0.04486082 Monocytes_2
10x_3288_t2_AGTATCCTAGCACT-1 Patient5 Monocytes nonactive 3 0.05947625 Monocytes_3
10x_3288_t2_AGTATCCTCGAGAG-1 Patient5 Monocytes nonactive 2 0.04245133 Monocytes_2
10x_3288_t2_AGTATCCTGAGGCA-1 Patient5 Monocytes nonactive 2 0.06908249 Monocytes_2
10x_3288_t2_AGTATCCTGGTGTT-1 Patient5 Monocytes nonactive 2 0.03945533 Monocytes_2
10x_3288_t2_AGTCACGACTTGTT-1 Patient5 Monocytes nonactive 3 0.06393063 Monocytes_3
10x_3288_t2_AGTCACGAGTTGAC-1 Patient5 Monocytes nonactive 2 0.09885232 Monocytes_2
10x_3288_t2_AGTCAGACACCATG-1 Patient5 Monocytes nonactive 2 0.06797286 Monocytes_2
10x_3288_t2_AGTCAGACGTGTTG-1 Patient5 Monocytes nonactive 2 0.044306 Monocytes_2
10x_3288_t2_AGTCCAGAATCAGC-1 Patient5 T cells nonactive 9 0.10587471 T cells_9
10x_3288_t2_AGTCCAGATAGCCA-1 Patient5 Monocytes nonactive 2 0.03436688 Monocytes_2
10x_3288_t2_AGTCCAGATTCACT-1 Patient5 Monocytes nonactive 2 0.01948196 Monocytes_2
10x_3288_t2_AGTCCAGATTCATC-1 Patient5 Monocytes nonactive 2 0.02553738

Monocytes_2
10x_3288_t2_AGTCGAACCCCTTG-1 Patient5 Monocytes nonactive 2 0.03563503 Monocytes_2
10x_3288_t2_AGTCGAACCCTCAC-1 Patient5 Monocytes nonactive 2 0.0501712 Monocytes_2
10x_3288_t2_AGTCGAACGCATCA-1 Patient5 Monocytes nonactive 2 0.02980153 Monocytes_2
10x_3288_t2_AGTCGAACGCTAAC-1 Patient5 Monocytes nonactive 2 0.03997844 Monocytes_2
10x_3288_t2_AGTCGCCTCACCAA-1 Patient5 Progenitors active 6 0.13673832 Progenitors_6
10x_3288_t2_AGTCGCCTCGAATC-1 Patient5 Monocytes nonactive 2 0.02474478 Monocytes_2
10x_3288_t2_AGTCGCCTCGTCTC-1 Patient5 Monocytes nonactive 2 0.06348678 Monocytes_2
10x_3288_t2_AGTCTACTACCATG-1 Patient5 Monocytes nonactive 3 0.0273762 Monocytes_3
10x_3288_t2_AGTCTACTGTCGAT-1Patient5 Monocytes nonactive 2 0.05963477 Monocytes_2
10x_3288_t2_AGTCTTACACTACG-1 Patient5 Monocytes nonactive 2 0.02880287 Monocytes_2
10x_3288_t2_AGTCTTACCTTACT-1 Patient5 Monocytes nonactive 2 0.02873946 Monocytes_2
10x_3288_t2_AGTCTTACGCGATT-1Patient5 Monocytes nonactive 3 0.04383045 Monocytes_3
10x_3288_t2_AGTCTTACTTGGTG-1 Patient5 Monocytes nonactive 3 0.06689493 Monocytes_3
10x_3288_t2_AGTGAAGACTTACT-1 Patient5 Monocytes nonactive 2 0.00041215 Monocytes_2
10x_3288_t2_AGTGACACAAGATG-1 Patient5 Monocytes nonactive 3 0.04511445 Monocytes_3
10x_3288_t2_AGTGACACCTGCTC-1 Patient5 Monocytes nonactive 2 0.05873122 Monocytes_2

10x_3288_t2_AGTGACTGGAATGA-1 Patient5 Monocytes nonactive 2 0.04857016 Monocytes_2
10x_3288_t2_AGTGCAACTTAGGC-1 Patient5 Monocytes nonactive 2 0.03332065 Monocytes_2
10x_3288_t2_AGTGTGACACACAC-1 Patient5 T cells nonactive 9 0.08560015 T cells_9
10x_3288_t2_AGTGTGACCATTCT-1 Patient5 Monocytes nonactive 2 0.0484592 Monocytes_2
10x_3288_t2_AGTGTGACTAGAGA-1 Patient5 Monocytes nonactive 2 0.0159787 Monocytes_2
10x_3288_t2_AGTGTGACTCGTAG-1 Patient5 Monocytes nonactive 2 0.02428508 Monocytes_2
10x_3288_t2_AGTTAAACGCGTTA-1 Patient5 Monocytes nonactive 2 0.0459546 Monocytes_2
10x_3288_t2_AGTTAAACGTGAGG-1 Patient5 Monocytes nonactive 2 0.05147105 Monocytes_2
10x_3288_t2_AGTTAAACTGAGAA-1 Patient5 Monocytes nonactive 3 0.00825883 Monocytes_3
10x_3288_t2_AGTTATGAAAACGA-1 Patient5 Monocytes nonactive 2 0.03674466 Monocytes_2
10x_3288_t2_AGTTATGAAAGAAC-1 Patient5 Monocytes nonactive 2 0.041738 Monocytes_2
10x_3288_t2_AGTTATGACATACG-1 Patient5 Progenitors nonactive 6 0.09660136 Progenitors_6
10x_3288_t2_AGTTATGACTGTAG-1 Patient5 Monocytes nonactive 2 0.05131254 Monocytes_2
10x_3288_t2_AGTTATGATTACCT-1 Patient5 Monocytes nonactive 2 0.02810538 Monocytes_2
10x_3288_t2_AGTTCTACACGTTG-1 Patient5 Monocytes nonactive 2 0.02888213 Monocytes_2
10x_3288_t2_AGTTCTACCCAACA-1 Patient5 Monocytes nonactive 2 0.06405745 Monocytes_2
10x_3288_t2_AGTTCTTGACCTTT-1 Patient5 Dendritic cells active 14 0.11777947

Dendritic cells_14
10x_3288_t2_AGTTCTTGCACTCC-1 Patient5 Monocytes nonactive 2 0.05565595 Monocytes_2
10x_3288_t2_AGTTCTTGCACTTT-1 Patient5 Monocytes nonactive 2 0.05265995 Monocytes_2
10x_3288_t2_AGTTCTTGCGAATC-1 Patient5 Monocytes nonactive 2 0.02155856 Monocytes_2
10x_3288_t2_AGTTCTTGCTGTCC-1 Patient5 Monocytes nonactive 3 0.06213937 Monocytes_3
10x_3288_t2_AGTTGTCTCCGTAA-1 Patient5 T cells nonactive 9 0.04425845 T cells_9
10x_3288_t2_AGTTGTCTGATAAG-1 Patient5 Monocytes nonactive 3 0.06695834 Monocytes_3
10x_3288_t2_AGTTTAGATACGAC-1 Patient5 Monocytes nonactive 3 0.05862025 Monocytes_3
10x_3288_t2_AGTTTGCTTGGTAC-1 Patient5 Monocytes nonactive 3 0.05843003 Monocytes_3
10x_3288_t2_ATAACAACCCTCCA-1 Patient5 Monocytes nonactive 3 0.06667301 Monocytes_3
10x_3288_t2_ATAACAACGAAGGC-1 Patient5 Monocytes nonactive 3 0.05823981 Monocytes_3
10x_3288_t2_ATAACAACGGGCAA-1 Patient5 Monocytes nonactive 2 0.02474478 Monocytes_2
10x_3288_t2_ATAACAACTTCATC-1 Patient5 Monocytes nonactive 2 0.05559254 Monocytes_2
10x_3288_t2_ATAACATGCCATGA-1 Patient5 Monocytes nonactive 2 0.04026378 Monocytes_2
10x_3288_t2_ATAACATGCCCTCA-1 Patient5 Monocytes nonactive 2 0.04319637 Monocytes_2
10x_3288_t2_ATAACATGCGTTAG-1 Patient5 Monocytes nonactive 2 0.02136833 Monocytes_2
10x_3288_t2_ATAACCCTAGTAGA-1 Patient5 Monocytes nonactive 2 0.06182233 Monocytes_2

10x_3288_t2_ATAACCCTAGTGTC-1 Patient5 Monocytes nonactive 2 0.06485004 Monocytes_2
10x_3288_t2_ATAACCCTCGGAGA-1 Patient5 Monocytes nonactive 2 0.07938622 Monocytes_2
10x_3288_t2_ATAACCCTCTCAAG-1 Patient5 Monocytes nonactive 3 0.03319384 Monocytes_3
10x_3288_t2_ATAACCCTGCCTTC-1 Patient5 Monocytes nonactive 2 0.02357175 Monocytes_2
10x_3288_t2_ATAACCCTGGTAAA-1 Patient5 Monocytes nonactive 3 0.03154524 Monocytes_3
10x_3288_t2_ATAACCCTTGAAGA-1 Patient5 Monocytes nonactive 2 0.05999937 Monocytes_2
10x_3288_t2_ATAACCCTTTGAGC-1 Patient5 Monocytes nonactive 2 0.05239046 Monocytes_2
10x_3288_t2_ATAAGTACACGCAT-1Patient5 Monocytes nonactive 3 0.07230042 Monocytes_3
10x_3288_t2_ATAAGTACAGTAGA-1 Patient5 Monocytes nonactive 2 0.02392049 Monocytes_2
10x_3288_t2_ATAAGTACCCTTCG-1 Patient5 T cells nonactive 9 0.09436624 T cells_9
10x_3288_t2_ATAAGTACCTGGAT-1Patient5 Monocytes nonactive 3 0.06884471 Monocytes_3
10x_3288_t2_ATAAGTACGCGTAT-1 Patient5 Monocytes nonactive 2 0.05134424 Monocytes_2
10x_3288_t2_ATAAGTACTTCACT-1 Patient5 Monocytes nonactive 3 0.0328768 Monocytes_3
10x_3288_t2_ATAAGTTGCCCTCA-1 Patient5 Monocytes nonactive 2 0.02179634 Monocytes_2
10x_3288_t2_ATAATCGAAGCAAA-1 Patient5 Monocytes nonactive 2 0.020227 Monocytes_2
10x_3288_t2_ATAATCGACAATCG-1 Patient5 Monocytes nonactive 3 0.03964555 Monocytes_3
10x_3288_t2_ATAATCGATGACAC-1 Patient5 Monocytes nonactive 2 0.03344747

Monocytes_2
10x_3288_t2_ATAATCGATTGGCA-1 Patient5 Monocytes nonactive 3 0.06232959 Monocytes_3
10x_3288_t2_ATAATCGATTTCGT-1 Patient5 Monocytes nonactive 2 0.02791516 Monocytes_2
10x_3288_t2_ATAATGACAGCTCA-1 Patient5 Monocytes nonactive 2 0.03154524 Monocytes_2
10x_3288_t2_ATAATGACTCCGAA-1 Patient5 Progenitors nonactive 1 0.10522478 Progenitors_1
10x_3288_t2_ATACAATGCTCCCA-1 Patient5 Monocytes nonactive 2 0.03617399 Monocytes_2
10x_3288_t2_ATACAATGGGTCAT-1 Patient5 Monocytes nonactive 3 0.03208421 Monocytes_3
10x_3288_t2_ATACAATGGTATCG-1 Patient5 Monocytes nonactive 2 0.03430347 Monocytes_2
10x_3288_t2_ATACACCTAATGCC-1 Patient5 Monocytes nonactive 3 0.06348678 Monocytes_3
10x_3288_t2_ATACACCTATTCTC-1 Patient5 Monocytes nonactive 2 0.04563756 Monocytes_2
10x_3288_t2_ATACCACTAATGCC-1 Patient5 T cells nonactive 9 0.06469152 T cells_9
10x_3288_t2_ATACCGGACATCAG-1 Patient5 T cells nonactive 9 0.06821064 T cells_9
10x_3288_t2_ATACCTACGTTGAC-1 Patient5 Monocytes nonactive 2 0.05846173 Monocytes_2
10x_3288_t2_ATACCTACTCTACT-1 Patient5 Monocytes nonactive 2 0.0257276 Monocytes_2
10x_3288_t2_ATACCTTGCAATCG-1 Patient5 B cells nonactive 7 0.04589119 B cells_7
10x_3288_t2_ATACCTTGCAGGAG-1 Patient5 Monocytes nonactive 2 0.02840657 Monocytes_2
10x_3288_t2_ATACCTTGCTCATT-1 Patient5 Monocytes nonactive 2 0.04392556 Monocytes_2
10x_3288_t2_ATACCTTGCTTATC-1 Patient5 Monocytes nonactive 2 0.02929427

Monocytes_2
10x_3288_t2_ATACCTTGGAATCC-1 Patient5 Monocytes nonactive 2 0.04062837 Monocytes_2
10x_3288_t2_ATACCTTGGTAGCT-1 Patient5 Monocytes nonactive 2 0.06369285 Monocytes_2
10x_3288_t2_ATACCTTGTTCCGC-1 Patient5 Monocytes nonactive 16 0.03725192 Monocytes_16
10x_3288_t2_ATACGGACTGAACC-1 Patient5 Monocytes nonactive 2 0.02734449 Monocytes_2
10x_3288_t2_ATACGGACTGCCTC-1 Patient5 Monocytes nonactive 2 0.05614736 Monocytes_2
10x_3288_t2_ATACGTCTATTGGC-1 Patient5 Monocytes nonactive 2 0.08907171 Monocytes_2
10x_3288_t2_ATACGTCTGGAAGC-1 Patient5 Monocytes nonactive 3 0.03569843 Monocytes_3
10x_3288_t2_ATACTCTGAAGGGC-1 Patient5 Monocytes nonactive 2 0.02436434 Monocytes_2
10x_3288_t2_ATACTCTGCGTACA-1 Patient5 Monocytes nonactive 3 0.0657853 Monocytes_3
10x_3288_t2_ATAGAACTGCAGAG-1 Patient5 Monocytes nonactive 2 0.01415573 Monocytes_2
10x_3288_t2_ATAGAACTTAGAAG-1 Patient5 Monocytes nonactive 3 0.05494262 Monocytes_3
10x_3288_t2_ATAGCCGATCGCTC-1 Patient5 Monocytes nonactive 2 0.06378797 Monocytes_2
10x_3288_t2_ATAGCGTGAAGCCT-1 Patient5 Monocytes nonactive 2 0.00391541 Monocytes_2
10x_3288_t2_ATAGCGTGACCAAC-1 Patient5 Monocytes nonactive 3 0.01742122 Monocytes_3
10x_3288_t2_ATAGCGTGGGACGA-1 Patient5 Monocytes nonactive 3 0.04864942 Monocytes_3
10x_3288_t2_ATAGCGTGTCCTGC-1 Patient5 Monocytes nonactive 2 0.02831146 Monocytes_2

10x_3288_t2_ATAGCTCTAGCTCA-1 Patient5 Monocytes nonactive 2 0.00867098 Monocytes_2
10x_3288_t2_ATAGCTCTCACTGA-1 Patient5 Monocytes nonactive 2 0.02398389 Monocytes_2
10x_3288_t2_ATAGCTCTGTTGCA-1 Patient5 Monocytes nonactive 2 0.03604718 Monocytes_2
10x_3288_t2_ATAGGAGAAGGGTG-1 Patient5 Monocytes nonactive 2 0.04070763 Monocytes_2
10x_3288_t2_ATAGGAGACCGATA-1 Patient5 Monocytes nonactive 3 0.05518039 Monocytes_3
10x_3288_t2_ATAGGAGACTCTCG-1 Patient5 Monocytes nonactive 2 0.03152939 Monocytes_2
10x_3288_t2_ATAGGAGATTGACG-1 Patient5 Monocytes nonactive 2 0.06608649 Monocytes_2
10x_3288_t2_ATAGGCTGGCAGTT-1 Patient5 Monocytes nonactive 2 0.00890876 Monocytes_2
10x_3288_t2_ATAGGCTGGGTAAA-1 Patient5 Monocytes nonactive 2 0.06857523 Monocytes_2
10x_3288_t2_ATAGGCTGTCAGGT-1 Patient5 Monocytes nonactive 2 0.01694566 Monocytes_2
10x_3288_t2_ATAGTCCTACACGT-1 Patient5 Monocytes nonactive 2 0.0240473 Monocytes_2
10x_3288_t2_ATAGTTGATTCAGG-1 Patient5 Monocytes nonactive 2 0.0227316 Monocytes_2
10x_3288_t2_ATAGTTGATTCCAT-1 Patient5 Monocytes nonactive 2 0.03003931 Monocytes_2
10x_3288_t2_ATATACGACTTGTT-1 Patient5 T cells nonactive 9 0.0556718 T cells_9
10x_3288_t2_ATATACGAGCAAGG-1 Patient5 Monocytes nonactive 2 0.04500349 Monocytes_2
10x_3288_t2_ATATACGAGCGGAA-1 Patient5 Monocytes nonactive 2 0.04207089 Monocytes_2
10x_3288_t2_ATATACGATATCTC-1 Patient5 Monocytes nonactive 3 0.02089278 Monocytes_3

10x_3288_t2_ATATAGTGAACTGC-1 Patient5 Monocytes nonactive 2 0.01853085 Monocytes_2
10x_3288_t2_ATATAGTGTCTAGG-1 Patient5 Monocytes nonactive 2 0.03065754 Monocytes_2
10x_3288_t2_ATATGAACGACAGG-1 Patient5 Monocytes nonactive 3 0.00618223 Monocytes_3
10x_3288_t2_ATATGAACGACGAG-1 Patient5 Monocytes nonactive 2 0.02674212 Monocytes_2
10x_3288_t2_ATATGAACTAAAGG-1 Patient5 Monocytes nonactive 2 0.03301947 Monocytes_2
10x_3288_t2_ATATGCCTTCAGTG-1 Patient5 T cells nonactive 9 0.06439034 T cells_9
10x_3288_t2_ATCAAATGCACTGA-1 Patient5 Monocytes nonactive 2 0.02120982 Monocytes_2
10x_3288_t2_ATCAAATGGACGTT-1 Patient5 Monocytes nonactive 2 0.04660453 Monocytes_2
10x_3288_t2_ATCAAATGTCTCAT-1 Patient5 Monocytes nonactive 3 0.05118572 Monocytes_3
10x_3288_t2_ATCAACCTATGACC-1 Patient5 Monocytes nonactive 2 0.02858094 Monocytes_2
10x_3288_t2_ATCAACCTCCCACT-1 Patient5 Monocytes nonactive 3 0.03357428 Monocytes_3
10x_3288_t2_ATCAACCTGTGCTA-1 Patient5 Monocytes nonactive 3 0.04451208 Monocytes_3
10x_3288_t2_ATCACACTCGAATC-1 Patient5 Monocytes nonactive 3 0.05960307 Monocytes_3
10x_3288_t2_ATCACACTGCTGTA-1 Patient5 Monocytes nonactive 2 0.02168537 Monocytes_2
10x_3288_t2_ATCACACTGGAAGC-1 Patient5 Monocytes nonactive 2 0.03308287 Monocytes_2
10x_3288_t2_ATCACACTTCCAAG-1 Patient5 Monocytes nonactive 2 0.04685816 Monocytes_2
10x_3288_t2_ATCACACTTGGAGG-1 Patient5 Monocytes nonactive 3 0.05518039 Monocytes_3

10x_3288_t2_ATCACGGAACCGAT-1 Patient5 Monocytes nonactive 2 0.06134678 Monocytes_2
10x_3288_t2_ATCACGGATGACTG-1 Patient5 Monocytes nonactive 2 0.04944201 Monocytes_2
10x_3288_t2_ATCACTACAAAAGC-1 Patient5 Monocytes nonactive 2 0.04769831 Monocytes_2
10x_3288_t2_ATCACTACAGGTTC-1 Patient5 Monocytes nonactive 2 0.04584364 Monocytes_2
10x_3288_t2_ATCACTACATACCG-1Patient5 Monocytes nonactive 2 0.04952127 Monocytes_2
10x_3288_t2_ATCACTACATGACC-1 Patient5 Monocytes nonactive 2 0.03087946 Monocytes_2
10x_3288_t2_ATCACTACCGAATC-1 Patient5 Monocytes nonactive 3 0.05280261 Monocytes_3
10x_3288_t2_ATCACTTGCAATCG-1 Patient5 Monocytes nonactive 3 0.07188828 Monocytes_3
10x_3288_t2_ATCACTTGCCGAAT-1Patient5 Monocytes nonactive 3 0.02628242 Monocytes_3
10x_3288_t2_ATCACTTGTGCCCT-1Patient5 Monocytes nonactive 3 0.05112231 Monocytes_3
10x_3288_t2_ATCAGGTGGGTGAG-1 Patient5 Monocytes nonactive 3 0.0788314 Monocytes_3
10x_3288_t2_ATCATCTGTTGCAG-1 Patient5 Monocytes nonactive 2 0.02915161 Monocytes_2
10x_3288_t2_ATCATGCTATCAGC-1 Patient5 Monocytes nonactive 3 0.04092955 Monocytes_3
10x_3288_t2_ATCATGCTCTCTCG-1 Patient5 Monocytes nonactive 2 0.04857016 Monocytes_2
10x_3288_t2_ATCATGCTGAGATA-1 Patient5 Monocytes nonactive 2 0.03309873 Monocytes_2
10x_3288_t2_ATCATGCTGGAAGC-1 Patient5 Monocytes nonactive 2 0.02376197 Monocytes_2
10x_3288_t2_ATCCAGGAAGTCGT-1 Patient5 Monocytes nonactive 2 0.01589944

Monocytes_2
10x_3288_t2_ATCCAGGAGAGGTG-1 Patient5 Monocytes nonactive 2 0.0695739
Monocytes_2
10x_3288_t2_ATCCAGGATAAGGA-1 Patient5 Monocytes nonactive 2 0.03504851
Monocytes_2
10x_3288_t2_ATCCAGGATTCCAT-1 Patient5 Monocytes nonactive 2 0.03558747
Monocytes_2
10x_3288_t2_ATCCATACGAGGGT-1 Patient5 Monocytes nonactive 2 0.02976983
Monocytes_2
10x_3288_t2_ATCCCGTGAGACTC-1 Patient5 Monocytes nonactive 3 0.0594287
Monocytes_3
10x_3288_t2_ATCCCGTGATCGGT-1 Patient5 Monocytes nonactive 3 0.04793609
Monocytes_3
10x_3288_t2_ATCCGCACACAGCT-1 Patient5 Monocytes nonactive 2 0.04987001
Monocytes_2
10x_3288_t2_ATCCGCACTGGTAC-1 Patient5 Monocytes nonactive 2 0.02988079
Monocytes_2
10x_3288_t2_ATCCGCACTGTGCA-1 Patient5 Monocytes nonactive 2 0.00431171
Monocytes_2
10x_3288_t2_ATCCGCACTTATCC-1 Patient5 Monocytes nonactive 2 0.05949211
Monocytes_2
10x_3288_t2_ATCCTAACCCACCT-1 Patient5 Monocytes nonactive 2 0.05229535
Monocytes_2
10x_3288_t2_ATCCTAACCTACTT-1 Patient5 Monocytes nonactive 2 0.07166635
Monocytes_2
10x_3288_t2_ATCCTAACCTGTAG-1 Patient5 Monocytes nonactive 2 0.01412403
Monocytes_2
10x_3288_t2_ATCCTAACGCAAGG-1 Patient5 Monocytes nonactive 2 0.04484497
Monocytes_2
10x_3288_t2_ATCCTAACGCGATT-1 Patient5 Monocytes nonactive 3 0.03988333
Monocytes_3
10x_3288_t2_ATCGACGATCAGGT-1 Patient5 Monocytes nonactive 2 0.0214793
Monocytes_2

10x_3288_t2_ATCGAGTGATTCCT-1 Patient5 Monocytes nonactive 3 0.06881301 Monocytes_3
10x_3288_t2_ATCGAGTGCTATTC-1 Patient5 Monocytes nonactive 2 0.05080528 Monocytes_2
10x_3288_t2_ATCGAGTGCTGTGA-1 Patient5 Monocytes nonactive 3 0.04016866 Monocytes_3
10x_3288_t2_ATCGAGTGGTCAAC-1 Patient5 Monocytes nonactive 2 0.00903557 Monocytes_2
10x_3288_t2_ATCGCAGAAAGAAC-1 Patient5 Monocytes nonactive 2 0.03070509 Monocytes_2
10x_3288_t2_ATCGCAGAGGAACG-1 Patient5 Monocytes nonactive 2 0.05381713 Monocytes_2
10x_3288_t2_ATCGCAGATGCCTC-1 Patient5 Monocytes nonactive 3 0.07475747 Monocytes_3
10x_3288_t2_ATCGCCACAACCAC-1 Patient5 Monocytes nonactive 2 0.04980661 Monocytes_2
10x_3288_t2_ATCGCCACGACGTT-1 Patient5 Monocytes nonactive 2 0.03729947 Monocytes_2
10x_3288_t2_ATCGCCACGGAGGT-1 Patient5 Monocytes nonactive 2 0.0278042 Monocytes_2
10x_3288_t2_ATCGCCACGGAGTG-1 Patient5 Progenitors nonactive 6 0.10720626 Progenitors_6
10x_3288_t2_ATCGCCACTGCCAA-1 Patient5 T cells nonactive 9 0.08929364 T cells_9
10x_3288_t2_ATCGCCTGCAGGAG-1 Patient5 Monocytes nonactive 2 0.04357682 Monocytes_2
10x_3288_t2_ATCGCCTGCTGTAG-1 Patient5 Monocytes nonactive 3 0.05743136 Monocytes_3
10x_3288_t2_ATCGCCTGGACGTT-1 Patient5 Monocytes nonactive 2 0.05698751 Monocytes_2
10x_3288_t2_ATCGCGCTGAGGGT-1 Patient5 Monocytes nonactive 3 0.05332572 Monocytes_3
10x_3288_t2_ATCGCGCTGATAGA-1 Patient5 Monocytes nonactive 2 0.02848583

Monocytes_2
10x_3288_t2_ATCGCGCTGGAGGT-1 Patient5 Monocytes nonactive 2 0.04169044 Monocytes_2
10x_3288_t2_ATCGCGCTTTCACT-1 Patient5 T cells nonactive 9 0.05253313 T cells_9
10x_3288_t2_ATCGGTGACCCTCA-1 Patient5 Monocytes nonactive 3 0.0708579 Monocytes_3
10x_3288_t2_ATCGGTGAGTCGAT-1 Patient5 Monocytes nonactive 2 0.0619967 Monocytes_2
10x_3288_t2_ATCGGTGATCAAGC-1 Patient5 Monocytes nonactive 2 0.0518198 Monocytes_2
10x_3288_t2_ATCGTTTGACACAC-1 Patient5 Monocytes nonactive 2 0.03433517 Monocytes_2
10x_3288_t2_ATCGTTTGCTGTGA-1 Patient5 Monocytes nonactive 2 0.02333397 Monocytes_2
10x_3288_t2_ATCGTTTGTTTCGT-1 Patient5 Monocytes nonactive 3 0.01493247 Monocytes_3
10x_3288_t2_ATCTACACATCACG-1 Patient5 Monocytes nonactive 2 0.03294021 Monocytes_2
10x_3288_t2_ATCTACACCCAATG-1 Patient5 Monocytes nonactive 2 0.03359013 Monocytes_2
10x_3288_t2_ATCTACACTGAGGG-1 Patient5 Monocytes nonactive 2 0.0282322 Monocytes_2
10x_3288_t2_ATCTACACTTCACT-1 Patient5 Monocytes nonactive 2 0.0501712 Monocytes_2
10x_3288_t2_ATCTACTGAGAAGT-1 Patient5 Monocytes nonactive 3 0.05717773 Monocytes_3
10x_3288_t2_ATCTACTGCCTGAA-1 Patient5 Monocytes nonactive 2 0.03384376 Monocytes_2
10x_3288_t2_ATCTACTGCTCAAG-1 Patient5 Monocytes nonactive 2 0.03308287 Monocytes_2
10x_3288_t2_ATCTACTGGACTAC-1 Patient5 Monocytes nonactive 2 0.0560364 Monocytes_2
10x_3288_t2_ATCTACTGGTTAGC-1 Patient5 Monocytes nonactive 2 0.02052818

Monocytes_2
10x_3288_t2_ATCTACTGTTTACC-1 Patient5 Monocytes nonactive 2 0.01710418
Monocytes_2
10x_3288_t2_ATCTCAACAGACTC-1 Patient5 Monocytes nonactive 2 0.03024539
Monocytes_2
10x_3288_t2_ATCTCAACCCTTTA-1 Patient5 Monocytes nonactive 2 0.0274396
Monocytes_2
10x_3288_t2_ATCTCAACGAACCT-1 Patient5 Monocytes nonactive 2 0.02387293
Monocytes_2
10x_3288_t2_ATCTGACTAAGCCT-1 Patient5 Monocytes nonactive 2 0.04734957
Monocytes_2
10x_3288_t2_ATCTGACTAAGTGA-1 Patient5 Monocytes nonactive 2 0.02981739
Monocytes_2
10x_3288_t2_ATCTGACTCTAGCA-1 Patient5 Monocytes nonactive 2 0.03891637
Monocytes_2
10x_3288_t2_ATCTGACTGACGAG-1 Patient5 Monocytes nonactive 2 0.03996259
Monocytes_2
10x_3288_t2_ATCTGACTTGGATC-1 Patient5 Monocytes nonactive 2 0.04560586
Monocytes_2
10x_3288_t2_ATCTGACTTTGCAG-1 Patient5 Monocytes nonactive 2 0.03484243
Monocytes_2
10x_3288_t2_ATCTGTTGACGGTT-1 Patient5 Monocytes nonactive 2 0.0311965
Monocytes_2
10x_3288_t2_ATCTGTTGGCCATA-1 Patient5 Monocytes nonactive 2 0.03989918
Monocytes_2
10x_3288_t2_ATCTTGACAAGAAC-1 Patient5 T cells nonactive 9 0.07074694 T cells_9
10x_3288_t2_ATCTTGACATGTCG-1 Patient5 Monocytes nonactive 2 0.04311711
Monocytes_2
10x_3288_t2_ATCTTGACGAACTC-1 Patient5 Monocytes nonactive 2 0.04215015
Monocytes_2
10x_3288_t2_ATCTTGACTTCCGC-1 Patient5 Monocytes nonactive 13 0.00924165
Monocytes_13

10x_3288_t2_ATCTTGACTTCGTT-1 Patient5 Progenitors active 6 0.17048697
Progenitors_6
10x_3288_t2_ATCTTTCTCTAGCA-1 Patient5 Monocytes nonactive 2 0.0392017
Monocytes_2
10x_3288_t2_ATCTTTCTGATACC-1 Patient5 Monocytes nonactive 3 0.06586456
Monocytes_3
10x_3288_t2_ATGAAACTATGACC-1 Patient5 Monocytes nonactive 2 0.03788599
Monocytes_2
10x_3288_t2_ATGAAACTCCTAAG-1 Patient5 Monocytes nonactive 2 0.04468645
Monocytes_2
10x_3288_t2_ATGAAACTGAACTC-1 Patient5 Monocytes nonactive 2 0.06919346
Monocytes_2
10x_3288_t2_ATGAAGGAAGTCAC-1 Patient5 Monocytes nonactive 2 0.02206582
Monocytes_2
10x_3288_t2_ATGAAGGACCATAG-1 Patient5 Monocytes nonactive 2 0.03909074
Monocytes_2
10x_3288_t2_ATGACGTGCCAAGT-1 Patient5 Monocytes nonactive 2 0.02503012
Monocytes_2
10x_3288_t2_ATGACGTGGGATTC-1 Patient5 Monocytes nonactive 2 0.04446452
Monocytes_2
10x_3288_t2_ATGACGTGGGTCTA-1 Patient5 Monocytes nonactive 2 0.0366654
Monocytes_2
10x_3288_t2_ATGAGAGAGAACCT-1 Patient5 Monocytes nonactive 2 0.03267072
Monocytes_2
10x_3288_t2_ATGAGAGAGGGAGT-1 Patient5 Monocytes nonactive 3 0.01234861
Monocytes_3
10x_3288_t2_ATGAGAGATTGGTG-1 Patient5 Monocytes nonactive 2 0.0547841
Monocytes_2
10x_3288_t2_ATGATAACACACCA-1 Patient5 Monocytes nonactive 3 0.03488999
Monocytes_3
10x_3288_t2_ATGATATGCCTTAT-1 Patient5 Monocytes nonactive 2 0.03753725
Monocytes_2
10x_3288_t2_ATGCACGATTCCCG-1 Patient5 Monocytes nonactive 2 0.02075011

Monocytes_2
10x_3288_t2_ATGCAGACATGCCA-1 Patient5 Monocytes nonactive 2 0.03707755
Monocytes_2
10x_3288_t2_ATGCAGACCACACA-1 Patient5 Monocytes nonactive 2 0.03514362
Monocytes_2
10x_3288_t2_ATGCAGACCTCCAC-1 Patient5 Monocytes nonactive 3 0.04278422
Monocytes_3
10x_3288_t2_ATGCAGACGAAAGT-1 Patient5 Monocytes nonactive 3 0.02599708
Monocytes_3
10x_3288_t2_ATGCAGACGTTACG-1 Patient5 Monocytes nonactive 2 0.02303278
Monocytes_2
10x_3288_t2_ATGCAGACTGTTTC-1 Patient5 Monocytes nonactive 2 0.0354131
Monocytes_2
10x_3288_t2_ATGCAGACTTTCTG-1 Patient5 Monocytes nonactive 2 0.0349851
Monocytes_2
10x_3250_t1_AAAGCCTGCCGTTC-1 Patient2 T cells nonactive 9 0.10214951 T cells_9
10x_3250_t1_AAGAGATGAGCACT-1 Patient2 T cells nonactive 9 0.06580115 T cells_9
10x_3250_t1_AAGTTATGAGTCTG-1 Patient2 T cells nonactive 9 0.10858538 T cells_9
10x_3250_t1_AATGGCTGGTCGAT-1 Patient2 T cells nonactive 9 0.06342337 T cells_9
10x_3250_t1_ACCCGTACCCCTAC-1 Patient2 Monocytes nonactive 16 0.03179887
Monocytes_16
10x_3250_t1_ACGTTGGAACCAAC-1 Patient2 Progenitors nonactive 5 0.07558176
Progenitors_5
10x_3250_t1_ACGTTGGACCCACT-1 Patient2 Progenitors nonactive 5 0.06361359
Progenitors_5
10x_3250_t1_ACTACTACAGTACC-1 Patient2 Monocytes nonactive 16 0.03563503
Monocytes_16
10x_3250_t1_ACTCAGGAAGTCAC-1 Patient2 T cells nonactive 9 0.08391985 T cells_9

10x_3250_t1_ACTCGCACTCTACT-1 Patient2 Progenitors nonactive 5 0.05183565 Progenitors_5
10x_3250_t1_ACTCGCACTGTTCT-1 Patient2 Progenitors nonactive 5 0.04688986 Progenitors_5
10x_3250_t1_ACTGAGACGTGAGG-1 Patient2 Monocytes nonactive 16 0.09116416 Monocytes_16
10x_3250_t1_ACTTAAGACCACCT-1 Patient2 T cells nonactive 9 0.08873882 T cells_9
10x_3250_t1_AGAGTCACTCTGGA-1 Patient2 T cells nonactive 9 0.11040834 T cells_9
10x_3250_t1_AGGATGCTTGTTTC-1 Patient2 T cells nonactive 9 0.08544163 T cells_9
10x_3250_t1_AGTGTTCTTTACCT-1 Patient2 Monocytes nonactive 16 0.09011794 Monocytes_16
10x_3250_t1_ATACACCTATCGGT-1 Patient2 T cells nonactive 9 0.06751316 T cells_9
10x_3250_t1_ATCATCTGCTCTAT-1 Patient2 Monocytes nonactive 16 0.0586044 Monocytes_16
10x_3250_t1_ATCGTTTGAACGGG-1 Patient2 Progenitors nonactive 5 0.08564771 Progenitors_5
10x_3250_t1_ATCGTTTGGTTCAG-1 Patient2 T cells nonactive 9 0.08975334 T cells_9
10x_3250_t1_ATGCCAGACGTCTC-1 Patient2 T cells nonactive 9 0.0741868 T cells_9
10x_3250_t1_ATGTAAACCTTAGG-1 Patient2 T cells nonactive 9 0.07004946 T cells_9
10x_3250_t1_ATTCAAGACCCACT-1 Patient2 T cells nonactive 9 0.10871219 T cells_9
10x_3250_t1_CAATAATGAAGTAG-1 Patient2 T cells nonactive 9 0.04993342 T cells_9
10x_3250_t1_CACAATCTCTGAGT-1 Patient2 T cells nonactive 9 0.05822396 T cells_9
10x_3250_t1_CACAGCCTCTCTCG-1 Patient2 T cells nonactive 9 0.09834506 T cells_9

10x_3250_t1_CACAGTGATGACAC-1 Patient2 CD4+ T cells nonactive 11 0.0784351 CD4+ T cells_11
10x_3250_t1_CACAGTGATGACTG-1 Patient2 Progenitors nonactive 5 0.09845603 Progenitors_5
10x_3250_t1_CACAGTGATGCATG-1 Patient2 Progenitors nonactive 5 0.07490013 Progenitors_5
10x_3250_t1_CACCGGGAAGATGA-1 Patient2 T cells nonactive 9 0.10075455 T cells_9
10x_3250_t1_CAGTTACTTTCGGA-1 Patient2 T cells nonactive 9 0.05665462 T cells_9
10x_3250_t1_CATGAGACGGGATG-1 Patient2 T cells nonactive 9 0.08376133 T cells_9
10x_3250_t1_CATGGCCTAGAGGC-1 Patient2 T cells nonactive 9 0.06938368 T cells_9
10x_3250_t1_CATTAGCTACGGTT-1 Patient2 T cells nonactive 9 0.08902416 T cells_9
10x_3250_t1_CCAGCACTTCACGA-1 Patient2 Monocytes nonactive 3 0.03680807 Monocytes_3
10x_3250_t1_CCCGAACTTTGACG-1 Patient2 Progenitors nonactive 5 0.05811299 Progenitors_5
10x_3250_t1_CCTCGAACATGTCG-1 Patient2 T cells nonactive 9 0.09723543 T cells_9
10x_3250_t1_CGACAAACCGTGAT-1 Patient2 T cells nonactive 9 0.11465665 T cells_9
10x_3250_t1_CGAGATTGATCGTG-1 Patient2 Progenitors nonactive 5 0.10121425 Progenitors_5
10x_3250_t1_CGAGCGTGATCGTG-1 Patient2 Progenitors nonactive 5 0.11083635 Progenitors_5
10x_3250_t1_CGAGCGTGCTGAGT-1 Patient2 T cells nonactive 9 0.06259907 T cells_9
10x_3250_t1_CGCACTTGATCGTG-1 Patient2 Progenitors nonactive 5 0.09785366 Progenitors_5
10x_3250_t1_CGCGATCTTTCCCG-1 Patient2 Dendritic cells nonactive 10 0.06551582 Dendritic cells_10

10x_3250_t1_CGCGATCTTTGACG-1 Patient2 Progenitors nonactive 5 0.10674656 Progenitors_5
10x_3250_t1_CTAAGGTGAGAACA-1 Patient2 T cells nonactive 9 0.07910088 T cells_9
10x_3250_t1_CTAGTTTGGACAGG-1 Patient2 T cells nonactive 9 0.10593811 T cells_9
10x_3250_t1_CTCAGCTGGGGACA-1 Patient2 T cells nonactive 9 0.08441126 T cells_9
10x_3250_t1_CTCGACTGCTCGAA-1 Patient2 T cells nonactive 9 0.04877623 T cells_9
10x_3250_t1_CTCGCATGCGACAT-1 Patient2 Monocytes nonactive 16 0.03993089 Monocytes_16
10x_3250_t1_CTCTAAACACACGT-1 Patient2 T cells nonactive 9 0.07884725 T cells_9
10x_3250_t1_GACACTGAAGCATC-1 Patient2 Progenitors active 5 0.16707882 Progenitors_5
10x_3250_t1_GACAGTACCTCATT-1 Patient2 Monocytes nonactive 16 0.05966648 Monocytes_16
10x_3250_t1_GAGATAGACGTAGT-1 Patient2 T cells nonactive 9 0.06572189 T cells_9
10x_3250_t1_GATAGCACCAACCA-1 Patient2 Progenitors active 5 0.12542007 Progenitors_5
10x_3250_t1_GATAGCACCAAGCT-1 Patient2 Progenitors active 5 0.15945406 Progenitors_5
10x_3250_t1_GATAGCACCTCGCT-1 Patient2 Progenitors active 5 0.16782385 Progenitors_5
10x_3250_t1_GATGCCCTAGATGA-1 Patient2 T cells nonactive 9 0.07694503 T cells_9
10x_3250_t1_GCCAAAACACGTGT-1 Patient2 T cells nonactive 9 0.08360282 T cells_9
10x_3250_t1_GCCGGAACCGCAAT-1 Patient2 T cells active 9 0.1268626 T cells_9
10x_3250_t1_GCCTCAACTTGGTG-1 Patient2 T cells nonactive 9 0.08434785 T cells_9

10x_3250_t1_GCGTACCTACCCAA-1 Patient2 Progenitors nonactive 5 0.07889481 Progenitors_5
10x_3250_t1_GCGTACCTACGCAT-1Patient2 Progenitors nonactive 5 0.09149705 Progenitors_5
10x_3250_t1_GGATGTTGTGAGGG-1 Patient2 T cells nonactive 9 0.04842749 T cells_9
10x_3250_t1_GGCGACTGTCACCC-1 Patient2 Progenitors nonactive 5 0.07822903 Progenitors_5
10x_3250_t1_GGCTAATGTCACCC-1 Patient2 Progenitors nonactive 5 0.04952127 Progenitors_5
10x_3250_t1_GGGCAGCTGCCAAT-1 Patient2 T cells nonactive 9 0.04287934 T cells_9
10x_3250_t1_GGGCAGCTTAGACC-1 Patient2 T cells nonactive 9 0.05424513 T cells_9
10x_3250_t1_GGTACATGCGAGTT-1 Patient2 T cells nonactive 9 0.08762919 T cells_9
10x_3250_t1_GGTGGAGAAAGAGT-1 Patient2 Progenitors active 5 0.11855621 Progenitors_5
10x_3250_t1_GTACCCTGGTTAGC-1 Patient2 Progenitors nonactive 5 0.08082874 Progenitors_5
10x_3250_t1_GTAGCATGGTTGCA-1 Patient2 Progenitors nonactive 5 0.08799379 Progenitors_5
10x_3250_t1_GTCACAGAGGTTAC-1 Patient2 T cells nonactive 9 0.04606556 T cells_9
10x_3250_t1_GTCCAAGATGCACA-1 Patient2 Progenitors nonactive 5 0.06112485 Progenitors_5
10x_3250_t1_GTCCAGCTTCGATG-1 Patient2 T cells nonactive 9 0.07521717 T cells_9
10x_3250_t1_GTGATGACCCTAAG-1 Patient2 T cells nonactive 9 0.05562425 T cells_9
10x_3250_t1_TAATGTGACTACCC-1 Patient2 T cells nonactive 9 0.03555577 T cells_9
10x_3250_t1_TACGACGACTTACT-1 Patient2 Monocytes nonactive 16 0.03428762

Monocytes_16
10x_3250_t1_TACTTTCTGCTACA-1 Patient2 T cells nonactive 9 0.07352102 T cells_9
10x_3250_t1_TAGCCCTGCATACG-1 Patient2 Monocytes nonactive 16 0.05513284 Monocytes_16
10x_3250_t1_TAGGTGTGCGTGAT-1 Patient2 T cells nonactive 9 0.09079957 T cells_9
10x_3250_t1_TAGTCTTGCTTCGC-1 Patient2 T cells active 9 0.12733815 T cells_9
10x_3250_t1_TAGTTCACGTAAGA-1 Patient2 T cells nonactive 9 0.02568005 T cells_9
10x_3250_t1_TATAAGTGGGGCAA-1 Patient2 Monocytes nonactive 8 0.08189081 Monocytes_8
10x_3250_t1_TATAGATGGGCATT-1 Patient2 Progenitors active 5 0.11901592 Progenitors_5
10x_3250_t1_TATCCAACACGACT-1 Patient2 T cells nonactive 9 0.08384059 T cells_9
10x_3250_t1_TATTGCTGGGCATT-1 Patient2 Progenitors nonactive 5 0.11432376 Progenitors_5
10x_3250_t1_TATTGCTGTTGCAG-1 Patient2 Progenitors nonactive 5 0.04505104 Progenitors_5
10x_3250_t1_TCACAACTGACTAC-1 Patient2 T cells nonactive 9 0.05676558 T cells_9
10x_3250_t1_TCACCGTGTTGGCA-1 Patient2 T cells nonactive 9 0.0755025 T cells_9
10x_3250_t1_TCATCATGTGGAAA-1 Patient2 T cells nonactive 9 0.05784351 T cells_9
10x_3250_t1_TCGCAGCTAGAGAT-1 Patient2 T cells nonactive 9 0.07334665 T cells_9
10x_3250_t1_TCTTGATGTCAGGT-1 Patient2 T cells nonactive 9 0.04194407 T cells_9
10x_3250_t1_TCTTGATGTTGCAG-1 Patient2 Progenitors nonactive 5 0.06971657 Progenitors_5
10x_3250_t1_TCTTGATGTTGGTG-1 Patient2 Progenitors nonactive 5 0.04123074 Progenitors_5
10x_3250_t1_TGAACCGATTCTCA-1 Patient2 T cells nonactive 9 0.07932281 T

cells_9

10x_3250_t1_TGTGGATGTTGCAG-1 Patient2 Progenitors nonactive 5 0.08032148 Progenitors_5

10x_3250_t1_TTAGGGTGCCTACC-1 Patient2 T cells nonactive 9 0.02889798 T cells_9

10x_3250_t1_TTATGCACAGCACT-1 Patient2 T cells nonactive 9 0.10841101 T cells_9

10x_3250_t1_TTCAGTACGGAGTG-1 Patient2 T cells nonactive 9 0.07751569 T cells_9

10x_3250_t1_TTTAGCTGGGCATT-1 Patient2 Progenitors nonactive 5 0.08154207 Progenitors_5

10x_3281_AAACATACCTGAGT-1 Patient4 Monocytes nonactive 3 0.0282322 Monocytes_3

10x_3281_AAACATACGTGTAC-1 Patient4 Monocytes nonactive 3 0.04674719 Monocytes_3

10x_3281_AAACATTGGGAAGC-1 Patient4 Monocytes nonactive 3 0.0560998 Monocytes_3

10x_3281_AAACGCACTTGCAG-1 Patient4 Monocytes nonactive 3 0.05724114 Monocytes_3

10x_3281_AAACGGCTCATGGT-1 Patient4 Monocytes nonactive 3 0.05250143 Monocytes_3

10x_3281_AAAGAGACCCTTAT-1 Patient4 Monocytes nonactive 3 0.04349756 Monocytes_3

10x_3281_AAAGATCTCCCTAC-1 Patient4 Monocytes nonactive 3 0.07696088 Monocytes_3

10x_3281_AAAGCCTGATGACC-1 Patient4 Monocytes nonactive 3 0.06036396 Monocytes_3

10x_3281_AAAGCCTGGGTATC-1 Patient4 Monocytes nonactive 2 0.04246719 Monocytes_2

10x_3281_AAAGGCCTATGACC-1 Patient4 Monocytes nonactive 3 0.03625325 Monocytes_3

10x_3281_AAATGGGAGCGAAG-1 Patient4 Monocytes nonactive 2 0.0138704 Monocytes_2

10x_3281_AAATGTTGCACTAG-1	Patient4 Monocytes	nonactive	3	0.03336821	Monocytes_3
10x_3281_AACACGTGCCGAAT-1	Patient4 Monocytes	nonactive	2	0.04349756	Monocytes_2
10x_3281_AACACTCTTGGGAG-1	Patient4 Monocytes	nonactive	3	0.02434849	Monocytes_3
10x_3281_AACAGAGAACGTAC-1	Patient4 Monocytes	nonactive	3	0.05243802	Monocytes_3
10x_3281_AACATTGATAGAGA-1	Patient4 Monocytes	nonactive	3	0.0155824	Monocytes_3
10x_3281_AACCACGATCTCCG-1	Patient4 Monocytes	nonactive	3	0.04023207	Monocytes_3
10x_3281_AACCCAGACCGTTC-1	Patient4 Monocytes	nonactive	3	0.02425338	Monocytes_3
10x_3281_AACCCAGAGTTAGC-1	Patient4 Monocytes	nonactive	3	0.03151354	Monocytes_3
10x_3281_AACGCAACCATTCT-1	Patient4 Monocytes	nonactive	3	0.04617653	Monocytes_3
10x_3281_AACGCATGGTGAGG-1	Patient4 Monocytes	nonactive	3	0.04696912	Monocytes_3
10x_3281_AACGCCCTAACGGG-1	Patient4 Monocytes	nonactive	3	0.07142857	Monocytes_3
10x_3281_AACGTCGACATTCT-1	Patient4 Monocytes	nonactive	3	0.05811299	Monocytes_3
10x_3281_AACGTCGATTCACT-1	Patient4 Monocytes	nonactive	3	0.03802866	Monocytes_3
10x_3281_AACTACCTAGCGGA-1	Patient4 Monocytes	nonactive	3	0.09809143	Monocytes_3
10x_3281_AACTCACTGCTTAG-1	Patient4 Monocytes	nonactive	2	0.06548412	Monocytes_2
10x_3281_AACTCGGAAGTCTG-1	Patient4 Monocytes	nonactive	3	0.02636168	Monocytes_3
10x_3281_AACTCTTGACTGGT-1	Patient4 Monocytes	nonactive	3	0.01626403	

Monocytes_3
10x_3281_AACTTGCTCGATAC-1 Patient4 Monocytes nonactive 3 0.03263902
Monocytes_3
10x_3281_AAGAACGAAGCATC-1 Patient4 Monocytes nonactive 3 0.05765329
Monocytes_3
10x_3281_AAGAACGAAGTGTC-1 Patient4 Monocytes nonactive 3 0.03154524
Monocytes_3
10x_3281_AAGAGATGGTATGC-1 Patient4 Monocytes nonactive 3 0.06928857
Monocytes_3
10x_3281_AAGATGGATCCTGC-1 Patient4 Monocytes nonactive 3 0.07497939
Monocytes_3
10x_3281_AAGATGGATGGTCA-1 Patient4 Monocytes nonactive 2 0.04909327
Monocytes_2
10x_3281_AAGCCAACAGGCGA-1 Patient4 Monocytes nonactive 3 0.03618984
Monocytes_3
10x_3281_AAGGTGCTAGTAGA-1 Patient4 Monocytes nonactive 3 0.03106968
Monocytes_3
10x_3281_AAGTATACATGACC-1 Patient4 Monocytes nonactive 2 0.08349185
Monocytes_2
10x_3281_AAGTGCACTCGATG-1 Patient4 Monocytes nonactive 3 0.04883964
Monocytes_3
10x_3281_AAGTGGCTGTTACG-1 Patient4 Monocytes nonactive 3 0.04211845
Monocytes_3
10x_3281_AAGTTCCTCTGTTT-1 Patient4 Monocytes nonactive 3 0.06548412
Monocytes_3
10x_3281_AATACCCTTGTGAC-1 Patient4 Monocytes nonactive 3 0.03267072
Monocytes_3
10x_3281_AATATCGAAACCGT-1 Patient4 Monocytes nonactive 3 0.04944201
Monocytes_3
10x_3281_AATATCGATTCGCC-1 Patient4 Monocytes nonactive 3 0.04503519
Monocytes_3
10x_3281_AATCAAACCCTAAG-1 Patient4 Monocytes nonactive 3 0.03446199
Monocytes_3

10x_3281_AATCAAACTTTGGG-1	Patient4	Monocytes	nonactive	3	0.04807875	Monocytes_3
10x_3281_AATCCGGACCTAAG-1	Patient4	Monocytes	nonactive	2	0.08177985	Monocytes_2
10x_3281_AATCCGGAGTTGTG-1	Patient4	Monocytes	nonactive	3	0.05126498	Monocytes_3
10x_3281_AATCGGTGACACGT-1	Patient4	Monocytes	nonactive	3	0.02219263	Monocytes_3
10x_3281_AATGGAGAGTGTTG-1	Patient4	Progenitors	active	6	0.122646	Progenitors_6
10x_3281_AATGGAGATGTAGC-1	Patient4	Monocytes	nonactive	3	0.02996005	Monocytes_3
10x_3281_AATGTAACACAGTC-1	Patient4	Monocytes	nonactive	3	0.05411832	Monocytes_3
10x_3281_AATGTAACTTACTC-1	Patient4	Monocytes	nonactive	3	0.03782259	Monocytes_3
10x_3281_AATGTTGACGCATA-1	Patient4	Monocytes	nonactive	3	0.07707184	Monocytes_3
10x_3281_AATGTTGATTGTCT-1	Patient4	Monocytes	nonactive	3	0.04419504	Monocytes_3
10x_3281_AATTCCTGCTGACA-1	Patient4	Monocytes	nonactive	3	0.03503265	Monocytes_3
10x_3281_AATTGTGATAGAGA-1	Patient4	Monocytes	nonactive	2	0.02415827	Monocytes_2
10x_3281_ACAAATTGCGTCTC-1	Patient4	Monocytes	nonactive	3	0.043022	Monocytes_3
10x_3281_ACAAATTGTTGACG-1	Patient4	Monocytes	nonactive	3	0.03924925	Monocytes_3
10x_3281_ACAAGCACTGATGC-1	Patient4	Monocytes	nonactive	2	0.01883203	Monocytes_2
10x_3281_ACACCCTGGGTCTA-1	Patient4	Monocytes	nonactive	3	0.04682645	Monocytes_3
10x_3281_ACACGATGGACTAC-1	Patient4	Monocytes	nonactive	3	0.0400894	

Monocytes_3
10x_3281_ACACGTGACGCATA-1 Patient4 Monocytes nonactive 3 0.05925433 Monocytes_3
10x_3281_ACAGCAACTCTTAC-1 Patient4 Monocytes nonactive 3 0.04646186 Monocytes_3
10x_3281_ACAGTGTGTAGACC-1 Patient4 Monocytes nonactive 2 0.04514615 Monocytes_2
10x_3281_ACATCACTGGTTTG-1 Patient4 Monocytes nonactive 3 0.04051741 Monocytes_3
10x_3281_ACCACAGATCTCTA-1 Patient4 Monocytes nonactive 3 0.03137087 Monocytes_3
10x_3281_ACCACGCTAAGCCT-1 Patient4 Monocytes nonactive 2 0.05966648 Monocytes_2
10x_3281_ACCAGCCTGCATCA-1 Patient4 Monocytes nonactive 2 0.05467313 Monocytes_2
10x_3281_ACCCGTACGTTGTG-1 Patient4 Monocytes nonactive 3 0.03723607 Monocytes_3
10x_3281_ACCCGTACTGAACC-1 Patient4 Monocytes nonactive 3 0.06397819 Monocytes_3
10x_3281_ACCCTCGATTCTGT-1 Patient4 Monocytes nonactive 3 0.06095048 Monocytes_3
10x_3281_ACCGTGCTGGAGCA-1 Patient4 Monocytes nonactive 3 0.04398897 Monocytes_3
10x_3281_ACCTGAGATCCGTC-1 Patient4 Monocytes nonactive 3 0.04942616 Monocytes_3
10x_3281_ACCTGGCTACCACA-1 Patient4 Monocytes nonactive 2 0.03921755 Monocytes_2
10x_3281_ACCTGGCTTGTGAC-1 Patient4 T cells nonactive 9 0.07623169 T cells_9
10x_3281_ACGAACTGAGCGTT-1 Patient4 Monocytes nonactive 3 0.04186482 Monocytes_3
10x_3281_ACGAAGCTCAACTG-1 Patient4 T cells nonactive 9 0.08523556 T cells_9
10x_3281_ACGATCGAACCTAG-1 Patient4 Monocytes nonactive 3 0.03921755 Monocytes_3

10x_3281_ACGATCGAGTCTAG-1 Patient4 Monocytes nonactive 3 0.03983577 Monocytes_3
10x_3281_ACGCCTTGCTCTTA-1 Patient4 Progenitors active 6 0.12215459 Progenitors_6
10x_3281_ACGCCTTGCTTCTA-1 Patient4 Monocytes nonactive 3 0.03271828 Monocytes_3
10x_3281_ACGCGGTGTGTAGC-1 Patient4 Monocytes nonactive 3 0.04390971 Monocytes_3
10x_3281_ACGCTCACACGTGT-1 Patient4 Monocytes nonactive 2 0.05578277 Monocytes_2
10x_3281_ACGGCGTGACTCTT-1 Patient4 Monocytes nonactive 2 0.02445945 Monocytes_2
10x_3281_ACGTAGACTTCGTT-1 Patient4 Monocytes nonactive 2 0.06064929 Monocytes_2
10x_3281_ACGTTGGAATGGTC-1 Patient4 Monocytes nonactive 3 0.03985163 Monocytes_3
10x_3281_ACGTTTACAGCTCA-1 Patient4 Monocytes nonactive 3 0.01269736 Monocytes_3
10x_3281_ACTACGGAGTCTGA-1 Patient4 Monocytes nonactive 3 0.0653573 Monocytes_3
10x_3281_ACTACGGATGACTG-1 Patient4 Monocytes nonactive 3 0.03937607 Monocytes_3
10x_3281_ACTATCACCTCTAT-1 Patient4 Monocytes nonactive 3 0.04514615 Monocytes_3
10x_3281_ACTCAGGATGAGCT-1 Patient4 Monocytes nonactive 3 0.06366115 Monocytes_3
10x_3281_ACTCCCGACCCTAC-1 Patient4 Monocytes nonactive 3 0.04262571 Monocytes_3
10x_3281_ACTCTCCTGCTTAG-1 Patient4 Monocytes nonactive 2 0.03263902 Monocytes_2
10x_3281_ACTCTCCTGTTACG-1 Patient4 Monocytes nonactive 3 0.02935768 Monocytes_3
10x_3281_ACTTCCCTCTCTAT-1 Patient4 Monocytes nonactive 3 0.05354765

Monocytes_3						
10x_3281_ACTTGACTTTGTGG-1	Patient4	Monocytes	nonactive	3	0.05668632	Monocytes_3
10x_3281_ACTTTGTGATGCTG-1	Patient4	Monocytes	nonactive	3	0.03742629	Monocytes_3
10x_3281_ACTTTGTGTCAGTG-1	Patient4	Monocytes	nonactive	2	0.03011857	Monocytes_2
10x_3281_AGAAACGATGTGCA-1	Patient4	Monocytes	nonactive	3	0.04215015	Monocytes_3
10x_3281_AGAATACTTTCATC-1	Patient4	Monocytes	nonactive	3	0.02632997	Monocytes_3
10x_3281_AGAATTTGGCTACA-1	Patient4	Monocytes	nonactive	3	0.03636421	Monocytes_3
10x_3281_AGACACTGGCTTCC-1	Patient4	Monocytes	nonactive	3	0.02384123	Monocytes_3
10x_3281_AGACGTACGAGAGC-1	Patient4	Monocytes	nonactive	3	0.03682392	Monocytes_3
10x_3281_AGAGAATGATGTGC-1	Patient4	Monocytes	nonactive	2	0.06662545	Monocytes_2
10x_3281_AGAGCTACGTCGAT-1	Patient4	Monocytes	nonactive	3	0.06932027	Monocytes_3
10x_3281_AGAGGTCTGCTAAC-1	Patient4	Monocytes	nonactive	3	0.04700082	Monocytes_3
10x_3281_AGATATTGCCCTTG-1	Patient4	Monocytes	nonactive	3	0.05987255	Monocytes_3
10x_3281_AGATCTCTAGCGGA-1	Patient4	Monocytes	nonactive	3	0.02425338	Monocytes_3
10x_3281_AGATTAACTGATGC-1	Patient4	Monocytes	nonactive	3	0.02728109	Monocytes_3
10x_3281_AGCAAGCTGCCTTC-1	Patient4	Monocytes	nonactive	2	0.02598123	Monocytes_2
10x_3281_AGCACAACGCGAGA-1	Patient4	Monocytes	nonactive	3	0.02204997	Monocytes_3

10x_3281_AGCATTCTGACGTT-1 Patient4 Monocytes nonactive 3 0.03106968 Monocytes_3
10x_3281_AGCCAATGCTAAGC-1 Patient4 Monocytes nonactive 3 0.03114894 Monocytes_3
10x_3281_AGCCACCTGTAGCT-1 Patient4 Monocytes nonactive 3 0.02618731 Monocytes_3
10x_3281_AGCCGGACACGTGT-1 Patient4 B cells nonactive 7 0.0354448 B cells_7
10x_3281_AGCCGTCTACGTTG-1 Patient4 T cells active 9 0.12064866 T cells_9
10x_3281_AGCCGTCTTATCGG-1 Patient4 Monocytes nonactive 3 0.06396234 Monocytes_3
10x_3281_AGCCTCTGACGTGT-1 Patient4 Monocytes nonactive 3 0.07215776 Monocytes_3
10x_3281_AGCGATTGCTATGG-1 Patient4 Monocytes nonactive 3 0.042594 Monocytes_3
10x_3281_AGCGGCTGGCGGAA-1 Patient4 Monocytes nonactive 3 0.0627893 Monocytes_3
10x_3281_AGCTCGCTCAAAGA-1 Patient4 Monocytes nonactive 3 0.03294021 Monocytes_3
10x_3281_AGCTCGCTCCTTCG-1 Patient4 Monocytes nonactive 3 0.02704331 Monocytes_3
10x_3281_AGCTGAACTGTCAG-1 Patient4 Monocytes nonactive 3 0.06551582 Monocytes_3
10x_3281_AGGAAATGTTTGTC-1 Patient4 Monocytes nonactive 3 0.05221609 Monocytes_3
10x_3281_AGGAACCTAGCTAC-1 Patient4 Monocytes nonactive 3 0.04314882 Monocytes_3
10x_3281_AGGCAGGACGTTGA-1 Patient4 Monocytes nonactive 3 0.02309619 Monocytes_3
10x_3281_AGGCCTCTCTTACT-1 Patient4 Monocytes nonactive 3 0.05835077 Monocytes_3
10x_3281_AGGGAGTGCCGATA-1 Patient4 T cells nonactive 9 0.05186735 T cells_9
10x_3281_AGGGCCTGTTCGGA-1 Patient4 Monocytes nonactive 3 0.02296937 Monocytes_3

10x_3281_AGGTCTGACATTTC-1 Patient4 Monocytes nonactive 3 0.0278042
Monocytes_3
10x_3281_AGGTGGGACGCAAT-1 Patient4 Monocytes nonactive 3 0.04734957
Monocytes_3
10x_3281_AGGTGTTGCACACA-1 Patient4 Monocytes nonactive 3 0.02970642
Monocytes_3
10x_3281_AGGTTCGATGCAGT-1 Patient4 Monocytes nonactive 3 0.06592797
Monocytes_3
10x_3281_AGTAATTGGGCATT-1 Patient4 Monocytes nonactive 3 0.07849851
Monocytes_3
10x_3281_AGTAGAGAAGAGAT-1 Patient4 T cells nonactive 9 0.06824234 T cells_9
10x_3281_AGTAGGCTGGTTAC-1 Patient4 Monocytes nonactive 3 0.06465982
Monocytes_3
10x_3281_AGTCCAGAGTTCTT-1 Patient4 Monocytes nonactive 3 0.02778835
Monocytes_3
10x_3281_AGTCCAGATCGCTC-1 Patient4 Monocytes nonactive 3 0.06982753
Monocytes_3
10x_3281_AGTCTTACAAAGCA-1 Patient4 Monocytes nonactive 3 0.01448862
Monocytes_3
10x_3281_AGTTTAGAGTCCTC-1 Patient4 Monocytes nonactive 3 0.03972481
Monocytes_3
10x_3281_ATAAACACAGCTCA-1 Patient4 Monocytes nonactive 3 0.04980661
Monocytes_3
10x_3281_ATAACCCTGCTTCC-1 Patient4 Monocytes nonactive 3 0.02834316
Monocytes_3
10x_3281_ATAACCCTGTGCAT-1 Patient4 T cells nonactive 9 0.04817386 T cells_9
10x_3281_ATAATCGACCCTAC-1 Patient4 Monocytes nonactive 3 0.04608142
Monocytes_3
10x_3281_ATAATGACACGACT-1 Patient4 Monocytes nonactive 2 0.06538901
Monocytes_2
10x_3281_ATACAATGACGCTA-1 Patient4 Monocytes nonactive 2 0.02160611
Monocytes_2
10x_3281_ATACAATGCTGGAT-1 Patient4 Monocytes nonactive 3 0.04579608

Monocytes_3
10x_3281_ATACACCTACCACA-1 Patient4 Monocytes nonactive 3 0.05235876
Monocytes_3
10x_3281_ATACCTTGACTGGT-1 Patient4 Monocytes nonactive 3 0.0328451
Monocytes_3
10x_3281_ATACGGACGACGAG-1 Patient4 Monocytes nonactive 2 0.03788599
Monocytes_2
10x_3281_ATACGTCTCATACG-1 Patient4 T cells nonactive 9 0.08957897 T cells_9
10x_3281_ATACTCTGCCTTAT-1 Patient4 Monocytes nonactive 3 0.05622662
Monocytes_3
10x_3281_ATAGATACTCCAGA-1 Patient4 Monocytes nonactive 3 0.00369349
Monocytes_3
10x_3281_ATAGCGTGTCTCCG-1 Patient4 T cells nonactive 9 0.11413354 T cells_9
10x_3281_ATAGGAGATGGTAC-1 Patient4 Monocytes nonactive 3 0.03197324
Monocytes_3
10x_3281_ATAGTCCTGACGTT-1 Patient4 Dendritic cells nonactive 14 0.11137531
Dendritic cells_14
10x_3281_ATAGTTGAGAGGGT-1 Patient4 Monocytes nonactive 3 0.0143618
Monocytes_3
10x_3281_ATATACGAGCCTTC-1 Patient4 Monocytes nonactive 3 0.03152939
Monocytes_3
10x_3281_ATCAAATGGTAGCT-1 Patient4 Monocytes nonactive 3 0.06727538
Monocytes_3
10x_3281_ATCAAATGTTCGCC-1 Patient4 Monocytes nonactive 3 0.05483165
Monocytes_3
10x_3281_ATCACGGAGTCTGA-1 Patient4 Monocytes nonactive 3 0.04390971
Monocytes_3
10x_3281_ATCACTACCTAGTG-1 Patient4 Monocytes nonactive 2 0.02369856
Monocytes_2
10x_3281_ATCACTTGAGCATC-1 Patient4 Monocytes nonactive 3 0.03756896
Monocytes_3
10x_3281_ATCAGGTGGGGTGA-1 Patient4 Monocytes nonactive 3 0.03504851
Monocytes_3

10x_3281_ATCATCTGATCTTC-1	Patient4 Monocytes	nonactive	3	0.02268404	Monocytes_3
10x_3281_ATCATCTGGCAAGG-1	Patient4 Monocytes	nonactive	3	0.11319828	Monocytes_3
10x_3281_ATCATGCTAACCTG-1	Patient4 Monocytes	nonactive	2	0.05243802	Monocytes_2
10x_3281_ATCCAGGACCATAG-1	Patient4 Monocytes	nonactive	3	0.04140511	Monocytes_3
10x_3281_ATCCAGGAGCTTCC-1	Patient4 Monocytes	nonactive	3	0.0543878	Monocytes_3
10x_3281_ATCCCGTGACCTAG-1	Patient4 Monocytes	nonactive	3	0.04717519	Monocytes_3
10x_3281_ATCCCGTGAGCCAT-1	Patient4 T cells	nonactive	9	0.05797033	T cells_9
10x_3281_ATCCGCACCCTACC-1	Patient4 Monocytes	nonactive	3	0.04137341	Monocytes_3
10x_3281_ATCCTAACTGTCAG-1	Patient4 T cells	nonactive	9	0.0788631	T cells_9
10x_3281_ATCGACGAGACGAG-1	Patient4 Monocytes	nonactive	3	0.05148691	Monocytes_3
10x_3281_ATCGACGATCGCTC-1	Patient4 Monocytes	nonactive	3	0.02365101	Monocytes_3
10x_3281_ATCGACGATGTTCT-1	Patient4 Monocytes	nonactive	3	0.06548412	Monocytes_3
10x_3281_ATCGCAGAACCTTT-1	Patient4 Monocytes	nonactive	3	0.02555323	Monocytes_3
10x_3281_ATCGCAGATGCTCC-1	Patient4 Monocytes	nonactive	2	0.08046414	Monocytes_2
10x_3281_ATCGCGCTTGCTAG-1	Patient4 Monocytes	nonactive	3	0.04073933	Monocytes_3
10x_3281_ATCGCGCTTTCTGT-1	Patient4 Monocytes	nonactive	3	0.01442521	Monocytes_3
10x_3281_ATCGGAACCTGAAC-1	Patient4 Monocytes	nonactive	3	0.05242217	Monocytes_3
10x_3281_ATCGGAACTCGCTC-1	Patient4 Monocytes	nonactive	3	0.05682899	

Monocytes_3
10x_3281_ATCGGTGATCACGA-1 Patient4 Monocytes nonactive 3 0.07637436 Monocytes_3
10x_3281_ATCTACACCGAGAG-1 Patient4 Monocytes nonactive 3 0.04860186 Monocytes_3
10x_3281_ATCTACACGCCCTT-1 Patient4 Monocytes nonactive 3 0.0560681 Monocytes_3
10x_3281_ATCTACTGAAGTGA-1 Patient4 Monocytes nonactive 3 0.07182487 Monocytes_3
10x_3281_ATCTGGGAGCAGTT-1 Patient4 Monocytes nonactive 3 0.04140511 Monocytes_3
10x_3281_ATCTTGACTTTCAC-1 Patient4 Monocytes nonactive 3 0.05327817 Monocytes_3
10x_3281_ATGACGTGTTCCGC-1 Patient4 Monocytes nonactive 3 0.04310126 Monocytes_3
10x_3281_ATGATAACGGTGAG-1 Patient4 Monocytes nonactive 3 0.07031894 Monocytes_3
10x_3281_ATGCCGCTACTCTT-1 Patient4 Monocytes nonactive 3 0.0353814 Monocytes_3
10x_3281_ATGCCGCTGGTATC-1 Patient4 Monocytes nonactive 3 0.04544734 Monocytes_3
10x_3281_ATGCTTTGCTCAGA-1 Patient4 Monocytes nonactive 3 0.02620316 Monocytes_3
10x_3281_ATGGGTACTTGTCT-1 Patient4 Monocytes nonactive 3 0.04923594 Monocytes_3
10x_3281_ATTAACGAGAGCAG-1 Patient4 Monocytes nonactive 2 0.05132839 Monocytes_2
10x_3281_ATTACCTGTGACAC-1 Patient4 Monocytes nonactive 3 0.03065754 Monocytes_3
10x_3281_ATTAGTGAAACCTG-1 Patient4 T cells nonactive 9 0.04901401 T cells_9
10x_3281_ATTCCATGCAATCG-1 Patient4 Monocytes nonactive 3 0.07100057 Monocytes_3
10x_3281_ATTCCATGGCGTTA-1 Patient4 Monocytes nonactive 3 0.04015281

Monocytes_3
10x_3281_ATTCGACTTCTCCG-1 Patient4 Monocytes nonactive 3 0.0514552
Monocytes_3
10x_3281_ATTCGGGAGGTAGG-1 Patient4 Monocytes nonactive 3 0.04332319
Monocytes_3
10x_3281_ATTGTAGAGGCGAA-1 Patient4 T cells nonactive 9 0.08788282 T cells_9
10x_3281_ATTTCTCTGAATGA-1 Patient4 Monocytes nonactive 3 0.06982753
Monocytes_3
10x_3281_ATTTCTCTTAGACC-1 Patient4 Monocytes nonactive 3 0.05570351
Monocytes_3
10x_3281_CAAACTCTGAGCTT-1 Patient4 Monocytes nonactive 3 0.03130746
Monocytes_3
10x_3281_CAAACTCTGTGTAC-1 Patient4 Monocytes nonactive 3 0.06901909
Monocytes_3
10x_3281_CAAAGCACAGGAGC-1 Patient4 Monocytes nonactive 2 0.05671803
Monocytes_2
10x_3281_CAAATTGACCCTCA-1 Patient4 Monocytes nonactive 3 0.04758734
Monocytes_3
10x_3281_CAAATTGACGTTGA-1 Patient4 Monocytes nonactive 3 0.01895885
Monocytes_3
10x_3281_CAACGAACTGCGTA-1 Patient4 T cells nonactive 9 0.06464397 T cells_9
10x_3281_CAACGATGTTAGGC-1 Patient4 Monocytes nonactive 3 0.04346586
Monocytes_3
10x_3281_CAAGACACGCGAGA-1 Patient4 Monocytes nonactive 3 0.01688225
Monocytes_3
10x_3281_CAAGACTGACGGTT-1 Patient4 Monocytes nonactive 3 0.03156109
Monocytes_3
10x_3281_CAAGCATGATCTCT-1 Patient4 Monocytes nonactive 3 0.07020798
Monocytes_3
10x_3281_CAAGCATGGAATAG-1 Patient4 T cells nonactive 9 0.03802866 T cells_9
10x_3281_CAAGCCCTTTGCAG-1 Patient4 Monocytes nonactive 3 0.06711686
Monocytes_3
10x_3281_CAAGCTGACGTCTC-1 Patient4 Monocytes nonactive 3 0.05568765

Monocytes_3
10x_3281_CAAGCTGATATGCG-1 Patient4 Monocytes nonactive 3 0.07383806
Monocytes_3
10x_3281_CAAGTCGATGGTGT-1 Patient4 Monocytes nonactive 3 0.02793101
Monocytes_3
10x_3281_CAATAAACCTTCCG-1 Patient4 Monocytes nonactive 3 0.04620823
Monocytes_3
10x_3281_CAATTCTGCACTAG-1 Patient4 Monocytes nonactive 3 0.0396614
Monocytes_3
10x_3281_CAATTCTGTAAGCC-1 Patient4 Monocytes nonactive 3 0.04933105
Monocytes_3
10x_3281_CACAGAACGCTATG-1 Patient4 Monocytes nonactive 3 0.04040644
Monocytes_3
10x_3281_CACAGATGGCCAAT-1 Patient4 Monocytes nonactive 3 0.04554245
Monocytes_3
10x_3281_CACAGTGATTTGTC-1 Patient4 Progenitors active 6 0.14033669
Progenitors_6
10x_3281_CACATACTACGACT-1 Patient4 Monocytes nonactive 3 0.04348171
Monocytes_3
10x_3281_CACCACTGAAGTAG-1 Patient4 Monocytes nonactive 3 0.02785175
Monocytes_3
10x_3281_CACCACTGCATGCA-1 Patient4 Monocytes nonactive 2 0.04704838
Monocytes_2
10x_3281_CACCGTACCTTCGC-1 Patient4 Monocytes nonactive 3 0.0374897
Monocytes_3
10x_3281_CACTCCGACTACGA-1 Patient4 Monocytes nonactive 3 0.05021876
Monocytes_3
10x_3281_CACTTAACAAAACG-1 Patient4 Monocytes nonactive 3 0.03839325
Monocytes_3
10x_3281_CACTTTGACCGCTT-1 Patient4 Monocytes nonactive 3 0.03573014
Monocytes_3
10x_3281_CACTTTGACTAGAC-1 Patient4 Monocytes nonactive 3 0.01543973
Monocytes_3

10x_3281_CAGACATGCGCATA-1	Patient4	Monocytes	nonactive	3	0.02937353	Monocytes_3
10x_3281_CAGACATGTGCGTA-1	Patient4	Monocytes	nonactive	3	0.02810538	Monocytes_3
10x_3281_CAGACCCTGTGTCA-1	Patient4	Monocytes	nonactive	3	0.04487667	Monocytes_3
10x_3281_CAGCCTACGGAACG-1	Patient4	Monocytes	nonactive	3	0.05844588	Monocytes_3
10x_3281_CAGCCTACGTAAGA-1	Patient4	Monocytes	nonactive	3	0.03420836	Monocytes_3
10x_3281_CAGCCTTGGTTGTG-1	Patient4	Monocytes	nonactive	2	0.03571429	Monocytes_2
10x_3281_CAGCGGACTCGCAA-1	Patient4	Monocytes	nonactive	2	0.080908	Monocytes_2
10x_3281_CAGGTATGGCCTTC-1	Patient4	Monocytes	nonactive	3	0.00605542	Monocytes_3
10x_3281_CAGGTTGAGAGGTG-1	Patient4	Monocytes	nonactive	3	0.03313043	Monocytes_3
10x_3281_CAGTGATGCAAGCT-1	Patient4	Monocytes	nonactive	3	0.04584364	Monocytes_3
10x_3281_CAGTGTGAGGGTGA-1	Patient4	Monocytes	nonactive	2	0.04409993	Monocytes_2
10x_3281_CAGTGTGATATCTC-1	Patient4	Monocytes	nonactive	3	0.07228457	Monocytes_3
10x_3281_CATAACCTGGTTAC-1	Patient4	Monocytes	nonactive	3	0.03891637	Monocytes_3
10x_3281_CATACTTGGCCAAT-1	Patient4	Monocytes	nonactive	3	0.02450701	Monocytes_3
10x_3281_CATATAGAGACAAA-1	Patient4	Monocytes	nonactive	3	0.03988333	Monocytes_3
10x_3281_CATCAGGAGGTCTA-1	Patient4	Monocytes	nonactive	3	0.05044068	Monocytes_3
10x_3281_CATCGCTGCCTACC-1	Patient4	Monocytes	nonactive	3	0.05773255	

Monocytes_3
10x_3281_CATCGGCTTTAGGC-1 Patient4 Monocytes nonactive 3 0.06047492
Monocytes_3
10x_3281_CATGGATGTAACCG-1 Patient4 Monocytes nonactive 3 0.05269165
Monocytes_3
10x_3281_CATGGCCTCTAGTG-1 Patient4 Monocytes nonactive 3 0.03354258
Monocytes_3
10x_3281_CATGTACTGTTTGG-1 Patient4 T cells nonactive 9 0.08028977 T cells_9
10x_3281_CATGTTTGCCAACA-1 Patient4 Progenitors nonactive 6 0.07802295
Progenitors_6
10x_3281_CATTAGCTACACTG-1 Patient4 Monocytes nonactive 3 0.0586361
Monocytes_3
10x_3281_CATTAGCTGTTAGC-1 Patient4 T cells nonactive 9 0.08374548 T cells_9
10x_3281_CATTCCCTGAGCTT-1 Patient4 T cells nonactive 9 0.0960941 T cells_9
10x_3281_CATTGACTACACAC-1 Patient4 Monocytes nonactive 2 0.03365354
Monocytes_2
10x_3281_CATTGACTGAGATA-1 Patient4 Monocytes nonactive 3 0.05326232
Monocytes_3
10x_3281_CATTGGGATCGTTT-1 Patient4 Monocytes nonactive 3 0.03273413
Monocytes_3
10x_3281_CATTGTACAACCTG-1 Patient4 Monocytes nonactive 2 0.02831146
Monocytes_2
10x_3281_CATTGTTGCGGAGA-1 Patient4 Monocytes nonactive 3 0.06090292
Monocytes_3
10x_3281_CATTTCGACTACCC-1 Patient4 Monocytes nonactive 3 0.07314057
Monocytes_3
10x_3281_CATTTCGAGCCTTC-1 Patient4 Monocytes nonactive 3 0.0510272
Monocytes_3
10x_3281_CATTTGACTCAGAC-1 Patient4 Monocytes nonactive 3 0.05151861
Monocytes_3
10x_3281_CATTTGACTCGTGA-1 Patient4 Monocytes nonactive 16 0.02805783
Monocytes_16
10x_3281_CCAAGAACTCTCGC-1 Patient4 Monocytes nonactive 3 0.0345571

Monocytes_3
10x_3281_CCAAGATGCTTCTA-1 Patient4 Monocytes nonactive 3 0.03664955
Monocytes_3
10x_3281_CCAATGGACTGACA-1 Patient4 Monocytes nonactive 3 0.08100311
Monocytes_3
10x_3281_CCAATTTGACCTAG-1 Patient4 Monocytes nonactive 3 0.04349756
Monocytes_3
10x_3281_CCACTGTGCTTACT-1 Patient4 Monocytes nonactive 2 0.06117241
Monocytes_2
10x_3281_CCAGAAACATGTCG-1 Patient4 T cells nonactive 9 0.06989094 T cells_9
10x_3281_CCAGATGAGGCGAA-1 Patient4 Monocytes nonactive 3 0.0619333
Monocytes_3
10x_3281_CCAGCACTTAACGC-1 Patient4 Monocytes nonactive 3 0.06370871
Monocytes_3
10x_3281_CCAGCTACCCCTAC-1 Patient4 Monocytes nonactive 2 0.03821888
Monocytes_2
10x_3281_CCAGTCACAAACAG-1 Patient4 Monocytes nonactive 3 0.03869444
Monocytes_3
10x_3281_CCATATACCTATTC-1 Patient4 Monocytes nonactive 3 0.03951874
Monocytes_3
10x_3281_CCATCCGAGAGAGC-1 Patient4 Monocytes nonactive 2 0.05105891
Monocytes_2
10x_3281_CCCGAACTTCGATG-1 Patient4 Monocytes nonactive 3 0.00502505
Monocytes_3
10x_3281_CCCTAGTGAGAGGC-1 Patient4 Monocytes nonactive 3 0.03452539
Monocytes_3
10x_3281_CCCTTACTTGTAGC-1 Patient4 Monocytes nonactive 2 0.04673134
Monocytes_2
10x_3281_CCGAAAACGGACTT-1 Patient4 Monocytes nonactive 3 0.01152432
Monocytes_3
10x_3281_CCGACTACCATGGT-1 Patient4 Monocytes nonactive 2 0.03390717
Monocytes_2
10x_3281_CCGATAGAATGCCA-1 Patient4 Monocytes nonactive 3 0.02805783

Monocytes_3
10x_3281_CCGATAGAATTCGG-1 Patient4 Monocytes nonactive 3 0.01528121
Monocytes_3
10x_3281_CCGGAGACTAGCGT-1 Patient4 Monocytes nonactive 3 0.01062076
Monocytes_3
10x_3281_CCGGTACTCTTCCG-1 Patient4 Monocytes nonactive 2 0.04788853
Monocytes_2
10x_3281_CCGTAAGACAGAGG-1 Patient4 Monocytes nonactive 3 0.05251728
Monocytes_3
10x_3281_CCGTACACCGAATC-1 Patient4 Monocytes nonactive 3 0.05246972
Monocytes_3
10x_3281_CCGTACACGCGTTA-1 Patient4 Monocytes nonactive 3 0.03573014
Monocytes_3
10x_3281_CCTAAGGAGCTCCT-1 Patient4 Monocytes nonactive 3 0.06557923
Monocytes_3
10x_3281_CCTATAACATGTGC-1 Patient4 Monocytes nonactive 3 0.06557923
Monocytes_3
10x_3281_CCTATAACTGTAGC-1 Patient4 Monocytes nonactive 3 0.04857016
Monocytes_3
10x_3281_CCTCGAACCTCAAG-1 Patient4 Monocytes nonactive 3 0.01543973
Monocytes_3
10x_3281_CCTGACTGGTTAGC-1 Patient4 Monocytes nonactive 2 0.0480946
Monocytes_2
10x_3281_CCTTAATGCTGTCC-1 Patient4 Monocytes nonactive 3 0.05296113
Monocytes_3
10x_3281_CCTTTAGAAACCAC-1 Patient4 Monocytes nonactive 3 0.0560681
Monocytes_3
10x_3281_CGAACATGTTCGCC-1 Patient4 Monocytes nonactive 3 0.04023207
Monocytes_3
10x_3281_CGACTCTGTTACCT-1 Patient4 Monocytes nonactive 3 0.05952381
Monocytes_3
10x_3281_CGAGAACTTGTCTT-1 Patient4 Monocytes nonactive 3 0.05589373
Monocytes_3

10x_3281_CGAGAACTTTCGTT-1 Patient4 Monocytes nonactive 3 0.0476349 Monocytes_3
10x_3281_CGAGGCACCCCTTG-1 Patient4 Monocytes nonactive 3 0.06160041 Monocytes_3
10x_3281_CGAGGCTGCTGGAT-1 Patient4 Monocytes nonactive 3 0.04146852 Monocytes_3
10x_3281_CGAGGGCTCGTTAG-1 Patient4 Monocytes nonactive 3 0.03422421 Monocytes_3
10x_3281_CGCAACCTATCGAC-1 Patient4 Monocytes nonactive 3 0.04175385 Monocytes_3
10x_3281_CGCACGGAACTGGT-1 Patient4 Monocytes nonactive 3 0.02786761 Monocytes_3
10x_3281_CGCACTACCCCGTT-1 Patient4 Monocytes nonactive 2 0.05999937 Monocytes_2
10x_3281_CGCAGGTGTTACCT-1 Patient4 Monocytes nonactive 3 0.04985416 Monocytes_3
10x_3281_CGCGAGACACGACT-1 Patient4 Monocytes nonactive 3 0.02217678 Monocytes_3
10x_3281_CGCTACACGGAAAT-1 Patient4 Monocytes nonactive 3 0.05537062 Monocytes_3
10x_3281_CGGACCGAGGACAG-1 Patient4 Monocytes nonactive 3 0.06982753 Monocytes_3
10x_3281_CGGAGGCTCTAGCA-1 Patient4 Monocytes nonactive 3 0.05741551 Monocytes_3
10x_3281_CGGATAACCACTGA-1 Patient4 Monocytes nonactive 3 0.03251221 Monocytes_3
10x_3281_CGGATATGACTCTT-1 Patient4 Monocytes nonactive 3 0.02957961 Monocytes_3
10x_3281_CGGATATGGTTCGA-1 Patient4 Monocytes nonactive 3 0.06042737 Monocytes_3
10x_3281_CGGATATGTAGACC-1 Patient4 Monocytes nonactive 3 0.01846744 Monocytes_3
10x_3281_CGGCATCTGTCAAC-1 Patient4 Monocytes nonactive 3 0.07559762

Monocytes_3
10x_3281_CGGCCAGATCACGA-1 Patient4 Monocytes nonactive 2 0.06908249
Monocytes_2
10x_3281_CGTACCACAGCTCA-1 Patient4 Monocytes nonactive 2 0.03508021
Monocytes_2
10x_3281_CGTACCTGGGCGAA-1 Patient4 Monocytes nonactive 3 0.07551836
Monocytes_3
10x_3281_CGTGAATGTGAACC-1 Patient4 Monocytes nonactive 2 0.07256991
Monocytes_2
10x_3281_CGTGATGAAAGCAA-1 Patient4 Monocytes nonactive 2 0.03487414
Monocytes_2
10x_3281_CGTGATGATGCAGT-1 Patient4 Monocytes nonactive 3 0.0703665
Monocytes_3
10x_3281_CGTGCACTGGTCTA-1 Patient4 Monocytes nonactive 3 0.07510621
Monocytes_3
10x_3281_CGTTAGGAAACCAC-1 Patient4 Monocytes nonactive 3 0.02821635
Monocytes_3
10x_3281_CGTTATACTTCAGG-1 Patient4 Monocytes nonactive 3 0.06042737
Monocytes_3
10x_3281_CGTTTAACCTGGTA-1 Patient4 T cells active 9 0.13510557 T cells_9
10x_3281_CTAACTACACTTTC-1 Patient4 Monocytes nonactive 2 0.04287934
Monocytes_2
10x_3281_CTAACTACTTTCGT-1 Patient4 Monocytes nonactive 3 0.09209942
Monocytes_3
10x_3281_CTAAGGACCCTGTC-1 Patient4 T cells nonactive 9 0.06145774 T cells_9
10x_3281_CTAAGGTGATGCTG-1 Patient4 Monocytes nonactive 3 0.01407647
Monocytes_3
10x_3281_CTAAGGTGTACAGC-1 Patient4 Monocytes nonactive 2 0.0455266
Monocytes_2
10x_3281_CTACGCACAAACAG-1 Patient4 Monocytes nonactive 3 0.06052248
Monocytes_3
10x_3281_CTACTATGACGGTT-1 Patient4 Monocytes nonactive 3 0.05733625
Monocytes_3

10x_3281_CTAGAGACAAAGCA-1 Patient4 Monocytes nonactive 3 0.04072348 Monocytes_3
10x_3281_CTAGGATGCGACTA-1 Patient4 Monocytes nonactive 3 0.03634836 Monocytes_3
10x_3281_CTAGGCCTGGTTAC-1 Patient4 Monocytes nonactive 3 0.08956312 Monocytes_3
10x_3281_CTAGGTGAGAAACA-1 Patient4 Monocytes nonactive 3 0.0459546 Monocytes_3
10x_3281_CTAGTTACTGTGGT-1 Patient4 Monocytes nonactive 3 0.05280261 Monocytes_3
10x_3281_CTAGTTTGCCGTAA-1 Patient4 Monocytes nonactive 3 0.0206233 Monocytes_3
10x_3281_CTATAAGAAGCCTA-1 Patient4 Monocytes nonactive 3 0.06355019 Monocytes_3
10x_3281_CTATTGACGATGAA-1 Patient4 Monocytes nonactive 2 0.06342337 Monocytes_2
10x_3281_CTATTGTGTCCAGA-1 Patient4 Monocytes nonactive 3 0.02674212 Monocytes_3
10x_3281_CTCAGCACCTCGCT-1 Patient4 Monocytes nonactive 3 0.03053072 Monocytes_3
10x_3281_CTCAGCTGCCACAA-1 Patient4 Monocytes nonactive 3 0.02128907 Monocytes_3
10x_3281_CTCAGCTGTCTTTG-1 Patient4 Monocytes nonactive 3 0.02536301 Monocytes_3
10x_3281_CTCAGGCTCGTGTA-1 Patient4 Monocytes nonactive 3 0.0737588 Monocytes_3
10x_3281_CTCAGGCTTCCTAT-1 Patient4 Monocytes nonactive 3 0.04826897 Monocytes_3
10x_3281_CTCATTGATGCTTT-1 Patient4 T cells nonactive 9 0.0881523 T cells_9
10x_3281_CTCCACGACCGTAA-1 Patient4 Monocytes nonactive 3 0.0265519 Monocytes_3
10x_3281_CTCCATCTTGTCAG-1 Patient4 Monocytes nonactive 3 0.06167967 Monocytes_3

10x_3281_CTGAACGATGTGAC-1 Patient4 Monocytes nonactive 3 0.04479741 Monocytes_3
10x_3281_CTGAAGTGCTACTT-1 Patient4 T cells nonactive 9 0.06315389 T cells_9
10x_3281_CTGAAGTGTATGGC-1 Patient4 Monocytes nonactive 3 0.02715427 Monocytes_3
10x_3281_CTGACAGATGAAGA-1 Patient4 Monocytes nonactive 3 0.05131254 Monocytes_3
10x_3281_CTGAGAACTTCTCA-1 Patient4 Monocytes nonactive 3 0.02669457 Monocytes_3
10x_3281_CTGATTTGAGGAGC-1 Patient4 Monocytes nonactive 3 0.02580686 Monocytes_3
10x_3281_CTGATTTGCTTGTT-1 Patient4 T cells nonactive 9 0.1016264 T cells_9
10x_3281_CTGCGACTTGAACC-1 Patient4 Monocytes nonactive 3 0.01891129 Monocytes_3
10x_3281_CTGTAACTTGGAGG-1 Patient4 Monocytes nonactive 3 0.05725699 Monocytes_3
10x_3281_CTGTGAGATTGGTG-1 Patient4 T cells nonactive 9 0.08187496 T cells_9
10x_3281_CTTAGACTGTTCGA-1 Patient4 Monocytes nonactive 3 0.03070509 Monocytes_3
10x_3281_CTTCATGACATTCT-1 Patient4 Monocytes nonactive 3 0.0370617 Monocytes_3
10x_3281_CTTCTAGATCTTCA-1 Patient4 Monocytes nonactive 3 0.04839579 Monocytes_3
10x_3281_CTTGAACTTTGGCA-1 Patient4 Monocytes nonactive 3 0.03885296 Monocytes_3
10x_3281_CTTGAGGAGTACCA-1 Patient4 Monocytes nonactive 3 0.02536301 Monocytes_3
10x_3281_CTTGATTGGAAAGT-1 Patient4 Monocytes nonactive 3 0.02480819 Monocytes_3
10x_3281_CTTTACGAAGTCGT-1 Patient4 Monocytes nonactive 3 0.0485226 Monocytes_3
10x_3281_CTTTACGACTCATT-1 Patient4 Monocytes nonactive 3 0.02934183 Monocytes_3

10x_3281_CTTTAGTGTGACTG-1	Patient4 Monocytes	nonactive	3	0.07187242
Monocytes_3
10x_3281_CTTTCAGAGACAGG-1	Patient4 Monocytes	nonactive	2	0.04337074
Monocytes_2
10x_3281_GAACCAACATTCGG-1	Patient4 Monocytes	nonactive	3	0.03571429
Monocytes_3
10x_3281_GAACCTGACGAACT-1	Patient4 Monocytes	nonactive	3	0.05250143
Monocytes_3
10x_3281_GAACCTGAGTATGC-1	Patient4 Monocytes	nonactive	2	0.03850422
Monocytes_2
10x_3281_GAACGTTGTGAGCT-1	Patient4 Monocytes	nonactive	3	0.05166128
Monocytes_3
10x_3281_GAAGCGGACTCTTA-1	Patient4 B cells	nonactive	7	0.059825	B cells_7
10x_3281_GAAGCTTGCGCCTT-1	Patient4 Monocytes	nonactive	3	0.02996005
Monocytes_3
10x_3281_GAAGGTCTTTTCTG-1	Patient4 Monocytes	nonactive	3	0.04454378
Monocytes_3
10x_3281_GAAGTAGAGGAACG-1	Patient4 Monocytes	nonactive	3	0.02957961
Monocytes_3
10x_3281_GAATGCTGTGAACC-1	Patient4 Monocytes	nonactive	3	0.02143174
Monocytes_3
10x_3281_GAATTAACAGGCGA-1	Patient4 Monocytes	nonactive	3	0.04479741
Monocytes_3
10x_3281_GACAACTGAGTCTG-1	Patient4 Monocytes	nonactive	3	0.06220278
Monocytes_3
10x_3281_GACAACTGTTGCTT-1	Patient4 Monocytes	nonactive	3	0.05075772
Monocytes_3
10x_3281_GACAGGGACTTACT-1	Patient4 Monocytes	nonactive	3	0.03322554
Monocytes_3
10x_3281_GACATTCTAGAGAT-1	Patient4 Monocytes	nonactive	3	0.05373787
Monocytes_3
10x_3281_GACGATTGAGGGTG-1	Patient4 Monocytes	nonactive	3	0.03571429
Monocytes_3

10x_3281_GACGATTGGGTCTA-1 Patient4 Monocytes nonactive 3 0.02548982 Monocytes_3
10x_3281_GACGCCGAAGGTCT-1 Patient4 Monocytes nonactive 3 0.02713842 Monocytes_3
10x_3281_GACTACGAGCTTAG-1 Patient4 T cells nonactive 9 0.04013696 T cells_9
10x_3281_GACTGAACGTCACA-1 Patient4 Monocytes nonactive 3 0.02856509 Monocytes_3
10x_3281_GACTGATGGGTCAT-1 Patient4 Monocytes nonactive 2 0.03576184 Monocytes_2
10x_3281_GACTGTGACGGTAT-1 Patient4 Monocytes nonactive 3 0.0506309 Monocytes_3
10x_3281_GACTTTACATACCG-1 Patient4 Monocytes nonactive 3 0.04332319 Monocytes_3
10x_3281_GAGATCACTCGCAA-1 Patient4 Monocytes nonactive 2 0.03289265 Monocytes_2
10x_3281_GAGATGCTCCAACA-1 Patient4 Monocytes nonactive 3 0.03804451 Monocytes_3
10x_3281_GAGATGCTCCCTTG-1 Patient4 Monocytes nonactive 3 0.0308002 Monocytes_3
10x_3281_GAGCAACTGTTGTG-1 Patient4 Progenitors nonactive 6 0.11123264 Progenitors_6
10x_3281_GAGCGAGAAGAGTA-1 Patient4 T cells nonactive 9 0.07566102 T cells_9
10x_3281_GAGCTCCTCTCAAG-1 Patient4 Monocytes nonactive 3 0.04145267 Monocytes_3
10x_3281_GAGGATCTGATAAG-1 Patient4 Monocytes nonactive 3 0.04893475 Monocytes_3
10x_3281_GAGGCAGAATCGAC-1 Patient4 Monocytes nonactive 3 0.03051487 Monocytes_3
10x_3281_GAGGCAGACTAGAC-1 Patient4 Monocytes nonactive 3 0.05914336 Monocytes_3
10x_3281_GAGGGTGACGACTA-1 Patient4 Monocytes nonactive 3 0.02498256 Monocytes_3
10x_3281_GAGGTGGAACGACT-1 Patient4 Progenitors nonactive 6 0.10765012

Progenitors_6
10x_3281_GAGGTGGACCGCTT-1 Patient4 Monocytes nonactive 3 0.07570858 Monocytes_3
10x_3281_GAGGTTTGCTCAAG-1 Patient4 Monocytes nonactive 3 0.061109 Monocytes_3
10x_3281_GAGTACACCAGAAA-1 Patient4 Monocytes nonactive 3 0.0624247 Monocytes_3
10x_3281_GAGTACACTGTCCC-1 Patient4 Monocytes nonactive 3 0.04435356 Monocytes_3
10x_3281_GAGTCTGAGGCAAG-1 Patient4 Monocytes nonactive 3 0.06423182 Monocytes_3
10x_3281_GAGTGTTGAGGGTG-1 Patient4 Monocytes nonactive 3 0.0358094 Monocytes_3
10x_3281_GATAAGGAGTGTCA-1 Patient4 Monocytes nonactive 3 0.06245641 Monocytes_3
10x_3281_GATACTCTGTATGC-1 Patient4 Monocytes nonactive 3 0.06102974 Monocytes_3
10x_3281_GATACTCTTCGCTC-1 Patient4 Monocytes nonactive 3 0.03620569 Monocytes_3
10x_3281_GATATATGGAGCTT-1 Patient4 Monocytes nonactive 3 0.02632997 Monocytes_3
10x_3281_GATATATGTCAGGT-1 Patient4 Monocytes nonactive 3 0.07745229 Monocytes_3
10x_3281_GATCCCTGTACTTC-1 Patient4 Monocytes nonactive 3 0.039598 Monocytes_3
10x_3281_GATCCCTGTGCTAG-1 Patient4 T cells nonactive 9 0.0776108 T cells_9
10x_3281_GATCGAACTGCGTA-1 Patient4 Monocytes nonactive 3 0.03661784 Monocytes_3
10x_3281_GATCTACTAGTCTG-1 Patient4 Monocytes nonactive 3 0.03997844 Monocytes_3
10x_3281_GATTACCTTGAACC-1 Patient4 Monocytes nonactive 3 0.05110646 Monocytes_3
10x_3281_GATTCTACCAGATC-1 Patient4 Monocytes nonactive 3 0.06982753

Monocytes_3
10x_3281_GATTCTTGCAGGAG-1 Patient4 Monocytes nonactive 3 0.0425623 Monocytes_3
10x_3281_GATTCTTGCTGTGA-1 Patient4 T cells nonactive 9 0.05571936 T cells_9
10x_3281_GATTCTTGTCTAGG-1 Patient4 Monocytes nonactive 3 0.05453047 Monocytes_3
10x_3281_GATTGGACAGAACA-1 Patient4 Monocytes nonactive 3 0.07716695 Monocytes_3
10x_3281_GATTGGTGTCGATG-1 Patient4 Monocytes nonactive 3 0.05590958 Monocytes_3
10x_3281_GATTTGCTCGCTAA-1 Patient4 Monocytes nonactive 3 0.08282607 Monocytes_3
10x_3281_GCAACTGACGACTA-1 Patient4 Monocytes nonactive 3 0.03891637 Monocytes_3
10x_3281_GCAACTGATCTCTA-1 Patient4 Monocytes nonactive 3 0.03642762 Monocytes_3
10x_3281_GCAAGACTCGATAC-1 Patient4 Monocytes nonactive 3 0.03866274 Monocytes_3
10x_3281_GCAATCGAAAAACG-1 Patient4 Monocytes nonactive 2 0.04744468 Monocytes_2
10x_3281_GCAATCGACGGGAA-1 Patient4 T cells nonactive 9 0.08005199 T cells_9
10x_3281_GCACCTTGGTACGT-1 Patient4 T cells nonactive 9 0.09328831 T cells_9
10x_3281_GCACGGACATGCCA-1 Patient4 Monocytes nonactive 2 0.05037727 Monocytes_2
10x_3281_GCACTAGACAGGAG-1 Patient4 Monocytes nonactive 3 0.04752394 Monocytes_3
10x_3281_GCACTAGATGTCTT-1 Patient4 Progenitors nonactive 6 0.10151544 Progenitors_6
10x_3281_GCACTGCTTGGAAA-1 Patient4 Monocytes nonactive 3 0.03999429 Monocytes_3
10x_3281_GCAGGCACCACTGA-1 Patient4 Monocytes nonactive 3 0.04980661 Monocytes_3
10x_3281_GCAGGCACCTGGTA-1 Patient4 Monocytes nonactive 3 0.03561917

Monocytes_3
10x_3281_GCAGTTGAACCACA-1 Patient4 T cells nonactive 9 0.09132268 T cells_9
10x_3281_GCATCAGACAACCA-1 Patient4 Monocytes nonactive 3 0.03064168 Monocytes_3
10x_3281_GCATCAGACCACAA-1 Patient4 T cells nonactive 9 0.04508275 T cells_9
10x_3281_GCATGTGACATCAG-1 Patient4 Monocytes nonactive 3 0.06716442 Monocytes_3
10x_3281_GCATTGGACGGAGA-1 Patient4 Monocytes nonactive 3 0.03978822 Monocytes_3
10x_3281_GCCAAATGTCAGGT-1 Patient4 Monocytes nonactive 3 0.03509606 Monocytes_3
10x_3281_GCCACTACGGAGTG-1 Patient4 Monocytes nonactive 3 0.02880287 Monocytes_3
10x_3281_GCCATGCTCTTTAC-1 Patient4 Monocytes nonactive 3 0.05090039 Monocytes_3
10x_3281_GCCCAACTGTCGTA-1 Patient4 Monocytes nonactive 3 0.05755818 Monocytes_3
10x_3281_GCCCAGGAAAGAGT-1 Patient4 Monocytes nonactive 3 0.04444867 Monocytes_3
10x_3281_GCCTACACGCGATT-1 Patient4 B cells nonactive 7 0.07532813 B cells_7
10x_3281_GCCTAGCTCTTCTA-1 Patient4 Monocytes nonactive 2 0.04010526 Monocytes_2
10x_3281_GCCTCATGCTGGTA-1 Patient4 Monocytes nonactive 2 0.04096126 Monocytes_2
10x_3281_GCGAAGGAGGTTTG-1 Patient4 Monocytes nonactive 3 0.04746053 Monocytes_3
10x_3281_GCGACTCTAGCCAT-1 Patient4 Monocytes nonactive 3 0.0328451 Monocytes_3
10x_3281_GCGATATGTACGCA-1 Patient4 Monocytes nonactive 3 0.05546573 Monocytes_3
10x_3281_GCGCACGAGCTTCC-1 Patient4 Monocytes nonactive 2 0.05922262 Monocytes_2
10x_3281_GCGCACGATTATCC-1 Patient4 Monocytes nonactive 3 0.03409739

Monocytes_3
10x_3281_GCGGAGCTGGGATG-1 Patient4 Monocytes nonactive 3 0.02690064
Monocytes_3
10x_3281_GCGTATGACTCCCA-1 Patient4 Monocytes nonactive 3 0.03931266
Monocytes_3
10x_3281_GCTATACTACCCTC-1 Patient4 Monocytes nonactive 3 0.0518198
Monocytes_3
10x_3281_GCTCAAGACATCAG-1 Patient4 Monocytes nonactive 3 0.02189145
Monocytes_3
10x_3281_GCTCACTGGACACT-1 Patient4 Monocytes nonactive 3 0.0362691
Monocytes_3
10x_3281_GCTCGACTCTATGG-1 Patient4 Monocytes nonactive 3 0.05456217
Monocytes_3
10x_3281_GCTGATGAATCTCT-1 Patient4 Monocytes nonactive 2 0.03829814
Monocytes_2
10x_3281_GCTTAACTTCGTTT-1 Patient4 Monocytes nonactive 2 0.04918838
Monocytes_2
10x_3281_GGAAGGACTACAGC-1 Patient4 Monocytes nonactive 3 0.06234544
Monocytes_3
10x_3281_GGAATCTGTCTCCG-1 Patient4 Monocytes nonactive 3 0.0661816
Monocytes_3
10x_3281_GGACAACTATCGGT-1 Patient4 Monocytes nonactive 3 0.05078942
Monocytes_3
10x_3281_GGACAGGATCGTTT-1 Patient4 Monocytes nonactive 3 0.02723353
Monocytes_3
10x_3281_GGACCCGATCGTTT-1 Patient4 Monocytes nonactive 3 0.05533891
Monocytes_3
10x_3281_GGACGAGACTATTC-1 Patient4 Monocytes nonactive 3 0.0198307
Monocytes_3
10x_3281_GGACGCACGTTTGG-1 Patient4 Monocytes nonactive 3 0.06340752
Monocytes_3
10x_3281_GGACGCACTCACGA-1 Patient4 Monocytes nonactive 3 0.01662862
Monocytes_3

10x_3281_GGACTATGTGTCCC-1 Patient4 Monocytes nonactive 3 0.03576184 Monocytes_3
10x_3281_GGAGAGACTAAGGA-1 Patient4 Monocytes nonactive 3 0.05376958 Monocytes_3
10x_3281_GGAGCCACAGCATC-1 Patient4 Monocytes nonactive 3 0.01757973 Monocytes_3
10x_3281_GGAGGATGAACGAA-1 Patient4 Monocytes nonactive 3 0.0695739 Monocytes_3
10x_3281_GGATACTGGTCACA-1 Patient4 Monocytes nonactive 3 0.03514362 Monocytes_3
10x_3281_GGATTGTGCATTTC-1 Patient4 Monocytes nonactive 3 0.04043815 Monocytes_3
10x_3281_GGATTGTGTGTGCA-1 Patient4 Monocytes nonactive 3 0.01921248 Monocytes_3
10x_3281_GGATTTCTTCCTGC-1 Patient4 Monocytes nonactive 2 0.04364023 Monocytes_2
10x_3281_GGATTTCTTCGCTC-1 Patient4 T cells nonactive 9 0.06145774 T cells_9
10x_3281_GGCACTCTCCTGAA-1 Patient4 Monocytes nonactive 2 0.01176209 Monocytes_2
10x_3281_GGCACTCTCTTCCG-1 Patient4 Monocytes nonactive 3 0.0185467 Monocytes_3
10x_3281_GGCACTCTGGATCT-1 Patient4 Monocytes nonactive 3 0.07681821 Monocytes_3
10x_3281_GGCACTCTTTTGCT-1 Patient4 Monocytes nonactive 3 0.0202587 Monocytes_3
10x_3281_GGCCACGACTACGA-1 Patient4 Monocytes nonactive 3 0.02308034 Monocytes_3
10x_3281_GGCCAGACCCCGTT-1 Patient4 Monocytes nonactive 3 0.06076026 Monocytes_3
10x_3281_GGCCCAGAAGTCGT-1 Patient4 Monocytes nonactive 3 0.04474986 Monocytes_3
10x_3281_GGCCCAGAGGTTAC-1 Patient4 Monocytes nonactive 3 0.03089531 Monocytes_3

10x_3281_GGCCGAACCGACAT-1 Patient4 Dendritic cells nonactive 14 0.07689747 Dendritic cells_14
10x_3281_GGCTAAACTTACCT-1 Patient4 Monocytes nonactive 2 0.07686577 Monocytes_2
10x_3281_GGCTAATGCCTTCG-1 Patient4 Monocytes nonactive 3 0.06806797 Monocytes_3
10x_3281_GGGAAGTGGACAGG-1 Patient4 Monocytes nonactive 3 0.0560998 Monocytes_3
10x_3281_GGGAAGTGTGGAGG-1 Patient4 Monocytes nonactive 2 0.01206328 Monocytes_2
10x_3281_GGGACCACTCGCCT-1 Patient4 Monocytes nonactive 2 0.07726206 Monocytes_2
10x_3281_GGGACCTGTTCTGT-1 Patient4 Monocytes nonactive 3 0.07084205 Monocytes_3
10x_3281_GGGATGGATAACGC-1 Patient4 Monocytes nonactive 3 0.06686323 Monocytes_3
10x_3281_GGGCAAGACTTGAG-1 Patient4 Monocytes nonactive 2 0.03235369 Monocytes_2
10x_3281_GGGCCAACTCTCAT-1 Patient4 Monocytes nonactive 3 0.01965633 Monocytes_3
10x_3281_GGGCCAACTGGATC-1 Patient4 T cells nonactive 9 0.07531228 T cells_9
10x_3281_GGGCCATGCATTCT-1 Patient4 Monocytes nonactive 2 0.05779595 Monocytes_2
10x_3281_GGGTTATGGGAAAT-1 Patient4 Monocytes nonactive 3 0.04536808 Monocytes_3
10x_3281_GGTAGTACCTTGCC-1 Patient4 Monocytes nonactive 3 0.01946611 Monocytes_3
10x_3281_GGTCAAACCACTAG-1 Patient4 Monocytes nonactive 3 0.02563249 Monocytes_3
10x_3281_GGTCTAGAGCGAGA-1 Patient4 Monocytes nonactive 3 0.04725445 Monocytes_3
10x_3281_GGTGGAGATCTGGA-1 Patient4 Monocytes nonactive 2 0.0159787 Monocytes_2

10x_3281_GGTTTACTCTGATG-1 Patient4 T cells nonactive 9 0.0607127 T cells_9
10x_3281_GTAACGTGGGTAGG-1 Patient4 Monocytes nonactive 3 0.03988333 Monocytes_3
10x_3281_GTAACGTGGTTACG-1 Patient4 Monocytes nonactive 3 0.01919663 Monocytes_3
10x_3281_GTAAGCACGGAGTG-1 Patient4 Monocytes nonactive 3 0.0492835 Monocytes_3
10x_3281_GTAAGCTGTCAGGT-1 Patient4 Monocytes nonactive 3 0.03618984 Monocytes_3
10x_3281_GTAATAACGGAAAT-1 Patient4 Monocytes nonactive 2 0.02548982 Monocytes_2
10x_3281_GTAATAACTTCAGG-1 Patient4 T cells nonactive 9 0.1163845 T cells_9
10x_3281_GTACGAACAGAGGC-1 Patient4 Monocytes nonactive 3 0.03942363 Monocytes_3
10x_3281_GTACGAACCCTACC-1 Patient4 T cells nonactive 9 0.04027963 T cells_9
10x_3281_GTACGAACTTGGCA-1 Patient4 Monocytes nonactive 3 0.01926003 Monocytes_3
10x_3281_GTAGACTGTCTTAC-1 Patient4 Monocytes nonactive 3 0.04398897 Monocytes_3
10x_3281_GTAGCAACGAGGGT-1 Patient4 Monocytes nonactive 3 0.04375119 Monocytes_3
10x_3281_GTAGCCCTGAATGA-1 Patient4 Monocytes nonactive 3 0.06886057 Monocytes_3
10x_3281_GTCAACGATCCAGA-1 Patient4 T cells nonactive 9 0.07567688 T cells_9
10x_3281_GTCACAGAGGTTTG-1 Patient4 Monocytes nonactive 3 0.04704838 Monocytes_3
10x_3281_GTCGAATGACCCTC-1 Patient4 Monocytes nonactive 3 0.03108554 Monocytes_3
10x_3281_GTCGACCTAGCTCA-1 Patient4 Monocytes nonactive 3 0.05927018 Monocytes_3
10x_3281_GTCGCACTTTATCC-1 Patient4 Monocytes nonactive 3 0.07726206 Monocytes_3
10x_3281_GTGATTCTGTGTTG-1 Patient4 Monocytes nonactive 3 0.04768246

Monocytes_3
10x_3281_GTGCAAACCCCACT-1 Patient4 T cells nonactive 9 0.07356858 T cells_9
10x_3281_GTGGAGGAGGACGA-1 Patient4 Monocytes nonactive 3 0.04389386
Monocytes_3
10x_3281_GTGTGATGACGACT-1 Patient4 Monocytes nonactive 3 0.03683977
Monocytes_3
10x_3281_GTTAAAACTGGAGG-1 Patient4 Monocytes nonactive 3 0.05334158
Monocytes_3
10x_3281_GTTAACCTAGCTCA-1 Patient4 Monocytes nonactive 3 0.07352102
Monocytes_3
10x_3281_GTTACGGAGCATCA-1 Patient4 Monocytes nonactive 3 0.03563503
Monocytes_3
10x_3281_GTTAGGTGCATGGT-1 Patient4 Monocytes nonactive 3 0.0594287
Monocytes_3
10x_3281_GTTCAGGACAGAGG-1 Patient4 Monocytes nonactive 3 0.04996513
Monocytes_3
10x_3281_GTTGACGATCTCTA-1 Patient4 Monocytes nonactive 3 0.042166
Monocytes_3
10x_3281_GTTGTACTGAGATA-1 Patient4 T cells nonactive 9 0.08663052 T cells_9
10x_3281_TAAATCGAATTCTC-1 Patient4 Monocytes nonactive 2 0.0585727
Monocytes_2
10x_3281_TAAATGTGAACTGC-1 Patient4 Monocytes nonactive 3 0.02926257
Monocytes_3
10x_3281_TAAATGTGGTAGGG-1 Patient4 B cells nonactive 7 0.0691776 B cells_7
10x_3281_TAAATGTGTGGTAC-1 Patient4 Monocytes nonactive 3 0.0337328
Monocytes_3
10x_3281_TAAATGTGTTCTAC-1 Patient4 Monocytes nonactive 3 0.05449876
Monocytes_3
10x_3281_TAACAATGGTCATG-1 Patient4 Monocytes nonactive 3 0.05159787
Monocytes_3
10x_3281_TAACCGGATTTGCT-1 Patient4 Monocytes nonactive 3 0.03823473
Monocytes_3
10x_3281_TAACTCACTTGACG-1 Patient4 Monocytes nonactive 3 0.04750808

Monocytes_3
10x_3281_TAAGATACTAGTCG-1 Patient4 Monocytes nonactive 3 0.02214508 Monocytes_3
10x_3281_TAAGGGCTGTTACG-1 Patient4 Monocytes nonactive 3 0.03934437 Monocytes_3
10x_3281_TAAGTCCTTACAGC-1 Patient4 Monocytes nonactive 3 0.05177224 Monocytes_3
10x_3281_TAATGATGTTCTGT-1 Patient4 Monocytes nonactive 2 0.06927272 Monocytes_2
10x_3281_TAATGCCTTCCTGC-1 Patient4 Monocytes nonactive 3 0.02718597 Monocytes_3
10x_3281_TAATGTGACTTGGA-1 Patient4 Monocytes nonactive 2 0.0594287 Monocytes_2
10x_3281_TACAAATGGCAGTT-1 Patient4 Monocytes nonactive 3 0.03270243 Monocytes_3
10x_3281_TACAATGACCTCAC-1 Patient4 Monocytes nonactive 3 0.03682392 Monocytes_3
10x_3281_TACATAGATTCGCC-1 Patient4 Monocytes nonactive 3 0.08122503 Monocytes_3
10x_3281_TACCGCTGGGATTC-1 Patient4 Monocytes nonactive 3 0.01856255 Monocytes_3
10x_3281_TACGACGAGAGCTT-1 Patient4 Monocytes nonactive 3 0.04879209 Monocytes_3
10x_3281_TACGAGACTTTCAC-1 Patient4 Monocytes nonactive 3 0.06977998 Monocytes_3
10x_3281_TACGTACTCATGGT-1 Patient4 T cells nonactive 9 0.06188574 T cells_9
10x_3281_TACTACTGACAGTC-1 Patient4 Monocytes nonactive 3 0.00115719 Monocytes_3
10x_3281_TACTCTGATGGAAA-1 Patient4 Monocytes nonactive 2 0.03848836 Monocytes_2
10x_3281_TACTGGGAACCTAG-1 Patient4 Monocytes nonactive 3 0.04333904 Monocytes_3
10x_3281_TACTGGGAGCTAAC-1 Patient4 Monocytes nonactive 3 0.01998922

Monocytes_3					
10x_3281_TACTTTCTGTTACG-1	Patient4	Monocytes	nonactive	3	0.06396234
Monocytes_3					
10x_3281_TAGAAACTGGGCAA-1	Patient4	Monocytes	nonactive	3	0.04684231
Monocytes_3					
10x_3281_TAGAATTGGCGTAT-1	Patient4	Monocytes	nonactive	3	0.09033986
Monocytes_3					
10x_3281_TAGCATCTTGCCCT-1	Patient4	Monocytes	nonactive	3	0.06903494
Monocytes_3					
10x_3281_TAGCCCACACTACG-1	Patient4	Monocytes	nonactive	3	0.05205757
Monocytes_3					
10x_3281_TAGCTACTTGGTAC-1	Patient4	Monocytes	nonactive	3	0.03972481
Monocytes_3					
10x_3281_TAGGACTGGAGAGC-1	Patient4	Monocytes	nonactive	2	0.01486906
Monocytes_2					
10x_3281_TAGGAGCTTCTCCG-1	Patient4	Monocytes	nonactive	3	0.05278676
Monocytes_3					
10x_3281_TAGGTGTGCTCTTA-1	Patient4	Monocytes	nonactive	3	0.04815801
Monocytes_3					
10x_3281_TAGTATGAGACGAG-1	Patient4	Monocytes	nonactive	3	0.02723353
Monocytes_3					
10x_3281_TAGTTGCTCATTGG-1	Patient4	Monocytes	nonactive	3	0.05896899
Monocytes_3					
10x_3281_TATAAGACTCTCGC-1	Patient4	Monocytes	nonactive	3	0.04383045
Monocytes_3					
10x_3281_TATACCACGCATCA-1	Patient4	Monocytes	nonactive	2	0.03733118
Monocytes_2					
10x_3281_TATACGCTCCCTAC-1	Patient4	Monocytes	nonactive	3	0.03523873
Monocytes_3					
10x_3281_TATACGCTGTACAC-1	Patient4	Monocytes	nonactive	2	0.03447784
Monocytes_2					
10x_3281_TATAGCCTGATACC-1	Patient4	Progenitors	nonactive	6	0.11492613
Progenitors_6					

10x_3281_TATCACTGCATTCT-1 Patient4 Monocytes nonactive 3 0.04660453 Monocytes_3
10x_3281_TATCAGCTTCGTAG-1 Patient4 Monocytes nonactive 3 0.04482912 Monocytes_3
10x_3281_TATCCAACTCTCGC-1 Patient4 Monocytes nonactive 3 0.05172468 Monocytes_3
10x_3281_TATCGTACAGCACT-1 Patient4 Monocytes nonactive 2 0.02972228 Monocytes_2
10x_3281_TATCTCGATCTATC-1 Patient4 T cells nonactive 9 0.06023714 T cells_9
10x_3281_TATCTGACCTAGTG-1 Patient4 Monocytes nonactive 3 0.04795194 Monocytes_3
10x_3281_TATCTTCTCATTCT-1 Patient4 Monocytes nonactive 3 0.04750808 Monocytes_3
10x_3281_TATGCGGAGAATAG-1 Patient4 Monocytes nonactive 3 0.01926003 Monocytes_3
10x_3281_TATGGGTGGTATCG-1 Patient4 T cells active 9 0.12063281 T cells_9
10x_3281_TATGTCACGTCCTC-1 Patient4 Monocytes nonactive 3 0.07141272 Monocytes_3
10x_3281_TATGTCTGGTAGGG-1 Patient4 Monocytes nonactive 3 0.02634582 Monocytes_3
10x_3281_TATTGCTGCCAGTA-1 Patient4 Monocytes nonactive 3 0.02859679 Monocytes_3
10x_3281_TCAACACTACTAGC-1 Patient4 Monocytes nonactive 3 0.05169298 Monocytes_3
10x_3281_TCAAGTCTCGATAC-1 Patient4 Monocytes nonactive 3 0.07600976 Monocytes_3
10x_3281_TCACATACGATGAA-1 Patient4 Monocytes nonactive 3 0.05340498 Monocytes_3
10x_3281_TCACGAGAAAGAGT-1 Patient4 Monocytes nonactive 2 0.07260161 Monocytes_2
10x_3281_TCACGAGATTTACC-1 Patient4 Monocytes nonactive 3 0.04157948 Monocytes_3
10x_3281_TCAGAGACGACAAA-1 Patient4 Monocytes nonactive 2 0.03482658

Monocytes_2
10x_3281_TCAGGATGCGCATA-1	Patient4 Monocytes	nonactive	3	0.05454632
Monocytes_3
10x_3281_TCAGGATGTGCTGA-1	Patient4 Monocytes	nonactive	2	0.01807114
Monocytes_2
10x_3281_TCAGTTACAGAGAT-1	Patient4 T cells	nonactive	9	0.08988016	T cells_9
10x_3281_TCCGAGCTAGGTTC-1	Patient4 Monocytes	nonactive	3	0.10273603
Monocytes_3
10x_3281_TCCTAATGACTTTC-1	Patient4 Monocytes	nonactive	3	0.06202841
Monocytes_3
10x_3281_TCGACGCTGAAACA-1	Patient4 Monocytes	nonactive	3	0.02704331
Monocytes_3
10x_3281_TCGATACTGTTTGG-1	Patient4 Monocytes	nonactive	3	0.03151354
Monocytes_3
10x_3281_TCGATTTGCCAATG-1	Patient4 Monocytes	nonactive	3	0.0354448
Monocytes_3
10x_3281_TCGATTTGGTCACA-1	Patient4 Monocytes	nonactive	3	0.05789107
Monocytes_3
10x_3281_TCGGTAGAGCGTAT-1	Patient4 Monocytes	nonactive	2	0.03003931
Monocytes_2
10x_3281_TCGTGAGAGTACAC-1	Patient4 Monocytes	nonactive	3	0.03385962
Monocytes_3
10x_3281_TCTCAAACCTTTAC-1	Patient4 Monocytes	nonactive	3	0.03146598
Monocytes_3
10x_3281_TCTCCACTCGCATA-1	Patient4 Monocytes	nonactive	16	0.05622662
Monocytes_16
10x_3281_TCTCTAGAGTCGTA-1	Patient4 Monocytes	nonactive	3	0.03672881
Monocytes_3
10x_3281_TCTTGATGCCCTAC-1	Patient4 Monocytes	nonactive	3	0.02666286
Monocytes_3
10x_3281_TGACCAGACACTCC-1	Patient4 Monocytes	nonactive	3	0.0383774
Monocytes_3
10x_3281_TGACGATGTCCAGA-1	Patient4 Monocytes	nonactive	2	0.04489252

Monocytes_2
10x_3281_TGACGCCTCCGTAA-1 Patient4 Monocytes nonactive 3 0.04167459 Monocytes_3
10x_3281_TGACTGGACGCCTT-1 Patient4 Monocytes nonactive 2 0.06174307 Monocytes_2
10x_3281_TGACTGGATTGGCA-1 Patient4 Monocytes nonactive 3 0.07967155 Monocytes_3
10x_3281_TGACTTTGTCATTC-1 Patient4 Monocytes nonactive 2 0.04360852 Monocytes_2
10x_3281_TGAGACACGTCACA-1 Patient4 Monocytes nonactive 3 0.05602054 Monocytes_3
10x_3281_TGAGGACTGCATCA-1 Patient4 Monocytes nonactive 3 0.02369856 Monocytes_3
10x_3281_TGAGTCGAAACGAA-1 Patient4 T cells nonactive 9 0.04429015 T cells_9
10x_3281_TGAGTCGACTGAGT-1 Patient4 Monocytes nonactive 3 0.04253059 Monocytes_3
10x_3281_TGATACCTAAAGCA-1 Patient4 T cells nonactive 9 0.04016866 T cells_9
10x_3281_TGATTAGACCTTTA-1 Patient4 Monocytes nonactive 3 0.04075518 Monocytes_3
10x_3281_TGATTCTGACCTGA-1 Patient4 Monocytes nonactive 3 0.04584364 Monocytes_3
10x_3281_TGATTCTGCCTTGC-1 Patient4 Monocytes nonactive 3 0.06619745 Monocytes_3
10x_3281_TGATTCTGTCAGGT-1 Patient4 Monocytes nonactive 3 0.03698244 Monocytes_3
10x_3281_TGCAACGACATTTC-1 Patient4 Monocytes nonactive 3 0.05749477 Monocytes_3
10x_3281_TGCAAGTGCAGAAA-1 Patient4 Monocytes nonactive 3 0.05697166 Monocytes_3
10x_3281_TGCACGCTGAATCC-1 Patient4 Monocytes nonactive 3 0.04305371 Monocytes_3
10x_3281_TGCCAGCTCCGAAT-1 Patient4 T cells nonactive 9 0.1121362 T cells_9
10x_3281_TGCCGACTGTCTTT-1 Patient4 Monocytes nonactive 2 0.03512777

Monocytes_2

10x_3281_TGCTAGGAAGCGGA-1	Patient4	Monocytes	nonactive	3	0.01750048	Monocytes_3
10x_3281_TGCTAGGAGAGACG-1	Patient4	Monocytes	nonactive	3	0.03439858	Monocytes_3
10x_3281_TGGAAAGACTGAGT-1	Patient4	Monocytes	nonactive	2	0.03186228	Monocytes_2
10x_3281_TGGAGACTGAACTC-1	Patient4	Monocytes	nonactive	2	0.041738	Monocytes_2
10x_3281_TGGATGACGTATCG-1	Patient4	Monocytes	nonactive	3	0.01905396	Monocytes_3
10x_3281_TGGATGACTGTGCA-1	Patient4	Monocytes	nonactive	3	0.01464714	Monocytes_3
10x_3281_TGGATTCTTGCATG-1	Patient4	Monocytes	nonactive	3	0.07577199	Monocytes_3
10x_3281_TGGTATCTGTCTTT-1	Patient4	Monocytes	nonactive	3	0.04001015	Monocytes_3
10x_3281_TGTATCTGTCCTCG-1	Patient4	Monocytes	nonactive	3	0.02426923	Monocytes_3
10x_3281_TGTCAGGAAAGTAG-1	Patient4	Monocytes	nonactive	2	0.06919346	Monocytes_2
10x_3281_TGTCTAACAGCGTT-1	Patient4	Monocytes	nonactive	2	0.04481326	Monocytes_2
10x_3281_TGTTACACAGTAGA-1	Patient4	Monocytes	nonactive	3	0.05245387	Monocytes_3
10x_3281_TGTTACTGCACTAG-1	Patient4	Monocytes	nonactive	2	0.04562171	Monocytes_2
10x_3281_TGTTACTGCCCTAC-1	Patient4	Monocytes	nonactive	3	0.06209181	Monocytes_3
10x_3281_TGTTACTGTGCAGT-1	Patient4	Monocytes	nonactive	3	0.04715934	Monocytes_3
10x_3281_TTACTCGAGTCCTC-1	Patient4	Monocytes	nonactive	3	0.06559508	Monocytes_3

10x_3281_TTAGACCTTGTGGT-1 Patient4 Monocytes nonactive 3 0.07413924 Monocytes_3
10x_3281_TTAGGGTGCTTGCC-1 Patient4 Monocytes nonactive 3 0.04471815 Monocytes_3
10x_3281_TTAGTCACTCGCTC-1 Patient4 Monocytes nonactive 3 0.05032972 Monocytes_3
10x_3281_TTCAAAGAAGAAGT-1 Patient4 Monocytes nonactive 3 0.03691903 Monocytes_3
10x_3281_TTCACAACTAGCCA-1 Patient4 Monocytes nonactive 3 0.04449623 Monocytes_3
10x_3281_TTCACCCTACGCAT-1 Patient4 Monocytes nonactive 3 0.0240473 Monocytes_3
10x_3281_TTCATTCTTCCGAA-1 Patient4 Progenitors active 6 0.15400101 Progenitors_6
10x_3281_TTCCATGAGGTAAA-1 Patient4 Monocytes nonactive 3 0.04211845 Monocytes_3
10x_3281_TTCCTAGACCACAA-1 Patient4 Monocytes nonactive 2 0.04947372 Monocytes_2
10x_3281_TTCCTAGATGCAAC-1 Patient4 Progenitors nonactive 6 0.11349946 Progenitors_6
10x_3281_TTCGAGGACTGTTT-1 Patient4 Monocytes nonactive 3 0.05429269 Monocytes_3
10x_3281_TTCTACGATGCACA-1 Patient4 Monocytes nonactive 3 0.0674656 Monocytes_3
10x_3281_TTCTAGTGGTCCTC-1 Patient4 Monocytes nonactive 3 0.0573204 Monocytes_3
10x_3281_TTCTTACTACCCTC-1 Patient4 T cells nonactive 9 0.04841164 T cells_9
10x_3281_TTGAATGAACCTTT-1 Patient4 Monocytes nonactive 2 0.03809207 Monocytes_2
10x_3281_TTGAGGTGGTCACA-1 Patient4 Monocytes nonactive 3 0.05456217 Monocytes_3
10x_3281_TTGTCATGTGACTG-1 Patient4 Monocytes nonactive 2 0.08279437 Monocytes_2

10x_3281_TTGTCATGTTACCT-1 Patient4 Monocytes nonactive 3 0.03184643 Monocytes_3
10x_3281_TTTAGGCTATGTCG-1 Patient4 Monocytes nonactive 3 0.0648976 Monocytes_3
10x_3281_TTTAGGCTCCAAGT-1 Patient4 Monocytes nonactive 3 0.05806544 Monocytes_3
10x_3281_TTTCACGATTCAGG-1 Patient4 T cells nonactive 9 0.10750745 T cells_9
10x_3281_TTTCAGTGCGTGAT-1 Patient4 Monocytes nonactive 3 0.03796525 Monocytes_3
10x_3281_TTTCAGTGTGGTTG-1 Patient4 Monocytes nonactive 3 0.04253059 Monocytes_3
10x_3281_TTTCGAACTATCTC-1 Patient4 Monocytes nonactive 3 0.05151861 Monocytes_3
10x_1976_AAAGTTTGACACGT-1 Patient1 Dendritic cells active 10 0.12488111 Dendritic cells_10
10x_1976_AAATACTGATGCCA-1 Patient1 T cells nonactive 9 0.08586963 T cells_9
10x_1976_AAATCAACAGCGGA-1 Patient1 Dendritic cells nonactive 10 0.04703253 Dendritic cells_10
10x_1976_AAATCCCTGACAAA-1 Patient1 Monocytes nonactive 3 0.02277915 Monocytes_3
10x_1976_AAATCCCTGTAAGA-1 Patient1 Dendritic cells nonactive 10 0.08525141 Dendritic cells_10
10x_1976_AAATGTTGGGTGTT-1 Patient1 Monocytes nonactive 15 0.07277598 Monocytes_15
10x_1976_AACAATACGGAAAT-1 Patient1 Dendritic cells nonactive 10 0.08366622 Dendritic cells_10
10x_1976_AACACGTGCCTGAA-1 Patient1 Dendritic cells nonactive 10 0.08055925 Dendritic cells_10
10x_1976_AACATTGATTACTC-1 Patient1 Progenitors active 1 0.13052438 Progenitors_1
10x_1976_AACCTACTTTCGCC-1 Patient1 Dendritic cells nonactive 10 0.04004185 Dendritic cells_10
10x_1976_AACGCAACTTACTC-1 Patient1 Monocytes nonactive 3 0.03519117

Monocytes_3

10x_1976_AACGCATGTGATGC-1	Patient1	Monocytes	nonactive	3	0.08200178	Monocytes_3
10x_1976_AACTGTCTGTTGCA-1	Patient1	Dendritic cells	nonactive	10	0.0725065	Dendritic cells_10
10x_1976_AAGAATCTAAGCCT-1	Patient1	Progenitors	nonactive	1	0.09691839	Progenitors_1
10x_1976_AAGGCTACATGACC-1	Patient1	Monocytes	nonactive	8	0.0400894	Monocytes_8
10x_1976_AAGTCTCTTGTTTC-1	Patient1	Progenitors	nonactive	1	0.07784858	Progenitors_1
10x_1976_AATCCGGAGGTGAG-1	Patient1	Monocytes	nonactive	3	0.03184643	Monocytes_3
10x_1976_AATCCTTGGGTAGG-1	Patient1	Dendritic cells	nonactive	10	0.0767548	Dendritic cells_10
10x_1976_AATCTCACATGCCA-1	Patient1	Monocytes	nonactive	4	0.0257276	Monocytes_4
10x_1976_AATCTCACTATGGC-1	Patient1	Progenitors	nonactive	1	0.0876609	Progenitors_1
10x_1976_ACAAGAGAGTCGTA-1	Patient1	Dendritic cells	nonactive	10	0.07707184	Dendritic cells_10
10x_1976_ACACGATGTCGCTC-1	Patient1	Monocytes	nonactive	3	0.01908566	Monocytes_3
10x_1976_ACAGTGTGCGAGTT-1	Patient1	Dendritic cells	nonactive	10	0.10555767	Dendritic cells_10
10x_1976_ACATGGTGTGCGTA-1	Patient1	Dendritic cells	nonactive	14	0.11613087	Dendritic cells_14
10x_1976_ACATTCTGTCTCTA-1	Patient1	Monocytes	nonactive	3	0.05213683	Monocytes_3
10x_1976_ACCAACGACCACAA-1	Patient1	Monocytes	nonactive	13	0.05010779	Monocytes_13
10x_1976_ACCAACGACCAGTA-1	Patient1	Dendritic cells	nonactive	10	0.08956312	Dendritic cells_10

10x_1976_ACCAACGACGTAAC-1 Patient1 Monocytes nonactive 0 0.06374041
Monocytes_0
10x_1976_ACCACGCTGTACCA-1 Patient1 Progenitors active 1 0.13508972
Progenitors_1
10x_1976_ACCAGTGAGCTAAC-1 Patient1 Monocytes nonactive 3 0.04062837
Monocytes_3
10x_1976_ACCAGTGATACGCA-1 Patient1 Monocytes nonactive 3 0.0724748
Monocytes_3
10x_1976_ACCATTTGAACCGT-1 Patient1 Monocytes nonactive 3 0.02967472
Monocytes_3
10x_1976_ACCCAAGAATCTTC-1 Patient1 Dendritic cells nonactive 10 0.03481073
Dendritic cells_10
10x_1976_ACCCGTACGAATGA-1 Patient1 Dendritic cells nonactive 10 0.06806797
Dendritic cells_10
10x_1976_ACCGTGCTGGAGCA-1 Patient1 Progenitors nonactive 1 0.10809397
Progenitors_1
10x_1976_ACCTCGTGCCCTCA-1 Patient1 Dendritic cells nonactive 10 0.08804134
Dendritic cells_10
10x_1976_ACCTTTGACTGTCC-1 Patient1 Monocytes nonactive 0 0.06335996
Monocytes_0
10x_1976_ACGAACTGCTTGAG-1 Patient1 Monocytes nonactive 3 0.05359521
Monocytes_3
10x_1976_ACGAAGCTGCCATA-1 Patient1 Dendritic cells active 10 0.13212542
Dendritic cells_10
10x_1976_ACGACAACAAAACG-1 Patient1 Dendritic cells nonactive 10 0.05548158
Dendritic cells_10
10x_1976_ACGACAACCAGGAG-1 Patient1 Dendritic cells nonactive 10 0.10598567
Dendritic cells_10
10x_1976_ACGCAATGAGGCGA-1 Patient1 Monocytes nonactive 3 0.04202333
Monocytes_3
10x_1976_ACGCACCTCAATCG-1 Patient1 Monocytes nonactive 3 0.00351912
Monocytes_3
10x_1976_ACGTAGACGCTACA-1 Patient1 Dendritic cells nonactive 10 0.03634836

Dendritic cells_10
10x_1976_ACGTTGGAAGTCTG-1 Patient1 Dendritic cells nonactive 10 0.08022636
Dendritic cells_10
10x_1976_ACGTTGGATTTCAC-1 Patient1 Dendritic cells nonactive 10 0.06905079
Dendritic cells_10
10x_1976_ACTAGGTGTCCTAT-1 Patient1 Dendritic cells nonactive 10 0.05116987
Dendritic cells_10
10x_1976_ACTGTTACGGGCAA-1 Patient1 Monocytes nonactive 3 0.05437195
Monocytes_3
10x_1976_ACTTAGCTATCAGC-1 Patient1 Dendritic cells nonactive 10 0.08063851
Dendritic cells_10
10x_1976_AGAAACGACGAATC-1 Patient1 Dendritic cells nonactive 10 0.07345761
Dendritic cells_10
10x_1976_AGAACGCTCAGAGG-1 Patient1 Dendritic cells nonactive 10 0.07445628
Dendritic cells_10
10x_1976_AGAACGCTCTGCAA-1 Patient1 Monocytes nonactive 3 0.07185657
Monocytes_3
10x_1976_AGAATACTCATCAG-1 Patient1 Monocytes nonactive 3 0.03392302
Monocytes_3
10x_1976_AGACACACGTTACG-1 Patient1 Monocytes nonactive 3 0.02832731
Monocytes_3
10x_1976_AGACGTACCGAGAG-1 Patient1 Monocytes active 8 0.13653224
Monocytes_8
10x_1976_AGAGAATGTTCGTT-1 Patient1 Dendritic cells nonactive 10 0.10417856
Dendritic cells_10
10x_1976_AGAGCTACTGGTCA-1 Patient1 Dendritic cells nonactive 10 0.11660643
Dendritic cells_10
10x_1976_AGATATACTGGATC-1 Patient1 Dendritic cells nonactive 10 0.06297952
Dendritic cells_10
10x_1976_AGATCGTGGTACGT-1 Patient1 Monocytes nonactive 3 0.05132839
Monocytes_3
10x_1976_AGCAACACACTGTG-1 Patient1 Dendritic cells nonactive 10 0.0889766
Dendritic cells_10

10x_1976_AGCATCGAGTGAGG-1 Patient1 Monocytes nonactive 3 0.0337645 Monocytes_3
10x_1976_AGCATGACTTGCTT-1 Patient1 Dendritic cells nonactive 10 0.07911673 Dendritic cells_10
10x_1976_AGCGATACTGGTTG-1 Patient1 Dendritic cells nonactive 10 0.07713525 Dendritic cells_10
10x_1976_AGCGCCGAACCATG-1 Patient1 Monocytes nonactive 0 0.04625579 Monocytes_0
10x_1976_AGCGCCGAAGTGTC-1 Patient1 Dendritic cells nonactive 10 0.0476032 Dendritic cells_10
10x_1976_AGCGGGCTACCTCC-1 Patient1 Monocytes nonactive 3 0.06882886 Monocytes_3
10x_1976_AGCGGGCTCGTGAT-1 Patient1 Dendritic cells nonactive 10 0.07664384 Dendritic cells_10
10x_1976_AGGACTTGCAGGAG-1 Patient1 Progenitors active 6 0.1479773 Progenitors_6
10x_1976_AGGACTTGCCTATT-1 Patient1 Monocytes nonactive 15 0.02661531 Monocytes_15
10x_1976_AGGCTAACACCATG-1 Patient1 Monocytes nonactive 13 0.04265741 Monocytes_13
10x_1976_AGGGACGAAAGATG-1 Patient1 Dendritic cells nonactive 10 0.08187496 Dendritic cells_10
10x_1976_AGGGAGTGATCGAC-1 Patient1 Monocytes nonactive 3 0.03027709 Monocytes_3
10x_1976_AGGGCGCTCACTGA-1 Patient1 Monocytes nonactive 0 0.11262761 Monocytes_0
10x_1976_AGGGTTTGTGAAGA-1 Patient1 Monocytes nonactive 3 0.05072602 Monocytes_3
10x_1976_AGGTACTGACCATG-1 Patient1 Dendritic cells nonactive 10 0.08547334 Dendritic cells_10
10x_1976_AGGTACTGGGAAGC-1 Patient1 Dendritic cells nonactive 10 0.06924101 Dendritic cells_10
10x_1976_AGTAGGCTTGTGGT-1 Patient1 Monocytes nonactive 2 0.02189145

Monocytes_2

10x_1976_AGTGACACACCTTT-1 Patient1 Dendritic cells nonactive 10 0.07493184 Dendritic cells_10

10x_1976_AGTGTGACGTGCTA-1 Patient1 Dendritic cells nonactive 10 0.05885803 Dendritic cells_10

10x_1976_AGTTAAACTCAAGC-1 Patient1 Dendritic cells nonactive 10 0.05725699 Dendritic cells_10

10x_1976_AGTTGTCTTAGCGT-1 Patient1 Dendritic cells nonactive 10 0.05887388 Dendritic cells_10

10x_1976_AGTTTAGATCACGA-1 Patient1 Progenitors nonactive 6 0.10183248 Progenitors_6

10x_1976_AGTTTGCTCAGAAA-1 Patient1 Progenitors nonactive 6 0.08915097 Progenitors_6

10x_1976_ATAAACACAGTCAC-1 Patient1 Dendritic cells nonactive 10 0.02170122 Dendritic cells_10

10x_1976_ATAATCGACTTGGA-1 Patient1 Dendritic cells nonactive 10 0.04652527 Dendritic cells_10

10x_1976_ATAATCGAGCTTCC-1 Patient1 Monocytes nonactive 3 0.03961385 Monocytes_3

10x_1976_ATAATCGAGTCTGA-1 Patient1 Monocytes nonactive 3 0.09070446 Monocytes_3

10x_1976_ATACCACTTCAGAC-1 Patient1 Monocytes nonactive 3 0.04425845 Monocytes_3

10x_1976_ATACCACTTTGCAG-1 Patient1 Monocytes nonactive 3 0.03087946 Monocytes_3

10x_1976_ATACCGGATGAACC-1 Patient1 Monocytes nonactive 3 0.01206328 Monocytes_3

10x_1976_ATACCTTGTGAGAA-1 Patient1 Dendritic cells nonactive 10 0.0923689 Dendritic cells_10

10x_1976_ATAGATTGTCCTGC-1 Patient1 Monocytes nonactive 4 0.06386722 Monocytes_4

10x_1976_ATAGGAGAGGTCAT-1 Patient1 Progenitors nonactive 1 0.10776108 Progenitors_1

10x_1976_ATATAGTGGGAGCA-1 Patient1 Monocytes nonactive 8 0.04292689
Monocytes_8
10x_1976_ATCAACCTCTATGG-1 Patient1 Dendritic cells nonactive 10 0.05984085
Dendritic cells_10
10x_1976_ATCACGGACTACTT-1 Patient1 Dendritic cells nonactive 10 0.06164796
Dendritic cells_10
10x_1976_ATCCATACGGAAGC-1 Patient1 Monocytes nonactive 8 0.02962716
Monocytes_8
10x_1976_ATCGACGAGTCTTT-1 Patient1 Progenitors active 1 0.12678334
Progenitors_1
10x_1976_ATCGCAGATGCCTC-1 Patient1 Monocytes nonactive 3 0.04731786
Monocytes_3
10x_1976_ATCGGTGATGGTGT-1 Patient1 Dendritic cells nonactive 10 0.03588866
Dendritic cells_10
10x_1976_ATCTGGGACTGTGA-1 Patient1 Monocytes nonactive 0 0.03852007
Monocytes_0
10x_1976_ATCTGGGATAGAAG-1 Patient1 Dendritic cells nonactive 10 0.05361106
Dendritic cells_10
10x_1976_ATGAAACTTACGAC-1 Patient1 Progenitors active 1 0.12274111
Progenitors_1
10x_1976_ATGACGTGATTCCT-1 Patient1 Dendritic cells nonactive 10 0.08363452
Dendritic cells_10
10x_1976_ATGCAGACTGGAAA-1 Patient1 Monocytes nonactive 3 0.04842749
Monocytes_3
10x_1976_ATGGGTACAGTCAC-1 Patient1 Dendritic cells nonactive 10 0.05208928
Dendritic cells_10
10x_1976_ATGTCACTGGCAAG-1 Patient1 Dendritic cells nonactive 10 0.0661499
Dendritic cells_10
10x_1976_ATGTCGGAACCTAG-1 Patient1 Dendritic cells nonactive 10 0.09149705
Dendritic cells_10
10x_1976_ATTACCTGAGAAGT-1 Patient1 Dendritic cells nonactive 10 0.05581447
Dendritic cells_10
10x_1976_ATTAGATGAGAGGC-1 Patient1 Monocytes nonactive 3 0.05516454

Monocytes_3
10x_1976_ATTAGTGAAAGGCG-1 Patient1 Dendritic cells nonactive 10 0.06527804
Dendritic cells_10
10x_1976_ATTCTGACACCGAT-1 Patient1 Dendritic cells nonactive 10 0.0839357
Dendritic cells_10
10x_1976_ATTGATGAACCCTC-1 Patient1 Dendritic cells nonactive 10 0.09492106
Dendritic cells_10
10x_1976_ATTGCGGAGCGGAA-1 Patient1 Monocytes nonactive 3 0.04708008
Monocytes_3
10x_1976_ATTGCTACGGTATC-1 Patient1 Progenitors active 1 0.12965253
Progenitors_1
10x_1976_ATTGTAGATGTTTC-1 Patient1 Dendritic cells nonactive 10 0.07938622
Dendritic cells_10
10x_1976_ATTTGCACATGTCG-1 Patient1 Monocytes nonactive 3 0.05846173
Monocytes_3
10x_1976_CAACAGACTCGTTT-1 Patient1 Monocytes nonactive 3 0.05468899
Monocytes_3
10x_1976_CAACGTGACCTTTA-1 Patient1 Monocytes nonactive 3 0.04639845
Monocytes_3
10x_1976_CAAGGTTGCGATAC-1 Patient1 Monocytes nonactive 3 0.04728616
Monocytes_3
10x_1976_CAATCGGATCTCTA-1 Patient1 Dendritic cells nonactive 10 0.05050409
Dendritic cells_10
10x_1976_CACACCTGCTGCTC-1 Patient1 Progenitors active 1 0.14811997
Progenitors_1
10x_1976_CACAGAACCTGTGA-1 Patient1 Progenitors nonactive 1 0.07095302
Progenitors_1
10x_1976_CACAGATGCAACTG-1 Patient1 Progenitors nonactive 1 0.09666476
Progenitors_1
10x_1976_CACATACTCGGGAA-1 Patient1 Dendritic cells nonactive 10 0.0893412
Dendritic cells_10
10x_1976_CACATGGAGAGGTG-1 Patient1 Monocytes nonactive 3 0.06806797
Monocytes_3

10x_1976_CACCACTGCTGCTC-1 Patient1 Dendritic cells nonactive 10 0.05071016
Dendritic cells_10
10x_1976_CACCGGGACGCCTT-1 Patient1 Monocytes nonactive 0 0.03490584
Monocytes_0
10x_1976_CACCGTACCTATTC-1 Patient1 Dendritic cells nonactive 10 0.07795955
Dendritic cells_10
10x_1976_CACCGTACGTGAGG-1 Patient1 Progenitors nonactive 1 0.10948894
Progenitors_1
10x_1976_CACTATACCCGTTC-1 Patient1 Progenitors nonactive 1 0.08116163
Progenitors_1
10x_1976_CACTCTCTTCGCCT-1 Patient1 Monocytes nonactive 3 0.02220848
Monocytes_3
10x_1976_CACTTTGATGATGC-1 Patient1 Dendritic cells nonactive 10 0.06594382
Dendritic cells_10
10x_1976_CAGATCGAAAGGCG-1 Patient1 Monocytes nonactive 3 0.0164384
Monocytes_3
10x_1976_CAGATGACAGCAAA-1 Patient1 Dendritic cells active 10 0.14574219
Dendritic cells_10
10x_1976_CAGCAATGCGGGAA-1 Patient1 Dendritic cells nonactive 10 0.08859616
Dendritic cells_10
10x_1976_CAGCTCACACAGTC-1 Patient1 Dendritic cells nonactive 10 0.06939953
Dendritic cells_10
10x_1976_CAGCTCACCGTAGT-1 Patient1 Monocytes nonactive 3 0.04528882
Monocytes_3
10x_1976_CAGGTATGCCTATT-1 Patient1 Monocytes nonactive 0 0.04853846
Monocytes_0
10x_1976_CAGGTTGAAGATCC-1 Patient1 Monocytes nonactive 3 0.04969564
Monocytes_3
10x_1976_CAGGTTGAGTTGGT-1 Patient1 Dendritic cells nonactive 10 0.09658551
Dendritic cells_10
10x_1976_CATAACCTAGAGAT-1 Patient1 Monocytes nonactive 3 0.06442204
Monocytes_3
10x_1976_CATAGTCTCCGTTC-1 Patient1 Monocytes nonactive 2 0.03254391

Monocytes_2
10x_1976_CATCAGGATCGCCT-1 Patient1 Monocytes nonactive 3 0.02341323
Monocytes_3
10x_1976_CATCATACAAGAGT-1 Patient1 Monocytes nonactive 3 0.04257815
Monocytes_3
10x_1976_CATGCCACCCTGAA-1 Patient1 Dendritic cells nonactive 10 0.07724621
Dendritic cells_10
10x_1976_CATGCCACTCTGGA-1 Patient1 B cells nonactive 7 0.04952127 B cells_7
10x_1976_CATTGACTGTTCTT-1 Patient1 Monocytes nonactive 2 0.06979583
Monocytes_2
10x_1976_CCAAAGTGCTTCCG-1 Patient1 Progenitors active 1 0.11880984
Progenitors_1
10x_1976_CCAGTCTGTTTGGG-1 Patient1 Progenitors nonactive 1 0.1117082
Progenitors_1
10x_1976_CCCAGACTCCCACT-1 Patient1 Monocytes nonactive 4 0.01038298
Monocytes_4
10x_1976_CCCTAGTGTGTCCC-1 Patient1 Dendritic cells nonactive 10 0.06345508
Dendritic cells_10
10x_1976_CCCTGAACTAAGGA-1 Patient1 Dendritic cells nonactive 10 0.06692664
Dendritic cells_10
10x_1976_CCGCGAGATGGTTG-1 Patient1 Dendritic cells nonactive 10 0.03550821
Dendritic cells_10
10x_1976_CCGGAGACTCAGTG-1 Patient1 Monocytes nonactive 8 0.08407837
Monocytes_8
10x_1976_CCGTAAGATTATCC-1 Patient1 Monocytes nonactive 4 0.03404984
Monocytes_4
10x_1976_CCTAGAGAGCAAGG-1 Patient1 Dendritic cells nonactive 10 0.08588549
Dendritic cells_10
10x_1976_CCTATAACGTCAAC-1 Patient1 Monocytes nonactive 3 0.01108046
Monocytes_3
10x_1976_CCTATTGACATCAG-1 Patient1 Monocytes nonactive 8 0.03902733
Monocytes_8
10x_1976_CGAACATGCCTAAG-1 Patient1 Monocytes nonactive 13 0.03574599

Monocytes_13
10x_1976_CGACCTTGATGTCG-1 Patient1 Progenitors active 1 0.12418363
Progenitors_1
10x_1976_CGACTCTGCCCACT-1 Patient1 Monocytes nonactive 3 0.0489189
Monocytes_3
10x_1976_CGAGCGTGTCCAGA-1 Patient1 Monocytes nonactive 3 0.05717773
Monocytes_3
10x_1976_CGAGGCACCTGGTA-1 Patient1 Progenitors nonactive 12 0.11243739
Progenitors_12
10x_1976_CGATCCACCTTACT-1 Patient1 Monocytes nonactive 3 0.02328641
Monocytes_3
10x_1976_CGCACGGAGCCATA-1 Patient1 Monocytes nonactive 4 0.03810792
Monocytes_4
10x_1976_CGCACTACCCTTTA-1 Patient1 Monocytes nonactive 4 0.04622408
Monocytes_4
10x_1976_CGCATAGACTGCTC-1 Patient1 Progenitors active 6 0.1214571
Progenitors_6
10x_1976_CGCCGAGACCAAGT-1 Patient1 Progenitors active 1 0.1399404
Progenitors_1
10x_1976_CGCCTAACATCGGT-1 Patient1 Dendritic cells nonactive 10 0.07497939
Dendritic cells_10
10x_1976_CGCGAGACTCGCAA-1 Patient1 Monocytes nonactive 3 0.03496925
Monocytes_3
10x_1976_CGCGGATGGGTTCA-1 Patient1 Dendritic cells nonactive 10 0.07093716
Dendritic cells_10
10x_1976_CGCGGATGTCAGTG-1 Patient1 Monocytes nonactive 3 0.0459863
Monocytes_3
10x_1976_CGGACCGATCGCTC-1 Patient1 Progenitors active 1 0.13761017
Progenitors_1
10x_1976_CGGCATCTTCTAGG-1 Patient1 Monocytes nonactive 3 0.04436941
Monocytes_3
10x_1976_CGGTACCTTTGCGA-1 Patient1 Monocytes nonactive 13 0.03642762
Monocytes_13

10x_1976_CGTACCACCAGATC-1 Patient1 Progenitors active 1 0.13057194 Progenitors_1
10x_1976_CGTCAAGATCGTGA-1 Patient1 Monocytes nonactive 4 0.00429586 Monocytes_4
10x_1976_CGTCCAACTGGAAA-1 Patient1 Dendritic cells nonactive 10 0.06486589 Dendritic cells_10
10x_1976_CTACTATGCACAAC-1 Patient1 Monocytes nonactive 3 0.09436624 Monocytes_3
10x_1976_CTACTATGTCCGAA-1 Patient1 Monocytes nonactive 15 0.02964302 Monocytes_15
10x_1976_CTAGAGACTATGCG-1 Patient1 Dendritic cells nonactive 10 0.06372456 Dendritic cells_10
10x_1976_CTAGGCCTCTTGGA-1 Patient1 Monocytes nonactive 3 0.01748462 Monocytes_3
10x_1976_CTAGGTGACTCGCT-1 Patient1 Progenitors active 1 0.17153319 Progenitors_1
10x_1976_CTAGTTACCCCGTT-1 Patient1 Dendritic cells nonactive 10 0.05960307 Dendritic cells_10
10x_1976_CTCAATTGGGTACT-1 Patient1 Monocytes nonactive 3 0.05324647 Monocytes_3
10x_1976_CTGAACGACCTGAA-1 Patient1 Monocytes nonactive 15 0.04165874 Monocytes_15
10x_1976_CTGAATCTTTGACG-1 Patient1 Progenitors active 1 0.1454727 Progenitors_1
10x_1976_CTGAGAACTACGCA-1 Patient1 Monocytes nonactive 0 0.04308541 Monocytes_0
10x_1976_CTGAGCCTCGTGTA-1 Patient1 Dendritic cells nonactive 10 0.03829814 Dendritic cells_10
10x_1976_CTGATTTGCTCAAG-1 Patient1 Dendritic cells nonactive 10 0.05023461 Dendritic cells_10
10x_1976_CTGCGACTCCAGTA-1 Patient1 Monocytes nonactive 3 0.07551836 Monocytes_3
10x_1976_CTGTATACTCTCCG-1 Patient1 Monocytes nonactive 3 0.01700907

Monocytes_3
10x_1976_CTTACATGTCATTC-1 Patient1 Dendritic cells nonactive 10 0.09140194
Dendritic cells_10
10x_1976_CTTTAGACGTATCG-1 Patient1 Dendritic cells nonactive 10 0.07442458
Dendritic cells_10
10x_1976_GAAAGCCTCTCAAG-1 Patient1 Dendritic cells nonactive 10 0.06581701
Dendritic cells_10
10x_1976_GAAATACTGAAAGT-1 Patient1 Dendritic cells nonactive 10 0.08043244
Dendritic cells_10
10x_1976_GAACAGCTGACGTT-1 Patient1 Monocytes nonactive 3 0.05809714
Monocytes_3
10x_1976_GAACGTTGACACCA-1 Patient1 Monocytes nonactive 3 0.03439858
Monocytes_3
10x_1976_GAAGAATGGAGGGT-1 Patient1 Progenitors active 1 0.15951747
Progenitors_1
10x_1976_GAAGCTTGGCAGTT-1 Patient1 Progenitors nonactive 1 0.10928286
Progenitors_1
10x_1976_GAAGTAGATTCACT-1 Patient1 Dendritic cells nonactive 10 0.09246402
Dendritic cells_10
10x_1976_GACAACACCAGAGG-1 Patient1 Monocytes nonactive 0 0.05836662
Monocytes_0
10x_1976_GACCATGACTGGAT-1 Patient1 Progenitors active 1 0.13971847
Progenitors_1
10x_1976_GACGATTGTGACTG-1 Patient1 Monocytes nonactive 3 0.0412783
Monocytes_3
10x_1976_GACGCTCTAAGAAC-1 Patient1 Dendritic cells nonactive 10 0.09736225
Dendritic cells_10
10x_1976_GACGGCACTGCATG-1 Patient1 Monocytes nonactive 3 0.05448291
Monocytes_3
10x_1976_GACTCCTGTAGACC-1 Patient1 Dendritic cells nonactive 10 0.07900577
Dendritic cells_10
10x_1976_GACTCCTGTTCTCA-1 Patient1 Dendritic cells nonactive 10 0.06835331
Dendritic cells_10

10x_1976_GACTGAACCGAATC-1 Patient1 Dendritic cells nonactive 10 0.07898992
Dendritic cells_10
10x_1976_GAGAAATGTGCTTT-1 Patient1 Progenitors active 1 0.1344715
Progenitors_1
10x_1976_GAGATGCTTCCCGT-1 Patient1 Monocytes nonactive 3 0.01375943
Monocytes_3
10x_1976_GAGCAGGATTTACC-1 Patient1 Monocytes nonactive 3 0.04135756
Monocytes_3
10x_1976_GAGGACGATACGAC-1 Patient1 Progenitors nonactive 1 0.08046414
Progenitors_1
10x_1976_GAGGCAGATGCATG-1 Patient1 Monocytes nonactive 3 0.04159533
Monocytes_3
10x_1976_GAGGCCACGTACAC-1 Patient1 Dendritic cells nonactive 10 0.04429015
Dendritic cells_10
10x_1976_GAGGGCCTTGGCAT-1 Patient1 Dendritic cells nonactive 10 0.09509543
Dendritic cells_10
10x_1976_GAGGTACTGGAAGC-1 Patient1 Progenitors nonactive 12 0.10473337
Progenitors_12
10x_1976_GAGGTGGAAATCGC-1 Patient1 Dendritic cells nonactive 10 0.07608902
Dendritic cells_10
10x_1976_GAGTACTGCATGAC-1 Patient1 Dendritic cells nonactive 10 0.05919092
Dendritic cells_10
10x_1976_GATACTCTCACTTT-1 Patient1 Monocytes nonactive 3 0.05123328
Monocytes_3
10x_1976_GATAGCACCTGAAC-1 Patient1 Monocytes nonactive 3 0.08352356
Monocytes_3
10x_1976_GATATATGAGAACA-1 Patient1 Dendritic cells nonactive 10 0.10519308
Dendritic cells_10
10x_1976_GATCGTGAAGTTCG-1 Patient1 Monocytes nonactive 3 0.04926764
Monocytes_3
10x_1976_GATCTTTGATTCTC-1 Patient1 Dendritic cells nonactive 10 0.10871219
Dendritic cells_10
10x_1976_GATGCAACGTACCA-1 Patient1 Dendritic cells nonactive 10 0.05351595

Dendritic cells_10
10x_1976_GATGCATGCCTGTC-1 Patient1 Progenitors active 12 0.13845032
Progenitors_12
10x_1976_GATTTGCTCCTACC-1 Patient1 Monocytes nonactive 4 0.02560079
Monocytes_4
10x_1976_GCACAAACACTGGT-1 Patient1 Monocytes nonactive 13 0.04104052
Monocytes_13
10x_1976_GCACCTACACCATG-1 Patient1 Dendritic cells nonactive 10 0.05976159
Dendritic cells_10
10x_1976_GCAGCCGAAAAGCA-1 Patient1 Dendritic cells nonactive 10 0.07930696
Dendritic cells_10
10x_1976_GCAGCCGACCGCTT-1 Patient1 Dendritic cells nonactive 10 0.10226048
Dendritic cells_10
10x_1976_GCAGGCACTCTAGG-1 Patient1 Monocytes nonactive 3 0.03400228
Monocytes_3
10x_1976_GCAGGGCTAAAACG-1 Patient1 Dendritic cells nonactive 10 0.06952635
Dendritic cells_10
10x_1976_GCAGTTGACCCACT-1 Patient1 Dendritic cells nonactive 10 0.08628178
Dendritic cells_10
10x_1976_GCATCAGATAGTCG-1 Patient1 Dendritic cells nonactive 10 0.03753725
Dendritic cells_10
10x_1976_GCATTGGACTTAGG-1 Patient1 Monocytes nonactive 16 0.00499334
Monocytes_16
10x_1976_GCCACGGACGCTAA-1 Patient1 Dendritic cells nonactive 10 0.08320652
Dendritic cells_10
10x_1976_GCCCAGGATTATCC-1 Patient1 Progenitors nonactive 5 0.05597299
Progenitors_5
10x_1976_GCCCATACGGATTC-1 Patient1 Monocytes nonactive 3 0.0674656
Monocytes_3
10x_1976_GCCGACGAAACCGT-1 Patient1 Monocytes nonactive 3 0.06545241
Monocytes_3
10x_1976_GCCGACGAGAAAGT-1 Patient1 Monocytes nonactive 3 0.03263902
Monocytes_3

10x_1976_GCCGACGAGCGTAT-1 Patient1 Monocytes nonactive 3 0.02777249 Monocytes_3
10x_1976_GCCGACGAGTAAAG-1 Patient1 Dendritic cells nonactive 10 0.09439795 Dendritic cells_10
10x_1976_GCCTGACTCGACAT-1 Patient1 Dendritic cells nonactive 10 0.06058589 Dendritic cells_10
10x_1976_GCGAGCACACGTAC-1 Patient1 Dendritic cells nonactive 10 0.03831399 Dendritic cells_10
10x_1976_GCGAGCACTTGCAG-1 Patient1 Progenitors active 5 0.12289963 Progenitors_5
10x_1976_GCGCGAACGTCGAT-1 Patient1 Dendritic cells nonactive 10 0.08236637 Dendritic cells_10
10x_1976_GCGTAAACTTACTC-1 Patient1 Dendritic cells active 10 0.13207786 Dendritic cells_10
10x_1976_GCGTATGAAAGAAC-1 Patient1 Monocytes nonactive 3 0.09160801 Monocytes_3
10x_1976_GCTATACTCGTTGA-1 Patient1 Monocytes nonactive 3 0.061109 Monocytes_3
10x_1976_GCTCGACTCTGTGA-1 Patient1 Dendritic cells nonactive 10 0.07490013 Dendritic cells_10
10x_1976_GGAAGGTGCATCAG-1 Patient1 Monocytes nonactive 3 0.05540232 Monocytes_3
10x_1976_GGACCTCTGCATAC-1 Patient1 Dendritic cells nonactive 10 0.10695263 Dendritic cells_10
10x_1976_GGACGAGACTCATT-1 Patient1 Dendritic cells nonactive 14 0.11034494 Dendritic cells_14
10x_1976_GGACGCTGGTCAAC-1 Patient1 Monocytes nonactive 3 0.03539725 Monocytes_3
10x_1976_GGAGCCACCCTTTA-1 Patient1 Dendritic cells nonactive 10 0.09081542 Dendritic cells_10
10x_1976_GGAGGATGGGACAG-1 Patient1 Dendritic cells nonactive 10 0.0472386 Dendritic cells_10
10x_1976_GGATAGCTTACTCT-1 Patient1 Dendritic cells nonactive 10 0.08957897

Dendritic cells_10
10x_1976_GGATGTTGACACCA-1 Patient1 Monocytes nonactive 3 0.04333904
Monocytes_3
10x_1976_GGATTGTGTAAGGA-1 Patient1 Dendritic cells nonactive 10 0.09206772
Dendritic cells_10
10x_1976_GGATTTCTCTGTTT-1 Patient1 Monocytes nonactive 3 0.02518864
Monocytes_3
10x_1976_GGCCGATGGGTAAA-1 Patient1 Monocytes nonactive 3 0.02683723
Monocytes_3
10x_1976_GGCGCATGCCTACC-1 Patient1 Monocytes nonactive 3 0.0455266
Monocytes_3
10x_1976_GGCGGACTCTACTT-1 Patient1 Dendritic cells nonactive 10 0.11264346
Dendritic cells_10
10x_1976_GGGAACGAAGCTCA-1 Patient1 Dendritic cells nonactive 10 0.09625262
Dendritic cells_10
10x_1976_GGGATTACTGGATC-1 Patient1 T cells active 9 0.11880984 T cells_9
10x_1976_GGTACTGAATTCTC-1 Patient1 Dendritic cells nonactive 10 0.09934373
Dendritic cells_10
10x_1976_GGTATGACACGACT-1 Patient1 Monocytes nonactive 3 0.11633695
Monocytes_3
10x_1976_GGTTGAACCTCAGA-1 Patient1 Dendritic cells nonactive 10 0.04468645
Dendritic cells_10
10x_1976_GTAATATGGTTACG-1 Patient1 Monocytes nonactive 3 0.0442426
Monocytes_3
10x_1976_GTACGTGAGGTTAC-1 Patient1 Dendritic cells nonactive 10 0.09869381
Dendritic cells_10
10x_1976_GTAGCATGTCTAGG-1 Patient1 Monocytes nonactive 3 0.05833492
Monocytes_3
10x_1976_GTAGTGACAGTGCT-1 Patient1 Dendritic cells nonactive 10 0.09100564
Dendritic cells_10
10x_1976_GTAGTGTGATCTCT-1 Patient1 Monocytes nonactive 3 0.05384884
Monocytes_3
10x_1976_GTATCTACTCAAGC-1 Patient1 Dendritic cells nonactive 10 0.06435863

Dendritic cells_10
10x_1976_GTATGGTGGTTACG-1 Patient1 Dendritic cells nonactive 10 0.09222624
Dendritic cells_10
10x_1976_GTCAATCTTGAGAA-1 Patient1 Dendritic cells nonactive 10 0.07724621
Dendritic cells_10
10x_1976_GTCACCTGTCTTCA-1 Patient1 Monocytes nonactive 8 0.08366622
Monocytes_8
10x_1976_GTCGCACTCTTGAG-1 Patient1 Monocytes nonactive 3 0.03886881
Monocytes_3
10x_1976_GTGTATCTAGGAGC-1 Patient1 Monocytes nonactive 0 0.01764314
Monocytes_0
10x_1976_GTGTATCTGTAAGA-1 Patient1 Monocytes nonactive 3 0.04822142
Monocytes_3
10x_1976_GTTCATACAAGAAC-1 Patient1 Dendritic cells nonactive 10 0.03515947
Dendritic cells_10
10x_1976_GTTGATCTTGGATC-1 Patient1 Dendritic cells nonactive 10 0.07640606
Dendritic cells_10
10x_1976_TAACCGGAAGAGAT-1 Patient1 Dendritic cells nonactive 10 0.05091624
Dendritic cells_10
10x_1976_TAAGATACTGTGCA-1 Patient1 Dendritic cells nonactive 10 0.08073363
Dendritic cells_10
10x_1976_TAAGGCTGGTATGC-1 Patient1 Dendritic cells nonactive 10 0.081764
Dendritic cells_10
10x_1976_TAAGTCCTAGGTCT-1 Patient1 Monocytes nonactive 3 0.05522795
Monocytes_3
10x_1976_TAATCGCTGACAAA-1 Patient1 Dendritic cells nonactive 10 0.07242724
Dendritic cells_10
10x_1976_TAATGTGAGGTCTA-1 Patient1 Dendritic cells active 10 0.11936466
Dendritic cells_10
10x_1976_TACAATGATAGAAG-1 Patient1 Dendritic cells nonactive 10 0.05051994
Dendritic cells_10
10x_1976_TACGACGAGGGACA-1 Patient1 Dendritic cells nonactive 10 0.05462558
Dendritic cells_10

10x_1976_TACGAGTGCGGAGA-1 Patient1 Monocytes nonactive 3 0.08166889
Monocytes_3
10x_1976_TACGGCCTCCTGTC-1 Patient1 Progenitors nonactive 6 0.09027646
Progenitors_6
10x_1976_TACGTACTGTCTGA-1 Patient1 Dendritic cells nonactive 10 0.07870458
Dendritic cells_10
10x_1976_TACTTTCTTCACGA-1 Patient1 Dendritic cells nonactive 10 0.06816308
Dendritic cells_10
10x_1976_TAGAAACTAACTGC-1 Patient1 Monocytes nonactive 3 0.06672056
Monocytes_3
10x_1976_TAGAATTGGTTAGC-1 Patient1 Monocytes nonactive 2 0.0476349
Monocytes_2
10x_1976_TAGACGTGGACGAG-1 Patient1 Dendritic cells nonactive 10 0.05440365
Dendritic cells_10
10x_1976_TAGAGAGAGCTGAT-1 Patient1 Monocytes nonactive 3 0.01507514
Monocytes_3
10x_1976_TAGCCCACCTCGAA-1 Patient1 Dendritic cells nonactive 10 0.07008116
Dendritic cells_10
10x_1976_TAGCCGCTCCTCAC-1 Patient1 Monocytes nonactive 3 0.05972988
Monocytes_3
10x_1976_TAGGCAACACCTTT-1 Patient1 Dendritic cells nonactive 10 0.05798618
Dendritic cells_10
10x_1976_TAGGGACTTGGTTG-1 Patient1 Dendritic cells nonactive 10 0.11608332
Dendritic cells_10
10x_1976_TAGTACCTCCAATG-1 Patient1 Monocytes nonactive 3 0.04484497
Monocytes_3
10x_1976_TAGTGGTGTTGCGA-1 Patient1 Dendritic cells nonactive 10 0.05281846
Dendritic cells_10
10x_1976_TATCAGCTAAAAGC-1 Patient1 Monocytes nonactive 8 0.06405745
Monocytes_8
10x_1976_TATCCAACAGCACT-1 Patient1 Monocytes nonactive 13 0.04636675
Monocytes_13
10x_1976_TATCCAACAGGTTC-1 Patient1 Dendritic cells active 10 0.12641874

Dendritic cells_10
10x_1976_TATCTCGATCGTGA-1 Patient1 Dendritic cells nonactive 10 0.09633187
Dendritic cells_10
10x_1976_TATCTTCTCACTTT-1 Patient1 Monocytes nonactive 3 0.0349851
Monocytes_3
10x_1976_TATGGGACCAAGCT-1 Patient1 Monocytes nonactive 3 0.04360852
Monocytes_3
10x_1976_TATGTGCTTTACCT-1 Patient1 Monocytes nonactive 3 0.06435863
Monocytes_3
10x_1976_TATTGCTGGTTACG-1 Patient1 Monocytes nonactive 3 0.04146852
Monocytes_3
10x_1976_TCAATAGATGGAAA-1 Patient1 Dendritic cells nonactive 10 0.06442204
Dendritic cells_10
10x_1976_TCACAACTGTATGC-1 Patient1 Monocytes nonactive 4 0.06290026
Monocytes_4
10x_1976_TCACCGTGGAACTC-1 Patient1 Dendritic cells nonactive 10 0.08865957
Dendritic cells_10
10x_1976_TCAGGATGCGCTAA-1 Patient1 Monocytes nonactive 3 0.03671295
Monocytes_3
10x_1976_TCCGAAGACTCAAG-1 Patient1 Monocytes nonactive 3 0.05275506
Monocytes_3
10x_1976_TCGACCTGTAGCGT-1 Patient1 Monocytes nonactive 4 0.08192252
Monocytes_4
10x_1976_TCGACGCTCCTGTC-1 Patient1 Monocytes nonactive 0 0.03710925
Monocytes_0
10x_1976_TCGAGAACGGTCAT-1 Patient1 Monocytes nonactive 3 0.06109315
Monocytes_3
10x_1976_TCGGACCTTGGTAC-1 Patient1 Monocytes nonactive 3 0.02219263
Monocytes_3
10x_1976_TCTAACTGGTAAAG-1 Patient1 Monocytes nonactive 3 0.04029548
Monocytes_3
10x_1976_TCTCCACTTGCCCT-1 Patient1 Dendritic cells nonactive 10 0.07109568
Dendritic cells_10

10x_1976_TCTCTAGACGATAC-1 Patient1 Dendritic cells nonactive 10 0.09217868 Dendritic cells_10
10x_1976_TCTTCAGAACCTTT-1 Patient1 Monocytes nonactive 3 0.06529389 Monocytes_3
10x_1976_TGAAGCACGTGCAT-1 Patient1 Dendritic cells nonactive 10 0.09305054 Dendritic cells_10
10x_1976_TGACCGCTGTGTCA-1 Patient1 Monocytes nonactive 8 0.05156617 Monocytes_8
10x_1976_TGACGATGTAAGCC-1 Patient1 Monocytes nonactive 15 0.06909835 Monocytes_15
10x_1976_TGACTTACGCTACA-1 Patient1 Monocytes nonactive 15 0.06030055 Monocytes_15
10x_1976_TGAGCAACTAGCCA-1 Patient1 Dendritic cells nonactive 10 0.0640733 Dendritic cells_10
10x_1976_TGAGGACTTGACCA-1 Patient1 Monocytes nonactive 15 0.03127576 Monocytes_15
10x_1976_TGAGTCGAGACTAC-1 Patient1 Dendritic cells nonactive 10 0.06733879 Dendritic cells_10
10x_1976_TGATTAGAAGTGTC-1 Patient1 Dendritic cells nonactive 10 0.07581954 Dendritic cells_10
10x_1976_TGATTCTGCCTAAG-1 Patient1 Monocytes nonactive 4 0.05976159 Monocytes_4
10x_1976_TGCAACGATAGAAG-1 Patient1 Monocytes nonactive 3 0.05583032 Monocytes_3
10x_1976_TGCACGCTTGGTCA-1 Patient1 Monocytes nonactive 3 0.06288441 Monocytes_3
10x_1976_TGCGAAACATTCCT-1 Patient1 Monocytes nonactive 3 0.03152939 Monocytes_3
10x_1976_TGCGTAGACGTGTA-1 Patient1 Dendritic cells nonactive 10 0.09133853 Dendritic cells_10
10x_1976_TGGAACACTCTAGG-1 Patient1 Progenitors nonactive 1 0.10817323 Progenitors_1
10x_1976_TGGCACCTGGATTC-1 Patient1 Dendritic cells nonactive 10 0.07937036

Dendritic cells_10

10x_1976_TGTATCTGGGTTAC-1 Patient1 Dendritic cells nonactive 14 0.0935895 Dendritic cells_14

10x_1976_TGTCTAACGAGAGC-1 Patient1 Dendritic cells nonactive 10 0.07535984 Dendritic cells_10

10x_1976_TTACGTACAGTAGA-1 Patient1 Dendritic cells nonactive 10 0.09049838 Dendritic cells_10

10x_1976_TTAGGGACGGCGAA-1 Patient1 Progenitors nonactive 1 0.08596475 Progenitors_1

10x_1976_TTAGTCTGGGCAAG-1 Patient1 Dendritic cells nonactive 10 0.05977744 Dendritic cells_10

10x_1976_TTCATGACTCGCTC-1 Patient1 Monocytes nonactive 3 0.06144189 Monocytes_3

10x_1976_TTCCAAACAGCCTA-1 Patient1 Monocytes nonactive 3 0.03618984 Monocytes_3

10x_1976_TTCTACGACATGAC-1 Patient1 Monocytes nonactive 3 0.04398897 Monocytes_3

10x_1976_TTCTCAGAGCCATA-1 Patient1 Dendritic cells nonactive 10 0.08173229 Dendritic cells_10

10x_1976_TTGAATGATTTGTC-1 Patient1 Monocytes nonactive 3 0.04929935 Monocytes_3

10x_1976_TTGAGGACCTGCAA-1 Patient1 Progenitors active 1 0.14078055 Progenitors_1

10x_1976_TTTCAGTGCATTGG-1 Patient1 Monocytes nonactive 8 0.04525712 Monocytes_8

10x_3290_AAACGCTGTTCTAC-1 Patient6 Monocytes nonactive 2 0.02560079 Monocytes_2

10x_3290_AAACTTGACGCCTT-1 Patient6 Monocytes nonactive 3 0.04627164 Monocytes_3

10x_3290_AAAGACGATTCACT-1 Patient6 Monocytes nonactive 3 0.04947372 Monocytes_3

10x_3290_AAAGCAGATGACAC-1 Patient6 Monocytes nonactive 3 0.04270496 Monocytes_3

10x_3290_AAAGTTTGCCTGAA-1 Patient6 Monocytes nonactive 2 0.0459229
Monocytes_2
10x_3290_AAAGTTTGGTCGTA-1 Patient6 Monocytes nonactive 3 0.05292943
Monocytes_3
10x_3290_AAATCAACGTCGTA-1 Patient6 Monocytes nonactive 3 0.0569558
Monocytes_3
10x_3290_AAATCCCTACTACG-1 Patient6 Monocytes nonactive 3 0.02258893
Monocytes_3
10x_3290_AAATCTGAACCTGA-1 Patient6 Progenitors active 6 0.16099169
Progenitors_6
10x_3290_AAATCTGATCGATG-1 Patient6 Monocytes nonactive 3 0.05827151
Monocytes_3
10x_3290_AAATGTTGCTCTAT-1 Patient6 Monocytes nonactive 3 0.03398643
Monocytes_3
10x_3290_AAATTGACTCTTAC-1 Patient6 Monocytes nonactive 2 0.02284256
Monocytes_2
10x_3290_AACAAACTGCTCCT-1 Patient6 Monocytes nonactive 3 0.04042229
Monocytes_3
10x_3290_AACACGTGACTGGT-1 Patient6 Monocytes nonactive 3 0.0316245
Monocytes_3
10x_3290_AACAGCACTGCTAG-1 Patient6 Monocytes nonactive 2 0.0467789
Monocytes_2
10x_3290_AACATATGAGCTCA-1 Patient6 Monocytes nonactive 2 0.06006277
Monocytes_2
10x_3290_AACATTGACGAGAG-1 Patient6 Monocytes nonactive 3 0.02682138
Monocytes_3
10x_3290_AACATTGAGGTGAG-1 Patient6 Monocytes nonactive 2 0.0168981
Monocytes_2
10x_3290_AACCACGATCTTCA-1 Patient6 Monocytes nonactive 2 0.05205757
Monocytes_2
10x_3290_AACCACGATGTGGT-1 Patient6 Monocytes nonactive 2 0.02384123
Monocytes_2
10x_3290_AACCAGTGGGAGTG-1 Patient6 Monocytes nonactive 2 0.06768753

Monocytes_2
10x_3290_AACCGCCTGGAAAT-1 Patient6 T cells nonactive 9 0.06599138 T cells_9
10x_3290_AACCGCCTGTTGTG-1 Patient6 Monocytes nonactive 3 0.05162957 Monocytes_3
10x_3290_AACCTACTGTTCGA-1 Patient6 Monocytes nonactive 2 0.02406315 Monocytes_2
10x_3290_AACCTTACCACACA-1 Patient6 Progenitors active 6 0.19642065 Progenitors_6
10x_3290_AACCTTACCACTTT-1 Patient6 Monocytes nonactive 2 0.0459863 Monocytes_2
10x_3290_AACCTTACGTCTGA-1 Patient6 Monocytes nonactive 2 0.04078689 Monocytes_2
10x_3290_AACGCAACCAGGAG-1 Patient6 Monocytes nonactive 2 0.07420265 Monocytes_2
10x_3290_AACGCAACCCGCTT-1 Patient6 Monocytes nonactive 2 0.01970389 Monocytes_2
10x_3290_AACGCAACTCTTTG-1 Patient6 Progenitors active 6 0.13998795 Progenitors_6
10x_3290_AACGCATGACGGGA-1 Patient6 Monocytes nonactive 2 0.0181187 Monocytes_2
10x_3290_AACGCCCTCGTCTC-1 Patient6 Monocytes nonactive 2 0.02235115 Monocytes_2
10x_3290_AACGGTACCCACCT-1 Patient6 Monocytes nonactive 3 0.06047492 Monocytes_3
10x_3290_AACGGTTGCCGCTT-1 Patient6 Monocytes nonactive 3 0.03099043 Monocytes_3
10x_3290_AACGGTTGTGGAGG-1 Patient6 Monocytes nonactive 2 0.04364023 Monocytes_2
10x_3290_AACGTCGATCGTTT-1 Patient6 Monocytes nonactive 2 0.01829307 Monocytes_2
10x_3290_AACGTTCTCAGCTA-1 Patient6 Progenitors active 6 0.13916365 Progenitors_6
10x_3290_AACTCGGAGTTGTG-1 Patient6 Progenitors active 6 0.12404096

Progenitors_6
10x_3290_AACTCGGATTCTTG-1 Patient6 Progenitors nonactive 6 0.11603576 Progenitors_6
10x_3290_AACTCTTGGTGTAC-1 Patient6 Monocytes nonactive 2 0.03929681 Monocytes_2
10x_3290_AACTGTCTCTTGGA-1 Patient6 Monocytes nonactive 2 0.02853338 Monocytes_2
10x_3290_AACTTGCTAAAAGC-1 Patient6 Monocytes nonactive 2 0.04628749 Monocytes_2
10x_3290_AACTTGCTATTCTC-1 Patient6 Monocytes nonactive 3 0.04240378 Monocytes_3
10x_3290_AAGACAGACTCGAA-1 Patient6 T cells nonactive 9 0.0742185 T cells_9
10x_3290_AAGAGATGCTATGG-1 Patient6 Monocytes nonactive 2 0.03157695 Monocytes_2
10x_3290_AAGATTACATGTCG-1 Patient6 Progenitors nonactive 6 0.0893729 Progenitors_6
10x_3290_AAGATTACGGACGA-1 Patient6 Progenitors nonactive 6 0.08911927 Progenitors_6
10x_3290_AAGATTACTAGAGA-1 Patient6 Monocytes nonactive 2 0.03414495 Monocytes_2
10x_3290_AAGCCAACCACTAG-1 Patient6 Monocytes nonactive 2 0.03909074 Monocytes_2
10x_3290_AAGGTCTGAGCTAC-1 Patient6 Monocytes nonactive 3 0.04896646 Monocytes_3
10x_3290_AAGGTGCTAATCGC-1 Patient6 Monocytes nonactive 3 0.06104559 Monocytes_3
10x_3290_AAGTAACTCCGCTT-1 Patient6 Monocytes nonactive 3 0.04665208 Monocytes_3
10x_3290_AAGTATACCCTTCG-1 Patient6 Monocytes nonactive 2 0.07948133 Monocytes_2
10x_3290_AAGTATACTAAGCC-1 Patient6 Progenitors active 6 0.13873565 Progenitors_6
10x_3290_AAGTCCGACCTGAA-1 Patient6 Progenitors active 6 0.1698846

Progenitors_6

10x_3290_AAGTCCGAGCTACA-1 Patient6 Monocytes nonactive 3 0.06377211 Monocytes_3

10x_3290_AAGTCCGAGGAGTG-1 Patient6 Progenitors active 6 0.2103703 Progenitors_6

10x_3290_AAGTCCGATGCTTT-1 Patient6 Monocytes nonactive 2 0.02872361 Monocytes_2

10x_3290_AAGTCTCTGCGAAG-1 Patient6 B cells nonactive 7 0.03850422 B cells_7

10x_3290_AAGTGGCTGACGGA-1 Patient6 Monocytes nonactive 2 0.05844588 Monocytes_2

10x_3290_AAGTTATGCTCGAA-1 Patient6 Monocytes nonactive 2 0.07285524 Monocytes_2

10x_3290_AAGTTATGGCAGAG-1 Patient6 Monocytes nonactive 2 0.02395219 Monocytes_2

10x_3290_AAGTTATGTATCGG-1 Patient6 Monocytes nonactive 3 0.04966394 Monocytes_3

10x_3290_AAGTTCCTCAACTG-1 Patient6 Monocytes nonactive 3 0.05044068 Monocytes_3

10x_3290_AATAAGCTCCTTAT-1 Patient6 Monocytes nonactive 2 0.03783844 Monocytes_2

10x_3290_AATAAGCTGACAGG-1 Patient6 Monocytes nonactive 3 0.05679729 Monocytes_3

10x_3290_AATAAGCTGTGCAT-1 Patient6 Monocytes nonactive 2 0.02617145 Monocytes_2

10x_3290_AATAAGCTTGTCGA-1 Patient6 Monocytes nonactive 3 0.02969057 Monocytes_3

10x_3290_AATACCCTGCTATG-1 Patient6 Progenitors active 6 0.16408281 Progenitors_6

10x_3290_AATACTGAAGGAGC-1 Patient6 Monocytes nonactive 3 0.08710608 Monocytes_3

10x_3290_AATACTGATTGGTG-1 Patient6 Progenitors nonactive 6 0.10137277 Progenitors_6

10x_3290_AATATCGAGTCTGA-1 Patient6 Monocytes nonactive 2 0.03192569

Monocytes_2

10x_3290_AATCCTACTCCAGA-1 Patient6 Monocytes nonactive 3 0.03221102

Monocytes_3

10x_3290_AATCTAGAGGACAG-1 Patient6 Progenitors active 6 0.13761017

Progenitors_6

10x_3290_AATGCGTGCTTCGC-1 Patient6 Monocytes nonactive 2 0.06511952

Monocytes_2

10x_3290_AATGCGTGTCGTGA-1 Patient6 Progenitors active 6 0.16673007

Progenitors_6

10x_3290_AATGGAGAAGAGTA-1 Patient6 Monocytes nonactive 2 0.0244753

Monocytes_2

10x_3290_AATGGAGATTTACC-1 Patient6 Monocytes nonactive 2 0.02639338

Monocytes_2

10x_3290_AATGGCTGGTGTCA-1 Patient6 Monocytes nonactive 2 0.05446706

Monocytes_2

10x_3290_AATGTAACCTGAGT-1 Patient6 Monocytes nonactive 3 0.06079196

Monocytes_3

10x_3290_AATGTTGAACGACT-1 Patient6 Monocytes nonactive 2 0.06464397

Monocytes_2

10x_3290_AATTACGATCATTC-1 Patient6 Monocytes nonactive 3 0.0190064

Monocytes_3

10x_3290_AATTCCTGGACGTT-1 Patient6 Monocytes nonactive 3 0.05107476

Monocytes_3

10x_3290_AATTGTGAGGGATG-1 Patient6 Monocytes nonactive 2 0.06415256

Monocytes_2

10x_3290_ACAAAGGAGCTCCT-1 Patient6 Monocytes nonactive 3 0.07580369

Monocytes_3

10x_3290_ACAAAGGATGGAGG-1 Patient6 Monocytes nonactive 2 0.02149515

Monocytes_2

10x_3290_ACAAATTGAGCGGA-1 Patient6 Progenitors active 6 0.14032084

Progenitors_6

10x_3290_ACAACCGAACACCA-1 Patient6 Monocytes nonactive 2 0.0282639

Monocytes_2

10x_3290_ACAAGAGATCCTTA-1 Patient6 Monocytes nonactive 2 0.0480946
Monocytes_2
10x_3290_ACAAGCACTCCGAA-1 Patient6 Monocytes nonactive 2 0.03443028
Monocytes_2
10x_3290_ACAATCCTCGGTAT-1 Patient6 Monocytes nonactive 2 0.03512777
Monocytes_2
10x_3290_ACACAGACCGACAT-1 Patient6 Progenitors active 6 0.13770528
Progenitors_6
10x_3290_ACACATCTACGGGA-1 Patient6 B cells nonactive 7 0.08610741 B cells_7
10x_3290_ACACCCTGACACCA-1 Patient6 Monocytes nonactive 3 0.04536808
Monocytes_3
10x_3290_ACACCCTGTCGCAA-1 Patient6 Progenitors active 6 0.15989791
Progenitors_6
10x_3290_ACACCCTGTCTTAC-1 Patient6 Monocytes nonactive 2 0.03060998
Monocytes_2
10x_3290_ACACGATGGAATAG-1 Patient6 Monocytes nonactive 2 0.01474225
Monocytes_2
10x_3290_ACAGACACGGTAGG-1 Patient6 Monocytes nonactive 3 0.03660199
Monocytes_3
10x_3290_ACAGCAACCTCGAA-1 Patient6 Progenitors active 6 0.1732769
Progenitors_6
10x_3290_ACAGCAACGTGTCA-1 Patient6 Monocytes nonactive 3 0.03179887
Monocytes_3
10x_3290_ACAGTTCTACCTCC-1 Patient6 Monocytes nonactive 3 0.03561917
Monocytes_3
10x_3290_ACATGGTGAGGCGA-1 Patient6 Monocytes nonactive 3 0.0518832
Monocytes_3
10x_3290_ACCAACGATCAGGT-1 Patient6 Monocytes nonactive 2 0.03146598
Monocytes_2
10x_3290_ACCAGCCTCTCGAA-1 Patient6 Monocytes nonactive 3 0.02938939
Monocytes_3
10x_3290_ACCAGTGAGAACTC-1 Patient6 Monocytes nonactive 2 0.01065246
Monocytes_2

10x_3290_ACCCACTGACCAGT-1 Patient6 Monocytes nonactive 2 0.04568512 Monocytes_2
10x_3290_ACCCACTGACGACT-1 Patient6 Monocytes nonactive 2 0.07742058 Monocytes_2
10x_3290_ACCCACTGCCAATG-1 Patient6 Monocytes nonactive 2 0.019799 Monocytes_2
10x_3290_ACCCAGCTCCTTTA-1 Patient6 Monocytes nonactive 2 0.01941855 Monocytes_2
10x_3290_ACCGCGGAGGACTT-1 Patient6 Monocytes nonactive 3 0.06123581 Monocytes_3
10x_3290_ACCGCGGAGGTAAA-1 Patient6 Monocytes nonactive 3 0.03365354 Monocytes_3
10x_3290_ACCGTGCTTTTCGT-1 Patient6 Progenitors active 6 0.18640226 Progenitors_6
10x_3290_ACCTCGTGGCTGAT-1 Patient6 Monocytes nonactive 3 0.03270243 Monocytes_3
10x_3290_ACCTTTGACAAGCT-1 Patient6 Monocytes nonactive 2 0.03057828 Monocytes_2
10x_3290_ACCTTTGACTACTT-1 Patient6 Monocytes nonactive 2 0.03690318 Monocytes_2
10x_3290_ACGAACACGAGGTG-1 Patient6 Monocytes nonactive 3 0.03607888 Monocytes_3
10x_3290_ACGAAGCTTTCTGT-1 Patient6 Monocytes nonactive 2 0.02713842 Monocytes_2
10x_3290_ACGACAACCACCAA-1 Patient6 Monocytes nonactive 3 0.05116987 Monocytes_3
10x_3290_ACGACAACCACTAG-1 Patient6 Monocytes nonactive 3 0.02231945 Monocytes_3
10x_3290_ACGACAACCTGAGT-1 Patient6 Monocytes nonactive 3 0.03352673 Monocytes_3
10x_3290_ACGACCCTAGAATG-1 Patient6 Monocytes nonactive 3 0.04525712 Monocytes_3
10x_3290_ACGACCCTAGGAGC-1 Patient6 Monocytes nonactive 2 0.0371251

Monocytes_2
10x_3290_ACGAGTACTACGCA-1 Patient6 Monocytes nonactive 2 0.02041722 Monocytes_2
10x_3290_ACGAGTACTGTTTC-1 Patient6 Progenitors active 6 0.13667491 Progenitors_6
10x_3290_ACGATTCTGAATAG-1 Patient6 Monocytes nonactive 3 0.03875785 Monocytes_3
10x_3290_ACGCAATGGCTACA-1 Patient6 Monocytes nonactive 2 0.04685816 Monocytes_2
10x_3290_ACGCACCTGGACTT-1 Patient6 Monocytes nonactive 2 0.04118318 Monocytes_2
10x_3290_ACGCACCTGTGCAT-1 Patient6 Monocytes nonactive 2 0.02759812 Monocytes_2
10x_3290_ACGCACCTTAAGGA-1 Patient6 Progenitors active 6 0.18654492 Progenitors_6
10x_3290_ACGCCGGAACCCTC-1 Patient6 Monocytes nonactive 2 0.02964302 Monocytes_2
10x_3290_ACGCCGGACGAACT-1 Patient6 Monocytes nonactive 2 0.01426669 Monocytes_2
10x_3290_ACGCCGGACTGCTC-1 Patient6 Monocytes nonactive 2 0.02132078 Monocytes_2
10x_3290_ACGCTGCTTGTCCC-1 Patient6 Monocytes nonactive 3 0.03574599 Monocytes_3
10x_3290_ACGGATTGTTGACG-1 Patient6 Monocytes nonactive 2 0.04061252 Monocytes_2
10x_3290_ACGGCGTGCCTAAG-1 Patient6 Monocytes nonactive 2 0.0223353 Monocytes_2
10x_3290_ACGGTAACTCGTTT-1 Patient6 Monocytes nonactive 2 0.03641177 Monocytes_2
10x_3290_ACGGTATGCGAACT-1 Patient6 Monocytes nonactive 3 0.03775918 Monocytes_3
10x_3290_ACGGTCCTATCGGT-1 Patient6 Monocytes nonactive 2 0.03817133 Monocytes_2

10x_3290_ACGTCCTGTGTTCT-1 Patient6 Monocytes nonactive 2 0.01326802 Monocytes_2
10x_3290_ACGTCGCTGAATCC-1 Patient6 Progenitors active 6 0.14225477 Progenitors_6
10x_3290_ACGTCGCTGTGCTA-1 Patient6 Monocytes nonactive 3 0.04102467 Monocytes_3
10x_3290_ACGTGCCTCATTGG-1 Patient6 Monocytes nonactive 2 0.02793101 Monocytes_2
10x_3290_ACGTTGGAGCGTAT-1 Patient6 Monocytes nonactive 3 0.05258069 Monocytes_3
10x_3290_ACGTTTACGTGCAT-1 Patient6 Monocytes nonactive 3 0.06928857 Monocytes_3
10x_3290_ACTAAAACACCTGA-1 Patient6 Monocytes nonactive 2 0.03246465 Monocytes_2
10x_3290_ACTAAAACCGCCTT-1 Patient6 Monocytes nonactive 3 0.03891637 Monocytes_3
10x_3290_ACTACGGAGACTAC-1 Patient6 Progenitors active 6 0.15089405 Progenitors_6
10x_3290_ACTAGGTGGATAAG-1 Patient6 Monocytes nonactive 2 0.02672627 Monocytes_2
10x_3290_ACTAGGTGTCTCAT-1 Patient6 Progenitors nonactive 6 0.08986431 Progenitors_6
10x_3290_ACTCCTCTGCCATA-1 Patient6 Monocytes nonactive 2 0.05242217 Monocytes_2
10x_3290_ACTCTATGTTGAGC-1 Patient6 Monocytes nonactive 2 0.01800774 Monocytes_2
10x_3290_ACTGCCACTAAAGG-1 Patient6 Monocytes nonactive 2 0.04717519 Monocytes_2
10x_3290_ACTGCCACTGCCCT-1 Patient6 Monocytes nonactive 2 0.04771416 Monocytes_2
10x_3290_ACTGCCTGGTGTAC-1 Patient6 Monocytes nonactive 3 0.03522288 Monocytes_3
10x_3290_ACTGGCCTTTTGCT-1 Patient6 Monocytes nonactive 2 0.06294782

Monocytes_2
10x_3290_ACTGTGGAGCTACA-1 Patient6 Monocytes nonactive 3 0.04660453
Monocytes_3
10x_3290_ACTTAGCTACTCTT-1 Patient6 Monocytes nonactive 2 0.05562425
Monocytes_2
10x_3290_ACTTAGCTAGCGGA-1 Patient6 Monocytes nonactive 3 0.07467821
Monocytes_3
10x_3290_ACTTAGCTGTTGAC-1 Patient6 Monocytes nonactive 2 0.03100628
Monocytes_2
10x_3290_ACTTCTGACGGGAA-1 Patient6 Monocytes nonactive 3 0.06968486
Monocytes_3
10x_3290_ACTTGACTGCGTTA-1 Patient6 Progenitors active 6 0.15274872
Progenitors_6
10x_3290_ACTTTGTGATCTTC-1 Patient6 Progenitors nonactive 6 0.11099486
Progenitors_6
10x_3290_ACTTTGTGATGTCG-1 Patient6 Monocytes nonactive 2 0.05162957
Monocytes_2
10x_3290_ACTTTGTGGTGTAC-1 Patient6 Monocytes nonactive 3 0.05204172
Monocytes_3
10x_3290_AGAAACGAATGCTG-1 Patient6 Monocytes nonactive 3 0.07386976
Monocytes_3
10x_3290_AGAAAGTGTGACAC-1 Patient6 Monocytes nonactive 2 0.02842242
Monocytes_2
10x_3290_AGAAAGTGTTCTTG-1 Patient6 Progenitors active 6 0.11939636
Progenitors_6
10x_3290_AGAACAGAGCGGAA-1 Patient6 Progenitors active 6 0.17627291
Progenitors_6
10x_3290_AGAAGATGTTCAGG-1 Patient6 Monocytes nonactive 3 0.06763997
Monocytes_3
10x_3290_AGAATTTGGGTATC-1 Patient6 Progenitors active 6 0.1345032
Progenitors_6
10x_3290_AGACACACCCTCCA-1 Patient6 Monocytes nonactive 3 0.05407076
Monocytes_3

10x_3290_AGACACACGGTACT-1 Patient6 Monocytes nonactive 3 0.06400989 Monocytes_3
10x_3290_AGACACTGACTACG-1 Patient6 Monocytes nonactive 3 0.05529136 Monocytes_3
10x_3290_AGACGTACAGAACA-1 Patient6 Monocytes nonactive 2 0.05589373 Monocytes_2
10x_3290_AGACTGACCGAGTT-1 Patient6 Monocytes nonactive 2 0.09206772 Monocytes_2
10x_3290_AGACTTCTGAGCAG-1 Patient6 Monocytes nonactive 2 0.03476317 Monocytes_2
10x_3290_AGAGAAACACCACA-1 Patient6 Monocytes nonactive 2 0.02371441 Monocytes_2
10x_3290_AGAGAATGCTCCAC-1 Patient6 Monocytes nonactive 13 0.07261746 Monocytes_13
10x_3290_AGAGAATGTATGCG-1 Patient6 Monocytes nonactive 2 0.02038552 Monocytes_2
10x_3290_AGAGATGAAGAATG-1 Patient6 Monocytes nonactive 2 0.03636421 Monocytes_2
10x_3290_AGAGCTACGCGGAA-1 Patient6 Progenitors active 6 0.12675163 Progenitors_6
10x_3290_AGAGGTCTACGTAC-1 Patient6 Progenitors active 6 0.14670915 Progenitors_6
10x_3290_AGAGGTCTTTGTCT-1 Patient6 Progenitors nonactive 6 0.10966331 Progenitors_6
10x_3290_AGAGTCACCCTTAT-1 Patient6 Progenitors nonactive 6 0.1171771 Progenitors_6
10x_3290_AGAGTCACTCGTAG-1 Patient6 Progenitors active 6 0.13665906 Progenitors_6
10x_3290_AGAGTGCTCACAAC-1 Patient6 Monocytes nonactive 2 0.02699575 Monocytes_2
10x_3290_AGATATTGACACAC-1 Patient6 Monocytes nonactive 2 0.04831653 Monocytes_2
10x_3290_AGATTAACCGATAC-1 Patient6 Monocytes nonactive 3 0.07745229

Monocytes_3
10x_3290_AGATTAACGAGGTG-1 Patient6 Monocytes nonactive 3 0.04146852
Monocytes_3
10x_3290_AGATTCCTAACGAA-1 Patient6 Progenitors active 6 0.15549109
Progenitors_6
10x_3290_AGATTCCTTCCCAC-1 Patient6 Monocytes nonactive 3 0.0590324
Monocytes_3
10x_3290_AGCAAGCTACGCTA-1 Patient6 Monocytes nonactive 2 0.04157948
Monocytes_2
10x_3290_AGCACAACACCAGT-1 Patient6 Monocytes nonactive 2 0.04452793
Monocytes_2
10x_3290_AGCACAACCCTTGC-1 Patient6 Monocytes nonactive 3 0.04581193
Monocytes_3
10x_3290_AGCACAACGCTTCC-1 Patient6 Monocytes nonactive 2 0.03298776
Monocytes_2
10x_3290_AGCCAATGGTAGCT-1 Patient6 Monocytes nonactive 3 0.05686069
Monocytes_3
10x_3290_AGCCACCTGGTGGA-1 Patient6 Monocytes nonactive 2 0.01780166
Monocytes_2
10x_3290_AGCCGGTGGAGGGT-1 Patient6 Monocytes nonactive 2 0.05036142
Monocytes_2
10x_3290_AGCCGGTGTCGCAA-1 Patient6 Monocytes nonactive 3 0.04126244
Monocytes_3
10x_3290_AGCCGTCTTGTGAC-1 Patient6 Monocytes nonactive 2 0.05116987
Monocytes_2
10x_3290_AGCCGTCTTTTCGT-1 Patient6 Progenitors active 6 0.14845286
Progenitors_6
10x_3290_AGCCTCACTGTGGT-1 Patient6 Progenitors active 6 0.1517342
Progenitors_6
10x_3290_AGCGATACCTCCCA-1 Patient6 Monocytes nonactive 2 0.04080274
Monocytes_2
10x_3290_AGCGATTGCTTGGA-1 Patient6 Monocytes nonactive 2 0.0290882
Monocytes_2

10x_3290_AGCGATTGGTAAAG-1 Patient6 Progenitors active 6 0.13145964 Progenitors_6
10x_3290_AGCGATTGTTCCCG-1 Patient6 Monocytes nonactive 2 0.03519117 Monocytes_2
10x_3290_AGCGCTCTCTTCTA-1 Patient6 Progenitors active 6 0.15628369 Progenitors_6
10x_3290_AGCGGCACCAGAAA-1 Patient6 Progenitors active 6 0.12874897 Progenitors_6
10x_3290_AGCGGCACTTCTTG-1 Patient6 Monocytes nonactive 2 0.02894553 Monocytes_2
10x_3290_AGCGTAACTTACCT-1 Patient6 Monocytes nonactive 2 0.0349851 Monocytes_2
10x_3290_AGCTTACTATCGGT-1 Patient6 Monocytes nonactive 2 0.01288758 Monocytes_2
10x_3290_AGCTTTACACGCAT-1 Patient6 Monocytes nonactive 3 0.0480946 Monocytes_3
10x_3290_AGCTTTACTGTCAG-1 Patient6 Monocytes nonactive 2 0.00825883 Monocytes_2
10x_3290_AGGAAATGCCCAAA-1 Patient6 Monocytes nonactive 3 0.06022129 Monocytes_3
10x_3290_AGGACTTGGTCATG-1 Patient6 Progenitors active 6 0.11909517 Progenitors_6
10x_3290_AGGAGTCTACAGCT-1 Patient6 Monocytes nonactive 2 0.03688732 Monocytes_2
10x_3290_AGGCAACTAAACAG-1 Patient6 Monocytes nonactive 2 0.06255152 Monocytes_2
10x_3290_AGGCAACTCCGATA-1 Patient6 Progenitors active 6 0.14494959 Progenitors_6
10x_3290_AGGCCTCTGATACC-1 Patient6 Monocytes nonactive 2 0.03611058 Monocytes_2
10x_3290_AGGCCTCTGTGTAC-1 Patient6 Monocytes nonactive 2 0.04671549 Monocytes_2
10x_3290_AGGCTAACGTCGAT-1 Patient6 Progenitors active 1 0.19187116

Progenitors_1

10x_3290_AGGGACGATACGCA-1 Patient6 Progenitors active 6 0.18209055 Progenitors_6

10x_3290_AGGGAGTGCTGTTT-1 Patient6 Monocytes nonactive 3 0.04709594 Monocytes_3

10x_3290_AGGGAGTGGGAAGC-1 Patient6 Progenitors active 6 0.1416524 Progenitors_6

10x_3290_AGGGCCTGGTTAGC-1 Patient6 Monocytes nonactive 2 0.01891129 Monocytes_2

10x_3290_AGGTCTGATGGTCA-1 Patient6 Monocytes nonactive 3 0.04543149 Monocytes_3

10x_3290_AGGTGGGAAACAGA-1 Patient6 Progenitors active 6 0.13394839 Progenitors_6

10x_3290_AGGTTGTGGTACAC-1 Patient6 Monocytes nonactive 2 0.03573014 Monocytes_2

10x_3290_AGTACGTGCTTACT-1 Patient6 Monocytes nonactive 3 0.03314628 Monocytes_3

10x_3290_AGTACGTGGACAGG-1 Patient6 Monocytes nonactive 2 0.03782259 Monocytes_2

10x_3290_AGTAGAGAGCGAGA-1 Patient6 Monocytes nonactive 2 0.04001015 Monocytes_2

10x_3290_AGTAGAGAGGCATT-1 Patient6 Progenitors active 6 0.13405935 Progenitors_6

10x_3290_AGTAGAGATCTTAC-1 Patient6 Monocytes nonactive 2 0.05129668 Monocytes_2

10x_3290_AGTATCCTTTCCCG-1 Patient6 Progenitors active 6 0.15404857 Progenitors_6

10x_3290_AGTCACGATGAAGA-1 Patient6 Progenitors active 6 0.15999303 Progenitors_6

10x_3290_AGTCGCCTTCCAGA-1 Patient6 Monocytes nonactive 2 0.02923087 Monocytes_2

10x_3290_AGTGACTGACCCAA-1 Patient6 Monocytes nonactive 2 0.04730201 Monocytes_2

10x_3290_AGTGACTGGAAACA-1 Patient6 Monocytes nonactive 16 0.03896392 Monocytes_16
10x_3290_AGTGACTGTCACCC-1 Patient6 Monocytes nonactive 3 0.0502029 Monocytes_3
10x_3290_AGTTATGAATTGGC-1 Patient6 Monocytes nonactive 2 0.03874199 Monocytes_2
10x_3290_AGTTCTACTCTTCA-1 Patient6 T cells nonactive 9 0.05568765 T cells_9
10x_3290_AGTTCTTGTTGAGC-1 Patient6 Monocytes nonactive 2 0.07022383 Monocytes_2
10x_3290_AGTTGTCTTCGCTC-1 Patient6 Monocytes nonactive 2 0.08063851 Monocytes_2
10x_3290_ATAAGTTGTTCTCA-1 Patient6 Monocytes nonactive 2 0.01333143 Monocytes_2
10x_3290_ATAATGACGGAAGC-1 Patient6 Monocytes nonactive 3 0.03641177 Monocytes_3
10x_3290_ATAATGACTCTCGC-1 Patient6 T cells nonactive 9 0.04909327 T cells_9
10x_3290_ATACAATGAGCGGA-1 Patient6 Progenitors active 6 0.16210133 Progenitors_6
10x_3290_ATACAATGGTCGTA-1 Patient6 Monocytes nonactive 2 0.0269799 Monocytes_2
10x_3290_ATACCACTCAGAAA-1 Patient6 Progenitors active 6 0.12816245 Progenitors_6
10x_3290_ATACTCTGAGGCGA-1 Patient6 Monocytes nonactive 2 0.08716949 Monocytes_2
10x_3290_ATAGCCGACAGAAA-1 Patient6 Monocytes nonactive 2 0.06732293 Monocytes_2
10x_3290_ATAGCCGAGCAAGG-1 Patient6 Monocytes nonactive 3 0.03286095 Monocytes_3
10x_3290_ATAGCCGATAACCG-1 Patient6 Monocytes nonactive 2 0.04280008 Monocytes_2
10x_3290_ATAGCCGATCTACT-1 Patient6 Monocytes nonactive 3 0.03332065 Monocytes_3
10x_3290_ATAGTCCTACGGAG-1 Patient6 Monocytes nonactive 3 0.06301122

Monocytes_3
10x_3290_ATAGTCCTCTCAGA-1 Patient6 Monocytes nonactive 2 0.01935515
Monocytes_2
10x_3290_ATATACGAATTCTC-1 Patient6 Progenitors active 6 0.12546763
Progenitors_6
10x_3290_ATATACGAGCGAAG-1 Patient6 Monocytes nonactive 2 0.04319637
Monocytes_2
10x_3290_ATATACGAGCTTAG-1 Patient6 Monocytes nonactive 2 0.03138672
Monocytes_2
10x_3290_ATCAACCTGAGCTT-1 Patient6 Monocytes nonactive 2 0.11080464
Monocytes_2
10x_3290_ATCAACCTGCGAGA-1 Patient6 Monocytes nonactive 16 0.040882
Monocytes_16
10x_3290_ATCAACCTTTTCTG-1 Patient6 Monocytes nonactive 2 0.0421343
Monocytes_2
10x_3290_ATCACGGAACCATG-1 Patient6 Progenitors active 6 0.18576818
Progenitors_6
10x_3290_ATCACTACCGGGAA-1 Patient6 Monocytes nonactive 2 0.05617906
Monocytes_2
10x_3290_ATCACTACGCTACA-1 Patient6 Monocytes nonactive 2 0.02717012
Monocytes_2
10x_3290_ATCAGGTGAGTAGA-1 Patient6 Monocytes nonactive 2 0.01895885
Monocytes_2
10x_3290_ATCATGCTGCGAAG-1 Patient6 Monocytes nonactive 3 0.02861264
Monocytes_3
10x_3290_ATCCAGGACCTCCA-1 Patient6 Monocytes nonactive 3 0.05075772
Monocytes_3
10x_3290_ATCGACGAGGACAG-1 Patient6 Monocytes nonactive 2 0.03484243
Monocytes_2
10x_3290_ATCGAGTGTCCTTA-1 Patient6 Monocytes nonactive 3 0.02753472
Monocytes_3
10x_3290_ATCGCCTGAAAACG-1 Patient6 Monocytes nonactive 3 0.04484497
Monocytes_3

10x_3290_ATCGCGCTAACAGA-1 Patient6 Monocytes nonactive 2 0.06331241 Monocytes_2
10x_3290_ATCGCGCTACTGGT-1 Patient6 Monocytes nonactive 2 0.03788599 Monocytes_2
10x_3290_ATCGGAACTCAGGT-1 Patient6 Monocytes nonactive 2 0.03168791 Monocytes_2
10x_3290_ATCGTTTGTTACTC-1 Patient6 B cells nonactive 7 0.10695263 B cells_7
10x_3290_ATCTACACCGTCTC-1 Patient6 Monocytes nonactive 2 0.01620062 Monocytes_2
10x_3290_ATCTACTGATCAGC-1 Patient6 Monocytes nonactive 2 0.01834063 Monocytes_2
10x_3290_ATCTACTGGCGATT-1 Patient6 Monocytes nonactive 3 0.04400482 Monocytes_3
10x_3290_ATCTACTGGTATCG-1 Patient6 Monocytes nonactive 2 0.00875024 Monocytes_2
10x_3290_ATCTACTGTGGTTG-1 Patient6 Monocytes nonactive 2 0.06873375 Monocytes_2
10x_3290_ATCTCAACCAATCG-1 Patient6 Monocytes nonactive 2 0.03710925 Monocytes_2
10x_3290_ATCTGGGACTCCCA-1 Patient6 Monocytes nonactive 2 0.04785683 Monocytes_2
10x_3290_ATCTGTTGAGTACC-1 Patient6 Monocytes nonactive 2 0.06794116 Monocytes_2
10x_3290_ATCTTGACTAGTCG-1 Patient6 Monocytes nonactive 3 0.09216283 Monocytes_3
10x_3290_ATCTTTCTCTCCAC-1 Patient6 Monocytes nonactive 2 0.05797033 Monocytes_2
10x_3290_ATCTTTCTGGTAAA-1 Patient6 Monocytes nonactive 2 0.03956629 Monocytes_2
10x_3290_ATGAAACTTCGTAG-1 Patient6 Monocytes nonactive 2 0.0657853 Monocytes_2
10x_3290_ATGACGTGTCGTTT-1 Patient6 Monocytes nonactive 2 0.04329148 Monocytes_2

10x_3290_ATGACGTGTTCCGC-1	Patient6	Monocytes	nonactive	2	0.08407837
Monocytes_2					
10x_3290_ATGAGAGACCGATA-1	Patient6	Monocytes	nonactive	2	0.01697736
Monocytes_2					
10x_3290_ATGAGAGACGTGAT-1	Patient6	Monocytes	nonactive	3	0.03450954
Monocytes_3					
10x_3290_ATGAGAGATGACAC-1	Patient6	Monocytes	nonactive	2	0.03249635
Monocytes_2					
10x_3290_ATGAGCACCCCTAC-1	Patient6	Monocytes	nonactive	2	0.04227696
Monocytes_2					
10x_3290_ATGATATGCAGTTG-1	Patient6	Progenitors	active	6	0.14941982
Progenitors_6					
10x_3290_ATGATATGTGGTCA-1	Patient6	Monocytes	nonactive	3	0.01418743
Monocytes_3					
10x_3290_ATGCACGACAGGAG-1	Patient6	Monocytes	nonactive	2	0.00825883
Monocytes_2					
10x_3290_ATGCAGACAGCTCA-1	Patient6	Monocytes	nonactive	2	0.03029294
Monocytes_2					
10x_3290_ATGCAGTGCAGGAG-1	Patient6	Monocytes	nonactive	3	0.04983831
Monocytes_3					
10x_3290_ATGCCAGAAACAGA-1	Patient6	Monocytes	nonactive	2	0.03298776
Monocytes_2					
10x_3290_ATGCCAGAGTCTAG-1	Patient6	Progenitors	active	6	0.12616511
Progenitors_6					
10x_3290_ATGCGATGACGTTG-1	Patient6	Monocytes	nonactive	3	0.06381967
Monocytes_3					
10x_3290_ATGCGCCTACTCTT-1	Patient6	Monocytes	nonactive	3	0.04024792
Monocytes_3					
10x_3290_ATGCTTTGAACGGG-1	Patient6	Monocytes	nonactive	2	0.02277915
Monocytes_2					
10x_3290_ATGCTTTGAGATCC-1	Patient6	Monocytes	nonactive	2	0.05180394
Monocytes_2					
10x_3290_ATGCTTTGCACTCC-1	Patient6	Monocytes	nonactive	2	0.02892968

Monocytes_2
10x_3290_ATGTACCTTCTTTG-1 Patient6 Monocytes nonactive 2 0.04195993
Monocytes_2
10x_3290_ATGTCGGATTAGGC-1 Patient6 Monocytes nonactive 2 0.04812631
Monocytes_2
10x_3290_ATGTTAGATACAGC-1 Patient6 Monocytes nonactive 2 0.0522478
Monocytes_2
10x_3290_ATGTTGCTAACAGA-1 Patient6 Monocytes nonactive 2 0.05026631
Monocytes_2
10x_3290_ATGTTGCTCAGATC-1 Patient6 Monocytes nonactive 3 0.02667871
Monocytes_3
10x_3290_ATGTTGCTTAAGGA-1 Patient6 Monocytes nonactive 2 0.03317798
Monocytes_2
10x_3290_ATTAACGACTCGAA-1 Patient6 Monocytes nonactive 3 0.07062013
Monocytes_3
10x_3290_ATTAAGACCCACAA-1 Patient6 Progenitors active 6 0.12738571
Progenitors_6
10x_3290_ATTAAGACGATAGA-1 Patient6 Monocytes nonactive 2 0.05307209
Monocytes_2
10x_3290_ATTAAGACGCAGAG-1 Patient6 Monocytes nonactive 3 0.02888213
Monocytes_3
10x_3290_ATTCAAGAAAGCCT-1 Patient6 T cells nonactive 9 0.07509036 T cells_9
10x_3290_ATTCCATGCCTGTC-1 Patient6 Monocytes nonactive 3 0.05351595
Monocytes_3
10x_3290_ATTCGACTGCGTAT-1 Patient6 Monocytes nonactive 2 0.04785683
Monocytes_2
10x_3290_ATTCGGGACTGGTA-1 Patient6 Monocytes nonactive 2 0.04639845
Monocytes_2
10x_3290_ATTCGGGAGGTTAC-1 Patient6 Progenitors active 6 0.12296303
Progenitors_6
10x_3290_ATTCTGACACAGCT-1 Patient6 Monocytes nonactive 3 0.05960307
Monocytes_3
10x_3290_ATTCTGACGGTGTT-1 Patient6 Monocytes nonactive 2 0.01485321

Monocytes_2
10x_3290_ATTCTTCTTGGAAA-1	Patient6 Monocytes	nonactive	2	0.06719612
Monocytes_2
10x_3290_ATTGAAACAAAACG-1	Patient6 Monocytes	nonactive	3	0.04394141
Monocytes_3
10x_3290_ATTGAAACTCCCAC-1	Patient6 Monocytes	nonactive	3	0.03955044
Monocytes_3
10x_3290_ATTGAATGGGAGCA-1	Patient6 Monocytes	nonactive	2	0.02667871
Monocytes_2
10x_3290_ATTGCACTCTCCCA-1	Patient6 Monocytes	nonactive	3	0.02877116
Monocytes_3
10x_3290_ATTGCACTTGCTTT-1	Patient6 Progenitors	active	6	0.1395441
Progenitors_6
10x_3290_ATTGCTACTGTCGA-1	Patient6 Monocytes	nonactive	3	0.06101389
Monocytes_3
10x_3290_ATTGGGTGATGCCA-1	Patient6 Monocytes	nonactive	2	0.02885042
Monocytes_2
10x_3290_ATTGGGTGTATCGG-1	Patient6 Monocytes	nonactive	3	0.04967979
Monocytes_3
10x_3290_ATTGGTCTGCGTAT-1	Patient6 Monocytes	nonactive	3	0.05085283
Monocytes_3
10x_3290_ATTTCCGATTACTC-1	Patient6 Progenitors	active	6	0.16206962
Progenitors_6
10x_3290_ATTTCTCTCCCTTG-1	Patient6 Progenitors	active	6	0.15790058
Progenitors_6
10x_3290_ATTTGCACGAGGGT-1	Patient6 Monocytes	nonactive	3	0.04958468
Monocytes_3
10x_3290_CAAACTCTTTGGCA-1	Patient6 Monocytes	nonactive	3	0.05503773
Monocytes_3
10x_3290_CAAAGCACCCCGTT-1	Patient6 Monocytes	nonactive	3	0.03644347
Monocytes_3
10x_3290_CAAAGCTGCGTAGT-1	Patient6 T cells	nonactive	9	0.06472323	T cells_9
10x_3290_CAAAGCTGGTCATG-1	Patient6 Progenitors	active	6	0.12347029

Progenitors_6
10x_3290_CAAAGCTGTCCCGT-1 Patient6 Monocytes nonactive 2 0.04075518
Monocytes_2
10x_3290_CAAAGCTGTTGCTT-1 Patient6 Monocytes nonactive 3 0.04023207
Monocytes_3
10x_3290_CAACAGACAAGTAG-1 Patient6 Monocytes nonactive 2 0.05245387
Monocytes_2
10x_3290_CAACGATGAGAGTA-1 Patient6 Monocytes nonactive 2 0.01957707
Monocytes_2
10x_3290_CAACTTTGCGTGAT-1 Patient6 Monocytes nonactive 3 0.04080274
Monocytes_3
10x_3290_CAACTTTGTCTAGG-1 Patient6 Monocytes nonactive 3 0.05931774
Monocytes_3
10x_3290_CAAGAAGAGAGACG-1 Patient6 Monocytes nonactive 2 0.04010526
Monocytes_2
10x_3290_CAAGACTGCCCGTT-1 Patient6 Monocytes nonactive 2 0.05449876
Monocytes_2
10x_3290_CAAGCATGTAGCCA-1 Patient6 Monocytes nonactive 3 0.05538647
Monocytes_3
10x_3290_CAAGCCCTAAAAGC-1 Patient6 Monocytes nonactive 2 0.04478156
Monocytes_2
10x_3290_CAAGCCCTACCCAA-1 Patient6 Monocytes nonactive 3 0.00979646
Monocytes_3
10x_3290_CAAGCCCTATCGAC-1 Patient6 T cells active 9 0.14601167 T cells_9
10x_3290_CAAGCTGACGCATA-1 Patient6 B cells nonactive 7 0.03503265 B cells_7
10x_3290_CAAGGACTCTCAAG-1 Patient6 Progenitors active 6 0.15320842
Progenitors_6
10x_3290_CAAGGTTGTTTCAC-1 Patient6 Progenitors active 6 0.14737493
Progenitors_6
10x_3290_CAAGTCGAAAAGCA-1 Patient6 Progenitors nonactive 6 0.10291041
Progenitors_6
10x_3290_CAAGTCGAAAGGCG-1 Patient6 Monocytes nonactive 2 0.04630334
Monocytes_2

10x_3290_CAATAATGCCGAAT-1 Patient6 Monocytes nonactive 2 0.04325978
Monocytes_2
10x_3290_CAATATGAGTCATG-1 Patient6 Monocytes nonactive 2 0.05050409
Monocytes_2
10x_3290_CAATCGGAAGAGGC-1 Patient6 Progenitors active 6 0.15685435
Progenitors_6
10x_3290_CACAACGACACTTT-1 Patient6 Monocytes nonactive 2 0.04129415
Monocytes_2
10x_3290_CACAACGATACTGG-1 Patient6 Monocytes nonactive 3 0.04053326
Monocytes_3
10x_3290_CACAATCTAAAGCA-1 Patient6 Monocytes nonactive 2 0.040454
Monocytes_2
10x_3290_CACAATCTCGAGTT-1 Patient6 Monocytes nonactive 2 0.05193076
Monocytes_2
10x_3290_CACACCTGGGAGCA-1 Patient6 Progenitors active 6 0.15292309
Progenitors_6
10x_3290_CACAGAACCATCAG-1 Patient6 Monocytes nonactive 2 0.03382791
Monocytes_2
10x_3290_CACAGAACCGACTA-1 Patient6 Monocytes nonactive 2 0.01632744
Monocytes_2
10x_3290_CACAGAACCTGACA-1 Patient6 Monocytes nonactive 3 0.05491091
Monocytes_3
10x_3290_CACAGAACGGAGTG-1 Patient6 Monocytes nonactive 2 0.00924165
Monocytes_2
10x_3290_CACAGATGGCTATG-1 Patient6 Monocytes nonactive 3 0.07870458
Monocytes_3
10x_3290_CACAGATGGCTTAG-1 Patient6 Monocytes nonactive 2 0.0244753
Monocytes_2
10x_3290_CACAGTGATGCCCT-1 Patient6 Monocytes nonactive 2 0.02106715
Monocytes_2
10x_3290_CACATGGACTTAGG-1 Patient6 Monocytes nonactive 2 0.04150022
Monocytes_2
10x_3290_CACCACTGCGAGAG-1 Patient6 Monocytes nonactive 2 0.0286602

Monocytes_2
10x_3290_CACCACTGGGTATC-1 Patient6 Monocytes nonactive 2 0.0851246 Monocytes_2
10x_3290_CACCCATGTTTGTC-1 Patient6 Monocytes nonactive 2 0.02986494 Monocytes_2
10x_3290_CACCGGGATTCTGT-1 Patient6 Monocytes nonactive 2 0.07404413 Monocytes_2
10x_3290_CACCGTACACCCTC-1 Patient6 Monocytes nonactive 3 0.04115148 Monocytes_3
10x_3290_CACCGTTGATTCTC-1 Patient6 Progenitors active 6 0.13090483 Progenitors_6
10x_3290_CACCGTTGGACGGA-1 Patient6 Monocytes nonactive 2 0.03430347 Monocytes_2
10x_3290_CACCTGACAGTCAC-1 Patient6 Monocytes nonactive 2 0.02152685 Monocytes_2
10x_3290_CACGACCTAAAGCA-1 Patient6 Progenitors active 6 0.15967599 Progenitors_6
10x_3290_CACGCTACAAAAGC-1 Patient6 Progenitors active 6 0.18824108 Progenitors_6
10x_3290_CACGGGACGCTTAG-1 Patient6 Monocytes nonactive 3 0.05234291 Monocytes_3
10x_3290_CACTAACTCGTTGA-1 Patient6 Monocytes nonactive 2 0.03649103 Monocytes_2
10x_3290_CACTTATGCCGTAA-1 Patient6 Monocytes nonactive 2 0.05758988 Monocytes_2
10x_3290_CACTTATGCCTAAG-1 Patient6 Monocytes nonactive 2 0.01677129 Monocytes_2
10x_3290_CACTTATGTTCTTG-1 Patient6 Progenitors active 6 0.15813836 Progenitors_6
10x_3290_CACTTTGAACAGTC-1 Patient6 Progenitors active 6 0.12881238 Progenitors_6
10x_3290_CACTTTGATTGTGG-1 Patient6 Progenitors active 6 0.16625452 Progenitors_6

10x_3290_CAGACCCTCTAGTG-1 Patient6 Monocytes nonactive 3 0.04855431 Monocytes_3
10x_3290_CAGAGGGAGCAGTT-1 Patient6 Monocytes nonactive 2 0.06340752 Monocytes_2
10x_3290_CAGCAATGCGTAAC-1 Patient6 Monocytes nonactive 2 0.06155285 Monocytes_2
10x_3290_CAGCATGAGTCATG-1 Patient6 Monocytes nonactive 2 0.01580432 Monocytes_2
10x_3290_CAGCATGATGCACA-1 Patient6 Monocytes nonactive 2 0.043022 Monocytes_2
10x_3290_CAGCCTACCACCAA-1 Patient6 Monocytes nonactive 3 0.04787268 Monocytes_3
10x_3290_CAGCCTTGCCGATA-1 Patient6 Monocytes nonactive 3 0.03294021 Monocytes_3
10x_3290_CAGCTAGAAACGAA-1 Patient6 Monocytes nonactive 3 0.05839833 Monocytes_3
10x_3290_CAGCTAGACCGATA-1 Patient6 Monocytes nonactive 2 0.05201002 Monocytes_2
10x_3290_CAGCTCACACACAC-1 Patient6 Monocytes nonactive 2 0.03522288 Monocytes_2
10x_3290_CAGCTCTGCCAACA-1 Patient6 Progenitors active 6 0.15753598 Progenitors_6
10x_3290_CAGCTCTGGAAAGT-1 Patient6 Monocytes active 2 0.16427303 Monocytes_2
10x_3290_CAGCTCTGGGTCAT-1 Patient6 Monocytes nonactive 2 0.04964809 Monocytes_2
10x_3290_CAGGAACTCCTGAA-1 Patient6 Monocytes nonactive 3 0.06171137 Monocytes_3
10x_3290_CAGGCCGAAAGTAG-1 Patient6 Monocytes nonactive 3 0.04872868 Monocytes_3
10x_3290_CAGGGCACAAGGGC-1 Patient6 Monocytes nonactive 2 0.05543402 Monocytes_2
10x_3290_CAGGTATGCATGCA-1 Patient6 Monocytes nonactive 3 0.0324488

Monocytes_3
10x_3290_CAGTCAGAACGTGT-1 Patient6 Monocytes nonactive 2 0.03355843
Monocytes_2
10x_3290_CAGTTACTGCATAC-1 Patient6 Progenitors active 6 0.16470103
Progenitors_6
10x_3290_CAGTTGGAGAATAG-1 Patient6 Monocytes nonactive 2 0.03710925
Monocytes_2
10x_3290_CAGTTGGAGGTATC-1 Patient6 Monocytes nonactive 2 0.01616892
Monocytes_2
10x_3290_CAGTTTACCTCATT-1 Patient6 Monocytes nonactive 3 0.04389386
Monocytes_3
10x_3290_CAGTTTACTCACCC-1 Patient6 Monocytes nonactive 2 0.05500602
Monocytes_2
10x_3290_CATACTACAGGCGA-1 Patient6 Monocytes nonactive 2 0.05923848
Monocytes_2
10x_3290_CATACTTGAGGGTG-1 Patient6 Monocytes nonactive 3 0.07179316
Monocytes_3
10x_3290_CATACTTGTGACAC-1 Patient6 Monocytes nonactive 3 0.05541817
Monocytes_3
10x_3290_CATAGTCTCGTAAC-1 Patient6 Monocytes nonactive 2 0.02246211
Monocytes_2
10x_3290_CATAGTCTGGATCT-1 Patient6 Progenitors active 6 0.12924038
Progenitors_6
10x_3290_CATCAACTTCACCC-1 Patient6 Monocytes nonactive 3 0.05847759
Monocytes_3
10x_3290_CATCATACAGAAGT-1 Patient6 Monocytes nonactive 2 0.02797857
Monocytes_2
10x_3290_CATCATACCCAGTA-1 Patient6 Dendritic cells active 14 0.18121869
Dendritic cells_14
10x_3290_CATCATACCCATGA-1 Patient6 Monocytes nonactive 3 0.04585949
Monocytes_3
10x_3290_CATCCCGATATCTC-1 Patient6 Monocytes nonactive 3 0.03571429
Monocytes_3

10x_3290_CATGAGACGCATCA-1 Patient6 Monocytes nonactive 2 0.0400577 Monocytes_2
10x_3290_CATGCCACTCTTTG-1 Patient6 Progenitors active 6 0.13838691 Progenitors_6
10x_3290_CATGCGCTCGGGAA-1 Patient6 Progenitors active 6 0.12776615 Progenitors_6
10x_3290_CATGGATGGATAGA-1 Patient6 Monocytes nonactive 2 0.05489506 Monocytes_2
10x_3290_CATGGATGTTGTGG-1 Patient6 Monocytes nonactive 2 0.03747384 Monocytes_2
10x_3290_CATGGCCTTTGTCT-1 Patient6 Monocytes nonactive 3 0.03717266 Monocytes_3
10x_3290_CATGTTACCAATCG-1 Patient6 Monocytes nonactive 2 0.05953966 Monocytes_2
10x_3290_CATTAGCTCGGAGA-1 Patient6 Monocytes nonactive 3 0.0729662 Monocytes_3
10x_3290_CATTCCCTGCAAGG-1 Patient6 Monocytes nonactive 3 0.04356097 Monocytes_3
10x_3290_CATTGTTGCCTTAT-1 Patient6 Monocytes nonactive 3 0.06778264 Monocytes_3
10x_3290_CATTTGACAAGGTA-1 Patient6 Monocytes nonactive 3 0.0502029 Monocytes_3
10x_3290_CATTTGACACGGGA-1 Patient6 Progenitors active 6 0.18752774 Progenitors_6
10x_3290_CATTTGACTGCACA-1 Patient6 Monocytes nonactive 3 0.04644601 Monocytes_3
10x_3290_CATTTGTGCTGTAG-1 Patient6 Progenitors active 6 0.12878067 Progenitors_6
10x_3290_CCAAGAACGGCGAA-1 Patient6 Monocytes nonactive 2 0.05462558 Monocytes_2
10x_3290_CCAAGATGCTCTAT-1 Patient6 Progenitors active 6 0.18559381 Progenitors_6
10x_3290_CCAAGTGAGGGTGA-1 Patient6 Monocytes nonactive 2 0.03612643

Monocytes_2
10x_3290_CCAATGGAATGACC-1 Patient6 Monocytes nonactive 2 0.040454 Monocytes_2
10x_3290_CCAATTTGTAGAGA-1 Patient6 Progenitors nonactive 6 0.10468582 Progenitors_6
10x_3290_CCACCATGCTCTCG-1 Patient6 Monocytes nonactive 2 0.0527075 Monocytes_2
10x_3290_CCACCTGATACTGG-1 Patient6 Monocytes nonactive 2 0.01875277 Monocytes_2
10x_3290_CCACCTGATCCGAA-1 Patient6 Monocytes nonactive 2 0.00055482 Monocytes_2
10x_3290_CCACCTGATCTCCG-1 Patient6 Monocytes nonactive 2 0.02159026 Monocytes_2
10x_3290_CCACGGGATCCAGA-1 Patient6 Monocytes nonactive 3 0.04584364 Monocytes_3
10x_3290_CCACTGACGCATCA-1 Patient6 Progenitors nonactive 6 0.11668569 Progenitors_6
10x_3290_CCACTGACTTGTGG-1 Patient6 Monocytes nonactive 2 0.03688732 Monocytes_2
10x_3290_CCAGACCTAACAGA-1 Patient6 Monocytes nonactive 2 0.0468106 Monocytes_2
10x_3290_CCAGATGAATGCTG-1 Patient6 Monocytes nonactive 3 0.07314057 Monocytes_3
10x_3290_CCAGCACTGTTCTT-1 Patient6 Monocytes nonactive 3 0.06147359 Monocytes_3
10x_3290_CCAGCTACAGAATG-1 Patient6 Monocytes nonactive 3 0.04833238 Monocytes_3
10x_3290_CCAGCTACCCAACA-1 Patient6 Progenitors active 6 0.16384503 Progenitors_6
10x_3290_CCAGCTACCCCTAC-1 Patient6 Monocytes nonactive 3 0.02330226 Monocytes_3
10x_3290_CCAGGTCTCTACGA-1 Patient6 Monocytes nonactive 2 0.02864435 Monocytes_2

10x_3290_CCAGGTCTTCGACA-1 Patient6 Progenitors active 6 0.14431552 Progenitors_6
10x_3290_CCAGTCTGAACCAC-1 Patient6 Monocytes nonactive 2 0.05023461 Monocytes_2
10x_3290_CCAGTCTGCGAGTT-1 Patient6 Monocytes nonactive 2 0.03313043 Monocytes_2
10x_3290_CCAGTCTGTGTTTC-1 Patient6 Monocytes nonactive 2 0.03021368 Monocytes_2
10x_3290_CCAGTGCTACCTCC-1 Patient6 Progenitors active 6 0.15068797 Progenitors_6
10x_3290_CCATATACGTTAGC-1 Patient6 Monocytes nonactive 2 0.01356921 Monocytes_2
10x_3290_CCATATACTCAGGT-1 Patient6 Monocytes nonactive 3 0.0737588 Monocytes_3
10x_3290_CCATCCGAGTAAGA-1 Patient6 Progenitors nonactive 6 0.08694756 Progenitors_6
10x_3290_CCATCCGATATGCG-1 Patient6 Monocytes nonactive 2 0.06440619 Monocytes_2
10x_3290_CCATGCTGGCCAAT-1 Patient6 Progenitors active 6 0.14008306 Progenitors_6
10x_3290_CCCAACACCTTGTT-1 Patient6 Progenitors active 6 0.13721387 Progenitors_6
10x_3290_CCCACATGCCTGTC-1 Patient6 Monocytes nonactive 2 0.03782259 Monocytes_2
10x_3290_CCCAGTTGCTGATG-1 Patient6 Monocytes nonactive 3 0.06932027 Monocytes_3
10x_3290_CCCATCGAAACGTC-1 Patient6 Monocytes nonactive 2 0.02715427 Monocytes_2
10x_3290_CCCATCGAGCCCTT-1 Patient6 Monocytes nonactive 3 0.04046985 Monocytes_3
10x_3290_CCCATCGATGTCCC-1 Patient6 Monocytes nonactive 2 0.04687401 Monocytes_2
10x_3290_CCCGATTGTGACTG-1 Patient6 Monocytes nonactive 2 0.04081859

Monocytes_2
10x_3290_CCCGGAGACTGTGA-1 Patient6 Progenitors active 6 0.14027329
Progenitors_6
10x_3290_CCCTAGTGGAGGCA-1 Patient6 Monocytes nonactive 2 0.02263648
Monocytes_2
10x_3290_CCCTAGTGGCAAGG-1 Patient6 Progenitors active 6 0.1597711
Progenitors_6
10x_3290_CCCTGAACAGTCGT-1 Patient6 Monocytes nonactive 2 0.03417665
Monocytes_2
10x_3290_CCCTGAACGTTCGA-1 Patient6 Monocytes nonactive 3 0.0417697
Monocytes_3
10x_3290_CCCTGATGGTCCTC-1 Patient6 Monocytes nonactive 2 0.0329085
Monocytes_2
10x_3290_CCGACACTTTACTC-1 Patient6 Monocytes nonactive 2 0.05040898
Monocytes_2
10x_3290_CCGACTACACCTGA-1 Patient6 Monocytes nonactive 2 0.02842242
Monocytes_2
10x_3290_CCGATAGATTTCAC-1 Patient6 Monocytes nonactive 2 0.02667871
Monocytes_2
10x_3290_CCGCGAGAAGCACT-1 Patient6 Progenitors active 6 0.1302549
Progenitors_6
10x_3290_CCGCTATGCGGTAT-1 Patient6 Progenitors active 6 0.13077801
Progenitors_6
10x_3290_CCGCTATGTGTAGC-1 Patient6 Monocytes nonactive 2 0.02926257
Monocytes_2
10x_3290_CCGGAGACACCAGT-1 Patient6 Monocytes nonactive 2 0.02146345
Monocytes_2
10x_3290_CCGGAGACACCTCC-1 Patient6 Monocytes nonactive 8 0.09387483
Monocytes_8
10x_3290_CCGGAGACGTACGT-1 Patient6 Monocytes nonactive 2 0.0767231
Monocytes_2
10x_3290_CCGGAGTGAGAGAT-1 Patient6 Monocytes nonactive 2 0.02808953
Monocytes_2

10x_3290_CCGGTACTGTGTTG-1 Patient6 Progenitors active 6 0.12042673 Progenitors_6
10x_3290_CCGTAAGAGTGTAC-1 Patient6 Progenitors nonactive 6 0.11622598 Progenitors_6
10x_3290_CCTACCGATGCGTA-1 Patient6 Monocytes nonactive 2 0.02583856 Monocytes_2
10x_3290_CCTAGAGAATTTCC-1 Patient6 Progenitors active 6 0.14241329 Progenitors_6
10x_3290_CCTCATCTAGCACT-1 Patient6 Monocytes nonactive 2 0.01155602 Monocytes_2
10x_3290_CCTCATCTCTAAGC-1 Patient6 Monocytes nonactive 2 0.04264156 Monocytes_2
10x_3290_CCTCTACTAAGGGC-1 Patient6 Monocytes nonactive 3 0.03560332 Monocytes_3
10x_3290_CCTGCAACCTAAGC-1 Patient6 Monocytes nonactive 2 0.02788346 Monocytes_2
10x_3290_CCTGCAACTAGTCG-1 Patient6 Monocytes nonactive 2 0.04186482 Monocytes_2
10x_3290_CCTGCAACTATCTC-1 Patient6 B cells nonactive 7 0.06302708 B cells_7
10x_3290_CCTGGACTTGGAGG-1 Patient6 Monocytes nonactive 3 0.03875785 Monocytes_3
10x_3290_CCTGGACTTTCACT-1 Patient6 Monocytes nonactive 3 0.05174054 Monocytes_3
10x_3290_CCTTCACTTCCTAT-1 Patient6 Monocytes nonactive 2 0.01870522 Monocytes_2
10x_3290_CCTTCACTTCTCAT-1 Patient6 Monocytes nonactive 3 0.04153193 Monocytes_3
10x_3290_CCTTCACTTTTCTG-1 Patient6 Progenitors active 6 0.14214381 Progenitors_6
10x_3290_CCTTTAGATTACCT-1 Patient6 Monocytes nonactive 3 0.03414495 Monocytes_3
10x_3290_CGAAGACTATCTCT-1 Patient6 Monocytes nonactive 3 0.04085029 Monocytes_3

10x_3290_CGAAGACTTCCTCG-1 Patient6 Progenitors active 6 0.13038171 Progenitors_6
10x_3290_CGAAGTACAGTTCG-1 Patient6 Monocytes nonactive 2 0.07838755 Monocytes_2
10x_3290_CGAAGTACGGTTTG-1 Patient6 T cells nonactive 9 0.10278359 T cells_9
10x_3290_CGACAAACTGCTGA-1 Patient6 Progenitors nonactive 6 0.11701858 Progenitors_6
10x_3290_CGACCACTGAGGGT-1 Patient6 Monocytes nonactive 2 0.01555069 Monocytes_2
10x_3290_CGACCGGACTCCCA-1 Patient6 Monocytes nonactive 2 0.03878955 Monocytes_2
10x_3290_CGACGTCTTGGTCA-1 Patient6 Monocytes nonactive 2 0.04663623 Monocytes_2
10x_3290_CGACTGCTCATGAC-1 Patient6 Monocytes nonactive 2 0.0476349 Monocytes_2
10x_3290_CGACTGCTCATTCT-1 Patient6 Monocytes nonactive 2 0.0278359 Monocytes_2
10x_3290_CGAGGAGACTACTT-1 Patient6 Monocytes nonactive 3 0.03045146 Monocytes_3
10x_3290_CGAGGCACTTGCTT-1 Patient6 Progenitors active 6 0.12656141 Progenitors_6
10x_3290_CGAGGCTGAATCGC-1 Patient6 Monocytes nonactive 2 0.02894553 Monocytes_2
10x_3290_CGAGGGCTCGGGAA-1 Patient6 Monocytes nonactive 2 0.02537886 Monocytes_2
10x_3290_CGAGGGCTTCTTCA-1 Patient6 Monocytes nonactive 2 0.01932344 Monocytes_2
10x_3290_CGAGGGCTTTTACC-1 Patient6 Progenitors active 6 0.12424704 Progenitors_6
10x_3290_CGAGTATGTGACAC-1 Patient6 Monocytes nonactive 3 0.02883457 Monocytes_3
10x_3290_CGATCAGACGTGTA-1 Patient6 Monocytes nonactive 2 0.02262063 Monocytes_2

10x_3290_CGATCAGAGGAAAT-1 Patient6 Monocytes nonactive 2 0.03465221
Monocytes_2
10x_3290_CGATCAGAGGACGA-1 Patient6 Monocytes nonactive 2 0.03294021
Monocytes_2
10x_3290_CGCATAGATCTTAC-1 Patient6 Monocytes nonactive 3 0.05175639
Monocytes_3
10x_3290_CGCCATACAGTCTG-1 Patient6 Progenitors active 6 0.14428381
Progenitors_6
10x_3290_CGCCATACCACACA-1 Patient6 Monocytes nonactive 2 0.04557416
Monocytes_2
10x_3290_CGCCATACTCCTGC-1 Patient6 Monocytes nonactive 3 0.0278042
Monocytes_3
10x_3290_CGCCTAACTGTCTT-1 Patient6 Progenitors active 6 0.13689684
Progenitors_6
10x_3290_CGCGAGACCTGAGT-1 Patient6 Progenitors active 6 0.16669837
Progenitors_6
10x_3290_CGCGAGACTAGAAG-1 Patient6 Monocytes nonactive 2 0.05159787
Monocytes_2
10x_3290_CGCTACACAAACGA-1 Patient6 Progenitors nonactive 6 0.10262507
Progenitors_6
10x_3290_CGCTACTGGACACT-1 Patient6 Monocytes nonactive 2 0.0509955
Monocytes_2
10x_3290_CGCTCATGCCGTAA-1 Patient6 Monocytes nonactive 3 0.04665208
Monocytes_3
10x_3290_CGGACCGACGTACA-1 Patient6 Monocytes nonactive 3 0.04356097
Monocytes_3
10x_3290_CGGACTCTTGCACA-1 Patient6 Progenitors active 6 0.1437924
Progenitors_6
10x_3290_CGGATATGTCTAGG-1 Patient6 Monocytes nonactive 2 0.04064422
Monocytes_2
10x_3290_CGGCACGAGAGCAG-1 Patient6 Monocytes nonactive 2 0.01537632
Monocytes_2
10x_3290_CGGCATCTCACACA-1 Patient6 Monocytes nonactive 2 0.0379494

Monocytes_2
10x_3290_CGGCGAACTCGACA-1 Patient6 Monocytes nonactive 2 0.02894553
Monocytes_2
10x_3290_CGGGACTGAATGCC-1 Patient6 Monocytes nonactive 2 0.04752394
Monocytes_2
10x_3290_CGGGACTGCCTTCG-1 Patient6 Monocytes nonactive 2 0.03075265
Monocytes_2
10x_3290_CGGGCATGACCCTC-1 Patient6 Progenitors active 6 0.15752013
Progenitors_6
10x_3290_CGGTAAACCGTTAG-1 Patient6 Monocytes nonactive 2 0.01542388
Monocytes_2
10x_3290_CGGTAAACGTCGTA-1 Patient6 Monocytes nonactive 2 0.02152685
Monocytes_2
10x_3290_CGGTACCTGCCAAT-1 Patient6 Monocytes nonactive 2 0.04433771
Monocytes_2
10x_3290_CGGTCACTGCGATT-1 Patient6 Monocytes nonactive 3 0.03270243
Monocytes_3
10x_3290_CGGTCACTTACTCT-1 Patient6 Monocytes nonactive 3 0.03008687
Monocytes_3
10x_3290_CGTAACGAATCTCT-1 Patient6 Monocytes nonactive 3 0.05246972
Monocytes_3
10x_3290_CGTAACGAGGGCAA-1 Patient6 Monocytes nonactive 3 0.04692156
Monocytes_3
10x_3290_CGTACAGATGGGAG-1 Patient6 Monocytes nonactive 2 0.04758734
Monocytes_2
10x_3290_CGTACCACGGATTC-1 Patient6 Progenitors nonactive 6 0.08560015
Progenitors_6
10x_3290_CGTAGCCTCAGCTA-1 Patient6 Monocytes nonactive 2 0.06166381
Monocytes_2
10x_3290_CGTCAAGATCGATG-1 Patient6 Monocytes nonactive 3 0.05277091
Monocytes_3
10x_3290_CGTCCATGCGCTAA-1 Patient6 Progenitors nonactive 6 0.10974257
Progenitors_6

10x_3290_CGTGAATGCGGTAT-1 Patient6 Monocytes nonactive 3 0.05272335 Monocytes_3
10x_3290_CGTGAATGCTGACA-1 Patient6 Monocytes nonactive 2 0.03812377 Monocytes_2
10x_3290_CGTGATGACCGTTC-1 Patient6 Monocytes nonactive 2 0.06643523 Monocytes_2
10x_3290_CGTGATGATTGCGA-1 Patient6 Progenitors active 5 0.123502 Progenitors_5
10x_3290_CGTGCACTAAGGTA-1 Patient6 Monocytes nonactive 3 0.06538901 Monocytes_3
10x_3290_CGTGTAGACCAAGT-1 Patient6 Monocytes nonactive 2 0.03048317 Monocytes_2
10x_3290_CTAAACCTATTTCC-1 Patient6 Monocytes nonactive 2 0.02859679 Monocytes_2
10x_3290_CTAAACCTTCCTGC-1 Patient6 Progenitors active 6 0.13063534 Progenitors_6
10x_3290_CTAAACCTTGCTAG-1 Patient6 Monocytes nonactive 2 0.01052565 Monocytes_2
10x_3290_CTAACGGACTCTTA-1 Patient6 Monocytes nonactive 2 0.0522795 Monocytes_2
10x_3290_CTAACGGAGTTCAG-1 Patient6 Progenitors active 6 0.13290216 Progenitors_6
10x_3290_CTAATAGAACTACG-1 Patient6 Monocytes nonactive 3 0.08439541 Monocytes_3
10x_3290_CTAATAGACAGTTG-1 Patient6 Monocytes nonactive 3 0.05128083 Monocytes_3
10x_3290_CTAATGCTGCTGTA-1 Patient6 Monocytes nonactive 3 0.03676051 Monocytes_3
10x_3290_CTACAACTAAGGCG-1 Patient6 Monocytes nonactive 2 0.02764568 Monocytes_2
10x_3290_CTACGGCTAACGAA-1 Patient6 Monocytes nonactive 2 0.08839008 Monocytes_2
10x_3290_CTACTATGTTTGGG-1 Patient6 Monocytes nonactive 2 0.01756388

Monocytes_2
10x_3290_CTATAAGAATTTCC-1 Patient6 Monocytes nonactive 3 0.08957897 Monocytes_3
10x_3290_CTATACTGTCAGGT-1 Patient6 B cells nonactive 7 0.0712542 B cells_7
10x_3290_CTATCAACAGGAGC-1 Patient6 Progenitors active 6 0.12708452 Progenitors_6
10x_3290_CTATCATGCGCATA-1 Patient6 Monocytes nonactive 2 0.05283432 Monocytes_2
10x_3290_CTATCATGGGCAAG-1 Patient6 Monocytes nonactive 3 0.0447023 Monocytes_3
10x_3290_CTATCATGGGTTTG-1 Patient6 Progenitors active 6 0.13252172 Progenitors_6
10x_3290_CTATCCCTCACTCC-1 Patient6 Progenitors active 6 0.15098916 Progenitors_6
10x_3290_CTATCCCTCCGTAA-1 Patient6 Progenitors active 6 0.15771035 Progenitors_6
10x_3290_CTATGACTTCTACT-1 Patient6 Monocytes nonactive 2 0.00648342 Monocytes_2
10x_3290_CTATGTTGGAGATA-1 Patient6 Monocytes nonactive 2 0.02843827 Monocytes_2
10x_3290_CTATGTTGGGAACG-1 Patient6 Monocytes nonactive 3 0.08560015 Monocytes_3
10x_3290_CTATTGACCACTAG-1 Patient6 Monocytes nonactive 3 0.02268404 Monocytes_3
10x_3290_CTATTGTGCCGCTT-1 Patient6 Monocytes nonactive 2 0.03940777 Monocytes_2
10x_3290_CTCAATTGCGTAAC-1 Patient6 Monocytes nonactive 3 0.04566927 Monocytes_3
10x_3290_CTCAATTGTCACGA-1 Patient6 Progenitors active 6 0.12323252 Progenitors_6
10x_3290_CTCAATTGTCGCCT-1 Patient6 Monocytes nonactive 2 0.04776171 Monocytes_2
10x_3290_CTCAGAGACGTTAG-1 Patient6 Monocytes nonactive 3 0.04398897

Monocytes_3
10x_3290_CTCAGCACAGAGTA-1 Patient6 Monocytes nonactive 2 0.03983577
Monocytes_2
10x_3290_CTCAGCACCGGGAA-1 Patient6 Monocytes nonactive 2 0.02602879
Monocytes_2
10x_3290_CTCATTGAGGGACA-1 Patient6 Progenitors nonactive 6 0.11579798
Progenitors_6
10x_3290_CTCGACTGCTGAGT-1 Patient6 Monocytes nonactive 2 0.0324805
Monocytes_2
10x_3290_CTCGCATGCTCCCA-1 Patient6 Progenitors active 6 0.11808065
Progenitors_6
10x_3290_CTCGCATGCTGGTA-1 Patient6 Monocytes nonactive 2 0.0485226
Monocytes_2
10x_3290_CTCGCATGGAGCAG-1 Patient6 Monocytes nonactive 2 0.039598
Monocytes_2
10x_3290_CTCGCATGTACGCA-1 Patient6 Monocytes nonactive 3 0.02973813
Monocytes_3
10x_3290_CTCGCATGTGATGC-1 Patient6 Monocytes nonactive 3 0.05868366
Monocytes_3
10x_3290_CTCTAAACGCGAAG-1 Patient6 Monocytes nonactive 2 0.04671549
Monocytes_2
10x_3290_CTCTAAACTGGTAC-1 Patient6 Monocytes nonactive 2 0.05979329
Monocytes_2
10x_3290_CTCTAATGTTTGGG-1 Patient6 Monocytes nonactive 2 0.07532813
Monocytes_2
10x_3290_CTGAACGAGGCATT-1 Patient6 Monocytes nonactive 3 0.05491091
Monocytes_3
10x_3290_CTGAAGACTAAGGA-1 Patient6 Monocytes nonactive 3 0.07160294
Monocytes_3
10x_3290_CTGACAGAAGAACA-1 Patient6 Progenitors active 6 0.14309492
Progenitors_6
10x_3290_CTGAGCCTCTTTAC-1 Patient6 Monocytes nonactive 3 0.04918838
Monocytes_3

10x_3290_CTGAGCCTGGAACG-1 Patient6 Progenitors active 6 0.15869317 Progenitors_6
10x_3290_CTGATGGAAGATGA-1 Patient6 Progenitors active 6 0.15603006 Progenitors_6
10x_3290_CTGATGGATTGACG-1 Patient6 Monocytes nonactive 2 0.06221863 Monocytes_2
10x_3290_CTGCGACTTCTTTG-1 Patient6 Monocytes nonactive 2 0.00729186 Monocytes_2
10x_3290_CTGGAAACTCTAGG-1 Patient6 Monocytes nonactive 2 0.01566166 Monocytes_2
10x_3290_CTGGATGATTCCCG-1 Patient6 Monocytes nonactive 2 0.01957707 Monocytes_2
10x_3290_CTGTAACTAATCGC-1 Patient6 Monocytes nonactive 2 0.06784605 Monocytes_2
10x_3290_CTGTATACGACGTT-1 Patient6 Progenitors active 6 0.19313931 Progenitors_6
10x_3290_CTGTATACGTCAAC-1 Patient6 Monocytes nonactive 3 0.05277091 Monocytes_3
10x_3290_CTGTGAGAATAAGG-1 Patient6 Monocytes nonactive 2 0.03680807 Monocytes_2
10x_3290_CTTAAAGAATACCG-1 Patient6 Progenitors active 6 0.15767865 Progenitors_6
10x_3290_CTTAACACACGTTG-1 Patient6 Monocytes nonactive 2 0.02114641 Monocytes_2
10x_3290_CTTAAGCTCCTGTC-1 Patient6 Monocytes nonactive 2 0.02243041 Monocytes_2
10x_3290_CTTACAACAATGCC-1 Patient6 Progenitors active 6 0.12386659 Progenitors_6
10x_3290_CTTACATGAAACGA-1 Patient6 Monocytes nonactive 3 0.02014774 Monocytes_3
10x_3290_CTTAGACTAGGTTC-1 Patient6 Monocytes nonactive 2 0.04215015 Monocytes_2
10x_3290_CTTAGGGACTACTT-1 Patient6 Monocytes nonactive 2 0.03910659

Monocytes_2
10x_3290_CTTATCGAACAGTC-1 Patient6 Monocytes nonactive 3 0.04257815
Monocytes_3
10x_3290_CTTCATGATTAGGC-1 Patient6 Progenitors active 6 0.13906854
Progenitors_6
10x_3290_CTTCTAGACCCACT-1 Patient6 Monocytes nonactive 3 0.01948196
Monocytes_3
10x_3290_CTTCTAGATTCTCA-1 Patient6 Monocytes nonactive 2 0.04324393
Monocytes_2
10x_3290_CTTGAGGAAGCATC-1 Patient6 Progenitors active 6 0.15293894
Progenitors_6
10x_3290_CTTGATTGGAAACA-1 Patient6 Monocytes nonactive 3 0.0303405
Monocytes_3
10x_3290_CTTGTATGGGTTTG-1 Patient6 Monocytes nonactive 2 0.01899055
Monocytes_2
10x_3290_CTTTACGAACCCAA-1 Patient6 Monocytes nonactive 3 0.07261746
Monocytes_3
10x_3290_CTTTAGTGAAGCAA-1 Patient6 Monocytes nonactive 2 0.03067339
Monocytes_2
10x_3290_CTTTCAGAATGACC-1 Patient6 Monocytes nonactive 2 0.00881365
Monocytes_2
10x_3290_CTTTGATGCTACGA-1 Patient6 Monocytes nonactive 3 0.03866274
Monocytes_3
10x_3290_CTTTGATGTACTCT-1 Patient6 Monocytes nonactive 2 0.04218185
Monocytes_2
10x_3290_GAAACAGACTAGAC-1 Patient6 Progenitors active 6 0.1631951
Progenitors_6
10x_3290_GAAACCTGAAAACG-1 Patient6 Monocytes nonactive 3 0.05253313
Monocytes_3
10x_3290_GAAAGATGCTTGAG-1 Patient6 Progenitors active 6 0.16666667
Progenitors_6
10x_3290_GAAAGCCTGCGGAA-1 Patient6 Monocytes nonactive 2 0.04061252
Monocytes_2

10x_3290_GAACACACAGGAGC-1 Patient6 Progenitors active 6 0.16890178
Progenitors_6
10x_3290_GAACACACATTCGG-1 Patient6 Progenitors nonactive 6 0.10780864
Progenitors_6
10x_3290_GAACACACCGCATA-1 Patient6 Progenitors nonactive 6 0.11321413
Progenitors_6
10x_3290_GAACACACGGATTC-1 Patient6 Monocytes nonactive 3 0.04609727
Monocytes_3
10x_3290_GAACAGCTAGGTTC-1 Patient6 Monocytes nonactive 2 0.03867859
Monocytes_2
10x_3290_GAACAGCTGTGCAT-1 Patient6 Monocytes nonactive 3 0.0206867
Monocytes_3
10x_3290_GAACAGCTTCCTTA-1 Patient6 Monocytes nonactive 3 0.10324329
Monocytes_3
10x_3290_GAACCTGAGCCATA-1 Patient6 Progenitors active 6 0.16711052
Progenitors_6
10x_3290_GAACTGTGCGCAAT-1 Patient6 Monocytes nonactive 2 0.01962463
Monocytes_2
10x_3290_GAAGATGACGTAAC-1 Patient6 Monocytes nonactive 3 0.0211147
Monocytes_3
10x_3290_GAAGCGGAGAAAGT-1 Patient6 Monocytes nonactive 2 0.01005009
Monocytes_2
10x_3290_GAAGGGTGGAACCT-1 Patient6 Monocytes nonactive 2 0.02466553
Monocytes_2
10x_3290_GAAGGGTGTGCGTA-1 Patient6 Monocytes nonactive 2 0.06749731
Monocytes_2
10x_3290_GAAGTAGAGAGGAC-1 Patient6 Monocytes nonactive 2 0.01778581
Monocytes_2
10x_3290_GAAGTCACGGAGCA-1 Patient6 Monocytes nonactive 2 0.06225033
Monocytes_2
10x_3290_GAATGCACCGCTAA-1 Patient6 Monocytes nonactive 2 0.04352926
Monocytes_2
10x_3290_GAATGCTGAGCAAA-1 Patient6 Monocytes nonactive 2 0.0050409

Monocytes_2
10x_3290_GAATGGCTCGTGTA-1 Patient6 Progenitors active 6 0.18367573 Progenitors_6
10x_3290_GACAACACTTGTGG-1 Patient6 Monocytes nonactive 3 0.03070509 Monocytes_3
10x_3290_GACAACTGGGCAAG-1 Patient6 Monocytes nonactive 3 0.05112231 Monocytes_3
10x_3290_GACAGGGACAGAAA-1 Patient6 Monocytes nonactive 3 0.04780927 Monocytes_3
10x_3290_GACAGTACAGGCGA-1 Patient6 Monocytes nonactive 2 0.05540232 Monocytes_2
10x_3290_GACAGTACGCGAGA-1 Patient6 Monocytes nonactive 3 0.04746053 Monocytes_3
10x_3290_GACAGTTGCTATTC-1 Patient6 Monocytes nonactive 2 0.03639592 Monocytes_2
10x_3290_GACAGTTGGGAAAT-1 Patient6 Progenitors active 6 0.1669837 Progenitors_6
10x_3290_GACAGTTGGTACGT-1 Patient6 Monocytes nonactive 2 0.07044575 Monocytes_2
10x_3290_GACCAAACTCTCGC-1 Patient6 Monocytes nonactive 3 0.0417697 Monocytes_3
10x_3290_GACCATGACACTAG-1 Patient6 Monocytes nonactive 3 0.08789867 Monocytes_3
10x_3290_GACCATGATAGAAG-1 Patient6 Monocytes nonactive 3 0.02304863 Monocytes_3
10x_3290_GACCCTACCTTGAG-1 Patient6 B cells nonactive 7 0.04994927 B cells_7
10x_3290_GACCTCACTAGCCA-1 Patient6 Monocytes nonactive 3 0.05193076 Monocytes_3
10x_3290_GACCTCTGGCTAAC-1 Patient6 Monocytes nonactive 16 0.07388561 Monocytes_16
10x_3290_GACGAGGAAACCTG-1 Patient6 Monocytes nonactive 2 0.01467884 Monocytes_2
10x_3290_GACGATTGAGCCAT-1 Patient6 Monocytes nonactive 2 0.04563756

Monocytes_2
10x_3290_GACGATTGTCTAGG-1 Patient6 Monocytes nonactive 3 0.04945787
Monocytes_3
10x_3290_GACGCCGATAACGC-1 Patient6 Monocytes nonactive 3 0.07310887
Monocytes_3
10x_3290_GACGGCACGATAAG-1 Patient6 Monocytes nonactive 2 0.02896138
Monocytes_2
10x_3290_GACGTAACCAACTG-1 Patient6 Progenitors active 6 0.16802993
Progenitors_6
10x_3290_GACGTCCTGAATGA-1 Patient6 Monocytes nonactive 4 0.08613912
Monocytes_4
10x_3290_GACGTCCTTGTAGC-1 Patient6 Monocytes nonactive 2 0.06044322
Monocytes_2
10x_3290_GACGTCCTTTTCTG-1 Patient6 Monocytes nonactive 3 0.04286348
Monocytes_3
10x_3290_GACTCCTGTTCCCG-1 Patient6 Progenitors active 6 0.14670915
Progenitors_6
10x_3290_GACTGATGAGAATG-1 Patient6 Progenitors active 6 0.12198022
Progenitors_6
10x_3290_GACTGATGTGCACA-1 Patient6 Monocytes nonactive 3 0.01827722
Monocytes_3
10x_3290_GACTTTACAAAGTG-1 Patient6 Monocytes nonactive 2 0.03704584
Monocytes_2
10x_3290_GAGCAGGATGCATG-1 Patient6 Monocytes nonactive 2 0.04866527
Monocytes_2
10x_3290_GAGCGAGACCACAA-1 Patient6 Progenitors active 6 0.11955488
Progenitors_6
10x_3290_GAGCGAGACTCCCA-1 Patient6 Monocytes nonactive 16 0.01375943
Monocytes_16
10x_3290_GAGCGAGATTCCCG-1 Patient6 Progenitors active 6 0.18102847
Progenitors_6
10x_3290_GAGCGCACAGAGAT-1 Patient6 Monocytes nonactive 3 0.04765075
Monocytes_3

10x_3290_GAGCGCACCGTGTA-1 Patient6 Monocytes nonactive 2 0.02558493 Monocytes_2
10x_3290_GAGCGCACGTATGC-1 Patient6 Monocytes nonactive 2 0.03211591 Monocytes_2
10x_3290_GAGCGGCTAAGGCG-1 Patient6 Monocytes nonactive 3 0.06293196 Monocytes_3
10x_3290_GAGCTCCTCTGAGT-1 Patient6 Monocytes nonactive 2 0.02306449 Monocytes_2
10x_3290_GAGGATCTGGTAAA-1 Patient6 Monocytes nonactive 2 0.05039313 Monocytes_2
10x_3290_GAGGCAGAATGGTC-1 Patient6 Monocytes nonactive 2 0.05278676 Monocytes_2
10x_3290_GAGGCCACCGTAGT-1 Patient6 Progenitors active 6 0.15924799 Progenitors_6
10x_3290_GAGGGAACTCAGAC-1 Patient6 Progenitors active 6 0.1357872 Progenitors_6
10x_3290_GAGGGATGCTCGCT-1 Patient6 Monocytes nonactive 3 0.08266755 Monocytes_3
10x_3290_GAGGGCCTATGCCA-1 Patient6 Monocytes nonactive 3 0.03617399 Monocytes_3
10x_3290_GAGGGCCTCCTCAC-1 Patient6 Monocytes nonactive 3 0.05128083 Monocytes_3
10x_3290_GAGGGTGACGTAGT-1 Patient6 Monocytes nonactive 2 0.07367954 Monocytes_2
10x_3290_GAGGGTGACTGTAG-1 Patient6 Monocytes nonactive 3 0.05884218 Monocytes_3
10x_3290_GAGGTACTTGCTCC-1 Patient6 Monocytes nonactive 2 0.04070763 Monocytes_2
10x_3290_GAGGTGGACGCAAT-1 Patient6 Monocytes nonactive 2 0.05156617 Monocytes_2
10x_3290_GAGGTTACGCCCTT-1 Patient6 Monocytes nonactive 3 0.03926511 Monocytes_3
10x_3290_GAGGTTTGCGTACA-1 Patient6 Progenitors active 6 0.17322935

Progenitors_6
10x_3290_GAGGTTTGTCGCAA-1 Patient6 Monocytes nonactive 2 0.02969057
Monocytes_2
10x_3290_GAGGTTTGTGGATC-1 Patient6 Progenitors active 6 0.14341196
Progenitors_6
10x_3290_GAGTAAGAATAAGG-1 Patient6 Monocytes nonactive 3 0.06730708
Monocytes_3
10x_3290_GAGTACACCACTTT-1 Patient6 Monocytes nonactive 2 0.04295859
Monocytes_2
10x_3290_GAGTACTGCCGAAT-1 Patient6 Monocytes nonactive 2 0.01399721
Monocytes_2
10x_3290_GAGTACTGTGTTTC-1 Patient6 Progenitors active 6 0.12121933
Progenitors_6
10x_3290_GAGTCAACGTTCTT-1 Patient6 Progenitors nonactive 6 0.11584554
Progenitors_6
10x_3290_GAGTGACTGTGCTA-1 Patient6 Monocytes nonactive 3 0.03469977
Monocytes_3
10x_3290_GAGTGACTTTACTC-1 Patient6 B cells nonactive 7 0.06679982 B cells_7
10x_3290_GAGTTGTGGGTGGA-1 Patient6 Monocytes nonactive 2 0.01735781
Monocytes_2
10x_3290_GAGTTGTGTGTGCA-1 Patient6 Monocytes nonactive 2 0.04097711
Monocytes_2
10x_3290_GATAAGGAGTGTTG-1 Patient6 Monocytes nonactive 2 0.03607888
Monocytes_2
10x_3290_GATACTCTATTTCC-1 Patient6 Monocytes nonactive 3 0.03251221
Monocytes_3
10x_3290_GATACTCTGTACGT-1 Patient6 Monocytes nonactive 2 0.02753472
Monocytes_2
10x_3290_GATAGAGACCACAA-1 Patient6 Monocytes nonactive 3 0.03427176
Monocytes_3
10x_3290_GATAGCACTGTTCT-1 Patient6 Progenitors active 6 0.16245007
Progenitors_6
10x_3290_GATATAACTGTGAC-1 Patient6 Monocytes nonactive 3 0.02539471

Monocytes_3
10x_3290_GATATATGGTCACA-1 Patient6 Progenitors active 6 0.14391922
Progenitors_6
10x_3290_GATATCCTTTGCGA-1 Patient6 Monocytes nonactive 2 0.04841164
Monocytes_2
10x_3290_GATATTGAAGAGTA-1 Patient6 Monocytes nonactive 2 0.04511445
Monocytes_2
10x_3290_GATCATCTTTGCAG-1 Patient6 Monocytes nonactive 2 0.05121742
Monocytes_2
10x_3290_GATCCCTGCGACAT-1 Patient6 Monocytes nonactive 3 0.04842749
Monocytes_3
10x_3290_GATCCGCTCAGAAA-1 Patient6 Progenitors nonactive 6 0.09254328
Progenitors_6
10x_3290_GATCCGCTTCGTTT-1 Patient6 Monocytes nonactive 2 0.05072602
Monocytes_2
10x_3290_GATCGATGCCCTTG-1 Patient6 Monocytes nonactive 3 0.03347917
Monocytes_3
10x_3290_GATCTACTTGGCAT-1 Patient6 Monocytes nonactive 3 0.00721261
Monocytes_3
10x_3290_GATCTTACAAGGGC-1 Patient6 Monocytes nonactive 3 0.02946864
Monocytes_3
10x_3290_GATCTTACCAAGCT-1 Patient6 Monocytes nonactive 3 0.01543973
Monocytes_3
10x_3290_GATCTTACCACTGA-1 Patient6 Progenitors nonactive 6 0.11069368
Progenitors_6
10x_3290_GATGACACGCGAGA-1 Patient6 Monocytes nonactive 3 0.04966394
Monocytes_3
10x_3290_GATGACACGGTGAG-1 Patient6 Monocytes nonactive 3 0.0147264
Monocytes_3
10x_3290_GATGCAACAGAGTA-1 Patient6 Monocytes nonactive 2 0.03926511
Monocytes_2
10x_3290_GATGCAACTCTAGG-1 Patient6 Progenitors active 6 0.13030245
Progenitors_6

10x_3290_GATGCATGCTAGAC-1 Patient6 Monocytes nonactive 3 0.05156617 Monocytes_3
10x_3290_GATTGGACTGCATG-1 Patient6 Progenitors nonactive 6 0.11682836 Progenitors_6
10x_3290_GATTGGTGTTTCAC-1 Patient6 Monocytes nonactive 2 0.0020766 Monocytes_2
10x_3290_GATTTAGAATCAGC-1 Patient6 Progenitors active 6 0.12811489 Progenitors_6
10x_3290_GATTTGCTCCAATG-1 Patient6 Monocytes nonactive 2 0.02040137 Monocytes_2
10x_3290_GCAACCCTGTCTAG-1 Patient6 Monocytes nonactive 2 0.04960053 Monocytes_2
10x_3290_GCAACTGATCGATG-1 Patient6 Monocytes nonactive 3 0.07334665 Monocytes_3
10x_3290_GCAAGACTCACTCC-1 Patient6 Monocytes nonactive 2 0.05311965 Monocytes_2
10x_3290_GCAATCGATCGTGA-1 Patient6 Monocytes nonactive 2 0.02788346 Monocytes_2
10x_3290_GCACAAACCACTCC-1 Patient6 Monocytes nonactive 2 0.03810792 Monocytes_2
10x_3290_GCACAAACGGAGTG-1 Patient6 Progenitors active 6 0.13123771 Progenitors_6
10x_3290_GCACAAACTAGACC-1 Patient6 Monocytes nonactive 2 0.03728362 Monocytes_2
10x_3290_GCACAATGGTCATG-1 Patient6 Monocytes nonactive 2 0.03190983 Monocytes_2
10x_3290_GCACCTTGCGTACA-1 Patient6 Monocytes nonactive 2 0.05681314 Monocytes_2
10x_3290_GCACGGACCGCTAA-1 Patient6 Progenitors active 6 0.17736669 Progenitors_6
10x_3290_GCACGGTGTATTCC-1 Patient6 Monocytes nonactive 2 0.05410247 Monocytes_2
10x_3290_GCACGGTGTCTTAC-1 Patient6 Monocytes nonactive 2 0.04668379

Monocytes_2
10x_3290_GCACTGCTAAGGGC-1 Patient6 Monocytes nonactive 2 0.04887135
Monocytes_2
10x_3290_GCAGCCGACCGTTC-1 Patient6 Monocytes nonactive 3 0.05226365
Monocytes_3
10x_3290_GCAGCGTGATTTCC-1 Patient6 Monocytes nonactive 2 0.04167459
Monocytes_2
10x_3290_GCAGCTCTACAGCT-1 Patient6 Monocytes nonactive 3 0.01413988
Monocytes_3
10x_3290_GCAGGGCTAGCCAT-1 Patient6 Monocytes nonactive 2 0.0236193
Monocytes_2
10x_3290_GCAGGGCTAGGAGC-1 Patient6 Progenitors active 6 0.13396424
Progenitors_6
10x_3290_GCAGTCCTCTTGAG-1 Patient6 Monocytes nonactive 2 0.04018452
Monocytes_2
10x_3290_GCAGTCCTTGCTAG-1 Patient6 Monocytes nonactive 2 0.03638006
Monocytes_2
10x_3290_GCAGTCCTTGGTAC-1 Patient6 Monocytes nonactive 2 0.03341576
Monocytes_2
10x_3290_GCATGATGTTATCC-1 Patient6 Monocytes nonactive 3 0.06145774
Monocytes_3
10x_3290_GCATTGGACAATCG-1 Patient6 Monocytes nonactive 2 0.0535001
Monocytes_2
10x_3290_GCCAAAACCGCTAA-1 Patient6 Monocytes nonactive 2 0.02564834
Monocytes_2
10x_3290_GCCAACCTACTTTC-1 Patient6 Progenitors active 6 0.1564105
Progenitors_6
10x_3290_GCCACTACAGTCGT-1 Patient6 Monocytes nonactive 2 0.07190413
Monocytes_2
10x_3290_GCCCAACTGGAAGC-1 Patient6 Monocytes nonactive 2 0.03809207
Monocytes_2
10x_3290_GCCGACGACTGAGT-1 Patient6 Monocytes nonactive 2 0.03135502
Monocytes_2

10x_3290_GCCGAGTGTCTCCG-1 Patient6 Monocytes nonactive 3 0.02087693 Monocytes_3
10x_3290_GCCGAGTGTGTAGC-1 Patient6 Monocytes nonactive 2 0.0328451 Monocytes_2
10x_3290_GCCGTACTAAGCAA-1 Patient6 Monocytes nonactive 2 0.05283432 Monocytes_2
10x_3290_GCCGTACTGAGGAC-1 Patient6 Monocytes nonactive 2 0.02748716 Monocytes_2
10x_3290_GCCTCAACCAGGAG-1 Patient6 Monocytes nonactive 3 0.06277344 Monocytes_3
10x_3290_GCGAGAGAGTCATG-1 Patient6 Monocytes nonactive 3 0.02980153 Monocytes_3
10x_3290_GCGAGCACCCTTCG-1 Patient6 Progenitors active 6 0.12047429 Progenitors_6
10x_3290_GCGAGCACGGACTT-1 Patient6 Monocytes nonactive 2 0.02255723 Monocytes_2
10x_3290_GCGCACGAGCTTAG-1 Patient6 Monocytes nonactive 3 0.04159533 Monocytes_3
10x_3290_GCGCATCTCAACCA-1 Patient6 Monocytes nonactive 3 0.06115655 Monocytes_3
10x_3290_GCGCATCTGACGAG-1 Patient6 Monocytes nonactive 3 0.03273413 Monocytes_3
10x_3290_GCGCATCTGCTTAG-1 Patient6 Monocytes nonactive 3 0.07228457 Monocytes_3
10x_3290_GCGCATCTTAGTCG-1 Patient6 Monocytes nonactive 2 0.05071016 Monocytes_2
10x_3290_GCGCGAACTCTATC-1 Patient6 Monocytes nonactive 3 0.06221863 Monocytes_3
10x_3290_GCGCGATGAGTGTC-1 Patient6 Monocytes nonactive 3 0.04367193 Monocytes_3
10x_3290_GCGGACTGGTTGCA-1 Patient6 Monocytes nonactive 2 0.00434342 Monocytes_2
10x_3290_GCGGAGCTATTCCT-1 Patient6 Monocytes nonactive 3 0.05847759

Monocytes_3
10x_3290_GCGTACCTGTCATG-1 Patient6 T cells nonactive 9 0.06290026 T cells_9
10x_3290_GCTAGAACCTATTC-1 Patient6 Monocytes nonactive 3 0.06018959
Monocytes_3
10x_3290_GCTAGAACTCGTGA-1 Patient6 Monocytes nonactive 2 0.05743136
Monocytes_2
10x_3290_GCTAGATGAGGGTG-1 Patient6 Progenitors active 6 0.13247416
Progenitors_6
10x_3290_GCTAGATGTCCCGT-1 Patient6 Progenitors active 6 0.14582144
Progenitors_6
10x_3290_GCTAGATGTTGCGA-1 Patient6 Monocytes nonactive 3 0.03517532
Monocytes_3
10x_3290_GCTATACTCTCTAT-1 Patient6 Progenitors active 6 0.12321666
Progenitors_6
10x_3290_GCTATACTGACTAC-1 Patient6 Progenitors active 6 0.13936973
Progenitors_6
10x_3290_GCTCAAGAAGTAGA-1 Patient6 Monocytes nonactive 2 0.0400577
Monocytes_2
10x_3290_GCTCAAGAGTCATG-1 Patient6 Monocytes nonactive 3 0.0231913
Monocytes_3
10x_3290_GCTCAGCTCTGGTA-1 Patient6 Monocytes nonactive 2 0.05847759
Monocytes_2
10x_3290_GGAACACTTCTTTG-1 Patient6 Monocytes nonactive 3 0.0648976
Monocytes_3
10x_3290_GGAAGGACGGACTT-1 Patient6 Monocytes nonactive 2 0.0354131
Monocytes_2
10x_3290_GGAATCTGCGTCTC-1 Patient6 Monocytes nonactive 3 0.06499271
Monocytes_3
10x_3290_GGAATCTGTAGCCA-1 Patient6 Monocytes nonactive 2 0.03454125
Monocytes_2
10x_3290_GGAATGCTATTTCC-1 Patient6 Progenitors active 6 0.12586393
Progenitors_6
10x_3290_GGAATGCTCCTGAA-1 Patient6 Progenitors nonactive 6 0.10806227

Progenitors_6
10x_3290_GGACCCGACGGGAA-1 Patient6 Monocytes nonactive 2 0.0130778 Monocytes_2
10x_3290_GGACCTCTCAATCG-1 Patient6 Monocytes nonactive 2 0.05996766 Monocytes_2
10x_3290_GGACGCACAGTCTG-1 Patient6 Progenitors nonactive 6 0.09374802 Progenitors_6
10x_3290_GGACGCACTATCTC-1 Patient6 Progenitors active 6 0.13554943 Progenitors_6
10x_3290_GGACGCTGCTGCTC-1 Patient6 Monocytes nonactive 3 0.04414749 Monocytes_3
10x_3290_GGACTATGACTTTC-1 Patient6 Monocytes nonactive 3 0.0459229 Monocytes_3
10x_3290_GGACTATGTCACGA-1 Patient6 Monocytes nonactive 3 0.02203411 Monocytes_3
10x_3290_GGAGACGAGAAGGC-1 Patient6 Monocytes nonactive 3 0.07651702 Monocytes_3
10x_3290_GGAGACGAGTCTTT-1 Patient6 Monocytes nonactive 2 0.03725192 Monocytes_2
10x_3290_GGAGACGATGGAGG-1 Patient6 Monocytes nonactive 2 0.05036142 Monocytes_2
10x_3290_GGAGCAGAGATAAG-1 Patient6 Monocytes nonactive 3 0.04474986 Monocytes_3
10x_3290_GGAGCAGATGAACC-1 Patient6 Monocytes nonactive 3 0.03831399 Monocytes_3
10x_3290_GGAGCCACGACAGG-1 Patient6 Monocytes nonactive 2 0.03133917 Monocytes_2
10x_3290_GGAGCCACGAGATA-1 Patient6 Monocytes nonactive 3 0.06093463 Monocytes_3
10x_3290_GGAGCCACTGCAAC-1 Patient6 T cells nonactive 9 0.07193583 T cells_9
10x_3290_GGAGCGCTTACGCA-1 Patient6 Monocytes nonactive 3 0.04565341 Monocytes_3
10x_3290_GGAGGCCTGCATCA-1 Patient6 Monocytes nonactive 3 0.03443028

Monocytes_3

10x_3290_GGAGGTGAGCCTTC-1 Patient6 Progenitors active 6 0.12966838 Progenitors_6

10x_3290_GGATGTACAGTGTC-1 Patient6 Progenitors active 6 0.12811489 Progenitors_6

10x_3290_GGATGTACGCTTAG-1 Patient6 Monocytes nonactive 2 0.02750301 Monocytes_2

10x_3290_GGATGTACTTTCAC-1 Patient6 Monocytes nonactive 2 0.04582779 Monocytes_2

10x_3290_GGATGTTGGGTATC-1 Patient6 Monocytes nonactive 2 0.06919346 Monocytes_2

10x_3290_GGCAAGGAAAGGCG-1 Patient6 Monocytes nonactive 2 0.02892968 Monocytes_2

10x_3290_GGCAAGGACCTTGC-1 Patient6 Monocytes nonactive 3 0.05422928 Monocytes_3

10x_3290_GGCAAGGACGCAAT-1 Patient6 Monocytes nonactive 2 0.02463382 Monocytes_2

10x_3290_GGCAAGGACTTCGC-1 Patient6 Monocytes nonactive 2 0.02892968 Monocytes_2

10x_3290_GGCAAGGATGAGAA-1 Patient6 Monocytes nonactive 2 0.04243548 Monocytes_2

10x_3290_GGCAATACCGATAC-1 Patient6 Monocytes nonactive 3 0.03907488 Monocytes_3

10x_3290_GGCATATGAGGTCT-1 Patient6 Monocytes nonactive 2 0.03145013 Monocytes_2

10x_3290_GGCCAGACCAACTG-1 Patient6 Monocytes nonactive 3 0.03634836 Monocytes_3

10x_3290_GGCCCAGACCGCTT-1 Patient6 Monocytes nonactive 2 0.02182804 Monocytes_2

10x_3290_GGCCGAACAGGAGC-1 Patient6 Monocytes nonactive 2 0.05980914 Monocytes_2

10x_3290_GGCCGAACCGACAT-1 Patient6 Monocytes nonactive 3 0.06892397 Monocytes_3

10x_3290_GGCCGATGAACCAC-1 Patient6 Monocytes nonactive 2 0.0383774 Monocytes_2
10x_3290_GGCCGATGGAAGGC-1 Patient6 Monocytes nonactive 2 0.03493754 Monocytes_2
10x_3290_GGCGACACGTATGC-1 Patient6 Progenitors active 6 0.12042673 Progenitors_6
10x_3290_GGCGACACGTTGAC-1 Patient6 T cells nonactive 9 0.06654619 T cells_9
10x_3290_GGCGGACTCCAACA-1 Patient6 Monocytes nonactive 2 0.05253313 Monocytes_2
10x_3290_GGCTAATGCTTGGA-1 Patient6 Progenitors active 6 0.12830512 Progenitors_6
10x_3290_GGCTAATGGTAGGG-1 Patient6 Progenitors active 6 0.14234988 Progenitors_6
10x_3290_GGGAAGACTCAAGC-1 Patient6 Progenitors active 6 0.14065373 Progenitors_6
10x_3290_GGGAAGTGAGAAGT-1 Patient6 Monocytes nonactive 2 0.02637753 Monocytes_2
10x_3290_GGGACCACAGATCC-1 Patient6 Monocytes nonactive 3 0.05866781 Monocytes_3
10x_3290_GGGATTACCCGTAA-1 Patient6 Monocytes nonactive 2 0.02831146 Monocytes_2
10x_3290_GGGCAAGATAGCCA-1 Patient6 Monocytes nonactive 2 0.01425084 Monocytes_2
10x_3290_GGGCACACCCCACT-1 Patient6 Monocytes nonactive 2 0.04102467 Monocytes_2
10x_3290_GGGCAGCTTCACCC-1 Patient6 Monocytes nonactive 2 0.04857016 Monocytes_2
10x_3290_GGGTTAACAAGTGA-1 Patient6 Progenitors active 6 0.15224146 Progenitors_6
10x_3290_GGGTTAACTGATGC-1 Patient6 Progenitors active 6 0.14494959 Progenitors_6
10x_3290_GGTACATGGTCTTT-1 Patient6 Monocytes nonactive 2 0.00773572 Monocytes_2

10x_3290_GGTACTGATCGACA-1 Patient6 Monocytes nonactive 2 0.03856762 Monocytes_2
10x_3290_GGTATCGAGAACCT-1 Patient6 Monocytes nonactive 2 0.02341323 Monocytes_2
10x_3290_GGTATCGATGGTTG-1 Patient6 Progenitors nonactive 6 0.1125325 Progenitors_6
10x_3290_GGTATGACCTGAGT-1 Patient6 Monocytes nonactive 2 0.04409993 Monocytes_2
10x_3290_GGTCTAGATGGTTG-1 Patient6 Progenitors active 6 0.14276203 Progenitors_6
10x_3290_GGTGATACGGATCT-1 Patient6 Monocytes nonactive 3 0.06935198 Monocytes_3
10x_3290_GGTTGAACGCTATG-1 Patient6 Progenitors active 6 0.13128527 Progenitors_6
10x_3290_GGTTGAACGTCTAG-1 Patient6 Monocytes nonactive 3 0.03347917 Monocytes_3
10x_3290_GGTTTACTTTCGCC-1 Patient6 Monocytes nonactive 2 0.02455456 Monocytes_2
10x_3290_GTAACGTGTACGCA-1 Patient6 Monocytes nonactive 2 0.04189652 Monocytes_2
10x_3290_GTAAGCACGTTCGA-1 Patient6 Monocytes nonactive 2 0.06050663 Monocytes_2
10x_3290_GTAATAACCTCTAT-1 Patient6 Monocytes nonactive 2 0.02878701 Monocytes_2
10x_3290_GTACCCTGTTATCC-1 Patient6 Progenitors active 6 0.13079386 Progenitors_6
10x_3290_GTACGTGAACGTAC-1 Patient6 Monocytes nonactive 2 0.01456788 Monocytes_2
10x_3290_GTACTACTAGTCGT-1 Patient6 T cells active 9 0.13378987 T cells_9
10x_3290_GTACTTTGCGATAC-1 Patient6 Monocytes nonactive 2 0.05280261 Monocytes_2
10x_3290_GTAGCCCTCTAGAC-1 Patient6 Monocytes nonactive 3 0.05246972 Monocytes_3

10x_3290_GTAGCTGACTCGAA-1 Patient6 Monocytes nonactive 2 0.08323822 Monocytes_2
10x_3290_GTAGTCGACCTTCG-1 Patient6 Monocytes nonactive 3 0.06369285 Monocytes_3
10x_3290_GTAGTCGATGGATC-1 Patient6 Monocytes nonactive 2 0.04221356 Monocytes_2
10x_3290_GTAGTGACCCTCCA-1 Patient6 Monocytes nonactive 3 0.05839833 Monocytes_3
10x_3290_GTATCACTTCCTCG-1 Patient6 Monocytes nonactive 2 0.04947372 Monocytes_2
10x_3290_GTCAACGAGTAGCT-1 Patient6 Monocytes nonactive 3 0.04479741 Monocytes_3
10x_3290_GTCACAGAAACGTC-1 Patient6 Monocytes nonactive 2 0.02181219 Monocytes_2
10x_3290_GTCACCTGACCCTC-1 Patient6 Progenitors active 6 0.18180521 Progenitors_6
10x_3290_GTCACCTGCCTTCG-1 Patient6 Monocytes nonactive 3 0.05804958 Monocytes_3
10x_3290_GTCACCTGTATTCC-1 Patient6 Monocytes nonactive 3 0.0413417 Monocytes_3
10x_3290_GTCATACTAAGATG-1 Patient6 Monocytes nonactive 3 0.01781751 Monocytes_3
10x_3290_GTCCACACCCTTAT-1 Patient6 Monocytes nonactive 2 0.07084205 Monocytes_2
10x_3290_GTCCACTGGGAGGT-1 Patient6 Monocytes nonactive 3 0.05050409 Monocytes_3
10x_3290_GTCCCATGTCTACT-1 Patient6 Monocytes nonactive 3 0.03676051 Monocytes_3
10x_3290_GTCGAATGGGTGGA-1 Patient6 Progenitors active 6 0.1281466 Progenitors_6
10x_3290_GTCGAATGTTGGCA-1 Patient6 Monocytes nonactive 2 0.04815801 Monocytes_2
10x_3290_GTCGCACTAGCATC-1 Patient6 Progenitors active 6 0.12819415

Progenitors_6

10x_3290_GTCTAACTGGAGTG-1 Patient6 Monocytes nonactive 2 0.06299537 Monocytes_2

10x_3290_GTCTGAGAGCGAGA-1 Patient6 Monocytes nonactive 3 0.05787521 Monocytes_3

10x_3290_GTCTGAGAGTGTCA-1 Patient6 Monocytes nonactive 2 0.02334982 Monocytes_2

10x_3290_GTCTGAGATTCTAC-1 Patient6 Monocytes nonactive 3 0.0459546 Monocytes_3

10x_3290_GTGAACACCTATTC-1 Patient6 Monocytes nonactive 2 0.04998098 Monocytes_2

10x_3290_GTGAACACGTCACA-1 Patient6 Progenitors active 6 0.16563629 Progenitors_6

10x_3290_GTGAACACTATGCG-1 Patient6 Monocytes nonactive 2 0.05804958 Monocytes_2

10x_3290_GTGACCCTCTTTAC-1 Patient6 Monocytes nonactive 2 0.03565088 Monocytes_2

10x_3290_GTGAGGGACTACCC-1 Patient6 Monocytes nonactive 3 0.06107729 Monocytes_3

10x_3290_GTGATGACGCTTCC-1 Patient6 Progenitors active 6 0.15488872 Progenitors_6

10x_3290_GTGATTCTTGGAGG-1 Patient6 Progenitors nonactive 6 0.10457485 Progenitors_6

10x_3290_GTGCAAACTACTGG-1 Patient6 Monocytes nonactive 2 0.04890305 Monocytes_2

10x_3290_GTGCTAGACTTAGG-1 Patient6 Monocytes nonactive 3 0.0383774 Monocytes_3

10x_3290_GTGCTAGATAGTCG-1 Patient6 Monocytes nonactive 3 0.09645869 Monocytes_3

10x_3290_GTGTATCTACGCAT-1 Patient6 Monocytes nonactive 3 0.04402067 Monocytes_3

10x_3290_GTGTCAGAGCCAAT-1 Patient6 Monocytes nonactive 2 0.06705345 Monocytes_2

10x_3290_GTTACGGATGCACA-1	Patient6	Monocytes	nonactive	2	0.04831653	Monocytes_2
10x_3290_GTTACTACGGTGAG-1	Patient6	Monocytes	nonactive	3	0.07684991	Monocytes_3
10x_3290_GTTATCTGCGAACT-1	Patient6	Monocytes	nonactive	2	0.03813962	Monocytes_2
10x_3290_GTTATGCTATCGAC-1	Patient6	Monocytes	nonactive	2	0.05066261	Monocytes_2
10x_3290_GTTCATACTTGAGC-1	Patient6	Monocytes	nonactive	2	0.03141843	Monocytes_2
10x_3290_GTTGACGACCTTTA-1	Patient6	Progenitors	active	6	0.15964428	Progenitors_6
10x_3290_GTTGACGATCCCAC-1	Patient6	Monocytes	nonactive	2	0.03728362	Monocytes_2
10x_3290_GTTGAGTGCCCACT-1	Patient6	Progenitors	active	6	0.17173927	Progenitors_6
10x_3290_GTTGGATGACGGTT-1	Patient6	Monocytes	nonactive	3	0.06356604	Monocytes_3
10x_3290_TAAAGTTGACGTAC-1	Patient6	Monocytes	nonactive	3	0.06472323	Monocytes_3
10x_3290_TAAATCGAGACGAG-1	Patient6	Monocytes	nonactive	2	0.03067339	Monocytes_2
10x_3290_TAACAATGTGAAGA-1	Patient6	Monocytes	nonactive	2	0.0181504	Monocytes_2
10x_3290_TAACATGAAGTAGA-1	Patient6	Monocytes	nonactive	2	0.03335236	Monocytes_2
10x_3290_TAACATGAGTGTCA-1	Patient6	Progenitors	nonactive	6	0.11099486	Progenitors_6
10x_3290_TAACGTCTTAGTCG-1	Patient6	Monocytes	nonactive	2	0.04151607	Monocytes_2
10x_3290_TAACTAGATGGAAA-1	Patient6	Monocytes	nonactive	3	0.03557162	Monocytes_3
10x_3290_TAACTCACCTGTGA-1	Patient6	Monocytes	nonactive	2	0.05969818	

Monocytes_2
10x_3290_TAAGAGGAAAGCCT-1 Patient6 Monocytes nonactive 2 0.01379114
Monocytes_2
10x_3290_TAAGCTCTCAAGCT-1 Patient6 Monocytes nonactive 3 0.02290597
Monocytes_3
10x_3290_TAAGGCTGGTCACA-1 Patient6 Progenitors nonactive 6 0.10798301
Progenitors_6
10x_3290_TAAGTAACTTCCGC-1 Patient6 Monocytes nonactive 3 0.05009194
Monocytes_3
10x_3290_TAAGTCCTCCACAA-1 Patient6 Monocytes nonactive 3 0.06418426
Monocytes_3
10x_3290_TAATCCACTGTCCC-1 Patient6 Monocytes nonactive 2 0.05827151
Monocytes_2
10x_3290_TAATGTGAAGCGTT-1 Patient6 Progenitors active 6 0.13784795
Progenitors_6
10x_3290_TAATGTGAATTTCC-1 Patient6 Progenitors active 6 0.14796145
Progenitors_6
10x_3290_TACAAATGCGAGTT-1 Patient6 Progenitors active 6 0.14783463
Progenitors_6
10x_3290_TACCATTGCCCGTT-1 Patient6 Monocytes nonactive 2 0.04584364
Monocytes_2
10x_3290_TACCATTGGGTGTT-1 Patient6 Monocytes nonactive 2 0.03813962
Monocytes_2
10x_3290_TACCGAGATCCGAA-1 Patient6 Monocytes nonactive 3 0.02100374
Monocytes_3
10x_3290_TACCGGCTGAGCTT-1 Patient6 Monocytes nonactive 3 0.04444867
Monocytes_3
10x_3290_TACGATCTGTTCGA-1 Patient6 Monocytes nonactive 3 0.04799949
Monocytes_3
10x_3290_TACGCAGAATTTCC-1 Patient6 Monocytes nonactive 3 0.02295352
Monocytes_3
10x_3290_TACGCAGAGTACCA-1 Patient6 Monocytes nonactive 2 0.07325154
Monocytes_2

10x_3290_TACGCGCTAAAAGC-1 Patient6 Monocytes nonactive 2 0.01198402
Monocytes_2
10x_3290_TACGGAACGACAGG-1 Patient6 Monocytes nonactive 2 0.04658868
Monocytes_2
10x_3290_TACGGAACGAGGTG-1 Patient6 Progenitors active 6 0.14474352
Progenitors_6
10x_3290_TACGTACTAAAGTG-1 Patient6 Monocytes nonactive 3 0.02886627
Monocytes_3
10x_3290_TACGTACTCGACAT-1 Patient6 Monocytes nonactive 3 0.03441443
Monocytes_3
10x_3290_TACTAAGACTGGTA-1 Patient6 Monocytes nonactive 3 0.06909835
Monocytes_3
10x_3290_TACTACACGTATCG-1 Patient6 Monocytes nonactive 2 0.0299125
Monocytes_2
10x_3290_TACTACACTGTTCT-1 Patient6 Monocytes nonactive 2 0.03607888
Monocytes_2
10x_3290_TACTCAACCGAACT-1 Patient6 Monocytes nonactive 2 0.03138672
Monocytes_2
10x_3290_TACTCCCTAAGAAC-1 Patient6 Monocytes nonactive 2 0.01989411
Monocytes_2
10x_3290_TACTCTGAACGTGT-1 Patient6 Monocytes nonactive 3 0.06637182
Monocytes_3
10x_3290_TACTCTGACTTGTT-1 Patient6 Monocytes nonactive 2 0.08544163
Monocytes_2
10x_3290_TACTGGGAACCAAC-1 Patient6 Progenitors active 6 0.1289709
Progenitors_6
10x_3290_TACTTGACTGGATC-1 Patient6 Progenitors active 6 0.15366813
Progenitors_6
10x_3290_TAGAGAGATAGCCA-1 Patient6 Progenitors active 6 0.166096
Progenitors_6
10x_3290_TAGATCCTAGAGTA-1 Patient6 Monocytes nonactive 2 0.04486082
Monocytes_2
10x_3290_TAGATTGATTTGTC-1 Patient6 Monocytes nonactive 2 0.01810285

Monocytes_2
10x_3290_TAGCATCTACTGGT-1 Patient6 Progenitors nonactive 6 0.10744404
Progenitors_6
10x_3290_TAGCCGCTGGACTT-1 Patient6 Monocytes nonactive 2 0.03100628
Monocytes_2
10x_3290_TAGGACTGACGGAG-1 Patient6 Monocytes nonactive 3 0.03433517
Monocytes_3
10x_3290_TAGGCAACTCTAGG-1 Patient6 Monocytes nonactive 2 0.02548982
Monocytes_2
10x_3290_TAGGCTGAGGACGA-1 Patient6 Monocytes nonactive 3 0.04405237
Monocytes_3
10x_3290_TAGGCTGATATGCG-1 Patient6 Progenitors active 6 0.15801154
Progenitors_6
10x_3290_TAGGCTGATTTCGT-1 Patient6 Monocytes nonactive 2 0.04655697
Monocytes_2
10x_3290_TAGGGACTGTGCAT-1 Patient6 Monocytes nonactive 2 0.0240156
Monocytes_2
10x_3290_TAGGTCGATTGGCA-1 Patient6 T cells nonactive 9 0.06692664 T cells_9
10x_3290_TAGGTGACCACTTT-1 Patient6 Monocytes nonactive 2 0.02271574
Monocytes_2
10x_3290_TAGGTGTGGTCACA-1 Patient6 Monocytes nonactive 2 0.0535001
Monocytes_2
10x_3290_TAGGTGTGTGGCAT-1 Patient6 Monocytes nonactive 2 0.04433771
Monocytes_2
10x_3290_TAGGTTCTCTGATG-1 Patient6 Monocytes nonactive 3 0.0556718
Monocytes_3
10x_3290_TAGGTTCTCTGTAG-1 Patient6 Monocytes nonactive 2 0.06293196
Monocytes_2
10x_3290_TAGGTTCTTGTCTT-1 Patient6 Monocytes nonactive 2 0.07726206
Monocytes_2
10x_3290_TAGTAATGAAGGGC-1 Patient6 Monocytes nonactive 2 0.05105891
Monocytes_2
10x_3290_TAGTAATGCAACTG-1 Patient6 Monocytes nonactive 3 0.02598123

Monocytes_3
10x_3290_TAGTCACTACGGAG-1 Patient6 Monocytes nonactive 2 0.04768246 Monocytes_2
10x_3290_TAGTCACTGGAAAT-1 Patient6 Progenitors active 6 0.18063217 Progenitors_6
10x_3290_TAGTCGGATCCAGA-1 Patient6 Monocytes nonactive 3 0.07179316 Monocytes_3
10x_3290_TAGTCTTGGAAACA-1 Patient6 Monocytes nonactive 3 0.08325407 Monocytes_3
10x_3290_TAGTCTTGGTCATG-1 Patient6 Monocytes nonactive 2 0.02545812 Monocytes_2
10x_3290_TAGTCTTGTGAGGG-1 Patient6 Monocytes nonactive 2 0.04359267 Monocytes_2
10x_3290_TAGTGGTGAACCAC-1 Patient6 Monocytes nonactive 2 0.020655 Monocytes_2
10x_3290_TAGTTAGATCGTAG-1 Patient6 Monocytes nonactive 2 0.04306956 Monocytes_2
10x_3290_TAGTTGCTTCAGTG-1 Patient6 Monocytes nonactive 2 0.0337011 Monocytes_2
10x_3290_TATAAGACACGACT-1 Patient6 Monocytes nonactive 2 0.0152178 Monocytes_2
10x_3290_TATAAGACGACAAA-1 Patient6 Monocytes nonactive 3 0.03715681 Monocytes_3
10x_3290_TATAAGTGAACCGT-1 Patient6 Progenitors nonactive 6 0.07153953 Progenitors_6
10x_3290_TATAAGTGAGGCGA-1 Patient6 Monocytes nonactive 2 0.02739205 Monocytes_2
10x_3290_TATACAGAAGTCTG-1 Patient6 Monocytes nonactive 2 0.03991503 Monocytes_2
10x_3290_TATACCACTTTGGG-1 Patient6 Progenitors active 6 0.17823854 Progenitors_6
10x_3290_TATACGCTGGGTGA-1 Patient6 Monocytes nonactive 2 0.03766407 Monocytes_2

10x_3290_TATAGATGTTCTCA-1 Patient6 Monocytes nonactive 3 0.04922009 Monocytes_3
10x_3290_TATCAAGACCAAGT-1 Patient6 Monocytes nonactive 3 0.03435102 Monocytes_3
10x_3290_TATCACTGAATCGC-1 Patient6 Monocytes nonactive 3 0.04113563 Monocytes_3
10x_3290_TATCACTGCAGATC-1 Patient6 Monocytes nonactive 3 0.08688415 Monocytes_3
10x_3290_TATCAGCTCTACTT-1 Patient6 Monocytes nonactive 2 0.02426923 Monocytes_2
10x_3290_TATCCAACCTTTAC-1 Patient6 Monocytes nonactive 2 0.04143681 Monocytes_2
10x_3290_TATCCAACTGCTCC-1 Patient6 Progenitors active 6 0.17395853 Progenitors_6
10x_3290_TATCCTGAAACCAC-1 Patient6 Monocytes nonactive 2 0.09056179 Monocytes_2
10x_3290_TATCGTACAATGCC-1 Patient6 Progenitors nonactive 6 0.106271 Progenitors_6
10x_3290_TATCGTACTGCGTA-1 Patient6 Monocytes nonactive 2 0.03482658 Monocytes_2
10x_3290_TATCTCGAGAATGA-1 Patient6 Progenitors active 6 0.12369222 Progenitors_6
10x_3290_TATGCGGAAGTACC-1 Patient6 Monocytes nonactive 2 0.02899309 Monocytes_2
10x_3290_TATGCGGACTCAGA-1 Patient6 Monocytes nonactive 3 0.04833238 Monocytes_3
10x_3290_TATGCGGAGCAGTT-1 Patient6 Monocytes nonactive 3 0.00694312 Monocytes_3
10x_3290_TATGCGGAGTTCGA-1 Patient6 Progenitors active 6 0.14972101 Progenitors_6
10x_3290_TATGGGTGGGACTT-1 Patient6 Monocytes nonactive 2 0.03349502 Monocytes_2
10x_3290_TATGGGTGTGCTTT-1 Patient6 Monocytes nonactive 2 0.03852007

Monocytes_2
10x_3290_TATGTCACTCTCTA-1	Patient6 Progenitors	active	6	0.13596158
Progenitors_6
10x_3290_TATGTCACTGCTGA-1	Patient6 Monocytes	nonactive	2	0.05630588
Monocytes_2
10x_3290_TATGTCTGGTGCAT-1	Patient6 Monocytes	nonactive	3	0.0645013
Monocytes_3
10x_3290_TATGTCTGGTTGGT-1	Patient6 Monocytes	nonactive	3	0.04029548
Monocytes_3
10x_3290_TATGTGCTGACAAA-1	Patient6 Progenitors	active	6	0.15609346
Progenitors_6
10x_3290_TATTGCTGCGTCTC-1	Patient6 Monocytes	nonactive	2	0.04562171
Monocytes_2
10x_3290_TCAACACTTCGACA-1	Patient6 Monocytes	nonactive	3	0.03525458
Monocytes_3
10x_3290_TCAAGGACATCAGC-1	Patient6 Monocytes	nonactive	2	0.03875785
Monocytes_2
10x_3290_TCAAGTCTTTTCTG-1	Patient6 Monocytes	nonactive	3	0.03611058
Monocytes_3
10x_3290_TCAATAGACCGTTC-1	Patient6 Monocytes	nonactive	2	0.03064168
Monocytes_2
10x_3290_TCACAACTCCACAA-1	Patient6 Monocytes	nonactive	3	0.05120157
Monocytes_3
10x_3290_TCACAACTCTAGCA-1	Patient6 Progenitors	active	1	0.14065373
Progenitors_1
10x_3290_TCACATACAGGGTG-1	Patient6 Monocytes	nonactive	2	0.05121742
Monocytes_2
10x_3290_TCACATACGTACCA-1	Patient6 Monocytes	nonactive	2	0.04178556
Monocytes_2
10x_3290_TCACCCGAACGTGT-1	Patient6 Monocytes	nonactive	3	0.06423182
Monocytes_3
10x_3290_TCACCCGAGTGCAT-1	Patient6 Monocytes	nonactive	2	0.061109
Monocytes_2

10x_3290_TCACTATGTCGACA-1 Patient6 Progenitors active 6 0.15293894
Progenitors_6
10x_3290_TCAGACGAAGAACA-1 Patient6 Monocytes nonactive 3 0.02327056
Monocytes_3
10x_3290_TCAGAGACGCATCA-1 Patient6 Monocytes nonactive 3 0.05189906
Monocytes_3
10x_3290_TCAGGATGCCTATT-1 Patient6 Monocytes nonactive 2 0.04348171
Monocytes_2
10x_3290_TCAGTACTAGGTTC-1 Patient6 Monocytes nonactive 3 0.05516454
Monocytes_3
10x_3290_TCAGTACTCGATAC-1 Patient6 Monocytes nonactive 3 0.06399404
Monocytes_3
10x_3290_TCAGTTACAGGGTG-1 Patient6 Monocytes nonactive 3 0.04073933
Monocytes_3
10x_3290_TCATCCCTGGTGTT-1 Patient6 Progenitors active 6 0.14588485
Progenitors_6
10x_3290_TCATGTACGAGCTT-1 Patient6 Monocytes nonactive 2 0.02165367
Monocytes_2
10x_3290_TCATGTACTCGTGA-1 Patient6 Monocytes nonactive 2 0.02938939
Monocytes_2
10x_3290_TCCACGTGAGCTCA-1 Patient6 Monocytes nonactive 2 0.03809207
Monocytes_2
10x_3290_TCCACGTGGGTCAT-1 Patient6 Monocytes nonactive 2 0.01875277
Monocytes_2
10x_3290_TCCATCCTCTATTC-1 Patient6 Monocytes nonactive 3 0.09094224
Monocytes_3
10x_3290_TCCCACGAAAGTGA-1 Patient6 Monocytes nonactive 3 0.01063661
Monocytes_3
10x_3290_TCCCACGATGGTAC-1 Patient6 Monocytes nonactive 3 0.041738
Monocytes_3
10x_3290_TCCCAGACATACCG-1 Patient6 Monocytes nonactive 2 0.03232198
Monocytes_2
10x_3290_TCCCATCTGCCTTC-1 Patient6 Monocytes nonactive 3 0.02856509

Monocytes_3
10x_3290_TCCCGAACAGAACA-1 Patient6 Monocytes nonactive 3 0.06121996
Monocytes_3
10x_3290_TCCCGAACCGAGAG-1 Patient6 Monocytes nonactive 2 0.01849914
Monocytes_2
10x_3290_TCCCGATGAATCGC-1 Patient6 Monocytes nonactive 2 0.04031133
Monocytes_2
10x_3290_TCCCTACTCTAAGC-1 Patient6 Monocytes nonactive 2 0.01672373
Monocytes_2
10x_3290_TCCCTACTTTCTTG-1 Patient6 Monocytes nonactive 2 0.02498256
Monocytes_2
10x_3290_TCCGAAGAACCTCC-1 Patient6 Progenitors active 6 0.13076216
Progenitors_6
10x_3290_TCCGAAGACTCATT-1 Patient6 Monocytes nonactive 2 0.06182233
Monocytes_2
10x_3290_TCCGAGCTAGTACC-1 Patient6 Monocytes nonactive 3 0.04188067
Monocytes_3
10x_3290_TCCTACCTGACGAG-1 Patient6 Monocytes nonactive 3 0.03774333
Monocytes_3
10x_3290_TCCTACCTTCCAGA-1 Patient6 Progenitors nonactive 6 0.08539408
Progenitors_6
10x_3290_TCCTATGAAGAGGC-1 Patient6 Progenitors active 6 0.14796145
Progenitors_6
10x_3290_TCCTATGACCCTTG-1 Patient6 Monocytes nonactive 2 0.07691332
Monocytes_2
10x_3290_TCGAGCCTACTCTT-1 Patient6 Monocytes nonactive 2 0.02680553
Monocytes_2
10x_3290_TCGAGCCTCTCAAG-1 Patient6 Progenitors nonactive 6 0.11451398
Progenitors_6
10x_3290_TCGATACTAACGTC-1 Patient6 B cells nonactive 7 0.05747892 B cells_7
10x_3290_TCGCACACCCTTGC-1 Patient6 Progenitors active 6 0.15290723
Progenitors_6
10x_3290_TCGCCATGGTTGCA-1 Patient6 Monocytes nonactive 3 0.06328071

Monocytes_3
10x_3290_TCGCCATGTCGCCT-1 Patient6 Progenitors active 6 0.17248431
Progenitors_6
10x_3290_TCGGACCTAGCAAA-1 Patient6 Monocytes nonactive 2 0.04040644
Monocytes_2
10x_3290_TCGGACCTGGACAG-1 Patient6 Monocytes nonactive 3 0.04688986
Monocytes_3
10x_3290_TCGGACCTTTGCAG-1 Patient6 Monocytes nonactive 2 0.05131254
Monocytes_2
10x_3290_TCGGCACTCTTCCG-1 Patient6 Monocytes nonactive 2 0.02339737
Monocytes_2
10x_3290_TCGGTAGACGATAC-1 Patient6 Monocytes nonactive 3 0.03639592
Monocytes_3
10x_3290_TCGTAGGATGTAGC-1 Patient6 Progenitors active 6 0.17050282
Progenitors_6
10x_3290_TCGTTATGCAAAGA-1 Patient6 Monocytes nonactive 2 0.04581193
Monocytes_2
10x_3290_TCGTTATGTATGCG-1 Patient6 Progenitors active 6 0.16550948
Progenitors_6
10x_3290_TCTAACTGCGACAT-1 Patient6 Monocytes nonactive 2 0.0417697
Monocytes_2
10x_3290_TCTAGACTATGCTG-1 Patient6 Monocytes nonactive 3 0.01314121
Monocytes_3
10x_3290_TCTAGTTGCCGAAT-1 Patient6 Monocytes nonactive 2 0.03813962
Monocytes_2
10x_3290_TCTATGTGCCACCT-1 Patient6 Monocytes nonactive 3 0.04708008
Monocytes_3
10x_3290_TCTCAAACCAGCTA-1 Patient6 Progenitors active 6 0.13217298
Progenitors_6
10x_3290_TCTCAAACCGAGTT-1 Patient6 Monocytes nonactive 3 0.05457802
Monocytes_3
10x_3290_TCTCAAACGCATCA-1 Patient6 Progenitors active 6 0.11801725
Progenitors_6

10x_3290_TCTGATACAAACAG-1 Patient6 Monocytes nonactive 2 0.03734703 Monocytes_2
10x_3290_TCTGATACCTGTCC-1 Patient6 Progenitors active 6 0.14390337 Progenitors_6
10x_3290_TCTTACGAACTGGT-1 Patient6 Monocytes nonactive 16 0.03595206 Monocytes_16
10x_3290_TGAATAACTCTCAT-1 Patient6 Monocytes nonactive 2 0.03785429 Monocytes_2
10x_3290_TGAATAACTTGTCT-1 Patient6 Progenitors active 6 0.12545178 Progenitors_6
10x_3290_TGACCAGAGAAGGC-1 Patient6 Monocytes nonactive 3 0.0535318 Monocytes_3
10x_3290_TGACCGCTCCGATA-1 Patient6 Monocytes nonactive 2 0.04960053 Monocytes_2
10x_3290_TGACCGCTTTACTC-1 Patient6 Monocytes nonactive 2 0.06714856 Monocytes_2
10x_3290_TGACTGGATAGAGA-1 Patient6 Monocytes nonactive 3 0.05143935 Monocytes_3
10x_3290_TGACTTACCGTACA-1 Patient6 B cells nonactive 7 0.0674339 B cells_7
10x_3290_TGACTTTGAGCGGA-1 Patient6 Monocytes nonactive 3 0.0332731 Monocytes_3
10x_3290_TGACTTTGCTGGTA-1 Patient6 Monocytes nonactive 3 0.03867859 Monocytes_3
10x_3290_TGACTTTGTTGAGC-1 Patient6 Monocytes nonactive 3 0.02173293 Monocytes_3
10x_3290_TGAGCTGACTGTGA-1 Patient6 Monocytes nonactive 3 0.05838247 Monocytes_3
10x_3290_TGAGGTACACCTAG-1 Patient6 Progenitors active 6 0.12809904 Progenitors_6
10x_3290_TGATACCTTGAACC-1 Patient6 Monocytes nonactive 16 0.06504026 Monocytes_16
10x_3290_TGATACCTTGCACA-1 Patient6 Monocytes nonactive 3 0.03607888 Monocytes_3

10x_3290_TGATCGGAATCTCT-1 Patient6 Monocytes nonactive 3 0.04075518 Monocytes_3
10x_3290_TGATCGGACCAACA-1 Patient6 Progenitors nonactive 6 0.10668315 Progenitors_6
10x_3290_TGATTAGAAGGAGC-1 Patient6 Monocytes nonactive 2 0.05053579 Monocytes_2
10x_3290_TGATTCTGCTCGCT-1 Patient6 Monocytes nonactive 3 0.07619999 Monocytes_3
10x_3290_TGCAAGACGTTGAC-1 Patient6 Monocytes nonactive 3 0.04365608 Monocytes_3
10x_3290_TGCAATCTTGATGC-1 Patient6 Monocytes nonactive 2 0.01383869 Monocytes_2
10x_3290_TGCACAGAGGTGGA-1 Patient6 Monocytes nonactive 2 0.06836916 Monocytes_2
10x_3290_TGCACAGAGTTCAG-1 Patient6 Monocytes nonactive 3 0.04768246 Monocytes_3
10x_3290_TGCCACTGAAGCAA-1 Patient6 Monocytes nonactive 3 0.01873692 Monocytes_3
10x_3290_TGCCAGCTTTGTCT-1 Patient6 Monocytes nonactive 3 0.02449115 Monocytes_3
10x_3290_TGCCCAACCCGCTT-1 Patient6 Monocytes nonactive 2 0.02387293 Monocytes_2
10x_3290_TGCCGACTTCCTGC-1 Patient6 Progenitors active 6 0.13929047 Progenitors_6
10x_3290_TGCGAAACTTGCGA-1 Patient6 Monocytes nonactive 2 0.03068924 Monocytes_2
10x_3290_TGCGATGAATGCTG-1 Patient6 Monocytes nonactive 2 0.06559508 Monocytes_2
10x_3290_TGCGATGATGCGTA-1 Patient6 Monocytes nonactive 2 0.05161372 Monocytes_2
10x_3290_TGCGTAGAACTAGC-1 Patient6 Monocytes nonactive 2 0.04027963 Monocytes_2
10x_3290_TGCGTAGATAGAAG-1 Patient6 Monocytes nonactive 3 0.0350168

Monocytes_3
10x_3290_TGCTGAGAACGACT-1 Patient6 Monocytes nonactive 2 0.01830892
Monocytes_2
10x_3290_TGGAAAGAAAAGTG-1 Patient6 Monocytes nonactive 3 0.05389639
Monocytes_3
10x_3290_TGGAAAGATTGCAG-1 Patient6 Monocytes nonactive 2 0.03815548
Monocytes_2
10x_3290_TGGAACTGAGTGCT-1 Patient6 Monocytes nonactive 2 0.00670535
Monocytes_2
10x_3290_TGGAACTGTTACCT-1 Patient6 Progenitors active 6 0.15209879
Progenitors_6
10x_3290_TGGAAGCTCTGTCC-1 Patient6 Monocytes nonactive 3 0.06465982
Monocytes_3
10x_3290_TGGACTGATTGTCT-1 Patient6 Progenitors nonactive 6 0.09685499
Progenitors_6
10x_3290_TGGAGACTCATTCT-1 Patient6 Progenitors active 6 0.14678841
Progenitors_6
10x_3290_TGGAGACTTTCAGG-1 Patient6 Monocytes nonactive 3 0.06174307
Monocytes_3
10x_3290_TGGAGGGACTGACA-1 Patient6 Monocytes nonactive 3 0.04295859
Monocytes_3
10x_3290_TGGATTCTTCCTGC-1 Patient6 Monocytes nonactive 2 0.02924672
Monocytes_2
10x_3290_TGGGTATGTTTACC-1 Patient6 Progenitors active 6 0.16305244
Progenitors_6
10x_3290_TGGTACGATCAGGT-1 Patient6 Monocytes nonactive 2 0.03341576
Monocytes_2
10x_3290_TGGTAGTGTACGCA-1 Patient6 Monocytes nonactive 2 0.02850168
Monocytes_2
10x_3290_TGGTCAGATACGAC-1 Patient6 Monocytes nonactive 3 0.0586361
Monocytes_3
10x_3290_TGGTCAGATCAGAC-1 Patient6 Monocytes nonactive 2 0.02182804
Monocytes_2

10x_3290_TGGTCAGATGCTAG-1	Patient6	Monocytes	nonactive	2	0.01998922	Monocytes_2
10x_3290_TGGTTACTTTATCC-1	Patient6	Monocytes	nonactive	2	0.02835901	Monocytes_2
10x_3290_TGTAAAACAAAGCA-1	Patient6	Progenitors	active	6	0.14875404	Progenitors_6
10x_3290_TGTAGGTGCTACTT-1	Patient6	Monocytes	nonactive	2	0.04611312	Monocytes_2
10x_3290_TGTCAGGACCGTAA-1	Patient6	T cells	nonactive	9	0.08648786	T cells_9
10x_3290_TGTCAGGATCTTTG-1	Patient6	Progenitors	active	6	0.13650054	Progenitors_6
10x_3290_TGTGACGACTATGG-1	Patient6	Monocytes	nonactive	2	0.02602879	Monocytes_2
10x_3290_TGTGACGATTCATC-1	Patient6	Monocytes	nonactive	2	0.05058335	Monocytes_2
10x_3290_TGTGAGACTCCTCG-1	Patient6	Progenitors	active	6	0.18644981	Progenitors_6
10x_3290_TGTGATCTTTACTC-1	Patient6	Monocytes	nonactive	3	0.08607571	Monocytes_3
10x_3290_TGTTAAGAGTCCTC-1	Patient6	Monocytes	nonactive	2	0.03996259	Monocytes_2
10x_3290_TGTTACTGAGTGTC-1	Patient6	B cells	nonactive	7	0.04773001	B cells_7
10x_3290_TGTTACTGTACGCA-1	Patient6	Progenitors	active	6	0.15417539	Progenitors_6
10x_3290_TGTTACTGTGTCAG-1	Patient6	Monocytes	nonactive	2	0.03400228	Monocytes_2
10x_3290_TTACACACACACAC-1	Patient6	Monocytes	nonactive	2	0.07058842	Monocytes_2
10x_3290_TTACACACGTCGAT-1	Patient6	B cells	nonactive	7	0.04714349	B cells_7
10x_3290_TTACACACTGCATG-1	Patient6	Monocytes	nonactive	2	0.04395726	Monocytes_2
10x_3290_TTACAGCTCAAGCT-1	Patient6	Monocytes	nonactive	3	0.06023714	Monocytes_3

10x_3290_TTACAGCTGCAGTT-1 Patient6 Monocytes nonactive 3 0.04333904 Monocytes_3
10x_3290_TTACGACTATTTCC-1 Patient6 Progenitors active 6 0.12580052 Progenitors_6
10x_3290_TTACGTACCTGCAA-1 Patient6 Monocytes nonactive 3 0.05032972 Monocytes_3
10x_3290_TTACTCGACAAGCT-1 Patient6 Monocytes nonactive 3 0.04148437 Monocytes_3
10x_3290_TTACTCGATTCGGA-1 Patient6 Monocytes nonactive 2 0.03390717 Monocytes_2
10x_3290_TTAGAATGGGAGGT-1 Patient6 Monocytes nonactive 2 0.04503519 Monocytes_2
10x_3290_TTAGAATGTCGCCT-1 Patient6 Monocytes nonactive 3 0.04761905 Monocytes_3
10x_3290_TTAGGGACCCTTCG-1 Patient6 Monocytes nonactive 3 0.06037981 Monocytes_3
10x_3290_TTAGGGTGAATGCC-1 Patient6 Monocytes nonactive 3 0.04608142 Monocytes_3
10x_3290_TTAGGTCTAACCGT-1 Patient6 Monocytes nonactive 3 0.02896138 Monocytes_3
10x_3290_TTAGGTCTTCCCAC-1 Patient6 Monocytes nonactive 3 0.03547651 Monocytes_3
10x_3290_TTAGGTCTTCTACT-1 Patient6 Progenitors nonactive 6 0.10387737 Progenitors_6
10x_3290_TTAGTCACGTCTAG-1 Patient6 Monocytes nonactive 2 0.0142984 Monocytes_2
10x_3290_TTATCCGACACTGA-1 Patient6 Monocytes nonactive 3 0.0800837 Monocytes_3
10x_3290_TTATGCACGTTTCT-1 Patient6 Monocytes nonactive 3 0.06244056 Monocytes_3
10x_3290_TTATGGCTGGTCAT-1 Patient6 Monocytes nonactive 2 0.04245133 Monocytes_2
10x_3290_TTCAAAGAGCTCCT-1 Patient6 Monocytes nonactive 2 0.01439351

Monocytes_2
10x_3290_TTCAACACGAAGGC-1 Patient6 Monocytes nonactive 2 0.05806544
Monocytes_2
10x_3290_TTCAAGCTGAGGCA-1 Patient6 Progenitors active 6 0.11774776
Progenitors_6
10x_3290_TTCACAACAGAGTA-1 Patient6 Progenitors active 6 0.13491535
Progenitors_6
10x_3290_TTCACAACAGCATC-1 Patient6 Progenitors nonactive 6 0.10129351
Progenitors_6
10x_3290_TTCACCCTAGACTC-1 Patient6 Progenitors active 6 0.12269355
Progenitors_6
10x_3290_TTCACCCTTGGTTG-1 Patient6 Monocytes nonactive 3 0.0450986
Monocytes_3
10x_3290_TTCAGTTGCTCTCG-1 Patient6 Monocytes nonactive 2 0.03072094
Monocytes_2
10x_3290_TTCAGTTGTACTGG-1 Patient6 Monocytes nonactive 3 0.03270243
Monocytes_3
10x_3290_TTCATGTGCCTAAG-1 Patient6 Monocytes nonactive 2 0.03500095
Monocytes_2
10x_3290_TTCATGTGCGACTA-1 Patient6 Monocytes nonactive 3 0.01375943
Monocytes_3
10x_3290_TTCCCACTCGAGTT-1 Patient6 Progenitors active 6 0.13152305
Progenitors_6
10x_3290_TTCCTAGAAAGCCT-1 Patient6 Progenitors active 6 0.15839199
Progenitors_6
10x_3290_TTCGATTGTGGTAC-1 Patient6 Progenitors nonactive 6 0.11340435
Progenitors_6
10x_3290_TTCGGAGAGGAACG-1 Patient6 Monocytes nonactive 2 0.07719866
Monocytes_2
10x_3290_TTCGGAGATAGCCA-1 Patient6 Monocytes nonactive 3 0.04040644
Monocytes_3
10x_3290_TTCTACGACAGGAG-1 Patient6 Monocytes nonactive 2 0.0442426
Monocytes_2

10x_3290_TTCTCAGACTTGTT-1 Patient6 Monocytes nonactive 3 0.04742883 Monocytes_3
10x_3290_TTCTCAGATTGTGG-1 Patient6 Monocytes nonactive 2 0.01834063 Monocytes_2
10x_3290_TTGACACTAGGCGA-1 Patient6 Monocytes nonactive 3 0.04543149 Monocytes_3
10x_3290_TTGAGGACGCTTCC-1 Patient6 Monocytes nonactive 3 0.04896646 Monocytes_3
10x_3290_TTGAGGTGGGAACG-1 Patient6 Monocytes nonactive 2 0.06798871 Monocytes_2
10x_3290_TTGAGGTGGGGATG-1 Patient6 Progenitors active 6 0.19291738 Progenitors_6
10x_3290_TTGATCTGGGGATG-1 Patient6 Monocytes nonactive 2 0.07743643 Monocytes_2
10x_3290_TTGCTAACCGAATC-1 Patient6 Monocytes nonactive 2 0.03463636 Monocytes_2
10x_3290_TTGCTAACGCAAGG-1 Patient6 B cells nonactive 7 0.06749731 B cells_7
10x_3290_TTGGTACTGAAACA-1 Patient6 Monocytes nonactive 3 0.07453554 Monocytes_3
10x_3290_TTGTACACGAACCT-1 Patient6 Monocytes nonactive 2 0.0396297 Monocytes_2
10x_3290_TTGTACACTCCTTA-1 Patient6 Monocytes nonactive 3 0.04035889 Monocytes_3
10x_3290_TTGTAGCTCCCTAC-1 Patient6 Progenitors active 6 0.18927145 Progenitors_6
10x_3290_TTGTAGCTGTCGTA-1 Patient6 Monocytes nonactive 2 0.02867605 Monocytes_2
10x_3290_TTTAGCTGGAATAG-1 Patient6 Monocytes nonactive 2 0.03411325 Monocytes_2
10x_3290_TTTAGGCTGAAGGC-1 Patient6 Monocytes nonactive 3 0.03332065 Monocytes_3
10x_3290_TTTCGAACCTCGAA-1 Patient6 Monocytes nonactive 2 0.0607127 Monocytes_2

10x_3290_TTTCTACTGGAGCA-1 Patient6 Monocytes nonactive 3 0.03817133 Monocytes_3
10x_3290_TTTGACTGAAACGA-1 Patient6 Monocytes nonactive 2 0.0687179 Monocytes_2
10x_3290_TTTGACTGGTTCGA-1 Patient6 Monocytes nonactive 2 0.04487667 Monocytes_2
10x_3266_AAACATACCCGTAA-1 Patient3 CD4+ T cells nonactive 11 0.06500856 CD4+ T cells_11
10x_3266_AAACATTGAGTACC-1 Patient3 B cells nonactive 7 0.05587788 B cells_7
10x_3266_AAACCGTGCAAAGA-1 Patient3 T cells nonactive 9 0.09224209 T cells_9
10x_3266_AAACTTGAGGAGTG-1 Patient3 CD4+ T cells nonactive 11 0.07566102 CD4+ T cells_11
10x_3266_AAAGACGAGTCGTA-1 Patient3 Monocytes nonactive 3 0.07106398 Monocytes_3
10x_3266_AAAGGCCTCGAACT-1 Patient3 B cells nonactive 7 0.0754708 B cells_7
10x_3266_AAAGTTTGATTCGG-1 Patient3 B cells nonactive 7 0.10505041 B cells_7
10x_3266_AAATCTGAAAGATG-1 Patient3 CD4+ T cells nonactive 11 0.0349851 CD4+ T cells_11
10x_3266_AAATGGGACTGCAA-1 Patient3 Monocytes nonactive 2 0.02203411 Monocytes_2
10x_3266_AAATGGGATGCATG-1 Patient3 B cells nonactive 7 0.08737556 B cells_7
10x_3266_AAATTGACGCGAGA-1 Patient3 B cells nonactive 7 0.11181916 B cells_7
10x_3266_AACAGAGACGGGAA-1 Patient3 Monocytes active 4 0.11766851 Monocytes_4
10x_3266_AACAGCACTCAAGC-1 Patient3 CD4+ T cells nonactive 11 0.07047746 CD4+ T cells_11
10x_3266_AACCACGAGTATCG-1 Patient3 Monocytes nonactive 0 0.03745799 Monocytes_0
10x_3266_AACCCAGAGCCTTC-1 Patient3 CD4+ T cells nonactive 11 0.05430854 CD4+ T cells_11
10x_3266_AACCTACTCCAACA-1 Patient3 Monocytes nonactive 3 0.02896138 Monocytes_3
10x_3266_AACCTTACTGAGCT-1 Patient3 CD4+ T cells nonactive 11 0.04053326

CD4+ T cells_11
10x_3266_AACGCAACTGCGTA-1 Patient3 B cells nonactive 7 0.06594382 B cells_7
10x_3266_AACGCATGGTATCG-1 Patient3 CD4+ T cells nonactive 11 0.0683216
CD4+ T cells_11
10x_3266_AACGCCCTGGACTT-1 Patient3 B cells nonactive 7 0.08631349 B cells_7
10x_3266_AACGCCCTTAGAAG-1 Patient3 B cells active 7 0.11844525 B cells_7
10x_3266_AACGGTTGAACCTG-1 Patient3 B cells nonactive 7 0.07956059 B cells_7
10x_3266_AACGTGTGATGTGC-1 Patient3 B cells nonactive 7 0.05632173 B cells_7
10x_3266_AACTACCTCAAAGA-1 Patient3 Dendritic cells nonactive 14 0.06172722
Dendritic cells_14
10x_3266_AACTTGCTAGTGCT-1 Patient3 B cells nonactive 7 0.06708516 B cells_7
10x_3266_AAGAACGATTGACG-1 Patient3 Monocytes nonactive 4 0.04454378
Monocytes_4
10x_3266_AAGAAGACGGATTC-1 Patient3 CD4+ T cells nonactive 11 0.04362437
CD4+ T cells_11
10x_3266_AAGAGATGGGTGAG-1 Patient3 Dendritic cells nonactive 14 0.11673324
Dendritic cells_14
10x_3266_AAGCACTGGCGAGA-1 Patient3 B cells nonactive 7 0.08821571 B cells_7
10x_3266_AAGCCAACCAAGCT-1 Patient3 CD4+ T cells nonactive 11 0.05779595
CD4+ T cells_11
10x_3266_AAGCCAACCATTCT-1 Patient3 CD4+ T cells nonactive 11 0.09221039
CD4+ T cells_11
10x_3266_AAGCCAACCCTCCA-1 Patient3 B cells nonactive 7 0.1171771 B cells_7
10x_3266_AAGCCAACTACGAC-1 Patient3 Monocytes nonactive 13 0.0464143
Monocytes_13
10x_3266_AAGCCATGATTCTC-1 Patient3 B cells nonactive 7 0.07011287 B cells_7
10x_3266_AAGGCTACCTAGAC-1 Patient3 T cells nonactive 9 0.09027646 T cells_9
10x_3266_AAGGTCACTTTCGT-1 Patient3 CD4+ T cells nonactive 11 0.05546573
CD4+ T cells_11
10x_3266_AAGTATACTATTCC-1 Patient3 B cells nonactive 7 0.10979012 B cells_7
10x_3266_AAGTCCGAGCCTTC-1 Patient3 B cells nonactive 7 0.04752394 B cells_7
10x_3266_AAGTGCACTCTCCG-1 Patient3 CD4+ T cells nonactive 11 0.05204172
CD4+ T cells_11

10x_3266_AAGTGGCTCTCTAT-1 Patient3 T cells nonactive 9 0.102847 T cells_9
10x_3266_AAGTTATGCAACTG-1 Patient3 B cells nonactive 7 0.08220785 B cells_7
10x_3266_AAGTTCCTAAACAG-1 Patient3 B cells nonactive 7 0.06171137 B cells_7
10x_3266_AATCCTACTAGTCG-1 Patient3 B cells nonactive 7 0.06128337 B cells_7
10x_3266_AATCGGTGATCGGT-1 Patient3 CD4+ T cells nonactive 11 0.07577199
CD4+ T cells_11
10x_3266_AATCTCACTAACCG-1 Patient3 Monocytes nonactive 0 0.05226365
Monocytes_0
10x_3266_AATGGCTGGAAAGT-1 Patient3 B cells nonactive 7 0.07975081 B cells_7
10x_3266_AATGGCTGTTGGTG-1 Patient3 CD4+ T cells nonactive 11 0.05931774
CD4+ T cells_11
10x_3266_AATGTTGACTGAAC-1 Patient3 T cells nonactive 9 0.08853275 T cells_9
10x_3266_AATTACGAGCGAAG-1 Patient3 Dendritic cells nonactive 14 0.08312726
Dendritic cells_14
10x_3266_AATTACGAGTATCG-1 Patient3 Dendritic cells nonactive 14 0.09626847
Dendritic cells_14
10x_3266_ACAAAGGAGTTACG-1 Patient3 Monocytes nonactive 3 0.06537315
Monocytes_3
10x_3266_ACAACCGATAGTCG-1 Patient3 CD4+ T cells nonactive 11 0.10298966
CD4+ T cells_11
10x_3266_ACAAGAGACCACCT-1 Patient3 CD4+ T cells nonactive 11 0.05519625
CD4+ T cells_11
10x_3266_ACAAGAGACTCAGA-1 Patient3 B cells nonactive 7 0.06990679 B cells_7
10x_3266_ACAATAACTCCGTC-1 Patient3 B cells nonactive 7 0.07314057 B cells_7
10x_3266_ACAATTGAAGAGAT-1 Patient3 Dendritic cells active 14 0.14696278
Dendritic cells_14
10x_3266_ACAATTGATCGCCT-1 Patient3 Dendritic cells active 14 0.13927462
Dendritic cells_14
10x_3266_ACACATCTGAATCC-1 Patient3 Monocytes nonactive 3 0.04768246
Monocytes_3
10x_3266_ACAGCAACCACACA-1 Patient3 CD4+ T cells nonactive 11 0.04516201
CD4+ T cells_11
10x_3266_ACAGCAACGTCAAC-1 Patient3 B cells nonactive 7 0.09723543 B cells_7

10x_3266_ACAGTGTGAAGGGC-1	Patient3 T cells	nonactive	9	0.03829814	T cells_9
10x_3266_ACAGTTCTGTAAAG-1	Patient3 B cells	nonactive	7	0.10092892	B cells_7
10x_3266_ACATACCTTTAGGC-1	Patient3 B cells	nonactive	7	0.10531989	B cells_7
10x_3266_ACATTCTGCGTCTC-1	Patient3 B cells	nonactive	7	0.06155285	B cells_7
10x_3266_ACATTCTGTCGTTT-1	Patient3 CD4+ T cells	nonactive	11	0.05026631	CD4+ T cells_11
10x_3266_ACCAACGATGAGAA-1	Patient3 CD4+ T cells	nonactive	11	0.0328768	CD4+ T cells_11
10x_3266_ACCACAGACTGGAT-1	Patient3 Dendritic cells	active	14	0.15872487	Dendritic cells_14
10x_3266_ACCACAGATTGAGC-1	Patient3 B cells	nonactive	7	0.07399658	B cells_7
10x_3266_ACCACCTGAGCTAC-1	Patient3 B cells	nonactive	7	0.11248494	B cells_7
10x_3266_ACCATTTGTAAGGA-1	Patient3 B cells	nonactive	7	0.05305624	B cells_7
10x_3266_ACCCACTGTGCTGA-1	Patient3 CD4+ T cells	nonactive	11	0.06017374	CD4+ T cells_11
10x_3266_ACCCAGCTACCTTT-1	Patient3 B cells	nonactive	7	0.06524634	B cells_7
10x_3266_ACCCAGCTCCGAAT-1	Patient3 B cells	nonactive	7	0.05552914	B cells_7
10x_3266_ACCCGTACTTCTAC-1	Patient3 T cells	nonactive	9	0.05633758	T cells_9
10x_3266_ACCCGTTGAGATCC-1	Patient3 B cells	nonactive	7	0.0969501	B cells_7
10x_3266_ACCGCGGAGCTCCT-1	Patient3 T cells	nonactive	9	0.04340245	T cells_9
10x_3266_ACCTGGCTTCTATC-1	Patient3 Monocytes	nonactive	4	0.03068924	Monocytes_4
10x_3266_ACGAACTGGCAGTT-1	Patient3 B cells	nonactive	7	0.06158455	B cells_7
10x_3266_ACGAAGCTATCGTG-1	Patient3 CD4+ T cells	nonactive	11	0.06251981	CD4+ T cells_11
10x_3266_ACGAGGGATGCTAG-1	Patient3 Progenitors	active	1	0.14930886	Progenitors_1
10x_3266_ACGAGTACCATGAC-1	Patient3 B cells	nonactive	7	0.06668886	B cells_7
10x_3266_ACGATGACAATGCC-1	Patient3 B cells	nonactive	7	0.0493152	B cells_7
10x_3266_ACGCCACTGAGACG-1	Patient3 B cells	nonactive	7	0.07990933	B cells_7
10x_3266_ACGGAGGACGCATA-1	Patient3 Monocytes	nonactive	3	0.03454125	Monocytes_3
10x_3266_ACGGCGTGATTCGG-1	Patient3 B cells	nonactive	7	0.064105	B cells_7

10x_3266_ACGGTATGGTCAAC-1 Patient3 Monocytes nonactive 0 0.05958722 Monocytes_0
10x_3266_ACGTAGACAAAACG-1 Patient3 B cells nonactive 7 0.04814216 B cells_7
10x_3266_ACGTCCTGGCCTTC-1 Patient3 B cells nonactive 7 0.0746465 B cells_7
10x_3266_ACGTCGCTGCCAAT-1 Patient3 CD4+ T cells nonactive 11 0.07188828 CD4+ T cells_11
10x_3266_ACGTGATGATGTGC-1 Patient3 CD4+ T cells nonactive 11 0.05835077 CD4+ T cells_11
10x_3266_ACGTGATGTCGCAA-1 Patient3 Monocytes nonactive 0 0.02892968 Monocytes_0
10x_3266_ACGTTGGAACTTTC-1 Patient3 Monocytes nonactive 3 0.04991757 Monocytes_3
10x_3266_ACGTTGGAATTCTC-1 Patient3 B cells nonactive 7 0.08425274 B cells_7
10x_3266_ACGTTGGACTCCAC-1 Patient3 B cells nonactive 7 0.08216029 B cells_7
10x_3266_ACTAAAACCCAAGT-1 Patient3 T cells nonactive 9 0.05537062 T cells_9
10x_3266_ACTACGGAACCGAT-1 Patient3 Progenitors nonactive 5 0.07001775 Progenitors_5
10x_3266_ACTACGGACCCAAA-1 Patient3 B cells nonactive 7 0.09853529 B cells_7
10x_3266_ACTACGGACCGTTC-1 Patient3 B cells nonactive 7 0.06305878 B cells_7
10x_3266_ACTATCACCTTGGA-1 Patient3 T cells nonactive 9 0.05191491 T cells_9
10x_3266_ACTCAGGAATCTTC-1 Patient3 Monocytes nonactive 3 0.05941285 Monocytes_3
10x_3266_ACTCCTCTGTTCAG-1 Patient3 B cells nonactive 7 0.06817894 B cells_7
10x_3266_ACTCTCCTTCAGAC-1 Patient3 B cells nonactive 7 0.08246148 B cells_7
10x_3266_ACTGAGACGGTGGA-1 Patient3 Dendritic cells active 14 0.12324837 Dendritic cells_14
10x_3266_ACTGGCCTTCACCC-1 Patient3 Dendritic cells active 14 0.1264663 Dendritic cells_14
10x_3266_ACTTCAACTAGAAG-1 Patient3 Monocytes nonactive 13 0.02081352 Monocytes_13
10x_3266_ACTTGTACGGATCT-1 Patient3 B cells nonactive 7 0.09848773 B cells_7
10x_3266_AGAAAGTGTTGGCA-1 Patient3 CD4+ T cells nonactive 11 0.03858348 CD4+ T cells_11

10x_3266_AGAAGATGCTAAGC-1 Patient3 B cells nonactive 7 0.07957644 B cells_7
10x_3266_AGAATGGATCTAGG-1 Patient3 CD4+ T cells nonactive 11 0.07959229 CD4+ T cells_11
10x_3266_AGACACTGGGTAGG-1 Patient3 B cells nonactive 7 0.07705599 B cells_7
10x_3266_AGACCTGAAGACTC-1 Patient3 Dendritic cells nonactive 14 0.09888403 Dendritic cells_14
10x_3266_AGACGTACAAGGGC-1 Patient3 B cells nonactive 7 0.06440619 B cells_7
10x_3266_AGACGTACGACGGA-1 Patient3 CD4+ T cells nonactive 11 0.09473084 CD4+ T cells_11
10x_3266_AGACTGACTTCGTT-1 Patient3 Monocytes nonactive 3 0.0480629 Monocytes_3
10x_3266_AGACTTCTGCTTAG-1 Patient3 B cells nonactive 7 0.05654366 B cells_7
10x_3266_AGAGATGACAACCA-1 Patient3 T cells nonactive 9 0.07436117 T cells_9
10x_3266_AGAGGTCTGGTATC-1 Patient3 B cells nonactive 7 0.06102974 B cells_7
10x_3266_AGAGTGCTTAGACC-1 Patient3 Monocytes nonactive 13 0.06316974 Monocytes_13
10x_3266_AGATATTGACCCAA-1 Patient3 Monocytes nonactive 3 0.0438463 Monocytes_3
10x_3266_AGCAAAGAAAGTAG-1 Patient3 CD4+ T cells nonactive 11 0.08829497 CD4+ T cells_11
10x_3266_AGCAACACGCAGAG-1 Patient3 Monocytes nonactive 15 0.04801534 Monocytes_15
10x_3266_AGCACTGACGTGAT-1 Patient3 Dendritic cells nonactive 14 0.08734386 Dendritic cells_14
10x_3266_AGCATCGAACCGAT-1 Patient3 B cells nonactive 7 0.07986177 B cells_7
10x_3266_AGCATGACCAGATC-1 Patient3 B cells nonactive 7 0.08753408 B cells_7
10x_3266_AGCCAATGTAAGCC-1 Patient3 CD4+ T cells nonactive 11 0.0358728 CD4+ T cells_11
10x_3266_AGCCACCTCGTAAC-1 Patient3 CD4+ T cells nonactive 11 0.05459387 CD4+ T cells_11
10x_3266_AGCCACCTGTTAGC-1 Patient3 B cells nonactive 7 0.08277852 B cells_7
10x_3266_AGCCGGTGTAGAAG-1 Patient3 Monocytes nonactive 3 0.06754486 Monocytes_3

10x_3266_AGCCGTCTATCGAC-1 Patient3 Monocytes nonactive 3 0.01158772 Monocytes_3
10x_3266_AGCGAACTCTTCGC-1 Patient3 CD4+ T cells nonactive 11 0.05705092 CD4+ T cells_11
10x_3266_AGCGATTGTGACCA-1 Patient3 CD4+ T cells nonactive 11 0.0582081 CD4+ T cells_11
10x_3266_AGCGCCGAACCGAT-1 Patient3 CD4+ T cells nonactive 11 0.06982753 CD4+ T cells_11
10x_3266_AGCGCTCTCCTAAG-1 Patient3 B cells nonactive 7 0.04912498 B cells_7
10x_3266_AGCGCTCTGCTGTA-1 Patient3 Progenitors nonactive 5 0.04324393 Progenitors_5
10x_3266_AGCGGGCTATTTCC-1 Patient3 CD4+ T cells nonactive 11 0.0703665 CD4+ T cells_11
10x_3266_AGCGTAACAACAGA-1 Patient3 T cells nonactive 9 0.07621584 T cells_9
10x_3266_AGCGTAACTGCCCT-1 Patient3 B cells nonactive 7 0.06394648 B cells_7
10x_3266_AGGAACCTCTAAGC-1 Patient3 Dendritic cells nonactive 14 0.09585632 Dendritic cells_14
10x_3266_AGGAATGAGGGATG-1 Patient3 Monocytes nonactive 3 0.05289772 Monocytes_3
10x_3266_AGGACACTAGATGA-1 Patient3 CD4+ T cells nonactive 11 0.05936529 CD4+ T cells_11
10x_3266_AGGACACTTGATGC-1 Patient3 B cells nonactive 7 0.05608395 B cells_7
10x_3266_AGGACTTGGGCAAG-1 Patient3 T cells nonactive 9 0.06465982 T cells_9
10x_3266_AGGATGCTGGTACT-1 Patient3 Progenitors active 12 0.1437607 Progenitors_12
10x_3266_AGGCTAACTTTGTC-1 Patient3 B cells nonactive 7 0.0839357 B cells_7
10x_3266_AGGGACGATGCTTT-1 Patient3 T cells nonactive 9 0.09308224 T cells_9
10x_3266_AGGGCCACAGAACA-1 Patient3 B cells nonactive 7 0.06420011 B cells_7
10x_3266_AGGGCGCTAAAAGC-1 Patient3 Dendritic cells nonactive 14 0.08834253 Dendritic cells_14
10x_3266_AGGGCGCTAACGGG-1 Patient3 B cells nonactive 7 0.05784351 B cells_7
10x_3266_AGGGTGGAGGTGAG-1 Patient3 B cells nonactive 7 0.06790945 B cells_7
10x_3266_AGGTACTGCGGGAA-1 Patient3 B cells nonactive 7 0.0754391 B cells_7

10x_3266_AGGTCATGTGTCCC-1 Patient3 B cells nonactive 7 0.0552438 B cells_7
10x_3266_AGGTCTGACATGGT-1 Patient3 B cells nonactive 7 0.08628178 B cells_7
10x_3266_AGGTCTGATGGTGT-1 Patient3 B cells nonactive 7 0.09343098 B cells_7
10x_3266_AGTAAGGATATCGG-1 Patient3 Monocytes nonactive 0 0.03010272 Monocytes_0
10x_3266_AGTAATTGAGAGTA-1 Patient3 B cells nonactive 7 0.08296874 B cells_7
10x_3266_AGTACGTGCCTGAA-1 Patient3 T cells nonactive 9 0.07171391 T cells_9
10x_3266_AGTATAACCAACTG-1 Patient3 T cells nonactive 9 0.06158455 T cells_9
10x_3266_AGTCACGAAGAAGT-1 Patient3 B cells nonactive 7 0.07654873 B cells_7
10x_3266_AGTCCAGAGAAACA-1 Patient3 B cells nonactive 7 0.07862532 B cells_7
10x_3266_AGTCCAGATGGTAC-1 Patient3 B cells nonactive 7 0.10563693 B cells_7
10x_3266_AGTCTACTGTGCTA-1 Patient3 T cells nonactive 9 0.06269419 T cells_9
10x_3266_AGTCTTACTACTGG-1 Patient3 CD4+ T cells nonactive 11 0.06908249 CD4+ T cells_11
10x_3266_AGTGACTGGCTGTA-1 Patient3 CD4+ T cells nonactive 11 0.06971657 CD4+ T cells_11
10x_3266_AGTTCTACTAGAGA-1 Patient3 Dendritic cells nonactive 14 0.1125642 Dendritic cells_14
10x_3266_AGTTCTTGTTTCAC-1 Patient3 T cells nonactive 9 0.08734386 T cells_9
10x_3266_AGTTTAGAATTGGC-1 Patient3 T cells nonactive 9 0.0994547 T cells_9
10x_3266_AGTTTCACCTTGAG-1 Patient3 CD4+ T cells nonactive 11 0.04828483 CD4+ T cells_11
10x_3266_ATAACATGGCATAC-1 Patient3 B cells nonactive 7 0.09555513 B cells_7
10x_3266_ATAACATGGGTAGG-1 Patient3 Monocytes nonactive 0 0.04482912 Monocytes_0
10x_3266_ATAACCCTAGTCGT-1 Patient3 Monocytes nonactive 3 0.06037981 Monocytes_3
10x_3266_ATAAGTACGGACTT-1 Patient3 T cells nonactive 9 0.02258893 T cells_9
10x_3266_ATACAATGCCTCGT-1 Patient3 T cells nonactive 9 0.10798301 T cells_9
10x_3266_ATACAATGCTCTAT-1 Patient3 CD4+ T cells nonactive 11 0.06182233 CD4+ T cells_11
10x_3266_ATACAATGGAGGAC-1 Patient3 CD4+ T cells nonactive 11 0.04627164 CD4+ T cells_11

10x_3266_ATACCACTACCACA-1 Patient3 CD4+ T cells nonactive 11 0.04917253 CD4+ T cells_11
10x_3266_ATACCACTTGCACA-1 Patient3 B cells nonactive 7 0.09953395 B cells_7
10x_3266_ATACCGGAATTCCT-1 Patient3 B cells nonactive 7 0.04460719 B cells_7
10x_3266_ATACCTACGCGTTA-1 Patient3 Monocytes nonactive 3 0.02262063 Monocytes_3
10x_3266_ATACGTCTGAGGTG-1 Patient3 B cells nonactive 7 0.08395156 B cells_7
10x_3266_ATACGTCTGGTTAC-1 Patient3 B cells active 7 0.13900514 B cells_7
10x_3266_ATACTCTGCCTGTC-1 Patient3 B cells nonactive 7 0.06939953 B cells_7
10x_3266_ATAGATTGTCGATG-1 Patient3 B cells nonactive 7 0.07794369 B cells_7
10x_3266_ATAGATTGTGTTTC-1 Patient3 CD4+ T cells nonactive 11 0.04097711 CD4+ T cells_11
10x_3266_ATAGCCGATATGGC-1 Patient3 Dendritic cells nonactive 14 0.06551582 Dendritic cells_14
10x_3266_ATAGCTCTTGCGTA-1 Patient3 B cells nonactive 7 0.09639528 B cells_7
10x_3266_ATATACGACGCATA-1 Patient3 CD4+ T cells nonactive 11 0.08718534 CD4+ T cells_11
10x_3266_ATATACGACTCCAC-1 Patient3 B cells nonactive 7 0.05330987 B cells_7
10x_3266_ATATGCCTAAGTGA-1 Patient3 B cells nonactive 7 0.10772938 B cells_7
10x_3266_ATCAAATGTTCTTG-1 Patient3 Monocytes nonactive 3 0.04292689 Monocytes_3
10x_3266_ATCAACCTCCTGAA-1 Patient3 CD4+ T cells nonactive 11 0.04205504 CD4+ T cells_11
10x_3266_ATCACACTTGAGGG-1 Patient3 B cells nonactive 7 0.10126181 B cells_7
10x_3266_ATCACGGATGTCCC-1 Patient3 Monocytes nonactive 3 0.01217424 Monocytes_3
10x_3266_ATCACTTGAGACTC-1 Patient3 CD4+ T cells nonactive 11 0.05972988 CD4+ T cells_11
10x_3266_ATCACTTGTGGTGT-1 Patient3 Dendritic cells active 14 0.11944392 Dendritic cells_14
10x_3266_ATCATGCTAGGTTC-1 Patient3 B cells nonactive 7 0.0286919 B cells_7
10x_3266_ATCATGCTGATAAG-1 Patient3 CD4+ T cells nonactive 11 0.04102467 CD4+ T cells_11

10x_3266_ATCCATACGCTATG-1 Patient3 Monocytes nonactive 3 0.02978568 Monocytes_3
10x_3266_ATCCATACGGTTTG-1 Patient3 B cells nonactive 7 0.04606556 B cells_7
10x_3266_ATCCGCACTTTCTG-1 Patient3 B cells nonactive 7 0.09205187 B cells_7
10x_3266_ATCCTAACATGTGC-1 Patient3 B cells nonactive 7 0.07291865 B cells_7
10x_3266_ATCGACGACTCTAT-1 Patient3 B cells nonactive 7 0.09205187 B cells_7
10x_3266_ATCGCAGAATTCGG-1 Patient3 CD4+ T cells nonactive 11 0.04700082 CD4+ T cells_11
10x_3266_ATCGCAGAGCCTTC-1 Patient3 B cells nonactive 7 0.07989348 B cells_7
10x_3266_ATCTACACCCAAGT-1 Patient3 CD4+ T cells nonactive 11 0.03015028 CD4+ T cells_11
10x_3266_ATCTACACTCCCGT-1 Patient3 B cells nonactive 7 0.06351848 B cells_7
10x_3266_ATCTACTGGCTTCC-1 Patient3 CD4+ T cells nonactive 11 0.06941538 CD4+ T cells_11
10x_3266_ATCTGACTAGTACC-1 Patient3 CD4+ T cells nonactive 11 0.03958214 CD4+ T cells_11
10x_3266_ATCTGACTGGTGTT-1 Patient3 CD4+ T cells nonactive 11 0.08022636 CD4+ T cells_11
10x_3266_ATCTTGACGTACGT-1 Patient3 Monocytes nonactive 15 0.01699322 Monocytes_15
10x_3266_ATCTTTCTAAAACG-1 Patient3 Monocytes nonactive 3 0.01973559 Monocytes_3
10x_3266_ATGAAGGATAGACC-1 Patient3 CD4+ T cells nonactive 11 0.06248811 CD4+ T cells_11
10x_3266_ATGAGAGATACTTC-1 Patient3 CD4+ T cells nonactive 11 0.04960053 CD4+ T cells_11
10x_3266_ATGAGCACAACGTC-1 Patient3 CD4+ T cells nonactive 11 0.03977237 CD4+ T cells_11
10x_3266_ATGAGCACAAGGTA-1 Patient3 B cells nonactive 7 0.07009701 B cells_7
10x_3266_ATGCAGTGGGTGAG-1 Patient3 B cells nonactive 7 0.10084966 B cells_7
10x_3266_ATGCCGCTCGCTAA-1 Patient3 Progenitors nonactive 5 0.07916429 Progenitors_5
10x_3266_ATGCCGCTGTGCTA-1 Patient3 Monocytes nonactive 0 0.06063344

Monocytes_0
10x_3266_ATGCGATGGAGACG-1 Patient3 Dendritic cells nonactive 14 0.11337265 Dendritic cells_14
10x_3266_ATGTAAACCCTTAT-1 Patient3 B cells nonactive 7 0.085188 B cells_7
10x_3266_ATGTTAGATAAGCC-1 Patient3 Monocytes nonactive 3 0.03574599 Monocytes_3
10x_3266_ATGTTCACATCGGT-1 Patient3 Monocytes nonactive 3 0.03688732 Monocytes_3
10x_3266_ATGTTGCTTTCTAC-1 Patient3 CD4+ T cells nonactive 11 0.0409137 CD4+ T cells_11
10x_3266_ATTACCACACGACT-1 Patient3 T cells nonactive 9 0.09617336 T cells_9
10x_3266_ATTACCACTTCCAT-1 Patient3 B cells nonactive 7 0.06439034 B cells_7
10x_3266_ATTACCTGTGGTTG-1 Patient3 Monocytes nonactive 3 0.02821635 Monocytes_3
10x_3266_ATTAGATGCTCTAT-1 Patient3 B cells nonactive 7 0.08208103 B cells_7
10x_3266_ATTAGATGTAGCGT-1 Patient3 CD4+ T cells nonactive 11 0.06374041 CD4+ T cells_11
10x_3266_ATTCAAGAAAGAGT-1 Patient3 Monocytes nonactive 0 0.02159026 Monocytes_0
10x_3266_ATTCAAGATCACGA-1 Patient3 Monocytes nonactive 3 0.02644094 Monocytes_3
10x_3266_ATTCCAACTGCTCC-1 Patient3 B cells nonactive 7 0.0809714 B cells_7
10x_3266_ATTCCATGTCGACA-1 Patient3 Monocytes nonactive 0 0.03435102 Monocytes_0
10x_3266_ATTCCATGTGGTGT-1 Patient3 Dendritic cells nonactive 14 0.07772177 Dendritic cells_14
10x_3266_ATTCTGACCATCAG-1 Patient3 Monocytes nonactive 0 0.019371 Monocytes_0
10x_3266_ATTCTGACTAACCG-1 Patient3 B cells nonactive 7 0.1171771 B cells_7
10x_3266_ATTGAAACTTCGTT-1 Patient3 T cells nonactive 9 0.09802803 T cells_9
10x_3266_ATTGCACTTGTTTC-1 Patient3 B cells nonactive 7 0.0763268 B cells_7
10x_3266_ATTGCGGATTGCGA-1 Patient3 CD4+ T cells nonactive 11 0.03852007 CD4+ T cells_11

10x_3266_ATTGCTACTCTTCA-1 Patient3 CD4+ T cells nonactive 11 0.06992264 CD4+ T cells_11
10x_3266_ATTGGGTGGAGCTT-1 Patient3 CD4+ T cells nonactive 11 0.05429269 CD4+ T cells_11
10x_3266_ATTTCGTGAATCGC-1 Patient3 B cells nonactive 7 0.06310633 B cells_7
10x_3266_ATTTCGTGAGTGCT-1 Patient3 Progenitors nonactive 5 0.11248494 Progenitors_5
10x_3266_ATTTCGTGGTAGGG-1 Patient3 Monocytes nonactive 13 0.08926194 Monocytes_13
10x_3266_ATTTCGTGGTCACA-1 Patient3 CD4+ T cells nonactive 11 0.05323061 CD4+ T cells_11
10x_3266_ATTTCTCTCTGGAT-1 Patient3 CD4+ T cells nonactive 11 0.04717519 CD4+ T cells_11
10x_3266_ATTTCTCTTACTTC-1 Patient3 B cells nonactive 7 0.07684991 B cells_7
10x_3266_ATTTGCACCTCTCG-1 Patient3 Monocytes nonactive 16 0.05334158 Monocytes_16
10x_3266_ATTTGCACTATTCC-1 Patient3 CD4+ T cells nonactive 11 0.05944455 CD4+ T cells_11
10x_3266_CAAATATGTTTGTC-1 Patient3 CD4+ T cells nonactive 11 0.06248811 CD4+ T cells_11
10x_3266_CAACAGACAGAAGT-1 Patient3 T cells active 9 0.13336187 T cells_9
10x_3266_CAACGTGAGGACTT-1 Patient3 T cells nonactive 9 0.07651702 T cells_9
10x_3266_CAACGTGAGGTATC-1 Patient3 Monocytes nonactive 0 0.01409232 Monocytes_0
10x_3266_CAACTTTGAGTACC-1 Patient3 Monocytes nonactive 13 0.03355843 Monocytes_13
10x_3266_CAAGTTCTCTACTT-1 Patient3 Dendritic cells active 14 0.13735654 Dendritic cells_14
10x_3266_CAATATGAACGTGT-1 Patient3 Dendritic cells active 14 0.12134614 Dendritic cells_14
10x_3266_CAATATGATGACAC-1 Patient3 T cells nonactive 9 0.08043244 T cells_9
10x_3266_CAATCGGAAACCTG-1 Patient3 Dendritic cells active 14 0.13060364 Dendritic cells_14

10x_3266_CAATCTACGCCAAT-1 Patient3 T cells nonactive 9 0.07052501 T cells_9
10x_3266_CAATTCTGAACCAC-1 Patient3 B cells nonactive 7 0.07600976 B cells_7
10x_3266_CAATTCTGGAGACG-1 Patient3 CD4+ T cells nonactive 11 0.0505675 CD4+ T cells_11
10x_3266_CAATTCTGGCCCTT-1 Patient3 Dendritic cells nonactive 14 0.10604908 Dendritic cells_14
10x_3266_CACAACGACTACGA-1 Patient3 B cells nonactive 7 0.0855843 B cells_7
10x_3266_CACAGCCTTGTGGT-1 Patient3 T cells nonactive 9 0.11026568 T cells_9
10x_3266_CACAGTGATTCGTT-1 Patient3 T cells nonactive 9 0.06882886 T cells_9
10x_3266_CACCACTGAACCTG-1 Patient3 CD4+ T cells nonactive 11 0.03974066 CD4+ T cells_11
10x_3266_CACCGGGAGCCATA-1 Patient3 B cells nonactive 7 0.05513284 B cells_7
10x_3266_CACCGTTGCGCCTT-1 Patient3 B cells nonactive 7 0.05570351 B cells_7
10x_3266_CACGATGATTGGTG-1 Patient3 Monocytes nonactive 0 0.03563503 Monocytes_0
10x_3266_CACGCTACGCCAAT-1 Patient3 T cells nonactive 9 0.07071524 T cells_9
10x_3266_CACGCTACTTACTC-1 Patient3 B cells nonactive 7 0.10915605 B cells_7
10x_3266_CACGGGTGGCCCTT-1 Patient3 B cells nonactive 7 0.09974003 B cells_7
10x_3266_CACGGGTGTCGACA-1 Patient3 B cells nonactive 7 0.07704014 B cells_7
10x_3266_CACTCCGACTAGCA-1 Patient3 B cells nonactive 7 0.07515376 B cells_7
10x_3266_CACTTAACCACTCC-1 Patient3 B cells nonactive 7 0.10107159 B cells_7
10x_3266_CACTTATGTTCCAT-1 Patient3 Monocytes nonactive 3 0.07608902 Monocytes_3
10x_3266_CAGAAGCTTGTAGC-1 Patient3 Dendritic cells active 14 0.12508719 Dendritic cells_14
10x_3266_CAGACAACTTTGTC-1 Patient3 T cells nonactive 9 0.06900323 T cells_9
10x_3266_CAGACATGCTACGA-1 Patient3 B cells nonactive 7 0.10408344 B cells_7
10x_3266_CAGACCCTAGATGA-1 Patient3 B cells nonactive 7 0.09517469 B cells_7
10x_3266_CAGACTGATGTTCT-1 Patient3 T cells nonactive 9 0.09747321 T cells_9
10x_3266_CAGAGGGACCTCGT-1 Patient3 Monocytes nonactive 3 0.0425623 Monocytes_3
10x_3266_CAGAGGGACCTTAT-1 Patient3 B cells nonactive 7 0.09133853 B cells_7
10x_3266_CAGAGGGAGCGTAT-1 Patient3 B cells nonactive 7 0.02682138 B cells_7

10x_3266_CAGAGGGATCGCAA-1	Patient3	B cells	nonactive	7	0.05784351	B cells_7
10x_3266_CAGAGGGATTCGTT-1	Patient3	B cells	active	7	0.12727474	B cells_7
10x_3266_CAGCACCTCATGGT-1	Patient3	B cells	nonactive	7	0.10167396	B cells_7
10x_3266_CAGCATGATCTCCG-1	Patient3	B cells	nonactive	7	0.0894046	B cells_7
10x_3266_CAGCCTACGTGTAC-1	Patient3	CD4+ T cells	nonactive	11	0.03875785	CD4+ T cells_11
10x_3266_CAGCCTTGGAAACA-1	Patient3	CD4+ T cells	nonactive	11	0.03561917	CD4+ T cells_11
10x_3266_CAGCGGACGTATCG-1	Patient3	CD4+ T cells	nonactive	11	0.07268087	CD4+ T cells_11
10x_3266_CAGCGGACTGAAGA-1	Patient3	Monocytes	nonactive	0	0.03883711	Monocytes_0
10x_3266_CAGGTAACAGGTTC-1	Patient3	Dendritic cells	nonactive	14	0.08770845	Dendritic cells_14
10x_3266_CAGGTTGAGAACTC-1	Patient3	Dendritic cells	active	14	0.1290026	Dendritic cells_14
10x_3266_CAGTTTACGAGCAG-1	Patient3	B cells	nonactive	7	0.03311458	B cells_7
10x_3266_CATAAAACCTGTTT-1	Patient3	Monocytes	nonactive	3	0.06729123	Monocytes_3
10x_3266_CATCCCGATTCTCA-1	Patient3	Monocytes	nonactive	4	0.04901401	Monocytes_4
10x_3266_CATGCGCTTGACCA-1	Patient3	B cells	nonactive	7	0.11321413	B cells_7
10x_3266_CATGGATGACGGAG-1	Patient3	B cells	nonactive	7	0.07103227	B cells_7
10x_3266_CATGTTACTTATCC-1	Patient3	B cells	nonactive	7	0.06408915	B cells_7
10x_3266_CATGTTTGACTGTG-1	Patient3	B cells	nonactive	7	0.06266248	B cells_7
10x_3266_CATTGACTTCTGGA-1	Patient3	B cells	nonactive	7	0.07545495	B cells_7
10x_3266_CATTGGGAAAGAGT-1	Patient3	B cells	nonactive	7	0.08249318	B cells_7
10x_3266_CATTGGGAATAAGG-1	Patient3	Monocytes	nonactive	4	0.02095619	Monocytes_4
10x_3266_CATTGGGACGTACA-1	Patient3	B cells	nonactive	7	0.07729377	B cells_7
10x_3266_CATTTGACGGACGA-1	Patient3	Monocytes	nonactive	3	0.07830829	Monocytes_3
10x_3266_CATTTGACGGGCAA-1	Patient3	B cells	nonactive	7	0.06335996	B cells_7

10x_3266_CATTTGTGGTAGCT-1 Patient3 B cells nonactive 7 0.10906093 B cells_7
10x_3266_CCAAGAACTGGTTG-1 Patient3 Progenitors nonactive 5 0.11579798 Progenitors_5
10x_3266_CCAAGATGAGATCC-1 Patient3 B cells nonactive 7 0.06301122 B cells_7
10x_3266_CCAATTTGTTCCAT-1 Patient3 Dendritic cells active 14 0.12354955 Dendritic cells_14
10x_3266_CCAATTTGTTTCGT-1 Patient3 B cells nonactive 7 0.08476 B cells_7
10x_3266_CCACCATGTAGTCG-1 Patient3 T cells nonactive 9 0.03886881 T cells_9
10x_3266_CCACCTGAAACCAC-1 Patient3 CD4+ T cells nonactive 11 0.05487921 CD4+ T cells_11
10x_3266_CCACTGACTAGCCA-1 Patient3 T cells nonactive 9 0.06369285 T cells_9
10x_3266_CCAGAAACTCTCGC-1 Patient3 Monocytes nonactive 3 0.02678968 Monocytes_3
10x_3266_CCAGATGAGCGTAT-1 Patient3 T cells nonactive 9 0.11077294 T cells_9
10x_3266_CCAGCACTGACGTT-1 Patient3 B cells nonactive 7 0.07098472 B cells_7
10x_3266_CCAGGTCTTGAAGA-1 Patient3 CD4+ T cells nonactive 11 0.06752901 CD4+ T cells_11
10x_3266_CCATAGGACTACGA-1 Patient3 B cells active 7 0.11896836 B cells_7
10x_3266_CCATTAACTGGGAG-1 Patient3 B cells nonactive 7 0.08298459 B cells_7
10x_3266_CCCAAAGATCGTAG-1 Patient3 B cells nonactive 7 0.09431869 B cells_7
10x_3266_CCCAACTGTTGCAG-1 Patient3 CD4+ T cells nonactive 11 0.0653256 CD4+ T cells_11
10x_3266_CCCATCGAGGGCAA-1 Patient3 CD4+ T cells nonactive 11 0.02503012 CD4+ T cells_11
10x_3266_CCCGAACTGCGAGA-1 Patient3 B cells nonactive 7 0.07994103 B cells_7
10x_3266_CCCGATTGAGGGTG-1 Patient3 B cells nonactive 7 0.07350517 B cells_7
10x_3266_CCCGATTGCCTTCG-1 Patient3 T cells nonactive 9 0.06890812 T cells_9
10x_3266_CCCTGAACAGATGA-1 Patient3 Monocytes nonactive 3 0.05713018 Monocytes_3
10x_3266_CCGAAAACGTTTCT-1 Patient3 T cells nonactive 9 0.07185657 T cells_9
10x_3266_CCGACACTACCAAC-1 Patient3 B cells nonactive 7 0.04850675 B cells_7
10x_3266_CCGACTACTGCAGT-1 Patient3 Monocytes nonactive 0 0.01453617 Monocytes_0

10x_3266_CCGCGAGAAGTGTC-1 Patient3 B cells nonactive 7 0.05888973 B cells_7
10x_3266_CCGCGAGATCACGA-1 Patient3 Dendritic cells nonactive 14 0.09691839 Dendritic cells_14
10x_3266_CCGCTATGAGGCGA-1 Patient3 B cells nonactive 7 0.08385645 B cells_7
10x_3266_CCGCTATGTCTTTG-1 Patient3 B cells nonactive 7 0.05307209 B cells_7
10x_3266_CCGGTACTAAAACG-1 Patient3 B cells nonactive 7 0.08231881 B cells_7
10x_3266_CCGTACACTATTCC-1 Patient3 B cells nonactive 7 0.10297381 B cells_7
10x_3266_CCTACCGAATTCCT-1 Patient3 B cells nonactive 7 0.08533067 B cells_7
10x_3266_CCTACCGACACAAC-1 Patient3 T cells nonactive 9 0.06727538 T cells_9
10x_3266_CCTACCGACTGACA-1 Patient3 B cells nonactive 7 0.06938368 B cells_7
10x_3266_CCTATTGAGAATCC-1 Patient3 T cells nonactive 9 0.0603164 T cells_9
10x_3266_CCTCATCTGACTAC-1 Patient3 B cells nonactive 7 0.07456724 B cells_7
10x_3266_CCTCATCTTTTCTG-1 Patient3 CD4+ T cells nonactive 11 0.0324805 CD4+ T cells_11
10x_3266_CCTCGAACGAAACA-1 Patient3 B cells nonactive 7 0.06951049 B cells_7
10x_3266_CCTGAGCTGTAGCT-1 Patient3 B cells active 7 0.14311077 B cells_7
10x_3266_CCTGCAACCCCACT-1 Patient3 CD4+ T cells nonactive 11 0.04351341 CD4+ T cells_11
10x_3266_CCTTAATGCGCCTT-1 Patient3 T cells nonactive 9 0.08162133 T cells_9
10x_3266_CCTTCACTGGCAAG-1 Patient3 CD4+ T cells nonactive 11 0.03588866 CD4+ T cells_11
10x_3266_CCTTTAGACTGAGT-1 Patient3 B cells nonactive 7 0.0708262 B cells_7
10x_3266_CGAACATGGGGACA-1 Patient3 Monocytes nonactive 2 0.02235115 Monocytes_2
10x_3266_CGAAGGGACGATAC-1 Patient3 Dendritic cells nonactive 14 0.1151322 Dendritic cells_14
10x_3266_CGAAGGGATTCGTT-1 Patient3 B cells nonactive 7 0.08074948 B cells_7
10x_3266_CGAATCGATATCTC-1 Patient3 CD4+ T cells nonactive 11 0.05733625 CD4+ T cells_11
10x_3266_CGACCTACTCGCCT-1 Patient3 CD4+ T cells nonactive 11 0.02317545 CD4+ T cells_11
10x_3266_CGACGTCTCTCCAC-1 Patient3 B cells nonactive 7 0.05112231 B cells_7
10x_3266_CGACGTCTCTTGAG-1 Patient3 CD4+ T cells nonactive 11 0.10527234

CD4+ T cells_11
10x_3266_CGAGAACTCTACGA-1 Patient3 B cells nonactive 7 0.04364023 B cells_7
10x_3266_CGAGATTGACCTGA-1 Patient3 B cells nonactive 7 0.06900323 B cells_7
10x_3266_CGAGATTGCGTACA-1 Patient3 B cells nonactive 7 0.09008623 B cells_7
10x_3266_CGAGCCGAACACCA-1 Patient3 B cells nonactive 7 0.06759242 B cells_7
10x_3266_CGAGCCGATTCGCC-1 Patient3 B cells nonactive 7 0.0775791 B cells_7
10x_3266_CGAGCGTGGGAGCA-1 Patient3 B cells nonactive 7 0.06366115 B cells_7
10x_3266_CGAGGAGAGAGGAC-1 Patient3 B cells nonactive 7 0.08797793 B cells_7
10x_3266_CGAGGCTGTCCTGC-1 Patient3 Monocytes nonactive 16 0.0383457 Monocytes_16
10x_3266_CGATAGACCATGGT-1 Patient3 B cells nonactive 7 0.06251981 B cells_7
10x_3266_CGCAAATGAAGTGA-1 Patient3 B cells nonactive 7 0.07028724 B cells_7
10x_3266_CGCACTACGAATCC-1 Patient3 T cells nonactive 9 0.08276267 T cells_9
10x_3266_CGCACTTGTCCCGT-1 Patient3 CD4+ T cells nonactive 11 0.06299537 CD4+ T cells_11
10x_3266_CGCAGGTGCTCGCT-1 Patient3 Monocytes nonactive 3 0.03687147 Monocytes_3
10x_3266_CGCATAGAATCGTG-1 Patient3 Dendritic cells nonactive 14 0.10408344 Dendritic cells_14
10x_3266_CGCCATTGCCCTCA-1 Patient3 B cells nonactive 7 0.03826644 B cells_7
10x_3266_CGCCATTGCTGAGT-1 Patient3 Progenitors active 1 0.13477268 Progenitors_1
10x_3266_CGCCTAACTTCCCG-1 Patient3 B cells nonactive 7 0.08117748 B cells_7
10x_3266_CGCGATCTAGTGTC-1 Patient3 CD4+ T cells nonactive 11 0.02545812 CD4+ T cells_11
10x_3266_CGCTCATGAAAGCA-1 Patient3 T cells nonactive 9 0.09723543 T cells_9
10x_3266_CGGAGGCTGGGAGT-1 Patient3 Monocytes nonactive 13 0.05140765 Monocytes_13
10x_3266_CGGATAACTGCATG-1 Patient3 B cells nonactive 7 0.07892651 B cells_7
10x_3266_CGGATATGTCCTAT-1 Patient3 Monocytes nonactive 15 0.04246719 Monocytes_15
10x_3266_CGGCATCTAAAACG-1 Patient3 B cells nonactive 7 0.07818147 B cells_7
10x_3266_CGGCATCTGTCCTC-1 Patient3 B cells nonactive 7 0.08452222 B cells_7

10x_3266_CGGCGATGGTATCG-1 Patient3 Dendritic cells active 14 0.14242914 Dendritic cells_14
10x_3266_CGGGACTGCTTACT-1 Patient3 CD4+ T cells nonactive 11 0.08577452 CD4+ T cells_11
10x_3266_CGGTAAACACCAGT-1 Patient3 B cells nonactive 7 0.06022129 B cells_7
10x_3266_CGTACCACGTTACG-1 Patient3 B cells nonactive 7 0.07705599 B cells_7
10x_3266_CGTACCACTTCTTG-1 Patient3 B cells nonactive 7 0.05554499 B cells_7
10x_3266_CGTACCTGACGTGT-1 Patient3 Monocytes nonactive 16 0.03869444 Monocytes_16
10x_3266_CGTCCAACGTTGTG-1 Patient3 T cells nonactive 9 0.1163211 T cells_9
10x_3266_CGTCGACTGCCAAT-1 Patient3 Dendritic cells nonactive 14 0.10352863 Dendritic cells_14
10x_3266_CGTCGACTTATCTC-1 Patient3 Monocytes nonactive 3 0.05472069 Monocytes_3
10x_3266_CGTGAAACACCTGA-1 Patient3 B cells nonactive 7 0.07631095 B cells_7
10x_3266_CGTGAAACGTTGAC-1 Patient3 CD4+ T cells nonactive 11 0.06467567 CD4+ T cells_11
10x_3266_CGTGAAACTTTGCT-1 Patient3 B cells nonactive 7 0.07269672 B cells_7
10x_3266_CGTGCACTTTCCCG-1 Patient3 CD4+ T cells nonactive 11 0.06014203 CD4+ T cells_11
10x_3266_CGTGTAGAAGCTAC-1 Patient3 B cells nonactive 7 0.0737905 B cells_7
10x_3266_CGTGTAGATTTACC-1 Patient3 T cells nonactive 9 0.06851183 T cells_9
10x_3266_CGTTAACTACCAAC-1 Patient3 CD4+ T cells active 11 0.11903177 CD4+ T cells_11
10x_3266_CGTTAACTACCGAT-1 Patient3 B cells nonactive 7 0.09024475 B cells_7
10x_3266_CGTTTAACCGCTAA-1 Patient3 B cells nonactive 7 0.07451969 B cells_7
10x_3266_CTAACACTACCACA-1 Patient3 B cells nonactive 7 0.06175892 B cells_7
10x_3266_CTAACACTACGGTT-1 Patient3 Monocytes nonactive 0 0.05736795 Monocytes_0
10x_3266_CTAACTACTCAGGT-1 Patient3 B cells nonactive 7 0.11646376 B cells_7
10x_3266_CTAAGGTGTGTCCC-1 Patient3 B cells nonactive 7 0.06854353 B cells_7
10x_3266_CTAATAGATGGGAG-1 Patient3 CD4+ T cells nonactive 11 0.06645108 CD4+ T cells_11

10x_3266_CTAGGATGCCTGAA-1 Patient3 Monocytes nonactive 3 0.05972988 Monocytes_3
10x_3266_CTAGGCCTACTTTC-1 Patient3 CD4+ T cells nonactive 11 0.02639338 CD4+ T cells_11
10x_3266_CTATAAGAAGAGAT-1 Patient3 B cells nonactive 7 0.07535984 B cells_7
10x_3266_CTATAGCTTGACAC-1 Patient3 Monocytes nonactive 0 0.00738698 Monocytes_0
10x_3266_CTATCATGCATTTC-1 Patient3 Monocytes nonactive 3 0.03606303 Monocytes_3
10x_3266_CTATCATGTGCTAG-1 Patient3 T cells nonactive 9 0.07832414 T cells_9
10x_3266_CTATGTACTCACCC-1 Patient3 B cells nonactive 7 0.07578784 B cells_7
10x_3266_CTATTGTGGTACCA-1 Patient3 B cells nonactive 7 0.04481326 B cells_7
10x_3266_CTCATTGATCATTC-1 Patient3 T cells nonactive 9 0.09029231 T cells_9
10x_3266_CTCCACGAAACTGC-1 Patient3 CD4+ T cells nonactive 11 0.06082366 CD4+ T cells_11
10x_3266_CTCCGAACAAGCCT-1 Patient3 B cells nonactive 7 0.06424767 B cells_7
10x_3266_CTCGACACAACGTC-1 Patient3 T cells nonactive 9 0.06714856 T cells_9
10x_3266_CTCGACACGTGTTG-1 Patient3 CD4+ T cells nonactive 11 0.07637436 CD4+ T cells_11
10x_3266_CTCGACTGCATGCA-1 Patient3 CD4+ T cells nonactive 11 0.0476032 CD4+ T cells_11
10x_3266_CTCGCATGTGTTTC-1 Patient3 CD4+ T cells nonactive 11 0.09492106 CD4+ T cells_11
10x_3266_CTCTAAACGGCATT-1 Patient3 Dendritic cells nonactive 14 0.10341767 Dendritic cells_14
10x_3266_CTGACAGAGACGGA-1 Patient3 B cells nonactive 7 0.06351848 B cells_7
10x_3266_CTGACCACTGAGCT-1 Patient3 B cells nonactive 7 0.09720373 B cells_7
10x_3266_CTGAGAACGCTATG-1 Patient3 B cells nonactive 7 0.04739712 B cells_7
10x_3266_CTGAGAACTGCGTA-1 Patient3 B cells nonactive 7 0.0518198 B cells_7
10x_3266_CTGCGACTTTTCAC-1 Patient3 Dendritic cells active 14 0.12418363 Dendritic cells_14
10x_3266_CTGTAACTGTCTAG-1 Patient3 T cells nonactive 9 0.09048253 T cells_9
10x_3266_CTGTGAGAAAGAAC-1 Patient3 B cells nonactive 7 0.09584047 B cells_7

10x_3266_CTTAAGCTATGCCA-1 Patient3 CD4+ T cells nonactive 11 0.1133885 CD4+ T cells_11
10x_3266_CTTACAACGTCCTC-1 Patient3 B cells nonactive 7 0.09926447 B cells_7
10x_3266_CTTACATGCTGCAA-1 Patient3 CD4+ T cells nonactive 11 0.04308541 CD4+ T cells_11
10x_3266_CTTACTGAGCTAAC-1 Patient3 B cells nonactive 7 0.05984085 B cells_7
10x_3266_CTTAGGGAACTACG-1 Patient3 Monocytes nonactive 3 0.040454 Monocytes_3
10x_3266_CTTAGGGAGAAAGT-1 Patient3 B cells nonactive 7 0.08994357 B cells_7
10x_3266_CTTATCGAAGGAGC-1 Patient3 CD4+ T cells nonactive 11 0.06313804 CD4+ T cells_11
10x_3266_CTTCACCTAGAACA-1 Patient3 B cells nonactive 7 0.0636453 B cells_7
10x_3266_CTTCATGAGGTGGA-1 Patient3 CD4+ T cells nonactive 11 0.04673134 CD4+ T cells_11
10x_3266_CTTGAGGACTGTGA-1 Patient3 B cells nonactive 7 0.05494262 B cells_7
10x_3266_CTTGAGGAGGTGGA-1 Patient3 B cells nonactive 7 0.07230042 B cells_7
10x_3266_CTTGATTGAGACTC-1 Patient3 Monocytes nonactive 3 0.04869698 Monocytes_3
10x_3266_CTTGATTGTCAGTG-1 Patient3 T cells nonactive 9 0.09626847 T cells_9
10x_3266_CTTGTATGAGCACT-1 Patient3 B cells nonactive 7 0.08759749 B cells_7
10x_3266_CTTTACGATCTACT-1 Patient3 CD4+ T cells nonactive 11 0.07306132 CD4+ T cells_11
10x_3266_CTTTAGACAGCTCA-1 Patient3 T cells nonactive 9 0.07112739 T cells_9
10x_3266_CTTTAGTGCGGAGA-1 Patient3 B cells nonactive 7 0.10771352 B cells_7
10x_3266_CTTTAGTGCTCGCT-1 Patient3 B cells nonactive 7 0.09782195 B cells_7
10x_3266_CTTTCAGAGCTATG-1 Patient3 B cells nonactive 7 0.06932027 B cells_7
10x_3266_GAAAGATGTTCGCC-1 Patient3 Monocytes nonactive 3 0.06626086 Monocytes_3
10x_3266_GAAAGTGAACTCAG-1 Patient3 Dendritic cells nonactive 14 0.11315072 Dendritic cells_14
10x_3266_GAAAGTGAAGATGA-1 Patient3 B cells nonactive 7 0.09641113 B cells_7
10x_3266_GAACCAACCGCTAA-1 Patient3 T cells nonactive 9 0.0548158 T cells_9
10x_3266_GAACCAACGGTGGA-1 Patient3 Monocytes nonactive 0 0.00240949

Monocytes_0
10x_3266_GAACGTTGTTGTGG-1 Patient3 CD4+ T cells nonactive 11 0.08837423
CD4+ T cells_11
10x_3266_GAAGATGACCATGA-1 Patient3 Dendritic cells nonactive 14 0.10871219
Dendritic cells_14
10x_3266_GAAGCGGAACTCTT-1 Patient3 CD4+ T cells nonactive 11 0.02669457
CD4+ T cells_11
10x_3266_GAAGCGGACTCGCT-1 Patient3 B cells nonactive 7 0.07081035 B cells_7
10x_3266_GAAGCGGACTCTTA-1 Patient3 B cells active 7 0.11914273 B cells_7
10x_3266_GAAGCGGATCTCGC-1 Patient3 T cells nonactive 9 0.08506119 T cells_9
10x_3266_GAAGGGTGTCGTAG-1 Patient3 B cells nonactive 7 0.05283432 B cells_7
10x_3266_GAAGGTCTCGGGAA-1 Patient3 B cells nonactive 7 0.0822237 B cells_7
10x_3266_GAAGTAGACGCCTT-1 Patient3 Progenitors active 12 0.12163148
Progenitors_12
10x_3266_GAAGTAGATTCTGT-1 Patient3 B cells nonactive 7 0.09850358 B cells_7
10x_3266_GAAGTCTGATGACC-1 Patient3 B cells nonactive 7 0.06998605 B cells_7
10x_3266_GAAGTGCTGATAAG-1 Patient3 CD4+ T cells nonactive 11 0.02464967
CD4+ T cells_11
10x_3266_GAATGCACGCTTAG-1 Patient3 CD4+ T cells nonactive 11 0.0539598
CD4+ T cells_11
10x_3266_GACAACTGGGGTGA-1 Patient3 B cells nonactive 7 0.09384313 B cells_7
10x_3266_GACAGTTGAGATCC-1 Patient3 Dendritic cells active 14 0.16409866
Dendritic cells_14
10x_3266_GACAGTTGTGGTGT-1 Patient3 CD4+ T cells nonactive 11 0.04910912
CD4+ T cells_11
10x_3266_GACATTCTTGCTAG-1 Patient3 B cells nonactive 7 0.09519054 B cells_7
10x_3266_GACCCTACTATCTC-1 Patient3 B cells nonactive 7 0.08395156 B cells_7
10x_3266_GACCTAGATGGTTG-1 Patient3 T cells nonactive 9 0.07241139 T cells_9
10x_3266_GACCTCTGAGCTAC-1 Patient3 Monocytes nonactive 0 0.02980153
Monocytes_0
10x_3266_GACCTCTGCTCTCG-1 Patient3 T cells nonactive 9 0.06328071 T cells_9
10x_3266_GACGAACTGTTGGT-1 Patient3 B cells nonactive 7 0.09311394 B cells_7
10x_3266_GACGAGGAAGGAGC-1 Patient3 B cells nonactive 7 0.07580369 B cells_7

10x_3266_GACGATTGGGAAGC-1 Patient3 Progenitors nonactive 5 0.11571872 Progenitors_5
10x_3266_GACGGCACCATGCA-1 Patient3 Monocytes nonactive 3 0.05511699 Monocytes_3
10x_3266_GACTACGAGCCCTT-1 Patient3 B cells nonactive 7 0.0721102 B cells_7
10x_3266_GACTGAACACGGGA-1 Patient3 Monocytes nonactive 2 0.06619745 Monocytes_2
10x_3266_GACTGAACTTGCTT-1 Patient3 Progenitors nonactive 5 0.06865449 Progenitors_5
10x_3266_GACTGTGAACTAGC-1 Patient3 B cells nonactive 7 0.11172405 B cells_7
10x_3266_GACTTTACACTACG-1 Patient3 T cells nonactive 9 0.05513284 T cells_9
10x_3266_GACTTTACGGTAGG-1 Patient3 T cells nonactive 9 0.09268594 T cells_9
10x_3266_GACTTTACTCACGA-1 Patient3 CD4+ T cells nonactive 11 0.04574853 CD4+ T cells_11
10x_3266_GAGATCACCGCTAA-1 Patient3 B cells nonactive 7 0.09731469 B cells_7
10x_3266_GAGATCACTCGCTC-1 Patient3 CD4+ T cells nonactive 11 0.03741044 CD4+ T cells_11
10x_3266_GAGATGCTCTTGGA-1 Patient3 B cells nonactive 7 0.0750745 B cells_7
10x_3266_GAGCAACTACGGAG-1 Patient3 B cells nonactive 7 0.0801154 B cells_7
10x_3266_GAGCAGGACATTTC-1 Patient3 B cells nonactive 7 0.07169805 B cells_7
10x_3266_GAGCGAGATTCTGT-1 Patient3 B cells nonactive 7 0.06716442 B cells_7
10x_3266_GAGGACGATGATGC-1 Patient3 B cells nonactive 7 0.08434785 B cells_7
10x_3266_GAGGCAGAGGTCAT-1 Patient3 B cells nonactive 7 0.0535635 B cells_7
10x_3266_GAGGGATGCCTTAT-1 Patient3 T cells nonactive 9 0.0121901 T cells_9
10x_3266_GAGGGTGACTAGTG-1 Patient3 B cells nonactive 7 0.04920424 B cells_7
10x_3266_GAGGGTGATCGCAA-1 Patient3 CD4+ T cells nonactive 11 0.08802549 CD4+ T cells_11
10x_3266_GAGGTTACGGACGA-1 Patient3 Progenitors nonactive 5 0.07428191 Progenitors_5
10x_3266_GAGGTTTGATTTCC-1 Patient3 CD4+ T cells nonactive 11 0.05530721 CD4+ T cells_11
10x_3266_GAGTGACTAGGTTC-1 Patient3 B cells nonactive 7 0.0454949 B cells_7
10x_3266_GAGTGGGAGTGCTA-1 Patient3 Monocytes nonactive 3 0.0181504

Monocytes_3
10x_3266_GAGTGTTGTGTGAC-1 Patient3 CD4+ T cells nonactive 11 0.03533384
CD4+ T cells_11
10x_3266_GAGTTGTGAGTCTG-1 Patient3 Dendritic cells active 14 0.11752584
Dendritic cells_14
10x_3266_GATAATACTCTTTG-1 Patient3 B cells nonactive 7 0.05687655 B cells_7
10x_3266_GATACTCTAGCCAT-1 Patient3 B cells nonactive 7 0.09279691 B cells_7
10x_3266_GATACTCTCAACCA-1 Patient3 CD4+ T cells nonactive 11 0.06622915
CD4+ T cells_11
10x_3266_GATACTCTTTTCTG-1 Patient3 CD4+ T cells nonactive 11 0.00824298
CD4+ T cells_11
10x_3266_GATATAACTGGTTG-1 Patient3 T cells nonactive 9 0.07112739 T cells_9
10x_3266_GATATATGATTCCT-1Patient3 Monocytes nonactive 0 0.03404984
Monocytes_0
10x_3266_GATATATGTTTGTC-1 Patient3 B cells nonactive 7 0.09382728 B cells_7
10x_3266_GATATCCTAACGGG-1 Patient3 T cells nonactive 9 0.09140194 T cells_9
10x_3266_GATATCCTCCAAGT-1 Patient3 CD4+ T cells nonactive 11 0.05172468
CD4+ T cells_11
10x_3266_GATATCCTTGACTG-1 Patient3 B cells nonactive 7 0.07352102 B cells_7
10x_3266_GATCCCTGTACTGG-1 Patient3 T cells nonactive 9 0.08418933 T cells_9
10x_3266_GATCTTTGTCTACT-1 Patient3 CD4+ T cells nonactive 11 0.06393063
CD4+ T cells_11
10x_3266_GATGACACCTGTAG-1 Patient3 B cells active 7 0.12849534 B cells_7
10x_3266_GATGCAACACCTGA-1 Patient3 B cells nonactive 7 0.10004121 B cells_7
10x_3266_GATGCAACCCGAAT-1 Patient3 Dendritic cells nonactive 14 0.10650878
Dendritic cells_14
10x_3266_GATTCGGAACGGAG-1 Patient3 CD4+ T cells nonactive 11 0.05548158
CD4+ T cells_11
10x_3266_GATTCGGAACGTTG-1 Patient3 Dendritic cells nonactive 14 0.10283115
Dendritic cells_14
10x_3266_GATTCTTGTCTCGC-1 Patient3 B cells nonactive 7 0.04143681 B cells_7
10x_3266_GATTGGTGAACAGA-1 Patient3 B cells nonactive 7 0.06180648 B cells_7
10x_3266_GATTTAGACAAGCT-1 Patient3 B cells nonactive 7 0.07696088 B cells_7

10x_3266_GCAAGACTTATGGC-1 Patient3 B cells nonactive 7 0.03832985 B cells_7
10x_3266_GCACACCTACGACT-1 Patient3 CD4+ T cells nonactive 11 0.03008687 CD4+ T cells_11
10x_3266_GCACACCTGCTTAG-1 Patient3 B cells nonactive 7 0.1003741 B cells_7
10x_3266_GCACCTACACAGTC-1 Patient3 CD4+ T cells nonactive 11 0.08016296 CD4+ T cells_11
10x_3266_GCACCTACTGCTAG-1 Patient3 Dendritic cells nonactive 14 0.08732801 Dendritic cells_14
10x_3266_GCACCTTGCTTCGC-1 Patient3 Progenitors nonactive 5 0.04448038 Progenitors_5
10x_3266_GCAGATACACCAGT-1 Patient3 B cells nonactive 7 0.10993279 B cells_7
10x_3266_GCAGCTCTGAGGCA-1 Patient3 Monocytes nonactive 3 0.00050726 Monocytes_3
10x_3266_GCAGGCACGTTGAC-1 Patient3 CD4+ T cells nonactive 11 0.0354448 CD4+ T cells_11
10x_3266_GCAGGGCTACACCA-1 Patient3 Dendritic cells nonactive 14 0.07065183 Dendritic cells_14
10x_3266_GCAGTTGAGGAACG-1 Patient3 B cells nonactive 7 0.08464904 B cells_7
10x_3266_GCCAAACACACGT-1 Patient3 B cells nonactive 7 0.11064612 B cells_7
10x_3266_GCCACGGAGACGGA-1 Patient3 B cells nonactive 7 0.08594889 B cells_7
10x_3266_GCCACTACGTGTTG-1 Patient3 Monocytes nonactive 3 0.04952127 Monocytes_3
10x_3266_GCCATGCTCGGTAT-1 Patient3 Dendritic cells nonactive 14 0.10097648 Dendritic cells_14
10x_3266_GCCGAGTGACGCTA-1 Patient3 B cells nonactive 7 0.05562425 B cells_7
10x_3266_GCCGAGTGCGGAGA-1 Patient3 Dendritic cells nonactive 14 0.08079703 Dendritic cells_14
10x_3266_GCCGTACTGACGAG-1 Patient3 B cells nonactive 7 0.0822237 B cells_7
10x_3266_GCGACTCTACCAGT-1 Patient3 Monocytes nonactive 2 0.05430854 Monocytes_2
10x_3266_GCGCACGACACTAG-1 Patient3 Progenitors nonactive 5 0.11700273 Progenitors_5
10x_3266_GCGCGATGAGCAAA-1 Patient3 Progenitors active 1 0.17313423

Progenitors_1
10x_3266_GCGGACTGTCGCAA-1 Patient3 T cells nonactive 9 0.05002853 T cells_9
10x_3266_GCGGAGCTGCTACA-1 Patient3 B cells nonactive 7 0.06057003 B cells_7
10x_3266_GCGGAGCTTACGAC-1 Patient3 Progenitors nonactive 5 0.10793545
Progenitors_5
10x_3266_GCGTAATGGGCGAA-1 Patient3 B cells nonactive 7 0.06767168 B cells_7
10x_3266_GCGTAATGTGTCAG-1 Patient3 CD4+ T cells nonactive 11 0.07326739
CD4+ T cells_11
10x_3266_GCTACGCTATCACG-1 Patient3 Monocytes nonactive 3 0.03102213
Monocytes_3
10x_3266_GCTACGCTCTAGTG-1 Patient3 Dendritic cells active 14 0.11987192
Dendritic cells_14
10x_3266_GCTAGAACGGTTTG-1 Patient3 CD4+ T cells nonactive 11 0.0455266
CD4+ T cells_11
10x_3266_GCTCAAGACATGAC-1 Patient3 Dendritic cells active 14 0.12618097
Dendritic cells_14
10x_3266_GCTCAAGAGGTGTT-1 Patient3 Monocytes nonactive 0 0.05676558
Monocytes_0
10x_3266_GCTCAGCTGGGATG-1 Patient3 B cells nonactive 7 0.05739966 B cells_7
10x_3266_GCTCAGCTTGTCCC-1 Patient3 T cells active 9 0.14217551 T cells_9
10x_3266_GCTTAACTTGGTAC-1 Patient3 B cells nonactive 7 0.05711432 B cells_7
10x_3266_GCTTAACTTTTCTG-1 Patient3 Monocytes nonactive 3 0.02831146
Monocytes_3
10x_3266_GGAACACTATGCCA-1 Patient3 Monocytes nonactive 0 0.06017374
Monocytes_0
10x_3266_GGAACACTGCATAC-1 Patient3 Progenitors nonactive 5 0.04597045
Progenitors_5
10x_3266_GGAACTACAAGAAC-1 Patient3 T cells nonactive 9 0.10810982 T cells_9
10x_3266_GGAATCTGCTCCCA-1 Patient3 CD4+ T cells nonactive 11 0.05427684
CD4+ T cells_11
10x_3266_GGACATTGCATTTC-1 Patient3 B cells nonactive 7 0.06508782 B cells_7
10x_3266_GGACCCGAGACAAA-1 Patient3 B cells nonactive 7 0.08374548 B cells_7
10x_3266_GGACCCGATCTCCG-1 Patient3 B cells nonactive 7 0.07957644 B cells_7

10x_3266_GGACTATGCTCAGA-1 Patient3 CD4+ T cells nonactive 11 0.05159787 CD4+ T cells_11
10x_3266_GGAGACGAGAATAG-1 Patient3 Monocytes nonactive 16 0.06251981 Monocytes_16
10x_3266_GGAGAGACCTGTGA-1 Patient3 B cells nonactive 7 0.07833999 B cells_7
10x_3266_GGAGGTGAACCCAA-1 Patient3 T cells nonactive 9 0.0776108 T cells_9
10x_3266_GGAGTTTGATGTGC-1 Patient3 B cells nonactive 7 0.08510874 B cells_7
10x_3266_GGAGTTTGCTCGCT-1 Patient3 Monocytes nonactive 0 0.03143428 Monocytes_0
10x_3266_GGATGTTGGAATCC-1 Patient3 CD4+ T cells nonactive 11 0.06776679 CD4+ T cells_11
10x_3266_GGCAATACCGTTGA-1 Patient3 B cells nonactive 7 0.07528058 B cells_7
10x_3266_GGCCCAGAGCCATA-1 Patient3 Monocytes nonactive 15 0.05624247 Monocytes_15
10x_3266_GGCGACACTGACAC-1 Patient3 Monocytes nonactive 15 0.06090292 Monocytes_15
10x_3266_GGCGCATGAGTAGA-1 Patient3 Monocytes nonactive 0 0.02426923 Monocytes_0
10x_3266_GGCGCATGGAGGTG-1 Patient3 Monocytes nonactive 13 0.03170376 Monocytes_13
10x_3266_GGCGCATGTACTGG-1 Patient3 B cells nonactive 7 0.08758164 B cells_7
10x_3266_GGCGGACTCTAAGC-1 Patient3 B cells nonactive 7 0.08517215 B cells_7
10x_3266_GGCGGACTTAACCG-1 Patient3 B cells active 7 0.12318496 B cells_7
10x_3266_GGCTCACTAGTAGA-1 Patient3 CD4+ T cells nonactive 11 0.04792023 CD4+ T cells_11
10x_3266_GGGAACGATTCGGA-1 Patient3 B cells nonactive 7 0.08599645 B cells_7
10x_3266_GGGAAGTGTAACCG-1 Patient3 B cells nonactive 7 0.05635343 B cells_7
10x_3266_GGGACCACTGTAGC-1 Patient3 B cells nonactive 7 0.07640606 B cells_7
10x_3266_GGGATTACAGTCTG-1 Patient3 CD4+ T cells nonactive 11 0.07691332 CD4+ T cells_11
10x_3266_GGGCACACCATTCT-1 Patient3 B cells nonactive 7 0.03598377 B cells_7
10x_3266_GGGCCATGGTAGCT-1 Patient3 T cells nonactive 9 0.08556845 T cells_9
10x_3266_GGTACTGACCTTAT-1 Patient3 Progenitors nonactive 5 0.05800203

Progenitors_5

10x_3266_GGTACTGATACTCT-1 Patient3 T cells nonactive 9 0.04544734 T cells_9

10x_3266_GGTAGTACCTGTCC-1 Patient3 T cells nonactive 9 0.07661214 T cells_9

10x_3266_GGTATCGACTACCC-1 Patient3 B cells nonactive 7 0.05608395 B cells_7

10x_3266_GGTATCGATACTTC-1 Patient3 Dendritic cells nonactive 14 0.11476761 Dendritic cells_14

10x_3266_GGTTGAACCACAAC-1 Patient3 T cells nonactive 9 0.10677826 T cells_9

10x_3266_GGTTGAACCGGTAT-1 Patient3 B cells nonactive 7 0.05727284 B cells_7

10x_3266_GGTTGAACTCATTC-1 Patient3 B cells nonactive 7 0.08553674 B cells_7

10x_3266_GTAATAACAGCTCA-1 Patient3 B cells nonactive 7 0.05655951 B cells_7

10x_3266_GTAATAACCCGCTT-1 Patient3 B cells nonactive 7 0.06350263 B cells_7

10x_3266_GTAATATGTCCTCG-1 Patient3 Monocytes nonactive 3 0.03683977 Monocytes_3

10x_3266_GTACTTTGGGGAGT-1 Patient3 B cells nonactive 7 0.06725953 B cells_7

10x_3266_GTAGCATGTGGTTG-1 Patient3 Monocytes nonactive 3 0.0552438 Monocytes_3

10x_3266_GTAGCCCTCCATAG-1 Patient3 T cells nonactive 9 0.07212605 T cells_9

10x_3266_GTAGCCCTTTTCTG-1 Patient3 B cells nonactive 7 0.09286031 B cells_7

10x_3266_GTAGTCGAGGTTAC-1 Patient3 Monocytes nonactive 15 0.03121235 Monocytes_15

10x_3266_GTAGTGACACAGCT-1 Patient3 T cells nonactive 9 0.06727538 T cells_9

10x_3266_GTAGTGACACAGTC-1 Patient3 B cells nonactive 7 0.06678397 B cells_7

10x_3266_GTAGTGACCGACTA-1 Patient3 B cells nonactive 7 0.09639528 B cells_7

10x_3266_GTAGTGTGTCTTCA-1 Patient3 B cells nonactive 7 0.10333841 B cells_7

10x_3266_GTATCACTCGTACA-1 Patient3 B cells nonactive 7 0.04937861 B cells_7

10x_3266_GTATCTACCGAGTT-1 Patient3 B cells nonactive 7 0.1070636 B cells_7

10x_3266_GTATTAGACCTTAT-1 Patient3 CD4+ T cells nonactive 11 0.05871536 CD4+ T cells_11

10x_3266_GTATTAGATCTCGC-1 Patient3 Progenitors nonactive 5 0.09962907 Progenitors_5

10x_3266_GTCACAGAGCATAC-1 Patient3 Dendritic cells nonactive 14 0.1171771 Dendritic cells_14

10x_3266_GTCACCTGCGCAAT-1 Patient3 B cells active 7 0.12236066 B cells_7

10x_3266_GTCACCTGGATGAA-1 Patient3 CD4+ T cells nonactive 11 0.06507197 CD4+ T cells_11
10x_3266_GTCCAAGAAGTCTG-1 Patient3 B cells nonactive 7 0.08144696 B cells_7
10x_3266_GTCTGAGAATCGTG-1 Patient3 CD4+ T cells nonactive 11 0.09928032 CD4+ T cells_11
10x_3266_GTCTGAGACATTGG-1 Patient3 T cells nonactive 9 0.08276267 T cells_9
10x_3266_GTGATGACGGTCAT-1 Patient3 Progenitors nonactive 6 0.11383235 Progenitors_6
10x_3266_GTGATTCTACCTCC-1 Patient3 B cells nonactive 7 0.07245894 B cells_7
10x_3266_GTGCAAACAGCGGA-1 Patient3 Dendritic cells nonactive 14 0.11034494 Dendritic cells_14
10x_3266_GTGCCACTGTAAAG-1 Patient3 B cells nonactive 7 0.07141272 B cells_7
10x_3266_GTGTACGAACCACA-1 Patient3 B cells nonactive 7 0.09856699 B cells_7
10x_3266_GTGTCAGAGACGGA-1 Patient3 Monocytes nonactive 15 0.0248399 Monocytes_15
10x_3266_GTTAAATGACAGTC-1 Patient3 B cells nonactive 7 0.07467821 B cells_7
10x_3266_GTTAACCTGCATCA-1 Patient3 B cells nonactive 7 0.09725128 B cells_7
10x_3266_GTTAGGTGCTCATT-1 Patient3 T cells nonactive 9 0.06792531 T cells_9
10x_3266_GTTATCTGACGGTT-1 Patient3 B cells nonactive 7 0.0771511 B cells_7
10x_3266_GTTATCTGCCTCCA-1 Patient3 T cells nonactive 9 0.05166128 T cells_9
10x_3266_GTTGACGAGAAAGT-1 Patient3 CD4+ T cells nonactive 11 0.07512206 CD4+ T cells_11
10x_3266_GTTGACGATCTCCG-1 Patient3 B cells nonactive 7 0.07440872 B cells_7
10x_3266_GTTGAGTGGGATTC-1 Patient3 Monocytes nonactive 15 0.06167967 Monocytes_15
10x_3266_GTTGATCTCGTGAT-1 Patient3 B cells nonactive 7 0.07967155 B cells_7
10x_3266_GTTGGATGAACAGA-1 Patient3 Dendritic cells active 14 0.14441063 Dendritic cells_14
10x_3266_GTTGTACTCTCAAG-1 Patient3 B cells nonactive 7 0.08564771 B cells_7
10x_3266_GTTTAAGAGTCCTC-1 Patient3 B cells nonactive 7 0.07022383 B cells_7
10x_3266_TAAATCGAGCTATG-1 Patient3 B cells nonactive 7 0.07163465 B cells_7
10x_3266_TAAATGTGCATTGG-1 Patient3 Monocytes nonactive 3 0.04146852 Monocytes_3

10x_3266_TAAATGTGTGGTTG-1 Patient3 B cells nonactive 7 0.08404667 B cells_7
10x_3266_TAACAATGCCCAAA-1 Patient3 B cells nonactive 7 0.05684484 B cells_7
10x_3266_TAACAATGGAGGTG-1 Patient3 Monocytes nonactive 3 0.05895314 Monocytes_3
10x_3266_TAACAATGGCTCCT-1 Patient3 Dendritic cells nonactive 14 0.1104876 Dendritic cells_14
10x_3266_TAACACCTTCGCTC-1 Patient3 B cells nonactive 7 0.0974098 B cells_7
10x_3266_TAAGCGTGGCATCA-1 Patient3 Dendritic cells nonactive 14 0.10547841 Dendritic cells_14
10x_3266_TAAGTAACAGCTCA-1 Patient3 Monocytes nonactive 0 0.06580115 Monocytes_0
10x_3266_TAATCCACCTTAGG-1 Patient3 B cells nonactive 7 0.08339674 B cells_7
10x_3266_TAATGCCTACACGT-1 Patient3 B cells nonactive 7 0.10392493 B cells_7
10x_3266_TAATGCCTGCTAAC-1 Patient3 B cells nonactive 7 0.07921184 B cells_7
10x_3266_TAATGTGAGAAAGT-1 Patient3 B cells nonactive 7 0.08590134 B cells_7
10x_3266_TACACACTAAGGTA-1 Patient3 B cells nonactive 7 0.09932788 B cells_7
10x_3266_TACATAGACAAAGA-1 Patient3 Monocytes nonactive 3 0.05280261 Monocytes_3
10x_3266_TACATAGATGAGGG-1 Patient3 Dendritic cells active 14 0.11999873 Dendritic cells_14
10x_3266_TACATCACATGTGC-1 Patient3 B cells nonactive 7 0.10252996 B cells_7
10x_3266_TACCGAGACTTAGG-1 Patient3 B cells nonactive 7 0.06527804 B cells_7
10x_3266_TACCGGCTTATCTC-1 Patient3 Monocytes nonactive 3 0.03460465 Monocytes_3
10x_3266_TACGACGAAAACAG-1 Patient3 Dendritic cells nonactive 14 0.0889766 Dendritic cells_14
10x_3266_TACGCCACCGGGAA-1 Patient3 T cells nonactive 9 0.06694249 T cells_9
10x_3266_TACGGAACCGCTAA-1 Patient3 CD4+ T cells nonactive 11 0.09479424 CD4+ T cells_11
10x_3266_TACTGGGAACCTTT-1 Patient3 Dendritic cells active 14 0.1293989 Dendritic cells_14
10x_3266_TACTTGACGTTTCT-1 Patient3 B cells nonactive 7 0.08930949 B cells_7
10x_3266_TAGAATACCTGACA-1 Patient3 B cells nonactive 7 0.06060174 B cells_7

10x_3266_TAGAATTGCCACAA-1	Patient3 B cells	nonactive	7	0.05611566	B cells_7
10x_3266_TAGACGTGTAACGC-1	Patient3 B cells	nonactive	7	0.0974098	B cells_7
10x_3266_TAGAGAGAGAACCT-1	Patient3 Monocytes	nonactive	0	0.04318052
Monocytes_0
10x_3266_TAGAGAGAGAGGGT-1	Patient3 B cells	nonactive	7	0.08060681	B cells_7
10x_3266_TAGAGCACTCTATC-1	Patient3 B cells	nonactive	7	0.08404667	B cells_7
10x_3266_TAGATTGAGCGGAA-1	Patient3 B cells	nonactive	7	0.08184326	B cells_7
10x_3266_TAGCCCACTGCAGT-1	Patient3 B cells	nonactive	7	0.07196754	B cells_7
10x_3266_TAGCCGCTCGCATA-1	Patient3 B cells	nonactive	7	0.0762951	B cells_7
10x_3266_TAGCCGCTCGTGAT-1	Patient3 B cells	nonactive	7	0.06694249	B cells_7
10x_3266_TAGCGATGGCGTTA-1	Patient3 T cells	nonactive	9	0.09343098	T cells_9
10x_3266_TAGGACTGGTTAGC-1	Patient3 T cells	nonactive	9	0.07444043	T cells_9
10x_3266_TAGGTGTGAGTACC-1	Patient3 CD4+ T cells	nonactive	11	0.0374897
CD4+ T cells_11
10x_3266_TAGGTGTGTCAAGC-1	Patient3 B cells	nonactive	7	0.10251411	B cells_7
10x_3266_TAGTACCTTTCCGC-1	Patient3 Dendritic cells	nonactive	14	0.11454569
Dendritic cells_14
10x_3266_TAGTCACTTCCAGA-1	Patient3 B cells	nonactive	7	0.09520639	B cells_7
10x_3266_TAGTCGGATCCAGA-1	Patient3 Monocytes	nonactive	0	0.06136263
Monocytes_0
10x_3266_TAGTCTTGAGCGTT-1	Patient3 B cells	nonactive	7	0.10972671	B cells_7
10x_3266_TAGTCTTGGGAAAT-1	Patient3 B cells	nonactive	7	0.05513284	B cells_7
10x_3266_TAGTTGCTTCCTGC-1	Patient3 B cells	nonactive	7	0.08165303	B cells_7
10x_3266_TATAAGTGCCTAAG-1	Patient3 B cells	nonactive	7	0.09758417	B cells_7
10x_3266_TATAGCCTACCTTT-1	Patient3 B cells	nonactive	7	0.08017881	B cells_7
10x_3266_TATCAAGAGTACAC-1	Patient3 B cells	nonactive	7	0.09935958	B cells_7
10x_3266_TATCAGCTTTCGCC-1	Patient3 T cells	nonactive	9	0.05235876	T cells_9
10x_3266_TATCCTGATAGACC-1	Patient3 CD4+ T cells	nonactive	11	0.06714856
CD4+ T cells_11
10x_3266_TATCGACTACCCAA-1	Patient3 CD4+ T cells	nonactive	11	0.06724368
CD4+ T cells_11
10x_3266_TATCTGACCCAAGT-1	Patient3 B cells	nonactive	7	0.06670471	B cells_7
10x_3266_TATCTTCTCTTTAC-1	Patient3 B cells	nonactive	7	0.06675227	B cells_7

10x_3266_TATGAATGCCTGAA-1 Patient3 B cells nonactive 7 0.07220531 B cells_7
10x_3266_TATGGGACCCTCCA-1 Patient3 CD4+ T cells nonactive 11 0.06681567 CD4+ T cells_11
10x_3266_TATGGGTGCGGTAT-1 Patient3 Dendritic cells nonactive 14 0.07822903 Dendritic cells_14
10x_3266_TATGGTCTCTGAAC-1 Patient3 B cells nonactive 7 0.06101389 B cells_7
10x_3266_TATGTCTGCCGATA-1 Patient3 B cells nonactive 7 0.104131 B cells_7
10x_3266_TATGTCTGGCGAGA-1 Patient3 T cells nonactive 9 0.08260415 T cells_9
10x_3266_TATTGCTGCCCTCA-1 Patient3 CD4+ T cells nonactive 11 0.05291358 CD4+ T cells_11
10x_3266_TCAACACTTTACCT-1 Patient3 B cells nonactive 7 0.080908 B cells_7
10x_3266_TCAAGGACCCTGAA-1 Patient3 T cells nonactive 9 0.04539978 T cells_9
10x_3266_TCAAGGTGCGTACA-1 Patient3 B cells nonactive 7 0.08935705 B cells_7
10x_3266_TCAATAGACTAAGC-1 Patient3 T cells active 9 0.14593241 T cells_9
10x_3266_TCAATCACAGGGTG-1 Patient3 CD4+ T cells nonactive 11 0.06044322 CD4+ T cells_11
10x_3266_TCACATACGGTACT-1 Patient3 CD4+ T cells nonactive 11 0.02498256 CD4+ T cells_11
10x_3266_TCACCGTGGCCAAT-1 Patient3 CD4+ T cells nonactive 11 0.04744468 CD4+ T cells_11
10x_3266_TCACTATGACCACA-1 Patient3 T cells nonactive 9 0.06732293 T cells_9
10x_3266_TCACTATGCTGAGT-1 Patient3 CD4+ T cells nonactive 11 0.04221356 CD4+ T cells_11
10x_3266_TCAGACGAGGGACA-1 Patient3 B cells nonactive 7 0.0758671 B cells_7
10x_3266_TCAGCAGAGGATCT-1 Patient3 B cells nonactive 7 0.05212098 B cells_7
10x_3266_TCAGTACTATTCGG-1 Patient3 Progenitors active 6 0.12816245 Progenitors_6
10x_3266_TCATCAACAAAAGC-1 Patient3 B cells nonactive 7 0.06523049 B cells_7
10x_3266_TCATCATGTTCCCG-1 Patient3 CD4+ T cells nonactive 11 0.05682899 CD4+ T cells_11
10x_3266_TCATTCGAAGAGAT-1 Patient3 Dendritic cells nonactive 14 0.0501395 Dendritic cells_14
10x_3266_TCATTGACTCTTCA-1 Patient3 T cells nonactive 9 0.08028977 T cells_9

10x_3266_TCCACTCTCGGTAT-1 Patient3 CD4+ T cells nonactive 11 0.07497939 CD4+ T cells_11
10x_3266_TCCACTCTTCTCAT-1 Patient3 Progenitors nonactive 5 0.04833238 Progenitors_5
10x_3266_TCCAGAGACATTGG-1 Patient3 Dendritic cells nonactive 14 0.11687591 Dendritic cells_14
10x_3266_TCCCACGACTTGAG-1 Patient3 B cells nonactive 7 0.06584871 B cells_7
10x_3266_TCCCGAACGACGAG-1 Patient3 B cells nonactive 7 0.07130176 B cells_7
10x_3266_TCCCGAACTGTGGT-1 Patient3 B cells nonactive 7 0.08696341 B cells_7
10x_3266_TCCCGATGCTGATG-1 Patient3 B cells nonactive 7 0.10149959 B cells_7
10x_3266_TCCGAAGAGCTCCT-1 Patient3 Dendritic cells nonactive 14 0.09235305 Dendritic cells_14
10x_3266_TCCGAAGAGGCAAG-1 Patient3 T cells nonactive 9 0.07509036 T cells_9
10x_3266_TCCGAGCTCCATAG-1 Patient3 B cells nonactive 7 0.0657853 B cells_7
10x_3266_TCCTAAACCCGAAT-1 Patient3 Monocytes nonactive 16 0.04844335 Monocytes_16
10x_3266_TCCTAATGAATCGC-1 Patient3 B cells nonactive 7 0.11315072 B cells_7
10x_3266_TCGAATCTCACTAG-1 Patient3 B cells nonactive 7 0.08125674 B cells_7
10x_3266_TCGACGCTTATGCG-1 Patient3 CD4+ T cells nonactive 11 0.05002853 CD4+ T cells_11
10x_3266_TCGCACACCTATGG-1 Patient3 B cells nonactive 7 0.07372709 B cells_7
10x_3266_TCGCACTGTCGTGA-1 Patient3 CD4+ T cells nonactive 11 0.10635026 CD4+ T cells_11
10x_3266_TCGCAGCTACTGGT-1 Patient3 B cells nonactive 7 0.08425274 B cells_7
10x_3266_TCGGCACTTGTGGT-1 Patient3 B cells nonactive 7 0.0973464 B cells_7
10x_3266_TCGTTATGAAAACG-1 Patient3 CD4+ T cells nonactive 11 0.05082113 CD4+ T cells_11
10x_3266_TCTAGACTGTCTAG-1 Patient3 Monocytes nonactive 0 0.04773001 Monocytes_0
10x_3266_TCTAGTTGCTTTAC-1 Patient3 B cells nonactive 7 0.09775854 B cells_7
10x_3266_TCTATGTGTTCCAT-1 Patient3 T cells nonactive 9 0.04749223 T cells_9
10x_3266_TCTCAAACACCAGT-1 Patient3 CD4+ T cells nonactive 11 0.06854353 CD4+ T cells_11

10x_3266_TCTCAAACTCTATC-1 Patient3 CD4+ T cells nonactive 11 0.05497432 CD4+ T cells_11
10x_3266_TCTCCACTCTTCCG-1 Patient3 B cells nonactive 7 0.09260668 B cells_7
10x_3266_TCTTACGACTCATT-1 Patient3 T cells nonactive 9 0.09961321 T cells_9
10x_3266_TCTTACGACTGTAG-1 Patient3 Dendritic cells nonactive 14 0.09972418 Dendritic cells_14
10x_3266_TCTTCAGAGTTCAG-1 Patient3 CD4+ T cells nonactive 11 0.05990425 CD4+ T cells_11
10x_3266_TGAAATTGGTCACA-1 Patient3 T cells nonactive 9 0.10002536 T cells_9
10x_3266_TGAACCGAAACGGG-1 Patient3 B cells nonactive 7 0.07734132 B cells_7
10x_3266_TGAACCGATCTGGA-1 Patient3 Monocytes nonactive 3 0.0354131 Monocytes_3
10x_3266_TGAAGCTGTGCCAA-1 Patient3 Monocytes nonactive 3 0.05804958 Monocytes_3
10x_3266_TGACCGCTTAGAGA-1 Patient3 Progenitors nonactive 12 0.1167491 Progenitors_12
10x_3266_TGACTTACTTGCTT-1 Patient3 Monocytes nonactive 15 0.061109 Monocytes_15
10x_3266_TGAGCAACCTGTAG-1 Patient3 Monocytes nonactive 16 0.03359013 Monocytes_16
10x_3266_TGATAAACTTACTC-1 Patient3 CD4+ T cells nonactive 11 0.01685055 CD4+ T cells_11
10x_3266_TGATCGGACACCAA-1 Patient3 Monocytes nonactive 0 0.09815484 Monocytes_0
10x_3266_TGATTCACGGATTC-1 Patient3 Monocytes nonactive 0 0.02098789 Monocytes_0
10x_3266_TGATTCTGTGCTAG-1 Patient3 B cells nonactive 7 0.09027646 B cells_7
10x_3266_TGCAACGAGCTAAC-1 Patient3 Dendritic cells nonactive 14 0.09974003 Dendritic cells_14
10x_3266_TGCAAGTGGAAAGT-1 Patient3 T cells nonactive 9 0.06472323 T cells_9
10x_3266_TGCCACTGTGTCGA-1 Patient3 T cells nonactive 9 0.03048317 T cells_9
10x_3266_TGCCAGCTAGCCTA-1 Patient3 Dendritic cells active 14 0.12879653 Dendritic cells_14

10x_3266_TGCCAGCTTATGGC-1 Patient3 B cells nonactive 7 0.07486843 B cells_7
10x_3266_TGCCAGCTTCTCAT-1 Patient3 B cells nonactive 7 0.04460719 B cells_7
10x_3266_TGCCAGCTTGCTTT-1 Patient3 T cells nonactive 9 0.08339674 T cells_9
10x_3266_TGCCCAACAAGCCT-1 Patient3 CD4+ T cells nonactive 11 0.0501712 CD4+ T cells_11
10x_3266_TGCCGACTATAAGG-1 Patient3 B cells nonactive 7 0.06225033 B cells_7
10x_3266_TGCGCACTTTGGTG-1 Patient3 B cells nonactive 7 0.05369032 B cells_7
10x_3266_TGCGTAGAACGTAC-1 Patient3 B cells nonactive 7 0.10497115 B cells_7
10x_3266_TGCTAGGAGGTAAA-1 Patient3 Progenitors nonactive 5 0.08609156 Progenitors_5
10x_3266_TGCTATACATCGGT-1 Patient3 B cells nonactive 7 0.04522541 B cells_7
10x_3266_TGGAAGCTAATGCC-1 Patient3 CD4+ T cells nonactive 11 0.04121489 CD4+ T cells_11
10x_3266_TGGAAGCTCAAGCT-1 Patient3 CD4+ T cells nonactive 11 0.02382538 CD4+ T cells_11
10x_3266_TGGACCCTCCTGTC-1 Patient3 Monocytes nonactive 0 0.04682645 Monocytes_0
10x_3266_TGGACCCTTGTAGC-1 Patient3 B cells nonactive 7 0.04872868 B cells_7
10x_3266_TGGACTGAAGAGAT-1 Patient3 CD4+ T cells nonactive 11 0.06977998 CD4+ T cells_11
10x_3266_TGGACTGACTTGTT-1 Patient3 CD4+ T cells nonactive 11 0.06041151 CD4+ T cells_11
10x_3266_TGGAGACTGGACGA-1 Patient3 Progenitors active 5 0.13730898 Progenitors_5
10x_3266_TGGAGGGAGCTTAG-1 Patient3 CD4+ T cells nonactive 11 0.03787014 CD4+ T cells_11
10x_3266_TGGATCGATCTCAT-1 Patient3 CD4+ T cells nonactive 11 0.04639845 CD4+ T cells_11
10x_3266_TGGATCGATGCCTC-1 Patient3 B cells nonactive 7 0.07841925 B cells_7
10x_3266_TGGATGACCGAGAG-1 Patient3 T cells nonactive 9 0.03393888 T cells_9
10x_3266_TGGATTCTCTTAGG-1 Patient3 B cells nonactive 7 0.05285017 B cells_7
10x_3266_TGGATTCTTGACCA-1 Patient3 CD4+ T cells nonactive 11 0.06722782 CD4+ T cells_11

10x_3266_TGGGTATGTATGGC-1 Patient3 CD4+ T cells nonactive 11 0.10969501 CD4+ T cells_11
10x_3266_TGGTAGACAGTCGT-1 Patient3 T cells nonactive 9 0.04812631 T cells_9
10x_3266_TGGTCAGACAGTTG-1 Patient3 Monocytes nonactive 2 0.03435102 Monocytes_2
10x_3266_TGGTTACTTTCACT-1 Patient3 B cells nonactive 7 0.07742058 B cells_7
10x_3266_TGTAACCTATTCCT-1 Patient3 CD4+ T cells nonactive 11 0.04129415 CD4+ T cells_11
10x_3266_TGTAATGATTCGCC-1 Patient3 B cells nonactive 7 0.09446135 B cells_7
10x_3266_TGTACTTGCAATCG-1 Patient3 T cells nonactive 9 0.08831082 T cells_9
10x_3266_TGTCAGGACATACG-1 Patient3 Monocytes nonactive 3 0.03016613 Monocytes_3
10x_3266_TGTGAGACCACAAC-1 Patient3 B cells nonactive 7 0.09290787 B cells_7
10x_3266_TGTTAAGAACCTCC-1 Patient3 Monocytes nonactive 3 0.02509353 Monocytes_3
10x_3266_TTACAGCTCCCTTG-1 Patient3 B cells nonactive 7 0.08669393 B cells_7
10x_3266_TTACCATGAAGGTA-1 Patient3 B cells nonactive 7 0.09915351 B cells_7
10x_3266_TTACGACTGCATCA-1 Patient3 Monocytes nonactive 15 0.03901148 Monocytes_15
10x_3266_TTACGTACAACAGA-1 Patient3 T cells nonactive 9 0.09336757 T cells_9
10x_3266_TTAGAATGAGAATG-1 Patient3 Monocytes nonactive 3 0.04192822 Monocytes_3
10x_3266_TTAGAATGTCTCGC-1 Patient3 CD4+ T cells nonactive 11 0.0648976 CD4+ T cells_11
10x_3266_TTAGAATGTTCTTG-1 Patient3 T cells nonactive 9 0.07312472 T cells_9
10x_3266_TTATCCGAGGCGAA-1 Patient3 B cells nonactive 7 0.07361613 B cells_7
10x_3266_TTCAAAGATTCTAC-1 Patient3 CD4+ T cells nonactive 11 0.06082366 CD4+ T cells_11
10x_3266_TTCACAACAGAATG-1 Patient3 B cells nonactive 7 0.10362374 B cells_7
10x_3266_TTCACAACATCAGC-1 Patient3 B cells nonactive 7 0.06529389 B cells_7
10x_3266_TTCAGTACTCAGTG-1 Patient3 B cells nonactive 7 0.10211781 B cells_7
10x_3266_TTCATGACGTTGAC-1 Patient3 B cells nonactive 7 0.08984846 B cells_7
10x_3266_TTCATGTGAACGTC-1 Patient3 B cells nonactive 7 0.10167396 B cells_7

10x_3266_TTCATGTGACCTCC-1	Patient3 T cells	nonactive	9	0.05508528	T cells_9
10x_3266_TTCCAAACTTCTCA-1	Patient3 B cells	nonactive	7	0.07410754	B cells_7
10x_3266_TTCCTAGAAAGCCT-1	Patient3 Progenitors	nonactive	5	0.09699765	Progenitors_5
10x_3266_TTCGGAGAAGCTCA-1	Patient3 Progenitors	nonactive	5	0.0619967	Progenitors_5
10x_3266_TTCTACGACTCATT-1	Patient3 CD4+ T cells	nonactive	11	0.08691586	CD4+ T cells_11
10x_3266_TTCTACGAGGAGCA-1	Patient3 Dendritic cells	nonactive	14	0.10841101	Dendritic cells_14
10x_3266_TTCTACGATACTCT-1	Patient3 Dendritic cells	nonactive	14	0.09498447	Dendritic cells_14
10x_3266_TTCTAGTGTCAGTG-1	Patient3 CD4+ T cells	nonactive	11	0.07412339	CD4+ T cells_11
10x_3266_TTCTAGTGTTACTC-1	Patient3 Dendritic cells	active	14	0.1328229	Dendritic cells_14
10x_3266_TTGAGGTGGCGGAA-1	Patient3 B cells	nonactive	7	0.10357618	B cells_7
10x_3266_TTGCTAACGAAACA-1	Patient3 Monocytes	nonactive	0	0.0173261	Monocytes_0
10x_3266_TTGCTATGGCGTTA-1	Patient3 B cells	nonactive	7	0.08659882	B cells_7
10x_3266_TTGGAGACTGAGAA-1	Patient3 CD4+ T cells	nonactive	11	0.05175639	CD4+ T cells_11
10x_3266_TTGGAGACTGTCGA-1	Patient3 T cells	nonactive	9	0.10986938	T cells_9
10x_3266_TTGTACACCACTCC-1	Patient3 B cells	active	7	0.14049521	B cells_7
10x_3266_TTGTACACGACGTT-1	Patient3 B cells	nonactive	7	0.084332	B cells_7
10x_3266_TTTAGAGACCCTTG-1	Patient3 B cells	nonactive	7	0.07548665	B cells_7
10x_3266_TTTAGCTGTAAAGG-1	Patient3 B cells	nonactive	7	0.10382981	B cells_7
10x_3266_TTTAGGCTGGATTC-1	Patient3 CD4+ T cells	nonactive	11	0.03595206	CD4+ T cells_11
10x_3266_TTTATCCTACGGTT-1	Patient3 Dendritic cells	active	14	0.12537252	Dendritic cells_14
10x_3266_TTTCACGAACCAGT-1	Patient3 T cells	nonactive	9	0.10860123	T cells_9
10x_3266_TTTCACGAGAATAG-1	Patient3 B cells	nonactive	7	0.07022383	B cells_7

10x_3266_TTTCACGAGAGGAC-1	Patient3	CD4+ T cells	nonactive	11	0.06884471	CD4+ T cells_11
10x_3266_TTTCAGTGCAATCG-1	Patient3	CD4+ T cells	nonactive	11	0.0539915	CD4+ T cells_11
10x_3266_TTTCGAACAGATCC-1	Patient3	T cells	nonactive	9	0.08751823	T cells_9
10x_3266_TTTGACTGCGCCTT-1	Patient3	T cells	nonactive	9	0.03731533	T cells_9
10x_3266_TTTGCATGGCCCTT-1	Patient3	B cells	nonactive	7	0.06256737	B cells_7
10x_3266_TTTGCATGTGTCCC-1	Patient3	T cells	nonactive	9	0.0582081	T cells_9
10x_3250_t2_AAACATTGTTCTGT-1	Patient2	Progenitors	active	12	0.15829687	Progenitors_12
10x_3250_t2_AAACTTGAGCTATG-1	Patient2	Progenitors	nonactive	5	0.11649547	Progenitors_5
10x_3250_t2_AAAGACGAAGTAGA-1	Patient2	Progenitors	nonactive	5	0.07572443	Progenitors_5
10x_3250_t2_AAAGAGACATTTCC-1	Patient2	Progenitors	nonactive	5	0.08810475	Progenitors_5
10x_3250_t2_AAAGGCCTTTTACC-1	Patient2	Progenitors	nonactive	5	0.09224209	Progenitors_5
10x_3250_t2_AAATACTGACGTAC-1	Patient2	Progenitors	nonactive	5	0.09517469	Progenitors_5
10x_3250_t2_AAATCCCTAAGCAA-1	Patient2	Progenitors	nonactive	12	0.11099486	Progenitors_12
10x_3250_t2_AACACGTGGGTTAC-1	Patient2	Progenitors	nonactive	5	0.11608332	Progenitors_5
10x_3250_t2_AACATTGAGGAGTG-1	Patient2	Progenitors	nonactive	5	0.07450384	Progenitors_5
10x_3250_t2_AACATTGAGTAAGA-1	Patient2	Progenitors	nonactive	5	0.0813677	Progenitors_5
10x_3250_t2_AACCAGTGGGGAGT-1	Patient2	Progenitors	nonactive	12	0.06939953	Progenitors_12
10x_3250_t2_AACCGCCTCGACAT-1	Patient2	Progenitors	nonactive	5	0.07486843	Progenitors_5
10x_3250_t2_AACCTTACAAGATG-1	Patient2	Progenitors	nonactive	5	0.07141272	

Progenitors_5
10x_3250_t2_AACCTTACATGTGC-1 Patient2 Progenitors active 12 0.13310824
Progenitors_12
10x_3250_t2_AACCTTACGGGCAA-1 Patient2 Progenitors nonactive 5 0.09867795
Progenitors_5
10x_3250_t2_AACGGTACTGTTTC-1 Patient2 Progenitors active 1 0.16842623
Progenitors_1
10x_3250_t2_AACTCGGAACTACG-1 Patient2 Progenitors nonactive 5 0.061965
Progenitors_5
10x_3250_t2_AACTCGGAAGCCTA-1 Patient2 Progenitors active 12 0.13215712
Progenitors_12
10x_3250_t2_AAGAACGAGGAGCA-1 Patient2 Progenitors active 5 0.15566546
Progenitors_5
10x_3250_t2_AAGATTACGAGCAG-1 Patient2 Progenitors active 6 0.12822586
Progenitors_6
10x_3250_t2_AAGGCTACTGACTG-1 Patient2 Progenitors nonactive 5 0.07748399
Progenitors_5
10x_3250_t2_AAGGTCACGGTAAA-1 Patient2 Progenitors active 12 0.14563122
Progenitors_12
10x_3250_t2_AAGTCTCTGGTGTT-1 Patient2 Progenitors nonactive 6 0.09477839
Progenitors_6
10x_3250_t2_AAGTTATGATACCG-1 Patient2 T cells nonactive 9 0.08206518 T
cells_9
10x_3250_t2_AATCTCACACCATG-1 Patient2 Progenitors active 12 0.13982943
Progenitors_12
10x_3250_t2_AATGATACGTGTTG-1 Patient2 Progenitors active 5 0.16552533
Progenitors_5
10x_3250_t2_AATGGAGATTACCT-1 Patient2 Progenitors active 12 0.14387166
Progenitors_12
10x_3250_t2_AATGTTGATTGTCT-1 Patient2 Progenitors nonactive 5 0.10875975
Progenitors_5
10x_3250_t2_AATTGATGGTGCTA-1 Patient2 Progenitors nonactive 5 0.11551265
Progenitors_5

10x_3250_t2_ACAAGCACCTTGAG-1 Patient2 Progenitors active 12 0.12621267
Progenitors_12
10x_3250_t2_ACAATCCTTGGTGT-1 Patient2 Progenitors nonactive 5 0.05074187
Progenitors_5
10x_3250_t2_ACAATTGAACCCAA-1 Patient2 Progenitors nonactive 5 0.09152876
Progenitors_5
10x_3250_t2_ACACGTGACGCAAT-1 Patient2 Progenitors nonactive 5 0.09450891
Progenitors_5
10x_3250_t2_ACAGTGACATGACC-1 Patient2 Progenitors active 12 0.16213303
Progenitors_12
10x_3250_t2_ACAGTGTGTCCCAC-1 Patient2 Progenitors nonactive 5 0.08567941
Progenitors_5
10x_3250_t2_ACATCACTGGGCAA-1 Patient2 Progenitors nonactive 5 0.09214698
Progenitors_5
10x_3250_t2_ACATGGTGAGGCGA-1 Patient2 Progenitors nonactive 5 0.07581954
Progenitors_5
10x_3250_t2_ACCAACGAGATAAG-1 Patient2 Progenitors active 12 0.14699448
Progenitors_12
10x_3250_t2_ACCAACGAGTTCAG-1 Patient2 Progenitors active 1 0.12865386
Progenitors_1
10x_3250_t2_ACCACCTGAGGTCT-1 Patient2 Progenitors nonactive 5 0.08986431
Progenitors_5
10x_3250_t2_ACCAGCCTCTTGTT-1 Patient2 Monocytes nonactive 13 0.09642699
Monocytes_13
10x_3250_t2_ACCATTACTACGCA-1 Patient2 Progenitors nonactive 5 0.06545241
Progenitors_5
10x_3250_t2_ACCCGTTGTACGCA-1 Patient2 Progenitors active 5 0.1290343
Progenitors_5
10x_3250_t2_ACGAACACTAGAGA-1 Patient2 Progenitors nonactive 5 0.10726967
Progenitors_5
10x_3250_t2_ACGAACTGGTAAGA-1 Patient2 Progenitors nonactive 5 0.09522224
Progenitors_5
10x_3250_t2_ACGACAACCTATGG-1 Patient2 Progenitors nonactive 5 0.09160801

Progenitors_5

10x_3250_t2_ACGACCCTAGTGCT-1 Patient2 Progenitors nonactive 5 0.09287617

Progenitors_5

10x_3250_t2_ACGGAGGAACCTAG-1 Patient2 Progenitors nonactive 5 0.06462811

Progenitors_5

10x_3250_t2_ACGGTCCTTGGTTG-1 Patient2 Progenitors nonactive 12 0.10958405

Progenitors_12

10x_3250_t2_ACGTCCTGGATAGA-1 Patient2 Progenitors nonactive 5 0.09287617

Progenitors_5

10x_3250_t2_ACGTCGCTTGAGAA-1 Patient2 Progenitors nonactive 5 0.07558176

Progenitors_5

10x_3250_t2_ACTACGGAGTTAGC-1 Patient2 Progenitors nonactive 5 0.08910342

Progenitors_5

10x_3250_t2_ACTACGGATACTTC-1 Patient2 Progenitors nonactive 6 0.11731976

Progenitors_6

10x_3250_t2_ACTACGGATTGTCT-1Patient2 Progenitors nonactive 5 0.11161309

Progenitors_5

10x_3250_t2_ACTAGGTGGTTTCT-1 Patient2 Progenitors active 12 0.16115021

Progenitors_12

10x_3250_t2_ACTATCACTGGAGG-1 Patient2 Progenitors active 5 0.1289709

Progenitors_5

10x_3250_t2_ACTCGCACGCGTAT-1 Patient2 Progenitors active 5 0.13176083

Progenitors_5

10x_3250_t2_ACTGCCTGCGCATA-1 Patient2 Progenitors active 5 0.16208547

Progenitors_5

10x_3250_t2_ACTTCTGAGCCATA-1 Patient2 Monocytes nonactive 0 0.03346332

Monocytes_0

10x_3250_t2_ACTTGTACTCTCTA-1Patient2 Progenitors nonactive 5 0.07139687

Progenitors_5

10x_3250_t2_AGAAAGTGCCGATA-1 Patient2 Progenitors active 5 0.1412561

Progenitors_5

10x_3250_t2_AGAAAGTGTGATGC-1 Patient2 Progenitors nonactive 5 0.09436624

Progenitors_5

10x_3250_t2_AGAACAGAACTCAG-1 Patient2 Progenitors nonactive 5 0.05234291 Progenitors_5
10x_3250_t2_AGAATGGAAGTCTG-1 Patient2 Progenitors nonactive 5 0.10866464 Progenitors_5
10x_3250_t2_AGAATGGATGTCTT-1 Patient2 Progenitors active 5 0.13656395 Progenitors_5
10x_3250_t2_AGAATTTGTGTCTT-1 Patient2 Progenitors nonactive 5 0.10015218 Progenitors_5
10x_3250_t2_AGAGTCACAACAGA-1 Patient2 Progenitors nonactive 5 0.09403335 Progenitors_5
10x_3250_t2_AGAGTCACAGTCAC-1 Patient2 Progenitors nonactive 6 0.09894744 Progenitors_6
10x_3250_t2_AGAGTCTGTTACTC-1 Patient2 Progenitors nonactive 5 0.05893729 Progenitors_5
10x_3250_t2_AGAGTGCTCGAATC-1 Patient2 Progenitors nonactive 5 0.08842179 Progenitors_5
10x_3250_t2_AGCAACACCAAAGA-1 Patient2 Progenitors nonactive 5 0.06887642 Progenitors_5
10x_3250_t2_AGCACAACGTGAGG-1 Patient2 Progenitors active 5 0.1188574 Progenitors_5
10x_3250_t2_AGCATTCTCTTGGA-1 Patient2 Progenitors active 5 0.16200621 Progenitors_5
10x_3250_t2_AGCGATTGGGTGTT-1 Patient2 Progenitors active 12 0.14534589 Progenitors_12
10x_3250_t2_AGCGCCGACCTCCA-1 Patient2 Progenitors nonactive 5 0.06943123 Progenitors_5
10x_3250_t2_AGCGGGCTGAGGCA-1 Patient2 Progenitors nonactive 5 0.09552343 Progenitors_5
10x_3250_t2_AGCGTAACGAGGTG-1 Patient2 Progenitors nonactive 5 0.08720119 Progenitors_5
10x_3250_t2_AGCTCGCTGGAGTG-1 Patient2 Progenitors nonactive 5 0.09417602 Progenitors_5
10x_3250_t2_AGCTGAACTGCATG-1 Patient2 Progenitors active 5 0.1235337

Progenitors_5
10x_3250_t2_AGCTGTGATTCGGA-1 Patient2 Progenitors nonactive 5 0.07946547 Progenitors_5
10x_3250_t2_AGGAACCTGCTATG-1 Patient2 Progenitors nonactive 12 0.10628686 Progenitors_12
10x_3250_t2_AGGAGTCTGTGTTG-1 Patient2 Progenitors nonactive 5 0.08038488 Progenitors_5
10x_3250_t2_AGGATGCTTGTAGC-1 Patient2 Progenitors nonactive 5 0.08024222 Progenitors_5
10x_3250_t2_AGGCAACTCCTTGC-1 Patient2 Progenitors nonactive 5 0.09842432 Progenitors_5
10x_3250_t2_AGGCAACTTTCCCG-1 Patient2 Monocytes active 13 0.11882569 Monocytes_13
10x_3250_t2_AGGCAGGAGCCAAT-1 Patient2 Progenitors nonactive 5 0.05460973 Progenitors_5
10x_3250_t2_AGGGCCTGCAGATC-1 Patient2 Progenitors active 5 0.12347029 Progenitors_5
10x_3250_t2_AGGGTGGAGTTGTG-1 Patient2 Progenitors nonactive 5 0.07372709 Progenitors_5
10x_3250_t2_AGGTACACTAAGGA-1 Patient2 Progenitors nonactive 5 0.08436371 Progenitors_5
10x_3250_t2_AGGTGGGAATGCCA-1 Patient2 Progenitors nonactive 5 0.08255659 Progenitors_5
10x_3250_t2_AGGTGTTGTGCTAG-1 Patient2 Progenitors nonactive 5 0.07596221 Progenitors_5
10x_3250_t2_AGTACGTGGTTCAG-1 Patient2 Progenitors nonactive 5 0.08074948 Progenitors_5
10x_3250_t2_AGTAGAGAGATACC-1 Patient2 Progenitors active 5 0.1348678 Progenitors_5
10x_3250_t2_AGTGACACTCCTTA-1 Patient2 Progenitors nonactive 5 0.05546573 Progenitors_5
10x_3250_t2_AGTGACTGGTTCAG-1 Patient2 Progenitors nonactive 12 0.11078879 Progenitors_12

10x_3250_t2_AGTGTTCTTGCTTT-1 Patient2 Progenitors nonactive 5 0.1003107 Progenitors_5
10x_3250_t2_AGTTATGAGTCACA-1 Patient2 Progenitors nonactive 5 0.10986938 Progenitors_5
10x_3250_t2_AGTTTAGATTCTCA-1 Patient2 Progenitors active 12 0.14783463 Progenitors_12
10x_3250_t2_ATAACATGCTCTTA-1 Patient2 Progenitors nonactive 12 0.1007387 Progenitors_12
10x_3250_t2_ATACAATGCAGTCA-1 Patient2 Progenitors nonactive 5 0.09910595 Progenitors_5
10x_3250_t2_ATACAATGGACGAG-1 Patient2 Progenitors nonactive 5 0.10551011 Progenitors_5
10x_3250_t2_ATACCTACCTTCGC-1 Patient2 Progenitors nonactive 5 0.1100279 Progenitors_5
10x_3250_t2_ATAGAACTCTGAGT-1 Patient2 Progenitors nonactive 5 0.0914812 Progenitors_5
10x_3250_t2_ATAGAACTGGGCAA-1 Patient2 Progenitors active 12 0.14991123 Progenitors_12
10x_3250_t2_ATAGCCGACGAACT-1 Patient2 Progenitors active 5 0.12532496 Progenitors_5
10x_3250_t2_ATAGGCTGCAAGCT-1 Patient2 Progenitors active 12 0.15357301 Progenitors_12
10x_3250_t2_ATAGTTGACGCTAA-1 Patient2 Progenitors nonactive 5 0.09507958 Progenitors_5
10x_3250_t2_ATATGAACTGCACA-1 Patient2 Progenitors active 1 0.12462748 Progenitors_1
10x_3250_t2_ATATGCCTGGATCT-1 Patient2 Progenitors active 12 0.14044766 Progenitors_12
10x_3250_t2_ATCAAATGTCTATC-1 Patient2 Progenitors nonactive 5 0.06451715 Progenitors_5
10x_3250_t2_ATCAACCTTTACCT-1 Patient2 Progenitors nonactive 5 0.07623169 Progenitors_5
10x_3250_t2_ATCACACTCTGATG-1 Patient2 Progenitors nonactive 5 0.07865703

Progenitors_5

10x_3250_t2_ATCACGGATTGGCA-1 Patient2 Progenitors active 6 0.12984275

Progenitors_6

10x_3250_t2_ATCACTACTTTCGT-1 Patient2 Progenitors active 6 0.15985036

Progenitors_6

10x_3250_t2_ATCAGGTGACACCA-1 Patient2 Progenitors nonactive 5 0.08271511

Progenitors_5

10x_3250_t2_ATCATGCTAGCTAC-1 Patient2 Monocytes nonactive 0 0.04855431

Monocytes_0

10x_3250_t2_ATCGAGTGACGTTG-1 Patient2 Progenitors nonactive 6 0.09125927

Progenitors_6

10x_3250_t2_ATCTACACCAAGCT-1 Patient2 Progenitors nonactive 6 0.11164479

Progenitors_6

10x_3250_t2_ATCTTGACGACGGA-1 Patient2 Progenitors nonactive 5 0.07686577

Progenitors_5

10x_3250_t2_ATGAAGGAAGGAGC-1 Patient2 Progenitors nonactive 5 0.1109156

Progenitors_5

10x_3250_t2_ATGATATGAAGCCT-1 Patient2 Progenitors nonactive 5 0.07226872

Progenitors_5

10x_3250_t2_ATGATATGCTGTAG-1 Patient2 Progenitors active 5 0.15308161

Progenitors_5

10x_3250_t2_ATGCCAGAGTCGAT-1 Patient2 Progenitors nonactive 5 0.08753408

Progenitors_5

10x_3250_t2_ATGCCAGATGCCTC-1 Patient2 Progenitors nonactive 5 0.11670154

Progenitors_5

10x_3250_t2_ATGCGATGACAGCT-1 Patient2 Progenitors active 5 0.13168157

Progenitors_5

10x_3250_t2_ATTAACGAGATACC-1 Patient2 Progenitors nonactive 5 0.11063027

Progenitors_5

10x_3250_t2_ATTAGTGACAACTG-1 Patient2 Progenitors nonactive 5 0.07778518

Progenitors_5

10x_3250_t2_ATTCAAGAAGCCTA-1 Patient2 Progenitors nonactive 5 0.10438463

Progenitors_5

10x_3250_t2_ATTCCAACGTGCTA-1 Patient2 Progenitors active 5 0.12174244 Progenitors_5
10x_3250_t2_ATTCTTCTTCTAGG-1 Patient2 Progenitors nonactive 5 0.07469406 Progenitors_5
10x_3250_t2_ATTGAAACGCCCTT-1 Patient2 Progenitors active 12 0.13577135 Progenitors_12
10x_3250_t2_ATTGCTTGCTTGAG-1 Patient2 Progenitors active 12 0.16829941 Progenitors_12
10x_3250_t2_ATTGGTCTGTTCAG-1 Patient2 Progenitors nonactive 5 0.08853275 Progenitors_5
10x_3250_t2_ATTTCGTGTTGACG-1 Patient2 Progenitors nonactive 5 0.09715617 Progenitors_5
10x_3250_t2_CAAACTCTGGTCAT-1 Patient2 Progenitors active 5 0.15561791 Progenitors_5
10x_3250_t2_CAAAGCACAGCGTT-1 Patient2 Progenitors active 5 0.13919536 Progenitors_5
10x_3250_t2_CAACGAACGAGCAG-1 Patient2 Progenitors active 5 0.17774713 Progenitors_5
10x_3250_t2_CAACTTTGGAGCTT-1 Patient2 Progenitors nonactive 5 0.07364783 Progenitors_5
10x_3250_t2_CAAGCATGCCACAA-1 Patient2 Progenitors active 5 0.12757593 Progenitors_5
10x_3250_t2_CAAGCTGAAGCATC-1 Patient2 Progenitors active 5 0.1589151 Progenitors_5
10x_3250_t2_CAAGGACTACTGTG-1 Patient2 Progenitors nonactive 5 0.09385898 Progenitors_5
10x_3250_t2_CAAGTTCTACCTCC-1 Patient2 Monocytes active 13 0.1399721 Monocytes_13
10x_3250_t2_CACAACGATTCCGC-1 Patient2 Progenitors nonactive 5 0.11277027 Progenitors_5
10x_3250_t2_CACACCTGTAACCG-1 Patient2 Progenitors nonactive 5 0.07778518 Progenitors_5
10x_3250_t2_CACCCATGGAGCAG-1 Patient2 Progenitors nonactive 5 0.06397819

Progenitors_5
10x_3250_t2_CACCCATGGTTGAC-1 Patient2 Progenitors active 6 0.12721134
Progenitors_6
10x_3250_t2_CACCCATGTGCCCT-1 Patient2 Progenitors nonactive 5 0.10400418
Progenitors_5
10x_3250_t2_CACCGGGAAGCTCA-1 Patient2 Progenitors nonactive 5 0.06275759
Progenitors_5
10x_3250_t2_CACCGTACGGTTCA-1 Patient2 Progenitors nonactive 5 0.08666223
Progenitors_5
10x_3250_t2_CACGACCTCTGCTC-1 Patient2 Monocytes nonactive 13 0.09141779
Monocytes_13
10x_3250_t2_CACGACCTTGCTTT-1 Patient2 Progenitors nonactive 5 0.10761841
Progenitors_5
10x_3250_t2_CACGACCTTTCCAT-1Patient2 Progenitors nonactive 5 0.1078879
Progenitors_5
10x_3250_t2_CACTATACAGGTTC-1 Patient2 Progenitors nonactive 5 0.1121679
Progenitors_5
10x_3250_t2_CACTGAGAAACGAA-1 Patient2 Progenitors nonactive 5 0.09352609
Progenitors_5
10x_3250_t2_CACTTAACCTACGA-1 Patient2 Progenitors nonactive 5 0.09417602
Progenitors_5
10x_3250_t2_CACTTAACGGAGGT-1 Patient2 Progenitors nonactive 5 0.07691332
Progenitors_5
10x_3250_t2_CACTTAACTGCCAA-1 Patient2 Progenitors nonactive 5 0.10035825
Progenitors_5
10x_3250_t2_CACTTATGGTGAGG-1 Patient2 Progenitors active 5 0.15504724
Progenitors_5
10x_3250_t2_CAGAAGCTCTGAGT-1 Patient2 Progenitors nonactive 5 0.04419504
Progenitors_5
10x_3250_t2_CAGAGGGAAGCACT-1 Patient2 Progenitors nonactive 5 0.0818274
Progenitors_5
10x_3250_t2_CAGAGGGAGCAGAG-1 Patient2 Progenitors active 12 0.12087059
Progenitors_12

10x_3250_t2_CAGATCGAGTAGCT-1 Patient2 Progenitors active 12 0.15945406 Progenitors_12
10x_3250_t2_CAGCACCTTAACCG-1 Patient2 Progenitors nonactive 5 0.04451208 Progenitors_5
10x_3250_t2_CAGTTTACTCAGGT-1 Patient2 Progenitors nonactive 5 0.10362374 Progenitors_5
10x_3250_t2_CATAAATGACAGCT-1 Patient2 Progenitors nonactive 5 0.08737556 Progenitors_5
10x_3250_t2_CATACTTGCGTTGA-1 Patient2 Progenitors nonactive 5 0.04264156 Progenitors_5
10x_3250_t2_CATATAGACCTACC-1 Patient2 Progenitors active 5 0.12819415 Progenitors_5
10x_3250_t2_CATCTCCTCCCAAA-1 Patient2 Progenitors nonactive 5 0.11633695 Progenitors_5
10x_3250_t2_CATCTTGAGCGATT-1 Patient2 Progenitors nonactive 5 0.06600723 Progenitors_5
10x_3250_t2_CATGAGACGATGAA-1 Patient2 Progenitors nonactive 5 0.0472069 Progenitors_5
10x_3250_t2_CATGCCACGCATAC-1 Patient2 Progenitors active 5 0.14553611 Progenitors_5
10x_3250_t2_CATGTTACTCGCCT-1 Patient2 Progenitors nonactive 5 0.06610234 Progenitors_5
10x_3250_t2_CATTACACGCTATG-1 Patient2 Progenitors nonactive 5 0.07930696 Progenitors_5
10x_3250_t2_CATTGTACACGTTG-1 Patient2 Progenitors active 5 0.12015725 Progenitors_5
10x_3250_t2_CCAAGAACAACGTC-1 Patient2 Progenitors active 5 0.19913132 Progenitors_5
10x_3250_t2_CCACTGACGAATAG-1 Patient2 Progenitors active 12 0.14953078 Progenitors_12
10x_3250_t2_CCACTGACGGCGAA-1 Patient2 Progenitors nonactive 5 0.05323061 Progenitors_5
10x_3250_t2_CCACTTCTAGGGTG-1 Patient2 Progenitors nonactive 5 0.10731723

Progenitors_5
10x_3250_t2_CCAGCTACGCAGAG-1 Patient2 Progenitors active 12 0.12416778 Progenitors_12
10x_3250_t2_CCAGTCACGGATTC-1 Patient2 Progenitors nonactive 5 0.11621013 Progenitors_5
10x_3250_t2_CCATAGGAATGGTC-1 Patient2 Progenitors nonactive 5 0.08691586 Progenitors_5
10x_3250_t2_CCATCGTGATCGAC-1 Patient2 Progenitors nonactive 5 0.0699702 Progenitors_5
10x_3250_t2_CCCAACTGCACAAC-1 Patient2 B cells nonactive 7 0.08357111 B cells_7
10x_3250_t2_CCCAGTTGGGGTGA-1 Patient2 Progenitors nonactive 5 0.10089722 Progenitors_5
10x_3250_t2_CCCGATTGACCACA-1 Patient2 Progenitors nonactive 5 0.09702936 Progenitors_5
10x_3250_t2_CCCGATTGCGGTAT-1 Patient2 Progenitors nonactive 5 0.11139116 Progenitors_5
10x_3250_t2_CCCGATTGTACGAC-1 Patient2 Progenitors active 12 0.13217298 Progenitors_12
10x_3250_t2_CCCTTACTGTAAGA-1 Patient2 Progenitors active 12 0.16614355 Progenitors_12
10x_3250_t2_CCGGTACTGGTCTA-1 Patient2 Progenitors nonactive 5 0.08466489 Progenitors_5
10x_3250_t2_CCGTAAGACCCAAA-1 Patient2 Progenitors nonactive 5 0.0387737 Progenitors_5
10x_3250_t2_CCTGAGCTGTTTCT-1Patient2 Progenitors nonactive 5 0.07743643 Progenitors_5
10x_3250_t2_CCTTCACTAGGTTC-1 Patient2 Progenitors nonactive 5 0.11070953 Progenitors_5
10x_3250_t2_CCTTTAGATCGTTT-1 Patient2 Progenitors active 5 0.12204362 Progenitors_5
10x_3250_t2_CGAAGTACACCGAT-1 Patient2 Progenitors nonactive 5 0.09481009 Progenitors_5

10x_3250_t2_CGACTCACCGTGAT-1 Patient2 Progenitors nonactive 5 0.07323569 Progenitors_5
10x_3250_t2_CGAGCCGAAATGCC-1 Patient2 Progenitors nonactive 5 0.05147105 Progenitors_5
10x_3250_t2_CGAGCCGAGTCAAC-1 Patient2 Progenitors active 5 0.12879653 Progenitors_5
10x_3250_t2_CGAGTATGCGATAC-1 Patient2 Progenitors nonactive 5 0.11546509 Progenitors_5
10x_3250_t2_CGATCAGAATGACC-1 Patient2 Progenitors active 12 0.13656395 Progenitors_12
10x_3250_t2_CGCAACCTTGCTTT-1 Patient2 Progenitors active 5 0.13600913 Progenitors_5
10x_3250_t2_CGCACTTGTCCCGT-1 Patient2 Progenitors nonactive 5 0.07405998 Progenitors_5
10x_3250_t2_CGCATAGACTTGGA-1 Patient2 Progenitors nonactive 5 0.08236637 Progenitors_5
10x_3250_t2_CGCGGATGCCCACT-1 Patient2 Progenitors active 12 0.14558367 Progenitors_12
10x_3250_t2_CGCTACACGGATCT-1 Patient2 Progenitors nonactive 5 0.05871536 Progenitors_5
10x_3250_t2_CGCTACTGCTTGGA-1 Patient2 Progenitors nonactive 12 0.11483102 Progenitors_12
10x_3250_t2_CGCTACTGGAGCAG-1 Patient2 Progenitors nonactive 5 0.08209689 Progenitors_5
10x_3250_t2_CGGAATTGGCATAC-1 Patient2 Progenitors nonactive 5 0.0657536 Progenitors_5
10x_3250_t2_CGGATAACTTTGTC-1 Patient2 Progenitors active 12 0.14954664 Progenitors_12
10x_3250_t2_CGGCGAACCTGATG-1 Patient2 Progenitors nonactive 5 0.08044829 Progenitors_5
10x_3250_t2_CGGGCATGCTGTAG-1 Patient2 Monocytes nonactive 13 0.08181155 Monocytes_13
10x_3250_t2_CGGTACCTTGAACC-1 Patient2 Progenitors nonactive 5 0.09171898

Progenitors_5
10x_3250_t2_CGTACCTGGGCATT-1 Patient2 Progenitors nonactive 5 0.08341259
Progenitors_5
10x_3250_t2_CGTACCTGGTTGGT-1 Patient2 Progenitors nonactive 5 0.10124596
Progenitors_5
10x_3250_t2_CGTCCAACTAGTCG-1 Patient2 Progenitors active 6 0.13773699
Progenitors_6
10x_3250_t2_CGTCGACTCCTCAC-1 Patient2 Progenitors nonactive 5 0.07478917
Progenitors_5
10x_3250_t2_CGTCGACTTCACGA-1 Patient2 Progenitors nonactive 5 0.11303976
Progenitors_5
10x_3250_t2_CGTGAAACGTGCTA-1 Patient2 Progenitors nonactive 5 0.08238222
Progenitors_5
10x_3250_t2_CGTGAATGCCCTTG-1 Patient2 Progenitors nonactive 5 0.08758164
Progenitors_5
10x_3250_t2_CGTGCACTTAACCG-1 Patient2 Progenitors nonactive 5 0.08292118
Progenitors_5
10x_3250_t2_CTAAGGTGAAGCAA-1 Patient2 Progenitors nonactive 5 0.06049077
Progenitors_5
10x_3250_t2_CTAGATCTTCTCGC-1 Patient2 Progenitors nonactive 5 0.09937544
Progenitors_5
10x_3250_t2_CTAGTTACTCCTAT-1 Patient2 Progenitors nonactive 5 0.1138482
Progenitors_5
10x_3250_t2_CTAGTTTGAGTACC-1 Patient2 Progenitors active 5 0.12178999
Progenitors_5
10x_3250_t2_CTATACTGGCGATT-1 Patient2 Progenitors active 5 0.12914527
Progenitors_5
10x_3250_t2_CTATACTGTAGCCA-1 Patient2 Progenitors nonactive 5 0.0510272
Progenitors_5
10x_3250_t2_CTATACTGTCTCCG-1 Patient2 T cells nonactive 9 0.07808636 T cells_9
10x_3250_t2_CTATGTACGAAGGC-1 Patient2 Progenitors nonactive 12 0.11110583
Progenitors_12
10x_3250_t2_CTATGTTGACCTAG-1 Patient2 Progenitors active 5 0.15985036

Progenitors_5

10x_3250_t2_CTCGACTGGCCATA-1 Patient2 Progenitors nonactive 12 0.10964745

Progenitors_12

10x_3250_t2_CTCGAGCTTTCACT-1 Patient2 Progenitors active 5 0.12098155

Progenitors_5

10x_3250_t2_CTGAACGAGTGTTG-1 Patient2 Progenitors nonactive 5 0.08212859

Progenitors_5

10x_3250_t2_CTGAACGATCCTGC-1 Patient2 Progenitors active 5 0.11898421

Progenitors_5

10x_3250_t2_CTGAAGACTGGTTG-1 Patient2 Progenitors active 12 0.14605922

Progenitors_12

10x_3250_t2_CTGAAGTGCTGTAG-1 Patient2 Progenitors active 5 0.14223892

Progenitors_5

10x_3250_t2_CTGACCACCCCTAC-1 Patient2 Progenitors active 5 0.14436307

Progenitors_5

10x_3250_t2_CTGATGGAGCGAGA-1 Patient2 Progenitors nonactive 5 0.06603893

Progenitors_5

10x_3250_t2_CTGATTTGACTGTG-1 Patient2 Progenitors nonactive 5 0.06464397

Progenitors_5

10x_3250_t2_CTGATTTGGTGTTG-1 Patient2 Progenitors nonactive 5 0.09604654

Progenitors_5

10x_3250_t2_CTGATTTGTCTGGA-1 Patient2 Progenitors nonactive 5 0.06313804

Progenitors_5

10x_3250_t2_CTGCCAACAGCACT-1 Patient2 Progenitors nonactive 5 0.09528565

Progenitors_5

10x_3250_t2_CTGGATGATGCTCC-1 Patient2 Progenitors nonactive 5 0.09018135

Progenitors_5

10x_3250_t2_CTGTATACGGTACT-1 Patient2 Progenitors active 5 0.13564454

Progenitors_5

10x_3250_t2_CTTACAACTACTTC-1Patient2 Progenitors nonactive 5 0.08935705

Progenitors_5

10x_3250_t2_CTTAGACTGAGGTG-1 Patient2 Progenitors nonactive 5 0.04235622

Progenitors_5

10x_3250_t2_CTTGAGGAACGACT-1 Patient2 Progenitors active 12 0.13302898 Progenitors_12
10x_3250_t2_CTTTCAGACTGTAG-1 Patient2 Progenitors active 5 0.1534462 Progenitors_5
10x_3250_t2_GAAACAGAGTTTCT-1 Patient2 Monocytes nonactive 13 0.09718788 Monocytes_13
10x_3250_t2_GAAAGTGATACTCT-1 Patient2 Progenitors active 6 0.14371314 Progenitors_6
10x_3250_t2_GAAAGTGATGCGTA-1 Patient2 Progenitors active 12 0.13753091 Progenitors_12
10x_3250_t2_GAAATACTTACAGC-1 Patient2 Progenitors nonactive 5 0.09049838 Progenitors_5
10x_3250_t2_GAACAGCTCGAGAG-1 Patient2 Progenitors active 12 0.17359394 Progenitors_12
10x_3250_t2_GAACGTTGACTGTG-1 Patient2 Progenitors nonactive 5 0.07218946 Progenitors_5
10x_3250_t2_GAAGATGACCCAAA-1 Patient2 Progenitors nonactive 5 0.09891573 Progenitors_5
10x_3250_t2_GAAGATGAGTTACG-1 Patient2 Progenitors nonactive 5 0.05529136 Progenitors_5
10x_3250_t2_GAAGCGGAATGTGC-1 Patient2 Progenitors active 6 0.12632363 Progenitors_6
10x_3250_t2_GAAGTGCTGAGGGT-1 Patient2 Progenitors nonactive 5 0.08203348 Progenitors_5
10x_3250_t2_GAATTAACAACCTG-1 Patient2 Progenitors active 12 0.14921375 Progenitors_12
10x_3250_t2_GACAGTTGCACTGA-1 Patient2 Progenitors nonactive 5 0.0784351 Progenitors_5
10x_3250_t2_GACCAAACCTTGGA-1 Patient2 Progenitors nonactive 5 0.11207279 Progenitors_5
10x_3250_t2_GACCATGACGCCTT-1 Patient2 Progenitors active 5 0.13480439 Progenitors_5
10x_3250_t2_GACGAACTCAAGCT-1 Patient2 Progenitors nonactive 5 0.10086551

Progenitors_5
10x_3250_t2_GACGATTGGTCTGA-1 Patient2 Progenitors nonactive 5 0.07249065 Progenitors_5
10x_3250_t2_GACTGATGCTCTCG-1 Patient2 Progenitors nonactive 5 0.0691776 Progenitors_5
10x_3250_t2_GACTTTACTGCCTC-1 Patient2 Progenitors active 12 0.16555704 Progenitors_12
10x_3250_t2_GAGATCACAAGAAC-1 Patient2 Progenitors nonactive 5 0.08697927 Progenitors_5
10x_3250_t2_GAGCAACTCCTCGT-1 Patient2 T cells active 9 0.13442394 T cells_9
10x_3250_t2_GAGGACGAAGAATG-1 Patient2 Progenitors nonactive 5 0.09779025 Progenitors_5
10x_3250_t2_GAGGCCACTTGCTT-1 Patient2 Progenitors nonactive 12 0.10611248 Progenitors_12
10x_3250_t2_GAGGGAACAACGGG-1 Patient2 Progenitors nonactive 5 0.09073616 Progenitors_5
10x_3250_t2_GAGGGCCTACACGT-1 Patient2 Progenitors nonactive 5 0.08970579 Progenitors_5
10x_3250_t2_GAGGTTTGTGCTTT-1 Patient2 Progenitors nonactive 5 0.11134361 Progenitors_5
10x_3250_t2_GAGTCTGACATTTC-1 Patient2 Progenitors nonactive 5 0.09270179 Progenitors_5
10x_3250_t2_GATACTCTCGAGAG-1 Patient2 Progenitors nonactive 5 0.09501617 Progenitors_5
10x_3250_t2_GATATAACGACAAA-1 Patient2 Progenitors nonactive 5 0.09850358 Progenitors_5
10x_3250_t2_GATATTGAACCATG-1 Patient2 Progenitors nonactive 5 0.10658804 Progenitors_5
10x_3250_t2_GATATTGATCGACA-1 Patient2 Progenitors active 12 0.16378162 Progenitors_12
10x_3250_t2_GATCGAACGTTGGT-1 Patient2 Progenitors active 12 0.14065373 Progenitors_12
10x_3250_t2_GATTCTACAATCGC-1 Patient2 Monocytes nonactive 13 0.09167142

Monocytes_13
10x_3250_t2_GCAAGACTGTCACA-1 Patient2 Progenitors nonactive 5 0.07512206 Progenitors_5
10x_3250_t2_GCAATTCTACGGTT-1Patient2 Progenitors active 6 0.13169742 Progenitors_6
10x_3250_t2_GCACAAACTAACCG-1 Patient2 Progenitors nonactive 5 0.08910342 Progenitors_5
10x_3250_t2_GCACACCTTCTCGC-1 Patient2 Progenitors active 12 0.12522985 Progenitors_12
10x_3250_t2_GCACCTACACAGCT-1 Patient2 Progenitors nonactive 5 0.08030562 Progenitors_5
10x_3250_t2_GCACGGTGGAATAG-1 Patient2 Progenitors nonactive 5 0.07610488 Progenitors_5
10x_3250_t2_GCACGTCTTCTACT-1Patient2 Progenitors active 12 0.15140131 Progenitors_12
10x_3250_t2_GCAGATACGTTTCT-1Patient2 Progenitors nonactive 5 0.06377211 Progenitors_5
10x_3250_t2_GCAGCCGAAACCGT-1 Patient2 Progenitors active 5 0.16517659 Progenitors_5
10x_3250_t2_GCAGTCCTTTGGTG-1 Patient2 Progenitors active 5 0.16879082 Progenitors_5
10x_3250_t2_GCAGTTGACGTTGA-1 Patient2 Progenitors nonactive 5 0.10500285 Progenitors_5
10x_3250_t2_GCCTACACGTAGGG-1 Patient2 Progenitors active 12 0.1526219 Progenitors_12
10x_3250_t2_GCCTCAACAAACAG-1 Patient2 Monocytes nonactive 3 0.04124659 Monocytes_3
10x_3250_t2_GCCTCAACAAGGGC-1 Patient2 Progenitors nonactive 5 0.0745831 Progenitors_5
10x_3250_t2_GCGAAGGACACTCC-1 Patient2 Progenitors nonactive 6 0.09463572 Progenitors_6
10x_3250_t2_GCGAAGGACGAATC-1 Patient2 Progenitors active 5 0.13508972 Progenitors_5

10x_3250_t2_GCGACTCTCCAACA-1 Patient2 Progenitors active 12 0.15821761 Progenitors_12
10x_3250_t2_GCGAGAGATCCCAC-1 Patient2 Monocytes nonactive 3 0.04116733 Monocytes_3
10x_3250_t2_GCGCATCTAGCACT-1 Patient2 Progenitors active 5 0.13583476 Progenitors_5
10x_3250_t2_GCGGAGCTCCAACA-1 Patient2 Progenitors nonactive 12 0.11335679 Progenitors_12
10x_3250_t2_GCGGCAACGGGTGA-1 Patient2 Progenitors nonactive 12 0.11556021 Progenitors_12
10x_3250_t2_GCGGCAACTTCTCA-1 Patient2 Progenitors nonactive 5 0.06340752 Progenitors_5
10x_3250_t2_GCTACAGACCTATT-1Patient2 Progenitors nonactive 5 0.07380635 Progenitors_5
10x_3250_t2_GCTTGAGAGTAAAG-1 Patient2 Progenitors nonactive 5 0.05467313 Progenitors_5
10x_3250_t2_GGAACTACTCACCC-1 Patient2 B cells nonactive 7 0.06935198 B cells_7
10x_3250_t2_GGAAGGTGCGTGAT-1 Patient2 Progenitors active 12 0.18196373 Progenitors_12
10x_3250_t2_GGACAGGAAGCACT-1 Patient2 Progenitors nonactive 1 0.09507958 Progenitors_1
10x_3250_t2_GGACAGGATCCAAG-1 Patient2 Progenitors nonactive 5 0.09046668 Progenitors_5
10x_3250_t2_GGACCCGAACCCTC-1 Patient2 Progenitors active 5 0.13050853 Progenitors_5
10x_3250_t2_GGACGCTGGACGGA-1 Patient2 Progenitors active 12 0.14916619 Progenitors_12
10x_3250_t2_GGAGACGACCACAA-1 Patient2 Progenitors active 1 0.124358 Progenitors_1
10x_3250_t2_GGAGAGACTTGCAG-1 Patient2 Progenitors active 6 0.12865386 Progenitors_6
10x_3250_t2_GGAGCCACCCACAA-1 Patient2 Progenitors active 1 0.1442204

Progenitors_1
10x_3250_t2_GGAGCGCTGGTATC-1 Patient2 Progenitors active 12 0.17857143
Progenitors_12
10x_3250_t2_GGAGGATGTCCTAT-1 Patient2 Progenitors active 5 0.14992708
Progenitors_5
10x_3250_t2_GGATAGCTAGCGGA-1 Patient2 Progenitors nonactive 5 0.07963985
Progenitors_5
10x_3250_t2_GGCAATACAGAACA-1 Patient2 Progenitors active 5 0.14006721
Progenitors_5
10x_3250_t2_GGCCCAGATTCGTT-1 Patient2 Progenitors nonactive 5 0.0657536
Progenitors_5
10x_3250_t2_GGCCGAACTGCATG-1 Patient2 Progenitors active 6 0.13845032
Progenitors_6
10x_3250_t2_GGCCGATGTCTAGG-1 Patient2 Progenitors nonactive 5 0.10446389
Progenitors_5
10x_3250_t2_GGCTAATGCTGAGT-1 Patient2 Progenitors active 12 0.13515313
Progenitors_12
10x_3250_t2_GGGAACGAGCATCA-1 Patient2 Progenitors nonactive 5 0.08333333
Progenitors_5
10x_3250_t2_GGGATGGAAAGAAC-1 Patient2 Progenitors active 12 0.16151481
Progenitors_12
10x_3250_t2_GGGATGGAAAGCCT-1 Patient2 Progenitors active 1 0.16806163
Progenitors_1
10x_3250_t2_GGGATGGATGGTGT-1 Patient2 Progenitors nonactive 5 0.06323315
Progenitors_5
10x_3250_t2_GGTACAACAGAATG-1 Patient2 Progenitors active 12 0.1311426
Progenitors_12
10x_3250_t2_GGTGATACAACCAC-1 Patient2 Progenitors nonactive 5 0.05383298
Progenitors_5
10x_3250_t2_GGTTGAACAAGTAG-1 Patient2 Monocytes nonactive 13 0.09124342
Monocytes_13
10x_3250_t2_GTAACGTGAAGAGT-1 Patient2 Progenitors nonactive 5 0.05527551
Progenitors_5

10x_3250_t2_GTAAGCACAGGGTG-1 Patient2 Progenitors active 12 0.15165494 Progenitors_12
10x_3250_t2_GTAATAACTTTCTG-1 Patient2 T cells nonactive 9 0.09591973 T cells_9
10x_3250_t2_GTAATATGAGCTCA-1 Patient2 Progenitors nonactive 5 0.06928857 Progenitors_5
10x_3250_t2_GTACCCTGGGAAGC-1 Patient2 Progenitors nonactive 5 0.10929871 Progenitors_5
10x_3250_t2_GTACGTGACAGTCA-1 Patient2 Progenitors active 12 0.12037918 Progenitors_12
10x_3250_t2_GTACGTGATCGCTC-1 Patient2 Progenitors active 5 0.11831843 Progenitors_5
10x_3250_t2_GTAGCCCTGTCACA-1 Patient2 Progenitors active 5 0.12153636 Progenitors_5
10x_3250_t2_GTATCTACTGAAGA-1 Patient2 Progenitors nonactive 5 0.09474669 Progenitors_5
10x_3250_t2_GTATTCACACTCTT-1 Patient2 Progenitors nonactive 5 0.08060681 Progenitors_5
10x_3250_t2_GTCACAGAAGAGTA-1 Patient2 Progenitors nonactive 5 0.09208357 Progenitors_5
10x_3250_t2_GTCACAGATTTCTG-1 Patient2 Monocytes nonactive 0 0.08360282 Monocytes_0
10x_3250_t2_GTCGAATGCGAGTT-1 Patient2 Progenitors active 12 0.13229979 Progenitors_12
10x_3250_t2_GTCTAGGAGACAGG-1 Patient2 Progenitors active 5 0.16420963 Progenitors_5
10x_3250_t2_GTCTAGGAGCATCA-1 Patient2 B cells nonactive 7 0.11037664 B cells_7
10x_3250_t2_GTCTGAGAAATGCC-1 Patient2 Progenitors active 1 0.13112675 Progenitors_1
10x_3250_t2_GTGACAACTGCATG-1 Patient2 Progenitors nonactive 5 0.07562932 Progenitors_5
10x_3250_t2_GTGACCCTTGGTCA-1 Patient2 Progenitors nonactive 5 0.06320145 Progenitors_5

10x_3250_t2_GTGACCCTTGTGCA-1 Patient2 Progenitors nonactive 5 0.08900831 Progenitors_5
10x_3250_t2_GTGATTCTCACTCC-1 Patient2 Progenitors nonactive 6 0.10302137 Progenitors_6
10x_3250_t2_GTGATTCTTTCACT-1 Patient2 Progenitors nonactive 5 0.07878384 Progenitors_5
10x_3250_t2_GTTGACGACTCAGA-1 Patient2 Progenitors active 12 0.13913195 Progenitors_12
10x_3250_t2_TAAATGTGTCAAGC-1 Patient2 Progenitors active 12 0.16424133 Progenitors_12
10x_3250_t2_TAACAATGGTTCAG-1 Patient2 Progenitors active 12 0.16782385 Progenitors_12
10x_3250_t2_TAACAATGTGCTAG-1 Patient2 Progenitors nonactive 5 0.10243485 Progenitors_5
10x_3250_t2_TAACACCTCAAGCT-1 Patient2 Progenitors active 5 0.12733815 Progenitors_5
10x_3250_t2_TAAGAGGAATTTCC-1 Patient2 Progenitors nonactive 5 0.06423182 Progenitors_5
10x_3250_t2_TAAGCGTGTGAACC-1 Patient2 Progenitors nonactive 5 0.08789867 Progenitors_5
10x_3250_t2_TAAGGCTGACCCTC-1 Patient2 Progenitors nonactive 5 0.07989348 Progenitors_5
10x_3250_t2_TAAGGCTGCGACAT-1 Patient2 Monocytes nonactive 13 0.07312472 Monocytes_13
10x_3250_t2_TAATGATGTTTCAC-1 Patient2 Progenitors nonactive 5 0.10188003 Progenitors_5
10x_3250_t2_TAATGCCTGAGATA-1 Patient2 Progenitors active 5 0.13011223 Progenitors_5
10x_3250_t2_TAATGTGATAGTCG-1 Patient2 Progenitors nonactive 5 0.03474732 Progenitors_5
10x_3250_t2_TACACACTATTTCC-1 Patient2 Progenitors nonactive 5 0.08873882 Progenitors_5
10x_3250_t2_TACCGCTGCAAAGA-1 Patient2 Progenitors active 5 0.12581637

Progenitors_5

10x_3250_t2_TACGACGAGCATCA-1 Patient2 Progenitors active 5 0.14447403 Progenitors_5

10x_3250_t2_TACGAGACCGAGAG-1 Patient2 Progenitors nonactive 5 0.06141018 Progenitors_5

10x_3250_t2_TACGCCACCATGCA-1 Patient2 Progenitors active 5 0.15989791 Progenitors_5

10x_3250_t2_TACGGCCTAGCGTT-1 Patient2 Progenitors active 12 0.14578974 Progenitors_12

10x_3250_t2_TACGTTACGAATAG-1 Patient2 Progenitors nonactive 5 0.10985353 Progenitors_5

10x_3250_t2_TACTAAGACCACCT-1 Patient2 B cells nonactive 7 0.07119079 B cells_7

10x_3250_t2_TACTACACACCAGT-1 Patient2 Monocytes active 8 0.12751252 Monocytes_8

10x_3250_t2_TACTACACGCGAAG-1 Patient2 Progenitors nonactive 5 0.08529897 Progenitors_5

10x_3250_t2_TACTCCCTTCGTTT-1 Patient2 Progenitors nonactive 5 0.10346522 Progenitors_5

10x_3250_t2_TACTGGGATGTTCT-1 Patient2 Progenitors active 12 0.16944075 Progenitors_12

10x_3250_t2_TAGAATACTCGATG-1 Patient2 Progenitors active 12 0.13903684 Progenitors_12

10x_3250_t2_TAGCCCTGCGAACT-1 Patient2 Progenitors nonactive 5 0.06519878 Progenitors_5

10x_3250_t2_TAGCCGCTTCTGGA-1 Patient2 Progenitors nonactive 5 0.09054594 Progenitors_5

10x_3250_t2_TAGGAGCTTCCGTC-1 Patient2 Progenitors nonactive 5 0.1049553 Progenitors_5

10x_3250_t2_TAGGCAACGGACGA-1 Patient2 Progenitors active 12 0.12898675 Progenitors_12

10x_3250_t2_TAGGTCGATACTGG-1 Patient2 Progenitors nonactive 5 0.05654366 Progenitors_5

10x_3250_t2_TAGGTTCTAACGAA-1 Patient2 Progenitors active 5 0.15715554 Progenitors_5
10x_3250_t2_TAGTAAACGGTGGA-1 Patient2 Progenitors nonactive 5 0.09454061 Progenitors_5
10x_3250_t2_TAGTCTTGGGAACG-1 Patient2 Progenitors nonactive 5 0.1117082 Progenitors_5
10x_3250_t2_TAGTGGTGGCCAAT-1 Patient2 Progenitors nonactive 5 0.08493437 Progenitors_5
10x_3250_t2_TAGTGGTGGGCAAG-1 Patient2 Progenitors nonactive 5 0.0957295 Progenitors_5
10x_3250_t2_TATAGCCTACGTGT-1 Patient2 Progenitors active 12 0.13554943 Progenitors_12
10x_3250_t2_TATCACTGGTTAGC-1Patient2 Progenitors nonactive 5 0.08098726 Progenitors_5
10x_3250_t2_TATCCTGATGCATG-1 Patient2 Progenitors nonactive 5 0.11318242 Progenitors_5
10x_3250_t2_TATCTGACTCAGGT-1Patient2 Progenitors nonactive 5 0.07615243 Progenitors_5
10x_3250_t2_TATGAATGCAGATC-1 Patient2 Progenitors nonactive 5 0.08709023 Progenitors_5
10x_3250_t2_TATGGGTGAGAGGC-1 Patient2 Progenitors nonactive 5 0.07773762 Progenitors_5
10x_3250_t2_TATGTCACTCAGGT-1Patient2 Progenitors nonactive 5 0.11435546 Progenitors_5
10x_3250_t2_TATGTGCTTCAGAC-1 Patient2 Progenitors nonactive 5 0.08409422 Progenitors_5
10x_3250_t2_TATTGCTGGAATCC-1Patient2 Progenitors nonactive 12 0.10573204 Progenitors_12
10x_3250_t2_TCAAGGTGACGCAT-1 Patient2 Progenitors nonactive 5 0.07545495 Progenitors_5
10x_3250_t2_TCAAGGTGTGGAGG-1 Patient2 Progenitors nonactive 5 0.07908503 Progenitors_5
10x_3250_t2_TCAAGTCTATTCGG-1 Patient2 Progenitors nonactive 5 0.06505612

Progenitors_5
10x_3250_t2_TCAATAGAGTCGTA-1 Patient2 Progenitors nonactive 5 0.05253313 Progenitors_5
10x_3250_t2_TCAATCACAAGTGA-1 Patient2 Progenitors nonactive 5 0.1040993 Progenitors_5
10x_3250_t2_TCACCTCTTGGTTG-1 Patient2 T cells nonactive 9 0.09874136 T cells_9
10x_3250_t2_TCAGAGACAACCGT-1 Patient2 Progenitors active 12 0.13932217 Progenitors_12
10x_3250_t2_TCAGAGACGTTTCT-1 Patient2 Progenitors active 5 0.1235654 Progenitors_5
10x_3250_t2_TCAGCGCTATGACC-1 Patient2 Progenitors nonactive 5 0.07478917 Progenitors_5
10x_3250_t2_TCATGTACAGATCC-1 Patient2 Progenitors nonactive 5 0.10750745 Progenitors_5
10x_3250_t2_TCATGTACGCAAGG-1 Patient2 Progenitors nonactive 6 0.06962146 Progenitors_6
10x_3250_t2_TCCACGTGTGCTAG-1 Patient2 Progenitors nonactive 5 0.06781434 Progenitors_5
10x_3250_t2_TCCACTCTCTTCGC-1 Patient2 Progenitors nonactive 5 0.0742185 Progenitors_5
10x_3250_t2_TCCCACGACCTCGT-1 Patient2 T cells nonactive 9 0.07526473 T cells_9
10x_3250_t2_TCCCGAACATTCGG-1 Patient2 Progenitors nonactive 12 0.10801471 Progenitors_12
10x_3250_t2_TCCCGATGACGCTA-1 Patient2 Progenitors active 12 0.18584744 Progenitors_12
10x_3250_t2_TCCTATGACTGACA-1 Patient2 Progenitors active 5 0.12129859 Progenitors_5
10x_3250_t2_TCGAATCTTTCATC-1 Patient2 Progenitors nonactive 5 0.09793291 Progenitors_5
10x_3250_t2_TCGACCTGATCTTC-1 Patient2 Progenitors nonactive 5 0.10105574 Progenitors_5

10x_3250_t2_TCGAGAACGATAGA-1 Patient2 Progenitors active 5 0.12421533 Progenitors_5
10x_3250_t2_TCGAGCCTCAAAGA-1 Patient2 Progenitors nonactive 6 0.06920931 Progenitors_6
10x_3250_t2_TCGAGCCTGGCGAA-1 Patient2 Progenitors nonactive 5 0.05586203 Progenitors_5
10x_3250_t2_TCGAGCCTGTGTCA-1 Patient2 Progenitors active 12 0.17811172 Progenitors_12
10x_3250_t2_TCGATTTGGTCTGA-1 Patient2 Progenitors active 5 0.17353053 Progenitors_5
10x_3250_t2_TCGCACACCTGTAG-1 Patient2 Progenitors nonactive 5 0.09311394 Progenitors_5
10x_3250_t2_TCGCACTGCTAGTG-1 Patient2 B cells nonactive 7 0.10562108 B cells_7
10x_3250_t2_TCGGTAGACAACCA-1 Patient2 Progenitors active 5 0.12652971 Progenitors_5
10x_3250_t2_TCTAGACTTGACAC-1 Patient2 Progenitors active 12 0.16625452 Progenitors_12
10x_3250_t2_TCTATGTGGGCGAA-1 Patient2 Progenitors nonactive 5 0.08468074 Progenitors_5
10x_3250_t2_TCTCAAACATCGGT-1 Patient2 Progenitors active 5 0.11942806 Progenitors_5
10x_3250_t2_TCTCCACTTGGTCA-1 Patient2 Progenitors active 5 0.12036333 Progenitors_5
10x_3250_t2_TCTTACGAAAGTGA-1 Patient2 Progenitors nonactive 5 0.08293704 Progenitors_5
10x_3250_t2_TCTTACGACTCCAC-1 Patient2 Progenitors nonactive 5 0.08406252 Progenitors_5
10x_3250_t2_TCTTCAGAATGTGC-1 Patient2 Progenitors nonactive 5 0.10107159 Progenitors_5
10x_3250_t2_TGACCAGACCTGTC-1 Patient2 Progenitors nonactive 5 0.07683406 Progenitors_5
10x_3250_t2_TGACGAACGGTAGG-1 Patient2 Progenitors active 5 0.12936719

Progenitors_5
10x_3250_t2_TGACTTACATCGAC-1 Patient2 Progenitors nonactive 5 0.1016264
Progenitors_5
10x_3250_t2_TGACTTACGAATGA-1 Patient2 Progenitors nonactive 5 0.10276774
Progenitors_5
10x_3250_t2_TGAGGACTTGTAGC-1 Patient2 Progenitors nonactive 5 0.08621838
Progenitors_5
10x_3250_t2_TGATAAACTGCATG-1 Patient2 Progenitors active 5 0.17433898
Progenitors_5
10x_3250_t2_TGATCACTACTGGT-1Patient2 T cells active 9 0.12751252 T cells_9
10x_3250_t2_TGATTAGAGAAAGT-1 Patient2 Progenitors active 12 0.17608268
Progenitors_12
10x_3250_t2_TGATTCACCAGTTG-1 Patient2 Progenitors active 12 0.14001966
Progenitors_12
10x_3250_t2_TGCAACGAGATGAA-1 Patient2 Progenitors nonactive 5 0.09553928
Progenitors_5
10x_3250_t2_TGCAAGTGCAACCA-1 Patient2 Progenitors nonactive 12 0.09855114
Progenitors_12
10x_3250_t2_TGCACAGACACAAC-1 Patient2 Progenitors active 12 0.15287553
Progenitors_12
10x_3250_t2_TGCCCAACCCCGTT-1 Patient2 Progenitors nonactive 5 0.09501617
Progenitors_5
10x_3250_t2_TGCCGACTGATAAG-1 Patient2 Progenitors nonactive 5 0.07134931
Progenitors_5
10x_3250_t2_TGGAACACGTACGT-1 Patient2 Progenitors active 12 0.14041595
Progenitors_12
10x_3250_t2_TGGATCGATTCTAC-1Patient2 Progenitors nonactive 5 0.11118509
Progenitors_5
10x_3250_t2_TGGATGACCCTTCG-1 Patient2 Progenitors nonactive 5 0.11012301
Progenitors_5
10x_3250_t2_TGGCAATGGGTCTA-1 Patient2 Progenitors active 12 0.15457168
Progenitors_12
10x_3250_t2_TGGCACCTTGGTCA-1 Patient2 Progenitors nonactive 5 0.11159724

Progenitors_5

10x_3250_t2_TGGTAGTGCGTTGA-1 Patient2 Progenitors active 6 0.14277788
Progenitors_6

10x_3250_t2_TGGTATCTCCCAAA-1 Patient2 Progenitors nonactive 5 0.09791706
Progenitors_5

10x_3250_t2_TGTAAAACGCAAGG-1 Patient2 Monocytes active 8 0.11968169
Monocytes_8

10x_3250_t2_TGTAATGACATCAG-1 Patient2 Progenitors active 5 0.1505136
Progenitors_5

10x_3250_t2_TGTACTTGAACGTC-1 Patient2 Progenitors active 1 0.20155665
Progenitors_1

10x_3250_t2_TGTACTTGTATGCG-1 Patient2 Progenitors nonactive 5 0.09907425
Progenitors_5

10x_3250_t2_TGTCAGGATCTTCA-1 Patient2 Progenitors active 12 0.19001649
Progenitors_12

10x_3250_t2_TGTGAGTGCTCAGA-1 Patient2 Progenitors active 12 0.13342527
Progenitors_12

10x_3250_t2_TGTGGATGCGTACA-1 Patient2 Progenitors active 12 0.13773699
Progenitors_12

10x_3250_t2_TGTGGATGGTCGAT-1 Patient2 Progenitors nonactive 5 0.07204679
Progenitors_5

10x_3250_t2_TGTTACTGAGTCGT-1 Patient2 Progenitors active 6 0.13445565
Progenitors_6

10x_3250_t2_TTAACCACCGAACT-1 Patient2 Progenitors nonactive 5 0.10901338
Progenitors_5

10x_3250_t2_TTACAGCTGTTGGT-1 Patient2 Progenitors nonactive 5 0.09479424
Progenitors_5

10x_3250_t2_TTACTCGAGCAGAG-1 Patient2 Progenitors nonactive 5 0.07537569
Progenitors_5

10x_3250_t2_TTAGAATGTGAGCT-1 Patient2 Progenitors active 5 0.12169488
Progenitors_5

10x_3250_t2_TTAGACCTGACTAC-1 Patient2 Progenitors nonactive 5 0.07394902
Progenitors_5

10x_3250_t2_TTCAAAGATCTTCA-1 Patient2 Progenitors nonactive 5 0.09967662
Progenitors_5
10x_3250_t2_TTCAAGCTAACCTG-1 Patient2 Progenitors nonactive 6 0.10072285
Progenitors_6
10x_3250_t2_TTCACAACGCCCTT-1 Patient2 Progenitors active 5 0.14513981
Progenitors_5
10x_3250_t2_TTCCATGACCTTCG-1 Patient2 Progenitors nonactive 5 0.07325154
Progenitors_5
10x_3250_t2_TTGACACTACCACA-1 Patient2 Progenitors nonactive 5 0.08358696
Progenitors_5
10x_3250_t2_TTGAGGTGAGCGTT-1 Patient2 Progenitors active 6 0.13191935
Progenitors_6
10x_3250_t2_TTGAGGTGTTGGCA-1 Patient2 Progenitors nonactive 5 0.08574282
Progenitors_5
10x_3250_t2_TTGGAGTGAGGCGA-1 Patient2 Progenitors nonactive 5 0.1172405
Progenitors_5
10x_3250_t2_TTGGGAACAAAGCA-1 Patient2 Progenitors nonactive 5 0.06884471
Progenitors_5
10x_3250_t2_TTGGTACTGTTACG-1 Patient2 Progenitors active 12 0.14138292
Progenitors_12
10x_3250_t2_TTGTCATGTGCTAG-1 Patient2 Progenitors nonactive 5 0.08377719
Progenitors_5
10x_3250_t2_TTTCAGTGTTTGCT-1 Patient2 Progenitors active 5 0.12744912
Progenitors_5
10x_3250_t2_TTTGACTGTTTCAC-1 Patient2 Progenitors nonactive 6 0.11058272
Progenitors_6

Table S4. Violin plot analysis for each cluster's TOP2 markers

CCL3	0	2.43773717	0.872	0.447	0	0
CCL18	2.84E-280	2.49175421	0.7	0.284	3.28E-276	0
DCN	0	5.98701779	0.991	0.235	0	1
FGF7	0	5.81771395	0.625	0.036	0	1
FABP5	0	2.59908181	0.954	0.176	0	2
GABARAP	0	2.2542635	0.959	0.448	0	2
FCN1	0	5.50601212	0.887	0.172	0	3
SERPINA1	0	3.95273473	0.889	0.302	0	3
CCL18	1.16E-205	1.53334767	0.686	0.301	1.34E-201	4
SDC3	9.60E-146	1.89427242	0.53	0.24	1.11E-141	4
HBB	0	7.18290752	0.735	0.093	0	5
SCNN1A	6.12E-258	8.38895015	0.523	0.058	7.07E-254	5
CFB	1.06E-268	4.93769632	0.964	0.298	1.22E-264	6
CCNB1	3.22E-166	4.10532551	0.535	0.097	3.71E-162	6
IGLL5	0	15.310638	0.997	0.16	0	7
MZB1	0	12.556912	1	0.068	0	7
MT-ND3	8.95E-51	2.2682047	0.601	0.306	1.03E-46	8
ADORA3	7.03E-26	0.95851169	0.662	0.41	8.12E-22	8
GNLY	8.68E-115	8.70651633	0.708	0.237	1.00E-110	9
CCL5	7.58E-84	8.27859011	0.523	0.145	8.75E-80	9
TSPAN12	0	6.74990785	0.775	0.017	0	10
PPP1R14A	0	6.48806852	0.63	0.03	0	10
MUC1	0	7.66928783	0.988	0.064	0	11
CTD-2538C1.2	0	7.60793182	0.988	0.026	0	11
TNC	9.33E-299	6.83008128	0.587	0.017	1.08E-294	12
PAEP	1.91E-144	6.5567769	0.641	0.049	2.21E-140	12
KIAA0101	1.25E-112	4.51631718	0.779	0.093	1.45E-108	13
CDK1	5.93E-84	4.09866808	0.547	0.056	6.84E-80	13
CCL17	0	13.9721697	0.649	0.007	0	14
CCL19	0	11.2846145	0.892	0.005	0	14

CST7	8.30E-59	3.1282237	0.594	0.074	9.59E-55	15
GZMA	1.92E-54	2.60793976	0.719	0.122	2.22E-50	15
IDH1	5.16E-09	2.39060942	0.694	0.375	5.96E-05	16
GLIPR2	0.00146556	2.35539088	0.556	0.383	1	16

Table S5. 28 genes obtained from the intersection of marker genes with strong cell subgroup specificity and oxidative stress response-related gene sets

gene	p_val	avg_log2FC	pct.1	pct.2	p_val_adj	cluster
IL6	0	5.113960873	0.525	0.049	0	1
SOD3	0	4.757115757	0.868	0.09	0	1
MMP2	0	4.12652004	0.524	0.058	0	1
COL1A1	0	3.44476703	0.967	0.203	0	1
CAV1	0	3.136016102	0.874	0.158	0	1
PTGIS	0	3.044227937	0.707	0.089	0	1
HP	0	3.030076319	0.615	0.096	0	1
CD36	0	3.243090746	0.781	0.188	0	3
HBB	0	7.182907524	0.735	0.093	0	5
OSER1	1.73E-156	4.139681307	0.747	0.225	2.00E-152	5
MAP1LC3A	3.42E-144	3.013190925	0.634	0.16	3.95E-140	5
PRDX2	1.97E-127	3.313361709	0.899	0.462	2.27E-123	5
AQP1	1.79E-291	3.238516989	0.736	0.108	2.06E-287	6
SDC1	0	5.694780537	0.639	0.038	0	7
RAC2	1.44E-142	4.905133684	0.72	0.187	1.66E-138	9
STK17A	2.66E-119	4.445653329	0.663	0.183	3.07E-115	9
DDIT4	1.50E-65	4.144028392	0.614	0.233	1.73E-61	9
GPX3	5.07E-147	4.922572015	0.872	0.209	5.85E-143	10
PXDN	2.87E-238	3.993747773	0.832	0.084	3.31E-234	11
PPP5C	1.92E-216	3.368714262	0.882	0.104	2.22E-212	11
CRYAB	4.22E-64	4.907555999	0.957	0.276	4.88E-60	12
TPM1	8.53E-59	3.299916321	0.967	0.326	9.85E-55	12
CLU	6.92E-57	4.151471078	0.946	0.284	8.00E-53	12
COL1A1	1.11E-52	3.688214952	0.989	0.315	1.29E-48	12
HP	3.29E-17	3.783141069	0.511	0.173	3.80E-13	12
CCL19	0	11.28461451	0.892	0.005	0	14
CCR7	3.18E-256	7.041303524	0.986	0.057	3.68E-252	14
CFLAR	1.57E-51	4.033923141	0.851	0.235	1.82E-47	14
PMAIP1	2.46E-29	3.150642477	0.635	0.186	2.84E-25	14

GADD45A	8.01E-16	3.129398819	0.568	0.247	9.25E-12	14

Table S6. 56 intersection genes as ROS_markers obtained from the intersection of oxidative stress response factors and specific markers of cell subsets.

gene	p_val	avg_log2FC	pct.1	pct.2	p_val_adj	cluster
IL6	0	5.113960873	0.525	0.049	0	1
SOD3	0	4.757115757	0.868	0.09	0	1
MMP2	0	4.12652004	0.524	0.058	0	1
COL1A1	0	3.44476703	0.967	0.203	0	1
CAV1	0	3.136016102	0.874	0.158	0	1
PTGIS	0	3.044227937	0.707	0.089	0	1
HP	0	3.030076319	0.615	0.096	0	1
CDKN1A	0	2.892789805	0.782	0.275	0	1
TPM1	0	2.150741321	0.841	0.239	0	1
GADD45A	0	2.063191171	0.689	0.169	0	1
CLU	0	1.966541389	0.891	0.181	0	1
CYB5R3	0	1.775496787	0.761	0.268	0	1
CAPN2	0	1.768563308	0.728	0.216	0	1
ARF4	0	1.597167116	0.941	0.465	0	1
ICAM1	4.33E-145	1.5137606	0.579	0.245	5.00E-141	1
JUN	9.10E-144	1.553186658	0.82	0.477	1.05E-139	1
CD36	0	3.243090746	0.781	0.188	0	3
ITGB2	3.63E-220	1.630354283	0.871	0.482	4.20E-216	3
TXNIP	1.82E-15	2.059459621	0.531	0.428	2.11E-11	3
HBB	0	7.182907524	0.735	0.093	0	5
OSER1	1.73E-156	4.139681307	0.747	0.225	2.00E-152	5
MAP1LC3A	3.42E-144	3.013190925	0.634	0.16	3.95E-140	5

PRDX2	1.97E-127	3.313361709	0.899	0.462	2.27E-123	5
HDAC2	2.05E-118	2.409119018	0.764	0.287	2.36E-114	5
ASS1	3.72E-115	2.631542522	0.571	0.149	4.29E-111	5
DDIT4	8.26E-108	2.898386655	0.687	0.224	9.54E-104	5
TRAP1	1.73E-99	2.1059056	0.542	0.152	2.00E-95	5
CLU	3.54E-88	2.057829845	0.728	0.271	4.08E-84	5
PCNA	4.77E-88	1.929856655	0.663	0.23	5.51E-84	5
CRYAB	9.01E-66	2.607262936	0.675	0.265	1.04E-61	5
TRA2B	3.49E-47	2.095207555	0.634	0.287	4.04E-43	5
GADD45A	6.32E-45	2.475056684	0.566	0.235	7.30E-41	5
TPM1	1.63E-43	1.53491263	0.701	0.315	1.89E-39	5
MAOB	0	2.9723631	0.716	0.081	0	6
AQP1	1.79E-291	3.238516989	0.736	0.108	2.06E-287	6
PTGIS	3.77E-284	2.450407181	0.881	0.155	4.35E-280	6
SOD3	8.23E-245	1.876808187	0.891	0.182	9.50E-241	6
COL1A1	8.10E-236	2.632414004	0.992	0.293	9.35E-232	6
CAV1	2.05E-235	2.24324733	0.974	0.24	2.36E-231	6

HP	1.87E-222	2.464022177	0.765	0.151	2.16E-218	6
TPM1	8.19E-212	2.42729317	0.974	0.305	9.46E-208	6
NQO1	4.02E-181	1.791742426	0.762	0.168	4.64E-177	6
CAPN2	2.62E-153	1.805497949	0.866	0.272	3.02E-149	6
CYB5R3	7.93E-134	1.541986836	0.915	0.32	9.16E-130	6
SDC1	0	5.694780537	0.639	0.038	0	7
RAC2	4.25E-186	2.290211815	0.743	0.179	4.91E-182	7
CFLAR	6.48E-142	2.483298316	0.738	0.219	7.48E-138	7
PRDX2	6.78E-132	1.631579367	0.958	0.461	7.83E-128	7
HNRNPD	2.74E-47	2.110617562	0.55	0.27	3.16E-43	7
RAC2	1.44E-142	4.905133684	0.72	0.187	1.66E-138	9
STK17A	2.66E-119	4.445653329	0.663	0.183	3.07E-115	9
DDIT4	1.50E-65	4.144028392	0.614	0.233	1.73E-61	9
TXNIP	5.17E-12	1.599216493	0.549	0.439	5.98E-08	9
GPX3	5.07E-147	4.922572015	0.872	0.209	5.85E-143	10
ASS1	3.18E-137	2.668711398	0.749	0.153	3.67E-133	10
HSF1	2.20E-104	2.359161984	0.797	0.221	2.55E-100	10

PAWR	3.40E-98	2.001449469	0.502	0.085	3.93E-94	10
CLU	1.09E-90	2.93357042	0.881	0.276	1.26E-86	10
SLC25A33	1.21E-88	1.685950447	0.604	0.126	1.40E-84	10
PDCD10	1.22E-71	1.696912414	0.833	0.295	1.41E-67	10
CRYAB	6.23E-54	2.633119431	0.758	0.271	7.19E-50	10
BNIP3	1.22E-43	1.580301533	0.758	0.284	1.40E-39	10
TFAP2A	0	2.723492458	0.627	0.027	0	11
PXDN	2.87E-238	3.993747773	0.832	0.084	3.31E-234	11
PPP5C	1.92E-216	3.368714262	0.882	0.104	2.22E-212	11
NOXA1	7.19E-202	2.280961075	0.547	0.037	8.30E-198	11
NET1	2.97E-180	1.869512596	0.571	0.046	3.43E-176	11
ASS1	1.96E-153	2.885603653	0.919	0.155	2.26E-149	11
PINK1	1.40E-119	1.958383859	0.727	0.109	1.62E-115	11
PAWR	1.20E-114	1.755329308	0.634	0.086	1.38E-110	11
RFK	5.79E-82	1.54022522	0.714	0.14	6.69E-78	11
CRYAB	4.22E-64	4.907555999	0.957	0.276	4.88E-60	12
TPM1	8.53E-59	3.299916321	0.967	0.326	9.85E-55	12

CLU	6.92E-57	4.151471078	0.946	0.284	8.00E-53	12
PTGIS	1.93E-55	2.628242299	0.815	0.178	2.23E-51	12
MMP2	1.12E-54	2.284453151	0.685	0.125	1.29E-50	12
COL1A1	1.11E-52	3.688214952	0.989	0.315	1.29E-48	12
ASS1	2.69E-51	2.230326421	0.772	0.162	3.11E-47	12
CYB5R3	1.20E-46	2.675470347	0.935	0.339	1.39E-42	12
CAV1	2.05E-43	2.037883609	0.913	0.263	2.37E-39	12
CAPN2	7.27E-43	2.227039855	0.902	0.29	8.40E-39	12
PYCR1	2.99E-41	1.880848785	0.63	0.13	3.45E-37	12
NQO1	1.10E-35	1.799685861	0.717	0.187	1.27E-31	12
ATP2B4	6.40E-30	2.912559325	0.511	0.111	7.40E-26	12
HP	3.29E-17	3.783141069	0.511	0.173	3.80E-13	12
CCL19	0	11.28461451	0.892	0.005	0	14
CCR7	3.18E-256	7.041303524	0.986	0.057	3.68E-252	14
CFLAR	1.57E-51	4.033923141	0.851	0.235	1.82E-47	14
CDKN1A	7.05E-35	2.229934723	0.919	0.349	8.14E-31	14
PMAIP1	2.46E-29	3.150642477	0.635	0.186	2.84E-25	14

GADD45A	8.01E-16	3.129398819	0.568	0.247	9.25E-12	14

Table S7: 2928 differentially expressed genes

gene	log2FC	pvalue	adjusted_pvalue
TOMM6	-3.0171639	0	0
XBP1	-2.7636323	0	0
KREMEN1	-2.2427757	0	0
TREX1	-2.2035696	0	0
OVCA2	-2.1326903	0	0
CEMP1	-1.5206557	9.019E-271	1.994E-267
GAGE12B	-2.4069061	9.585E-302	2.422E-298
C1QTNF5	-1.8392013	1.675E-246	3.291E-243
CLDN22	4.07339326	0	2.051E-306
RP11-724O16.1	-2.0472986	1.206E-209	1.94E-206
PIGY	-1.7720972	4.445E-209	6.552E-206
PTPRCAP	-1.3088842	3.211E-198	4.056E-195
MSMP	1.8908252	8.381E-211	1.482E-207
HEPN1	2.52522778	1.883E-199	2.562E-196
SERPINA7	-5.6048955	1.08E-175	1.274E-172
BIVM-ERCC5	-3.3204061	4.682E-171	5.175E-168
ACSM2B	-4.2482667	8.517E-163	8.861E-160
ADGRG4	-4.5941323	1.418E-152	1.393E-149
RP11-514P8.6	-0.778539	9.834E-133	9.154E-130
BOLA2	-0.8334717	9.823E-130	8.687E-127
CCL15-CCL14	-2.9453051	7.024E-127	5.916E-124
CLPS	-5.0738931	1.489E-123	1.197E-120
INS	-2.1703039	8.223E-123	6.324E-120
RP4-539M6.19	-1.3687256	2.306E-121	1.699E-118
RP1-317E23.6	-1.1454073	1.151E-120	8.144E-118
RP11-162P23.2	-2.1268424	5.011E-120	3.409E-117
SPINK1	-5.7620678	3.175E-117	2.08E-114
PRR23A	-4.5503666	4.332E-116	2.736E-113
C1QTNF3-AMACR	-1.5487058	4.608E-114	2.81E-111

RP11-216L13.16	-1.7892918	1.04E-113	6.129E-111
CTRC	-2.8157892	1.213E-112	6.918E-110
RP4-777O23.3	-1.4644695	2.575E-107	1.423E-104
SFTPA1	-2.7373489	2.331E-103	1.249E-100
LMAN1L	-5.7560088	1.83E-101	9.519E-99
TNFRSF6B	-1.2525079	1.953E-100	9.868E-98
C22orf31	2.23027593	9.155E-100	4.4981E-97
MYL1	-2.5075406	2.906E-98	1.3891E-95
TBC1D3I	-2.0129602	3.2709E-95	1.5224E-92
CGA	-2.9594463	7.6721E-93	3.3924E-90
TSSK2	1.94043496	4.3896E-93	1.9908E-90
TDGF1P3	-2.3146638	1.2269E-91	5.2928E-89
RP11-343C2.12	-1.2041219	2.169E-91	9.1341E-89
GKN1	-1.6717785	2.4149E-90	9.933E-88
ERAS	-2.8300606	2.5812E-90	1.0376E-87
F11	-2.8513876	2.3738E-87	9.3303E-85
CELA3B	-1.7826609	1.2103E-86	4.6536E-84
PAGE2B	-4.1064149	4.5694E-86	1.7196E-83
DEFA3	-3.439514	2.2532E-83	8.3025E-81
RP11-542C16.2	-1.5377531	1.2295E-81	4.4379E-79
FAM9C	-3.3084323	5.1847E-78	1.834E-75
RFPL1	-1.6505831	5.5132E-78	1.912E-75
PHGR1	-2.7044782	2.763E-77	9.3978E-75
TTR	-4.0231124	6.0234E-77	2.0101E-74
PI16	-2.3812651	1.2389E-76	4.0578E-74
CCL27	-0.9472275	1.2921E-74	4.1552E-72
CPA1	-2.1045582	1.5136E-74	4.7805E-72
TNP1	-2.1365396	3.7737E-74	1.171E-71
SPATA22	-3.5972163	6.1595E-74	1.8783E-71
PAGE4	-3.5628046	8.8762E-73	2.6609E-70
APOA2	-1.6001767	1.412E-72	4.1624E-70
MIA-RAB4B	-1.2847131	3.6065E-69	1.0457E-66

EPB42	-2.7437719	8.8402E-69	2.5219E-66
MUCL1	-4.7360085	2.4855E-68	6.9781E-66
SFTPC	-1.2755061	3.1304E-68	8.651E-66
HTR1A	-2.5387667	8.2262E-68	2.2384E-65
RP4-583P15.14	-0.8477415	1.4002E-67	3.7524E-65
RP11-403P17.5	-1.7159185	5.2457E-66	1.3848E-63
ORM2	-3.2439465	5.8083E-66	1.5108E-63
RAG2	-1.9306107	6.2187E-66	1.5941E-63
RP11-468E2.1	-1.0610421	1.8802E-65	4.7508E-63
IL13	-3.5148792	3.8618E-64	9.6203E-62
NPPB	-2.5180624	9.4965E-64	2.3328E-61
MYH1	-1.9497429	4.948E-63	1.1988E-60
CYP11B1	-1.2388444	9.8286E-62	2.3492E-59
AMBP	-2.2800763	1.2707E-61	2.9965E-59
PTGES3L-AARSD1	-3.27871	3.5775E-61	8.3256E-59
TBC1D3D	-2.8609463	1.7292E-60	3.972E-58
DNAJC5G	-2.9319877	3.6747E-60	8.3326E-58
NDST4	-2.2423324	8.3366E-60	1.8665E-57
DDX3Y	-1.224921	8.4504E-60	1.8683E-57
PNLIPRP1	-1.0368724	8.6658E-60	1.8922E-57
TMPRSS11B	-3.1293299	1.1918E-59	2.5706E-57
RP11-618P17.4	-3.362338	2.6952E-58	5.7434E-56
APOC3	-1.9253316	4.1028E-58	8.6389E-56
APOH	-5.6709838	1.4155E-57	2.9453E-55
PRM1	-2.1931614	2.2249E-57	4.5757E-55
KDM5D	-1.1327401	1.8905E-56	3.8434E-54
SUPT20HL2	-1.6098392	8.8152E-56	1.7717E-53
RIPPLY1	-2.5323093	3.6481E-55	7.1693E-53
AC011530.4	-1.0368578	1.9989E-54	3.8852E-52
TEKT5	-1.874303	3.3342E-54	6.4099E-52
SPINK7	-1.5249107	4.8408E-54	9.2064E-52
USP50	-2.8525218	6.5652E-54	1.2237E-51

SYCN	-1.7205805	6.5726E-54	1.2237E-51
NEDD8-MDP1	-1.7054264	1.0281E-53	1.8942E-51
OPTC	-1.7817537	3.501E-53	6.3837E-51
CELA3A	-1.1425994	1.0747E-52	1.9395E-50
RP11-321N4.5	-0.9219522	2.8381E-52	5.0704E-50
XIRP2	-1.3049907	3.1204E-52	5.5191E-50
TMEM145	-1.838781	3.5096E-52	6.146E-50
TUBA3E	-2.8919371	1.3722E-51	2.3793E-49
SERPINB3	-2.9454729	1.416E-51	2.4316E-49
FAM187A	2.41703962	2.6059E-51	4.4317E-49
ZNF536	-2.8786686	1.5897E-50	2.6777E-48
OR2A42	-4.0962732	3.7124E-50	6.0797E-48
TMEM229A	-2.4365185	2.5917E-50	4.284E-48
SPRR1A	-2.548153	2.5757E-50	4.284E-48
NPFF	0.63557198	4.3319E-50	7.0292E-48
RP11-234B24.6	-0.6182548	7.5477E-50	1.2136E-47
MYH2	-1.497488	5.9545E-49	9.4881E-47
SEZ6L	-4.7564473	9.8182E-49	1.5505E-46
DHRS2	-1.5080841	1.0763E-48	1.6847E-46
PRM2	-1.5780863	1.6766E-48	2.6012E-46
SHISA3	-4.0116752	2.939E-48	4.5201E-46
DLX1	-1.8674355	7.3582E-48	1.1219E-45
OLFM4	-4.9924689	8.1391E-48	1.2304E-45
TIGD3	-1.9442167	9.2232E-48	1.3825E-45
DSG3	-3.0931846	1.5307E-47	2.2751E-45
FGB	-1.1671221	1.7624E-47	2.5976E-45
GDF1	2.02193119	4.7018E-47	6.8727E-45
ICAM4	0.93858527	9.1328E-46	1.324E-43
AQP8	-1.6045586	1.1933E-45	1.716E-43
PMP2	-1.3946102	1.4342E-45	2.0458E-43
SEZ6	-3.1398083	1.8306E-45	2.5903E-43
FBXO40	-1.2546009	2.0995E-45	2.9471E-43
CPA2	-5.9314639	4.4762E-45	6.2339E-43

AFP	-1.7677352	1.3677E-44	1.8899E-42
NDUFC2-KCTD14	0.81747217	2.3425E-44	3.1871E-42
AC084219.2	-0.6594815	1.7656E-44	2.4208E-42
GOT1L1	-1.0084358	2.8991E-44	3.9142E-42
RAB4B-EGLN2	-0.6983008	3.0696E-44	4.1131E-42
MUC13	-3.4327552	5.2233E-44	6.8944E-42
SCGN	-1.8050146	5.017E-44	6.6718E-42
EDN3	-3.5196226	6.6248E-44	8.6795E-42
DCD	-1.5973843	9.1637E-44	1.1917E-41
HOXA11	-4.2596876	2.1545E-43	2.7815E-41
INSC	-1.6811331	3.8969E-43	4.9946E-41
HSPE1-MOB4	-1.1904571	3.3501E-42	4.2628E-40
TXNDC2	-1.82033	5.5048E-42	6.9052E-40
YIPF7	-1.4129937	5.2105E-42	6.5827E-40
SLC6A4	-1.9004737	9.7263E-42	1.2115E-39
MYO1A	-1.322398	1.396E-41	1.7267E-39
CEACAM5	-2.7320614	1.7194E-41	2.0974E-39
NCBP2L	-1.7013992	1.6607E-41	2.0397E-39
KLK3	-2.269002	4.9479E-41	5.9941E-39
DCAF8L2	-3.7771468	6.2201E-41	7.484E-39
HOXD11	-4.9409467	8.9462E-41	1.062E-38
PNLIP	-0.6323924	7.9007E-41	9.4418E-39
PLA2G2F	-3.2441428	1.9498E-40	2.299E-38
FXYD6-FXYD2	1.32208763	2.8471E-40	3.3349E-38
QRFPR	-1.4440963	3.4968E-40	4.0689E-38
PPAN-P2RY11	-0.790199	3.5501E-40	4.1039E-38
RBAK-RBAKDN	-4.468564	6.554E-40	7.5274E-38
TNFSF12-TNFSF13	-4.441116	1.2691E-39	1.4207E-37
CRCT1	-1.6961324	7.2844E-40	8.3122E-38
OR51B4	-2.7899853	9.2451E-40	1.0482E-37

NXNL1	-3.6680976	9.3098E-40	1.0488E-37
UBE2F-SCLY	-4.2902848	1.7469E-39	1.9433E-37
RFPL3	-2.9327325	3.0212E-39	3.3397E-37
GP5	-1.8085461	3.1683E-39	3.4806E-37
NEFL	-2.2736824	3.7916E-39	4.1396E-37
TMOD4	-0.6771394	4.1687E-39	4.5234E-37
SLC26A3	-1.8181262	6.7686E-39	7.2998E-37
HSFX2	-1.0738963	8.7629E-39	9.3933E-37
VGF	-5.1814367	2.0384E-38	2.146E-36
REG1A	-2.655328	1.5125E-38	1.6116E-36
DLK1	-2.4399749	1.7377E-38	1.8404E-36
CLCA4	-3.181162	3.1636E-38	3.3109E-36
SPRR1B	-2.6617965	3.487E-38	3.6279E-36
LMOD2	-1.2873257	4.2956E-38	4.443E-36
RNF133	-2.2568485	5.604E-38	5.7627E-36
KLRC4-KLRK1	-1.9999224	1.7893E-37	1.8293E-35
KLK2	-4.3121075	2.3324E-37	2.3709E-35
HNF4A	-5.5659236	3.4435E-37	3.4605E-35
CHURC1-FNTB	-1.0886692	2.733E-37	2.7622E-35
CORO7-PAM16	-5.9557687	9.514E-37	9.507E-35
VCX3B	-2.2935725	1.8662E-36	1.8543E-34
ITIH2	-5.1884414	4.4324E-36	4.3313E-34
RNF186	-1.4356511	3.463E-36	3.4218E-34
GDPD4	-1.371455	3.926E-36	3.8578E-34
PRL	-1.7003118	7.5172E-36	7.3053E-34
TAC3	-1.4987084	8.4621E-36	8.1787E-34
RP11-426L16.10	-2.1418141	1.0921E-35	1.0497E-33
CCL16	-1.4832709	2.3039E-35	2.2026E-33
C12orf50	-1.6057519	2.5112E-35	2.3879E-33
CHRNB2	-3.6202588	5.8826E-35	5.564E-33
F2	-2.2383807	9.5036E-35	8.941E-33
MSLNL	4.07606544	1.0966E-34	1.0262E-32

GH1	-1.3057774	2.0052E-34	1.8569E-32
FAM163B	-1.3278299	2.0017E-34	1.8569E-32
CAMP	-4.7123163	3.5127E-34	3.2359E-32
HPSE2	-2.7956722	4.657E-34	4.2678E-32
XXbac-BPG246D15.9	-3.1518843	7.843E-34	7.1505E-32
SLC5A8	-0.8942396	8.4801E-34	7.6916E-32
SENP3-EIF4A1	-2.889493	8.9964E-34	8.1183E-32
MYH6	-2.8424848	9.7551E-34	8.7583E-32
IVL	-1.9206781	4.6664E-33	4.1684E-31
CHGA	-3.2283528	5.6205E-33	4.9954E-31
PSORS1C2	-2.1082366	6.2401E-33	5.5184E-31
ADH4	-2.304918	7.3141E-33	6.4361E-31
CEL	-1.7418434	7.4819E-33	6.5511E-31
RP5-877J2.1	-2.5645866	1.2982E-32	1.1311E-30
KRT222	-4.092297	2.3613E-32	2.0274E-30
CDHR4	-2.7857097	2.0546E-32	1.7813E-30
IL13RA2	-3.1707139	2.2957E-32	1.9807E-30
USP17L2	-2.6102541	5.0921E-32	4.3509E-30
ACTA1	-1.2386913	2.0014E-31	1.7019E-29
CALHM1	-1.940965	6.893E-31	5.8334E-29
PSMA8	-3.8174531	1.3231E-30	1.1143E-28
SLC17A1	-4.1296183	2.1288E-30	1.7844E-28
SAA2	-1.9100078	2.2652E-30	1.8898E-28
PAH	-1.7665948	2.9059E-30	2.413E-28
CRYGD	-1.6262684	3.0487E-30	2.5197E-28
SPRR2G	-4.0961915	5.4973E-30	4.5014E-28
STMN4	-2.1288855	4.4257E-30	3.6408E-28
CRP	-0.8619199	6.0208E-30	4.9073E-28
STON1-GTF2A1L	-1.0464144	6.8448E-30	5.5534E-28
FGF20	-1.154838	1.2862E-29	1.0388E-27
LY6H	-1.8920444	1.4956E-29	1.2024E-27

SFRP5	-2.586946	1.9517E-29	1.555E-27
CLEC4G	-1.4950227	1.9105E-29	1.529E-27
RP11-977G19.10	-4.2843793	4.7407E-29	3.7266E-27
FGA	-0.8587691	2.5886E-29	2.0531E-27
SPATA21	-3.0024678	2.9094E-29	2.2972E-27
IL5	-1.4812413	5.1693E-29	4.0455E-27
ERICH4	-0.9800982	6.2377E-29	4.8602E-27
TFF1	-4.7168227	3.1295E-28	2.4066E-26
URGCP-MRPS24	-3.2916047	4.2752E-28	3.2734E-26
SERPINI2	-4.0532647	2.7583E-28	2.1397E-26
CTSG	-2.8033341	2.991E-28	2.3102E-26
SPTA1	-4.3837854	6.5109E-28	4.9637E-26
RBPJL	-4.7673444	9.6553E-28	7.298E-26
PRDM7	-3.7602231	1.1232E-27	8.4534E-26
NPY	-3.1867653	8.0336E-28	6.0983E-26
UNC13C	-0.9519532	1.3378E-27	1.0026E-25
CRYGS	-1.0176896	1.5522E-27	1.1584E-25
SCNN1B	-1.3829532	1.9012E-27	1.4128E-25
LMOD3	-2.1599333	2.63E-27	1.9463E-25
CLEC2L	-2.238529	3.1584E-27	2.3276E-25
GJA9	-2.4915048	5.1262E-27	3.7465E-25
FMO3	-1.9119759	4.8174E-27	3.5355E-25
OTOF	-3.1609981	5.578E-27	4.06E-25
GFRA1	-1.3828044	6.1804E-27	4.48E-25
SLC35F4	-2.0435876	9.3643E-27	6.7328E-25
CA1	-1.0388286	8.9845E-27	6.4861E-25
RP11-330H6.5	-0.6815935	1.0166E-26	7.2798E-25
CFHR1	-1.8741851	1.0775E-26	7.6849E-25
DND1	0.84949239	2.0008E-26	1.4212E-24
PNLDC1	-1.8200943	2.6393E-26	1.8598E-24
ASIC4	-1.8598029	2.6238E-26	1.8563E-24

CTD-2535L24.2	-4.6449621	1.1961E-25	8.1684E-24
NAT2	-1.3110512	3.95E-26	2.7724E-24
CASR	-2.5117599	4.5565E-26	3.1854E-24
MROH2B	-4.4929652	9.1986E-26	6.3553E-24
PLA1A	-1.630381	6.3312E-26	4.4087E-24
SMLR1	-1.0660218	8.5374E-26	5.9216E-24
MUSK	-2.5686469	1.0531E-25	7.2473E-24
USP41	-1.5074184	1.1902E-25	8.1594E-24
CHRNA4	-1.6944601	1.3656E-25	9.2898E-24
FCER2	-3.3554525	1.3951E-25	9.454E-24
GALNT9	-1.054303	1.4762E-25	9.9656E-24
EYA1	-3.9620143	1.7481E-25	1.1756E-23
PAX9	-1.9563474	2.2635E-25	1.5164E-23
CDH20	-2.1772997	5.3935E-25	3.5998E-23
COL2A1	-2.2411668	7.4834E-25	4.9759E-23
TCHH	-4.3872146	1.3919E-24	9.1519E-23
AC037459.4	-2.0366258	8.0068E-25	5.304E-23
SFTPB	-1.7778486	1.2436E-24	8.2072E-23
NPY5R	-4.389583	2.2659E-24	1.4734E-22
ELANE	-1.7853045	1.7319E-24	1.1345E-22
DIO1	-1.4334322	2.1681E-24	1.415E-22
RP11-729L2.2	-1.0492531	2.2827E-24	1.4789E-22
CEND1	-2.266389	3.0384E-24	1.9613E-22
RGS8	-1.262363	3.8063E-24	2.4392E-22
SLN	-1.29169	3.628E-24	2.3334E-22
CD177	-1.8537168	3.93E-24	2.5094E-22
ZPLD1	-1.5489152	4.413E-24	2.8076E-22
OR56B1	-3.430471	7.9233E-24	4.9871E-22
SPACA5	-3.9599762	1.381E-23	8.5403E-22
TRIM50	-1.5041342	5.334E-24	3.3815E-22
GSTA3	-1.4315647	7.5002E-24	4.7377E-22
TMEM132E	-1.2118721	9.2033E-24	5.7519E-22
TRIM63	-1.2078533	9.0658E-24	5.6861E-22

NUTM2E	-2.4718925	1.0296E-23	6.3897E-22
CTRB1	-0.6086629	9.6306E-24	5.9977E-22
HBG1	-4.1506845	3.4944E-23	2.1312E-21
TMEM178B	-1.7067585	1.5254E-23	9.4004E-22
MS4A1	-2.371038	1.7991E-23	1.1049E-21
NANOGP8	-2.485397	2.7149E-23	1.6615E-21
NT5C1A	-2.0731068	3.9902E-23	2.4169E-21
SLURP1	-4.5633763	3.8177E-23	2.3204E-21
UCMA	-1.6708971	4.1699E-23	2.5172E-21
CYP3A43	-2.2113282	4.4497E-23	2.677E-21
GAL	-3.409192	5.965E-23	3.5764E-21
CA10	-0.8639784	6.0024E-23	3.5866E-21
GATA5	-2.78669	7.1091E-23	4.2336E-21
SLC14A1	-1.3682813	8.1239E-23	4.8217E-21
AMIGO3	-5.0303051	2.7947E-22	1.5945E-20
UTS2B	-0.7593316	9.655E-23	5.7113E-21
TMEM215	-3.3452237	1.4174E-22	8.274E-21
IGDCC3	-1.4652922	1.0758E-22	6.3425E-21
VIL1	-4.1912251	1.583E-22	9.1796E-21
MLC1	-2.7478525	1.4055E-22	8.2316E-21
PRSS1	-2.0147584	1.375E-22	8.0794E-21
TFF3	-2.9199936	1.541E-22	8.9655E-21
WDR64	-2.5588664	2.0944E-22	1.2066E-20
UPK1A	-3.6480625	1.998E-22	1.1549E-20
SPHKAP	-0.7862745	2.6751E-22	1.5362E-20
AP000295.9	-3.0069386	3.7533E-22	2.1277E-20
CELA2A	-0.6986546	2.7095E-22	1.5509E-20
C1QTNF9B	-0.9763717	2.9566E-22	1.6815E-20
KRT2	-0.8065342	5.4664E-22	3.0791E-20
SCNN1G	-3.5913052	1.1111E-21	6.084E-20
SPRR2F	-1.1158381	6.4724E-22	3.6342E-20
SCGB2A2	-4.4965539	1.0057E-21	5.5762E-20
CHGB	-1.1462359	7.1726E-22	4.002E-20

PRSS57	-2.3880689	7.0878E-22	3.9672E-20
SOX2	-4.1964361	7.4198E-22	4.1269E-20
MYOC	-1.096268	1.0769E-21	5.9336E-20
CNDP1	-1.2677804	1.1282E-21	6.1586E-20
TDRD12	-1.2049419	1.1092E-21	6.084E-20
RIPPLY2	-1.4103313	1.0481E-21	5.793E-20
PLAC1	-2.0038575	1.2716E-21	6.92E-20
ALX1	-1.8831581	1.357E-21	7.3622E-20
AL928654.7	-2.7675541	1.7333E-21	9.375E-20
DEFB126	2.74547303	1.7482E-21	9.4268E-20
SERPINA6	-4.2242437	1.8078E-21	9.7186E-20
RP11-108K14.8	-3.3086798	3.1272E-21	1.666E-19
RP11-565P22.6	-2.743952	2.7373E-21	1.4671E-19
PENK	-1.1196935	2.774E-21	1.4823E-19
KLHL38	-1.0822261	3.7777E-21	2.0065E-19
RP11-745O10.4	-6.3652747	4.4432E-21	2.3529E-19
GOLGA6B	-3.183563	7.9864E-21	4.1916E-19
FAM133A	-1.4452577	5.1366E-21	2.7119E-19
RP11-195F19.29	-5.9182816	8.6826E-21	4.5167E-19
BEND4	-1.5416009	8.1202E-21	4.2468E-19
GAGE10	-2.4934625	8.1397E-21	4.2468E-19
CITED1	-1.0168638	1.0158E-20	5.269E-19
AZU1	-1.1097196	1.0574E-20	5.4686E-19
PTPRQ	-2.81262	1.0952E-20	5.6476E-19
RSPH10B2	-2.699069	1.2963E-20	6.665E-19
HDC	-2.0832256	1.446E-20	7.4134E-19
ATP1A4	-1.8056378	1.6629E-20	8.5003E-19
FGF17	-1.5898232	1.9344E-20	9.86E-19
RP11-500M8.7	-0.6563719	2.0193E-20	1.0263E-18
GRIA1	-0.7362975	2.3474E-20	1.1896E-18
SLC12A1	-1.5493378	2.7745E-20	1.4021E-18
OPCML	-2.0349689	3.5454E-20	1.7865E-18
CD8B	-1.6085775	3.5659E-20	1.7918E-18

CTC-429P9.4	-2.4220109	5.6179E-20	2.8148E-18
RP4-559A3.7	-4.6320721	5.5771E-21	2.9358E-19
GPR22	-3.2310043	9.9133E-20	4.8977E-18
CALB1	-1.1672729	6.0776E-20	3.0366E-18
GPC5	-1.5877874	6.7392E-20	3.3576E-18
RP11-123K3.4	-3.1560298	1.3901E-19	6.7733E-18
MYL4	-0.8540846	6.826E-20	3.3913E-18
CTD-2207O23.12	-2.655895	1.0471E-19	5.1447E-18
TFF2	-3.4009844	1.0783E-19	5.2684E-18
UPK2	-3.4804048	1.0319E-19	5.0841E-18
PTPRR	-1.9155765	9.2569E-20	4.5862E-18
IL1RAPL1	-3.1666676	1.0648E-19	5.2168E-18
CYSLTR1	-1.324305	1.4319E-19	6.9578E-18
APOBEC2	-1.2939922	1.5317E-19	7.4225E-18
KDM4E	-2.181178	2.0601E-19	9.9016E-18
KCNE5	-1.0327062	1.916E-19	9.2592E-18
BLID	-1.6954841	2.3653E-19	1.1337E-17
MYL2	-0.6154393	1.957E-19	9.4315E-18
S100P	-1.490418	3.7111E-19	1.7692E-17
SLC28A2	-1.2187841	3.5402E-19	1.6923E-17
RP11-1021N1.1	-5.3099661	1.0547E-18	4.9089E-17
FFAR2	-2.9871756	4.8856E-19	2.3166E-17
TPSAB1	-1.8342042	3.9076E-19	1.8579E-17
FAM217A	-4.08791	1.3664E-18	6.2612E-17
RP4-613B23.5	-3.54514	1.4186E-18	6.4835E-17
CTD-2410N18.5	-3.4654817	1.0714E-18	4.9606E-17
ADCY10	-1.7789595	5.7541E-19	2.7212E-17
MANSC4	-1.4529009	6.517E-19	3.0737E-17
TNFSF8	-1.4010281	7.9711E-19	3.7397E-17
FFAR3	-1.0041438	7.9699E-19	3.7397E-17
DDC	-1.0229565	8.7961E-19	4.1158E-17
TAS2R3	-1.7207711	1.0477E-18	4.8892E-17

EEF1A2	-1.5389062	1.0594E-18	4.918E-17
STPG2	-1.8935844	1.1228E-18	5.1849E-17
TUBB2B	-2.6984707	1.1428E-18	5.2638E-17
RP11-571M6.15	-0.7182546	1.2759E-18	5.8616E-17
CABP7	-1.6970442	1.4461E-18	6.5921E-17
TAS2R43	-2.3520572	2.581E-18	1.1735E-16
RBP2	-3.0007565	2.7965E-18	1.265E-16
USH1C	-2.9621758	2.6491E-18	1.2014E-16
GRIA4	-0.9929094	2.9852E-18	1.3469E-16
RP5-874C20.8	-2.0251976	3.6191E-18	1.6246E-16
AC003006.7	-2.2257659	3.5864E-18	1.6141E-16
CXCR2	-2.1845222	3.7507E-18	1.6795E-16
PPEF2	-3.6560727	9.0933E-18	3.9909E-16
OLAH	-1.8176407	4.3426E-18	1.9396E-16
LY6D	-1.3104185	5.0064E-18	2.2305E-16
CLEC18C	-3.3200799	5.6889E-18	2.5281E-16
GGTLC3	-3.9021085	1.6589E-17	7.0678E-16
OR52B6	-2.4186159	6.2868E-18	2.7868E-16
FBXW10	-1.4771319	6.7261E-18	2.9667E-16
SLC6A13	-1.5807161	6.437E-18	2.8463E-16
ARPP21	-3.0476058	1.2456E-17	5.3865E-16
RORB	-1.5435451	7.4554E-18	3.2802E-16
CDCP2	-3.6359733	2.2824E-17	9.5434E-16
GRID2IP	-0.9359491	9.4162E-18	4.1224E-16
MYBPC2	-1.8078426	9.5622E-18	4.176E-16
LTF	-2.7138907	9.9253E-18	4.3239E-16
IQCF1	-2.7937614	1.3184E-17	5.6738E-16
GSG1	-2.8157831	1.7648E-17	7.4673E-16
CYP2W1	-1.1387524	1.2253E-17	5.3118E-16
RP11-514P8.7	-0.6463334	1.1484E-17	4.9907E-16
CDRT1	-0.8233984	1.2581E-17	5.4272E-16
SCRT1	-2.5334589	1.6762E-17	7.1096E-16
MSX2	-1.5352499	1.4233E-17	6.1103E-16

ACY3	-2.6252807	1.5363E-17	6.5794E-16
ISLR2	-1.2943037	1.5508E-17	6.6254E-16
THRSP	-2.9016924	1.7883E-17	7.549E-16
TAS2R46	-2.2402614	2.1545E-17	9.0301E-16
AC068533.7	-0.6693025	1.6624E-17	7.0678E-16
NMRK2	-0.761677	2.0073E-17	8.4532E-16
WSCD2	-1.2700691	2.075E-17	8.7176E-16
ETNPPL	-2.7198491	3.0487E-17	1.254E-15
SLC8A2	-1.1958141	2.4111E-17	1.0058E-15
GPR83	-1.705304	2.4951E-17	1.0384E-15
NPY1R	-1.0421993	2.9126E-17	1.2036E-15
PTCHD4	-1.1852572	2.8221E-17	1.1717E-15
HRH4	-3.1053302	5.7334E-17	2.3312E-15
GDF10	-1.4422147	2.8998E-17	1.2012E-15
GRM4	-1.3032243	3.9511E-17	1.6177E-15
OR13C2	-2.924249	9.6302E-17	3.8536E-15
PZP	-1.1253359	4.3715E-17	1.7857E-15
FGF3	3.33231985	8.0362E-17	3.2445E-15
GS1-114I9.3	-3.5144911	1.1043E-16	4.3793E-15
ZFR2	-1.744901	5.1676E-17	2.106E-15
TPSD1	-3.1138996	7.3901E-17	2.991E-15
CLEC18B	-1.222989	6.6405E-17	2.6938E-15
CELF3	-3.253096	8.3747E-17	3.3664E-15
TMEM236	-1.7658535	8.0531E-17	3.2445E-15
TAS2R30	-2.6345935	1.0318E-16	4.1009E-15
IRX6	-1.5043781	8.7096E-17	3.4931E-15
ANP32D	-2.4606049	1.1183E-16	4.4247E-15
GSTA2	-3.3720967	1.579E-16	6.1516E-15
PLA2G2A	-1.2791276	1.0159E-16	4.0468E-15
GSTA1	-1.9235914	9.7732E-17	3.902E-15
PRG2	-2.8540526	1.5351E-16	5.9937E-15
TNFRSF18	-1.8035363	1.1361E-16	4.4855E-15
POU5F2	-2.5623842	1.64E-16	6.3335E-15

HOXC10	-1.7029418	1.3203E-16	5.2008E-15
SLC26A7	-1.5607013	1.4028E-16	5.5013E-15
RET	-1.1811911	1.3605E-16	5.3475E-15
HMGCS2	-3.4595862	1.4558E-16	5.6965E-15
LHX1	2.69461176	1.6074E-16	6.2484E-15
LEFTY1	-1.1319122	1.6264E-16	6.2945E-15
CSF3	-1.1217055	1.6229E-16	6.2945E-15
TAS2R50	-2.2659426	1.981E-16	7.6336E-15
SERPINA12	-2.9726248	2.0068E-16	7.6829E-15
CYP3A4	-2.213696	1.9949E-16	7.6681E-15
FRMD1	-1.0040389	1.9986E-16	7.6681E-15
PRSS3	-0.913956	2.0138E-16	7.693E-15
MUC6	-3.4317295	2.6456E-16	1.0063E-14
B3GALT5	-1.1472331	2.6178E-16	9.9787E-15
GFRA3	-1.1740911	2.921E-16	1.1063E-14
POPDC3	-1.2758871	2.9013E-16	1.1012E-14
RP11-277P12.6	-3.3121969	6.1203E-16	2.2599E-14
CROCC2	-1.1051164	3.5402E-16	1.3322E-14
ANKRD30BL	-1.1732212	3.4097E-16	1.2886E-14
ELMOD1	-2.6203343	4.2098E-16	1.5675E-14
TMEM151A	-2.8348837	4.0832E-16	1.5236E-14
LGALS7B	-1.2000073	3.4757E-16	1.3107E-14
MYLK2	-1.8280945	3.8848E-16	1.4526E-14
RP11-449H3.3	-1.0751787	3.8629E-16	1.4475E-14
AGXT	-4.475507	8.2371E-16	3.0039E-14
FAM181A	2.0169486	3.8454E-16	1.444E-14
DPP6	-1.46427	4.6153E-16	1.7149E-14
AC005943.2	-4.0053982	2.1839E-15	7.6337E-14
SYT9	-0.9732347	5.3087E-16	1.9685E-14
GPR37L1	-2.157439	5.7219E-16	2.1172E-14
SLCO1C1	-2.6803889	7.9568E-16	2.9077E-14
NKAIN2	-1.4987351	6.3313E-16	2.333E-14
STMN2	-1.135195	6.7296E-16	2.4746E-14

ERICH3	-1.2651972	7.7172E-16	2.826E-14
NNAT	-0.8808666	7.7065E-16	2.826E-14
CAMK2N2	-2.4058462	9.0013E-16	3.2624E-14
RP11-1012A1.4	-3.8482548	1.5371E-15	5.4591E-14
CHIT1	-2.8803988	1.0047E-15	3.6192E-14
TNNT3	-1.1080148	9.3245E-16	3.3727E-14
TMC2	-0.6556739	9.9703E-16	3.5989E-14
AXDND1	-2.1166028	1.1201E-15	4.0267E-14
NANOG	-3.3324153	1.7184E-15	6.0642E-14
CHST13	-1.9424494	1.1607E-15	4.1642E-14
A4GNT	-1.9893635	1.4165E-15	5.0512E-14
TAF7L	-2.1419008	1.2616E-15	4.5171E-14
HNRNPCL1	-2.9879826	5.4876E-15	1.8176E-13
AP000350.10	-4.1203931	5.6756E-15	1.8728E-13
OR1L8	-2.4411463	1.8092E-15	6.3615E-14
KCNJ13	-0.8869864	1.403E-15	5.0132E-14
KIAA1210	-2.7202617	1.5977E-15	5.6629E-14
SP5	-1.9911204	1.5072E-15	5.3636E-14
ARL2-SNX15	-5.2625279	3.6182E-17	1.4848E-15
KCNJ16	-1.7722796	1.7212E-15	6.0642E-14
AC069368.3	-2.2527158	2.2652E-15	7.8866E-14
CCL13	-2.6796581	2.2603E-15	7.8851E-14
SPINK9	-2.5570864	6.0587E-15	1.9881E-13
RP11-315D16.2	-3.2433471	4.5103E-15	1.5109E-13
RP1-66C13.4	-3.8233122	5.9046E-15	1.9412E-13
DPYSL5	-4.0458397	2.362E-15	8.1917E-14
LRP1B	-2.2577122	2.1364E-15	7.4972E-14
CCL18	-2.9497062	2.6415E-15	9.143E-14
DNAI1	-2.374392	2.167E-15	7.5896E-14
KCNH4	-2.3894081	2.7109E-15	9.3648E-14
LBX2	-1.5832717	2.334E-15	8.1102E-14
CLDN34	-2.2603485	3.6116E-15	1.2284E-13
SIX2	-2.586859	3.2947E-15	1.1293E-13

PGPEP1L	-2.2960555	3.362E-15	1.1501E-13
GFAP	-1.1282323	3.136E-15	1.0791E-13
RXRG	-1.3662682	3.0613E-15	1.0555E-13
GPR52	-3.0206247	6.8405E-15	2.2405E-13
RP11-762I7.5	-1.8029079	3.3684E-15	1.1501E-13
AC003002.6	-1.0574333	3.2616E-15	1.1202E-13
TAT	-2.4960725	4.7683E-15	1.5912E-13
MORN5	-1.4493226	3.6443E-15	1.2372E-13
C9orf24	-2.1114969	3.5725E-15	1.2175E-13
FABP4	-1.5822045	3.8194E-15	1.2941E-13
ADTRP	-2.219233	4.11E-15	1.3873E-13
C11orf53	-1.2509643	4.0034E-15	1.3539E-13
NCCRP1	-2.0159408	4.2141E-15	1.4197E-13
LRRC3B	-1.2641591	4.3455E-15	1.4612E-13
SV2B	-2.696843	5.7228E-15	1.8849E-13
FAM183A	-2.2635257	4.4242E-15	1.4848E-13
TRPA1	-0.7751006	4.7056E-15	1.5733E-13
CAPN11	-1.9678368	5.1943E-15	1.7237E-13
SIM2	-1.1684613	4.8428E-15	1.6131E-13
LOXHD1	-1.1427982	4.892E-15	1.6264E-13
PIP	-3.4100503	7.9824E-15	2.6001E-13
SCGB2B2	-0.7305121	5.603E-15	1.8523E-13
MAGEC3	-2.7053581	7.8678E-15	2.5675E-13
IDI2	-3.1810436	2.2088E-14	6.8539E-13
PPIAL4C	-3.0175195	1.873E-14	5.8632E-13
RP11-49K24.6	-3.8499991	1.629E-14	5.1357E-13
KRT36	-2.4053218	9.9766E-15	3.2141E-13
RBFOX1	-1.3072309	7.4253E-15	2.4276E-13
WIF1	-4.0360191	1.1215E-14	3.5934E-13
PXT1	-2.4701086	1.3967E-14	4.4429E-13
CSRP3	-2.2443147	9.9486E-15	3.211E-13
AC002985.3	-3.2408502	1.6386E-15	5.7965E-14
ABCC11	-2.2937431	8.6831E-15	2.818E-13

GNG8	-2.3783159	1.0706E-14	3.4365E-13
CALY	-0.9451869	8.5986E-15	2.7957E-13
TSPEAR	-2.5107261	9.4249E-15	3.0475E-13
RGS7	-1.7016938	9.1229E-15	2.9553E-13
CC2D2B	-1.8435249	1.0403E-14	3.3453E-13
C2CD4C	-0.955018	1.2291E-14	3.9239E-13
SYN3	-0.9586244	1.1531E-14	3.6881E-13
MSMB	-3.9435851	2.1301E-14	6.6213E-13
CTD-2116N17.1	-2.9605033	2.6731E-14	8.2081E-13
PRSS37	-1.0842622	1.3757E-14	4.3841E-13
SLITRK5	-1.9924821	1.4999E-14	4.7542E-13
NLRP14	-2.4122697	1.8052E-14	5.671E-13
TCN1	-3.894389	3.0192E-14	9.2069E-13
HTR2A	-1.1859421	1.6336E-14	5.1412E-13
PSCA	-1.6803384	1.4261E-14	4.5286E-13
MYH7	-0.8083844	1.517E-14	4.7998E-13
PVALB	-2.4670072	1.8452E-14	5.7865E-13
ORM1	-1.2110479	1.6113E-14	5.0891E-13
FRG2B	-2.5478411	7.7706E-14	2.2568E-12
NKAIN4	-2.8296992	1.9324E-14	6.028E-13
P2RY4	-1.6337887	1.8877E-14	5.899E-13
DGKI	-2.0928953	2.0733E-14	6.4561E-13
KRT86	-2.1580458	2.3603E-14	7.273E-13
C10orf99	-3.1841868	2.7897E-14	8.526E-13
OR2A25	-4.3857472	5.2673E-14	1.5737E-12
CD207	-2.3092411	2.3126E-14	7.1635E-13
IMPG2	-2.3204501	2.322E-14	7.1801E-13
GAP43	-1.0366599	2.343E-14	7.2321E-13
CPLX2	-3.2999318	2.7625E-14	8.468E-13
CTC-454I21.3	-3.468853	6.6136E-14	1.9431E-12
MS4A12	-3.6651321	9.1161E-14	2.6175E-12
FOXD4L4	-2.7806753	5.1886E-14	1.5528E-12
ANKRD2	-1.4183016	2.6192E-14	8.0567E-13

MAGEA8	-1.1734363	2.7911E-14	8.526E-13
SMCP	-2.8029818	5.944E-14	1.7493E-12
TSHR	-1.1213633	3.3826E-14	1.0262E-12
DSC3	-1.5117731	3.3613E-14	1.0215E-12
PPP1R42	-2.1840088	3.5207E-14	1.0645E-12
PDIA2	-1.2276407	3.3612E-14	1.0215E-12
TAL2	-1.642721	3.6035E-14	1.0876E-12
KIF19	-1.152402	3.4857E-14	1.0557E-12
RP11-446E24.4	-2.3627301	5.4692E-14	1.6241E-12
FDCSP	-3.434953	7.0045E-14	2.0478E-12
C16orf89	-1.1354299	4.0238E-14	1.2124E-12
RP11-26J3.4	-2.6713344	8.6647E-14	2.5E-12
RP11-1035H13.3	-2.1749252	5.8376E-14	1.7208E-12
CAPN13	-2.43294	5.4727E-14	1.6241E-12
TDRD9	-1.2143504	4.5486E-14	1.3659E-12
HHATL	-0.9322421	4.4372E-14	1.3347E-12
PROK2	-3.2642116	5.3842E-14	1.6032E-12
ZCCHC12	-1.1258467	4.9042E-14	1.4702E-12
CALML3	-1.2526553	5.3493E-14	1.5955E-12
HMGA2	-1.6823683	5.6633E-14	1.675E-12
DQX1	-2.2104075	5.7363E-14	1.6938E-12
FCGR3B	-0.9603342	5.6177E-14	1.6643E-12
C1orf194	-2.7786266	6.795E-14	1.9931E-12
BNC1	-1.8387818	6.9519E-14	2.0357E-12
HS3ST2	-2.2031851	7.2711E-14	2.1222E-12
RNF103-CHMP3	-2.8737007	1.4745E-13	4.1928E-12
AGR2	-2.4583049	7.6717E-14	2.2354E-12
SLC38A4	-1.6911213	8.0103E-14	2.3226E-12
LRRC71	-1.0970148	7.7136E-14	2.2439E-12
NLRP11	-1.340548	8.0919E-14	2.3424E-12
TNFRSF17	-2.1557827	8.4111E-14	2.4308E-12

VNN1	-1.6018259	9.0618E-14	2.6061E-12
MYBPC3	-0.8540719	8.9581E-14	2.5805E-12
MEIOB	-2.214662	9.3079E-14	2.6682E-12
RAD51AP2	-2.4019498	1.135E-13	3.2431E-12
FAM3D	-2.5922574	1.3162E-13	3.7486E-12
SPINK13	-1.1187524	1.0595E-13	3.0322E-12
HBZ	-1.9852532	1.518E-13	4.3027E-12
RP11-468E2.6	-3.5392761	5.5769E-13	1.5152E-11
CFAP61	-1.8887852	1.2377E-13	3.5309E-12
P2RX2	-2.9508178	1.9001E-13	5.3515E-12
SLFN14	-2.7389714	4.2269E-13	1.1645E-11
GRM8	-0.9110408	1.6755E-13	4.7415E-12
CPLX3	-3.7571804	7.0658E-13	1.9051E-11
SLC25A31	-4.6501879	7.4411E-13	2.0002E-11
GZMB	-1.7499951	1.8737E-13	5.2854E-12
MSX1	-1.9583259	1.7386E-13	4.9122E-12
CCL20	-2.4063751	2.3367E-13	6.5393E-12
KIF25	-1.0986987	1.9594E-13	5.5095E-12
DLX6	-1.2704412	2.0247E-13	5.6841E-12
ONECUT2	-3.11508	2.6196E-13	7.2849E-12
CYTL1	-1.2327654	2.1134E-13	5.9239E-12
HOXA9	-1.1793398	2.543E-13	7.0943E-12
ATP1A3	-2.3715799	2.5966E-13	7.2324E-12
CETP	-0.8233163	2.4435E-13	6.8275E-12
RP11-20I23.3	-3.8866763	1.2881E-12	3.3852E-11
SLC5A11	-2.0365332	3.278E-13	9.1016E-12
SPACA3	-2.0891025	4.6167E-13	1.264E-11
CPA4	-2.1727839	3.4542E-13	9.5758E-12
POMC	-0.9509559	3.5675E-13	9.8746E-12
SERPINC1	-1.9190399	3.849E-13	1.0637E-11
CXCL5	-2.6876652	4.8853E-13	1.3334E-11
UBD	-2.2617096	4.3986E-13	1.208E-11
USP17L7	-2.2276728	6.1656E-13	1.67E-11

CLDN18	-0.5863581	4.3523E-13	1.1972E-11
HP	-2.0399361	4.1899E-13	1.1561E-11
CCL19	-1.363665	4.569E-13	1.2529E-11
ZACN	-0.8550895	4.7437E-13	1.2968E-11
ITIH1	-0.8088758	5.2343E-13	1.4243E-11
TDRD1	-0.8415412	5.6501E-13	1.5327E-11
ZPBP	-2.1037776	6.2646E-13	1.6942E-11
ALOX15	-2.5335688	6.472E-13	1.7476E-11
LAMB4	-2.6726605	8.8903E-13	2.3646E-11
GTSF1	-3.2053838	8.2475E-13	2.2069E-11
KRT81	-3.2293948	1.0615E-12	2.8064E-11
ASCL1	-1.51343	7.3819E-13	1.9873E-11
PTGER1	-2.4656764	8.055E-13	2.1619E-11
BCAN	-0.882831	8.0944E-13	2.1692E-11
RP11-447L10.1	-5.2124341	4.2199E-12	1.0483E-10
KANK4	-1.6831825	8.2601E-13	2.2069E-11
REG3G	-2.529889	9.7442E-13	2.58E-11
UTF1	-1.077007	8.5402E-13	2.2783E-11
OR2J3	-4.478104	1.1114E-12	2.9338E-11
EDN2	-1.9491285	9.3962E-13	2.4916E-11
CEBPE	-2.0875746	1.9055E-12	4.8914E-11
MMP13	2.03660979	1.1651E-12	3.0712E-11
ADARB2	-1.0189699	8.7625E-13	2.3341E-11
POU4F3	-2.8882713	1.7745E-12	4.5953E-11
CTSE	-2.7419705	1.2988E-12	3.4084E-11
SCG3	-3.243306	1.6491E-12	4.2893E-11
GPC3	-1.4675764	9.0218E-13	2.3959E-11
TMPRSS11F	-4.8657121	5.0048E-12	1.2294E-10
DUOXA2	-1.9858362	1.1806E-12	3.1074E-11
XDH	-2.4783155	1.7122E-12	4.4469E-11
TMEM190	-2.8794241	1.7956E-12	4.6363E-11
TOX3	-0.9048674	1.4096E-12	3.6936E-11
TACR1	-1.1474479	1.4675E-12	3.8339E-11

SMIM24	-1.9571112	1.4628E-12	3.8273E-11
TMPRSS6	-1.3373195	1.503E-12	3.9209E-11
CFHR3	-1.2138559	1.5537E-12	4.047E-11
C19orf67	-1.6959087	1.7744E-12	4.5953E-11
KCNQ3	-1.0646368	1.791E-12	4.6312E-11
OR51E2	-0.714702	1.8117E-12	4.6712E-11
REG3A	-0.8363192	1.8843E-12	4.8463E-11
MS4A8	-2.6390451	2.3443E-12	5.992E-11
CLEC12B	-2.4916253	4.3591E-12	1.0798E-10
PKHD1L1	-1.0550164	2.1337E-12	5.4614E-11
RP11-257K9.8	-3.6614003	7.2173E-12	1.7391E-10
AHSP	-2.2740646	4.3387E-12	1.0763E-10
LIPF	-0.6401444	2.0995E-12	5.3818E-11
LIPJ	-2.0572246	2.5313E-12	6.4419E-11
GHRHR	-2.5992333	6.5057E-12	1.5762E-10
XPNPEP2	-0.7503699	2.5855E-12	6.561E-11
RHOXF1	-1.0351633	2.4169E-12	6.1684E-11
SMIM9	-4.2053314	2.8479E-12	7.1754E-11
RP11-404P21.8	-2.9995171	1.0073E-11	2.3881E-10
FSD2	-2.3289115	3.9551E-12	9.8666E-11
HOXA10	-1.0318257	2.6132E-12	6.6217E-11
SDR42E2	-1.6109064	2.6208E-12	6.6219E-11
ROPN1L	-1.4524377	2.4955E-12	6.3598E-11
SLC6A2	-2.607705	3.8841E-12	9.703E-11
RNF148	-1.6758519	2.6179E-12	6.6219E-11
RP11-65B7.2	-2.2143762	4.7507E-12	1.1719E-10
RP11-190A12.7	-1.5771287	2.8137E-12	7.0994E-11
HSD17B2	-2.7023057	5.3527E-12	1.3113E-10
IL12RB2	-1.1598351	3.0387E-12	7.6452E-11
SNX31	-2.2518545	3.0706E-12	7.7146E-11
LDHAL6B	-1.9289355	4.8046E-12	1.1836E-10
ACOT6	-1.2479342	3.1828E-12	7.9772E-11
FGFBP2	-1.2282065	3.2279E-12	8.0752E-11

FAM205A	-3.5090871	8.2729E-12	1.98E-10
TAS1R3	-0.8831249	3.9829E-12	9.9218E-11
TM4SF20	-4.9078302	1.4809E-11	3.4556E-10
HOXA5	-1.3363195	4.0858E-12	1.0164E-10
BCAS1	-1.0953147	4.466E-12	1.1048E-10
MCHR1	-0.6878075	4.5959E-12	1.1353E-10
OR52H1	-2.093916	2.0354E-11	4.6813E-10
MCOLN2	-0.8498166	4.8218E-12	1.1861E-10
AC104534.3	-4.1875218	5.1738E-13	1.41E-11
LDHAL6A	-1.0137075	5.2898E-12	1.2977E-10
PRH2	-1.9507193	7.8658E-12	1.8903E-10
KRT4	-1.1651706	5.5481E-12	1.3573E-10
HIGD1C	-2.5345293	1.2254E-11	2.8745E-10
SLC25A2	-1.3738292	6.2015E-12	1.5067E-10
ZG16B	-1.3499265	5.7096E-12	1.3948E-10
S100Z	-2.3052314	9.6055E-12	2.2866E-10
IZUMO1R	-2.3622093	8.5828E-12	2.0514E-10
OR52I1	-2.5536096	2.3063E-11	5.2635E-10
CXCR1	-1.9640795	6.9353E-12	1.678E-10
CH17-140K24.8	-4.8851806	3.3959E-11	7.603E-10
COL9A3	-1.0882693	6.1766E-12	1.5027E-10
PRAP1	-1.1115999	6.0853E-12	1.4825E-10
OBP2B	-2.0204503	7.2025E-12	1.7379E-10
BEX1	-1.5485842	5.9508E-12	1.4517E-10
RP11-540D14.8	-3.8958887	3.1392E-11	7.0819E-10
FAM163A	-2.0735818	7.5653E-12	1.8205E-10
IGFBPL1	-2.966119	1.0978E-11	2.5924E-10
HTR1F	-1.6118248	6.9931E-12	1.6897E-10
TFPI2	-1.240894	6.4411E-12	1.5627E-10
NRAP	-2.3481035	8.104E-12	1.9422E-10
C3orf22	-2.1073642	1.1008E-11	2.5961E-10
ABRA	-2.7489555	1.894E-11	4.3848E-10
NPIPB15	-0.7512885	8.0093E-12	1.9221E-10

DLX5	-1.1811861	8.8961E-12	2.1234E-10
TDO2	-1.9853274	9.4233E-12	2.2462E-10
AC104532.2	-2.2416669	1.5022E-11	3.5006E-10
ITLN1	-0.9903874	1.0017E-11	2.378E-10
LBP	-2.3420774	1.1435E-11	2.6895E-10
CTD-2370N5.3	-2.4645685	4.8928E-11	1.079E-09
AVPR2	-1.1056121	9.7757E-12	2.324E-10
MCEMP1	-1.8642624	1.0617E-11	2.5104E-10
SCGB3A1	-2.1484316	1.0243E-11	2.4254E-10
TBC1D3H	-5.8246734	4.2287E-11	9.4079E-10
C12orf71	-1.8813274	1.3425E-11	3.1368E-10
SOSTDC1	-1.9291533	1.1111E-11	2.6168E-10
MTRNR2L12	-1.0419331	1.2703E-11	2.9719E-10
CCL23	-1.4961024	1.1584E-11	2.721E-10
CNBD1	-2.8246613	4.7426E-11	1.0472E-09
OR2AE1	-2.4231086	4.7391E-11	1.0472E-09
WDR38	-2.6529368	1.6647E-11	3.869E-10
KLRC4	-2.216102	2.0457E-11	4.6989E-10
AL021546.6	-4.4964932	7.0155E-11	1.5225E-09
ATP4A	-2.0217979	1.7287E-11	4.0126E-10
C10orf90	-2.6396423	3.8581E-11	8.6159E-10
CCL24	-2.0719109	1.771E-11	4.1054E-10
TBX15	-0.7429215	1.6465E-11	3.8319E-10
FAM43B	-1.6395177	1.9358E-11	4.4757E-10
NPAS4	-0.9680138	2.0248E-11	4.6631E-10
COCH	-0.8278126	1.9918E-11	4.599E-10
FCER1A	-2.2028729	2.3061E-11	5.2635E-10
RNF151	-2.3183699	4.2979E-11	9.5498E-10
GREM1	-2.3371037	2.1192E-11	4.8615E-10
TMEM61	-1.9530164	2.7652E-11	6.2702E-10
TP53TG3	-4.1822808	1.0032E-10	2.143E-09
SPOCK3	-0.8386733	2.3028E-11	5.2635E-10
RP11-613M10.8	-3.712221	9.447E-11	2.0253E-09

TEDDM1	-1.8674958	2.4057E-11	5.4761E-10
IL31RA	-2.4097469	3.2117E-11	7.227E-10
ALK	-1.6493395	2.3219E-11	5.2923E-10
MME	-0.8460444	2.437E-11	5.5403E-10
S100A7	-2.4185325	2.6296E-11	5.9704E-10
BCAT1	-1.8376177	2.2923E-11	5.2518E-10
BTLA	-2.071701	3.2911E-11	7.3776E-10
ZNF648	-1.958735	3.0152E-11	6.811E-10
POU6F2	-1.5804486	2.9627E-11	6.7008E-10
KCNE1	-1.2272755	2.944E-11	6.6671E-10
FBN2	-1.1882664	3.1576E-11	7.1144E-10
TGFBR3L	-1.7514853	3.2659E-11	7.3399E-10
L34079.2	-3.1015358	7.9806E-11	1.7277E-09
SPX	-1.4376692	3.2894E-11	7.3776E-10
GNG3	-0.6021217	3.4755E-11	7.7712E-10
TBC1D3K	-6.3972601	6.0306E-11	1.3184E-09
TBC1D28	-4.3442945	1.7959E-10	3.7298E-09
PTTG2	-1.8472854	4.4269E-11	9.8119E-10
SLITRK2	-0.9998327	4.2109E-11	9.3801E-10
VPREB3	-2.0857114	5.3941E-11	1.1866E-09
UNC79	-1.4513988	4.3407E-11	9.6328E-10
DGAT2L6	-4.3107945	1.7643E-10	3.6742E-09
KLK1	-1.0395546	4.0152E-11	8.9554E-10
NOG	-1.8087408	5.2669E-11	1.1601E-09
CFAP57	-0.9412086	4.6041E-11	1.0192E-09
C2orf72	-1.948134	5.7826E-11	1.2674E-09
LCE5A	-2.1707569	1.1929E-10	2.5329E-09
DDI1	-2.1421637	8.6457E-11	1.8603E-09
TENM2	-0.7001081	5.7697E-11	1.2661E-09
NRTN	-1.6558933	5.7209E-11	1.257E-09
ANTXRL	-2.3759055	9.9704E-11	2.1349E-09
USP17L1	-4.4138606	2.814E-10	5.7605E-09
SCT	-1.7789678	6.4877E-11	1.4132E-09

TAC1	-2.7758607	7.8552E-11	1.7026E-09
DPEP1	-0.8052091	6.3092E-11	1.3777E-09
LRCOL1	-0.9497116	5.9555E-11	1.3036E-09
MEOX2	-0.6802062	6.6816E-11	1.4536E-09
CTD-2616J11.11	-1.483317	6.902E-11	1.4997E-09
SERPINA5	-2.0458762	6.3928E-11	1.3942E-09
CARTPT	-3.4328201	1.467E-10	3.0926E-09
RBMXL2	-2.2746903	1.457E-10	3.0752E-09
PKD1L3	-1.7987017	8.5215E-11	1.8358E-09
KLRC3	-3.3301559	3.1501E-10	6.3894E-09
CD164L2	-2.1939036	9.9974E-11	2.1381E-09
MMP1	-2.5568226	1.0714E-10	2.2859E-09
CCDC169-SOHLH2	-2.9216597	4.4287E-10	8.8111E-09
PMCH	-2.6617558	1.816E-10	3.7655E-09
TAS2R13	-1.9991398	1.0792E-10	2.297E-09
OCM	-1.4207707	8.2095E-11	1.7729E-09
JAKMIP2	-1.2371014	8.3937E-11	1.8105E-09
PRSS2	-1.6635195	8.0463E-11	1.7398E-09
PROP1	-2.4680278	3.4672E-10	6.9528E-09
DCC	-2.150656	1.3241E-10	2.8047E-09
JPH3	-1.1711799	9.4147E-11	2.0208E-09
TNNI1	-0.9084752	9.3335E-11	2.0059E-09
RNASE3	-2.1082797	1.2245E-10	2.5968E-09
GP9	-1.9792183	2.8385E-10	5.804E-09
BHLHA15	-1.6448888	1.1584E-10	2.4625E-09
XXcos-LUCA11.5	-4.0993538	7.1129E-10	1.3795E-08
GLRA2	-3.002623	1.8834E-10	3.8961E-09
CCL21	-1.3842639	1.0785E-10	2.297E-09
CFAP74	-2.308359	1.4187E-10	2.9978E-09
PLEKHS1	-2.1550584	1.4793E-10	3.1148E-09

PNPLA1	-2.029518	1.7658E-10	3.6742E-09
RP11-248J23.7	-2.8530551	7.0206E-10	1.363E-08
PNMT	-0.9887467	1.3737E-10	2.9062E-09
NTSR1	-2.1973319	1.9221E-10	3.9714E-09
FOXH1	-1.9494132	1.4989E-10	3.1523E-09
NOTUM	-3.202053	2.3772E-10	4.8946E-09
CCDC38	-2.2994336	3.2553E-10	6.5803E-09
DHH	-1.0463355	1.581E-10	3.3132E-09
GSTM1	-2.0574738	1.7967E-10	3.7298E-09
AIM2	-1.5871668	1.5448E-10	3.2449E-09
CERS3	-0.658189	1.6158E-10	3.3781E-09
CRYM	-0.8866984	1.5796E-10	3.3132E-09
FRG2	-6.854384	3.4274E-10	6.8809E-09
CMTM5	-0.690774	1.6135E-10	3.3773E-09
EFCAB5	-2.5949511	3.335E-10	6.7105E-09
OVGP1	-2.6375774	1.8519E-10	3.8355E-09
ZMAT4	-0.7093713	1.7256E-10	3.599E-09
C1orf141	-2.5249239	2.5228E-10	5.1884E-09
ZC2HC1B	-5.7518113	8.3943E-10	1.6138E-08
C8A	4.22733733	4.8795E-10	9.6644E-09
OR13C5	-2.5484264	8.4707E-10	1.6267E-08
RP11-613M10.9	-2.1089752	1.0681E-09	2.0314E-08
HSD3B2	-0.7295698	2.0813E-10	4.2955E-09
SPINK2	-1.9112328	2.6132E-10	5.3619E-09
MUC19	-2.5209281	1.2014E-09	2.2701E-08
PRR5-ARHGAP8	-1.812541	3.2667E-10	6.5956E-09
RP5-864K19.6	-1.562671	2.5257E-10	5.1884E-09
C1QL1	-1.5763071	2.2607E-10	4.6603E-09
KHDC1L	-2.6587245	3.311E-10	6.6775E-09
KBTBD13	-2.0391595	5.1127E-10	1.0104E-08
PDC	-2.2900314	4.8519E-10	9.6206E-09
GZMH	-1.4641182	2.6729E-10	5.4781E-09

CPNE6	-2.3596946	3.6308E-10	7.2645E-09
KLRC2	-1.7483342	3.0482E-10	6.2112E-09
RCVRN	-1.4166719	2.9235E-10	5.971E-09
IMPG1	-1.2698896	2.955E-10	6.0283E-09
GDPD2	-1.898616	3.1375E-10	6.3784E-09
CHST4	-1.9152215	3.387E-10	6.8076E-09
MPP4	-0.7518045	3.1681E-10	6.4187E-09
ZP1	-2.241533	5.2903E-10	1.0443E-08
KRT14	-1.463619	3.1123E-10	6.3345E-09
PROM1	-2.1650578	3.3187E-10	6.6854E-09
CLCA2	-2.41516	6.1251E-10	1.201E-08
SUCNR1	-1.9739916	4.3661E-10	8.6964E-09
VHLL	-2.2392627	2.1095E-09	3.8149E-08
AADAC	-1.4925562	3.5855E-10	7.182E-09
IL2	-3.024579	2.5509E-09	4.5852E-08
MOG	-2.5616704	8.168E-10	1.5737E-08
C1QL4	-2.333445	5.4643E-10	1.0774E-08
HPGD	-1.7216102	3.9928E-10	7.9707E-09
CLDN20	-1.0707643	4.0583E-10	8.0924E-09
IZUMO1	-1.6279447	4.7651E-10	9.4696E-09
SIRPD	-4.0001876	1.9648E-09	3.5715E-08
CTD-2583A14.9	-2.4163544	8.8979E-10	1.7032E-08
SALL1	-0.7435473	4.8147E-10	9.5576E-09
SEMA5B	-1.1431692	5.0287E-10	9.9489E-09
TMC3	-2.2421915	6.9489E-10	1.3523E-08
HLA-G	-1.0367642	5.5563E-10	1.0944E-08
IL25	-2.4321092	1.3181E-09	2.4722E-08
SLC51B	-0.7314161	5.853E-10	1.1503E-08
HOXA6	-2.046013	6.1683E-10	1.2082E-08
BTNL2	-0.9005923	5.6709E-10	1.1157E-08
LMO1	-1.8545949	6.8632E-10	1.3398E-08
CALML5	-2.3449212	6.958E-10	1.3524E-08
LYG2	-2.363985	9.6392E-10	1.8372E-08

IRX5	-1.3449764	6.2392E-10	1.2207E-08
IRGM	-2.4686156	1.3094E-09	2.4586E-08
TNS4	-1.3389325	6.3788E-10	1.2467E-08
CXCL6	-2.0390198	7.7501E-10	1.4965E-08
RARRES1	-1.4604153	6.0731E-10	1.1922E-08
RP11-544M22.13	-1.5750698	7.2435E-10	1.4032E-08
CXCL11	-1.8393211	8.9561E-10	1.7125E-08
ANKRD55	-1.792199	9.1737E-10	1.7522E-08
FOXI3	1.62722413	7.5533E-10	1.4616E-08
PKD2L1	-1.9293781	1.0886E-09	2.0682E-08
SPATA45	-1.7592731	1.3693E-09	2.5629E-08
S100A8	-1.3377854	6.9295E-10	1.3513E-08
TBC1D3	-3.8815075	4.8264E-09	8.2878E-08
C9orf153	-1.8631472	1.1176E-09	2.1209E-08
FOXD2	-1.1491668	7.7221E-10	1.4927E-08
NECAB2	-0.6669836	8.0024E-10	1.5435E-08
GABRP	-1.7772328	8.3695E-10	1.6108E-08
GYS2	-1.3147327	8.617E-10	1.6512E-08
IQCJ	-3.1096891	4.3723E-09	7.5743E-08
DUX4	-5.1925889	2.7917E-09	4.9977E-08
HSPB3	-0.619608	8.5469E-10	1.6396E-08
S100A5	-2.4938356	1.2725E-09	2.3943E-08
MEIKIN	-3.5662802	4.54E-09	7.8534E-08
SIGLEC5	-1.6135631	1.1748E-09	2.2224E-08
DLL3	-1.6716224	9.3561E-10	1.7851E-08
RFPL4B	3.05701819	4.6352E-09	7.9906E-08
WFDC10B	-2.2036297	1.3843E-09	2.5855E-08
ZAR1L	-4.8113753	3.9359E-09	6.8586E-08
TMCO2	-3.6270764	5.2246E-09	8.9368E-08
FNDC8	-1.8988116	1.5912E-09	2.9531E-08
STARD6	-2.652101	3.4441E-09	6.0674E-08
CTB-54O9.9	-2.0815724	1.6561E-09	3.0544E-08

TSHB	-2.386749	4.052E-09	7.0539E-08
OR2K2	-2.4118784	6.5606E-09	1.1009E-07
MLIP	-1.7150802	1.3536E-09	2.5362E-08
HPD	-0.636443	1.1262E-09	2.135E-08
PLA2G1B	-0.5942263	1.1586E-09	2.194E-08
TREH	-1.8821414	1.388E-09	2.5895E-08
BARX1	-1.8521078	1.3836E-09	2.5855E-08
NGFR	-0.9282232	1.2937E-09	2.4316E-08
TSACC	-1.0664637	1.2076E-09	2.2794E-08
C14orf39	-0.7344802	1.2326E-09	2.3241E-08
AGR3	-2.5083109	2.0046E-09	3.6365E-08
PCP2	-1.5836901	1.4351E-09	2.6719E-08
POU5F1B	-1.5407937	1.3975E-09	2.6046E-08
AKR1B15	-2.3093147	2.9718E-09	5.2721E-08
FRZB	-1.0270752	1.2672E-09	2.387E-08
GFRA2	-0.750144	1.4651E-09	2.7249E-08
KCNF1	-1.9436587	1.7429E-09	3.1878E-08
OR51I1	-3.6474587	6.7627E-09	1.1327E-07
TLL1	-0.7914418	1.5041E-09	2.7945E-08
SCGB1A1	-2.497041	2.0968E-09	3.7959E-08
FOXA2	-2.3347024	2.1518E-09	3.8875E-08
KRT32	-2.596559	2.8579E-09	5.0997E-08
LGALS2	-1.6581095	1.8089E-09	3.2983E-08
DAND5	-2.4515327	2.916E-09	5.1782E-08
ISM2	-1.8778117	1.6848E-09	3.1009E-08
PBK	-1.127842	1.6908E-09	3.1087E-08
FOXD1	-3.0178179	2.0089E-09	3.6405E-08
CDHR1	-0.948144	1.7134E-09	3.1437E-08
SYT8	-1.9329765	1.739E-09	3.1839E-08
VSX1	-0.6213093	1.6464E-09	3.0397E-08
KCNK4	-3.1855793	4.5935E-09	7.9341E-08
GCNT3	-1.8579794	1.8478E-09	3.3657E-08
CX3CR1	-1.3175286	1.6806E-09	3.0964E-08

ITGB6	-1.1431755	1.7169E-09	3.1469E-08
C8orf89	-0.911764	1.7092E-09	3.1392E-08
HOXA7	-0.8307484	1.8071E-09	3.2983E-08
CACNA1B	-1.5530394	1.9467E-09	3.5424E-08
CFAP126	1.18977357	5.6607E-09	9.6085E-08
KLK13	-1.3153343	1.7983E-09	3.2858E-08
SV2C	-0.8952048	2.0014E-09	3.6344E-08
OR13D1	-3.4573624	4.315E-09	7.4823E-08
CCNB3	-1.2963725	2.1695E-09	3.9155E-08
CTD-2528L19.4	-5.0272077	6.9501E-10	1.3523E-08
IFNA21	-3.0756683	4.6122E-09	7.9587E-08
GNRHR	-1.1484584	2.3621E-09	4.2588E-08
KLF17	-2.7037648	1.0342E-08	1.684E-07
CCL25	-2.2087153	3.4938E-09	6.1365E-08
CHRNA3	-1.766937	3.1149E-09	5.5094E-08
CTXN3	4.42551999	8.7931E-09	1.4535E-07
TTLL13P	-0.8476461	2.5569E-09	4.5913E-08
NEFH	-0.9742814	2.4831E-09	4.4724E-08
HTR1B	-1.2603541	2.7612E-09	4.9481E-08
MOXD1	-1.0760728	2.8362E-09	5.0722E-08
RP11-371E8.4	-3.8700465	2.1321E-08	3.2849E-07
PDE6G	-2.1482778	3.2067E-09	5.6603E-08
CCDC182	-2.6906041	1.3626E-08	2.179E-07
MYEOV	-1.0707486	2.7229E-09	4.8843E-08
MZB1	-1.3210402	2.8797E-09	5.1292E-08
ANKRD1	-0.7704965	2.8603E-09	5.0997E-08
NPPC	-0.8672481	2.8436E-09	5.0803E-08
ARG1	-0.7065783	2.8896E-09	5.1418E-08
KCNIP1	-0.9831423	2.9087E-09	5.1705E-08
BEND2	4.1558187	1.0963E-08	1.7773E-07
TMEM88B	-1.8509631	3.8875E-09	6.7809E-08
CAV3	-2.0016808	4.9833E-09	8.5407E-08
RP11-178C3.1	-2.7765082	1.3519E-08	2.1639E-07

RBP4	-0.9040447	3.1486E-09	5.5633E-08
IL4	-2.333492	1.475E-08	2.3398E-07
CPNE4	-2.1384485	3.7056E-09	6.4893E-08
VSIG2	-1.7538918	2.9845E-09	5.2893E-08
BLK	-2.1480748	4.0654E-09	7.0703E-08
HOXA2	-0.8496867	3.488E-09	6.1324E-08
ST6GAL2	-1.0138742	3.3895E-09	5.9771E-08
DYDC2	-1.523594	3.6126E-09	6.3388E-08
SLC26A9	-1.9970515	4.1711E-09	7.247E-08
FNDC9	-2.9034377	1.7264E-08	2.7118E-07
C9orf131	-1.4827794	3.7902E-09	6.6243E-08
MUC3A	-2.1961852	4.178E-09	7.2519E-08
CPXM2	-1.060918	3.4621E-09	6.093E-08
C4orf45	-1.865222	5.9963E-09	1.011E-07
SFTPA2	-0.5895763	3.66E-09	6.4157E-08
CCDC85A	-1.6906255	3.8848E-09	6.7809E-08
C2CD4B	-1.3168664	3.7813E-09	6.6152E-08
CRYAA	-5.062427	2.2461E-08	3.4454E-07
RHOXF2	-2.6272057	9.7645E-09	1.5962E-07
SETSIP	-1.4809331	6.3731E-09	1.0715E-07
ADGRF1	-1.9447834	5.5456E-09	9.4313E-08
PGAM4	-1.3204373	4.8845E-09	8.3795E-08
UPK1B	-1.9276186	5.2938E-09	9.0378E-08
TAS2R42	-3.1263581	2.2503E-08	3.446E-07
CTNNA2	-1.5878179	5.0665E-09	8.6749E-08
TMC1	-1.5564492	4.6803E-09	8.0525E-08
SCN11A	-1.5937996	4.7827E-09	8.2208E-08
ERVH48-1	-0.9232811	4.5423E-09	7.8534E-08
TRIM75P	-2.4305031	1.1302E-08	1.8254E-07
OR52W1	-2.8344904	2.3755E-08	3.622E-07
RNF165	-1.622817	5.3574E-09	9.1376E-08
SLC10A4	-0.9244438	4.6749E-09	8.0511E-08
C7orf57	-1.6461335	5.6979E-09	9.6358E-08

REC114	-3.2538726	0.000000019	2.9713E-07
LCNL1	-0.8395737	5.2698E-09	9.0055E-08
MATN4	-2.5509835	9.9468E-09	1.6215E-07
AMELX	3.32571704	1.6408E-09	3.0357E-08
TBR1	2.84255644	3.108E-08	4.6311E-07
KERA	-1.9183047	7.4907E-09	1.2452E-07
ACKR2	-1.1582119	5.4898E-09	9.3453E-08
ODF3	-2.4086688	9.6052E-09	1.5745E-07
CDRT15	-1.4725001	6.5994E-09	1.1064E-07
IL17B	-0.7202801	5.4318E-09	9.2555E-08
DLGAP1	-0.6572181	5.6894E-09	9.6358E-08
ARR3	-1.7473102	8.8493E-09	0.000000146
OR56B4	-1.8233011	1.4933E-08	2.3603E-07
VAV3	-1.4267357	5.704E-09	9.6358E-08
LRRC43	-0.7485338	5.5807E-09	9.4817E-08
MESP2	-1.7226007	7.3674E-09	1.2293E-07
FXYD7	-1.0423404	5.6996E-09	9.6358E-08
PCDHA8	-1.4161803	7.4846E-09	1.2452E-07
KLRG2	-2.1851983	8.6833E-09	1.4367E-07
CHRDL2	-0.6041063	6.0057E-09	1.0117E-07
WNT11	-1.4791341	5.6676E-09	9.6111E-08
RAET1E	-1.2497959	6.1269E-09	1.0311E-07
NLRP7	-2.4709019	9.5247E-09	1.5627E-07
SLC47A2	-1.571175	6.3946E-09	1.0741E-07
TSPAN16	-2.6818736	2.7124E-08	4.0794E-07
LRRC3C	-1.8545923	1.0488E-08	1.7033E-07
KLHDC7B	-1.6795439	7.0468E-09	1.1791E-07
SPEM1	-1.516827	2.1004E-08	3.2417E-07
CA5A	-2.0494993	2.2268E-08	3.4189E-07
GOLT1A	-1.1464941	7.0726E-09	1.1812E-07
CTD-2207O23.10	-3.7249203	2.9961E-09	5.3045E-08
FGFBP1	-1.5522564	7.0581E-09	1.1799E-07

FAIM2	-0.7722788	7.4637E-09	1.243E-07
CTAGE6	-2.5840996	2.549E-08	3.8633E-07
OR2AG2	-3.6732427	5.0962E-08	7.3229E-07
TUBB1	-1.4403679	8.8404E-09	1.4599E-07
TMEM252	-0.919465	7.5857E-09	1.2598E-07
SEMA3E	-2.091002	9.761E-09	1.5962E-07
CCND2	-1.0418419	7.4162E-09	1.2363E-07
KCNA2	-1.3316463	8.8884E-09	1.4651E-07
TNNC1	-1.231349	8.0065E-09	1.3284E-07
HCRTR2	-1.9699626	1.4302E-08	2.2789E-07
POU3F1	-0.6479572	8.6292E-09	1.4291E-07
RAPSN	-1.9857966	9.8328E-09	1.6058E-07
HOXA4	-0.9836832	9.0893E-09	1.4955E-07
GABRR1	-1.6797438	1.4722E-08	2.3374E-07
TTC9B	-1.2938648	9.103E-09	1.4963E-07
OVCH1	-1.8519572	1.0959E-08	1.7773E-07
XAGE2	1.61923379	9.7594E-09	1.5962E-07
CTSK	-1.3531645	8.1806E-09	1.356E-07
TSPO2	-2.4770525	1.9295E-08	3.0067E-07
S100A3	-0.8225191	9.5107E-09	1.5619E-07
EPHX3	-1.3654584	9.0715E-09	1.4939E-07
HAO2	-1.7242577	1.2948E-08	0.000000208
ANO3	-1.8797855	1.1311E-08	1.8254E-07
RP11-322E11.6	-3.6043957	4.5642E-08	6.6115E-07
DLGAP2	-0.6125814	1.035E-08	1.684E-07
MUC5B	-2.0052672	1.1323E-08	1.8256E-07
PRSS33	1.81816744	1.1304E-08	1.8254E-07
TMEM89	-2.0848571	1.6703E-08	2.6284E-07
TMEFF2	-2.0251698	1.4914E-08	2.3603E-07
NXPE2	-1.7117297	1.2902E-08	2.0764E-07
ACBD7	-1.0546401	1.0374E-08	1.6865E-07
AQP9	-1.3341364	1.1208E-08	1.8153E-07
KISS1	-2.8023921	2.0388E-08	3.1577E-07

CHRM2	-2.1323647	2.1427E-08	3.2984E-07
C11orf91	-1.7528848	1.7933E-08	2.8119E-07
FGF14	-1.6353212	1.4256E-08	2.2736E-07
ENKUR	-0.8701361	1.1291E-08	1.8254E-07
OR13J1	-1.4229983	1.5239E-08	2.4065E-07
TPO	-0.9023807	1.1846E-08	1.9081E-07
OVOL3	-2.2735356	2.1944E-08	3.3749E-07
C17orf99	-1.6196024	1.4691E-08	2.3346E-07
SLC35F1	-1.0627552	1.2948E-08	0.000000208
CXCL13	-1.7467634	1.3017E-08	2.0893E-07
CDH16	-2.3935553	1.9915E-08	3.0951E-07
CYP7A1	-2.007756	3.3707E-08	4.9848E-07
WEE2	-1.4083788	1.4652E-08	2.3305E-07
DYNAP	-3.3894384	8.8465E-08	1.2148E-06
APOC4	-3.7658072	6.806E-08	9.5387E-07
LMO3	-0.9352499	1.4244E-08	2.2736E-07
ELOVL3	-1.4592099	1.3189E-08	2.113E-07
DGKB	-2.0834881	2.2044E-08	3.3875E-07
DKK1	-0.7175186	1.4619E-08	2.3273E-07
SFTA2	-3.2457403	2.8277E-08	4.2385E-07
ANPEP	-1.3068248	1.3065E-08	2.0951E-07
CALHM3	-1.6048984	1.415E-08	2.2608E-07
OR1E2	-3.5645752	3.554E-08	5.2514E-07
KRT40	3.777621	5.2007E-08	7.4542E-07
SLC22A7	-1.9429829	3.1785E-08	4.7281E-07
CST2	-3.8628439	3.6761E-08	5.4227E-07
NPW	-1.5000753	1.4827E-08	2.3498E-07
EPO	-0.9513045	1.4928E-08	2.3603E-07
SYT12	-1.4742307	1.5972E-08	0.000000252
XCL2	-1.7452534	1.8822E-08	2.9461E-07
LKAAEAR1	-1.9709419	2.2833E-08	3.4934E-07
CLCA1	-2.9658901	4.9532E-08	0.000000714
RASD1	-0.8381947	1.6126E-08	2.542E-07

NXPE4	-2.0719096	2.6675E-08	4.0222E-07
ODF3L2	-1.6521998	1.9179E-08	2.9913E-07
TNF	-1.7302384	1.8791E-08	2.9438E-07
LGI1	-2.505932	3.9267E-08	5.754E-07
PLCXD2	-1.5145964	1.9937E-08	3.096E-07
KLK15	-2.9822468	1.2143E-07	1.6246E-06
SERPINE1	-1.275969	1.7261E-08	2.7118E-07
PLET1	-1.8664049	7.9134E-08	1.0943E-06
S1PR5	-0.6153921	2.0302E-08	3.1471E-07
CAPN6	-1.0345995	1.9028E-08	2.9731E-07
C2orf50	-0.7683738	1.9086E-08	2.9794E-07
FLG	-0.7786217	2.0511E-08	3.1739E-07
INA	-1.9122829	2.352E-08	3.5925E-07
SFTPD	-0.9941301	1.9486E-08	3.0339E-07
DNER	-0.6265729	1.9826E-08	3.0842E-07
CHRM5	-1.303017	2.1296E-08	3.2839E-07
KCNS1	-1.9493408	2.703E-08	4.0688E-07
SBK2	-3.4423102	5.0091E-08	7.2147E-07
OMP	-1.7339778	2.9081E-08	4.3479E-07
COL11A1	-2.035457	2.0204E-08	3.1347E-07
C2orf73	-0.7931625	2.0541E-08	3.1758E-07
MAEL	-2.0662609	2.7982E-08	4.1977E-07
GKN2	-2.3554998	8.0594E-08	1.1128E-06
THBS2	-1.2468148	2.0608E-08	3.1834E-07
RNF175	-1.0997631	2.2489E-08	3.446E-07
C1orf189	-3.0871781	4.4246E-08	6.4304E-07
KREMEN2	-1.7234691	2.7878E-08	4.1858E-07
NKAIN1	-1.9413633	2.5636E-08	3.8821E-07
GPR179	-0.6870051	2.4148E-08	3.6747E-07
FOXA1	-2.2378663	2.731E-08	4.104E-07
CHST6	-1.5251933	2.6207E-08	3.9618E-07
GPR85	-1.0844856	2.4568E-08	3.7299E-07
CUX2	1.9937419	4.553E-08	6.6007E-07

KIRREL2	-2.2936213	4.1593E-08	6.0698E-07
KRT6A	-1.5694427	2.3429E-08	3.5816E-07
PTX4	-1.870226	4.2055E-08	6.1322E-07
NDUFA4L2	-1.057715	2.3682E-08	3.6139E-07
SLC10A6	-1.4044863	3.0555E-08	4.5567E-07
HLA-DQB2	-1.4005795	2.6031E-08	3.9384E-07
PAEP	-2.5891512	3.9887E-08	5.8352E-07
S100A2	-1.2064404	2.4163E-08	3.6747E-07
RP11-77K12.1	-3.4831196	1.7323E-07	2.2529E-06
RP11-212D19.4	-2.6288683	1.0199E-07	1.3823E-06
MFSD6L	-1.0496411	2.9644E-08	4.4283E-07
RGS20	-0.6054367	2.6434E-08	3.9893E-07
GIPR	-1.102946	2.8537E-08	4.2738E-07
NTM	-1.0643347	2.6729E-08	4.0268E-07
C3orf49	-1.3513142	3.2904E-08	4.8783E-07
OR52N5	-2.7706052	5.9449E-08	8.4118E-07
RGS13	-2.0209516	7.6023E-08	1.0571E-06
MFAP4	-1.343223	2.6421E-08	3.9893E-07
ALAS2	-1.4617358	2.8844E-08	4.3161E-07
F8A3	-1.0364069	3.0513E-08	4.5543E-07
ATP8A2	-0.8910598	3.1159E-08	4.639E-07
OR52D1	-2.4894963	8.7569E-08	1.2044E-06
FAM71C	-3.0615229	1.6382E-07	2.1448E-06
NR2E3	-1.8443196	3.6912E-08	5.4405E-07
PADI4	-2.1972591	4.2786E-08	6.2285E-07
RP11-697E2.12	-1.71724	4.8625E-08	7.0322E-07
RAB44	-2.2512383	5.1232E-08	7.3538E-07
RNASE2	-0.791225	3.2631E-08	4.8419E-07
SERPINB2	-1.8349007	3.6353E-08	5.3671E-07
TP53TG3D	-1.023202	3.3093E-08	4.9021E-07
S100A4	-1.0935925	3.2178E-08	4.7794E-07
PRR23C	4.45346006	1.5299E-07	2.0149E-06
C19orf81	-1.1043138	3.8797E-08	5.6898E-07

KCNK12	-1.268107	3.8214E-08	5.6184E-07
NRN1	-0.8315848	3.7342E-08	5.4948E-07
CCL17	-2.6161596	9.0969E-08	1.2453E-06
NTRK1	-1.4441689	3.8415E-08	5.6432E-07
EYS	-1.2713109	4.1465E-08	6.0561E-07
C9orf135	-2.3659605	6.1862E-08	8.7293E-07
SAA1	-1.4145346	3.8512E-08	5.6529E-07
FOXG1	-0.6460683	4.0321E-08	5.8939E-07
ASCL2	-1.2952166	3.9467E-08	5.7786E-07
COL17A1	-0.6677134	4.2902E-08	6.2402E-07
PGLYRP1	-1.3375833	5.4447E-08	7.7661E-07
ATOH1	1.96867265	3.4778E-07	4.2895E-06
CHRNA1	-1.8090614	6.028E-08	8.5158E-07
SLC10A1	-2.3482081	7.7483E-08	1.0732E-06
DEFA6	-3.1181805	1.8762E-07	2.4239E-06
C19orf73	-1.0228559	4.4963E-08	6.5239E-07
DAZL	4.01346029	2.5365E-07	3.1976E-06
GPRIN2	-1.1675553	4.9138E-08	7.0948E-07
AGXT2	-4.3005614	1.9918E-07	2.5603E-06
BBOX1	-1.5083057	5.0967E-08	7.3229E-07
RP11-101E3.5	-2.478556	5.4608E-08	7.7766E-07
PRSS55	-3.6354914	3.1539E-07	3.9091E-06
SFRP4	-1.1804351	4.2314E-08	6.1649E-07
ZNF80	-1.689715	6.189E-08	8.7293E-07
C17orf78	-1.7276153	6.7407E-08	9.4621E-07
FOLR3	-1.7665732	6.2338E-08	8.7784E-07
KRTAP3-3	3.1754562	1.6562E-07	2.1619E-06
METTL21C	-1.668236	9.2618E-08	1.2669E-06
RIMKLA	-1.2894342	5.2829E-08	7.5537E-07
IFIT1B	-3.0892304	2.5987E-07	3.2644E-06
ALOX15B	-0.8810244	5.0437E-08	7.2586E-07
CPA3	-1.201071	4.9321E-08	7.1154E-07
RUFY4	-1.0122438	4.9002E-08	7.0809E-07

KCNJ15	-1.4152274	5.278E-08	7.5528E-07
CTAGE15	-1.211945	5.4179E-08	7.7342E-07
PCDHA9	-1.4728738	8.1955E-08	1.1307E-06
CTD-3105H18.18	-1.6821112	7.7082E-08	1.0693E-06
RP1-138B7.6	-4.9015333	3.7026E-07	4.5331E-06
OR52N1	-3.3609592	7.0018E-08	9.7897E-07
CLDN9	-1.49407	5.4779E-08	7.7946E-07
NR5A1	-0.6094938	5.1265E-08	7.3538E-07
LRRC26	-2.6018736	9.8409E-08	1.3399E-06
CALB2	-0.9130312	5.5512E-08	7.8926E-07
XIRP1	-1.861773	7.0614E-08	9.8498E-07
PON1	-0.7136209	5.8139E-08	8.2462E-07
RP4-614O4.11	-1.3177422	7.4535E-08	1.0372E-06
FIBCD1	-1.414021	5.8349E-08	8.2694E-07
GPX3	-1.3749835	5.4506E-08	7.7684E-07
GPR151	-6.8215829	3.7201E-08	5.4786E-07
PTGER2	-0.7389068	5.9782E-08	8.4521E-07
WNT10B	-1.4780813	6.6015E-08	9.2889E-07
PAK6	-0.8033154	6.2187E-08	8.7641E-07
PATE2	-1.7191975	1.6767E-07	2.1871E-06
HBB	-1.2354288	5.9328E-08	8.4014E-07
SOWAHB	-1.2967226	7.3812E-08	1.0288E-06
THEGL	-1.4361846	9.987E-08	1.3577E-06
RTP5	-1.7705799	1.1074E-07	0.000001494
HMGCLL1	-1.5070571	7.038E-08	9.8326E-07
APOC4-APOC2	-0.6787745	6.7395E-08	9.4621E-07
SMPX	-0.6222781	6.6726E-08	9.3814E-07
AARD	-1.2870275	7.0475E-08	9.8381E-07
S100A9	-1.37755	6.7975E-08	9.5343E-07
CD70	-2.483854	1.322E-07	1.7567E-06
RP11-310N16.1	-3.8028273	3.6407E-07	4.4717E-06
PTGDS	-1.3219618	6.9203E-08	9.6835E-07

INSL5	-2.8337217	2.2402E-07	2.8608E-06
KRT5	-1.4852787	6.9026E-08	9.6663E-07
AKAP14	-2.2865228	1.3467E-07	1.7856E-06
C11orf94	-1.175153	7.7274E-08	1.0711E-06
CFHR5	-6.416549	3.7034E-07	4.5331E-06
ASTL	-1.2880909	8.2936E-08	1.1433E-06
TPBGL	-1.3286868	7.4066E-08	1.0315E-06
FAM166A	-1.0746879	7.6842E-08	1.0668E-06
KHDC3L	-1.7330477	1.6488E-07	2.1538E-06
CFTR	-0.8949933	7.8811E-08	1.0907E-06
GALR2	-2.3006831	1.4135E-07	1.8671E-06
TAC4	-1.5497123	1.0762E-07	1.4553E-06
CADM3	-1.3696354	8.7781E-08	1.2064E-06
DEFB124	-0.7244388	8.5222E-08	0.000001173
NKX6-1	-1.934204	9.2724E-08	1.2674E-06
COL5A2	-1.0577815	8.4249E-08	1.1605E-06
SCGB2A1	-1.3764602	9.0918E-08	1.2453E-06
KCNG3	-0.6344871	9.4519E-08	1.2902E-06
C8orf34	-1.3973373	1.0036E-07	1.3634E-06
KLRF1	-1.2850986	9.0296E-08	0.000001238
ARID3C	-1.5838722	1.0972E-07	1.4814E-06
OR52B2	-2.8309125	2.7174E-07	3.4015E-06
ERMN	-1.50329	1.1358E-07	1.5288E-06
GBP6	-0.8156404	9.612E-08	1.3098E-06
IGSF5	-0.9399926	9.4538E-08	1.2902E-06
HBD	-1.7416446	1.6905E-07	2.2034E-06
RPL10L	-2.4965897	2.4786E-07	3.1313E-06
LRRIQ1	-1.3199545	1.0161E-07	1.3782E-06
C8G	-0.8181801	9.5347E-08	1.3002E-06
LECT2	-1.5581661	3.1109E-07	3.8612E-06
SPESP1	-0.8634791	1.0213E-07	1.3831E-06
OXTR	-1.1703078	9.904E-08	1.3475E-06
C3orf20	-1.697068	1.7152E-07	2.2323E-06

MT1H	-2.180865	1.3388E-07	1.7764E-06
C16orf92	-3.050332	9.0577E-07	0.00001043
COX6A2	-0.6739967	1.0122E-07	1.3739E-06
RSPO3	-1.8032904	1.0714E-07	1.4499E-06
MRGPRD	-1.6278573	2.2921E-07	2.9208E-06
SAMD11	-1.1121907	1.0874E-07	1.4692E-06
CD1E	-1.9539338	1.6975E-07	2.2109E-06
GAL3ST2	-1.286297	1.1491E-07	0.000001542
FAM71D	-0.9579115	1.1393E-07	1.5315E-06
RSPO4	-1.5614836	1.1395E-07	1.5315E-06
FAM3B	-1.2364049	1.1126E-07	1.4999E-06
ANGPTL7	-0.6834845	1.1653E-07	1.5625E-06
KRT78	-1.3267974	1.2498E-07	0.000001667
POU2F3	-1.1734555	1.1415E-07	1.5331E-06
SHOX2	-0.6683265	1.2264E-07	1.6383E-06
UBL4B	-1.6620009	1.4961E-07	1.9733E-06
SLC6A7	-2.5407023	3.3119E-07	4.0992E-06
TMPRSS4	-1.2021762	1.306E-07	1.7381E-06
CCDC146	-1.0458141	1.1353E-07	1.5288E-06
AMDHD1	-0.6186189	1.2199E-07	1.6308E-06
CLEC12A	-1.2361271	1.2322E-07	1.6449E-06
FOSB	-1.4735946	1.2129E-07	0.000001624
UNC45B	-2.61324	2.5727E-07	3.2363E-06
PDE6H	-3.4243729	7.4543E-07	8.6682E-06
COLEC11	-0.7538287	1.2709E-07	1.6926E-06
FCRL6	-0.7530271	1.3147E-07	1.7484E-06
REN	-0.7896141	1.3977E-07	1.8503E-06
GATA2	-0.7585136	1.3928E-07	1.8452E-06
CLEC9A	-1.4352448	1.6455E-07	2.1511E-06
TPPP3	-0.9155034	1.2594E-07	1.6786E-06
NTS	-1.9357447	1.5505E-07	0.000002039
SH3TC2	-0.9360554	1.4934E-07	1.9712E-06
CCL8	-1.1556393	1.4003E-07	1.8524E-06

AC015688.3	-2.8922137	5.6869E-07	6.7415E-06
COL5A1	-1.2233997	1.3238E-07	1.7578E-06
PRSS22	-1.1880931	1.4999E-07	1.9768E-06
SMIM6	-1.9094873	1.7567E-07	2.2829E-06
KCNH1	-2.0410386	2.1265E-07	2.7274E-06
MYCL	-1.0459944	1.4031E-07	1.8548E-06
TM4SF19	-1.4926534	1.8187E-07	2.3583E-06
PCK1	-1.0473318	1.5611E-07	2.0514E-06
IGLON5	-1.2715124	1.6418E-07	2.1478E-06
TP53AIP1	-0.8688611	1.6201E-07	2.1242E-06
GDF3	-2.2054678	2.6066E-07	0.000003272
TSPYL6	-1.6278495	2.2288E-07	2.8504E-06
TMSB15A	-0.9683999	1.5966E-07	2.0949E-06
LTBP4	-0.8042732	1.5319E-07	0.000002016
SPATA4	-1.4379283	1.7907E-07	2.3254E-06
KRT6C	-1.7571353	2.1353E-07	2.7368E-06
EFCAB9	-3.4962729	8.3224E-07	9.6271E-06
GOLGA8H	-0.7367723	1.6252E-07	2.1293E-06
C1orf100	-1.1202182	1.9691E-07	2.5366E-06
E2F8	-0.9640112	1.8287E-07	2.3695E-06
ZC3H11B	-1.5821541	2.8834E-07	3.5889E-06
SLC30A2	-1.9911378	2.4886E-07	3.1403E-06
AREG	-0.8528042	1.9113E-07	2.4657E-06
SIT1	-1.2559442	1.8591E-07	2.4037E-06
TMEM59L	-0.7149378	1.8513E-07	0.000002397
COL26A1	-1.4808074	1.8589E-07	2.4037E-06
FBN3	-1.4879563	2.0326E-07	2.6108E-06
ADCYAP1R1	-0.906404	1.9519E-07	2.5163E-06
FXYD4	-3.14124	4.6681E-07	5.6243E-06
RAB6C	-1.3506614	2.3104E-07	2.9377E-06
HOXB9	-1.9789403	2.3769E-07	3.0137E-06
HSPB6	-0.8066396	1.8181E-07	2.3583E-06
OR6A2	2.07453281	1.1074E-06	0.000012547

CNTFR	-1.1947759	1.9074E-07	2.4625E-06
FIGLA	-1.5798288	3.056E-07	3.7984E-06
TUBA3D	-0.6789153	2.171E-07	2.7805E-06
PATE4	-1.9344936	8.2032E-07	9.5016E-06
PPP1R1B	-1.2533225	1.9749E-07	2.5422E-06
GRK7	-1.339249	2.4893E-07	3.1403E-06
MEST	-0.7740181	1.9914E-07	2.5603E-06
NEK2	-0.7689845	2.196E-07	2.8105E-06
ARC	-0.7408662	2.2373E-07	2.8592E-06
CTD-3214H19.4	-4.6695298	1.0587E-06	0.000012065
RASSF10	-1.5972238	2.7944E-07	0.000003488
WNT7B	-1.3981892	2.5947E-07	3.2617E-06
TSGA13	2.68847668	1.2352E-06	0.000013835
VCX3A	-2.0671884	5.3675E-07	6.3929E-06
OR1G1	-1.9948149	2.0372E-06	0.000021998
FIGNL2	-1.4369506	2.3608E-07	2.9953E-06
CD79A	-1.1861182	2.3062E-07	2.9345E-06
ZNF114	-0.7283092	2.2597E-07	2.8836E-06
TNFRSF13B	-1.565811	3.5965E-07	4.4266E-06
RPL36A-<br>HNRNPH2	-1.4181094	2.8437E-07	3.5446E-06
SLC7A3	-0.6412277	2.3944E-07	3.0314E-06
ECEL1	-0.7297685	2.327E-07	2.9567E-06
MUC21	-0.7412339	2.4228E-07	3.0653E-06
CKMT1B	-1.1887423	2.3841E-07	3.0205E-06
C10orf62	-1.8534198	4.8428E-07	5.8071E-06
LDLRAD1	-2.5614009	4.1709E-07	5.0632E-06
LYNX1	-0.8432262	2.2657E-07	2.8893E-06
SLC9C2	-1.2075682	2.5675E-07	3.2322E-06
NDP	-0.7956778	2.4557E-07	3.1047E-06
SYT6	-0.5953251	2.673E-07	3.3482E-06
MGP	-0.77341	2.3326E-07	2.9617E-06
ALPP	-1.6723116	2.7448E-07	3.4309E-06

IQCA1L	-2.5361447	6.7513E-07	7.8923E-06
LCE2B	-1.897697	1.0852E-06	0.000012319
ICAM5	-0.7265158	2.6141E-07	3.2791E-06
FABP6	-1.4159229	3.4936E-07	0.000004306
CXorf49B	-1.7223609	5.4521E-07	6.4773E-06
RP11-434D12.1	-1.4665169	3.5137E-07	4.3278E-06
CCR2	-0.7879867	2.6253E-07	3.2908E-06
LCE6A	-1.8590092	0.000001357	0.000015124
EYA4	-0.8993277	2.7483E-07	3.4329E-06
NCR1	-1.5907403	4.7178E-07	5.6803E-06
HAS2	-1.2604955	2.9556E-07	3.6762E-06
TCP10L2	-2.2921887	1.2559E-06	0.00001405
SFRP1	-0.8570515	2.7285E-07	3.4129E-06
MORC1	-2.0833882	6.5784E-07	7.7055E-06
CD3D	-0.9237668	2.8693E-07	3.5739E-06
AC003005.4	-5.9371861	3.1204E-07	3.8703E-06
EGF	-1.7190361	4.3589E-07	0.000005277
GYPE	-1.5135412	3.9591E-07	4.8258E-06
KRT6B	-0.9089249	3.2496E-07	0.000004025
KCNG2	-1.3489103	3.8087E-07	4.6522E-06
SYCE3	-2.5547049	6.6265E-07	7.7515E-06
APLP1	-1.0592493	3.1005E-07	0.000003851
CATSPER4	-1.7853775	1.3082E-06	0.000014589
GJA3	-1.807091	4.1158E-07	4.9997E-06
P2RY13	-1.0761882	3.4028E-07	4.2088E-06
F12	-1.290637	3.4537E-07	4.2627E-06
MRGPRX3	2.70152423	1.8753E-06	0.000020462
WNT2	-1.4302109	3.6536E-07	0.000004484
GZMK	-0.7618545	3.4272E-07	0.000004236
SHANK1	-0.8204092	3.6006E-07	4.4286E-06
LGALS12	-1.4221326	3.9617E-07	4.8258E-06
SEC14L4	-0.6749618	3.6558E-07	0.000004484
PDZD9	-1.4727084	4.1932E-07	5.0868E-06

PIANP	-0.8775604	3.7625E-07	0.000004599
SRMS	-1.6979526	5.105E-07	6.0926E-06
RNF222	-1.2759923	4.3481E-07	5.2675E-06
HNRNPCL2	3.09222478	1.1397E-06	0.000012872
MEP1A	4.92079789	1.9316E-06	0.000020972
PTN	-1.1959482	3.4435E-07	4.2532E-06
TEKT2	-0.8829005	3.7097E-07	4.5376E-06
PRR9	-2.2229305	6.1572E-07	0.000007265
CXorf65	-1.7561912	6.3255E-07	7.4437E-06
SLC22A14	-1.5399575	4.9887E-07	5.9699E-06
GRIK3	-1.7505819	3.9228E-07	4.7883E-06
POU1F1	-1.6462776	1.0185E-06	0.000011652
CEACAM4	-1.3825877	4.392E-07	5.3098E-06
UCHL1	-1.2530745	3.621E-07	4.4506E-06
PCDHA5	-1.3780162	4.0892E-07	4.9709E-06
SIGLECL1	3.23185122	1.9152E-06	0.000020833
KCNH3	-0.8779137	4.0328E-07	4.9057E-06
C5orf49	-0.831707	4.026E-07	4.9007E-06
IHH	-2.0368897	6.2778E-07	7.3925E-06
IGFL4	-1.5206676	5.7106E-07	6.7651E-06
BARX2	-1.4428048	4.5006E-07	5.4373E-06
CATSPER1	-2.0195351	6.5951E-07	7.7199E-06
FABP3	-0.8126158	3.9271E-07	4.7902E-06
GPR84	-1.0722667	4.2554E-07	5.1587E-06
MFSD2B	-1.2307428	5.0813E-07	6.0725E-06
ENTPD8	-0.8402636	4.3728E-07	5.2901E-06
SLC35G5	-1.329648	5.3767E-07	6.3996E-06
KCNG1	-0.9299211	4.6309E-07	5.5833E-06
CAPSL	-2.9490638	8.8354E-07	0.000010187
LEP	-1.7768851	7.3671E-07	8.5725E-06
GLOD5	-2.6025117	1.0057E-06	0.000011513
APOBEC3H	-1.088409	4.5454E-07	5.4877E-06
C2CD4D	-1.3845739	5.0804E-07	6.0725E-06

ENPP7	-3.1234954	3.7428E-06	0.00003842
GNLY	-0.7791517	4.9166E-07	5.8915E-06
NANOS3	-0.6320019	4.6246E-07	5.5795E-06
KIR3DL2	-2.1983789	2.3729E-06	0.000025298
NPTX2	-1.0578334	4.7225E-07	5.6821E-06
TEKT1	-1.776556	7.0513E-07	8.2158E-06
MTRNR2L3	-1.6178518	9.0085E-07	0.00001038
CYYR1	-0.9072653	4.7978E-07	5.7649E-06
SH2D6	-0.7236111	4.8274E-07	5.7925E-06
GRIN2D	-0.9092345	5.2098E-07	6.2135E-06
SPEF1	-0.7984714	4.9592E-07	5.9386E-06
CDC25C	-0.9113134	5.4632E-07	0.000006485
MDFI	-0.8715404	4.7492E-07	5.7104E-06
C2orf88	-1.1761984	4.8198E-07	5.7873E-06
AOC1	-1.4759102	5.8554E-07	6.9227E-06
CLEC4C	-2.8139557	2.4448E-06	0.000025955
ZNF888	-1.2169197	6.379E-07	7.5017E-06
TMEM100	-0.9496933	5.2465E-07	0.000006253
PKP1	-0.6390268	5.453E-07	6.4773E-06
HSF5	4.8608866	3.0336E-06	0.000031693
SNCB	-1.7469307	6.5536E-07	7.6815E-06
BTN1A1	-1.6274105	8.1825E-07	9.4838E-06
DIO2	-0.925709	5.8347E-07	6.9076E-06
LCE1A	-1.2392361	0.000004207	0.000042659
KCNK13	-1.3087435	6.422E-07	7.5473E-06
C9orf57	2.58106059	0.00000227	0.000024318
HGD	-0.8850632	6.2095E-07	7.3169E-06
CLEC11A	-0.7456581	5.5868E-07	6.6273E-06
TNFRSF11B	-1.171323	6.0122E-07	7.0987E-06
CENPA	-0.6972801	6.2057E-07	7.3169E-06
TBX1	-0.6881528	6.481E-07	7.6065E-06
KRTAP2-2	-4.7355736	1.8989E-06	0.000020668
CENPW	-0.8851491	5.8501E-07	6.9211E-06

SERPINB7	-2.3596262	1.0775E-06	0.000012248
RSPH4A	-0.613733	6.5058E-07	7.6305E-06
FAM90A26	-2.8804745	5.2696E-06	0.000052509
VEPH1	-0.9712361	6.4607E-07	7.5877E-06
SLC22A16	-1.7767908	9.7163E-07	0.000011159
GATA3	-0.7790275	6.8826E-07	8.0298E-06
S100A12	-0.6057484	6.7588E-07	7.8958E-06
C9orf152	-1.3806379	8.2385E-07	9.5362E-06
OR2L3	-2.106326	4.3535E-06	0.000043875
SCGB3A2	-2.1934961	1.3826E-06	0.00001537
C4orf50	-2.8327095	2.5973E-06	0.000027492
SLC22A2	-2.0226588	2.6043E-06	0.000027549
RP11-364B14.3	-1.4324231	8.7462E-07	0.000010097
FN1	-1.3079657	7.1835E-07	8.3643E-06
RP3-370M22.8	-1.6353712	1.6151E-06	0.000017786
AIPL1	-1.810614	2.2483E-06	0.000024115
CREG2	-1.5952103	1.1435E-06	0.000012907
RLN2	-1.3206532	9.9312E-07	0.000011384
GJD3	-1.7922878	1.4527E-06	0.000016059
CCNO	-0.896801	7.9683E-07	9.2416E-06
SOD3	-0.8159717	7.7132E-07	8.9516E-06
FOXR1	-2.0891713	2.6135E-06	0.00002763
DHRS9	-1.5702729	9.2256E-07	0.000010603
SLC7A4	-0.9213265	8.7077E-07	0.00001006
MYB	-1.0087666	9.0843E-07	0.000010454
OR9A2	-3.7216168	8.6626E-06	0.000082197
CAPS	-0.8440764	8.5604E-07	9.8959E-06
KIAA1549L	-1.5887006	1.0266E-06	0.00001173
TPSB2	-0.8014962	9.7811E-07	0.000011219
GJB5	-1.5295608	1.0023E-06	0.000011482
NEIL3	-1.4099901	1.2245E-06	0.000013734
CYP4B1	-1.2847732	9.1761E-07	0.000010553
PRR23B	2.89144973	6.4049E-06	0.000062761

SLC26A8	-1.2309239	1.0725E-06	0.000012198
TAGLN3	-1.6720936	1.1517E-06	0.000012982
PRSS48	-1.7929717	2.7924E-06	0.000029363
CYP17A1	-0.6709335	1.0462E-06	0.000011939
TMPRSS13	-1.1614281	9.7805E-07	0.000011219
UNC93A	-2.2944448	2.3553E-06	0.000025126
CCDC70	-2.1519693	0.00000672	0.000065631
RSPH6A	-1.5718638	1.8089E-06	0.000019774
ZMYND10	-0.755764	1.0401E-06	0.000011876
FAM167A	-1.2884819	1.1873E-06	0.000013358
THSD7B	-1.5216636	1.2067E-06	0.000013543
ST8SIA6	-0.749035	1.0646E-06	0.000012117
OSM	-0.6832948	1.0583E-06	0.000012065
LIPN	-1.4991964	5.7007E-06	0.000056423
CA9	-1.3588788	1.0644E-06	0.000012117
LRRC46	-0.7169074	0.000001137	0.00001285
P2RY12	-1.3375453	1.1616E-06	0.000013086
S1PR4	-0.9257576	1.1014E-06	0.000012492
PIGR	-1.7341847	1.2645E-06	0.000014129
MS4A4E	-0.7863455	1.1189E-06	0.000012653
PLSCR2	-1.1331915	1.5664E-06	0.000017273
KNG1	-2.1100486	3.7381E-06	0.000038395
ACTA2	-0.9228327	1.1018E-06	0.000012492
FOS	-1.0215882	1.1122E-06	0.000012585
MEIG1	-1.3480374	1.4238E-06	0.000015798
ERP27	-1.175417	1.1443E-06	0.000012908
SOWAHA	-0.8970831	0.000001196	0.000013445
C4orf19	-0.8115777	1.1718E-06	0.000013193
DEFB119	-1.4831198	7.3155E-06	0.000070628
KRTAP5-7	-1.7698767	0.000002296	0.000024567
C16orf90	-2.5226826	3.5092E-06	0.000036318
ODF3L1	-1.2337103	1.2837E-06	0.000014334
IGLL1	-1.9278238	2.2344E-06	0.000024004

DRD1	-1.6762916	2.0425E-06	0.000022028
GTSF1L	-2.3611025	3.5455E-06	0.000036672
GOLGA7B	-1.1400481	1.4356E-06	0.00001591
FAM166B	-0.9099881	1.3059E-06	0.000014572
PDE11A	-0.596072	1.3691E-06	0.000015239
PPP1R14A	-0.7704589	1.2628E-06	0.000014118
LUZP4	3.30818119	7.6686E-06	0.000073754
ASGR2	-1.0637033	1.4695E-06	0.000016234
SMKR1	-0.7949861	1.3774E-06	0.000015322
KRTAP2-1	2.57451441	9.0536E-06	0.000085449
CCDC169	-0.7597346	1.4154E-06	0.000015715
EGFL6	-1.0660278	1.4999E-06	0.000016549
CAPN9	-1.2901893	0.000001484	0.000016384
RIBC2	-0.6926701	0.000001426	0.000015813
OR52L1	-3.839756	9.6666E-06	0.00009075
GPR141	-1.307696	1.9222E-06	0.000020883
MMP11	-1.3463508	1.4465E-06	0.000016
NGB	-2.5591814	3.9304E-06	0.000040114
CXCR3	-1.1664875	1.6853E-06	0.000018503
C19orf84	-1.4128297	2.2426E-06	0.000024068
NLGN4Y	-3.0640177	9.5115E-06	0.000089436
FAM81B	-1.7362538	2.1866E-06	0.000023524
CRYAB	-0.9687495	1.4425E-06	0.000015976
TSNAX-DISC1	-2.2543771	4.2107E-06	0.000042659
PGA5	-0.5936318	1.5984E-06	0.000017614
ZBED2	-1.1330106	1.7223E-06	0.000018863
WNT16	-1.8669228	2.2842E-06	0.000024455
SSX2B	-4.2420829	0.000013756	0.00012558
FAM155B	-0.8253275	0.000001616	0.000017786
CTB-60B18.10	-1.571912	2.8133E-06	0.000029565
ANKS4B	4.44355376	9.5317E-06	0.000089579
MPC1L	2.67385146	5.5675E-06	0.000055228
LRRN4	-1.2397449	1.6616E-06	0.000018254

SALL4	-0.7390428	1.6519E-06	0.000018158
MS4A2	-1.686213	2.4698E-06	0.000026189
CTD-2568A17.1	-1.8241898	3.6465E-06	0.000037498
OR2A4	-4.1167538	0.000015831	0.00014171
FZD9	-1.0889571	1.6872E-06	0.000018513
SLC9C1	-2.2722623	4.6795E-06	0.000046973
RELN	-0.6472756	1.7666E-06	0.000019336
LIX1	-2.0975015	2.6845E-06	0.00002833
PKLR	-2.2883639	6.2658E-06	0.000061569
EBF2	-1.4040129	1.8828E-06	0.000020519
GABRQ	-1.8278414	3.9435E-06	0.000040216
KIR2DL1	-1.653782	4.3995E-06	0.000044313
PNOC	-1.3481407	1.8827E-06	0.000020519
SMIM18	-2.0722834	0.000004866	0.000048707
DUSP13	-1.6823454	3.2728E-06	0.000034031
TSPAN7	-0.7969678	1.6914E-06	0.000018535
RP11-38C17.1	-0.8116293	1.7941E-06	0.000019625
LCE2C	-1.5970977	0.000011628	0.00010779
EBI3	-0.6159832	1.8333E-06	0.000020028
RNF113B	-1.4971647	3.1019E-06	0.000032387
NPFFR1	-1.2464611	2.0686E-06	0.000022295
IL20	-1.8075151	4.6583E-06	0.000046813
TEX38	-1.415772	2.2687E-06	0.000024318
OR10A2	-2.3214438	0.000014137	0.00012882
TEX33	2.65482262	0.000013449	0.00012287
KRTAP5-11	-4.2444315	0.00001052	0.000098192
EGR1	-1.0072732	1.8446E-06	0.000020139
UPP2	-1.0425512	2.0317E-06	0.000021952
POF1B	-1.2809327	2.0258E-06	0.000021915
ADGRV1	-0.7722607	2.0311E-06	0.000021952
LGR5	-1.4540821	1.9801E-06	0.000021473
SGCG	-1.279404	2.0227E-06	0.000021895
ZNF683	-1.5276773	2.4385E-06	0.000025903

RDM1	-1.5819215	3.2595E-06	0.000033932
AP000721.4	-3.1545369	0.000010044	0.000094141
CCDC3	-0.7913926	1.9537E-06	0.000021199
SLC30A8	-1.9268513	4.8135E-06	0.000048236
APOA1	-1.2129329	0.00000204	0.000022014
LYZ	-1.1001853	2.0042E-06	0.000021708
PRELP	-0.7669332	1.9923E-06	0.000021592
SOHLH2	-1.5619058	2.8945E-06	0.000030329
SULT2B1	-1.1312476	2.1907E-06	0.000023555
MEI4	1.84277348	4.0343E-06	0.000041079
KYNU	-0.9607819	2.1587E-06	0.000023239
GLYATL2	-1.9162545	3.7966E-06	0.000038927
PROC	-0.592342	2.1568E-06	0.000023232
MUC15	-1.4432267	2.6769E-06	0.000028267
LAMP3	-1.1161766	0.0000023	0.000024594
SERPINB5	-1.37521	2.4185E-06	0.000025722
B3GNT3	-1.0101047	2.4336E-06	0.000025867
DTHD1	-1.7785735	3.3799E-06	0.000035061
PRR30	-4.3251812	0.000015274	0.00013755
TRIM72	-1.754357	4.2991E-06	0.000043351
RAD21L1	-3.1772994	0.000018322	0.0001613
NEU4	-0.6443207	2.3956E-06	0.000025494
WNT6	-0.9442365	2.3179E-06	0.000024756
IRGC	-1.7062939	5.4594E-06	0.000054278
ALG1L2	-0.811998	2.3893E-06	0.000025458
ADAMDEC1	-1.3733599	2.7869E-06	0.00002934
DCAF12L1	-0.6824086	2.4499E-06	0.000025994
CXCL2	-0.7967563	2.5574E-06	0.000027085
KLHDC8A	-0.5971064	0.000002393	0.000025481
RP11-691N7.6	-1.4454825	4.8337E-06	0.000048411
TMCO5A	-3.4669789	0.00001771	0.00015677
DUSP1	-0.8918372	2.3314E-06	0.000024886
PRKCG	-1.5696438	3.2672E-06	0.000033993

TEX15	-1.3784826	2.9526E-06	0.000030919
FGFR3	-0.9095908	2.5508E-06	0.000027032
TTLL10	-1.7343627	3.5763E-06	0.000036904
ADGRG7	2.30030042	0.000015324	0.00013786
CD1C	-1.260461	3.1227E-06	0.000032585
P2RY14	-0.8636554	2.6325E-06	0.000027814
CEACAM6	-1.260848	2.8627E-06	0.000030031
SOHLH1	-1.9549156	4.6256E-06	0.000046511
IGFL1	-2.4745114	5.7835E-06	0.000057211
PPP1R27	-1.0240265	0.000002733	0.000028824
LIPH	-0.6867826	2.8534E-06	0.000029951
GC	-2.3659148	6.8218E-06	0.000066478
C1orf116	-1.157383	0.000002872	0.000030111
FOXD4L1	-1.3831302	4.2064E-06	0.000042659
SPINK6	-2.1998756	7.1059E-06	0.000068867
KRT16	-1.0187293	2.9784E-06	0.000031171
AEBP1	-1.0181243	2.7359E-06	0.000028838
PCSK9	-1.6471926	3.9184E-06	0.000040014
FCGBP	-0.8195163	3.0012E-06	0.000031391
MMP10	-3.0059937	6.2181E-06	0.000061134
KCNJ10	-0.8533231	3.0269E-06	0.000031641
SPOCK2	-1.0483856	2.7886E-06	0.00002934
ID1	-0.8059041	2.7877E-06	0.00002934
PDZD3	-1.3500818	3.6208E-06	0.000037303
PTGDR2	-0.9550053	3.2353E-06	0.00003372
IBSP	-2.7939611	0.000007304	0.000070555
GRM7	-1.1802586	3.6305E-06	0.000037355
HS3ST6	-1.6441752	5.6804E-06	0.000056254
ULBP2	-1.3724381	4.2111E-06	0.000042659
PCDH17	-0.614501	3.6217E-06	0.000037303
GALNT13	-0.6152465	3.4678E-06	0.000035911
ARL13A	-1.1080166	4.2755E-06	0.000043187
FLT3	-1.4317784	0.000005034	0.000050303

DNAJB13	-0.9895262	0.000003418	0.000035436
CFAP52	-0.5934368	3.4391E-06	0.000035634
LRRC19	-1.0224636	3.8759E-06	0.000039649
THSD4	-1.0805015	3.3723E-06	0.000035003
ANO1	-0.7924267	3.5725E-06	0.000036887
MAS1L	-2.5010564	0.000027637	0.00023637
ZNF488	-0.6463918	0.000003662	0.000037635
TNNI3	-0.872766	3.6234E-06	0.000037303
C3orf56	-2.4044714	0.000029771	0.00025328
CCL11	-1.166817	4.2512E-06	0.000042966
NMU	1.26492276	3.5651E-06	0.000036832
C20orf85	-2.1267038	6.0082E-06	0.000059301
ACR	-0.7342547	0.000003799	0.00003893
C17orf98	-1.9720879	8.4636E-06	0.000080568
SH3GL3	-0.7369084	3.8936E-06	0.000039806
IL1B	-0.7977056	3.8989E-06	0.000039839
CREB3L3	-2.1714941	9.1772E-06	0.000086569
TMC5	-0.9479249	3.9449E-06	0.000040216
MMP2	-0.9169733	3.7883E-06	0.000038866
SIX1	-1.6218825	4.2139E-06	0.000042663
PASD1	1.81807469	0.000015399	0.00013832
MYCN	-1.0477674	4.0453E-06	0.000041144
OR2B11	-1.8196735	0.00003339	0.00028068
TTC16	-1.052222	4.1609E-06	0.000042272
OR2I1P	-2.7438269	9.7657E-06	0.000091583
AC004076.9	-0.9878206	4.4295E-06	0.00004459
ZAR1	-2.5593849	0.000023771	0.00020503
CHI3L1	-0.9344622	3.9675E-06	0.000040422
AC004076.7	-2.8457362	0.000014233	0.00012943
DNASE2B	-1.7905441	0.000010822	0.00010074
SHISA2	-1.3063435	4.2846E-06	0.000043254
CYP3A7	-0.8842705	4.2454E-06	0.000042932
BTBD16	-1.2783453	6.0313E-06	0.00005943

OR7C1	2.34300476	0.000040333	0.00033258
MMEL1	-0.7579336	4.2946E-06	0.000043331
GGTLC2	-3.3986097	0.000025883	0.0002219
RETN	-1.4452641	6.0515E-06	0.000059595
FZD10	-1.0495235	4.5502E-06	0.000045779
SULF1	-0.9793876	4.2215E-06	0.000042715
ACTRT1	-1.4446485	0.000036554	0.00030397
PRSS58	-4.5380811	5.1536E-06	0.00005144
CDH17	-1.8706932	8.5457E-06	0.000081218
TMEM210	-2.5116105	0.000014717	0.00013301
LGALS9C	-1.5846079	8.9596E-06	0.000084697
SHISA7	-1.699561	7.2805E-06	0.000070405
HCRTR1	-1.5208497	0.000010664	0.000099424
SLC6A15	-0.6174022	4.6776E-06	0.000046973
SIGLEC14	-0.9882954	4.7117E-06	0.000047269
MAB21L3	1.73233273	0.00001824	0.00016066
LDHC	-1.179702	5.6715E-06	0.000056197
CDH12	-1.4847001	8.1854E-06	0.000078299
OR2L5	-2.0447786	0.000038886	0.00032176
XKRX	-1.0087029	0.000005019	0.000050181
FAM83E	-1.1558784	5.3962E-06	0.000053679
LCN2	-1.0353658	4.7843E-06	0.000047971
SLC5A7	-1.8858196	0.00001164	0.00010784
SIGLEC10	-0.6048558	5.1263E-06	0.000051196
DRC1	-1.4271698	6.9863E-06	0.000067857
C1orf146	-1.9562638	0.000015216	0.00013717
RRAD	-0.632354	4.9865E-06	0.000049885
IFI6	-1.0242589	5.1895E-06	0.00005174
SEBOX	-4.3962634	0.000008907	0.000084245
CITED4	-0.6780186	5.1607E-06	0.000051482
CCL7	-2.6629389	0.000011876	0.00010986
ZNF157	-0.737946	5.4631E-06	0.000054284
ARSI	-0.591279	5.5533E-06	0.000055118

PDYN	-2.1426392	0.000011167	0.00010374
CYP1A1	-1.7122184	7.7472E-06	0.00007447
SLC40A1	-0.8099544	0.000005365	0.000053399
GLDC	-1.2464643	5.8657E-06	0.000057992
HKDC1	-1.1312404	6.1986E-06	0.000060976
CIDEC	-0.6897384	6.0181E-06	0.000059366
PTF1A	-2.1237659	0.00001376	0.00012558
COL3A1	-1.0980687	5.6653E-06	0.000056167
POU3F2	2.32423699	0.000025773	0.00022118
RAB19	-2.2076141	0.000014026	0.00012787
PALM3	-1.1347523	5.9523E-06	0.000058782
SP9	2.0704549	0.000030549	0.00025939
SLC7A11	-0.6907887	6.1048E-06	0.000060086
APOF	-1.867137	0.000028935	0.00024688
NLRP12	-1.3170838	8.4757E-06	0.00008064
GPR39	-1.4357154	7.9521E-06	0.000076191
CXorf49	-1.6671125	0.000012484	0.00011452
GALNT14	-0.8582661	6.0303E-06	0.00005943
OR2C1	-1.4849881	8.3895E-06	0.000080078
IL27	-1.3472712	0.000010241	0.000095692
PPIAL4G	-3.0058059	0.000035693	0.00029722
TTLL2	-1.2765377	0.000007532	0.000072559
ARNTL2	-0.660071	6.5446E-06	0.000064059
RPTN	4.73923057	0.000048765	0.0003951
AZGP1	-0.7549417	6.7393E-06	0.000065783
XKR9	-0.6842595	6.3962E-06	0.00006271
KMO	-0.9006865	6.6508E-06	0.00006499
C12orf42	-2.2004135	0.000016267	0.00014516
FA2H	-0.9851	6.9343E-06	0.000067426
S100A6	-0.8089834	0.000006293	0.000061801
ASIC2	-1.3775963	8.4564E-06	0.000080543
PCSK1N	-1.0463619	6.4657E-06	0.000063321
VSTM2L	-1.4181372	6.9575E-06	0.000067614

DCN	-0.7679079	6.3631E-06	0.000062421
CRTAM	-1.2674439	8.2736E-06	0.000079015
HLA-DRB5	-1.0565057	6.5989E-06	0.000064555
AGT	-0.6107434	7.1222E-06	0.000068987
ADAMTS14	-1.1153952	0.000007094	0.000068789
KLRB1	-0.7156485	6.8325E-06	0.000066545
PLA2G12B	-2.095811	0.000035848	0.00029837
CXCL1	-1.192438	6.7915E-06	0.000066219
SERPINF1	-0.8339016	6.6316E-06	0.000064839
GAPT	-1.0416435	7.3823E-06	0.000071195
PPIAL4D	-4.2295934	0.000057669	0.00045842
HOXB5	-1.3339353	7.2671E-06	0.000070314
OR2A14	-2.5924575	0.000046878	0.00038121
OR5V1	2.75948355	0.000043513	0.00035613
MXRA8	-0.8795932	6.8753E-06	0.000066925
TUNAR	-1.7626498	0.000010052	0.000094171
SCGB1D2	-1.0490503	7.1612E-06	0.000069327
PRR15L	-0.8153246	7.2991E-06	0.000070546
ZFP36	-0.7350867	6.9169E-06	0.000067294
DEFB108B	-2.4968588	0.000071164	0.00055693
KCTD16	-1.4833374	0.000015139	0.00013654
ZFP57	-1.7415892	0.000014305	0.00013001
KCND3	-0.7664364	8.2274E-06	0.000078658
TNFRSF13C	-0.6815946	7.6254E-06	0.000073379
SKA1	-0.8349225	7.8185E-06	0.000075074
GSC	-1.2984258	0.00000875	0.000082982
KRT23	-1.1283984	7.8833E-06	0.000075614
CHRM4	-1.2856517	9.2259E-06	0.000086861
DLX2	-1.9772291	0.000017532	0.00015543
TSPAN19	-1.4785587	0.000013129	0.00012013
COL10A1	-1.3448504	7.9105E-06	0.000075834
MYOM3	-0.7049879	8.0762E-06	0.000077296
SERPINA1	-0.8304358	7.3616E-06	0.000071034

MMP12	-2.4530731	0.0000189	0.00016606
F5	-1.5755711	0.000011278	0.00010471
DEFB4A	-2.8121106	0.000029264	0.00024944
CLRN3	-2.5081784	0.000031354	0.00026546
SCX	-0.9579466	7.7665E-06	0.000074615
SPARC	-0.825059	7.8317E-06	0.00007516
NGEF	-0.6773232	8.5792E-06	0.000081493
METTL7B	-0.8467149	8.2463E-06	0.000078796
PYHIN1	-1.1291848	8.7923E-06	0.000083294
FGF5	-2.2406698	0.000021067	0.00018364
EDARADD	-0.6865998	8.4152E-06	0.000080237
NXPH4	-1.0926892	9.2278E-06	0.000086861
C9orf50	-1.0629879	9.0399E-06	0.000085365
COL1A2	-0.981782	8.0648E-06	0.00007723
ATP1B2	-0.6556099	8.3991E-06	0.000080127
CLDN14	-2.0998738	0.000024903	0.00021413
HOXB6	-1.295979	8.8569E-06	0.000083816
TRIM31	-1.3387773	0.000011911	0.00011007
LY6G6C	-1.1786955	8.7555E-06	0.00008299
SLC22A13	-1.2407687	0.000014445	0.00013102
DRD2	-0.6171732	9.3818E-06	0.000088264
NMNAT2	-0.7851922	9.5783E-06	0.000089969
SLC9A2	-1.425198	0.000012037	0.000111
KRTAP10-10	-2.3049194	0.000078214	0.00060808
TFAP2A	-1.0072069	9.1841E-06	0.000086588
HLA-DOA	-0.9270755	9.1934E-06	0.000086629
SPATA18	-0.6369354	0.000010273	0.000095932
FAM111B	-0.8387017	9.7093E-06	0.000091103
ADCYAP1	-2.2516359	0.000018917	0.00016613
OASL	-0.981174	0.000010068	0.00009427
KAAG1	-1.6130002	0.000017993	0.00015887
GRIA2	-0.7769173	0.000010568	0.000098585
AMH	-0.6227312	0.000010152	0.000095009

TUBA4B	-1.7765183	0.000017763	0.00015717
AICDA	-3.1862024	0.000069518	0.00054478
LINGO3	-1.2570434	0.000012231	0.00011262
UPK3A	-1.2792975	0.000015247	0.00013738
FAM177B	-1.2382859	0.000013189	0.00012062
PHOX2A	1.3377489	0.00001099	0.0001022
FOXC1	-0.776176	0.000010242	0.000095692
OR51B5	-1.3416286	0.000017203	0.00015267
LUM	-1.1182864	0.000010681	0.000099531
NOX4	-0.5980043	0.000011556	0.00010718
DUSP9	-0.7071614	0.000011419	0.00010596
SLC25A52	-1.4121914	0.000023211	0.00020056
HBQ1	-1.5736467	0.000016523	0.00014723
MYH4	2.82095411	0.00005446	0.00043625
VSTM5	-1.1125496	0.000012435	0.00011414
GPR160	-0.7272659	0.00001111	0.00010326
MEOX1	-1.3251915	0.000011861	0.00010978
LCE1D	-2.635567	0.00008995	0.00068902
NIPAL1	-0.8293176	0.000011942	0.0001103
FAM209A	-0.9419733	0.000012037	0.000111
CLPSL1	-2.5839036	0.00003016	0.00025622
ASB10	-1.8530369	0.000034558	0.000289
HES2	-1.3433296	0.000016388	0.00014617
DEFA5	-2.8004234	0.000050125	0.00040538
BIK	-0.7887988	0.000012007	0.00011084
CASP5	-1.1189104	0.000018864	0.00016591
CELA2B	-0.6192747	0.000012251	0.00011274
RPA4	-0.8081711	0.000013048	0.00011951
PKD2L2	-0.6917264	0.000012975	0.00011891
IDO1	-1.1158443	0.000012419	0.00011404
COL9A2	-0.8660119	0.00001189	0.00010993
CDH22	-0.6794988	0.000013291	0.00012149
KPRP	-2.4588227	0.000047637	0.00038703

TRIM29	-0.9151028	0.000012215	0.00011253
CALCA	-1.7027859	0.000022102	0.00019172
CD24	-0.7220379	0.000012185	0.0001123
FKBP6	-1.4022318	0.000016474	0.00014686
LRRC10	2.23121549	0.00011795	0.00088324
HRCT1	-0.7962049	0.000013111	0.00012003
PCOLCE	-0.6306627	0.0000123	0.00011313
OR5C1	-1.6699577	0.00012096	0.00090273
CTHRC1	-0.9867275	0.000012373	0.0001137
CDC42EP5	-0.7358772	0.000012375	0.0001137
TUBB4A	-1.0492483	0.000012866	0.00011796
PRB3	-3.0084948	0.000048365	0.00039222
LHB	-1.0016972	0.000015818	0.00014166
VN1R2	-1.6530673	0.000088384	0.00067849
DLX3	-0.7689157	0.000013801	0.00012589
MT1A	-0.8021697	0.000014199	0.00012932
RNF212B	-2.1186828	0.000048573	0.00039373
CTD-3214H19.16	-2.3869696	0.00013736	0.00101226
SLC5A4	-1.0807615	0.000015612	0.00014002
VSTM1	-2.4466317	0.000031143	0.00026387
DMBX1	-2.7683562	0.000037795	0.0003134
NKX3-2	-0.7755169	0.000014682	0.00013276
CA8	-0.924548	0.000014613	0.00013227
APOBEC3B	-0.8210344	0.000014227	0.00012943
PI3	-1.1514048	0.000014532	0.00013175
BAAT	-1.5647598	0.000033934	0.00028459
TOMM20L	-0.81794	0.000016962	0.00015083
KCP	-1.2328128	0.000015422	0.00013846
CNFN	-0.8497455	0.00001436	0.00013038
GPR19	-0.9128068	0.000016003	0.00014309
CKMT1A	-1.0610899	0.000016036	0.00014332
ANXA8	-0.6071973	0.000015025	0.00013559

COL6A3	-0.7032144	0.000014211	0.00012936
COL1A1	-0.9623476	0.000014549	0.00013183
OPN4	5.2872015	0.00010126	0.00076765
GGT2	-1.4365026	0.000052484	0.00042291
HOXD9	-0.6166964	0.000014639	0.00013244
SPSB4	-1.4143669	0.000021539	0.00018757
COL9A1	-1.3441948	0.000018092	0.0001596
XG	-0.6199966	0.000015793	0.00014151
COL6A1	-0.8052628	0.000014745	0.00013319
FAM71A	-1.9582318	0.00013753	0.00101312
TGM2	-0.7070355	0.000015001	0.00013544
MIA2	-1.1807409	0.000022496	0.00019485
STX19	-1.0687084	0.000015752	0.00014121
HTR3C	-2.5919964	0.000065541	0.00051567
A1CF	2.76850245	0.00012185	0.00090783
CRHR1	-1.3791529	0.000020046	0.0001757
KRT17	-0.9993925	0.000015535	0.0001394
IGLL5	-1.3188455	0.000016896	0.0001504
ITM2C	-0.8967831	0.000015396	0.00013832
HOXD1	-1.2528418	0.000016046	0.00014334
CDKN2C	-0.6105826	0.000015332	0.00013787
CXCL9	-1.2087737	0.000017023	0.00015123
GSDMC	-1.3357505	0.000023452	0.00020244
KRTAP5-3	-2.261131	0.00020955	0.00147484
RBBP8NL	0.77479535	0.000016176	0.00014442
CTSW	-0.6131814	0.000016775	0.00014939
FAM107A	-1.00936	0.0000159	0.00014225
ARSF	-2.6941534	0.0001098	0.00082636
LILRA2	-0.7173037	0.000016929	0.00015061
TRDN	-1.2414167	0.000020222	0.00017698
ANKRD22	-1.0064518	0.000018702	0.00016457
HLA-DQA2	-1.0819395	0.000018	0.00015887
CKAP2L	-0.7595413	0.000018209	0.00016047

ACSBG2	-0.8627241	0.000017906	0.00015835
KRTAP11-1	2.09755547	0.00014907	0.00108953
C2CD4A	-1.5118502	0.000026477	0.00022678
OR10H2	-3.293429	0.00016965	0.00122324
ACSL6	2.37091601	0.000070445	0.0005518
ERBB4	-1.0515495	0.000020197	0.00017685
MYO1H	-1.3401896	0.000024723	0.00021268
FAM187B	-1.9459943	0.000056116	0.00044768
POTEJ	-2.327436	0.0001441	0.00105754
IFIT1	-0.8358929	0.000017957	0.00015865
UGT2B17	-1.4810183	0.000030965	0.00026255
SSMEM1	-1.3624112	0.00014556	0.00106738
OPN1SW	-0.8801723	0.000021608	0.00018799
GCG	2.38535116	0.00013895	0.00102313
WFDC13	-2.0690671	0.000079495	0.00061614
RASAL1	-0.7114245	0.00002031	0.00017757
C15orf48	-0.8681262	0.00001912	0.00016783
C7	-0.6925349	0.000018897	0.00016606
TEX101	-1.6792691	0.000040511	0.00033389
BNIPL	-0.9888276	0.00002009	0.00017599
IFNA5	-1.4950509	0.00016069	0.00116573
RRH	-0.9719175	0.000021724	0.00018872
DPPA3	-2.5323727	0.000088231	0.00067761
TMEM176A	-0.620428	0.000019211	0.00016855
OTC	-1.5105106	0.000031886	0.00026933
TAGLN	-0.7905775	0.000019523	0.0001712
NKX2-5	-1.6040427	0.000039263	0.00032466
ALPL	-0.8945206	0.000020356	0.00017788
LTB	-1.0051751	0.000020415	0.00017831
IGFBP5	-0.7970251	0.000020279	0.00017739
GAGE2E	-1.878741	0.0001091	0.00082182
LHX2	-1.5755719	0.00002861	0.00024434
ENHO	-0.75641	0.000021451	0.0001869

PLA2G10	-1.1029711	0.000028362	0.00024246
IRX3	-1.230572	0.000021688	0.0001885
ASPDH	-0.8301547	0.000022599	0.00019565
GALNTL6	-1.5779309	0.000042585	0.00034952
CNTF	-0.7394268	0.000023357	0.00020171
TMEM176B	-0.7671033	0.000021631	0.0001881
RGS18	-0.8179993	0.00002296	0.00019858
DLGAP3	-0.7113442	0.000023896	0.00020597
CCNE1	-0.7194393	0.000021807	0.00018935
EMILIN1	-0.6169356	0.000022203	0.0001925
ICAM1	-0.767295	0.000022396	0.00019408
SPECC1L-ADORA2A	-2.5193535	0.00011102	0.00083383
TMEM95	-3.2167741	0.00020361	0.00143995
OR10A3	-2.6453136	0.00016377	0.00118612
OR2B6	-2.094559	0.000063012	0.00049754
CXCL14	-1.2344944	0.000023776	0.00020503
MIXL1	-0.8303894	0.00002456	0.00021148
NPY4R	-1.8039262	0.000045394	0.0003705
CLRN1	-4.467447	0.0001695	0.00122265
MYH8	2.36516171	0.00020214	0.0014316
GRIFIN	-2.3845151	0.000068409	0.00053704
C20orf202	-0.9872296	0.000029366	0.00025019
RP11-20I23.1	-2.3862809	0.00012141	0.00090533
MAGEA2B	-4.1264198	0.00011019	0.00082838
DMBT1	-1.576544	0.000033927	0.00028459
DLGAP5	-0.6411164	0.000027052	0.00023159
OR5B2	1.09470963	0.000082964	0.0006403
CLDN1	-0.7693554	0.000025319	0.00021749
ACTL8	-2.5893947	0.00005793	0.00046009
RHAG	-2.0667021	0.000070521	0.00055215
APOC2	-1.0789106	0.000031648	0.00026757
MT1F	-0.8037695	0.000025794	0.00022125

FANCD2OS	-2.20712	0.00012694	0.00094171
IL12RB1	-0.8224198	0.00002755	0.00023574
CATSPERB	-1.01758	0.000030563	0.00025939
ZNF703	-0.6902809	0.000025975	0.00022259
ROPN1	-2.2359234	0.00010605	0.00080052
IP6K3	-0.7215551	0.000029636	0.00025237
VCAN	-0.7917863	0.000028782	0.00024569
DSCAM	2.59844427	0.000097579	0.00074231
PHOSPHO1	-0.8611902	0.000029185	0.00024889
SLC6A5	3.53462416	0.00028644	0.00194335
PNLIPRP3	4.33277906	0.00024625	0.00170131
CRISP3	-1.6917924	0.000035182	0.00029338
SPTSSB	-1.2044151	0.000033749	0.0002833
TP63	-0.6112929	0.000030091	0.00025575
PPP1R14D	-2.0958583	0.000057363	0.00045661
PDX1	2.70433419	0.00032074	0.00214723
LRRC4C	-0.5919899	0.000031151	0.00026387
LYZL1	-0.8510375	0.0002977	0.00200585
CTCFL	-1.0706143	0.000038402	0.00031813
TEX12	-1.0580816	0.000033432	0.00028091
GPR45	-1.3148583	0.000071548	0.00055969
C1orf127	-1.20835	0.000043291	0.00035465
TMEM158	-0.8766574	0.000029745	0.00025318
KRT85	-1.8789341	0.00011088	0.00083314
ISG15	-0.8893788	0.000030028	0.00025534
TSGA10IP	-1.5260704	0.00010287	0.00077888
IL10	-0.843326	0.00003142	0.00026577
RBP3	-1.5584755	0.00012254	0.00091179
ZIC1	-1.2816886	0.000039663	0.00032766
CMA1	-2.1299575	0.00008657	0.00066649
GJB1	-0.9485882	0.000034099	0.0002857
ARHGAP40	-1.24537	0.000047745	0.00038773
NUTM2F	3.51761414	0.00034122	0.00226374

OXT	-1.0408603	0.000042619	0.00034963
PLEK2	-0.5856039	0.000032989	0.00027758
COL6A2	-0.7590799	0.00003201	0.00027024
INHBA	-0.6452525	0.000032734	0.00027569
ADGRG2	-1.038465	0.000034488	0.00028855
GJB3	-0.832734	0.000033526	0.00028157
SPOCD1	-0.6901782	0.000034657	0.00028968
SPNS3	-1.2114574	0.000042076	0.00034565
MX1	-0.8105343	0.000032512	0.00027409
CDH2	-0.9549856	0.000032775	0.00027592
MC3R	-3.6226534	0.00049535	0.00317093
PTPRT	-1.7644521	0.000045669	0.00037224
AGBL4	-0.728085	0.000035941	0.000299
NFE2	-0.926165	0.000034702	0.00028993
CD2	-0.8032369	0.000035633	0.00029686
TEX37	-1.3711468	0.00019922	0.00141396
SPDEF	-1.0797932	0.000035332	0.0002945
TNFSF10	-0.7612077	0.000034211	0.0002865
MDGA2	-1.1900566	0.000078949	0.00061325
C5orf46	-1.4250979	0.000064764	0.00051001
BGN	-0.7109524	0.000035027	0.00029223
C4orf48	-0.6701451	0.000034967	0.00029187
GZMA	-0.7038291	0.00003688	0.00030653
MT1G	-1.1207765	0.000037322	0.00031006
DEFB103B	-2.4215843	0.0004995	0.00319055
MYPN	-1.8209094	0.000094298	0.0007189
COL22A1	-0.588606	0.000038894	0.00032176
ATP1B4	2.60212663	0.00025055	0.00172563
TRIM58	-0.8701215	0.000041621	0.00034239
STAC2	-0.8746739	0.000040776	0.00033591
LRRTM1	-1.0877375	0.000039819	0.00032864
MPL	-0.6296442	0.000039914	0.00032927
COX8C	-2.099688	0.00007184	0.00056145

MBOAT4	-0.8392991	0.000043242	0.00035441
RP11-514O12.4	-2.0823905	0.000091711	0.0007022
B3GALT1	-0.6143987	0.000042037	0.00034549
IL37	-2.0255016	0.00010047	0.00076263
GADL1	-1.7888324	0.000093853	0.00071643
SSX3	-2.8693493	0.00048398	0.00310488
LYPD6B	-0.7226365	0.000041748	0.00034328
LILRA5	-0.6319027	0.000042876	0.00035158
HORMAD2	-2.1101841	0.00025747	0.0017664
HIGD2B	-3.5279362	0.0003464	0.0022938
UGT1A6	-1.9330136	0.00024903	0.00171784
HECW1	-0.6483614	0.000045581	0.00037169
APOL1	-0.7720398	0.000042502	0.00034899
CAPZA3	-1.6906933	0.00033483	0.00222721
ALDH3B2	-0.8819916	0.000046666	0.00038001
FST	-0.8684188	0.000048341	0.0003922
QPCT	-0.6568534	0.000045514	0.00037131
SIRPG	-1.0727154	0.000054138	0.00043406
MT1E	-0.9884088	0.000045051	0.00036788
SPIC	-1.8969701	0.0002889	0.0019555
LRRC55	-1.1222416	0.000056606	0.00045139
FAM189A1	-0.6134448	0.000049185	0.00039832
TSPAN1	-1.0652699	0.000046153	0.000376
PITX1	-1.281472	0.000047917	0.00038895
GRP	-2.718627	0.00013513	0.00099706
SMR3B	-3.8963884	0.00029419	0.00198452
NKD2	-0.7473721	0.000051296	0.00041465
KIF26B	-0.6898393	0.00005203	0.00041983
GPR152	-1.2460605	0.00025454	0.00174836
TMPRSS3	-0.6557514	0.000052526	0.00042306
CDC20B	-2.0469193	0.00010183	0.00077163
LY9	-0.7234939	0.000054371	0.00043573
GPR37	-0.8773216	0.00005475	0.00043837

KCNQ5	-1.3484565	0.000068748	0.00053946
TTC24	-1.2621486	0.000074829	0.00058304
TKTL2	-1.6882829	0.00020013	0.00141987
CXCL8	-0.8949866	0.000054131	0.00043406
DACT2	-0.9896782	0.000055466	0.0004433
SMCO2	-1.3872025	0.000082968	0.0006403
ISX	2.05206835	0.00031565	0.00211715
FUT9	1.9681785	0.00022205	0.00155293
GJA1	-0.7271377	0.000051671	0.0004175
CCND1	-0.8398316	0.000051847	0.00041861
SDK2	-0.6663314	0.000057091	0.00045485
KIF18B	-0.6689268	0.000055605	0.00044402
PRAMEF6	-4.11515	0.00067253	0.00415184
MS4A15	-1.1763596	0.000066743	0.00052466
HOXD13	-2.0301079	0.00017078	0.00122985
SMCO1	-2.0292147	0.00034193	0.00226714
CPXM1	-0.7523894	0.000053414	0.00042923
KRTAP10-3	2.52776738	0.00067997	0.00418901
ST6GALNAC5	-0.7973446	0.00005353	0.00042996
TBC1D3F	-2.0776963	0.00027076	0.00185186
ASRGL1	-0.7203438	0.000053838	0.00043224
SCEL	-0.9457917	0.000055769	0.00044512
ISLR	-0.8142305	0.000054093	0.00043406
MROH6	-0.6196133	0.000055298	0.00044236
CYP4A11	-1.5506906	0.000079058	0.00061351
TMEM211	-1.4256015	0.00010528	0.00079573
CCR5	-0.6405249	0.000059641	0.00047303
MT2A	-0.6517499	0.000056735	0.00045222
KLHL31	-0.6004088	0.00006109	0.00048344
UNC5A	-1.0776098	0.000060268	0.00047758
PHLDA2	-0.6927243	0.000057462	0.00045719
SECTM1	-0.7692512	0.000057187	0.00045541
CLDN24	-1.695802	0.00025281	0.00173918

CYP2J2	-0.7154619	0.000060164	0.00047697
RP1-27O5.3	-1.9478851	0.00037544	0.00246949
TBX22	-1.2919314	0.00020428	0.00144353
FOXN1	-1.3272073	0.000092913	0.00071018
CDSN	-1.9340399	0.00015165	0.00110675
LENEP	-0.8967079	0.000066577	0.00052358
DLX4	-1.2155006	0.000078152	0.00060786
CYP4X1	-0.7846236	0.000061027	0.00048316
TRPM1	-1.8260703	0.00015969	0.00115939
LAMP5	-0.7747699	0.000063621	0.00050168
CPSF4L	-1.1186784	0.000086987	0.00066906
CCL22	-1.1996825	0.000094958	0.00072331
DHRS3	-0.6252671	0.000061807	0.00048868
TGM4	-1.8458444	0.00016981	0.00122391
PCP4	-1.1778351	0.000067211	0.0005281
LGALS9B	-1.6155511	0.00019742	0.00140229
CKB	-0.6715039	0.000064889	0.00051076
MC4R	-1.4515532	0.00010367	0.00078423
TMPRSS11D	-2.0240494	0.00017031	0.00122698
KRTAP7-1	-5.9835194	0.00023479	0.00163108
PRSS51	-1.1788711	0.000079065	0.00061351
THEM5	-0.9620037	0.000079825	0.00061843
E2F2	-0.6696811	0.000074232	0.00057865
CRTAC1	-1.0575812	0.000069429	0.00054432
RNASE1	-0.626823	0.000067724	0.0005319
RSPO1	-1.2358786	0.000071868	0.00056145
DRC7	-0.9615826	0.00008322	0.00064192
OR52E5	-5.0599336	0.0002791	0.00189838
C10orf67	-0.9973272	0.000081981	0.00063346
CHST1	-0.6406169	0.000071599	0.00055985
HLA-DQB1	-0.8143299	0.000072469	0.00056554
RHO	-1.3560843	0.00012203	0.00090881
ATP4B	-1.5030152	0.00012505	0.00092901

RPGRIP1	-0.9790302	0.00008472	0.00065291
DUSP21	2.07358629	0.00101054	0.00593409
SLC9A4	-1.3788309	0.00011006	0.00082802
APOC1	-0.609805	0.000074186	0.00057854
CACNG4	-0.8154827	0.000081617	0.0006312
VGLL2	-1.5601808	0.00015336	0.00111856
DIRAS2	-1.0568471	0.000094231	0.0007189
EFNA2	-1.7517228	0.00015168	0.00110675
TGM1	-0.7184417	0.000075757	0.00059001
CNTN2	-1.4448954	0.00012234	0.00091069
GRIK1	-0.5866121	0.000086045	0.00066284
CLU	-0.7775106	0.000076421	0.00059492
FGF6	-7.4911235	0.00039512	0.0025845
MMP3	-1.4934455	0.00012556	0.00093228
FAM72D	-0.892735	0.000089942	0.00068902
PAPOLB	-1.9824145	0.00032266	0.00215681
AIRE	-2.1140093	0.00022056	0.00154495
IGF2BP1	-1.6557654	0.00017218	0.00123943
TACSTD2	-0.6948073	0.000079304	0.00061493
ERN2	-1.3983172	0.00014897	0.00108953
IL1F10	-5.6839026	0.0004706	0.00303006
FBXO43	-0.5898742	0.000084615	0.00065239
SUSD2	-0.9014812	0.000081334	0.00062929
THBS1	-0.5933581	0.00008079	0.00062563
PLAU	-0.8051533	0.000081711	0.00063166
NT5DC4	-1.8882001	0.00036695	0.0024163
SPP1	-0.7495354	0.000082975	0.0006403
CALCB	-1.6374005	0.00014832	0.00108629
KCNK17	-0.9650449	0.00009653	0.00073497
TMEM171	-1.0875858	0.00011861	0.00088741
OR52E6	-2.6299954	0.00141156	0.00792343
AGMO	-1.235059	0.00011225	0.00084268
CEACAM3	-1.4290916	0.0001899	0.00135542

PPIAL4A	-2.8493014	0.00056552	0.00356336
TTYH1	-0.70533	0.000089407	0.00068575
CABS1	-1.7983741	0.000796	0.00482651
IGSF23	-2.1608663	0.00023773	0.0016489
FBXO2	-0.7883499	0.000087285	0.00067064
TM6SF2	-0.791319	0.000094262	0.0007189
AKR1B10	-0.585638	0.000092781	0.00070947
LCE1E	-1.9034134	0.00084182	0.00506437
RASGEF1C	-0.8167216	0.000093619	0.00071496
ZNF365	-0.8542822	0.00010677	0.00080565
MCIDAS	-2.2658071	0.00024408	0.00168902
KISS1R	-1.5237347	0.00019299	0.00137496
LRRC10B	-0.6511312	0.0001019	0.00077185
SIGLEC15	-1.1971974	0.00012506	0.00092901
BDKRB1	-1.0441855	0.00011326	0.00084994
PSMB9	-0.6110332	0.000092656	0.00070883
AWAT2	-2.4177488	0.00047928	0.00307922
FNDC1	-0.862458	0.00010017	0.00076104
IFIT3	-0.7046144	0.000093121	0.00071146
ALOXE3	-1.1695531	0.00012699	0.00094171
NKX2-8	-1.9069792	0.00024711	0.00170596
NT5C1B	-0.9445642	0.00010948	0.00082437
NME8	-1.3907502	0.00015447	0.00112524
TRHR	-1.4671472	0.00050087	0.00319582
MT1M	-0.7241571	0.00010565	0.00079819
WFDC9	-2.692338	0.00133975	0.00759004
FAM83A	-1.0772999	0.00010739	0.00080962
CIB3	1.48688586	0.00121973	0.00699075
MACC1	-0.7866253	0.00010788	0.00081301
KRTAP5-8	-1.1417056	0.0001592	0.00115636
KRTAP5-4	-2.4273494	0.00077725	0.00472273
SLAMF6	-0.6960217	0.00010569	0.00079819
FXYD3	-0.7299927	0.00010041	0.00076251

CNGA1	-1.2203839	0.00012695	0.00094171
SDC1	-0.7130069	0.00010052	0.00076269
JCHAIN	-1.0258123	0.00010704	0.00080731
PLAT	-0.6427846	0.00010074	0.00076408
FAM71F1	-1.1115435	0.00015866	0.0011534
SNCG	-0.9654864	0.00010489	0.00079314
RP11-1099M24.7	-4.1721054	0.0009804	0.00578587
BMP5	2.76898437	0.0003395	0.00225487
MPO	-1.0654664	0.00014696	0.00107717
PLSCR5	1.11853367	0.00133742	0.00757927
HRH2	-0.7082131	0.00011611	0.00087017
CRB2	-0.8418548	0.00011857	0.00088741
ODF1	-2.2664996	0.00051158	0.00325829
SLC15A1	-1.169475	0.00014876	0.00108857
SCGB1D4	-3.291732	0.00033942	0.00225487
RNF182	-0.738294	0.00011606	0.00087016
OR6F1	-1.5955609	0.0019036	0.01026809
SOST	-1.6891612	0.00024476	0.00169303
LEMD1	-0.770929	0.00011666	0.00087391
CST5	-3.4443279	0.00044396	0.0028721
KCNC2	1.92548214	0.00074803	0.00456219
SUN3	-1.8740599	0.00032615	0.00217601
SPACA4	-1.5361856	0.00023528	0.00163385
TIMD4	-1.1246745	0.00016791	0.0012122
HES4	-0.6120696	0.00011942	0.00089276
SCN1A	2.51656459	0.00035241	0.00233013
MMP14	-0.5955961	0.00012067	0.00090093
CD52	-0.738918	0.00012309	0.00091554
UBQLN3	-2.7397306	0.00039382	0.00257698
CRYBB3	-0.9649093	0.00015658	0.00113875
TNFAIP2	-0.6714905	0.00012339	0.00091738
IL1RAPL2	-1.1461604	0.00016891	0.00121888

LPA	-1.1030846	0.000167	0.00120661
CTAGE9	-2.6773246	0.00076939	0.00467794
CCNA1	-0.8927748	0.00012753	0.00094498
ANKRD7	-0.7343026	0.00013575	0.00100129
OR7D2	1.4890385	0.00027659	0.00188376
KLK4	-1.3802541	0.00023685	0.00164348
RIMBP3	-1.3700856	0.00025031	0.00172465
CHRNB3	1.8605334	0.00056268	0.00354926
CD300E	-0.9494661	0.00014937	0.00109125
MAPK15	-0.6624187	0.00013171	0.00097515
GRHL3	-0.8572354	0.00014146	0.00104011
RP1-37E16.12	-6.3782524	0.00111673	0.00646957
CLGN	-0.8972389	0.00013632	0.00100506
IL32	-0.6000543	0.00013382	0.00098825
C17orf50	-1.1880764	0.00025758	0.00176652
GMNC	-1.1335828	0.00015975	0.00115939
FAM209B	-0.8673567	0.0001485	0.00108715
CCIN	-1.1906074	0.0002028	0.00143537
FAT2	-0.7529646	0.00014385	0.00105614
GPR150	-1.0124113	0.00020882	0.00147144
NR2E1	-3.4022986	0.00118674	0.0068231
A3GALT2	-1.1399163	0.00023165	0.00161373
GPNMB	-0.624124	0.0001404	0.00103296
PRAMEF13	-3.8932916	0.00179171	0.00974778
ANXA10	-1.1954605	0.00023035	0.00160657
PTH2R	-0.7794752	0.00018267	0.00130861
ATP12A	-2.432376	0.00038679	0.0025347
KLRC1	-1.1133064	0.00019094	0.00136179
CXorf58	-1.1429112	0.00024937	0.00171954
DAPL1	-0.9053034	0.00015542	0.00113123
CNR2	1.3937227	0.00067359	0.00415692
HLA-DMA	-0.6862002	0.00015131	0.00110495
ST6GALNAC1	-0.7313136	0.00015353	0.00111934

KLB	-1.4567726	0.0003284	0.00219023
MFAP2	-0.6996313	0.00015466	0.00112616
CCDC83	-1.4675163	0.00128979	0.00734405
PRAMEF2	1.96468822	0.00253096	0.01315075
STRA8	-2.5138532	0.00047447	0.00305275
KRT31	-1.9962006	0.0004733	0.00304631
EPPK1	-0.789555	0.00016771	0.00121119
OR2AK2	-2.0594375	0.00188036	0.01016438
UPK3B	-0.8522097	0.00016134	0.00117001
APOE	-0.6030479	0.00015906	0.00115585
OR51Q1	-0.9961676	0.00099704	0.00586646
MTRNR2L1	-3.2883117	0.00075374	0.00459227
HPN	-0.7364115	0.00016571	0.00119871
OR2T8	-2.0652608	0.0006678	0.00413129
HEATR9	-1.2509743	0.00028242	0.00191828
SYT14	1.46803913	0.00040825	0.00266543
BTNL8	-1.0548995	0.00023378	0.00162533
EPPIN-WFDC6	-2.103099	0.00071299	0.00436658
MYO18B	-1.3810995	0.00027257	0.00186284
SLC5A1	-0.9762038	0.00019142	0.00136462
OR14K1	-1.8905775	0.00111439	0.00646236
HOXB8	-1.181417	0.00020427	0.00144353
MYBPHL	-1.4670542	0.00029236	0.00197443
GUCA2A	-1.3509713	0.00050043	0.00319418
OR8U1	-1.7608923	0.00290906	0.01478523
HCAR1	-0.9329132	0.00018563	0.00132761
MUC16	-0.8786063	0.00019302	0.00137496
SST	-1.1027498	0.00019671	0.00139894
SERPINE3	-0.9245483	0.00023132	0.00161201
NRGN	-0.6589591	0.00017751	0.0012752
SAA2-SAA4	-1.6332227	0.00042574	0.00276844
PF4	-1.4784005	0.0003601	0.00237744
GAL3ST3	-1.0180325	0.00020484	0.00144691

GPR65	-0.6566105	0.0001909	0.00136179
SCARA3	-0.6113257	0.00017991	0.00129146
OAS2	-0.7427377	0.00018146	0.001302
ARHGAP19-SLIT1	-1.7357	0.00106598	0.0062163
CYBB	-0.6831947	0.00018703	0.00133712
COMP	-1.1280437	0.00019681	0.00139912
SLC4A11	-0.8152065	0.00018904	0.00134982
IGFL2	-1.1902113	0.00027369	0.00186831
ITGAD	-1.1371596	0.00027659	0.00188376
KAZALD1	-0.7050308	0.00020172	0.00142999
RP11-407P15.2	-2.2227745	0.00118701	0.0068231
IFNA1	-1.5262803	0.00323943	0.01614419
GDF15	-0.6037369	0.00019885	0.0014119
HCN2	-0.8777194	0.00023059	0.00160759
GRAP2	-0.8798187	0.00020949	0.00147484
CRYBA1	-1.878067	0.00079711	0.00483048
DEFB103A	-3.6364891	0.00332936	0.01651182
SLC35G6	-0.9589803	0.00025941	0.001777
ARL11	-0.7955825	0.00021748	0.00152478
ZSWIM2	-2.1900804	0.00172371	0.0094213
SUSD3	-0.6019844	0.00019698	0.00139978
KLHL34	1.41870135	0.00064496	0.00400684
WDR87	1.18975911	0.00063092	0.00393064
AGRP	-0.8287123	0.00023216	0.00161665
TRIML2	-3.5206214	0.00200355	0.0107384
IFITM5	-1.843634	0.00055557	0.00351192
FOXN4	-1.1751709	0.00030011	0.0020206
MYBPH	-1.2862223	0.00030518	0.00205157
ZSCAN4	-1.1775801	0.00042036	0.00273747
IL4I1	-0.6621923	0.00020774	0.00146561
GPR6	-2.0781018	0.00191483	0.01031918
LHX5	1.89063497	0.00152052	0.00844115

TBX21	-0.8671074	0.00024048	0.00166537
E2F7	-0.7471494	0.00022273	0.00155648
CTXN1	-0.6179923	0.00020952	0.00147484
IKZF3	-0.6620023	0.00023286	0.00162022
ZNF560	-1.0039065	0.00026581	0.00181872
TREML1	-0.8973153	0.00024681	0.00170456
FNDC7	-1.7079476	0.00099465	0.0058563
NEURL3	-0.8916845	0.00027613	0.00188205
NNMT	-0.8313538	0.00022185	0.00155213
ID4	-0.6193755	0.00022065	0.00154496
RHOXF2B	-1.4349406	0.0007218	0.00441747
RD3L	-2.5123202	0.0036733	0.01794742
RP1-4G17.5	-3.5271115	0.00420832	0.02016048
CRYBA2	-2.5453548	0.00169	0.00925707
SCG5	-0.652245	0.00023914	0.00165671
MMP7	-0.8334877	0.00023901	0.00165649
TMEFF1	-1.0416021	0.00029731	0.00200403
C1QA	-0.6734327	0.0002336	0.00162475
KCNA10	-2.2526242	0.00296506	0.01501374
MMP9	-0.9212423	0.00023886	0.00165608
TP53	-0.6323875	0.00023438	0.00162888
CA7	-1.2903218	0.00050455	0.0032182
OTOA	-1.4724205	0.00052794	0.00335462
ABCG4	-0.8381049	0.00025847	0.00177127
OR13C3	-1.5028805	0.00266958	0.01374583
TPTE2	-0.9268334	0.00029165	0.00197117
DNMT3L	-2.6464547	0.00465549	0.02185868
GNG5P2	0.70456921	0.00026137	0.00178973
EPYC	-1.1821988	0.00028168	0.00191469
RARRES2	-0.5991856	0.0002454	0.00169615
RP3-468K18.5	-6.8987417	0.0011754	0.00676956
NKX2-6	1.6098471	0.00445702	0.02111743
GPRC6A	-2.4259215	0.00298925	0.01511323

GDF5	-1.1589505	0.00033984	0.00225546
S100G	-3.4186971	0.00159069	0.00876464
GALNTL5	-2.746838	0.00232461	0.01221491
LIPI	-0.9300962	0.00031739	0.00212719
LGALS7	-1.2031566	0.00043055	0.00279766
SCUBE3	-0.6226563	0.00028218	0.00191738
HSPA1A	-0.6392546	0.00025485	0.00174978
TREM2	-0.6431168	0.00026543	0.00181679
IL22RA1	-0.5900102	0.00028301	0.00192077
AANAT	-0.8239292	0.00027522	0.00187704
CFC1B	2.87318816	0.00400127	0.01928878
DEFA4	-0.9894008	0.0015381	0.00852001
NOTO	-1.824791	0.00267513	0.01376637
GYPA	4.58008109	0.00238215	0.01248021
FAM72C	-0.8914401	0.00032434	0.00216719
OR4M1	-2.3856213	0.00476154	0.02228563
ADIG	-1.0524161	0.00317405	0.01586755
PRPS1L1	-1.2527165	0.00385686	0.01872529
LRIT1	1.17604026	0.00446778	0.02115708
KRT33A	-1.6207125	0.00144333	0.00807595
GABRB2	-1.2475311	0.0003641	0.00239937
KIF1A	-1.0409955	0.00027426	0.00187149
DEFB125	2.11613854	0.00493905	0.02294037
VN1R4	-2.1375643	0.0049864	0.02313601
TMEM45B	-0.7152309	0.00028273	0.00191965
AGBL1	-1.6210258	0.00289108	0.01470649
GJD2	1.96770982	0.00226835	0.01195479
OAS1	-0.5921087	0.00027529	0.00187704
FAM43A	-0.6097908	0.0002776	0.00188915
HEMGN	-1.7989458	0.00124686	0.00713233
KRT80	-0.7355877	0.00029166	0.00197117
FCAR	-0.8566936	0.00032885	0.002192
ADGRE3	1.55430353	0.00148794	0.00828106

RIMS1	-1.4431123	0.00044259	0.00286431
RAB27B	-0.834035	0.000326	0.00217584
SLC45A2	-1.1055194	0.00072805	0.00445264
KRT20	-1.2417479	0.00070107	0.00430101
GJB4	1.91448804	0.00048833	0.00313052
WFDC12	-1.9798489	0.00127277	0.00726411
SLCO6A1	-1.8562931	0.00106824	0.00622329
GOLGA6C	-1.4989885	0.00264227	0.01363549
TBX2	-0.5986496	0.00029702	0.00200285
LHFPL1	-1.002789	0.0003453	0.00228736
ZNF750	-0.7101528	0.00031008	0.00208137
MSI1	-0.7826016	0.00030574	0.00205454
SSX4B	-2.2423834	0.00162589	0.00895025
WFDC5	-1.2133767	0.0005699	0.00358966
OR52E4	-4.8825148	0.00291526	0.01480822
GAS1	-0.5901437	0.00030272	0.00203663
PRTN3	-1.2236192	0.00054694	0.00346234
SPANXD	2.06407482	0.00581943	0.0263716
C19orf33	-0.6505237	0.00030933	0.00207712
IGFBP2	-0.6041544	0.00030902	0.00207584
SLAMF9	-1.9694938	0.00091235	0.00544243
THY1	-0.6300666	0.00031076	0.00208512
SHD	-1.4737796	0.000558	0.0035235
CLIC5	-0.6886786	0.00032178	0.00215253
OR7G1	2.00739375	0.00612046	0.02751717
OAS3	-0.624998	0.00032111	0.00214889
FOXF2	-0.5925025	0.00033984	0.00225546
PRR36	-0.6193422	0.00032974	0.00219583
CRLF2	2.38980134	0.00268901	0.01382974
SYT13	-0.6787612	0.00036197	0.00238792
APOL5	-1.6366894	0.00250309	0.01303273
ASB5	-1.4001689	0.00068271	0.00420298
GABRA1	2.21637474	0.00165617	0.00909711

CTC-360G5.8	-3.9969024	0.00727025	0.03162989
KRTAP5-9	-0.828243	0.00038634	0.00253268
RTBDN	-1.6894047	0.00103689	0.00607065
PPBP	-1.1845685	0.00059349	0.00372367
WFDC2	-0.6393996	0.00034277	0.00227146
NAT8L	-0.5938002	0.00035035	0.00231822
AC006486.9	-1.8175247	0.00743606	0.03225148
REG4	-2.2179792	0.00135908	0.00768236
SLPI	-0.6765311	0.00035295	0.00233282
KRTAP19-3	-4.0169936	0.00457159	0.02155058
SYNGR4	-2.1365949	0.00098377	0.00580193
LCT	2.10413855	0.00211716	0.0112451
VTCN1	-0.8386025	0.00036277	0.00239149
PROM2	-0.7162887	0.00035701	0.0023588
TMEM244	-5.0005331	0.00478758	0.02238973
NMUR1	-0.9483399	0.00041104	0.00268266
AVP	-2.0619942	0.00128574	0.00732398
POSTN	-1.1454242	0.00037358	0.00245906
C1QC	-0.7002042	0.00036573	0.00240917
CTAG1B	1.01200497	0.00086871	0.00520317
TXNDC8	-3.8256624	0.00521337	0.02408171
DNAJC5B	-1.3327999	0.00066477	0.00411833
TUBB8	-0.9695779	0.00045188	0.00291729
ADM2	-0.8114198	0.00043596	0.00282861
SLC44A4	-0.676438	0.00038167	0.00250486
ZFP42	-2.707786	0.00150562	0.00836101
C6orf58	-1.0074653	0.00058526	0.00367726
SULT2A1	5.89017797	0.00328854	0.01633832
HLA-DPA1	-0.7168669	0.00039976	0.0026129
C1QL2	-1.34042	0.00061093	0.00382358
ETV4	-0.6250628	0.00041355	0.0026971
L1CAM	-0.858387	0.00042234	0.00274931
PRSS21	-0.7489161	0.00045193	0.00291729

TNFAIP6	-0.6491889	0.00043574	0.00282821
HLA-DPB1	-0.7117691	0.00041475	0.00270291
GPR82	-0.8769354	0.00045265	0.00292084
C1QTNF9	-1.1100856	0.00053208	0.00337912
SSX4	-2.5073236	0.00275781	0.01413021
OR9A4	-2.0701657	0.00227632	0.01199325
C1QB	-0.6685149	0.00042767	0.0027799
CCDC42	-1.2185	0.00071945	0.00440461
CRABP1	-0.9851458	0.0004657	0.00299958
PRRX2	-0.7262976	0.00043902	0.00284536
OR2W3	-1.1537586	0.00069012	0.00423822
POTEE	-1.1554132	0.00094864	0.00563038
GPR18	-0.7643299	0.00049231	0.00315376
TNP2	-1.4090173	0.00165896	0.00910393
CRABP2	-0.6443905	0.00044822	0.00289752
KLK14	-0.6709728	0.0004575	0.00294997
GLT6D1	-1.7271923	0.0031056	0.01559149
KCNA7	-1.4566966	0.00173255	0.00946084
BANF2	-2.5330685	0.00167452	0.00918363
XCL1	-0.8434761	0.00049834	0.00318542
OR6C4	1.99089727	0.00956911	0.03967389
TFCP2L1	-0.8476326	0.00049574	0.0031723
CIB4	-1.0728934	0.0009038	0.00539686
RP11-834C11.12	-6.1720639	0.01000815	0.04117566
ITGB4	-0.6348395	0.00047629	0.00306221
CACNG6	-1.5493409	0.00104209	0.0060971
FGF18	-0.7034602	0.00048457	0.00310755
OLFM2	-0.6117016	0.00047886	0.00307763
GJA10	1.177804	0.00827672	0.03526629
RP11-152F13.10	-1.9026823	0.01014848	0.04164168
POTEM	-0.6608908	0.00423344	0.02024792
RNF224	-0.6666356	0.0005168	0.00329034

KLC3	-0.6309479	0.00053551	0.00339846
ADGRA1	1.07833496	0.00108672	0.00632262
SMIM2	-1.3930368	0.00126	0.00719586
RPRML	-1.169124	0.00088851	0.00531636
HLA-DQA1	-0.750338	0.00051137	0.00325814
TCHHL1	1.91547206	0.00510271	0.02362609
SLC38A8	-1.4061667	0.00106805	0.00622329
CACNA1I	-1.1598028	0.00061795	0.00385817
GSG1L	-1.161359	0.00085728	0.00514688
DEFB1	-0.8730513	0.00054249	0.00343662
ARMC3	-1.0676777	0.00086285	0.00517682
BECN2	-1.7085126	0.00138207	0.00779235
CXCL17	-0.9316837	0.00055442	0.0035059
KLF1	-1.1976417	0.00095026	0.0056326
CYP4Z1	-0.9526926	0.00068213	0.00420086
ARGFX	-1.6479257	0.01158972	0.04628299
SLC8A3	1.49765486	0.00200907	0.01076148
ADAMTS18	-1.0561903	0.00078784	0.00478286
LILRA1	-0.7358353	0.00060486	0.00378831
FEZF2	-2.3620108	0.00226268	0.01193202
LAIR2	-1.7986462	0.00168065	0.00921441
OVOL1	-0.7794192	0.00062525	0.00389667
SYCP1	-1.7561753	0.00198743	0.01066173
OR52K2	-0.9606452	0.00536398	0.02464227
RCOR2	-0.5993244	0.00057792	0.00363243
SEMA3A	-0.5933272	0.00061518	0.00384475
CCDC33	-0.8169387	0.00068618	0.0042199
S100A1	-0.7030615	0.00059052	0.00370708
OR5M1	-2.6793598	0.01263635	0.04961135
RPL3L	-0.8070672	0.00064983	0.00403422
CA12	-0.727597	0.00061798	0.00385817
IL11	-0.9128661	0.00066148	0.00409995
RP11-201K10.3	-2.2624494	0.00651876	0.02891831

BRINP1	-1.0890719	0.00082992	0.00500303
PDCL2	-2.4712896	0.00207075	0.01104505
ZNF695	-0.787463	0.00067858	0.00418334
INHBB	-0.7264602	0.00061743	0.00385744
SLC6A17	-1.0703082	0.00098179	0.00579218
TEX36	-1.9897358	0.00463294	0.02178752
WNT8A	-1.4804642	0.00226706	0.01195159
RTP4	-0.6095116	0.0006351	0.00395249
SI	3.1371229	0.00609348	0.02742378
OTUD6A	-1.8463119	0.01124362	0.04520706
OXGR1	-0.8063208	0.00068357	0.00420531
RUNX3	-0.7945296	0.0006668	0.00412801
OR2T12	-1.3410517	0.00906623	0.03799869
AMER3	2.03947916	0.00409843	0.01969273
DMRT3	-1.6710178	0.00175538	0.00956776
RLN1	-1.2139688	0.00137216	0.00774389
SLC18A3	-2.5198405	0.00253981	0.01318897
CCDC87	-0.7418502	0.00071068	0.00435391
ADRB1	-0.9120489	0.00083637	0.00503504
OR7E24	-1.6006703	0.00733636	0.03188162
CTLA4	-0.8056608	0.00078799	0.00478286
CDH6	-0.7143555	0.00069964	0.00429373
ATCAY	-1.7254592	0.0023861	0.01249716
CDX2	2.37183888	0.0025122	0.01306863
PLEKHN1	-0.6478704	0.00075171	0.00458151
CLDN10	-0.8716468	0.00073052	0.00446464
FCRL1	-1.3801967	0.00184931	0.01001797
SPATA31A6	-2.7377687	0.00667579	0.02952607
ATP2B2	-0.7695674	0.00080948	0.00489313
TMEM72	-1.4077373	0.00134598	0.00762293
ANGPTL6	-0.6886702	0.00076424	0.00464827
PDZK1IP1	-0.7198609	0.00073551	0.00449204
PADI6	-2.9895563	0.00555312	0.02535186

KLK11	-0.7141326	0.0007379	0.00450352
PRMT8	-1.0552076	0.00113833	0.00658392
BOLL	-1.7060783	0.00240697	0.01259156
COLEC10	-2.0814263	0.00274205	0.01406163
UCN2	-0.8672202	0.00083269	0.00501797
LRRIQ3	-0.7238644	0.00080142	0.00485438
GPR42	-1.9844156	0.00562013	0.02560619
GPR33	-1.2595515	0.01207248	0.047858
DOK5	-0.687252	0.00075813	0.00461742
TMC7	-0.6782577	0.00081707	0.00493562
LALBA	-1.8167621	0.00814912	0.03479806
KLK10	-0.6290888	0.00077729	0.00472273
RXFP3	0.93766649	0.00984532	0.04063806
RP11-385D13.1	-1.7356483	0.00356984	0.01750966
SLC13A2	-1.1213343	0.00174901	0.00953596
TMEM74	-0.9155513	0.00099523	0.00585778
LANCL3	-1.1827636	0.00137187	0.00774389
WNT10A	-0.7023373	0.00086724	0.00519961
MAGEA4	-1.9408478	0.00237769	0.01246051
MAL	-0.7605515	0.00083938	0.00505139
RP11-219A15.1	-1.5781909	0.00380402	0.01849924
PSG3	-1.543058	0.00548885	0.02510507
SIX3	-1.9181654	0.00188737	0.01019608
CST6	-0.6213504	0.00085308	0.00512338
UBB	-0.6747452	0.00083415	0.00502507
GYPB	-2.6381433	0.0060348	0.0272012
CCR9	-1.2557117	0.00180967	0.00983034
TMEM179	-1.0853751	0.00113289	0.00655674
KLK5	-0.7995529	0.00088186	0.00527831
FAM180B	-0.9361926	0.00127485	0.00726893
AQP12B	-1.2328441	0.00207237	0.01105036
PRKG2	-0.820398	0.001134	0.00656101
C11orf87	1.35366678	0.00223044	0.01178662

LY6G6F	-1.8775811	0.00431301	0.02056734
NPAS1	-0.6052127	0.00094102	0.0055908
DCDC2	-0.6084862	0.0009337	0.00556225
RPEL1	-1.9110951	0.00431964	0.02058785
USP26	1.10644364	0.010802	0.04373969
MUC7	-2.7078645	0.00359854	0.01763086
IL17REL	-0.971557	0.00140497	0.00789882
NKG7	-0.6903157	0.00095028	0.0056326
TMPRSS12	-1.4408976	0.00460886	0.02169157
DACH1	-0.6480138	0.00095861	0.00567303
RNASE13	-1.016746	0.00138181	0.00779235
IFI27	-0.6772553	0.00093894	0.00558034
NIPAL4	-0.9289386	0.00149847	0.00832653
PLVAP	-0.5973037	0.00093545	0.00556706
FOXI1	-2.7917556	0.00433786	0.02066354
CEACAM7	1.54902438	0.00243786	0.01272305
CELA1	-1.8209154	0.00386553	0.01876223
WFDC6	-1.7619184	0.00265659	0.01368687
OR7A5	-2.8116543	0.00869365	0.03673306
CDH10	-1.1709307	0.00184065	0.00997413
CRYGC	-2.8190497	0.00348655	0.0171454
ADCY8	-1.2386016	0.00176007	0.0095904
CLSPN	-0.6976244	0.00105892	0.00618273
DOC2B	-0.660295	0.00101594	0.00595982
KRTAP2-3	-3.1773886	0.00491379	0.02285907
KCNH7	1.65063686	0.00491026	0.02284867
HLA-DRB1	-0.6009792	0.00099042	0.00583527
PPP1R14C	-0.7283364	0.00101983	0.0059787
SSX1	-2.3693742	0.00393207	0.01900698
HES5	-0.9823271	0.00153067	0.00848502
AQP5	-0.7644897	0.00101358	0.00594799
CD1A	-1.6255047	0.00253328	0.0131589
SHANK2	-0.7586039	0.00121787	0.00698461

OR51E1	-0.5879891	0.0011157	0.00646589
FRMPD1	-0.729144	0.00130367	0.00741655
PSG9	-1.8243711	0.01110836	0.04473441
SPINK4	-1.6117721	0.00429025	0.02048641
CLPSL2	-2.6269888	0.0055158	0.02522183
BPIFB1	-1.4822466	0.00236525	0.01241003
TRPM6	-0.8713767	0.00174369	0.00951285
MLANA	-0.7096496	0.00120191	0.00689977
PP2D1	-0.7208394	0.00121709	0.00698235
NXF2	-1.2947909	0.00665061	0.02942941
CFHR4	-1.367979	0.00345636	0.01702384
CSTL1	-2.3402817	0.00407762	0.01961404
DAW1	-1.1725292	0.00204721	0.01093267
CCDC63	-1.5480366	0.01028056	0.04206177
MYO3B	-0.9956535	0.00157932	0.00871016
IL9R	1.9470097	0.00297556	0.01505398
FPR3	-0.5932319	0.00114511	0.00661451
VSTM2B	-1.2846537	0.00230002	0.01210725
RDH12	-0.8449011	0.0013229	0.00750902
FAM86B2	-0.7295849	0.00117785	0.00677927
FMR1NB	-1.6030395	0.00459885	0.02165597
OTOP1	-2.5629037	0.00570292	0.02590335
GABRA3	-1.2292979	0.00241889	0.01263898
OTOS	-2.7452215	0.00441022	0.02094057
LIPM	-2.1272775	0.00391915	0.01896523
WNT1	-1.4054502	0.00438269	0.02084342
POTEG	-2.0873344	0.00686307	0.03018973
VWA3A	-0.5982942	0.00127733	0.00728073
EIF4E1B	-1.3401425	0.0028402	0.01447684
CSAG3	-2.2468288	0.00400802	0.01930549
TMEM132D	1.29262988	0.00532976	0.02452329
CYP4F22	-1.5492451	0.00331243	0.01644317
KRTAP1-5	-1.5391602	0.00500354	0.02320335

AQP6	-0.9463067	0.00154985	0.00856897
GFI1B	-1.1801142	0.00361324	0.01768329
KRTAP5-6	-1.3257626	0.00757715	0.032751
FOXJ1	-0.6904497	0.00122052	0.00699297
SPDYE4	-2.504628	0.0092698	0.03868687
CCL5	-0.6920291	0.00123027	0.00704428
ST18	-2.9082483	0.01232133	0.04861197
ANKRD30B	-2.1819821	0.0063558	0.02836616
IFNB1	-1.284488	0.0036986	0.01804611
ERVFRD-1	-1.1741643	0.00307814	0.01548181
IL12B	-0.8992548	0.00852545	0.03616926
KRTAP10-2	-2.2704039	0.00695103	0.03052206
ALOX5AP	-0.5908927	0.00127447	0.00726893
KIR3DL1	-1.19118	0.00417216	0.02000895
ODAM	-1.4392975	0.00430704	0.02054441
CD5L	-1.3636933	0.00338565	0.01673149
SOX3	-1.1984879	0.00208651	0.0111023
GPRIN1	-0.6007651	0.00139215	0.00783673
PROKR2	-1.7288909	0.00402613	0.01937691
NWD1	-0.9691933	0.00172703	0.00943651
CXCL10	-0.6817733	0.001386	0.00780955
PROZ	-0.8593772	0.00149	0.0082873
CD74	-0.5928103	0.00136792	0.00772489
RP11-467J12.4	-0.6460303	0.00139103	0.0078329
C6orf118	-1.0894949	0.00229104	0.01206361
KCTD4	-1.2338902	0.00593524	0.0268071
VWDE	-0.8331645	0.00170898	0.00935814
CYP27C1	-0.6072168	0.00155766	0.0086063
CP	-0.5932502	0.00143982	0.0080589
TMPRSS11A	-1.2528934	0.00396304	0.01911935
FATE1	-0.9125273	0.00214025	0.01135069
PAPPA2	-1.8688066	0.0043408	0.02066564
FRMD7	-1.4226305	0.00492336	0.02288711

GATA1	-0.9872168	0.0025822	0.01338157
FUT6	-1.211969	0.00320698	0.01602313
LCN1	-3.0746575	0.00584597	0.02646472
CD3G	-0.757881	0.00180475	0.00980966
C16orf54	-0.76543	0.0017196	0.00940319
GPR12	-1.0639407	0.00249421	0.01299031
PTX3	-0.596307	0.00156481	0.00863548
LYPD1	-0.6867868	0.0015453	0.00855183
CLNK	-1.2026608	0.00421853	0.02019845
C20orf141	-0.935035	0.00258947	0.01340389
KRT83	-1.2482516	0.00392445	0.01898052
GABRD	-0.6681059	0.00180126	0.00979669
AMY1B	-1.3978249	0.00373716	0.01819912
DNAH3	-0.8799778	0.00234742	0.01232379
SSTR5	-1.1457608	0.00679954	0.02996846
CTD-2006C1.10	-2.1257829	0.00837028	0.03558777
SERPINB10	-1.256735	0.0065262	0.02893678
P2RX3	-1.7531176	0.00469704	0.02201871
PAX2	-1.5103071	0.00309879	0.0155661
GPR87	-1.011695	0.00312395	0.01566734
BLACAT1	-0.5942032	0.00182675	0.0099049
VWA2	-0.7132246	0.00189054	0.01021006
ZG16	-0.904983	0.00361324	0.01768329
METTL11B	-1.8226391	0.00861677	0.03646046
CYP2C8	-0.7454835	0.0019897	0.01066925
AMPD1	-1.0388088	0.002828	0.01442714
GNG13	-1.7250875	0.00567589	0.02581369
SLC14A2	1.49894153	0.00464134	0.0218212
CLECL1	-0.7188335	0.00233107	0.0122452
CLEC4D	-0.8643486	0.00315003	0.01576529
GCSAM	-0.8448033	0.00230642	0.01213374
GJB6	-0.922929	0.00241247	0.0126123
CCL15	-1.0286558	0.00700199	0.0306849

HNF1A	1.09364208	0.00647836	0.02879686
GUCA1C	-1.7041454	0.00648496	0.02880957
MAGEA11	-1.2886012	0.00669186	0.02958235
LY6G6D	-2.0110061	0.00720062	0.03137654
GJB2	-0.7545164	0.0020873	0.01110316
CST11	-2.5058336	0.00820004	0.03497327
SLC28A3	-0.640557	0.00225995	0.01192472
TUBAL3	-1.2388652	0.00630426	0.02818591
TMEM212	0.87813038	0.00621076	0.02786648
EPHX4	-0.5855871	0.0021845	0.01155419
GPR25	-2.3179951	0.008091	0.03460834
TM4SF4	-1.280593	0.0047704	0.02232116
CCKBR	-0.7964923	0.00276404	0.01415799
PSG4	-1.7468628	0.00986801	0.04070325
CD28	-0.6192004	0.00243328	0.01270291
EYA2	-0.5879249	0.00226165	0.01193018
FAM221B	-0.9081048	0.00506486	0.02346312
MYOD1	-1.4467818	0.00703374	0.03079351
UNC5D	1.68940739	0.00568705	0.02584449
MYO3A	-0.8712149	0.00313969	0.01572689
CASP14	-1.2806564	0.00411057	0.01974569
CGB7	-0.7834654	0.00265222	0.01366837
SHISA9	-0.8060448	0.00269923	0.01387423
AADACL2	-1.0908258	0.00764092	0.03296218
TUBA3C	1.14073798	0.00850328	0.03610116
RP11-894J14.5	-0.6114545	0.00262872	0.01358288
CRYBB2	-0.7112773	0.00281457	0.01437103
FAM170A	-1.3744412	0.01230982	0.04857736
LHFPL3	-1.0572061	0.00428409	0.02046253
CGB2	-2.3890102	0.01207433	0.047858
TMEM249	-0.61195	0.00264276	0.01363549
PCDHA12	0.81618171	0.00311004	0.01560652
MEGF10	-0.9818501	0.00325343	0.01619693

CES5A	-2.1485217	0.01198455	0.04756973
CSAG1	-2.8155736	0.01086967	0.04395333
AKAP4	-1.1336049	0.01089574	0.04401848
OLR1	-0.6420646	0.00279127	0.01428095
PAGE5	-2.6143961	0.01257156	0.0494338
TNR	1.52112409	0.01211691	0.0479874
NMBR	-0.7614909	0.00425096	0.02030977
IL2RA	-0.7370411	0.00307649	0.01548049
GPR26	-1.1720453	0.00915239	0.03827818
KCNK9	-0.9381838	0.00392038	0.01896603
SPATA32	-0.9405978	0.0048784	0.02271236
BTBD18	-0.6669832	0.00335983	0.01663641
OSTN	-1.0047814	0.00461577	0.02171832
FCRL5	-0.8598889	0.00357944	0.01755187
LRG1	-0.6026781	0.00281315	0.01436795
KCNJ1	-0.9963193	0.00566914	0.02578963
SPATA12	-0.7118516	0.00347857	0.01711416
EPGN	-0.9989305	0.00545917	0.02498224
COL19A1	3.93901814	0.01007957	0.04143503
PIK3C2G	1.90584036	0.00726828	0.03162989
INHBE	-0.8789044	0.00365191	0.01785275
PCDHA13	-0.7643341	0.00458381	0.02159667
SBK3	-1.0665736	0.00492918	0.02290659
DCSTAMP	-1.1044167	0.00568033	0.02582059
FAM25A	-1.2810369	0.01194067	0.04741742
C11orf97	-1.9253061	0.0092013	0.03842818
KRTAP4-1	-1.5798454	0.01049566	0.04282122
CTAG2	-1.7100187	0.00844154	0.0358649
MAGEC2	-1.5351218	0.00718822	0.03133024
RBM46	1.26000948	0.00931859	0.03882637
FOXA3	-0.7178173	0.00387978	0.01881077
CASKIN1	-0.5900226	0.00312441	0.01566734
RFPL2	-1.0229877	0.00572243	0.02597859

NEUROD2	1.37938065	0.00862556	0.03648022
CLDN19	-0.9921226	0.00482807	0.02253734
CNTN5	-0.9274646	0.00468827	0.02198339
CARD18	-1.783171	0.01177668	0.04691309
TUBB3	-0.6799689	0.00363794	0.01779429
PITX3	-1.4643442	0.01080084	0.04373969
KRTAP5-10	-0.6812558	0.00468188	0.02195929
RLN3	-1.1977993	0.01042287	0.04259457
TMEM169	-0.5908618	0.00331586	0.01645556
ERICH6	-0.7536449	0.00446766	0.02115708
TMPRSS11E	-0.9525113	0.00625883	0.02801113
GABBR2	-0.9061369	0.00570458	0.02590421
XKR5	-0.7270961	0.00394469	0.01904713
CR2	0.92245964	0.00516899	0.02389543
CDK5R2	-1.1172957	0.00655273	0.02904714
ASMT	1.22213262	0.00858603	0.03640008
PTCRA	-1.6585637	0.01197705	0.04755063
OTOR	-1.1646072	0.01171653	0.04674719
CR1L	-0.9865791	0.00738245	0.03205828
AADACL4	-1.4007735	0.0126251	0.04957817
OBP2A	1.01600415	0.00387941	0.01881077
SOX11	-1.0952785	0.00840049	0.03570762
CLC	-1.5310881	0.01235092	0.04868524
KSR2	-1.1400283	0.00914771	0.03827669
SH2D4B	-1.0411663	0.0075231	0.03257308
SAA4	-1.1501994	0.00792494	0.03403799
ATP10B	-0.9271659	0.00528497	0.02435876
SOX30	-0.6873766	0.00429374	0.02049754
LCN8	-1.0243	0.00580074	0.02629361
ILDR2	-0.7804379	0.00448524	0.02122836
RFX4	2.93584042	0.01145282	0.04587092
CAPN14	-0.694605	0.00528336	0.02435876
CYP4F3	-0.7557339	0.0046265	0.021763

FRMPD2	-1.3434013	0.01014967	0.04164168
VCX	-1.0773034	0.00892108	0.03748803
LRRIQ4	-0.8357121	0.00611142	0.02749752
SH2D1A	-0.6867523	0.00536558	0.02464253
IL36RN	1.58744289	0.00973706	0.04024759
CCDC158	-0.8256686	0.00681009	0.02998506
WDR49	-0.7283712	0.00611619	0.02751195
SH2D7	-0.7276126	0.00704807	0.03082573
ZNF804A	-0.7049078	0.00697509	0.03058989
RNF223	-1.0924478	0.01233484	0.04864163
ACRV1	-0.8165516	0.00774888	0.03337095
LIN28B	-1.040403	0.01150083	0.04601114
NKAIN3	-0.8881975	0.00929183	0.03875137
FCRLA	-0.9170323	0.00915739	0.03828265
CCK	-0.7779462	0.01077059	0.04367251
NPHS1	-1.4649499	0.0124354	0.04897458
GRIN2A	-1.0633388	0.00559409	0.02549411
PLA2G2D	-0.8218032	0.00895223	0.03760999
SLC1A2	-0.9720176	0.00746035	0.03234098
GPR15	-0.8527035	0.01104685	0.04453742
SIGLEC8	-0.796964	0.0071773	0.0313027
SYNDIG1	-0.6624051	0.00591655	0.02675664
NELL1	-1.0257485	0.00900358	0.03779881
DDN	-0.7802689	0.00749304	0.03246679
TCF24	-0.7700678	0.00891031	0.03746757
IL21R	-0.6958319	0.00710931	0.03106282
MAB21L2	-0.7825627	0.00970501	0.04015265
FBXW12	-0.6548164	0.00643097	0.02862938
SLC7A10	-0.873306	0.00999397	0.04114604
IL17C	-0.7430712	0.01129756	0.04536066
PPP1R36	-0.6052038	0.00717286	0.03130185
SLC6A11	-0.6724415	0.00873024	0.03685244
CYP26A1	-0.7407735	0.0087147	0.03679564

TMIGD2	-0.8695665	0.01055287	0.04298678
SLC35F3	-0.7268199	0.00845376	0.03590816
LAX1	-0.697378	0.00919033	0.03839146
SHISA8	-0.8721737	0.01255998	0.04939923
TLR8	-0.6312908	0.00901678	0.03783625
ATP2B3	-0.8466833	0.01146112	0.04589376
ADIPOQ	-1.0211909	0.01189633	0.04728319
SYT5	-0.7536105	0.01134809	0.04550299
CDKL4	-0.7999395	0.01050012	0.04282122
MFAP5	-0.7139678	0.00860302	0.03643718
ST8SIA2	-0.666407	0.01260895	0.04954744
STEAP1B	-0.6573328	0.01071102	0.04349078
SFRP2	-0.776224	0.00968088	0.04008091
HCAR2	-0.6998092	0.01053713	0.04294244
NPTX1	-0.7629624	0.01081407	0.04377855
CST1	-0.7736833	0.01060181	0.04316156
P2RY10	-0.6744521	0.0121416	0.04806367
FUT3	-0.5952906	0.01134669	0.04550299
CPNE9	-0.6478046	0.01132733	0.04545065
CLDN16	-0.6231458	0.0106031	0.04316156
COL23A1	-0.6436257	0.01180403	0.04699805

Table S8: Intersection of differentially expressed genes and marker genes of active cell populations to obtain 151 differentially active marker genes

gene	log2FC	pvalue	adjusted_pvalue
KREMEN1	-2.2427757	0	0
SERPINB3	-2.9454729	1.416E-51	2.4316E-49
IL13RA2	-3.1707139	2.2957E-32	1.9807E-30
SAA2	-1.9100078	2.2652E-30	1.8898E-28

PLA2G2A	-1.2791276	1.0159E-16	4.0468E-15
TMEM151A	-2.8348837	4.0832E-16	1.5236E-14
DSC3	-1.5117731	3.3613E-14	1.0215E-12
FDCSP	-3.434953	7.0045E-14	2.0478E-12
VNN1	-1.6018259	9.0618E-14	2.6061E-12
CYTL1	-1.2327654	2.1134E-13	5.9239E-12
CPA4	-2.1727839	3.4542E-13	9.5758E-12
HP	-2.0399361	4.1899E-13	1.1561E-11
ALOX15	-2.5335688	6.472E-13	1.7476E-11
GPC3	-1.4675764	9.0218E-13	2.3959E-11
HOXA5	-1.3363195	4.0858E-12	1.0164E-10
TFPI2	-1.240894	6.4411E-12	1.5627E-10
TDO2	-1.9853274	9.4233E-12	2.2462E-10
BCAT1	-1.8376177	2.2923E-11	5.2518E-10
KLK1	-1.0395546	4.0152E-11	8.9554E-10
C1QL1	-1.5763071	2.2607E-10	4.6603E-09
AADAC	-1.4925562	3.5855E-10	7.182E-09
CXCL6	-2.0390198	7.7501E-10	1.4965E-08
RARRES1	-1.4604153	6.0731E-10	1.1922E-08
HSPB3	-0.619608	8.5469E-10	1.6396E-08
LGALS2	-1.6581095	1.8089E-09	3.2983E-08
RBP4	-0.9040447	3.1486E-09	5.5633E-08
VSIG2	-1.7538918	2.9845E-09	5.2893E-08
UPK1B	-1.9276186	5.2938E-09	9.0378E-08
DLGAP1	-0.6572181	5.6894E-09	9.6358E-08
CTSK	-1.3531645	8.1806E-09	1.356E-07
S100A3	-0.8225191	9.5107E-09	1.5619E-07
DKK1	-0.7175186	1.4619E-08	2.3273E-07
SERPINE1	-1.275969	1.7261E-08	2.7118E-07
THBS2	-1.2468148	2.0608E-08	3.1834E-07
PAEP	-2.5891512	3.9887E-08	5.8352E-07
MFAP4	-1.343223	2.6421E-08	3.9893E-07
SERPINB2	-1.8349007	3.6353E-08	5.3671E-07

SAA1	-1.4145346	3.8512E-08	5.6529E-07
CALB2	-0.9130312	5.5512E-08	7.8926E-07
PTGDS	-1.3219618	6.9203E-08	9.6835E-07
CADM3	-1.3696354	8.7781E-08	1.2064E-06
COL5A2	-1.0577815	8.4249E-08	1.1605E-06
TPPP3	-0.9155034	1.2594E-07	1.6786E-06
COL5A1	-1.2233997	1.3238E-07	1.7578E-06
LTBP4	-0.8042732	1.5319E-07	0.000002016
HSPB6	-0.8066396	1.8181E-07	2.3583E-06
MEST	-0.7740181	1.9914E-07	2.5603E-06
ARC	-0.7408662	2.2373E-07	2.8592E-06
MGP	-0.77341	2.3326E-07	2.9617E-06
APLP1	-1.0592493	3.1005E-07	0.000003851
WNT2	-1.4302109	3.6536E-07	0.000004484
PTN	-1.1959482	3.4435E-07	4.2532E-06
PRR9	-2.2229305	6.1572E-07	0.000007265
UCHL1	-1.2530745	3.621E-07	4.4506E-06
MDFI	-0.8715404	4.7492E-07	5.7104E-06
CLEC11A	-0.7456581	5.5868E-07	6.6273E-06
TNFRSF11B	-1.171323	6.0122E-07	7.0987E-06
CENPW	-0.8851491	5.8501E-07	6.9211E-06
FN1	-1.3079657	7.1835E-07	8.3643E-06
SOD3	-0.8159717	7.7132E-07	8.9516E-06
CA9	-1.3588788	1.0644E-06	0.000012117
ACTA2	-0.9228327	1.1018E-06	0.000012492
FOS	-1.0215882	1.1122E-06	0.000012585
EGFL6	-1.0660278	1.4999E-06	0.000016549
CRYAB	-0.9687495	1.4425E-06	0.000015976
LRRN4	-1.2397449	1.6616E-06	0.000018254
TSPAN7	-0.7969678	1.6914E-06	0.000018535
EGR1	-1.0072732	1.8446E-06	0.000020139
APOA1	-1.2129329	0.00000204	0.000022014
CXCL2	-0.7967563	2.5574E-06	0.000027085

DUSP1	-0.8918372	2.3314E-06	0.000024886
AEBP1	-1.0181243	2.7359E-06	0.000028838
ID1	-0.8059041	2.7877E-06	0.00002934
NMU	1.26492276	3.5651E-06	0.000036832
MMP2	-0.9169733	3.7883E-06	0.000038866
CHI3L1	-0.9344622	3.9675E-06	0.000040422
MMEL1	-0.7579336	4.2946E-06	0.000043331
SULF1	-0.9793876	4.2215E-06	0.000042715
RRAD	-0.632354	4.9865E-06	0.000049885
ARSI	-0.591279	5.5533E-06	0.000055118
SLC40A1	-0.8099544	0.000005365	0.000053399
COL3A1	-1.0980687	5.6653E-06	0.000056167
S100A6	-0.8089834	0.000006293	0.000061801
DCN	-0.7679079	6.3631E-06	0.000062421
CXCL1	-1.192438	6.7915E-06	0.000066219
MXRA8	-0.8795932	6.8753E-06	0.000066925
SPARC	-0.825059	7.8317E-06	0.00007516
EDARADD	-0.6865998	8.4152E-06	0.000080237
COL1A2	-0.981782	8.0648E-06	0.00007723
GRIA2	-0.7769173	0.000010568	0.000098585
LUM	-1.1182864	0.000010681	0.000099531
PCOLCE	-0.6306627	0.0000123	0.00011313
CTHRC1	-0.9867275	0.000012373	0.0001137
COL1A1	-0.9623476	0.000014549	0.00013183
COL6A1	-0.8052628	0.000014745	0.00013319
TMEM176A	-0.620428	0.000019211	0.00016855
TAGLN	-0.7905775	0.000019523	0.0001712
IGFBP5	-0.7970251	0.000020279	0.00017739
EMILIN1	-0.6169356	0.000022203	0.0001925
ICAM1	-0.767295	0.000022396	0.00019408
CXCL14	-1.2344944	0.000023776	0.00020503
CLDN1	-0.7693554	0.000025319	0.00021749
IL10	-0.843326	0.00003142	0.00026577

COL6A2	-0.7590799	0.00003201	0.00027024
CDH2	-0.9549856	0.000032775	0.00027592
BGN	-0.7109524	0.000035027	0.00029223
APOL1	-0.7720398	0.000042502	0.00034899
FST	-0.8684188	0.000048341	0.0003922
MT1E	-0.9884088	0.000045051	0.00036788
GJA1	-0.7271377	0.000051671	0.0004175
CPXM1	-0.7523894	0.000053414	0.00042923
ISLR	-0.8142305	0.000054093	0.00043406
MT2A	-0.6517499	0.000056735	0.00045222
PHLDA2	-0.6927243	0.000057462	0.00045719
TGM1	-0.7184417	0.000075757	0.00059001
CLU	-0.7775106	0.000076421	0.00059492
THBS1	-0.5933581	0.00008079	0.00062563
BDKRB1	-1.0441855	0.00011326	0.00084994
MT1M	-0.7241571	0.00010565	0.00079819
PLAT	-0.6427846	0.00010074	0.00076408
SNCG	-0.9654864	0.00010489	0.00079314
CRB2	-0.8418548	0.00011857	0.00088741
HES4	-0.6120696	0.00011942	0.00089276
MMP14	-0.5955961	0.00012067	0.00090093
UPK3B	-0.8522097	0.00016134	0.00117001
IGFL2	-1.1902113	0.00027369	0.00186831
GDF15	-0.6037369	0.00019885	0.0014119
NNMT	-0.8313538	0.00022185	0.00155213
ID4	-0.6193755	0.00022065	0.00154496
SCG5	-0.652245	0.00023914	0.00165671
RARRES2	-0.5991856	0.0002454	0.00169615
GAS1	-0.5901437	0.00030272	0.00203663
IGFBP2	-0.6041544	0.00030902	0.00207584
THY1	-0.6300666	0.00031076	0.00208512
SLPI	-0.6765311	0.00035295	0.00233282
POSTN	-1.1454242	0.00037358	0.00245906

TNFAIP6	-0.6491889	0.00043574	0.00282821
PRRX2	-0.7262976	0.00043902	0.00284536
CRABP2	-0.6443905	0.00044822	0.00289752
CA12	-0.727597	0.00061798	0.00385817
CDH6	-0.7143555	0.00069964	0.00429373
PDZK1IP1	-0.7198609	0.00073551	0.00449204
KLK11	-0.7141326	0.0007379	0.00450352
DOK5	-0.687252	0.00075813	0.00461742
UBB	-0.6747452	0.00083415	0.00502507
KLK5	-0.7995529	0.00088186	0.00527831
IFI27	-0.6772553	0.00093894	0.00558034
CXCL10	-0.6817733	0.001386	0.00780955
PTX3	-0.596307	0.00156481	0.00863548
GJB2	-0.7545164	0.0020873	0.01110316
EPGN	-0.9989305	0.00545917	0.02498224

Table S9: HALLMARK pathway enrichment analysis of differentially expressed genes by GSEA

ID Description setSize enrichmentScore NES pvalue p.adjust qvalues rank leading_edge core_enrichment

HALLMARK_INTERFERON_GAMMA_RESPONSE HALLMARK_INTERFERON_GAMMA_RESPONSE 33 0.426037 2.698705 3.52E-06 0.000106 0.0001 789 "tags=82%, list=27%,

signal=60%"CD74/HLA-DRB1/RTP4/PSMB9/OAS3/TNFAIP6/MT2A/TNFAIP2/IFI27/CXCL10/HLA-DMA/CCL5/GZMA/IFIT3/OAS2/HLA-DQA1/TNFSF10/GPR18/ICAM1/SECTM1/MX1/GBP6/IFIT1/XCL1/METTL7B/P2RY14/ISG15

HALLMARK_EPITHELIAL_MESENCHYMAL_TRANSITION HALLMARK_EPITHELIAL_MESENCHYMAL_TRANSITION 61 0.296948 2.330671 1.10E-05 0.000165 0.000156 1430 "tags=89%, list=49%, signal=46%" GAS1/THBS1/MMP14/PTX3/IL32/IGFBP2/THY1/PCOLCE/INHBA/COL6A3/TGM2/BGN/SDC1/MFAP5/CDH6/DKK1/GJA1/SPP1/COL6A2/DCN/MGP/MEST/TAGLN/VCAN/SPARC/NNMT/AREG/SFRP1/CXCL8/MMP2/ACTA2/CDH2/COL1A1/COL1A2/CTHRC1/COL5A2/APLP1/NTM/COL3A1/LUM/COMP/POSTN/OXTR/TNFRSF11B/SFRP4/FBN2/CXCL1/COL5A1/TFPI2/THBS2/SERPINE1/SGCG/ANPEP/FN1

Table S10: Identification of differentially expressed active population marker genes using univariate Cox analysis to obtain the 12 prognostic genes associated with ovarian cancer

gene	coef	p.value	Hazard_Ratio	lower_.95	upper_.95	logrank_pvalue	wald_pvalue
FDCSP	-0.1846838	0.04782918	0.83136708	0.69239267	0.9982359	0.04744187	0.04782918
AADAC	-0.2927959	0.000059949	0.74617445	0.64674831	0.8608856	0.000058826	0.000059949
RARRES1	0.08064691	0.02404908	1.08398808	1.01064962	1.1626484	0.02392447	0.02404908
DKK1	0.36161314	0.00187447	1.43564344	1.14302475	1.80317362	0.00199848	0.00187447
FOS	0.09658518	0.03647236	1.10140339	1.0060981	1.20573673	0.0363693	0.03647236
CRYAB	0.08913141	0.03412641	1.09322431	1.00669529	1.1871908	0.03403897	0.03412641
EGR1	0.092237	0.03833536	1.09662469	1.00496813	1.19664064	0.0382301	0.03833536
RRAD	0.10281249	0.04512647	1.10828358	1.00223528	1.22555304	0.04500788	0.04512647
ARSI	0.32102909	0.0177534	1.37854568	1.05719837	1.79757011	0.01754352	0.0177534
GAS1	0.17290078	0.0018766	1.18874815	1.06599087	1.3256419	0.00186787	0.0018766
UBB	-0.1475057	0.00107717	0.86285753	0.78983649	0.94262943	0.00102201	0.00107717

CXCL10	-0.0662393	0.04709373	0.93590688	0.87666696	0.99914988		0.0467964	0.04709373

Table S11: Identification of prognostic gene signatures using Lasso cox analysis

symbol	coef
FDCSP	-0.1855586
AADAC	-0.2258057
RARRES1	0.06190599
DKK1	0.04368029
CRYAB	0.07873196
EGR1	-0.0041924
GAS1	0.08911218
UBB	-0.1522447
CXCL10	-0.0494712

Table S12: Dividing samples into high and low TMB groups according to upper quartile

sample	TMB_log	riskscore	riskgroup
TCGA-10-0937	-0.920818754	-0.977308664	Low
TCGA-10-0928	-0.853871964	0.118253647	High
TCGA-57-1586	-0.853871964	-1.630494817	Low
TCGA-13-0714	-0.657577319	-0.853132512	Low
TCGA-25-1312	-0.619788758	-0.555991411	High
TCGA-13-1485	-0.552841969	-0.015643406	High
TCGA-25-1316	-0.494850022	-0.893932967	Low

TCGA-13-0766	-0.443697499	-0.877210993	Low
TCGA-13-0797	-0.420216403	-0.853007001	Low
TCGA-13-1511	-0.420216403	-0.219630799	High
TCGA-24-2038	-0.420216403	0.157234719	High
TCGA-24-1416	-0.397940009	-0.815204203	Low
TCGA-61-2088	-0.397940009	-0.584454358	High
TCGA-13-0768	-0.37675071	-0.10654785	High
TCGA-13-1495	-0.37675071	-0.435666541	High
TCGA-13-0720	-0.356547324	-0.385060323	High
TCGA-13-0911	-0.356547324	-0.838843582	Low
TCGA-24-0966	-0.337242168	-1.463932918	Low
TCGA-25-2396	-0.337242168	-0.451969165	High
TCGA-WR-A838	-0.318758763	-0.279700588	High
TCGA-09-0364	-0.301029996	-0.337328861	High
TCGA-10-0927	-0.301029996	-0.614512761	High
TCGA-5X-AA5U	-0.283996656	-0.336193993	High

TCGA-25-2409	-0.26760624	-0.703626606	High
TCGA-59-2355	-0.251811973	-0.538035063	High
TCGA-13-0916	-0.236572006	-0.793790653	Low
TCGA-61-1741	-0.22184875	-0.320142786	High
TCGA-13-1506	-0.207608311	-1.019133749	Low
TCGA-24-1562	-0.193820026	-0.716362095	High
TCGA-13-0906	-0.167491087	-1.576610888	Low
TCGA-24-1565	-0.167491087	-0.670940623	High
TCGA-10-0931	-0.142667504	-1.029099063	Low
TCGA-24-1603	-0.13076828	-1.823882044	Low
TCGA-09-1670	-0.107905397	-0.770357937	High
TCGA-13-0765	-0.096910013	0.089382313	High
TCGA-24-1419	-0.096910013	-0.945014023	Low
TCGA-29-1694	-0.096910013	-0.071348901	High
TCGA-24-1467	-0.086186148	-0.955334707	Low
TCGA-09-2045	-0.075720714	-0.772435985	High

TCGA-29-1705	-0.075720714	-0.565640549	High
TCGA-09-1673	-0.065501549	-0.718946725	High
TCGA-13-0804	-0.065501549	-0.396952637	High
TCGA-13-0924	-0.036212173	-1.088774391	Low
TCGA-24-1430	-0.036212173	-0.661021225	High
TCGA-OY-A56Q	-0.036212173	-0.571429741	High
TCGA-09-0367	-0.026872146	-0.672902859	High
TCGA-61-1733	-0.026872146	-0.984785673	Low
TCGA-04-1341	-0.017728767	-0.477100378	High
TCGA-25-1321	-0.017728767	-1.215856061	Low
TCGA-29-A5NZ	-0.017728767	-0.379815652	High
TCGA-20-0987	-0.008773924	-0.748849572	High
TCGA-24-1551	0	-1.100172785	Low
TCGA-25-2398	0	-0.269748541	High
TCGA-29-1697	0	-1.163524308	Low
TCGA-57-1994	0	-1.636599912	Low

TCGA-13-0897	0.017033339	-0.966512037	Low
TCGA-24-1413	0.017033339	-1.405332411	Low
TCGA-29-1768	0.017033339	-0.731498737	High
TCGA-59-2350	0.017033339	-1.088098971	Low
TCGA-61-2002	0.017033339	-1.520995961	Low
TCGA-29-1711	0.025305865	-0.840651066	Low
TCGA-13-0891	0.033423755	-1.057321427	Low
TCGA-23-1809	0.033423755	-0.599731212	High
TCGA-29-1695	0.033423755	-0.905703334	Low
TCGA-61-1738	0.033423755	-0.681411216	High
TCGA-61-1911	0.033423755	-1.239148185	Low
TCGA-24-1471	0.041392685	-1.110030584	Low
TCGA-24-2290	0.049218023	-0.901892118	Low
TCGA-29-1696	0.049218023	-0.768250453	High
TCGA-29-1776	0.049218023	-0.328337741	High
TCGA-61-2101	0.049218023	-1.095683659	Low

TCGA-25-2404	0.056904851	-0.800603124	Low
TCGA-13-0724	0.064457989	-0.683577666	High
TCGA-25-1319	0.064457989	-0.676014635	High
TCGA-25-2399	0.064457989	-0.286424433	High
TCGA-29-1778	0.064457989	-0.680483843	High
TCGA-24-1418	0.071882007	-0.79471646	Low
TCGA-24-1842	0.071882007	-0.649142917	High
TCGA-24-2036	0.071882007	-0.759834241	High
TCGA-23-1027	0.079181246	-1.028298625	Low
TCGA-24-1550	0.079181246	-1.066928378	Low
TCGA-29-1785	0.079181246	-0.816239128	Low
TCGA-13-1403	0.086359831	-0.540236306	High
TCGA-13-1404	0.086359831	-0.784942901	High
TCGA-24-1552	0.086359831	0.346055864	High
TCGA-29-1710	0.086359831	-0.17443323	High
TCGA-24-1464	0.093421685	-0.8592932	Low

TCGA-24-1616	0.093421685	-0.35514895	High
TCGA-24-2261	0.093421685	-1.288590931	Low
TCGA-13-0886	0.100370545	-1.225490511	Low
TCGA-13-1512	0.100370545	-0.902094563	Low
TCGA-30-1891	0.100370545	-0.448219247	High
TCGA-23-1111	0.10720997	0.17215153	High
TCGA-13-1411	0.113943352	-0.851253198	Low
TCGA-20-1687	0.113943352	-0.260227427	High
TCGA-23-1113	0.113943352	-0.659887784	High
TCGA-24-1103	0.120573931	-1.299246307	Low
TCGA-24-1427	0.120573931	-0.361839969	High
TCGA-29-2427	0.120573931	-1.316284385	Low
TCGA-04-1542	0.127104798	-1.265641417	Low
TCGA-13-1496	0.127104798	-1.124003647	Low
TCGA-29-1703	0.127104798	-0.221310386	High
TCGA-61-2104	0.133538908	-1.008711174	Low

TCGA-13-0923	0.139879086	-1.10520478	Low
TCGA-30-1714	0.139879086	-0.927277525	Low
TCGA-59-2354	0.146128036	-0.745834844	High
TCGA-61-2102	0.146128036	0.380184772	High
TCGA-29-1688	0.152288344	-0.66854042	High
TCGA-13-1499	0.158362492	-1.965869578	Low
TCGA-24-1424	0.158362492	-0.646595444	High
TCGA-04-1332	0.164352856	-0.432708011	High
TCGA-13-1409	0.164352856	-0.595095579	High
TCGA-29-1783	0.164352856	-0.766343284	High
TCGA-24-1470	0.170261715	-1.249629587	Low
TCGA-VG-A8LO	0.170261715	-0.867674708	Low
TCGA-04-1655	0.176091259	-0.068643844	High
TCGA-25-2391	0.176091259	-0.227968857	High
TCGA-13-0883	0.181843588	-0.599402361	High
TCGA-13-1405	0.181843588	-0.438048212	High

TCGA-13-2060	0.181843588	-1.455524592	Low
TCGA-23-1030	0.187520721	-0.791146069	Low
TCGA-24-1474	0.187520721	-0.49073913	High
TCGA-61-2008	0.187520721	-0.637061699	High
TCGA-04-1651	0.193124598	-1.427652929	Low
TCGA-25-1328	0.193124598	-0.539541317	High
TCGA-59-2348	0.193124598	-0.876538201	Low
TCGA-13-A5FT	0.198657087	-1.130000451	Low
TCGA-29-1769	0.198657087	-1.055683355	Low
TCGA-25-1315	0.204119983	-1.155057745	Low
TCGA-04-1362	0.209515015	-0.920061531	Low
TCGA-29-1762	0.209515015	-0.96597436	Low
TCGA-13-1483	0.214843848	-1.045718107	Low
TCGA-24-1417	0.214843848	-1.236356086	Low
TCGA-24-1843	0.214843848	-0.930391808	Low
TCGA-25-2401	0.214843848	-1.160856941	Low

TCGA-59-2352	0.220108088	-0.639122736	High
TCGA-59-2363	0.220108088	-0.244603531	High
TCGA-61-2109	0.220108088	-1.523427287	Low
TCGA-13-0795	0.225309282	-0.392051771	High
TCGA-13-1507	0.225309282	-0.815196963	Low
TCGA-20-1683	0.225309282	0.060691575	High
TCGA-24-1434	0.225309282	-0.394334109	High
TCGA-61-1910	0.225309282	-1.150834759	Low
TCGA-23-1123	0.230448921	-0.950508096	Low
TCGA-25-2393	0.230448921	-1.357300322	Low
TCGA-13-0726	0.235528447	-0.799795859	Low
TCGA-29-1774	0.240549248	-0.883152265	Low
TCGA-13-1501	0.245512668	-1.121066072	Low
TCGA-61-1900	0.250420002	-0.67884585	High
TCGA-09-0366	0.255272505	-0.838703635	Low
TCGA-13-1509	0.255272505	-1.342101591	Low

TCGA-13-1487	0.264817823	-0.275410863	High
TCGA-30-1853	0.264817823	0.175884977	High
TCGA-25-1329	0.274157849	0.018794058	High
TCGA-20-1686	0.278753601	-0.971696378	Low
TCGA-61-2009	0.278753601	-0.677459406	High
TCGA-13-1410	0.28780173	-0.966541568	Low
TCGA-31-1959	0.28780173	-0.892902451	Low
TCGA-10-0933	0.292256071	-0.631726572	High
TCGA-20-1682	0.292256071	-2.377441431	Low
TCGA-24-1428	0.292256071	-1.054274386	Low
TCGA-61-2092	0.301029996	-1.184083682	Low
TCGA-30-1862	0.309630167	-0.413716326	High
TCGA-04-1356	0.31386722	-1.326957915	Low
TCGA-13-1489	0.31386722	-1.620456965	Low
TCGA-29-1781	0.31386722	-0.554390139	High
TCGA-24-0979	0.326335861	-0.284991427	High

TCGA-24-1104	0.326335861	-0.461279727	High
TCGA-30-1857	0.334453751	-0.586100478	High
TCGA-61-1907	0.334453751	-1.227131046	Low
TCGA-13-1505	0.338456494	-1.058994125	Low
TCGA-24-1847	0.338456494	-0.773565012	High
TCGA-24-1423	0.346352974	-1.169421778	Low
TCGA-24-1435	0.346352974	-0.763685978	High
TCGA-24-2254	0.346352974	-0.487894404	High
TCGA-24-1850	0.350248018	-0.504291971	High
TCGA-25-2400	0.354108439	-0.346896692	High
TCGA-24-2033	0.365487985	-0.33350082	High
TCGA-13-1498	0.369215857	-0.960678774	Low
TCGA-61-1725	0.372912003	-0.955932727	Low
TCGA-23-1118	0.376576957	-1.257918876	Low
TCGA-31-1950	0.383815366	-1.004085507	Low
TCGA-31-1953	0.387389826	-0.892795373	Low

TCGA-29-1701	0.394451681	-1.083364817	Low
TCGA-61-1995	0.397940009	-0.341532433	High
TCGA-29-1784	0.401400541	-1.249954797	Low
TCGA-04-1648	0.408239965	-0.156868377	High
TCGA-59-2351	0.408239965	-1.012749514	Low
TCGA-61-1914	0.411619706	-1.293994871	Low
TCGA-13-1510	0.414973348	-1.005154808	Low
TCGA-24-2271	0.414973348	-1.265248059	Low
TCGA-61-1728	0.418301291	-0.068973288	High
TCGA-04-1343	0.421603927	-0.946590252	Low
TCGA-25-1323	0.421603927	-0.397728947	High
TCGA-25-2392	0.421603927	-0.590238813	High
TCGA-29-1777	0.421603927	-1.427468499	Low
TCGA-04-1347	0.424881637	-1.394779704	Low
TCGA-24-2267	0.424881637	-0.711227389	High
TCGA-59-A5PD	0.437750563	-1.10784039	Low

TCGA-13-1497	0.440909082	-0.590826062	High
TCGA-25-1320	0.45331834	-1.048397966	Low
TCGA-09-0369	0.462397998	-0.156645403	High
TCGA-24-1425	0.465382851	-0.625189329	High
TCGA-13-0887	0.471291711	-1.129349735	Low
TCGA-23-2077	0.471291711	-1.140080562	Low
TCGA-29-1766	0.471291711	-0.80603764	Low
TCGA-24-2024	0.477121255	-0.644618441	High
TCGA-61-2000	0.496929648	-0.834376076	Low
TCGA-29-1693	0.499687083	-1.513324279	Low
TCGA-24-0982	0.50242712	-0.812638848	Low
TCGA-23-1122	0.507855872	-0.957631318	Low
TCGA-24-2281	0.507855872	-1.095281186	Low
TCGA-13-1488	0.5132176	-2.591186223	Low
TCGA-24-1844	0.515873844	-0.83861623	Low
TCGA-24-2262	0.51851394	-0.73876012	High

TCGA-61-1919	0.51851394	-1.457601256	Low
TCGA-04-1331	0.526339277	-0.323527916	High
TCGA-61-1737	0.526339277	-0.680269525	High
TCGA-57-1993	0.541579244	-0.104337513	High
TCGA-24-1846	0.553883027	-0.977361625	Low
TCGA-30-1718	0.565847819	-1.255263167	Low
TCGA-13-0885	0.572871602	-1.37460334	Low
TCGA-13-0888	0.575187845	-0.621714655	High
TCGA-24-2289	0.588831726	-0.751564004	High
TCGA-24-2298	0.588831726	-0.231398387	High
TCGA-29-1761	0.588831726	-0.900826504	Low
TCGA-61-2012	0.588831726	-0.707910304	High
TCGA-61-2111	0.588831726	-1.074435923	Low
TCGA-04-1361	0.595496222	-0.883053984	Low
TCGA-13-0800	0.595496222	-1.383754371	Low
TCGA-23-1022	0.599883072	-1.070730854	Low

TCGA-29-1691	0.631443769	-0.822562705	Low
TCGA-24-1469	0.643452676	-1.2998347	Low
TCGA-24-1422	0.704150517	-0.769972417	High
TCGA-25-1313	0.712649702	-0.823865914	Low
TCGA-24-2288	0.727541257	-0.052918998	High
TCGA-24-2035	0.764922985	-1.565094785	Low
TCGA-23-2084	0.766412847	-0.704937649	High
TCGA-24-2280	0.770852012	-0.518646469	High
TCGA-23-1110	0.77524626	-0.533319505	High
TCGA-25-1326	0.797959644	-0.860129923	Low
TCGA-24-2293	0.802089258	-0.717729669	High
TCGA-29-1770	0.810232518	-1.303646717	Low
TCGA-13-0920	0.829946696	-1.621648386	Low
TCGA-61-2003	0.833784375	-0.378513718	High
TCGA-13-1477	0.858537198	-0.111371546	High
TCGA-25-2042	0.914871818	-0.365945964	High

TCGA-61-2110	0.920123326	-1.024939778	Low
TCGA-23-1116	0.926342447	-0.608917827	High
TCGA-29-1763	0.928395852	-0.814248537	Low
TCGA-24-1845	0.966610987	-1.127130723	Low
TCGA-23-2078	0.992111488	-1.466187256	Low
TCGA-61-1998	0.994756945	-0.767660558	High
TCGA-09-2051	1.037426498	-1.141528347	Low
TCGA-24-1426	1.037426498	-0.346483463	High
TCGA-13-1492	1.038222638	-0.918794866	Low
TCGA-24-1431	1.168497484	-0.234423987	High
TCGA-61-2113	1.176669933	-0.617750798	High
TCGA-20-0991	1.19368103	-0.98728661	Low

Table S13: The proportion of 22 immune infiltrating cells in the high expression group of OV samples was estimated using the CIBERSORT algorithm

HighSamples	Celltype	Fraction
TCGA-04-1331-01A	B_cells_naive	0.064124023
TCGA-04-1331-01A	B_cells_memory	0
TCGA-04-1331-01A	Plasma_cells	0.000703522
TCGA-04-1331-01A	T_cells_CD8	0.01851536
TCGA-04-1331-01A	T_cells_CD4_naive	0
TCGA-04-1331-01A	T_cells_CD4_memory_resting	0.183748744
TCGA-04-1331-01A	T_cells_CD4_memory_activated	0
TCGA-04-1331-01A	T_cells_follicular_helper	0.087365716
TCGA-04-1331-01A	T_cells_regulatory_(Tregs)	0.044562701
TCGA-04-1331-01A	T_cells_gamma_delta	0

TCGA-04-1331-01A	NK_cells_resting	0
TCGA-04-1331-01A	NK_cells_activated	0.052207722
TCGA-04-1331-01A	Monocytes	0.015573902
TCGA-04-1331-01A	Macrophages_M0	0.260994201
TCGA-04-1331-01A	Macrophages_M1	0.162005813
TCGA-04-1331-01A	Macrophages_M2	0.10688744
TCGA-04-1331-01A	Dendritic_cells_resting	0
TCGA-04-1331-01A	Dendritic_cells_activated	0
TCGA-04-1331-01A	Mast_cells_resting	0
TCGA-04-1331-01A	Mast_cells_activated	0
TCGA-04-1331-01A	Eosinophils	0.003310855
TCGA-04-1331-01A	Neutrophils	0
TCGA-04-1332-01A	B_cells_naive	0.06162598
TCGA-04-1332-01A	B_cells_memory	0
TCGA-04-1332-01A	Plasma_cells	0.014076426
TCGA-04-1332-01A	T_cells_CD8	0.039977007
TCGA-04-1332-01A	T_cells_CD4_naive	0
TCGA-04-1332-01A	T_cells_CD4_memory_resting	0.265299737
TCGA-04-1332-01A	T_cells_CD4_memory_activated	0
TCGA-04-1332-01A	T_cells_follicular_helper	0.020740035
TCGA-04-1332-01A	T_cells_regulatory_(Tregs)	0.005448038
TCGA-04-1332-01A	T_cells_gamma_delta	0
TCGA-04-1332-01A	NK_cells_resting	0
TCGA-04-1332-01A	NK_cells_activated	0.052185964
TCGA-04-1332-01A	Monocytes	0.074116045
TCGA-04-1332-01A	Macrophages_M0	0.057748785
TCGA-04-1332-01A	Macrophages_M1	0.051472901
TCGA-04-1332-01A	Macrophages_M2	0.225939001
TCGA-04-1332-01A	Dendritic_cells_resting	0.012511708
TCGA-04-1332-01A	Dendritic_cells_activated	0.013225187
TCGA-04-1332-01A	Mast_cells_resting	0.070427271
TCGA-04-1332-01A	Mast_cells_activated	0.029927472
TCGA-04-1332-01A	Eosinophils	0

TCGA-04-1332-01A	Neutrophils	0.005278444
TCGA-04-1341-01A	B_cells_naive	0.060640384
TCGA-04-1341-01A	B_cells_memory	0
TCGA-04-1341-01A	Plasma_cells	0.391051632
TCGA-04-1341-01A	T_cells_CD8	0.090028148
TCGA-04-1341-01A	T_cells_CD4_naive	0
TCGA-04-1341-01A	T_cells_CD4_memory_resting	0.097023117
TCGA-04-1341-01A	T_cells_CD4_memory_activated	0
TCGA-04-1341-01A	T_cells_follicular_helper	0.074335408
TCGA-04-1341-01A	T_cells_regulatory_(Tregs)	0.032208285
TCGA-04-1341-01A	T_cells_gamma_delta	0
TCGA-04-1341-01A	NK_cells_resting	0
TCGA-04-1341-01A	NK_cells_activated	0.056973548
TCGA-04-1341-01A	Monocytes	0
TCGA-04-1341-01A	Macrophages_M0	0.084359156
TCGA-04-1341-01A	Macrophages_M1	0.047434494
TCGA-04-1341-01A	Macrophages_M2	0.041749634
TCGA-04-1341-01A	Dendritic_cells_resting	0
TCGA-04-1341-01A	Dendritic_cells_activated	0.009918327
TCGA-04-1341-01A	Mast_cells_resting	0
TCGA-04-1341-01A	Mast_cells_activated	0.014277866
TCGA-04-1341-01A	Eosinophils	0
TCGA-04-1341-01A	Neutrophils	0
TCGA-04-1350-01A	B_cells_naive	0
TCGA-04-1350-01A	B_cells_memory	0
TCGA-04-1350-01A	Plasma_cells	0.073907221
TCGA-04-1350-01A	T_cells_CD8	0.007848873
TCGA-04-1350-01A	T_cells_CD4_naive	0
TCGA-04-1350-01A	T_cells_CD4_memory_resting	0.095114773
TCGA-04-1350-01A	T_cells_CD4_memory_activated	0
TCGA-04-1350-01A	T_cells_follicular_helper	0.037187684
TCGA-04-1350-01A	T_cells_regulatory_(Tregs)	0.029883472
TCGA-04-1350-01A	T_cells_gamma_delta	0.10042106

TCGA-04-1350-01A	NK_cells_resting	0
TCGA-04-1350-01A	NK_cells_activated	0
TCGA-04-1350-01A	Monocytes	0
TCGA-04-1350-01A	Macrophages_M0	0.355474507
TCGA-04-1350-01A	Macrophages_M1	0.025200289
TCGA-04-1350-01A	Macrophages_M2	0
TCGA-04-1350-01A	Dendritic_cells_resting	0
TCGA-04-1350-01A	Dendritic_cells_activated	0
TCGA-04-1350-01A	Mast_cells_resting	0.268562917
TCGA-04-1350-01A	Mast_cells_activated	0
TCGA-04-1350-01A	Eosinophils	0
TCGA-04-1350-01A	Neutrophils	0.006399204
TCGA-04-1364-01A	B_cells_naive	0.355071917
TCGA-04-1364-01A	B_cells_memory	0
TCGA-04-1364-01A	Plasma_cells	0.006220817
TCGA-04-1364-01A	T_cells_CD8	0.04648388
TCGA-04-1364-01A	T_cells_CD4_naive	0
TCGA-04-1364-01A	T_cells_CD4_memory_resting	0.147782673
TCGA-04-1364-01A	T_cells_CD4_memory_activated	0
TCGA-04-1364-01A	T_cells_follicular_helper	0.017326493
TCGA-04-1364-01A	T_cells_regulatory_(Tregs)	0
TCGA-04-1364-01A	T_cells_gamma_delta	0
TCGA-04-1364-01A	NK_cells_resting	0.092285169
TCGA-04-1364-01A	NK_cells_activated	0
TCGA-04-1364-01A	Monocytes	0
TCGA-04-1364-01A	Macrophages_M0	0.320597162
TCGA-04-1364-01A	Macrophages_M1	0.010405738
TCGA-04-1364-01A	Macrophages_M2	0.003826151
TCGA-04-1364-01A	Dendritic_cells_resting	0
TCGA-04-1364-01A	Dendritic_cells_activated	0
TCGA-04-1364-01A	Mast_cells_resting	0
TCGA-04-1364-01A	Mast_cells_activated	0
TCGA-04-1364-01A	Eosinophils	0

TCGA-04-1364-01A	Neutrophils	0
TCGA-04-1536-01A	B_cells_naive	0
TCGA-04-1536-01A	B_cells_memory	0.020896805
TCGA-04-1536-01A	Plasma_cells	0
TCGA-04-1536-01A	T_cells_CD8	0.049139668
TCGA-04-1536-01A	T_cells_CD4_naive	0
TCGA-04-1536-01A	T_cells_CD4_memory_resting	0.200410516
TCGA-04-1536-01A	T_cells_CD4_memory_activated	0
TCGA-04-1536-01A	T_cells_follicular_helper	0.003302987
TCGA-04-1536-01A	T_cells_regulatory_(Tregs)	0.0664436
TCGA-04-1536-01A	T_cells_gamma_delta	0.007983787
TCGA-04-1536-01A	NK_cells_resting	0
TCGA-04-1536-01A	NK_cells_activated	0.033910333
TCGA-04-1536-01A	Monocytes	0.102856916
TCGA-04-1536-01A	Macrophages_M0	0.101733642
TCGA-04-1536-01A	Macrophages_M1	0.035596963
TCGA-04-1536-01A	Macrophages_M2	0.363010239
TCGA-04-1536-01A	Dendritic_cells_resting	0
TCGA-04-1536-01A	Dendritic_cells_activated	0
TCGA-04-1536-01A	Mast_cells_resting	0
TCGA-04-1536-01A	Mast_cells_activated	0.014714544
TCGA-04-1536-01A	Eosinophils	0
TCGA-04-1536-01A	Neutrophils	0
TCGA-04-1648-01A	B_cells_naive	0.102215654
TCGA-04-1648-01A	B_cells_memory	0
TCGA-04-1648-01A	Plasma_cells	0.025970556
TCGA-04-1648-01A	T_cells_CD8	0.039185507
TCGA-04-1648-01A	T_cells_CD4_naive	0
TCGA-04-1648-01A	T_cells_CD4_memory_resting	0.242144555
TCGA-04-1648-01A	T_cells_CD4_memory_activated	0
TCGA-04-1648-01A	T_cells_follicular_helper	0.022576649
TCGA-04-1648-01A	T_cells_regulatory_(Tregs)	0.053190529
TCGA-04-1648-01A	T_cells_gamma_delta	0

TCGA-04-1648-01A	NK_cells_resting	0.007251549
TCGA-04-1648-01A	NK_cells_activated	0.066291963
TCGA-04-1648-01A	Monocytes	0.158636263
TCGA-04-1648-01A	Macrophages_M0	0.052542684
TCGA-04-1648-01A	Macrophages_M1	0.090771134
TCGA-04-1648-01A	Macrophages_M2	0.119167667
TCGA-04-1648-01A	Dendritic_cells_resting	0.008654008
TCGA-04-1648-01A	Dendritic_cells_activated	0
TCGA-04-1648-01A	Mast_cells_resting	0.011401282
TCGA-04-1648-01A	Mast_cells_activated	0
TCGA-04-1648-01A	Eosinophils	0
TCGA-04-1648-01A	Neutrophils	0
TCGA-04-1655-01A	B_cells_naive	0
TCGA-04-1655-01A	B_cells_memory	0.044381399
TCGA-04-1655-01A	Plasma_cells	0.083475264
TCGA-04-1655-01A	T_cells_CD8	0.04611406
TCGA-04-1655-01A	T_cells_CD4_naive	0
TCGA-04-1655-01A	T_cells_CD4_memory_resting	0.107951944
TCGA-04-1655-01A	T_cells_CD4_memory_activated	0
TCGA-04-1655-01A	T_cells_follicular_helper	0.053623775
TCGA-04-1655-01A	T_cells_regulatory_(Tregs)	0
TCGA-04-1655-01A	T_cells_gamma_delta	0
TCGA-04-1655-01A	NK_cells_resting	0
TCGA-04-1655-01A	NK_cells_activated	0.13316102
TCGA-04-1655-01A	Monocytes	0
TCGA-04-1655-01A	Macrophages_M0	0.171159437
TCGA-04-1655-01A	Macrophages_M1	0
TCGA-04-1655-01A	Macrophages_M2	0
TCGA-04-1655-01A	Dendritic_cells_resting	0
TCGA-04-1655-01A	Dendritic_cells_activated	0
TCGA-04-1655-01A	Mast_cells_resting	0.134199666
TCGA-04-1655-01A	Mast_cells_activated	0
TCGA-04-1655-01A	Eosinophils	0.225933436

TCGA-04-1655-01A	Neutrophils	0
TCGA-09-0364-01A	B_cells_naive	0.328714903
TCGA-09-0364-01A	B_cells_memory	0
TCGA-09-0364-01A	Plasma_cells	0.029995003
TCGA-09-0364-01A	T_cells_CD8	0.019116319
TCGA-09-0364-01A	T_cells_CD4_naive	0
TCGA-09-0364-01A	T_cells_CD4_memory_resting	0.288608378
TCGA-09-0364-01A	T_cells_CD4_memory_activated	0
TCGA-09-0364-01A	T_cells_follicular_helper	0
TCGA-09-0364-01A	T_cells_regulatory_(Tregs)	0
TCGA-09-0364-01A	T_cells_gamma_delta	0
TCGA-09-0364-01A	NK_cells_resting	0
TCGA-09-0364-01A	NK_cells_activated	0.054263178
TCGA-09-0364-01A	Monocytes	0.043405138
TCGA-09-0364-01A	Macrophages_M0	0.019021
TCGA-09-0364-01A	Macrophages_M1	0
TCGA-09-0364-01A	Macrophages_M2	0.216876081
TCGA-09-0364-01A	Dendritic_cells_resting	0
TCGA-09-0364-01A	Dendritic_cells_activated	0
TCGA-09-0364-01A	Mast_cells_resting	0
TCGA-09-0364-01A	Mast_cells_activated	0
TCGA-09-0364-01A	Eosinophils	0
TCGA-09-0364-01A	Neutrophils	0
TCGA-09-0367-01A	B_cells_naive	0
TCGA-09-0367-01A	B_cells_memory	0.033968547
TCGA-09-0367-01A	Plasma_cells	0.001129611
TCGA-09-0367-01A	T_cells_CD8	0.049330931
TCGA-09-0367-01A	T_cells_CD4_naive	0
TCGA-09-0367-01A	T_cells_CD4_memory_resting	0.190737742
TCGA-09-0367-01A	T_cells_CD4_memory_activated	0
TCGA-09-0367-01A	T_cells_follicular_helper	0.019332016
TCGA-09-0367-01A	T_cells_regulatory_(Tregs)	0.039361729
TCGA-09-0367-01A	T_cells_gamma_delta	0

TCGA-09-0367-01A	NK_cells_resting	0
TCGA-09-0367-01A	NK_cells_activated	0.094264232
TCGA-09-0367-01A	Monocytes	0.012800159
TCGA-09-0367-01A	Macrophages_M0	0.188736737
TCGA-09-0367-01A	Macrophages_M1	0.094162902
TCGA-09-0367-01A	Macrophages_M2	0.18062225
TCGA-09-0367-01A	Dendritic_cells_resting	0.073923541
TCGA-09-0367-01A	Dendritic_cells_activated	0
TCGA-09-0367-01A	Mast_cells_resting	0
TCGA-09-0367-01A	Mast_cells_activated	0.021454717
TCGA-09-0367-01A	Eosinophils	0
TCGA-09-0367-01A	Neutrophils	0.000174885
TCGA-09-0369-01A	B_cells_naive	0.031163389
TCGA-09-0369-01A	B_cells_memory	0
TCGA-09-0369-01A	Plasma_cells	0.00167055
TCGA-09-0369-01A	T_cells_CD8	0
TCGA-09-0369-01A	T_cells_CD4_naive	0
TCGA-09-0369-01A	T_cells_CD4_memory_resting	0.156353198
TCGA-09-0369-01A	T_cells_CD4_memory_activated	0.000151752
TCGA-09-0369-01A	T_cells_follicular_helper	0.00118566
TCGA-09-0369-01A	T_cells_regulatory_(Tregs)	0.069522131
TCGA-09-0369-01A	T_cells_gamma_delta	0
TCGA-09-0369-01A	NK_cells_resting	0.034881163
TCGA-09-0369-01A	NK_cells_activated	0
TCGA-09-0369-01A	Monocytes	0
TCGA-09-0369-01A	Macrophages_M0	0.41393528
TCGA-09-0369-01A	Macrophages_M1	0.022933517
TCGA-09-0369-01A	Macrophages_M2	0.267735325
TCGA-09-0369-01A	Dendritic_cells_resting	0
TCGA-09-0369-01A	Dendritic_cells_activated	0
TCGA-09-0369-01A	Mast_cells_resting	0
TCGA-09-0369-01A	Mast_cells_activated	0.000468034
TCGA-09-0369-01A	Eosinophils	0

TCGA-09-0369-01A	Neutrophils	0
TCGA-09-1662-01A	B_cells_naive	0.000793316
TCGA-09-1662-01A	B_cells_memory	0
TCGA-09-1662-01A	Plasma_cells	0
TCGA-09-1662-01A	T_cells_CD8	0.029088644
TCGA-09-1662-01A	T_cells_CD4_naive	0
TCGA-09-1662-01A	T_cells_CD4_memory_resting	0.245865129
TCGA-09-1662-01A	T_cells_CD4_memory_activated	0
TCGA-09-1662-01A	T_cells_follicular_helper	0.005934576
TCGA-09-1662-01A	T_cells_regulatory_(Tregs)	0.050079162
TCGA-09-1662-01A	T_cells_gamma_delta	0
TCGA-09-1662-01A	NK_cells_resting	0
TCGA-09-1662-01A	NK_cells_activated	0.066519558
TCGA-09-1662-01A	Monocytes	0.069465339
TCGA-09-1662-01A	Macrophages_M0	0.342804197
TCGA-09-1662-01A	Macrophages_M1	0.052528857
TCGA-09-1662-01A	Macrophages_M2	0.109239292
TCGA-09-1662-01A	Dendritic_cells_resting	0.021970943
TCGA-09-1662-01A	Dendritic_cells_activated	0
TCGA-09-1662-01A	Mast_cells_resting	0.005126854
TCGA-09-1662-01A	Mast_cells_activated	0
TCGA-09-1662-01A	Eosinophils	0
TCGA-09-1662-01A	Neutrophils	0.000584132
TCGA-09-1670-01A	B_cells_naive	0
TCGA-09-1670-01A	B_cells_memory	0.062053431
TCGA-09-1670-01A	Plasma_cells	0.002719919
TCGA-09-1670-01A	T_cells_CD8	0.033617941
TCGA-09-1670-01A	T_cells_CD4_naive	0
TCGA-09-1670-01A	T_cells_CD4_memory_resting	0.291777981
TCGA-09-1670-01A	T_cells_CD4_memory_activated	0
TCGA-09-1670-01A	T_cells_follicular_helper	0.013890495
TCGA-09-1670-01A	T_cells_regulatory_(Tregs)	0.047881188
TCGA-09-1670-01A	T_cells_gamma_delta	0

TCGA-09-1670-01A	NK_cells_resting	0
TCGA-09-1670-01A	NK_cells_activated	0.068305253
TCGA-09-1670-01A	Monocytes	0.011247267
TCGA-09-1670-01A	Macrophages_M0	0
TCGA-09-1670-01A	Macrophages_M1	0.090374025
TCGA-09-1670-01A	Macrophages_M2	0.326031016
TCGA-09-1670-01A	Dendritic_cells_resting	0.001268352
TCGA-09-1670-01A	Dendritic_cells_activated	0.012016611
TCGA-09-1670-01A	Mast_cells_resting	0.009354682
TCGA-09-1670-01A	Mast_cells_activated	0
TCGA-09-1670-01A	Eosinophils	0
TCGA-09-1670-01A	Neutrophils	0.029461838
TCGA-09-1673-01A	B_cells_naive	0
TCGA-09-1673-01A	B_cells_memory	0.065212361
TCGA-09-1673-01A	Plasma_cells	0
TCGA-09-1673-01A	T_cells_CD8	0.080147682
TCGA-09-1673-01A	T_cells_CD4_naive	0
TCGA-09-1673-01A	T_cells_CD4_memory_resting	0.093595019
TCGA-09-1673-01A	T_cells_CD4_memory_activated	0
TCGA-09-1673-01A	T_cells_follicular_helper	0.040667737
TCGA-09-1673-01A	T_cells_regulatory_(Tregs)	0.054387691
TCGA-09-1673-01A	T_cells_gamma_delta	0
TCGA-09-1673-01A	NK_cells_resting	0
TCGA-09-1673-01A	NK_cells_activated	0.02052212
TCGA-09-1673-01A	Monocytes	0.025649823
TCGA-09-1673-01A	Macrophages_M0	0.124646822
TCGA-09-1673-01A	Macrophages_M1	0.059175489
TCGA-09-1673-01A	Macrophages_M2	0.282425218
TCGA-09-1673-01A	Dendritic_cells_resting	0.0040271
TCGA-09-1673-01A	Dendritic_cells_activated	0
TCGA-09-1673-01A	Mast_cells_resting	0.13254134
TCGA-09-1673-01A	Mast_cells_activated	0
TCGA-09-1673-01A	Eosinophils	0.008899313

TCGA-09-1673-01A	Neutrophils	0.008102286
TCGA-09-2045-01A	B_cells_naive	0.052481961
TCGA-09-2045-01A	B_cells_memory	0
TCGA-09-2045-01A	Plasma_cells	0.008628261
TCGA-09-2045-01A	T_cells_CD8	0.048957272
TCGA-09-2045-01A	T_cells_CD4_naive	0
TCGA-09-2045-01A	T_cells_CD4_memory_resting	0.226236023
TCGA-09-2045-01A	T_cells_CD4_memory_activated	0
TCGA-09-2045-01A	T_cells_follicular_helper	0.029869676
TCGA-09-2045-01A	T_cells_regulatory_(Tregs)	0.088276884
TCGA-09-2045-01A	T_cells_gamma_delta	0
TCGA-09-2045-01A	NK_cells_resting	0
TCGA-09-2045-01A	NK_cells_activated	0.09833821
TCGA-09-2045-01A	Monocytes	0.024503129
TCGA-09-2045-01A	Macrophages_M0	0.070647162
TCGA-09-2045-01A	Macrophages_M1	0.012792314
TCGA-09-2045-01A	Macrophages_M2	0.191323097
TCGA-09-2045-01A	Dendritic_cells_resting	0
TCGA-09-2045-01A	Dendritic_cells_activated	0.107709055
TCGA-09-2045-01A	Mast_cells_resting	0.032431501
TCGA-09-2045-01A	Mast_cells_activated	0
TCGA-09-2045-01A	Eosinophils	0
TCGA-09-2045-01A	Neutrophils	0.007805454
TCGA-09-2054-01A	B_cells_naive	0.085491374
TCGA-09-2054-01A	B_cells_memory	0
TCGA-09-2054-01A	Plasma_cells	0.150030798
TCGA-09-2054-01A	T_cells_CD8	0.073882457
TCGA-09-2054-01A	T_cells_CD4_naive	0
TCGA-09-2054-01A	T_cells_CD4_memory_resting	0.096899945
TCGA-09-2054-01A	T_cells_CD4_memory_activated	0
TCGA-09-2054-01A	T_cells_follicular_helper	0.021144343
TCGA-09-2054-01A	T_cells_regulatory_(Tregs)	0.071888672
TCGA-09-2054-01A	T_cells_gamma_delta	0

TCGA-09-2054-01A	NK_cells_resting	0
TCGA-09-2054-01A	NK_cells_activated	0.027074528
TCGA-09-2054-01A	Monocytes	0.008401757
TCGA-09-2054-01A	Macrophages_M0	0.139509555
TCGA-09-2054-01A	Macrophages_M1	0.067019045
TCGA-09-2054-01A	Macrophages_M2	0.253490102
TCGA-09-2054-01A	Dendritic_cells_resting	0
TCGA-09-2054-01A	Dendritic_cells_activated	0
TCGA-09-2054-01A	Mast_cells_resting	0.00071894
TCGA-09-2054-01A	Mast_cells_activated	0
TCGA-09-2054-01A	Eosinophils	0
TCGA-09-2054-01A	Neutrophils	0.004448482
TCGA-10-0927-01A	B_cells_naive	0
TCGA-10-0927-01A	B_cells_memory	0
TCGA-10-0927-01A	Plasma_cells	0.169589466
TCGA-10-0927-01A	T_cells_CD8	0
TCGA-10-0927-01A	T_cells_CD4_naive	0
TCGA-10-0927-01A	T_cells_CD4_memory_resting	0.073745418
TCGA-10-0927-01A	T_cells_CD4_memory_activated	0
TCGA-10-0927-01A	T_cells_follicular_helper	0.027820419
TCGA-10-0927-01A	T_cells_regulatory_(Tregs)	0.001076964
TCGA-10-0927-01A	T_cells_gamma_delta	0.191535306
TCGA-10-0927-01A	NK_cells_resting	0
TCGA-10-0927-01A	NK_cells_activated	0.101711139
TCGA-10-0927-01A	Monocytes	0.028559317
TCGA-10-0927-01A	Macrophages_M0	0.206817348
TCGA-10-0927-01A	Macrophages_M1	0
TCGA-10-0927-01A	Macrophages_M2	0.020829027
TCGA-10-0927-01A	Dendritic_cells_resting	0
TCGA-10-0927-01A	Dendritic_cells_activated	0
TCGA-10-0927-01A	Mast_cells_resting	0.159484977
TCGA-10-0927-01A	Mast_cells_activated	0
TCGA-10-0927-01A	Eosinophils	0.018830619

TCGA-10-0927-01A	Neutrophils	0
TCGA-10-0928-01A	B_cells_naive	0.07234449
TCGA-10-0928-01A	B_cells_memory	0
TCGA-10-0928-01A	Plasma_cells	0
TCGA-10-0928-01A	T_cells_CD8	0
TCGA-10-0928-01A	T_cells_CD4_naive	0
TCGA-10-0928-01A	T_cells_CD4_memory_resting	0.352553071
TCGA-10-0928-01A	T_cells_CD4_memory_activated	0
TCGA-10-0928-01A	T_cells_follicular_helper	0
TCGA-10-0928-01A	T_cells_regulatory_(Tregs)	0.028998248
TCGA-10-0928-01A	T_cells_gamma_delta	0
TCGA-10-0928-01A	NK_cells_resting	0
TCGA-10-0928-01A	NK_cells_activated	0.063987766
TCGA-10-0928-01A	Monocytes	0.012389014
TCGA-10-0928-01A	Macrophages_M0	0.153961112
TCGA-10-0928-01A	Macrophages_M1	0
TCGA-10-0928-01A	Macrophages_M2	0.102927894
TCGA-10-0928-01A	Dendritic_cells_resting	0.074737907
TCGA-10-0928-01A	Dendritic_cells_activated	0.109279012
TCGA-10-0928-01A	Mast_cells_resting	0
TCGA-10-0928-01A	Mast_cells_activated	0.028821485
TCGA-10-0928-01A	Eosinophils	0
TCGA-10-0928-01A	Neutrophils	0
TCGA-10-0933-01A	B_cells_naive	0.100475939
TCGA-10-0933-01A	B_cells_memory	0
TCGA-10-0933-01A	Plasma_cells	0
TCGA-10-0933-01A	T_cells_CD8	0.132573163
TCGA-10-0933-01A	T_cells_CD4_naive	0
TCGA-10-0933-01A	T_cells_CD4_memory_resting	0.17690976
TCGA-10-0933-01A	T_cells_CD4_memory_activated	0
TCGA-10-0933-01A	T_cells_follicular_helper	0.013106218
TCGA-10-0933-01A	T_cells_regulatory_(Tregs)	0.057600537
TCGA-10-0933-01A	T_cells_gamma_delta	0

TCGA-10-0933-01A	NK_cells_resting	0
TCGA-10-0933-01A	NK_cells_activated	0.159386294
TCGA-10-0933-01A	Monocytes	0.088825439
TCGA-10-0933-01A	Macrophages_M0	0.050794023
TCGA-10-0933-01A	Macrophages_M1	0.036696815
TCGA-10-0933-01A	Macrophages_M2	0.087642079
TCGA-10-0933-01A	Dendritic_cells_resting	0
TCGA-10-0933-01A	Dendritic_cells_activated	0.039038724
TCGA-10-0933-01A	Mast_cells_resting	0.049942849
TCGA-10-0933-01A	Mast_cells_activated	0
TCGA-10-0933-01A	Eosinophils	0.007008161
TCGA-10-0933-01A	Neutrophils	0
TCGA-10-0936-01A	B_cells_naive	0.039926166
TCGA-10-0936-01A	B_cells_memory	0
TCGA-10-0936-01A	Plasma_cells	0.102715976
TCGA-10-0936-01A	T_cells_CD8	0.081714538
TCGA-10-0936-01A	T_cells_CD4_naive	0
TCGA-10-0936-01A	T_cells_CD4_memory_resting	0.216360456
TCGA-10-0936-01A	T_cells_CD4_memory_activated	0
TCGA-10-0936-01A	T_cells_follicular_helper	0.10910657
TCGA-10-0936-01A	T_cells_regulatory_(Tregs)	0.079395058
TCGA-10-0936-01A	T_cells_gamma_delta	0
TCGA-10-0936-01A	NK_cells_resting	0
TCGA-10-0936-01A	NK_cells_activated	0.059368129
TCGA-10-0936-01A	Monocytes	0.005063642
TCGA-10-0936-01A	Macrophages_M0	0.008125436
TCGA-10-0936-01A	Macrophages_M1	0.065756395
TCGA-10-0936-01A	Macrophages_M2	0.121736638
TCGA-10-0936-01A	Dendritic_cells_resting	0
TCGA-10-0936-01A	Dendritic_cells_activated	0.110730994
TCGA-10-0936-01A	Mast_cells_resting	0
TCGA-10-0936-01A	Mast_cells_activated	0
TCGA-10-0936-01A	Eosinophils	0

TCGA-10-0936-01A	Neutrophils	0
TCGA-10-0938-01A	B_cells_naive	0
TCGA-10-0938-01A	B_cells_memory	0.073718439
TCGA-10-0938-01A	Plasma_cells	0.041288783
TCGA-10-0938-01A	T_cells_CD8	0
TCGA-10-0938-01A	T_cells_CD4_naive	0
TCGA-10-0938-01A	T_cells_CD4_memory_resting	0.054503233
TCGA-10-0938-01A	T_cells_CD4_memory_activated	0
TCGA-10-0938-01A	T_cells_follicular_helper	0.118805906
TCGA-10-0938-01A	T_cells_regulatory_(Tregs)	0.019142364
TCGA-10-0938-01A	T_cells_gamma_delta	0.053241179
TCGA-10-0938-01A	NK_cells_resting	0
TCGA-10-0938-01A	NK_cells_activated	0.052164001
TCGA-10-0938-01A	Monocytes	0.040091787
TCGA-10-0938-01A	Macrophages_M0	0.30214049
TCGA-10-0938-01A	Macrophages_M1	0.054135788
TCGA-10-0938-01A	Macrophages_M2	0
TCGA-10-0938-01A	Dendritic_cells_resting	0
TCGA-10-0938-01A	Dendritic_cells_activated	0
TCGA-10-0938-01A	Mast_cells_resting	0.142010148
TCGA-10-0938-01A	Mast_cells_activated	0
TCGA-10-0938-01A	Eosinophils	0.048757881
TCGA-10-0938-01A	Neutrophils	0
TCGA-13-0720-01A	B_cells_naive	0.004441756
TCGA-13-0720-01A	B_cells_memory	0
TCGA-13-0720-01A	Plasma_cells	0.066065864
TCGA-13-0720-01A	T_cells_CD8	0.024243368
TCGA-13-0720-01A	T_cells_CD4_naive	0
TCGA-13-0720-01A	T_cells_CD4_memory_resting	0.273963102
TCGA-13-0720-01A	T_cells_CD4_memory_activated	0
TCGA-13-0720-01A	T_cells_follicular_helper	0.077456099
TCGA-13-0720-01A	T_cells_regulatory_(Tregs)	0
TCGA-13-0720-01A	T_cells_gamma_delta	0

TCGA-13-0720-01A	NK_cells_resting	0
TCGA-13-0720-01A	NK_cells_activated	0.066225727
TCGA-13-0720-01A	Monocytes	0.022113827
TCGA-13-0720-01A	Macrophages_M0	0.185751407
TCGA-13-0720-01A	Macrophages_M1	0.047023659
TCGA-13-0720-01A	Macrophages_M2	0.012955328
TCGA-13-0720-01A	Dendritic_cells_resting	0
TCGA-13-0720-01A	Dendritic_cells_activated	0.205781798
TCGA-13-0720-01A	Mast_cells_resting	0.013978066
TCGA-13-0720-01A	Mast_cells_activated	0
TCGA-13-0720-01A	Eosinophils	0
TCGA-13-0720-01A	Neutrophils	0
TCGA-13-0724-01A	B_cells_naive	0.177910304
TCGA-13-0724-01A	B_cells_memory	0
TCGA-13-0724-01A	Plasma_cells	0.003205773
TCGA-13-0724-01A	T_cells_CD8	0.146625719
TCGA-13-0724-01A	T_cells_CD4_naive	0
TCGA-13-0724-01A	T_cells_CD4_memory_resting	0.124732507
TCGA-13-0724-01A	T_cells_CD4_memory_activated	0
TCGA-13-0724-01A	T_cells_follicular_helper	0.024332237
TCGA-13-0724-01A	T_cells_regulatory_(Tregs)	0.075195571
TCGA-13-0724-01A	T_cells_gamma_delta	0
TCGA-13-0724-01A	NK_cells_resting	0
TCGA-13-0724-01A	NK_cells_activated	0.043792158
TCGA-13-0724-01A	Monocytes	0.019884572
TCGA-13-0724-01A	Macrophages_M0	0.163634726
TCGA-13-0724-01A	Macrophages_M1	0.040676568
TCGA-13-0724-01A	Macrophages_M2	0.098094497
TCGA-13-0724-01A	Dendritic_cells_resting	0
TCGA-13-0724-01A	Dendritic_cells_activated	0.064586123
TCGA-13-0724-01A	Mast_cells_resting	0.017329245
TCGA-13-0724-01A	Mast_cells_activated	0
TCGA-13-0724-01A	Eosinophils	0

TCGA-13-0724-01A	Neutrophils	0
TCGA-13-0727-01A	B_cells_naive	0
TCGA-13-0727-01A	B_cells_memory	0.037286847
TCGA-13-0727-01A	Plasma_cells	0
TCGA-13-0727-01A	T_cells_CD8	0.124444526
TCGA-13-0727-01A	T_cells_CD4_naive	0
TCGA-13-0727-01A	T_cells_CD4_memory_resting	0.130870276
TCGA-13-0727-01A	T_cells_CD4_memory_activated	0
TCGA-13-0727-01A	T_cells_follicular_helper	0.003504136
TCGA-13-0727-01A	T_cells_regulatory_(Tregs)	0.070955487
TCGA-13-0727-01A	T_cells_gamma_delta	0
TCGA-13-0727-01A	NK_cells_resting	0
TCGA-13-0727-01A	NK_cells_activated	0.012353031
TCGA-13-0727-01A	Monocytes	0.039273442
TCGA-13-0727-01A	Macrophages_M0	0
TCGA-13-0727-01A	Macrophages_M1	0.034116059
TCGA-13-0727-01A	Macrophages_M2	0.521012042
TCGA-13-0727-01A	Dendritic_cells_resting	0.000591279
TCGA-13-0727-01A	Dendritic_cells_activated	0
TCGA-13-0727-01A	Mast_cells_resting	0.024068784
TCGA-13-0727-01A	Mast_cells_activated	0
TCGA-13-0727-01A	Eosinophils	0
TCGA-13-0727-01A	Neutrophils	0.001524091
TCGA-13-0730-01A	B_cells_naive	0.022039676
TCGA-13-0730-01A	B_cells_memory	0
TCGA-13-0730-01A	Plasma_cells	0
TCGA-13-0730-01A	T_cells_CD8	0.190156162
TCGA-13-0730-01A	T_cells_CD4_naive	0
TCGA-13-0730-01A	T_cells_CD4_memory_resting	0.063413882
TCGA-13-0730-01A	T_cells_CD4_memory_activated	0
TCGA-13-0730-01A	T_cells_follicular_helper	0.020787102
TCGA-13-0730-01A	T_cells_regulatory_(Tregs)	0.068466065
TCGA-13-0730-01A	T_cells_gamma_delta	0

TCGA-13-0730-01A	NK_cells_resting	0
TCGA-13-0730-01A	NK_cells_activated	0.084308961
TCGA-13-0730-01A	Monocytes	0.009546754
TCGA-13-0730-01A	Macrophages_M0	0.172966348
TCGA-13-0730-01A	Macrophages_M1	0.018025828
TCGA-13-0730-01A	Macrophages_M2	0.203595676
TCGA-13-0730-01A	Dendritic_cells_resting	0.056268668
TCGA-13-0730-01A	Dendritic_cells_activated	0.090424878
TCGA-13-0730-01A	Mast_cells_resting	0
TCGA-13-0730-01A	Mast_cells_activated	0
TCGA-13-0730-01A	Eosinophils	0
TCGA-13-0730-01A	Neutrophils	0
TCGA-13-0765-01A	B_cells_naive	0.016936048
TCGA-13-0765-01A	B_cells_memory	0
TCGA-13-0765-01A	Plasma_cells	0.001811061
TCGA-13-0765-01A	T_cells_CD8	0.059410552
TCGA-13-0765-01A	T_cells_CD4_naive	0
TCGA-13-0765-01A	T_cells_CD4_memory_resting	0.165459462
TCGA-13-0765-01A	T_cells_CD4_memory_activated	0
TCGA-13-0765-01A	T_cells_follicular_helper	0
TCGA-13-0765-01A	T_cells_regulatory_(Tregs)	0.080662709
TCGA-13-0765-01A	T_cells_gamma_delta	0
TCGA-13-0765-01A	NK_cells_resting	0
TCGA-13-0765-01A	NK_cells_activated	0.034932586
TCGA-13-0765-01A	Monocytes	0.06772915
TCGA-13-0765-01A	Macrophages_M0	0.456721969
TCGA-13-0765-01A	Macrophages_M1	0.005829101
TCGA-13-0765-01A	Macrophages_M2	0.059510959
TCGA-13-0765-01A	Dendritic_cells_resting	0
TCGA-13-0765-01A	Dendritic_cells_activated	0.006591761
TCGA-13-0765-01A	Mast_cells_resting	0
TCGA-13-0765-01A	Mast_cells_activated	0.044404641
TCGA-13-0765-01A	Eosinophils	0

TCGA-13-0765-01A	Neutrophils	0
TCGA-13-0768-01A	B_cells_naive	0.028423602
TCGA-13-0768-01A	B_cells_memory	0
TCGA-13-0768-01A	Plasma_cells	0.04327207
TCGA-13-0768-01A	T_cells_CD8	0.286431071
TCGA-13-0768-01A	T_cells_CD4_naive	0
TCGA-13-0768-01A	T_cells_CD4_memory_resting	0.051784208
TCGA-13-0768-01A	T_cells_CD4_memory_activated	0.146608425
TCGA-13-0768-01A	T_cells_follicular_helper	0
TCGA-13-0768-01A	T_cells_regulatory_(Tregs)	0.076226255
TCGA-13-0768-01A	T_cells_gamma_delta	0
TCGA-13-0768-01A	NK_cells_resting	0.009824686
TCGA-13-0768-01A	NK_cells_activated	0.000235029
TCGA-13-0768-01A	Monocytes	0.05367805
TCGA-13-0768-01A	Macrophages_M0	0.045548309
TCGA-13-0768-01A	Macrophages_M1	0.114635057
TCGA-13-0768-01A	Macrophages_M2	0.120351411
TCGA-13-0768-01A	Dendritic_cells_resting	0.00517415
TCGA-13-0768-01A	Dendritic_cells_activated	0
TCGA-13-0768-01A	Mast_cells_resting	0.017807676
TCGA-13-0768-01A	Mast_cells_activated	0
TCGA-13-0768-01A	Eosinophils	0
TCGA-13-0768-01A	Neutrophils	0
TCGA-13-0795-01A	B_cells_naive	0.003856138
TCGA-13-0795-01A	B_cells_memory	0
TCGA-13-0795-01A	Plasma_cells	0.014681624
TCGA-13-0795-01A	T_cells_CD8	0.10441456
TCGA-13-0795-01A	T_cells_CD4_naive	0
TCGA-13-0795-01A	T_cells_CD4_memory_resting	0.21241436
TCGA-13-0795-01A	T_cells_CD4_memory_activated	0
TCGA-13-0795-01A	T_cells_follicular_helper	0.016463393
TCGA-13-0795-01A	T_cells_regulatory_(Tregs)	0.045499532
TCGA-13-0795-01A	T_cells_gamma_delta	0

TCGA-13-0795-01A	NK_cells_resting	0
TCGA-13-0795-01A	NK_cells_activated	0.053681272
TCGA-13-0795-01A	Monocytes	0.139819284
TCGA-13-0795-01A	Macrophages_M0	0
TCGA-13-0795-01A	Macrophages_M1	0.032853215
TCGA-13-0795-01A	Macrophages_M2	0.308581451
TCGA-13-0795-01A	Dendritic_cells_resting	0
TCGA-13-0795-01A	Dendritic_cells_activated	0.047602672
TCGA-13-0795-01A	Mast_cells_resting	0.019775528
TCGA-13-0795-01A	Mast_cells_activated	0
TCGA-13-0795-01A	Eosinophils	0.000356972
TCGA-13-0795-01A	Neutrophils	0
TCGA-13-0804-01A	B_cells_naive	0
TCGA-13-0804-01A	B_cells_memory	0.119300701
TCGA-13-0804-01A	Plasma_cells	0
TCGA-13-0804-01A	T_cells_CD8	0.121255696
TCGA-13-0804-01A	T_cells_CD4_naive	0
TCGA-13-0804-01A	T_cells_CD4_memory_resting	0.109054694
TCGA-13-0804-01A	T_cells_CD4_memory_activated	0
TCGA-13-0804-01A	T_cells_follicular_helper	0.012258364
TCGA-13-0804-01A	T_cells_regulatory_(Tregs)	0.061197365
TCGA-13-0804-01A	T_cells_gamma_delta	0
TCGA-13-0804-01A	NK_cells_resting	0
TCGA-13-0804-01A	NK_cells_activated	0.159316782
TCGA-13-0804-01A	Monocytes	0.019721803
TCGA-13-0804-01A	Macrophages_M0	0
TCGA-13-0804-01A	Macrophages_M1	0.043220008
TCGA-13-0804-01A	Macrophages_M2	0.310097308
TCGA-13-0804-01A	Dendritic_cells_resting	0.02090899
TCGA-13-0804-01A	Dendritic_cells_activated	0
TCGA-13-0804-01A	Mast_cells_resting	0
TCGA-13-0804-01A	Mast_cells_activated	0.017841105
TCGA-13-0804-01A	Eosinophils	0.002186807

TCGA-13-0804-01A	Neutrophils	0.003640375
TCGA-13-0883-01A	B_cells_naive	0.023471272
TCGA-13-0883-01A	B_cells_memory	0
TCGA-13-0883-01A	Plasma_cells	0
TCGA-13-0883-01A	T_cells_CD8	0.098763046
TCGA-13-0883-01A	T_cells_CD4_naive	0
TCGA-13-0883-01A	T_cells_CD4_memory_resting	0.111284861
TCGA-13-0883-01A	T_cells_CD4_memory_activated	0.007935388
TCGA-13-0883-01A	T_cells_follicular_helper	0.00622158
TCGA-13-0883-01A	T_cells_regulatory_(Tregs)	0.059288655
TCGA-13-0883-01A	T_cells_gamma_delta	0
TCGA-13-0883-01A	NK_cells_resting	0
TCGA-13-0883-01A	NK_cells_activated	0.015987842
TCGA-13-0883-01A	Monocytes	0
TCGA-13-0883-01A	Macrophages_M0	0.368876569
TCGA-13-0883-01A	Macrophages_M1	0.05527221
TCGA-13-0883-01A	Macrophages_M2	0.199292242
TCGA-13-0883-01A	Dendritic_cells_resting	0.022641196
TCGA-13-0883-01A	Dendritic_cells_activated	0
TCGA-13-0883-01A	Mast_cells_resting	0
TCGA-13-0883-01A	Mast_cells_activated	0.005382484
TCGA-13-0883-01A	Eosinophils	0
TCGA-13-0883-01A	Neutrophils	0.025582656
TCGA-13-0888-01A	B_cells_naive	0
TCGA-13-0888-01A	B_cells_memory	0
TCGA-13-0888-01A	Plasma_cells	0.031736957
TCGA-13-0888-01A	T_cells_CD8	0
TCGA-13-0888-01A	T_cells_CD4_naive	0
TCGA-13-0888-01A	T_cells_CD4_memory_resting	0.165497621
TCGA-13-0888-01A	T_cells_CD4_memory_activated	0
TCGA-13-0888-01A	T_cells_follicular_helper	0.022437634
TCGA-13-0888-01A	T_cells_regulatory_(Tregs)	0.070611558
TCGA-13-0888-01A	T_cells_gamma_delta	0

TCGA-13-0888-01A	NK_cells_resting	0
TCGA-13-0888-01A	NK_cells_activated	0.040683434
TCGA-13-0888-01A	Monocytes	0
TCGA-13-0888-01A	Macrophages_M0	0.395072125
TCGA-13-0888-01A	Macrophages_M1	0.001332779
TCGA-13-0888-01A	Macrophages_M2	0.029892779
TCGA-13-0888-01A	Dendritic_cells_resting	0
TCGA-13-0888-01A	Dendritic_cells_activated	0
TCGA-13-0888-01A	Mast_cells_resting	0.224679743
TCGA-13-0888-01A	Mast_cells_activated	0
TCGA-13-0888-01A	Eosinophils	0.01805537
TCGA-13-0888-01A	Neutrophils	0
TCGA-13-1403-01A	B_cells_naive	0
TCGA-13-1403-01A	B_cells_memory	0
TCGA-13-1403-01A	Plasma_cells	0.000152304
TCGA-13-1403-01A	T_cells_CD8	0.040965114
TCGA-13-1403-01A	T_cells_CD4_naive	0
TCGA-13-1403-01A	T_cells_CD4_memory_resting	0.283459401
TCGA-13-1403-01A	T_cells_CD4_memory_activated	0
TCGA-13-1403-01A	T_cells_follicular_helper	0.035151801
TCGA-13-1403-01A	T_cells_regulatory_(Tregs)	0.091252925
TCGA-13-1403-01A	T_cells_gamma_delta	0
TCGA-13-1403-01A	NK_cells_resting	0
TCGA-13-1403-01A	NK_cells_activated	0.161161142
TCGA-13-1403-01A	Monocytes	0.007555372
TCGA-13-1403-01A	Macrophages_M0	0.038147483
TCGA-13-1403-01A	Macrophages_M1	0.001687926
TCGA-13-1403-01A	Macrophages_M2	0.029836031
TCGA-13-1403-01A	Dendritic_cells_resting	0
TCGA-13-1403-01A	Dendritic_cells_activated	0.25292703
TCGA-13-1403-01A	Mast_cells_resting	0.056061948
TCGA-13-1403-01A	Mast_cells_activated	0
TCGA-13-1403-01A	Eosinophils	0.000589108

TCGA-13-1403-01A	Neutrophils	0.001052415
TCGA-13-1404-01A	B_cells_naive	0.039230132
TCGA-13-1404-01A	B_cells_memory	0
TCGA-13-1404-01A	Plasma_cells	0.113603178
TCGA-13-1404-01A	T_cells_CD8	0.051708025
TCGA-13-1404-01A	T_cells_CD4_naive	0
TCGA-13-1404-01A	T_cells_CD4_memory_resting	0.126481284
TCGA-13-1404-01A	T_cells_CD4_memory_activated	0
TCGA-13-1404-01A	T_cells_follicular_helper	0.03729412
TCGA-13-1404-01A	T_cells_regulatory_(Tregs)	0.084387424
TCGA-13-1404-01A	T_cells_gamma_delta	0
TCGA-13-1404-01A	NK_cells_resting	0
TCGA-13-1404-01A	NK_cells_activated	0.058714609
TCGA-13-1404-01A	Monocytes	0.040799806
TCGA-13-1404-01A	Macrophages_M0	0.115112657
TCGA-13-1404-01A	Macrophages_M1	0.12832397
TCGA-13-1404-01A	Macrophages_M2	0.188626877
TCGA-13-1404-01A	Dendritic_cells_resting	0.013957842
TCGA-13-1404-01A	Dendritic_cells_activated	0
TCGA-13-1404-01A	Mast_cells_resting	0
TCGA-13-1404-01A	Mast_cells_activated	0.001760076
TCGA-13-1404-01A	Eosinophils	0
TCGA-13-1404-01A	Neutrophils	0
TCGA-13-1405-01A	B_cells_naive	0.087537579
TCGA-13-1405-01A	B_cells_memory	0
TCGA-13-1405-01A	Plasma_cells	0.05477678
TCGA-13-1405-01A	T_cells_CD8	0.1743814
TCGA-13-1405-01A	T_cells_CD4_naive	0
TCGA-13-1405-01A	T_cells_CD4_memory_resting	0.257998751
TCGA-13-1405-01A	T_cells_CD4_memory_activated	0
TCGA-13-1405-01A	T_cells_follicular_helper	0.016925259
TCGA-13-1405-01A	T_cells_regulatory_(Tregs)	0.033660765
TCGA-13-1405-01A	T_cells_gamma_delta	0.006092184

TCGA-13-1405-01A	NK_cells_resting	0
TCGA-13-1405-01A	NK_cells_activated	0.029851037
TCGA-13-1405-01A	Monocytes	0.079042552
TCGA-13-1405-01A	Macrophages_M0	0.034776466
TCGA-13-1405-01A	Macrophages_M1	0.112389338
TCGA-13-1405-01A	Macrophages_M2	0.108653615
TCGA-13-1405-01A	Dendritic_cells_resting	0
TCGA-13-1405-01A	Dendritic_cells_activated	0
TCGA-13-1405-01A	Mast_cells_resting	0.003914273
TCGA-13-1405-01A	Mast_cells_activated	0
TCGA-13-1405-01A	Eosinophils	0
TCGA-13-1405-01A	Neutrophils	0
TCGA-13-1409-01A	B_cells_naive	0
TCGA-13-1409-01A	B_cells_memory	0.07524945
TCGA-13-1409-01A	Plasma_cells	0.089874504
TCGA-13-1409-01A	T_cells_CD8	0
TCGA-13-1409-01A	T_cells_CD4_naive	0
TCGA-13-1409-01A	T_cells_CD4_memory_resting	0.110292467
TCGA-13-1409-01A	T_cells_CD4_memory_activated	0
TCGA-13-1409-01A	T_cells_follicular_helper	0.023415731
TCGA-13-1409-01A	T_cells_regulatory_(Tregs)	0.084331397
TCGA-13-1409-01A	T_cells_gamma_delta	0.022496288
TCGA-13-1409-01A	NK_cells_resting	0.021851165
TCGA-13-1409-01A	NK_cells_activated	0.02164042
TCGA-13-1409-01A	Monocytes	0
TCGA-13-1409-01A	Macrophages_M0	0.428175605
TCGA-13-1409-01A	Macrophages_M1	0
TCGA-13-1409-01A	Macrophages_M2	0.017307364
TCGA-13-1409-01A	Dendritic_cells_resting	0
TCGA-13-1409-01A	Dendritic_cells_activated	0
TCGA-13-1409-01A	Mast_cells_resting	0.105365607
TCGA-13-1409-01A	Mast_cells_activated	0
TCGA-13-1409-01A	Eosinophils	0

TCGA-13-1409-01A	Neutrophils	0
TCGA-13-1477-01A	B_cells_naive	0.036433908
TCGA-13-1477-01A	B_cells_memory	0
TCGA-13-1477-01A	Plasma_cells	0.002593742
TCGA-13-1477-01A	T_cells_CD8	0.036411051
TCGA-13-1477-01A	T_cells_CD4_naive	0
TCGA-13-1477-01A	T_cells_CD4_memory_resting	0.221726247
TCGA-13-1477-01A	T_cells_CD4_memory_activated	0
TCGA-13-1477-01A	T_cells_follicular_helper	0
TCGA-13-1477-01A	T_cells_regulatory_(Tregs)	0.066825917
TCGA-13-1477-01A	T_cells_gamma_delta	0
TCGA-13-1477-01A	NK_cells_resting	0
TCGA-13-1477-01A	NK_cells_activated	0.082665769
TCGA-13-1477-01A	Monocytes	0.005178339
TCGA-13-1477-01A	Macrophages_M0	0.242133802
TCGA-13-1477-01A	Macrophages_M1	0.132627487
TCGA-13-1477-01A	Macrophages_M2	0.111742558
TCGA-13-1477-01A	Dendritic_cells_resting	0.029291349
TCGA-13-1477-01A	Dendritic_cells_activated	0
TCGA-13-1477-01A	Mast_cells_resting	0
TCGA-13-1477-01A	Mast_cells_activated	0.032369832
TCGA-13-1477-01A	Eosinophils	0
TCGA-13-1477-01A	Neutrophils	0
TCGA-13-1485-01A	B_cells_naive	0
TCGA-13-1485-01A	B_cells_memory	0
TCGA-13-1485-01A	Plasma_cells	0.018860476
TCGA-13-1485-01A	T_cells_CD8	0.019448495
TCGA-13-1485-01A	T_cells_CD4_naive	0
TCGA-13-1485-01A	T_cells_CD4_memory_resting	0.138461202
TCGA-13-1485-01A	T_cells_CD4_memory_activated	0
TCGA-13-1485-01A	T_cells_follicular_helper	0.021128221
TCGA-13-1485-01A	T_cells_regulatory_(Tregs)	0.112789544
TCGA-13-1485-01A	T_cells_gamma_delta	0

TCGA-13-1485-01A	NK_cells_resting	0
TCGA-13-1485-01A	NK_cells_activated	0.056599158
TCGA-13-1485-01A	Monocytes	0
TCGA-13-1485-01A	Macrophages_M0	0.358293119
TCGA-13-1485-01A	Macrophages_M1	0
TCGA-13-1485-01A	Macrophages_M2	0.23527505
TCGA-13-1485-01A	Dendritic_cells_resting	0.018442318
TCGA-13-1485-01A	Dendritic_cells_activated	0.005436292
TCGA-13-1485-01A	Mast_cells_resting	0
TCGA-13-1485-01A	Mast_cells_activated	0.01311671
TCGA-13-1485-01A	Eosinophils	0.002149415
TCGA-13-1485-01A	Neutrophils	0
TCGA-13-1487-01A	B_cells_naive	0.096918031
TCGA-13-1487-01A	B_cells_memory	0
TCGA-13-1487-01A	Plasma_cells	0.008922728
TCGA-13-1487-01A	T_cells_CD8	0.101211033
TCGA-13-1487-01A	T_cells_CD4_naive	0
TCGA-13-1487-01A	T_cells_CD4_memory_resting	0.162664669
TCGA-13-1487-01A	T_cells_CD4_memory_activated	0
TCGA-13-1487-01A	T_cells_follicular_helper	0.014302313
TCGA-13-1487-01A	T_cells_regulatory_(Tregs)	0.06650476
TCGA-13-1487-01A	T_cells_gamma_delta	0
TCGA-13-1487-01A	NK_cells_resting	0
TCGA-13-1487-01A	NK_cells_activated	0.083613199
TCGA-13-1487-01A	Monocytes	0
TCGA-13-1487-01A	Macrophages_M0	0.182814087
TCGA-13-1487-01A	Macrophages_M1	0.108842518
TCGA-13-1487-01A	Macrophages_M2	0.113133552
TCGA-13-1487-01A	Dendritic_cells_resting	0.034025739
TCGA-13-1487-01A	Dendritic_cells_activated	0.008844511
TCGA-13-1487-01A	Mast_cells_resting	0.018202859
TCGA-13-1487-01A	Mast_cells_activated	0
TCGA-13-1487-01A	Eosinophils	0

TCGA-13-1487-01A	Neutrophils	0
TCGA-13-1495-01A	B_cells_naive	0
TCGA-13-1495-01A	B_cells_memory	0.030411023
TCGA-13-1495-01A	Plasma_cells	0
TCGA-13-1495-01A	T_cells_CD8	0.057619451
TCGA-13-1495-01A	T_cells_CD4_naive	0
TCGA-13-1495-01A	T_cells_CD4_memory_resting	0.330610543
TCGA-13-1495-01A	T_cells_CD4_memory_activated	0
TCGA-13-1495-01A	T_cells_follicular_helper	0.047994657
TCGA-13-1495-01A	T_cells_regulatory_(Tregs)	0
TCGA-13-1495-01A	T_cells_gamma_delta	0
TCGA-13-1495-01A	NK_cells_resting	0
TCGA-13-1495-01A	NK_cells_activated	0.071919286
TCGA-13-1495-01A	Monocytes	0.036771066
TCGA-13-1495-01A	Macrophages_M0	0.060337892
TCGA-13-1495-01A	Macrophages_M1	0.232749126
TCGA-13-1495-01A	Macrophages_M2	0.07829145
TCGA-13-1495-01A	Dendritic_cells_resting	0
TCGA-13-1495-01A	Dendritic_cells_activated	0
TCGA-13-1495-01A	Mast_cells_resting	0.053295505
TCGA-13-1495-01A	Mast_cells_activated	0
TCGA-13-1495-01A	Eosinophils	0
TCGA-13-1495-01A	Neutrophils	0
TCGA-13-1497-01A	B_cells_naive	0.021082347
TCGA-13-1497-01A	B_cells_memory	0
TCGA-13-1497-01A	Plasma_cells	0.182314789
TCGA-13-1497-01A	T_cells_CD8	0.023811805
TCGA-13-1497-01A	T_cells_CD4_naive	0
TCGA-13-1497-01A	T_cells_CD4_memory_resting	0.226571805
TCGA-13-1497-01A	T_cells_CD4_memory_activated	0
TCGA-13-1497-01A	T_cells_follicular_helper	0.036331143
TCGA-13-1497-01A	T_cells_regulatory_(Tregs)	0.017087931
TCGA-13-1497-01A	T_cells_gamma_delta	0

TCGA-13-1497-01A	NK_cells_resting	0.02746368
TCGA-13-1497-01A	NK_cells_activated	0
TCGA-13-1497-01A	Monocytes	0.005258154
TCGA-13-1497-01A	Macrophages_M0	0.216389094
TCGA-13-1497-01A	Macrophages_M1	0.140520656
TCGA-13-1497-01A	Macrophages_M2	0.091646581
TCGA-13-1497-01A	Dendritic_cells_resting	0
TCGA-13-1497-01A	Dendritic_cells_activated	0
TCGA-13-1497-01A	Mast_cells_resting	0
TCGA-13-1497-01A	Mast_cells_activated	0.011522016
TCGA-13-1497-01A	Eosinophils	0
TCGA-13-1497-01A	Neutrophils	0
TCGA-13-1511-01A	B_cells_naive	0.02718248
TCGA-13-1511-01A	B_cells_memory	0
TCGA-13-1511-01A	Plasma_cells	0.020854278
TCGA-13-1511-01A	T_cells_CD8	0.034944854
TCGA-13-1511-01A	T_cells_CD4_naive	0
TCGA-13-1511-01A	T_cells_CD4_memory_resting	0.166533044
TCGA-13-1511-01A	T_cells_CD4_memory_activated	0
TCGA-13-1511-01A	T_cells_follicular_helper	0
TCGA-13-1511-01A	T_cells_regulatory_(Tregs)	0.067921845
TCGA-13-1511-01A	T_cells_gamma_delta	0
TCGA-13-1511-01A	NK_cells_resting	0
TCGA-13-1511-01A	NK_cells_activated	0.077172285
TCGA-13-1511-01A	Monocytes	0.009658413
TCGA-13-1511-01A	Macrophages_M0	0.140404668
TCGA-13-1511-01A	Macrophages_M1	0.124688123
TCGA-13-1511-01A	Macrophages_M2	0.194432673
TCGA-13-1511-01A	Dendritic_cells_resting	0
TCGA-13-1511-01A	Dendritic_cells_activated	0.128353171
TCGA-13-1511-01A	Mast_cells_resting	0.007854166
TCGA-13-1511-01A	Mast_cells_activated	0
TCGA-13-1511-01A	Eosinophils	0

TCGA-13-1511-01A	Neutrophils	0
TCGA-20-0987-01A	B_cells_naive	0
TCGA-20-0987-01A	B_cells_memory	0
TCGA-20-0987-01A	Plasma_cells	0.052774037
TCGA-20-0987-01A	T_cells_CD8	0.051420463
TCGA-20-0987-01A	T_cells_CD4_naive	0
TCGA-20-0987-01A	T_cells_CD4_memory_resting	0.142597802
TCGA-20-0987-01A	T_cells_CD4_memory_activated	0.005358089
TCGA-20-0987-01A	T_cells_follicular_helper	0.010750039
TCGA-20-0987-01A	T_cells_regulatory_(Tregs)	0.035111607
TCGA-20-0987-01A	T_cells_gamma_delta	0
TCGA-20-0987-01A	NK_cells_resting	0.022656933
TCGA-20-0987-01A	NK_cells_activated	0.029238593
TCGA-20-0987-01A	Monocytes	0.051859001
TCGA-20-0987-01A	Macrophages_M0	0.317122591
TCGA-20-0987-01A	Macrophages_M1	0.089939027
TCGA-20-0987-01A	Macrophages_M2	0.162267876
TCGA-20-0987-01A	Dendritic_cells_resting	0
TCGA-20-0987-01A	Dendritic_cells_activated	0
TCGA-20-0987-01A	Mast_cells_resting	0.028903942
TCGA-20-0987-01A	Mast_cells_activated	0
TCGA-20-0987-01A	Eosinophils	0
TCGA-20-0987-01A	Neutrophils	0
TCGA-20-1683-01A	B_cells_naive	0.057108747
TCGA-20-1683-01A	B_cells_memory	0
TCGA-20-1683-01A	Plasma_cells	0.459274536
TCGA-20-1683-01A	T_cells_CD8	0.075892167
TCGA-20-1683-01A	T_cells_CD4_naive	0
TCGA-20-1683-01A	T_cells_CD4_memory_resting	0.067034907
TCGA-20-1683-01A	T_cells_CD4_memory_activated	0.003625009
TCGA-20-1683-01A	T_cells_follicular_helper	0.007408961
TCGA-20-1683-01A	T_cells_regulatory_(Tregs)	0.02262016
TCGA-20-1683-01A	T_cells_gamma_delta	0

TCGA-20-1683-01A	NK_cells_resting	0
TCGA-20-1683-01A	NK_cells_activated	0.021572129
TCGA-20-1683-01A	Monocytes	0
TCGA-20-1683-01A	Macrophages_M0	0.135319842
TCGA-20-1683-01A	Macrophages_M1	0.025194564
TCGA-20-1683-01A	Macrophages_M2	0.100487971
TCGA-20-1683-01A	Dendritic_cells_resting	0
TCGA-20-1683-01A	Dendritic_cells_activated	0.002294326
TCGA-20-1683-01A	Mast_cells_resting	0.022166681
TCGA-20-1683-01A	Mast_cells_activated	0
TCGA-20-1683-01A	Eosinophils	0
TCGA-20-1683-01A	Neutrophils	0
TCGA-20-1687-01A	B_cells_naive	0.088198272
TCGA-20-1687-01A	B_cells_memory	0
TCGA-20-1687-01A	Plasma_cells	0.034163779
TCGA-20-1687-01A	T_cells_CD8	0.062417281
TCGA-20-1687-01A	T_cells_CD4_naive	0
TCGA-20-1687-01A	T_cells_CD4_memory_resting	0.246664298
TCGA-20-1687-01A	T_cells_CD4_memory_activated	0
TCGA-20-1687-01A	T_cells_follicular_helper	0.048481
TCGA-20-1687-01A	T_cells_regulatory_(Tregs)	0.054610194
TCGA-20-1687-01A	T_cells_gamma_delta	0
TCGA-20-1687-01A	NK_cells_resting	0
TCGA-20-1687-01A	NK_cells_activated	0.033064299
TCGA-20-1687-01A	Monocytes	0.148023889
TCGA-20-1687-01A	Macrophages_M0	0.058847171
TCGA-20-1687-01A	Macrophages_M1	0.107673556
TCGA-20-1687-01A	Macrophages_M2	0.095624213
TCGA-20-1687-01A	Dendritic_cells_resting	0.012374161
TCGA-20-1687-01A	Dendritic_cells_activated	0.002120324
TCGA-20-1687-01A	Mast_cells_resting	0.007737563
TCGA-20-1687-01A	Mast_cells_activated	0
TCGA-20-1687-01A	Eosinophils	0

TCGA-20-1687-01A	Neutrophils	0
TCGA-23-1024-01A	B_cells_naive	0.067267052
TCGA-23-1024-01A	B_cells_memory	0
TCGA-23-1024-01A	Plasma_cells	0
TCGA-23-1024-01A	T_cells_CD8	0.136971244
TCGA-23-1024-01A	T_cells_CD4_naive	0
TCGA-23-1024-01A	T_cells_CD4_memory_resting	0.141438947
TCGA-23-1024-01A	T_cells_CD4_memory_activated	0
TCGA-23-1024-01A	T_cells_follicular_helper	0.111561514
TCGA-23-1024-01A	T_cells_regulatory_(Tregs)	0.048832584
TCGA-23-1024-01A	T_cells_gamma_delta	0
TCGA-23-1024-01A	NK_cells_resting	0
TCGA-23-1024-01A	NK_cells_activated	0.038725215
TCGA-23-1024-01A	Monocytes	0.0388386
TCGA-23-1024-01A	Macrophages_M0	0.050382744
TCGA-23-1024-01A	Macrophages_M1	0.0377025
TCGA-23-1024-01A	Macrophages_M2	0.141291891
TCGA-23-1024-01A	Dendritic_cells_resting	0
TCGA-23-1024-01A	Dendritic_cells_activated	0.172634
TCGA-23-1024-01A	Mast_cells_resting	0.01435371
TCGA-23-1024-01A	Mast_cells_activated	0
TCGA-23-1024-01A	Eosinophils	0
TCGA-23-1024-01A	Neutrophils	0
TCGA-23-1107-01A	B_cells_naive	0.031599815
TCGA-23-1107-01A	B_cells_memory	0
TCGA-23-1107-01A	Plasma_cells	0.209128157
TCGA-23-1107-01A	T_cells_CD8	0.054884878
TCGA-23-1107-01A	T_cells_CD4_naive	0
TCGA-23-1107-01A	T_cells_CD4_memory_resting	0.060094399
TCGA-23-1107-01A	T_cells_CD4_memory_activated	0
TCGA-23-1107-01A	T_cells_follicular_helper	0.008042761
TCGA-23-1107-01A	T_cells_regulatory_(Tregs)	0.06850308
TCGA-23-1107-01A	T_cells_gamma_delta	0

TCGA-23-1107-01A	NK_cells_resting	0
TCGA-23-1107-01A	NK_cells_activated	0.046765977
TCGA-23-1107-01A	Monocytes	0.012663814
TCGA-23-1107-01A	Macrophages_M0	0.232057333
TCGA-23-1107-01A	Macrophages_M1	0.048283609
TCGA-23-1107-01A	Macrophages_M2	0.184680078
TCGA-23-1107-01A	Dendritic_cells_resting	0
TCGA-23-1107-01A	Dendritic_cells_activated	0
TCGA-23-1107-01A	Mast_cells_resting	0
TCGA-23-1107-01A	Mast_cells_activated	0.043296101
TCGA-23-1107-01A	Eosinophils	0
TCGA-23-1107-01A	Neutrophils	0
TCGA-23-1110-01A	B_cells_naive	0
TCGA-23-1110-01A	B_cells_memory	0.019785779
TCGA-23-1110-01A	Plasma_cells	0.068034684
TCGA-23-1110-01A	T_cells_CD8	0.077875977
TCGA-23-1110-01A	T_cells_CD4_naive	0
TCGA-23-1110-01A	T_cells_CD4_memory_resting	0.25099492
TCGA-23-1110-01A	T_cells_CD4_memory_activated	0
TCGA-23-1110-01A	T_cells_follicular_helper	0.01231638
TCGA-23-1110-01A	T_cells_regulatory_(Tregs)	0.04375932
TCGA-23-1110-01A	T_cells_gamma_delta	0
TCGA-23-1110-01A	NK_cells_resting	0
TCGA-23-1110-01A	NK_cells_activated	0
TCGA-23-1110-01A	Monocytes	0.114284474
TCGA-23-1110-01A	Macrophages_M0	0.296512286
TCGA-23-1110-01A	Macrophages_M1	0
TCGA-23-1110-01A	Macrophages_M2	0.116436179
TCGA-23-1110-01A	Dendritic_cells_resting	0
TCGA-23-1110-01A	Dendritic_cells_activated	0
TCGA-23-1110-01A	Mast_cells_resting	0
TCGA-23-1110-01A	Mast_cells_activated	0
TCGA-23-1110-01A	Eosinophils	0

TCGA-23-1110-01A	Neutrophils	0
TCGA-23-1111-01A	B_cells_naive	0.124912314
TCGA-23-1111-01A	B_cells_memory	0
TCGA-23-1111-01A	Plasma_cells	0.01476599
TCGA-23-1111-01A	T_cells_CD8	0.025215095
TCGA-23-1111-01A	T_cells_CD4_naive	0
TCGA-23-1111-01A	T_cells_CD4_memory_resting	0.301336829
TCGA-23-1111-01A	T_cells_CD4_memory_activated	0
TCGA-23-1111-01A	T_cells_follicular_helper	0.033308087
TCGA-23-1111-01A	T_cells_regulatory_(Tregs)	0.033188307
TCGA-23-1111-01A	T_cells_gamma_delta	0
TCGA-23-1111-01A	NK_cells_resting	0
TCGA-23-1111-01A	NK_cells_activated	0.045516622
TCGA-23-1111-01A	Monocytes	0.066093248
TCGA-23-1111-01A	Macrophages_M0	0.094751731
TCGA-23-1111-01A	Macrophages_M1	0.003803764
TCGA-23-1111-01A	Macrophages_M2	0.084732854
TCGA-23-1111-01A	Dendritic_cells_resting	0
TCGA-23-1111-01A	Dendritic_cells_activated	0.172375157
TCGA-23-1111-01A	Mast_cells_resting	0
TCGA-23-1111-01A	Mast_cells_activated	0
TCGA-23-1111-01A	Eosinophils	0
TCGA-23-1111-01A	Neutrophils	0
TCGA-23-1113-01A	B_cells_naive	0
TCGA-23-1113-01A	B_cells_memory	0.056372369
TCGA-23-1113-01A	Plasma_cells	0.007874741
TCGA-23-1113-01A	T_cells_CD8	0
TCGA-23-1113-01A	T_cells_CD4_naive	0
TCGA-23-1113-01A	T_cells_CD4_memory_resting	0.127100328
TCGA-23-1113-01A	T_cells_CD4_memory_activated	0
TCGA-23-1113-01A	T_cells_follicular_helper	0.007731418
TCGA-23-1113-01A	T_cells_regulatory_(Tregs)	0.021686147
TCGA-23-1113-01A	T_cells_gamma_delta	0

TCGA-23-1113-01A	NK_cells_resting	0.02464459
TCGA-23-1113-01A	NK_cells_activated	0.01339115
TCGA-23-1113-01A	Monocytes	0.010931771
TCGA-23-1113-01A	Macrophages_M0	0.196335696
TCGA-23-1113-01A	Macrophages_M1	0
TCGA-23-1113-01A	Macrophages_M2	0.303149358
TCGA-23-1113-01A	Dendritic_cells_resting	0.023750902
TCGA-23-1113-01A	Dendritic_cells_activated	0
TCGA-23-1113-01A	Mast_cells_resting	0.207031533
TCGA-23-1113-01A	Mast_cells_activated	0
TCGA-23-1113-01A	Eosinophils	0
TCGA-23-1113-01A	Neutrophils	0
TCGA-23-1116-01A	B_cells_naive	0
TCGA-23-1116-01A	B_cells_memory	0.035337554
TCGA-23-1116-01A	Plasma_cells	0
TCGA-23-1116-01A	T_cells_CD8	0.010233838
TCGA-23-1116-01A	T_cells_CD4_naive	0
TCGA-23-1116-01A	T_cells_CD4_memory_resting	0.069482022
TCGA-23-1116-01A	T_cells_CD4_memory_activated	0
TCGA-23-1116-01A	T_cells_follicular_helper	0.022300991
TCGA-23-1116-01A	T_cells_regulatory_(Tregs)	0.036237148
TCGA-23-1116-01A	T_cells_gamma_delta	0.006329823
TCGA-23-1116-01A	NK_cells_resting	0
TCGA-23-1116-01A	NK_cells_activated	0.013745212
TCGA-23-1116-01A	Monocytes	0
TCGA-23-1116-01A	Macrophages_M0	0.297650916
TCGA-23-1116-01A	Macrophages_M1	0.060213388
TCGA-23-1116-01A	Macrophages_M2	0.235154715
TCGA-23-1116-01A	Dendritic_cells_resting	0.005973794
TCGA-23-1116-01A	Dendritic_cells_activated	0
TCGA-23-1116-01A	Mast_cells_resting	0
TCGA-23-1116-01A	Mast_cells_activated	0.120235181
TCGA-23-1116-01A	Eosinophils	0

TCGA-23-1116-01A	Neutrophils	0.087105418
TCGA-23-1809-01A	B_cells_naive	0.080897616
TCGA-23-1809-01A	B_cells_memory	0
TCGA-23-1809-01A	Plasma_cells	0.025313459
TCGA-23-1809-01A	T_cells_CD8	0.001591732
TCGA-23-1809-01A	T_cells_CD4_naive	0
TCGA-23-1809-01A	T_cells_CD4_memory_resting	0.173324523
TCGA-23-1809-01A	T_cells_CD4_memory_activated	0
TCGA-23-1809-01A	T_cells_follicular_helper	0.030453883
TCGA-23-1809-01A	T_cells_regulatory_(Tregs)	0.01935645
TCGA-23-1809-01A	T_cells_gamma_delta	0
TCGA-23-1809-01A	NK_cells_resting	0
TCGA-23-1809-01A	NK_cells_activated	0.014434396
TCGA-23-1809-01A	Monocytes	0
TCGA-23-1809-01A	Macrophages_M0	0.510078162
TCGA-23-1809-01A	Macrophages_M1	0.057343577
TCGA-23-1809-01A	Macrophages_M2	0.081808229
TCGA-23-1809-01A	Dendritic_cells_resting	0
TCGA-23-1809-01A	Dendritic_cells_activated	0
TCGA-23-1809-01A	Mast_cells_resting	0
TCGA-23-1809-01A	Mast_cells_activated	0.005397972
TCGA-23-1809-01A	Eosinophils	0
TCGA-23-1809-01A	Neutrophils	0
TCGA-23-2084-01A	B_cells_naive	0
TCGA-23-2084-01A	B_cells_memory	0
TCGA-23-2084-01A	Plasma_cells	0
TCGA-23-2084-01A	T_cells_CD8	0.012849168
TCGA-23-2084-01A	T_cells_CD4_naive	0
TCGA-23-2084-01A	T_cells_CD4_memory_resting	0.345400394
TCGA-23-2084-01A	T_cells_CD4_memory_activated	0
TCGA-23-2084-01A	T_cells_follicular_helper	0.04396912
TCGA-23-2084-01A	T_cells_regulatory_(Tregs)	0.019996014
TCGA-23-2084-01A	T_cells_gamma_delta	0

TCGA-23-2084-01A	NK_cells_resting	0
TCGA-23-2084-01A	NK_cells_activated	0.060067133
TCGA-23-2084-01A	Monocytes	0.159599594
TCGA-23-2084-01A	Macrophages_M0	0
TCGA-23-2084-01A	Macrophages_M1	0.077131716
TCGA-23-2084-01A	Macrophages_M2	0.162237433
TCGA-23-2084-01A	Dendritic_cells_resting	0
TCGA-23-2084-01A	Dendritic_cells_activated	0.104386643
TCGA-23-2084-01A	Mast_cells_resting	0.008729945
TCGA-23-2084-01A	Mast_cells_activated	0
TCGA-23-2084-01A	Eosinophils	0
TCGA-23-2084-01A	Neutrophils	0.00563284
TCGA-24-0979-01A	B_cells_naive	0
TCGA-24-0979-01A	B_cells_memory	0
TCGA-24-0979-01A	Plasma_cells	0.022034379
TCGA-24-0979-01A	T_cells_CD8	0.107308517
TCGA-24-0979-01A	T_cells_CD4_naive	0
TCGA-24-0979-01A	T_cells_CD4_memory_resting	0.159467671
TCGA-24-0979-01A	T_cells_CD4_memory_activated	0
TCGA-24-0979-01A	T_cells_follicular_helper	0.082910027
TCGA-24-0979-01A	T_cells_regulatory_(Tregs)	0
TCGA-24-0979-01A	T_cells_gamma_delta	0
TCGA-24-0979-01A	NK_cells_resting	0
TCGA-24-0979-01A	NK_cells_activated	0.066001425
TCGA-24-0979-01A	Monocytes	0.046457684
TCGA-24-0979-01A	Macrophages_M0	0.207224067
TCGA-24-0979-01A	Macrophages_M1	0.121666578
TCGA-24-0979-01A	Macrophages_M2	0.012443371
TCGA-24-0979-01A	Dendritic_cells_resting	0.002743486
TCGA-24-0979-01A	Dendritic_cells_activated	0.010553997
TCGA-24-0979-01A	Mast_cells_resting	0.146280492
TCGA-24-0979-01A	Mast_cells_activated	0
TCGA-24-0979-01A	Eosinophils	0.014908307

TCGA-24-0979-01A	Neutrophils	0
TCGA-24-1104-01A	B_cells_naive	0.029494779
TCGA-24-1104-01A	B_cells_memory	0
TCGA-24-1104-01A	Plasma_cells	0.066384024
TCGA-24-1104-01A	T_cells_CD8	0.12879347
TCGA-24-1104-01A	T_cells_CD4_naive	0
TCGA-24-1104-01A	T_cells_CD4_memory_resting	0.236565622
TCGA-24-1104-01A	T_cells_CD4_memory_activated	0
TCGA-24-1104-01A	T_cells_follicular_helper	0.050379167
TCGA-24-1104-01A	T_cells_regulatory_(Tregs)	0.081635752
TCGA-24-1104-01A	T_cells_gamma_delta	0
TCGA-24-1104-01A	NK_cells_resting	0
TCGA-24-1104-01A	NK_cells_activated	0.086911818
TCGA-24-1104-01A	Monocytes	0.068989
TCGA-24-1104-01A	Macrophages_M0	0
TCGA-24-1104-01A	Macrophages_M1	0.058645166
TCGA-24-1104-01A	Macrophages_M2	0.150254154
TCGA-24-1104-01A	Dendritic_cells_resting	0
TCGA-24-1104-01A	Dendritic_cells_activated	0
TCGA-24-1104-01A	Mast_cells_resting	0
TCGA-24-1104-01A	Mast_cells_activated	0.041947048
TCGA-24-1104-01A	Eosinophils	0
TCGA-24-1104-01A	Neutrophils	0
TCGA-24-1105-01A	B_cells_naive	0.065277523
TCGA-24-1105-01A	B_cells_memory	0
TCGA-24-1105-01A	Plasma_cells	0.099943304
TCGA-24-1105-01A	T_cells_CD8	0.079935397
TCGA-24-1105-01A	T_cells_CD4_naive	0
TCGA-24-1105-01A	T_cells_CD4_memory_resting	0.192778612
TCGA-24-1105-01A	T_cells_CD4_memory_activated	0.017144526
TCGA-24-1105-01A	T_cells_follicular_helper	0.037268246
TCGA-24-1105-01A	T_cells_regulatory_(Tregs)	0.053437573
TCGA-24-1105-01A	T_cells_gamma_delta	0

TCGA-24-1105-01A	NK_cells_resting	0
TCGA-24-1105-01A	NK_cells_activated	0.031337312
TCGA-24-1105-01A	Monocytes	0.009121169
TCGA-24-1105-01A	Macrophages_M0	0.151257222
TCGA-24-1105-01A	Macrophages_M1	0.056438196
TCGA-24-1105-01A	Macrophages_M2	0.20606092
TCGA-24-1105-01A	Dendritic_cells_resting	0
TCGA-24-1105-01A	Dendritic_cells_activated	0
TCGA-24-1105-01A	Mast_cells_resting	0
TCGA-24-1105-01A	Mast_cells_activated	0
TCGA-24-1105-01A	Eosinophils	0
TCGA-24-1105-01A	Neutrophils	0
TCGA-24-1422-01A	B_cells_naive	0.001002368
TCGA-24-1422-01A	B_cells_memory	0
TCGA-24-1422-01A	Plasma_cells	0.00576105
TCGA-24-1422-01A	T_cells_CD8	0.130499635
TCGA-24-1422-01A	T_cells_CD4_naive	0
TCGA-24-1422-01A	T_cells_CD4_memory_resting	0.125620372
TCGA-24-1422-01A	T_cells_CD4_memory_activated	0.008907677
TCGA-24-1422-01A	T_cells_follicular_helper	0.041241412
TCGA-24-1422-01A	T_cells_regulatory_(Tregs)	0.008161639
TCGA-24-1422-01A	T_cells_gamma_delta	0
TCGA-24-1422-01A	NK_cells_resting	0
TCGA-24-1422-01A	NK_cells_activated	0.12022785
TCGA-24-1422-01A	Monocytes	0.013425617
TCGA-24-1422-01A	Macrophages_M0	0.149122035
TCGA-24-1422-01A	Macrophages_M1	0.015168923
TCGA-24-1422-01A	Macrophages_M2	0.304557863
TCGA-24-1422-01A	Dendritic_cells_resting	0.005487153
TCGA-24-1422-01A	Dendritic_cells_activated	0.004256261
TCGA-24-1422-01A	Mast_cells_resting	0.066560147
TCGA-24-1422-01A	Mast_cells_activated	0
TCGA-24-1422-01A	Eosinophils	0

TCGA-24-1422-01A	Neutrophils	0
TCGA-24-1424-01A	B_cells_naive	0.050148948
TCGA-24-1424-01A	B_cells_memory	0
TCGA-24-1424-01A	Plasma_cells	0.050480911
TCGA-24-1424-01A	T_cells_CD8	0.044805859
TCGA-24-1424-01A	T_cells_CD4_naive	0
TCGA-24-1424-01A	T_cells_CD4_memory_resting	0.143642153
TCGA-24-1424-01A	T_cells_CD4_memory_activated	0
TCGA-24-1424-01A	T_cells_follicular_helper	0.055532198
TCGA-24-1424-01A	T_cells_regulatory_(Tregs)	0.029449229
TCGA-24-1424-01A	T_cells_gamma_delta	0
TCGA-24-1424-01A	NK_cells_resting	0
TCGA-24-1424-01A	NK_cells_activated	0.041614155
TCGA-24-1424-01A	Monocytes	0.010307034
TCGA-24-1424-01A	Macrophages_M0	0.154753678
TCGA-24-1424-01A	Macrophages_M1	0.135752181
TCGA-24-1424-01A	Macrophages_M2	0.240443331
TCGA-24-1424-01A	Dendritic_cells_resting	0
TCGA-24-1424-01A	Dendritic_cells_activated	0
TCGA-24-1424-01A	Mast_cells_resting	0.014685945
TCGA-24-1424-01A	Mast_cells_activated	0
TCGA-24-1424-01A	Eosinophils	0
TCGA-24-1424-01A	Neutrophils	0.028384377
TCGA-24-1425-01A	B_cells_naive	0.099200899
TCGA-24-1425-01A	B_cells_memory	0
TCGA-24-1425-01A	Plasma_cells	0.025519162
TCGA-24-1425-01A	T_cells_CD8	0.011996148
TCGA-24-1425-01A	T_cells_CD4_naive	0
TCGA-24-1425-01A	T_cells_CD4_memory_resting	0.172456096
TCGA-24-1425-01A	T_cells_CD4_memory_activated	0.002101771
TCGA-24-1425-01A	T_cells_follicular_helper	0.021700585
TCGA-24-1425-01A	T_cells_regulatory_(Tregs)	0.07314439
TCGA-24-1425-01A	T_cells_gamma_delta	0

TCGA-24-1425-01A	NK_cells_resting	0.023228216
TCGA-24-1425-01A	NK_cells_activated	0.011548447
TCGA-24-1425-01A	Monocytes	0
TCGA-24-1425-01A	Macrophages_M0	0.279114245
TCGA-24-1425-01A	Macrophages_M1	0.070346483
TCGA-24-1425-01A	Macrophages_M2	0.181339092
TCGA-24-1425-01A	Dendritic_cells_resting	0
TCGA-24-1425-01A	Dendritic_cells_activated	0
TCGA-24-1425-01A	Mast_cells_resting	0.015806111
TCGA-24-1425-01A	Mast_cells_activated	0
TCGA-24-1425-01A	Eosinophils	0
TCGA-24-1425-01A	Neutrophils	0.012498355
TCGA-24-1426-01A	B_cells_naive	0
TCGA-24-1426-01A	B_cells_memory	0.008601053
TCGA-24-1426-01A	Plasma_cells	0
TCGA-24-1426-01A	T_cells_CD8	0
TCGA-24-1426-01A	T_cells_CD4_naive	0
TCGA-24-1426-01A	T_cells_CD4_memory_resting	0.291638012
TCGA-24-1426-01A	T_cells_CD4_memory_activated	0
TCGA-24-1426-01A	T_cells_follicular_helper	0.005688187
TCGA-24-1426-01A	T_cells_regulatory_(Tregs)	0.129084431
TCGA-24-1426-01A	T_cells_gamma_delta	0
TCGA-24-1426-01A	NK_cells_resting	0.007487286
TCGA-24-1426-01A	NK_cells_activated	0.023310622
TCGA-24-1426-01A	Monocytes	0.051331715
TCGA-24-1426-01A	Macrophages_M0	0.046562866
TCGA-24-1426-01A	Macrophages_M1	0.003945902
TCGA-24-1426-01A	Macrophages_M2	0.237666107
TCGA-24-1426-01A	Dendritic_cells_resting	0
TCGA-24-1426-01A	Dendritic_cells_activated	0.057131273
TCGA-24-1426-01A	Mast_cells_resting	0.134010742
TCGA-24-1426-01A	Mast_cells_activated	0
TCGA-24-1426-01A	Eosinophils	0

TCGA-24-1426-01A	Neutrophils	0.003541804
TCGA-24-1427-01A	B_cells_naive	0.011102688
TCGA-24-1427-01A	B_cells_memory	0
TCGA-24-1427-01A	Plasma_cells	0.125545648
TCGA-24-1427-01A	T_cells_CD8	0.140456858
TCGA-24-1427-01A	T_cells_CD4_naive	0
TCGA-24-1427-01A	T_cells_CD4_memory_resting	0.235587092
TCGA-24-1427-01A	T_cells_CD4_memory_activated	0
TCGA-24-1427-01A	T_cells_follicular_helper	0.004099262
TCGA-24-1427-01A	T_cells_regulatory_(Tregs)	0.068017686
TCGA-24-1427-01A	T_cells_gamma_delta	0.002049234
TCGA-24-1427-01A	NK_cells_resting	0
TCGA-24-1427-01A	NK_cells_activated	0.007596944
TCGA-24-1427-01A	Monocytes	0.131368549
TCGA-24-1427-01A	Macrophages_M0	0.015242921
TCGA-24-1427-01A	Macrophages_M1	0.070216367
TCGA-24-1427-01A	Macrophages_M2	0.158002688
TCGA-24-1427-01A	Dendritic_cells_resting	0.006545676
TCGA-24-1427-01A	Dendritic_cells_activated	0.00414361
TCGA-24-1427-01A	Mast_cells_resting	0.016114387
TCGA-24-1427-01A	Mast_cells_activated	0
TCGA-24-1427-01A	Eosinophils	0
TCGA-24-1427-01A	Neutrophils	0.003910391
TCGA-24-1430-01A	B_cells_naive	0.014844479
TCGA-24-1430-01A	B_cells_memory	0
TCGA-24-1430-01A	Plasma_cells	0.044328439
TCGA-24-1430-01A	T_cells_CD8	0
TCGA-24-1430-01A	T_cells_CD4_naive	0
TCGA-24-1430-01A	T_cells_CD4_memory_resting	0.451498852
TCGA-24-1430-01A	T_cells_CD4_memory_activated	0
TCGA-24-1430-01A	T_cells_follicular_helper	0.021583303
TCGA-24-1430-01A	T_cells_regulatory_(Tregs)	0.053031031
TCGA-24-1430-01A	T_cells_gamma_delta	0

TCGA-24-1430-01A	NK_cells_resting	0.027473907
TCGA-24-1430-01A	NK_cells_activated	0.027094438
TCGA-24-1430-01A	Monocytes	0
TCGA-24-1430-01A	Macrophages_M0	0.261746876
TCGA-24-1430-01A	Macrophages_M1	0
TCGA-24-1430-01A	Macrophages_M2	0.027427671
TCGA-24-1430-01A	Dendritic_cells_resting	0
TCGA-24-1430-01A	Dendritic_cells_activated	0
TCGA-24-1430-01A	Mast_cells_resting	0.070971005
TCGA-24-1430-01A	Mast_cells_activated	0
TCGA-24-1430-01A	Eosinophils	0
TCGA-24-1430-01A	Neutrophils	0
TCGA-24-1431-01A	B_cells_naive	0.007459202
TCGA-24-1431-01A	B_cells_memory	0
TCGA-24-1431-01A	Plasma_cells	0.002439752
TCGA-24-1431-01A	T_cells_CD8	0.076904835
TCGA-24-1431-01A	T_cells_CD4_naive	0
TCGA-24-1431-01A	T_cells_CD4_memory_resting	0.241007885
TCGA-24-1431-01A	T_cells_CD4_memory_activated	0
TCGA-24-1431-01A	T_cells_follicular_helper	0.031875516
TCGA-24-1431-01A	T_cells_regulatory_(Tregs)	0.048736383
TCGA-24-1431-01A	T_cells_gamma_delta	0
TCGA-24-1431-01A	NK_cells_resting	0
TCGA-24-1431-01A	NK_cells_activated	0.046858997
TCGA-24-1431-01A	Monocytes	0.150296364
TCGA-24-1431-01A	Macrophages_M0	0
TCGA-24-1431-01A	Macrophages_M1	0.087935854
TCGA-24-1431-01A	Macrophages_M2	0.298448995
TCGA-24-1431-01A	Dendritic_cells_resting	0
TCGA-24-1431-01A	Dendritic_cells_activated	0
TCGA-24-1431-01A	Mast_cells_resting	0
TCGA-24-1431-01A	Mast_cells_activated	0.007968929
TCGA-24-1431-01A	Eosinophils	6.72881E-05

TCGA-24-1431-01A	Neutrophils	0
TCGA-24-1434-01A	B_cells_naive	0.01196381
TCGA-24-1434-01A	B_cells_memory	0
TCGA-24-1434-01A	Plasma_cells	0
TCGA-24-1434-01A	T_cells_CD8	0.027456993
TCGA-24-1434-01A	T_cells_CD4_naive	0
TCGA-24-1434-01A	T_cells_CD4_memory_resting	0.245259741
TCGA-24-1434-01A	T_cells_CD4_memory_activated	0
TCGA-24-1434-01A	T_cells_follicular_helper	0.029292444
TCGA-24-1434-01A	T_cells_regulatory_(Tregs)	0.036453505
TCGA-24-1434-01A	T_cells_gamma_delta	0
TCGA-24-1434-01A	NK_cells_resting	0
TCGA-24-1434-01A	NK_cells_activated	0.046769558
TCGA-24-1434-01A	Monocytes	0.126102405
TCGA-24-1434-01A	Macrophages_M0	0.037330042
TCGA-24-1434-01A	Macrophages_M1	0.099804877
TCGA-24-1434-01A	Macrophages_M2	0.217295554
TCGA-24-1434-01A	Dendritic_cells_resting	0
TCGA-24-1434-01A	Dendritic_cells_activated	0
TCGA-24-1434-01A	Mast_cells_resting	0
TCGA-24-1434-01A	Mast_cells_activated	0.098353181
TCGA-24-1434-01A	Eosinophils	0.022497118
TCGA-24-1434-01A	Neutrophils	0.001420774
TCGA-24-1435-01A	B_cells_naive	0
TCGA-24-1435-01A	B_cells_memory	0.001492974
TCGA-24-1435-01A	Plasma_cells	0
TCGA-24-1435-01A	T_cells_CD8	0.089607896
TCGA-24-1435-01A	T_cells_CD4_naive	0
TCGA-24-1435-01A	T_cells_CD4_memory_resting	0.177742966
TCGA-24-1435-01A	T_cells_CD4_memory_activated	0
TCGA-24-1435-01A	T_cells_follicular_helper	0.009818312
TCGA-24-1435-01A	T_cells_regulatory_(Tregs)	0.042637497
TCGA-24-1435-01A	T_cells_gamma_delta	0

TCGA-24-1435-01A	NK_cells_resting	0
TCGA-24-1435-01A	NK_cells_activated	0.025669756
TCGA-24-1435-01A	Monocytes	0.015860826
TCGA-24-1435-01A	Macrophages_M0	0.149438091
TCGA-24-1435-01A	Macrophages_M1	0.189815428
TCGA-24-1435-01A	Macrophages_M2	0.259005882
TCGA-24-1435-01A	Dendritic_cells_resting	0.006836508
TCGA-24-1435-01A	Dendritic_cells_activated	0.008277482
TCGA-24-1435-01A	Mast_cells_resting	0.021527569
TCGA-24-1435-01A	Mast_cells_activated	0
TCGA-24-1435-01A	Eosinophils	0
TCGA-24-1435-01A	Neutrophils	0.002268814
TCGA-24-1474-01A	B_cells_naive	0
TCGA-24-1474-01A	B_cells_memory	0.006144331
TCGA-24-1474-01A	Plasma_cells	0.004635733
TCGA-24-1474-01A	T_cells_CD8	0.064536802
TCGA-24-1474-01A	T_cells_CD4_naive	0
TCGA-24-1474-01A	T_cells_CD4_memory_resting	0.136098254
TCGA-24-1474-01A	T_cells_CD4_memory_activated	0
TCGA-24-1474-01A	T_cells_follicular_helper	0.02128918
TCGA-24-1474-01A	T_cells_regulatory_(Tregs)	0.004859204
TCGA-24-1474-01A	T_cells_gamma_delta	0
TCGA-24-1474-01A	NK_cells_resting	0
TCGA-24-1474-01A	NK_cells_activated	0.038430506
TCGA-24-1474-01A	Monocytes	0.089151097
TCGA-24-1474-01A	Macrophages_M0	0
TCGA-24-1474-01A	Macrophages_M1	0.050822
TCGA-24-1474-01A	Macrophages_M2	0.428847733
TCGA-24-1474-01A	Dendritic_cells_resting	0
TCGA-24-1474-01A	Dendritic_cells_activated	0.000740434
TCGA-24-1474-01A	Mast_cells_resting	0.135207494
TCGA-24-1474-01A	Mast_cells_activated	0
TCGA-24-1474-01A	Eosinophils	0

TCGA-24-1474-01A	Neutrophils	0.019237233
TCGA-24-1552-01A	B_cells_naive	0.135591132
TCGA-24-1552-01A	B_cells_memory	0
TCGA-24-1552-01A	Plasma_cells	0.015893918
TCGA-24-1552-01A	T_cells_CD8	0.059620246
TCGA-24-1552-01A	T_cells_CD4_naive	0
TCGA-24-1552-01A	T_cells_CD4_memory_resting	0.082661756
TCGA-24-1552-01A	T_cells_CD4_memory_activated	0
TCGA-24-1552-01A	T_cells_follicular_helper	0.028547546
TCGA-24-1552-01A	T_cells_regulatory_(Tregs)	0.052015931
TCGA-24-1552-01A	T_cells_gamma_delta	0.004750984
TCGA-24-1552-01A	NK_cells_resting	0
TCGA-24-1552-01A	NK_cells_activated	0.056447289
TCGA-24-1552-01A	Monocytes	0
TCGA-24-1552-01A	Macrophages_M0	0.200788559
TCGA-24-1552-01A	Macrophages_M1	0.088007839
TCGA-24-1552-01A	Macrophages_M2	0.222472198
TCGA-24-1552-01A	Dendritic_cells_resting	0
TCGA-24-1552-01A	Dendritic_cells_activated	0
TCGA-24-1552-01A	Mast_cells_resting	0
TCGA-24-1552-01A	Mast_cells_activated	0.049965134
TCGA-24-1552-01A	Eosinophils	0.003237468
TCGA-24-1552-01A	Neutrophils	0
TCGA-24-1553-01A	B_cells_naive	0
TCGA-24-1553-01A	B_cells_memory	0
TCGA-24-1553-01A	Plasma_cells	0.112355704
TCGA-24-1553-01A	T_cells_CD8	0.328830876
TCGA-24-1553-01A	T_cells_CD4_naive	0
TCGA-24-1553-01A	T_cells_CD4_memory_resting	0.004241403
TCGA-24-1553-01A	T_cells_CD4_memory_activated	0
TCGA-24-1553-01A	T_cells_follicular_helper	0.043609484
TCGA-24-1553-01A	T_cells_regulatory_(Tregs)	0.105025523
TCGA-24-1553-01A	T_cells_gamma_delta	0.032598632

TCGA-24-1553-01A	NK_cells_resting	0
TCGA-24-1553-01A	NK_cells_activated	0.040129877
TCGA-24-1553-01A	Monocytes	0.032938895
TCGA-24-1553-01A	Macrophages_M0	0.066269404
TCGA-24-1553-01A	Macrophages_M1	0.104406704
TCGA-24-1553-01A	Macrophages_M2	0.129593499
TCGA-24-1553-01A	Dendritic_cells_resting	0
TCGA-24-1553-01A	Dendritic_cells_activated	0
TCGA-24-1553-01A	Mast_cells_resting	0
TCGA-24-1553-01A	Mast_cells_activated	0
TCGA-24-1553-01A	Eosinophils	0
TCGA-24-1553-01A	Neutrophils	0
TCGA-24-1557-01A	B_cells_naive	0.001954439
TCGA-24-1557-01A	B_cells_memory	0
TCGA-24-1557-01A	Plasma_cells	0.021834273
TCGA-24-1557-01A	T_cells_CD8	0.017911158
TCGA-24-1557-01A	T_cells_CD4_naive	0
TCGA-24-1557-01A	T_cells_CD4_memory_resting	0.196016655
TCGA-24-1557-01A	T_cells_CD4_memory_activated	0
TCGA-24-1557-01A	T_cells_follicular_helper	0.000797386
TCGA-24-1557-01A	T_cells_regulatory_(Tregs)	0.033114438
TCGA-24-1557-01A	T_cells_gamma_delta	0
TCGA-24-1557-01A	NK_cells_resting	0
TCGA-24-1557-01A	NK_cells_activated	0.020842382
TCGA-24-1557-01A	Monocytes	0.084441911
TCGA-24-1557-01A	Macrophages_M0	0.256542991
TCGA-24-1557-01A	Macrophages_M1	0.108350583
TCGA-24-1557-01A	Macrophages_M2	0.126922548
TCGA-24-1557-01A	Dendritic_cells_resting	0
TCGA-24-1557-01A	Dendritic_cells_activated	0
TCGA-24-1557-01A	Mast_cells_resting	0.131271237
TCGA-24-1557-01A	Mast_cells_activated	0
TCGA-24-1557-01A	Eosinophils	0

TCGA-24-1557-01A	Neutrophils	0
TCGA-24-1562-01A	B_cells_naive	0
TCGA-24-1562-01A	B_cells_memory	0.003289273
TCGA-24-1562-01A	Plasma_cells	0
TCGA-24-1562-01A	T_cells_CD8	0.058777368
TCGA-24-1562-01A	T_cells_CD4_naive	0
TCGA-24-1562-01A	T_cells_CD4_memory_resting	0.220034691
TCGA-24-1562-01A	T_cells_CD4_memory_activated	0
TCGA-24-1562-01A	T_cells_follicular_helper	0
TCGA-24-1562-01A	T_cells_regulatory_(Tregs)	0.123493419
TCGA-24-1562-01A	T_cells_gamma_delta	0
TCGA-24-1562-01A	NK_cells_resting	0
TCGA-24-1562-01A	NK_cells_activated	0.080496399
TCGA-24-1562-01A	Monocytes	0.001952269
TCGA-24-1562-01A	Macrophages_M0	0.315416518
TCGA-24-1562-01A	Macrophages_M1	0.039418191
TCGA-24-1562-01A	Macrophages_M2	0.090720038
TCGA-24-1562-01A	Dendritic_cells_resting	0.045390124
TCGA-24-1562-01A	Dendritic_cells_activated	0.000273098
TCGA-24-1562-01A	Mast_cells_resting	0.020738612
TCGA-24-1562-01A	Mast_cells_activated	0
TCGA-24-1562-01A	Eosinophils	0
TCGA-24-1562-01A	Neutrophils	0
TCGA-24-1563-01A	B_cells_naive	0.010922904
TCGA-24-1563-01A	B_cells_memory	0.008120942
TCGA-24-1563-01A	Plasma_cells	0
TCGA-24-1563-01A	T_cells_CD8	0.030243197
TCGA-24-1563-01A	T_cells_CD4_naive	0
TCGA-24-1563-01A	T_cells_CD4_memory_resting	0.224514407
TCGA-24-1563-01A	T_cells_CD4_memory_activated	0
TCGA-24-1563-01A	T_cells_follicular_helper	0.020092568
TCGA-24-1563-01A	T_cells_regulatory_(Tregs)	0.033667887
TCGA-24-1563-01A	T_cells_gamma_delta	0

TCGA-24-1563-01A	NK_cells_resting	0
TCGA-24-1563-01A	NK_cells_activated	0.120377861
TCGA-24-1563-01A	Monocytes	0.072298664
TCGA-24-1563-01A	Macrophages_M0	0.17228083
TCGA-24-1563-01A	Macrophages_M1	0.031141992
TCGA-24-1563-01A	Macrophages_M2	0.123364443
TCGA-24-1563-01A	Dendritic_cells_resting	0.006732176
TCGA-24-1563-01A	Dendritic_cells_activated	0.027166697
TCGA-24-1563-01A	Mast_cells_resting	0
TCGA-24-1563-01A	Mast_cells_activated	0.09383826
TCGA-24-1563-01A	Eosinophils	0.016655905
TCGA-24-1563-01A	Neutrophils	0.008581268
TCGA-24-1565-01A	B_cells_naive	0.053545216
TCGA-24-1565-01A	B_cells_memory	0
TCGA-24-1565-01A	Plasma_cells	0.055558705
TCGA-24-1565-01A	T_cells_CD8	0.164868631
TCGA-24-1565-01A	T_cells_CD4_naive	0
TCGA-24-1565-01A	T_cells_CD4_memory_resting	0.097030534
TCGA-24-1565-01A	T_cells_CD4_memory_activated	0
TCGA-24-1565-01A	T_cells_follicular_helper	0.016262855
TCGA-24-1565-01A	T_cells_regulatory_(Tregs)	0.121675827
TCGA-24-1565-01A	T_cells_gamma_delta	0
TCGA-24-1565-01A	NK_cells_resting	0
TCGA-24-1565-01A	NK_cells_activated	0.050743456
TCGA-24-1565-01A	Monocytes	0.083489583
TCGA-24-1565-01A	Macrophages_M0	0
TCGA-24-1565-01A	Macrophages_M1	0.021046122
TCGA-24-1565-01A	Macrophages_M2	0.287359557
TCGA-24-1565-01A	Dendritic_cells_resting	0
TCGA-24-1565-01A	Dendritic_cells_activated	0.01362543
TCGA-24-1565-01A	Mast_cells_resting	0.014581008
TCGA-24-1565-01A	Mast_cells_activated	0
TCGA-24-1565-01A	Eosinophils	0

TCGA-24-1565-01A	Neutrophils	0.020213075
TCGA-24-1567-01A	B_cells_naive	0.127638693
TCGA-24-1567-01A	B_cells_memory	0
TCGA-24-1567-01A	Plasma_cells	0
TCGA-24-1567-01A	T_cells_CD8	0.193302389
TCGA-24-1567-01A	T_cells_CD4_naive	0
TCGA-24-1567-01A	T_cells_CD4_memory_resting	0.030157422
TCGA-24-1567-01A	T_cells_CD4_memory_activated	0
TCGA-24-1567-01A	T_cells_follicular_helper	0.017741076
TCGA-24-1567-01A	T_cells_regulatory_(Tregs)	0.07070149
TCGA-24-1567-01A	T_cells_gamma_delta	0
TCGA-24-1567-01A	NK_cells_resting	0
TCGA-24-1567-01A	NK_cells_activated	0.069300378
TCGA-24-1567-01A	Monocytes	0.011177623
TCGA-24-1567-01A	Macrophages_M0	0.303569505
TCGA-24-1567-01A	Macrophages_M1	0.003196965
TCGA-24-1567-01A	Macrophages_M2	0.113730728
TCGA-24-1567-01A	Dendritic_cells_resting	0
TCGA-24-1567-01A	Dendritic_cells_activated	0.049614936
TCGA-24-1567-01A	Mast_cells_resting	0
TCGA-24-1567-01A	Mast_cells_activated	0.009868795
TCGA-24-1567-01A	Eosinophils	0
TCGA-24-1567-01A	Neutrophils	0
TCGA-24-1604-01A	B_cells_naive	0
TCGA-24-1604-01A	B_cells_memory	0.022597806
TCGA-24-1604-01A	Plasma_cells	0.025171883
TCGA-24-1604-01A	T_cells_CD8	0.054205283
TCGA-24-1604-01A	T_cells_CD4_naive	0
TCGA-24-1604-01A	T_cells_CD4_memory_resting	0.176879922
TCGA-24-1604-01A	T_cells_CD4_memory_activated	0
TCGA-24-1604-01A	T_cells_follicular_helper	0.049050187
TCGA-24-1604-01A	T_cells_regulatory_(Tregs)	0.063288655
TCGA-24-1604-01A	T_cells_gamma_delta	0

TCGA-24-1604-01A	NK_cells_resting	0
TCGA-24-1604-01A	NK_cells_activated	0.087687897
TCGA-24-1604-01A	Monocytes	0.13369118
TCGA-24-1604-01A	Macrophages_M0	0.003383344
TCGA-24-1604-01A	Macrophages_M1	0.048383457
TCGA-24-1604-01A	Macrophages_M2	0.121034582
TCGA-24-1604-01A	Dendritic_cells_resting	0
TCGA-24-1604-01A	Dendritic_cells_activated	0.177196342
TCGA-24-1604-01A	Mast_cells_resting	0.034709258
TCGA-24-1604-01A	Mast_cells_activated	0
TCGA-24-1604-01A	Eosinophils	0
TCGA-24-1604-01A	Neutrophils	0.002720204
TCGA-24-1616-01A	B_cells_naive	0.049054709
TCGA-24-1616-01A	B_cells_memory	0
TCGA-24-1616-01A	Plasma_cells	0
TCGA-24-1616-01A	T_cells_CD8	0.030623941
TCGA-24-1616-01A	T_cells_CD4_naive	0
TCGA-24-1616-01A	T_cells_CD4_memory_resting	0.264709369
TCGA-24-1616-01A	T_cells_CD4_memory_activated	0
TCGA-24-1616-01A	T_cells_follicular_helper	0.00804571
TCGA-24-1616-01A	T_cells_regulatory_(Tregs)	0.054047831
TCGA-24-1616-01A	T_cells_gamma_delta	0
TCGA-24-1616-01A	NK_cells_resting	0
TCGA-24-1616-01A	NK_cells_activated	0.118919096
TCGA-24-1616-01A	Monocytes	0.021857255
TCGA-24-1616-01A	Macrophages_M0	0.157914209
TCGA-24-1616-01A	Macrophages_M1	0.07380845
TCGA-24-1616-01A	Macrophages_M2	0.117113295
TCGA-24-1616-01A	Dendritic_cells_resting	0
TCGA-24-1616-01A	Dendritic_cells_activated	0.086068525
TCGA-24-1616-01A	Mast_cells_resting	0.01783761
TCGA-24-1616-01A	Mast_cells_activated	0
TCGA-24-1616-01A	Eosinophils	0

TCGA-24-1616-01A	Neutrophils	0
TCGA-24-1842-01A	B_cells_naive	0.002830301
TCGA-24-1842-01A	B_cells_memory	0
TCGA-24-1842-01A	Plasma_cells	0.029886246
TCGA-24-1842-01A	T_cells_CD8	0.136776165
TCGA-24-1842-01A	T_cells_CD4_naive	0
TCGA-24-1842-01A	T_cells_CD4_memory_resting	0.11628221
TCGA-24-1842-01A	T_cells_CD4_memory_activated	0.041092364
TCGA-24-1842-01A	T_cells_follicular_helper	0.024931318
TCGA-24-1842-01A	T_cells_regulatory_(Tregs)	0.09518094
TCGA-24-1842-01A	T_cells_gamma_delta	0
TCGA-24-1842-01A	NK_cells_resting	0.000318078
TCGA-24-1842-01A	NK_cells_activated	0.064263296
TCGA-24-1842-01A	Monocytes	0.015389752
TCGA-24-1842-01A	Macrophages_M0	0.287094297
TCGA-24-1842-01A	Macrophages_M1	0.072050359
TCGA-24-1842-01A	Macrophages_M2	0.097363101
TCGA-24-1842-01A	Dendritic_cells_resting	0.000328845
TCGA-24-1842-01A	Dendritic_cells_activated	0
TCGA-24-1842-01A	Mast_cells_resting	0.016212726
TCGA-24-1842-01A	Mast_cells_activated	0
TCGA-24-1842-01A	Eosinophils	0
TCGA-24-1842-01A	Neutrophils	0
TCGA-24-1847-01A	B_cells_naive	0.012926018
TCGA-24-1847-01A	B_cells_memory	0
TCGA-24-1847-01A	Plasma_cells	0.02258643
TCGA-24-1847-01A	T_cells_CD8	0.306335118
TCGA-24-1847-01A	T_cells_CD4_naive	0
TCGA-24-1847-01A	T_cells_CD4_memory_resting	0.125011939
TCGA-24-1847-01A	T_cells_CD4_memory_activated	0.059218925
TCGA-24-1847-01A	T_cells_follicular_helper	0.011119084
TCGA-24-1847-01A	T_cells_regulatory_(Tregs)	0.080096124
TCGA-24-1847-01A	T_cells_gamma_delta	0

TCGA-24-1847-01A	NK_cells_resting	0.026344725
TCGA-24-1847-01A	NK_cells_activated	0
TCGA-24-1847-01A	Monocytes	0.014848019
TCGA-24-1847-01A	Macrophages_M0	0.109049278
TCGA-24-1847-01A	Macrophages_M1	0.088077562
TCGA-24-1847-01A	Macrophages_M2	0.137639278
TCGA-24-1847-01A	Dendritic_cells_resting	0
TCGA-24-1847-01A	Dendritic_cells_activated	0
TCGA-24-1847-01A	Mast_cells_resting	0.0067475
TCGA-24-1847-01A	Mast_cells_activated	0
TCGA-24-1847-01A	Eosinophils	0
TCGA-24-1847-01A	Neutrophils	0
TCGA-24-1850-01A	B_cells_naive	0
TCGA-24-1850-01A	B_cells_memory	0
TCGA-24-1850-01A	Plasma_cells	0.08035531
TCGA-24-1850-01A	T_cells_CD8	0.165954957
TCGA-24-1850-01A	T_cells_CD4_naive	0
TCGA-24-1850-01A	T_cells_CD4_memory_resting	0.091672269
TCGA-24-1850-01A	T_cells_CD4_memory_activated	0
TCGA-24-1850-01A	T_cells_follicular_helper	0.018415787
TCGA-24-1850-01A	T_cells_regulatory_(Tregs)	0.08742342
TCGA-24-1850-01A	T_cells_gamma_delta	0
TCGA-24-1850-01A	NK_cells_resting	0
TCGA-24-1850-01A	NK_cells_activated	0.0300673
TCGA-24-1850-01A	Monocytes	0.004173085
TCGA-24-1850-01A	Macrophages_M0	0.074359093
TCGA-24-1850-01A	Macrophages_M1	0.153270615
TCGA-24-1850-01A	Macrophages_M2	0.27933258
TCGA-24-1850-01A	Dendritic_cells_resting	0
TCGA-24-1850-01A	Dendritic_cells_activated	0
TCGA-24-1850-01A	Mast_cells_resting	0
TCGA-24-1850-01A	Mast_cells_activated	0.006369366
TCGA-24-1850-01A	Eosinophils	0

TCGA-24-1850-01A	Neutrophils	0.00860622
TCGA-24-1923-01A	B_cells_naive	0.099213986
TCGA-24-1923-01A	B_cells_memory	0
TCGA-24-1923-01A	Plasma_cells	0.000611227
TCGA-24-1923-01A	T_cells_CD8	0.015430333
TCGA-24-1923-01A	T_cells_CD4_naive	0
TCGA-24-1923-01A	T_cells_CD4_memory_resting	0.212704012
TCGA-24-1923-01A	T_cells_CD4_memory_activated	0
TCGA-24-1923-01A	T_cells_follicular_helper	0.011851569
TCGA-24-1923-01A	T_cells_regulatory_(Tregs)	0.017792921
TCGA-24-1923-01A	T_cells_gamma_delta	0
TCGA-24-1923-01A	NK_cells_resting	0
TCGA-24-1923-01A	NK_cells_activated	0.026003611
TCGA-24-1923-01A	Monocytes	0.156788989
TCGA-24-1923-01A	Macrophages_M0	0.076078478
TCGA-24-1923-01A	Macrophages_M1	0.03949941
TCGA-24-1923-01A	Macrophages_M2	0.229846013
TCGA-24-1923-01A	Dendritic_cells_resting	0
TCGA-24-1923-01A	Dendritic_cells_activated	0.092275919
TCGA-24-1923-01A	Mast_cells_resting	0
TCGA-24-1923-01A	Mast_cells_activated	0.021903533
TCGA-24-1923-01A	Eosinophils	0
TCGA-24-1923-01A	Neutrophils	0
TCGA-24-1928-01A	B_cells_naive	0.005031479
TCGA-24-1928-01A	B_cells_memory	0
TCGA-24-1928-01A	Plasma_cells	0
TCGA-24-1928-01A	T_cells_CD8	0.096617521
TCGA-24-1928-01A	T_cells_CD4_naive	0
TCGA-24-1928-01A	T_cells_CD4_memory_resting	0.34119276
TCGA-24-1928-01A	T_cells_CD4_memory_activated	0
TCGA-24-1928-01A	T_cells_follicular_helper	0.024620278
TCGA-24-1928-01A	T_cells_regulatory_(Tregs)	0.048052628
TCGA-24-1928-01A	T_cells_gamma_delta	0

TCGA-24-1928-01A	NK_cells_resting	0
TCGA-24-1928-01A	NK_cells_activated	0.010872601
TCGA-24-1928-01A	Monocytes	0.064151457
TCGA-24-1928-01A	Macrophages_M0	0
TCGA-24-1928-01A	Macrophages_M1	0.110651335
TCGA-24-1928-01A	Macrophages_M2	0.246475209
TCGA-24-1928-01A	Dendritic_cells_resting	0
TCGA-24-1928-01A	Dendritic_cells_activated	0
TCGA-24-1928-01A	Mast_cells_resting	0
TCGA-24-1928-01A	Mast_cells_activated	0.052334732
TCGA-24-1928-01A	Eosinophils	0
TCGA-24-1928-01A	Neutrophils	0
TCGA-24-2024-01A	B_cells_naive	0.011661044
TCGA-24-2024-01A	B_cells_memory	0.027505149
TCGA-24-2024-01A	Plasma_cells	0.02898968
TCGA-24-2024-01A	T_cells_CD8	0.052660111
TCGA-24-2024-01A	T_cells_CD4_naive	0
TCGA-24-2024-01A	T_cells_CD4_memory_resting	0.133540175
TCGA-24-2024-01A	T_cells_CD4_memory_activated	0
TCGA-24-2024-01A	T_cells_follicular_helper	0.062508415
TCGA-24-2024-01A	T_cells_regulatory_(Tregs)	0.021899282
TCGA-24-2024-01A	T_cells_gamma_delta	0
TCGA-24-2024-01A	NK_cells_resting	0
TCGA-24-2024-01A	NK_cells_activated	0.042584752
TCGA-24-2024-01A	Monocytes	0.048272033
TCGA-24-2024-01A	Macrophages_M0	0.215756665
TCGA-24-2024-01A	Macrophages_M1	0.085624235
TCGA-24-2024-01A	Macrophages_M2	0.18361449
TCGA-24-2024-01A	Dendritic_cells_resting	0
TCGA-24-2024-01A	Dendritic_cells_activated	0
TCGA-24-2024-01A	Mast_cells_resting	0
TCGA-24-2024-01A	Mast_cells_activated	0.075834068
TCGA-24-2024-01A	Eosinophils	0

TCGA-24-2024-01A	Neutrophils	0.009549901
TCGA-24-2026-01A	B_cells_naive	0.020531686
TCGA-24-2026-01A	B_cells_memory	0
TCGA-24-2026-01A	Plasma_cells	0.121649732
TCGA-24-2026-01A	T_cells_CD8	0.092386907
TCGA-24-2026-01A	T_cells_CD4_naive	0
TCGA-24-2026-01A	T_cells_CD4_memory_resting	0.082789682
TCGA-24-2026-01A	T_cells_CD4_memory_activated	0
TCGA-24-2026-01A	T_cells_follicular_helper	0.02833076
TCGA-24-2026-01A	T_cells_regulatory_(Tregs)	0.097354322
TCGA-24-2026-01A	T_cells_gamma_delta	0
TCGA-24-2026-01A	NK_cells_resting	0.018010701
TCGA-24-2026-01A	NK_cells_activated	0.049720537
TCGA-24-2026-01A	Monocytes	0.014432117
TCGA-24-2026-01A	Macrophages_M0	0.173033687
TCGA-24-2026-01A	Macrophages_M1	0.11288826
TCGA-24-2026-01A	Macrophages_M2	0.171496537
TCGA-24-2026-01A	Dendritic_cells_resting	0
TCGA-24-2026-01A	Dendritic_cells_activated	0
TCGA-24-2026-01A	Mast_cells_resting	0
TCGA-24-2026-01A	Mast_cells_activated	0.012171368
TCGA-24-2026-01A	Eosinophils	0
TCGA-24-2026-01A	Neutrophils	0.005203703
TCGA-24-2027-01A	B_cells_naive	0.065393704
TCGA-24-2027-01A	B_cells_memory	0
TCGA-24-2027-01A	Plasma_cells	0
TCGA-24-2027-01A	T_cells_CD8	0.080340148
TCGA-24-2027-01A	T_cells_CD4_naive	0
TCGA-24-2027-01A	T_cells_CD4_memory_resting	0.121166859
TCGA-24-2027-01A	T_cells_CD4_memory_activated	0
TCGA-24-2027-01A	T_cells_follicular_helper	0.113172636
TCGA-24-2027-01A	T_cells_regulatory_(Tregs)	0.043879756
TCGA-24-2027-01A	T_cells_gamma_delta	0

TCGA-24-2027-01A	NK_cells_resting	0
TCGA-24-2027-01A	NK_cells_activated	0.118465221
TCGA-24-2027-01A	Monocytes	0.011082308
TCGA-24-2027-01A	Macrophages_M0	0.109461897
TCGA-24-2027-01A	Macrophages_M1	0.070355148
TCGA-24-2027-01A	Macrophages_M2	0.18674503
TCGA-24-2027-01A	Dendritic_cells_resting	0
TCGA-24-2027-01A	Dendritic_cells_activated	0.056231637
TCGA-24-2027-01A	Mast_cells_resting	0.023705655
TCGA-24-2027-01A	Mast_cells_activated	0
TCGA-24-2027-01A	Eosinophils	0
TCGA-24-2027-01A	Neutrophils	0
TCGA-24-2033-01A	B_cells_naive	0.038553874
TCGA-24-2033-01A	B_cells_memory	0
TCGA-24-2033-01A	Plasma_cells	0.034083506
TCGA-24-2033-01A	T_cells_CD8	0.05540616
TCGA-24-2033-01A	T_cells_CD4_naive	0
TCGA-24-2033-01A	T_cells_CD4_memory_resting	0.090356069
TCGA-24-2033-01A	T_cells_CD4_memory_activated	0
TCGA-24-2033-01A	T_cells_follicular_helper	0.030501201
TCGA-24-2033-01A	T_cells_regulatory_(Tregs)	0.025485111
TCGA-24-2033-01A	T_cells_gamma_delta	0
TCGA-24-2033-01A	NK_cells_resting	0
TCGA-24-2033-01A	NK_cells_activated	0.04620713
TCGA-24-2033-01A	Monocytes	0.025425426
TCGA-24-2033-01A	Macrophages_M0	0.081508525
TCGA-24-2033-01A	Macrophages_M1	0.068139235
TCGA-24-2033-01A	Macrophages_M2	0.263889879
TCGA-24-2033-01A	Dendritic_cells_resting	0
TCGA-24-2033-01A	Dendritic_cells_activated	0
TCGA-24-2033-01A	Mast_cells_resting	0
TCGA-24-2033-01A	Mast_cells_activated	0.159032453
TCGA-24-2033-01A	Eosinophils	0.066447109

TCGA-24-2033-01A	Neutrophils	0.014964321
TCGA-24-2036-01A	B_cells_naive	0.049275986
TCGA-24-2036-01A	B_cells_memory	0
TCGA-24-2036-01A	Plasma_cells	0.061029643
TCGA-24-2036-01A	T_cells_CD8	0
TCGA-24-2036-01A	T_cells_CD4_naive	0
TCGA-24-2036-01A	T_cells_CD4_memory_resting	0.177669826
TCGA-24-2036-01A	T_cells_CD4_memory_activated	0
TCGA-24-2036-01A	T_cells_follicular_helper	0.015894264
TCGA-24-2036-01A	T_cells_regulatory_(Tregs)	0.050598194
TCGA-24-2036-01A	T_cells_gamma_delta	0
TCGA-24-2036-01A	NK_cells_resting	0
TCGA-24-2036-01A	NK_cells_activated	0.059983613
TCGA-24-2036-01A	Monocytes	0.024865356
TCGA-24-2036-01A	Macrophages_M0	0.166018405
TCGA-24-2036-01A	Macrophages_M1	0
TCGA-24-2036-01A	Macrophages_M2	0.264608351
TCGA-24-2036-01A	Dendritic_cells_resting	0
TCGA-24-2036-01A	Dendritic_cells_activated	0.050351931
TCGA-24-2036-01A	Mast_cells_resting	0.07970443
TCGA-24-2036-01A	Mast_cells_activated	0
TCGA-24-2036-01A	Eosinophils	0
TCGA-24-2036-01A	Neutrophils	0
TCGA-24-2038-01A	B_cells_naive	0.025540213
TCGA-24-2038-01A	B_cells_memory	0
TCGA-24-2038-01A	Plasma_cells	0.002775044
TCGA-24-2038-01A	T_cells_CD8	0.027460876
TCGA-24-2038-01A	T_cells_CD4_naive	0
TCGA-24-2038-01A	T_cells_CD4_memory_resting	0.22797378
TCGA-24-2038-01A	T_cells_CD4_memory_activated	0
TCGA-24-2038-01A	T_cells_follicular_helper	0
TCGA-24-2038-01A	T_cells_regulatory_(Tregs)	0.042300861
TCGA-24-2038-01A	T_cells_gamma_delta	0

TCGA-24-2038-01A	NK_cells_resting	0
TCGA-24-2038-01A	NK_cells_activated	0.120882156
TCGA-24-2038-01A	Monocytes	0.233594792
TCGA-24-2038-01A	Macrophages_M0	0.123933314
TCGA-24-2038-01A	Macrophages_M1	0.004650542
TCGA-24-2038-01A	Macrophages_M2	0.133982759
TCGA-24-2038-01A	Dendritic_cells_resting	0
TCGA-24-2038-01A	Dendritic_cells_activated	0.015691325
TCGA-24-2038-01A	Mast_cells_resting	0
TCGA-24-2038-01A	Mast_cells_activated	0.041214337
TCGA-24-2038-01A	Eosinophils	0
TCGA-24-2038-01A	Neutrophils	0
TCGA-24-2254-01A	B_cells_naive	0.00224134
TCGA-24-2254-01A	B_cells_memory	0
TCGA-24-2254-01A	Plasma_cells	0
TCGA-24-2254-01A	T_cells_CD8	0
TCGA-24-2254-01A	T_cells_CD4_naive	0
TCGA-24-2254-01A	T_cells_CD4_memory_resting	0.148634333
TCGA-24-2254-01A	T_cells_CD4_memory_activated	0
TCGA-24-2254-01A	T_cells_follicular_helper	0.013719955
TCGA-24-2254-01A	T_cells_regulatory_(Tregs)	0.037155784
TCGA-24-2254-01A	T_cells_gamma_delta	0
TCGA-24-2254-01A	NK_cells_resting	0.002312711
TCGA-24-2254-01A	NK_cells_activated	0
TCGA-24-2254-01A	Monocytes	0.036391066
TCGA-24-2254-01A	Macrophages_M0	0.375818548
TCGA-24-2254-01A	Macrophages_M1	0.018531099
TCGA-24-2254-01A	Macrophages_M2	0.201994564
TCGA-24-2254-01A	Dendritic_cells_resting	0
TCGA-24-2254-01A	Dendritic_cells_activated	0
TCGA-24-2254-01A	Mast_cells_resting	0
TCGA-24-2254-01A	Mast_cells_activated	0.160250629
TCGA-24-2254-01A	Eosinophils	0.000718634

TCGA-24-2254-01A	Neutrophils	0.002231337
TCGA-24-2262-01A	B_cells_naive	0.059790923
TCGA-24-2262-01A	B_cells_memory	0
TCGA-24-2262-01A	Plasma_cells	0.08335937
TCGA-24-2262-01A	T_cells_CD8	0.107990487
TCGA-24-2262-01A	T_cells_CD4_naive	0
TCGA-24-2262-01A	T_cells_CD4_memory_resting	0.147335902
TCGA-24-2262-01A	T_cells_CD4_memory_activated	0
TCGA-24-2262-01A	T_cells_follicular_helper	0.026821394
TCGA-24-2262-01A	T_cells_regulatory_(Tregs)	0.075632856
TCGA-24-2262-01A	T_cells_gamma_delta	0
TCGA-24-2262-01A	NK_cells_resting	0.043030122
TCGA-24-2262-01A	NK_cells_activated	0
TCGA-24-2262-01A	Monocytes	0.007139783
TCGA-24-2262-01A	Macrophages_M0	0.19610588
TCGA-24-2262-01A	Macrophages_M1	0.120176965
TCGA-24-2262-01A	Macrophages_M2	0.070968961
TCGA-24-2262-01A	Dendritic_cells_resting	0.005856068
TCGA-24-2262-01A	Dendritic_cells_activated	0
TCGA-24-2262-01A	Mast_cells_resting	0
TCGA-24-2262-01A	Mast_cells_activated	0.046485567
TCGA-24-2262-01A	Eosinophils	0
TCGA-24-2262-01A	Neutrophils	0.009305721
TCGA-24-2267-01A	B_cells_naive	0.002637541
TCGA-24-2267-01A	B_cells_memory	0
TCGA-24-2267-01A	Plasma_cells	0.173897642
TCGA-24-2267-01A	T_cells_CD8	0.008654857
TCGA-24-2267-01A	T_cells_CD4_naive	0
TCGA-24-2267-01A	T_cells_CD4_memory_resting	0.166380319
TCGA-24-2267-01A	T_cells_CD4_memory_activated	0
TCGA-24-2267-01A	T_cells_follicular_helper	0.041130022
TCGA-24-2267-01A	T_cells_regulatory_(Tregs)	0.065328168
TCGA-24-2267-01A	T_cells_gamma_delta	0

TCGA-24-2267-01A	NK_cells_resting	0.014125348
TCGA-24-2267-01A	NK_cells_activated	0.001096628
TCGA-24-2267-01A	Monocytes	0.003064022
TCGA-24-2267-01A	Macrophages_M0	0.219785865
TCGA-24-2267-01A	Macrophages_M1	0.109574207
TCGA-24-2267-01A	Macrophages_M2	0.184728505
TCGA-24-2267-01A	Dendritic_cells_resting	0
TCGA-24-2267-01A	Dendritic_cells_activated	0
TCGA-24-2267-01A	Mast_cells_resting	0
TCGA-24-2267-01A	Mast_cells_activated	0.009596877
TCGA-24-2267-01A	Eosinophils	0
TCGA-24-2267-01A	Neutrophils	0
TCGA-24-2280-01A	B_cells_naive	0.037668958
TCGA-24-2280-01A	B_cells_memory	0
TCGA-24-2280-01A	Plasma_cells	0.211556787
TCGA-24-2280-01A	T_cells_CD8	0.049890499
TCGA-24-2280-01A	T_cells_CD4_naive	0
TCGA-24-2280-01A	T_cells_CD4_memory_resting	0.164018596
TCGA-24-2280-01A	T_cells_CD4_memory_activated	0
TCGA-24-2280-01A	T_cells_follicular_helper	0.016522777
TCGA-24-2280-01A	T_cells_regulatory_(Tregs)	0.037092112
TCGA-24-2280-01A	T_cells_gamma_delta	0.013270164
TCGA-24-2280-01A	NK_cells_resting	0
TCGA-24-2280-01A	NK_cells_activated	0.033011088
TCGA-24-2280-01A	Monocytes	0.026658146
TCGA-24-2280-01A	Macrophages_M0	0.127581191
TCGA-24-2280-01A	Macrophages_M1	0.018711929
TCGA-24-2280-01A	Macrophages_M2	0.24271082
TCGA-24-2280-01A	Dendritic_cells_resting	0
TCGA-24-2280-01A	Dendritic_cells_activated	0
TCGA-24-2280-01A	Mast_cells_resting	0.021306932
TCGA-24-2280-01A	Mast_cells_activated	0
TCGA-24-2280-01A	Eosinophils	0

TCGA-24-2280-01A	Neutrophils	0
TCGA-24-2288-01A	B_cells_naive	0.062613828
TCGA-24-2288-01A	B_cells_memory	0
TCGA-24-2288-01A	Plasma_cells	0.191752036
TCGA-24-2288-01A	T_cells_CD8	0.12949821
TCGA-24-2288-01A	T_cells_CD4_naive	0
TCGA-24-2288-01A	T_cells_CD4_memory_resting	0.184708821
TCGA-24-2288-01A	T_cells_CD4_memory_activated	0.021282343
TCGA-24-2288-01A	T_cells_follicular_helper	0.047651753
TCGA-24-2288-01A	T_cells_regulatory_(Tregs)	0.044285977
TCGA-24-2288-01A	T_cells_gamma_delta	0
TCGA-24-2288-01A	NK_cells_resting	0.029353583
TCGA-24-2288-01A	NK_cells_activated	0
TCGA-24-2288-01A	Monocytes	0.039610267
TCGA-24-2288-01A	Macrophages_M0	0.010034256
TCGA-24-2288-01A	Macrophages_M1	0.093719678
TCGA-24-2288-01A	Macrophages_M2	0.129834719
TCGA-24-2288-01A	Dendritic_cells_resting	0
TCGA-24-2288-01A	Dendritic_cells_activated	0.009731323
TCGA-24-2288-01A	Mast_cells_resting	0
TCGA-24-2288-01A	Mast_cells_activated	0.005923206
TCGA-24-2288-01A	Eosinophils	0
TCGA-24-2288-01A	Neutrophils	0
TCGA-24-2289-01A	B_cells_naive	0.026236942
TCGA-24-2289-01A	B_cells_memory	0
TCGA-24-2289-01A	Plasma_cells	0.001001649
TCGA-24-2289-01A	T_cells_CD8	0.019357849
TCGA-24-2289-01A	T_cells_CD4_naive	0
TCGA-24-2289-01A	T_cells_CD4_memory_resting	0.19046981
TCGA-24-2289-01A	T_cells_CD4_memory_activated	0
TCGA-24-2289-01A	T_cells_follicular_helper	0.009435584
TCGA-24-2289-01A	T_cells_regulatory_(Tregs)	0.059610732
TCGA-24-2289-01A	T_cells_gamma_delta	0

TCGA-24-2289-01A	NK_cells_resting	0
TCGA-24-2289-01A	NK_cells_activated	0.032282138
TCGA-24-2289-01A	Monocytes	0.192546278
TCGA-24-2289-01A	Macrophages_M0	0.003245789
TCGA-24-2289-01A	Macrophages_M1	0.037116896
TCGA-24-2289-01A	Macrophages_M2	0.328028982
TCGA-24-2289-01A	Dendritic_cells_resting	0
TCGA-24-2289-01A	Dendritic_cells_activated	0.008391546
TCGA-24-2289-01A	Mast_cells_resting	0
TCGA-24-2289-01A	Mast_cells_activated	0.083808776
TCGA-24-2289-01A	Eosinophils	0.008467028
TCGA-24-2289-01A	Neutrophils	0
TCGA-24-2293-01A	B_cells_naive	0.036785407
TCGA-24-2293-01A	B_cells_memory	0
TCGA-24-2293-01A	Plasma_cells	0.001531901
TCGA-24-2293-01A	T_cells_CD8	0.079589162
TCGA-24-2293-01A	T_cells_CD4_naive	0
TCGA-24-2293-01A	T_cells_CD4_memory_resting	0.203445287
TCGA-24-2293-01A	T_cells_CD4_memory_activated	0
TCGA-24-2293-01A	T_cells_follicular_helper	0
TCGA-24-2293-01A	T_cells_regulatory_(Tregs)	0.074183641
TCGA-24-2293-01A	T_cells_gamma_delta	0
TCGA-24-2293-01A	NK_cells_resting	0
TCGA-24-2293-01A	NK_cells_activated	0.017286704
TCGA-24-2293-01A	Monocytes	0.049386733
TCGA-24-2293-01A	Macrophages_M0	0.2389626
TCGA-24-2293-01A	Macrophages_M1	0.099567568
TCGA-24-2293-01A	Macrophages_M2	0.171791675
TCGA-24-2293-01A	Dendritic_cells_resting	0.001047581
TCGA-24-2293-01A	Dendritic_cells_activated	0
TCGA-24-2293-01A	Mast_cells_resting	0.02524086
TCGA-24-2293-01A	Mast_cells_activated	0
TCGA-24-2293-01A	Eosinophils	0

TCGA-24-2293-01A	Neutrophils	0.001180882
TCGA-24-2298-01A	B_cells_naive	0
TCGA-24-2298-01A	B_cells_memory	0.000639002
TCGA-24-2298-01A	Plasma_cells	0.03871331
TCGA-24-2298-01A	T_cells_CD8	0.01750014
TCGA-24-2298-01A	T_cells_CD4_naive	0
TCGA-24-2298-01A	T_cells_CD4_memory_resting	0.213744258
TCGA-24-2298-01A	T_cells_CD4_memory_activated	0
TCGA-24-2298-01A	T_cells_follicular_helper	0.070351884
TCGA-24-2298-01A	T_cells_regulatory_(Tregs)	0.049156366
TCGA-24-2298-01A	T_cells_gamma_delta	0
TCGA-24-2298-01A	NK_cells_resting	0
TCGA-24-2298-01A	NK_cells_activated	0.05422451
TCGA-24-2298-01A	Monocytes	0
TCGA-24-2298-01A	Macrophages_M0	0.23081784
TCGA-24-2298-01A	Macrophages_M1	0.020391777
TCGA-24-2298-01A	Macrophages_M2	0.117837188
TCGA-24-2298-01A	Dendritic_cells_resting	0
TCGA-24-2298-01A	Dendritic_cells_activated	0.13522241
TCGA-24-2298-01A	Mast_cells_resting	0
TCGA-24-2298-01A	Mast_cells_activated	0.051401316
TCGA-24-2298-01A	Eosinophils	0
TCGA-24-2298-01A	Neutrophils	0
TCGA-25-1312-01A	B_cells_naive	0.033709211
TCGA-25-1312-01A	B_cells_memory	0
TCGA-25-1312-01A	Plasma_cells	0
TCGA-25-1312-01A	T_cells_CD8	0.05034945
TCGA-25-1312-01A	T_cells_CD4_naive	0
TCGA-25-1312-01A	T_cells_CD4_memory_resting	0.17996474
TCGA-25-1312-01A	T_cells_CD4_memory_activated	0
TCGA-25-1312-01A	T_cells_follicular_helper	0.007394634
TCGA-25-1312-01A	T_cells_regulatory_(Tregs)	0.06623572
TCGA-25-1312-01A	T_cells_gamma_delta	0

TCGA-25-1312-01A	NK_cells_resting	0
TCGA-25-1312-01A	NK_cells_activated	0.133848067
TCGA-25-1312-01A	Monocytes	0.134869403
TCGA-25-1312-01A	Macrophages_M0	0.015369465
TCGA-25-1312-01A	Macrophages_M1	0.037865489
TCGA-25-1312-01A	Macrophages_M2	0.253200774
TCGA-25-1312-01A	Dendritic_cells_resting	0
TCGA-25-1312-01A	Dendritic_cells_activated	0.082611874
TCGA-25-1312-01A	Mast_cells_resting	0
TCGA-25-1312-01A	Mast_cells_activated	0
TCGA-25-1312-01A	Eosinophils	0.004581174
TCGA-25-1312-01A	Neutrophils	0
TCGA-25-1317-01A	B_cells_naive	0.077981481
TCGA-25-1317-01A	B_cells_memory	0
TCGA-25-1317-01A	Plasma_cells	0.275743722
TCGA-25-1317-01A	T_cells_CD8	0.065906817
TCGA-25-1317-01A	T_cells_CD4_naive	0
TCGA-25-1317-01A	T_cells_CD4_memory_resting	0.107331255
TCGA-25-1317-01A	T_cells_CD4_memory_activated	0
TCGA-25-1317-01A	T_cells_follicular_helper	0.024526112
TCGA-25-1317-01A	T_cells_regulatory_(Tregs)	0.058469736
TCGA-25-1317-01A	T_cells_gamma_delta	0
TCGA-25-1317-01A	NK_cells_resting	0
TCGA-25-1317-01A	NK_cells_activated	0.05360145
TCGA-25-1317-01A	Monocytes	0.01871502
TCGA-25-1317-01A	Macrophages_M0	0.158400934
TCGA-25-1317-01A	Macrophages_M1	0.059612711
TCGA-25-1317-01A	Macrophages_M2	0.086247491
TCGA-25-1317-01A	Dendritic_cells_resting	0
TCGA-25-1317-01A	Dendritic_cells_activated	0
TCGA-25-1317-01A	Mast_cells_resting	0
TCGA-25-1317-01A	Mast_cells_activated	0.013463272
TCGA-25-1317-01A	Eosinophils	0

TCGA-25-1317-01A	Neutrophils	0
TCGA-25-1318-01A	B_cells_naive	0
TCGA-25-1318-01A	B_cells_memory	0
TCGA-25-1318-01A	Plasma_cells	0.011826589
TCGA-25-1318-01A	T_cells_CD8	0.026767418
TCGA-25-1318-01A	T_cells_CD4_naive	0
TCGA-25-1318-01A	T_cells_CD4_memory_resting	0.15452628
TCGA-25-1318-01A	T_cells_CD4_memory_activated	0
TCGA-25-1318-01A	T_cells_follicular_helper	0.037342661
TCGA-25-1318-01A	T_cells_regulatory_(Tregs)	0.029834299
TCGA-25-1318-01A	T_cells_gamma_delta	0
TCGA-25-1318-01A	NK_cells_resting	0
TCGA-25-1318-01A	NK_cells_activated	0.084294581
TCGA-25-1318-01A	Monocytes	0.008771708
TCGA-25-1318-01A	Macrophages_M0	0.285587156
TCGA-25-1318-01A	Macrophages_M1	0.0062712
TCGA-25-1318-01A	Macrophages_M2	0.257278911
TCGA-25-1318-01A	Dendritic_cells_resting	0
TCGA-25-1318-01A	Dendritic_cells_activated	0
TCGA-25-1318-01A	Mast_cells_resting	0
TCGA-25-1318-01A	Mast_cells_activated	0.080507382
TCGA-25-1318-01A	Eosinophils	0.016991814
TCGA-25-1318-01A	Neutrophils	0
TCGA-25-1319-01A	B_cells_naive	0.007361849
TCGA-25-1319-01A	B_cells_memory	0
TCGA-25-1319-01A	Plasma_cells	0.013185571
TCGA-25-1319-01A	T_cells_CD8	0.098017412
TCGA-25-1319-01A	T_cells_CD4_naive	0
TCGA-25-1319-01A	T_cells_CD4_memory_resting	0.1597595
TCGA-25-1319-01A	T_cells_CD4_memory_activated	0
TCGA-25-1319-01A	T_cells_follicular_helper	0.040202895
TCGA-25-1319-01A	T_cells_regulatory_(Tregs)	0.042329509
TCGA-25-1319-01A	T_cells_gamma_delta	0

TCGA-25-1319-01A	NK_cells_resting	0
TCGA-25-1319-01A	NK_cells_activated	0.02679557
TCGA-25-1319-01A	Monocytes	0.015421533
TCGA-25-1319-01A	Macrophages_M0	0.033080419
TCGA-25-1319-01A	Macrophages_M1	0.116085767
TCGA-25-1319-01A	Macrophages_M2	0.340499716
TCGA-25-1319-01A	Dendritic_cells_resting	0
TCGA-25-1319-01A	Dendritic_cells_activated	0.059329638
TCGA-25-1319-01A	Mast_cells_resting	0.045907431
TCGA-25-1319-01A	Mast_cells_activated	0
TCGA-25-1319-01A	Eosinophils	0
TCGA-25-1319-01A	Neutrophils	0.002023191
TCGA-25-1322-01A	B_cells_naive	0.041077211
TCGA-25-1322-01A	B_cells_memory	0
TCGA-25-1322-01A	Plasma_cells	0.008584978
TCGA-25-1322-01A	T_cells_CD8	0
TCGA-25-1322-01A	T_cells_CD4_naive	0
TCGA-25-1322-01A	T_cells_CD4_memory_resting	0.195115064
TCGA-25-1322-01A	T_cells_CD4_memory_activated	0
TCGA-25-1322-01A	T_cells_follicular_helper	0.053405956
TCGA-25-1322-01A	T_cells_regulatory_(Tregs)	0.00718426
TCGA-25-1322-01A	T_cells_gamma_delta	0
TCGA-25-1322-01A	NK_cells_resting	0
TCGA-25-1322-01A	NK_cells_activated	0.036174807
TCGA-25-1322-01A	Monocytes	0.193750338
TCGA-25-1322-01A	Macrophages_M0	0.055481708
TCGA-25-1322-01A	Macrophages_M1	0.113701299
TCGA-25-1322-01A	Macrophages_M2	0.161564071
TCGA-25-1322-01A	Dendritic_cells_resting	0
TCGA-25-1322-01A	Dendritic_cells_activated	0.094792797
TCGA-25-1322-01A	Mast_cells_resting	0
TCGA-25-1322-01A	Mast_cells_activated	0.029218673
TCGA-25-1322-01A	Eosinophils	0

TCGA-25-1322-01A	Neutrophils	0.009948839
TCGA-25-1323-01A	B_cells_naive	0
TCGA-25-1323-01A	B_cells_memory	0.005185961
TCGA-25-1323-01A	Plasma_cells	0.009839048
TCGA-25-1323-01A	T_cells_CD8	0
TCGA-25-1323-01A	T_cells_CD4_naive	0
TCGA-25-1323-01A	T_cells_CD4_memory_resting	0.293714467
TCGA-25-1323-01A	T_cells_CD4_memory_activated	0
TCGA-25-1323-01A	T_cells_follicular_helper	0.027820858
TCGA-25-1323-01A	T_cells_regulatory_(Tregs)	0.042966741
TCGA-25-1323-01A	T_cells_gamma_delta	0
TCGA-25-1323-01A	NK_cells_resting	0
TCGA-25-1323-01A	NK_cells_activated	0.158886077
TCGA-25-1323-01A	Monocytes	0.014414187
TCGA-25-1323-01A	Macrophages_M0	0.248592271
TCGA-25-1323-01A	Macrophages_M1	0.024643962
TCGA-25-1323-01A	Macrophages_M2	0.094356394
TCGA-25-1323-01A	Dendritic_cells_resting	0
TCGA-25-1323-01A	Dendritic_cells_activated	0
TCGA-25-1323-01A	Mast_cells_resting	0
TCGA-25-1323-01A	Mast_cells_activated	0.062584691
TCGA-25-1323-01A	Eosinophils	0
TCGA-25-1323-01A	Neutrophils	0.016995344
TCGA-25-1328-01A	B_cells_naive	0.03029583
TCGA-25-1328-01A	B_cells_memory	0
TCGA-25-1328-01A	Plasma_cells	0.013273502
TCGA-25-1328-01A	T_cells_CD8	0.044984113
TCGA-25-1328-01A	T_cells_CD4_naive	0
TCGA-25-1328-01A	T_cells_CD4_memory_resting	0.198557021
TCGA-25-1328-01A	T_cells_CD4_memory_activated	0
TCGA-25-1328-01A	T_cells_follicular_helper	0.008278138
TCGA-25-1328-01A	T_cells_regulatory_(Tregs)	0.065616443
TCGA-25-1328-01A	T_cells_gamma_delta	0

TCGA-25-1328-01A	NK_cells_resting	0.00163738
TCGA-25-1328-01A	NK_cells_activated	0.053464572
TCGA-25-1328-01A	Monocytes	0.054112757
TCGA-25-1328-01A	Macrophages_M0	0.211747724
TCGA-25-1328-01A	Macrophages_M1	0.048725199
TCGA-25-1328-01A	Macrophages_M2	0.186375147
TCGA-25-1328-01A	Dendritic_cells_resting	0.055442617
TCGA-25-1328-01A	Dendritic_cells_activated	0
TCGA-25-1328-01A	Mast_cells_resting	0.025794705
TCGA-25-1328-01A	Mast_cells_activated	0
TCGA-25-1328-01A	Eosinophils	0
TCGA-25-1328-01A	Neutrophils	0.001694854
TCGA-25-1329-01A	B_cells_naive	0.028249505
TCGA-25-1329-01A	B_cells_memory	0
TCGA-25-1329-01A	Plasma_cells	0.041020907
TCGA-25-1329-01A	T_cells_CD8	0.086623369
TCGA-25-1329-01A	T_cells_CD4_naive	0
TCGA-25-1329-01A	T_cells_CD4_memory_resting	0.193918126
TCGA-25-1329-01A	T_cells_CD4_memory_activated	0
TCGA-25-1329-01A	T_cells_follicular_helper	0.002553152
TCGA-25-1329-01A	T_cells_regulatory_(Tregs)	0.034354103
TCGA-25-1329-01A	T_cells_gamma_delta	0
TCGA-25-1329-01A	NK_cells_resting	0.016845405
TCGA-25-1329-01A	NK_cells_activated	0
TCGA-25-1329-01A	Monocytes	0.006424742
TCGA-25-1329-01A	Macrophages_M0	0.259400651
TCGA-25-1329-01A	Macrophages_M1	0.027065506
TCGA-25-1329-01A	Macrophages_M2	0.286356704
TCGA-25-1329-01A	Dendritic_cells_resting	0
TCGA-25-1329-01A	Dendritic_cells_activated	0.008165491
TCGA-25-1329-01A	Mast_cells_resting	0
TCGA-25-1329-01A	Mast_cells_activated	0.003609011
TCGA-25-1329-01A	Eosinophils	0

TCGA-25-1329-01A	Neutrophils	0.00541333
TCGA-25-1623-01A	B_cells_naive	0.013203702
TCGA-25-1623-01A	B_cells_memory	0
TCGA-25-1623-01A	Plasma_cells	0.080299298
TCGA-25-1623-01A	T_cells_CD8	0.101206457
TCGA-25-1623-01A	T_cells_CD4_naive	0
TCGA-25-1623-01A	T_cells_CD4_memory_resting	0.213677772
TCGA-25-1623-01A	T_cells_CD4_memory_activated	0.008421142
TCGA-25-1623-01A	T_cells_follicular_helper	0.017542196
TCGA-25-1623-01A	T_cells_regulatory_(Tregs)	0.094768808
TCGA-25-1623-01A	T_cells_gamma_delta	0
TCGA-25-1623-01A	NK_cells_resting	0
TCGA-25-1623-01A	NK_cells_activated	0.045863607
TCGA-25-1623-01A	Monocytes	0.011637917
TCGA-25-1623-01A	Macrophages_M0	0.144756137
TCGA-25-1623-01A	Macrophages_M1	0.091518965
TCGA-25-1623-01A	Macrophages_M2	0.147152015
TCGA-25-1623-01A	Dendritic_cells_resting	0
TCGA-25-1623-01A	Dendritic_cells_activated	0
TCGA-25-1623-01A	Mast_cells_resting	0.018056528
TCGA-25-1623-01A	Mast_cells_activated	0
TCGA-25-1623-01A	Eosinophils	0
TCGA-25-1623-01A	Neutrophils	0.011895457
TCGA-25-1626-01A	B_cells_naive	0.031760241
TCGA-25-1626-01A	B_cells_memory	0
TCGA-25-1626-01A	Plasma_cells	0.025734911
TCGA-25-1626-01A	T_cells_CD8	0.142306121
TCGA-25-1626-01A	T_cells_CD4_naive	0
TCGA-25-1626-01A	T_cells_CD4_memory_resting	0.137334035
TCGA-25-1626-01A	T_cells_CD4_memory_activated	0
TCGA-25-1626-01A	T_cells_follicular_helper	0.024788254
TCGA-25-1626-01A	T_cells_regulatory_(Tregs)	0.046845529
TCGA-25-1626-01A	T_cells_gamma_delta	0

TCGA-25-1626-01A	NK_cells_resting	0
TCGA-25-1626-01A	NK_cells_activated	0.078082373
TCGA-25-1626-01A	Monocytes	0.116390481
TCGA-25-1626-01A	Macrophages_M0	0.042868336
TCGA-25-1626-01A	Macrophages_M1	0.025615088
TCGA-25-1626-01A	Macrophages_M2	0.107786606
TCGA-25-1626-01A	Dendritic_cells_resting	0.011513709
TCGA-25-1626-01A	Dendritic_cells_activated	0.039423147
TCGA-25-1626-01A	Mast_cells_resting	0
TCGA-25-1626-01A	Mast_cells_activated	0.160909412
TCGA-25-1626-01A	Eosinophils	0.004659045
TCGA-25-1626-01A	Neutrophils	0.003982712
TCGA-25-1627-01A	B_cells_naive	0.069543205
TCGA-25-1627-01A	B_cells_memory	0
TCGA-25-1627-01A	Plasma_cells	0.055212807
TCGA-25-1627-01A	T_cells_CD8	0.02594242
TCGA-25-1627-01A	T_cells_CD4_naive	0
TCGA-25-1627-01A	T_cells_CD4_memory_resting	0.18181439
TCGA-25-1627-01A	T_cells_CD4_memory_activated	0
TCGA-25-1627-01A	T_cells_follicular_helper	0.021094087
TCGA-25-1627-01A	T_cells_regulatory_(Tregs)	0.032839026
TCGA-25-1627-01A	T_cells_gamma_delta	0
TCGA-25-1627-01A	NK_cells_resting	0
TCGA-25-1627-01A	NK_cells_activated	0.07382152
TCGA-25-1627-01A	Monocytes	0.001460866
TCGA-25-1627-01A	Macrophages_M0	0.30767928
TCGA-25-1627-01A	Macrophages_M1	0.037522246
TCGA-25-1627-01A	Macrophages_M2	0.133380187
TCGA-25-1627-01A	Dendritic_cells_resting	0.026375118
TCGA-25-1627-01A	Dendritic_cells_activated	0
TCGA-25-1627-01A	Mast_cells_resting	0
TCGA-25-1627-01A	Mast_cells_activated	0.027775703
TCGA-25-1627-01A	Eosinophils	0

TCGA-25-1627-01A	Neutrophils	0.005539143
TCGA-25-1630-01A	B_cells_naive	0.004333196
TCGA-25-1630-01A	B_cells_memory	0.021691578
TCGA-25-1630-01A	Plasma_cells	0
TCGA-25-1630-01A	T_cells_CD8	0.013681353
TCGA-25-1630-01A	T_cells_CD4_naive	0
TCGA-25-1630-01A	T_cells_CD4_memory_resting	0.094265197
TCGA-25-1630-01A	T_cells_CD4_memory_activated	0
TCGA-25-1630-01A	T_cells_follicular_helper	0.007172168
TCGA-25-1630-01A	T_cells_regulatory_(Tregs)	0.068897743
TCGA-25-1630-01A	T_cells_gamma_delta	0
TCGA-25-1630-01A	NK_cells_resting	0
TCGA-25-1630-01A	NK_cells_activated	0.038263403
TCGA-25-1630-01A	Monocytes	0.008954891
TCGA-25-1630-01A	Macrophages_M0	0.440121291
TCGA-25-1630-01A	Macrophages_M1	0.031628381
TCGA-25-1630-01A	Macrophages_M2	0.114264314
TCGA-25-1630-01A	Dendritic_cells_resting	0.006948434
TCGA-25-1630-01A	Dendritic_cells_activated	0
TCGA-25-1630-01A	Mast_cells_resting	0
TCGA-25-1630-01A	Mast_cells_activated	0.07224574
TCGA-25-1630-01A	Eosinophils	0
TCGA-25-1630-01A	Neutrophils	0.07753231
TCGA-25-1631-01A	B_cells_naive	0.025804971
TCGA-25-1631-01A	B_cells_memory	0
TCGA-25-1631-01A	Plasma_cells	0.004357244
TCGA-25-1631-01A	T_cells_CD8	0.007512615
TCGA-25-1631-01A	T_cells_CD4_naive	0
TCGA-25-1631-01A	T_cells_CD4_memory_resting	0.246636155
TCGA-25-1631-01A	T_cells_CD4_memory_activated	0
TCGA-25-1631-01A	T_cells_follicular_helper	0.002927765
TCGA-25-1631-01A	T_cells_regulatory_(Tregs)	0.068367396
TCGA-25-1631-01A	T_cells_gamma_delta	0

TCGA-25-1631-01A	NK_cells_resting	0
TCGA-25-1631-01A	NK_cells_activated	0.049908169
TCGA-25-1631-01A	Monocytes	0.036998491
TCGA-25-1631-01A	Macrophages_M0	0.203398007
TCGA-25-1631-01A	Macrophages_M1	0.037790453
TCGA-25-1631-01A	Macrophages_M2	0.173923916
TCGA-25-1631-01A	Dendritic_cells_resting	0
TCGA-25-1631-01A	Dendritic_cells_activated	0.060695837
TCGA-25-1631-01A	Mast_cells_resting	0.08167898
TCGA-25-1631-01A	Mast_cells_activated	0
TCGA-25-1631-01A	Eosinophils	0
TCGA-25-1631-01A	Neutrophils	0
TCGA-25-1633-01A	B_cells_naive	0.053444767
TCGA-25-1633-01A	B_cells_memory	0
TCGA-25-1633-01A	Plasma_cells	0
TCGA-25-1633-01A	T_cells_CD8	0.190041087
TCGA-25-1633-01A	T_cells_CD4_naive	0
TCGA-25-1633-01A	T_cells_CD4_memory_resting	0.155914322
TCGA-25-1633-01A	T_cells_CD4_memory_activated	0.042122073
TCGA-25-1633-01A	T_cells_follicular_helper	0.026313001
TCGA-25-1633-01A	T_cells_regulatory_(Tregs)	0.080202797
TCGA-25-1633-01A	T_cells_gamma_delta	0
TCGA-25-1633-01A	NK_cells_resting	0.000251625
TCGA-25-1633-01A	NK_cells_activated	0
TCGA-25-1633-01A	Monocytes	0.078709252
TCGA-25-1633-01A	Macrophages_M0	0.029466304
TCGA-25-1633-01A	Macrophages_M1	0.084549708
TCGA-25-1633-01A	Macrophages_M2	0.190480473
TCGA-25-1633-01A	Dendritic_cells_resting	0
TCGA-25-1633-01A	Dendritic_cells_activated	0.009279345
TCGA-25-1633-01A	Mast_cells_resting	0
TCGA-25-1633-01A	Mast_cells_activated	0.03609115
TCGA-25-1633-01A	Eosinophils	0.00919722

TCGA-25-1633-01A	Neutrophils	0.013936877
TCGA-25-1634-01A	B_cells_naive	0
TCGA-25-1634-01A	B_cells_memory	0.00020615
TCGA-25-1634-01A	Plasma_cells	0.001150995
TCGA-25-1634-01A	T_cells_CD8	0
TCGA-25-1634-01A	T_cells_CD4_naive	0
TCGA-25-1634-01A	T_cells_CD4_memory_resting	0.305204736
TCGA-25-1634-01A	T_cells_CD4_memory_activated	0
TCGA-25-1634-01A	T_cells_follicular_helper	0.002919175
TCGA-25-1634-01A	T_cells_regulatory_(Tregs)	0.134258682
TCGA-25-1634-01A	T_cells_gamma_delta	0
TCGA-25-1634-01A	NK_cells_resting	0
TCGA-25-1634-01A	NK_cells_activated	0.064568877
TCGA-25-1634-01A	Monocytes	0.054887668
TCGA-25-1634-01A	Macrophages_M0	0.250283232
TCGA-25-1634-01A	Macrophages_M1	0
TCGA-25-1634-01A	Macrophages_M2	0.186520483
TCGA-25-1634-01A	Dendritic_cells_resting	0
TCGA-25-1634-01A	Dendritic_cells_activated	0
TCGA-25-1634-01A	Mast_cells_resting	0
TCGA-25-1634-01A	Mast_cells_activated	0
TCGA-25-1634-01A	Eosinophils	0
TCGA-25-1634-01A	Neutrophils	0
TCGA-25-1635-01A	B_cells_naive	0
TCGA-25-1635-01A	B_cells_memory	0
TCGA-25-1635-01A	Plasma_cells	0.014255678
TCGA-25-1635-01A	T_cells_CD8	0.12124455
TCGA-25-1635-01A	T_cells_CD4_naive	0
TCGA-25-1635-01A	T_cells_CD4_memory_resting	0.293118394
TCGA-25-1635-01A	T_cells_CD4_memory_activated	0.008359928
TCGA-25-1635-01A	T_cells_follicular_helper	0
TCGA-25-1635-01A	T_cells_regulatory_(Tregs)	0.047365346
TCGA-25-1635-01A	T_cells_gamma_delta	0

TCGA-25-1635-01A	NK_cells_resting	0
TCGA-25-1635-01A	NK_cells_activated	0
TCGA-25-1635-01A	Monocytes	0.111998324
TCGA-25-1635-01A	Macrophages_M0	0.043522612
TCGA-25-1635-01A	Macrophages_M1	0.070188386
TCGA-25-1635-01A	Macrophages_M2	0.255871315
TCGA-25-1635-01A	Dendritic_cells_resting	0
TCGA-25-1635-01A	Dendritic_cells_activated	0.009995518
TCGA-25-1635-01A	Mast_cells_resting	0.024079948
TCGA-25-1635-01A	Mast_cells_activated	0
TCGA-25-1635-01A	Eosinophils	0
TCGA-25-1635-01A	Neutrophils	0
TCGA-25-1870-01A	B_cells_naive	0
TCGA-25-1870-01A	B_cells_memory	0.014273939
TCGA-25-1870-01A	Plasma_cells	0
TCGA-25-1870-01A	T_cells_CD8	0.013472506
TCGA-25-1870-01A	T_cells_CD4_naive	0
TCGA-25-1870-01A	T_cells_CD4_memory_resting	0.099564182
TCGA-25-1870-01A	T_cells_CD4_memory_activated	0
TCGA-25-1870-01A	T_cells_follicular_helper	0.040468132
TCGA-25-1870-01A	T_cells_regulatory_(Tregs)	0.024378691
TCGA-25-1870-01A	T_cells_gamma_delta	0
TCGA-25-1870-01A	NK_cells_resting	0
TCGA-25-1870-01A	NK_cells_activated	0.10614099
TCGA-25-1870-01A	Monocytes	0.094763145
TCGA-25-1870-01A	Macrophages_M0	0
TCGA-25-1870-01A	Macrophages_M1	0.016428434
TCGA-25-1870-01A	Macrophages_M2	0.358848324
TCGA-25-1870-01A	Dendritic_cells_resting	0
TCGA-25-1870-01A	Dendritic_cells_activated	0.005708111
TCGA-25-1870-01A	Mast_cells_resting	0
TCGA-25-1870-01A	Mast_cells_activated	0.225953546
TCGA-25-1870-01A	Eosinophils	0

TCGA-25-1870-01A	Neutrophils	0
TCGA-25-1877-01A	B_cells_naive	0.039347716
TCGA-25-1877-01A	B_cells_memory	0
TCGA-25-1877-01A	Plasma_cells	0.084611822
TCGA-25-1877-01A	T_cells_CD8	0.148535546
TCGA-25-1877-01A	T_cells_CD4_naive	0
TCGA-25-1877-01A	T_cells_CD4_memory_resting	0
TCGA-25-1877-01A	T_cells_CD4_memory_activated	0.074791886
TCGA-25-1877-01A	T_cells_follicular_helper	0.013219392
TCGA-25-1877-01A	T_cells_regulatory_(Tregs)	0.0123542
TCGA-25-1877-01A	T_cells_gamma_delta	0
TCGA-25-1877-01A	NK_cells_resting	0
TCGA-25-1877-01A	NK_cells_activated	0.043442949
TCGA-25-1877-01A	Monocytes	0
TCGA-25-1877-01A	Macrophages_M0	0.279476915
TCGA-25-1877-01A	Macrophages_M1	0.117628984
TCGA-25-1877-01A	Macrophages_M2	0.180924665
TCGA-25-1877-01A	Dendritic_cells_resting	0
TCGA-25-1877-01A	Dendritic_cells_activated	0
TCGA-25-1877-01A	Mast_cells_resting	0
TCGA-25-1877-01A	Mast_cells_activated	0
TCGA-25-1877-01A	Eosinophils	0.005665925
TCGA-25-1877-01A	Neutrophils	0
TCGA-25-2042-01A	B_cells_naive	0.066109698
TCGA-25-2042-01A	B_cells_memory	0
TCGA-25-2042-01A	Plasma_cells	0.099598579
TCGA-25-2042-01A	T_cells_CD8	0.074789742
TCGA-25-2042-01A	T_cells_CD4_naive	0
TCGA-25-2042-01A	T_cells_CD4_memory_resting	0.219825399
TCGA-25-2042-01A	T_cells_CD4_memory_activated	0
TCGA-25-2042-01A	T_cells_follicular_helper	0
TCGA-25-2042-01A	T_cells_regulatory_(Tregs)	0.030409384
TCGA-25-2042-01A	T_cells_gamma_delta	0

TCGA-25-2042-01A	NK_cells_resting	0
TCGA-25-2042-01A	NK_cells_activated	0.019464265
TCGA-25-2042-01A	Monocytes	0.093808109
TCGA-25-2042-01A	Macrophages_M0	0
TCGA-25-2042-01A	Macrophages_M1	0.022002378
TCGA-25-2042-01A	Macrophages_M2	0.170746487
TCGA-25-2042-01A	Dendritic_cells_resting	0
TCGA-25-2042-01A	Dendritic_cells_activated	0.064785263
TCGA-25-2042-01A	Mast_cells_resting	0
TCGA-25-2042-01A	Mast_cells_activated	0.085286202
TCGA-25-2042-01A	Eosinophils	0.028001573
TCGA-25-2042-01A	Neutrophils	0.025172922
TCGA-25-2391-01A	B_cells_naive	0.046306208
TCGA-25-2391-01A	B_cells_memory	0
TCGA-25-2391-01A	Plasma_cells	0.012140255
TCGA-25-2391-01A	T_cells_CD8	0.102839841
TCGA-25-2391-01A	T_cells_CD4_naive	0
TCGA-25-2391-01A	T_cells_CD4_memory_resting	0.075813099
TCGA-25-2391-01A	T_cells_CD4_memory_activated	0
TCGA-25-2391-01A	T_cells_follicular_helper	0.060019893
TCGA-25-2391-01A	T_cells_regulatory_(Tregs)	0.046566731
TCGA-25-2391-01A	T_cells_gamma_delta	0
TCGA-25-2391-01A	NK_cells_resting	0
TCGA-25-2391-01A	NK_cells_activated	0.075533548
TCGA-25-2391-01A	Monocytes	0.07027556
TCGA-25-2391-01A	Macrophages_M0	0.03481869
TCGA-25-2391-01A	Macrophages_M1	0
TCGA-25-2391-01A	Macrophages_M2	0.152824199
TCGA-25-2391-01A	Dendritic_cells_resting	0
TCGA-25-2391-01A	Dendritic_cells_activated	0.263612733
TCGA-25-2391-01A	Mast_cells_resting	0
TCGA-25-2391-01A	Mast_cells_activated	0
TCGA-25-2391-01A	Eosinophils	0

TCGA-25-2391-01A	Neutrophils	0.059249243
TCGA-25-2392-01A	B_cells_naive	0
TCGA-25-2392-01A	B_cells_memory	0.003908274
TCGA-25-2392-01A	Plasma_cells	0.012727349
TCGA-25-2392-01A	T_cells_CD8	0.032171308
TCGA-25-2392-01A	T_cells_CD4_naive	0
TCGA-25-2392-01A	T_cells_CD4_memory_resting	0.193438941
TCGA-25-2392-01A	T_cells_CD4_memory_activated	0
TCGA-25-2392-01A	T_cells_follicular_helper	0.020004439
TCGA-25-2392-01A	T_cells_regulatory_(Tregs)	0.046184126
TCGA-25-2392-01A	T_cells_gamma_delta	0
TCGA-25-2392-01A	NK_cells_resting	0
TCGA-25-2392-01A	NK_cells_activated	0.015501738
TCGA-25-2392-01A	Monocytes	0.227636085
TCGA-25-2392-01A	Macrophages_M0	0
TCGA-25-2392-01A	Macrophages_M1	0.054002625
TCGA-25-2392-01A	Macrophages_M2	0.373039552
TCGA-25-2392-01A	Dendritic_cells_resting	0
TCGA-25-2392-01A	Dendritic_cells_activated	6.63699E-05
TCGA-25-2392-01A	Mast_cells_resting	0.021319193
TCGA-25-2392-01A	Mast_cells_activated	0
TCGA-25-2392-01A	Eosinophils	0
TCGA-25-2392-01A	Neutrophils	0
TCGA-25-2396-01A	B_cells_naive	0.014008731
TCGA-25-2396-01A	B_cells_memory	0
TCGA-25-2396-01A	Plasma_cells	0.033122004
TCGA-25-2396-01A	T_cells_CD8	0.139312142
TCGA-25-2396-01A	T_cells_CD4_naive	0
TCGA-25-2396-01A	T_cells_CD4_memory_resting	0.212221366
TCGA-25-2396-01A	T_cells_CD4_memory_activated	0
TCGA-25-2396-01A	T_cells_follicular_helper	0.008470701
TCGA-25-2396-01A	T_cells_regulatory_(Tregs)	0.090046423
TCGA-25-2396-01A	T_cells_gamma_delta	0

TCGA-25-2396-01A	NK_cells_resting	0
TCGA-25-2396-01A	NK_cells_activated	0.110101728
TCGA-25-2396-01A	Monocytes	0.035158003
TCGA-25-2396-01A	Macrophages_M0	0.029727888
TCGA-25-2396-01A	Macrophages_M1	0.119602823
TCGA-25-2396-01A	Macrophages_M2	0.173355648
TCGA-25-2396-01A	Dendritic_cells_resting	0
TCGA-25-2396-01A	Dendritic_cells_activated	0
TCGA-25-2396-01A	Mast_cells_resting	0.020333765
TCGA-25-2396-01A	Mast_cells_activated	0
TCGA-25-2396-01A	Eosinophils	0
TCGA-25-2396-01A	Neutrophils	0.014538778
TCGA-25-2398-01A	B_cells_naive	0.009095369
TCGA-25-2398-01A	B_cells_memory	0
TCGA-25-2398-01A	Plasma_cells	0
TCGA-25-2398-01A	T_cells_CD8	0.034461862
TCGA-25-2398-01A	T_cells_CD4_naive	0
TCGA-25-2398-01A	T_cells_CD4_memory_resting	0.137210627
TCGA-25-2398-01A	T_cells_CD4_memory_activated	0
TCGA-25-2398-01A	T_cells_follicular_helper	0.010403326
TCGA-25-2398-01A	T_cells_regulatory_(Tregs)	0.025673611
TCGA-25-2398-01A	T_cells_gamma_delta	0
TCGA-25-2398-01A	NK_cells_resting	0
TCGA-25-2398-01A	NK_cells_activated	0.051682692
TCGA-25-2398-01A	Monocytes	0
TCGA-25-2398-01A	Macrophages_M0	0.540397472
TCGA-25-2398-01A	Macrophages_M1	0.050987275
TCGA-25-2398-01A	Macrophages_M2	0.136710425
TCGA-25-2398-01A	Dendritic_cells_resting	0
TCGA-25-2398-01A	Dendritic_cells_activated	0
TCGA-25-2398-01A	Mast_cells_resting	0.003377341
TCGA-25-2398-01A	Mast_cells_activated	0
TCGA-25-2398-01A	Eosinophils	0

TCGA-25-2398-01A	Neutrophils	0
TCGA-25-2399-01A	B_cells_naive	0.077699947
TCGA-25-2399-01A	B_cells_memory	0
TCGA-25-2399-01A	Plasma_cells	0.014422695
TCGA-25-2399-01A	T_cells_CD8	0.078877145
TCGA-25-2399-01A	T_cells_CD4_naive	0
TCGA-25-2399-01A	T_cells_CD4_memory_resting	0.16506281
TCGA-25-2399-01A	T_cells_CD4_memory_activated	0.036153134
TCGA-25-2399-01A	T_cells_follicular_helper	0.017587087
TCGA-25-2399-01A	T_cells_regulatory_(Tregs)	0.03042623
TCGA-25-2399-01A	T_cells_gamma_delta	0
TCGA-25-2399-01A	NK_cells_resting	0.02779669
TCGA-25-2399-01A	NK_cells_activated	0.095460175
TCGA-25-2399-01A	Monocytes	0.027232215
TCGA-25-2399-01A	Macrophages_M0	0.128890273
TCGA-25-2399-01A	Macrophages_M1	0.092657803
TCGA-25-2399-01A	Macrophages_M2	0.179755311
TCGA-25-2399-01A	Dendritic_cells_resting	0
TCGA-25-2399-01A	Dendritic_cells_activated	0.001520909
TCGA-25-2399-01A	Mast_cells_resting	0.026251265
TCGA-25-2399-01A	Mast_cells_activated	0
TCGA-25-2399-01A	Eosinophils	0
TCGA-25-2399-01A	Neutrophils	0.00020631
TCGA-25-2400-01A	B_cells_naive	0.102434923
TCGA-25-2400-01A	B_cells_memory	0
TCGA-25-2400-01A	Plasma_cells	0.000541236
TCGA-25-2400-01A	T_cells_CD8	0.069237201
TCGA-25-2400-01A	T_cells_CD4_naive	0
TCGA-25-2400-01A	T_cells_CD4_memory_resting	0.124797576
TCGA-25-2400-01A	T_cells_CD4_memory_activated	0
TCGA-25-2400-01A	T_cells_follicular_helper	0.050599608
TCGA-25-2400-01A	T_cells_regulatory_(Tregs)	0.027567841
TCGA-25-2400-01A	T_cells_gamma_delta	0

TCGA-25-2400-01A	NK_cells_resting	0
TCGA-25-2400-01A	NK_cells_activated	0.065259254
TCGA-25-2400-01A	Monocytes	0.123964972
TCGA-25-2400-01A	Macrophages_M0	0.008642222
TCGA-25-2400-01A	Macrophages_M1	0.111021287
TCGA-25-2400-01A	Macrophages_M2	0.265878807
TCGA-25-2400-01A	Dendritic_cells_resting	0
TCGA-25-2400-01A	Dendritic_cells_activated	0.036317392
TCGA-25-2400-01A	Mast_cells_resting	0.013052044
TCGA-25-2400-01A	Mast_cells_activated	0
TCGA-25-2400-01A	Eosinophils	0
TCGA-25-2400-01A	Neutrophils	0.000685637
TCGA-25-2409-01A	B_cells_naive	0.008199643
TCGA-25-2409-01A	B_cells_memory	0
TCGA-25-2409-01A	Plasma_cells	0.020432708
TCGA-25-2409-01A	T_cells_CD8	0.031048926
TCGA-25-2409-01A	T_cells_CD4_naive	0
TCGA-25-2409-01A	T_cells_CD4_memory_resting	0.162702327
TCGA-25-2409-01A	T_cells_CD4_memory_activated	0
TCGA-25-2409-01A	T_cells_follicular_helper	0.007341833
TCGA-25-2409-01A	T_cells_regulatory_(Tregs)	0.087708132
TCGA-25-2409-01A	T_cells_gamma_delta	0
TCGA-25-2409-01A	NK_cells_resting	0.069319866
TCGA-25-2409-01A	NK_cells_activated	0.037288803
TCGA-25-2409-01A	Monocytes	0.082320509
TCGA-25-2409-01A	Macrophages_M0	0.017571282
TCGA-25-2409-01A	Macrophages_M1	0.120051299
TCGA-25-2409-01A	Macrophages_M2	0.295349951
TCGA-25-2409-01A	Dendritic_cells_resting	0.000345263
TCGA-25-2409-01A	Dendritic_cells_activated	0.027970116
TCGA-25-2409-01A	Mast_cells_resting	0.032349341
TCGA-25-2409-01A	Mast_cells_activated	0
TCGA-25-2409-01A	Eosinophils	0

TCGA-25-2409-01A	Neutrophils	0
TCGA-29-1688-01A	B_cells_naive	0.041757721
TCGA-29-1688-01A	B_cells_memory	0
TCGA-29-1688-01A	Plasma_cells	0.002641899
TCGA-29-1688-01A	T_cells_CD8	0.054586779
TCGA-29-1688-01A	T_cells_CD4_naive	0
TCGA-29-1688-01A	T_cells_CD4_memory_resting	0.272254227
TCGA-29-1688-01A	T_cells_CD4_memory_activated	0
TCGA-29-1688-01A	T_cells_follicular_helper	0.01856826
TCGA-29-1688-01A	T_cells_regulatory_(Tregs)	0.045958214
TCGA-29-1688-01A	T_cells_gamma_delta	0
TCGA-29-1688-01A	NK_cells_resting	0.03005425
TCGA-29-1688-01A	NK_cells_activated	0.023736399
TCGA-29-1688-01A	Monocytes	0.026624475
TCGA-29-1688-01A	Macrophages_M0	0.132324699
TCGA-29-1688-01A	Macrophages_M1	0.117530571
TCGA-29-1688-01A	Macrophages_M2	0.208727237
TCGA-29-1688-01A	Dendritic_cells_resting	0
TCGA-29-1688-01A	Dendritic_cells_activated	0
TCGA-29-1688-01A	Mast_cells_resting	0.020054501
TCGA-29-1688-01A	Mast_cells_activated	0
TCGA-29-1688-01A	Eosinophils	0
TCGA-29-1688-01A	Neutrophils	0.005180767
TCGA-29-1690-01A	B_cells_naive	0.002586967
TCGA-29-1690-01A	B_cells_memory	0
TCGA-29-1690-01A	Plasma_cells	0
TCGA-29-1690-01A	T_cells_CD8	0.040667926
TCGA-29-1690-01A	T_cells_CD4_naive	0
TCGA-29-1690-01A	T_cells_CD4_memory_resting	0.189302866
TCGA-29-1690-01A	T_cells_CD4_memory_activated	0.01683441
TCGA-29-1690-01A	T_cells_follicular_helper	0
TCGA-29-1690-01A	T_cells_regulatory_(Tregs)	0.032186685
TCGA-29-1690-01A	T_cells_gamma_delta	0

TCGA-29-1690-01A	NK_cells_resting	0
TCGA-29-1690-01A	NK_cells_activated	0.005320434
TCGA-29-1690-01A	Monocytes	0.152981925
TCGA-29-1690-01A	Macrophages_M0	0.171328004
TCGA-29-1690-01A	Macrophages_M1	0.00166376
TCGA-29-1690-01A	Macrophages_M2	0.21905722
TCGA-29-1690-01A	Dendritic_cells_resting	0.05580905
TCGA-29-1690-01A	Dendritic_cells_activated	0.066630432
TCGA-29-1690-01A	Mast_cells_resting	0.02732177
TCGA-29-1690-01A	Mast_cells_activated	0
TCGA-29-1690-01A	Eosinophils	0.016611421
TCGA-29-1690-01A	Neutrophils	0.001697131
TCGA-29-1694-01A	B_cells_naive	0.006794324
TCGA-29-1694-01A	B_cells_memory	0
TCGA-29-1694-01A	Plasma_cells	0
TCGA-29-1694-01A	T_cells_CD8	0.058580908
TCGA-29-1694-01A	T_cells_CD4_naive	0
TCGA-29-1694-01A	T_cells_CD4_memory_resting	0.325969671
TCGA-29-1694-01A	T_cells_CD4_memory_activated	0
TCGA-29-1694-01A	T_cells_follicular_helper	0.009680361
TCGA-29-1694-01A	T_cells_regulatory_(Tregs)	0.049338506
TCGA-29-1694-01A	T_cells_gamma_delta	0
TCGA-29-1694-01A	NK_cells_resting	0.021073966
TCGA-29-1694-01A	NK_cells_activated	0.038555246
TCGA-29-1694-01A	Monocytes	0.064654019
TCGA-29-1694-01A	Macrophages_M0	0.11532499
TCGA-29-1694-01A	Macrophages_M1	0.055655821
TCGA-29-1694-01A	Macrophages_M2	0.088468418
TCGA-29-1694-01A	Dendritic_cells_resting	0.032675669
TCGA-29-1694-01A	Dendritic_cells_activated	0.096515974
TCGA-29-1694-01A	Mast_cells_resting	0.03448176
TCGA-29-1694-01A	Mast_cells_activated	0
TCGA-29-1694-01A	Eosinophils	0

TCGA-29-1694-01A	Neutrophils	0.002230365
TCGA-29-1696-01A	B_cells_naive	0.049080299
TCGA-29-1696-01A	B_cells_memory	0
TCGA-29-1696-01A	Plasma_cells	0.130612021
TCGA-29-1696-01A	T_cells_CD8	0.052370907
TCGA-29-1696-01A	T_cells_CD4_naive	0
TCGA-29-1696-01A	T_cells_CD4_memory_resting	0.264172121
TCGA-29-1696-01A	T_cells_CD4_memory_activated	0
TCGA-29-1696-01A	T_cells_follicular_helper	0.082689873
TCGA-29-1696-01A	T_cells_regulatory_(Tregs)	0.005835711
TCGA-29-1696-01A	T_cells_gamma_delta	0
TCGA-29-1696-01A	NK_cells_resting	0
TCGA-29-1696-01A	NK_cells_activated	0.052181729
TCGA-29-1696-01A	Monocytes	0.037035839
TCGA-29-1696-01A	Macrophages_M0	0.026955936
TCGA-29-1696-01A	Macrophages_M1	0.10625276
TCGA-29-1696-01A	Macrophages_M2	0.153146383
TCGA-29-1696-01A	Dendritic_cells_resting	0
TCGA-29-1696-01A	Dendritic_cells_activated	0.033679609
TCGA-29-1696-01A	Mast_cells_resting	0.005986812
TCGA-29-1696-01A	Mast_cells_activated	0
TCGA-29-1696-01A	Eosinophils	0
TCGA-29-1696-01A	Neutrophils	0
TCGA-29-1703-01A	B_cells_naive	0.002562717
TCGA-29-1703-01A	B_cells_memory	0
TCGA-29-1703-01A	Plasma_cells	0
TCGA-29-1703-01A	T_cells_CD8	0.050949774
TCGA-29-1703-01A	T_cells_CD4_naive	0
TCGA-29-1703-01A	T_cells_CD4_memory_resting	0.248833319
TCGA-29-1703-01A	T_cells_CD4_memory_activated	0
TCGA-29-1703-01A	T_cells_follicular_helper	0
TCGA-29-1703-01A	T_cells_regulatory_(Tregs)	0.084492883
TCGA-29-1703-01A	T_cells_gamma_delta	0

TCGA-29-1703-01A	NK_cells_resting	0
TCGA-29-1703-01A	NK_cells_activated	0.087067246
TCGA-29-1703-01A	Monocytes	0.007033075
TCGA-29-1703-01A	Macrophages_M0	0.190510492
TCGA-29-1703-01A	Macrophages_M1	0.103778833
TCGA-29-1703-01A	Macrophages_M2	0.177057103
TCGA-29-1703-01A	Dendritic_cells_resting	0.044287344
TCGA-29-1703-01A	Dendritic_cells_activated	0
TCGA-29-1703-01A	Mast_cells_resting	0
TCGA-29-1703-01A	Mast_cells_activated	0
TCGA-29-1703-01A	Eosinophils	0
TCGA-29-1703-01A	Neutrophils	0.003427214
TCGA-29-1705-01A	B_cells_naive	0.040154545
TCGA-29-1705-01A	B_cells_memory	0
TCGA-29-1705-01A	Plasma_cells	0
TCGA-29-1705-01A	T_cells_CD8	0.040633707
TCGA-29-1705-01A	T_cells_CD4_naive	0
TCGA-29-1705-01A	T_cells_CD4_memory_resting	0.194777736
TCGA-29-1705-01A	T_cells_CD4_memory_activated	0
TCGA-29-1705-01A	T_cells_follicular_helper	0.012113799
TCGA-29-1705-01A	T_cells_regulatory_(Tregs)	0.040858562
TCGA-29-1705-01A	T_cells_gamma_delta	0
TCGA-29-1705-01A	NK_cells_resting	0
TCGA-29-1705-01A	NK_cells_activated	0.007860317
TCGA-29-1705-01A	Monocytes	0
TCGA-29-1705-01A	Macrophages_M0	0.358696561
TCGA-29-1705-01A	Macrophages_M1	0.050127451
TCGA-29-1705-01A	Macrophages_M2	0.232403371
TCGA-29-1705-01A	Dendritic_cells_resting	0
TCGA-29-1705-01A	Dendritic_cells_activated	0
TCGA-29-1705-01A	Mast_cells_resting	0
TCGA-29-1705-01A	Mast_cells_activated	0.011955977
TCGA-29-1705-01A	Eosinophils	0

TCGA-29-1705-01A	Neutrophils	0.010417975
TCGA-29-1710-01A	B_cells_naive	0.033470244
TCGA-29-1710-01A	B_cells_memory	0
TCGA-29-1710-01A	Plasma_cells	0.064994165
TCGA-29-1710-01A	T_cells_CD8	0.061146358
TCGA-29-1710-01A	T_cells_CD4_naive	0
TCGA-29-1710-01A	T_cells_CD4_memory_resting	0.193666193
TCGA-29-1710-01A	T_cells_CD4_memory_activated	0
TCGA-29-1710-01A	T_cells_follicular_helper	0.010049937
TCGA-29-1710-01A	T_cells_regulatory_(Tregs)	0.118289726
TCGA-29-1710-01A	T_cells_gamma_delta	0
TCGA-29-1710-01A	NK_cells_resting	0
TCGA-29-1710-01A	NK_cells_activated	0.097998134
TCGA-29-1710-01A	Monocytes	0.023708981
TCGA-29-1710-01A	Macrophages_M0	0.076371579
TCGA-29-1710-01A	Macrophages_M1	0.055114382
TCGA-29-1710-01A	Macrophages_M2	0.18357735
TCGA-29-1710-01A	Dendritic_cells_resting	0.055750578
TCGA-29-1710-01A	Dendritic_cells_activated	0
TCGA-29-1710-01A	Mast_cells_resting	0.024235902
TCGA-29-1710-01A	Mast_cells_activated	0
TCGA-29-1710-01A	Eosinophils	0
TCGA-29-1710-01A	Neutrophils	0.001626471
TCGA-29-1768-01A	B_cells_naive	0.062103585
TCGA-29-1768-01A	B_cells_memory	0
TCGA-29-1768-01A	Plasma_cells	0.070228339
TCGA-29-1768-01A	T_cells_CD8	0.120866332
TCGA-29-1768-01A	T_cells_CD4_naive	0
TCGA-29-1768-01A	T_cells_CD4_memory_resting	0.19609409
TCGA-29-1768-01A	T_cells_CD4_memory_activated	0
TCGA-29-1768-01A	T_cells_follicular_helper	0.011251373
TCGA-29-1768-01A	T_cells_regulatory_(Tregs)	0.023072195
TCGA-29-1768-01A	T_cells_gamma_delta	0

TCGA-29-1768-01A	NK_cells_resting	0
TCGA-29-1768-01A	NK_cells_activated	0.056269957
TCGA-29-1768-01A	Monocytes	0.013924183
TCGA-29-1768-01A	Macrophages_M0	0.069552239
TCGA-29-1768-01A	Macrophages_M1	0.119076636
TCGA-29-1768-01A	Macrophages_M2	0.170578599
TCGA-29-1768-01A	Dendritic_cells_resting	0.025473471
TCGA-29-1768-01A	Dendritic_cells_activated	0
TCGA-29-1768-01A	Mast_cells_resting	0.057264336
TCGA-29-1768-01A	Mast_cells_activated	0
TCGA-29-1768-01A	Eosinophils	0
TCGA-29-1768-01A	Neutrophils	0.004244663
TCGA-29-1776-01A	B_cells_naive	0.074044327
TCGA-29-1776-01A	B_cells_memory	0
TCGA-29-1776-01A	Plasma_cells	0.072514858
TCGA-29-1776-01A	T_cells_CD8	0.090968708
TCGA-29-1776-01A	T_cells_CD4_naive	0
TCGA-29-1776-01A	T_cells_CD4_memory_resting	0.25512511
TCGA-29-1776-01A	T_cells_CD4_memory_activated	0
TCGA-29-1776-01A	T_cells_follicular_helper	0.042975806
TCGA-29-1776-01A	T_cells_regulatory_(Tregs)	0.035606568
TCGA-29-1776-01A	T_cells_gamma_delta	0
TCGA-29-1776-01A	NK_cells_resting	0
TCGA-29-1776-01A	NK_cells_activated	0.034565363
TCGA-29-1776-01A	Monocytes	0.092889697
TCGA-29-1776-01A	Macrophages_M0	0.031222772
TCGA-29-1776-01A	Macrophages_M1	0.031621676
TCGA-29-1776-01A	Macrophages_M2	0.142694457
TCGA-29-1776-01A	Dendritic_cells_resting	0
TCGA-29-1776-01A	Dendritic_cells_activated	0.081484451
TCGA-29-1776-01A	Mast_cells_resting	0
TCGA-29-1776-01A	Mast_cells_activated	0.014286208
TCGA-29-1776-01A	Eosinophils	0

TCGA-29-1776-01A	Neutrophils	0
TCGA-29-1778-01A	B_cells_naive	0.054518225
TCGA-29-1778-01A	B_cells_memory	0
TCGA-29-1778-01A	Plasma_cells	0.113070156
TCGA-29-1778-01A	T_cells_CD8	0.070862931
TCGA-29-1778-01A	T_cells_CD4_naive	0
TCGA-29-1778-01A	T_cells_CD4_memory_resting	0.218940198
TCGA-29-1778-01A	T_cells_CD4_memory_activated	0
TCGA-29-1778-01A	T_cells_follicular_helper	0.016060454
TCGA-29-1778-01A	T_cells_regulatory_(Tregs)	0.07677968
TCGA-29-1778-01A	T_cells_gamma_delta	0
TCGA-29-1778-01A	NK_cells_resting	0
TCGA-29-1778-01A	NK_cells_activated	0.061193831
TCGA-29-1778-01A	Monocytes	0
TCGA-29-1778-01A	Macrophages_M0	0.084371876
TCGA-29-1778-01A	Macrophages_M1	0.049282628
TCGA-29-1778-01A	Macrophages_M2	0.19839986
TCGA-29-1778-01A	Dendritic_cells_resting	0
TCGA-29-1778-01A	Dendritic_cells_activated	0.035594797
TCGA-29-1778-01A	Mast_cells_resting	0.020925365
TCGA-29-1778-01A	Mast_cells_activated	0
TCGA-29-1778-01A	Eosinophils	0
TCGA-29-1778-01A	Neutrophils	0
TCGA-29-1781-01A	B_cells_naive	0
TCGA-29-1781-01A	B_cells_memory	0.002334809
TCGA-29-1781-01A	Plasma_cells	0.226242589
TCGA-29-1781-01A	T_cells_CD8	0.190382769
TCGA-29-1781-01A	T_cells_CD4_naive	0
TCGA-29-1781-01A	T_cells_CD4_memory_resting	0.000819575
TCGA-29-1781-01A	T_cells_CD4_memory_activated	0.04767822
TCGA-29-1781-01A	T_cells_follicular_helper	0.014380858
TCGA-29-1781-01A	T_cells_regulatory_(Tregs)	0.009751439
TCGA-29-1781-01A	T_cells_gamma_delta	0.014343256

TCGA-29-1781-01A	NK_cells_resting	0
TCGA-29-1781-01A	NK_cells_activated	0.007222706
TCGA-29-1781-01A	Monocytes	0
TCGA-29-1781-01A	Macrophages_M0	0.191326814
TCGA-29-1781-01A	Macrophages_M1	0.072500903
TCGA-29-1781-01A	Macrophages_M2	0.223016063
TCGA-29-1781-01A	Dendritic_cells_resting	0
TCGA-29-1781-01A	Dendritic_cells_activated	0
TCGA-29-1781-01A	Mast_cells_resting	0
TCGA-29-1781-01A	Mast_cells_activated	0
TCGA-29-1781-01A	Eosinophils	0
TCGA-29-1781-01A	Neutrophils	0
TCGA-29-1783-01A	B_cells_naive	0
TCGA-29-1783-01A	B_cells_memory	0.001416711
TCGA-29-1783-01A	Plasma_cells	0.062398855
TCGA-29-1783-01A	T_cells_CD8	0.035666637
TCGA-29-1783-01A	T_cells_CD4_naive	0
TCGA-29-1783-01A	T_cells_CD4_memory_resting	0.20798441
TCGA-29-1783-01A	T_cells_CD4_memory_activated	0
TCGA-29-1783-01A	T_cells_follicular_helper	0.017271584
TCGA-29-1783-01A	T_cells_regulatory_(Tregs)	0.054461028
TCGA-29-1783-01A	T_cells_gamma_delta	0
TCGA-29-1783-01A	NK_cells_resting	0
TCGA-29-1783-01A	NK_cells_activated	0.043977438
TCGA-29-1783-01A	Monocytes	0.012135652
TCGA-29-1783-01A	Macrophages_M0	0.177193132
TCGA-29-1783-01A	Macrophages_M1	0.096196502
TCGA-29-1783-01A	Macrophages_M2	0.261167599
TCGA-29-1783-01A	Dendritic_cells_resting	0.013784347
TCGA-29-1783-01A	Dendritic_cells_activated	0
TCGA-29-1783-01A	Mast_cells_resting	0
TCGA-29-1783-01A	Mast_cells_activated	0
TCGA-29-1783-01A	Eosinophils	0

TCGA-29-1783-01A	Neutrophils	0.016346104
TCGA-29-2414-01A	B_cells_naive	0.117763398
TCGA-29-2414-01A	B_cells_memory	0
TCGA-29-2414-01A	Plasma_cells	0.029210549
TCGA-29-2414-01A	T_cells_CD8	0.073553772
TCGA-29-2414-01A	T_cells_CD4_naive	0
TCGA-29-2414-01A	T_cells_CD4_memory_resting	0.18063257
TCGA-29-2414-01A	T_cells_CD4_memory_activated	0
TCGA-29-2414-01A	T_cells_follicular_helper	0.044409885
TCGA-29-2414-01A	T_cells_regulatory_(Tregs)	0.00667694
TCGA-29-2414-01A	T_cells_gamma_delta	0
TCGA-29-2414-01A	NK_cells_resting	0
TCGA-29-2414-01A	NK_cells_activated	0.025759847
TCGA-29-2414-01A	Monocytes	0.072481174
TCGA-29-2414-01A	Macrophages_M0	0.279566271
TCGA-29-2414-01A	Macrophages_M1	0.031944742
TCGA-29-2414-01A	Macrophages_M2	0.130720863
TCGA-29-2414-01A	Dendritic_cells_resting	0
TCGA-29-2414-01A	Dendritic_cells_activated	0.007279987
TCGA-29-2414-01A	Mast_cells_resting	0
TCGA-29-2414-01A	Mast_cells_activated	0
TCGA-29-2414-01A	Eosinophils	0
TCGA-29-2414-01A	Neutrophils	0
TCGA-29-2425-01A	B_cells_naive	0.022734376
TCGA-29-2425-01A	B_cells_memory	0
TCGA-29-2425-01A	Plasma_cells	0.108994652
TCGA-29-2425-01A	T_cells_CD8	0.047447562
TCGA-29-2425-01A	T_cells_CD4_naive	0
TCGA-29-2425-01A	T_cells_CD4_memory_resting	0.079242074
TCGA-29-2425-01A	T_cells_CD4_memory_activated	0
TCGA-29-2425-01A	T_cells_follicular_helper	0.041662749
TCGA-29-2425-01A	T_cells_regulatory_(Tregs)	0.067943685
TCGA-29-2425-01A	T_cells_gamma_delta	0

TCGA-29-2425-01A	NK_cells_resting	0.026662107
TCGA-29-2425-01A	NK_cells_activated	0.0334745
TCGA-29-2425-01A	Monocytes	0
TCGA-29-2425-01A	Macrophages_M0	0.343497626
TCGA-29-2425-01A	Macrophages_M1	0.059180104
TCGA-29-2425-01A	Macrophages_M2	0.16674849
TCGA-29-2425-01A	Dendritic_cells_resting	0
TCGA-29-2425-01A	Dendritic_cells_activated	0
TCGA-29-2425-01A	Mast_cells_resting	0.002412076
TCGA-29-2425-01A	Mast_cells_activated	0
TCGA-29-2425-01A	Eosinophils	0
TCGA-29-2425-01A	Neutrophils	0
TCGA-29-A5NZ-01A	B_cells_naive	0
TCGA-29-A5NZ-01A	B_cells_memory	0
TCGA-29-A5NZ-01A	Plasma_cells	0.021341259
TCGA-29-A5NZ-01A	T_cells_CD8	0.03228339
TCGA-29-A5NZ-01A	T_cells_CD4_naive	0
TCGA-29-A5NZ-01A	T_cells_CD4_memory_resting	0.313793718
TCGA-29-A5NZ-01A	T_cells_CD4_memory_activated	0
TCGA-29-A5NZ-01A	T_cells_follicular_helper	0
TCGA-29-A5NZ-01A	T_cells_regulatory_(Tregs)	0.025831361
TCGA-29-A5NZ-01A	T_cells_gamma_delta	0

TCGA-29-A5NZ-01A	NK_cells_resting	0.032713088
TCGA-29-A5NZ-01A	NK_cells_activated	0.000412287
TCGA-29-A5NZ-01A	Monocytes	0.005084681
TCGA-29-A5NZ-01A	Macrophages_M0	0.342194598
TCGA-29-A5NZ-01A	Macrophages_M1	0.061707034
TCGA-29-A5NZ-01A	Macrophages_M2	0.092504217
TCGA-29-A5NZ-01A	Dendritic_cells_resting	0.026074302
TCGA-29-A5NZ-01A	Dendritic_cells_activated	0.040280678
TCGA-29-A5NZ-01A	Mast_cells_resting	0
TCGA-29-A5NZ-01A	Mast_cells_activated	0.005779386
TCGA-29-A5NZ-01A	Eosinophils	0
TCGA-29-A5NZ-01A	Neutrophils	0
TCGA-30-1853-01A	B_cells_naive	0.017530773
TCGA-30-1853-01A	B_cells_memory	0
TCGA-30-1853-01A	Plasma_cells	0.090448604
TCGA-30-1853-01A	T_cells_CD8	0
TCGA-30-1853-01A	T_cells_CD4_naive	0
TCGA-30-1853-01A	T_cells_CD4_memory_resting	0.217778644
TCGA-30-1853-01A	T_cells_CD4_memory_activated	0
TCGA-30-1853-01A	T_cells_follicular_helper	0.029203238
TCGA-30-1853-01A	T_cells_regulatory_(Tregs)	0.096658903

TCGA-30-1853-01A	T_cells_gamma_delta	0
TCGA-30-1853-01A	NK_cells_resting	0.065758095
TCGA-30-1853-01A	NK_cells_activated	0
TCGA-30-1853-01A	Monocytes	0
TCGA-30-1853-01A	Macrophages_M0	0.29990232
TCGA-30-1853-01A	Macrophages_M1	0
TCGA-30-1853-01A	Macrophages_M2	0.047918655
TCGA-30-1853-01A	Dendritic_cells_resting	0
TCGA-30-1853-01A	Dendritic_cells_activated	0
TCGA-30-1853-01A	Mast_cells_resting	0.134800767
TCGA-30-1853-01A	Mast_cells_activated	0
TCGA-30-1853-01A	Eosinophils	0
TCGA-30-1853-01A	Neutrophils	0
TCGA-30-1857-01A	B_cells_naive	0
TCGA-30-1857-01A	B_cells_memory	0.01945899
TCGA-30-1857-01A	Plasma_cells	0
TCGA-30-1857-01A	T_cells_CD8	0.078368572
TCGA-30-1857-01A	T_cells_CD4_naive	0
TCGA-30-1857-01A	T_cells_CD4_memory_resting	0.181694414
TCGA-30-1857-01A	T_cells_CD4_memory_activated	0
TCGA-30-1857-01A	T_cells_follicular_helper	0
TCGA-30-1857-01A	T_cells_regulatory_(Tregs)	0.078827832
TCGA-30-1857-01A	T_cells_gamma_delta	0
TCGA-30-1857-01A	NK_cells_resting	0
TCGA-30-1857-01A	NK_cells_activated	0.035713073
TCGA-30-1857-01A	Monocytes	0.00868889
TCGA-30-1857-01A	Macrophages_M0	0.318665941
TCGA-30-1857-01A	Macrophages_M1	0.090110294
TCGA-30-1857-01A	Macrophages_M2	0.138317701
TCGA-30-1857-01A	Dendritic_cells_resting	0.018978276
TCGA-30-1857-01A	Dendritic_cells_activated	0
TCGA-30-1857-01A	Mast_cells_resting	0.031176018
TCGA-30-1857-01A	Mast_cells_activated	0

TCGA-30-1857-01A	Eosinophils	0
TCGA-30-1857-01A	Neutrophils	0
TCGA-30-1861-01A	B_cells_naive	0.014566162
TCGA-30-1861-01A	B_cells_memory	0
TCGA-30-1861-01A	Plasma_cells	0
TCGA-30-1861-01A	T_cells_CD8	0.043373349
TCGA-30-1861-01A	T_cells_CD4_naive	0
TCGA-30-1861-01A	T_cells_CD4_memory_resting	0.327266224
TCGA-30-1861-01A	T_cells_CD4_memory_activated	0
TCGA-30-1861-01A	T_cells_follicular_helper	0
TCGA-30-1861-01A	T_cells_regulatory_(Tregs)	0
TCGA-30-1861-01A	T_cells_gamma_delta	0
TCGA-30-1861-01A	NK_cells_resting	0.017573177
TCGA-30-1861-01A	NK_cells_activated	0.007040946
TCGA-30-1861-01A	Monocytes	0
TCGA-30-1861-01A	Macrophages_M0	0.276560683
TCGA-30-1861-01A	Macrophages_M1	0.091358707
TCGA-30-1861-01A	Macrophages_M2	0.186683283
TCGA-30-1861-01A	Dendritic_cells_resting	0
TCGA-30-1861-01A	Dendritic_cells_activated	0
TCGA-30-1861-01A	Mast_cells_resting	0
TCGA-30-1861-01A	Mast_cells_activated	0.035577468
TCGA-30-1861-01A	Eosinophils	0
TCGA-30-1861-01A	Neutrophils	0
TCGA-30-1862-01A	B_cells_naive	0.018380669
TCGA-30-1862-01A	B_cells_memory	0
TCGA-30-1862-01A	Plasma_cells	0.249199236
TCGA-30-1862-01A	T_cells_CD8	0.112023025
TCGA-30-1862-01A	T_cells_CD4_naive	0
TCGA-30-1862-01A	T_cells_CD4_memory_resting	0.21674985
TCGA-30-1862-01A	T_cells_CD4_memory_activated	0
TCGA-30-1862-01A	T_cells_follicular_helper	0.029180776
TCGA-30-1862-01A	T_cells_regulatory_(Tregs)	0.040195257

TCGA-30-1862-01A	T_cells_gamma_delta	0
TCGA-30-1862-01A	NK_cells_resting	0
TCGA-30-1862-01A	NK_cells_activated	0.029033802
TCGA-30-1862-01A	Monocytes	0.083652976
TCGA-30-1862-01A	Macrophages_M0	0.000977364
TCGA-30-1862-01A	Macrophages_M1	0.073573496
TCGA-30-1862-01A	Macrophages_M2	0.101628765
TCGA-30-1862-01A	Dendritic_cells_resting	0
TCGA-30-1862-01A	Dendritic_cells_activated	0
TCGA-30-1862-01A	Mast_cells_resting	0
TCGA-30-1862-01A	Mast_cells_activated	0.045404785
TCGA-30-1862-01A	Eosinophils	0
TCGA-30-1862-01A	Neutrophils	0
TCGA-30-1866-01A	B_cells_naive	0
TCGA-30-1866-01A	B_cells_memory	0.026235258
TCGA-30-1866-01A	Plasma_cells	0.001541279
TCGA-30-1866-01A	T_cells_CD8	0.384875751
TCGA-30-1866-01A	T_cells_CD4_naive	0
TCGA-30-1866-01A	T_cells_CD4_memory_resting	0
TCGA-30-1866-01A	T_cells_CD4_memory_activated	0
TCGA-30-1866-01A	T_cells_follicular_helper	0.03763766
TCGA-30-1866-01A	T_cells_regulatory_(Tregs)	0.096974837
TCGA-30-1866-01A	T_cells_gamma_delta	0
TCGA-30-1866-01A	NK_cells_resting	0
TCGA-30-1866-01A	NK_cells_activated	0.068386927
TCGA-30-1866-01A	Monocytes	0.034162357
TCGA-30-1866-01A	Macrophages_M0	0.110917015
TCGA-30-1866-01A	Macrophages_M1	0.090961673
TCGA-30-1866-01A	Macrophages_M2	0.141089591
TCGA-30-1866-01A	Dendritic_cells_resting	0
TCGA-30-1866-01A	Dendritic_cells_activated	0
TCGA-30-1866-01A	Mast_cells_resting	0
TCGA-30-1866-01A	Mast_cells_activated	0.007217652

TCGA-30-1866-01A	Eosinophils	0
TCGA-30-1866-01A	Neutrophils	0
TCGA-30-1891-01A	B_cells_naive	0.038712792
TCGA-30-1891-01A	B_cells_memory	0
TCGA-30-1891-01A	Plasma_cells	0.06693207
TCGA-30-1891-01A	T_cells_CD8	0.133134568
TCGA-30-1891-01A	T_cells_CD4_naive	0
TCGA-30-1891-01A	T_cells_CD4_memory_resting	0.198630018
TCGA-30-1891-01A	T_cells_CD4_memory_activated	0.000687062
TCGA-30-1891-01A	T_cells_follicular_helper	0
TCGA-30-1891-01A	T_cells_regulatory_(Tregs)	0.038325901
TCGA-30-1891-01A	T_cells_gamma_delta	0
TCGA-30-1891-01A	NK_cells_resting	0
TCGA-30-1891-01A	NK_cells_activated	0.019656525
TCGA-30-1891-01A	Monocytes	0.023942264
TCGA-30-1891-01A	Macrophages_M0	0.206596362
TCGA-30-1891-01A	Macrophages_M1	0.074394021
TCGA-30-1891-01A	Macrophages_M2	0.148075845
TCGA-30-1891-01A	Dendritic_cells_resting	0
TCGA-30-1891-01A	Dendritic_cells_activated	0
TCGA-30-1891-01A	Mast_cells_resting	0.020502969
TCGA-30-1891-01A	Mast_cells_activated	0.007685518
TCGA-30-1891-01A	Eosinophils	0
TCGA-30-1891-01A	Neutrophils	0.022724084
TCGA-30-1892-01A	B_cells_naive	0.065854706
TCGA-30-1892-01A	B_cells_memory	0
TCGA-30-1892-01A	Plasma_cells	0.003909016
TCGA-30-1892-01A	T_cells_CD8	0.074586282
TCGA-30-1892-01A	T_cells_CD4_naive	0
TCGA-30-1892-01A	T_cells_CD4_memory_resting	0.213709578
TCGA-30-1892-01A	T_cells_CD4_memory_activated	0
TCGA-30-1892-01A	T_cells_follicular_helper	0.02357696
TCGA-30-1892-01A	T_cells_regulatory_(Tregs)	0.019636641

TCGA-30-1892-01A	T_cells_gamma_delta	0
TCGA-30-1892-01A	NK_cells_resting	0.019030495
TCGA-30-1892-01A	NK_cells_activated	0
TCGA-30-1892-01A	Monocytes	0.001331365
TCGA-30-1892-01A	Macrophages_M0	0.336461957
TCGA-30-1892-01A	Macrophages_M1	0.050785504
TCGA-30-1892-01A	Macrophages_M2	0.165619366
TCGA-30-1892-01A	Dendritic_cells_resting	0
TCGA-30-1892-01A	Dendritic_cells_activated	0
TCGA-30-1892-01A	Mast_cells_resting	0
TCGA-30-1892-01A	Mast_cells_activated	0.014800561
TCGA-30-1892-01A	Eosinophils	0
TCGA-30-1892-01A	Neutrophils	0.010697569
TCGA-31-1946-01A	B_cells_naive	0.017421402
TCGA-31-1946-01A	B_cells_memory	0
TCGA-31-1946-01A	Plasma_cells	0.015539069
TCGA-31-1946-01A	T_cells_CD8	0.029218033
TCGA-31-1946-01A	T_cells_CD4_naive	0
TCGA-31-1946-01A	T_cells_CD4_memory_resting	0.120705237
TCGA-31-1946-01A	T_cells_CD4_memory_activated	0
TCGA-31-1946-01A	T_cells_follicular_helper	0.023463562
TCGA-31-1946-01A	T_cells_regulatory_(Tregs)	0.043851511
TCGA-31-1946-01A	T_cells_gamma_delta	0
TCGA-31-1946-01A	NK_cells_resting	0.003358154
TCGA-31-1946-01A	NK_cells_activated	0.001754999
TCGA-31-1946-01A	Monocytes	0
TCGA-31-1946-01A	Macrophages_M0	0.363797149
TCGA-31-1946-01A	Macrophages_M1	0.068361437
TCGA-31-1946-01A	Macrophages_M2	0.295110827
TCGA-31-1946-01A	Dendritic_cells_resting	0
TCGA-31-1946-01A	Dendritic_cells_activated	0
TCGA-31-1946-01A	Mast_cells_resting	0
TCGA-31-1946-01A	Mast_cells_activated	0.01741862

TCGA-31-1946-01A	Eosinophils	0
TCGA-31-1946-01A	Neutrophils	0
TCGA-36-1569-01A	B_cells_naive	0.031586765
TCGA-36-1569-01A	B_cells_memory	0
TCGA-36-1569-01A	Plasma_cells	0.034084853
TCGA-36-1569-01A	T_cells_CD8	0.167355523
TCGA-36-1569-01A	T_cells_CD4_naive	0
TCGA-36-1569-01A	T_cells_CD4_memory_resting	0.13861748
TCGA-36-1569-01A	T_cells_CD4_memory_activated	0
TCGA-36-1569-01A	T_cells_follicular_helper	0.036737554
TCGA-36-1569-01A	T_cells_regulatory_(Tregs)	0.064789989
TCGA-36-1569-01A	T_cells_gamma_delta	0
TCGA-36-1569-01A	NK_cells_resting	0
TCGA-36-1569-01A	NK_cells_activated	0.039356145
TCGA-36-1569-01A	Monocytes	0.030217762
TCGA-36-1569-01A	Macrophages_M0	0.118599736
TCGA-36-1569-01A	Macrophages_M1	0.097994286
TCGA-36-1569-01A	Macrophages_M2	0.132889509
TCGA-36-1569-01A	Dendritic_cells_resting	0.068234485
TCGA-36-1569-01A	Dendritic_cells_activated	0
TCGA-36-1569-01A	Mast_cells_resting	0.029980846
TCGA-36-1569-01A	Mast_cells_activated	0
TCGA-36-1569-01A	Eosinophils	0
TCGA-36-1569-01A	Neutrophils	0.009555065
TCGA-36-1570-01A	B_cells_naive	0
TCGA-36-1570-01A	B_cells_memory	0
TCGA-36-1570-01A	Plasma_cells	0.022312728
TCGA-36-1570-01A	T_cells_CD8	0.002341094
TCGA-36-1570-01A	T_cells_CD4_naive	0
TCGA-36-1570-01A	T_cells_CD4_memory_resting	0.114186749
TCGA-36-1570-01A	T_cells_CD4_memory_activated	0
TCGA-36-1570-01A	T_cells_follicular_helper	0.037695501
TCGA-36-1570-01A	T_cells_regulatory_(Tregs)	0.035599538

TCGA-36-1570-01A	T_cells_gamma_delta	0
TCGA-36-1570-01A	NK_cells_resting	0
TCGA-36-1570-01A	NK_cells_activated	0.092593888
TCGA-36-1570-01A	Monocytes	0.077877601
TCGA-36-1570-01A	Macrophages_M0	0.118115232
TCGA-36-1570-01A	Macrophages_M1	0.11318813
TCGA-36-1570-01A	Macrophages_M2	0.213530708
TCGA-36-1570-01A	Dendritic_cells_resting	0.007064146
TCGA-36-1570-01A	Dendritic_cells_activated	0.001453693
TCGA-36-1570-01A	Mast_cells_resting	0.129302332
TCGA-36-1570-01A	Mast_cells_activated	0
TCGA-36-1570-01A	Eosinophils	0.03473866
TCGA-36-1570-01A	Neutrophils	0
TCGA-36-1571-01A	B_cells_naive	0.071541667
TCGA-36-1571-01A	B_cells_memory	0
TCGA-36-1571-01A	Plasma_cells	0.018065571
TCGA-36-1571-01A	T_cells_CD8	0
TCGA-36-1571-01A	T_cells_CD4_naive	0
TCGA-36-1571-01A	T_cells_CD4_memory_resting	0.326314788
TCGA-36-1571-01A	T_cells_CD4_memory_activated	0
TCGA-36-1571-01A	T_cells_follicular_helper	0.0762248
TCGA-36-1571-01A	T_cells_regulatory_(Tregs)	0
TCGA-36-1571-01A	T_cells_gamma_delta	0
TCGA-36-1571-01A	NK_cells_resting	0
TCGA-36-1571-01A	NK_cells_activated	0.087383491
TCGA-36-1571-01A	Monocytes	0.059360689
TCGA-36-1571-01A	Macrophages_M0	0.057669826
TCGA-36-1571-01A	Macrophages_M1	0.05270288
TCGA-36-1571-01A	Macrophages_M2	0.146633465
TCGA-36-1571-01A	Dendritic_cells_resting	0
TCGA-36-1571-01A	Dendritic_cells_activated	0.055587773
TCGA-36-1571-01A	Mast_cells_resting	0.048515049
TCGA-36-1571-01A	Mast_cells_activated	0

TCGA-36-1571-01A	Eosinophils	0
TCGA-36-1571-01A	Neutrophils	0
TCGA-36-1574-01A	B_cells_naive	0.03284307
TCGA-36-1574-01A	B_cells_memory	0
TCGA-36-1574-01A	Plasma_cells	0.002767323
TCGA-36-1574-01A	T_cells_CD8	0.011225662
TCGA-36-1574-01A	T_cells_CD4_naive	0
TCGA-36-1574-01A	T_cells_CD4_memory_resting	0.216227077
TCGA-36-1574-01A	T_cells_CD4_memory_activated	0
TCGA-36-1574-01A	T_cells_follicular_helper	0.017348618
TCGA-36-1574-01A	T_cells_regulatory_(Tregs)	0.035208399
TCGA-36-1574-01A	T_cells_gamma_delta	0
TCGA-36-1574-01A	NK_cells_resting	0
TCGA-36-1574-01A	NK_cells_activated	0.035518822
TCGA-36-1574-01A	Monocytes	0
TCGA-36-1574-01A	Macrophages_M0	0.285583934
TCGA-36-1574-01A	Macrophages_M1	0.142015666
TCGA-36-1574-01A	Macrophages_M2	0.124547058
TCGA-36-1574-01A	Dendritic_cells_resting	0
TCGA-36-1574-01A	Dendritic_cells_activated	0.089238404
TCGA-36-1574-01A	Mast_cells_resting	0
TCGA-36-1574-01A	Mast_cells_activated	0.007475967
TCGA-36-1574-01A	Eosinophils	0
TCGA-36-1574-01A	Neutrophils	0
TCGA-36-1576-01A	B_cells_naive	0.032606359
TCGA-36-1576-01A	B_cells_memory	0
TCGA-36-1576-01A	Plasma_cells	0.053958899
TCGA-36-1576-01A	T_cells_CD8	0.14285345
TCGA-36-1576-01A	T_cells_CD4_naive	0
TCGA-36-1576-01A	T_cells_CD4_memory_resting	0.184538284
TCGA-36-1576-01A	T_cells_CD4_memory_activated	0
TCGA-36-1576-01A	T_cells_follicular_helper	0.00324332
TCGA-36-1576-01A	T_cells_regulatory_(Tregs)	0.087421693

TCGA-36-1576-01A	T_cells_gamma_delta	0
TCGA-36-1576-01A	NK_cells_resting	0
TCGA-36-1576-01A	NK_cells_activated	0.067805941
TCGA-36-1576-01A	Monocytes	0.053913232
TCGA-36-1576-01A	Macrophages_M0	0.106293999
TCGA-36-1576-01A	Macrophages_M1	0.041621896
TCGA-36-1576-01A	Macrophages_M2	0.203095684
TCGA-36-1576-01A	Dendritic_cells_resting	0.002796923
TCGA-36-1576-01A	Dendritic_cells_activated	0
TCGA-36-1576-01A	Mast_cells_resting	0.014347721
TCGA-36-1576-01A	Mast_cells_activated	0
TCGA-36-1576-01A	Eosinophils	0.001093126
TCGA-36-1576-01A	Neutrophils	0.004409473
TCGA-36-1577-01A	B_cells_naive	0.020005247
TCGA-36-1577-01A	B_cells_memory	0
TCGA-36-1577-01A	Plasma_cells	0
TCGA-36-1577-01A	T_cells_CD8	0.045212975
TCGA-36-1577-01A	T_cells_CD4_naive	0
TCGA-36-1577-01A	T_cells_CD4_memory_resting	0.156772207
TCGA-36-1577-01A	T_cells_CD4_memory_activated	0
TCGA-36-1577-01A	T_cells_follicular_helper	0
TCGA-36-1577-01A	T_cells_regulatory_(Tregs)	0.086402683
TCGA-36-1577-01A	T_cells_gamma_delta	0
TCGA-36-1577-01A	NK_cells_resting	0
TCGA-36-1577-01A	NK_cells_activated	0.071090651
TCGA-36-1577-01A	Monocytes	0.03244512
TCGA-36-1577-01A	Macrophages_M0	0.423089222
TCGA-36-1577-01A	Macrophages_M1	0
TCGA-36-1577-01A	Macrophages_M2	0.132414414
TCGA-36-1577-01A	Dendritic_cells_resting	0
TCGA-36-1577-01A	Dendritic_cells_activated	0.002584703
TCGA-36-1577-01A	Mast_cells_resting	0
TCGA-36-1577-01A	Mast_cells_activated	0

TCGA-36-1577-01A	Eosinophils	0
TCGA-36-1577-01A	Neutrophils	0.029982778
TCGA-36-1580-01A	B_cells_naive	0.013435312
TCGA-36-1580-01A	B_cells_memory	0
TCGA-36-1580-01A	Plasma_cells	0
TCGA-36-1580-01A	T_cells_CD8	0.075971252
TCGA-36-1580-01A	T_cells_CD4_naive	0
TCGA-36-1580-01A	T_cells_CD4_memory_resting	0.215883574
TCGA-36-1580-01A	T_cells_CD4_memory_activated	0
TCGA-36-1580-01A	T_cells_follicular_helper	0.0088059
TCGA-36-1580-01A	T_cells_regulatory_(Tregs)	0.06270766
TCGA-36-1580-01A	T_cells_gamma_delta	0
TCGA-36-1580-01A	NK_cells_resting	0
TCGA-36-1580-01A	NK_cells_activated	0.036938616
TCGA-36-1580-01A	Monocytes	0.006547671
TCGA-36-1580-01A	Macrophages_M0	0.298932504
TCGA-36-1580-01A	Macrophages_M1	0.072863839
TCGA-36-1580-01A	Macrophages_M2	0.17131992
TCGA-36-1580-01A	Dendritic_cells_resting	0.035357822
TCGA-36-1580-01A	Dendritic_cells_activated	0
TCGA-36-1580-01A	Mast_cells_resting	0
TCGA-36-1580-01A	Mast_cells_activated	0
TCGA-36-1580-01A	Eosinophils	0
TCGA-36-1580-01A	Neutrophils	0.00123593
TCGA-57-1582-01A	B_cells_naive	0.075908706
TCGA-57-1582-01A	B_cells_memory	0
TCGA-57-1582-01A	Plasma_cells	0.111176047
TCGA-57-1582-01A	T_cells_CD8	0.078341392
TCGA-57-1582-01A	T_cells_CD4_naive	0
TCGA-57-1582-01A	T_cells_CD4_memory_resting	0.178887918
TCGA-57-1582-01A	T_cells_CD4_memory_activated	0.016212028
TCGA-57-1582-01A	T_cells_follicular_helper	0.032421497
TCGA-57-1582-01A	T_cells_regulatory_(Tregs)	0.047093063

TCGA-57-1582-01A	T_cells_gamma_delta	0
TCGA-57-1582-01A	NK_cells_resting	0
TCGA-57-1582-01A	NK_cells_activated	0.006886226
TCGA-57-1582-01A	Monocytes	0.020810708
TCGA-57-1582-01A	Macrophages_M0	0.140652884
TCGA-57-1582-01A	Macrophages_M1	0.067929987
TCGA-57-1582-01A	Macrophages_M2	0.195094021
TCGA-57-1582-01A	Dendritic_cells_resting	0
TCGA-57-1582-01A	Dendritic_cells_activated	0
TCGA-57-1582-01A	Mast_cells_resting	0.024687916
TCGA-57-1582-01A	Mast_cells_activated	0
TCGA-57-1582-01A	Eosinophils	0.003897607
TCGA-57-1582-01A	Neutrophils	0
TCGA-57-1583-01A	B_cells_naive	0.031291371
TCGA-57-1583-01A	B_cells_memory	0
TCGA-57-1583-01A	Plasma_cells	0.007510575
TCGA-57-1583-01A	T_cells_CD8	0.008340988
TCGA-57-1583-01A	T_cells_CD4_naive	0
TCGA-57-1583-01A	T_cells_CD4_memory_resting	0.067963297
TCGA-57-1583-01A	T_cells_CD4_memory_activated	0
TCGA-57-1583-01A	T_cells_follicular_helper	0.011566148
TCGA-57-1583-01A	T_cells_regulatory_(Tregs)	0.014462444
TCGA-57-1583-01A	T_cells_gamma_delta	0
TCGA-57-1583-01A	NK_cells_resting	0.013334413
TCGA-57-1583-01A	NK_cells_activated	0.009127906
TCGA-57-1583-01A	Monocytes	0
TCGA-57-1583-01A	Macrophages_M0	0.70730453
TCGA-57-1583-01A	Macrophages_M1	0.041006099
TCGA-57-1583-01A	Macrophages_M2	0.030836832
TCGA-57-1583-01A	Dendritic_cells_resting	0
TCGA-57-1583-01A	Dendritic_cells_activated	0.047300541
TCGA-57-1583-01A	Mast_cells_resting	0.009954857
TCGA-57-1583-01A	Mast_cells_activated	0

TCGA-57-1583-01A	Eosinophils	0
TCGA-57-1583-01A	Neutrophils	0
TCGA-57-1585-01A	B_cells_naive	0.027440414
TCGA-57-1585-01A	B_cells_memory	0
TCGA-57-1585-01A	Plasma_cells	0.069338327
TCGA-57-1585-01A	T_cells_CD8	0.031761299
TCGA-57-1585-01A	T_cells_CD4_naive	0
TCGA-57-1585-01A	T_cells_CD4_memory_resting	0.204680035
TCGA-57-1585-01A	T_cells_CD4_memory_activated	0
TCGA-57-1585-01A	T_cells_follicular_helper	0.013426955
TCGA-57-1585-01A	T_cells_regulatory_(Tregs)	0.037028807
TCGA-57-1585-01A	T_cells_gamma_delta	0
TCGA-57-1585-01A	NK_cells_resting	0
TCGA-57-1585-01A	NK_cells_activated	0.012412987
TCGA-57-1585-01A	Monocytes	0.03779344
TCGA-57-1585-01A	Macrophages_M0	0.311091984
TCGA-57-1585-01A	Macrophages_M1	0.044801888
TCGA-57-1585-01A	Macrophages_M2	0.108953133
TCGA-57-1585-01A	Dendritic_cells_resting	0.053203979
TCGA-57-1585-01A	Dendritic_cells_activated	0
TCGA-57-1585-01A	Mast_cells_resting	0
TCGA-57-1585-01A	Mast_cells_activated	0.043190673
TCGA-57-1585-01A	Eosinophils	0
TCGA-57-1585-01A	Neutrophils	0.00487608
TCGA-57-1993-01A	B_cells_naive	0.045747384
TCGA-57-1993-01A	B_cells_memory	0
TCGA-57-1993-01A	Plasma_cells	0.076337005
TCGA-57-1993-01A	T_cells_CD8	0.008628774
TCGA-57-1993-01A	T_cells_CD4_naive	0
TCGA-57-1993-01A	T_cells_CD4_memory_resting	0.118492085
TCGA-57-1993-01A	T_cells_CD4_memory_activated	0
TCGA-57-1993-01A	T_cells_follicular_helper	0
TCGA-57-1993-01A	T_cells_regulatory_(Tregs)	0.02893196

TCGA-57-1993-01A	T_cells_gamma_delta	0
TCGA-57-1993-01A	NK_cells_resting	0
TCGA-57-1993-01A	NK_cells_activated	0.025315022
TCGA-57-1993-01A	Monocytes	0
TCGA-57-1993-01A	Macrophages_M0	0.311750768
TCGA-57-1993-01A	Macrophages_M1	0.028952712
TCGA-57-1993-01A	Macrophages_M2	0.321251277
TCGA-57-1993-01A	Dendritic_cells_resting	0
TCGA-57-1993-01A	Dendritic_cells_activated	0.007540571
TCGA-57-1993-01A	Mast_cells_resting	0.027052442
TCGA-57-1993-01A	Mast_cells_activated	0
TCGA-57-1993-01A	Eosinophils	0
TCGA-57-1993-01A	Neutrophils	0
TCGA-59-2352-01A	B_cells_naive	0.002751083
TCGA-59-2352-01A	B_cells_memory	0
TCGA-59-2352-01A	Plasma_cells	0.232337875
TCGA-59-2352-01A	T_cells_CD8	0.063751292
TCGA-59-2352-01A	T_cells_CD4_naive	0
TCGA-59-2352-01A	T_cells_CD4_memory_resting	0.065834303
TCGA-59-2352-01A	T_cells_CD4_memory_activated	0
TCGA-59-2352-01A	T_cells_follicular_helper	0.03292776
TCGA-59-2352-01A	T_cells_regulatory_(Tregs)	0.049374537
TCGA-59-2352-01A	T_cells_gamma_delta	0
TCGA-59-2352-01A	NK_cells_resting	0
TCGA-59-2352-01A	NK_cells_activated	0.084442543
TCGA-59-2352-01A	Monocytes	0
TCGA-59-2352-01A	Macrophages_M0	0.239335092
TCGA-59-2352-01A	Macrophages_M1	0.087532156
TCGA-59-2352-01A	Macrophages_M2	0.075087161
TCGA-59-2352-01A	Dendritic_cells_resting	0
TCGA-59-2352-01A	Dendritic_cells_activated	0.038818162
TCGA-59-2352-01A	Mast_cells_resting	0.027808037
TCGA-59-2352-01A	Mast_cells_activated	0

TCGA-59-2352-01A	Eosinophils	0
TCGA-59-2352-01A	Neutrophils	0
TCGA-59-2354-01A	B_cells_naive	0.071059535
TCGA-59-2354-01A	B_cells_memory	0
TCGA-59-2354-01A	Plasma_cells	0.025658695
TCGA-59-2354-01A	T_cells_CD8	0.062584271
TCGA-59-2354-01A	T_cells_CD4_naive	0
TCGA-59-2354-01A	T_cells_CD4_memory_resting	0.213999212
TCGA-59-2354-01A	T_cells_CD4_memory_activated	0.008185667
TCGA-59-2354-01A	T_cells_follicular_helper	0.047674394
TCGA-59-2354-01A	T_cells_regulatory_(Tregs)	0.096911907
TCGA-59-2354-01A	T_cells_gamma_delta	0
TCGA-59-2354-01A	NK_cells_resting	0.012395533
TCGA-59-2354-01A	NK_cells_activated	0.010934813
TCGA-59-2354-01A	Monocytes	0.002061439
TCGA-59-2354-01A	Macrophages_M0	0.160558276
TCGA-59-2354-01A	Macrophages_M1	0.095817799
TCGA-59-2354-01A	Macrophages_M2	0.168003382
TCGA-59-2354-01A	Dendritic_cells_resting	0.013355395
TCGA-59-2354-01A	Dendritic_cells_activated	0
TCGA-59-2354-01A	Mast_cells_resting	0.010799681
TCGA-59-2354-01A	Mast_cells_activated	0
TCGA-59-2354-01A	Eosinophils	0
TCGA-59-2354-01A	Neutrophils	0
TCGA-59-2355-01A	B_cells_naive	0.041010466
TCGA-59-2355-01A	B_cells_memory	0
TCGA-59-2355-01A	Plasma_cells	0
TCGA-59-2355-01A	T_cells_CD8	0.171528187
TCGA-59-2355-01A	T_cells_CD4_naive	0
TCGA-59-2355-01A	T_cells_CD4_memory_resting	0.201722333
TCGA-59-2355-01A	T_cells_CD4_memory_activated	0
TCGA-59-2355-01A	T_cells_follicular_helper	0.040580802
TCGA-59-2355-01A	T_cells_regulatory_(Tregs)	0.07509131

TCGA-59-2355-01A	T_cells_gamma_delta	0
TCGA-59-2355-01A	NK_cells_resting	0
TCGA-59-2355-01A	NK_cells_activated	0.056354211
TCGA-59-2355-01A	Monocytes	0.015085481
TCGA-59-2355-01A	Macrophages_M0	0.086592572
TCGA-59-2355-01A	Macrophages_M1	0.155376306
TCGA-59-2355-01A	Macrophages_M2	0.109406922
TCGA-59-2355-01A	Dendritic_cells_resting	0
TCGA-59-2355-01A	Dendritic_cells_activated	0.031178811
TCGA-59-2355-01A	Mast_cells_resting	0.016072599
TCGA-59-2355-01A	Mast_cells_activated	0
TCGA-59-2355-01A	Eosinophils	0
TCGA-59-2355-01A	Neutrophils	0
TCGA-59-2363-01A	B_cells_naive	0.04469985
TCGA-59-2363-01A	B_cells_memory	0
TCGA-59-2363-01A	Plasma_cells	0
TCGA-59-2363-01A	T_cells_CD8	0.080097435
TCGA-59-2363-01A	T_cells_CD4_naive	0
TCGA-59-2363-01A	T_cells_CD4_memory_resting	0.163001964
TCGA-59-2363-01A	T_cells_CD4_memory_activated	0
TCGA-59-2363-01A	T_cells_follicular_helper	0.1047355
TCGA-59-2363-01A	T_cells_regulatory_(Tregs)	0.04364025
TCGA-59-2363-01A	T_cells_gamma_delta	0
TCGA-59-2363-01A	NK_cells_resting	0
TCGA-59-2363-01A	NK_cells_activated	0.064455112
TCGA-59-2363-01A	Monocytes	0.104210819
TCGA-59-2363-01A	Macrophages_M0	0.107157455
TCGA-59-2363-01A	Macrophages_M1	0.053552206
TCGA-59-2363-01A	Macrophages_M2	0.073152732
TCGA-59-2363-01A	Dendritic_cells_resting	0
TCGA-59-2363-01A	Dendritic_cells_activated	0.119654081
TCGA-59-2363-01A	Mast_cells_resting	0
TCGA-59-2363-01A	Mast_cells_activated	0.001760907

TCGA-59-2363-01A	Eosinophils	0
TCGA-59-2363-01A	Neutrophils	0.039881688
TCGA-5X-AA5U-01A	B_cells_naive	0.000202128
TCGA-5X-AA5U-01A	B_cells_memory	0.002948291
TCGA-5X-AA5U-01A	Plasma_cells	0.003178328
TCGA-5X-AA5U-01A	T_cells_CD8	0
TCGA-5X-AA5U-01A	T_cells_CD4_naive	0
TCGA-5X-AA5U-01A	T_cells_CD4_memory_resting	0.22794982
TCGA-5X-AA5U-01A	T_cells_CD4_memory_activated	0
TCGA-5X-AA5U-01A	T_cells_follicular_helper	0.036914537
TCGA-5X-AA5U-01A	T_cells_regulatory_(Tregs)	0.029157457
TCGA-5X-AA5U-01A	T_cells_gamma_delta	0
TCGA-5X-AA5U-01A	NK_cells_resting	0.062801787
TCGA-5X-AA5U-01A	NK_cells_activated	0
TCGA-5X-AA5U-01A	Monocytes	0
TCGA-5X-AA5U-01A	Macrophages_M0	0.625372365
TCGA-5X-AA5U-01A	Macrophages_M1	0.005088787

TCGA-5X-AA5U-01A	Macrophages_M2	0
TCGA-5X-AA5U-01A	Dendritic_cells_resting	0
TCGA-5X-AA5U-01A	Dendritic_cells_activated	0
TCGA-5X-AA5U-01A	Mast_cells_resting	0
TCGA-5X-AA5U-01A	Mast_cells_activated	0.006386499
TCGA-5X-AA5U-01A	Eosinophils	0
TCGA-5X-AA5U-01A	Neutrophils	0
TCGA-61-1721-01A	B_cells_naive	0.017318383
TCGA-61-1721-01A	B_cells_memory	0
TCGA-61-1721-01A	Plasma_cells	0.004183765
TCGA-61-1721-01A	T_cells_CD8	0.057958355
TCGA-61-1721-01A	T_cells_CD4_naive	0
TCGA-61-1721-01A	T_cells_CD4_memory_resting	0.192244902
TCGA-61-1721-01A	T_cells_CD4_memory_activated	0
TCGA-61-1721-01A	T_cells_follicular_helper	0
TCGA-61-1721-01A	T_cells_regulatory_(Tregs)	0.00785831
TCGA-61-1721-01A	T_cells_gamma_delta	0.009586957
TCGA-61-1721-01A	NK_cells_resting	0
TCGA-61-1721-01A	NK_cells_activated	0.011234216
TCGA-61-1721-01A	Monocytes	0.008794473
TCGA-61-1721-01A	Macrophages_M0	0.008866418
TCGA-61-1721-01A	Macrophages_M1	0.011085663
TCGA-61-1721-01A	Macrophages_M2	0.552806384
TCGA-61-1721-01A	Dendritic_cells_resting	0.003547647
TCGA-61-1721-01A	Dendritic_cells_activated	0
TCGA-61-1721-01A	Mast_cells_resting	0.111972332

TCGA-61-1721-01A	Mast_cells_activated	0
TCGA-61-1721-01A	Eosinophils	0
TCGA-61-1721-01A	Neutrophils	0.002542195
TCGA-61-1724-01A	B_cells_naive	0.052402302
TCGA-61-1724-01A	B_cells_memory	0
TCGA-61-1724-01A	Plasma_cells	0.105751192
TCGA-61-1724-01A	T_cells_CD8	0.050967244
TCGA-61-1724-01A	T_cells_CD4_naive	0
TCGA-61-1724-01A	T_cells_CD4_memory_resting	0.202025177
TCGA-61-1724-01A	T_cells_CD4_memory_activated	0
TCGA-61-1724-01A	T_cells_follicular_helper	0.00741923
TCGA-61-1724-01A	T_cells_regulatory_(Tregs)	0.058232042
TCGA-61-1724-01A	T_cells_gamma_delta	0
TCGA-61-1724-01A	NK_cells_resting	0.016598332
TCGA-61-1724-01A	NK_cells_activated	0.016576179
TCGA-61-1724-01A	Monocytes	0.008642584
TCGA-61-1724-01A	Macrophages_M0	0.230589187
TCGA-61-1724-01A	Macrophages_M1	0.084209755
TCGA-61-1724-01A	Macrophages_M2	0.146575158
TCGA-61-1724-01A	Dendritic_cells_resting	0
TCGA-61-1724-01A	Dendritic_cells_activated	0
TCGA-61-1724-01A	Mast_cells_resting	0.020011618
TCGA-61-1724-01A	Mast_cells_activated	0
TCGA-61-1724-01A	Eosinophils	0
TCGA-61-1724-01A	Neutrophils	0
TCGA-61-1728-01A	B_cells_naive	0.031083663
TCGA-61-1728-01A	B_cells_memory	0
TCGA-61-1728-01A	Plasma_cells	0.016892197
TCGA-61-1728-01A	T_cells_CD8	0.017864366
TCGA-61-1728-01A	T_cells_CD4_naive	0
TCGA-61-1728-01A	T_cells_CD4_memory_resting	0.167330036
TCGA-61-1728-01A	T_cells_CD4_memory_activated	0
TCGA-61-1728-01A	T_cells_follicular_helper	0.002161958

TCGA-61-1728-01A	T_cells_regulatory_(Tregs)	0.054763045
TCGA-61-1728-01A	T_cells_gamma_delta	0
TCGA-61-1728-01A	NK_cells_resting	0
TCGA-61-1728-01A	NK_cells_activated	0.058665426
TCGA-61-1728-01A	Monocytes	0.007817268
TCGA-61-1728-01A	Macrophages_M0	0.39986158
TCGA-61-1728-01A	Macrophages_M1	0.046732504
TCGA-61-1728-01A	Macrophages_M2	0.154254673
TCGA-61-1728-01A	Dendritic_cells_resting	0.003155925
TCGA-61-1728-01A	Dendritic_cells_activated	0
TCGA-61-1728-01A	Mast_cells_resting	0
TCGA-61-1728-01A	Mast_cells_activated	0.025861017
TCGA-61-1728-01A	Eosinophils	0
TCGA-61-1728-01A	Neutrophils	0.01355634
TCGA-61-1737-01A	B_cells_naive	0.013331403
TCGA-61-1737-01A	B_cells_memory	0
TCGA-61-1737-01A	Plasma_cells	0
TCGA-61-1737-01A	T_cells_CD8	0.033747496
TCGA-61-1737-01A	T_cells_CD4_naive	0
TCGA-61-1737-01A	T_cells_CD4_memory_resting	0.195146737
TCGA-61-1737-01A	T_cells_CD4_memory_activated	0
TCGA-61-1737-01A	T_cells_follicular_helper	0.02632422
TCGA-61-1737-01A	T_cells_regulatory_(Tregs)	0.043956856
TCGA-61-1737-01A	T_cells_gamma_delta	0
TCGA-61-1737-01A	NK_cells_resting	0
TCGA-61-1737-01A	NK_cells_activated	0.031378528
TCGA-61-1737-01A	Monocytes	0
TCGA-61-1737-01A	Macrophages_M0	0.257612207
TCGA-61-1737-01A	Macrophages_M1	0.078142988
TCGA-61-1737-01A	Macrophages_M2	0.230767569
TCGA-61-1737-01A	Dendritic_cells_resting	0.043246835
TCGA-61-1737-01A	Dendritic_cells_activated	0.001820611
TCGA-61-1737-01A	Mast_cells_resting	0

TCGA-61-1737-01A	Mast_cells_activated	0.044524551
TCGA-61-1737-01A	Eosinophils	0
TCGA-61-1737-01A	Neutrophils	0
TCGA-61-1738-01A	B_cells_naive	0.023567119
TCGA-61-1738-01A	B_cells_memory	0
TCGA-61-1738-01A	Plasma_cells	0.102894773
TCGA-61-1738-01A	T_cells_CD8	0.053325905
TCGA-61-1738-01A	T_cells_CD4_naive	0
TCGA-61-1738-01A	T_cells_CD4_memory_resting	0.040717355
TCGA-61-1738-01A	T_cells_CD4_memory_activated	0
TCGA-61-1738-01A	T_cells_follicular_helper	0.047170283
TCGA-61-1738-01A	T_cells_regulatory_(Tregs)	0.010664052
TCGA-61-1738-01A	T_cells_gamma_delta	0
TCGA-61-1738-01A	NK_cells_resting	0
TCGA-61-1738-01A	NK_cells_activated	0.056331105
TCGA-61-1738-01A	Monocytes	0.102742336
TCGA-61-1738-01A	Macrophages_M0	0.029562977
TCGA-61-1738-01A	Macrophages_M1	0.124939399
TCGA-61-1738-01A	Macrophages_M2	0.170952771
TCGA-61-1738-01A	Dendritic_cells_resting	0
TCGA-61-1738-01A	Dendritic_cells_activated	0.213500444
TCGA-61-1738-01A	Mast_cells_resting	0
TCGA-61-1738-01A	Mast_cells_activated	0
TCGA-61-1738-01A	Eosinophils	0
TCGA-61-1738-01A	Neutrophils	0.023631481
TCGA-61-1741-01A	B_cells_naive	0.046099608
TCGA-61-1741-01A	B_cells_memory	0
TCGA-61-1741-01A	Plasma_cells	0.165159146
TCGA-61-1741-01A	T_cells_CD8	0.045372921
TCGA-61-1741-01A	T_cells_CD4_naive	0
TCGA-61-1741-01A	T_cells_CD4_memory_resting	0.156929305
TCGA-61-1741-01A	T_cells_CD4_memory_activated	1.606E-06
TCGA-61-1741-01A	T_cells_follicular_helper	0.053148727

TCGA-61-1741-01A	T_cells_regulatory_(Tregs)	0.060923214
TCGA-61-1741-01A	T_cells_gamma_delta	0
TCGA-61-1741-01A	NK_cells_resting	0
TCGA-61-1741-01A	NK_cells_activated	0.009378938
TCGA-61-1741-01A	Monocytes	0
TCGA-61-1741-01A	Macrophages_M0	0.253010136
TCGA-61-1741-01A	Macrophages_M1	0.076728384
TCGA-61-1741-01A	Macrophages_M2	0.131483769
TCGA-61-1741-01A	Dendritic_cells_resting	0
TCGA-61-1741-01A	Dendritic_cells_activated	0
TCGA-61-1741-01A	Mast_cells_resting	0.001764248
TCGA-61-1741-01A	Mast_cells_activated	0
TCGA-61-1741-01A	Eosinophils	0
TCGA-61-1741-01A	Neutrophils	0
TCGA-61-1900-01A	B_cells_naive	0.096354579
TCGA-61-1900-01A	B_cells_memory	0
TCGA-61-1900-01A	Plasma_cells	0.23869942
TCGA-61-1900-01A	T_cells_CD8	0.067673775
TCGA-61-1900-01A	T_cells_CD4_naive	0
TCGA-61-1900-01A	T_cells_CD4_memory_resting	0.100817068
TCGA-61-1900-01A	T_cells_CD4_memory_activated	0
TCGA-61-1900-01A	T_cells_follicular_helper	0.018050483
TCGA-61-1900-01A	T_cells_regulatory_(Tregs)	0.040459887
TCGA-61-1900-01A	T_cells_gamma_delta	0
TCGA-61-1900-01A	NK_cells_resting	0
TCGA-61-1900-01A	NK_cells_activated	0.10430996
TCGA-61-1900-01A	Monocytes	0.020059131
TCGA-61-1900-01A	Macrophages_M0	0.087098985
TCGA-61-1900-01A	Macrophages_M1	0.07620027
TCGA-61-1900-01A	Macrophages_M2	0.128477746
TCGA-61-1900-01A	Dendritic_cells_resting	0
TCGA-61-1900-01A	Dendritic_cells_activated	0
TCGA-61-1900-01A	Mast_cells_resting	0.021798696

TCGA-61-1900-01A	Mast_cells_activated	0
TCGA-61-1900-01A	Eosinophils	0
TCGA-61-1900-01A	Neutrophils	0
TCGA-61-1995-01A	B_cells_naive	0
TCGA-61-1995-01A	B_cells_memory	0.004725394
TCGA-61-1995-01A	Plasma_cells	0
TCGA-61-1995-01A	T_cells_CD8	0.058288688
TCGA-61-1995-01A	T_cells_CD4_naive	0
TCGA-61-1995-01A	T_cells_CD4_memory_resting	0.300373295
TCGA-61-1995-01A	T_cells_CD4_memory_activated	0
TCGA-61-1995-01A	T_cells_follicular_helper	0
TCGA-61-1995-01A	T_cells_regulatory_(Tregs)	0.102762629
TCGA-61-1995-01A	T_cells_gamma_delta	0
TCGA-61-1995-01A	NK_cells_resting	0
TCGA-61-1995-01A	NK_cells_activated	0.031087472
TCGA-61-1995-01A	Monocytes	0.030876243
TCGA-61-1995-01A	Macrophages_M0	0.128686762
TCGA-61-1995-01A	Macrophages_M1	0.062940095
TCGA-61-1995-01A	Macrophages_M2	0.254850412
TCGA-61-1995-01A	Dendritic_cells_resting	0.002772346
TCGA-61-1995-01A	Dendritic_cells_activated	0
TCGA-61-1995-01A	Mast_cells_resting	0.022636663
TCGA-61-1995-01A	Mast_cells_activated	0
TCGA-61-1995-01A	Eosinophils	0
TCGA-61-1995-01A	Neutrophils	0
TCGA-61-1998-01A	B_cells_naive	0.004290558
TCGA-61-1998-01A	B_cells_memory	0
TCGA-61-1998-01A	Plasma_cells	0.000306591
TCGA-61-1998-01A	T_cells_CD8	0.008133947
TCGA-61-1998-01A	T_cells_CD4_naive	0
TCGA-61-1998-01A	T_cells_CD4_memory_resting	0.087868854
TCGA-61-1998-01A	T_cells_CD4_memory_activated	0
TCGA-61-1998-01A	T_cells_follicular_helper	0.029370867

TCGA-61-1998-01A	T_cells_regulatory_(Tregs)	0.054756025
TCGA-61-1998-01A	T_cells_gamma_delta	0
TCGA-61-1998-01A	NK_cells_resting	0
TCGA-61-1998-01A	NK_cells_activated	0.058573841
TCGA-61-1998-01A	Monocytes	0
TCGA-61-1998-01A	Macrophages_M0	0.513634762
TCGA-61-1998-01A	Macrophages_M1	0.0169693
TCGA-61-1998-01A	Macrophages_M2	0.212826907
TCGA-61-1998-01A	Dendritic_cells_resting	0.008896352
TCGA-61-1998-01A	Dendritic_cells_activated	0
TCGA-61-1998-01A	Mast_cells_resting	0
TCGA-61-1998-01A	Mast_cells_activated	0
TCGA-61-1998-01A	Eosinophils	0
TCGA-61-1998-01A	Neutrophils	0.004371996
TCGA-61-2003-01A	B_cells_naive	0.002523085
TCGA-61-2003-01A	B_cells_memory	0.009301318
TCGA-61-2003-01A	Plasma_cells	0
TCGA-61-2003-01A	T_cells_CD8	0.054896168
TCGA-61-2003-01A	T_cells_CD4_naive	0
TCGA-61-2003-01A	T_cells_CD4_memory_resting	0.170800023
TCGA-61-2003-01A	T_cells_CD4_memory_activated	0
TCGA-61-2003-01A	T_cells_follicular_helper	0.014110991
TCGA-61-2003-01A	T_cells_regulatory_(Tregs)	0.047755331
TCGA-61-2003-01A	T_cells_gamma_delta	0
TCGA-61-2003-01A	NK_cells_resting	0
TCGA-61-2003-01A	NK_cells_activated	0.054614968
TCGA-61-2003-01A	Monocytes	0.016574624
TCGA-61-2003-01A	Macrophages_M0	0.343453325
TCGA-61-2003-01A	Macrophages_M1	0.046077318
TCGA-61-2003-01A	Macrophages_M2	0.158822859
TCGA-61-2003-01A	Dendritic_cells_resting	0.063455398
TCGA-61-2003-01A	Dendritic_cells_activated	0
TCGA-61-2003-01A	Mast_cells_resting	0.017614593

TCGA-61-2003-01A	Mast_cells_activated	0
TCGA-61-2003-01A	Eosinophils	0
TCGA-61-2003-01A	Neutrophils	0
TCGA-61-2008-01A	B_cells_naive	0.01484779
TCGA-61-2008-01A	B_cells_memory	0
TCGA-61-2008-01A	Plasma_cells	0.209243588
TCGA-61-2008-01A	T_cells_CD8	0.081859008
TCGA-61-2008-01A	T_cells_CD4_naive	0
TCGA-61-2008-01A	T_cells_CD4_memory_resting	0.093723283
TCGA-61-2008-01A	T_cells_CD4_memory_activated	0
TCGA-61-2008-01A	T_cells_follicular_helper	0.039994233
TCGA-61-2008-01A	T_cells_regulatory_(Tregs)	0.035085012
TCGA-61-2008-01A	T_cells_gamma_delta	0
TCGA-61-2008-01A	NK_cells_resting	0
TCGA-61-2008-01A	NK_cells_activated	0.056494723
TCGA-61-2008-01A	Monocytes	0.081513713
TCGA-61-2008-01A	Macrophages_M0	0.010853465
TCGA-61-2008-01A	Macrophages_M1	0.100241698
TCGA-61-2008-01A	Macrophages_M2	0.240474426
TCGA-61-2008-01A	Dendritic_cells_resting	0
TCGA-61-2008-01A	Dendritic_cells_activated	0
TCGA-61-2008-01A	Mast_cells_resting	0
TCGA-61-2008-01A	Mast_cells_activated	0.035669061
TCGA-61-2008-01A	Eosinophils	0
TCGA-61-2008-01A	Neutrophils	0
TCGA-61-2009-01A	B_cells_naive	0.003755406
TCGA-61-2009-01A	B_cells_memory	0
TCGA-61-2009-01A	Plasma_cells	0.007304481
TCGA-61-2009-01A	T_cells_CD8	0
TCGA-61-2009-01A	T_cells_CD4_naive	0
TCGA-61-2009-01A	T_cells_CD4_memory_resting	0.189328299
TCGA-61-2009-01A	T_cells_CD4_memory_activated	0
TCGA-61-2009-01A	T_cells_follicular_helper	0.005644919

TCGA-61-2009-01A	T_cells_regulatory_(Tregs)	0.013489644
TCGA-61-2009-01A	T_cells_gamma_delta	0.082423259
TCGA-61-2009-01A	NK_cells_resting	0
TCGA-61-2009-01A	NK_cells_activated	0.01218257
TCGA-61-2009-01A	Monocytes	0
TCGA-61-2009-01A	Macrophages_M0	0.097508902
TCGA-61-2009-01A	Macrophages_M1	0.116259589
TCGA-61-2009-01A	Macrophages_M2	0.389637533
TCGA-61-2009-01A	Dendritic_cells_resting	0.066157946
TCGA-61-2009-01A	Dendritic_cells_activated	0
TCGA-61-2009-01A	Mast_cells_resting	0
TCGA-61-2009-01A	Mast_cells_activated	0.016307453
TCGA-61-2009-01A	Eosinophils	0
TCGA-61-2009-01A	Neutrophils	0
TCGA-61-2012-01A	B_cells_naive	0
TCGA-61-2012-01A	B_cells_memory	0
TCGA-61-2012-01A	Plasma_cells	0.171298041
TCGA-61-2012-01A	T_cells_CD8	0.017099375
TCGA-61-2012-01A	T_cells_CD4_naive	0
TCGA-61-2012-01A	T_cells_CD4_memory_resting	0.148505833
TCGA-61-2012-01A	T_cells_CD4_memory_activated	0
TCGA-61-2012-01A	T_cells_follicular_helper	0.0259626
TCGA-61-2012-01A	T_cells_regulatory_(Tregs)	0.074823728
TCGA-61-2012-01A	T_cells_gamma_delta	0.027007751
TCGA-61-2012-01A	NK_cells_resting	0
TCGA-61-2012-01A	NK_cells_activated	0.002356422
TCGA-61-2012-01A	Monocytes	0
TCGA-61-2012-01A	Macrophages_M0	0.277533155
TCGA-61-2012-01A	Macrophages_M1	0.114312254
TCGA-61-2012-01A	Macrophages_M2	0.063393701
TCGA-61-2012-01A	Dendritic_cells_resting	0
TCGA-61-2012-01A	Dendritic_cells_activated	0
TCGA-61-2012-01A	Mast_cells_resting	0

TCGA-61-2012-01A	Mast_cells_activated	0.061240513
TCGA-61-2012-01A	Eosinophils	0
TCGA-61-2012-01A	Neutrophils	0.016466627
TCGA-61-2088-01A	B_cells_naive	0
TCGA-61-2088-01A	B_cells_memory	0.011405031
TCGA-61-2088-01A	Plasma_cells	0.099632127
TCGA-61-2088-01A	T_cells_CD8	0
TCGA-61-2088-01A	T_cells_CD4_naive	0
TCGA-61-2088-01A	T_cells_CD4_memory_resting	0.266749281
TCGA-61-2088-01A	T_cells_CD4_memory_activated	0
TCGA-61-2088-01A	T_cells_follicular_helper	0.099488573
TCGA-61-2088-01A	T_cells_regulatory_(Tregs)	0
TCGA-61-2088-01A	T_cells_gamma_delta	0
TCGA-61-2088-01A	NK_cells_resting	0
TCGA-61-2088-01A	NK_cells_activated	0.14728197
TCGA-61-2088-01A	Monocytes	0.029363822
TCGA-61-2088-01A	Macrophages_M0	0.183221938
TCGA-61-2088-01A	Macrophages_M1	0
TCGA-61-2088-01A	Macrophages_M2	0.02869995
TCGA-61-2088-01A	Dendritic_cells_resting	0.00370789
TCGA-61-2088-01A	Dendritic_cells_activated	0
TCGA-61-2088-01A	Mast_cells_resting	0.125532322
TCGA-61-2088-01A	Mast_cells_activated	0
TCGA-61-2088-01A	Eosinophils	0
TCGA-61-2088-01A	Neutrophils	0.004917096
TCGA-61-2102-01A	B_cells_naive	0.00278741
TCGA-61-2102-01A	B_cells_memory	0
TCGA-61-2102-01A	Plasma_cells	0.005216062
TCGA-61-2102-01A	T_cells_CD8	0
TCGA-61-2102-01A	T_cells_CD4_naive	0
TCGA-61-2102-01A	T_cells_CD4_memory_resting	0.140286457
TCGA-61-2102-01A	T_cells_CD4_memory_activated	0
TCGA-61-2102-01A	T_cells_follicular_helper	0.002059924

TCGA-61-2102-01A	T_cells_regulatory_(Tregs)	0.051421597
TCGA-61-2102-01A	T_cells_gamma_delta	0
TCGA-61-2102-01A	NK_cells_resting	0.04093878
TCGA-61-2102-01A	NK_cells_activated	0.003755884
TCGA-61-2102-01A	Monocytes	0
TCGA-61-2102-01A	Macrophages_M0	0.462847307
TCGA-61-2102-01A	Macrophages_M1	0.006026543
TCGA-61-2102-01A	Macrophages_M2	0.236796491
TCGA-61-2102-01A	Dendritic_cells_resting	0
TCGA-61-2102-01A	Dendritic_cells_activated	0
TCGA-61-2102-01A	Mast_cells_resting	0.044104342
TCGA-61-2102-01A	Mast_cells_activated	0
TCGA-61-2102-01A	Eosinophils	0
TCGA-61-2102-01A	Neutrophils	0.003759204
TCGA-61-2113-01A	B_cells_naive	0.05987882
TCGA-61-2113-01A	B_cells_memory	0
TCGA-61-2113-01A	Plasma_cells	0.049129175
TCGA-61-2113-01A	T_cells_CD8	0.039262698
TCGA-61-2113-01A	T_cells_CD4_naive	0
TCGA-61-2113-01A	T_cells_CD4_memory_resting	0.271121273
TCGA-61-2113-01A	T_cells_CD4_memory_activated	0.006432744
TCGA-61-2113-01A	T_cells_follicular_helper	0.040890622
TCGA-61-2113-01A	T_cells_regulatory_(Tregs)	0.010072607
TCGA-61-2113-01A	T_cells_gamma_delta	0
TCGA-61-2113-01A	NK_cells_resting	0.037493693
TCGA-61-2113-01A	NK_cells_activated	0
TCGA-61-2113-01A	Monocytes	0.014852051
TCGA-61-2113-01A	Macrophages_M0	0.132529733
TCGA-61-2113-01A	Macrophages_M1	0.099491369
TCGA-61-2113-01A	Macrophages_M2	0.135767305
TCGA-61-2113-01A	Dendritic_cells_resting	0
TCGA-61-2113-01A	Dendritic_cells_activated	0.061042449
TCGA-61-2113-01A	Mast_cells_resting	0

TCGA-61-2113-01A	Mast_cells_activated	0.030262424
TCGA-61-2113-01A	Eosinophils	0
TCGA-61-2113-01A	Neutrophils	0.011773037
TCGA-OY-A56Q-01A	B_cells_naive	0.191666542
TCGA-OY-A56Q-01A	B_cells_memory	0
TCGA-OY-A56Q-01A	Plasma_cells	0.053743884
TCGA-OY-A56Q-01A	T_cells_CD8	0.018783076
TCGA-OY-A56Q-01A	T_cells_CD4_naive	0
TCGA-OY-A56Q-01A	T_cells_CD4_memory_resting	0.166198533
TCGA-OY-A56Q-01A	T_cells_CD4_memory_activated	0
TCGA-OY-A56Q-01A	T_cells_follicular_helper	0.026528437
TCGA-OY-A56Q-01A	T_cells_regulatory_(Tregs)	0.0559811
TCGA-OY-A56Q-01A	T_cells_gamma_delta	0
TCGA-OY-A56Q-01A	NK_cells_resting	0
TCGA-OY-A56Q-01A	NK_cells_activated	0.099316125
TCGA-OY-A56Q-01A	Monocytes	0.008504547
TCGA-OY-A56Q-01A	Macrophages_M0	0.034344239
TCGA-OY-A56Q-01A	Macrophages_M1	0.006745025

TCGA-OY-A56Q-01A	Macrophages_M2	0.261456648
TCGA-OY-A56Q-01A	Dendritic_cells_resting	0
TCGA-OY-A56Q-01A	Dendritic_cells_activated	0.000211327
TCGA-OY-A56Q-01A	Mast_cells_resting	0.07652052
TCGA-OY-A56Q-01A	Mast_cells_activated	0
TCGA-OY-A56Q-01A	Eosinophils	0
TCGA-OY-A56Q-01A	Neutrophils	0
TCGA-WR-A838-01A	B_cells_naive	0.02880762
TCGA-WR-A838-01A	B_cells_memory	0
TCGA-WR-A838-01A	Plasma_cells	0.090434684
TCGA-WR-A838-01A	T_cells_CD8	0
TCGA-WR-A838-01A	T_cells_CD4_naive	0
TCGA-WR-A838-01A	T_cells_CD4_memory_resting	0.334344581
TCGA-WR-A838-01A	T_cells_CD4_memory_activated	0
TCGA-WR-A838-01A	T_cells_follicular_helper	0.023042105
TCGA-WR-A838-01A	T_cells_regulatory_(Tregs)	0.086698204

TCGA-WR-A838-01A	T_cells_gamma_delta	0
TCGA-WR-A838-01A	NK_cells_resting	0
TCGA-WR-A838-01A	NK_cells_activated	0.087551475
TCGA-WR-A838-01A	Monocytes	0
TCGA-WR-A838-01A	Macrophages_M0	0.223164999
TCGA-WR-A838-01A	Macrophages_M1	0.011313906
TCGA-WR-A838-01A	Macrophages_M2	0
TCGA-WR-A838-01A	Dendritic_cells_resting	0
TCGA-WR-A838-01A	Dendritic_cells_activated	0
TCGA-WR-A838-01A	Mast_cells_resting	0.114642426
TCGA-WR-A838-01A	Mast_cells_activated	0
TCGA-WR-A838-01A	Eosinophils	0
TCGA-WR-A838-01A	Neutrophils	0

Table S14: The proportion of 22 immune infiltrating cells in the low expression group of OV samples was estimated using the CIBERSORT algorithm

LowSamples	Celltype	Fraction
TCGA-04-1338-01A	B_cells_naive	0.035332734
TCGA-04-1338-01A	B_cells_memory	0
TCGA-04-1338-01A	Plasma_cells	0.025245569
TCGA-04-1338-01A	T_cells_CD8	0.184326124
TCGA-04-1338-01A	T_cells_CD4_naive	0
TCGA-04-1338-01A	T_cells_CD4_memory_resting	0.041827732
TCGA-04-1338-01A	T_cells_CD4_memory_activated	0.148281714
TCGA-04-1338-01A	T_cells_follicular_helper	0.028728984
TCGA-04-1338-01A	T_cells_regulatory_(Tregs)	0
TCGA-04-1338-01A	T_cells_gamma_delta	0.021958216
TCGA-04-1338-01A	NK_cells_resting	0
TCGA-04-1338-01A	NK_cells_activated	0.05388621
TCGA-04-1338-01A	Monocytes	0.013223421
TCGA-04-1338-01A	Macrophages_M0	0.085423518
TCGA-04-1338-01A	Macrophages_M1	0.075829541
TCGA-04-1338-01A	Macrophages_M2	0.195808066
TCGA-04-1338-01A	Dendritic_cells_resting	0
TCGA-04-1338-01A	Dendritic_cells_activated	0

TCGA-04-1338-01A	Mast_cells_resting	0
TCGA-04-1338-01A	Mast_cells_activated	0.079874838
TCGA-04-1338-01A	Eosinophils	0.010253331
TCGA-04-1338-01A	Neutrophils	0
TCGA-04-1343-01A	B_cells_naive	0.031365023
TCGA-04-1343-01A	B_cells_memory	0
TCGA-04-1343-01A	Plasma_cells	0.246309342
TCGA-04-1343-01A	T_cells_CD8	0.105805202
TCGA-04-1343-01A	T_cells_CD4_naive	0
TCGA-04-1343-01A	T_cells_CD4_memory_resting	0.116644584
TCGA-04-1343-01A	T_cells_CD4_memory_activated	0
TCGA-04-1343-01A	T_cells_follicular_helper	0.014321952
TCGA-04-1343-01A	T_cells_regulatory_(Tregs)	0.060562836
TCGA-04-1343-01A	T_cells_gamma_delta	0
TCGA-04-1343-01A	NK_cells_resting	0
TCGA-04-1343-01A	NK_cells_activated	0.052927711
TCGA-04-1343-01A	Monocytes	0.030463718
TCGA-04-1343-01A	Macrophages_M0	0.028243181
TCGA-04-1343-01A	Macrophages_M1	0.103855721
TCGA-04-1343-01A	Macrophages_M2	0.120858752
TCGA-04-1343-01A	Dendritic_cells_resting	0
TCGA-04-1343-01A	Dendritic_cells_activated	0
TCGA-04-1343-01A	Mast_cells_resting	0
TCGA-04-1343-01A	Mast_cells_activated	0.088641977
TCGA-04-1343-01A	Eosinophils	0
TCGA-04-1343-01A	Neutrophils	0
TCGA-04-1347-01A	B_cells_naive	0.087455513
TCGA-04-1347-01A	B_cells_memory	0
TCGA-04-1347-01A	Plasma_cells	0.103757493
TCGA-04-1347-01A	T_cells_CD8	0.047376588
TCGA-04-1347-01A	T_cells_CD4_naive	0
TCGA-04-1347-01A	T_cells_CD4_memory_resting	0.017105415
TCGA-04-1347-01A	T_cells_CD4_memory_activated	0

TCGA-04-1347-01A	T_cells_follicular_helper	0.022061873
TCGA-04-1347-01A	T_cells_regulatory_(Tregs)	0.044606079
TCGA-04-1347-01A	T_cells_gamma_delta	0
TCGA-04-1347-01A	NK_cells_resting	0
TCGA-04-1347-01A	NK_cells_activated	0.048981051
TCGA-04-1347-01A	Monocytes	0
TCGA-04-1347-01A	Macrophages_M0	0.430508113
TCGA-04-1347-01A	Macrophages_M1	0.053708524
TCGA-04-1347-01A	Macrophages_M2	0.107946854
TCGA-04-1347-01A	Dendritic_cells_resting	0
TCGA-04-1347-01A	Dendritic_cells_activated	0
TCGA-04-1347-01A	Mast_cells_resting	0.036492497
TCGA-04-1347-01A	Mast_cells_activated	0
TCGA-04-1347-01A	Eosinophils	0
TCGA-04-1347-01A	Neutrophils	0
TCGA-04-1356-01A	B_cells_naive	0.036095163
TCGA-04-1356-01A	B_cells_memory	0
TCGA-04-1356-01A	Plasma_cells	0.002229556
TCGA-04-1356-01A	T_cells_CD8	0.043782806
TCGA-04-1356-01A	T_cells_CD4_naive	0
TCGA-04-1356-01A	T_cells_CD4_memory_resting	0.151946321
TCGA-04-1356-01A	T_cells_CD4_memory_activated	0
TCGA-04-1356-01A	T_cells_follicular_helper	0.028515604
TCGA-04-1356-01A	T_cells_regulatory_(Tregs)	0.033096606
TCGA-04-1356-01A	T_cells_gamma_delta	0.009979073
TCGA-04-1356-01A	NK_cells_resting	0
TCGA-04-1356-01A	NK_cells_activated	0.028504016
TCGA-04-1356-01A	Monocytes	0
TCGA-04-1356-01A	Macrophages_M0	0.499942684
TCGA-04-1356-01A	Macrophages_M1	0.033673752
TCGA-04-1356-01A	Macrophages_M2	0.039021024
TCGA-04-1356-01A	Dendritic_cells_resting	0
TCGA-04-1356-01A	Dendritic_cells_activated	0

TCGA-04-1356-01A	Mast_cells_resting	0
TCGA-04-1356-01A	Mast_cells_activated	0.093213396
TCGA-04-1356-01A	Eosinophils	0
TCGA-04-1356-01A	Neutrophils	0
TCGA-04-1361-01A	B_cells_naive	0.049012847
TCGA-04-1361-01A	B_cells_memory	0
TCGA-04-1361-01A	Plasma_cells	0.009074731
TCGA-04-1361-01A	T_cells_CD8	0.085620953
TCGA-04-1361-01A	T_cells_CD4_naive	0
TCGA-04-1361-01A	T_cells_CD4_memory_resting	0.325449053
TCGA-04-1361-01A	T_cells_CD4_memory_activated	0
TCGA-04-1361-01A	T_cells_follicular_helper	0.017423547
TCGA-04-1361-01A	T_cells_regulatory_(Tregs)	0.029803486
TCGA-04-1361-01A	T_cells_gamma_delta	0
TCGA-04-1361-01A	NK_cells_resting	0
TCGA-04-1361-01A	NK_cells_activated	0.048019737
TCGA-04-1361-01A	Monocytes	0.073911165
TCGA-04-1361-01A	Macrophages_M0	0
TCGA-04-1361-01A	Macrophages_M1	0.033773389
TCGA-04-1361-01A	Macrophages_M2	0.145087531
TCGA-04-1361-01A	Dendritic_cells_resting	0
TCGA-04-1361-01A	Dendritic_cells_activated	0.163731767
TCGA-04-1361-01A	Mast_cells_resting	0.019091792
TCGA-04-1361-01A	Mast_cells_activated	0
TCGA-04-1361-01A	Eosinophils	0
TCGA-04-1361-01A	Neutrophils	0
TCGA-04-1362-01A	B_cells_naive	0.023471901
TCGA-04-1362-01A	B_cells_memory	0
TCGA-04-1362-01A	Plasma_cells	0
TCGA-04-1362-01A	T_cells_CD8	0.033861194
TCGA-04-1362-01A	T_cells_CD4_naive	0
TCGA-04-1362-01A	T_cells_CD4_memory_resting	0.280462566
TCGA-04-1362-01A	T_cells_CD4_memory_activated	0

TCGA-04-1362-01A	T_cells_follicular_helper	0.015159511
TCGA-04-1362-01A	T_cells_regulatory_(Tregs)	0.041562563
TCGA-04-1362-01A	T_cells_gamma_delta	0
TCGA-04-1362-01A	NK_cells_resting	0
TCGA-04-1362-01A	NK_cells_activated	0.08314697
TCGA-04-1362-01A	Monocytes	0.144964959
TCGA-04-1362-01A	Macrophages_M0	0
TCGA-04-1362-01A	Macrophages_M1	0.015439756
TCGA-04-1362-01A	Macrophages_M2	0.145638955
TCGA-04-1362-01A	Dendritic_cells_resting	0
TCGA-04-1362-01A	Dendritic_cells_activated	0.180691911
TCGA-04-1362-01A	Mast_cells_resting	0
TCGA-04-1362-01A	Mast_cells_activated	0.035599715
TCGA-04-1362-01A	Eosinophils	0
TCGA-04-1362-01A	Neutrophils	0
TCGA-04-1365-01A	B_cells_naive	0
TCGA-04-1365-01A	B_cells_memory	0.029601144
TCGA-04-1365-01A	Plasma_cells	0.011826425
TCGA-04-1365-01A	T_cells_CD8	0.024072156
TCGA-04-1365-01A	T_cells_CD4_naive	0
TCGA-04-1365-01A	T_cells_CD4_memory_resting	0.235440094
TCGA-04-1365-01A	T_cells_CD4_memory_activated	0.005604534
TCGA-04-1365-01A	T_cells_follicular_helper	0.07086876
TCGA-04-1365-01A	T_cells_regulatory_(Tregs)	0.011444503
TCGA-04-1365-01A	T_cells_gamma_delta	0.020496513
TCGA-04-1365-01A	NK_cells_resting	0
TCGA-04-1365-01A	NK_cells_activated	0.054409282
TCGA-04-1365-01A	Monocytes	0.02222601
TCGA-04-1365-01A	Macrophages_M0	0.215024894
TCGA-04-1365-01A	Macrophages_M1	0.150665424
TCGA-04-1365-01A	Macrophages_M2	0.139248108
TCGA-04-1365-01A	Dendritic_cells_resting	0.007917469
TCGA-04-1365-01A	Dendritic_cells_activated	0

TCGA-04-1365-01A	Mast_cells_resting	0.001154685
TCGA-04-1365-01A	Mast_cells_activated	0
TCGA-04-1365-01A	Eosinophils	0
TCGA-04-1365-01A	Neutrophils	0
TCGA-04-1514-01A	B_cells_naive	0
TCGA-04-1514-01A	B_cells_memory	0
TCGA-04-1514-01A	Plasma_cells	0
TCGA-04-1514-01A	T_cells_CD8	0.032668969
TCGA-04-1514-01A	T_cells_CD4_naive	0
TCGA-04-1514-01A	T_cells_CD4_memory_resting	0.186296339
TCGA-04-1514-01A	T_cells_CD4_memory_activated	0
TCGA-04-1514-01A	T_cells_follicular_helper	0.112722566
TCGA-04-1514-01A	T_cells_regulatory_(Tregs)	0.116109078
TCGA-04-1514-01A	T_cells_gamma_delta	0
TCGA-04-1514-01A	NK_cells_resting	0
TCGA-04-1514-01A	NK_cells_activated	0.129717108
TCGA-04-1514-01A	Monocytes	0.070685401
TCGA-04-1514-01A	Macrophages_M0	0.142247962
TCGA-04-1514-01A	Macrophages_M1	0.094361259
TCGA-04-1514-01A	Macrophages_M2	0.085906192
TCGA-04-1514-01A	Dendritic_cells_resting	0
TCGA-04-1514-01A	Dendritic_cells_activated	0
TCGA-04-1514-01A	Mast_cells_resting	0
TCGA-04-1514-01A	Mast_cells_activated	0
TCGA-04-1514-01A	Eosinophils	0.029285124
TCGA-04-1514-01A	Neutrophils	0
TCGA-04-1519-01A	B_cells_naive	0
TCGA-04-1519-01A	B_cells_memory	0.046605452
TCGA-04-1519-01A	Plasma_cells	0.002263928
TCGA-04-1519-01A	T_cells_CD8	0
TCGA-04-1519-01A	T_cells_CD4_naive	0
TCGA-04-1519-01A	T_cells_CD4_memory_resting	0.192851535
TCGA-04-1519-01A	T_cells_CD4_memory_activated	0

TCGA-04-1519-01A	T_cells_follicular_helper	0.001979788
TCGA-04-1519-01A	T_cells_regulatory_(Tregs)	0.061769007
TCGA-04-1519-01A	T_cells_gamma_delta	0
TCGA-04-1519-01A	NK_cells_resting	0.012021616
TCGA-04-1519-01A	NK_cells_activated	0.059739738
TCGA-04-1519-01A	Monocytes	0.025961912
TCGA-04-1519-01A	Macrophages_M0	0.183021469
TCGA-04-1519-01A	Macrophages_M1	0.028813174
TCGA-04-1519-01A	Macrophages_M2	0.119963393
TCGA-04-1519-01A	Dendritic_cells_resting	0
TCGA-04-1519-01A	Dendritic_cells_activated	0
TCGA-04-1519-01A	Mast_cells_resting	0.26500899
TCGA-04-1519-01A	Mast_cells_activated	0
TCGA-04-1519-01A	Eosinophils	0
TCGA-04-1519-01A	Neutrophils	0
TCGA-04-1530-01A	B_cells_naive	0
TCGA-04-1530-01A	B_cells_memory	0.012755073
TCGA-04-1530-01A	Plasma_cells	0
TCGA-04-1530-01A	T_cells_CD8	0.073118871
TCGA-04-1530-01A	T_cells_CD4_naive	0
TCGA-04-1530-01A	T_cells_CD4_memory_resting	0.236941471
TCGA-04-1530-01A	T_cells_CD4_memory_activated	0
TCGA-04-1530-01A	T_cells_follicular_helper	0.039531527
TCGA-04-1530-01A	T_cells_regulatory_(Tregs)	0.001684246
TCGA-04-1530-01A	T_cells_gamma_delta	0
TCGA-04-1530-01A	NK_cells_resting	0
TCGA-04-1530-01A	NK_cells_activated	0.07594256
TCGA-04-1530-01A	Monocytes	0
TCGA-04-1530-01A	Macrophages_M0	0.224849961
TCGA-04-1530-01A	Macrophages_M1	0.137890031
TCGA-04-1530-01A	Macrophages_M2	0.178701056
TCGA-04-1530-01A	Dendritic_cells_resting	0.018567901
TCGA-04-1530-01A	Dendritic_cells_activated	0

TCGA-04-1530-01A	Mast_cells_resting	0
TCGA-04-1530-01A	Mast_cells_activated	0
TCGA-04-1530-01A	Eosinophils	0
TCGA-04-1530-01A	Neutrophils	1.73024E-05
TCGA-04-1542-01A	B_cells_naive	0
TCGA-04-1542-01A	B_cells_memory	0.082822627
TCGA-04-1542-01A	Plasma_cells	0.008701765
TCGA-04-1542-01A	T_cells_CD8	0.048122717
TCGA-04-1542-01A	T_cells_CD4_naive	0
TCGA-04-1542-01A	T_cells_CD4_memory_resting	0.169993177
TCGA-04-1542-01A	T_cells_CD4_memory_activated	0
TCGA-04-1542-01A	T_cells_follicular_helper	0
TCGA-04-1542-01A	T_cells_regulatory_(Tregs)	0.126632476
TCGA-04-1542-01A	T_cells_gamma_delta	0
TCGA-04-1542-01A	NK_cells_resting	0
TCGA-04-1542-01A	NK_cells_activated	0.059377624
TCGA-04-1542-01A	Monocytes	0.021680027
TCGA-04-1542-01A	Macrophages_M0	0.176386152
TCGA-04-1542-01A	Macrophages_M1	0
TCGA-04-1542-01A	Macrophages_M2	0.177218572
TCGA-04-1542-01A	Dendritic_cells_resting	0
TCGA-04-1542-01A	Dendritic_cells_activated	0.092716597
TCGA-04-1542-01A	Mast_cells_resting	0
TCGA-04-1542-01A	Mast_cells_activated	0.036348266
TCGA-04-1542-01A	Eosinophils	0
TCGA-04-1542-01A	Neutrophils	0
TCGA-04-1651-01A	B_cells_naive	0.110867894
TCGA-04-1651-01A	B_cells_memory	0
TCGA-04-1651-01A	Plasma_cells	0.037257683
TCGA-04-1651-01A	T_cells_CD8	0.004572506
TCGA-04-1651-01A	T_cells_CD4_naive	0
TCGA-04-1651-01A	T_cells_CD4_memory_resting	0.29879358
TCGA-04-1651-01A	T_cells_CD4_memory_activated	0

TCGA-04-1651-01A	T_cells_follicular_helper	0.06998617
TCGA-04-1651-01A	T_cells_regulatory_(Tregs)	0.005328674
TCGA-04-1651-01A	T_cells_gamma_delta	0
TCGA-04-1651-01A	NK_cells_resting	0
TCGA-04-1651-01A	NK_cells_activated	0.090948989
TCGA-04-1651-01A	Monocytes	0.063131858
TCGA-04-1651-01A	Macrophages_M0	0
TCGA-04-1651-01A	Macrophages_M1	0.059071196
TCGA-04-1651-01A	Macrophages_M2	0.057755148
TCGA-04-1651-01A	Dendritic_cells_resting	0
TCGA-04-1651-01A	Dendritic_cells_activated	0.160539197
TCGA-04-1651-01A	Mast_cells_resting	0.039536417
TCGA-04-1651-01A	Mast_cells_activated	0
TCGA-04-1651-01A	Eosinophils	0.002210688
TCGA-04-1651-01A	Neutrophils	0
TCGA-09-0366-01A	B_cells_naive	0.007174758
TCGA-09-0366-01A	B_cells_memory	0
TCGA-09-0366-01A	Plasma_cells	0
TCGA-09-0366-01A	T_cells_CD8	0.058143992
TCGA-09-0366-01A	T_cells_CD4_naive	0
TCGA-09-0366-01A	T_cells_CD4_memory_resting	0.073437624
TCGA-09-0366-01A	T_cells_CD4_memory_activated	0
TCGA-09-0366-01A	T_cells_follicular_helper	0.016065316
TCGA-09-0366-01A	T_cells_regulatory_(Tregs)	0.023020791
TCGA-09-0366-01A	T_cells_gamma_delta	0
TCGA-09-0366-01A	NK_cells_resting	0
TCGA-09-0366-01A	NK_cells_activated	0.047540528
TCGA-09-0366-01A	Monocytes	0.362813155
TCGA-09-0366-01A	Macrophages_M0	0
TCGA-09-0366-01A	Macrophages_M1	0.011367819
TCGA-09-0366-01A	Macrophages_M2	0.349118292
TCGA-09-0366-01A	Dendritic_cells_resting	0
TCGA-09-0366-01A	Dendritic_cells_activated	0.025667835

TCGA-09-0366-01A	Mast_cells_resting	0
TCGA-09-0366-01A	Mast_cells_activated	0.003801492
TCGA-09-0366-01A	Eosinophils	0.021848398
TCGA-09-0366-01A	Neutrophils	0
TCGA-09-1666-01A	B_cells_naive	0.02762291
TCGA-09-1666-01A	B_cells_memory	0
TCGA-09-1666-01A	Plasma_cells	0
TCGA-09-1666-01A	T_cells_CD8	0.108748524
TCGA-09-1666-01A	T_cells_CD4_naive	0
TCGA-09-1666-01A	T_cells_CD4_memory_resting	0.243991062
TCGA-09-1666-01A	T_cells_CD4_memory_activated	0.004028838
TCGA-09-1666-01A	T_cells_follicular_helper	0.027584898
TCGA-09-1666-01A	T_cells_regulatory_(Tregs)	0.033148005
TCGA-09-1666-01A	T_cells_gamma_delta	0
TCGA-09-1666-01A	NK_cells_resting	0.021552218
TCGA-09-1666-01A	NK_cells_activated	0
TCGA-09-1666-01A	Monocytes	0
TCGA-09-1666-01A	Macrophages_M0	0.176277661
TCGA-09-1666-01A	Macrophages_M1	0.18526187
TCGA-09-1666-01A	Macrophages_M2	0.160752677
TCGA-09-1666-01A	Dendritic_cells_resting	0.002199591
TCGA-09-1666-01A	Dendritic_cells_activated	0
TCGA-09-1666-01A	Mast_cells_resting	0
TCGA-09-1666-01A	Mast_cells_activated	0.006484656
TCGA-09-1666-01A	Eosinophils	0
TCGA-09-1666-01A	Neutrophils	0.002347091
TCGA-09-1669-01A	B_cells_naive	0.001853493
TCGA-09-1669-01A	B_cells_memory	0
TCGA-09-1669-01A	Plasma_cells	0.217374599
TCGA-09-1669-01A	T_cells_CD8	0.121148355
TCGA-09-1669-01A	T_cells_CD4_naive	0
TCGA-09-1669-01A	T_cells_CD4_memory_resting	0.154047621
TCGA-09-1669-01A	T_cells_CD4_memory_activated	0.003891463

TCGA-09-1669-01A	T_cells_follicular_helper	0.060298863
TCGA-09-1669-01A	T_cells_regulatory_(Tregs)	0.066193879
TCGA-09-1669-01A	T_cells_gamma_delta	0
TCGA-09-1669-01A	NK_cells_resting	0
TCGA-09-1669-01A	NK_cells_activated	0.038815507
TCGA-09-1669-01A	Monocytes	0.001835276
TCGA-09-1669-01A	Macrophages_M0	0.149043991
TCGA-09-1669-01A	Macrophages_M1	0.058572152
TCGA-09-1669-01A	Macrophages_M2	0.11844336
TCGA-09-1669-01A	Dendritic_cells_resting	0
TCGA-09-1669-01A	Dendritic_cells_activated	0
TCGA-09-1669-01A	Mast_cells_resting	0.008481441
TCGA-09-1669-01A	Mast_cells_activated	0
TCGA-09-1669-01A	Eosinophils	0
TCGA-09-1669-01A	Neutrophils	0
TCGA-09-2048-01A	B_cells_naive	0
TCGA-09-2048-01A	B_cells_memory	0
TCGA-09-2048-01A	Plasma_cells	0.054992413
TCGA-09-2048-01A	T_cells_CD8	0.093977532
TCGA-09-2048-01A	T_cells_CD4_naive	0
TCGA-09-2048-01A	T_cells_CD4_memory_resting	0.214919077
TCGA-09-2048-01A	T_cells_CD4_memory_activated	0
TCGA-09-2048-01A	T_cells_follicular_helper	0.056893928
TCGA-09-2048-01A	T_cells_regulatory_(Tregs)	0.05791789
TCGA-09-2048-01A	T_cells_gamma_delta	0
TCGA-09-2048-01A	NK_cells_resting	0
TCGA-09-2048-01A	NK_cells_activated	0
TCGA-09-2048-01A	Monocytes	0.04812847
TCGA-09-2048-01A	Macrophages_M0	0.074085989
TCGA-09-2048-01A	Macrophages_M1	0.104547034
TCGA-09-2048-01A	Macrophages_M2	0.168571658
TCGA-09-2048-01A	Dendritic_cells_resting	0.012629586
TCGA-09-2048-01A	Dendritic_cells_activated	0

TCGA-09-2048-01A	Mast_cells_resting	0
TCGA-09-2048-01A	Mast_cells_activated	0.084922149
TCGA-09-2048-01A	Eosinophils	0.015112201
TCGA-09-2048-01A	Neutrophils	0.013302072
TCGA-09-2051-01A	B_cells_naive	0.044893712
TCGA-09-2051-01A	B_cells_memory	0
TCGA-09-2051-01A	Plasma_cells	0.009423603
TCGA-09-2051-01A	T_cells_CD8	0.011137946
TCGA-09-2051-01A	T_cells_CD4_naive	0
TCGA-09-2051-01A	T_cells_CD4_memory_resting	0.244148581
TCGA-09-2051-01A	T_cells_CD4_memory_activated	0
TCGA-09-2051-01A	T_cells_follicular_helper	0.074879992
TCGA-09-2051-01A	T_cells_regulatory_(Tregs)	0.041710961
TCGA-09-2051-01A	T_cells_gamma_delta	0
TCGA-09-2051-01A	NK_cells_resting	0.043186857
TCGA-09-2051-01A	NK_cells_activated	0.013944327
TCGA-09-2051-01A	Monocytes	0.077786507
TCGA-09-2051-01A	Macrophages_M0	0.047718722
TCGA-09-2051-01A	Macrophages_M1	0.05450627
TCGA-09-2051-01A	Macrophages_M2	0.17285837
TCGA-09-2051-01A	Dendritic_cells_resting	0
TCGA-09-2051-01A	Dendritic_cells_activated	0.135486178
TCGA-09-2051-01A	Mast_cells_resting	0.02199666
TCGA-09-2051-01A	Mast_cells_activated	0
TCGA-09-2051-01A	Eosinophils	0
TCGA-09-2051-01A	Neutrophils	0.006321312
TCGA-10-0931-01A	B_cells_naive	0.087415907
TCGA-10-0931-01A	B_cells_memory	0
TCGA-10-0931-01A	Plasma_cells	0.030820343
TCGA-10-0931-01A	T_cells_CD8	0.07088374
TCGA-10-0931-01A	T_cells_CD4_naive	0
TCGA-10-0931-01A	T_cells_CD4_memory_resting	0.224296535
TCGA-10-0931-01A	T_cells_CD4_memory_activated	0

TCGA-10-0931-01A	T_cells_follicular_helper	0.062685435
TCGA-10-0931-01A	T_cells_regulatory_(Tregs)	0.011347534
TCGA-10-0931-01A	T_cells_gamma_delta	0
TCGA-10-0931-01A	NK_cells_resting	0.003338588
TCGA-10-0931-01A	NK_cells_activated	0.048746523
TCGA-10-0931-01A	Monocytes	0.07776465
TCGA-10-0931-01A	Macrophages_M0	0.091309058
TCGA-10-0931-01A	Macrophages_M1	0.030121194
TCGA-10-0931-01A	Macrophages_M2	0.120996389
TCGA-10-0931-01A	Dendritic_cells_resting	0
TCGA-10-0931-01A	Dendritic_cells_activated	0.07364594
TCGA-10-0931-01A	Mast_cells_resting	0
TCGA-10-0931-01A	Mast_cells_activated	0.066628162
TCGA-10-0931-01A	Eosinophils	0
TCGA-10-0931-01A	Neutrophils	0
TCGA-10-0937-01A	B_cells_naive	0
TCGA-10-0937-01A	B_cells_memory	0.000822207
TCGA-10-0937-01A	Plasma_cells	0
TCGA-10-0937-01A	T_cells_CD8	0.124567842
TCGA-10-0937-01A	T_cells_CD4_naive	0
TCGA-10-0937-01A	T_cells_CD4_memory_resting	0.147863136
TCGA-10-0937-01A	T_cells_CD4_memory_activated	0
TCGA-10-0937-01A	T_cells_follicular_helper	0.043801378
TCGA-10-0937-01A	T_cells_regulatory_(Tregs)	0.118123614
TCGA-10-0937-01A	T_cells_gamma_delta	0
TCGA-10-0937-01A	NK_cells_resting	0
TCGA-10-0937-01A	NK_cells_activated	0.102817737
TCGA-10-0937-01A	Monocytes	0.053155425
TCGA-10-0937-01A	Macrophages_M0	0.151079382
TCGA-10-0937-01A	Macrophages_M1	0.038528557
TCGA-10-0937-01A	Macrophages_M2	0.197968338
TCGA-10-0937-01A	Dendritic_cells_resting	0
TCGA-10-0937-01A	Dendritic_cells_activated	0.017571952

TCGA-10-0937-01A	Mast_cells_resting	0
TCGA-10-0937-01A	Mast_cells_activated	0.003700431
TCGA-10-0937-01A	Eosinophils	0
TCGA-10-0937-01A	Neutrophils	0
TCGA-13-0714-01A	B_cells_naive	0
TCGA-13-0714-01A	B_cells_memory	0.06899293
TCGA-13-0714-01A	Plasma_cells	0.006324259
TCGA-13-0714-01A	T_cells_CD8	0.15325327
TCGA-13-0714-01A	T_cells_CD4_naive	0
TCGA-13-0714-01A	T_cells_CD4_memory_resting	0.186602889
TCGA-13-0714-01A	T_cells_CD4_memory_activated	0
TCGA-13-0714-01A	T_cells_follicular_helper	0.008763663
TCGA-13-0714-01A	T_cells_regulatory_(Tregs)	0.091114312
TCGA-13-0714-01A	T_cells_gamma_delta	0
TCGA-13-0714-01A	NK_cells_resting	0
TCGA-13-0714-01A	NK_cells_activated	0.082444448
TCGA-13-0714-01A	Monocytes	0.022604757
TCGA-13-0714-01A	Macrophages_M0	0.146349429
TCGA-13-0714-01A	Macrophages_M1	0.058287784
TCGA-13-0714-01A	Macrophages_M2	0.119302687
TCGA-13-0714-01A	Dendritic_cells_resting	0.003591949
TCGA-13-0714-01A	Dendritic_cells_activated	0
TCGA-13-0714-01A	Mast_cells_resting	0.052367621
TCGA-13-0714-01A	Mast_cells_activated	0
TCGA-13-0714-01A	Eosinophils	0
TCGA-13-0714-01A	Neutrophils	0
TCGA-13-0725-01A	B_cells_naive	0.063075869
TCGA-13-0725-01A	B_cells_memory	0
TCGA-13-0725-01A	Plasma_cells	0.083443316
TCGA-13-0725-01A	T_cells_CD8	0.045011436
TCGA-13-0725-01A	T_cells_CD4_naive	0
TCGA-13-0725-01A	T_cells_CD4_memory_resting	0.107642819
TCGA-13-0725-01A	T_cells_CD4_memory_activated	0

TCGA-13-0725-01A	T_cells_follicular_helper	0.041864234
TCGA-13-0725-01A	T_cells_regulatory_(Tregs)	0.028407034
TCGA-13-0725-01A	T_cells_gamma_delta	0.002007269
TCGA-13-0725-01A	NK_cells_resting	0
TCGA-13-0725-01A	NK_cells_activated	0.012853855
TCGA-13-0725-01A	Monocytes	0.026301867
TCGA-13-0725-01A	Macrophages_M0	0.100240644
TCGA-13-0725-01A	Macrophages_M1	0.044077353
TCGA-13-0725-01A	Macrophages_M2	0.32765464
TCGA-13-0725-01A	Dendritic_cells_resting	0
TCGA-13-0725-01A	Dendritic_cells_activated	0.060829365
TCGA-13-0725-01A	Mast_cells_resting	0.03792652
TCGA-13-0725-01A	Mast_cells_activated	0
TCGA-13-0725-01A	Eosinophils	0
TCGA-13-0725-01A	Neutrophils	0.01866378
TCGA-13-0726-01A	B_cells_naive	0
TCGA-13-0726-01A	B_cells_memory	0.001659277
TCGA-13-0726-01A	Plasma_cells	0.017225288
TCGA-13-0726-01A	T_cells_CD8	0.037682429
TCGA-13-0726-01A	T_cells_CD4_naive	0
TCGA-13-0726-01A	T_cells_CD4_memory_resting	0.401067562
TCGA-13-0726-01A	T_cells_CD4_memory_activated	0
TCGA-13-0726-01A	T_cells_follicular_helper	0
TCGA-13-0726-01A	T_cells_regulatory_(Tregs)	0.103959819
TCGA-13-0726-01A	T_cells_gamma_delta	0
TCGA-13-0726-01A	NK_cells_resting	0.002487996
TCGA-13-0726-01A	NK_cells_activated	0.10022721
TCGA-13-0726-01A	Monocytes	0.005847985
TCGA-13-0726-01A	Macrophages_M0	0.045166047
TCGA-13-0726-01A	Macrophages_M1	0.06553449
TCGA-13-0726-01A	Macrophages_M2	0.166609528
TCGA-13-0726-01A	Dendritic_cells_resting	0
TCGA-13-0726-01A	Dendritic_cells_activated	0.031532807

TCGA-13-0726-01A	Mast_cells_resting	0.018133517
TCGA-13-0726-01A	Mast_cells_activated	0
TCGA-13-0726-01A	Eosinophils	0
TCGA-13-0726-01A	Neutrophils	0.002866045
TCGA-13-0762-01A	B_cells_naive	0
TCGA-13-0762-01A	B_cells_memory	0.014055915
TCGA-13-0762-01A	Plasma_cells	0.000187483
TCGA-13-0762-01A	T_cells_CD8	0.019230539
TCGA-13-0762-01A	T_cells_CD4_naive	0
TCGA-13-0762-01A	T_cells_CD4_memory_resting	0.151288335
TCGA-13-0762-01A	T_cells_CD4_memory_activated	0
TCGA-13-0762-01A	T_cells_follicular_helper	0
TCGA-13-0762-01A	T_cells_regulatory_(Tregs)	0.066351987
TCGA-13-0762-01A	T_cells_gamma_delta	0
TCGA-13-0762-01A	NK_cells_resting	0.002859761
TCGA-13-0762-01A	NK_cells_activated	0.091409492
TCGA-13-0762-01A	Monocytes	0.000887174
TCGA-13-0762-01A	Macrophages_M0	0.461594445
TCGA-13-0762-01A	Macrophages_M1	0.016561393
TCGA-13-0762-01A	Macrophages_M2	0.121277359
TCGA-13-0762-01A	Dendritic_cells_resting	0
TCGA-13-0762-01A	Dendritic_cells_activated	0
TCGA-13-0762-01A	Mast_cells_resting	0.054296116
TCGA-13-0762-01A	Mast_cells_activated	0
TCGA-13-0762-01A	Eosinophils	0
TCGA-13-0762-01A	Neutrophils	0
TCGA-13-0766-01A	B_cells_naive	0.063423712
TCGA-13-0766-01A	B_cells_memory	0
TCGA-13-0766-01A	Plasma_cells	0.000895975
TCGA-13-0766-01A	T_cells_CD8	0.06664058
TCGA-13-0766-01A	T_cells_CD4_naive	0
TCGA-13-0766-01A	T_cells_CD4_memory_resting	0.112031452
TCGA-13-0766-01A	T_cells_CD4_memory_activated	0

TCGA-13-0766-01A	T_cells_follicular_helper	0.044346038
TCGA-13-0766-01A	T_cells_regulatory_(Tregs)	0.014474816
TCGA-13-0766-01A	T_cells_gamma_delta	0
TCGA-13-0766-01A	NK_cells_resting	0
TCGA-13-0766-01A	NK_cells_activated	0.065831126
TCGA-13-0766-01A	Monocytes	0.059287745
TCGA-13-0766-01A	Macrophages_M0	0.134125906
TCGA-13-0766-01A	Macrophages_M1	0.039984822
TCGA-13-0766-01A	Macrophages_M2	0.155016992
TCGA-13-0766-01A	Dendritic_cells_resting	0
TCGA-13-0766-01A	Dendritic_cells_activated	0.216675505
TCGA-13-0766-01A	Mast_cells_resting	0.027068292
TCGA-13-0766-01A	Mast_cells_activated	0
TCGA-13-0766-01A	Eosinophils	0.00019704
TCGA-13-0766-01A	Neutrophils	0
TCGA-13-0797-01A	B_cells_naive	0.000687423
TCGA-13-0797-01A	B_cells_memory	0.014038016
TCGA-13-0797-01A	Plasma_cells	0.046478884
TCGA-13-0797-01A	T_cells_CD8	0.153460683
TCGA-13-0797-01A	T_cells_CD4_naive	0
TCGA-13-0797-01A	T_cells_CD4_memory_resting	0.262899694
TCGA-13-0797-01A	T_cells_CD4_memory_activated	0
TCGA-13-0797-01A	T_cells_follicular_helper	0.031153297
TCGA-13-0797-01A	T_cells_regulatory_(Tregs)	0.097768111
TCGA-13-0797-01A	T_cells_gamma_delta	0
TCGA-13-0797-01A	NK_cells_resting	0
TCGA-13-0797-01A	NK_cells_activated	0.086809645
TCGA-13-0797-01A	Monocytes	0.063678033
TCGA-13-0797-01A	Macrophages_M0	0.018123616
TCGA-13-0797-01A	Macrophages_M1	0.03580545
TCGA-13-0797-01A	Macrophages_M2	0.103896663
TCGA-13-0797-01A	Dendritic_cells_resting	0
TCGA-13-0797-01A	Dendritic_cells_activated	0.076674944

TCGA-13-0797-01A	Mast_cells_resting	0.008525539
TCGA-13-0797-01A	Mast_cells_activated	0
TCGA-13-0797-01A	Eosinophils	0
TCGA-13-0797-01A	Neutrophils	0
TCGA-13-0800-01A	B_cells_naive	0
TCGA-13-0800-01A	B_cells_memory	0
TCGA-13-0800-01A	Plasma_cells	0.025150091
TCGA-13-0800-01A	T_cells_CD8	0
TCGA-13-0800-01A	T_cells_CD4_naive	0
TCGA-13-0800-01A	T_cells_CD4_memory_resting	0.20998333
TCGA-13-0800-01A	T_cells_CD4_memory_activated	0
TCGA-13-0800-01A	T_cells_follicular_helper	0.120734791
TCGA-13-0800-01A	T_cells_regulatory_(Tregs)	0.060391614
TCGA-13-0800-01A	T_cells_gamma_delta	0
TCGA-13-0800-01A	NK_cells_resting	0
TCGA-13-0800-01A	NK_cells_activated	0.065124473
TCGA-13-0800-01A	Monocytes	0.094470621
TCGA-13-0800-01A	Macrophages_M0	0.099507
TCGA-13-0800-01A	Macrophages_M1	0
TCGA-13-0800-01A	Macrophages_M2	0.04266796
TCGA-13-0800-01A	Dendritic_cells_resting	0
TCGA-13-0800-01A	Dendritic_cells_activated	0.104460118
TCGA-13-0800-01A	Mast_cells_resting	0.142429799
TCGA-13-0800-01A	Mast_cells_activated	0
TCGA-13-0800-01A	Eosinophils	0.035080204
TCGA-13-0800-01A	Neutrophils	0
TCGA-13-0885-01A	B_cells_naive	0
TCGA-13-0885-01A	B_cells_memory	0.022919194
TCGA-13-0885-01A	Plasma_cells	0
TCGA-13-0885-01A	T_cells_CD8	0.188216009
TCGA-13-0885-01A	T_cells_CD4_naive	0
TCGA-13-0885-01A	T_cells_CD4_memory_resting	0.070194467
TCGA-13-0885-01A	T_cells_CD4_memory_activated	0.032437161

TCGA-13-0885-01A	T_cells_follicular_helper	0.028250843
TCGA-13-0885-01A	T_cells_regulatory_(Tregs)	0.097864261
TCGA-13-0885-01A	T_cells_gamma_delta	0
TCGA-13-0885-01A	NK_cells_resting	0
TCGA-13-0885-01A	NK_cells_activated	0.043426606
TCGA-13-0885-01A	Monocytes	0.010415341
TCGA-13-0885-01A	Macrophages_M0	0.035477194
TCGA-13-0885-01A	Macrophages_M1	0.134129729
TCGA-13-0885-01A	Macrophages_M2	0.246551111
TCGA-13-0885-01A	Dendritic_cells_resting	0.033443266
TCGA-13-0885-01A	Dendritic_cells_activated	0
TCGA-13-0885-01A	Mast_cells_resting	0.056674817
TCGA-13-0885-01A	Mast_cells_activated	0
TCGA-13-0885-01A	Eosinophils	0
TCGA-13-0885-01A	Neutrophils	0
TCGA-13-0886-01A	B_cells_naive	0
TCGA-13-0886-01A	B_cells_memory	0.042502706
TCGA-13-0886-01A	Plasma_cells	0
TCGA-13-0886-01A	T_cells_CD8	0.136129793
TCGA-13-0886-01A	T_cells_CD4_naive	0
TCGA-13-0886-01A	T_cells_CD4_memory_resting	0.087648146
TCGA-13-0886-01A	T_cells_CD4_memory_activated	0
TCGA-13-0886-01A	T_cells_follicular_helper	0.052213586
TCGA-13-0886-01A	T_cells_regulatory_(Tregs)	0.073556394
TCGA-13-0886-01A	T_cells_gamma_delta	0
TCGA-13-0886-01A	NK_cells_resting	0
TCGA-13-0886-01A	NK_cells_activated	0.074109815
TCGA-13-0886-01A	Monocytes	0.004382102
TCGA-13-0886-01A	Macrophages_M0	0.191479891
TCGA-13-0886-01A	Macrophages_M1	0.115587937
TCGA-13-0886-01A	Macrophages_M2	0.186839488
TCGA-13-0886-01A	Dendritic_cells_resting	0.011263044
TCGA-13-0886-01A	Dendritic_cells_activated	0

TCGA-13-0886-01A	Mast_cells_resting	0.02006194
TCGA-13-0886-01A	Mast_cells_activated	0
TCGA-13-0886-01A	Eosinophils	0.004225158
TCGA-13-0886-01A	Neutrophils	0
TCGA-13-0887-01A	B_cells_naive	0.061478012
TCGA-13-0887-01A	B_cells_memory	0
TCGA-13-0887-01A	Plasma_cells	0.034519148
TCGA-13-0887-01A	T_cells_CD8	0.046408862
TCGA-13-0887-01A	T_cells_CD4_naive	0
TCGA-13-0887-01A	T_cells_CD4_memory_resting	0.109109156
TCGA-13-0887-01A	T_cells_CD4_memory_activated	0
TCGA-13-0887-01A	T_cells_follicular_helper	0.05233315
TCGA-13-0887-01A	T_cells_regulatory_(Tregs)	0.037346348
TCGA-13-0887-01A	T_cells_gamma_delta	0
TCGA-13-0887-01A	NK_cells_resting	0
TCGA-13-0887-01A	NK_cells_activated	0.0777473
TCGA-13-0887-01A	Monocytes	0.056766326
TCGA-13-0887-01A	Macrophages_M0	0.035563984
TCGA-13-0887-01A	Macrophages_M1	0.022452034
TCGA-13-0887-01A	Macrophages_M2	0.133889038
TCGA-13-0887-01A	Dendritic_cells_resting	0
TCGA-13-0887-01A	Dendritic_cells_activated	0.244144275
TCGA-13-0887-01A	Mast_cells_resting	0
TCGA-13-0887-01A	Mast_cells_activated	0.08727926
TCGA-13-0887-01A	Eosinophils	0
TCGA-13-0887-01A	Neutrophils	0.000963107
TCGA-13-0891-01A	B_cells_naive	0.041500987
TCGA-13-0891-01A	B_cells_memory	0
TCGA-13-0891-01A	Plasma_cells	0.023895747
TCGA-13-0891-01A	T_cells_CD8	0.072024217
TCGA-13-0891-01A	T_cells_CD4_naive	0
TCGA-13-0891-01A	T_cells_CD4_memory_resting	0.121763618
TCGA-13-0891-01A	T_cells_CD4_memory_activated	0

TCGA-13-0891-01A	T_cells_follicular_helper	0.055437475
TCGA-13-0891-01A	T_cells_regulatory_(Tregs)	0.009264452
TCGA-13-0891-01A	T_cells_gamma_delta	0
TCGA-13-0891-01A	NK_cells_resting	0
TCGA-13-0891-01A	NK_cells_activated	0.092767727
TCGA-13-0891-01A	Monocytes	0.001982136
TCGA-13-0891-01A	Macrophages_M0	0.23744378
TCGA-13-0891-01A	Macrophages_M1	0.056527196
TCGA-13-0891-01A	Macrophages_M2	0.216624322
TCGA-13-0891-01A	Dendritic_cells_resting	0
TCGA-13-0891-01A	Dendritic_cells_activated	0.027732476
TCGA-13-0891-01A	Mast_cells_resting	0.043035866
TCGA-13-0891-01A	Mast_cells_activated	0
TCGA-13-0891-01A	Eosinophils	0
TCGA-13-0891-01A	Neutrophils	0
TCGA-13-0897-01A	B_cells_naive	0.000969606
TCGA-13-0897-01A	B_cells_memory	0
TCGA-13-0897-01A	Plasma_cells	0
TCGA-13-0897-01A	T_cells_CD8	0.098960335
TCGA-13-0897-01A	T_cells_CD4_naive	0
TCGA-13-0897-01A	T_cells_CD4_memory_resting	0.1191592
TCGA-13-0897-01A	T_cells_CD4_memory_activated	0.033226877
TCGA-13-0897-01A	T_cells_follicular_helper	0.032057195
TCGA-13-0897-01A	T_cells_regulatory_(Tregs)	0.126338325
TCGA-13-0897-01A	T_cells_gamma_delta	0
TCGA-13-0897-01A	NK_cells_resting	0
TCGA-13-0897-01A	NK_cells_activated	0.088584455
TCGA-13-0897-01A	Monocytes	0
TCGA-13-0897-01A	Macrophages_M0	0.10461171
TCGA-13-0897-01A	Macrophages_M1	0.136693998
TCGA-13-0897-01A	Macrophages_M2	0.203683463
TCGA-13-0897-01A	Dendritic_cells_resting	0.003737922
TCGA-13-0897-01A	Dendritic_cells_activated	0.016454629

TCGA-13-0897-01A	Mast_cells_resting	0.035522285
TCGA-13-0897-01A	Mast_cells_activated	0
TCGA-13-0897-01A	Eosinophils	0
TCGA-13-0897-01A	Neutrophils	0
TCGA-13-0906-01A	B_cells_naive	0.077680471
TCGA-13-0906-01A	B_cells_memory	0
TCGA-13-0906-01A	Plasma_cells	0.024865446
TCGA-13-0906-01A	T_cells_CD8	0.10483739
TCGA-13-0906-01A	T_cells_CD4_naive	0
TCGA-13-0906-01A	T_cells_CD4_memory_resting	0.202937183
TCGA-13-0906-01A	T_cells_CD4_memory_activated	0
TCGA-13-0906-01A	T_cells_follicular_helper	0.068658981
TCGA-13-0906-01A	T_cells_regulatory_(Tregs)	0.0674448
TCGA-13-0906-01A	T_cells_gamma_delta	0
TCGA-13-0906-01A	NK_cells_resting	0
TCGA-13-0906-01A	NK_cells_activated	0.07275208
TCGA-13-0906-01A	Monocytes	0.010265717
TCGA-13-0906-01A	Macrophages_M0	0.069738432
TCGA-13-0906-01A	Macrophages_M1	0.128026348
TCGA-13-0906-01A	Macrophages_M2	0.029256362
TCGA-13-0906-01A	Dendritic_cells_resting	0
TCGA-13-0906-01A	Dendritic_cells_activated	0.140474375
TCGA-13-0906-01A	Mast_cells_resting	0
TCGA-13-0906-01A	Mast_cells_activated	0
TCGA-13-0906-01A	Eosinophils	0.003062416
TCGA-13-0906-01A	Neutrophils	0
TCGA-13-0911-01A	B_cells_naive	0
TCGA-13-0911-01A	B_cells_memory	0.044812749
TCGA-13-0911-01A	Plasma_cells	0
TCGA-13-0911-01A	T_cells_CD8	0.035487505
TCGA-13-0911-01A	T_cells_CD4_naive	0
TCGA-13-0911-01A	T_cells_CD4_memory_resting	0.036884553
TCGA-13-0911-01A	T_cells_CD4_memory_activated	0

TCGA-13-0911-01A	T_cells_follicular_helper	0
TCGA-13-0911-01A	T_cells_regulatory_(Tregs)	0.07236762
TCGA-13-0911-01A	T_cells_gamma_delta	0
TCGA-13-0911-01A	NK_cells_resting	0
TCGA-13-0911-01A	NK_cells_activated	0.042683585
TCGA-13-0911-01A	Monocytes	0.006516221
TCGA-13-0911-01A	Macrophages_M0	0.547182051
TCGA-13-0911-01A	Macrophages_M1	0.07497764
TCGA-13-0911-01A	Macrophages_M2	0.110305806
TCGA-13-0911-01A	Dendritic_cells_resting	0.021828494
TCGA-13-0911-01A	Dendritic_cells_activated	0
TCGA-13-0911-01A	Mast_cells_resting	0.006953776
TCGA-13-0911-01A	Mast_cells_activated	0
TCGA-13-0911-01A	Eosinophils	0
TCGA-13-0911-01A	Neutrophils	0
TCGA-13-0916-01A	B_cells_naive	0.060702038
TCGA-13-0916-01A	B_cells_memory	0
TCGA-13-0916-01A	Plasma_cells	0.158498585
TCGA-13-0916-01A	T_cells_CD8	0.136449565
TCGA-13-0916-01A	T_cells_CD4_naive	0
TCGA-13-0916-01A	T_cells_CD4_memory_resting	0.090645236
TCGA-13-0916-01A	T_cells_CD4_memory_activated	0
TCGA-13-0916-01A	T_cells_follicular_helper	0.02877897
TCGA-13-0916-01A	T_cells_regulatory_(Tregs)	0.07128309
TCGA-13-0916-01A	T_cells_gamma_delta	0
TCGA-13-0916-01A	NK_cells_resting	0.004950194
TCGA-13-0916-01A	NK_cells_activated	0.013565608
TCGA-13-0916-01A	Monocytes	0
TCGA-13-0916-01A	Macrophages_M0	0.165113832
TCGA-13-0916-01A	Macrophages_M1	0.158136653
TCGA-13-0916-01A	Macrophages_M2	0.095794263
TCGA-13-0916-01A	Dendritic_cells_resting	0
TCGA-13-0916-01A	Dendritic_cells_activated	0

TCGA-13-0916-01A	Mast_cells_resting	0.016081966
TCGA-13-0916-01A	Mast_cells_activated	0
TCGA-13-0916-01A	Eosinophils	0
TCGA-13-0916-01A	Neutrophils	0
TCGA-13-0920-01A	B_cells_naive	0.039524069
TCGA-13-0920-01A	B_cells_memory	0
TCGA-13-0920-01A	Plasma_cells	0.286077104
TCGA-13-0920-01A	T_cells_CD8	0.019757049
TCGA-13-0920-01A	T_cells_CD4_naive	0
TCGA-13-0920-01A	T_cells_CD4_memory_resting	0.137919755
TCGA-13-0920-01A	T_cells_CD4_memory_activated	0.059159354
TCGA-13-0920-01A	T_cells_follicular_helper	0
TCGA-13-0920-01A	T_cells_regulatory_(Tregs)	0
TCGA-13-0920-01A	T_cells_gamma_delta	0.030519936
TCGA-13-0920-01A	NK_cells_resting	0
TCGA-13-0920-01A	NK_cells_activated	0.083775866
TCGA-13-0920-01A	Monocytes	0.004473269
TCGA-13-0920-01A	Macrophages_M0	0.0457485
TCGA-13-0920-01A	Macrophages_M1	0.098805128
TCGA-13-0920-01A	Macrophages_M2	0.102699725
TCGA-13-0920-01A	Dendritic_cells_resting	0
TCGA-13-0920-01A	Dendritic_cells_activated	0.007982853
TCGA-13-0920-01A	Mast_cells_resting	0.08355739
TCGA-13-0920-01A	Mast_cells_activated	0
TCGA-13-0920-01A	Eosinophils	0
TCGA-13-0920-01A	Neutrophils	0
TCGA-13-0923-01A	B_cells_naive	0.038287352
TCGA-13-0923-01A	B_cells_memory	0
TCGA-13-0923-01A	Plasma_cells	0.02582845
TCGA-13-0923-01A	T_cells_CD8	0.29219904
TCGA-13-0923-01A	T_cells_CD4_naive	0
TCGA-13-0923-01A	T_cells_CD4_memory_resting	0
TCGA-13-0923-01A	T_cells_CD4_memory_activated	0.043972501

TCGA-13-0923-01A	T_cells_follicular_helper	0.142572208
TCGA-13-0923-01A	T_cells_regulatory_(Tregs)	0.013611596
TCGA-13-0923-01A	T_cells_gamma_delta	0
TCGA-13-0923-01A	NK_cells_resting	0.001231277
TCGA-13-0923-01A	NK_cells_activated	0.067294806
TCGA-13-0923-01A	Monocytes	0.02882234
TCGA-13-0923-01A	Macrophages_M0	0.07554072
TCGA-13-0923-01A	Macrophages_M1	0.073427333
TCGA-13-0923-01A	Macrophages_M2	0.065313679
TCGA-13-0923-01A	Dendritic_cells_resting	0
TCGA-13-0923-01A	Dendritic_cells_activated	0.084404543
TCGA-13-0923-01A	Mast_cells_resting	0
TCGA-13-0923-01A	Mast_cells_activated	0.04574831
TCGA-13-0923-01A	Eosinophils	0
TCGA-13-0923-01A	Neutrophils	0.001745846
TCGA-13-0924-01A	B_cells_naive	0.012254126
TCGA-13-0924-01A	B_cells_memory	0
TCGA-13-0924-01A	Plasma_cells	0.087723297
TCGA-13-0924-01A	T_cells_CD8	0.128923304
TCGA-13-0924-01A	T_cells_CD4_naive	0
TCGA-13-0924-01A	T_cells_CD4_memory_resting	0.066301431
TCGA-13-0924-01A	T_cells_CD4_memory_activated	0
TCGA-13-0924-01A	T_cells_follicular_helper	0.062159282
TCGA-13-0924-01A	T_cells_regulatory_(Tregs)	0.077606889
TCGA-13-0924-01A	T_cells_gamma_delta	0
TCGA-13-0924-01A	NK_cells_resting	0
TCGA-13-0924-01A	NK_cells_activated	0.079809665
TCGA-13-0924-01A	Monocytes	0.030260903
TCGA-13-0924-01A	Macrophages_M0	0.110899981
TCGA-13-0924-01A	Macrophages_M1	0.127685461
TCGA-13-0924-01A	Macrophages_M2	0.151255999
TCGA-13-0924-01A	Dendritic_cells_resting	0.009743942
TCGA-13-0924-01A	Dendritic_cells_activated	0

TCGA-13-0924-01A	Mast_cells_resting	0
TCGA-13-0924-01A	Mast_cells_activated	0.05537572
TCGA-13-0924-01A	Eosinophils	0
TCGA-13-0924-01A	Neutrophils	0
TCGA-13-1407-01A	B_cells_naive	0
TCGA-13-1407-01A	B_cells_memory	0.008366733
TCGA-13-1407-01A	Plasma_cells	0.043228003
TCGA-13-1407-01A	T_cells_CD8	0.103079221
TCGA-13-1407-01A	T_cells_CD4_naive	0
TCGA-13-1407-01A	T_cells_CD4_memory_resting	0.148870968
TCGA-13-1407-01A	T_cells_CD4_memory_activated	0
TCGA-13-1407-01A	T_cells_follicular_helper	0.099793082
TCGA-13-1407-01A	T_cells_regulatory_(Tregs)	0.050153078
TCGA-13-1407-01A	T_cells_gamma_delta	0
TCGA-13-1407-01A	NK_cells_resting	0
TCGA-13-1407-01A	NK_cells_activated	0.124428421
TCGA-13-1407-01A	Monocytes	0.023629915
TCGA-13-1407-01A	Macrophages_M0	0.339999156
TCGA-13-1407-01A	Macrophages_M1	0.044303396
TCGA-13-1407-01A	Macrophages_M2	0.014148029
TCGA-13-1407-01A	Dendritic_cells_resting	0
TCGA-13-1407-01A	Dendritic_cells_activated	0
TCGA-13-1407-01A	Mast_cells_resting	0
TCGA-13-1407-01A	Mast_cells_activated	0
TCGA-13-1407-01A	Eosinophils	0
TCGA-13-1407-01A	Neutrophils	0
TCGA-13-1408-01A	B_cells_naive	0.006240418
TCGA-13-1408-01A	B_cells_memory	0
TCGA-13-1408-01A	Plasma_cells	0.203380926
TCGA-13-1408-01A	T_cells_CD8	0.0365895
TCGA-13-1408-01A	T_cells_CD4_naive	0
TCGA-13-1408-01A	T_cells_CD4_memory_resting	0.2558968
TCGA-13-1408-01A	T_cells_CD4_memory_activated	0

TCGA-13-1408-01A	T_cells_follicular_helper	0.043302677
TCGA-13-1408-01A	T_cells_regulatory_(Tregs)	0.019185788
TCGA-13-1408-01A	T_cells_gamma_delta	0
TCGA-13-1408-01A	NK_cells_resting	0
TCGA-13-1408-01A	NK_cells_activated	0.036193165
TCGA-13-1408-01A	Monocytes	0.024635178
TCGA-13-1408-01A	Macrophages_M0	0.201049558
TCGA-13-1408-01A	Macrophages_M1	0.106870252
TCGA-13-1408-01A	Macrophages_M2	0.061178859
TCGA-13-1408-01A	Dendritic_cells_resting	0
TCGA-13-1408-01A	Dendritic_cells_activated	0
TCGA-13-1408-01A	Mast_cells_resting	0
TCGA-13-1408-01A	Mast_cells_activated	0.005476879
TCGA-13-1408-01A	Eosinophils	0
TCGA-13-1408-01A	Neutrophils	0
TCGA-13-1410-01A	B_cells_naive	0.046079058
TCGA-13-1410-01A	B_cells_memory	0
TCGA-13-1410-01A	Plasma_cells	0.051147565
TCGA-13-1410-01A	T_cells_CD8	0
TCGA-13-1410-01A	T_cells_CD4_naive	0
TCGA-13-1410-01A	T_cells_CD4_memory_resting	0.187104248
TCGA-13-1410-01A	T_cells_CD4_memory_activated	0
TCGA-13-1410-01A	T_cells_follicular_helper	0.064414114
TCGA-13-1410-01A	T_cells_regulatory_(Tregs)	0.019056282
TCGA-13-1410-01A	T_cells_gamma_delta	0.038525871
TCGA-13-1410-01A	NK_cells_resting	0
TCGA-13-1410-01A	NK_cells_activated	0.016478388
TCGA-13-1410-01A	Monocytes	0
TCGA-13-1410-01A	Macrophages_M0	0.145585955
TCGA-13-1410-01A	Macrophages_M1	0.161126281
TCGA-13-1410-01A	Macrophages_M2	0.17225589
TCGA-13-1410-01A	Dendritic_cells_resting	0
TCGA-13-1410-01A	Dendritic_cells_activated	0.069093015

TCGA-13-1410-01A	Mast_cells_resting	0
TCGA-13-1410-01A	Mast_cells_activated	0.029133334
TCGA-13-1410-01A	Eosinophils	0
TCGA-13-1410-01A	Neutrophils	0
TCGA-13-1411-01A	B_cells_naive	0.006207934
TCGA-13-1411-01A	B_cells_memory	0
TCGA-13-1411-01A	Plasma_cells	0.00783135
TCGA-13-1411-01A	T_cells_CD8	0.084441898
TCGA-13-1411-01A	T_cells_CD4_naive	0
TCGA-13-1411-01A	T_cells_CD4_memory_resting	0.136233692
TCGA-13-1411-01A	T_cells_CD4_memory_activated	0.00483183
TCGA-13-1411-01A	T_cells_follicular_helper	0
TCGA-13-1411-01A	T_cells_regulatory_(Tregs)	0.044691013
TCGA-13-1411-01A	T_cells_gamma_delta	0
TCGA-13-1411-01A	NK_cells_resting	0.021144844
TCGA-13-1411-01A	NK_cells_activated	0.018581358
TCGA-13-1411-01A	Monocytes	0
TCGA-13-1411-01A	Macrophages_M0	0.34179436
TCGA-13-1411-01A	Macrophages_M1	0.078056805
TCGA-13-1411-01A	Macrophages_M2	0.205497735
TCGA-13-1411-01A	Dendritic_cells_resting	0.012849231
TCGA-13-1411-01A	Dendritic_cells_activated	0
TCGA-13-1411-01A	Mast_cells_resting	0
TCGA-13-1411-01A	Mast_cells_activated	0.009186735
TCGA-13-1411-01A	Eosinophils	0
TCGA-13-1411-01A	Neutrophils	0.028651215
TCGA-13-1483-01A	B_cells_naive	0.0122047
TCGA-13-1483-01A	B_cells_memory	0
TCGA-13-1483-01A	Plasma_cells	0
TCGA-13-1483-01A	T_cells_CD8	0.042635834
TCGA-13-1483-01A	T_cells_CD4_naive	0
TCGA-13-1483-01A	T_cells_CD4_memory_resting	0.197443002
TCGA-13-1483-01A	T_cells_CD4_memory_activated	0

TCGA-13-1483-01A	T_cells_follicular_helper	0.019834665
TCGA-13-1483-01A	T_cells_regulatory_(Tregs)	0.057806399
TCGA-13-1483-01A	T_cells_gamma_delta	0
TCGA-13-1483-01A	NK_cells_resting	0
TCGA-13-1483-01A	NK_cells_activated	0.026279005
TCGA-13-1483-01A	Monocytes	0.032519936
TCGA-13-1483-01A	Macrophages_M0	0.308100717
TCGA-13-1483-01A	Macrophages_M1	0.025635232
TCGA-13-1483-01A	Macrophages_M2	0.097872342
TCGA-13-1483-01A	Dendritic_cells_resting	0
TCGA-13-1483-01A	Dendritic_cells_activated	0.151554067
TCGA-13-1483-01A	Mast_cells_resting	0
TCGA-13-1483-01A	Mast_cells_activated	0.0281141
TCGA-13-1483-01A	Eosinophils	0
TCGA-13-1483-01A	Neutrophils	0
TCGA-13-1488-01A	B_cells_naive	0.045866007
TCGA-13-1488-01A	B_cells_memory	0
TCGA-13-1488-01A	Plasma_cells	0.026086846
TCGA-13-1488-01A	T_cells_CD8	0.022511225
TCGA-13-1488-01A	T_cells_CD4_naive	0
TCGA-13-1488-01A	T_cells_CD4_memory_resting	0.129612367
TCGA-13-1488-01A	T_cells_CD4_memory_activated	0.030827304
TCGA-13-1488-01A	T_cells_follicular_helper	0.031869979
TCGA-13-1488-01A	T_cells_regulatory_(Tregs)	0.012307765
TCGA-13-1488-01A	T_cells_gamma_delta	0
TCGA-13-1488-01A	NK_cells_resting	0
TCGA-13-1488-01A	NK_cells_activated	0.095289434
TCGA-13-1488-01A	Monocytes	0
TCGA-13-1488-01A	Macrophages_M0	0.254365074
TCGA-13-1488-01A	Macrophages_M1	0.219212546
TCGA-13-1488-01A	Macrophages_M2	0.096090983
TCGA-13-1488-01A	Dendritic_cells_resting	0
TCGA-13-1488-01A	Dendritic_cells_activated	0.003631993

TCGA-13-1488-01A	Mast_cells_resting	0.032328477
TCGA-13-1488-01A	Mast_cells_activated	0
TCGA-13-1488-01A	Eosinophils	0
TCGA-13-1488-01A	Neutrophils	0
TCGA-13-1489-01A	B_cells_naive	0
TCGA-13-1489-01A	B_cells_memory	0.114410176
TCGA-13-1489-01A	Plasma_cells	0
TCGA-13-1489-01A	T_cells_CD8	0.019054723
TCGA-13-1489-01A	T_cells_CD4_naive	0
TCGA-13-1489-01A	T_cells_CD4_memory_resting	0.221310619
TCGA-13-1489-01A	T_cells_CD4_memory_activated	0
TCGA-13-1489-01A	T_cells_follicular_helper	0
TCGA-13-1489-01A	T_cells_regulatory_(Tregs)	0.045542264
TCGA-13-1489-01A	T_cells_gamma_delta	0
TCGA-13-1489-01A	NK_cells_resting	0
TCGA-13-1489-01A	NK_cells_activated	0.122023006
TCGA-13-1489-01A	Monocytes	0.107034101
TCGA-13-1489-01A	Macrophages_M0	0.132661702
TCGA-13-1489-01A	Macrophages_M1	0.073700447
TCGA-13-1489-01A	Macrophages_M2	0.070375088
TCGA-13-1489-01A	Dendritic_cells_resting	0
TCGA-13-1489-01A	Dendritic_cells_activated	0.085214889
TCGA-13-1489-01A	Mast_cells_resting	0
TCGA-13-1489-01A	Mast_cells_activated	0
TCGA-13-1489-01A	Eosinophils	0.008672985
TCGA-13-1489-01A	Neutrophils	0
TCGA-13-1492-01A	B_cells_naive	0.068424197
TCGA-13-1492-01A	B_cells_memory	0
TCGA-13-1492-01A	Plasma_cells	0.064742871
TCGA-13-1492-01A	T_cells_CD8	0.139346696
TCGA-13-1492-01A	T_cells_CD4_naive	0
TCGA-13-1492-01A	T_cells_CD4_memory_resting	0.025729745
TCGA-13-1492-01A	T_cells_CD4_memory_activated	0

TCGA-13-1492-01A	T_cells_follicular_helper	0.005004916
TCGA-13-1492-01A	T_cells_regulatory_(Tregs)	0.064038969
TCGA-13-1492-01A	T_cells_gamma_delta	0
TCGA-13-1492-01A	NK_cells_resting	0
TCGA-13-1492-01A	NK_cells_activated	0.042058825
TCGA-13-1492-01A	Monocytes	0.041609282
TCGA-13-1492-01A	Macrophages_M0	0.106881311
TCGA-13-1492-01A	Macrophages_M1	0.052935874
TCGA-13-1492-01A	Macrophages_M2	0.241043018
TCGA-13-1492-01A	Dendritic_cells_resting	0
TCGA-13-1492-01A	Dendritic_cells_activated	0.103266819
TCGA-13-1492-01A	Mast_cells_resting	0.044917476
TCGA-13-1492-01A	Mast_cells_activated	0
TCGA-13-1492-01A	Eosinophils	0
TCGA-13-1492-01A	Neutrophils	0
TCGA-13-1496-01A	B_cells_naive	0
TCGA-13-1496-01A	B_cells_memory	0.100626054
TCGA-13-1496-01A	Plasma_cells	0.06338841
TCGA-13-1496-01A	T_cells_CD8	0.092491655
TCGA-13-1496-01A	T_cells_CD4_naive	0
TCGA-13-1496-01A	T_cells_CD4_memory_resting	0.153891172
TCGA-13-1496-01A	T_cells_CD4_memory_activated	0.021573824
TCGA-13-1496-01A	T_cells_follicular_helper	0.052653513
TCGA-13-1496-01A	T_cells_regulatory_(Tregs)	0.019126922
TCGA-13-1496-01A	T_cells_gamma_delta	0.060456476
TCGA-13-1496-01A	NK_cells_resting	0
TCGA-13-1496-01A	NK_cells_activated	0.031309099
TCGA-13-1496-01A	Monocytes	0
TCGA-13-1496-01A	Macrophages_M0	0.020148506
TCGA-13-1496-01A	Macrophages_M1	0.127686719
TCGA-13-1496-01A	Macrophages_M2	0.126313794
TCGA-13-1496-01A	Dendritic_cells_resting	0
TCGA-13-1496-01A	Dendritic_cells_activated	0.08767035

TCGA-13-1496-01A	Mast_cells_resting	0.020026239
TCGA-13-1496-01A	Mast_cells_activated	0
TCGA-13-1496-01A	Eosinophils	0
TCGA-13-1496-01A	Neutrophils	0.022637268
TCGA-13-1498-01A	B_cells_naive	0
TCGA-13-1498-01A	B_cells_memory	0.025384897
TCGA-13-1498-01A	Plasma_cells	0
TCGA-13-1498-01A	T_cells_CD8	0.045827343
TCGA-13-1498-01A	T_cells_CD4_naive	0
TCGA-13-1498-01A	T_cells_CD4_memory_resting	0.209078661
TCGA-13-1498-01A	T_cells_CD4_memory_activated	0
TCGA-13-1498-01A	T_cells_follicular_helper	0.037674189
TCGA-13-1498-01A	T_cells_regulatory_(Tregs)	0.029625726
TCGA-13-1498-01A	T_cells_gamma_delta	0
TCGA-13-1498-01A	NK_cells_resting	0
TCGA-13-1498-01A	NK_cells_activated	0.136387184
TCGA-13-1498-01A	Monocytes	0.020206334
TCGA-13-1498-01A	Macrophages_M0	0.30417408
TCGA-13-1498-01A	Macrophages_M1	0.113104235
TCGA-13-1498-01A	Macrophages_M2	0.056036241
TCGA-13-1498-01A	Dendritic_cells_resting	0.015641735
TCGA-13-1498-01A	Dendritic_cells_activated	0
TCGA-13-1498-01A	Mast_cells_resting	0.006859376
TCGA-13-1498-01A	Mast_cells_activated	0
TCGA-13-1498-01A	Eosinophils	0
TCGA-13-1498-01A	Neutrophils	0
TCGA-13-1499-01A	B_cells_naive	0.045884034
TCGA-13-1499-01A	B_cells_memory	0.029942603
TCGA-13-1499-01A	Plasma_cells	0.09085963
TCGA-13-1499-01A	T_cells_CD8	0.158672142
TCGA-13-1499-01A	T_cells_CD4_naive	0
TCGA-13-1499-01A	T_cells_CD4_memory_resting	0.105076539
TCGA-13-1499-01A	T_cells_CD4_memory_activated	0.000787326

TCGA-13-1499-01A	T_cells_follicular_helper	0.008992965
TCGA-13-1499-01A	T_cells_regulatory_(Tregs)	0.121668837
TCGA-13-1499-01A	T_cells_gamma_delta	0
TCGA-13-1499-01A	NK_cells_resting	0
TCGA-13-1499-01A	NK_cells_activated	0.10694423
TCGA-13-1499-01A	Monocytes	0.035461763
TCGA-13-1499-01A	Macrophages_M0	0.062695716
TCGA-13-1499-01A	Macrophages_M1	0.105531881
TCGA-13-1499-01A	Macrophages_M2	0.074160314
TCGA-13-1499-01A	Dendritic_cells_resting	0.00014468
TCGA-13-1499-01A	Dendritic_cells_activated	0
TCGA-13-1499-01A	Mast_cells_resting	0.052534889
TCGA-13-1499-01A	Mast_cells_activated	0
TCGA-13-1499-01A	Eosinophils	0
TCGA-13-1499-01A	Neutrophils	0.000642451
TCGA-13-1501-01A	B_cells_naive	0.084540305
TCGA-13-1501-01A	B_cells_memory	0
TCGA-13-1501-01A	Plasma_cells	0.089443638
TCGA-13-1501-01A	T_cells_CD8	0.056740607
TCGA-13-1501-01A	T_cells_CD4_naive	0
TCGA-13-1501-01A	T_cells_CD4_memory_resting	0.227717833
TCGA-13-1501-01A	T_cells_CD4_memory_activated	0
TCGA-13-1501-01A	T_cells_follicular_helper	0.012397075
TCGA-13-1501-01A	T_cells_regulatory_(Tregs)	0.06990214
TCGA-13-1501-01A	T_cells_gamma_delta	0
TCGA-13-1501-01A	NK_cells_resting	0
TCGA-13-1501-01A	NK_cells_activated	0.037743006
TCGA-13-1501-01A	Monocytes	0.024090382
TCGA-13-1501-01A	Macrophages_M0	0.19199535
TCGA-13-1501-01A	Macrophages_M1	0.053651472
TCGA-13-1501-01A	Macrophages_M2	0.113727235
TCGA-13-1501-01A	Dendritic_cells_resting	0.005209832
TCGA-13-1501-01A	Dendritic_cells_activated	0.019429043

TCGA-13-1501-01A	Mast_cells_resting	0.013412082
TCGA-13-1501-01A	Mast_cells_activated	0
TCGA-13-1501-01A	Eosinophils	0
TCGA-13-1501-01A	Neutrophils	0
TCGA-13-1505-01A	B_cells_naive	0.036339561
TCGA-13-1505-01A	B_cells_memory	0
TCGA-13-1505-01A	Plasma_cells	0.200413001
TCGA-13-1505-01A	T_cells_CD8	0.108692804
TCGA-13-1505-01A	T_cells_CD4_naive	0
TCGA-13-1505-01A	T_cells_CD4_memory_resting	0.204693067
TCGA-13-1505-01A	T_cells_CD4_memory_activated	0
TCGA-13-1505-01A	T_cells_follicular_helper	0.05799246
TCGA-13-1505-01A	T_cells_regulatory_(Tregs)	0.039443913
TCGA-13-1505-01A	T_cells_gamma_delta	0
TCGA-13-1505-01A	NK_cells_resting	0
TCGA-13-1505-01A	NK_cells_activated	0.048154018
TCGA-13-1505-01A	Monocytes	0.053136876
TCGA-13-1505-01A	Macrophages_M0	0.057465386
TCGA-13-1505-01A	Macrophages_M1	0.074255858
TCGA-13-1505-01A	Macrophages_M2	0.081265334
TCGA-13-1505-01A	Dendritic_cells_resting	0.008355794
TCGA-13-1505-01A	Dendritic_cells_activated	0.001001591
TCGA-13-1505-01A	Mast_cells_resting	0.028401993
TCGA-13-1505-01A	Mast_cells_activated	0
TCGA-13-1505-01A	Eosinophils	0
TCGA-13-1505-01A	Neutrophils	0.000388346
TCGA-13-1506-01A	B_cells_naive	0.099927176
TCGA-13-1506-01A	B_cells_memory	0
TCGA-13-1506-01A	Plasma_cells	0.032622868
TCGA-13-1506-01A	T_cells_CD8	0.039747085
TCGA-13-1506-01A	T_cells_CD4_naive	0
TCGA-13-1506-01A	T_cells_CD4_memory_resting	0.138012025
TCGA-13-1506-01A	T_cells_CD4_memory_activated	0

TCGA-13-1506-01A	T_cells_follicular_helper	0.003609473
TCGA-13-1506-01A	T_cells_regulatory_(Tregs)	0.055086626
TCGA-13-1506-01A	T_cells_gamma_delta	0
TCGA-13-1506-01A	NK_cells_resting	0
TCGA-13-1506-01A	NK_cells_activated	0.064715714
TCGA-13-1506-01A	Monocytes	0.022382348
TCGA-13-1506-01A	Macrophages_M0	0.119471091
TCGA-13-1506-01A	Macrophages_M1	0.029250733
TCGA-13-1506-01A	Macrophages_M2	0.060670036
TCGA-13-1506-01A	Dendritic_cells_resting	0
TCGA-13-1506-01A	Dendritic_cells_activated	0.253047052
TCGA-13-1506-01A	Mast_cells_resting	0
TCGA-13-1506-01A	Mast_cells_activated	0.066068373
TCGA-13-1506-01A	Eosinophils	0
TCGA-13-1506-01A	Neutrophils	0.015389398
TCGA-13-1507-01A	B_cells_naive	0.016579335
TCGA-13-1507-01A	B_cells_memory	0
TCGA-13-1507-01A	Plasma_cells	0.190064577
TCGA-13-1507-01A	T_cells_CD8	0
TCGA-13-1507-01A	T_cells_CD4_naive	0
TCGA-13-1507-01A	T_cells_CD4_memory_resting	0.076195449
TCGA-13-1507-01A	T_cells_CD4_memory_activated	0.121862004
TCGA-13-1507-01A	T_cells_follicular_helper	0.026720387
TCGA-13-1507-01A	T_cells_regulatory_(Tregs)	0
TCGA-13-1507-01A	T_cells_gamma_delta	0.046183633
TCGA-13-1507-01A	NK_cells_resting	0
TCGA-13-1507-01A	NK_cells_activated	0.069046435
TCGA-13-1507-01A	Monocytes	0.011301306
TCGA-13-1507-01A	Macrophages_M0	0.109706663
TCGA-13-1507-01A	Macrophages_M1	0.143756524
TCGA-13-1507-01A	Macrophages_M2	0.137290653
TCGA-13-1507-01A	Dendritic_cells_resting	0.004262062
TCGA-13-1507-01A	Dendritic_cells_activated	0

TCGA-13-1507-01A	Mast_cells_resting	0
TCGA-13-1507-01A	Mast_cells_activated	0.029127193
TCGA-13-1507-01A	Eosinophils	0.011211358
TCGA-13-1507-01A	Neutrophils	0.006692421
TCGA-13-1509-01A	B_cells_naive	0
TCGA-13-1509-01A	B_cells_memory	0
TCGA-13-1509-01A	Plasma_cells	0.331048478
TCGA-13-1509-01A	T_cells_CD8	0.035091777
TCGA-13-1509-01A	T_cells_CD4_naive	0
TCGA-13-1509-01A	T_cells_CD4_memory_resting	0.153454225
TCGA-13-1509-01A	T_cells_CD4_memory_activated	0
TCGA-13-1509-01A	T_cells_follicular_helper	0.040220128
TCGA-13-1509-01A	T_cells_regulatory_(Tregs)	0.014987681
TCGA-13-1509-01A	T_cells_gamma_delta	0.016563663
TCGA-13-1509-01A	NK_cells_resting	0
TCGA-13-1509-01A	NK_cells_activated	0.030591198
TCGA-13-1509-01A	Monocytes	0.048498087
TCGA-13-1509-01A	Macrophages_M0	0.076719862
TCGA-13-1509-01A	Macrophages_M1	0.100734935
TCGA-13-1509-01A	Macrophages_M2	0.130079486
TCGA-13-1509-01A	Dendritic_cells_resting	0.004899681
TCGA-13-1509-01A	Dendritic_cells_activated	0
TCGA-13-1509-01A	Mast_cells_resting	0
TCGA-13-1509-01A	Mast_cells_activated	0.007664202
TCGA-13-1509-01A	Eosinophils	0.009446597
TCGA-13-1509-01A	Neutrophils	0
TCGA-13-1510-01A	B_cells_naive	0.064782483
TCGA-13-1510-01A	B_cells_memory	0
TCGA-13-1510-01A	Plasma_cells	0.004551202
TCGA-13-1510-01A	T_cells_CD8	0.10115335
TCGA-13-1510-01A	T_cells_CD4_naive	0
TCGA-13-1510-01A	T_cells_CD4_memory_resting	0.228538108
TCGA-13-1510-01A	T_cells_CD4_memory_activated	0

TCGA-13-1510-01A	T_cells_follicular_helper	0.035103944
TCGA-13-1510-01A	T_cells_regulatory_(Tregs)	0.108275578
TCGA-13-1510-01A	T_cells_gamma_delta	0
TCGA-13-1510-01A	NK_cells_resting	0
TCGA-13-1510-01A	NK_cells_activated	0.060442673
TCGA-13-1510-01A	Monocytes	0.017587241
TCGA-13-1510-01A	Macrophages_M0	0.104546858
TCGA-13-1510-01A	Macrophages_M1	0.107763501
TCGA-13-1510-01A	Macrophages_M2	0.060108671
TCGA-13-1510-01A	Dendritic_cells_resting	0
TCGA-13-1510-01A	Dendritic_cells_activated	0.076616411
TCGA-13-1510-01A	Mast_cells_resting	0.030529981
TCGA-13-1510-01A	Mast_cells_activated	0
TCGA-13-1510-01A	Eosinophils	0
TCGA-13-1510-01A	Neutrophils	0
TCGA-13-1512-01A	B_cells_naive	0
TCGA-13-1512-01A	B_cells_memory	0.042852107
TCGA-13-1512-01A	Plasma_cells	0.022370124
TCGA-13-1512-01A	T_cells_CD8	0.081820306
TCGA-13-1512-01A	T_cells_CD4_naive	0
TCGA-13-1512-01A	T_cells_CD4_memory_resting	0.041759463
TCGA-13-1512-01A	T_cells_CD4_memory_activated	0
TCGA-13-1512-01A	T_cells_follicular_helper	0.018738596
TCGA-13-1512-01A	T_cells_regulatory_(Tregs)	0.067776553
TCGA-13-1512-01A	T_cells_gamma_delta	0
TCGA-13-1512-01A	NK_cells_resting	0
TCGA-13-1512-01A	NK_cells_activated	0.039839272
TCGA-13-1512-01A	Monocytes	0
TCGA-13-1512-01A	Macrophages_M0	0.36855671
TCGA-13-1512-01A	Macrophages_M1	0.172893179
TCGA-13-1512-01A	Macrophages_M2	0.115122611
TCGA-13-1512-01A	Dendritic_cells_resting	0.016547596
TCGA-13-1512-01A	Dendritic_cells_activated	0

TCGA-13-1512-01A	Mast_cells_resting	0.011723482
TCGA-13-1512-01A	Mast_cells_activated	0
TCGA-13-1512-01A	Eosinophils	0
TCGA-13-1512-01A	Neutrophils	0
TCGA-13-2060-01A	B_cells_naive	0.067572888
TCGA-13-2060-01A	B_cells_memory	0
TCGA-13-2060-01A	Plasma_cells	0.120265355
TCGA-13-2060-01A	T_cells_CD8	0.112470145
TCGA-13-2060-01A	T_cells_CD4_naive	0
TCGA-13-2060-01A	T_cells_CD4_memory_resting	0.182818765
TCGA-13-2060-01A	T_cells_CD4_memory_activated	0.097407055
TCGA-13-2060-01A	T_cells_follicular_helper	0.033230224
TCGA-13-2060-01A	T_cells_regulatory_(Tregs)	0.015637247
TCGA-13-2060-01A	T_cells_gamma_delta	0.013125049
TCGA-13-2060-01A	NK_cells_resting	0
TCGA-13-2060-01A	NK_cells_activated	0.01302197
TCGA-13-2060-01A	Monocytes	0.008841607
TCGA-13-2060-01A	Macrophages_M0	0
TCGA-13-2060-01A	Macrophages_M1	0.145214495
TCGA-13-2060-01A	Macrophages_M2	0.16538612
TCGA-13-2060-01A	Dendritic_cells_resting	0
TCGA-13-2060-01A	Dendritic_cells_activated	0.001290569
TCGA-13-2060-01A	Mast_cells_resting	0.023718512
TCGA-13-2060-01A	Mast_cells_activated	0
TCGA-13-2060-01A	Eosinophils	0
TCGA-13-2060-01A	Neutrophils	0
TCGA-13-A5FT-01A	B_cells_naive	0.041901551
TCGA-13-A5FT-01A	B_cells_memory	0
TCGA-13-A5FT-01A	Plasma_cells	0.088565569

TCGA-13-A5FT-01A	T_cells_CD8	0.005583865
TCGA-13-A5FT-01A	T_cells_CD4_naive	0
TCGA-13-A5FT-01A	T_cells_CD4_memory_resting	0.157208117
TCGA-13-A5FT-01A	T_cells_CD4_memory_activated	0
TCGA-13-A5FT-01A	T_cells_follicular_helper	0.09907174
TCGA-13-A5FT-01A	T_cells_regulatory_(Tregs)	0.068741797
TCGA-13-A5FT-01A	T_cells_gamma_delta	0
TCGA-13-A5FT-01A	NK_cells_resting	0
TCGA-13-A5FT-01A	NK_cells_activated	0.041679245
TCGA-13-A5FT-01A	Monocytes	0.0127951
TCGA-13-A5FT-01A	Macrophages_M0	0.135487298
TCGA-13-A5FT-01A	Macrophages_M1	0.064622525
TCGA-13-A5FT-01A	Macrophages_M2	0.048432161
TCGA-13-A5FT-01A	Dendritic_cells_resting	0
TCGA-13-A5FT-01A	Dendritic_cells_activated	0.235911034
TCGA-13-A5FT-01A	Mast_cells_resting	0

TCGA-13-A5FT-01A	Mast_cells_activated	0
TCGA-13-A5FT-01A	Eosinophils	0
TCGA-13-A5FT-01A	Neutrophils	0
TCGA-20-0991-01A	B_cells_naive	0
TCGA-20-0991-01A	B_cells_memory	0.069091926
TCGA-20-0991-01A	Plasma_cells	0.112958894
TCGA-20-0991-01A	T_cells_CD8	0.158499906
TCGA-20-0991-01A	T_cells_CD4_naive	0
TCGA-20-0991-01A	T_cells_CD4_memory_resting	0.027975498
TCGA-20-0991-01A	T_cells_CD4_memory_activated	0.059507736
TCGA-20-0991-01A	T_cells_follicular_helper	0.01351052
TCGA-20-0991-01A	T_cells_regulatory_(Tregs)	0.048451434
TCGA-20-0991-01A	T_cells_gamma_delta	0
TCGA-20-0991-01A	NK_cells_resting	0
TCGA-20-0991-01A	NK_cells_activated	0.062070238
TCGA-20-0991-01A	Monocytes	0.011328398
TCGA-20-0991-01A	Macrophages_M0	0.163058924
TCGA-20-0991-01A	Macrophages_M1	0.064878615
TCGA-20-0991-01A	Macrophages_M2	0.179700879
TCGA-20-0991-01A	Dendritic_cells_resting	0.013621183
TCGA-20-0991-01A	Dendritic_cells_activated	0
TCGA-20-0991-01A	Mast_cells_resting	0
TCGA-20-0991-01A	Mast_cells_activated	0
TCGA-20-0991-01A	Eosinophils	0
TCGA-20-0991-01A	Neutrophils	0.015345848
TCGA-20-1682-01A	B_cells_naive	0.074198633
TCGA-20-1682-01A	B_cells_memory	0
TCGA-20-1682-01A	Plasma_cells	0.070034872
TCGA-20-1682-01A	T_cells_CD8	0.160199783
TCGA-20-1682-01A	T_cells_CD4_naive	0

TCGA-20-1682-01A	T_cells_CD4_memory_resting	0.193580624
TCGA-20-1682-01A	T_cells_CD4_memory_activated	0.10564223
TCGA-20-1682-01A	T_cells_follicular_helper	0.007795692
TCGA-20-1682-01A	T_cells_regulatory_(Tregs)	0.029153388
TCGA-20-1682-01A	T_cells_gamma_delta	0
TCGA-20-1682-01A	NK_cells_resting	0
TCGA-20-1682-01A	NK_cells_activated	0.020969995
TCGA-20-1682-01A	Monocytes	0.069582854
TCGA-20-1682-01A	Macrophages_M0	0.067337492
TCGA-20-1682-01A	Macrophages_M1	0.061943452
TCGA-20-1682-01A	Macrophages_M2	0.135032966
TCGA-20-1682-01A	Dendritic_cells_resting	0
TCGA-20-1682-01A	Dendritic_cells_activated	0.00232539
TCGA-20-1682-01A	Mast_cells_resting	0.002202629
TCGA-20-1682-01A	Mast_cells_activated	0
TCGA-20-1682-01A	Eosinophils	0
TCGA-20-1682-01A	Neutrophils	0
TCGA-20-1686-01A	B_cells_naive	0.007855875
TCGA-20-1686-01A	B_cells_memory	0
TCGA-20-1686-01A	Plasma_cells	0.227553493
TCGA-20-1686-01A	T_cells_CD8	0
TCGA-20-1686-01A	T_cells_CD4_naive	0
TCGA-20-1686-01A	T_cells_CD4_memory_resting	0.172645803
TCGA-20-1686-01A	T_cells_CD4_memory_activated	0
TCGA-20-1686-01A	T_cells_follicular_helper	0
TCGA-20-1686-01A	T_cells_regulatory_(Tregs)	0.055968961
TCGA-20-1686-01A	T_cells_gamma_delta	0.00525368
TCGA-20-1686-01A	NK_cells_resting	0.054827842
TCGA-20-1686-01A	NK_cells_activated	0
TCGA-20-1686-01A	Monocytes	0.035608346
TCGA-20-1686-01A	Macrophages_M0	0.138213969
TCGA-20-1686-01A	Macrophages_M1	0.072262193
TCGA-20-1686-01A	Macrophages_M2	0.135040853

TCGA-20-1686-01A	Dendritic_cells_resting	0
TCGA-20-1686-01A	Dendritic_cells_activated	0.014465726
TCGA-20-1686-01A	Mast_cells_resting	0.080303257
TCGA-20-1686-01A	Mast_cells_activated	0
TCGA-20-1686-01A	Eosinophils	0
TCGA-20-1686-01A	Neutrophils	0
TCGA-23-1022-01A	B_cells_naive	0
TCGA-23-1022-01A	B_cells_memory	0
TCGA-23-1022-01A	Plasma_cells	0
TCGA-23-1022-01A	T_cells_CD8	0
TCGA-23-1022-01A	T_cells_CD4_naive	0
TCGA-23-1022-01A	T_cells_CD4_memory_resting	0.152858595
TCGA-23-1022-01A	T_cells_CD4_memory_activated	0
TCGA-23-1022-01A	T_cells_follicular_helper	0.001739867
TCGA-23-1022-01A	T_cells_regulatory_(Tregs)	0.115153312
TCGA-23-1022-01A	T_cells_gamma_delta	0
TCGA-23-1022-01A	NK_cells_resting	0
TCGA-23-1022-01A	NK_cells_activated	0.095680506
TCGA-23-1022-01A	Monocytes	0
TCGA-23-1022-01A	Macrophages_M0	0.277999495
TCGA-23-1022-01A	Macrophages_M1	0.054791107
TCGA-23-1022-01A	Macrophages_M2	0.188544625
TCGA-23-1022-01A	Dendritic_cells_resting	0.011462304
TCGA-23-1022-01A	Dendritic_cells_activated	0
TCGA-23-1022-01A	Mast_cells_resting	0.101613522
TCGA-23-1022-01A	Mast_cells_activated	0
TCGA-23-1022-01A	Eosinophils	0
TCGA-23-1022-01A	Neutrophils	0.000156667
TCGA-23-1023-01A	B_cells_naive	0
TCGA-23-1023-01A	B_cells_memory	0
TCGA-23-1023-01A	Plasma_cells	0.089206038
TCGA-23-1023-01A	T_cells_CD8	0.112727065
TCGA-23-1023-01A	T_cells_CD4_naive	0

TCGA-23-1023-01A	T_cells_CD4_memory_resting	0.097180126
TCGA-23-1023-01A	T_cells_CD4_memory_activated	0
TCGA-23-1023-01A	T_cells_follicular_helper	0.006906057
TCGA-23-1023-01A	T_cells_regulatory_(Tregs)	0.040717346
TCGA-23-1023-01A	T_cells_gamma_delta	0
TCGA-23-1023-01A	NK_cells_resting	0
TCGA-23-1023-01A	NK_cells_activated	0.070848133
TCGA-23-1023-01A	Monocytes	0.072069109
TCGA-23-1023-01A	Macrophages_M0	0.105608052
TCGA-23-1023-01A	Macrophages_M1	0.132386772
TCGA-23-1023-01A	Macrophages_M2	0.152210343
TCGA-23-1023-01A	Dendritic_cells_resting	0.001776963
TCGA-23-1023-01A	Dendritic_cells_activated	0
TCGA-23-1023-01A	Mast_cells_resting	0.111158177
TCGA-23-1023-01A	Mast_cells_activated	0
TCGA-23-1023-01A	Eosinophils	0
TCGA-23-1023-01A	Neutrophils	0.007205818
TCGA-23-1027-01A	B_cells_naive	0
TCGA-23-1027-01A	B_cells_memory	0.015334144
TCGA-23-1027-01A	Plasma_cells	0
TCGA-23-1027-01A	T_cells_CD8	0.000945491
TCGA-23-1027-01A	T_cells_CD4_naive	0
TCGA-23-1027-01A	T_cells_CD4_memory_resting	0.149385938
TCGA-23-1027-01A	T_cells_CD4_memory_activated	0
TCGA-23-1027-01A	T_cells_follicular_helper	0.012339749
TCGA-23-1027-01A	T_cells_regulatory_(Tregs)	0.03043665
TCGA-23-1027-01A	T_cells_gamma_delta	0
TCGA-23-1027-01A	NK_cells_resting	0.006525728
TCGA-23-1027-01A	NK_cells_activated	0.002876906
TCGA-23-1027-01A	Monocytes	0
TCGA-23-1027-01A	Macrophages_M0	0.496973166
TCGA-23-1027-01A	Macrophages_M1	0.054318206
TCGA-23-1027-01A	Macrophages_M2	0.174006881

TCGA-23-1027-01A	Dendritic_cells_resting	0
TCGA-23-1027-01A	Dendritic_cells_activated	0
TCGA-23-1027-01A	Mast_cells_resting	0
TCGA-23-1027-01A	Mast_cells_activated	0.056857141
TCGA-23-1027-01A	Eosinophils	0
TCGA-23-1027-01A	Neutrophils	0
TCGA-23-1028-01A	B_cells_naive	0.019841782
TCGA-23-1028-01A	B_cells_memory	0
TCGA-23-1028-01A	Plasma_cells	0.019228332
TCGA-23-1028-01A	T_cells_CD8	0.050835819
TCGA-23-1028-01A	T_cells_CD4_naive	0
TCGA-23-1028-01A	T_cells_CD4_memory_resting	0.244773825
TCGA-23-1028-01A	T_cells_CD4_memory_activated	0.040599567
TCGA-23-1028-01A	T_cells_follicular_helper	0.008554264
TCGA-23-1028-01A	T_cells_regulatory_(Tregs)	0.060967757
TCGA-23-1028-01A	T_cells_gamma_delta	0
TCGA-23-1028-01A	NK_cells_resting	0
TCGA-23-1028-01A	NK_cells_activated	0.090445351
TCGA-23-1028-01A	Monocytes	0.01428822
TCGA-23-1028-01A	Macrophages_M0	0.169973779
TCGA-23-1028-01A	Macrophages_M1	0.19323663
TCGA-23-1028-01A	Macrophages_M2	0.016456693
TCGA-23-1028-01A	Dendritic_cells_resting	0
TCGA-23-1028-01A	Dendritic_cells_activated	0.04648387
TCGA-23-1028-01A	Mast_cells_resting	0
TCGA-23-1028-01A	Mast_cells_activated	0.024314111
TCGA-23-1028-01A	Eosinophils	0
TCGA-23-1028-01A	Neutrophils	0
TCGA-23-1030-01A	B_cells_naive	0.032646376
TCGA-23-1030-01A	B_cells_memory	0
TCGA-23-1030-01A	Plasma_cells	0
TCGA-23-1030-01A	T_cells_CD8	0.032834101
TCGA-23-1030-01A	T_cells_CD4_naive	0

TCGA-23-1030-01A	T_cells_CD4_memory_resting	0.168050091
TCGA-23-1030-01A	T_cells_CD4_memory_activated	0
TCGA-23-1030-01A	T_cells_follicular_helper	0
TCGA-23-1030-01A	T_cells_regulatory_(Tregs)	0.082863152
TCGA-23-1030-01A	T_cells_gamma_delta	0
TCGA-23-1030-01A	NK_cells_resting	0.00208593
TCGA-23-1030-01A	NK_cells_activated	0.028507741
TCGA-23-1030-01A	Monocytes	0.010673246
TCGA-23-1030-01A	Macrophages_M0	0.453601334
TCGA-23-1030-01A	Macrophages_M1	0.076252025
TCGA-23-1030-01A	Macrophages_M2	0.110183925
TCGA-23-1030-01A	Dendritic_cells_resting	0
TCGA-23-1030-01A	Dendritic_cells_activated	0
TCGA-23-1030-01A	Mast_cells_resting	0.002302079
TCGA-23-1030-01A	Mast_cells_activated	0
TCGA-23-1030-01A	Eosinophils	0
TCGA-23-1030-01A	Neutrophils	0
TCGA-23-1109-01A	B_cells_naive	0.014460291
TCGA-23-1109-01A	B_cells_memory	0.023440396
TCGA-23-1109-01A	Plasma_cells	0.01228163
TCGA-23-1109-01A	T_cells_CD8	0.060852897
TCGA-23-1109-01A	T_cells_CD4_naive	0
TCGA-23-1109-01A	T_cells_CD4_memory_resting	0.103586879
TCGA-23-1109-01A	T_cells_CD4_memory_activated	0
TCGA-23-1109-01A	T_cells_follicular_helper	0.021412204
TCGA-23-1109-01A	T_cells_regulatory_(Tregs)	0.046267218
TCGA-23-1109-01A	T_cells_gamma_delta	0
TCGA-23-1109-01A	NK_cells_resting	0
TCGA-23-1109-01A	NK_cells_activated	0.042973836
TCGA-23-1109-01A	Monocytes	0.011844116
TCGA-23-1109-01A	Macrophages_M0	0.328326348
TCGA-23-1109-01A	Macrophages_M1	0.04381332
TCGA-23-1109-01A	Macrophages_M2	0.176751068

TCGA-23-1109-01A	Dendritic_cells_resting	0.113786265
TCGA-23-1109-01A	Dendritic_cells_activated	0
TCGA-23-1109-01A	Mast_cells_resting	0
TCGA-23-1109-01A	Mast_cells_activated	0.000203531
TCGA-23-1109-01A	Eosinophils	0
TCGA-23-1109-01A	Neutrophils	0
TCGA-23-1118-01A	B_cells_naive	0
TCGA-23-1118-01A	B_cells_memory	0.022624911
TCGA-23-1118-01A	Plasma_cells	0
TCGA-23-1118-01A	T_cells_CD8	0.076304964
TCGA-23-1118-01A	T_cells_CD4_naive	0
TCGA-23-1118-01A	T_cells_CD4_memory_resting	0.155943834
TCGA-23-1118-01A	T_cells_CD4_memory_activated	0
TCGA-23-1118-01A	T_cells_follicular_helper	0.015653408
TCGA-23-1118-01A	T_cells_regulatory_(Tregs)	0.036076838
TCGA-23-1118-01A	T_cells_gamma_delta	0
TCGA-23-1118-01A	NK_cells_resting	0
TCGA-23-1118-01A	NK_cells_activated	0.051611162
TCGA-23-1118-01A	Monocytes	0.006715642
TCGA-23-1118-01A	Macrophages_M0	0.185993586
TCGA-23-1118-01A	Macrophages_M1	0.133932679
TCGA-23-1118-01A	Macrophages_M2	0.137286868
TCGA-23-1118-01A	Dendritic_cells_resting	0
TCGA-23-1118-01A	Dendritic_cells_activated	0.147065174
TCGA-23-1118-01A	Mast_cells_resting	0.030790933
TCGA-23-1118-01A	Mast_cells_activated	0
TCGA-23-1118-01A	Eosinophils	0
TCGA-23-1118-01A	Neutrophils	0
TCGA-23-1119-01A	B_cells_naive	0.03209434
TCGA-23-1119-01A	B_cells_memory	0
TCGA-23-1119-01A	Plasma_cells	0.192799209
TCGA-23-1119-01A	T_cells_CD8	0.0715574
TCGA-23-1119-01A	T_cells_CD4_naive	0

TCGA-23-1119-01A	T_cells_CD4_memory_resting	0.159604667
TCGA-23-1119-01A	T_cells_CD4_memory_activated	0.011168751
TCGA-23-1119-01A	T_cells_follicular_helper	0.011509131
TCGA-23-1119-01A	T_cells_regulatory_(Tregs)	0.011199525
TCGA-23-1119-01A	T_cells_gamma_delta	0.016921686
TCGA-23-1119-01A	NK_cells_resting	0
TCGA-23-1119-01A	NK_cells_activated	0.091003084
TCGA-23-1119-01A	Monocytes	0.004853171
TCGA-23-1119-01A	Macrophages_M0	0.011842685
TCGA-23-1119-01A	Macrophages_M1	0.110392758
TCGA-23-1119-01A	Macrophages_M2	0.229240084
TCGA-23-1119-01A	Dendritic_cells_resting	0.011570817
TCGA-23-1119-01A	Dendritic_cells_activated	0
TCGA-23-1119-01A	Mast_cells_resting	0.031425995
TCGA-23-1119-01A	Mast_cells_activated	0
TCGA-23-1119-01A	Eosinophils	0
TCGA-23-1119-01A	Neutrophils	0.002816699
TCGA-23-1120-01A	B_cells_naive	0
TCGA-23-1120-01A	B_cells_memory	0.005423351
TCGA-23-1120-01A	Plasma_cells	0
TCGA-23-1120-01A	T_cells_CD8	0.025190509
TCGA-23-1120-01A	T_cells_CD4_naive	0
TCGA-23-1120-01A	T_cells_CD4_memory_resting	0.2171685
TCGA-23-1120-01A	T_cells_CD4_memory_activated	0
TCGA-23-1120-01A	T_cells_follicular_helper	0.042447272
TCGA-23-1120-01A	T_cells_regulatory_(Tregs)	0.07388517
TCGA-23-1120-01A	T_cells_gamma_delta	0
TCGA-23-1120-01A	NK_cells_resting	0
TCGA-23-1120-01A	NK_cells_activated	0.066910576
TCGA-23-1120-01A	Monocytes	0.015372636
TCGA-23-1120-01A	Macrophages_M0	0.18456414
TCGA-23-1120-01A	Macrophages_M1	0.179279539
TCGA-23-1120-01A	Macrophages_M2	0.145036996

TCGA-23-1120-01A	Dendritic_cells_resting	0
TCGA-23-1120-01A	Dendritic_cells_activated	0
TCGA-23-1120-01A	Mast_cells_resting	0.044627796
TCGA-23-1120-01A	Mast_cells_activated	0
TCGA-23-1120-01A	Eosinophils	0
TCGA-23-1120-01A	Neutrophils	9.35169E-05
TCGA-23-1122-01A	B_cells_naive	0.031359191
TCGA-23-1122-01A	B_cells_memory	0
TCGA-23-1122-01A	Plasma_cells	0.010183226
TCGA-23-1122-01A	T_cells_CD8	0.008384655
TCGA-23-1122-01A	T_cells_CD4_naive	0
TCGA-23-1122-01A	T_cells_CD4_memory_resting	0.153906612
TCGA-23-1122-01A	T_cells_CD4_memory_activated	0
TCGA-23-1122-01A	T_cells_follicular_helper	0.066789879
TCGA-23-1122-01A	T_cells_regulatory_(Tregs)	0.025648424
TCGA-23-1122-01A	T_cells_gamma_delta	0
TCGA-23-1122-01A	NK_cells_resting	0
TCGA-23-1122-01A	NK_cells_activated	0.085583378
TCGA-23-1122-01A	Monocytes	0.08437092
TCGA-23-1122-01A	Macrophages_M0	0.113612428
TCGA-23-1122-01A	Macrophages_M1	0.08546811
TCGA-23-1122-01A	Macrophages_M2	0.16467374
TCGA-23-1122-01A	Dendritic_cells_resting	0.077842807
TCGA-23-1122-01A	Dendritic_cells_activated	0.092176631
TCGA-23-1122-01A	Mast_cells_resting	0
TCGA-23-1122-01A	Mast_cells_activated	0
TCGA-23-1122-01A	Eosinophils	0
TCGA-23-1122-01A	Neutrophils	0
TCGA-23-1123-01A	B_cells_naive	0.014388398
TCGA-23-1123-01A	B_cells_memory	0
TCGA-23-1123-01A	Plasma_cells	0.029233616
TCGA-23-1123-01A	T_cells_CD8	0.144891507
TCGA-23-1123-01A	T_cells_CD4_naive	0

TCGA-23-1123-01A	T_cells_CD4_memory_resting	0.164521708
TCGA-23-1123-01A	T_cells_CD4_memory_activated	0.008784867
TCGA-23-1123-01A	T_cells_follicular_helper	0.054792466
TCGA-23-1123-01A	T_cells_regulatory_(Tregs)	0.038268736
TCGA-23-1123-01A	T_cells_gamma_delta	0
TCGA-23-1123-01A	NK_cells_resting	0.021317859
TCGA-23-1123-01A	NK_cells_activated	0.002636015
TCGA-23-1123-01A	Monocytes	0.030645176
TCGA-23-1123-01A	Macrophages_M0	0.114052639
TCGA-23-1123-01A	Macrophages_M1	0.119196546
TCGA-23-1123-01A	Macrophages_M2	0.231643396
TCGA-23-1123-01A	Dendritic_cells_resting	0
TCGA-23-1123-01A	Dendritic_cells_activated	0.010610765
TCGA-23-1123-01A	Mast_cells_resting	0.014246896
TCGA-23-1123-01A	Mast_cells_activated	0
TCGA-23-1123-01A	Eosinophils	0
TCGA-23-1123-01A	Neutrophils	0.000769411
TCGA-23-2077-01A	B_cells_naive	0
TCGA-23-2077-01A	B_cells_memory	0
TCGA-23-2077-01A	Plasma_cells	0
TCGA-23-2077-01A	T_cells_CD8	0.082727406
TCGA-23-2077-01A	T_cells_CD4_naive	0
TCGA-23-2077-01A	T_cells_CD4_memory_resting	0.122353709
TCGA-23-2077-01A	T_cells_CD4_memory_activated	0
TCGA-23-2077-01A	T_cells_follicular_helper	0.013712465
TCGA-23-2077-01A	T_cells_regulatory_(Tregs)	0.105494098
TCGA-23-2077-01A	T_cells_gamma_delta	0
TCGA-23-2077-01A	NK_cells_resting	0
TCGA-23-2077-01A	NK_cells_activated	0
TCGA-23-2077-01A	Monocytes	0.017046154
TCGA-23-2077-01A	Macrophages_M0	0.204460341
TCGA-23-2077-01A	Macrophages_M1	0.073942011
TCGA-23-2077-01A	Macrophages_M2	0.316144457

TCGA-23-2077-01A	Dendritic_cells_resting	0.012134547
TCGA-23-2077-01A	Dendritic_cells_activated	0
TCGA-23-2077-01A	Mast_cells_resting	0.051984812
TCGA-23-2077-01A	Mast_cells_activated	0
TCGA-23-2077-01A	Eosinophils	0
TCGA-23-2077-01A	Neutrophils	0
TCGA-23-2078-01A	B_cells_naive	0.120272358
TCGA-23-2078-01A	B_cells_memory	0
TCGA-23-2078-01A	Plasma_cells	0.037910095
TCGA-23-2078-01A	T_cells_CD8	0.135470735
TCGA-23-2078-01A	T_cells_CD4_naive	0
TCGA-23-2078-01A	T_cells_CD4_memory_resting	0.202221488
TCGA-23-2078-01A	T_cells_CD4_memory_activated	0
TCGA-23-2078-01A	T_cells_follicular_helper	0.008221153
TCGA-23-2078-01A	T_cells_regulatory_(Tregs)	0.130363355
TCGA-23-2078-01A	T_cells_gamma_delta	0
TCGA-23-2078-01A	NK_cells_resting	0
TCGA-23-2078-01A	NK_cells_activated	0.026886003
TCGA-23-2078-01A	Monocytes	0.018474446
TCGA-23-2078-01A	Macrophages_M0	0.032803562
TCGA-23-2078-01A	Macrophages_M1	0.150524432
TCGA-23-2078-01A	Macrophages_M2	0.116460769
TCGA-23-2078-01A	Dendritic_cells_resting	0.005338742
TCGA-23-2078-01A	Dendritic_cells_activated	0
TCGA-23-2078-01A	Mast_cells_resting	0.015052863
TCGA-23-2078-01A	Mast_cells_activated	0
TCGA-23-2078-01A	Eosinophils	0
TCGA-23-2078-01A	Neutrophils	0
TCGA-24-0966-01A	B_cells_naive	0
TCGA-24-0966-01A	B_cells_memory	0.004660336
TCGA-24-0966-01A	Plasma_cells	0.058015439
TCGA-24-0966-01A	T_cells_CD8	0.164132887
TCGA-24-0966-01A	T_cells_CD4_naive	0

TCGA-24-0966-01A	T_cells_CD4_memory_resting	0.116443478
TCGA-24-0966-01A	T_cells_CD4_memory_activated	0
TCGA-24-0966-01A	T_cells_follicular_helper	0.006993636
TCGA-24-0966-01A	T_cells_regulatory_(Tregs)	0.093668187
TCGA-24-0966-01A	T_cells_gamma_delta	0
TCGA-24-0966-01A	NK_cells_resting	0
TCGA-24-0966-01A	NK_cells_activated	0.079343052
TCGA-24-0966-01A	Monocytes	0.046332479
TCGA-24-0966-01A	Macrophages_M0	0
TCGA-24-0966-01A	Macrophages_M1	0.02411954
TCGA-24-0966-01A	Macrophages_M2	0.277842416
TCGA-24-0966-01A	Dendritic_cells_resting	0
TCGA-24-0966-01A	Dendritic_cells_activated	0.126987746
TCGA-24-0966-01A	Mast_cells_resting	0
TCGA-24-0966-01A	Mast_cells_activated	0.000906945
TCGA-24-0966-01A	Eosinophils	0
TCGA-24-0966-01A	Neutrophils	0.000553861
TCGA-24-0982-01A	B_cells_naive	0.088198357
TCGA-24-0982-01A	B_cells_memory	0
TCGA-24-0982-01A	Plasma_cells	0.242519449
TCGA-24-0982-01A	T_cells_CD8	0.042407142
TCGA-24-0982-01A	T_cells_CD4_naive	0
TCGA-24-0982-01A	T_cells_CD4_memory_resting	0.211190177
TCGA-24-0982-01A	T_cells_CD4_memory_activated	0
TCGA-24-0982-01A	T_cells_follicular_helper	0.023508834
TCGA-24-0982-01A	T_cells_regulatory_(Tregs)	0.05553956
TCGA-24-0982-01A	T_cells_gamma_delta	0
TCGA-24-0982-01A	NK_cells_resting	0
TCGA-24-0982-01A	NK_cells_activated	0.066161793
TCGA-24-0982-01A	Monocytes	0.00572913
TCGA-24-0982-01A	Macrophages_M0	0.144507069
TCGA-24-0982-01A	Macrophages_M1	0.054387968
TCGA-24-0982-01A	Macrophages_M2	0.062385149

TCGA-24-0982-01A	Dendritic_cells_resting	0
TCGA-24-0982-01A	Dendritic_cells_activated	0.003465373
TCGA-24-0982-01A	Mast_cells_resting	0
TCGA-24-0982-01A	Mast_cells_activated	0
TCGA-24-0982-01A	Eosinophils	0
TCGA-24-0982-01A	Neutrophils	0
TCGA-24-1103-01A	B_cells_naive	0.004200257
TCGA-24-1103-01A	B_cells_memory	0
TCGA-24-1103-01A	Plasma_cells	0.03856764
TCGA-24-1103-01A	T_cells_CD8	0.025054378
TCGA-24-1103-01A	T_cells_CD4_naive	0
TCGA-24-1103-01A	T_cells_CD4_memory_resting	0.215668261
TCGA-24-1103-01A	T_cells_CD4_memory_activated	0
TCGA-24-1103-01A	T_cells_follicular_helper	0.116365034
TCGA-24-1103-01A	T_cells_regulatory_(Tregs)	0.025171192
TCGA-24-1103-01A	T_cells_gamma_delta	0.006422848
TCGA-24-1103-01A	NK_cells_resting	0
TCGA-24-1103-01A	NK_cells_activated	0.092823298
TCGA-24-1103-01A	Monocytes	0.033153806
TCGA-24-1103-01A	Macrophages_M0	0.155221286
TCGA-24-1103-01A	Macrophages_M1	0.083591335
TCGA-24-1103-01A	Macrophages_M2	0
TCGA-24-1103-01A	Dendritic_cells_resting	0
TCGA-24-1103-01A	Dendritic_cells_activated	0.146283869
TCGA-24-1103-01A	Mast_cells_resting	0.054363376
TCGA-24-1103-01A	Mast_cells_activated	0
TCGA-24-1103-01A	Eosinophils	0.003023829
TCGA-24-1103-01A	Neutrophils	8.95889E-05
TCGA-24-1413-01A	B_cells_naive	0
TCGA-24-1413-01A	B_cells_memory	0
TCGA-24-1413-01A	Plasma_cells	0.072497618
TCGA-24-1413-01A	T_cells_CD8	0
TCGA-24-1413-01A	T_cells_CD4_naive	0

TCGA-24-1413-01A	T_cells_CD4_memory_resting	0.126450331
TCGA-24-1413-01A	T_cells_CD4_memory_activated	0
TCGA-24-1413-01A	T_cells_follicular_helper	0.113815641
TCGA-24-1413-01A	T_cells_regulatory_(Tregs)	0.020407407
TCGA-24-1413-01A	T_cells_gamma_delta	0.004078531
TCGA-24-1413-01A	NK_cells_resting	0
TCGA-24-1413-01A	NK_cells_activated	0.046284066
TCGA-24-1413-01A	Monocytes	0.016021561
TCGA-24-1413-01A	Macrophages_M0	0.165114616
TCGA-24-1413-01A	Macrophages_M1	0.042676002
TCGA-24-1413-01A	Macrophages_M2	0.165102101
TCGA-24-1413-01A	Dendritic_cells_resting	0
TCGA-24-1413-01A	Dendritic_cells_activated	0.198018675
TCGA-24-1413-01A	Mast_cells_resting	0
TCGA-24-1413-01A	Mast_cells_activated	0
TCGA-24-1413-01A	Eosinophils	0.015111639
TCGA-24-1413-01A	Neutrophils	0.014421812
TCGA-24-1416-01A	B_cells_naive	0.015641305
TCGA-24-1416-01A	B_cells_memory	0
TCGA-24-1416-01A	Plasma_cells	0.039879611
TCGA-24-1416-01A	T_cells_CD8	0.005030011
TCGA-24-1416-01A	T_cells_CD4_naive	0
TCGA-24-1416-01A	T_cells_CD4_memory_resting	0.236758852
TCGA-24-1416-01A	T_cells_CD4_memory_activated	0
TCGA-24-1416-01A	T_cells_follicular_helper	0.061466339
TCGA-24-1416-01A	T_cells_regulatory_(Tregs)	0.033277614
TCGA-24-1416-01A	T_cells_gamma_delta	0
TCGA-24-1416-01A	NK_cells_resting	0
TCGA-24-1416-01A	NK_cells_activated	0.100086323
TCGA-24-1416-01A	Monocytes	0.091212446
TCGA-24-1416-01A	Macrophages_M0	0
TCGA-24-1416-01A	Macrophages_M1	0.004984656
TCGA-24-1416-01A	Macrophages_M2	0.148385016

TCGA-24-1416-01A	Dendritic_cells_resting	0
TCGA-24-1416-01A	Dendritic_cells_activated	0.134067119
TCGA-24-1416-01A	Mast_cells_resting	0
TCGA-24-1416-01A	Mast_cells_activated	0.124405976
TCGA-24-1416-01A	Eosinophils	6.45782E-06
TCGA-24-1416-01A	Neutrophils	0.004798274
TCGA-24-1417-01A	B_cells_naive	0
TCGA-24-1417-01A	B_cells_memory	0.020600992
TCGA-24-1417-01A	Plasma_cells	0.017170154
TCGA-24-1417-01A	T_cells_CD8	0
TCGA-24-1417-01A	T_cells_CD4_naive	0
TCGA-24-1417-01A	T_cells_CD4_memory_resting	0.251539746
TCGA-24-1417-01A	T_cells_CD4_memory_activated	0
TCGA-24-1417-01A	T_cells_follicular_helper	0.021939304
TCGA-24-1417-01A	T_cells_regulatory_(Tregs)	0.030800488
TCGA-24-1417-01A	T_cells_gamma_delta	0.002650112
TCGA-24-1417-01A	NK_cells_resting	0
TCGA-24-1417-01A	NK_cells_activated	0.0572631
TCGA-24-1417-01A	Monocytes	0
TCGA-24-1417-01A	Macrophages_M0	0.196081514
TCGA-24-1417-01A	Macrophages_M1	0.085789627
TCGA-24-1417-01A	Macrophages_M2	0.223897018
TCGA-24-1417-01A	Dendritic_cells_resting	0
TCGA-24-1417-01A	Dendritic_cells_activated	0.046638719
TCGA-24-1417-01A	Mast_cells_resting	0.045629226
TCGA-24-1417-01A	Mast_cells_activated	0
TCGA-24-1417-01A	Eosinophils	0
TCGA-24-1417-01A	Neutrophils	0
TCGA-24-1418-01A	B_cells_naive	0.081468825
TCGA-24-1418-01A	B_cells_memory	0
TCGA-24-1418-01A	Plasma_cells	0.027475067
TCGA-24-1418-01A	T_cells_CD8	0.030809854
TCGA-24-1418-01A	T_cells_CD4_naive	0

TCGA-24-1418-01A	T_cells_CD4_memory_resting	0.34334961
TCGA-24-1418-01A	T_cells_CD4_memory_activated	0
TCGA-24-1418-01A	T_cells_follicular_helper	0.011708547
TCGA-24-1418-01A	T_cells_regulatory_(Tregs)	0.062788608
TCGA-24-1418-01A	T_cells_gamma_delta	0
TCGA-24-1418-01A	NK_cells_resting	0
TCGA-24-1418-01A	NK_cells_activated	0.006883866
TCGA-24-1418-01A	Monocytes	0.066215294
TCGA-24-1418-01A	Macrophages_M0	0.085637357
TCGA-24-1418-01A	Macrophages_M1	0.085147754
TCGA-24-1418-01A	Macrophages_M2	0.138207028
TCGA-24-1418-01A	Dendritic_cells_resting	0
TCGA-24-1418-01A	Dendritic_cells_activated	0.053805356
TCGA-24-1418-01A	Mast_cells_resting	0
TCGA-24-1418-01A	Mast_cells_activated	0.006502836
TCGA-24-1418-01A	Eosinophils	0
TCGA-24-1418-01A	Neutrophils	0
TCGA-24-1419-01A	B_cells_naive	0.04821022
TCGA-24-1419-01A	B_cells_memory	0
TCGA-24-1419-01A	Plasma_cells	0.050471902
TCGA-24-1419-01A	T_cells_CD8	0
TCGA-24-1419-01A	T_cells_CD4_naive	0
TCGA-24-1419-01A	T_cells_CD4_memory_resting	0.166325099
TCGA-24-1419-01A	T_cells_CD4_memory_activated	0
TCGA-24-1419-01A	T_cells_follicular_helper	0.027194286
TCGA-24-1419-01A	T_cells_regulatory_(Tregs)	0.094566599
TCGA-24-1419-01A	T_cells_gamma_delta	0
TCGA-24-1419-01A	NK_cells_resting	0
TCGA-24-1419-01A	NK_cells_activated	0.017270394
TCGA-24-1419-01A	Monocytes	0.143586757
TCGA-24-1419-01A	Macrophages_M0	0.111140205
TCGA-24-1419-01A	Macrophages_M1	0
TCGA-24-1419-01A	Macrophages_M2	0.23056545

TCGA-24-1419-01A	Dendritic_cells_resting	0
TCGA-24-1419-01A	Dendritic_cells_activated	0.075740179
TCGA-24-1419-01A	Mast_cells_resting	0
TCGA-24-1419-01A	Mast_cells_activated	0.03492891
TCGA-24-1419-01A	Eosinophils	0
TCGA-24-1419-01A	Neutrophils	0
TCGA-24-1423-01A	B_cells_naive	0.040503047
TCGA-24-1423-01A	B_cells_memory	0
TCGA-24-1423-01A	Plasma_cells	0.000974321
TCGA-24-1423-01A	T_cells_CD8	0.075464141
TCGA-24-1423-01A	T_cells_CD4_naive	0
TCGA-24-1423-01A	T_cells_CD4_memory_resting	0.192869977
TCGA-24-1423-01A	T_cells_CD4_memory_activated	0
TCGA-24-1423-01A	T_cells_follicular_helper	0.073186763
TCGA-24-1423-01A	T_cells_regulatory_(Tregs)	0.06031456
TCGA-24-1423-01A	T_cells_gamma_delta	0
TCGA-24-1423-01A	NK_cells_resting	0
TCGA-24-1423-01A	NK_cells_activated	0.095399212
TCGA-24-1423-01A	Monocytes	0.017102279
TCGA-24-1423-01A	Macrophages_M0	0.156914571
TCGA-24-1423-01A	Macrophages_M1	0.147387628
TCGA-24-1423-01A	Macrophages_M2	0.12830774
TCGA-24-1423-01A	Dendritic_cells_resting	0
TCGA-24-1423-01A	Dendritic_cells_activated	0.01157576
TCGA-24-1423-01A	Mast_cells_resting	0
TCGA-24-1423-01A	Mast_cells_activated	0
TCGA-24-1423-01A	Eosinophils	0
TCGA-24-1423-01A	Neutrophils	0
TCGA-24-1428-01A	B_cells_naive	0
TCGA-24-1428-01A	B_cells_memory	0.003381762
TCGA-24-1428-01A	Plasma_cells	0.06157187
TCGA-24-1428-01A	T_cells_CD8	0.026536179
TCGA-24-1428-01A	T_cells_CD4_naive	0

TCGA-24-1428-01A	T_cells_CD4_memory_resting	0.096490299
TCGA-24-1428-01A	T_cells_CD4_memory_activated	0
TCGA-24-1428-01A	T_cells_follicular_helper	0.017273831
TCGA-24-1428-01A	T_cells_regulatory_(Tregs)	0.084635616
TCGA-24-1428-01A	T_cells_gamma_delta	0
TCGA-24-1428-01A	NK_cells_resting	0
TCGA-24-1428-01A	NK_cells_activated	0.047119534
TCGA-24-1428-01A	Monocytes	0.015398257
TCGA-24-1428-01A	Macrophages_M0	0.407997307
TCGA-24-1428-01A	Macrophages_M1	0.07576952
TCGA-24-1428-01A	Macrophages_M2	0.14596492
TCGA-24-1428-01A	Dendritic_cells_resting	0
TCGA-24-1428-01A	Dendritic_cells_activated	0.017860905
TCGA-24-1428-01A	Mast_cells_resting	0
TCGA-24-1428-01A	Mast_cells_activated	0
TCGA-24-1428-01A	Eosinophils	0
TCGA-24-1428-01A	Neutrophils	0
TCGA-24-1464-01A	B_cells_naive	0
TCGA-24-1464-01A	B_cells_memory	0
TCGA-24-1464-01A	Plasma_cells	0.013466219
TCGA-24-1464-01A	T_cells_CD8	0.322414071
TCGA-24-1464-01A	T_cells_CD4_naive	0
TCGA-24-1464-01A	T_cells_CD4_memory_resting	0.107840167
TCGA-24-1464-01A	T_cells_CD4_memory_activated	0
TCGA-24-1464-01A	T_cells_follicular_helper	0.045965996
TCGA-24-1464-01A	T_cells_regulatory_(Tregs)	0.111871541
TCGA-24-1464-01A	T_cells_gamma_delta	0
TCGA-24-1464-01A	NK_cells_resting	0
TCGA-24-1464-01A	NK_cells_activated	0.117442745
TCGA-24-1464-01A	Monocytes	0.028416048
TCGA-24-1464-01A	Macrophages_M0	0
TCGA-24-1464-01A	Macrophages_M1	0.069356974
TCGA-24-1464-01A	Macrophages_M2	0.18322624

TCGA-24-1464-01A	Dendritic_cells_resting	0
TCGA-24-1464-01A	Dendritic_cells_activated	0
TCGA-24-1464-01A	Mast_cells_resting	0
TCGA-24-1464-01A	Mast_cells_activated	0
TCGA-24-1464-01A	Eosinophils	0
TCGA-24-1464-01A	Neutrophils	0
TCGA-24-1467-01A	B_cells_naive	0
TCGA-24-1467-01A	B_cells_memory	0
TCGA-24-1467-01A	Plasma_cells	0
TCGA-24-1467-01A	T_cells_CD8	0.057456228
TCGA-24-1467-01A	T_cells_CD4_naive	0
TCGA-24-1467-01A	T_cells_CD4_memory_resting	0.056881036
TCGA-24-1467-01A	T_cells_CD4_memory_activated	0
TCGA-24-1467-01A	T_cells_follicular_helper	0.05239485
TCGA-24-1467-01A	T_cells_regulatory_(Tregs)	0.09315599
TCGA-24-1467-01A	T_cells_gamma_delta	0.016985025
TCGA-24-1467-01A	NK_cells_resting	0
TCGA-24-1467-01A	NK_cells_activated	0.031260839
TCGA-24-1467-01A	Monocytes	0.03229493
TCGA-24-1467-01A	Macrophages_M0	0.189963255
TCGA-24-1467-01A	Macrophages_M1	0.108806588
TCGA-24-1467-01A	Macrophages_M2	0.19749067
TCGA-24-1467-01A	Dendritic_cells_resting	0
TCGA-24-1467-01A	Dendritic_cells_activated	0
TCGA-24-1467-01A	Mast_cells_resting	0.16331059
TCGA-24-1467-01A	Mast_cells_activated	0
TCGA-24-1467-01A	Eosinophils	0
TCGA-24-1467-01A	Neutrophils	0
TCGA-24-1469-01A	B_cells_naive	0.040479464
TCGA-24-1469-01A	B_cells_memory	0
TCGA-24-1469-01A	Plasma_cells	0.073040334
TCGA-24-1469-01A	T_cells_CD8	0.013712625
TCGA-24-1469-01A	T_cells_CD4_naive	0

TCGA-24-1469-01A	T_cells_CD4_memory_resting	0.14744279
TCGA-24-1469-01A	T_cells_CD4_memory_activated	0
TCGA-24-1469-01A	T_cells_follicular_helper	0.060149985
TCGA-24-1469-01A	T_cells_regulatory_(Tregs)	0.071033415
TCGA-24-1469-01A	T_cells_gamma_delta	0
TCGA-24-1469-01A	NK_cells_resting	0
TCGA-24-1469-01A	NK_cells_activated	0.04057892
TCGA-24-1469-01A	Monocytes	0
TCGA-24-1469-01A	Macrophages_M0	0.345615927
TCGA-24-1469-01A	Macrophages_M1	0.083787248
TCGA-24-1469-01A	Macrophages_M2	0.124159293
TCGA-24-1469-01A	Dendritic_cells_resting	0
TCGA-24-1469-01A	Dendritic_cells_activated	0
TCGA-24-1469-01A	Mast_cells_resting	0
TCGA-24-1469-01A	Mast_cells_activated	0
TCGA-24-1469-01A	Eosinophils	0
TCGA-24-1469-01A	Neutrophils	0
TCGA-24-1470-01A	B_cells_naive	0.048000195
TCGA-24-1470-01A	B_cells_memory	0
TCGA-24-1470-01A	Plasma_cells	0.025536625
TCGA-24-1470-01A	T_cells_CD8	0.115253284
TCGA-24-1470-01A	T_cells_CD4_naive	0
TCGA-24-1470-01A	T_cells_CD4_memory_resting	0.192402593
TCGA-24-1470-01A	T_cells_CD4_memory_activated	0.012104105
TCGA-24-1470-01A	T_cells_follicular_helper	0.067113072
TCGA-24-1470-01A	T_cells_regulatory_(Tregs)	0.102390855
TCGA-24-1470-01A	T_cells_gamma_delta	0
TCGA-24-1470-01A	NK_cells_resting	0.025429681
TCGA-24-1470-01A	NK_cells_activated	0
TCGA-24-1470-01A	Monocytes	0.024124748
TCGA-24-1470-01A	Macrophages_M0	0.06981732
TCGA-24-1470-01A	Macrophages_M1	0.082939075
TCGA-24-1470-01A	Macrophages_M2	0.168857979

TCGA-24-1470-01A	Dendritic_cells_resting	0.039983936
TCGA-24-1470-01A	Dendritic_cells_activated	0.024016622
TCGA-24-1470-01A	Mast_cells_resting	0.002029909
TCGA-24-1470-01A	Mast_cells_activated	0
TCGA-24-1470-01A	Eosinophils	0
TCGA-24-1470-01A	Neutrophils	0
TCGA-24-1471-01A	B_cells_naive	0.025750106
TCGA-24-1471-01A	B_cells_memory	0
TCGA-24-1471-01A	Plasma_cells	0.004453964
TCGA-24-1471-01A	T_cells_CD8	0.055343522
TCGA-24-1471-01A	T_cells_CD4_naive	0
TCGA-24-1471-01A	T_cells_CD4_memory_resting	0.24143478
TCGA-24-1471-01A	T_cells_CD4_memory_activated	0
TCGA-24-1471-01A	T_cells_follicular_helper	0.032386784
TCGA-24-1471-01A	T_cells_regulatory_(Tregs)	0.027225421
TCGA-24-1471-01A	T_cells_gamma_delta	0
TCGA-24-1471-01A	NK_cells_resting	0
TCGA-24-1471-01A	NK_cells_activated	0.05176864
TCGA-24-1471-01A	Monocytes	0.254192022
TCGA-24-1471-01A	Macrophages_M0	0
TCGA-24-1471-01A	Macrophages_M1	0.087533752
TCGA-24-1471-01A	Macrophages_M2	0.170169421
TCGA-24-1471-01A	Dendritic_cells_resting	0.008508936
TCGA-24-1471-01A	Dendritic_cells_activated	0.002266898
TCGA-24-1471-01A	Mast_cells_resting	0.038965753
TCGA-24-1471-01A	Mast_cells_activated	0
TCGA-24-1471-01A	Eosinophils	0
TCGA-24-1471-01A	Neutrophils	0
TCGA-24-1544-01A	B_cells_naive	0.020734974
TCGA-24-1544-01A	B_cells_memory	0
TCGA-24-1544-01A	Plasma_cells	0.050052989
TCGA-24-1544-01A	T_cells_CD8	0.321664335
TCGA-24-1544-01A	T_cells_CD4_naive	0

TCGA-24-1544-01A	T_cells_CD4_memory_resting	0
TCGA-24-1544-01A	T_cells_CD4_memory_activated	0.012070549
TCGA-24-1544-01A	T_cells_follicular_helper	0.044555152
TCGA-24-1544-01A	T_cells_regulatory_(Tregs)	0.012455518
TCGA-24-1544-01A	T_cells_gamma_delta	0
TCGA-24-1544-01A	NK_cells_resting	0
TCGA-24-1544-01A	NK_cells_activated	0.035464623
TCGA-24-1544-01A	Monocytes	0.034180553
TCGA-24-1544-01A	Macrophages_M0	0.33063492
TCGA-24-1544-01A	Macrophages_M1	0.021100676
TCGA-24-1544-01A	Macrophages_M2	0.085523528
TCGA-24-1544-01A	Dendritic_cells_resting	0
TCGA-24-1544-01A	Dendritic_cells_activated	0.030906167
TCGA-24-1544-01A	Mast_cells_resting	0
TCGA-24-1544-01A	Mast_cells_activated	0
TCGA-24-1544-01A	Eosinophils	0
TCGA-24-1544-01A	Neutrophils	0.000656018
TCGA-24-1546-01A	B_cells_naive	0.010433122
TCGA-24-1546-01A	B_cells_memory	0.001081518
TCGA-24-1546-01A	Plasma_cells	0.033572817
TCGA-24-1546-01A	T_cells_CD8	0.01426857
TCGA-24-1546-01A	T_cells_CD4_naive	0
TCGA-24-1546-01A	T_cells_CD4_memory_resting	0.168205097
TCGA-24-1546-01A	T_cells_CD4_memory_activated	0
TCGA-24-1546-01A	T_cells_follicular_helper	0.002041114
TCGA-24-1546-01A	T_cells_regulatory_(Tregs)	0.029233835
TCGA-24-1546-01A	T_cells_gamma_delta	0
TCGA-24-1546-01A	NK_cells_resting	0
TCGA-24-1546-01A	NK_cells_activated	0.012461911
TCGA-24-1546-01A	Monocytes	0.045167805
TCGA-24-1546-01A	Macrophages_M0	0.294808265
TCGA-24-1546-01A	Macrophages_M1	0.053683558
TCGA-24-1546-01A	Macrophages_M2	0.156243391

TCGA-24-1546-01A	Dendritic_cells_resting	0.136499981
TCGA-24-1546-01A	Dendritic_cells_activated	0
TCGA-24-1546-01A	Mast_cells_resting	0.010514166
TCGA-24-1546-01A	Mast_cells_activated	0.020361709
TCGA-24-1546-01A	Eosinophils	0
TCGA-24-1546-01A	Neutrophils	0.011423141
TCGA-24-1549-01A	B_cells_naive	0.019104533
TCGA-24-1549-01A	B_cells_memory	0
TCGA-24-1549-01A	Plasma_cells	0.011922872
TCGA-24-1549-01A	T_cells_CD8	0.08148528
TCGA-24-1549-01A	T_cells_CD4_naive	0
TCGA-24-1549-01A	T_cells_CD4_memory_resting	0.192137741
TCGA-24-1549-01A	T_cells_CD4_memory_activated	0.058590962
TCGA-24-1549-01A	T_cells_follicular_helper	0.044847245
TCGA-24-1549-01A	T_cells_regulatory_(Tregs)	0.061150772
TCGA-24-1549-01A	T_cells_gamma_delta	0.012249051
TCGA-24-1549-01A	NK_cells_resting	0.049610995
TCGA-24-1549-01A	NK_cells_activated	0.013418281
TCGA-24-1549-01A	Monocytes	0
TCGA-24-1549-01A	Macrophages_M0	0.210829726
TCGA-24-1549-01A	Macrophages_M1	0.107606555
TCGA-24-1549-01A	Macrophages_M2	0.056931876
TCGA-24-1549-01A	Dendritic_cells_resting	0
TCGA-24-1549-01A	Dendritic_cells_activated	0.048926857
TCGA-24-1549-01A	Mast_cells_resting	0.031187254
TCGA-24-1549-01A	Mast_cells_activated	0
TCGA-24-1549-01A	Eosinophils	0
TCGA-24-1549-01A	Neutrophils	0
TCGA-24-1550-01A	B_cells_naive	0.067161747
TCGA-24-1550-01A	B_cells_memory	0
TCGA-24-1550-01A	Plasma_cells	0.071671354
TCGA-24-1550-01A	T_cells_CD8	0.034944598
TCGA-24-1550-01A	T_cells_CD4_naive	0

TCGA-24-1550-01A	T_cells_CD4_memory_resting	0.063988106
TCGA-24-1550-01A	T_cells_CD4_memory_activated	0.021333677
TCGA-24-1550-01A	T_cells_follicular_helper	0.025427764
TCGA-24-1550-01A	T_cells_regulatory_(Tregs)	0.020021627
TCGA-24-1550-01A	T_cells_gamma_delta	0
TCGA-24-1550-01A	NK_cells_resting	0
TCGA-24-1550-01A	NK_cells_activated	0.018412993
TCGA-24-1550-01A	Monocytes	0
TCGA-24-1550-01A	Macrophages_M0	0.303680114
TCGA-24-1550-01A	Macrophages_M1	0.03932207
TCGA-24-1550-01A	Macrophages_M2	0.308193802
TCGA-24-1550-01A	Dendritic_cells_resting	0
TCGA-24-1550-01A	Dendritic_cells_activated	0
TCGA-24-1550-01A	Mast_cells_resting	0
TCGA-24-1550-01A	Mast_cells_activated	0.020729963
TCGA-24-1550-01A	Eosinophils	0
TCGA-24-1550-01A	Neutrophils	0.005112185
TCGA-24-1551-01A	B_cells_naive	0
TCGA-24-1551-01A	B_cells_memory	0.001932807
TCGA-24-1551-01A	Plasma_cells	0.00579062
TCGA-24-1551-01A	T_cells_CD8	0.090502794
TCGA-24-1551-01A	T_cells_CD4_naive	0
TCGA-24-1551-01A	T_cells_CD4_memory_resting	0.169508462
TCGA-24-1551-01A	T_cells_CD4_memory_activated	0
TCGA-24-1551-01A	T_cells_follicular_helper	0
TCGA-24-1551-01A	T_cells_regulatory_(Tregs)	0.068405293
TCGA-24-1551-01A	T_cells_gamma_delta	0
TCGA-24-1551-01A	NK_cells_resting	0.000602525
TCGA-24-1551-01A	NK_cells_activated	0.069223929
TCGA-24-1551-01A	Monocytes	0.034467557
TCGA-24-1551-01A	Macrophages_M0	0.322021987
TCGA-24-1551-01A	Macrophages_M1	0.09931835
TCGA-24-1551-01A	Macrophages_M2	0.108913742

TCGA-24-1551-01A	Dendritic_cells_resting	0.006502682
TCGA-24-1551-01A	Dendritic_cells_activated	0
TCGA-24-1551-01A	Mast_cells_resting	0
TCGA-24-1551-01A	Mast_cells_activated	0.011688293
TCGA-24-1551-01A	Eosinophils	0
TCGA-24-1551-01A	Neutrophils	0.01112096
TCGA-24-1558-01A	B_cells_naive	0.006165527
TCGA-24-1558-01A	B_cells_memory	0
TCGA-24-1558-01A	Plasma_cells	0.003783538
TCGA-24-1558-01A	T_cells_CD8	0.018125647
TCGA-24-1558-01A	T_cells_CD4_naive	0
TCGA-24-1558-01A	T_cells_CD4_memory_resting	0.065873462
TCGA-24-1558-01A	T_cells_CD4_memory_activated	0
TCGA-24-1558-01A	T_cells_follicular_helper	0.025246799
TCGA-24-1558-01A	T_cells_regulatory_(Tregs)	0.073802702
TCGA-24-1558-01A	T_cells_gamma_delta	0
TCGA-24-1558-01A	NK_cells_resting	0
TCGA-24-1558-01A	NK_cells_activated	0.047857874
TCGA-24-1558-01A	Monocytes	0
TCGA-24-1558-01A	Macrophages_M0	0.558553529
TCGA-24-1558-01A	Macrophages_M1	0.01503678
TCGA-24-1558-01A	Macrophages_M2	0.171567735
TCGA-24-1558-01A	Dendritic_cells_resting	0
TCGA-24-1558-01A	Dendritic_cells_activated	0
TCGA-24-1558-01A	Mast_cells_resting	0.013986407
TCGA-24-1558-01A	Mast_cells_activated	0
TCGA-24-1558-01A	Eosinophils	0
TCGA-24-1558-01A	Neutrophils	0
TCGA-24-1560-01A	B_cells_naive	0.094123231
TCGA-24-1560-01A	B_cells_memory	0
TCGA-24-1560-01A	Plasma_cells	0.018838046
TCGA-24-1560-01A	T_cells_CD8	0.044497903
TCGA-24-1560-01A	T_cells_CD4_naive	0

TCGA-24-1560-01A	T_cells_CD4_memory_resting	0.25609787
TCGA-24-1560-01A	T_cells_CD4_memory_activated	0
TCGA-24-1560-01A	T_cells_follicular_helper	0.128894998
TCGA-24-1560-01A	T_cells_regulatory_(Tregs)	0
TCGA-24-1560-01A	T_cells_gamma_delta	0
TCGA-24-1560-01A	NK_cells_resting	0
TCGA-24-1560-01A	NK_cells_activated	0.034024263
TCGA-24-1560-01A	Monocytes	0.010587543
TCGA-24-1560-01A	Macrophages_M0	0.111981748
TCGA-24-1560-01A	Macrophages_M1	0.072549922
TCGA-24-1560-01A	Macrophages_M2	0.134826266
TCGA-24-1560-01A	Dendritic_cells_resting	0
TCGA-24-1560-01A	Dendritic_cells_activated	0.09357821
TCGA-24-1560-01A	Mast_cells_resting	0
TCGA-24-1560-01A	Mast_cells_activated	0
TCGA-24-1560-01A	Eosinophils	0
TCGA-24-1560-01A	Neutrophils	0
TCGA-24-1603-01A	B_cells_naive	0
TCGA-24-1603-01A	B_cells_memory	0
TCGA-24-1603-01A	Plasma_cells	0.023132408
TCGA-24-1603-01A	T_cells_CD8	0.016538006
TCGA-24-1603-01A	T_cells_CD4_naive	0
TCGA-24-1603-01A	T_cells_CD4_memory_resting	0.110864736
TCGA-24-1603-01A	T_cells_CD4_memory_activated	0
TCGA-24-1603-01A	T_cells_follicular_helper	0.03837341
TCGA-24-1603-01A	T_cells_regulatory_(Tregs)	0.054476311
TCGA-24-1603-01A	T_cells_gamma_delta	0.031072955
TCGA-24-1603-01A	NK_cells_resting	0
TCGA-24-1603-01A	NK_cells_activated	0.052879688
TCGA-24-1603-01A	Monocytes	0.004076873
TCGA-24-1603-01A	Macrophages_M0	0.313219493
TCGA-24-1603-01A	Macrophages_M1	0.105925274
TCGA-24-1603-01A	Macrophages_M2	0.121361939

TCGA-24-1603-01A	Dendritic_cells_resting	0
TCGA-24-1603-01A	Dendritic_cells_activated	0
TCGA-24-1603-01A	Mast_cells_resting	0.10779401
TCGA-24-1603-01A	Mast_cells_activated	0
TCGA-24-1603-01A	Eosinophils	0
TCGA-24-1603-01A	Neutrophils	0.020284895
TCGA-24-1843-01A	B_cells_naive	0
TCGA-24-1843-01A	B_cells_memory	0.000617631
TCGA-24-1843-01A	Plasma_cells	0.11976662
TCGA-24-1843-01A	T_cells_CD8	0.103773657
TCGA-24-1843-01A	T_cells_CD4_naive	0
TCGA-24-1843-01A	T_cells_CD4_memory_resting	0.125995557
TCGA-24-1843-01A	T_cells_CD4_memory_activated	0
TCGA-24-1843-01A	T_cells_follicular_helper	0
TCGA-24-1843-01A	T_cells_regulatory_(Tregs)	0.038345989
TCGA-24-1843-01A	T_cells_gamma_delta	0
TCGA-24-1843-01A	NK_cells_resting	0
TCGA-24-1843-01A	NK_cells_activated	0.050761506
TCGA-24-1843-01A	Monocytes	0.006358806
TCGA-24-1843-01A	Macrophages_M0	0.33329436
TCGA-24-1843-01A	Macrophages_M1	0.04778391
TCGA-24-1843-01A	Macrophages_M2	0.166565677
TCGA-24-1843-01A	Dendritic_cells_resting	0
TCGA-24-1843-01A	Dendritic_cells_activated	0
TCGA-24-1843-01A	Mast_cells_resting	0
TCGA-24-1843-01A	Mast_cells_activated	0.005316172
TCGA-24-1843-01A	Eosinophils	0
TCGA-24-1843-01A	Neutrophils	0.001420116
TCGA-24-1844-01A	B_cells_naive	0
TCGA-24-1844-01A	B_cells_memory	0
TCGA-24-1844-01A	Plasma_cells	0.084176543
TCGA-24-1844-01A	T_cells_CD8	0
TCGA-24-1844-01A	T_cells_CD4_naive	0

TCGA-24-1844-01A	T_cells_CD4_memory_resting	0.20931706
TCGA-24-1844-01A	T_cells_CD4_memory_activated	0
TCGA-24-1844-01A	T_cells_follicular_helper	0.00445103
TCGA-24-1844-01A	T_cells_regulatory_(Tregs)	0.029427586
TCGA-24-1844-01A	T_cells_gamma_delta	0
TCGA-24-1844-01A	NK_cells_resting	0
TCGA-24-1844-01A	NK_cells_activated	0.103808432
TCGA-24-1844-01A	Monocytes	0.050790607
TCGA-24-1844-01A	Macrophages_M0	0.153958701
TCGA-24-1844-01A	Macrophages_M1	0
TCGA-24-1844-01A	Macrophages_M2	0.146347254
TCGA-24-1844-01A	Dendritic_cells_resting	0.010448582
TCGA-24-1844-01A	Dendritic_cells_activated	0
TCGA-24-1844-01A	Mast_cells_resting	0.2008133
TCGA-24-1844-01A	Mast_cells_activated	0
TCGA-24-1844-01A	Eosinophils	0.006460903
TCGA-24-1844-01A	Neutrophils	0
TCGA-24-1845-01A	B_cells_naive	0
TCGA-24-1845-01A	B_cells_memory	0.000464324
TCGA-24-1845-01A	Plasma_cells	0
TCGA-24-1845-01A	T_cells_CD8	0
TCGA-24-1845-01A	T_cells_CD4_naive	0
TCGA-24-1845-01A	T_cells_CD4_memory_resting	0.15597913
TCGA-24-1845-01A	T_cells_CD4_memory_activated	0
TCGA-24-1845-01A	T_cells_follicular_helper	0.035984275
TCGA-24-1845-01A	T_cells_regulatory_(Tregs)	0.026551938
TCGA-24-1845-01A	T_cells_gamma_delta	0
TCGA-24-1845-01A	NK_cells_resting	0.033656237
TCGA-24-1845-01A	NK_cells_activated	0
TCGA-24-1845-01A	Monocytes	0.045558895
TCGA-24-1845-01A	Macrophages_M0	0.315690061
TCGA-24-1845-01A	Macrophages_M1	0.13149282
TCGA-24-1845-01A	Macrophages_M2	0.131302841

TCGA-24-1845-01A	Dendritic_cells_resting	0
TCGA-24-1845-01A	Dendritic_cells_activated	0.068632053
TCGA-24-1845-01A	Mast_cells_resting	0
TCGA-24-1845-01A	Mast_cells_activated	0.05089471
TCGA-24-1845-01A	Eosinophils	0
TCGA-24-1845-01A	Neutrophils	0.003792717
TCGA-24-1846-01A	B_cells_naive	0.020352515
TCGA-24-1846-01A	B_cells_memory	0
TCGA-24-1846-01A	Plasma_cells	0.003546434
TCGA-24-1846-01A	T_cells_CD8	0.050104098
TCGA-24-1846-01A	T_cells_CD4_naive	0
TCGA-24-1846-01A	T_cells_CD4_memory_resting	0.112124808
TCGA-24-1846-01A	T_cells_CD4_memory_activated	0
TCGA-24-1846-01A	T_cells_follicular_helper	0.010410012
TCGA-24-1846-01A	T_cells_regulatory_(Tregs)	0.093718623
TCGA-24-1846-01A	T_cells_gamma_delta	0
TCGA-24-1846-01A	NK_cells_resting	0
TCGA-24-1846-01A	NK_cells_activated	0.067478092
TCGA-24-1846-01A	Monocytes	0
TCGA-24-1846-01A	Macrophages_M0	0.388733981
TCGA-24-1846-01A	Macrophages_M1	0.089905646
TCGA-24-1846-01A	Macrophages_M2	0.144036366
TCGA-24-1846-01A	Dendritic_cells_resting	0
TCGA-24-1846-01A	Dendritic_cells_activated	0
TCGA-24-1846-01A	Mast_cells_resting	0.019589424
TCGA-24-1846-01A	Mast_cells_activated	0
TCGA-24-1846-01A	Eosinophils	0
TCGA-24-1846-01A	Neutrophils	0
TCGA-24-1924-01A	B_cells_naive	0.039311427
TCGA-24-1924-01A	B_cells_memory	0
TCGA-24-1924-01A	Plasma_cells	0.080485117
TCGA-24-1924-01A	T_cells_CD8	0.166997911
TCGA-24-1924-01A	T_cells_CD4_naive	0

TCGA-24-1924-01A	T_cells_CD4_memory_resting	0.105730219
TCGA-24-1924-01A	T_cells_CD4_memory_activated	0.015539247
TCGA-24-1924-01A	T_cells_follicular_helper	0.083320135
TCGA-24-1924-01A	T_cells_regulatory_(Tregs)	0
TCGA-24-1924-01A	T_cells_gamma_delta	0
TCGA-24-1924-01A	NK_cells_resting	0
TCGA-24-1924-01A	NK_cells_activated	0.038892804
TCGA-24-1924-01A	Monocytes	0.095647517
TCGA-24-1924-01A	Macrophages_M0	0.006434844
TCGA-24-1924-01A	Macrophages_M1	0.153343936
TCGA-24-1924-01A	Macrophages_M2	0.178608437
TCGA-24-1924-01A	Dendritic_cells_resting	0.01260313
TCGA-24-1924-01A	Dendritic_cells_activated	0
TCGA-24-1924-01A	Mast_cells_resting	0
TCGA-24-1924-01A	Mast_cells_activated	0.010637761
TCGA-24-1924-01A	Eosinophils	0
TCGA-24-1924-01A	Neutrophils	0.012447517
TCGA-24-1930-01A	B_cells_naive	0.014094987
TCGA-24-1930-01A	B_cells_memory	0
TCGA-24-1930-01A	Plasma_cells	0.093562952
TCGA-24-1930-01A	T_cells_CD8	0.02119793
TCGA-24-1930-01A	T_cells_CD4_naive	0
TCGA-24-1930-01A	T_cells_CD4_memory_resting	0.226106832
TCGA-24-1930-01A	T_cells_CD4_memory_activated	0
TCGA-24-1930-01A	T_cells_follicular_helper	0.040673366
TCGA-24-1930-01A	T_cells_regulatory_(Tregs)	0.030917883
TCGA-24-1930-01A	T_cells_gamma_delta	0
TCGA-24-1930-01A	NK_cells_resting	0
TCGA-24-1930-01A	NK_cells_activated	0.023652999
TCGA-24-1930-01A	Monocytes	0.025278814
TCGA-24-1930-01A	Macrophages_M0	0.149288007
TCGA-24-1930-01A	Macrophages_M1	0.089370132
TCGA-24-1930-01A	Macrophages_M2	0.272600987

TCGA-24-1930-01A	Dendritic_cells_resting	0
TCGA-24-1930-01A	Dendritic_cells_activated	0
TCGA-24-1930-01A	Mast_cells_resting	0.010287768
TCGA-24-1930-01A	Mast_cells_activated	0
TCGA-24-1930-01A	Eosinophils	0
TCGA-24-1930-01A	Neutrophils	0.002967343
TCGA-24-2020-01A	B_cells_naive	0.07256256
TCGA-24-2020-01A	B_cells_memory	0
TCGA-24-2020-01A	Plasma_cells	0
TCGA-24-2020-01A	T_cells_CD8	0.045095646
TCGA-24-2020-01A	T_cells_CD4_naive	0
TCGA-24-2020-01A	T_cells_CD4_memory_resting	0.066920142
TCGA-24-2020-01A	T_cells_CD4_memory_activated	0
TCGA-24-2020-01A	T_cells_follicular_helper	0.052530215
TCGA-24-2020-01A	T_cells_regulatory_(Tregs)	0.025718242
TCGA-24-2020-01A	T_cells_gamma_delta	0
TCGA-24-2020-01A	NK_cells_resting	0.042864816
TCGA-24-2020-01A	NK_cells_activated	0
TCGA-24-2020-01A	Monocytes	0.01621356
TCGA-24-2020-01A	Macrophages_M0	0.415153981
TCGA-24-2020-01A	Macrophages_M1	0.074504758
TCGA-24-2020-01A	Macrophages_M2	0.180578829
TCGA-24-2020-01A	Dendritic_cells_resting	0
TCGA-24-2020-01A	Dendritic_cells_activated	0
TCGA-24-2020-01A	Mast_cells_resting	0
TCGA-24-2020-01A	Mast_cells_activated	0.007857252
TCGA-24-2020-01A	Eosinophils	0
TCGA-24-2020-01A	Neutrophils	0
TCGA-24-2023-01A	B_cells_naive	0.029903613
TCGA-24-2023-01A	B_cells_memory	0
TCGA-24-2023-01A	Plasma_cells	0.001648321
TCGA-24-2023-01A	T_cells_CD8	0.072764276
TCGA-24-2023-01A	T_cells_CD4_naive	0

TCGA-24-2023-01A	T_cells_CD4_memory_resting	0.227993999
TCGA-24-2023-01A	T_cells_CD4_memory_activated	0
TCGA-24-2023-01A	T_cells_follicular_helper	0.044706613
TCGA-24-2023-01A	T_cells_regulatory_(Tregs)	0.053668889
TCGA-24-2023-01A	T_cells_gamma_delta	0
TCGA-24-2023-01A	NK_cells_resting	0.001018331
TCGA-24-2023-01A	NK_cells_activated	0.100814014
TCGA-24-2023-01A	Monocytes	0.006918632
TCGA-24-2023-01A	Macrophages_M0	0.011422158
TCGA-24-2023-01A	Macrophages_M1	0.114526842
TCGA-24-2023-01A	Macrophages_M2	0.229808566
TCGA-24-2023-01A	Dendritic_cells_resting	0
TCGA-24-2023-01A	Dendritic_cells_activated	0.0901045
TCGA-24-2023-01A	Mast_cells_resting	0.00228841
TCGA-24-2023-01A	Mast_cells_activated	0
TCGA-24-2023-01A	Eosinophils	0
TCGA-24-2023-01A	Neutrophils	0.012412838
TCGA-24-2035-01A	B_cells_naive	0.049245362
TCGA-24-2035-01A	B_cells_memory	0
TCGA-24-2035-01A	Plasma_cells	0.022998321
TCGA-24-2035-01A	T_cells_CD8	0.048553891
TCGA-24-2035-01A	T_cells_CD4_naive	0
TCGA-24-2035-01A	T_cells_CD4_memory_resting	0.183033076
TCGA-24-2035-01A	T_cells_CD4_memory_activated	0.017214312
TCGA-24-2035-01A	T_cells_follicular_helper	0.022134955
TCGA-24-2035-01A	T_cells_regulatory_(Tregs)	0.03187752
TCGA-24-2035-01A	T_cells_gamma_delta	0
TCGA-24-2035-01A	NK_cells_resting	0
TCGA-24-2035-01A	NK_cells_activated	0.042806821
TCGA-24-2035-01A	Monocytes	0.079412857
TCGA-24-2035-01A	Macrophages_M0	0
TCGA-24-2035-01A	Macrophages_M1	0.087394537
TCGA-24-2035-01A	Macrophages_M2	0.314316758

TCGA-24-2035-01A	Dendritic_cells_resting	0.026214887
TCGA-24-2035-01A	Dendritic_cells_activated	0
TCGA-24-2035-01A	Mast_cells_resting	0.050252033
TCGA-24-2035-01A	Mast_cells_activated	0
TCGA-24-2035-01A	Eosinophils	0
TCGA-24-2035-01A	Neutrophils	0.024544671
TCGA-24-2261-01A	B_cells_naive	0.027261424
TCGA-24-2261-01A	B_cells_memory	0
TCGA-24-2261-01A	Plasma_cells	0.020883258
TCGA-24-2261-01A	T_cells_CD8	0.204205808
TCGA-24-2261-01A	T_cells_CD4_naive	0
TCGA-24-2261-01A	T_cells_CD4_memory_resting	0.162406189
TCGA-24-2261-01A	T_cells_CD4_memory_activated	0.042449479
TCGA-24-2261-01A	T_cells_follicular_helper	0.048019774
TCGA-24-2261-01A	T_cells_regulatory_(Tregs)	0.079551449
TCGA-24-2261-01A	T_cells_gamma_delta	0
TCGA-24-2261-01A	NK_cells_resting	0.00207846
TCGA-24-2261-01A	NK_cells_activated	0.004071331
TCGA-24-2261-01A	Monocytes	0.099691058
TCGA-24-2261-01A	Macrophages_M0	0
TCGA-24-2261-01A	Macrophages_M1	0.0983243
TCGA-24-2261-01A	Macrophages_M2	0.208129487
TCGA-24-2261-01A	Dendritic_cells_resting	0.002927983
TCGA-24-2261-01A	Dendritic_cells_activated	0
TCGA-24-2261-01A	Mast_cells_resting	0
TCGA-24-2261-01A	Mast_cells_activated	0
TCGA-24-2261-01A	Eosinophils	0
TCGA-24-2261-01A	Neutrophils	0
TCGA-24-2271-01A	B_cells_naive	0.07916295
TCGA-24-2271-01A	B_cells_memory	0
TCGA-24-2271-01A	Plasma_cells	0.055344264
TCGA-24-2271-01A	T_cells_CD8	0.082029439
TCGA-24-2271-01A	T_cells_CD4_naive	0

TCGA-24-2271-01A	T_cells_CD4_memory_resting	0.304573879
TCGA-24-2271-01A	T_cells_CD4_memory_activated	0
TCGA-24-2271-01A	T_cells_follicular_helper	0.046793336
TCGA-24-2271-01A	T_cells_regulatory_(Tregs)	0.110359814
TCGA-24-2271-01A	T_cells_gamma_delta	0
TCGA-24-2271-01A	NK_cells_resting	0
TCGA-24-2271-01A	NK_cells_activated	0.059493348
TCGA-24-2271-01A	Monocytes	0.028700611
TCGA-24-2271-01A	Macrophages_M0	0.060034302
TCGA-24-2271-01A	Macrophages_M1	0.115266471
TCGA-24-2271-01A	Macrophages_M2	0.047449305
TCGA-24-2271-01A	Dendritic_cells_resting	0.010792282
TCGA-24-2271-01A	Dendritic_cells_activated	0
TCGA-24-2271-01A	Mast_cells_resting	0
TCGA-24-2271-01A	Mast_cells_activated	0
TCGA-24-2271-01A	Eosinophils	0
TCGA-24-2271-01A	Neutrophils	0
TCGA-24-2281-01A	B_cells_naive	0.020203118
TCGA-24-2281-01A	B_cells_memory	0
TCGA-24-2281-01A	Plasma_cells	0.033480067
TCGA-24-2281-01A	T_cells_CD8	0.075275892
TCGA-24-2281-01A	T_cells_CD4_naive	0
TCGA-24-2281-01A	T_cells_CD4_memory_resting	0.086522678
TCGA-24-2281-01A	T_cells_CD4_memory_activated	0.012118533
TCGA-24-2281-01A	T_cells_follicular_helper	0.030743351
TCGA-24-2281-01A	T_cells_regulatory_(Tregs)	0.074913933
TCGA-24-2281-01A	T_cells_gamma_delta	0
TCGA-24-2281-01A	NK_cells_resting	0
TCGA-24-2281-01A	NK_cells_activated	0.023737401
TCGA-24-2281-01A	Monocytes	0.01778783
TCGA-24-2281-01A	Macrophages_M0	0.377526432
TCGA-24-2281-01A	Macrophages_M1	0.066226002
TCGA-24-2281-01A	Macrophages_M2	0.166282553

TCGA-24-2281-01A	Dendritic_cells_resting	0.007342012
TCGA-24-2281-01A	Dendritic_cells_activated	0
TCGA-24-2281-01A	Mast_cells_resting	0
TCGA-24-2281-01A	Mast_cells_activated	0.007840196
TCGA-24-2281-01A	Eosinophils	0
TCGA-24-2281-01A	Neutrophils	0
TCGA-24-2290-01A	B_cells_naive	0.000371869
TCGA-24-2290-01A	B_cells_memory	9.06511E-05
TCGA-24-2290-01A	Plasma_cells	0
TCGA-24-2290-01A	T_cells_CD8	0.00048068
TCGA-24-2290-01A	T_cells_CD4_naive	0
TCGA-24-2290-01A	T_cells_CD4_memory_resting	0.14147714
TCGA-24-2290-01A	T_cells_CD4_memory_activated	0
TCGA-24-2290-01A	T_cells_follicular_helper	0.034383657
TCGA-24-2290-01A	T_cells_regulatory_(Tregs)	0.052774826
TCGA-24-2290-01A	T_cells_gamma_delta	0
TCGA-24-2290-01A	NK_cells_resting	0.027202289
TCGA-24-2290-01A	NK_cells_activated	0
TCGA-24-2290-01A	Monocytes	0.057962228
TCGA-24-2290-01A	Macrophages_M0	0.199650405
TCGA-24-2290-01A	Macrophages_M1	0.104633985
TCGA-24-2290-01A	Macrophages_M2	0.252048998
TCGA-24-2290-01A	Dendritic_cells_resting	0
TCGA-24-2290-01A	Dendritic_cells_activated	0.042517406
TCGA-24-2290-01A	Mast_cells_resting	0
TCGA-24-2290-01A	Mast_cells_activated	0.077718631
TCGA-24-2290-01A	Eosinophils	0.000265626
TCGA-24-2290-01A	Neutrophils	0.008421609
TCGA-24-2297-01A	B_cells_naive	0.084388228
TCGA-24-2297-01A	B_cells_memory	0
TCGA-24-2297-01A	Plasma_cells	0.0194321
TCGA-24-2297-01A	T_cells_CD8	0.013010253
TCGA-24-2297-01A	T_cells_CD4_naive	0

TCGA-24-2297-01A	T_cells_CD4_memory_resting	0.292051184
TCGA-24-2297-01A	T_cells_CD4_memory_activated	0
TCGA-24-2297-01A	T_cells_follicular_helper	0.035148728
TCGA-24-2297-01A	T_cells_regulatory_(Tregs)	0.056255172
TCGA-24-2297-01A	T_cells_gamma_delta	0
TCGA-24-2297-01A	NK_cells_resting	0
TCGA-24-2297-01A	NK_cells_activated	0.102298037
TCGA-24-2297-01A	Monocytes	0.054708437
TCGA-24-2297-01A	Macrophages_M0	0.121410077
TCGA-24-2297-01A	Macrophages_M1	0.050417272
TCGA-24-2297-01A	Macrophages_M2	0.106990374
TCGA-24-2297-01A	Dendritic_cells_resting	0
TCGA-24-2297-01A	Dendritic_cells_activated	0.063890139
TCGA-24-2297-01A	Mast_cells_resting	0
TCGA-24-2297-01A	Mast_cells_activated	0
TCGA-24-2297-01A	Eosinophils	0
TCGA-24-2297-01A	Neutrophils	0
TCGA-25-1313-01A	B_cells_naive	0
TCGA-25-1313-01A	B_cells_memory	0.036624063
TCGA-25-1313-01A	Plasma_cells	0.018462761
TCGA-25-1313-01A	T_cells_CD8	0
TCGA-25-1313-01A	T_cells_CD4_naive	0
TCGA-25-1313-01A	T_cells_CD4_memory_resting	0.288737685
TCGA-25-1313-01A	T_cells_CD4_memory_activated	0
TCGA-25-1313-01A	T_cells_follicular_helper	0.022208043
TCGA-25-1313-01A	T_cells_regulatory_(Tregs)	0.003628838
TCGA-25-1313-01A	T_cells_gamma_delta	0
TCGA-25-1313-01A	NK_cells_resting	0
TCGA-25-1313-01A	NK_cells_activated	0.06101713
TCGA-25-1313-01A	Monocytes	0.116380315
TCGA-25-1313-01A	Macrophages_M0	0
TCGA-25-1313-01A	Macrophages_M1	0.070000987
TCGA-25-1313-01A	Macrophages_M2	0.287395357

TCGA-25-1313-01A	Dendritic_cells_resting	0
TCGA-25-1313-01A	Dendritic_cells_activated	0.067989201
TCGA-25-1313-01A	Mast_cells_resting	0.012574399
TCGA-25-1313-01A	Mast_cells_activated	0
TCGA-25-1313-01A	Eosinophils	7.98321E-05
TCGA-25-1313-01A	Neutrophils	0.014901389
TCGA-25-1315-01A	B_cells_naive	0
TCGA-25-1315-01A	B_cells_memory	0
TCGA-25-1315-01A	Plasma_cells	0.014190704
TCGA-25-1315-01A	T_cells_CD8	0
TCGA-25-1315-01A	T_cells_CD4_naive	0
TCGA-25-1315-01A	T_cells_CD4_memory_resting	0.299617154
TCGA-25-1315-01A	T_cells_CD4_memory_activated	0
TCGA-25-1315-01A	T_cells_follicular_helper	0.012082864
TCGA-25-1315-01A	T_cells_regulatory_(Tregs)	0.032812902
TCGA-25-1315-01A	T_cells_gamma_delta	0
TCGA-25-1315-01A	NK_cells_resting	0
TCGA-25-1315-01A	NK_cells_activated	0.109280193
TCGA-25-1315-01A	Monocytes	0.023678205
TCGA-25-1315-01A	Macrophages_M0	0.154761179
TCGA-25-1315-01A	Macrophages_M1	0.028855764
TCGA-25-1315-01A	Macrophages_M2	0.045395546
TCGA-25-1315-01A	Dendritic_cells_resting	0
TCGA-25-1315-01A	Dendritic_cells_activated	0.029058978
TCGA-25-1315-01A	Mast_cells_resting	0.237781099
TCGA-25-1315-01A	Mast_cells_activated	0
TCGA-25-1315-01A	Eosinophils	0.002959174
TCGA-25-1315-01A	Neutrophils	0.009526238
TCGA-25-1316-01A	B_cells_naive	0.020290579
TCGA-25-1316-01A	B_cells_memory	0
TCGA-25-1316-01A	Plasma_cells	0
TCGA-25-1316-01A	T_cells_CD8	0.3178036
TCGA-25-1316-01A	T_cells_CD4_naive	0

TCGA-25-1316-01A	T_cells_CD4_memory_resting	0.066258762
TCGA-25-1316-01A	T_cells_CD4_memory_activated	0
TCGA-25-1316-01A	T_cells_follicular_helper	0.022462402
TCGA-25-1316-01A	T_cells_regulatory_(Tregs)	0.115804743
TCGA-25-1316-01A	T_cells_gamma_delta	0
TCGA-25-1316-01A	NK_cells_resting	0
TCGA-25-1316-01A	NK_cells_activated	0.144230841
TCGA-25-1316-01A	Monocytes	0.01934645
TCGA-25-1316-01A	Macrophages_M0	0.052567072
TCGA-25-1316-01A	Macrophages_M1	0.08219385
TCGA-25-1316-01A	Macrophages_M2	0.115719011
TCGA-25-1316-01A	Dendritic_cells_resting	0
TCGA-25-1316-01A	Dendritic_cells_activated	0
TCGA-25-1316-01A	Mast_cells_resting	0.043322689
TCGA-25-1316-01A	Mast_cells_activated	0
TCGA-25-1316-01A	Eosinophils	0
TCGA-25-1316-01A	Neutrophils	0
TCGA-25-1320-01A	B_cells_naive	0.030128696
TCGA-25-1320-01A	B_cells_memory	0
TCGA-25-1320-01A	Plasma_cells	0.014591384
TCGA-25-1320-01A	T_cells_CD8	0.056547103
TCGA-25-1320-01A	T_cells_CD4_naive	0
TCGA-25-1320-01A	T_cells_CD4_memory_resting	0.155656209
TCGA-25-1320-01A	T_cells_CD4_memory_activated	0
TCGA-25-1320-01A	T_cells_follicular_helper	0.011982215
TCGA-25-1320-01A	T_cells_regulatory_(Tregs)	0.044107503
TCGA-25-1320-01A	T_cells_gamma_delta	0
TCGA-25-1320-01A	NK_cells_resting	0
TCGA-25-1320-01A	NK_cells_activated	0.061476683
TCGA-25-1320-01A	Monocytes	0.014827892
TCGA-25-1320-01A	Macrophages_M0	0.221238362
TCGA-25-1320-01A	Macrophages_M1	0.042703034
TCGA-25-1320-01A	Macrophages_M2	0.185188312

TCGA-25-1320-01A	Dendritic_cells_resting	0.074908243
TCGA-25-1320-01A	Dendritic_cells_activated	0.028357447
TCGA-25-1320-01A	Mast_cells_resting	0
TCGA-25-1320-01A	Mast_cells_activated	0.050340477
TCGA-25-1320-01A	Eosinophils	0
TCGA-25-1320-01A	Neutrophils	0.007946439
TCGA-25-1321-01A	B_cells_naive	0.000727706
TCGA-25-1321-01A	B_cells_memory	0
TCGA-25-1321-01A	Plasma_cells	0.02178282
TCGA-25-1321-01A	T_cells_CD8	0
TCGA-25-1321-01A	T_cells_CD4_naive	0
TCGA-25-1321-01A	T_cells_CD4_memory_resting	0.226933603
TCGA-25-1321-01A	T_cells_CD4_memory_activated	0
TCGA-25-1321-01A	T_cells_follicular_helper	0.030212704
TCGA-25-1321-01A	T_cells_regulatory_(Tregs)	0.093671156
TCGA-25-1321-01A	T_cells_gamma_delta	0
TCGA-25-1321-01A	NK_cells_resting	0.001086223
TCGA-25-1321-01A	NK_cells_activated	0.027173989
TCGA-25-1321-01A	Monocytes	0.054383914
TCGA-25-1321-01A	Macrophages_M0	0.238530031
TCGA-25-1321-01A	Macrophages_M1	0.093220162
TCGA-25-1321-01A	Macrophages_M2	0.181721971
TCGA-25-1321-01A	Dendritic_cells_resting	0.003980637
TCGA-25-1321-01A	Dendritic_cells_activated	0.010455127
TCGA-25-1321-01A	Mast_cells_resting	0.003431553
TCGA-25-1321-01A	Mast_cells_activated	0
TCGA-25-1321-01A	Eosinophils	0.003539227
TCGA-25-1321-01A	Neutrophils	0.009149176
TCGA-25-1326-01A	B_cells_naive	0.000151334
TCGA-25-1326-01A	B_cells_memory	0
TCGA-25-1326-01A	Plasma_cells	0.035059157
TCGA-25-1326-01A	T_cells_CD8	0.035015786
TCGA-25-1326-01A	T_cells_CD4_naive	0

TCGA-25-1326-01A	T_cells_CD4_memory_resting	0.236775538
TCGA-25-1326-01A	T_cells_CD4_memory_activated	0.001726266
TCGA-25-1326-01A	T_cells_follicular_helper	0.021148093
TCGA-25-1326-01A	T_cells_regulatory_(Tregs)	0.007423059
TCGA-25-1326-01A	T_cells_gamma_delta	0
TCGA-25-1326-01A	NK_cells_resting	0
TCGA-25-1326-01A	NK_cells_activated	0.039976362
TCGA-25-1326-01A	Monocytes	0.001625585
TCGA-25-1326-01A	Macrophages_M0	0.21829458
TCGA-25-1326-01A	Macrophages_M1	0.129933062
TCGA-25-1326-01A	Macrophages_M2	0.221645729
TCGA-25-1326-01A	Dendritic_cells_resting	0.009456187
TCGA-25-1326-01A	Dendritic_cells_activated	0
TCGA-25-1326-01A	Mast_cells_resting	0.041769263
TCGA-25-1326-01A	Mast_cells_activated	0
TCGA-25-1326-01A	Eosinophils	0
TCGA-25-1326-01A	Neutrophils	0
TCGA-25-1628-01A	B_cells_naive	0.072069052
TCGA-25-1628-01A	B_cells_memory	0
TCGA-25-1628-01A	Plasma_cells	0.039037393
TCGA-25-1628-01A	T_cells_CD8	0.281462264
TCGA-25-1628-01A	T_cells_CD4_naive	0
TCGA-25-1628-01A	T_cells_CD4_memory_resting	0.050775392
TCGA-25-1628-01A	T_cells_CD4_memory_activated	0.037899678
TCGA-25-1628-01A	T_cells_follicular_helper	0.020265034
TCGA-25-1628-01A	T_cells_regulatory_(Tregs)	0.070158872
TCGA-25-1628-01A	T_cells_gamma_delta	0
TCGA-25-1628-01A	NK_cells_resting	0
TCGA-25-1628-01A	NK_cells_activated	0.001623174
TCGA-25-1628-01A	Monocytes	0.006615882
TCGA-25-1628-01A	Macrophages_M0	0.043270914
TCGA-25-1628-01A	Macrophages_M1	0.168747798
TCGA-25-1628-01A	Macrophages_M2	0.154695181

TCGA-25-1628-01A	Dendritic_cells_resting	0.017781068
TCGA-25-1628-01A	Dendritic_cells_activated	0
TCGA-25-1628-01A	Mast_cells_resting	0.035598297
TCGA-25-1628-01A	Mast_cells_activated	0
TCGA-25-1628-01A	Eosinophils	0
TCGA-25-1628-01A	Neutrophils	0
TCGA-25-1632-01A	B_cells_naive	0.168543711
TCGA-25-1632-01A	B_cells_memory	0
TCGA-25-1632-01A	Plasma_cells	0.00247902
TCGA-25-1632-01A	T_cells_CD8	0
TCGA-25-1632-01A	T_cells_CD4_naive	0
TCGA-25-1632-01A	T_cells_CD4_memory_resting	0.152725139
TCGA-25-1632-01A	T_cells_CD4_memory_activated	0
TCGA-25-1632-01A	T_cells_follicular_helper	0.001501051
TCGA-25-1632-01A	T_cells_regulatory_(Tregs)	0.067248295
TCGA-25-1632-01A	T_cells_gamma_delta	0
TCGA-25-1632-01A	NK_cells_resting	0.012894531
TCGA-25-1632-01A	NK_cells_activated	0.007183805
TCGA-25-1632-01A	Monocytes	0.024008184
TCGA-25-1632-01A	Macrophages_M0	0.155748796
TCGA-25-1632-01A	Macrophages_M1	0
TCGA-25-1632-01A	Macrophages_M2	0.163202028
TCGA-25-1632-01A	Dendritic_cells_resting	0
TCGA-25-1632-01A	Dendritic_cells_activated	0.203540393
TCGA-25-1632-01A	Mast_cells_resting	0
TCGA-25-1632-01A	Mast_cells_activated	0.03791247
TCGA-25-1632-01A	Eosinophils	0
TCGA-25-1632-01A	Neutrophils	0.003012578
TCGA-25-2393-01A	B_cells_naive	0.07695953
TCGA-25-2393-01A	B_cells_memory	0
TCGA-25-2393-01A	Plasma_cells	0
TCGA-25-2393-01A	T_cells_CD8	0.074491591
TCGA-25-2393-01A	T_cells_CD4_naive	0

TCGA-25-2393-01A	T_cells_CD4_memory_resting	0.104778011
TCGA-25-2393-01A	T_cells_CD4_memory_activated	0.010121069
TCGA-25-2393-01A	T_cells_follicular_helper	0.013955027
TCGA-25-2393-01A	T_cells_regulatory_(Tregs)	0.015435093
TCGA-25-2393-01A	T_cells_gamma_delta	0
TCGA-25-2393-01A	NK_cells_resting	0.011335445
TCGA-25-2393-01A	NK_cells_activated	0
TCGA-25-2393-01A	Monocytes	0.00254969
TCGA-25-2393-01A	Macrophages_M0	0.393461252
TCGA-25-2393-01A	Macrophages_M1	0.062489222
TCGA-25-2393-01A	Macrophages_M2	0.112601617
TCGA-25-2393-01A	Dendritic_cells_resting	0.004743994
TCGA-25-2393-01A	Dendritic_cells_activated	0
TCGA-25-2393-01A	Mast_cells_resting	0
TCGA-25-2393-01A	Mast_cells_activated	0.068971736
TCGA-25-2393-01A	Eosinophils	0.027212804
TCGA-25-2393-01A	Neutrophils	0.020893921
TCGA-25-2401-01A	B_cells_naive	0.005207319
TCGA-25-2401-01A	B_cells_memory	0.003286966
TCGA-25-2401-01A	Plasma_cells	0
TCGA-25-2401-01A	T_cells_CD8	0.020994937
TCGA-25-2401-01A	T_cells_CD4_naive	0
TCGA-25-2401-01A	T_cells_CD4_memory_resting	0.181567901
TCGA-25-2401-01A	T_cells_CD4_memory_activated	0
TCGA-25-2401-01A	T_cells_follicular_helper	0.045294646
TCGA-25-2401-01A	T_cells_regulatory_(Tregs)	0.037673361
TCGA-25-2401-01A	T_cells_gamma_delta	0
TCGA-25-2401-01A	NK_cells_resting	0
TCGA-25-2401-01A	NK_cells_activated	0.036638007
TCGA-25-2401-01A	Monocytes	0.130010533
TCGA-25-2401-01A	Macrophages_M0	0.111841206
TCGA-25-2401-01A	Macrophages_M1	0.133601694
TCGA-25-2401-01A	Macrophages_M2	0.26915603

TCGA-25-2401-01A	Dendritic_cells_resting	0.002231799
TCGA-25-2401-01A	Dendritic_cells_activated	0.018247354
TCGA-25-2401-01A	Mast_cells_resting	0.003965723
TCGA-25-2401-01A	Mast_cells_activated	0
TCGA-25-2401-01A	Eosinophils	0.000282524
TCGA-25-2401-01A	Neutrophils	0
TCGA-25-2404-01A	B_cells_naive	0.049657367
TCGA-25-2404-01A	B_cells_memory	0
TCGA-25-2404-01A	Plasma_cells	0.014165544
TCGA-25-2404-01A	T_cells_CD8	0.069773599
TCGA-25-2404-01A	T_cells_CD4_naive	0
TCGA-25-2404-01A	T_cells_CD4_memory_resting	0.269783949
TCGA-25-2404-01A	T_cells_CD4_memory_activated	0
TCGA-25-2404-01A	T_cells_follicular_helper	0.062052143
TCGA-25-2404-01A	T_cells_regulatory_(Tregs)	0.004007671
TCGA-25-2404-01A	T_cells_gamma_delta	0
TCGA-25-2404-01A	NK_cells_resting	0
TCGA-25-2404-01A	NK_cells_activated	0.031343264
TCGA-25-2404-01A	Monocytes	0.083483616
TCGA-25-2404-01A	Macrophages_M0	0.090657432
TCGA-25-2404-01A	Macrophages_M1	0.179157479
TCGA-25-2404-01A	Macrophages_M2	0.119869814
TCGA-25-2404-01A	Dendritic_cells_resting	0
TCGA-25-2404-01A	Dendritic_cells_activated	0
TCGA-25-2404-01A	Mast_cells_resting	0.026048123
TCGA-25-2404-01A	Mast_cells_activated	0
TCGA-25-2404-01A	Eosinophils	0
TCGA-25-2404-01A	Neutrophils	0
TCGA-29-1691-01A	B_cells_naive	0.057024276
TCGA-29-1691-01A	B_cells_memory	0
TCGA-29-1691-01A	Plasma_cells	0
TCGA-29-1691-01A	T_cells_CD8	0.112090855
TCGA-29-1691-01A	T_cells_CD4_naive	0

TCGA-29-1691-01A	T_cells_CD4_memory_resting	0.200668487
TCGA-29-1691-01A	T_cells_CD4_memory_activated	0
TCGA-29-1691-01A	T_cells_follicular_helper	0.023367823
TCGA-29-1691-01A	T_cells_regulatory_(Tregs)	0.08787778
TCGA-29-1691-01A	T_cells_gamma_delta	0
TCGA-29-1691-01A	NK_cells_resting	0
TCGA-29-1691-01A	NK_cells_activated	0.050980369
TCGA-29-1691-01A	Monocytes	0.103922395
TCGA-29-1691-01A	Macrophages_M0	0.030633197
TCGA-29-1691-01A	Macrophages_M1	0.076946046
TCGA-29-1691-01A	Macrophages_M2	0.227808563
TCGA-29-1691-01A	Dendritic_cells_resting	0
TCGA-29-1691-01A	Dendritic_cells_activated	0
TCGA-29-1691-01A	Mast_cells_resting	0.02868021
TCGA-29-1691-01A	Mast_cells_activated	0
TCGA-29-1691-01A	Eosinophils	0
TCGA-29-1691-01A	Neutrophils	0
TCGA-29-1693-01A	B_cells_naive	0
TCGA-29-1693-01A	B_cells_memory	0.080893928
TCGA-29-1693-01A	Plasma_cells	0.009170641
TCGA-29-1693-01A	T_cells_CD8	0.039270932
TCGA-29-1693-01A	T_cells_CD4_naive	0
TCGA-29-1693-01A	T_cells_CD4_memory_resting	0.133897078
TCGA-29-1693-01A	T_cells_CD4_memory_activated	0
TCGA-29-1693-01A	T_cells_follicular_helper	0.012918973
TCGA-29-1693-01A	T_cells_regulatory_(Tregs)	0.022568193
TCGA-29-1693-01A	T_cells_gamma_delta	0
TCGA-29-1693-01A	NK_cells_resting	0
TCGA-29-1693-01A	NK_cells_activated	0.113287501
TCGA-29-1693-01A	Monocytes	0.009526602
TCGA-29-1693-01A	Macrophages_M0	0.188723501
TCGA-29-1693-01A	Macrophages_M1	0.137992261
TCGA-29-1693-01A	Macrophages_M2	0.081003031

TCGA-29-1693-01A	Dendritic_cells_resting	0
TCGA-29-1693-01A	Dendritic_cells_activated	0.137222192
TCGA-29-1693-01A	Mast_cells_resting	0.033525167
TCGA-29-1693-01A	Mast_cells_activated	0
TCGA-29-1693-01A	Eosinophils	0
TCGA-29-1693-01A	Neutrophils	0
TCGA-29-1695-01A	B_cells_naive	0
TCGA-29-1695-01A	B_cells_memory	0.003299016
TCGA-29-1695-01A	Plasma_cells	0
TCGA-29-1695-01A	T_cells_CD8	0.01720718
TCGA-29-1695-01A	T_cells_CD4_naive	0
TCGA-29-1695-01A	T_cells_CD4_memory_resting	0.089581379
TCGA-29-1695-01A	T_cells_CD4_memory_activated	0
TCGA-29-1695-01A	T_cells_follicular_helper	0.013484941
TCGA-29-1695-01A	T_cells_regulatory_(Tregs)	0.039028467
TCGA-29-1695-01A	T_cells_gamma_delta	0
TCGA-29-1695-01A	NK_cells_resting	0
TCGA-29-1695-01A	NK_cells_activated	0.035285104
TCGA-29-1695-01A	Monocytes	0
TCGA-29-1695-01A	Macrophages_M0	0.519752646
TCGA-29-1695-01A	Macrophages_M1	0.067890355
TCGA-29-1695-01A	Macrophages_M2	0.197759081
TCGA-29-1695-01A	Dendritic_cells_resting	0
TCGA-29-1695-01A	Dendritic_cells_activated	0
TCGA-29-1695-01A	Mast_cells_resting	0
TCGA-29-1695-01A	Mast_cells_activated	0.016711832
TCGA-29-1695-01A	Eosinophils	0
TCGA-29-1695-01A	Neutrophils	0
TCGA-29-1697-01A	B_cells_naive	0
TCGA-29-1697-01A	B_cells_memory	0.005653979
TCGA-29-1697-01A	Plasma_cells	0.025877056
TCGA-29-1697-01A	T_cells_CD8	0.043311778
TCGA-29-1697-01A	T_cells_CD4_naive	0

TCGA-29-1697-01A	T_cells_CD4_memory_resting	0.052333361
TCGA-29-1697-01A	T_cells_CD4_memory_activated	0
TCGA-29-1697-01A	T_cells_follicular_helper	0.06059565
TCGA-29-1697-01A	T_cells_regulatory_(Tregs)	0.007368459
TCGA-29-1697-01A	T_cells_gamma_delta	0
TCGA-29-1697-01A	NK_cells_resting	0
TCGA-29-1697-01A	NK_cells_activated	0.131843034
TCGA-29-1697-01A	Monocytes	0.002738449
TCGA-29-1697-01A	Macrophages_M0	0.136970686
TCGA-29-1697-01A	Macrophages_M1	0.048279721
TCGA-29-1697-01A	Macrophages_M2	0.130210771
TCGA-29-1697-01A	Dendritic_cells_resting	0
TCGA-29-1697-01A	Dendritic_cells_activated	0.207474115
TCGA-29-1697-01A	Mast_cells_resting	0.14734294
TCGA-29-1697-01A	Mast_cells_activated	0
TCGA-29-1697-01A	Eosinophils	0
TCGA-29-1697-01A	Neutrophils	0
TCGA-29-1701-01A	B_cells_naive	0.032046113
TCGA-29-1701-01A	B_cells_memory	0
TCGA-29-1701-01A	Plasma_cells	0.076292179
TCGA-29-1701-01A	T_cells_CD8	0.157513596
TCGA-29-1701-01A	T_cells_CD4_naive	0
TCGA-29-1701-01A	T_cells_CD4_memory_resting	0.086870857
TCGA-29-1701-01A	T_cells_CD4_memory_activated	0.06416164
TCGA-29-1701-01A	T_cells_follicular_helper	0.040905005
TCGA-29-1701-01A	T_cells_regulatory_(Tregs)	0.031496304
TCGA-29-1701-01A	T_cells_gamma_delta	0.041287999
TCGA-29-1701-01A	NK_cells_resting	0
TCGA-29-1701-01A	NK_cells_activated	0
TCGA-29-1701-01A	Monocytes	0.06453555
TCGA-29-1701-01A	Macrophages_M0	0.041244097
TCGA-29-1701-01A	Macrophages_M1	0.119577413
TCGA-29-1701-01A	Macrophages_M2	0.216988867

TCGA-29-1701-01A	Dendritic_cells_resting	0
TCGA-29-1701-01A	Dendritic_cells_activated	0.003196384
TCGA-29-1701-01A	Mast_cells_resting	0
TCGA-29-1701-01A	Mast_cells_activated	0.014389375
TCGA-29-1701-01A	Eosinophils	0.005799352
TCGA-29-1701-01A	Neutrophils	0.003695269
TCGA-29-1711-01A	B_cells_naive	0.07089936
TCGA-29-1711-01A	B_cells_memory	0
TCGA-29-1711-01A	Plasma_cells	0.0063451
TCGA-29-1711-01A	T_cells_CD8	0.193573907
TCGA-29-1711-01A	T_cells_CD4_naive	0
TCGA-29-1711-01A	T_cells_CD4_memory_resting	0.098802651
TCGA-29-1711-01A	T_cells_CD4_memory_activated	0.001382857
TCGA-29-1711-01A	T_cells_follicular_helper	0.041158997
TCGA-29-1711-01A	T_cells_regulatory_(Tregs)	0.084759342
TCGA-29-1711-01A	T_cells_gamma_delta	0
TCGA-29-1711-01A	NK_cells_resting	0
TCGA-29-1711-01A	NK_cells_activated	0.048260848
TCGA-29-1711-01A	Monocytes	0.002928578
TCGA-29-1711-01A	Macrophages_M0	0.109704372
TCGA-29-1711-01A	Macrophages_M1	0.163616684
TCGA-29-1711-01A	Macrophages_M2	0.14547567
TCGA-29-1711-01A	Dendritic_cells_resting	0.010757
TCGA-29-1711-01A	Dendritic_cells_activated	0
TCGA-29-1711-01A	Mast_cells_resting	0.022334634
TCGA-29-1711-01A	Mast_cells_activated	0
TCGA-29-1711-01A	Eosinophils	0
TCGA-29-1711-01A	Neutrophils	0
TCGA-29-1761-01A	B_cells_naive	0.026221713
TCGA-29-1761-01A	B_cells_memory	0
TCGA-29-1761-01A	Plasma_cells	0.004643087
TCGA-29-1761-01A	T_cells_CD8	0.156255545
TCGA-29-1761-01A	T_cells_CD4_naive	0

TCGA-29-1761-01A	T_cells_CD4_memory_resting	0.093373767
TCGA-29-1761-01A	T_cells_CD4_memory_activated	0
TCGA-29-1761-01A	T_cells_follicular_helper	0.013814876
TCGA-29-1761-01A	T_cells_regulatory_(Tregs)	0.045697681
TCGA-29-1761-01A	T_cells_gamma_delta	0
TCGA-29-1761-01A	NK_cells_resting	0
TCGA-29-1761-01A	NK_cells_activated	0.003516435
TCGA-29-1761-01A	Monocytes	0
TCGA-29-1761-01A	Macrophages_M0	0.286608325
TCGA-29-1761-01A	Macrophages_M1	0.118153546
TCGA-29-1761-01A	Macrophages_M2	0.230248754
TCGA-29-1761-01A	Dendritic_cells_resting	0
TCGA-29-1761-01A	Dendritic_cells_activated	0
TCGA-29-1761-01A	Mast_cells_resting	0.010578333
TCGA-29-1761-01A	Mast_cells_activated	0
TCGA-29-1761-01A	Eosinophils	0
TCGA-29-1761-01A	Neutrophils	0.010887936
TCGA-29-1762-01A	B_cells_naive	0
TCGA-29-1762-01A	B_cells_memory	0.000385694
TCGA-29-1762-01A	Plasma_cells	0
TCGA-29-1762-01A	T_cells_CD8	0
TCGA-29-1762-01A	T_cells_CD4_naive	0
TCGA-29-1762-01A	T_cells_CD4_memory_resting	0.250914948
TCGA-29-1762-01A	T_cells_CD4_memory_activated	0
TCGA-29-1762-01A	T_cells_follicular_helper	0
TCGA-29-1762-01A	T_cells_regulatory_(Tregs)	0.026459361
TCGA-29-1762-01A	T_cells_gamma_delta	0
TCGA-29-1762-01A	NK_cells_resting	0
TCGA-29-1762-01A	NK_cells_activated	0.076183252
TCGA-29-1762-01A	Monocytes	0.187346893
TCGA-29-1762-01A	Macrophages_M0	0.046636606
TCGA-29-1762-01A	Macrophages_M1	0
TCGA-29-1762-01A	Macrophages_M2	0.333304521

TCGA-29-1762-01A	Dendritic_cells_resting	0
TCGA-29-1762-01A	Dendritic_cells_activated	0
TCGA-29-1762-01A	Mast_cells_resting	0
TCGA-29-1762-01A	Mast_cells_activated	0.020243037
TCGA-29-1762-01A	Eosinophils	0.015779497
TCGA-29-1762-01A	Neutrophils	0.042746191
TCGA-29-1763-01A	B_cells_naive	0.092165769
TCGA-29-1763-01A	B_cells_memory	0
TCGA-29-1763-01A	Plasma_cells	0
TCGA-29-1763-01A	T_cells_CD8	0.014688083
TCGA-29-1763-01A	T_cells_CD4_naive	0
TCGA-29-1763-01A	T_cells_CD4_memory_resting	0.232474247
TCGA-29-1763-01A	T_cells_CD4_memory_activated	0
TCGA-29-1763-01A	T_cells_follicular_helper	0.017739429
TCGA-29-1763-01A	T_cells_regulatory_(Tregs)	0.023264277
TCGA-29-1763-01A	T_cells_gamma_delta	0
TCGA-29-1763-01A	NK_cells_resting	0
TCGA-29-1763-01A	NK_cells_activated	0.04985496
TCGA-29-1763-01A	Monocytes	0.027465066
TCGA-29-1763-01A	Macrophages_M0	0.150018968
TCGA-29-1763-01A	Macrophages_M1	0.055975511
TCGA-29-1763-01A	Macrophages_M2	0.267302672
TCGA-29-1763-01A	Dendritic_cells_resting	0.006638678
TCGA-29-1763-01A	Dendritic_cells_activated	0.033979282
TCGA-29-1763-01A	Mast_cells_resting	0.021065832
TCGA-29-1763-01A	Mast_cells_activated	0
TCGA-29-1763-01A	Eosinophils	0
TCGA-29-1763-01A	Neutrophils	0.007367226
TCGA-29-1766-01A	B_cells_naive	0.042356994
TCGA-29-1766-01A	B_cells_memory	0
TCGA-29-1766-01A	Plasma_cells	0.043294598
TCGA-29-1766-01A	T_cells_CD8	0.149289595
TCGA-29-1766-01A	T_cells_CD4_naive	0

TCGA-29-1766-01A	T_cells_CD4_memory_resting	0.127312844
TCGA-29-1766-01A	T_cells_CD4_memory_activated	0
TCGA-29-1766-01A	T_cells_follicular_helper	0.033927787
TCGA-29-1766-01A	T_cells_regulatory_(Tregs)	0.029368302
TCGA-29-1766-01A	T_cells_gamma_delta	0.008443097
TCGA-29-1766-01A	NK_cells_resting	0
TCGA-29-1766-01A	NK_cells_activated	0.081968005
TCGA-29-1766-01A	Monocytes	0.015854941
TCGA-29-1766-01A	Macrophages_M0	0.184874833
TCGA-29-1766-01A	Macrophages_M1	0.041983812
TCGA-29-1766-01A	Macrophages_M2	0.133016124
TCGA-29-1766-01A	Dendritic_cells_resting	0
TCGA-29-1766-01A	Dendritic_cells_activated	0.07465373
TCGA-29-1766-01A	Mast_cells_resting	0.027210471
TCGA-29-1766-01A	Mast_cells_activated	0
TCGA-29-1766-01A	Eosinophils	0.000791506
TCGA-29-1766-01A	Neutrophils	0.005653359
TCGA-29-1769-01A	B_cells_naive	0.041611316
TCGA-29-1769-01A	B_cells_memory	0
TCGA-29-1769-01A	Plasma_cells	0.027249032
TCGA-29-1769-01A	T_cells_CD8	0.104602346
TCGA-29-1769-01A	T_cells_CD4_naive	0
TCGA-29-1769-01A	T_cells_CD4_memory_resting	0.285529697
TCGA-29-1769-01A	T_cells_CD4_memory_activated	0
TCGA-29-1769-01A	T_cells_follicular_helper	0.033043284
TCGA-29-1769-01A	T_cells_regulatory_(Tregs)	0.023710236
TCGA-29-1769-01A	T_cells_gamma_delta	0
TCGA-29-1769-01A	NK_cells_resting	0
TCGA-29-1769-01A	NK_cells_activated	0.058957258
TCGA-29-1769-01A	Monocytes	0.160034403
TCGA-29-1769-01A	Macrophages_M0	0.004029767
TCGA-29-1769-01A	Macrophages_M1	0.049605971
TCGA-29-1769-01A	Macrophages_M2	0.168901914

TCGA-29-1769-01A	Dendritic_cells_resting	0
TCGA-29-1769-01A	Dendritic_cells_activated	0.04125984
TCGA-29-1769-01A	Mast_cells_resting	0.001464935
TCGA-29-1769-01A	Mast_cells_activated	0
TCGA-29-1769-01A	Eosinophils	0
TCGA-29-1769-01A	Neutrophils	0
TCGA-29-1770-01A	B_cells_naive	0.001893702
TCGA-29-1770-01A	B_cells_memory	0
TCGA-29-1770-01A	Plasma_cells	0.002126393
TCGA-29-1770-01A	T_cells_CD8	0.030855951
TCGA-29-1770-01A	T_cells_CD4_naive	0
TCGA-29-1770-01A	T_cells_CD4_memory_resting	0.112714214
TCGA-29-1770-01A	T_cells_CD4_memory_activated	0
TCGA-29-1770-01A	T_cells_follicular_helper	0.015914259
TCGA-29-1770-01A	T_cells_regulatory_(Tregs)	0.017960591
TCGA-29-1770-01A	T_cells_gamma_delta	0
TCGA-29-1770-01A	NK_cells_resting	0
TCGA-29-1770-01A	NK_cells_activated	0.106440898
TCGA-29-1770-01A	Monocytes	0.041545285
TCGA-29-1770-01A	Macrophages_M0	0.277846522
TCGA-29-1770-01A	Macrophages_M1	0.018829064
TCGA-29-1770-01A	Macrophages_M2	0.177814132
TCGA-29-1770-01A	Dendritic_cells_resting	0
TCGA-29-1770-01A	Dendritic_cells_activated	0.078938047
TCGA-29-1770-01A	Mast_cells_resting	0
TCGA-29-1770-01A	Mast_cells_activated	0.096283877
TCGA-29-1770-01A	Eosinophils	0.019053985
TCGA-29-1770-01A	Neutrophils	0.001783081
TCGA-29-1774-01A	B_cells_naive	0.209801414
TCGA-29-1774-01A	B_cells_memory	0
TCGA-29-1774-01A	Plasma_cells	0
TCGA-29-1774-01A	T_cells_CD8	0.10978865
TCGA-29-1774-01A	T_cells_CD4_naive	0

TCGA-29-1774-01A	T_cells_CD4_memory_resting	0.092061311
TCGA-29-1774-01A	T_cells_CD4_memory_activated	0
TCGA-29-1774-01A	T_cells_follicular_helper	0
TCGA-29-1774-01A	T_cells_regulatory_(Tregs)	0.02549279
TCGA-29-1774-01A	T_cells_gamma_delta	0
TCGA-29-1774-01A	NK_cells_resting	0
TCGA-29-1774-01A	NK_cells_activated	0.036207794
TCGA-29-1774-01A	Monocytes	0.050147873
TCGA-29-1774-01A	Macrophages_M0	0.26165864
TCGA-29-1774-01A	Macrophages_M1	0
TCGA-29-1774-01A	Macrophages_M2	0.208996947
TCGA-29-1774-01A	Dendritic_cells_resting	0
TCGA-29-1774-01A	Dendritic_cells_activated	0
TCGA-29-1774-01A	Mast_cells_resting	0.005844581
TCGA-29-1774-01A	Mast_cells_activated	0
TCGA-29-1774-01A	Eosinophils	0
TCGA-29-1774-01A	Neutrophils	0
TCGA-29-1777-01A	B_cells_naive	0.005305497
TCGA-29-1777-01A	B_cells_memory	0
TCGA-29-1777-01A	Plasma_cells	0.13502894
TCGA-29-1777-01A	T_cells_CD8	0.047872563
TCGA-29-1777-01A	T_cells_CD4_naive	0
TCGA-29-1777-01A	T_cells_CD4_memory_resting	0.16803036
TCGA-29-1777-01A	T_cells_CD4_memory_activated	0
TCGA-29-1777-01A	T_cells_follicular_helper	0.042777705
TCGA-29-1777-01A	T_cells_regulatory_(Tregs)	0
TCGA-29-1777-01A	T_cells_gamma_delta	0
TCGA-29-1777-01A	NK_cells_resting	0
TCGA-29-1777-01A	NK_cells_activated	0.066901272
TCGA-29-1777-01A	Monocytes	0
TCGA-29-1777-01A	Macrophages_M0	0.122080871
TCGA-29-1777-01A	Macrophages_M1	0.12927578
TCGA-29-1777-01A	Macrophages_M2	0.138541986

TCGA-29-1777-01A	Dendritic_cells_resting	0.030548428
TCGA-29-1777-01A	Dendritic_cells_activated	0.050656441
TCGA-29-1777-01A	Mast_cells_resting	0.016981151
TCGA-29-1777-01A	Mast_cells_activated	0.010296721
TCGA-29-1777-01A	Eosinophils	0.008373858
TCGA-29-1777-01A	Neutrophils	0.027328427
TCGA-29-1784-01A	B_cells_naive	0
TCGA-29-1784-01A	B_cells_memory	0.037990481
TCGA-29-1784-01A	Plasma_cells	0.148555387
TCGA-29-1784-01A	T_cells_CD8	0.028893995
TCGA-29-1784-01A	T_cells_CD4_naive	0
TCGA-29-1784-01A	T_cells_CD4_memory_resting	0.164664691
TCGA-29-1784-01A	T_cells_CD4_memory_activated	0
TCGA-29-1784-01A	T_cells_follicular_helper	0.007952966
TCGA-29-1784-01A	T_cells_regulatory_(Tregs)	0.053778876
TCGA-29-1784-01A	T_cells_gamma_delta	0.005173821
TCGA-29-1784-01A	NK_cells_resting	0
TCGA-29-1784-01A	NK_cells_activated	0.046362203
TCGA-29-1784-01A	Monocytes	0.014799476
TCGA-29-1784-01A	Macrophages_M0	0.114670276
TCGA-29-1784-01A	Macrophages_M1	0.107986877
TCGA-29-1784-01A	Macrophages_M2	0.203490255
TCGA-29-1784-01A	Dendritic_cells_resting	0.012204979
TCGA-29-1784-01A	Dendritic_cells_activated	0
TCGA-29-1784-01A	Mast_cells_resting	0.052726565
TCGA-29-1784-01A	Mast_cells_activated	0
TCGA-29-1784-01A	Eosinophils	0
TCGA-29-1784-01A	Neutrophils	0.000749153
TCGA-29-1785-01A	B_cells_naive	0.007294617
TCGA-29-1785-01A	B_cells_memory	0
TCGA-29-1785-01A	Plasma_cells	0
TCGA-29-1785-01A	T_cells_CD8	0.221400389
TCGA-29-1785-01A	T_cells_CD4_naive	0

TCGA-29-1785-01A	T_cells_CD4_memory_resting	0.031948971
TCGA-29-1785-01A	T_cells_CD4_memory_activated	0.008744557
TCGA-29-1785-01A	T_cells_follicular_helper	0.015908032
TCGA-29-1785-01A	T_cells_regulatory_(Tregs)	0.156318345
TCGA-29-1785-01A	T_cells_gamma_delta	0
TCGA-29-1785-01A	NK_cells_resting	0
TCGA-29-1785-01A	NK_cells_activated	0.105396838
TCGA-29-1785-01A	Monocytes	0.05189537
TCGA-29-1785-01A	Macrophages_M0	0
TCGA-29-1785-01A	Macrophages_M1	0.136724631
TCGA-29-1785-01A	Macrophages_M2	0.225896514
TCGA-29-1785-01A	Dendritic_cells_resting	0
TCGA-29-1785-01A	Dendritic_cells_activated	0
TCGA-29-1785-01A	Mast_cells_resting	0.033711825
TCGA-29-1785-01A	Mast_cells_activated	0
TCGA-29-1785-01A	Eosinophils	0
TCGA-29-1785-01A	Neutrophils	0.004759913
TCGA-29-2427-01A	B_cells_naive	0.01210787
TCGA-29-2427-01A	B_cells_memory	0
TCGA-29-2427-01A	Plasma_cells	0.08143358
TCGA-29-2427-01A	T_cells_CD8	0.195621945
TCGA-29-2427-01A	T_cells_CD4_naive	0
TCGA-29-2427-01A	T_cells_CD4_memory_resting	0.066655681
TCGA-29-2427-01A	T_cells_CD4_memory_activated	0
TCGA-29-2427-01A	T_cells_follicular_helper	0.019668992
TCGA-29-2427-01A	T_cells_regulatory_(Tregs)	0.035402901
TCGA-29-2427-01A	T_cells_gamma_delta	0
TCGA-29-2427-01A	NK_cells_resting	0.016780693
TCGA-29-2427-01A	NK_cells_activated	0.022453446
TCGA-29-2427-01A	Monocytes	0
TCGA-29-2427-01A	Macrophages_M0	0.165231886
TCGA-29-2427-01A	Macrophages_M1	0.131133499
TCGA-29-2427-01A	Macrophages_M2	0.217194859

TCGA-29-2427-01A	Dendritic_cells_resting	0
TCGA-29-2427-01A	Dendritic_cells_activated	0
TCGA-29-2427-01A	Mast_cells_resting	0.036314646
TCGA-29-2427-01A	Mast_cells_activated	0
TCGA-29-2427-01A	Eosinophils	0
TCGA-29-2427-01A	Neutrophils	0
TCGA-29-2428-01A	B_cells_naive	0.001343696
TCGA-29-2428-01A	B_cells_memory	0
TCGA-29-2428-01A	Plasma_cells	0.100145801
TCGA-29-2428-01A	T_cells_CD8	0.148586832
TCGA-29-2428-01A	T_cells_CD4_naive	0
TCGA-29-2428-01A	T_cells_CD4_memory_resting	0.028641082
TCGA-29-2428-01A	T_cells_CD4_memory_activated	0.054151048
TCGA-29-2428-01A	T_cells_follicular_helper	0.036365563
TCGA-29-2428-01A	T_cells_regulatory_(Tregs)	0.069576173
TCGA-29-2428-01A	T_cells_gamma_delta	0.056179038
TCGA-29-2428-01A	NK_cells_resting	0
TCGA-29-2428-01A	NK_cells_activated	0.008388052
TCGA-29-2428-01A	Monocytes	0
TCGA-29-2428-01A	Macrophages_M0	0.185007825
TCGA-29-2428-01A	Macrophages_M1	0.068727675
TCGA-29-2428-01A	Macrophages_M2	0.202892779
TCGA-29-2428-01A	Dendritic_cells_resting	0.034864251
TCGA-29-2428-01A	Dendritic_cells_activated	0
TCGA-29-2428-01A	Mast_cells_resting	0
TCGA-29-2428-01A	Mast_cells_activated	0.005130184
TCGA-29-2428-01A	Eosinophils	0
TCGA-29-2428-01A	Neutrophils	0
TCGA-30-1714-01A	B_cells_naive	0.046626336
TCGA-30-1714-01A	B_cells_memory	0
TCGA-30-1714-01A	Plasma_cells	0
TCGA-30-1714-01A	T_cells_CD8	0.040203789
TCGA-30-1714-01A	T_cells_CD4_naive	0

TCGA-30-1714-01A	T_cells_CD4_memory_resting	0.17164977
TCGA-30-1714-01A	T_cells_CD4_memory_activated	0
TCGA-30-1714-01A	T_cells_follicular_helper	0.083757795
TCGA-30-1714-01A	T_cells_regulatory_(Tregs)	0.015495454
TCGA-30-1714-01A	T_cells_gamma_delta	0
TCGA-30-1714-01A	NK_cells_resting	0
TCGA-30-1714-01A	NK_cells_activated	0.077992702
TCGA-30-1714-01A	Monocytes	0.052121971
TCGA-30-1714-01A	Macrophages_M0	0.139328643
TCGA-30-1714-01A	Macrophages_M1	0.032809124
TCGA-30-1714-01A	Macrophages_M2	0.209690345
TCGA-30-1714-01A	Dendritic_cells_resting	0.007883151
TCGA-30-1714-01A	Dendritic_cells_activated	0
TCGA-30-1714-01A	Mast_cells_resting	0
TCGA-30-1714-01A	Mast_cells_activated	0.099521326
TCGA-30-1714-01A	Eosinophils	0.022919595
TCGA-30-1714-01A	Neutrophils	0
TCGA-30-1718-01A	B_cells_naive	0.041262563
TCGA-30-1718-01A	B_cells_memory	0
TCGA-30-1718-01A	Plasma_cells	0.095540599
TCGA-30-1718-01A	T_cells_CD8	0.093370099
TCGA-30-1718-01A	T_cells_CD4_naive	0
TCGA-30-1718-01A	T_cells_CD4_memory_resting	0.209226038
TCGA-30-1718-01A	T_cells_CD4_memory_activated	0.01109118
TCGA-30-1718-01A	T_cells_follicular_helper	0.037664383
TCGA-30-1718-01A	T_cells_regulatory_(Tregs)	0.036312155
TCGA-30-1718-01A	T_cells_gamma_delta	0
TCGA-30-1718-01A	NK_cells_resting	0
TCGA-30-1718-01A	NK_cells_activated	0.043778113
TCGA-30-1718-01A	Monocytes	0.031444227
TCGA-30-1718-01A	Macrophages_M0	0.081199266
TCGA-30-1718-01A	Macrophages_M1	0.067758477
TCGA-30-1718-01A	Macrophages_M2	0.176752955

TCGA-30-1718-01A	Dendritic_cells_resting	0
TCGA-30-1718-01A	Dendritic_cells_activated	0.051275746
TCGA-30-1718-01A	Mast_cells_resting	0.014041436
TCGA-30-1718-01A	Mast_cells_activated	0
TCGA-30-1718-01A	Eosinophils	0
TCGA-30-1718-01A	Neutrophils	0.009282763
TCGA-30-1860-01A	B_cells_naive	0.018342605
TCGA-30-1860-01A	B_cells_memory	0
TCGA-30-1860-01A	Plasma_cells	0.068522769
TCGA-30-1860-01A	T_cells_CD8	0.074774786
TCGA-30-1860-01A	T_cells_CD4_naive	0
TCGA-30-1860-01A	T_cells_CD4_memory_resting	0.153526406
TCGA-30-1860-01A	T_cells_CD4_memory_activated	0.045979062
TCGA-30-1860-01A	T_cells_follicular_helper	0.074285539
TCGA-30-1860-01A	T_cells_regulatory_(Tregs)	0.023497615
TCGA-30-1860-01A	T_cells_gamma_delta	0.029794766
TCGA-30-1860-01A	NK_cells_resting	0
TCGA-30-1860-01A	NK_cells_activated	0.105339472
TCGA-30-1860-01A	Monocytes	0.011321682
TCGA-30-1860-01A	Macrophages_M0	0.08577773
TCGA-30-1860-01A	Macrophages_M1	0.175598735
TCGA-30-1860-01A	Macrophages_M2	0.097481415
TCGA-30-1860-01A	Dendritic_cells_resting	0.018765008
TCGA-30-1860-01A	Dendritic_cells_activated	0
TCGA-30-1860-01A	Mast_cells_resting	0.01699241
TCGA-30-1860-01A	Mast_cells_activated	0
TCGA-30-1860-01A	Eosinophils	0
TCGA-30-1860-01A	Neutrophils	0
TCGA-31-1944-01A	B_cells_naive	0.026699729
TCGA-31-1944-01A	B_cells_memory	0
TCGA-31-1944-01A	Plasma_cells	0.069127165
TCGA-31-1944-01A	T_cells_CD8	0.041169596
TCGA-31-1944-01A	T_cells_CD4_naive	0

TCGA-31-1944-01A	T_cells_CD4_memory_resting	0.249892896
TCGA-31-1944-01A	T_cells_CD4_memory_activated	0.028429524
TCGA-31-1944-01A	T_cells_follicular_helper	0.035788329
TCGA-31-1944-01A	T_cells_regulatory_(Tregs)	0.014798749
TCGA-31-1944-01A	T_cells_gamma_delta	0
TCGA-31-1944-01A	NK_cells_resting	0
TCGA-31-1944-01A	NK_cells_activated	0.037089274
TCGA-31-1944-01A	Monocytes	0.013778861
TCGA-31-1944-01A	Macrophages_M0	0.154951459
TCGA-31-1944-01A	Macrophages_M1	0.09851936
TCGA-31-1944-01A	Macrophages_M2	0.174160624
TCGA-31-1944-01A	Dendritic_cells_resting	0.015363282
TCGA-31-1944-01A	Dendritic_cells_activated	0
TCGA-31-1944-01A	Mast_cells_resting	0.021817898
TCGA-31-1944-01A	Mast_cells_activated	0
TCGA-31-1944-01A	Eosinophils	0.001836022
TCGA-31-1944-01A	Neutrophils	0.016577232
TCGA-31-1950-01A	B_cells_naive	0.08058222
TCGA-31-1950-01A	B_cells_memory	0
TCGA-31-1950-01A	Plasma_cells	0.174885025
TCGA-31-1950-01A	T_cells_CD8	0.061449231
TCGA-31-1950-01A	T_cells_CD4_naive	0
TCGA-31-1950-01A	T_cells_CD4_memory_resting	0.111252704
TCGA-31-1950-01A	T_cells_CD4_memory_activated	0.008389585
TCGA-31-1950-01A	T_cells_follicular_helper	0.027724664
TCGA-31-1950-01A	T_cells_regulatory_(Tregs)	0.087824161
TCGA-31-1950-01A	T_cells_gamma_delta	0
TCGA-31-1950-01A	NK_cells_resting	0
TCGA-31-1950-01A	NK_cells_activated	0.01689253
TCGA-31-1950-01A	Monocytes	0.016255186
TCGA-31-1950-01A	Macrophages_M0	0.020638978
TCGA-31-1950-01A	Macrophages_M1	0.181841384
TCGA-31-1950-01A	Macrophages_M2	0.104972517

TCGA-31-1950-01A	Dendritic_cells_resting	0
TCGA-31-1950-01A	Dendritic_cells_activated	0.074494272
TCGA-31-1950-01A	Mast_cells_resting	0.032264424
TCGA-31-1950-01A	Mast_cells_activated	0
TCGA-31-1950-01A	Eosinophils	0
TCGA-31-1950-01A	Neutrophils	0.000533118
TCGA-31-1951-01A	B_cells_naive	0.036438648
TCGA-31-1951-01A	B_cells_memory	0
TCGA-31-1951-01A	Plasma_cells	0.003658997
TCGA-31-1951-01A	T_cells_CD8	0.026894959
TCGA-31-1951-01A	T_cells_CD4_naive	0
TCGA-31-1951-01A	T_cells_CD4_memory_resting	0.260144191
TCGA-31-1951-01A	T_cells_CD4_memory_activated	0.000734886
TCGA-31-1951-01A	T_cells_follicular_helper	0.057218457
TCGA-31-1951-01A	T_cells_regulatory_(Tregs)	0.061913502
TCGA-31-1951-01A	T_cells_gamma_delta	0
TCGA-31-1951-01A	NK_cells_resting	0.072557494
TCGA-31-1951-01A	NK_cells_activated	0
TCGA-31-1951-01A	Monocytes	0.008158992
TCGA-31-1951-01A	Macrophages_M0	0.165059701
TCGA-31-1951-01A	Macrophages_M1	0.121322334
TCGA-31-1951-01A	Macrophages_M2	0.121115856
TCGA-31-1951-01A	Dendritic_cells_resting	0
TCGA-31-1951-01A	Dendritic_cells_activated	0.041318184
TCGA-31-1951-01A	Mast_cells_resting	0
TCGA-31-1951-01A	Mast_cells_activated	0.013511875
TCGA-31-1951-01A	Eosinophils	0
TCGA-31-1951-01A	Neutrophils	0.009951926
TCGA-31-1953-01A	B_cells_naive	0.001529014
TCGA-31-1953-01A	B_cells_memory	0
TCGA-31-1953-01A	Plasma_cells	0
TCGA-31-1953-01A	T_cells_CD8	0.042978267
TCGA-31-1953-01A	T_cells_CD4_naive	0

TCGA-31-1953-01A	T_cells_CD4_memory_resting	0.106189906
TCGA-31-1953-01A	T_cells_CD4_memory_activated	0
TCGA-31-1953-01A	T_cells_follicular_helper	0.026302654
TCGA-31-1953-01A	T_cells_regulatory_(Tregs)	0.080714294
TCGA-31-1953-01A	T_cells_gamma_delta	0
TCGA-31-1953-01A	NK_cells_resting	0
TCGA-31-1953-01A	NK_cells_activated	0.090860782
TCGA-31-1953-01A	Monocytes	0.028874776
TCGA-31-1953-01A	Macrophages_M0	0.2432781
TCGA-31-1953-01A	Macrophages_M1	0.074334843
TCGA-31-1953-01A	Macrophages_M2	0.178667144
TCGA-31-1953-01A	Dendritic_cells_resting	0.096150987
TCGA-31-1953-01A	Dendritic_cells_activated	0
TCGA-31-1953-01A	Mast_cells_resting	0.024297883
TCGA-31-1953-01A	Mast_cells_activated	0
TCGA-31-1953-01A	Eosinophils	0
TCGA-31-1953-01A	Neutrophils	0.00582135
TCGA-31-1956-01A	B_cells_naive	0.003335763
TCGA-31-1956-01A	B_cells_memory	0
TCGA-31-1956-01A	Plasma_cells	0.077263832
TCGA-31-1956-01A	T_cells_CD8	0.132405634
TCGA-31-1956-01A	T_cells_CD4_naive	0
TCGA-31-1956-01A	T_cells_CD4_memory_resting	0.073082436
TCGA-31-1956-01A	T_cells_CD4_memory_activated	0.053388709
TCGA-31-1956-01A	T_cells_follicular_helper	0.014905779
TCGA-31-1956-01A	T_cells_regulatory_(Tregs)	0.07558258
TCGA-31-1956-01A	T_cells_gamma_delta	0
TCGA-31-1956-01A	NK_cells_resting	0
TCGA-31-1956-01A	NK_cells_activated	0.023173406
TCGA-31-1956-01A	Monocytes	0
TCGA-31-1956-01A	Macrophages_M0	0.202439527
TCGA-31-1956-01A	Macrophages_M1	0.056222188
TCGA-31-1956-01A	Macrophages_M2	0.237894627

TCGA-31-1956-01A	Dendritic_cells_resting	0.025138238
TCGA-31-1956-01A	Dendritic_cells_activated	0
TCGA-31-1956-01A	Mast_cells_resting	0.016341761
TCGA-31-1956-01A	Mast_cells_activated	0
TCGA-31-1956-01A	Eosinophils	0
TCGA-31-1956-01A	Neutrophils	0.008825521
TCGA-31-1959-01A	B_cells_naive	0.008343721
TCGA-31-1959-01A	B_cells_memory	0.012186743
TCGA-31-1959-01A	Plasma_cells	0
TCGA-31-1959-01A	T_cells_CD8	0.060284575
TCGA-31-1959-01A	T_cells_CD4_naive	0
TCGA-31-1959-01A	T_cells_CD4_memory_resting	0.225245718
TCGA-31-1959-01A	T_cells_CD4_memory_activated	0
TCGA-31-1959-01A	T_cells_follicular_helper	0.032511624
TCGA-31-1959-01A	T_cells_regulatory_(Tregs)	0.061723165
TCGA-31-1959-01A	T_cells_gamma_delta	0
TCGA-31-1959-01A	NK_cells_resting	0
TCGA-31-1959-01A	NK_cells_activated	0.036717498
TCGA-31-1959-01A	Monocytes	0.056181566
TCGA-31-1959-01A	Macrophages_M0	0.100308084
TCGA-31-1959-01A	Macrophages_M1	0.046388139
TCGA-31-1959-01A	Macrophages_M2	0.294576102
TCGA-31-1959-01A	Dendritic_cells_resting	0.010922581
TCGA-31-1959-01A	Dendritic_cells_activated	0.035588254
TCGA-31-1959-01A	Mast_cells_resting	0
TCGA-31-1959-01A	Mast_cells_activated	0
TCGA-31-1959-01A	Eosinophils	0
TCGA-31-1959-01A	Neutrophils	0.01902223
TCGA-36-1568-01A	B_cells_naive	0
TCGA-36-1568-01A	B_cells_memory	0.002990842
TCGA-36-1568-01A	Plasma_cells	0.0295707
TCGA-36-1568-01A	T_cells_CD8	0.036667824
TCGA-36-1568-01A	T_cells_CD4_naive	0

TCGA-36-1568-01A	T_cells_CD4_memory_resting	0.235491225
TCGA-36-1568-01A	T_cells_CD4_memory_activated	0
TCGA-36-1568-01A	T_cells_follicular_helper	0.002457885
TCGA-36-1568-01A	T_cells_regulatory_(Tregs)	0.056881018
TCGA-36-1568-01A	T_cells_gamma_delta	0
TCGA-36-1568-01A	NK_cells_resting	0
TCGA-36-1568-01A	NK_cells_activated	0.06587715
TCGA-36-1568-01A	Monocytes	0.001512883
TCGA-36-1568-01A	Macrophages_M0	0.232204802
TCGA-36-1568-01A	Macrophages_M1	0.071027875
TCGA-36-1568-01A	Macrophages_M2	0.162891266
TCGA-36-1568-01A	Dendritic_cells_resting	0.057038448
TCGA-36-1568-01A	Dendritic_cells_activated	0
TCGA-36-1568-01A	Mast_cells_resting	0.045388083
TCGA-36-1568-01A	Mast_cells_activated	0
TCGA-36-1568-01A	Eosinophils	0
TCGA-36-1568-01A	Neutrophils	0
TCGA-36-1581-01A	B_cells_naive	0.101980535
TCGA-36-1581-01A	B_cells_memory	0
TCGA-36-1581-01A	Plasma_cells	0.138153542
TCGA-36-1581-01A	T_cells_CD8	0.066425106
TCGA-36-1581-01A	T_cells_CD4_naive	0
TCGA-36-1581-01A	T_cells_CD4_memory_resting	0.163969214
TCGA-36-1581-01A	T_cells_CD4_memory_activated	0.011883064
TCGA-36-1581-01A	T_cells_follicular_helper	0.058527341
TCGA-36-1581-01A	T_cells_regulatory_(Tregs)	0.048842888
TCGA-36-1581-01A	T_cells_gamma_delta	0.005212357
TCGA-36-1581-01A	NK_cells_resting	0.003531186
TCGA-36-1581-01A	NK_cells_activated	0
TCGA-36-1581-01A	Monocytes	0.005246163
TCGA-36-1581-01A	Macrophages_M0	0.180794528
TCGA-36-1581-01A	Macrophages_M1	0.084660032
TCGA-36-1581-01A	Macrophages_M2	0.124330364

TCGA-36-1581-01A	Dendritic_cells_resting	0
TCGA-36-1581-01A	Dendritic_cells_activated	0
TCGA-36-1581-01A	Mast_cells_resting	0.005431577
TCGA-36-1581-01A	Mast_cells_activated	0
TCGA-36-1581-01A	Eosinophils	0
TCGA-36-1581-01A	Neutrophils	0.001012104
TCGA-57-1584-01A	B_cells_naive	0.016229457
TCGA-57-1584-01A	B_cells_memory	0
TCGA-57-1584-01A	Plasma_cells	0.092512185
TCGA-57-1584-01A	T_cells_CD8	0.022279587
TCGA-57-1584-01A	T_cells_CD4_naive	0
TCGA-57-1584-01A	T_cells_CD4_memory_resting	0.256368271
TCGA-57-1584-01A	T_cells_CD4_memory_activated	0
TCGA-57-1584-01A	T_cells_follicular_helper	0
TCGA-57-1584-01A	T_cells_regulatory_(Tregs)	0.013964046
TCGA-57-1584-01A	T_cells_gamma_delta	0
TCGA-57-1584-01A	NK_cells_resting	0
TCGA-57-1584-01A	NK_cells_activated	0.049161779
TCGA-57-1584-01A	Monocytes	0.156558243
TCGA-57-1584-01A	Macrophages_M0	0
TCGA-57-1584-01A	Macrophages_M1	0.047658227
TCGA-57-1584-01A	Macrophages_M2	0.216063976
TCGA-57-1584-01A	Dendritic_cells_resting	0
TCGA-57-1584-01A	Dendritic_cells_activated	0.076475599
TCGA-57-1584-01A	Mast_cells_resting	0.05272863
TCGA-57-1584-01A	Mast_cells_activated	0
TCGA-57-1584-01A	Eosinophils	0
TCGA-57-1584-01A	Neutrophils	0
TCGA-57-1586-01A	B_cells_naive	0.147910538
TCGA-57-1586-01A	B_cells_memory	0
TCGA-57-1586-01A	Plasma_cells	0.141490511
TCGA-57-1586-01A	T_cells_CD8	0.046974067
TCGA-57-1586-01A	T_cells_CD4_naive	0

TCGA-57-1586-01A	T_cells_CD4_memory_resting	0.129476652
TCGA-57-1586-01A	T_cells_CD4_memory_activated	0
TCGA-57-1586-01A	T_cells_follicular_helper	0.005266499
TCGA-57-1586-01A	T_cells_regulatory_(Tregs)	0.057867398
TCGA-57-1586-01A	T_cells_gamma_delta	0
TCGA-57-1586-01A	NK_cells_resting	0
TCGA-57-1586-01A	NK_cells_activated	0.025022263
TCGA-57-1586-01A	Monocytes	0.002696565
TCGA-57-1586-01A	Macrophages_M0	0.154730833
TCGA-57-1586-01A	Macrophages_M1	0.160062498
TCGA-57-1586-01A	Macrophages_M2	0.128502175
TCGA-57-1586-01A	Dendritic_cells_resting	0
TCGA-57-1586-01A	Dendritic_cells_activated	0
TCGA-57-1586-01A	Mast_cells_resting	0
TCGA-57-1586-01A	Mast_cells_activated	0
TCGA-57-1586-01A	Eosinophils	0
TCGA-57-1586-01A	Neutrophils	0
TCGA-57-1994-01A	B_cells_naive	0.028783157
TCGA-57-1994-01A	B_cells_memory	0
TCGA-57-1994-01A	Plasma_cells	0.045244923
TCGA-57-1994-01A	T_cells_CD8	0.159270541
TCGA-57-1994-01A	T_cells_CD4_naive	0
TCGA-57-1994-01A	T_cells_CD4_memory_resting	0.205504923
TCGA-57-1994-01A	T_cells_CD4_memory_activated	0
TCGA-57-1994-01A	T_cells_follicular_helper	0.022894205
TCGA-57-1994-01A	T_cells_regulatory_(Tregs)	0.101084387
TCGA-57-1994-01A	T_cells_gamma_delta	0
TCGA-57-1994-01A	NK_cells_resting	0
TCGA-57-1994-01A	NK_cells_activated	0.03797369
TCGA-57-1994-01A	Monocytes	0
TCGA-57-1994-01A	Macrophages_M0	0.179891782
TCGA-57-1994-01A	Macrophages_M1	0.0752467
TCGA-57-1994-01A	Macrophages_M2	0.142818834

TCGA-57-1994-01A	Dendritic_cells_resting	0
TCGA-57-1994-01A	Dendritic_cells_activated	0
TCGA-57-1994-01A	Mast_cells_resting	0
TCGA-57-1994-01A	Mast_cells_activated	0
TCGA-57-1994-01A	Eosinophils	0
TCGA-57-1994-01A	Neutrophils	0.001286857
TCGA-59-2348-01A	B_cells_naive	0.052780405
TCGA-59-2348-01A	B_cells_memory	0
TCGA-59-2348-01A	Plasma_cells	0.137782038
TCGA-59-2348-01A	T_cells_CD8	0.023111298
TCGA-59-2348-01A	T_cells_CD4_naive	0
TCGA-59-2348-01A	T_cells_CD4_memory_resting	0.135090805
TCGA-59-2348-01A	T_cells_CD4_memory_activated	0
TCGA-59-2348-01A	T_cells_follicular_helper	0.051342439
TCGA-59-2348-01A	T_cells_regulatory_(Tregs)	0.032331171
TCGA-59-2348-01A	T_cells_gamma_delta	0
TCGA-59-2348-01A	NK_cells_resting	0
TCGA-59-2348-01A	NK_cells_activated	0.023102541
TCGA-59-2348-01A	Monocytes	0.010312733
TCGA-59-2348-01A	Macrophages_M0	0.314422363
TCGA-59-2348-01A	Macrophages_M1	0.067336528
TCGA-59-2348-01A	Macrophages_M2	0.120399145
TCGA-59-2348-01A	Dendritic_cells_resting	0
TCGA-59-2348-01A	Dendritic_cells_activated	0
TCGA-59-2348-01A	Mast_cells_resting	0.027074057
TCGA-59-2348-01A	Mast_cells_activated	0
TCGA-59-2348-01A	Eosinophils	0
TCGA-59-2348-01A	Neutrophils	0.004914477
TCGA-59-2350-01A	B_cells_naive	0.027597251
TCGA-59-2350-01A	B_cells_memory	0
TCGA-59-2350-01A	Plasma_cells	0
TCGA-59-2350-01A	T_cells_CD8	0.077915169
TCGA-59-2350-01A	T_cells_CD4_naive	0

TCGA-59-2350-01A	T_cells_CD4_memory_resting	0.09235304
TCGA-59-2350-01A	T_cells_CD4_memory_activated	0
TCGA-59-2350-01A	T_cells_follicular_helper	0.044295656
TCGA-59-2350-01A	T_cells_regulatory_(Tregs)	0.030388341
TCGA-59-2350-01A	T_cells_gamma_delta	0
TCGA-59-2350-01A	NK_cells_resting	0.011165203
TCGA-59-2350-01A	NK_cells_activated	0.005971476
TCGA-59-2350-01A	Monocytes	0
TCGA-59-2350-01A	Macrophages_M0	0.35955433
TCGA-59-2350-01A	Macrophages_M1	0.104507472
TCGA-59-2350-01A	Macrophages_M2	0.125494999
TCGA-59-2350-01A	Dendritic_cells_resting	0
TCGA-59-2350-01A	Dendritic_cells_activated	0.106283255
TCGA-59-2350-01A	Mast_cells_resting	0.014473807
TCGA-59-2350-01A	Mast_cells_activated	0
TCGA-59-2350-01A	Eosinophils	0
TCGA-59-2350-01A	Neutrophils	0
TCGA-59-2351-01A	B_cells_naive	0
TCGA-59-2351-01A	B_cells_memory	0
TCGA-59-2351-01A	Plasma_cells	0.203653516
TCGA-59-2351-01A	T_cells_CD8	0.008497653
TCGA-59-2351-01A	T_cells_CD4_naive	0
TCGA-59-2351-01A	T_cells_CD4_memory_resting	0.074707757
TCGA-59-2351-01A	T_cells_CD4_memory_activated	0.010365592
TCGA-59-2351-01A	T_cells_follicular_helper	0.006452622
TCGA-59-2351-01A	T_cells_regulatory_(Tregs)	0.031642577
TCGA-59-2351-01A	T_cells_gamma_delta	0.003985921
TCGA-59-2351-01A	NK_cells_resting	0
TCGA-59-2351-01A	NK_cells_activated	0.056902548
TCGA-59-2351-01A	Monocytes	0
TCGA-59-2351-01A	Macrophages_M0	0.267490651
TCGA-59-2351-01A	Macrophages_M1	0.092447327
TCGA-59-2351-01A	Macrophages_M2	0.201675374

TCGA-59-2351-01A	Dendritic_cells_resting	0.004985005
TCGA-59-2351-01A	Dendritic_cells_activated	0
TCGA-59-2351-01A	Mast_cells_resting	0.037136006
TCGA-59-2351-01A	Mast_cells_activated	0
TCGA-59-2351-01A	Eosinophils	5.74496E-05
TCGA-59-2351-01A	Neutrophils	0
TCGA-59-A5PD-01A	B_cells_naive	0.054040101
TCGA-59-A5PD-01A	B_cells_memory	0
TCGA-59-A5PD-01A	Plasma_cells	0.05824553
TCGA-59-A5PD-01A	T_cells_CD8	0.020537773
TCGA-59-A5PD-01A	T_cells_CD4_naive	0
TCGA-59-A5PD-01A	T_cells_CD4_memory_resting	0.233856509
TCGA-59-A5PD-01A	T_cells_CD4_memory_activated	0.013062261
TCGA-59-A5PD-01A	T_cells_follicular_helper	0.022134374
TCGA-59-A5PD-01A	T_cells_regulatory_(Tregs)	0.029303991
TCGA-59-A5PD-01A	T_cells_gamma_delta	0
TCGA-59-A5PD-01A	NK_cells_resting	0
TCGA-59-A5PD-01A	NK_cells_activated	0.025753603
TCGA-59-A5PD-01A	Monocytes	0

TCGA-59-A5PD-01A	Macrophages_M0	0.386986377
TCGA-59-A5PD-01A	Macrophages_M1	0.046728279
TCGA-59-A5PD-01A	Macrophages_M2	0.0852135
TCGA-59-A5PD-01A	Dendritic_cells_resting	0
TCGA-59-A5PD-01A	Dendritic_cells_activated	0
TCGA-59-A5PD-01A	Mast_cells_resting	0
TCGA-59-A5PD-01A	Mast_cells_activated	0.024137702
TCGA-59-A5PD-01A	Eosinophils	0
TCGA-59-A5PD-01A	Neutrophils	0
TCGA-61-1725-01A	B_cells_naive	0.016769308
TCGA-61-1725-01A	B_cells_memory	0
TCGA-61-1725-01A	Plasma_cells	0.026982585
TCGA-61-1725-01A	T_cells_CD8	0.105612575
TCGA-61-1725-01A	T_cells_CD4_naive	0
TCGA-61-1725-01A	T_cells_CD4_memory_resting	0.215137554
TCGA-61-1725-01A	T_cells_CD4_memory_activated	0
TCGA-61-1725-01A	T_cells_follicular_helper	0.07246534
TCGA-61-1725-01A	T_cells_regulatory_(Tregs)	0.017292652
TCGA-61-1725-01A	T_cells_gamma_delta	0
TCGA-61-1725-01A	NK_cells_resting	0
TCGA-61-1725-01A	NK_cells_activated	0.029694152
TCGA-61-1725-01A	Monocytes	0.05712943
TCGA-61-1725-01A	Macrophages_M0	0.04787008
TCGA-61-1725-01A	Macrophages_M1	0.153738625

TCGA-61-1725-01A	Macrophages_M2	0.229789886
TCGA-61-1725-01A	Dendritic_cells_resting	0
TCGA-61-1725-01A	Dendritic_cells_activated	0
TCGA-61-1725-01A	Mast_cells_resting	0.005648495
TCGA-61-1725-01A	Mast_cells_activated	0
TCGA-61-1725-01A	Eosinophils	0
TCGA-61-1725-01A	Neutrophils	0.021869319
TCGA-61-1733-01A	B_cells_naive	0
TCGA-61-1733-01A	B_cells_memory	0
TCGA-61-1733-01A	Plasma_cells	0.011108999
TCGA-61-1733-01A	T_cells_CD8	0.031843239
TCGA-61-1733-01A	T_cells_CD4_naive	0
TCGA-61-1733-01A	T_cells_CD4_memory_resting	0.265259243
TCGA-61-1733-01A	T_cells_CD4_memory_activated	0
TCGA-61-1733-01A	T_cells_follicular_helper	0.055870415
TCGA-61-1733-01A	T_cells_regulatory_(Tregs)	0.057796394
TCGA-61-1733-01A	T_cells_gamma_delta	0
TCGA-61-1733-01A	NK_cells_resting	0
TCGA-61-1733-01A	NK_cells_activated	0.001807409
TCGA-61-1733-01A	Monocytes	0.061898187
TCGA-61-1733-01A	Macrophages_M0	0.086071509
TCGA-61-1733-01A	Macrophages_M1	0.080537293
TCGA-61-1733-01A	Macrophages_M2	0.301109773
TCGA-61-1733-01A	Dendritic_cells_resting	0
TCGA-61-1733-01A	Dendritic_cells_activated	0.008341127
TCGA-61-1733-01A	Mast_cells_resting	0
TCGA-61-1733-01A	Mast_cells_activated	0.038054697
TCGA-61-1733-01A	Eosinophils	0
TCGA-61-1733-01A	Neutrophils	0.000301715
TCGA-61-1907-01A	B_cells_naive	0
TCGA-61-1907-01A	B_cells_memory	0
TCGA-61-1907-01A	Plasma_cells	0.073192341
TCGA-61-1907-01A	T_cells_CD8	0.099655875

TCGA-61-1907-01A	T_cells_CD4_naive	0
TCGA-61-1907-01A	T_cells_CD4_memory_resting	0.162698288
TCGA-61-1907-01A	T_cells_CD4_memory_activated	0.015904173
TCGA-61-1907-01A	T_cells_follicular_helper	0.020419732
TCGA-61-1907-01A	T_cells_regulatory_(Tregs)	0.071671794
TCGA-61-1907-01A	T_cells_gamma_delta	0.004538741
TCGA-61-1907-01A	NK_cells_resting	0
TCGA-61-1907-01A	NK_cells_activated	0.028770096
TCGA-61-1907-01A	Monocytes	0
TCGA-61-1907-01A	Macrophages_M0	0.194870734
TCGA-61-1907-01A	Macrophages_M1	0.077629558
TCGA-61-1907-01A	Macrophages_M2	0.240227643
TCGA-61-1907-01A	Dendritic_cells_resting	0.002876548
TCGA-61-1907-01A	Dendritic_cells_activated	0
TCGA-61-1907-01A	Mast_cells_resting	0
TCGA-61-1907-01A	Mast_cells_activated	0.007544476
TCGA-61-1907-01A	Eosinophils	0
TCGA-61-1907-01A	Neutrophils	0
TCGA-61-1910-01A	B_cells_naive	0.011647691
TCGA-61-1910-01A	B_cells_memory	0
TCGA-61-1910-01A	Plasma_cells	0
TCGA-61-1910-01A	T_cells_CD8	0.014526583
TCGA-61-1910-01A	T_cells_CD4_naive	0
TCGA-61-1910-01A	T_cells_CD4_memory_resting	0.093176658
TCGA-61-1910-01A	T_cells_CD4_memory_activated	0
TCGA-61-1910-01A	T_cells_follicular_helper	0.010659355
TCGA-61-1910-01A	T_cells_regulatory_(Tregs)	0.061398468
TCGA-61-1910-01A	T_cells_gamma_delta	0
TCGA-61-1910-01A	NK_cells_resting	0
TCGA-61-1910-01A	NK_cells_activated	0.048978222
TCGA-61-1910-01A	Monocytes	0
TCGA-61-1910-01A	Macrophages_M0	0.271969249
TCGA-61-1910-01A	Macrophages_M1	0.10388932

TCGA-61-1910-01A	Macrophages_M2	0.242402113
TCGA-61-1910-01A	Dendritic_cells_resting	0
TCGA-61-1910-01A	Dendritic_cells_activated	0.04754989
TCGA-61-1910-01A	Mast_cells_resting	0
TCGA-61-1910-01A	Mast_cells_activated	0.074735324
TCGA-61-1910-01A	Eosinophils	0
TCGA-61-1910-01A	Neutrophils	0.019067128
TCGA-61-1911-01A	B_cells_naive	0.020546579
TCGA-61-1911-01A	B_cells_memory	0
TCGA-61-1911-01A	Plasma_cells	0.171697672
TCGA-61-1911-01A	T_cells_CD8	0.281108899
TCGA-61-1911-01A	T_cells_CD4_naive	0
TCGA-61-1911-01A	T_cells_CD4_memory_resting	0.02807319
TCGA-61-1911-01A	T_cells_CD4_memory_activated	0
TCGA-61-1911-01A	T_cells_follicular_helper	0.033891106
TCGA-61-1911-01A	T_cells_regulatory_(Tregs)	0.095914063
TCGA-61-1911-01A	T_cells_gamma_delta	0
TCGA-61-1911-01A	NK_cells_resting	0
TCGA-61-1911-01A	NK_cells_activated	0.088582231
TCGA-61-1911-01A	Monocytes	0.003776159
TCGA-61-1911-01A	Macrophages_M0	0.044025686
TCGA-61-1911-01A	Macrophages_M1	0.090607099
TCGA-61-1911-01A	Macrophages_M2	0.117543916
TCGA-61-1911-01A	Dendritic_cells_resting	0.001411413
TCGA-61-1911-01A	Dendritic_cells_activated	0
TCGA-61-1911-01A	Mast_cells_resting	0.017100265
TCGA-61-1911-01A	Mast_cells_activated	0
TCGA-61-1911-01A	Eosinophils	0
TCGA-61-1911-01A	Neutrophils	0.005721721
TCGA-61-1914-01A	B_cells_naive	0.042703163
TCGA-61-1914-01A	B_cells_memory	0
TCGA-61-1914-01A	Plasma_cells	0.101895115
TCGA-61-1914-01A	T_cells_CD8	0.041854517

TCGA-61-1914-01A	T_cells_CD4_naive	0
TCGA-61-1914-01A	T_cells_CD4_memory_resting	0.076677608
TCGA-61-1914-01A	T_cells_CD4_memory_activated	0
TCGA-61-1914-01A	T_cells_follicular_helper	0.032951621
TCGA-61-1914-01A	T_cells_regulatory_(Tregs)	0
TCGA-61-1914-01A	T_cells_gamma_delta	0.027380737
TCGA-61-1914-01A	NK_cells_resting	0
TCGA-61-1914-01A	NK_cells_activated	0.040127167
TCGA-61-1914-01A	Monocytes	0.074044954
TCGA-61-1914-01A	Macrophages_M0	0.085379172
TCGA-61-1914-01A	Macrophages_M1	0.191915965
TCGA-61-1914-01A	Macrophages_M2	0.222649154
TCGA-61-1914-01A	Dendritic_cells_resting	0
TCGA-61-1914-01A	Dendritic_cells_activated	0
TCGA-61-1914-01A	Mast_cells_resting	0
TCGA-61-1914-01A	Mast_cells_activated	0.021286448
TCGA-61-1914-01A	Eosinophils	0.036892989
TCGA-61-1914-01A	Neutrophils	0.004241389
TCGA-61-1918-01A	B_cells_naive	0.027541045
TCGA-61-1918-01A	B_cells_memory	0
TCGA-61-1918-01A	Plasma_cells	0.008648166
TCGA-61-1918-01A	T_cells_CD8	0.054832888
TCGA-61-1918-01A	T_cells_CD4_naive	0
TCGA-61-1918-01A	T_cells_CD4_memory_resting	0.110452959
TCGA-61-1918-01A	T_cells_CD4_memory_activated	0
TCGA-61-1918-01A	T_cells_follicular_helper	0.009535595
TCGA-61-1918-01A	T_cells_regulatory_(Tregs)	0.083305639
TCGA-61-1918-01A	T_cells_gamma_delta	0
TCGA-61-1918-01A	NK_cells_resting	0
TCGA-61-1918-01A	NK_cells_activated	0.049196764
TCGA-61-1918-01A	Monocytes	0.043064244
TCGA-61-1918-01A	Macrophages_M0	0.078298539
TCGA-61-1918-01A	Macrophages_M1	0.109909198

TCGA-61-1918-01A	Macrophages_M2	0.293362026
TCGA-61-1918-01A	Dendritic_cells_resting	0.072448544
TCGA-61-1918-01A	Dendritic_cells_activated	0
TCGA-61-1918-01A	Mast_cells_resting	0.059404392
TCGA-61-1918-01A	Mast_cells_activated	0
TCGA-61-1918-01A	Eosinophils	0
TCGA-61-1918-01A	Neutrophils	0
TCGA-61-1919-01A	B_cells_naive	0.021904398
TCGA-61-1919-01A	B_cells_memory	0
TCGA-61-1919-01A	Plasma_cells	0.051450776
TCGA-61-1919-01A	T_cells_CD8	0.033952721
TCGA-61-1919-01A	T_cells_CD4_naive	0
TCGA-61-1919-01A	T_cells_CD4_memory_resting	0.086469034
TCGA-61-1919-01A	T_cells_CD4_memory_activated	0.00968007
TCGA-61-1919-01A	T_cells_follicular_helper	0.004441357
TCGA-61-1919-01A	T_cells_regulatory_(Tregs)	0.0303312
TCGA-61-1919-01A	T_cells_gamma_delta	0.00625512
TCGA-61-1919-01A	NK_cells_resting	0
TCGA-61-1919-01A	NK_cells_activated	0.027425328
TCGA-61-1919-01A	Monocytes	0
TCGA-61-1919-01A	Macrophages_M0	0.385678106
TCGA-61-1919-01A	Macrophages_M1	0.103378827
TCGA-61-1919-01A	Macrophages_M2	0.192390242
TCGA-61-1919-01A	Dendritic_cells_resting	0.037387771
TCGA-61-1919-01A	Dendritic_cells_activated	0
TCGA-61-1919-01A	Mast_cells_resting	0.006170955
TCGA-61-1919-01A	Mast_cells_activated	0
TCGA-61-1919-01A	Eosinophils	0
TCGA-61-1919-01A	Neutrophils	0.003084094
TCGA-61-2000-01A	B_cells_naive	0.032546668
TCGA-61-2000-01A	B_cells_memory	0
TCGA-61-2000-01A	Plasma_cells	0.002600201
TCGA-61-2000-01A	T_cells_CD8	0.035904286

TCGA-61-2000-01A	T_cells_CD4_naive	0
TCGA-61-2000-01A	T_cells_CD4_memory_resting	0.293185145
TCGA-61-2000-01A	T_cells_CD4_memory_activated	0.006125726
TCGA-61-2000-01A	T_cells_follicular_helper	0.028483511
TCGA-61-2000-01A	T_cells_regulatory_(Tregs)	0.018616689
TCGA-61-2000-01A	T_cells_gamma_delta	0
TCGA-61-2000-01A	NK_cells_resting	0
TCGA-61-2000-01A	NK_cells_activated	0.158611374
TCGA-61-2000-01A	Monocytes	0.077996909
TCGA-61-2000-01A	Macrophages_M0	0.121750098
TCGA-61-2000-01A	Macrophages_M1	0.041047846
TCGA-61-2000-01A	Macrophages_M2	0.11016814
TCGA-61-2000-01A	Dendritic_cells_resting	0.025863413
TCGA-61-2000-01A	Dendritic_cells_activated	0.033609158
TCGA-61-2000-01A	Mast_cells_resting	0
TCGA-61-2000-01A	Mast_cells_activated	0.006782729
TCGA-61-2000-01A	Eosinophils	0
TCGA-61-2000-01A	Neutrophils	0.006708106
TCGA-61-2002-01A	B_cells_naive	0.019594652
TCGA-61-2002-01A	B_cells_memory	0
TCGA-61-2002-01A	Plasma_cells	0.015393993
TCGA-61-2002-01A	T_cells_CD8	0.147747865
TCGA-61-2002-01A	T_cells_CD4_naive	0
TCGA-61-2002-01A	T_cells_CD4_memory_resting	0.176868574
TCGA-61-2002-01A	T_cells_CD4_memory_activated	0.042709303
TCGA-61-2002-01A	T_cells_follicular_helper	0.001450363
TCGA-61-2002-01A	T_cells_regulatory_(Tregs)	0.126140126
TCGA-61-2002-01A	T_cells_gamma_delta	0
TCGA-61-2002-01A	NK_cells_resting	0
TCGA-61-2002-01A	NK_cells_activated	0.056149803
TCGA-61-2002-01A	Monocytes	0.057767647
TCGA-61-2002-01A	Macrophages_M0	0.079965839
TCGA-61-2002-01A	Macrophages_M1	0.088096489

TCGA-61-2002-01A	Macrophages_M2	0.117913841
TCGA-61-2002-01A	Dendritic_cells_resting	0.024613556
TCGA-61-2002-01A	Dendritic_cells_activated	0
TCGA-61-2002-01A	Mast_cells_resting	0.039912661
TCGA-61-2002-01A	Mast_cells_activated	0
TCGA-61-2002-01A	Eosinophils	0
TCGA-61-2002-01A	Neutrophils	0.005675289
TCGA-61-2092-01A	B_cells_naive	0.153564797
TCGA-61-2092-01A	B_cells_memory	0
TCGA-61-2092-01A	Plasma_cells	0.387871882
TCGA-61-2092-01A	T_cells_CD8	0.01317556
TCGA-61-2092-01A	T_cells_CD4_naive	0
TCGA-61-2092-01A	T_cells_CD4_memory_resting	0.080974371
TCGA-61-2092-01A	T_cells_CD4_memory_activated	0
TCGA-61-2092-01A	T_cells_follicular_helper	0
TCGA-61-2092-01A	T_cells_regulatory_(Tregs)	0.013537209
TCGA-61-2092-01A	T_cells_gamma_delta	0
TCGA-61-2092-01A	NK_cells_resting	0
TCGA-61-2092-01A	NK_cells_activated	0.016585859
TCGA-61-2092-01A	Monocytes	0
TCGA-61-2092-01A	Macrophages_M0	0.200364439
TCGA-61-2092-01A	Macrophages_M1	0.002425337
TCGA-61-2092-01A	Macrophages_M2	0.120438856
TCGA-61-2092-01A	Dendritic_cells_resting	0
TCGA-61-2092-01A	Dendritic_cells_activated	0.007192489
TCGA-61-2092-01A	Mast_cells_resting	0.003869202
TCGA-61-2092-01A	Mast_cells_activated	0
TCGA-61-2092-01A	Eosinophils	0
TCGA-61-2092-01A	Neutrophils	0
TCGA-61-2097-01A	B_cells_naive	0.041873405
TCGA-61-2097-01A	B_cells_memory	0
TCGA-61-2097-01A	Plasma_cells	0.002820295
TCGA-61-2097-01A	T_cells_CD8	0

TCGA-61-2097-01A	T_cells_CD4_naive	0
TCGA-61-2097-01A	T_cells_CD4_memory_resting	0.147733788
TCGA-61-2097-01A	T_cells_CD4_memory_activated	0
TCGA-61-2097-01A	T_cells_follicular_helper	0.029785776
TCGA-61-2097-01A	T_cells_regulatory_(Tregs)	0.018978931
TCGA-61-2097-01A	T_cells_gamma_delta	0
TCGA-61-2097-01A	NK_cells_resting	0
TCGA-61-2097-01A	NK_cells_activated	0.057542966
TCGA-61-2097-01A	Monocytes	0.086284977
TCGA-61-2097-01A	Macrophages_M0	0.176440566
TCGA-61-2097-01A	Macrophages_M1	0.008268284
TCGA-61-2097-01A	Macrophages_M2	0.168144992
TCGA-61-2097-01A	Dendritic_cells_resting	0
TCGA-61-2097-01A	Dendritic_cells_activated	0.121264333
TCGA-61-2097-01A	Mast_cells_resting	0
TCGA-61-2097-01A	Mast_cells_activated	0.119392399
TCGA-61-2097-01A	Eosinophils	0.007006474
TCGA-61-2097-01A	Neutrophils	0.014462814
TCGA-61-2098-01A	B_cells_naive	0
TCGA-61-2098-01A	B_cells_memory	0.00426549
TCGA-61-2098-01A	Plasma_cells	0.01616862
TCGA-61-2098-01A	T_cells_CD8	0
TCGA-61-2098-01A	T_cells_CD4_naive	0
TCGA-61-2098-01A	T_cells_CD4_memory_resting	0.282270699
TCGA-61-2098-01A	T_cells_CD4_memory_activated	0
TCGA-61-2098-01A	T_cells_follicular_helper	0.022810469
TCGA-61-2098-01A	T_cells_regulatory_(Tregs)	0.012750514
TCGA-61-2098-01A	T_cells_gamma_delta	0
TCGA-61-2098-01A	NK_cells_resting	0
TCGA-61-2098-01A	NK_cells_activated	0.114510829
TCGA-61-2098-01A	Monocytes	0.016987359
TCGA-61-2098-01A	Macrophages_M0	0.418218389
TCGA-61-2098-01A	Macrophages_M1	0.007679323

TCGA-61-2098-01A	Macrophages_M2	0.070215579
TCGA-61-2098-01A	Dendritic_cells_resting	0
TCGA-61-2098-01A	Dendritic_cells_activated	0
TCGA-61-2098-01A	Mast_cells_resting	0.017957127
TCGA-61-2098-01A	Mast_cells_activated	0
TCGA-61-2098-01A	Eosinophils	0.016165602
TCGA-61-2098-01A	Neutrophils	0
TCGA-61-2101-01A	B_cells_naive	0.03398552
TCGA-61-2101-01A	B_cells_memory	0
TCGA-61-2101-01A	Plasma_cells	0.012242765
TCGA-61-2101-01A	T_cells_CD8	0.037123032
TCGA-61-2101-01A	T_cells_CD4_naive	0
TCGA-61-2101-01A	T_cells_CD4_memory_resting	0.219132856
TCGA-61-2101-01A	T_cells_CD4_memory_activated	0
TCGA-61-2101-01A	T_cells_follicular_helper	0.005130312
TCGA-61-2101-01A	T_cells_regulatory_(Tregs)	0.067791291
TCGA-61-2101-01A	T_cells_gamma_delta	0
TCGA-61-2101-01A	NK_cells_resting	0
TCGA-61-2101-01A	NK_cells_activated	0.014418397
TCGA-61-2101-01A	Monocytes	0
TCGA-61-2101-01A	Macrophages_M0	0.226619835
TCGA-61-2101-01A	Macrophages_M1	0.064723073
TCGA-61-2101-01A	Macrophages_M2	0.176087994
TCGA-61-2101-01A	Dendritic_cells_resting	0.108423953
TCGA-61-2101-01A	Dendritic_cells_activated	0.003009754
TCGA-61-2101-01A	Mast_cells_resting	0.031311217
TCGA-61-2101-01A	Mast_cells_activated	0
TCGA-61-2101-01A	Eosinophils	0
TCGA-61-2101-01A	Neutrophils	0
TCGA-61-2104-01A	B_cells_naive	0.069479577
TCGA-61-2104-01A	B_cells_memory	0
TCGA-61-2104-01A	Plasma_cells	0.05928551
TCGA-61-2104-01A	T_cells_CD8	0.046554923

TCGA-61-2104-01A	T_cells_CD4_naive	0
TCGA-61-2104-01A	T_cells_CD4_memory_resting	0.192317111
TCGA-61-2104-01A	T_cells_CD4_memory_activated	0
TCGA-61-2104-01A	T_cells_follicular_helper	0
TCGA-61-2104-01A	T_cells_regulatory_(Tregs)	0.05874228
TCGA-61-2104-01A	T_cells_gamma_delta	0
TCGA-61-2104-01A	NK_cells_resting	0.013079521
TCGA-61-2104-01A	NK_cells_activated	0.001837272
TCGA-61-2104-01A	Monocytes	0.011565891
TCGA-61-2104-01A	Macrophages_M0	0.210676462
TCGA-61-2104-01A	Macrophages_M1	0.155766985
TCGA-61-2104-01A	Macrophages_M2	0.127557972
TCGA-61-2104-01A	Dendritic_cells_resting	0
TCGA-61-2104-01A	Dendritic_cells_activated	0.036750765
TCGA-61-2104-01A	Mast_cells_resting	0
TCGA-61-2104-01A	Mast_cells_activated	0.010090688
TCGA-61-2104-01A	Eosinophils	0
TCGA-61-2104-01A	Neutrophils	0.006295042
TCGA-61-2109-01A	B_cells_naive	0.017855245
TCGA-61-2109-01A	B_cells_memory	0
TCGA-61-2109-01A	Plasma_cells	0
TCGA-61-2109-01A	T_cells_CD8	0.0295925
TCGA-61-2109-01A	T_cells_CD4_naive	0
TCGA-61-2109-01A	T_cells_CD4_memory_resting	0.169462142
TCGA-61-2109-01A	T_cells_CD4_memory_activated	0
TCGA-61-2109-01A	T_cells_follicular_helper	0
TCGA-61-2109-01A	T_cells_regulatory_(Tregs)	0.104057439
TCGA-61-2109-01A	T_cells_gamma_delta	0
TCGA-61-2109-01A	NK_cells_resting	0
TCGA-61-2109-01A	NK_cells_activated	0.090292081
TCGA-61-2109-01A	Monocytes	0
TCGA-61-2109-01A	Macrophages_M0	0.33016489
TCGA-61-2109-01A	Macrophages_M1	0.007428511

TCGA-61-2109-01A	Macrophages_M2	0.191334707
TCGA-61-2109-01A	Dendritic_cells_resting	0
TCGA-61-2109-01A	Dendritic_cells_activated	0.026659891
TCGA-61-2109-01A	Mast_cells_resting	0.027966403
TCGA-61-2109-01A	Mast_cells_activated	0
TCGA-61-2109-01A	Eosinophils	0
TCGA-61-2109-01A	Neutrophils	0.005186191
TCGA-61-2110-01A	B_cells_naive	0.011264056
TCGA-61-2110-01A	B_cells_memory	0
TCGA-61-2110-01A	Plasma_cells	0.015289977
TCGA-61-2110-01A	T_cells_CD8	0
TCGA-61-2110-01A	T_cells_CD4_naive	0
TCGA-61-2110-01A	T_cells_CD4_memory_resting	0.374763466
TCGA-61-2110-01A	T_cells_CD4_memory_activated	0
TCGA-61-2110-01A	T_cells_follicular_helper	0
TCGA-61-2110-01A	T_cells_regulatory_(Tregs)	0.090754329
TCGA-61-2110-01A	T_cells_gamma_delta	0
TCGA-61-2110-01A	NK_cells_resting	0
TCGA-61-2110-01A	NK_cells_activated	0.096514596
TCGA-61-2110-01A	Monocytes	0.072166238
TCGA-61-2110-01A	Macrophages_M0	0.132896504
TCGA-61-2110-01A	Macrophages_M1	0.019696461
TCGA-61-2110-01A	Macrophages_M2	0.167512521
TCGA-61-2110-01A	Dendritic_cells_resting	0.005551173
TCGA-61-2110-01A	Dendritic_cells_activated	0
TCGA-61-2110-01A	Mast_cells_resting	0
TCGA-61-2110-01A	Mast_cells_activated	0
TCGA-61-2110-01A	Eosinophils	0
TCGA-61-2110-01A	Neutrophils	0.01359068
TCGA-61-2111-01A	B_cells_naive	0.069281684
TCGA-61-2111-01A	B_cells_memory	0
TCGA-61-2111-01A	Plasma_cells	0.02414376
TCGA-61-2111-01A	T_cells_CD8	0.078389141

TCGA-61-2111-01A	T_cells_CD4_naive	0
TCGA-61-2111-01A	T_cells_CD4_memory_resting	0.181829363
TCGA-61-2111-01A	T_cells_CD4_memory_activated	0
TCGA-61-2111-01A	T_cells_follicular_helper	0.011779952
TCGA-61-2111-01A	T_cells_regulatory_(Tregs)	0.103449568
TCGA-61-2111-01A	T_cells_gamma_delta	0
TCGA-61-2111-01A	NK_cells_resting	0
TCGA-61-2111-01A	NK_cells_activated	0.047895147
TCGA-61-2111-01A	Monocytes	0.012228966
TCGA-61-2111-01A	Macrophages_M0	0.118958408
TCGA-61-2111-01A	Macrophages_M1	0.048282494
TCGA-61-2111-01A	Macrophages_M2	0.21814628
TCGA-61-2111-01A	Dendritic_cells_resting	0.041119389
TCGA-61-2111-01A	Dendritic_cells_activated	0
TCGA-61-2111-01A	Mast_cells_resting	0.041257089
TCGA-61-2111-01A	Mast_cells_activated	0
TCGA-61-2111-01A	Eosinophils	0
TCGA-61-2111-01A	Neutrophils	0.003238759
TCGA-VG-A8LO-01A	B_cells_naive	0.123927274
TCGA-VG-A8LO-01A	B_cells_memory	0
TCGA-VG-A8LO-01A	Plasma_cells	0.029940078
TCGA-VG-A8LO-01A	T_cells_CD8	0.059410629
TCGA-VG-A8LO-01A	T_cells_CD4_naive	0
TCGA-VG-A8LO-01A	T_cells_CD4_memory_resting	0.275399148
TCGA-VG-A8LO-01A	T_cells_CD4_memory_activated	0

TCGA-VG-A8LO-01A	T_cells_follicular_helper	0.101127642
TCGA-VG-A8LO-01A	T_cells_regulatory_(Tregs)	0.042306571
TCGA-VG-A8LO-01A	T_cells_gamma_delta	0
TCGA-VG-A8LO-01A	NK_cells_resting	0
TCGA-VG-A8LO-01A	NK_cells_activated	0.039676742
TCGA-VG-A8LO-01A	Monocytes	0
TCGA-VG-A8LO-01A	Macrophages_M0	0.090464782
TCGA-VG-A8LO-01A	Macrophages_M1	0.027557433
TCGA-VG-A8LO-01A	Macrophages_M2	0.047068964
TCGA-VG-A8LO-01A	Dendritic_cells_resting	0
TCGA-VG-A8LO-01A	Dendritic_cells_activated	0.136607984
TCGA-VG-A8LO-01A	Mast_cells_resting	0
TCGA-VG-A8LO-01A	Mast_cells_activated	0.026512752
TCGA-VG-A8LO-01A	Eosinophils	0
TCGA-VG-A8LO-01A	Neutrophils	0

Table S15: Correlations between five classes of immunomodulators and chemokines and the expression of 9 model genes

model_gene CCL1 CCL2 CCL3 CCL4 CCL5 CCL7 CCL8 CCL11 CCL13 CCL14 CCL15 CCL16 CCL17 CCL18 CCL19 CCL20 CCL21 CCL22 CCL23 CCL24 CCL25 CCL26 CCL27 CCL28 CX3CL1 CXCL1 CXCL2 CXCL3 CXCL5 CXCL6 CXCL8 CXCL9 CXCL10 CXCL11 CXCL12 CXCL13 CXCL14 CXCL16 CXCL17 XCL1 XCL2 CCR1 CCR2 CCR3 CCR4 CCR5 CCR6 CCR7 CCR8 CCR9 CCR10 CXCR1 CXCR2 CXCR3 CXCR4 CXCR5 CXCR6 XCR1 CX3CR1 B2M HLA-A HLA-B HLA-C HLA-DMA HLA-DMB HLA-DOA HLA-DOB HLA-DPA1 HLA-DPB1 HLA-DQA1 HLA-DQA2 HLA-DQB1 HLA-DRA HLA-DRB1 HLA-E HLA-F HLA-G TAP1 TAP2 TAPBP CD160 CD96 CSF1R IDO1 IL10 IL10RB KIR2DL1 KIR2DL3 LAG3 LGALS9 TGFB1 TGFBR1 TIGIT BTNL2 CD27 CD28 CD40 CD40LG CD48 CD70 CXCL12.1 CXCR4.1 ICOS IL6 IL6R LTA MICB RAET1E TMIGD2 ULBP1

FDCSP 0.188034025 0.389483588 0.35165429 0.448544161

0.451431016 0.235685556 0.274293934 0.5661117890.241147491
0.206705296 -0.091434523 0.145798535 0.370101331
0.378684156 0.579156682 0.170473993 0.409676808 0.44561284
0.296152008 0.181425478 0.021201454 0.430340947 -
0.01185593 -0.004047958 0.105373286 0.272337328 0.349074449
0.262765483 0.230887884 0.351890231 0.2411185330.445339132
0.30295158 0.301897604 0.484469161 0.535373846 0.478810947
0.098556341 0.154065674 0.142092544 0.358871322
0.359313886 0.426457542 0.255620593 0.526637472
0.452664235 0.241089889 0.46601594 0.348660768 0.113032644
0.083403838 0.105547676 0.218570284 0.408890492
0.081651722 0.321031644 0.43066117 0.366064908 0.14635921
0.247267886 0.213460601 0.26045918 0.190758698 0.198047394
0.201935219 0.185058638 0.144946749 0.29364839 0.324671902
0.350756821 0.255403138 0.262571315 0.258927735
0.207504579 0.256976987 0.236355277 0.147872522
0.195815996 0.109088564 0.108897325 0.066300034
0.412333349 0.348099564 0.212973647 0.392947647
0.043512759 0.306317897 0.28696331 0.254966957 0.122243033
0.36262674 0.16181538 0.458174274 -0.087920511 0.419252261
0.307549123 0.066828133 0.41553009 0.45150369 0.335395683
0.484469161 0.081651722 0.460186217 0.503556497
0.043645971 0.387553233 0.192418333 -0.006782531
0.368730849 0.0899848
AADAC -0.067732727 0.000855967 0.035892263 0.101639351
0.151464801 0.080762345 0.135026171 0.129839881
0.070649576 -0.140190121 -0.033973777 -0.042077439
0.080109283 0.082740735 0.1179964240.230467125 0.002265056
0.051780062 0.089408007 0.025426601 0.010880227
0.051899146 -0.040912129 0.005790912 -0.000259734
0.095895488 0.019705389 0.005759821 0.008379172
0.092289605 0.053270502 0.162085829 0.238313897
0.277204932 0.03546992 0.154176917 -0.004295791 0.081492634

0.23525597 0.063030203 0.140650133 0.1144257460.030565516
0.023175431 0.0821190550.120183641 -0.097344373 0.055633421
0.106328785 -0.115182063 -0.025450627 -0.051028825
0.024883841 0.122842612 0.237721517 0.065306397
0.1148659730.027677903 0.077606529 0.142298723 0.136907042
0.146441259 0.1311312630.1850533 0.138087951 0.099632465
0.289587144 0.155677223 0.151566053 0.160479275
0.049698885 0.134444246 0.192589438 0.205423708
0.103768674 0.065465342 0.083567204 0.150368085
0.165923508 0.173054637 -0.000764895 0.158303178
0.008324969 0.097949973 0.0608977110.194294492 0.048726687
0.083717031 0.098440551 0.034193537 -0.078920057 -
0.020195417 0.154543374 -0.087312572 0.103479775 -0.001442294
0.007557048 0.135215553 0.13623432 0.062180985 0.03546992
0.237721517 0.13051051 -0.029250062 0.280933931 0.162528543
0.017540848 0.01461521 0.045734303 0.018829308
RARRES1 0.155618545 0.400519688 0.33105846 0.403965015
0.371692244 0.284978208 0.318447605 0.331676887 0.172734
0.172712725 -0.104454037 0.099553528 0.224180415
0.1775430110.319359527 0.148501522 0.158912135 0.279308136
0.29265827 0.207516304 -0.043127454 0.302874295 -0.047840231
0.139919571 0.252253137 0.3926115590.336809476 0.281938749
0.249717154 0.47668678 0.253746883 0.29744052 0.284449694
0.278677767 0.326954718 0.290988522 0.204425768
0.198887986 0.288693483 0.037707239 0.338226222
0.432000326 0.434719278 0.271846495 0.353485441
0.435307256 0.172950983 0.3320575 0.153250544 0.092532785
0.014671943 0.193137918 0.304496093 0.383906069 -
0.032798292 0.187362821 0.36030467 0.337720718 0.304556515
0.323386128 0.2517911740.317268897 0.255840162 0.238787692
0.09094651 0.2172559110.11246701 0.295930816 0.333207043
0.35314135 0.217108985 0.307357735 0.303661203 0.295969611
0.334096302 0.29155771 0.152074515 0.204968898 0.15943236

0.177986831 0.196507334 0.336347788 0.440466718
0.282028996 0.306812679 0.107769237 0.232134587
0.3090117490.2902161180.25473134 0.418452063 0.108993344
0.306620926 -0.153055587 0.2775505110.285823478 0.050547422
0.324583968 0.442909153 0.337495943 0.326954718 -
0.032798292 0.314400534 0.406303171 0.035855394 0.308379083
0.186857131 0.144235042 0.365288619 0.072653844
DKK1 0.08209338 0.221207869 0.125839752 0.194564214
0.185279959 0.051929767 0.077432649 0.30708465 0.084037499
0.189157469 0.050653606 0.175096915 0.15328901 0.217001726
0.228066672 0.0117569740.187922581 0.215928723 0.193804309
0.118486766-0.124174195 0.287054268 -0.078107754 -0.117819104
0.146538037 0.277991775 0.19202654 0.220442988 0.178807513
0.299470766 0.1173834060.169579956 -0.006171888 0.027879464
0.384762362 0.193304788 0.3611163 0.038228056 0.051353503
-0.004825573 0.17264916 0.192828852 0.186964952 0.223155928
0.233989146 0.2110896490.134543271 0.145413699 0.049622302
0.07560245 0.051639473 0.17233381 0.144632865 0.130939597 -
0.044253794 0.106301564 0.194499258 0.109351286 0.154598044
0.096696405 0.02429259 0.088290427 0.038052456 0.038656323
0.061712003 0.087068105 -0.03137606 0.091236552 0.154619787
0.1734211470.160730023 0.120689539 0.106965454 0.061506953
0.108526317 0.032977657 -0.045722453 -0.045793188 -
0.044730778 -0.01657773 0.003250673 0.202887621 0.221421177
0.000935527 0.257824969 0.005699584 0.167160743
0.2116847250.004439554 -0.038458704 0.257428355 0.179612027
0.189828519 0.0611898760.141242775 0.144827997 0.040516367
0.162412202 0.220181239 0.129553959 0.384762362 -
0.044253794 0.170825961 0.258472875 -0.074121011 0.121006068
-0.07170912 -0.058637132 0.161843215 -0.03658332
CRYAB 0.157975489 0.212310785 0.1951188170.218626943
0.158380768 -0.009078211 0.0658420110.188693564 -0.004538746
0.066033519 -0.112639416 0.040023593 0.071482451

0.004389067 0.103830898 0.159627436 0.079205679
0.081646306 0.060404067 -0.060429503 -0.136796898
0.160761524 -0.067357951 0.1449211 -0.012285912 0.100061137
0.158864467 0.111325172 0.061408374 0.067400233 0.029590413
0.096204747 0.088761608 0.124774108 0.085288767 0.05334453
0.083306094 0.222527355 0.104282364 -0.057512919 0.21636266
0.114814521 0.080750851 0.132400392 0.116886071 0.135851047
0.016728745 0.075099161 0.15307637 0.016666199 -0.177395275
0.068672361 0.075365109 0.148362025 0.030510762
0.027154317 0.146823982 0.067188831 0.248793778
0.220206533 0.119059411 0.14949671 0.126533366 0.214283277
0.049292775 0.123356302 0.092395408 0.190243302
0.201613817 0.182156056 0.104043541 0.181305042
0.171530625 0.211266896 0.186823013 0.146099808 0.08904583
0.04654851 0.107414029 0.122364416 -0.055137756 0.142746929
0.175092062 0.123247621 0.113113869 0.204166034 0.080407504
0.164241892 0.011941985 0.109474842 0.107814361 -0.202531141
0.071158033 -0.155584442 0.126148442 0.023503998 0.038068636
0.110832691 0.1454048 0.190946887 0.085288767 0.030510762
0.106693818 0.205637493 -0.05303333 0.122596231 -0.032230675
0.003463644 0.214546306 -0.117936413
EGR1 -0.090539709 0.198290653 0.376301696 0.27252862 -0.0142983
0.009264623 0.10163825 0.11554408 0.086735977 0.236245607 -
0.009362661 0.119758668 0.038538098 0.029831733 0.015131613
0.117737354 0.140057034 0.137197326 0.053998631 0.083943488
-0.008392665 0.169761837 0.021484037 -0.055665062
0.046500635 0.224246166 0.434089203 0.302508359
0.095162235 0.18248235 0.240891702 0.006200323 -0.008736856
0.000768747 0.186672236 -0.014131843 0.176716225
0.073855795 0.074746704 -0.183023178 0.015086981
0.121404721 0.042975792 0.15194255 0.129274641 0.125100152
0.110120517 0.133590756 0.095455941 0.111981127 -0.101450878
0.281179407 0.237097352 0.02451597 -0.074579968 0.169894353

0.080488916 0.05917861 0.232412103 0.039054469 -0.058817638
0.006723918 0.038017184 0.103893863 0.04199216 0.184827959
-0.024681881 0.125583851 0.104785874 0.08541148 0.024016588
0.102408923 0.079659363 0.10110585 0.121406922 -0.010046257
0.007595293 0.017541398 0.094283431 0.1195499890.181281255
0.06297063 0.248496625 0.070344159 0.401613927 0.022389674
0.062886103 0.225968242 0.056734539 0.056946948
0.247337177 0.055382216 0.056190862 0.109450216
0.020277959 0.180408905 0.035408839 0.1121586330.017249747
0.089476642 0.186672236 -0.074579968 0.099776947 0.50921808
0.092331575 0.074240861 -0.031788522 0.11784424 0.11662931 -
0.015044572
GAS1 0.167685072 0.273387888 0.329479696 0.32188908
0.190881907 0.085494329 0.15686336 0.423152952 0.227330056
0.222770895 -0.038661096 0.064184853 0.214954313
0.271545863 0.297592424 0.04122644 0.261019033 0.268986462
0.199490957 0.210329298 -0.028251061 0.418905418
0.028015102 -0.203815228 0.129237461 0.201679576
0.217459791 0.180006647 0.13252293 0.249726302 0.183160599
0.247061759 0.085747153 0.069320355 0.536299198
0.210399157 0.384437643 0.006510958 0.020369582 -
0.006474778 0.189896265 0.283515587 0.264381393 0.151765468
0.342315065 0.281915637 0.183235082 0.26780691 0.16744096
0.026987178 0.150461083 0.188357579 0.209798438
0.256613036 0.066916716 0.186971932 0.27468931 0.166582555
0.170555523 0.083430458 -0.02900326 0.055978449 0.052971423
0.069768562 0.055668089 0.159021573 -0.007000986
0.151887969 0.210771641 0.2060111360.160738459 0.15176498
0.147172036 0.095716646 0.077957611-0.028213878 0.003125612
0.013519924 -0.031716159 -0.032932837 0.09852764 0.248679319
0.34338867 0.024772678 0.359775022 0.1116415850.196608707
0.120732552 0.100552265 -0.00990786 0.360512127 0.340895334
0.255901537 0.041937363 0.201504586 0.364077416

0.0385171170.24200906 0.284922388 0.231093354 0.536299198
0.066916716 0.257191414 0.348660664 0.025335618
0.225075459 0.004835894 -0.022550624 0.193595057
0.109912678
UBB 0.061751575 0.1911680540.159807654 0.212331696
0.238654248 0.158861264 0.210669563 0.130874472
0.159752398 0.101028139 -0.024045357 0.017577596
0.1167221750.168405679 0.149704654 0.145169278 0.121289706
0.1190309420.250391746 0.180969741 0.072986997 0.150917311-
0.037496036 0.012186036 0.033700483 0.124991471 0.126248869
0.149716274 0.147141771 0.104034329 0.154462747
0.1378111580.201049226 0.19729519 0.013346309 0.231852253
0.018371226 0.077482991 0.130768901 0.103487486
0.217007873 0.168568723 0.145548149 0.126720014
0.165889377 0.147941333 -0.012498317 0.164452875
0.054922235 -0.096847634 0.11281424 0.138910088 0.08698607
0.212421392 0.0700211420.105040856 0.205471307 0.112005784-
0.025877648 0.252505717 0.288865997 0.262353597 0.274769651
0.134002919 0.1181297060.045237183 0.1114649440.176255363
0.181490763 0.158564287 0.073297532 0.147192672 0.16916523
0.158039042 0.1911611760.266254009 0.1091127550.218639324
0.064509224 0.066247295 -0.066121147 0.155364937 0.06518002
0.198029544 0.125596233 0.092820777 0.051252229
0.106932665 0.182639205 0.127012388 0.043145279 -
0.1105789870.205518233 -0.144427764 0.212294276 0.054160036
0.096686246 0.1190381790.242107554 0.182689583 0.013346309
0.0700211420.219663006 0.120956239 0.014919295 0.197689069
0.163525647 -0.060902328 0.158399871 0.018110771
CXCL10 0.223601057 0.518664527 0.461623756 0.607390092
0.730519677 0.587489629 0.789890075 0.324423463
0.501555117-0.058679398 0.005463472 -0.018960394 0.340273191
0.301471006 0.46400756 0.300664633 0.123323083 0.452444364
0.452129423 0.273007819 0.282126234 0.175354771 -

0.073383482 0.16917486 0.203507619 0.249537486 0.203803672
0.198597987 0.163083769 0.208022709 0.269208483
0.696137668 1 0.945111939 0.043715924 0.67944527 0.052223037
0.369155986 0.285640508 0.266993196 0.609067707
0.643233655 0.551017915 0.376766455 0.513494196
0.676526928 0.216597629 0.550926018 0.461285085
0.047946565 -0.05103745 0.050697086 0.273043338 0.687061011
0.214437632 0.210893111 0.695626729 0.511644256 0.133941562
0.767282489 0.719887093 0.76970759 0.690913273 0.54787772
0.511516065 0.451806967 0.581588658 0.619008785 0.567780936
0.602794947 0.365586845 0.477674985 0.620450804
0.608327225 0.689656425 0.725047379 0.471492821
0.839814852 0.643695618 0.531006018 0.109762648
0.634319332 0.410641334 0.682380297 0.345945564 0.19711167
0.435304279 0.484659749 0.687086049 0.538575722
0.338062473 -0.027790434 0.698178031 -0.171785268
0.566495749 0.300061247 0.328169195 0.561325579
0.690702514 0.463896719 0.043715924 0.214437632
0.763996336 0.238050311 0.363674884 0.706183148 0.497995591
0.133638578 0.548530702 0.128887812

Table S16: Calculation of HALLMARK and KEGG pathway enrichment scores based on expression profiles of ovarian cancer samples

PathwayTCGA-04-1331-01A TCGA-04-1332-01A TCGA-04-1338-01A TCGA-04-1341-01A TCGA-04-1343-01A TCGA-04-1347-01A TCGA-04-1350-01A TCGA-04-1356-01A TCGA-04-1361-01A TCGA-04-1362-01A TCGA-04-1364-01A TCGA-04-1365-01A TCGA-04-1514-01A TCGA-04-1519-01A TCGA-04-1530-01A TCGA-04-1536-01A TCGA-04-1542-01A TCGA-04-1648-01A TCGA-04-1651-01A TCGA-04-1655-01A TCGA-09-0364-01A TCGA-09-0366-01A TCGA-09-0367-01A TCGA-09-0369-01A TCGA-09-1662-01A TCGA-09-1666-01A TCGA-09-1669-01A TCGA-09-1670-01A TCGA-09-1673-01A TCGA-09-2045-01A TCGA-09-2048-01A TCGA-09-2051-01A TCGA-09-2054-01A TCGA-10-0927-01A TCGA-10-0928-01A TCGA-10-0931-01A TCGA-10-0933-01A TCGA-10-0936-01A TCGA-10-0937-01A TCGA-10-0938-01A TCGA-13-0714-01A TCGA-13-0720-01A TCGA-13-0724-01A TCGA-13-0725-01A TCGA-13-0726-01A TCGA-13-0727-01A TCGA-13-0730-01A TCGA-13-0762-01A TCGA-13-0765-01A TCGA-13-0766-01A TCGA-13-0768-01A TCGA-13-0795-01A TCGA-13-0797-01A TCGA-13-0800-01A TCGA-13-0804-01A

TCGA-13-0883-01A TCGA-13-0885-01A TCGA-13-0886-01A TCGA-13-0887-01A TCGA-13-0888-01A TCGA-13-0891-01A TCGA-13-0897-01A TCGA-13-0906-01A TCGA-13-0911-01A TCGA-13-0916-01A TCGA-13-0920-01A

TCGA-13-0923-01A TCGA-13-0924-01A TCGA-13-1403-01A TCGA-13-1404-01A TCGA-13-1405-01A TCGA-13-1407-01A TCGA-13-1408-01A TCGA-13-1409-01A TCGA-13-1410-01A TCGA-13-1411-01A TCGA-13-1477-01A

TCGA-13-1483-01A TCGA-13-1485-01A TCGA-13-1487-01A TCGA-13-1488-01A TCGA-13-1489-01A TCGA-13-1492-01A TCGA-13-1495-01A TCGA-13-1496-01A TCGA-13-1497-01A TCGA-13-1498-01A TCGA-13-1499-01A

TCGA-13-1501-01A TCGA-13-1505-01A TCGA-13-1506-01A TCGA-13-1507-01A TCGA-13-1509-01A TCGA-13-1510-01A TCGA-13-1511-01A TCGA-13-1512-01A TCGA-13-2060-01A TCGA-13-A5FT-01ATCGA-20-0987-01A

TCGA-20-0991-01A TCGA-20-1682-01A TCGA-20-1683-01A TCGA-20-1686-01A TCGA-20-1687-01A TCGA-23-1022-01A TCGA-23-1023-01A TCGA-23-1024-01A TCGA-23-1027-01A TCGA-23-1028-01A TCGA-23-1030-01A

TCGA-23-1107-01A TCGA-23-1109-01A TCGA-23-1110-01A TCGA-23-1111-01A TCGA-23-1113-01A TCGA-23-1116-01A TCGA-23-1118-01A TCGA-23-1119-01A TCGA-23-1120-01A TCGA-23-1122-01A TCGA-23-1123-01A

TCGA-23-1809-01A TCGA-23-2077-01A TCGA-23-2078-01A TCGA-23-2084-01A TCGA-24-0966-01A TCGA-24-0979-01A TCGA-24-0982-01A TCGA-24-1103-01A TCGA-24-1104-01A TCGA-24-1105-01A TCGA-24-1413-01A

TCGA-24-1416-01A TCGA-24-1417-01A TCGA-24-1418-01A TCGA-24-1419-01A TCGA-24-1422-01A TCGA-24-1423-01A TCGA-24-1424-01A TCGA-24-1425-01A TCGA-24-1426-01A TCGA-24-1427-01A TCGA-24-1428-01A

TCGA-24-1430-01A TCGA-24-1431-01A TCGA-24-1434-01A TCGA-24-1435-01A TCGA-24-1464-01A TCGA-24-1467-01A TCGA-24-1469-01A TCGA-24-1470-01A TCGA-24-1471-01A TCGA-24-1474-01A TCGA-24-1544-01A

TCGA-24-1546-01A TCGA-24-1549-01A TCGA-24-1550-01A TCGA-24-1551-01A TCGA-24-1552-01A TCGA-24-1553-01A TCGA-24-1557-01A TCGA-24-1558-01A TCGA-24-1560-01A TCGA-24-1562-01A TCGA-24-1563-01A

TCGA-24-1565-01A TCGA-24-1567-01A TCGA-24-1603-01A TCGA-24-1604-01A TCGA-24-1616-01A TCGA-24-1842-01A TCGA-24-1843-01A TCGA-24-1844-01A TCGA-24-1845-01A TCGA-24-1846-01A TCGA-24-1847-01A

TCGA-24-1850-01A TCGA-24-1923-01A TCGA-24-1924-01A TCGA-24-1928-01A TCGA-24-1930-01A TCGA-24-2020-01A TCGA-24-2023-01A TCGA-24-2024-01A TCGA-24-2026-01A TCGA-24-2027-01A TCGA-24-2033-01A

TCGA-24-2035-01A TCGA-24-2036-01A TCGA-24-2038-01A TCGA-24-2254-01A TCGA-24-2261-01A TCGA-24-2262-01A TCGA-24-2267-01A TCGA-24-2271-01A TCGA-24-2280-01A TCGA-24-2281-01A TCGA-24-2288-01A

TCGA-24-2289-01A TCGA-24-2290-01A TCGA-24-2293-01A TCGA-24-2297-01A TCGA-24-2298-01A TCGA-25-1312-01A TCGA-25-1313-01A TCGA-25-1315-01A TCGA-25-1316-01A TCGA-25-1317-01A TCGA-25-1318-01A

TCGA-25-1319-01A TCGA-25-1320-01A TCGA-25-1321-01A TCGA-25-1322-01A TCGA-25-1323-01A TCGA-25-1326-01A TCGA-25-1328-01A TCGA-25-1329-01A TCGA-25-1623-01A TCGA-25-1626-01A TCGA-25-1627-01A

TCGA-25-1628-01A TCGA-25-1630-01A TCGA-25-1631-01A TCGA-25-1632-01A TCGA-25-1633-01A TCGA-25-1634-01A TCGA-25-1635-01A TCGA-25-1870-01A TCGA-25-1877-01A TCGA-25-2042-01A TCGA-25-2391-01A

TCGA-25-2392-01A TCGA-25-2393-01A TCGA-25-2396-01A TCGA-25-2398-01A TCGA-25-2399-01A TCGA-25-2400-01A TCGA-25-2401-01A TCGA-25-2404-01A TCGA-25-2409-01A TCGA-29-1688-01A TCGA-29-1690-01A

TCGA-29-1691-01A TCGA-29-1693-01A TCGA-29-1694-01A TCGA-29-1695-01A TCGA-29-1696-01A TCGA-29-1697-01A TCGA-29-1701-01A TCGA-29-1703-01A TCGA-29-1705-01A TCGA-29-1710-01A TCGA-29-1711-01A

TCGA-29-1761-01A TCGA-29-1762-01A TCGA-29-1763-01A TCGA-29-1766-01A TCGA-29-1768-01A TCGA-29-1769-01A TCGA-29-1770-01A TCGA-29-1774-01A TCGA-29-1776-01A TCGA-29-1777-01A TCGA-29-1778-01A

TCGA-29-1781-01A TCGA-29-1783-01A TCGA-29-1784-01A TCGA-29-1785-01A TCGA-29-2414-01A TCGA-29-2425-01A TCGA-29-2427-01A TCGA-29-2428-01A TCGA-29-A5NZ-01A TCGA-30-1714-01A TCGA-30-1718-01ATCGA-30-1853-01A TCGA-30-1857-01A TCGA-30-1860-01A TCGA-30-1861-01A TCGA-30-1862-01A TCGA-30-1866-01A TCGA-30-1891-01A TCGA-30-1892-01A TCGA-31-1944-01A TCGA-31-1946-01A TCGA-31-1950-01A

TCGA-31-1951-01A TCGA-31-1953-01A TCGA-31-1956-01A TCGA-31-1959-01A TCGA-36-1568-01A TCGA-36-1569-01A TCGA-36-1570-01A TCGA-36-1571-01A TCGA-36-1574-01A TCGA-36-1576-01A TCGA-36-1577-01A

TCGA-36-1580-01A TCGA-36-1581-01A TCGA-57-1582-01A TCGA-57-1583-01A TCGA-57-1584-01A TCGA-57-1585-01A TCGA-57-1586-01A TCGA-57-1993-01A TCGA-57-1994-01A TCGA-59-2348-01A TCGA-59-2350-01A

TCGA-59-2351-01A TCGA-59-2352-01A TCGA-59-2354-01A TCGA-59-2355-01A TCGA-59-2363-01A TCGA-59-A5PD-01A TCGA-5X-AA5U-01A

TCGA-61-1721-01A TCGA-61-1724-01A TCGA-61-1725-01A TCGA-61-1728-01A TCGA-61-1733-01A TCGA-61-1737-01A TCGA-61-1738-01A TCGA-61-1741-01A TCGA-61-1900-01A TCGA-61-1907-01A TCGA-61-1910-01A

TCGA-61-1911-01A TCGA-61-1914-01A TCGA-61-1918-01A TCGA-61-1919-01A TCGA-61-1995-01A TCGA-61-1998-01A TCGA-61-2000-01A TCGA-61-2002-01A TCGA-61-2003-01A TCGA-61-2008-01A TCGA-61-2009-01A

TCGA-61-2012-01A TCGA-61-2088-01A TCGA-61-2092-01A TCGA-61-2097-01A TCGA-61-2098-01A TCGA-61-2101-01A TCGA-61-2102-01A TCGA-61-2104-01A TCGA-61-2109-01A TCGA-61-2110-01A TCGA-61-2111-01A

TCGA-61-2113-01A TCGA-OY-A56Q-01A TCGA-VG-A8LO-01A TCGA-WR-A838-01A

KEGG_ABC_TRANSPORTERS 0.0022117330.027571717 0.002518406

0.007185398 0.013770877 -0.035524588 -0.071438941

0.005837186 0.055329434 0.054557754 -0.028277827

0.053713413 -0.055481297 -0.039649432 0.046890153

0.015403428 -0.008706396 0.036141335 0.024020423 -0.084569674 -0.071584584 0.045301241 0.027262336 -0.073206078

0.079906334 0.0541189980.02459673 0.028623654 0.015741367

0.07546963 -0.100724082 0.072199928 -0.025790749 -0.028008048

0.042918016 0.053537527 0.039617451 0.037998598

0.0526969110.002669768 0.007819307 0.029483706 0.045747196

0.039330439 0.0139114 -0.01613951 -0.028826075 0.016995002

0.061021693 0.027871293 0.074962607 0.068769567

0.050833024 -0.003237847 0.016755688 0.0038114820.083527677

0.079909278 0.017457955 -0.108372651 0.020730225

0.064174035 0.02762944 0.039489593 0.08348341 0.029338054

0.060617383 0.018198822 0.038184708 0.020890884

0.042640219 -0.043751552 0.012639754 -0.038179822

0.066025732 0.06944807 -0.058171464 0.028687947 0.002312555
-0.021613215 0.100340787 -0.033793485 0.056494978
0.006810162 0.082398191 0.034513224 0.073753957 -
0.000503219 -0.05197238 -0.00323466 0.010934759 0.023795059 -
0.0151132470.057755957 0.025727369 0.031408639 0.042063579
0.021764776 0.068746941 0.077406626 0.009379407 -
0.009413706 0.002252884 0.042370421 -0.100396626 0.078476678
0.055957562 0.039410731 0.048916969 0.061649326 0.0052862
0.013273915 -0.083252339 0.018058611-0.053042921 0.032995732
0.069734156 0.005991391 0.090918005 0.039627668 0.04769949
0.024450543 0.01450187 -0.011462583 0.087974052 0.019424313
0.078277961 0.03453569 0.018030146 0.006785227 0.024007434
0.080177358 0.016138377 0.102431299 -0.001792135 -
0.003136058 0.044264544 0.08614244 0.031606486 0.052503764 -
0.072065431 -0.005346642 0.044493118-0.048601519 0.036438582
0.013640982 0.0691165380.06312164 0.064353327 0.03486151
0.065846954 0.028304658 0.03210813 -0.020176887 0.02758065
0.075439205 -0.006664135 0.067359978 0.002036315
0.070617389 0.035514278 0.004263317 0.013739854
0.013884203 0.018799751 0.033795159 -0.034139253
0.030178656 0.09616513 -0.013191215 0.058010482 0.012207628
-0.018343997 0.100120172 0.090479672 0.088532303 -
0.026025022 0.010421343 0.06792994 0.019801743 0.062672221 -
0.066360261 0.040421589 0.015230466 0.059865085 -0.034768138
-0.052832765 0.034507077 -0.023493349 -0.028953639
0.0303113860.098282391 0.064483818 0.071816999 0.060473153
-0.024263053 0.065245767 0.063042902 0.023206314
0.062957337 0.0490431110.079709449 0.032201869 -0.008390139
0.088078685 -0.010038856 -0.018491135 -0.026090209
0.034764958 0.024501687 -0.014608321 -0.008469993
0.036206741 -0.004539259 0.024222498 0.003227924
0.005157405 0.067213345 0.020750163 -0.005254475
0.042906283 0.019852067 0.039056358 -0.042968549

0.025431425	-0.117632989	0.039880399	0.011605374-0.029969523
0.006362994	0.031580546	0.045666118-0.013654543	0.046560941
-0.001159994	0.062194615	0.045646414	0.043613874
0.058584932	0.051815944	0.059707383	-0.018822678
0.033385333	0.03895538 0.043736793	0.055212327	0.019427203
0.026744591	-0.010029862	0.003201652	-0.007073273
0.075747628	0.086719987	0.007221296	-0.050936608
0.033269632	-0.017237065	0.056773476	-0.010914577	0.00209449
-0.056485925	0.052692545	0.010946067	0.061076344
0.024573506	0.006323906	0.051905656	0.055634045	-
0.028653858	-0.033371517	0.06892949 0.033443813	0.018824934	-
0.02687912 0.036951047	-0.033973213	0.045090577	0.028380153	-
0.045890651	0.021392688	0.000588629	-0.018353268	-0.042966787
0.054002553	0.071237975	0.099461514	0.093381088
0.080277029	0.078585803	0.063097423	0.073464106
0.032026546	0.10354816 -0.006989449	0.0351186250.008170596	-
0.02930747 0.03323356 0.08471973 -0.013024301	0.039432816
0.078044234	0.019528926	0.064684887	0.04775824 0.062903358
0.032580039	0.050385024	0.03986308 0.039465456	0.069568427
0.071177561-0.007186268	0.020978154	-0.073243164	-0.002152751
0.04821851 0.025050454	-0.033129913	-0.019582025	0.067078559
0.043694914	0.056282843	0.0116728020.058857538	0.085075129
0.058040536	0.043052386	0.070429653	0.032802578
0.015985666	0.013718254	0.0560811710.050978631	0.033760282
0.072105954	-0.005535245	0.044949899	-0.052534417
0.064500331	0.043061257	-0.072007414	-0.00737948-0.06757825
0.129406505	0.063048466	-0.009156776	0.028838164
0.056171485	-0.028977543	0.057867805	-0.012696604
KEGG_ACUTE_MYELOID_LEUKEMIA0.2042841160.248093708
0.216312212	0.207804093	0.237992255	0.2232117370.202981727
0.2232139110.23220302 0.247668881	0.204631963	0.220438452
0.210457843	0.220239738	0.238258426	0.210249985
0.214576213	0.214030695	0.22056482 0.20718423 0.207157016

0.242768182	0.209982403	0.206333807	0.23610318 0.238734843
0.228027178	0.237376188	0.255543094	0.240624379
0.184794856	0.23856245 0.223551782	0.217770128	0.233743251
0.22614927 0.209889735	0.221683083	0.213934771	0.206376388
0.244388087	0.228090174	0.214561545	0.215293096
0.227253907	0.215210963	0.219196732	0.213082486
0.234401012	0.223281303	0.2443711950.21100538 0.233054742
0.220669183	0.206415726	0.242366936	0.235493831
0.250887915	0.19769077 0.210258375	0.210555438	0.237065208
0.208334159	0.233187818	0.224922466	0.200297153
0.207900895	0.235587483	0.233853278	0.206331786
0.258393384	0.196918978	0.23225683 0.19423519 0.240897757
0.265972886	0.193202736	0.227506284	0.2311087710.212621624
0.220684515	0.192139199	0.210327642	0.238636543
0.214618688	0.209419812	0.246307149	0.245894978
0.243913412	0.23933722 0.229238963	0.2040721130.219075286
0.208682779	0.236584749	0.221663153	0.209580257
0.228942127	0.24458025 0.2411764870.244715281	0.262496348
0.1757496110.247701499	0.209968399	0.227285691	0.223691342
0.222450876	0.224336246	0.226555003	0.218182153	0.23469411
0.201078148	0.228645401	0.22044478 0.2540113170.233393512
0.202071378	0.230303139	0.240135101	0.236992035
0.202290546	0.230909498	0.249984636	0.26103156 0.19851366
0.235172964	0.218089572	0.203499797	0.237412374
0.225712401	0.225843712	0.227357604	0.250976032
0.2428081130.223868756	0.247235314	0.232202149	0.23230618
0.260399875	0.212082867	0.26081157 0.235223221	0.234481305
0.221634499	0.246948605	0.245918061	0.214360564
0.223638425	0.238003746	0.2411678940.237759521	0.251896108
0.212766556	0.249863069	0.238570089	0.239726839
0.242597482	0.22268268 0.25527161 0.217887267	0.230895127
0.229487099	0.248107949	0.223055287	0.219012476
0.213540969	0.237059372	0.234683527	0.222764363

0.250539929 0.242590415 0.216130209 0.224158337

0.234393553 0.2611684370.23386386 0.235643 0.228517925

0.245018973 0.240921837 0.199577554 0.245425239

0.203343143 0.230943984 0.2061165910.206946022 0.243153511

0.212506944 0.2301161180.235155544 0.252734761 0.24863523

0.262202325 0.248107622 0.253498317 0.229654089

0.252870131 0.265825695 0.247836652 0.269833767

0.214548356 0.25298476 0.232507065 0.244903937 0.229590195

0.205942401 0.22435415 0.233821013 0.233086417 0.259969471

0.225071659 0.25421147 0.22440865 0.235182348 0.250266812

0.254229237 0.261455365 0.268377874 0.241230905

0.256270979 0.241726271 0.234715483 0.208000685

0.259871833 0.201795423 0.2595565 0.229660129 0.187487954

0.280148564 0.228988422 0.249214895 0.238421056

0.232272242 0.248758863 0.253000734 0.241725078

0.263978525 0.244830427 0.234315816 0.236139986

0.235645505 0.219124197 0.204756763 0.237724705

0.247654399 0.191357284 0.216683228 0.206575531

0.231693483 0.244867685 0.247272665 0.245314166

0.223920394 0.20561607 0.2359161180.214899667 0.252774716

0.202663504 0.218681429 0.183336889 0.226185377

0.208038235 0.234175224 0.2118424840.247142955 0.206785835

0.230366599 0.22829928 0.213583617 0.235626809 0.220931777

0.234381376 0.17871686 0.223036358 0.196799621 0.227974063

0.223805775 0.216034378 0.232291056 0.206837415

0.239404936 0.210402726 0.238516014 0.2418261130.244153812

0.247422602 0.26033113 0.242672336 0.2511920390.250478379

0.257752685 0.218069713 0.225580016 0.230216958

0.240971645 0.231449009 0.2384021120.235475427 0.222866413

0.201077999 0.2315781140.251962513 0.209986079 0.256352609

0.253478645 0.244304413 0.230526144 0.249320589

0.248922681 0.259064036 0.234784729 0.233447726

0.197982352 0.232138509 0.259402318 0.246268567

0.206461038 0.21785945 0.230713557 0.23870832 0.214960161
0.238040824 0.241988414 0.227920965 0.202122242
0.235437648 0.213136935 0.242641998 0.227258594
0.267228639 0.236140861 0.236917694 0.247266057
0.253832517 0.25677439 0.2049291190.23891481 0.234672036
0.191485993 0.2557216 0.217007784 0.218028664 0.227610407
0.24530102 0.231246965 0.238458213 0.215725684 0.264950439
0.2115833210.219934751 0.216444171

KEGG_ADHERENS_JUNCTION0.2799112350.296104597 0.308623394
0.269255478 0.272406905 0.28357673 0.294833492 0.283747073
0.290504779 0.303397661 0.271271955 0.288355066
0.318881236 0.283702778 0.2911166430.287235789 0.288467056
0.284800839 0.288637021 0.298269884 0.320175201
0.283095882 0.272509683 0.288993741 0.310845007
0.290136012 0.289469883 0.284938447 0.288447487
0.297439713 0.265626197 0.271457557 0.302492279
0.2811289650.313999539 0.252444191 0.312367392 0.248609283
0.258173721 0.267269047 0.301673388 0.272640029
0.275014888 0.273037514 0.275097407 0.268807822
0.286161606 0.2802112 0.256463188 0.267802827 0.283078805
0.269807496 0.267883144 0.294374321 0.272252027
0.313121475 0.285332075 0.287866959 0.248599104
0.306725207 0.264541901 0.282653927 0.3081136260.261469134
0.279235236 0.286764187 0.252959655 0.289663168
0.284867604 0.280374288 0.293598678 0.290301842
0.292667624 0.292269973 0.291054193 0.315847387
0.295206234 0.290839259 0.296008424 0.303969928 0.26266798
0.268410975 0.298700195 0.2842110450.2801133880.260238339
0.273255372 0.316075867 0.297418319 0.2993155 0.273406379
0.264744148 0.286550768 0.273870602 0.304822143
0.303061971 0.292207536 0.302996638 0.270380607
0.250872243 0.281021602 0.259299181 0.280652077
0.292260706 0.284565616 0.293023283 0.269010649

0.259629434 0.284994157 0.300797374 0.256490144

0.282226542 0.2970921 0.304837823 0.276191372 0.29709375

0.267669219 0.278153351 0.297803621 0.291847265

0.279793477 0.282794024 0.283981416 0.294706502

0.2714541140.240667574 0.288788419 0.301726383 0.271092381

0.270715695 0.293753764 0.29293199 0.257704086 0.291663909

0.30300509 0.300633525 0.300635737 0.295206263 0.32651176

0.310335803 0.272289994 0.329207543 0.264837499 0.30925995

0.278872533 0.3115515940.290474189 0.282452324 0.278942204

0.288568657 0.293289431 0.274651644 0.300054794

0.283577351 0.286947745 0.286237864 0.313398334

0.307708968 0.282737648 0.307632303 0.289079505

0.291807225 0.264352622 0.29772006 0.284618183 0.273001988

0.304174629 0.270269895 0.298388707 0.294020734

0.283612633 0.279383452 0.26825369 0.260148409 0.274244125

0.290702351 0.290839692 0.290007572 0.27006161 0.292834664

0.287588818 0.290401649 0.289006299 0.29288494 0.29125639

0.285819257 0.300923747 0.291779807 0.284996405

0.305376812 0.310810812 0.298749273 0.28144903 0.293455491

0.287346683 0.31419502 0.286075978 0.301278213 0.296163124

0.285179091 0.298450037 0.278770173 0.293952857

0.298710256 0.315200613 0.288362307 0.299248153

0.3115471860.298715044 0.281903595 0.295189831 0.296335889

0.2897614 0.304475861 0.282105158 0.322501917 0.286246067

0.314737206 0.312164578 0.305679788 0.310085321

0.289156231 0.295872732 0.297920658 0.345963256

0.315268821 0.298445145 0.29233487 0.264809686 0.328526081

0.291517591 0.305448037 0.300128182 0.259427061

0.312819329 0.296254904 0.305547357 0.304234706

0.2963511870.294627757 0.264397629 0.290522168 0.286256393

0.263770012 0.285346796 0.297108665 0.247436736

0.266832807 0.243698262 0.293891568 0.296100582

0.305583544 0.289906306 0.257075124 0.283265555

0.310356725	0.275684692	0.291353932	0.280989427
0.283508412	0.286109044	0.303994397	0.253578988	0.30110329
0.263226839	0.30662809 0.245360262	0.286331614	0.308919869
0.295969966	0.2716119150.268484546	0.327264437	0.263302747
0.278048246	0.279706181	0.283868708	0.263581466
0.290370769	0.279862084	0.275218384	0.3027793	0.282808959
0.298501029	0.290263076	0.297317684	0.294168474	0.29963846
0.284513084	0.303907424	0.293427529	0.30589387 0.272848164
0.289050173	0.268032634	0.314137036	0.287975327
0.294579388	0.284957948	0.292066871	0.2628011860.283015058
0.309615519	0.2895117790.294393607	0.298487894	0.284614875
0.269639688	0.284885281	0.318406839	0.298395623
0.290791854	0.309040132	0.290538415	0.324429432	0.29886118
0.271544664	0.264265474	0.274902128	0.281622766
0.281041937	0.255008588	0.308741819	0.3117480820.264724615
0.271424904	0.26905483 0.258684665	0.265953862	0.299400325
0.288009967	0.294577475	0.274975432	0.281287451
0.307000769	0.2821119270.273924886	0.27955709 0.288322567
0.25646848 0.293574347	0.3216234	0.299325424	0.30400762
0.295637086	0.291955915	0.291443667	0.323069048
0.320270489	0.326785938	0.301376704	0.291392008
KEGG_ADIPOCYTOKINE_SIGNALING_PATHWAY 0.105278027
0.121771426	0.121630021	0.105017036	0.177576219
0.1143888820.080284202	0.155328209	0.100057763	0.159715892
0.085192434	0.1485491130.071000216	0.1184564080.171646336
0.141604255	0.127920177	0.125683326	0.104520708
0.090175642	0.098867033	0.127363059	0.140104291	0.09513118
0.184386477	0.125651826	0.124957987	0.1183757080.119734213
0.149307984	0.1114868810.134689662	0.102648795	0.10667867
0.17237395 0.137882883	0.081841839	0.09977239 0.130559377
0.090107788	0.12283729 0.090159786	0.160577912	0.084321441
0.080803192	0.129151488	0.167343396	0.104881513
0.158618078	0.098927429	0.1294117480.120773822	0.139213991

0.080006924 0.134174392 0.151898957 0.157537693

0.125512997 0.137793899 0.106650969 0.1161511270.107929971

0.144292552 0.169437744 0.1184361350.095157072 0.107244026

0.137453715 0.16289865 0.124057977 0.124091026 0.105302046

0.11567285 0.1174304140.1175367760.17133909 0.096313853

0.139554937 0.1142728540.12506844 0.160825432 0.104541091

0.1173130120.104590787 0.148586479 0.140438878 0.160196794

0.1148259810.07133886 0.102859089 0.147104077 0.133132804

0.1128029870.090297926 0.173431903 0.105706226 0.098270075

0.160489273 0.1130235150.170794394 0.105232387 0.106413109

0.085500767 0.1101764230.1177395540.136893436 0.10657208

0.173134614 0.141081725 0.146710275 0.1154190110.16125225

0.089588216 0.1185385940.1165922010.164162417 0.136885085

0.10981086 0.137569882 0.124839033 0.1102534010.125820789

0.1176181670.105241233 0.157040437 0.083236082 0.159585912

0.128052936 0.1122759970.11285277 0.0977321120.124849537

0.076733044 0.174278482 0.136216931 0.104900474

0.109386546 0.159995523 0.1255416 0.146995245 0.111121986

0.1449641150.171250442 0.107292028 0.129474705 0.12396466

0.1511986860.1128352430.093743005 0.161026362 0.131318787

0.147972484 0.127641041 0.104795704 0.160216576

0.126562265 0.1131665240.1680691150.134319415 0.161713002

0.125835278 0.131412781 0.094569936 0.180324942

0.165928519 0.101234072 0.108862243 0.153258365

0.143930371 0.125747532 0.13110361 0.1524117180.083667172

0.157127507 0.186789386 0.1411415390.147956158 0.157254133

0.152451792 0.095546683 0.160693735 0.09271743 0.150090664

0.123250779 0.149006668 0.105297161 0.1149957470.11708188

0.098388847 0.154591259 0.169759636 0.1469553110.17861108

0.146814385 0.156793464 0.122575346 0.172618616

0.156876531 0.143135796 0.202584188 0.1859119290.11751829

0.138144455 0.15603385 0.126918916 0.072024208 0.110776078

0.133172718 0.13778145 0.100228685 0.135420646 0.139296525

0.157649882 0.1139422910.139469376 0.129677091 0.112293381
0.181774393 0.1820321150.151620055 0.128831208 0.157621233
0.1381119920.132046816 0.129569178 0.11450212 0.108828157
0.149378412 0.108334743 0.121856234 0.144278383
0.146968264 0.140543037 0.13904746 0.182375224 0.134677955
0.104137762 0.1465118810.133208629 0.1171357080.146484645
0.128910582 0.1011397080.123302808 0.1101089780.190750065
0.096743418 0.109683496 0.139992961 0.122413894
0.145377396 0.175654671 0.139456807 0.130187502
0.1399116780.1155175470.132131067 0.108918463 0.130337356
0.162263668 0.10845727 0.10857269 0.138020041 0.113120993
0.142306287 0.1149802230.13897224 0.14133109 0.105548858
0.084094168 0.120129752 0.132901749 0.1151701210.144877297
0.136250146 0.0782112740.135353905 0.129743498 0.09198659
0.129510515 0.123559746 0.1110133290.0936711060.124066211
0.130904652 0.132677719 0.125884512 0.165361825
0.185752323 0.173495813 0.14199045 0.137458741 0.102509383
0.090054107 0.159752203 0.17048245 0.139952885 0.169214616
0.1132410850.1184354820.151436563 0.1107698520.172587319
0.1115611880.126692958 0.137361299 0.1151923080.126741665
0.1170205550.150540796 0.120655553 0.124672318 0.164814249
0.138052397 0.1411882450.132203953 0.126316142 0.111103085
0.131649478 0.089173392 0.189758253 0.1183817950.118500188
0.12833032 0.170633196 0.149552003 0.123972962 0.148098995
0.144069364 0.167389792 0.1125293490.163173969 0.161005589
0.1539111170.185848077 0.158718186 0.121670162 0.180059798
0.1140563930.105231615 0.189255104 0.109014434 0.104158156
0.10045278 0.172594971 0.159826022 0.12139156 0.155358047
0.127446638 0.1102300610.120921886 0.113368596

KEGG_ALANINE_ASPARTATE_AND_GLUTAMATE_METABOLISM
0.095467324 0.096777807 0.1103837950.146267752 0.14009414
0.139683924 0.11875075 0.1123165250.127406537 0.082901439
0.138814488 0.131208199 0.101429288 0.153470705

0.139209896 0.14141625 0.131698612 0.139974995 0.128880349
0.1071191510.165756688 0.120424336 0.154166021 0.133412371
0.130776864 0.148717282 0.1162084760.155083125 0.146328742
0.1128883580.105296063 0.14012361 0.101937253 0.10884116
0.154024582 0.158479814 0.083213914 0.157092096
0.138432508 0.12374487 0.12675897 0.14737674 0.140898965
0.136854262 0.121292074 0.1173097040.1190653090.130282357
0.149729723 0.181764699 0.149259062 0.146065213
0.152642104 0.122229532 0.145717886 0.1116930530.130663091
0.139796476 0.154231358 0.1011900780.159734795 0.135735498
0.139626469 0.1176127810.169225218 0.151238169 0.181637937
0.147573603 0.164140457 0.108584609 0.109229496
0.139806096 0.1188728710.13345051 0.134827196 0.113515504
0.102473218 0.136787196 0.1177713320.134908659 0.145152457
0.143399337 0.104279391 0.164410403 0.1132391840.134072917
0.135666715 0.101264763 0.105346034 0.1421116420.16426283
0.131849193 0.127595552 0.16883494 0.146279654 0.123683544
0.142746603 0.1156492380.148146092 0.143300376 0.115781047
0.126643981 0.130636984 0.123436583 0.127139762
0.138190225 0.156073469 0.144168588 0.134351777 0.1261464
0.163908913 0.100830821 0.1104391740.1136031230.117989154
0.1186794020.144047256 0.148339107 0.101947874 0.12539596
0.123479047 0.13829776 0.135365258 0.105941296 0.128476707
0.1405531110.153485681 0.121085418 0.138485712 0.124588475
0.146298289 0.1219555110.1190185380.129034191 0.117622347
0.139609886 0.1297211450.129985402 0.1153327070.126124392
0.101430681 0.1113721950.163392668 0.129181565 0.165403214
0.104061649 0.12273885 0.131440765 0.13889966 0.13143191
0.141886355 0.150317816 0.123925545 0.1441137220.173187913
0.159534942 0.099222348 0.147866532 0.123224258
0.134343919 0.146902805 0.14623729 0.137786347 0.144528426
0.123409397 0.132577046 0.087412412 0.15324342 0.146459067
0.147867865 0.156702924 0.157240799 0.123430401

0.161375536 0.129157469 0.1105052080.126873874 0.120380644
0.1631147740.133237654 0.121004414 0.16146912 0.159181518
0.1279411180.147430062 0.11752138 0.140535149 0.119785182
0.100218389 0.12218208 0.1130458790.148658716 0.130899931
0.150297383 0.130521093 0.1101483230.167196874 0.128906839
0.11797273 0.146953981 0.124561649 0.131299658 0.126760316
0.143187597 0.1194785910.1134924820.130771878 0.114713203
0.142056208 0.14545386 0.1182747050.123942381 0.126175297
0.130074741 0.139827009 0.108626774 0.1125438730.131142973
0.104924583 0.1219311210.104271227 0.137502744 0.130395846
0.107157814 0.100343267 0.103373905 0.1189893920.155430895
0.123354521 0.090213175 0.1158334480.1176936380.140316835
0.15233514 0.1178272410.156845409 0.168713503 0.13758278
0.1196926170.139338362 0.138672157 0.1106508270.114017125
0.152681549 0.137124665 0.144623903 0.139503876
0.171347947 0.154229418 0.14843467 0.134065729 0.115239663
0.122001357 0.163669686 0.124413076 0.098486392
0.126870095 0.136687993 0.138342763 0.152384914
0.123650905 0.1196820880.146940007 0.144530083 0.12347463
0.122737457 0.162798079 0.134364333 0.1147024770.130081491
0.161294727 0.136635526 0.1184787260.136829085 0.136914442
0.136249828 0.109830217 0.159845605 0.1165920120.145630752
0.125503871 0.139018629 0.1143189670.124163096 0.138658331
0.161262399 0.1181889 0.151460608 0.131088278 0.135823773
0.1128361050.1160811190.149705593 0.136991656 0.131544857
0.121000825 0.1182526130.145558003 0.1233511410.155749294
0.140786893 0.130400302 0.128435526 0.127647921
0.144520019 0.106381297 0.156136726 0.143786059
0.147674047 0.1172079680.1111876230.1140118190.088566353
0.155194819 0.1140196670.060369183 0.132416357 0.147956527
0.143024451 0.103770972 0.109494395 0.163215652
0.123402284 0.07058172 0.154758069 0.146901792 0.142707647
0.141486651 0.156588861 0.135123933 0.141443253

0.137823358 0.152582307 0.145097562 0.099919442
0.144392948 0.168539805 0.167984648 0.084052503
0.220034396 0.11908958 0.093762089 0.134397674 0.1034704
0.144322445 0.162543423 0.145590508 0.122314085
0.134987216 0.13865997 0.170582265 0.158199516
KEGG_ALDOSTERONE_REGULATED_SODIUM_REABSORPTION
0.060550254 0.084784459 0.067683799 0.052470775
0.0652051160.039520346 0.081382409 0.0455011910.072454598
0.06984901 0.078308409 0.067878569 0.058180304 0.040072036
0.09186801 0.06987587 0.068460608 0.078768679 0.070023248
0.090989161 0.041814229 0.074002873 0.077710256
0.033477138 0.066605798 0.047988514 0.063817562
0.0887342110.068797701 0.066340009 0.01760297 0.061482154
0.045284492 0.067094045 0.084208467 0.058183204
0.047059598 0.025963958 0.029328094 0.034838133
0.076757677 0.073402177 0.05802174 0.072806871 0.033802741
0.051770679 0.043797075 0.0753911870.078593543 0.022651644
0.083663194 0.062040138 0.059470351 0.059882762
0.056310409 0.040949714 0.0511146410.061990499 0.045559031
0.0641115860.079259606 0.065707952 0.060962738 0.074782667
0.094123048 0.034778278 0.034749864 0.058010618
0.055582076 0.064829987 0.070158359 0.050733508
0.043708438 0.087580028 0.0380117990.0728891110.055387104
0.056486028 0.030919198 0.090285391 0.0525011740.046860306
0.050821568 0.06278729 0.0414112020.044649679 0.078571345
0.047703691 0.063808377 0.068230357 0.049502628
0.021932178 0.042541796 0.084633316 0.09841526 0.04984428
0.027432158 0.071510669 0.0593183110.073474791 0.117009459
0.0561162140.069595248 0.09453002 0.076686979 0.085717603
0.02925561 0.039897478 0.079143056 0.055638503 0.061993876
0.037800038 0.051377791 0.028047813 0.061433892
0.061484379 0.060087239 0.0591117840.063289864 0.069236481
0.042224763 0.044255016 0.06695415 0.075903549 0.063619844

0.008076306 0.059901388 0.074439726 0.038012296
0.096309607 0.086362783 0.065123178 0.062988467
0.071345995 0.060844059 0.08669931 0.055651925 0.076295729
0.053248869 0.074246482 0.063119281 0.099819868 0.045859207
0.074535458 0.05787576 0.059858227 0.057784634 0.040809429
0.055965703 0.058975554 0.090296441 0.0723604 0.071633394
0.062344039 0.056853614 0.047932787 0.055162164
0.104857728 0.056830008 0.106667632 0.081336952
0.079460877 0.058821091 0.075712985 0.07296694 0.029357892
0.09539055 0.06557442 0.051400738 0.074489296 0.092474001
0.059952549 0.037557547 0.042593601 0.070883315
0.084429201 0.068204017 0.111102532 0.052813231 0.043314734
0.064581743 0.040250806 0.058068147 0.057138949
0.075859227 0.060181603 0.07094699 0.076093336 0.058835379
0.055246856 0.079220351 0.076381354 0.065887528
0.082191652 0.087287922 0.054473331 0.062642924
0.072365701 0.073125799 0.092131323 0.084607759
0.057059822 0.07715151 0.081396422 0.066417133 0.05387748
0.048220511 0.087816456 0.106302368 0.061761332 0.07717826
0.064979623 0.086514391 0.085274492 0.068070009
0.052699593 0.073171274 0.084321363 0.099391649
0.076355273 0.071234581 0.064672064 0.082333808
0.065125396 0.055679426 0.059292005 0.073857059
0.090977038 -0.003749715 0.11163246 0.070637736 0.071116243
0.064014151 0.059317438 0.068172006 0.075307478
0.078435428 0.052425503 0.072799033 0.034638934
0.053400324 0.059354577 0.080712507 0.022811915 0.081219886
0.074860488 0.035716745 0.025993697 0.039338451
0.085689936 0.064833778 0.0584654 0.040649731 0.073440567
0.062752133 0.048332829 0.063431258 0.059811118 0.041916207
0.077603625 0.056724687 0.065415018 0.036330551
0.054289186 0.06153604 0.066451375 0.06301608 0.031164286
0.052833736 0.035875235 0.060367272 0.084455949 0.07592612

0.052534083 0.02819678 0.085028616 0.057884913 0.08971112
0.018697751 0.040279318 0.038003111 0.057715871 0.044121424
0.060333734 0.074358031 0.090380878 0.102899165
0.089813545 0.085972897 0.089657391 0.059003928
0.093351267 0.056902289 0.072030623 0.056558789 0.08491385
0.062951499 0.084583277 0.076358283 0.077687707 0.07746233
0.07306011 0.087171229 0.058396218 0.063143499 0.080810179
0.073146663 0.066226553 0.064950489 0.090063151
0.068081945 0.065573592 0.043801324 0.045918858
0.063856119 0.111144505 0.088943222 0.020428547 0.071970481
0.032169318 0.061207174 0.048284309 0.06772993 0.081438545
0.036477105 0.051429768 0.078557438 0.039858463
0.074767767 0.046001321 0.08579414 0.085550978 0.073312125
0.06724392 0.071890739 0.080419617 0.064029919 0.062186966
0.054545106 0.062916366 0.08575619 0.038021265 0.064743052
0.041619996 0.062118566 0.077022917 0.075930307 0.132333539
0.063488321 0.091979238 0.061539213 0.075979213
KEGG_ALLOGRAFT_REJECTION 0.186564295 0.275426892
0.351410676 0.240030468 0.346543638 0.193539384
0.156547177 0.264720203 0.376942663 0.280089647
0.130765361 0.391156345 0.050917897 0.268216709 0.381377835
0.29142914 0.34838976 0.426982009 0.27945138 0.251087341
0.045166958 0.348926364 0.386818405 0.184718182
0.384649466 0.367276514 0.337483349 0.325060493
0.291643539 0.307400282 0.225261722 0.393289587
0.193013514 0.262250223 0.320265312 0.324977863 0.1932522
0.343667223 0.394680794 0.11915638 0.299149338 0.249685027
0.32423661 0.335552815 0.275393507 0.298344033 0.302761823
0.25515313 0.40710869 0.308773723 0.395727153 0.409866741
0.333454107 0.101936145 0.386076911 0.355499786 0.416021949
0.408435465 0.381258308 0.143568467 0.374144053
0.373379606 0.261558934 0.40436013 0.441330453 0.280615639
0.343214098 0.376172977 0.348452878 0.350791509

0.286965051 0.128819399 0.290607191 0.245888093

0.298616567 0.402879922 0.210221039 0.373217602 0.26040538

0.263945092 0.423038576 0.159534976 0.207586583

0.268494439 0.437186517 0.267630308 0.452834322

0.367848153 0.241403342 0.335460988 0.324443853

0.395647349 0.306484252 0.413567893 0.323829677

0.321289375 0.382606367 0.275057957 0.39652077 0.425776728

0.287671791 0.242328751 0.222226641 0.392574562

0.195073149 0.40913722 0.247043948 0.367939477 0.412713268

0.333928582 0.286354108 0.350046271 0.058455362

0.166580484 0.227743336 0.360297249 0.370807462

0.349622315 0.393556735 0.268440267 0.33881341 0.183877823

0.380483884 0.291516357 0.301841516 0.33879846 0.352752474

0.3024117710.223020997 0.343762391 0.308105175 0.369348173

0.279716176 0.394814693 0.285385304 0.208443248

0.371774563 0.353573045 0.235320606 0.335683324

0.258058489 0.347705552 0.381344356 0.227863716

0.321852942 0.376457087 0.386545814 0.334047345

0.292457533 0.324142631 0.4114588630.338058842 0.323835129

0.1622119370.353527571 0.420922695 0.301052996 0.406687836

0.202247654 0.422268464 0.310395517 0.247637143

0.253418133 0.364579135 0.405700766 0.403773022

0.201286422 0.381084179 0.325691754 0.271867404

0.378133316 0.353543233 0.237678651 0.315335023

0.429848974 0.401398657 0.332658128 0.349487573

0.402543125 0.248460698 0.3995119090.1162946910.313474047

0.375969316 0.36664408 0.1631103940.3511485620.348469959

0.210542953 0.300446633 0.33752707 0.408452716 0.319610865

0.387532646 0.372204177 0.2643491170.410334054 0.331214431

0.32446301 0.332770818 0.374342769 0.27963658 0.307513766

0.337126048 0.348950724 0.11854219 0.319028042 0.233540164

0.32039565 0.378454455 0.294727405 0.29088407 0.255277243

0.185263121 0.340320222 0.343466801 0.208837697

0.378121854	0.360048657	0.260201812	0.348621242
0.336471024	0.298265524	0.199685841	0.367647491
0.163645999	0.323412764	0.380780265	0.312363316
0.300209725	0.386743305	0.360314032	0.305168399	0.34246545
0.357664558	0.373475416	0.292805708	0.361111375 0.315972389
0.376671539	0.359780171	0.374420368	0.343299151
0.341743288	0.370733854	0.365284873	0.336601465	0.16337557
0.348766251	0.319887427	0.348885674	0.441798855	0.38863845
0.366135382	0.188083669	0.34486629 0.324274929	0.39394482
0.292921297	0.318641438	0.123175288	0.31785845 0.358655517
0.327314026	0.402129197	0.340439598	0.406701544
0.423349245	0.298880631	0.235748518	0.456377502
0.381308669	0.208059821	0.363614371	0.303292755
0.192637799	0.38512597 0.409470431	0.242989607	0.28477236
0.316226027	0.331207787	0.263889353	0.298534623
0.371336576	0.415337197	0.350461441	0.416265466
0.471336692	0.37078743 0.360432657	0.380354549	0.37927134
0.145310101	0.358776001	0.35676337 0.177989684	0.393767923
0.396559177	0.251898572	0.238713992	0.356091793
0.378028898	0.322433053	0.280328479	0.385282168	0.26479013
0.290889118 0.330727555	0.344928268	0.384557189	0.33102031
0.224970844	0.23816551 0.137114983 0.303654618	0.278249146
0.370498098	0.324705582	0.258023365	0.373204519
0.389961303	0.320711551 0.228746771	0.441360094	0.277969858
0.363217684	0.345957354	0.393310582	0.395639859
0.359839315	0.345631409	0.411511296 0.434020312	0.357869843
0.382575757	0.360784036	0.378425592	0.121929078
0.268260061	0.322321217	0.087346121	0.287870763
0.199370194	0.422364932	0.343634467	0.242481563
0.302952404	0.296752176	0.136131535	0.212281464	0.20090308
KEGG_ALPHA_LINOLENIC_ACID_METABOLISM -0.118321668	-
0.084330168	-0.108323423	-0.123496018	-0.116549592	-0.119024019
-0.132086638	-0.099931659	-0.161591579	-0.155940364	-

0.084234984	-0.109824802	-0.183141522	-0.123218193	-0.101374392
	-0.160577918	-0.145214795	-0.086901307	-0.14658895 -0.160726714
	-0.126506263	-0.141043616	-0.119668868	-0.099999288	-
0.117870035-0.142582846	-0.144992388	-0.176430739	-0.062285029	-
0.116278845-0.148380189	-0.146688539	-0.117594587	-0.046573564	-
0.149433712	-0.086314139	-0.148709828	-0.105484855	-0.141462542
	-0.146839333	-0.09833989 -0.101458315	-0.120062267	-0.133498989
	-0.111142444	-0.168944945	-0.13795147 -0.060502335	-0.149721703
	-0.102121754	-0.064739604	-0.166863125	-0.136505154	-
0.043978999	-0.134685788	-0.176077028	-0.143858908	-0.131418712
	-0.143955556	-0.100606275	-0.108698934	-0.159967341	-
0.10165031 -0.111775565	-0.127864776	-0.094891614	-0.085298552	-
0.082413729	-0.138099339	-0.121082463	-0.113439403	-0.105850335
	-0.146369776	-0.148971303	-0.13574256 -0.095220246	-0.08261097 -
0.123077568	-0.147278532	-0.132146226	-0.066713921	-0.140785822
	-0.104430266	-0.168126365	-0.129767874	-0.130941191	-
0.151167097-0.055898326	-0.118651507	-0.082069171	-0.155471269	-
0.118060515-0.169776831	-0.13989251 -0.093275539	-0.152207929	-
0.077802285	-0.156960604	-0.03578044 -0.057096745	-0.113433687	-
0.08800842 0.009709856	-0.121908163	-0.175768034	-0.053667401	-
0.132239154	-0.150500197	-0.097670119	-0.093791794	-0.104759849
	-0.114282243	-0.130137812	-0.134747209	-0.169825098	-
0.11665645 -0.111296645	-0.105794589	-0.093347769	-0.110952703	-
0.138988348	-0.190763688	-0.123684394	-0.088915778	-0.143176181
	-0.118700295	-0.158617253	-0.108919289	-0.117440336	-
0.116980123-0.084043603	-0.156391723	-0.113023425	-0.178666821	-
0.128670435	-0.10798298 -0.169001203	-0.148170647	-0.168976527	-
0.151507578	-0.168196419	-0.149654164	-0.11579192 -0.10758945 -
0.101467233	-0.159697254	-0.1561864 -0.14579041 -0.112209578	-
0.134807726	-0.106509683	-0.157752245	-0.108415299	-0.136306995
	-0.108503225	-0.11821055 -0.134140917	-0.116516862	-0.156310787
	-0.114207888	-0.148971601	-0.099953224	-0.119454893	-
0.145337408	-0.136446369	-0.110101185	-0.128079555	-0.155422433

-0.163979984	-0.176942351	-0.137186756	-0.125376365	-
0.112698086-0.148536489	-0.175968701	-0.109089252	-0.105400035	-
0.099051162-0.126513044	-0.146288355	-0.173334326	-0.047339348	-
0.18637358	-0.130738499	-0.137280193	-0.173423862	-0.11622082	-
0.053460598	-0.103404939	-0.16862224	-0.128023005	-0.129565307	-
0.100110512-0.112452469	-0.121464529	-0.088240617	-0.060343868	-
0.152975999	-0.086202154	-0.147496622	-0.114867607	-0.143432017
-0.12638733	-0.134353437	-0.137009371	-0.109630942	-0.150113564
-0.09513276	-0.14805388	-0.083958554	-0.131162948	-0.164882944	-
0.144944706	-0.177439918	-0.128069076	-0.120073171	-0.180415646
-0.12761476	-0.154709603	-0.113292442	-0.14409929	-0.100457267	-
0.179626617	-0.147501145	-0.084495393	-0.153674681	-0.139172325
-0.156275928	-0.114455429	-0.124043005	-0.142305259	-
0.138802713	-0.109771579	-0.102732402	-0.098512771	-0.116611764
-0.123589869	-0.121152314	-0.103610859	-0.124285515	-
0.124277744	-0.154115267	-0.086825049	-0.096589327	-0.127914156
-0.116150548	-0.136306784	-0.174762454	-0.140399103	-
0.127508019	-0.149594489	-0.138371874	-0.124492658	-0.131669821
-0.118957831	-0.120685707	-0.104706275	-0.070559124	-
0.097020337	-0.158313317	-0.128167824	-0.1825258	-0.175671044	-
0.080032708	-0.11039618	-0.124745584	-0.11109478	-0.149220786	-
0.116420763-0.091163298	-0.110494055	-0.129855419	-0.113993834	-
0.11721804	-0.129849356	-0.125031185	-0.096118908	-0.080108636	-
0.170237666	-0.132266882	-0.095334062	-0.133508688	-0.124235359
-0.086453218	-0.128536012	-0.147278798	-0.151719766	-
0.131693595	-0.105665865	-0.133913002	-0.131212147	-0.118381147
-0.130442632	-0.118193958	-0.109701174	-0.125245328	-0.1459108
-0.135141736	-0.057173326	-0.130404054	-0.082934149	-
0.171097843	-0.092898797	-0.110966521	-0.134358611	-0.134185388
-0.122654675	-0.161268778	-0.086593096	-0.120931307	-
0.116096795-0.140667061	-0.169391957	-0.084345699	-0.111007291	-
0.051360732	-0.130816338	-0.157713077	-0.164735376	-0.149058729
-0.160555524	-0.112797327	-0.12045558	-0.138922825	-0.132752814

-0.1117615 -0.128644569 -0.091321947 -0.094786346 -0.091120684
-0.143583037 -0.110668171 -0.112914922 -0.083614 -0.112416133
-0.155555694 -0.184792989 -0.123258921 -0.076615793 -
0.100776341 -0.127406005 -0.126649615 -0.078080565 -0.084242058
-0.152721764 -0.170019396 -0.175844815 -0.110978312 -
0.122017599 -0.109112579 -0.157615257 -0.138506374
KEGG_ALZHEIMERS_DISEASE 0.290004714 0.284062643
0.323605168 0.307399236 0.332127107 0.2933511960.313539787
0.329699524 0.301685241 0.306029438 0.297460853
0.305734927 0.25955014 0.285950278 0.301084924 0.315156077
0.302712154 0.284513253 0.303210658 0.300822689
0.269024937 0.284043582 0.328950761 0.308929291
0.312294253 0.3102186 0.291244693 0.296813268 0.259105455
0.303796548 0.310281428 0.294093801 0.267686697
0.293954588 0.317250684 0.314816556 0.297771317
0.286003121 0.328802756 0.293613658 0.297476629
0.294558386 0.316432291 0.305238976 0.309006136
0.301341237 0.288382349 0.300948983 0.334012679
0.309237137 0.285276956 0.313794553 0.315258554
0.280802125 0.3117606990.302994706 0.318161566 0.300772615
0.323759121 0.294343313 0.3141139810.308177098 0.309634128
0.323753329 0.281748275 0.308671027 0.312286717
0.324870262 0.316788094 0.305842954 0.297850041
0.3113831660.304776372 0.308800666 0.30297056 0.309284173
0.308079826 0.324147654 0.286766158 0.312767922
0.321577732 0.3079711590.288776558 0.302584656 0.309302203
0.316664762 0.317883017 0.297161757 0.321366415
0.283715469 0.31515703 0.320551683 0.31917964 0.297040505
0.305165005 0.309038893 0.291206426 0.312899181
0.296440802 0.315273525 0.302513869 0.288850308
0.303969817 0.307898832 0.3119365590.304355908 0.287782315
0.316597049 0.319984443 0.298325818 0.327282579
0.308796209 0.287967469 0.287992234 0.300480992 0.31524116

0.320656761 0.319522183 0.31248412 0.29843544 0.283957257
0.306136003 0.29539557 0.302318106 0.300660475 0.309724279
0.317336588 0.315234283 0.30480335 0.313601628 0.30497948
0.291854292 0.305391697 0.304538601 0.2958112020.290289395
0.299749302 0.3116970170.278050456 0.279177667 0.294091256
0.293589785 0.308328498 0.283268929 0.307692477 0.29306068
0.31056199 0.305318469 0.252285 0.307001542 0.291894195
0.314556171 0.27074771 0.286239134 0.299126038 0.261895225
0.284040042 0.310222797 0.295193966 0.2968118940.298381337
0.287766944 0.297079863 0.32777047 0.317610663 0.292557512
0.281303771 0.312404449 0.279674025 0.298074212
0.296842964 0.324094712 0.299979877 0.314427718
0.3118571290.293474819 0.304997004 0.313167957 0.315615881
0.290050229 0.3229113610.289043944 0.3011512990.301424845
0.297783766 0.285701327 0.296678952 0.288474336
0.282410908 0.29712283 0.281771021 0.277423606 0.319798239
0.292328733 0.30696062 0.277918697 0.300661719 0.298760604
0.289273123 0.308829894 0.293970999 0.299850222
0.302759265 0.303548773 0.314876271 0.298034027
0.288575931 0.309178016 0.294294849 0.277651872
0.288531561 0.296459241 0.286863005 0.301846939
0.297722928 0.27659651 0.278754582 0.299562749 0.293119499
0.305490185 0.275979012 0.307847924 0.293750875
0.295775473 0.292405909 0.295333177 0.277330752
0.284159149 0.298490248 0.286883036 0.310093126
0.298880358 0.300346373 0.301688424 0.2911302240.288575408
0.27394398 0.3063211960.298132809 0.292080707 0.317271777
0.300372062 0.28194568 0.323644766 0.29708953 0.308893333
0.303653546 0.302694454 0.319056937 0.293205137
0.3118628590.297246168 0.299937579 0.320767887 0.315407057
0.28054148 0.3331139380.313388424 0.312458365 0.321295007
0.296199774 0.288476983 0.327228359 0.276323345
0.320025985 0.294648829 0.312286376 0.308160531

0.286104165 0.298106495 0.31206277 0.310740625 0.235177091 0.334653107 0.306629487 0.306490546 0.315855857 0.318453195 0.29721248 0.29251325 0.319819153 0.317464009 0.314003498 0.291882146 0.288318379 0.274965183 0.287600945 0.304756836 0.32404991 0.305437037 0.303922179 0.293035244 0.303073877 0.278237215 0.300121719 0.318171873 0.285667697 0.313763294 0.276314054 0.292536927 0.302879957 0.294529085 0.30079168 0.282815986 0.280695103 0.274679385 0.285651827 0.313450392 0.297748476 0.286532121 0.2949757110.309318219 0.304695398 0.291329662 0.270021727 0.294168343 0.288190071 0.308561609 0.299292224 0.29122634 0.321886563 0.300757767 0.275320248 0.285425151 0.3251101180.310277513 0.32492134 0.3241101250.289781 0.334207784 0.29829445 0.299040688 0.31183676 0.302223267 0.298055494 0.31495126 0.333235929 0.309621503 0.243604352 0.301785516 0.294256235 0.278013913 0.303413682 0.2733896 0.276710532 0.305031057 0.303808588 0.282660044 0.274154369 0.257032332 0.2888076 0.274543055

KEGG_AMINO_SUGAR_AND_NUCLEOTIDE_SUGAR_METABOLISM

0.157918625 0.186582134 0.214200691 0.188067252 0.207059333 0.185456637 0.1911611630.196605644 0.203594119 0.144195835 0.205332468 0.198050587 0.132938051 0.1811160980.197741513 0.207728064 0.203054587 0.227873882 0.19574008 0.175883521 0.16013325 0.220053306 0.212072321 0.220325748 0.178354157 0.1994381140.177364558 0.183005352 0.207596825 0.201252496 0.176575995 0.226421484 0.190345961 0.166446712 0.167878506 0.222673526 0.164715466 0.188108754 0.2069026 0.172265267 0.209927057 0.212602424 0.2113902680.219093718 0.229536001 0.185965854 0.198910246 0.180997712 0.243967898 0.244620962 0.210336864 0.22432809 0.219867377 0.18075215 0.197218253 0.195023328 0.199593508 0.21338675 0.218812031 0.169460679

0.215242335 0.229031572 0.184249862 0.243067326

0.186143256 0.201720652 0.23453322 0.235477091 0.202628674

0.196179529 0.2149385110.155164625 0.195199077 0.152508539

0.199987456 0.219834736 0.168772238 0.208699152

0.186942504 0.192129408 0.205378357 0.205826546

0.180252317 0.202088577 0.206670679 0.19940877 0.19843389

0.18722649 0.195655792 0.18381948 0.208520978 0.23945823

0.208981413 0.206079771 0.210971694 0.216752986

0.2113761480.151044991 0.257682261 0.258773288 0.203221307

0.199309914 0.222669926 0.206826459 0.177332915

0.208340786 0.207105894 0.229747822 0.2116790440.18949667

0.244451069 0.1722565110.168869169 0.190301085 0.198698256

0.218448388 0.199516041 0.218240496 0.178124262

0.181001247 0.184178677 0.195956827 0.224367758

0.195753905 0.209989212 0.202123146 0.198289838

0.164177426 0.185755615 0.203583927 0.205412326

0.182172852 0.190326353 0.20448429 0.171460403 0.191663713

0.202859996 0.192929008 0.1646114020.194744385 0.178236662

0.1766521110.224361247 0.16646126 0.2176014110.206833619

0.190615983 0.203230565 0.180247473 0.169255882

0.173255712 0.201039509 0.203334463 0.213836544

0.2119771830.214609545 0.220569582 0.207806102 0.228298271

0.193937627 0.178417475 0.182155891 0.2119950540.207484028

0.206608565 0.182805471 0.1828389 0.200638943 0.189569164

0.181224464 0.230301453 0.21242525 0.18768845 0.213218386

0.209035899 0.225527693 0.223691902 0.180757554

0.214376015 0.1956181110.21058867 0.2076363 0.216011489

0.189854003 0.22795265 0.169538657 0.201298485 0.186897539

0.149756338 0.17195632 0.175341882 0.193617196 0.210263489

0.215364394 0.192746793 0.200422814 0.206521477

0.190485356 0.205059748 0.216066868 0.219780513

0.185916425 0.195646154 0.191662626 0.197244523

0.198454353 0.1481118460.165549077 0.189932905 0.184132612

0.202754132	0.180808396	0.186619888	0.182169601
0.221729963	0.178263214	0.193708325	0.212329557
0.205246679	0.189121691	0.179967954	0.230544284
0.188654163	0.170047808	0.187378798	0.138473572
0.207089553	0.199517849	0.240266179	0.207689129
0.214523192	0.200407059	0.188720408	0.19995139 0.187694484
0.226866046	0.171565266	0.2115427020.21086762 0.221021877
0.208951973	0.213029679	0.206150842	0.217496979
0.205458541	0.215864261	0.213914494	0.188691642
0.214878829	0.198700582	0.193238407	0.206709091
0.214147219	0.245247837	0.195424322	0.183376993
0.2110415780.230283279	0.2177692 0.207820657	0.196280482
0.187829124	0.235820868	0.179564771	0.236289025
0.194889672	0.221008518	0.20672955 0.168792127	0.176586214
0.234769796	0.201749388	0.146034816	0.213074497
0.220795562	0.177950139	0.208163635	0.228250722
0.198695927	0.207603537	0.184742361	0.2110723410.198736154
0.221074831	0.22515354 0.20618914 0.2030118890.208389688
0.236138942	0.209315277	0.1804472110.179666624	0.202971904
0.196727359	0.2289380110.214251412	0.212551535	0.207030626
0.238301329	0.218697589	0.185388174	0.215557604
0.213857422	0.184129407	0.187801347	0.1754075110.19963919
0.199379298	0.217871945	0.179551778	0.207559963
0.177656732	0.161314359	0.218755068	0.172500067	0.20530525
0.215301697	0.208748429	0.178344302	0.195245475
0.199862943	0.239566639	0.174191017	0.17143818 0.220562006
0.17471839 0.222354058	0.228940904	0.20581408 0.209393583
0.2118160160.21341189 0.209121496	0.213994414	0.206552635
0.203918589	0.230574532	0.220867164	0.176682244	0.27007208
0.214285184	0.169447751	0.187235534	0.192392794
0.208840471	0.1926011540.186028465	0.194308329	0.193402039
0.153069682	0.182363714	0.183572669
KEGG_AMINOACYL_TRNA_BIOSYNTHESIS	0.275904353	0.279067011

0.267319579 0.297132186 0.279322838 0.343001708 0.27497455
0.289462171 0.272936651 0.238210803 0.307301539
0.2706066110.310109454 0.267523422 0.281725313 0.286449027
0.31768708 0.248059683 0.255036666 0.29659139 0.374656033
0.282221986 0.294015429 0.320142879 0.3030451140.290570914
0.285781593 0.302868007 0.322908504 0.258450759
0.328176633 0.294066378 0.318436442 0.310491521
0.297969908 0.31735919 0.286541956 0.281078056 0.284034377
0.325442842 0.300522634 0.259046646 0.3114711940.271751117
0.275768201 0.273855328 0.275343917 0.304843566
0.301413957 0.313217753 0.256138525 0.294453909
0.280151866 0.344469729 0.285942791 0.275936225
0.300788702 0.30562021 0.314461098 0.270130012 0.288139911
0.304546001 0.265248819 0.259080061 0.336961055
0.315338345 0.32279677 0.303419825 0.295697824 0.2803648
0.251594765 0.305154297 0.276750568 0.306567961
0.293639307 0.299648328 0.256193594 0.321577606
0.259352763 0.303825905 0.291068463 0.319999026
0.258239349 0.273181491 0.270361762 0.30111834 0.310067555
0.242037883 0.259799095 0.288315653 0.290745645
0.235755195 0.258108362 0.281508882 0.282975674
0.306428881 0.309428258 0.320153329 0.3058958110.29191904
0.220589803 0.267068067 0.275207128 0.274286404
0.289318392 0.286434164 0.358374452 0.302252653
0.281765277 0.329410289 0.2954331120.248799832 0.318253946
0.286177004 0.2462011040.280857499 0.288386808 0.273157148
0.291250836 0.282738741 0.274416054 0.274004105
0.299543276 0.270676236 0.29020001 0.327538673 0.325049065
0.3198225110.245847236 0.268828667 0.309557785 0.274569817
0.2343116970.301539122 0.286224173 0.26970488 0.275547206
0.262616105 0.315557893 0.290043519 0.272777292 0.26576326
0.313787141 0.295086434 0.31730183 0.243561338 0.266296799
0.35118235 0.288660039 0.279903395 0.2611044590.283601864

0.323051319 0.303586523 0.280060928 0.28619585 0.251559327
0.31684154 0.286399401 0.254077377 0.259289162 0.304706898
0.288668066 0.290903856 0.267822189 0.294654624
0.272537922 0.315244731 0.331349329 0.278323688
0.289703757 0.304300891 0.280538554 0.319100447
0.304426804 0.258688407 0.2672117710.226779142 0.283253024
0.276553003 0.27423958 0.270961435 0.331888022 0.299228858
0.315607286 0.249161767 0.306430066 0.219750533
0.307415806 0.2514313 0.287485714 0.289707067 0.260981751
0.293435846 0.245547723 0.317900038 0.320238984
0.295202947 0.293346685 0.299232134 0.28229098 0.291739171
0.252872649 0.309185775 0.2696881180.268806571 0.262866803
0.291446151 0.270771963 0.278390587 0.294831071
0.293629902 0.244533516 0.297764564 0.278083739
0.269689528 0.251586235 0.282120343 0.238256147
0.287485443 0.243714304 0.271877244 0.289602038
0.3112946740.276917973 0.299935045 0.261555456 0.304331999
0.304259843 0.2414901 0.284372413 0.297428355 0.314369825
0.251583473 0.281994771 0.310284305 0.299154858 0.30061733
0.2827555110.27542088 0.281448486 0.252362648 0.261394244
0.273630123 0.248103162 0.304904207 0.257805698
0.2819546110.234733555 0.273830795 0.278500497 0.279426485
0.256364721 0.293532723 0.265178871 0.301907926
0.262712662 0.26141037 0.244098725 0.283284829 0.315285105
0.272044079 0.243848418 0.3119592850.256155072 0.279872425
0.283014449 0.274629191 0.308929982 0.303005598
0.284456633 0.248591341 0.295210892 0.298275076
0.294694169 0.307780142 0.251806216 0.249841686
0.291934629 0.273814322 0.242312382 0.245287043
0.234308642 0.297143086 0.295472304 0.320753359
0.2611642670.304457108 0.26619208 0.265886883 0.301026519
0.306784482 0.343063352 0.307577123 0.276355165
0.292558042 0.297016274 0.299646409 0.297388235

0.338196076 0.308125547 0.286122643 0.264692793 0.31291038
0.288600791 0.274496598 0.269483502 0.26354463 0.301737823
0.283939186 0.273772075 0.299953297 0.307223164
0.305297802 0.33458491 0.202289682 0.261519223 0.303263082
0.254334201 0.269685874 0.28464607 0.267501838 0.303673845
0.268513719 0.276284101 0.343405278 0.2370011930.269812251
0.3110346040.292668058 0.285989432 0.290762602 0.298951868
0.282188803 0.287960145 0.30616642 0.29601708 0.27809512
0.301888667 0.36742307 0.26199497 0.321701958 0.273208498
0.303049384 0.30275773 0.293560588 0.277368703 0.300260172
0.308880314 0.32346247 0.30277528 0.339716458

KEGG_AMYOTROPHIC_LATERAL_SCLEROSIS_ALS 0.122832974
0.148001749 0.162903143 0.147061305 0.202358257
0.121734856 0.131795831 0.210203169 0.147312439
0.202167861 0.147938334 0.194175819 0.1178000180.124542182
0.212629506 0.170685999 0.150793457 0.14653834 0.147311024
0.127618557 0.12755121 0.1444118220.166329613 0.135930957
0.223847191 0.159807539 0.150614903 0.136545213
0.124778342 0.177302609 0.158941469 0.183350048
0.124133658 0.108181401 0.20071539 0.16424974 0.145723813
0.123618597 0.18234054 0.1165901010.142486532 0.128296696
0.2022937 0.132161774 0.1163305580.153776989 0.151479139
0.150662907 0.20921874 0.132748542 0.150527897 0.159864362
0.156217649 0.1370811660.15819169 0.188362489 0.190109824
0.147844378 0.168244547 0.122112 0.155705694 0.138151766
0.161743913 0.21534545 0.148562885 0.1301125430.141631318
0.17636597 0.200301735 0.154512392 0.14323896 0.166682856
0.140900121 0.133261021 0.1521131270.196926841 0.127841798
0.186802003 0.142651955 0.161033307 0.192860686
0.160249777 0.157749264 0.136958779 0.180142219
0.194624168 0.197373648 0.13810941 0.1387911620.135656591
0.187789631 0.169324101 0.171473129 0.122736172
0.178825014 0.1570011370.158542656 0.188799803 0.157348755

0.197161029 0.135467885 0.130627639 0.1187055710.13989785
0.140830549 0.171387055 0.128795382 0.205414158
0.198365194 0.185335094 0.149394399 0.193775663
0.122200068 0.133161352 0.131479803 0.2091127180.192878855
0.166166694 0.198901889 0.148010664 0.135313774
0.184802905 0.164438755 0.139446351 0.182886346
0.121500386 0.181808223 0.155024598 0.131262723
0.133186874 0.130766716 0.134033784 0.133786216
0.200942306 0.1662911910.152147193 0.160642037 0.173150535
0.172052666 0.132514771 0.144006356 0.141792088
0.184023738 0.125161292 0.13480116 0.1631311620.199667158
0.162339941 0.124579467 0.190712196 0.134146042
0.142302427 0.147320754 0.122828684 0.170481262
0.149747665 0.14382905 0.209242362 0.166359418 0.171917233
0.169368231 0.155846825 0.133210129 0.223025504
0.213649686 0.137413126 0.155182092 0.176568365
0.152339821 0.149819394 0.144857749 0.209521746
0.127748482 0.187610801 0.213314526 0.181441718 0.15730828
0.203392239 0.181728168 0.140931205 0.184184645 0.13394467
0.17595278 0.168924522 0.187014703 0.1171955350.153843526
0.124770147 0.125827034 0.160464465 0.203426857 0.16556054
0.209427825 0.159813648 0.178751088 0.126793917
0.201022061 0.163707713 0.170679874 0.210703004
0.213573804 0.1364168 0.1199082890.176201323 0.161247458
0.11456323 0.127837754 0.164368397 0.148450415 0.142552103
0.166313061 0.166249967 0.166206178 0.145378519
0.159809707 0.139677774 0.132542357 0.198471719
0.200465122 0.162840152 0.144853878 0.196061054
0.144218844 0.165233486 0.1672091150.128561755 0.136275887
0.149248968 0.170854898 0.129970545 0.184907573 0.15952243
0.175885132 0.155390539 0.2140511050.160149425 0.140303271
0.187012862 0.155256519 0.139138561 0.190978187
0.1640381120.122262729 0.156062964 0.134367425 0.218806787

0.120884845 0.132720143 0.173417297 0.140837939
0.161941507 0.223033931 0.182029487 0.156874693
0.194274142 0.142199994 0.191847325 0.144536219
0.156793259 0.177319445 0.139767437 0.13838595 0.171521759
0.138424596 0.181678593 0.132790603 0.187077008
0.180495903 0.122347019 0.1123170220.166915577 0.164744309
0.158679859 0.202035246 0.149131271 0.105669254
0.160571901 0.180665898 0.147332548 0.13306424 0.158716117
0.138322792 0.1177712590.142744002 0.155132907 0.162747237
0.15502979 0.191789912 0.228526983 0.220955815 0.181379749
0.139207144 0.13664439 0.11347563 0.2047886110.204090616
0.143251035 0.1878827 0.148024431 0.166364795 0.160796435
0.160086746 0.19436663 0.146740009 0.159350458 0.159169661
0.135796585 0.166286921 0.138294557 0.177955745
0.1568512110.158638192 0.192929613 0.200489079 0.161606774
0.137002748 0.146969376 0.128419752 0.168373419
0.1133082150.224469039 0.159293827 0.151800636 0.147415018
0.2111919360.163124629 0.170497433 0.193001993 0.163193685
0.209154022 0.149413737 0.18979191 0.196474863 0.176954685
0.213487942 0.196055252 0.193323314 0.215415091
0.135651423 0.144610412 0.220769382 0.13683329 0.128825439
0.1381155180.200585259 0.1864298 0.1666071120.175055464
0.146987844 0.133459098 0.125860738 0.138291164
KEGG_ANTIGEN_PROCESSING_AND_PRESENTATION -0.039615069
0.000735125 0.050069699 -0.017119752 0.02443961 -0.044210726
-0.064538229 -0.004130964 0.075335391 0.016520629 -
0.071583819 0.09001587 -0.097686457 -0.006423834 0.064002659
0.016144903 0.034053141 0.087504203 0.013739864 -
0.026930838 -0.125164934 0.0591162530.073660096 -0.051983597
0.068736597 0.06130138 0.046999844 0.040151548 0.021162461
0.030623101 -0.031213063 0.088795576 -0.04005063 -0.003847359
0.036172589 0.044462933 -0.019599246 0.044135521
0.089133785 -0.075912165 0.041557095 0.001306301

0.025004437	0.042449295	0.037974775	0.002594166
0.002623603	-0.000709265	0.0726853110.017894286	0.090940776
0.074472474	0.042203138	-0.062462855	0.079326934
0.030562712	0.09421884 0.094903312	0.051548611-0.084565047
0.05878648 0.098381861	0.010618261	0.070181424	0.107086576
0.019837994	0.067986027	0.062776292	0.036794619
0.041488615	0.049819119-0.067029779	0.015096641	-0.008530705
0.035418322	0.086154367	-0.038576808	0.051615811-0.005334746
-0.014132612	0.095478715	-0.053422457	-0.019194959
0.008103528	0.1109993620.0110528310.1123326760.062042695	-
0.017013316	0.034916686	0.032241487	0.080915102	0.014376391
0.092524058	0.031539142	0.049608613	0.071795253	0.00410181
0.134160032	0.10190838 0.020865879	-0.015898944	-0.018635676
0.070635626	-0.057829572	0.083895834	0.00416982 0.045618258
0.095316772	0.044157292	0.006127653	0.033108362	-
0.119453437-0.043336271	-0.03417122 0.041789174	0.06522174
0.046852553	0.089682379	0.017178824	0.067790499	-
0.044223085	0.054516122	0.01090205 0.034797403	0.047026172
0.05530716 0.026608109	-0.008679465	0.072889665	0.031737712
0.069859556	0.018626539	0.101267439	0.003390778	-
0.030360195	0.099986431	0.060027146	-0.004974167	0.05410598 -
0.014013399	0.036530854	0.079616628	-0.025145235	0.048608192
0.061081317	0.066536764	0.054676892	0.025705522
0.041243869	0.097817348	0.039462848	0.040258531	-
0.0471311660.043915667	0.127625084	0.025206546	0.102741435	-
0.03292405 0.09948935 0.033133281	-0.009313357	0.010042989
0.052738148	0.061072799	0.101016182	-0.029030646
0.051585203	0.055084213	0.00258025 0.089338987	0.042734363
-0.014324464	0.03982053 0.097812308	0.084487244	0.036374999
0.031826904	0.076246378	-0.001453398	0.074707761	-
0.088246344	0.039043049	0.051919225	0.066862958	-0.063769216
0.024713243	0.055047356	-0.025908885	0.010629936
0.028274489	0.096153298	0.046729376	0.077465448

0.053817717 0.009928825 0.083609568 0.04279343 0.032651662
0.027726895 0.050950293 0.023954796 0.029002251
0.030859526 0.056519353 -0.066769631 0.039329912 -
0.018430863 0.02144529 0.069854326 0.008975458 0.016292165 -
0.000148313 -0.039801861 0.041645464 0.039547942 -0.019988841
0.077010485 0.040957551 -0.007807225 0.051040455
0.032297492 0.024143169 -0.040683912 0.057394725 -
0.075697106 0.035363413 0.064338403 0.015823703 0.022828869
0.059696698 0.062751298 0.013889222 0.057660296
0.028877213 0.084033033 0.024314156 0.061958618
0.038361448 0.083973787 0.069495002 0.058177832
0.043730259 0.048409025 0.058656978 0.047421379
0.045296421 -0.037229389 0.028163016 0.028260069
0.039487359 0.13310903 0.087839025 0.052015823 -0.053756094
0.055186714 0.016135353 0.080710098 0.016193848
0.020284236 -0.07492949 0.029704846 0.04359621 0.054435414
0.070042023 0.046882038 0.080677094 0.121031938
0.013585256 -0.019333587 0.127320612 0.061663489 -
0.025797469 0.0231173220.030463678 -0.04944349 0.067761634
0.10688402 -0.020363593 0.017174059 0.036382131 0.033858976
-0.009929287 0.030785743 0.1161722260.097968628 0.071647536
0.121659665 0.11646491 0.047915608 0.061009613 0.066162273
0.077571029 -0.046402686 0.058799823 0.035625472 -
0.044743849 0.072839283 0.09459634 -0.011598439 -0.011727579
0.060614539 0.049773142 0.044376502 0.01493286 0.074105197
0.019744856 0.015730432 0.044080246 0.054102441
0.092630454 0.050992592 -0.031309293 -0.019357785 -
0.0731186570.017626794 0.018676186 0.066698179 0.022585769 -
0.0080106110.056020943 0.066807924 0.034801167-0.012616541
0.089157193 0.007016287 0.086156543 0.037226565
0.081484934 0.05912075 0.047303473 0.02910807 0.087668152
0.094414739 0.0456211030.066349547 0.041246008 0.060405314
-0.077591975 0.000185227 0.014677461 -0.101421483

0.010645979	-0.050107382	0.098784934	0.048273729	-
0.015150782	0.018203822	0.02965458	-0.071901421	-0.01953016	-
0.021869897
KEGG_APOPTOSIS	0.148875262	0.146646805	0.160235779
0.127949654	0.194966753	0.097166122	0.13302062	0.204682515
0.152386022	0.202285477	0.101300307	0.184147223
0.111743418	0.151348728	0.184552918	0.177017301	0.14169171
0.161731219	0.158193362	0.128395541	0.087661358
0.166256139	0.165757082	0.109186482	0.217926934
0.184793207	0.163407264	0.161643801	0.129212125
0.197497106	0.136728109	0.180865567	0.102560163
0.114084464	0.181193186	0.163772441	0.110125396	0.134324267
0.180718727	0.099144903	0.15283635	0.16570204	0.173945203
0.136138777	0.15320629	0.142060744	0.126420379	0.13244685
0.207706127	0.138988183	0.158421964	0.152354924	0.18440271
0.105769713	0.160257844	0.195461119	0.188297618	0.166673121
0.169545921	0.097966201	0.14581538	0.132508066	0.171024256
0.196270635	0.1308297	0.1194392	0.136572981	0.166165504
0.20474403	0.151972368	0.180251212	0.123398481	0.148738602
0.120576445	0.155354095	0.198781274	0.153258073
0.192871583	0.16750545	0.156407296	0.191832482	0.140518424
0.110720086	0.173202317	0.185593907	0.179000454	0.202330354
0.146331158	0.152066561	0.130651906	0.183606632	0.170837215
0.161609961	0.142965251	0.18501348	0.150905059	0.147999033
0.166982477	0.160961318	0.185938313	0.15018868	0.148177596
0.131326519	0.168789865	0.133454255	0.16592689	0.145571863
0.200728024	0.210917493	0.192907025	0.148396615
0.184028043	0.090416066	0.14686285	0.136083448	0.186380684
0.197473089	0.150005374	0.19274453	0.15713273	0.162140775
0.167414743	0.161170093	0.167075175	0.180076268	0.134371029
0.168292837	0.15642637	0.146666314	0.175920007	0.127937157
0.143226653	0.150054067	0.196222403	0.166890165
0.143674335	0.163724143	0.19311609	0.164239524	0.16780937

0.158121981 0.158433153 0.200713931 0.139244588

0.158875218 0.179145561 0.202369965 0.14458221 0.148704686

0.18542264 0.169319254 0.185907794 0.138525866 0.090815152

0.179840068 0.171916996 0.143361776 0.2174311040.161998991

0.1946011340.166343845 0.161938504 0.161674773 0.205123384

0.202169541 0.167512863 0.124505775 0.188902313

0.162178602 0.159006553 0.164454095 0.188208356

0.134470992 0.186379872 0.216754373 0.208874459

0.156052335 0.206715544 0.189784884 0.144756412

0.193521956 0.099680773 0.198866552 0.170059564

0.198757383 0.125614482 0.132062443 0.178500766

0.139972623 0.183364004 0.212302046 0.167096395

0.233095787 0.193427405 0.199308905 0.137106765

0.187531361 0.17140965 0.19621657 0.216924896 0.219230477

0.152210883 0.152680356 0.152831965 0.179361257

0.124249963 0.147987389 0.150855041 0.161564595

0.1491374110.169956475 0.160590842 0.18710519 0.126758397

0.172905734 0.182305467 0.139652274 0.1997728110.209516746

0.147839597 0.156024352 0.209741235 0.156854105

0.1710011960.156599518 0.109645371 0.152258178 0.158508182

0.1154301650.168196317 0.181859354 0.160430723 0.158599684

0.18277043 0.188507998 0.170754152 0.132827082 0.18208087

0.160033914 0.17017831 0.191215876 0.17803851 0.134165063

0.171893654 0.181510932 0.2119340420.145291809 0.157111907

0.163720659 0.144363593 0.177410108 0.209877755

0.198645702 0.1483625 0.166180816 0.150368593 0.167650754

0.159657949 0.165349982 0.185422189 0.1126741810.156311371

0.171914451 0.145428096 0.177317592 0.14904693 0.163411109

0.183680918 0.1065411060.144423697 0.182150446 0.163596474

0.152905484 0.192278887 0.164376134 0.124576282

0.183907613 0.195880723 0.139882023 0.158918757

0.162704508 0.161304533 0.155126848 0.155550079

0.156772734 0.164542366 0.184071452 0.204934312

0.234293343 0.219862895 0.18965147 0.170369823 0.148082009
0.107845356 0.203505183 0.185999722 0.133999718
0.182610965 0.167969027 0.144177747 0.171718042
0.170133979 0.195987326 0.134962361 0.149706344
0.163327518 0.160831701 0.182153193 0.156927431
0.166355629 0.16282774 0.16311866 0.162747508 0.17566606
0.158041959 0.165970743 0.170030828 0.1362592 0.175416676
0.142549626 0.208633812 0.158163609 0.141492058
0.127907259 0.197780013 0.164013554 0.1711847150.185601694
0.168641259 0.201352529 0.172993479 0.1832606110.202028357
0.194728108 0.2116911010.192770096 0.166852922 0.210498443
0.130796537 0.162004379 0.233812543 0.123512651 0.14200379
0.1115150310.205122981 0.191332091 0.161835779 0.151604296
0.160473074 0.104123006 0.154794513 0.129800597
KEGG_ARACHIDONIC_ACID_METABOLISM -0.035755365 -0.008051338
-0.007271434 -0.016159346 0.012213123 -0.066953773 -
0.055756571 -0.029695367 -0.045398551 -0.044030986 -0.055363205
-0.041084329 -0.089953002 -0.019198954 -0.032620115 -
0.049783283 -0.045805818 -0.006710723 -0.03415261 -0.08310767 -
0.089284824 0.011026025-0.018830588 -0.049743385 -0.04009177 -
0.046202535 -0.056238564 -0.03686847 -0.049660441 -0.035402216 -
0.079425485 -0.048185825 -0.063958226 -0.020838274 -0.057079942
-0.01297749 -0.097422412 -0.005308483 -0.010875471 -0.069899883
-0.013344407 -0.004279053 -0.029769586 -0.00233553 -0.031633327
-0.019017053 -0.056007624 -0.009555996 -0.046924765 -
0.038619319 7.29E-05 -0.024465824 -0.042593541 -0.039724914 -
0.023713487 -0.046832832 -0.03758873 -0.025982213 -0.036974573 -
0.076806132 -0.017758144 -0.034060894 -0.044627482 0.005229073
-0.040239007 -0.057532521 -0.004707345 0.001181847-0.027873526
-0.03862969 -0.019253904 -0.071965727 -0.050885693 -0.06133493 -
0.051685725 -0.022609065 -0.033079706 -0.051558876 -0.0312904 -
0.053881201 -0.006326762 -0.068190718 -0.054425121 -0.047021155
-0.050334047 -0.047842511 -0.044272902 -0.02026946 -0.031922261

-0.011809674 -0.03089669 -0.043870502 -0.042624745 -0.023584491
-0.028738121 -0.056305315 -0.044645199 -0.08438163 0.025860396
-0.004512347 0.000374391 -0.05212693 0.013442626 -0.008919764
-0.064335677 0.007851864 -0.045763821 -0.036053373 -
0.018058911 -0.018968905 -0.031597908 -0.016943566 -0.087654591 -
0.029722969 -0.049780259 -0.018228771 -0.051648345 -0.027799616
-0.041266066 -0.036427377 -0.034838207 -0.071367829 -
0.039155441 -0.03145004 -0.064261052 -0.017999839 -0.040383617 -
0.056012403 -0.053518212 -0.027213251 -0.009614544 -0.044286747
-0.011502102 -0.041399625 -0.055349575 -0.027517522 -
0.042698638 -0.042321786 -0.063649804 -0.030280344 -0.049147904
-0.030315182 -0.03810422 -0.05657524 -0.054807628 -0.0377507 -
0.036480636 -0.057273732 -0.079938783 -0.037261597 0.006755681
-0.046722684 -0.023211738 -0.06187172 -0.008996064 -0.048887913
-0.065402001 -0.013830183 -0.045605384 -0.000838414 -
0.013951423 -0.065046695 -0.040017813 -0.024137431 -0.033101869
0.007861465 -0.083324472 -0.023050394 -0.055432858 -
0.067234673 -0.032797721 -0.029391404 -0.018362286 -0.046868182
-0.00874513 0.004251407 -0.032600694 -0.003003317 -0.020849227
-0.030921028 -0.017485819 -0.043995117 -0.048914087 -
0.01387405 -0.046626611 -0.067237062 -0.041148246 0.007622113 -
0.033974216 -0.023711198 -0.023301729 -0.026736792 -0.02887521 -
0.015475125 -0.004252029 -0.047525809 -0.002493223 -0.053080447
-0.028878728 -0.067986681 -0.016008481 -0.03302258 -0.033163113
-0.061252985 -0.040475248 -0.046671203 -0.08432782 -0.030084605
-0.048469 -0.028415054 -0.034517192 -0.061896572 -0.037810878
-0.066385956 -0.028029031 -0.015861682 -0.037258944 -
0.038975331 -0.01874234 -0.050741092 -0.047897425 -0.029512846 -
0.056327321 -0.052732113 -0.010618451 -0.097413953 -0.02179733 -
0.03632768 -0.068408223 -0.022406203 -0.015455912 -0.02682307 -
0.047580571 -0.00662846 -0.025243628 -0.045154125 -0.035155961 -
0.028397258 -0.008193438 -0.007963755 -0.037036665 0.013369904
-0.016152102 -0.041080687 -0.013159225 -0.036247328 -

0.02367648 -0.052453546 -0.027266142 -0.028646203 -0.045724511 -
0.016127392 -0.014921997 -0.038946763 -0.034900618 -0.027351361
-0.014525739 0.00583377 -0.043334853 -0.03369598 -0.062366831 -
0.049625417 -0.030402597 -0.018691308 -0.015385609 -0.048841003
-0.005958859 -0.039956473 -0.041427509 -0.064239314 -
0.000694811-0.015695816 -0.06566577 -0.04914709 -0.062503547 -
0.048790482 -0.004602929 -0.013756749 -0.069151433 -0.047807847
-0.042378105 -0.027868047 -0.021731828 -0.019152405 -
0.009107063 -0.022636927 -0.035111784 -0.026006382 0.003940964
-0.03045085 -0.040999334 -0.03328645 -0.055186699 -0.049873526 -
0.030479576 -0.029369352 -0.080159886 -0.029724726 -0.025542384
-0.056687771 -0.059295574 -0.042131683 -0.044710878 -
0.042224028 -0.029746162 -0.045885862 -0.045224403 -0.02430156 -
0.041742852 -0.046916409 -0.028575212 -0.027262171 -0.08740524 -
0.053323849 -0.094034465 -0.011019894 -0.030175225 -0.022567742
-0.045024824 -0.065143336 -0.067281458 -0.035902566 -
0.049280235 -0.083801926 -0.008271602 -0.065708304 -0.006090414
-0.019351907 -0.039878956 -0.030066449 -0.016626151 -
0.035043624 -0.041869005 0.002714708 -0.030769275 -0.044566645
-0.026849345 -0.003188411 -0.058188727 -0.039043353 -
0.021725648 -0.080872125 -0.035518615 -0.072591145 -0.042566733
-0.057281533 -0.043941486 -0.05777364 -0.055096398 -0.065761316
-0.039107576 -0.073904722

KEGG_ARGININE_AND_PROLINE_METABOLISM 0.1350611470.171907229
0.171520928 0.199073724 0.19745482 0.162569185 0.157490435
0.147756092 0.202710773 0.152473254 0.181932065
0.152230816 0.156357431 0.174341631 0.151366812
0.190955795 0.175210098 0.174192849 0.16958192 0.158394345
0.192583063 0.159186937 0.172438688 0.170529194
0.146021985 0.146531709 0.169764088 0.176641465
0.165202732 0.141282943 0.176890667 0.156707135
0.159217491 0.174152385 0.198912213 0.184731802
0.170047782 0.168810195 0.169506908 0.1567911520.181165491

0.193343229 0.174794279 0.199302641 0.156769907

0.167176881 0.178990315 0.170719593 0.174341355

0.192582567 0.180015796 0.204580342 0.163867691 0.18939504

0.178382241 0.167285441 0.168599847 0.158109548

0.200160225 0.135967143 0.174380817 0.1741406 0.179629887

0.191449495 0.180448663 0.184594468 0.191944028

0.203479245 0.178307403 0.1711358490.154447709 0.150725785

0.156703964 0.151823375 0.15411518 0.155003171 0.159674134

0.149977698 0.1728734110.164990467 0.187920665 0.193281408

0.176354743 0.15920036 0.1359307 0.155054143 0.143060112

0.151591514 0.164475825 0.148282135 0.199830688

0.177065865 0.184927321 0.159886683 0.171269193

0.175497579 0.152534136 0.159362542 0.182941814 0.17971917

0.179487577 0.182821522 0.2103377 0.168553034 0.189188257

0.182982036 0.195440537 0.177851383 0.172929848

0.152089956 0.201901753 0.1491144330.145724579 0.163347763

0.18555394 0.185056247 0.154769498 0.210861921 0.143216768

0.153963253 0.142262777 0.162645334 0.170145488

0.153945464 0.154524707 0.173975242 0.175189697

0.160906352 0.190277427 0.166695776 0.1761172340.155004361

0.188291254 0.156083099 0.160497268 0.17391639 0.162594539

0.165144091 0.134368566 0.174230172 0.163362533

0.149945756 0.178124559 0.142955827 0.176752215

0.1446881110.144193777 0.168227796 0.142181631 0.152066137

0.157538281 0.181242986 0.158886028 0.188802714

0.158786636 0.157188515 0.149436624 0.183383814

0.158962044 0.162198966 0.177659368 0.161446135

0.185912935 0.175870193 0.169829184 0.167255179

0.191692142 0.194936836 0.168518194 0.191417797

0.159368059 0.165165637 0.177009186 0.1720786110.177300285

0.1681142570.171689544 0.168684603 0.1711085060.178325413

0.163733469 0.208125384 0.180419718 0.158410263

0.145150207 0.1890251150.176355049 0.153823545 0.163212017

0.174953123 0.149797683 0.132534326 0.15337674 0.159759765

0.155507463 0.143846745 0.151552633 0.159270446

0.152972173 0.164042526 0.182585601 0.1467353 0.161465372

0.163265225 0.1419511950.179546071 0.147452229 0.175437034

0.183935015 0.13726472 0.151332282 0.159179417 0.167272672

0.191753674 0.174476754 0.155346281 0.167309489

0.178906335 0.170619724 0.175243433 0.15245848 0.173016331

0.199323923 0.145410231 0.148041813 0.146836056

0.177496367 0.165172351 0.189245649 0.152532039

0.178388916 0.164154429 0.152614673 0.15672789 0.15318191

0.148527183 0.195266796 0.173965098 0.169060608

0.157440121 0.175266527 0.175820477 0.173815172

0.147240158 0.17183868 0.150995166 0.180039157 0.196359242

0.179195187 0.1658117730.164235904 0.150246673 0.13029133

0.206799944 0.186614194 0.15654616 0.175052594 0.179786428

0.158777128 0.173549684 0.2006678110.147638699 0.186331365

0.153427179 0.187922475 0.175420087 0.187937138

0.152147256 0.168340932 0.144148265 0.186273315

0.174418497 0.1174080030.183092358 0.154718734 0.198738004

0.179032368 0.18405367 0.187159172 0.157329537 0.162372123

0.165860558 0.169759425 0.172059043 0.176386568

0.180743784 0.133315388 0.171476005 0.167291587

0.175713401 0.177362541 0.162782141 0.169450876

0.186882043 0.181477221 0.184748563 0.177835696

0.182635586 0.179715918 0.179351771 0.150527635

0.207923869 0.177774066 0.157923618 0.195683682

0.138010474 0.164382404 0.185789346 0.177094544 0.17431732

0.175308889 0.175842824 0.164946178 0.15898137 0.127858702

0.175923093 0.1659608 0.197478504 0.173949296 0.13118372

0.1619981120.208054052 0.138505443 0.161506597 0.188011268

0.154012334 0.175102829 0.197380888 0.168958361

0.155292337 0.194241294 0.17605502 0.161767191 0.170593282

0.161485103 0.163746059 0.19872805 0.177758266 0.127043171

0.269029042 0.189628981 0.165788198 0.163614467
0.1241101030.158981551 0.179243815 0.201098617 0.176843636
0.148822756 0.155259693 0.183598998 0.178320309
KEGG_ARRHYTHMOGENIC_RIGHT_VENTRICULAR_CARDIOMYOPATHY_ARVC 0.105026596 0.1361142280.104480561 0.092268613
0.091900694 0.064381866 0.073940506 0.086567466
0.088735022 0.1123616940.054306984 0.0940466110.122937724
0.097740841 0.1154345880.071409461 0.07524782 0.074304347
0.07607609 0.0880171130.079346106 0.085703449 0.076585602
0.100203142 0.091725815 0.100906442 0.094319737 0.10890747
0.099243792 0.090212593 0.076035541 0.081034373
0.1101322280.120860647 0.121996532 0.055145897 0.083449913
0.067847354 0.074513685 0.050786152 0.1130094040.087046228
0.074581683 0.081056868 0.094668104 0.071040916
0.091869772 0.11291863 0.04477693 0.0651168960.118864095
0.074495315 0.070026807 0.100287859 0.065447704
0.122633839 0.079297643 0.086984973 0.068160635
0.123048246 0.080166392 0.095748223 0.097488422
0.084057788 0.101098933 0.092678204 0.070310365
0.089596766 0.096704169 0.094894342 0.121247527
0.082986207 0.1180804720.094165402 0.090949475 0.123355297
0.100405321 0.097324021 0.138655226 0.108048956
0.078631458 0.076040612 0.088179769 0.108883087
0.072989521 0.079279343 0.078992612 0.101401012
0.108629721 0.128767781 0.085635323 0.074870286
0.099383962 0.083444074 0.104345073 0.093854726
0.103614286 0.099582701 0.072504684 0.053791736
0.109334393 0.07977558 0.071614464 0.088713404 0.075607243
0.102672783 0.046758135 0.089734503 0.083537252
0.1183215440.049004018 0.096641074 0.086950825 0.112690763
0.101728552 0.1103130110.072096354 0.094196574 0.082507811
0.108230065 0.073319232 0.105531743 0.089593997
0.1148652410.105366266 0.049972827 0.086841758 0.111361638

0.091714964 0.077610731 0.120104953 0.099069573 0.08217169
0.106224994 0.120309122 0.090707875 0.122593084
0.1119913930.1157683520.129505742 0.081523203 0.133516851
0.069995932 0.1139706150.060522317 0.133873602 0.125554911
0.075208889 0.100825203 0.11952779 0.090688934 0.08847761
0.083025369 0.092990403 0.106045235 0.129956124
0.1174557860.085912333 0.1161246460.097205735 0.087287932
0.101446125 0.098502497 0.097163666 0.1128356670.083267548
0.1110672030.095786817 0.089146661 0.088441917 0.09519437
0.075046071 0.1185601920.075331058 0.078683547 0.108341616
0.11536543 0.0921128010.09198251 0.107452237 0.105019635
0.066016938 0.074851783 0.11300968 0.096345181 0.093482201
0.100356039 0.133075986 0.109079519 0.100484521
0.1155334980.094319581 0.123532144 0.078347426 0.097336555
0.139813538 0.102846883 0.1153628910.138995346 0.091011008
0.133159043 0.1153747760.139063781 0.088541318 0.111281679
0.093460123 0.107016675 0.109385129 0.1105985540.10897029
0.1179767210.091439758 0.102305766 0.099156633 0.104948083
0.141316606 0.125040435 0.1169327670.146870768 0.10708372
0.127328482 0.096690574 0.103586502 0.1103894060.149343132
0.093167065 0.131307657 0.087420673 0.061817728
0.150882206 0.092335024 0.102438415 0.090991349
0.083162464 0.133553236 0.1128308250.088049536 0.11553464
0.132529542 0.090391792 0.074834361 0.122503747 0.0798105
0.105036531 0.127345828 0.0987713 0.094073347 0.097295141
0.0629111020.1105084860.1174404880.102741308 0.11057723
0.035226218 0.097388099 0.103907969 0.0992114680.12111868
0.08867016 0.1190135130.092318233 0.1011349690.072003744
0.098984765 0.0811771910.084989952 0.06642914 0.076833514
0.1133995470.09740091 0.070622623 0.10042654 0.143961266
0.087966475 0.085267672 0.089644222 0.09285262 0.074244725
0.1169367170.095047646 0.089039098 0.1113456120.125494829
0.108626388 0.104998058 0.094735763 0.129810543

0.1146617140.095503051 0.1151361060.100578306 0.124664996
0.075408429 0.108862191 0.109462425 0.124543538
0.093856686 0.1181743180.075141783 0.09878039 0.10249512
0.098663804 0.132909578 0.0950022110.108335282 0.125672464
0.1171251340.126139406 0.106961793 0.10793701 0.124040156
0.1186531620.078658647 0.090168596 0.138719431 0.127023102
0.086508059 0.086574935 0.104629937 0.107524192
0.097399572 0.047003126 0.122980602 0.1055511210.067333417
0.081924988 0.083471318 0.07660475 0.086833275 0.112892461
0.092157793 0.1121772220.070325823 0.097356525 0.137375844
0.079726203 0.10471591 0.12104799 0.1128119970.044047956
0.126071712 0.107104046 0.12437753 0.102700188 0.085453685
0.078189618 0.097739893 0.095995352 0.129381575
0.1131324730.091722961 0.094106278
KEGG_ASCORBATE_AND_ALDARATE_METABOLISM -0.155887375 -0.144700101 -0.082522187 -0.13758997 -0.126991744 -0.12549918 -0.126278561 -0.16392502 -0.128136932 -0.178703223 -0.146822423 -0.152031802 -0.176641246 -0.105662386 -0.142911089 -0.120137726 -0.136056583 -0.131079452 -0.193493746 -0.127979963 -0.108271433 -0.18147768 -0.144927631 -0.167886535 -0.195765834 -0.170418633 -0.136056527 -0.122584871 -0.10087058 -0.154090132 -0.058119214-0.167275393 -0.180608691 -0.146812707 -0.137027891 -0.124502262 -0.180067681 -0.140804918 -0.145301171 -0.128825258 -0.148377577 -0.152353117 -0.138088683 -0.154477219 -0.171866345 -0.152922838 -0.165880427 -0.145974609 -0.178854901 -0.123066532 -0.156940431 -0.145486284 -0.157467797 -0.105663039 -0.149461102 -0.142977476 -0.152583736 -0.126920991 -0.138746306 -0.178439273 -0.130794918 -0.202037453 -0.121156014-0.155879987 -0.181939337 -0.085309436 -0.161252605 -0.142694293 -0.154592502 -0.178120937 -0.133920095 -0.171122227 -0.146134771 -0.112426816 -0.14094792 -0.11571451 -0.156309539 -0.161529686 -0.136303054 -0.12342647 -0.132947726 -0.147576273 -0.20037194 -0.142287296 -0.144677971 -0.110077571 -0.130086523 -

0.124785904 -0.134131138 -0.122395507 -0.130111921 -0.090913433 -0.127754117 -0.109380282 -0.11848487 -0.171554775 -0.087648992 -0.167095833 -0.143815785 -0.134628376 -0.168864575 -0.139280287 -0.059725465 -0.151913595 -0.136634225 -0.144989566 -0.163467129 -0.167896784 -0.137349007 -0.134373827 -0.150214159 -0.156805647 -0.164984129 -0.145281888 -0.159149826 -0.147915815 -0.136396778 -0.120251963 -0.18310396 -0.172305666 -0.179104437 -0.170511728 -0.15644082 -0.162295652 -0.1866408 -0.150981358 -0.148749782 -0.088245662 -0.148508836 -0.141090109 -0.180214804 -0.16308723 -0.142006151 -0.14364189 -0.156698211 -0.202555669 -0.131356031 -0.105486088 -0.153048754 -0.147256044 -0.153576959 -0.109845742 -0.161504421 -0.118138681 -0.120195575 -0.151938375 -0.167418291 -0.048275839 -0.14678556 -0.117531329 -0.16969342 -0.108422284 -0.154881537 -0.098722636 -0.146825828 -0.199242964 -0.175018574 -0.146703237 -0.154399509 -0.131851056 -0.147662177 -0.118806148 -0.110101066 -0.138460104 -0.157803276 -0.143445127 -0.117295976 -0.151507601 -0.117583039 -0.067394193 -0.123088338 -0.092885567 -0.123925716 -0.114052282 -0.158165435 -0.160578919 -0.131994555 -0.176412562 -0.069816289 -0.167702141 -0.152204405 -0.144017794 -0.171774651 -0.130505798 -0.135039374 -0.168703513 -0.16007655 -0.155560916 -0.113709804 -0.125375084 -0.145677022 -0.152175175 -0.13219935 -0.148783656 -0.149626497 -0.143226857 -0.168076792 -0.185953268 -0.148269419 -0.181800037 -0.13722106 -0.164251077 -0.155443 -0.163591264 -0.170964149 -0.088087307 -0.18378573 -0.129221899 -0.12101481 -0.125454498 -0.140549471 -0.143809699 -0.174072131 -0.112920985 -0.141439557 -0.132921525 -0.199950055 -0.165672801 -0.137826276 -0.116850046 -0.150279924 -0.145798283 -0.162964589 -0.119024966 -0.155985363 -0.153538105 -0.145993151 -0.111548344 -0.180122525 -0.154577309 -0.148342431 -0.143462893 -0.103048814 -0.122154214 -0.111232694 -0.144703533 -0.137088619 -0.183282755 -0.166345779 -0.140029258 -0.141729245 -0.137065627 -

0.161134802-0.132365529 -0.167295099 -0.144765355 -0.149017318 -
0.172752313 -0.053283023 -0.136107804 -0.154741152 -0.146259976
-0.156515729 -0.144259274 -0.138699686 -0.141948385 -
0.110507903-0.096906686 -0.146402946 -0.133463305 -0.148379739 -
0.137331697 -0.121378134 -0.09250764 -0.174707889 -0.140256542 -
0.124557592 -0.149609319 -0.111063516 -0.102709186 -0.095954615
-0.175356056 -0.142592479 -0.107384624 -0.116563373 -
0.129278131 -0.15233894 -0.166324945 -0.176707087 -0.134543762 -
0.150898312 -0.149806377 -0.139754947 -0.191276913 -0.153258546
-0.170852668 -0.14326 -0.131389684 -0.154716588 -0.136552823
-0.165683991 -0.097334792 -0.144954973 -0.111128432 -
0.131326825 -0.140501328 -0.16241111 -0.117402456 -0.142256445 -
0.169910545 -0.120213671 -0.151953911 -0.146221606 -0.120900892
-0.179039806 -0.139858794 -0.143128464 -0.161798933 -
0.169757404 -0.142474821 -0.145127592 -0.154453762 -0.201449724
-0.059305588 -0.105840939 -0.153796952 -0.134405102 -
0.147086081 -0.142781908 -0.159934342 -0.116391205 -0.116607424
-0.144784082 -0.150625974 -0.18369001 -0.161020202 -0.129350974
-0.12464645 -0.142810234 -0.147191801 -0.151381381 -0.144349745
-0.187502643 -0.13729221 -0.168181463 -0.15605751 -0.141028505 -
0.1490119 -0.164906175 -0.02967226 -0.137434093 -0.177516894 -
0.126847594 -0.208284283 -0.192752816 -0.166365095 -0.13027531 -
0.07178824 -0.202884348 -0.097548598 -0.141541402 -0.119266033
KEGG_ASTHMA 0.1191217270.235768108 0.336233171 0.185900689
0.334065637 0.142932869 0.086543599 0.232705108 0.34390677
0.255784014 0.033786786 0.384020981 -0.084788852
0.249199046 0.370850953 0.25571021 0.344229949 0.419428165
0.231403095 0.223019398 -0.056752025 0.323331413 0.37820857
0.1211038070.386233012 0.349258681 0.31728036 0.314332097
0.271061406 0.265366103 0.204190753 0.371617681
0.140546982 0.227516309 0.302259567 0.28545796 0.08866758
0.310282314 0.386086078 0.002688152 0.274456831
0.202976545 0.299288194 0.31868096 0.220831999 0.262659477

0.273610949 0.192799364 0.404515267 0.257861291
0.364972413 0.402861383 0.294369854 -0.022889571
0.386286555 0.354469372 0.401295633 0.380653468
0.377206646 0.036990309 0.336015355 0.349526435 0.21943299
0.385554887 0.405701465 0.224312884 0.285892707 0.3558656
0.330892537 0.341097696 0.243146417 0.051591332
0.247857506 0.208953535 0.240163323 0.394496516
0.161967659 0.36351621 0.229095564 0.231354716 0.396316875
0.090316106 0.134993453 0.233696773 0.391039619
0.212157259 0.420730552 0.347489444 0.206906629
0.325441499 0.289059733 0.377269085 0.279653248
0.365965223 0.30400696 0.265178968 0.364208861 0.230776609
0.371871442 0.385715245 0.257162865 0.204067516
0.163766805 0.394696543 0.157603041 0.383944165
0.162887651 0.363254382 0.403287375 0.277673042
0.2473075110.352381302 -0.048512038 0.070541856 0.193183742
0.340217463 0.361709248 0.330584109 0.379067319
0.234976679 0.313213453 0.108708593 0.375409317
0.256971356 0.251230613 0.317092541 0.329793632 0.27269696
0.167325565 0.315356857 0.288797608 0.321405034
0.246060253 0.379552134 0.258016675 0.159035426
0.368037049 0.332108091 0.184294964 0.314033676
0.226995502 0.339190152 0.363193614 0.197206209
0.312241468 0.373785214 0.371612724 0.296464493
0.262992081 0.292492414 0.4065611830.338085205 0.309575375
0.067891345 0.350373971 0.378155073 0.271317024
0.393461052 0.156219127 0.417379783 0.278139325
0.213172754 0.20598826 0.350908919 0.422522441 0.397514179
0.1298116770.376605755 0.285677302 0.223304934 0.362316002
0.330004899 0.204567743 0.271846867 0.429725109
0.387287755 0.31222575 0.335975151 0.353023681 0.199516895
0.38612936 0.032246641 0.28472174 0.390082825 0.344070781
0.089637902 0.357243756 0.328752293 0.1689481180.288697876

0.34164836 0.40253548 0.265047583 0.368256925 0.36615085
0.2261261170.407492039 0.293608633 0.3043085 0.309481642
0.360481072 0.245022489 0.272547524 0.324771231
0.307726948 0.022207864 0.292264627 0.188687616
0.304893689 0.37019807 0.27150209 0.265697463 0.208744921
0.10221628 0.3281181530.336074189 0.153362239 0.359405757
0.349640898 0.234632077 0.3217911590.308267304 0.256400553
0.149255972 0.342676397 0.108401799 0.296524241
0.389470138 0.276299355 0.2776621120.397640194 0.354148396
0.293812669 0.309869866 0.355236599 0.335905961
0.2651195970.340322441 0.286601017 0.349470097 0.334487681
0.379662864 0.335041567 0.318708945 0.364144218
0.362950557 0.317175556 0.058573493 0.333843177
0.305020419 0.348969479 0.454540188 0.370162584
0.347383563 0.142767423 0.313505639 0.304532293
0.381312478 0.253526573 0.26683617 0.042758257 0.302522841
0.331460348 0.304388346 0.389230815 0.318248485
0.408672799 0.415668289 0.281458554 0.197635291
0.428031765 0.369929362 0.144036999 0.365453225
0.255020259 0.147599655 0.366452509 0.400571214
0.2011764770.250852963 0.285908547 0.308307126 0.225315552
0.278601743 0.356788038 0.414094965 0.313712952
0.408428598 0.465904332 0.358912201 0.354878918
0.380745873 0.368590941 0.052027256 0.3417115490.345321772
0.104440452 0.399379682 0.371706551 0.226489662
0.171434172 0.333172265 0.386213585 0.285635872
0.221308015 0.382609644 0.219362403 0.24501905 0.30125438
0.306906892 0.379010122 0.287492349 0.178248152
0.191213259 0.0771186630.269280661 0.234587059 0.353336896
0.314866581 0.207663308 0.3771188820.376137935 0.281775108
0.162681731 0.43580755 0.232664467 0.335574964 0.337600066
0.4064411610.391532982 0.349161541 0.3422931110.419539726
0.442326855 0.357352974 0.369473302 0.350370478

0.361010279	0.06202736	0.22627356	0.29650369	-0.007057591
0.27179506	0.146532785	0.412738917	0.313063998	0.195420808
0.265054955	0.253203891	0.073264946	0.128638249
0.122845446
KEGG_AUTOIMMUNE_THYROID_DISEASE	0.153339434	0.245506656
0.312399909	0.201687873	0.288728547	0.16917383	0.130291593
0.207726241	0.348735678	0.228164851	0.098770948
0.346639424	0.029967747	0.237986861	0.331024002
0.247736677	0.31251528	0.400896913	0.250834212	0.229542225
0.026271036	0.324678434	0.345420522	0.160843077
0.327029087	0.328415357	0.302222634	0.296353092
0.265740505	0.266589525	0.192702728	0.355406143
0.168474763	0.236277342	0.273354887	0.279141783
0.165354781	0.313819614	0.342563539	0.093626528
0.271400495	0.216963537	0.271230131	0.308342463
0.250220322	0.26085137	0.265033052	0.222224351	0.355345958
0.280619466	0.367105959	0.375247981	0.2892241130.081056263
0.346779863	0.305225917	0.370359417	0.378151264
0.334301772	0.1180011470.341535905	0.343738134	0.218349219
0.350310427	0.415361864	0.253219583	0.314170103
0.336642249	0.28964929	0.3117257570.259725728	0.095597719
0.260483235	0.218450031	0.268539093	0.3585747110.179916502
0.320346141	0.230521601	0.222486749	0.375020575
0.123261977	0.18441918	0.231888105	0.395888741	0.21608705
0.40373376	0.34161603	0.213583416	0.309076277	0.279660787
0.356914265	0.26520019	0.387752977	0.277342092	0.285254611
0.351585092	0.226466945	0.364587492	0.380205973
0.260644024	0.220167062	0.197887722	0.360275355
0.156583894	0.372140357	0.218787297	0.315239128
0.363474649	0.284955875	0.250125892	0.299766458
0.030556951	0.141297431	0.194727582	0.3116914090.323432227
0.310618014	0.350074491	0.236523891	0.3118402320.146058013
0.346757003	0.258933784	0.259109606	0.308664949	0.30386659

0.263619836 0.193633363 0.315413371 0.285419746
0.337862871 0.247634919 0.349761043 0.246848561
0.180323204 0.340740087 0.313153433 0.20097927 0.308387246
0.223918672 0.315556128 0.329816832 0.201857378
0.291267848 0.340196062 0.33223396 0.295108421 0.262793388
0.279301429 0.38073182 0.301266443 0.299657218 0.139154624
0.313699787 0.391209477 0.277222388 0.35994823 0.165426493
0.384602598 0.274384136 0.208127752 0.226290881
0.3119099050.347763751 0.375206746 0.177750697 0.342410217
0.293793132 0.235224762 0.34572285 0.3079323110.210481945
0.264814363 0.373108133 0.359602508 0.295790738
0.297164012 0.358329651 0.223445127 0.352527562
0.091082568 0.269705616 0.334237145 0.322032448
0.138853703 0.320315923 0.319738234 0.186605418
0.260022868 0.283473308 0.374603497 0.266383307
0.348098203 0.324105321 0.241274247 0.3577761180.297872274
0.284164889 0.274609383 0.324656661 0.2456014 0.279024977
0.2982335110.31301695 0.096833455 0.28615927 0.196425463
0.283363897 0.353007267 0.252932186 0.249947089
0.214285708 0.15755751 0.302403659 0.309879622 0.185150586
0.329999053 0.313702761 0.221442033 0.319579848 0.28834971
0.2664341170.157366707 0.329844261 0.135012801 0.297849035
0.345382967 0.281960292 0.275056497 0.343310469
0.329353087 0.266015712 0.304686647 0.303740475
0.334881768 0.265072422 0.321707816 0.286849181
0.346190792 0.310057926 0.339204735 0.318480685
0.302443382 0.340292713 0.310858881 0.306099838
0.137206559 0.312385367 0.290126138 0.308568873
0.392690409 0.341441909 0.329127269 0.142310218
0.3178664110.27710711 0.36189374 0.253948487 0.272746408
0.102142403 0.288185508 0.316038193 0.29871717 0.360311045
0.31143978 0.367691643 0.379203161 0.274600932 0.20675225
0.421486581 0.343217264 0.172599456 0.302096954

0.269670475 0.16997444 0.345718797 0.370173037 0.213209402
0.255548099 0.280857934 0.297775209 0.238424601
0.265774881 0.336038334 0.3859142 0.316929571 0.373678126
0.417447018 0.315093188 0.317275614 0.353449492 0.34696338
0.123094825 0.309964175 0.304830387 0.14792276 0.349687921
0.370303352 0.21739761 0.192212343 0.324873762 0.334659315
0.292160172 0.250489819 0.352405916 0.239981484
0.252361279 0.300203023 0.300881889 0.353089592
0.294492565 0.175633173 0.183719789 0.090031591
0.276698615 0.245805434 0.339787976 0.283206976
0.236221951 0.3133114890.360257354 0.291315484 0.200696854
0.387542599 0.233983656 0.326524126 0.298276333 0.35733377
0.342688103 0.331428078 0.297729373 0.358815317
0.391971295 0.306488557 0.337844749 0.316752479
0.331779656 0.096531032 0.237402508 0.261963049
0.062271071 0.258747614 0.175444456 0.374587405
0.295840966 0.209807683 0.261655003 0.26515487 0.112959967
0.182283848 0.171354609
KEGG_AXON_GUIDANCE 0.166700161 0.188242289 0.164324575
0.138929383 0.162948616 0.170222691 0.1585896 0.141936416
0.134550606 0.150792656 0.146749729 0.146741066
0.179968181 0.1500511340.154661221 0.146471833 0.147858354
0.132086841 0.155416442 0.170131574 0.178408483
0.146820744 0.14032973 0.192568765 0.158127737 0.136348356
0.137041695 0.135067461 0.148597979 0.170647583
0.135199266 0.134561434 0.181295193 0.14770691 0.177891265
0.1111989860.162984512 0.1164318590.130462885 0.111595479
0.177755595 0.140496517 0.126350059 0.149459498
0.160044864 0.167644375 0.19031763 0.1601105860.132022036
0.125223644 0.147485742 0.12425029 0.1172954190.151760431
0.137493451 0.162291856 0.122231807 0.127429705
0.1153106490.18203772 0.145276815 0.154080447 0.145856183
0.138167643 0.13702265 0.1153981730.1139070230.141247391

0.128423395 0.150232616 0.194357326 0.1498411180.171037056
0.150405053 0.155517808 0.179029394 0.146534624
0.141658744 0.17409369 0.176853941 0.122194808 0.129663241
0.134915598 0.159656718 0.093966369 0.134365087
0.1153528730.170787487 0.154238746 0.168414791 0.134978039
0.108191739 0.137915056 0.1144248660.172234373 0.155360974
0.1239928110.143364387 0.146898772 0.129391436 0.163549047
0.1406347110.102543382 0.16200225 0.1631191590.152823427
0.12241716 0.156530051 0.1152300840.164725432 0.123395598
0.157321448 0.16010766 0.151815619 0.161735047 0.141467048
0.124248125 0.126180646 0.155991951 0.171233139
0.152566383 0.171513442 0.153156477 0.168917938
0.133003386 0.1159900120.132427946 0.139320333 0.130721599
0.1471151550.169645039 0.141215955 0.133079084 0.152222965
0.163817601 0.157665141 0.156242203 0.153510177
0.191338363 0.189209081 0.156772687 0.190385991
0.122605037 0.175344142 0.121569696 0.184269915
0.155978144 0.108439375 0.129285124 0.141983326
0.149994408 0.11171829 0.164736445 0.144427309 0.176239812
0.155368014 0.182973545 0.145689475 0.154149025
0.186995456 0.170413713 0.147820167 0.11423447 0.169656942
0.145152513 0.1116055470.1372811820.143167414 0.138493147
0.147623142 0.160173296 0.1131371910.147967062 0.107986584
0.127267093 0.156774028 0.15771219 0.143577966 0.111459676
0.150817976 0.149202526 0.134019583 0.134171051
0.151390369 0.134456447 0.151282248 0.159600338
0.174586264 0.147783962 0.16605816 0.176538106 0.1575381
0.167603324 0.150312483 0.170957161 0.181431287
0.146452998 0.150481344 0.168490648 0.153342303
0.180676447 0.13966603 0.162930403 0.132722141 0.125227896
0.137345471 0.196054054 0.173892784 0.163756232
0.1195222450.172630992 0.158074335 0.166092673 0.169889542
0.150001724 0.182328384 0.182569643 0.171273971

0.1958611590.16946827 0.18383558 0.148633199 0.141858984
0.161481885 0.18417798 0.173045198 0.172325508 0.145274696
0.141528258 0.2110771580.14857694 0.129137265 0.141841003
0.129215347 0.188288605 0.141205263 0.140392563
0.154420249 0.169839616 0.169951821 0.130794414
0.1611958030.1291158380.130799034 0.171026703 0.160288865
0.105947384 0.136636221 0.10968342 0.15512509 0.158012735
0.146212045 0.146374481 0.098386248 0.167004551
0.158297862 0.125299589 0.128251962 0.132139316
0.156034662 0.131286756 0.146100045 0.121627477
0.133657377 0.137230049 0.1648271140.1198974530.134740539
0.163724568 0.147228721 0.130785269 0.163510646
0.196890074 0.109720572 0.1390911770.164589315 0.17310608
0.133521801 0.169646848 0.153762049 0.142883472
0.170239825 0.151613494 0.147609477 0.152026002
0.153510489 0.1827114610.165743601 0.143745785 0.152193816
0.148735909 0.174541517 0.1138202940.139935836 0.14033961
0.174224964 0.129742966 0.154931336 0.143190269 0.16064032
0.152326706 0.132895731 0.175144286 0.166204722
0.156563723 0.165319629 0.165219575 0.158627728
0.144944695 0.155617241 0.164239617 0.1565339110.134938959
0.138095498 0.186633357 0.158536209 0.14512097 0.120567623
0.157522701 0.174094894 0.150433149 0.1157377210.168500726
0.165642602 0.100196841 0.126776191 0.136870725
0.104793451 0.145522278 0.150216662 0.17869017 0.168552102
0.137547668 0.14141596 0.178313912 0.1721180560.148147202
0.153527586 0.155852609 0.098919392 0.150621539
0.200006556 0.180390293 0.178185776 0.168984437
0.144417463 0.159754549 0.1511728190.173604975 0.157812766
0.149249685 0.119031219

KEGG_B_CELL_RECEPTOR_SIGNALING_PATHWAY 0.130153697
0.156029857 0.18036923 0.124391303 0.153394885 0.122099359
0.1176035510.156961014 0.142807029 0.142994505 0.111286593

0.181650824 0.1152743660.144190554 0.1611203060.153885848

0.120520396 0.1544115870.159289679 0.120922394 0.122231157

0.209508278 0.149442166 0.1181589590.184649505 0.160022242

0.141527907 0.167124664 0.154297069 0.16741905 0.094008464

0.166715441 0.138994226 0.127476648 0.147939352

0.130598391 0.1229031150.142505373 0.144946807 0.099969107

0.171776927 0.147319904 0.139581488 0.149239663

0.153060499 0.14241396 0.140995656 0.1171374440.180205056

0.151933372 0.201289636 0.172670327 0.165953371

0.1187886760.161334264 0.158506926 0.171497742 0.178004739

0.122347938 0.1164639410.133201457 0.168056479 0.142544249

0.173012396 0.147547356 0.1142017130.127193635 0.164840209

0.144256486 0.141435343 0.181847824 0.1173962970.158786725

0.121676832 0.156871526 0.216498203 0.137324455

0.145617355 0.159536983 0.144660192 0.157399013

0.1145949510.134820277 0.150136016 0.183955346 0.137276168

0.18547887 0.186596927 0.158513714 0.147850043 0.145983676

0.1604117350.147377176 0.127207069 0.162500942 0.154139452

0.16437175 0.139159823 0.204166722 0.2282411220.171644605

0.156052218 0.126736729 0.160971756 0.1144996110.162442177

0.131861659 0.159751756 0.138561345 0.135732462

0.157990833 0.163025435 0.094757371 0.133097838

0.138557717 0.18395927 0.155278907 0.149315445 0.15600386

0.154460293 0.185909303 0.136775529 0.167166177

0.189154352 0.173230044 0.130841653 0.146690667

0.131778245 0.1165584580.188363326 0.153288892 0.137170575

0.140044166 0.174916605 0.174265212 0.145967482

0.174277769 0.166508398 0.15034274 0.171967219 0.146223275

0.178238126 0.175714338 0.130240252 0.158458726 0.19209979

0.164637527 0.146166747 0.136072629 0.149929147

0.184502729 0.153100008 0.170561579 0.11772877 0.170958356

0.1852118450.167867672 0.170577484 0.16847856 0.225365898

0.156275102 0.149429263 0.144504023 0.16863441 0.14360156

0.156105478 0.125659122 0.164024849 0.137031241
0.138200606 0.184918497 0.167788508 0.109163547
0.151917569 0.160448275 0.198021791 0.171821956 0.14824745
0.165071612 0.162557142 0.187172596 0.1141936350.156400446
0.141488459 0.178246221 0.130556927 0.137124735
0.191884884 0.131322633 0.154789242 0.154364082
0.191768059 0.190479422 0.2117393680.19574861 0.163373375
0.18374471 0.18965569 0.198672891 0.195740444 0.197170501
0.126497077 0.167063139 0.142410898 0.179275854
0.1199345430.163493003 0.143041383 0.157619206 0.15415387
0.177400301 0.156234561 0.185717074 0.137219205
0.171830045 0.172560489 0.170620587 0.19987117 0.19765817
0.165174636 0.17524652 0.187664337 0.149477745 0.135452514
0.1666751170.1134192230.180109003 0.153772725 0.105609685
0.190868622 0.169043099 0.206845047 0.145291002
0.157753964 0.166067899 0.164894158 0.146435265
0.190558182 0.171826227 0.17019595 0.169354524 0.168391586
0.125258968 0.134358092 0.172847063 0.191933483
0.123808258 0.129166431 0.154641629 0.170891021
0.152093932 0.2011040840.174620976 0.150860655 0.121517526
0.142288191 0.141523431 0.170713754 0.147609131
0.143995985 0.1130565140.1447801190.135688455 0.162394293
0.198014855 0.169801941 0.182451918 0.183872523
0.1192678360.138827038 0.210987859 0.183527656 0.137165029
0.129169339 0.163129317 0.1105532220.161651543 0.180812169
0.1133890290.15369616 0.147206343 0.158275021 0.142214434
0.151049186 0.166251646 0.171585741 0.182338106
0.209469288 0.221430962 0.183878045 0.162707952 0.19132679
0.137274219 0.1169544870.169809513 0.176341794 0.137163558
0.169018784 0.190688142 0.146405037 0.143212836
0.149070812 0.176996206 0.127659518 0.166866297
0.1911019280.185904563 0.146807322 0.180469475 0.167494673
0.192274787 0.151532102 0.1296701110.1471131870.132556116

0.168397101	0.160901426	0.132648648	0.15017256	0.14437646
0.159394292	0.171068824	0.16117524	0.1185924490.166046569
0.1191827490.181261663	0.154242925	0.157316351	0.177944619
0.199147838	0.163214428	0.160721861	0.170635487
0.192146676	0.162623661	0.151604061	0.191529163
0.132513985	0.154287781	0.166142096	0.12822463	0.139526425
0.1400965	0.19239487	0.1485119960.167849631	0.173073028
0.183584437	0.127652885	0.134839635	0.11828519
KEGG_BASAL_CELL_CARCINOMA	0.064302243	0.096862682
0.053261428	0.071076129	0.014997594	0.062810574	0.10038963
0.085337878	0.046390691	0.0539076	0.043812885	0.026749246
0.1117858150.103604298	0.054801471	-0.006918573	0.103471068
0.02011474	0.0711370730.1182625440.138553336	0.038603886
0.026780199	0.096356901	0.078560598	0.031731443
0.073746104	0.030702636	0.067548337	0.052861231
0.089153083	0.0318115890.0969119370.142993302	0.100971284
0.003653583	0.122237942	0.00658497	0.038419008	0.058595186
0.081377696	-0.011485025	0.0251034110.006399596	0.070160044
0.052589272	0.1114735060.079506338	-0.000235019	0.024349449
0.026131158-0.02382112	-0.00324749	0.08769713	-0.006330582
0.101060433	-0.008749272	0.036596674	0.025429464
0.138914609	-0.027153328	0.057272124	0.097247799
0.032406875	0.044657206	0.054590841	0.010000034
0.016491224	0.055170262	0.060035788	0.034725452
0.103903827	0.074309504	0.100510489	0.03148351	0.058834906
0.098016101	0.072478907	0.095671844	0.081478551	-
0.0031931170.014097356	0.1007803	0.061583195	-0.042218262
0.02566261	0.067598212	0.121261275	0.066167921	0.123160318
0.051934739	0.0114099960.052251298	0.0111542350.01996584
0.05531008	-0.004053183	0.078800691	-0.02417815	-0.013020016
0.000500465	0.038160258	-0.001061053	0.061208267	0.0957429
0.061297741	0.066556895	0.074902367	0.027458474	0.08698208
-0.041133961	0.026077642	0.073335008	0.058177055

0.063201007	0.062154879	0.001237921	0.05546985 0.034913906
0.052257489	0.064851895	0.087748782	0.0831731170.094535494
-0.008482221	0.001445913	0.020990968	0.083338406
0.033694905	-0.016420241	0.08868431 0.071689624	0.047456435
0.017254085	0.064805021	0.092752819	0.034707373
0.058001952	0.1165504540.047760041	0.073474236	0.111435786-
0.012819125	0.089703586	-0.029415867	0.07439533 0.043525248
0.043370086	0.069957333	0.04099011 0.004430082	-0.017231789
0.027242709	0.077643299	0.070663264	0.039908594	0.10489446
0.02762966 -0.013429457	0.064294515	0.001812654	0.065417071
0.020763572	0.08904517 0.082946809	0.026445022	0.137233291
0.028873457	0.066155766	0.078208059	-0.015945546
0.039278775	0.056286302	-0.071304009	0.00561958 0.039137173
0.046957492	0.07290673 -0.011802415	0.073245504	0.017180154
0.006344134	0.026689229	0.084415197	-0.049337519	0.06260779
0.077831342	0.075802244	0.088467399	0.065685752
0.099639846	0.028876845	-0.008716192	-0.002181005
0.047858106	0.083120474	0.0411865850.0211765490.096597501	-
0.03167264 0.0794551140.024200848	0.0113858350.041570297
0.036442898	0.076234508	0.101427613	0.1148507730.04175172
0.041326577	0.070461903	0.044690083	0.077385902
0.108719438	0.029465995	0.107481751	0.058137863
0.077619705	0.1145204190.053668187	0.10322279 0.050007043
0.079309241	0.087510754	0.120789346	0.120689761	0.07795732
0.049805499	0.065364456	0.103496132	0.044878499
0.061772344	0.084848931	0.0042541170.123668887	0.057460308
0.059385003	0.052808017	0.059479541	0.066509601	-
0.034978334	0.080600973	0.037531094	0.01161523 0.072466322
0.028739381	0.007406256	0.045590171	-0.005259086
0.059399855	0.05345357 -0.00290216 0.009389576	-0.027037822
0.054665467	0.094452563	0.050564604	0.040296588
0.051444144	0.03165117 0.074065476	0.091058378	0.002275028
0.041342212	-0.011162322	0.069094339	-0.053406928	-

0.003451396 0.099779941 0.094102392 -0.000657249 0.006070828
0.105523483 0.037722498 0.038602276 0.096149264
0.067339341 -0.01962076 0.077716824 0.051882271 0.014332843
0.067067372 0.024186712 0.089769044 0.057119119 0.028661685
0.032151433 -0.000452329 0.040252909 0.074505887
0.077868375 0.098397604 -0.008284875 0.067534557
0.012083472 0.07962062 0.109132286 0.065555434 0.008629083
0.027485808 0.007806917 0.04362554 0.075179909 0.052245783
0.046899874 0.038049882 -0.030589818 0.058511594 0.032170728
0.124240218 0.094256113 0.085076526 0.103630204 0.022294767
0.103612972 0.08441271 -0.039657156 0.06197243 0.06461951
0.090717652 0.043500143 -0.027804689 0.081654779
0.097716144 0.006866722 0.050827538 -0.006319195 -
0.008785323 0.014237594 0.067235367 0.066487939 0.058570315
0.067009587 0.064403872 0.084660204 0.074034889 0.04510344
0.069081791 0.117655095 -0.039121489 0.11854837 0.148485747
0.046473586 0.114035487 0.025994219 0.02147043 0.063313006
0.056691835 0.081880537 0.102270758 0.028532764 0.03660788
KEGG_BASAL_TRANSCRIPTION_FACTORS 0.134803234 0.08629753
0.140931438 0.120993858 0.127174078 0.148772769
0.163182465 0.146612121 0.129979964 0.141003137
0.174642176 0.136392376 0.140820088 0.143910243
0.114432047 0.145962745 0.149673335 0.136576702 0.115852367
0.157356583 0.166187687 0.127850874 0.133110556 0.110032844
0.156935464 0.140129418 0.163780569 0.137429908
0.170679787 0.114010795 0.199356675 0.16112241 0.15816296
0.129434504 0.124831389 0.129199523 0.156977822
0.123377101 0.131211733 0.165726483 0.130050465 0.134448769
0.150868505 0.112786421 0.125283096 0.133799602 0.131284681
0.118517323 0.128363006 0.146876941 0.108297109 0.167888166
0.133672405 0.147582348 0.130021985 0.126467041 0.17855064
0.150462745 0.144213882 0.132919337 0.16590446 0.109622129
0.162396688 0.115469695 0.133676184 0.158039186 0.123151

0.1449115290.141840557 0.142272942 0.138722729 0.196292536
0.134190631 0.150123578 0.167014167 0.130485196
0.122585722 0.152878158 0.1197143090.143937584 0.121963816
0.165029713 0.170019578 0.143306538 0.163881303
0.165958999 0.166618949 0.121615062 0.143464738
0.143177059 0.157373457 0.156597368 0.162042124
0.146009499 0.140094866 0.148810713 0.198319613
0.152463213 0.103961752 0.123687296 0.130786924
0.163760091 0.134379421 0.1159380420.123527966 0.099037194
0.142165717 0.1254588 0.144695444 0.152283466 0.163035481
0.159029937 0.156777618 0.150983223 0.130986841
0.122041052 0.138189783 0.134769937 0.134728134
0.147586531 0.145379043 0.130132009 0.125272499
0.139734978 0.161874516 0.132923815 0.147581594
0.156197416 0.1365115930.12763533 0.138542873 0.140828529
0.1183418780.1331661190.163509707 0.173233209 0.140516409
0.139474135 0.138480782 0.1158830910.156692426 0.129221498
0.1131959660.143828958 0.176215761 0.123439949 0.121456488
0.181877505 0.145822231 0.161429759 0.1192372070.160961425
0.160725104 0.124191669 0.1132301350.109922485 0.126957461
0.1197961450.16786229 0.1106432920.149507968 0.13694355 0.15911472
0.141688958 0.143074882 0.132907038 0.125723067
0.160203276 0.131483017 0.127318041 0.140839603
0.1867114940.146988208 0.157831501 0.146970763 0.124618123
0.142152337 0.128263322 0.145935938 0.148973436
0.129918758 0.165036582 0.174280308 0.1429229110.172876572
0.1187548520.156521279 0.1157128880.12415109 0.143492625
0.127706272 0.140039458 0.142957052 0.122405639
0.1160992240.1209113360.148747255 0.125216323 0.131218338
0.155016375 0.1113747370.1125906370.1104848210.14987847
0.150458412 0.15581677 0.143817199 0.136044935 0.11369096
0.160138718 0.157902809 0.151806105 0.131089829
0.155356633 0.125662728 0.1154392360.135269232 0.139724122

0.09676684 0.15095411 0.125987829 0.1189863560.12104532
0.156292063 0.1411204330.15564638 0.129303057 0.163007295
0.1997611850.1109797640.135576418 0.1198876040.188606241
0.151488595 0.130417794 0.150781218 0.168979565
0.133838506 0.129655057 0.120205422 0.150444244
0.1175796940.149604925 0.164755982 0.1119375640.146920115
0.143401866 0.149754282 0.168909638 0.154850993
0.155486226 0.148856625 0.154903269 0.186196806
0.146506021 0.122239741 0.151202279 0.134173888
0.155389998 0.140551672 0.16137943 0.1411849190.144278058
0.147776214 0.134919304 0.140747503 0.14593872 0.168412208
0.1368785110.148529098 0.1198848310.13595519 0.126709931
0.185727895 0.158867601 0.1611347950.102390501 0.151028905
0.125229109 0.150045764 0.154051996 0.139776338
0.157754579 0.1511842890.1143579190.161977661 0.138002401
0.14744825 0.1183194920.136637091 0.142344079 0.13490809
0.148431677 0.145675828 0.13765615 0.152498723 0.151440942
0.129781846 0.136147616 0.182462245 0.1511822690.128749252
0.1106349120.12291081 0.13015624 0.130984644 0.157603831
0.148746187 0.141250801 0.1132342790.129441549 0.151360276
0.174429914 0.167624 0.149578913 0.134329841 0.113773858
0.142966609 0.138579789 0.150842247 0.1539285 0.163831321
0.151053419 0.136183212 0.154702619 0.145910851
0.145550836 0.176038044 0.143641819 0.155486433
0.135846139 0.137833698 0.165900886 0.125705469
0.145431991 0.143660817 0.158642222 0.127004478
0.1685031180.166459469 0.1156037480.124975216 0.11630109
0.148363889 0.13493161 0.135263609 0.121618577 0.150423406
0.127364664 0.157889276 0.140475845 0.167724744
KEGG_BASE_EXCISION_REPAIR 0.289108449 0.272261745
0.240659697 0.301243228 0.279829631 0.304099794 0.29028821
0.277746853 0.269792095 0.272046339 0.2884796110.31761958
0.265308424 0.297891731 0.285533047 0.299605562

0.272826431 0.261775861 0.296733243 0.272865568
0.324135793 0.271836607 0.2711577020.307698106 0.302430554
0.278600002 0.319877709 0.274299873 0.259007599
0.263735241 0.300082766 0.276595231 0.304499844
0.284078923 0.268080174 0.310102156 0.288306206
0.309433044 0.291724147 0.310034736 0.27915949 0.256778043
0.285490012 0.301762302 0.252063755 0.308356155
0.273043505 0.263618868 0.287698671 0.288354271
0.234109938 0.267658625 0.315620395 0.2970824 0.319540806
0.265379618 0.280987386 0.262988539 0.29227092 0.26717645
0.307221775 0.285503923 0.271786447 0.28536607 0.313091336
0.278552991 0.281633473 0.286862557 0.263822399
0.307036641 0.268258828 0.31372055 0.256777367 0.286665451
0.263855955 0.246406543 0.281086797 0.273901945
0.268551023 0.273325673 0.278201397 0.298548024
0.300460075 0.282979816 0.24918398 0.312337059 0.273649066
0.258656796 0.258686193 0.276352997 0.31505051 0.262895854
0.268431465 0.278096042 0.250039779 0.26446146 0.282673886
0.270915017 0.259796955 0.290227996 0.293028942
0.285490692 0.26836463 0.237376723 0.302508781 0.28075927
0.29784716 0.295929978 0.288071653 0.280698985 0.298975521
0.316562962 0.329742445 0.269933619 0.299868913
0.260342963 0.297508395 0.244742074 0.277109619
0.277643325 0.2811650330.2975711180.24823935 0.271677198
0.30798575 0.3018811440.260323696 0.267382835 0.278563434
0.254060049 0.286912824 0.273224571 0.265407513
0.280425834 0.265465498 0.266781028 0.229588243
0.243728297 0.260831295 0.244386653 0.275254122
0.256825875 0.321056401 0.299051626 0.293746669 0.23300768
0.273340707 0.282718669 0.257797918 0.297605567
0.283759907 0.270991498 0.300876543 0.273002079
0.260507784 0.284341417 0.250807702 0.297029188
0.301678772 0.266682388 0.30441211 0.272041688 0.273142989

0.274374133 0.276668821 0.279916477 0.28055948 0.310361786
0.254705246 0.287138225 0.286925807 0.285948599
0.264641051 0.294696554 0.3111053540.265467415 0.285221879
0.272007723 0.29443366 0.249851485 0.242054045 0.279918771
0.279980751 0.27439041 0.276757962 0.2924421170.300104432
0.258817712 0.287413007 0.26631072 0.273108019 0.283260407
0.265850144 0.264553349 0.273432522 0.252653555 0.28314842
0.260525757 0.245307536 0.2711274410.241834439 0.258575217
0.258082255 0.290796594 0.246479577 0.281069697
0.295375508 0.28488225 0.250967459 0.260407428 0.265695214
0.276512632 0.269404226 0.267926931 0.267999426
0.242477948 0.281790259 0.246594672 0.204528442
0.284109581 0.261238552 0.273399789 0.260308599
0.279472236 0.22583156 0.287422272 0.252807698 0.264916792
0.324070946 0.197510134 0.296057082 0.270153857 0.29945984
0.303349962 0.268680348 0.270542872 0.259619421
0.264226418 0.24411764 0.271427765 0.301479145 0.259062828
0.314464401 0.254574853 0.244624296 0.254754559
0.280884761 0.299183373 0.310656213 0.286745766
0.277470265 0.227272362 0.255628994 0.279267453
0.313121715 0.231992881 0.267683028 0.260641861
0.328562958 0.282139349 0.307367929 0.276796762 0.29155291
0.276049192 0.322795085 0.253667749 0.306413077
0.287247457 0.26233641 0.26514165 0.2781182660.292657644
0.2499151180.294762126 0.280008474 0.283908357 0.252853258
0.303140506 0.259959178 0.23936089 0.29499047 0.251761729
0.255242989 0.279794467 0.270235185 0.288682597
0.255529695 0.262590456 0.273657151 0.2522011540.261483116
0.225208882 0.276471454 0.277676106 0.278183583
0.2840993110.28510433 0.256308228 0.278496442 0.31399966
0.284983195 0.287916777 0.257897189 0.287621668
0.262040274 0.2556114360.269551704 0.28585383 0.282738832
0.254845347 0.246071038 0.2495622110.287874581 0.281608535

0.277030816	0.2223191	0.263236282	0.317851038	0.290908982
0.261607402	0.285829632	0.270781641	0.2729110920.286996601
0.307989439	0.2822112760.26380547	0.277888919	0.27398794
0.27114022	0.296965538	0.285153189	0.299068082	0.286505781
0.247520288	0.2702091130.268014001	0.285528789	0.282800379
0.307239956	0.244551836	0.267319158	0.257756473	0.27943962
0.253963229	0.293829057	0.276213286	0.261666669
0.236148861	0.27339476	0.277137318	0.295245457
KEGG_BETA_ALANINE_METABOLISM	0.11739102	0.13433157
0.1182417020.127820952	0.155178667	0.142292937	0.161104104
0.1112618220.158382349	0.092933887	0.142400128	0.130209144
0.14289263	0.178079459	0.1197402520.155862898	0.115796727
0.156835583	0.092413637	0.158314459	0.133651942
0.1174765080.130674919	0.150304756	0.133993806	0.15679189
0.130301399	0.155818126	0.1321119480.11390586	0.118114191
0.13698334	0.140716569	0.125676952	0.130325372	0.151925038
0.128648768	0.1144712560.122917591	0.1187676980.129808189
0.133145065	0.126109312	0.174260962	0.101458338
0.1193713820.124419617	0.106875966	0.142599957	0.162697255
0.152090088	0.142626514	0.143439162	0.127295516
0.153420399	0.127507426	0.155949861	0.147396453
0.125924608	0.1107089230.1601122390.084815166	0.1430719
0.135730059	0.145289129	0.137102388	0.124100382
0.1411750260.099216277	0.1411343210.145634199	0.132907649
0.122469452	0.161593014	0.129937709	0.120337082	0.11523273
0.1161061960.120586055	0.137523318	0.159589343	0.110877366
0.128312151	0.156243585	0.14814398	0.165093842	0.120175538
0.153075235	0.130740358	0.191628839	0.147516934
0.131491581	0.147322466	0.181059576	0.146456403
0.103024282	0.13993462	0.1150830970.1281198210.106595242
0.139068788	0.157069044	0.143989637	0.153976554
0.137431206	0.138242176	0.144068572	0.106927125	0.1501416
0.124975597	0.176122602	0.1171184390.083983412	0.11580468

0.1136389240.1147165130.146640486 0.138743879 0.097649796
0.125332977 0.122092052 0.130451802 0.1146696510.093449753
0.1109286 0.12872851 0.101017666 0.137026057 0.110379883
0.153031383 0.1126557 0.108207005 0.122006491 0.164615663
0.1196321390.1145098530.1405418110.105789162 0.109353091
0.190608192 0.124651656 0.139707051 0.145013039
0.158566017 0.153960178 0.140273413 0.1351145280.106568009
0.1237116890.122894258 0.131600448 0.189245966 0.159775007
0.15623422 0.139259016 0.107534722 0.1103096160.186838598
0.136593229 0.155077239 0.157034414 0.129656392
0.128521405 0.164285041 0.1121604370.165412046 0.142085913
0.163573093 0.137705245 0.162152922 0.161437466
0.144137295 0.1561157950.190824641 0.13947321 0.104484229
0.138054418 0.156338893 0.162065985 0.134658169
0.149812694 0.123312909 0.1122555160.107695152 0.157815877
0.097422696 0.136998192 0.133654338 0.143647765
0.1543953110.140912158 0.156338997 0.157028676 0.14648669
0.158309787 0.146224485 0.139661588 0.1143155460.132408113
0.126701672 0.145265668 0.10032368 0.154560826 0.115499576
0.1155492790.1091157020.1118105950.1171803250.1157238450.157956772
0.130799477 0.121004922 0.102104649 0.1149462160.145297721
0.155209451 0.120275425 0.142905319 0.162397959
0.125912385 0.129467013 0.1139127650.137493706 0.11734466
0.136373312 0.1158219290.175263383 0.146623286 0.109180331
0.144358658 0.133081837 0.120528898 0.133872128
0.131886529 0.100206754 0.145493449 0.142144563
0.1162230710.135038354 0.136688316 0.155459694 0.117587197
0.151022885 0.139313076 0.1168015690.1584241170.146418106
0.146510033 0.131567854 0.141533649 0.128606221
0.151588687 0.120556891 0.155769096 0.126676475
0.121646555 0.151846829 0.166060066 0.135057339
0.147281493 0.136153777 0.127378592 0.143275522
0.140090132 0.141405534 0.127260292 0.160165977

0.135740943 0.111081955 0.116687113 0.158199942 0.113577907
0.105258112 0.119076771 0.130936978 0.151173569 0.119721931
0.149358715 0.097491045 0.151424822 0.127641154 0.169212737
0.142120705 0.091098718 0.122348952 0.122770404
0.139723778 0.144942589 0.132262653 0.152179036
0.105145203 0.145436758 0.1234389 0.11158877 0.136073106
0.142443674 0.147909748 0.15638146 0.171461272 0.130810618
0.160888064 0.123538965 0.138561384 0.11371112 0.147840863
0.127250594 0.165433698 0.149393066 0.139331566
0.123522282 0.136835232 0.137405985 0.125499876
0.130241903 0.140873017 0.195857413 0.141826628
0.136155465 0.132104795 0.114373807 0.150302016 0.157834517
0.131032662 0.150234954 0.152934755 0.118641923 0.150396507
0.123164379 0.153689654 0.127949115 0.156939225 0.116744119
0.129839865 0.128850454 0.133463216 0.125392077
0.146887996 0.129753591 0.085094205 0.19704966 0.137033935
0.096840825 0.116201171 0.121649603 0.136014097 0.111399183
0.1748452 0.122516498 0.093377667 0.131600206 0.119305855
0.135175737

KEGG_BIOSYNTHESIS_OF_UNSATURATED_FATTY_ACIDS 0.15054608
0.11417159 0.153531844 0.153162874 0.142448607 0.114661486
0.182867367 0.178827847 0.179700299 0.182500975
0.205726222 0.165016611 0.14364746 0.14897677 0.200709091
0.143081346 0.144708903 0.130138918 0.160155175
0.118787438 0.189594835 0.124836983 0.184410453 0.179025809
0.166043991 0.154633872 0.155607752 0.099534983
0.170489838 0.178297879 0.17093313 0.165881048 0.169850202
0.13056181 0.187198142 0.161445354 0.16223672 0.138501202
0.195970574 0.108625535 0.150085769 0.130427839
0.126306592 0.190938137 0.192556072 0.147379749 0.13758143
0.133746116 0.178631347 0.196564285 0.173975886 0.099068165
0.146611882 0.16767363 0.115469521 0.157458473 0.119504464
0.151271835 0.207737917 0.169727632 0.222174621

0.205304565 0.19076104 0.182807 0.192122688 0.173596412
0.157920134 0.212471641 0.140628593 0.176830946
0.213687429 0.156467151 0.14512963 0.074781839 0.102435698
0.161923867 0.184367401 0.187152082 0.145422545
0.133787398 0.2032511030.202237312 0.195801045 0.153122644
0.1177176230.181535597 0.130828201 0.152085 0.188953782
0.163091282 0.185699125 0.198229426 0.143193238
0.1381108620.163472521 0.204954303 0.1511198370.174737599
0.212771655 0.18555199 0.193626098 0.184131747 0.165147781
0.158455388 0.124608074 0.199523797 0.1127048390.189207746
0.190898724 0.16105132 0.156500463 0.177268236 0.18401688
0.140129307 0.1184943960.161868262 0.18435837 0.170781248
0.165703224 0.16436445 0.1166548740.199779507 0.162011603
0.169058701 0.178890572 0.120473391 0.161378714
0.124467147 0.236001943 0.14791761 0.186817578 0.153061359
0.154805568 0.13183067 0.182750192 0.180120778 0.105077268
0.151305162 0.151393208 0.1529811440.135102096 0.140962472
0.161627792 0.146153381 0.172454908 0.147606255
0.174618305 0.120270607 0.165262917 0.180372717
0.1177432680.101280655 0.193208518 0.129136024 0.17190503
0.203352129 0.166129937 0.125369948 0.161277196
0.1367911450.163274935 0.204022934 0.19562145 0.140460481
0.177950616 0.1184415860.207757279 0.167612872 0.150613718
0.1136532710.1876653 0.135762419 0.139746312 0.109686419
0.14242043 0.184083331 0.167904006 0.141077636 0.171239144
0.134973453 0.127622701 0.147027375 0.1431183380.132975883
0.133943828 0.190064897 0.169219175 0.159028887 0.16544647
0.097839955 0.140325654 0.126752355 0.193034902
0.1139279870.148600236 0.1155054720.171056228 0.128305731
0.194567141 0.137049493 0.166964488 0.175324754
0.181026474 0.152451587 0.101291626 0.198836045
0.201646037 0.135402892 0.175518065 0.176736781
0.159872934 0.1193199420.139643903 0.1199414180.157231727

0.13484974 0.144335618 0.200840076 0.155120092 0.194230337
0.164071235 0.180827538 0.1511153870.145326949 0.172632111
0.106667069 0.193379974 0.1146022450.191824599 0.18460273
0.192463917 0.158676305 0.193335539 0.199678823
0.178620464 0.184620838 0.168909792 0.180492669
0.200468386 0.148031051 0.204654448 0.153432508
0.249513351 0.1870041140.174605337 0.1138554780.174415929
0.224643371 0.199926183 0.166350218 0.183866338 0.09737297
0.1791185640.173962884 0.19635672 0.138816949 0.153035777
0.17450705 0.202996039 0.162551887 0.252626537 0.171588631
0.1911854020.145970252 0.197377106 0.137026439 0.189049747
0.131738705 0.18122115 0.130197643 0.15196704 0.175576862
0.169677442 0.172542536 0.194060416 0.143294462
0.178551424 0.22020537 0.183483501 0.152450134 0.231944221
0.189237179 0.204066966 0.16196236 0.186645616 0.140586837
0.138815334 0.140675935 0.193163628 0.15873919 0.202815508
0.121601372 0.174834543 0.179881983 0.225784514
0.198406103 0.185377561 0.170396142 0.178869136
0.140066397 0.215065681 0.169725359 0.183202818
0.124635631 0.167331759 0.147171779 0.226753576
0.217561514 0.175614295 0.187019375 0.208019398
0.171478285 0.17699769 0.173576138 0.15973334 0.133496391
0.195219544 0.200868193 0.168589852 0.1611024690.21273111
0.2148561190.134333425 0.1731785 0.130379438 0.159856315
0.15498399 0.193023791 0.152026823 0.181218382 0.128218924
0.1693115270.144872988 0.180846108 0.150972815 0.205533012
0.151931207 0.201331858 0.1011467010.154354029 0.170978593
0.192322382 0.15919831 0.173633263 0.135789528 0.126744774
0.11479601 0.132471814 0.141581879 0.094629064 0.150002118
0.17646574
KEGG_BLADDER_CANCER 0.2116049660.273928136 0.261794549
0.254153634 0.239661962 0.215823505 0.215903598
0.279884025 0.219765477 0.21851104 0.218254752 0.275255173

0.212655451 0.200353062 0.305825394 0.234134048

0.223064603 0.2031176470.234619167 0.217478345 0.22372266

0.247846602 0.241412085 0.2466134110.26326976 0.230332296

0.239090795 0.214328791 0.242180555 0.240038907

0.205297795 0.251491591 0.246066241 0.210661364

0.241259741 0.224702857 0.261817888 0.2116992950.230508303

0.2119454130.262247184 0.238958429 0.220321317 0.223243713

0.263399712 0.22255989 0.26641546 0.233086464 0.225929574

0.254758531 0.279882434 0.227718285 0.245864694

0.206003913 0.233136854 0.292922753 0.222950641 0.24843904

0.249748935 0.2359331110.230795817 0.25655281 0.245716519

0.2847325110.243192383 0.2099961150.2119140470.23784289

0.250563964 0.254325671 0.26379894 0.230953848 0.285627142

0.180576419 0.256508223 0.29774282 0.249899924 0.281970033

0.284435585 0.247523031 0.239855084 0.199777185

0.213396057 0.244941382 0.217169389 0.261705607

0.259788193 0.256314439 0.253086371 0.228259184

0.2474118260.258487044 0.22741172 0.2278113530.258612626

0.291774012 0.244935532 0.246928321 0.262445449

0.260497748 0.251355543 0.246328876 0.223917602

0.234767096 0.215236762 0.23324496 0.20191396 0.286552896

0.252228496 0.281487559 0.229547724 0.272510032 0.23433049

0.230856853 0.241980175 0.315335454 0.253399583

0.212109823 0.247786134 0.273209499 0.262672484

0.254561703 0.235140674 0.278677422 0.244545061

0.215937595 0.252866693 0.240664176 0.213148266

0.251671818 0.259723832 0.209718931 0.2309116 0.269338924

0.261715973 0.224327795 0.254558543 0.254135055 0.28257048

0.282328187 0.216698731 0.26147513 0.260006478 0.230518498

0.227381344 0.297719585 0.286019982 0.22472516 0.2405889

0.260359525 0.261486336 0.2117651210.256699555 0.229007438

0.252049386 0.248816819 0.310796547 0.265571778

0.246635874 0.23479197 0.229370141 0.247606281 0.238195986

0.264298285 0.276487145 0.174516457 0.214792786

0.236038683 0.206532884 0.253663825 0.282877072

0.247497994 0.209241745 0.23621672 0.23781367 0.272003251

0.267031233 0.2420101190.237055392 0.299760943 0.253137408

0.262067036 0.250773963 0.259472429 0.27366594 0.233667751

0.253958537 0.265131987 0.232752201 0.224665844

0.280990109 0.247201732 0.297490476 0.2546411560.2390598

0.252368537 0.258908972 0.277357675 0.256300409

0.288685524 0.273223414 0.248459082 0.264819267

0.244204843 0.21653706 0.222162723 0.189237794 0.23088926

0.259371359 0.222161279 0.288637788 0.22464775 0.267432042

0.253837991 0.264198236 0.25944887 0.256979333 0.284990696

0.281965626 0.271725566 0.27126784 0.274904613 0.230244526

0.224824471 0.262177999 0.203231654 0.257938097

0.199868217 0.255755193 0.276912105 0.246189161

0.233545252 0.255032209 0.234453572 0.279650168

0.274299721 0.239601043 0.278827972 0.289275223

0.242010491 0.252590221 0.249176145 0.2111693710.249249867

0.264399156 0.28240632 0.175994954 0.219282519 0.258166234

0.241836921 0.25698189 0.258359973 0.264837661 0.23048121

0.24567496 0.261292182 0.251934504 0.237648617 0.256521769

0.275525121 0.214328198 0.223369749 0.264756429

0.239229627 0.245722077 0.252248735 0.228964281

0.256604695 0.219840752 0.264044894 0.239260742

0.277092825 0.292699745 0.2114107540.262986652 0.206801414

0.250566426 0.259284343 0.25129677 0.228983405 0.247844505

0.275792364 0.248389962 0.264795164 0.292047675

0.2422110460.287633876 0.284781305 0.307043906 0.281471437

0.238868443 0.215240831 0.2114752720.234244951 0.291703863

0.303591951 0.257488229 0.258064234 0.26127383 0.252190162

0.238369436 0.188495301 0.270821609 0.230632374

0.265253858 0.262055863 0.266679863 0.234231789

0.257946319 0.236153146 0.257189698 0.232869935

0.271956019 0.276042718 0.258293307 0.206479153 0.25082281
0.2409161 0.260014352 0.249275141 0.297128336 0.203482293
0.253027933 0.240748843 0.23056815 0.2311870910.228264637
0.237327499 0.247821676 0.297733994 0.256952367
0.299750622 0.270370687 0.260753559 0.2711210380.245711058
0.287642275 0.27649428 0.22137345 0.228835238 0.270075594
0.262387454 0.245410933 0.262760687 0.269050193 0.23920982
0.238893452 0.242758941 0.338307626 0.180453842
0.236552639 0.212858722
KEGG_BUTANOATE_METABOLISM 0.101348304 0.100178995
0.095998629 0.134282134 0.1661130160.124764132 0.139003593
0.09015418 0.109138743 0.081987406 0.094071437 0.114894448
0.096453707 0.146300507 0.098037312 0.137316147
0.073582446 0.175854436 0.1108188270.108249976 0.142654628
0.110811133 0.130740132 0.143179645 0.087607371 0.121016685
0.1110687770.143857947 0.100135164 0.133133205 0.134472528
0.135356234 0.090033899 0.102645739 0.122854574 0.13659609
0.109213523 0.123149661 0.082402609 0.089763831
0.094397299 0.11431915 0.1144572440.160132746 0.086986638
0.10530612 0.080630376 0.069386178 0.1711851490.141552155
0.098449122 0.1151856340.137863059 0.109279494 0.155697403
0.108438209 0.10934639 0.104003493 0.135375586 0.079361295
0.190225028 0.077185401 0.122017744 0.12212622 0.145649593
0.141019954 0.144429934 0.120567319 0.080129031
0.124899887 0.107192097 0.104173239 0.097710812
0.1145510240.095556422 0.053506944 0.109193009 0.06830452
0.09849667 0.128864684 0.169736235 0.126531606 0.152408702
0.108779735 0.1277557 0.153232453 0.1093611440.09980175
0.091233391 0.149775728 0.160099035 0.149641686
0.1170379650.1496113270.126559227 0.063498799 0.120762462
0.093806597 0.142384815 0.088189625 0.123596098
0.121870661 0.140145453 0.1159315410.132787922 0.13970215
0.143341846 0.085641371 0.12928696 0.096025362 0.160252347

0.093698901 0.085283558 0.082161254 0.104744659 0.05925661

0.121775555 0.129657212 0.07253551 0.1297011730.113696782

0.1166102180.109933912 0.046648363 0.085628322 0.158120367

0.128896162 0.103615609 0.164039534 0.1357117590.089799863

0.088836179 0.1117562410.1198498740.102062946 0.115262537

0.071852588 0.1150887050.086489068 0.1158670720.108939279

0.091012168 0.140092441 0.149850554 0.147635133

0.104212868 0.100185566 0.083906401 0.102041687

0.125084656 0.12891088 0.164625045 0.164633547 0.136396141

0.122343257 0.090606764 0.036466002 0.148356917

0.136026438 0.128039952 0.102809069 0.12588656 0.138521792

0.129290344 0.092275663 0.143336404 0.0979068110.164199752

0.1180501830.168747555 0.107703002 0.148468052 0.17627571

0.153368298 0.099962474 0.100305306 0.126535989

0.130305595 0.144000168 0.105627619 0.1135216920.108764431

0.094126991 0.06903427 0.150438884 0.080390535 0.101963972

0.077082378 0.138759193 0.12089081 0.128485604 0.092360191

0.143818632 0.095773374 0.1205535110.093470179 0.098142948

0.092416719 0.067865674 0.1170390690.082307633 0.112381537

0.140928676 0.096272248 0.08811398 0.11094329 0.076639533

0.097806291 0.107534028 0.179650279 0.071263433

0.1188738920.066668319 0.0911192140.13793795 0.112762989

0.077664463 0.08780651 0.077375838 0.1198690270.080851572

0.0760112720.106575503 0.107664042 0.088943579 0.0885613

0.1119910670.13191473 0.092603333 0.064779057 0.10841885

0.109402499 0.07995766 0.13592299 0.079029736 0.119158624

0.177501879 0.103555986 0.128510181 0.123629186

0.102613428 0.070168901 0.172277765 0.12810683 0.075153943

0.126804146 0.132830207 0.2104124110.162049806 0.140135611

0.104177158 0.100463231 0.1126542430.16091323 0.139285514

0.09046035 0.130120983 0.090320531 0.123055725 0.11303916

0.145267612 0.108104535 0.1188545360.123100567 0.116395726

0.078024136 0.165252767 0.090447645 0.097993312

0.100645972 0.132302639 0.1211318740.053354732 0.113459589
0.1157609770.138630365 0.0844115640.142969204 0.084806315
0.1192972770.124278897 0.127877647 0.1146341820.102709614
0.103508023 0.130647098 0.090331948 0.109583229 0.09015124
0.1181044410.087008472 0.097220273 0.100214691 0.096614784
0.128988406 0.092052419 0.13748355 0.080909371 0.172042669
0.127821315 0.15077731 0.122139516 0.10201681 0.098175869
0.169077203 0.09347516 0.1162938060.144820133 0.145824253
0.100010006 0.123005488 0.09841371 0.091263256 0.143747483
0.094677806 0.125261335 0.14627374 0.109378246 0.141817233
0.08796204 0.102442619 0.184560405 0.128030743 0.10385682
0.133629547 0.089888883 0.11402563 0.143318345 0.148535402
0.086446937 0.145836687 0.09366102 0.068068361 0.076315058
0.084593147 0.1167712070.138813664 0.133374955 0.089861062
0.215739976 0.095526229 0.08377137 0.076838345 0.044802995
0.09446596 0.120123183 0.131774389 0.1159498120.051116952
0.1128964070.140520104 0.128222176
KEGG_CALCIUM_SIGNALING_PATHWAY -0.05807538 -0.016876511 -
0.042140669 -0.055135827 -0.048724103 -0.079303795 -0.070136287
-0.047776171 -0.05039632 -0.052113624 -0.069173012 -0.064545344
-0.052146213 -0.050755399 -0.042753751 -0.056324331 -
0.052333424 -0.063392448 -0.042487238 -0.060128225 -0.068792526
-0.063743657 -0.061363588 -0.036974385 -0.048616451 -
0.065201391 -0.065951038 -0.054475055 -0.074005784 -0.046045192
-0.087106883 -0.053855437 -0.062262138 -0.056944589 -
0.05344078 -0.071681177 -0.063823492 -0.052570186 -0.062117633 -
0.072037366 -0.030980367 -0.057787403 -0.060978572 -0.057307018
-0.037383085 -0.063796467 -0.040945913 -0.057397812 -
0.055540364 -0.071757597 -0.023439052 -0.05236452 -0.063648236 -
0.068531342 -0.056516438 -0.043692617 -0.061840757 -0.046193083
-0.074282726 -0.072216299 -0.049645149 -0.028971464 -
0.057014947 -0.044045107 -0.051851796 -0.085407398 -0.067129174
-0.04173487 -0.055816652 -0.057541677 -0.023455102 -0.081762866

-0.038860777 -0.049421848 -0.048433305 -0.030056805 -
0.046669468 -0.055275271 -0.031973474 -0.052794707 -0.056229198
-0.065427705 -0.071820151 -0.067256356 -0.066257244 -
0.072252349 -0.061165283 -0.05090182 -0.029660972 -0.055535343 -
0.06206204 -0.060439199 -0.050342809 -0.070816053 -0.047012555 -
0.04318281 -0.062051439 -0.067104842 -0.043441341 -0.047571712 -
0.033503845 -0.041758886 -0.049640221 -0.036393221 -0.066169635
-0.033320306 -0.061785706 -0.053372278 -0.069293874 -
0.055364582 -0.076349321 -0.047395788 -0.065450574 -0.061607561
-0.042167429 -0.030978315 -0.059920348 -0.06263328 -0.048000956
-0.060557316 -0.05560214 -0.040611602 -0.043946798 -0.037054552
-0.037443197 -0.064725364 -0.061878544 -0.059023291 -
0.063962617 -0.047708729 -0.037096303 -0.054993231 -0.050716761
-0.033695428 -0.045519114 -0.060631002 -0.03377181 -0.045156789
-0.053539219 -0.021382969 -0.062509391 -0.022793871 -
0.061312086 -0.065196481 -0.059810474 -0.028903653 -0.036339772
-0.062307937 -0.064570727 -0.064405996 -0.044188188 -
0.065928524 -0.049226631 -0.07629681 -0.02334829 -0.031948567 -
0.019713596 -0.048191517 -0.037092791 -0.02748786 -0.040803876 -
0.051690413 -0.061655493 -0.039005856 -0.044954723 -0.053840925
-0.031796705 -0.046586434 -0.047086391 -0.049539153 -
0.043269392 -0.064582306 -0.058818165 -0.083663886 -0.061460916
-0.032862807 -0.051932316 -0.02887505 -0.063386105 -0.030110353
-0.041132085 -0.042150101 -0.070538313 -0.051314935 -
0.059811762-0.04666433 -0.039662585 -0.022476591 -0.056010158 -
0.065814927 -0.042429109 -0.043738908 -0.019266914 -0.048639431
-0.029672163 -0.039917327 -0.052193813 -0.03674202 -0.033160631
-0.056518588 -0.013982601 -0.069541083 -0.040402928 -
0.059710291 -0.04160651 -0.063792757 -0.024072448 -0.043880582 -
0.021708951 -0.048374403 -0.045821071 -0.050149909 -0.045187507
-0.050497474 -0.041677747 -0.036990124 -0.02358871 -0.024234877
-0.013730967 -0.056060859 -0.034812556 -0.060690964 -
0.048404351 -0.05554783 -0.026310048 -0.062648609 -0.037744611 -

0.072576502 -0.071642174 0.00218085 -0.061066875 -0.032488235 -
0.057317168 -0.053989076 -0.037146421 -0.035298855 -0.056650997
-0.03537358 -0.026559515 -0.04893048 -0.058462246 -0.042466799 -
0.064910184 -0.063477899 -0.032170599 -0.044147568 -0.075822641
-0.064281749 -0.065975362 -0.061081258 -0.037304848 -
0.034066068 -0.04625268 -0.073840839 -0.067298256 -0.057385087 -
0.056365919 -0.042320887 -0.067516918 -0.046134894 -0.073184626
-0.047560039 -0.060213266 -0.053398147 -0.051440467 -
0.044626508 -0.05240996 -0.046980751 -0.047652403 -0.060159663 -
0.031661277 -0.037736464 -0.048756588 -0.073623532 -0.057850997
-0.056394815 -0.036609362 -0.035682891 -0.03614105 -0.046943643
-0.061131112 -0.042927321 -0.042193631 -0.042650568 -
0.049302395 -0.060585978 -0.042805133 -0.036791776 -0.025210343
-0.03752091 -0.045287341 -0.029110786 -0.066591234 -0.062635828
-0.039477501 -0.036350719 -0.066871627 -0.04092088 -0.05063774 -
0.049200123 -0.04190628 -0.04604611 -0.023951909 -0.071487746 -
0.039566172 -0.050839164 -0.026356106 -0.048114741 -0.037505199
-0.048083969 -0.029322591 -0.038916211 -0.069117129 -
0.060519755 -0.062983913 -0.001793023 -0.035090994 -0.058018518
-0.052341574 -0.051500153 -0.048432171 -0.059869976 -0.0614704
-0.056216988 -0.073715559 -0.064661847 -0.043076706 -
0.06790438 -0.059206344 -0.050330306 -0.042196237 -0.054077084 -
0.052159255 -0.041077711 -0.016535122 -0.029888927 -0.046222923
-0.044805041 -0.059250059 -0.06400827 -0.039760309 -0.051068573
-0.032341138 -0.011449402 -0.062663623 -0.050176771 -
0.049127771 -0.046761427 -0.048248442 -0.049591706 -0.05684269 -
0.067000131
KEGG_CARDIAC_MUSCLE_CONTRACTION 0.133667612 0.145873033
0.145624294 0.1568119610.154926044 0.124246371 0.158214086
0.131090552 0.1395715 0.138124921 0.171807615 0.128495596
0.148401239 0.139705143 0.150365375 0.142523518
0.150173798 0.102919676 0.12848076 0.159664837 0.115048066
0.1189581940.1511496340.155989475 0.109848873 0.114307829

0.126802031 0.129850426 0.124868608 0.11601357 0.157435655

0.103506408 0.1421121860.152832695 0.159370783 0.145756923

0.161029802 0.142813027 0.1362111970.1178218830.138220569

0.1189974040.135631505 0.135480055 0.142395385 0.136203468

0.15967205 0.15242089 0.142453939 0.142091267 0.140211443

0.142988557 0.1230460110.157297833 0.12834558 0.126959689

0.1189187450.1199696510.138809908 0.165746906 0.136279057

0.1399311310.138127122 0.152336087 0.165498723 0.144825781

0.136029685 0.147210135 0.132906332 0.14213683 0.133034046

0.163692366 0.167813523 0.165875779 0.134414036

0.135372173 0.150740267 0.135141218 0.145879434 0.13180463

0.122754367 0.137407473 0.127478332 0.132432401

0.133875689 0.134385235 0.1153738560.1469111360.17250372

0.129920793 0.140964197 0.12935961 0.134237734 0.112006958

0.1211649180.149102914 0.122480871 0.151379653 0.125065547

0.131323509 0.13345792 0.1174915880.135995148 0.132471742

0.16610625 0.132790932 0.1165899290.131274892 0.138593279

0.137086739 0.149224245 0.134471932 0.148999387

0.126950293 0.165252143 0.13304174 0.125219999 0.154134694

0.1147897120.131111109 0.097416757 0.1128989160.130144281

0.133988636 0.1175782120.135259726 0.138721741 0.13897755

0.125741584 0.140824098 0.1811319880.135850373 0.153935262

0.1130912630.126806802 0.142657683 0.107670817 0.136060292

0.1108949 0.1193272490.137561881 0.134889531 0.120807153

0.1142299040.1168212870.116884311 0.127084165 0.131430882

0.097203162 0.153914583 0.121506398 0.125456156

0.095245567 0.137067656 0.125419284 0.122933924

0.1174621570.132762019 0.126958444 0.130628585 0.136265443

0.122270894 0.11885617 0.132903507 0.130076466 0.128853368

0.132565739 0.121666808 0.104185685 0.1130458310.124372886

0.139038152 0.133497605 0.124252342 0.1124351870.119475176

0.146704814 0.150153517 0.1173857340.120245752 0.131210644

0.132144701 0.1115200770.1286011120.132509825 0.140372622

0.132151577 0.1406118210.131569097 0.124654275 0.108676351
0.109651826 0.122730241 0.102220751 0.134219924
0.154822673 0.120937392 0.128823754 0.136199936
0.099231581 0.135020453 0.140689593 0.147735363
0.134286572 0.151009983 0.136309199 0.143282474 0.14596492
0.129974968 0.088934317 0.1139218550.132102786 0.124366122
0.155304533 0.1195200010.120826141 0.126668003 0.120340054
0.130524795 0.133802863 0.132055731 0.12606783 0.129077384
0.130640867 0.130246319 0.132461927 0.1194574440.12223753
0.171850629 0.145883138 0.130848854 0.132003876
0.123474044 0.107203546 0.127375344 0.1167260670.104913582
0.1186515 0.13234708 0.1168182520.1152535890.1196049960.131367071
0.131920849 0.135753568 0.10098662 0.122195378 0.133765801
0.130968788 0.122580539 0.129858072 0.108802249
0.1186552930.131740869 0.141099286 0.1113803920.160633019
0.139239468 0.138770931 0.158739355 0.123305836
0.1180550920.12895207 0.1175367790.140930779 0.123353291
0.130369545 0.11035219 0.131552409 0.123250771 0.114247095
0.146877398 0.102100721 0.142932237 0.1171713690.177859756
0.140056309 0.122640453 0.138246268 0.1178099740.160675684
0.142025865 0.156028942 0.125267215 0.132262321
0.128310962 0.122397143 0.122587508 0.1190111970.110328756
0.125174431 0.126580523 0.121376143 0.133888625
0.108905461 0.1331311530.13878321 0.145785101 0.097486869
0.122449259 0.1176903450.1186280480.120505928 0.119231631
0.135522299 0.12045691 0.1173222790.143196598 0.123881109
0.1194788650.133779053 0.14272393 0.1148895330.116328859
0.141402405 0.1511066230.126921541 0.144318205 0.134481665
0.14856801 0.1132481530.1001169980.1263110690.131067921
0.124059465 0.121043507 0.162593945 0.125617766
0.108669722 0.148389124 0.1180372950.12789466 0.112247494
0.1191583340.123202431 0.124355507 0.171204327 0.123862162
0.139335275 0.106651054 0.1102325450.145717425 0.139785069

0.122929728 0.099854385 0.136578883 0.144507573
0.138129688 0.1171396830.127272639 0.132327184 0.13454467
KEGG_CELL_ADHESION_MOLECULES_CAMS 0.147713855
0.234661337 0.263440012 0.180361422 0.244187979
0.153753364 0.122568616 0.178348942 0.28496048 0.195517153
0.09377274 0.2812041160.078761447 0.200720527 0.276597839
0.201730977 0.251367998 0.313702377 0.207508419
0.196422827 0.062389359 0.260556345 0.260362515
0.158868329 0.270410745 0.260751445 0.255292561 0.24796046
0.21619563 0.227477856 0.16878533 0.277329897 0.159283567
0.200697296 0.234148497 0.216340203 0.156131386
0.244885598 0.26799111 0.079650488 0.238312313 0.172955203
0.215192932 0.262710407 0.2114551340.220521844 0.223300242
0.1921113740.2658031130.2288116870.319342892 0.29567084
0.229081407 0.1004011340.275099591 0.26208436 0.292273301
0.306180518 0.260722939 0.135007802 0.256724523
0.277899559 0.191200928 0.293148252 0.321309099
0.203926837 0.240842747 0.277795086 0.233352657
0.256022429 0.229298172 0.095783346 0.225410037
0.182809952 0.231314799 0.3110803310.160871035 0.260639924
0.203499506 0.195615549 0.286302285 0.1178580120.153739508
0.19906549 0.300617895 0.186943207 0.308046209 0.297699115
0.191642731 0.260076057 0.232438865 0.285742893
0.221729272 0.295443324 0.233894513 0.242154428 0.28472861
0.197663547 0.296454732 0.312293479 0.225087686
0.174165606 0.170416723 0.287896475 0.151558754
0.309663677 0.171089201 0.258958165 0.289094049
0.2411096960.20754076 0.246710309 0.064028928 0.13982632
0.176034594 0.272706467 0.257635875 0.263849625
0.284615818 0.2062034 0.260651893 0.146687309 0.286591221
0.239108426 0.219948041 0.23268954 0.244566793 0.2255184
0.1711983390.246522749 0.251298306 0.271834619 0.204595209
0.289466638 0.221456674 0.163741677 0.279714358

0.260913594 0.1904930110.264099372 0.199455803 0.279061738

0.262135755 0.170379236 0.222283122 0.291806493

0.281079809 0.235929127 0.219455039 0.238247785

0.307839917 0.23332598 0.246828348 0.134525615 0.272876354

0.318662374 0.250658672 0.291690318 0.156327806

0.329410543 0.2395576110.182805645 0.1844511690.266318787

0.2862996 0.27937501 0.1627719 0.276420781 0.235966326

0.208651233 0.28696325 0.251021314 0.179798296 0.204543488

0.287658745 0.305491925 0.245974129 0.251249383

0.291526748 0.199260403 0.298204258 0.1124130950.220832672

0.268906481 0.254476328 0.153775997 0.256521288

0.283695903 0.174903306 0.219225752 0.252754576

0.303599872 0.2366114050.2911345350.279818586 0.224523993

0.293458274 0.246070572 0.255318617 0.236321617 0.28864126

0.205578164 0.23519809 0.237542048 0.262187789 0.112216814

0.252260787 0.19026064 0.231968468 0.274478343 0.229960661

0.214134369 0.205745926 0.16306638 0.26074279 0.267017808

0.181337901 0.296301657 0.289026555 0.202346253

0.289058232 0.257013573 0.230942612 0.148689227

0.292797216 0.136917394 0.259488097 0.278131971

0.226889514 0.259204474 0.275249057 0.271908123

0.219709412 0.242635721 0.268302751 0.274169581

0.223486321 0.282314491 0.248382772 0.282451586

0.255714337 0.293656694 0.252594014 0.249466768

0.281233918 0.261012947 0.24248791 0.126293792 0.254180898

0.242783238 0.258704342 0.322573956 0.280153008

0.250877973 0.145502594 0.26586884 0.24120995 0.30361163

0.216532663 0.238591635 0.1115526470.242905969 0.257271557

0.248651907 0.3072447 0.267490255 0.294518695 0.302897141

0.232151964 0.178031278 0.339533973 0.288049381

0.172703056 0.233642178 0.234356477 0.149594281 0.28463567

0.301593382 0.195126395 0.216862981 0.228958182

0.258180401 0.220798023 0.2285111520.282533844 0.30005058

0.272931028	0.3081172390.353051074	0.278997826	0.254981773
0.30771394 0.26586188 0.125548979	0.259057279	0.278935118
0.155959723	0.298949479	0.300088393	0.193300785
0.166604668	0.265917161	0.284372184	0.242854644
0.210453334	0.298309171	0.206386599	0.222645503
0.268440434	0.263205778	0.298022587	0.2491405	0.165166479
0.164990975	0.1128643460.255643124	0.207481674	0.269431862
0.233969318	0.21303934 0.256031531	0.2812636	0.257220208
0.185259822	0.303390425	0.191622177	0.269401852
0.242197769	0.284644631	0.28921506 0.274545263	0.263807816
0.278722795	0.3218292110.280008396	0.2761125140.269128272
0.283752502	0.101483977	0.175630375	0.235496108
0.099189343	0.229575365	0.160870944	0.302718293
0.239559612	0.194887906	0.233383891	0.241926459
0.1153342360.159982094	0.139757839
KEGG_CELL_CYCLE	0.277213623	0.236087825	0.258270542
0.259694686	0.240128819	0.241694402	0.264850365
0.254481932	0.246208804	0.256752541	0.304129329	0.29315611
0.272086549	0.22677648 0.284207877	0.267567404	0.24186589
0.209567974	0.2595118110.254288558	0.282558493	0.23208805
0.232223223	0.256033327	0.252240436	0.267209393
0.273230043	0.264185896	0.285793792	0.188470541
0.272697325	0.280908043	0.290077197	0.232743163
0.279380739	0.249178655	0.296668408	0.246137351
0.262072686	0.30018789 0.243553107	0.234210574	0.267866783
0.266392314	0.230782495	0.263876882	0.273353848
0.231765514	0.224375447	0.275707185	0.2404701110.262563636
0.274023194	0.257725361	0.262091516	0.238663287
0.281522915	0.26344822 0.269427739	0.255367233	0.262522308
0.262581651	0.29161554 0.25822305 0.2738061160.280580415
0.244609743	0.263546899	0.24781979 0.283372047	0.268791288
0.300171029	0.252231575	0.228570338	0.276836597
0.250019572	0.235786371	0.256147864	0.218089721

0.273099766 0.272571873 0.267683277 0.290060326 0.27667801

0.272679737 0.278557482 0.261583047 0.258760906

0.262187721 0.249142942 0.287960991 0.268821898

0.2621117010.267510534 0.249275797 0.277014254 0.287323279

0.279849094 0.246240573 0.262366796 0.270935094

0.300030319 0.2513118310.228746494 0.254812791 0.236502876

0.27650386 0.254526694 0.256758192 0.262857178 0.284109239

0.293317246 0.296569774 0.248337529 0.272091943

0.260438835 0.279678778 0.2404114090.262780858 0.294910595

0.267966848 0.292730258 0.239952519 0.282857315

0.291019151 0.252585166 0.271454665 0.27742952 0.253197221

0.22488023 0.258540534 0.226891605 0.275739583 0.273121378

0.28641174 0.26318082 0.244066738 0.237720016 0.291965841

0.241763622 0.254256699 0.248369712 0.270487532

0.260842995 0.270519139 0.258251301 0.273929303

0.253767217 0.256042391 0.2971177150.2720118290.249381896

0.321387158 0.265153737 0.202317876 0.288746856

0.273327813 0.271406937 0.28171034 0.219163542 0.258637827

0.265642501 0.260807399 0.254225047 0.249807371

0.197795095 0.26451824 0.276017312 0.225806702 0.270844838

0.280571977 0.296003148 0.241957484 0.254482353

0.265098813 0.256992324 0.260651279 0.253343684

0.276033296 0.269278425 0.227242607 0.287290973

0.273057374 0.220778717 0.266925155 0.2909809110.268918943

0.248168435 0.262730842 0.185996569 0.259103442

0.284850836 0.270559484 0.240151274 0.224998303

0.255980325 0.277887833 0.2560115620.224884486 0.29527439

0.245507165 0.270755899 0.251497414 0.262655959

0.217554029 0.269760483 0.255024147 0.234934247

0.229646223 0.258334933 0.2748546110.245788963 0.287537532

0.269483842 0.254409289 0.196972495 0.257182905

0.273373442 0.240006391 0.2681401180.278863938 0.258986637

0.251040608 0.240751705 0.234858043 0.245464362

0.268923499	0.21419005	0.28092326	0.226390815	0.265077801
0.232238348	0.295843751	0.279852188	0.242647777
0.267612388	0.265200345	0.265979976	0.267307148
0.226722067	0.286467831	0.204415406	0.301882363
0.237887959	0.219853315	0.278385109	0.235366756
0.291790373	0.290596046	0.290797902	0.268719098
0.232549069	0.241615565	0.275525035	0.2887423	0.242135557
0.259890566	0.2347191140.286041343	0.262246912	0.266360747
0.231038609	0.263808504	0.24639288	0.272898854	0.241084115
0.267792136	0.269684904	0.233972543	0.262936109
0.247588658	0.258647742	0.272527572	0.273772774
0.272708146	0.235768954	0.236386236	0.273039997
0.253863226	0.213631668	0.291402382	0.260853782
0.251040943	0.264155153	0.257428739	0.264776067	0.26325327
0.269807856	0.256981616	0.241062696	0.223862965
0.208379291	0.278764404	0.272615775	0.2931119990.276896801
0.2911979470.22073349	0.28587799	0.291209028	0.277057707
0.206957539	0.242269265	0.2558117420.283636559	0.260564572
0.253842937	0.283532324	0.278586426	0.223550443
0.244706553	0.229315465	0.286521725	0.297268664
0.287443735	0.175408997	0.253487224	0.256126328
0.259978549	0.249067529	0.279822427	0.252329551
0.289487641	0.268218967	0.241409304	0.239492943
0.242978865	0.250497566	0.240907648	0.271820813
0.282258501	0.28593683	0.290085309	0.264467561	0.252534875
0.251864279	0.237728306	0.2676357110.21984739	0.24574483
0.212438246	0.271089138	0.237347772	0.287468819
0.245274412	0.278496932	0.2481311410.251814088	0.280602331
0.232679898	0.26695263	0.273801476
KEGG_CHEMOKINE_SIGNALING_PATHWAY	0.050409544	0.091176303
0.137783091	0.050897628	0.102696822	0.022752296
0.022836688	0.094629663	0.072855129	0.08215481	0.005577353
0.1183833880.010676571	0.029721374	0.106694196	0.079276749

0.027256355 0.07317 0.073029667 0.023952921 0.019982148
0.127698191 0.094508954 0.04167012 0.111074382 0.087873304
0.079065397 0.066759559 0.054208082 0.080866775
0.014837317 0.087769786 0.05682779 0.040141395 0.050259004
0.084582165 0.046816047 0.066607232 0.089893408 0.00973514
0.088655229 0.073287642 0.059225944 0.075313345
0.057148537 0.052173592 0.0708199 0.039321378 0.096080603
0.079175125 0.169475375 0.103752108 0.085660612 -
0.004989666 0.111968981 0.102868292 0.114569249 0.129449942
0.09437408 0.010803738 0.052312179 0.089690704 0.083483314
0.120380464 0.07943133 0.037665795 0.059513858 0.143474594
0.055385565 0.087441484 0.099879958 0.008419531
0.081439721 0.007294426 0.084957111 0.165531794 0.054607407
0.096257125 0.062005515 0.052096935 0.103127522
0.026794448 0.03572082 0.072294489 0.124884442 0.083053627
0.126848349 0.141024672 0.066199957 0.059448386
0.072469485 0.136133338 0.090561414 0.057350174
0.066137643 0.095392239 0.092288573 0.04871469 0.130312467
0.175092592 0.109585218 0.052737144 0.056042539
0.097491645 0.035672945 0.104714257 0.040134429
0.097899942 0.104420735 0.083308523 0.06648966 0.100580208
-0.001038114 0.034571083 0.034576434 0.153795544
0.090257844 0.09175846 0.097040373 0.083076368 0.098423662
0.055081562 0.076594437 0.100641101 0.081553103 0.062146142
0.056357366 0.053157516 0.050474093 0.123718439 0.08350783
0.049112355 0.080203476 0.107239305 0.080011406 0.038655054
0.12104835 0.08472951 0.076100534 0.108122714 0.031092683
0.095827276 0.123551642 0.025428553 0.072966662
0.139603446 0.10161519 0.070533558 0.044193856 0.076829803
0.117719097 0.068661393 0.074610189 0.02484576 0.093394375
0.093826724 0.135956961 0.120734843 0.069440152
0.148401974 0.06673378 0.055063633 0.042953095 0.099283673
0.115528498 0.060855478 0.032464115 0.067835844 0.070676252

0.058414269 0.1110730250.108741585 0.027027619 0.091060878

0.0968911860.139322469 0.100650894 0.072353573 0.126095094

0.089158798 0.11851599 0.034805781 0.092581045 0.083509287

0.1190419440.03392026 0.069409056 0.148528083 0.026703742

0.06089464 0.099331619 0.1191405680.1136094050.133571441

0.1134324640.07682843 0.128184144 0.108917485 0.122958039

0.107624488 0.1197339110.032300351 0.083318194 0.07066699

0.1086191 0.009957212 0.072713272 0.065235649 0.090523493

0.063753861 0.091985594 0.057517015 0.105568647

0.058661928 0.098256317 0.085519975 0.067372998 0.15502579

0.165292049 0.073807657 0.101509554 0.1491148680.059212101

0.0311237680.1117045260.016095444 0.107890506 0.077327559

0.065450654 0.1196177370.09137811 0.090451668 0.115015088

0.098358669 0.090448453 0.096301419 0.055292338

0.137949702 0.086692148 0.085063955 0.105983862 0.12117145

0.048382861 0.072557586 0.08934807 0.132415029 0.057255784

0.035876514 0.102092057 0.071368937 0.071787289

0.138254368 0.1140049030.080577981 0.0471182920.061530318

0.095463677 0.1179952340.08725523 0.072000909 0.011789561

0.08496733 0.11071851 0.096841549 0.126769968 0.105418006

0.109519429 0.1134261980.032592337 0.071776181 0.13734123

0.128221398 0.058265094 0.055166019 0.090806817

0.002292493 0.107515671 0.132617599 0.042083258

0.076991456 0.096860521 0.091641283 0.067465866

0.090456426 0.1140559710.084312762 0.108177413 0.141561741

0.1728388 0.1169158110.0820511350.126555566 0.0525641

0.029358196 0.1126884310.138998174 0.032702183 0.114124958

0.1163805110.054147854 0.052644047 0.060826498 0.121387109

0.049904437 0.044336924 0.093604272 0.07671004 0.066378538

0.104672282 0.089343961 0.104725975 0.085080132

0.053185099 0.066100898 0.028992836 0.11989965 0.073296892

0.0790171110.070307006 0.069704527 0.102974589 0.079791786

0.069767783 0.05472828 0.131293767 0.054556068 0.123552227

0.103120849 0.071600249 0.147104891 0.089366223
0.084980629 0.097598916 0.131635385 0.126524854
0.101718477 0.081894256 0.136196749 0.03192581 0.036299187
0.130867634 0.0251104330.0639011160.068519408 0.121830256
0.066595637 0.054204972 0.082999281 0.125925396
0.017745618 0.042464522 0.00151735
KEGG_CHRONIC_MYELOID_LEUKEMIA 0.232678107 0.261403108
0.249640453 0.237333353 0.236773578 0.230232001
0.226255601 0.23152073 0.238770588 0.257690239 0.223830091
0.245953853 0.246298397 0.239621585 0.264278767
0.243853093 0.239568355 0.223328329 0.233040655
0.229948055 0.255156004 0.260577699 0.231030928
0.221863426 0.268299848 0.254424817 0.241715602
0.261317427 0.264238993 0.26385171 0.214295777 0.244439009
0.245368124 0.223365804 0.258159735 0.208901349
0.228747006 0.214773451 0.223609582 0.223984774
0.256167286 0.232410441 0.226779709 0.2117527740.240242416
0.231657069 0.237559713 0.226098624 0.232074256 0.22613006
0.23314186 0.236594507 0.247189329 0.243691732 0.226805843
0.267438102 0.245021596 0.250225892 0.2117168210.239583343
0.232431087 0.22910943 0.250065451 0.235121947 0.249490762
0.233054548 0.203738699 0.2311945580.24607033 0.239447789
0.268389547 0.233776481 0.247670563 0.217124837
0.253972807 0.282481673 0.238953392 0.242015624
0.251645244 0.248626652 0.229364671 0.21893475 0.233101009
0.247134179 0.248542693 0.235594136 0.255358785
0.243921282 0.25325765 0.247830965 0.237530352 0.231150875
0.223304726 0.225243214 0.266050726 0.243916753
0.241976183 0.245676684 0.234800513 0.250842969
0.253891467 0.264577991 0.219786468 0.254869947
0.239818368 0.241510838 0.235068506 0.243832764
0.250378965 0.247851354 0.218439396 0.263773205
0.233662651 0.24880889 0.239762803 0.254608315 0.248853932

0.206571065 0.25886009 0.266620924 0.246812664 0.21934964

0.252257377 0.2757941110.268016155 0.205718969 0.253488726

0.245153107 0.227685846 0.257274682 0.248531574 0.22997087

0.235593878 0.256213297 0.272841918 0.256826348

0.269225191 0.268173296 0.271748039 0.275498593

0.236922503 0.282779133 0.243082367 0.254174955

0.248609788 0.28351998 0.273406887 0.237364859 0.256703867

0.262512338 0.258780317 0.249732589 0.285393314

0.218812203 0.256642468 0.263677622 0.267275651

0.265248449 0.258506446 0.271659695 0.2481178530.278949722

0.237299395 0.263721084 0.244152429 0.229213498

0.245948305 0.252686875 0.242091052 0.2491135830.267122115

0.255420761 0.230517901 0.245149447 0.257581917

0.273241341 0.243322381 0.251702033 0.239955122

0.267563713 0.26025913 0.246407068 0.251758544 0.234075847

0.267488648 0.24348024 0.25240992 0.265920286 0.238353604

0.260436944 0.270423897 0.281963407 0.263049213 0.26800595

0.255481782 0.266229425 0.25480167 0.265049106 0.268854895

0.277019496 0.282902441 0.244851881 0.265470746 0.25620494

0.257753044 0.237049603 0.239413971 0.258610444

0.273268469 0.243309205 0.281665819 0.254709341

0.2849711290.258436751 0.267522049 0.278846131 0.268866986

0.278662582 0.28805504 0.281989414 0.279520225 0.277608058

0.248140713 0.2361170790.272322159 0.228709904 0.269353582

0.2411402290.2118910850.279937774 0.251826284 0.258894396

0.2691110090.256030138 0.280306721 0.266819197 0.262196752

0.263026144 0.271028644 0.248802389 0.247606941

0.249913072 0.228502066 0.230503949 0.248819735

0.285997017 0.194433732 0.233365028 0.230553146

0.254344088 0.258971481 0.264998798 0.255701065

0.234817044 0.233199856 0.247223997 0.24104995 0.250563183

0.23458943 0.256760658 0.230149789 0.251704443 0.220643017

0.254501421 0.221625219 0.262463122 0.226067789

0.256837557 0.232275631 0.25168356 0.252409407 0.239666344
0.278793512 0.215934091 0.2411640140.222642249 0.238626581
0.236596031 0.246796013 0.254220314 0.234668315
0.261268089 0.23819037 0.26040572 0.256650016 0.252427489
0.2706914 0.281386823 0.261856401 0.268016216 0.261138075
0.27589047 0.236008632 0.246241412 0.2514501170.268484704
0.246385631 0.259523771 0.251730524 0.257833497
0.230425615 0.236354804 0.275833476 0.252579845
0.276166981 0.275978623 0.265363995 0.243075665
0.274016321 0.265437121 0.260769682 0.2461179110.252296841
0.254254615 0.271282684 0.252453073 0.253237677
0.216691477 0.244150729 0.255123475 0.252122764
0.233467796 0.26471539 0.2732011 0.235280253 0.237413686
0.254805439 0.225177416 0.252202323 0.258982872
0.276668698 0.269818735 0.2622618 0.25400191 0.272223498
0.268793755 0.239396067 0.254454104 0.229531854
0.232224132 0.272341447 0.263901097 0.249137539
0.246573082 0.279158275 0.255605749 0.251351399
0.246913837 0.284061226 0.235882856 0.253800772
0.243124235

KEGG_CIRCADIAN_RHYTHM_MAMMAL 0.17252347 0.195737258
0.214153513 0.185135226 0.217211 0.161077965 0.213490262
0.218267805 0.182144021 0.252357055 0.163517869
0.223500571 0.168817569 0.224855698 0.239615497 0.15918078
0.172930535 0.223914019 0.227332124 0.234366888
0.143994209 0.249181334 0.201746094 0.146836671
0.225823658 0.163053187 0.177854317 0.183247852
0.218256307 0.247937888 0.055789923 0.180213266
0.159468568 0.207399132 0.190047036 0.1933597 0.159335688
0.215972562 0.140682713 0.174090792 0.229936786
0.283784572 0.219891268 0.1731355110.266734865 0.206801835
0.135319785 0.160014295 0.207173272 0.167241463
0.189876761 0.180014007 0.188955383 0.155054021 0.20534409

0.201279009 0.1198955390.168019285 0.182802452 0.214449849
0.167165089 0.202029933 0.204889357 0.222835204
0.179372524 0.172426045 0.16471339 0.175428558 0.273291245
0.158250866 0.281274537 0.153100581 0.24168576 0.202829948
0.208932445 0.231397243 0.17572982 0.246582087 0.238719675
0.234684865 0.180156924 0.212693906 0.157603085
0.239474729 0.170681204 0.183510607 0.238079675
0.209048235 0.19855145 0.21939298 0.190371937 0.149622154
0.158013464 0.236770087 0.303453307 0.244700146
0.162474504 0.212305365 0.266000896 0.183558774
0.182485066 0.260785051 0.159992228 0.25653905 0.165463643
0.244210712 0.140766565 0.2071116790.160039191 0.230103567
0.166292794 0.221803037 0.158156109 0.234623982
0.193003314 0.227407166 0.19933828 0.150306277 0.243273241
0.1882581140.171716163 0.216446929 0.178483192 0.240049968
0.202057531 0.178514962 0.23625847 0.180321881 0.250179413
0.238029308 0.230678303 0.238244972 0.256268132
0.178894253 0.233887001 0.185833179 0.2436305 0.248246906
0.172427804 0.241895508 0.19023101 0.246778062 0.217956297
0.210496726 0.190688152 0.258413565 0.220946404
0.173838237 0.261039083 0.219595528 0.233658847
0.249954721 0.190588785 0.160128908 0.245536268
0.235318043 0.185672757 0.2115999130.19473077 0.230469987
0.216631564 0.208168799 0.215294951 0.22592154 0.234358389
0.240750534 0.213023359 0.190746992 0.219017483
0.210756278 0.231655434 0.175817699 0.200669547
0.185856282 0.234389512 0.2275461160.207167707 0.239671527
0.172712996 0.274642344 0.205796533 0.136878158
0.239236551 0.262657746 0.24699751 0.142435188 0.184575511
0.242248558 0.199708022 0.281073623 0.182979902
0.239065555 0.232737605 0.218217534 0.286202444
0.234258637 0.229532317 0.239377971 0.238991555 0.27022707
0.259568513 0.263720943 0.294491696 0.19920692 0.225470922

0.167660804 0.135155236 0.233892071 0.231259325
0.200632356 0.26596435 0.191984934 0.231974373 0.202575242
0.224679476 0.299534403 0.212080972 0.222277746
0.253561321 0.236960498 0.169821298 0.207770177 0.19770153
0.299674389 0.207526709 0.195580454 0.216350025
0.227973017 0.051656568 0.334633759 0.231098841
0.177496776 0.192344314 0.179144397 0.248606841
0.234725701 0.223062772 0.18688475 0.253440204 0.267322118
0.153318938 0.283658482 0.155125946 0.19309705 0.258093271
0.178418834 0.184354695 0.175736997 0.139443057
0.210192361 0.252907216 0.244390834 0.215447955
0.231809108 0.124224626 0.192691709 0.162604987
0.1831112780.18393577 0.194740081 0.124490439 0.235871336
0.188079823 0.166222893 0.198695228 0.227701874
0.124030269 0.238801221 0.21305708 0.190956215 0.191953345
0.256671891 0.273530571 0.178869451 0.213290296
0.212864074 0.205103464 0.150120432 0.203884654
0.246833887 0.148826443 0.22932766 0.202357098 0.236340294
0.250248464 0.224372097 0.261686477 0.228123636
0.236796769 0.210920558 0.257478219 0.264562598
0.2113558740.178529997 0.192988877 0.177218946 0.171697543
0.265301265 0.220407184 0.150240344 0.206576544
0.232513137 0.247854641 0.205823078 0.221761807
0.246284893 0.256685522 0.157207392 0.196105093
0.1774280110.241421357 0.1941151640.207334072 0.272379196
0.219214101 0.252722455 0.234217147 0.140159164
0.209847133 0.244695834 0.19175607 0.144843031 0.178355941
0.169565001 0.151872522 0.233257564 0.237225399
0.159358124 0.22439872 0.195447843 0.203889728 0.229279468
0.247714717 0.193864925 0.249181433 0.231791567
0.189943259 0.248146327 0.195419942 0.204296565
0.251613584 0.200337874 0.29463683 0.206846028 0.237736687
0.200320331 0.195341407 0.225998996 0.232553872

0.196177358 0.260231927 0.232303761
KEGG_CITRATE_CYCLE_TCA_CYCLE0.319242646 0.334867644
0.33452002 0.350678541 0.356697183 0.315248821 0.332159076
0.33441007 0.328478796 0.328152731 0.3411444680.342131388
0.299885283 0.342022502 0.335866965 0.360768485
0.314985026 0.335809222 0.327589263 0.306127023
0.335946357 0.321856354 0.378763417 0.355572092
0.356875794 0.345381269 0.347032476 0.3693561120.299362815
0.346042155 0.336796606 0.3501139970.3107081 0.301510606
0.379350222 0.334376454 0.306094242 0.348044179
0.328433459 0.305276996 0.355107426 0.353344686 0.31417226
0.352480677 0.326728258 0.331032304 0.341403468
0.319848635 0.370514855 0.341669454 0.306550792
0.320287386 0.351523274 0.312361936 0.336314603 0.36180613
0.360909151 0.332033254 0.368869165 0.282183683
0.384493131 0.345101656 0.372185226 0.341587042
0.3247113550.381923896 0.344088647 0.360690787 0.355460475
0.356641437 0.348459626 0.328798263 0.328833198 0.32062705
0.336098789 0.326932271 0.32036471 0.331596235 0.315016592
0.341751935 0.384573945 0.368205019 0.322512078
0.340592464 0.29924876 0.358409327 0.322598864 0.30414938
0.330881901 0.315925289 0.380427665 0.350886867
0.345673096 0.333272307 0.361677292 0.339466183
0.338887008 0.341658384 0.350563606 0.339403135
0.346736856 0.34166013 0.344880637 0.3609421190.340345318
0.347606946 0.353614959 0.345587557 0.327629806
0.329094833 0.356383458 0.3032111430.329086457 0.318983727
0.3378890110.3111385960.33499871 0.351793952 0.348277165
0.366894675 0.359181046 0.3587117740.313054461 0.337875261
0.363885743 0.392088444 0.361890054 0.339687996 0.38021982
0.389047913 0.369964526 0.343274044 0.366738681
0.3682119750.341486877 0.340584508 0.323325221 0.36993723
0.3117501850.356037334 0.321627183 0.353907504 0.37855039

0.329721024 0.355390385 0.325891715 0.355762222
0.327249386 0.317059522 0.324501995 0.364694875
0.366606087 0.350166255 0.315882876 0.360833322
0.309590396 0.300738023 0.39742533 0.352030289 0.339408126
0.388492413 0.330285651 0.340708314 0.358884354
0.3494118550.335047331 0.30931307 0.372328926 0.338761014
0.341856868 0.36890337 0.368475127 0.388093891 0.359842643
0.359644942 0.342206558 0.365333835 0.310013686
0.375242397 0.362864742 0.37800352 0.315071654 0.373160141
0.324880657 0.341748481 0.365458727 0.353590994 0.31637958
0.356995808 0.319825086 0.323524184 0.326143059
0.334949312 0.357797153 0.337855381 0.303459603 0.37281267
0.383632359 0.325601744 0.380810408 0.335276502
0.379070229 0.371469152 0.339748355 0.326674135
0.327038907 0.329230389 0.320050956 0.360150144
0.329129147 0.318798328 0.348965607 0.349207531
0.326928852 0.394945828 0.328844324 0.326423953
0.353757712 0.309403064 0.351078304 0.312869174
0.342516173 0.366560127 0.358589353 0.307087495
0.315450369 0.347492717 0.34694194 0.339779017 0.348232561
0.354278743 0.365087715 0.309284174 0.3671144580.308877754
0.339597143 0.363510452 0.352008482 0.383134421
0.344383668 0.35595168 0.315835013 0.34539877 0.331070675
0.355883502 0.334778848 0.334369595 0.382917439
0.369899781 0.350953144 0.368314423 0.310830188
0.321297615 0.360127102 0.375403426 0.307966159
0.323371896 0.327662058 0.362319055 0.372037996
0.373585105 0.338440389 0.3364683 0.326527587 0.355171034
0.327510237 0.39112876 0.337665365 0.345782858 0.330192786
0.375944737 0.376246757 0.284582049 0.338219938
0.352414567 0.335285133 0.325169719 0.352775712
0.346623435 0.33279784 0.361355997 0.344351303 0.341437859
0.35305531 0.367346014 0.333165468 0.327019932 0.374498563

0.340019568	0.34399255	0.34788285	0.329878321	0.386232023
0.32265895	0.348885636	0.360319251	0.327716397	0.345319044
0.342524966	0.387302231	0.342352008	0.358881053
0.325556984	0.335888543	0.383570392	0.319777637
0.330198036	0.369748138	0.362344099	0.334375124
0.348747429	0.345009999	0.33628195	0.377090014	0.297894523
0.293523557	0.345098301	0.383666012	0.36677192	0.287771497
0.361408921	0.350378197	0.307679192	0.321626201
0.352924934	0.379858175	0.355074791	0.350902107
0.383412197	0.353227769	0.369248517	0.334435704
0.317414286	0.329806255	0.33220262	0.365957766	0.362523746
0.354760155	0.264740159	0.386882921	0.310476665
0.3301166130.349946148	0.304462478	0.327264958	0.34857674
0.352021905	0.334865494	0.333391514	0.292386426
0.356333541	0.333893791
KEGG_COLORECTAL_CANCER	0.214231376	0.223719283
0.2156390110.19152987	0.201698272	0.202603894	0.216304763
0.208302922	0.232570536	0.214943137	0.199772856
0.216739397	0.2518266	0.230382607	0.227393175	0.207034375
0.19746397	0.2100871180.208464902	0.224638084	0.226526439
0.221888287	0.197207579	0.219173242	0.236971359
0.248109657	0.22532182	0.236041724	0.256549037	0.233854814
0.203743848	0.217452981	0.225585434	0.228955359
0.245552028	0.196839292	0.222225762	0.179959364
0.198983953	0.19672987	0.234553916	0.221706518	0.196157791
0.202367086	0.218509138	0.18641523	0.2251073110.196676235
0.203050281	0.20922849	0.217712596	0.20157914	0.204203301
0.223524098	0.2020116350.23988856	0.21963289	0.223267132
0.158337104	0.22131014	0.194906086	0.20627607	0.2267496
0.198469313	0.21774015	0.19422711	0.175308073	0.217458203
0.2064116650.221574198	0.236981292	0.217857632	0.216347814
0.208978546	0.217126691	0.242064198	0.207015125
0.229426443	0.222610824	0.217068815	0.206986824

0.201421799 0.222466773 0.227175607 0.202357352

0.214416531 0.234228024 0.235407195 0.231378452

0.248217602 0.208091463 0.219129643 0.2118000010.206386329

0.212167687 0.232932694 0.207919474 0.223726464

0.190806921 0.204769351 0.226021979 0.2401172220.186440082

0.231347463 0.200868599 0.223970009 0.220855564

0.206700853 0.218107627 0.2143938 0.179915204 0.220074021

0.205682434 0.214132286 0.197265531 0.230636084

0.218596268 0.194673182 0.220516153 0.224088964

0.223534656 0.212051713 0.224022365 0.251873808

0.226847526 0.181371096 0.223979738 0.224848019

0.192621206 0.230314137 0.21960671 0.208186105 0.218726316

0.217804906 0.232960139 0.240428499 0.231944878 0.21933441

0.256177951 0.236021035 0.1988201140.25265283 0.211020505

0.230518924 0.212460087 0.246675771 0.23180533 0.203789972

0.229214198 0.216847867 0.22152348 0.229073387 0.243699139

0.213327205 0.229927558 0.241260259 0.234837008

0.251548291 0.216493224 0.2409114210.206221399 0.219526846

0.22070145 0.238971756 0.228332173 0.2061126460.251397309

0.225073055 0.209225538 0.244462707 0.226998366

0.224618817 0.201493159 0.188058536 0.217950989

0.233342667 0.224539267 0.2211358560.209364825 0.225219416

0.209137453 0.206920928 0.225299059 0.217369649 0.22013567

0.192820364 0.235469387 0.242465164 0.22638606 0.243568113

0.237721854 0.235362835 0.227154608 0.221443328

0.223307477 0.242720646 0.208680494 0.223928266

0.252703773 0.23950718 0.243232551 0.206134196 0.236297838

0.199901271 0.227216909 0.226276306 0.224937449

0.225791915 0.22360625 0.230579728 0.235336747 0.205294051

0.2269118740.230569424 0.233209032 0.247040064 0.233295995

0.2471170380.2552811930.232467618 0.244527327 0.230833621

0.221356717 0.215889096 0.247430147 0.212308401

0.250713175 0.215085578 0.203620354 0.256439675

0.237403337 0.246138297 0.239738589 0.213145701
0.243056252 0.241800191 0.244267675 0.250318369
0.231499821 0.226495695 0.213998647 0.212629282
0.216505241 0.2051110620.217945428 0.249762541 0.193580134
0.232591524 0.207755529 0.238330717 0.238248795
0.218222667 0.217098696 0.19337163 0.209633197 0.223808065
0.217998716 0.236441647 0.216661234 0.23790458 0.216455633
0.238040534 0.200379074 0.229791957 0.207759046
0.219990258 0.195496777 0.212505597 0.221739884
0.224887058 0.219520319 0.195785003 0.2539818 0.190854836
0.213381239 0.217041888 0.206847735 0.225530257
0.222459361 0.222826352 0.210233217 0.245782905
0.220542033 0.22239479 0.227299202 0.210654926 0.242304087
0.239505492 0.241487227 0.246143084 0.229924389
0.261350148 0.204537914 0.223426188 0.232178683
0.226507254 0.25057089 0.236884065 0.226530529 0.22440671
0.207618825 0.207988321 0.246204207 0.202409195 0.22328237
0.229681467 0.237095208 0.233016686 0.229007641 0.25407629
0.24413729 0.213308737 0.220370363 0.201377681 0.237171917
0.246645823 0.207913644 0.210926944 0.212171904
0.232519548 0.226155552 0.210293155 0.231325335
0.255516524 0.224183381 0.221876414 0.217160935 0.20430722
0.217728876 0.230638201 0.25728562 0.237334282 0.236227797
0.221513327 0.236652279 0.250601483 0.213060962
0.210575539 0.22604585 0.192151417 0.234832886 0.241401478
0.2110876830.246520226 0.22314877 0.203097141 0.235096061
0.224551827 0.24533616 0.228778076 0.207817089 0.212289046
KEGG_COMPLEMENT_AND_COAGULATION_CASCADES0.071343686
0.161548282 0.108502054 0.090560668 0.147923665 -
0.015455919 -0.029562376 0.046729666 0.0734207110.085032791 -
0.027089992 0.084956546 -0.03669468 0.053806203 0.082865416
0.094476438 0.079582154 0.136100086 0.101460837 -
0.048936729 -0.094514514 0.182171867 0.11946141 0.003063278

0.108242678 0.089782026 0.1189375190.100136795 0.073582061
0.094601038 -0.055677562 0.14922084 0.1139259710.043763994
0.081808414 0.146954054 -0.003484766 0.037034505
0.163807075 -0.098000612 0.1968101160.091290042 0.106242395
0.169715816 0.076793646 0.078031208 -0.030735319
0.0711042720.145236207 0.1148598190.182585292 0.124511129
0.07285797 -0.057933151 0.120454075 0.123450177 0.143701077
0.143649204 0.137140894 -0.022379182 0.109206306
0.136519771 0.063756078 0.203373554 0.1711738390.044983583
0.15602001 0.213832981 0.145385204 0.1150667240.17309553 -
0.06882524 0.098370745 0.041679587 0.1211050270.126583384
0.054801903 0.10759216 0.1211684170.074368482 0.121821848
0.055067657 0.004040681 0.145019243 0.104636286
0.043652979 0.102728205 0.133998393 0.041898521
0.082640528 0.076603856 0.091089075 0.0486112980.113583793
0.186918162 0.058440796 0.1117772910.136991724 0.202657101
0.1145425360.13186521 0.0501411830.092929089 0.132184069
0.030277971 0.162473642 -0.001616795 0.147493864
0.1574111670.141384196 0.091769571 0.041136962-0.018351016
0.133815432 0.001264655 0.139508142 0.127508446
0.140994764 0.107376258 0.140277848 0.040876409
0.040407223 0.190135106 0.053594058 0.140127252
0.145214404 0.098215868 0.07393528 0.069227552 0.058647529
0.104941569 0.1160543190.082616605 0.144353743 0.106759166
0.063267194 0.151024689 0.172565307 0.066404597
0.204497387 0.0231176090.073340961 0.1151243210.059121589
0.063507657 0.160389274 0.135701864 0.062147938
0.0522377110.075459306 0.198842255 -0.019302891 0.103068597
-0.009196904 0.102504268 0.048090919 0.086723585
0.172649731 -0.013909841 0.10530673 0.11621547 0.022715019
0.042403786 0.161431077 0.177335953 0.146926879 -
0.022762787 0.101054969 0.165650905 0.084721308 0.157812329
0.060088315 0.067056039 0.104128974 0.134893824

0.135097505	0.036304586	0.138190584	0.153753763	0.07579234
0.170480415	0.048155454	0.139005054	0.1189304880.110921352-
0.019953935	0.097926045	0.155841553	0.049679085	0.111879932
0.14274949 0.174579667	0.127657152	0.122029683	0.175977827
0.181510002	0.1119565880.14403824 0.086755557	0.163465556
0.229449777	0.147576983	0.108789772	0.076940355
0.159148406	-0.052766312	-0.058429872	0.069703141
0.191456717	0.120589431	0.082101763	0.0581154810.148749422
0.062203392	0.149302388	0.146549412	0.158648572
0.136536716	0.177710403	0.079549719	0.083150626
0.148312596	0.137601072	0.029190045	0.119890431-0.092506844
0.127906417	0.16908568 -0.050564665	0.186818168	0.118412175
0.136826379	-0.004307499	0.049956642	0.1142447660.09288767
0.072530294	0.136403413	0.158152628	0.1170815750.182901589
0.14934849 0.06765549 0.098785739	0.173424345	0.132351166
0.121508653	0.0119636820.071503182	0.169341567	0.140248536
0.138555736	0.079025944	0.038599338	0.0471141830.166329862
0.1110979250.125597036	0.091981959	0.144543747	-0.01719117
0.136778293	0.1571192580.101206288	0.099415808	0.108046572
0.10082097 0.092890929	0.068462326	-0.010822712	0.211878032
0.06267853 0.120916326	0.049726786	0.141021077	0.000452979
0.130467058	0.193225542	0.012387469	0.094815828
0.022370562	0.062374345	0.123328199	0.097947649
0.169240339	0.126487656	0.188063065	0.185846192
0.180272147	0.156872905	0.162329778	0.143585181
0.129943196	-0.024545796	0.093416065	0.105043524	-
0.038191292	0.18019907 0.209261987	-0.015180833	0.121418407
0.167008331	0.142856371	0.052368476	0.160777788
0.127237636	0.168187196	0.053342879	0.149789514
0.096244759	0.158187262	0.179871708	0.1221069	-0.047933666
0.048241327	0.123025474	0.163970284	0.095748396
0.044186076	0.093052904	0.120607334	0.067631909
0.087466855	-0.047233835	0.148299368	0.044258923

0.1163465510.09383635 0.107404853 0.147476292 0.042111573
0.121733053 0.078493598 0.155079085 0.205193045
0.151262175 0.093883476 0.174596643 0.008062853 -
0.056599572 0.208864383 -0.033632695 0.108038437 0.09288226
0.141084555 0.14075865 0.085333559 0.03547303 0.123616104 -
0.084296047 0.099165399 -0.026167291
KEGG_CYSTEINE_AND_METHIONINE_METABOLISM 0.191150198
0.188776297 0.205460003 0.206516722 0.206704468
0.202424734 0.187266335 0.1891180710.203132427 0.202942912
0.208545139 0.219665162 0.194540133 0.201691437
0.202942958 0.205335177 0.197957271 0.195209814
0.205976034 0.172930937 0.225445927 0.193264486
0.199920004 0.192786144 0.195627125 0.218842338
0.217038979 0.214094917 0.216222235 0.200246692
0.194832057 0.214687183 0.19709308 0.183339052 0.206542349
0.203878924 0.199029935 0.208869838 0.202270263
0.219183869 0.188103827 0.1924811350.210121415 0.203816074
0.194260916 0.195148224 0.202046268 0.177500407 0.21179619
0.216459293 0.200141674 0.22431966 0.215678996 0.234615012
0.2133375 0.184092676 0.217692542 0.213555896 0.217084344
0.196522619 0.215292819 0.193850717 0.215394459
0.192402353 0.213028978 0.22350293 0.206845018 0.208320138
0.202506854 0.193334186 0.196766001 0.243517892
0.174726347 0.184325173 0.217757713 0.208136804
0.196357905 0.207557703 0.161266963 0.2050117430.221069464
0.200140076 0.216845437 0.222326881 0.207808514
0.215096189 0.1921112980.194187845 0.2118343050.192573794
0.227444592 0.227804265 0.232276714 0.2124115060.209792205
0.219617716 0.224658304 0.218079453 0.190061901
0.216601053 0.212926294 0.195882867 0.207124968
0.180545155 0.210561469 0.203745985 0.229562362
0.200328147 0.218863853 0.205034358 0.225950855
0.203098147 0.194780188 0.199553736 0.194527368

0.2115365450.227628386 0.197217322 0.188426552 0.208963867
0.221087765 0.204343833 0.207276821 0.200667308
0.221075418 0.2011327730.218426182 0.209751398 0.183592858
0.199964968 0.207503508 0.200055181 0.191570624 0.20663352
0.198141249 0.200187702 0.191596524 0.194853847
0.218392763 0.187422367 0.210392448 0.192653979
0.214674604 0.1778362110.225463492 0.197310682 0.198791352
0.217279171 0.191566473 0.222754426 0.227647425
0.212317068 0.226700241 0.200972396 0.188128981 0.21854425
0.198837055 0.218619683 0.22600538 0.1983054 0.19265283
0.23278853 0.207631739 0.1999341160.204923668 0.193690118
0.195830736 0.224982785 0.203063796 0.2114123530.224732117
0.235830219 0.180528899 0.225322382 0.230968714
0.198404026 0.233826514 0.204442244 0.215492066
0.200295806 0.200143817 0.206592221 0.214425241
0.204083838 0.225533101 0.202701916 0.180177093
0.1911988840.188671033 0.185732183 0.193106999 0.210192355
0.209497129 0.207103555 0.183582587 0.190259413 0.2097798
0.206067459 0.213017694 0.226266465 0.198813035
0.198549069 0.195884875 0.226559331 0.190870627
0.203063258 0.197409474 0.184332078 0.188594418
0.227951562 0.189713139 0.2114335540.217656133 0.198275114
0.205025509 0.178009195 0.180727578 0.206157478
0.192319404 0.213806994 0.20247071 0.202650082 0.196565303
0.195641579 0.188379887 0.192612887 0.195168805
0.200067858 0.258950912 0.179474656 0.208085099
0.197462262 0.231355914 0.226105569 0.198946847
0.214298525 0.221934236 0.214852677 0.191634647 0.20195082
0.214932877 0.183881357 0.224503422 0.213531016
0.194846573 0.212522893 0.188366679 0.237185466
0.232345663 0.216989434 0.191071939 0.196671021
0.197751812 0.237675392 0.228992255 0.195891457
0.207920045 0.2019408 0.204743887 0.208509263 0.207181781

0.181808556 0.210459625 0.210932524 0.216474267
0.208676884 0.209679992 0.209138044 0.208380199 0.20935894
0.205157259 0.186638086 0.203193643 0.208168309
0.225679569 0.193414492 0.188327874 0.218096786 0.18112972
0.207558939 0.236387953 0.194026465 0.180689402
0.213539426 0.20386481 0.191076625 0.190335802 0.207414967
0.182726379 0.206705985 0.194168815 0.192797653
0.213585961 0.223035614 0.209229969 0.20355415 0.203452441
0.185765176 0.201671217 0.20746562 0.222581219 0.208924006
0.207328068 0.198675464 0.222487828 0.189995638
0.206975527 0.238062529 0.22406457 0.206532633 0.194027514
0.194552335 0.220467919 0.2118595960.217321856 0.193259411
0.195993844 0.208302748 0.204596922 0.179937862
0.204357054 0.225813159 0.225755876 0.18015507 0.213249789
0.220173128 0.203542325 0.227349655 0.212910506
0.213765438 0.22170444 0.198936698 0.225749713 0.197642306
0.190260405 0.196054661 0.212562128 0.216174678
0.162227931 0.232385717 0.207366221 0.2119217810.196131534
0.180849788 0.194397462 0.212918941 0.209056291 0.2111976
0.205678838 0.180909528 0.207759874 0.245044739
KEGG_CYTOKINE_CYTOKINE_RECEPTOR_INTERACTION -0.081094223
-0.051878986 0.017909327 -0.062478739 0.0115138 -0.112123664
-0.119667144 0.004771221 -0.061213092 -0.040609265 -
0.160759074 -0.00184853 -0.141793187 -0.103270384 0.017760681 -
0.05475909 -0.090571709 -0.06458937 -0.057726713 -0.11128341 -
0.1847119210.003306668 -0.010819099 -0.108258873 0.011943247-
0.038429763 -0.033177948 -0.067275706 -0.079384973 -0.047971991
-0.102806582 -0.033713328 -0.085222444 -0.103261495 -
0.053908302 -0.040647176 -0.0938016 -0.064151101 -0.018101307 -
0.131051683 -0.031091576 -0.067457365 -0.043457333 -0.051112305
-0.05186247 -0.080174537 -0.054995778 -0.065758297 -0.014117379
-0.066157867 0.059240337 -0.018667136 -0.020647668 -
0.176154329 0.010521113-0.000811843 0.015189582 0.011101681-

0.013552634 -0.135813369 -0.078102586 -0.025173663 -0.027435974
0.02950949 -0.07216154 -0.103304215 -0.057068157 0.03137645 -
0.05010828 -0.034418664 -0.034631406 -0.125154049 -0.036207423 -
0.135719675 -0.038318196 0.059959173 -0.067872439 -0.004511756
-0.044757836 -0.061344009 -0.014110006 -0.095037022 -
0.115960137-0.055792543 0.011185813-0.0440667 0.02513429 0.033395676
-0.077845669 -0.067240739 -0.028644084 0.039682749 -
0.034448448 -0.063793179 -0.038866858 -0.030344933 -0.030378893
-0.046438027 0.01269208 0.068256473 -0.02848345 -0.098584237 -
0.090133457 -0.026632863 -0.088492436 -0.024741443 -0.100837712
0.010929019 0.011892244-0.005657032 -0.076014438 -0.006965033
-0.151604363 -0.092760298 -0.111860203 0.06244542 -0.019128611
-0.020594527 0.004000583 -0.059580623 -0.021285786 -
0.086973009 -0.045366574 -0.018119261 -0.055196353 -0.059935019
-0.065334299 -0.067105111 -0.094013116 -0.021149179 -
0.055449564 -0.06576503 -0.060794329 0.006562359 -0.043594732 -
0.128579728 -0.009224648 -0.035135813 -0.053554668 -0.020313405
-0.103540945 -0.031126712 0.016532549 -0.114011051 -
0.06607898 0.0301828110.001730672 -0.037080975 -0.096213631 -
0.033385148 -0.006025692 -0.057472405 -0.092983971 -0.132922988
-0.028909648 -0.030708542 0.002648579 0.013974463 -
0.092660887 0.027017729 -0.086169647 -0.07906717 -0.105131089
0.009915414 0.03231684 -0.06637548 -0.114305122 -0.062725546 -
0.048023841 -0.063790581 -0.002682128 0.007457657 -0.121875764
-0.032101803 0.008252341 0.030476789 -0.030140784 -
0.057714944 0.014373871 -0.052228174 0.003977129 -0.123039993
-0.022167917 -0.023297791 -0.003558013 -0.115044095 -
0.0670721120.027176137 -0.12256083 -0.045963978 -0.017080065 -
0.014344251 0.007678752 0.008692738 0.012636295 -0.050980526
0.027170498 -0.009218973 0.00595671 -0.010585473 0.02853288 -
0.103352897 -0.047617382 -0.064527695 -0.027184312 -0.140303862
-0.054183097 -0.066309899 -0.041075109 -0.07623404 -0.022957716
-0.076642553 -0.022973949 -0.075964219 -0.014572708 -

0.012671053 -0.080941133 0.061613378 0.054985322 -0.062042906 -0.025413173 0.038233283 -0.069993385 -0.093526213 -0.003667618 -0.13281013 -0.042473261 -0.041965847 -0.049287111 -0.004105746 -0.019003039 -0.043735767 -0.015851994 -0.01658964 -0.004449798 -0.024776587 -0.105215448 0.031037682 -0.047263489 -0.026809645 -0.003457095 0.026002671 -0.089148459 -0.026067667 -0.027773006 0.014122823 -0.063261119 -0.124338412 -0.010899982 -0.05384199 -0.049100162 0.040946948 0.011893164 -0.046416262 -0.093902149 -0.061472584 0.00431441 0.006460265 -0.017445727 -0.041835777 -0.156052127 -0.034684155 0.016175549 -0.018686662 0.021688557 -0.024424893 -0.004926975 0.020156258 -0.116325939 -0.06121869 0.014828057 0.005328 -0.066520075 -0.028897322 -0.027348947 -0.133979119 0.010631888 0.013903937 -0.092639688 -0.042492641 -0.026561487 -0.028198066 -0.044966013 -0.034922715 -0.010783602 -0.040416084 -0.009478402 0.028977311 0.085109141 0.024504075 -0.013242892 -0.002857378 -0.099233832 -0.119442739 0.000738121 0.042380395 -0.085333528 0.027384521 -0.011854814 -0.087655957 -0.070699393 -0.074138276 0.004028705 -0.073893606 -0.107103834 -0.021772245 -0.080740447 -0.065634713 -0.025656486 -0.008566914 -0.014826092 -0.033218919 -0.054447434 -0.037563088 -0.098538635 -0.035141583 -0.059165159 -0.040242768 -0.049378852 -0.061597231 0.009933982 -0.050973739 -0.067142872 -0.100122897 0.036151802 -0.052274629 0.005824918 -0.00954665 -0.062757444 0.047073006 -0.044197714 -0.009903658 0.002599774 0.040196374 0.017423722 -0.041248329 -0.029466571 0.052593196 -0.116801713 -0.129616492 0.034914185 -0.129329856 -0.066603047 -0.084162233 0.003928141 -0.040529781 -0.086463407 -0.032092421 0.007123463 -0.135841556 -0.092516203 -0.145118961

KEGG_CYTOSOLIC_DNA_SENSING_PATHWAY 0.055870886 0.085520105 0.167552197 0.089602768 0.150418869 0.031763402 0.019725611 0.111763703 0.109267758 0.124761732

0.012020266 0.156887722 -0.017108494 0.0595064 0.108356254

0.101803709 0.017506247 0.10155816 0.120073506 0.046302905

-0.022933993 0.180225881 0.147694084 0.036847427

0.127144343 0.1181862120.1123395790.1115713970.066265008

0.104182886 0.023688808 0.123629579 0.049816032

0.065522646 0.048082254 0.144740944 0.067575861

0.150189378 0.128289542 0.07991893 0.1108110860.08984101

0.102466733 0.1116009930.108207146 0.1124908860.103551298

0.0594421120.133293883 0.098449124 0.196039879 0.178220946

0.132444983 0.004427333 0.162678002 0.1048772110.161692441

0.165806219 0.157750958 0.004181033 0.090564842

0.1411220250.106162184 0.139868817 0.097805865 0.060317384

0.138273289 0.169999066 0.092332551 0.104672483

0.1195245420.028635389 0.1211735450.049638967 0.14328338

0.16375777 0.077899755 0.1179804110.062030632 0.07030814

0.158791289 0.06619201 0.077626363 0.089024852 0.185177848

0.107985271 0.173077718 0.1738107110.043229367 0.060294675

0.1135676490.180068526 0.11508544 0.096513793 0.119644425

0.124822419 0.126761259 0.0735586110.173658714 0.199593916

0.105542773 0.052675691 0.068512397 0.127082994

0.040251044 0.1139639020.068175626 0.149669809 0.153893668

0.139000865 0.1114529710.118130017-0.007115802 0.084558606

0.044774469 0.180295706 0.1132535290.103607256 0.14816795

0.108807494 0.14365535 0.074139094 0.10083633 0.091982507

0.138629129 0.123139002 0.089994266 0.0796117570.087615198

0.160307978 0.094166942 0.1106995810.135973197 0.148530746

0.098285278 0.020735401 0.165333456 0.124194832

0.073403548 0.10612693 0.049537926 0.089184794 0.159832031

-0.000599798 0.124946206 0.154720531 0.123831753 0.15299273

0.051300613 0.102842167 0.137714391 0.109273426

0.060927596 0.04412504 0.102093413 0.132880019 0.110293099

0.166036638 0.09262818 0.172042705 0.0808119020.051810062

0.070534428 0.135150482 0.155656579 0.126071777

0.018986845 0.087179581 0.1143347480.065173875 0.142406035
0.1451711830.022543006 0.150457948 0.134548523 0.166200011
0.098448655 0.096595973 0.169143722 0.099247579
0.177274209 0.028673495 0.135451815 0.1196742430.151701807
0.022273755 0.099567708 0.1122769390.007743891 0.072200937
0.070256036 0.147158942 0.1115813770.182990281 0.153171667
0.064081558 0.147506697 0.128567294 0.1481157190.125368893
0.124645121 0.051664617 0.1123105590.1166433160.147383757 -
0.014646015 0.096406104 0.099317058 0.108839792 0.080180508
0.1171351390.085408697 0.128236891 0.109775251 0.101102792
0.078682459 0.062650356 0.164741696 0.184283321 0.05848185
0.1101577590.176658367 0.06773565 0.043948906 0.138763943
0.006957415 0.097786957 0.136530882 0.090034995
0.120539982 0.13242509 0.124968368 0.098070759 0.165042489
0.074977843 0.1140315470.054078924 0.150191305 0.124480008
0.12217728 0.179549233 0.1130936570.064693073 0.111368576
0.0911827580.158425725 0.102576263 0.061395521 0.144950978
0.092614607 0.074446024 0.164659242 0.143761053
0.098442122 0.031295141 0.09776107 0.126365425 0.139177121
0.131686174 0.099003727 -0.014006357 0.108270795
0.139008447 0.1171642610.1791471140.125239252 0.158024505
0.1629271190.034205122 0.073209281 0.164698419 0.136545674
0.044325619 0.088325637 0.1148140410.016239803 0.144700862
0.164405877 0.033768921 0.124256288 0.12080397 0.078940973
0.083098984 0.07867226 0.123144528 0.135672132 0.133519161
0.177377022 0.174559229 0.1170936580.11877449 0.134365992
0.1144744460.031088196 0.12517486 0.150743963 0.017184941
0.128821371 0.13567587 0.043131872 0.0921153590.096187373
0.132702727 0.108677561 0.073392315 0.1130210470.08918792
0.105722226 0.127627171 0.1129511860.131376164 0.123744885
0.064958681 0.06812827 0.021575842 0.066037774 0.075478773
0.109047283 0.089752505 0.080960789 0.102387689
0.136353897 0.091227798 0.01946052 0.174830997 0.082008025

0.1681149680.131513674 0.092132524 0.15500673 0.082938081
0.0799811040.106084774 0.137343994 0.144389206 0.11888695
0.1101114880.127327521 0.031668706 0.057463338 0.143954223
0.019029274 0.075922843 0.040219952 0.165649598
0.1139145680.067472688 0.086521899 0.131922635 -0.001365384
0.0503644110.031912167
KEGG_DILATED_CARDIOMYOPATHY 0.045876843 0.068159016
0.043163221 0.041578534 0.073427882 -0.026759862 -
0.004567089 0.054754025 0.014205399 0.070602849 -0.001892447
0.037440803 0.037363837 0.02744758 0.082966448 0.035641651
0.019713587 -0.012823371 0.015045182 0.011248341-0.023719187
0.005661798 0.029194956 0.013787174 0.060409257
0.028516383 0.032682019 0.02364452 0.005255329 0.02780265
0.003814907 0.015737244 0.014944857 0.018599407
0.074440141 0.017297154 0.013167783 0.006660753
0.038395569 -0.040200112 0.029675173 0.014883022
0.059477318 0.005370015 0.012848898 0.012326434
0.046698097 0.0301194480.033606618 -0.010716483 0.063702285
0.010819546 0.031267483 0.003474417 0.0163991140.082143471
0.020808214 0.017783907 0.030650958 0.034091553
0.003777454 0.021627007 0.050778628 0.067965237
0.025411605-0.017446003 -0.012771243 0.044862918 0.086382908
0.041284327 0.046639523 0.02011587 0.05357396 0.018628309
0.028258633 0.063705041 0.026400142 0.063093425
0.069401064 0.052448093 0.038781345 0.012830065 -
0.017971983 0.028962037 0.023552236 0.045637241 0.03080958
0.009481458 0.029442044 0.02994643 0.038458639 0.027563333
0.056111447-0.015980754 0.053663927 0.025866836 0.013466632
0.050191819 0.010518438 0.034880858 0.023260684
0.003582138 -0.003779788 0.020439137 0.016953409
0.060436035 -0.00360859 0.075466275 0.041983668 0.069885802
-0.017761037 0.06280585 0.008133137 0.022213389 0.038876368
0.075392591 0.032608555 0.032239639 0.045233284

0.016760094 -0.018857244 0.031294926 0.0211282890.048644428
0.0544879 -0.013894087 0.047931729 0.035781203 0.007945874
0.012266501 0.031919301 0.023761838 0.014983553
0.058589736 0.045972514 0.0115629390.043094664 0.057578794
0.037238758 0.055933398 0.022866383 0.057824727
0.043989064 0.001844307 -0.002966143 0.061410068
0.0891103940.012753828 0.0091611730.056752289 0.025284048
0.025330891 -0.011452918 -0.00199598 0.072048639 0.032476679
0.034722958 0.046912471 0.045189157 0.043998507
0.031357477 0.045200825 -0.001906931 0.072578564
0.095041644 0.002789382 0.025039797 0.025849858
0.024799873 0.035494985 0.023904955 0.04395496 0.024584548
0.042887004 0.063456513 0.057275735 0.042753223
0.0709344110.040649725 0.035991925 0.061888025 0.000442934
0.025485946 0.054916168 0.041737048 0.023806832
0.024566245 0.064923725 0.008185819 0.036306237
0.081366594 0.032657066 0.08634379 0.031047034 0.069992366
0.049769101 0.067672582 0.047401315 0.071634049
0.0588085110.11043261 0.027773792 0.055937659 0.026403431
0.040139665 -0.006556552 0.039798616 0.0591107960.055386977
0.0009111350.051041502 0.0336114980.055578502 0.021687303
0.054192251 0.066757004 0.035900894 0.083896624 0.1134223
0.025191427 0.043128322 0.065644505 0.03426258 0.042717643
0.076202709 0.002491444 0.041389319 0.009617302
0.008415188 0.079333226 0.045410491 0.038077964
0.023373424 0.032071559 0.096792615 0.049491979
0.010645169 0.060530796 0.045108068 0.0170638 0.049742559
0.04356867 -0.000732066 0.046141704 0.054532807 0.066136234
0.014262081 0.0029397 0.004725313 0.021425922 0.049009998
0.068684793 0.073952602 -0.006882205 0.034986582
0.010914963 0.054860695 0.048089289 0.025989084
0.080144618 -0.013391904 0.027248635 0.035219766
0.024533037 0.033375086 0.023999723 0.014568553

0.036482858 0.025190705 0.009607501 0.024356728
0.043026216 0.052956858 0.05615156 0.019384191 0.008624246
0.044982309 0.023824253 0.043799768 0.036849815
0.028929806 0.037750973 0.046097197 0.046098706
0.044201351 0.003564665 0.051876585 0.0585119580.075408894
0.090456925 0.055707893 0.039574396 -0.001504495
0.001565761 0.069707093 0.084502033 0.013669543
0.075590846 -6.44E-05 0.02197999 0.056737529 0.026719708
0.080056965 0.006861612 0.035158684 0.038155358
0.022749746 0.056718497 0.022789223 0.059767139
0.056138661 0.048062768 0.034368535 0.049641503
0.075589544 0.074171583 0.034622138 0.018659939 0.04025655
0.018646243 0.078516609 -0.018502231 0.017684409
0.031633327 0.045077085 0.037208496 0.025330752
0.039806962 0.020749021 0.065956882 0.012496207
0.063519267 0.050198822 0.050480997 0.1162140540.046823205
0.057802868 0.067947 0.050577046 -0.025803543 0.111534612
0.038366106 0.04444879 0.036373494 0.044520714 0.055940625
0.016968976 0.044894179 0.046942718 0.015313831 0.02436308
0.008751648
KEGG_DNA_REPLICATION 0.316238771 0.241063543 0.264205908
0.291307662 0.25328649 0.303829008 0.294986791 0.269962607
0.293835701 0.286810874 0.322087424 0.348200041
0.293890865 0.259250005 0.3119464360.345690548 0.277790732
0.2411616320.323386224 0.277884994 0.33327355 0.238775008
0.28025271 0.324692281 0.315307985 0.328544243 0.35602913
0.28812933 0.314201268 0.219725875 0.326694641 0.317986836
0.330750707 0.271826001 0.293371376 0.303609514
0.330874609 0.264074945 0.33305186 0.31033546 0.274704192
0.26370809 0.280227023 0.337536084 0.231465557 0.289573267
0.297073439 0.21499097 0.265400362 0.333303044 0.241018873
0.281784763 0.338702349 0.275746275 0.334267847
0.261215917 0.317236999 0.254206705 0.320158183

0.274363495 0.320037584 0.323021852 0.316675893
0.294039204 0.357367325 0.312648945 0.284101099
0.317984065 0.2496581170.357087978 0.293263175 0.338351412
0.251816736 0.276842703 0.281684524 0.240758585
0.270751768 0.302052504 0.220192078 0.297874309
0.314877131 0.338696006 0.353534519 0.303210198 0.27577867
0.339582962 0.277051736 0.274280289 0.275799405
0.278684839 0.337712526 0.289303841 0.3028114660.304746898
0.2111581470.305070795 0.303173903 0.303133801 0.277096396
0.299165233 0.335897855 0.31826695 0.299416886 0.229265407
0.315696651 0.27877328 0.300685121 0.304306302 0.314868209
0.268287172 0.327651628 0.327345548 0.366216058
0.291038643 0.288498648 0.269863903 0.3111949520.246484909
0.2780141150.291886253 0.280057366 0.342578844 0.250350821
0.282799056 0.331477043 0.298929259 0.275448499
0.264896224 0.314595307 0.240792349 0.28257806 0.2428827
0.277666164 0.295715616 0.295314808 0.288518371
0.221601795 0.244213783 0.296585433 0.2711037250.2704376
0.245344088 0.359294639 0.305545515 0.303423519
0.253746768 0.286367468 0.274815446 0.235890808
0.324745955 0.319457085 0.286932978 0.34211726 0.287450297
0.23149825 0.31169308 0.277148157 0.322345189 0.306735441
0.243599991 0.319875494 0.26992925 0.261474333 0.268272872
0.293987008 0.254690816 0.31303513 0.340617443 0.2383467
0.308235894 0.303803143 0.321648432 0.268469594
0.304929391 0.340787672 0.276220912 0.287505021 0.26942824
0.305386698 0.274379289 0.226351917 0.337734146
0.287957319 0.259329618 0.29739584 0.344447255 0.308901792
0.277242051 0.310979974 0.2228511240.287646082 0.318511044
0.307437405 0.263673105 0.233345654 0.238295238
0.289507487 0.252204197 0.225710958 0.301540901
0.2311951090.273032187 0.243158406 0.28744527 0.228406918
0.296671034 0.310070547 0.260860019 0.217417416

0.264432191	0.275300083	0.242294539	0.276879188
0.283733467	0.28599235	0.185530545	0.259671404	0.259868475
0.201289239	0.297886524	0.28588144	0.270602817	0.25522113
0.245829809	0.199914317	0.274531435	0.292223008
0.229086762	0.341782157	0.199781726	0.326831758
0.249667598	0.312057885	0.305342044	0.2565159110.267488678
0.286191008	0.284421739	0.267333935	0.253768934
0.326132625	0.22027051	0.362033407	0.2617991170.244728416
0.296462166	0.273572529	0.355094566	0.327745532
0.289881447	0.30843135	0.225530485	0.260394139	0.304134061
0.364895288	0.242441369	0.267926575	0.266578072
0.367476159	0.312681232	0.333163647	0.270978377
0.316889102	0.278656689	0.339381051	0.236795104
0.306519885	0.288845031	0.244812957	0.282089453
0.295043206	0.282703578	0.278721859	0.313469736
0.3119095120.283076322	0.270748047	0.350789354	0.261977645
0.235334607	0.316957204	0.27532825	0.258268362	0.29213832
0.287356918	0.287801879	0.268231202	0.280795048
0.302718774	0.252549563	0.239074043	0.21277311	0.285514052
0.2755394110.326907753	0.291376517	0.310782343	0.237064009
0.291097404	0.340807204	0.289348938	0.243747545
0.250218604	0.25655652	0.2711151410.236942078	0.277106046
0.334879913	0.305405535	0.219804034	0.215690796
0.230614199	0.34364481	0.325272242	0.306796861	0.197575362
0.254656966	0.331708731	0.271567171	0.261646369	0.32781823
0.270636429	0.32712146	0.327082249	0.294452676	0.278726047
0.254012628	0.283290173	0.257460875	0.310824978
0.320714029	0.292886462	0.3118398780.295466917	0.249735047
0.278447925	0.242442801	0.271745026	0.228966938
0.292446807	0.215219201	0.293730697	0.252733458	0.31798896
0.259210893	0.333224998	0.28222573	0.25946773	0.273508498
0.253461672	0.287395531	0.298135506
KEGG_DORSO_VENTRAL_AXIS_FORMATION	0.0664347110.1280217

0.077307455 0.030713569 0.053651313 0.038767764
0.007425605 0.071349371 0.057522179 0.074740338
0.0334211250.064737378 0.085833265 0.035826781 0.084307727
0.063290738 0.0747866110.031985704 0.060192774 0.040967855
0.061092194 0.060553526 0.049798571 0.1126137470.080322628
0.0587118050.040225923 0.05214868 0.083629243 0.10060866
0.004259562 0.064510706 0.094342672 0.045264978
0.087732325 0.02177251 0.070712418 0.0336488110.035495082
0.03222298 0.07686784 0.060834033 0.044368999 0.050963215
0.040206801 0.0162480110.056300818 0.076594348 0.042837928
0.0203351140.1104721290.067807316 0.07018234 0.043286734
0.057265175 0.08742951 0.060145203 0.076396561 0.026736411
0.076733039 0.049432513 0.074328437 0.04916532 0.041017955
0.094910233 0.055606715 0.027878794 0.037749921 0.06079697
0.064537568 0.081439223 0.027872069 0.07216087 0.041071653
0.080446227 0.125923004 0.054155641 0.07337096 0.071836246
0.056841226 0.055392009 0.051206387 0.060266341
0.064951475 0.082343422 0.08359179 0.069705608 0.065684384
0.063839615 0.052717103 0.050073932 0.010846333
0.046822665 0.055734354 0.104560304 0.053693025
0.067876523 0.067862223 0.068143895 0.077398661
0.092071837 0.070005175 0.049622017 0.075854837
0.049926578 0.1142233310.062541034 0.047987762 0.066456285
0.064019402 0.021396236 0.0658291180.054156686 0.049636593
0.03389285 0.06710653 0.0699061190.051528941 0.075593273
0.08496079 0.0758655110.053906041 0.047389514 0.078781476
0.076390865 0.03197564 0.060806919 0.053669095 0.076587341
0.069796443 0.067600232 0.084866345 0.038661272
0.094620498 0.071467224 0.051490163 0.079977691
0.098170106 0.107546775 0.144645699 0.042170769
0.082785813 0.050497653 0.06206998 0.026417306 0.082671917
0.088975598 0.018355782 0.073305377 0.066610248
0.1118285560.049952088 0.106323526 0.034276762 0.067214549

0.079925385 0.1149898080.098389845 0.084687348 0.095550113
0.050178804 0.078685665 0.046160332 0.062679067
0.068046924 0.046216243 0.092436104 0.062262925
0.069567899 0.052549528 0.088376849 0.051449198
0.046330387 0.077244887 0.079080274 0.079939434
0.049429955 0.054367606 0.052637679 0.082008434
0.087516378 0.073648533 0.040523501 0.039188625 0.06916059
0.042141392 0.007340209 0.093574643 0.059691405
0.031691265 0.082935622 0.09622319 0.127964121 0.098479733
0.068515813 0.120410424 0.0581154040.107955535 0.101377608
0.104714628 0.1147721310.074298496 0.093744765 0.066909478
0.07856656 0.065905301 0.071036313 0.055348394 0.089805827
0.086515268 0.077545247 0.086980338 0.089917175
0.063164708 0.077508042 0.079710163 0.083588385
0.092125191 0.1134757970.084388474 0.083019427 0.054600031
0.065130019 0.049824301 0.084659826 0.050281015
0.070133242 0.0821641160.028280055 0.1155342140.06319924
0.093468098 0.03797789 0.052710357 0.0711020350.071018142
0.0791181680.089717848 0.075851474 0.07149972 0.069082684
0.044085341 0.062509837 0.047500789 0.069614395
0.088133599 0.013962966 0.043438183 0.0122221120.059921743
0.077066058 0.10033445 0.073012227 0.025383525 0.052629225
0.073700398 0.016443475 0.076585531 0.026922567
0.067737454 0.026263165 0.050615899 0.030134171
0.0741511190.04508153 0.059271686 0.039077046 0.047731979
0.050341904 0.04642206 0.055139486 0.045278652 0.111708373
0.012792946 0.050905827 0.042840209 0.0784811470.038948009
0.0611790410.062318673 0.053496872 0.047523996 0.058217387
0.06081035 0.082424496 0.080663255 0.099560197 0.12676319
0.109457448 0.09309929 0.067131864 0.063297716 0.053074393
0.076852188 0.061549542 0.073296866 0.057667079
0.059251999 0.092000276 0.034550597 0.064772915
0.062581371 0.086890961 0.069736986 0.066709295 0.09212108

0.095978155 0.053995149 0.078920557 0.07308027 0.085157183
0.06944573 0.060014483 0.0148571140.087757862 0.14351566
0.084551844 0.030616736 0.040969923 0.057325924
0.077404399 0.05108946 0.074678316 0.063849285 0.057510645
0.087715823 0.056526625 0.022345465 0.066797456
0.058656602 0.09336776 0.090741744 0.064647447 0.086668333
0.09892359 0.046957109 0.070052613 0.087862607 0.088552878
0.047748585 0.0851413 0.083649898 0.084531062 0.128539004
0.091377017 0.071027121 0.044125067 0.091397765
0.127950664 0.071512814 0.058433744 0.048652242
KEGG_DRUG_METABOLISM_CYTOCHROME_P450 -0.127113086 -
0.115827107-0.108411724 -0.099915551 -0.058158169 -0.123778023 -
0.118908699-0.165407249 -0.100597885 -0.123830356 -0.145266764 -
0.15725765 -0.154628336 -0.126177122 -0.145895861 -0.071497585 -
0.127400125 -0.088182275 -0.124868475 -0.109031197 -0.055592579
-0.093260409 -0.085283758 -0.096398309 -0.113691798 -
0.131593398 -0.110161698 -0.09379116 -0.105321695 -0.128215725 -
0.061275582 -0.145242184 -0.148401909 -0.096844677 -0.138816964
-0.130358636 -0.094657732 -0.096018329 -0.115607184 -
0.129840916 -0.124962682 -0.086447894 -0.103251395 -0.104432193
-0.128614698 -0.104615879 -0.16550599 -0.055464082 -0.103506484
-0.09909914 -0.095877684 -0.126742969 -0.144926422 -0.099130363
-0.045830837 -0.119864306 -0.130464447 -0.086166574 -
0.117722326-0.131477035 -0.039223697 -0.176055425 -0.132382799 -
0.155057931 -0.149899444 -0.118169226 -0.108243827 -0.088348884
-0.120001718 -0.127183491 -0.127704902 -0.11812368 -0.131471483
-0.085268887 -0.130993834 -0.087203946 -0.096015627 -
0.14292348 -0.107924488 -0.099421765 -0.099089114 -0.129592184 -
0.137496075 -0.142694229 -0.104841773 -0.154243226 -0.128480742
-0.08755177 -0.141039502 -0.099012314 -0.140573024 -0.072600278
-0.104582928 -0.104819159 -0.134528009 -0.171250448 -
0.115005833-0.174981245 -0.127454736 -0.145642296 -0.118104881 -
0.151882097 0.003203913 -0.129360778 -0.10434937 -0.085685756 -

0.092481293 -0.124086303 -0.130809446 -0.139011797 -0.106212157
-0.151452472 -0.160832834 -0.144535294 -0.112444044 -
0.12046898 -0.123704015 -0.129908177 -0.146915579 -0.130991959 -
0.142209616 -0.167652047 -0.115874461 -0.163807763 -0.16465654 -
0.08512343 -0.151893063 -0.115487207 -0.102080794 -0.101476233 -
0.171875688 -0.123648208 -0.132361905 -0.164850867 -0.142875048
-0.130266886 -0.149501049 -0.119818018 -0.132918655 -
0.112761875-0.134131397 -0.119604397 -0.155479932 -0.106571533 -
0.120090991 -0.123953254 -0.171206014 -0.096437071 -0.152338483
-0.117267431 -0.099689119 -0.115989896 -0.178009137 -
0.086020798 -0.147215624 -0.176868579 -0.172587033 -0.100358043
-0.154569526 -0.08962613 -0.142524314 -0.151882649 -0.120281188
-0.103660261 -0.139790217 -0.063366237 -0.129159632 -
0.102878276 -0.108233165 -0.059580375 -0.171063727 -0.111899751
-0.131876101 -0.099704848 -0.141420346 -0.146465645 -
0.125258877 -0.084545074 -0.066845007 -0.183470621 -0.130785442
-0.059772007 -0.129352047 -0.105757334 -0.103146955 -
0.13404029 -0.108926287 -0.092368838 -0.101868396 -0.091514391 -
0.140910336 -0.114238381 -0.144822415 -0.128885452 -0.116264443
-0.131072133 -0.147430171 -0.10828634 -0.154894979 -0.151122391
-0.120524555 -0.147486017 -0.138662167 -0.158140887 -
0.118301798-0.110237539 -0.152737036 -0.111828472 -0.121726601 -
0.127295813 -0.153997291 -0.144013643 -0.148303297 -0.099734547
-0.145818139 -0.129141533 -0.168795598 -0.149170236 -
0.103769544 -0.089518202 -0.166132723 -0.117306696 -0.11169671 -
0.118836728-0.140557883 -0.113450002 -0.15056155 -0.104613409 -
0.128035816 -0.128308284 -0.130925992 -0.142322387 -0.143245881
-0.154116616 -0.113252597 -0.126929137 -0.121845599 -
0.159432939 -0.121373947 -0.107076843 -0.142534801 -0.114291551
-0.134183133 -0.134706109 -0.146858136 -0.134511232 -
0.086308221 -0.147034071 -0.061638276 -0.147420223 -0.149936255
-0.133355654 -0.141677956 -0.149319001 -0.114772515 -
0.113457296-0.120449927 -0.095803537 -0.118691096 -0.127955937 -

0.084809808 -0.120560481 -0.10211181 -0.112070077 -0.147416602 -
0.13616818 -0.12680682 -0.152508474 -0.124023959 -0.087231227 -
0.098518745 -0.134347464 -0.168831368 -0.09919819 -0.127945099 -
0.114170148 -0.111235159 -0.136766684 -0.1600973 -0.145778781 -
0.132988309 -0.130510948 -0.108902353 -0.11715909 -0.15705129 -
0.115178689 -0.169241628 -0.163913811 -0.111126981 -0.121276851 -
0.121347605 -0.125839368 -0.134323664 -0.149429229 -0.141563508
-0.122832858 -0.156827189 -0.138100357 -0.091980232 -
0.178169963 -0.114395227 -0.117143663 -0.150588024 -0.11603136 -
0.130998621 -0.133759649 -0.138897458 -0.138821562 -0.133573381
-0.143428041 -0.140228837 -0.1047066 -0.138064837 -0.146657837
-0.153450634 -0.06411382 -0.148308696 -0.128084643 -0.166621211
-0.146937106 -0.128067453 -0.06600361 -0.125742981 -0.114711783
-0.154051449 -0.164423463 -0.120181309 -0.098744514 -
0.117316596 -0.132985402 -0.134534432 -0.136199031 -0.153001473 -
0.135911423 -0.12894588 -0.146246392 -0.132004288 -0.143269659 -
0.111249155 -0.030013786 -0.117803937 -0.1176484 -0.13603061 -
0.174213369 -0.133465478 -0.114495792 -0.088453116 -0.093302628
-0.181805313 -0.097981942 -0.147196062 -0.123557917
KEGG_DRUG_METABOLISM_OTHER_ENZYMES -0.070807587 -
0.051385337 -0.022379815 -0.056762779 -0.052625304 -0.070708349
-0.095131365 -0.060424159 -0.066857449 -0.057758334 -
0.079275717 -0.059053664 -0.10334231 -0.078930029 -0.061414044 -
0.021429547 -0.058267399 -0.011199797 -0.063964011 -0.057400341
-0.062645516 -0.058406224 -0.034631152 -0.089940139 -
0.064196065 -0.070700373 -0.053378593 -0.063083253 -0.054471633
-0.074216526 -0.059965853 -0.047878235 -0.10544395 -0.072815048
-0.071224356 -0.050729007 -0.057715204 -0.032880234 -
0.06865731 -0.059197112 -0.048604822 -0.06077892 -0.060778126 -
0.027773096 -0.059876418 -0.058456581 -0.074712043 -0.068008334
-0.045259478 -0.03931222 -0.047243683 -0.049823447 -0.071262115
-0.052284161 -0.036471847 -0.065559657 -0.053862669 -
0.035314568 -0.066497875 -0.108428786 -0.054672531 -0.058076633

-0.073549305 -0.035699371 -0.086397813 -0.060328587 -
0.061638183 -0.037037507 -0.061763188 -0.081956584 -0.043647541
-0.088222802 -0.071340728 -0.074542367 -0.031909482 -
0.038508448 -0.08615871 -0.06441878 -0.054453667 -0.054488036 -
0.04554011 -0.057920578 -0.058320207 -0.06943021 -0.053273763 -
0.058700136 -0.041916618 -0.071851241 -0.060321405 -0.07011284 -
0.055454824 -0.033528698 -0.040932363 -0.045604642 -0.06750262 -
0.06397385 -0.044273989 -0.069994448 -0.043895566 -0.031148778 -
0.07948477 -0.074077925 -0.024692196 -0.079567106 -0.085217993 -
0.054182031 -0.075040669 -0.053911349 -0.058655908 -0.071069573
-0.058563984 -0.077069709 -0.083047284 -0.041811886 -
0.077752267 -0.041617408 -0.064428908 -0.044500296 -0.073523454
-0.07316627 -0.064023941 -0.068974266 -0.069581101 -0.056110406
-0.053614849 -0.051634227 -0.06630593 -0.030293329 -0.075429183
-0.054217431 -0.074413331 -0.079632516 -0.073520993 -
0.06978204 -0.076993011 -0.103500563 -0.06682038 -0.05200518 -
0.065489902 -0.053566413 -0.078253174 -0.057573094 -0.05788672 -
0.085446818 -0.041122536 -0.06103418 -0.069124559 -0.024484643 -
0.076905452 -0.069242155 -0.039035771 -0.059666625 -0.079962377
-0.042721741 -0.047283805 -0.086160264 -0.081805947 -
0.063847614 -0.065494963 -0.060666725 -0.067492805 -0.080762461
-0.064171511 -0.070439388 -0.06038721 -0.079463082 -0.055625604
-0.053852167 -0.05291819 -0.029881081 -0.046964631 -0.05290826 -
0.079438165 -0.050701386 -0.060489794 -0.051843497 -0.056846422
-0.081877655 -0.016608117 -0.070515397 -0.061805103 -
0.063232688 -0.045279603 -0.046696415 -0.058535633 -0.060572527
-0.077761525 -0.043283372 -0.062748397 -0.05812516 -0.053922391
-0.058613095 -0.050675515 -0.048628111 -0.071035082 -
0.063287674 -0.054398303 -0.07211296 -0.064758111 -0.056639015 -
0.057811097-0.083197104 -0.094074142 -0.070495975 -0.053892569 -
0.04806673 -0.094898701 -0.078755326 -0.06796759 -0.058195488 -
0.071471056 -0.072399983 -0.066963997 -0.069325159 -0.061269911
-0.085560451 -0.072280014 -0.058482968 -0.050696048 -

0.049130155 -0.067694973 -0.038906592 -0.071976884 -0.076690584 -0.093690785 -0.083558649 -0.054544238 -0.081393979 -0.067095005 -0.07095241 -0.065393454 -0.071978263 -0.046237958 -0.06902527 -0.054222742 -0.066186367 -0.057358171 -0.053711616 -0.052582141 -0.063416391 -0.059603736 -0.030213812 -0.09741203 -0.055191836 -0.05569229 -0.059273551 -0.056396858 -0.087596722 -0.012521482 -0.083535916 -0.053388988 -0.041600045 -0.070704813 -0.06896498 -0.06925276 -0.08273699 -0.054833439 -0.046457857 -0.045910259 -0.048850976 -0.076082448 -0.052919741 -0.044258636 -0.049202021 -0.066737608 -0.083481688 -0.05396191 -0.057127435 -0.067457258 -0.050212952 -0.028828831 -0.073303084 -0.069855989 -0.038447218 -0.043053899 -0.094957195 -0.032307978 -0.057576369 -0.071873965 -0.068861538 -0.062976143 -0.030263076 -0.051859027 -0.049958123 -0.058860653 -0.062911974 -0.068924525 -0.053749804 -0.0346405 -0.066748023 -0.048491903 -0.06711305 -0.072504693 -0.075474149 -0.046186825 -0.064512081 -0.092564131 -0.053891262 -0.075542812 -0.067021118 -0.068048361 -0.073772673 -0.061168909 -0.061723131 -0.057284001 -0.068103302 -0.063254585 -0.07357734 -0.070861986 -0.079472143 -0.068981539 -0.071027314 -0.099734631 -0.011171183 -0.09191883 -0.079694224 -0.068436536 -0.040040796 -0.065718508 -0.074208249 -0.038903772 -0.048265756 -0.072580557 -0.064197759 -0.062656423 -0.061936336 -0.056867595 -0.044829385 -0.062420684 -0.066117978 -0.069914921 -0.048014075 -0.084516607 -0.052002715 -0.052466992 -0.058968972 -0.062165231 -0.051562376 -0.071141479 -0.016252815 -0.063442174 -0.091919464 -0.056083842 -0.088013272 -0.066945763 -0.060556768 -0.049742994 -0.037691946 -0.06522457 -0.063767852 -0.066329006 -0.085524483

KEGG_ECM_RECEPTOR_INTERACTION 0.097744371 0.179554253 0.102413545 0.068020554 0.108230083 0.054674804 0.009096103 0.074032762 0.080757597 0.063845579 0.016670559 0.0381136460.097222474 0.0192480110.148521095

0.051728702 0.079145417 0.036843871 0.044399072
0.080228996 0.055685291 0.052092671 0.023476083
0.096457814 0.099218249 0.02618862 0.064687346 0.043586269
0.033273835 0.073130442 0.031572996 0.055277075 0.11986841
0.102301765 0.056391098 0.016667946 0.079844007
0.033083158 0.041377301 -0.005097883 0.11432562 0.046105844
0.061222887 0.070542566 0.069109571 0.123023312
0.124120747 0.092857323 0.004297383 0.037398902
0.151602859 0.080543282 0.0375002 0.014570396 0.050805455
0.136109613 0.0652172110.105423666 0.048509442 0.111102476
0.03734233 0.102458381 0.057986765 0.106073838 0.057409317
0.022195632 0.021602094 0.069016722 0.09554734 0.14086796
0.1408968 0.016781709 0.1311060520.100713424 0.138377083
0.146813408 0.074581419 0.109622207 0.137032184 0.11140051
0.055935008 0.0011576780.015878741 0.070766767 0.029178846
0.062099835 0.077417539 0.106377537 0.087225028
0.105408837 0.021961623 0.062672086 0.098464564
0.040145991 0.128482382 0.065901364 0.074314919
0.060770643 0.04090546 0.039365429 0.098256305 0.07866441
0.062472096 0.085414008 0.051302146 0.13531078 -0.020080978
0.104233205 0.036179104 0.080731893 0.0112815040.127510351
0.066309638 0.10292082 0.093022985 0.135794259 0.06583941
0.072229083 0.093266125 0.1116108140.036891623 0.140301778
0.098882842 0.143484223 0.123176807 0.01340671 0.079350449
0.1122230420.05967042 0.074288317 0.132797043 0.049078858
0.042858127 0.1164340010.1186098660.053659416 0.140761249
0.103421059 0.107397806 0.175778447 0.078044256
0.130072298 0.092915931 0.079671051 0.057140348
0.1527221120.129790474 0.03376246 0.056899423 0.069088175
0.095037539 0.031734639 0.069803768 0.040021737
0.122446987 0.089504155 0.135413342 0.0837968110.083711607
0.077407016 0.066038944 0.069884824 0.046887836
0.1111675820.13504814 -0.00729909 0.085521064 0.049405764

0.081226419 0.071366647 0.093629862 0.051254702

0.074255945 0.032030449 0.1152618980.101516786 0.099021696

0.107649873 0.03872135 0.1103852450.10771026 0.072930342

0.055719627 0.094803062 0.062212786 0.101379954

0.079958824 0.165533337 0.095471829 0.052589878

0.144463438 0.122873691 0.13135293 0.087108556 0.102801244

0.155552954 0.077387638 0.122555504 0.14725426 0.084936431

0.161989793 0.084384638 0.150624661 0.0722801180.073777888

0.045096789 0.078589022 0.1167198290.145582475 0.058534035

0.130291943 0.05615494 0.125928546 0.1151284130.133659172

0.149171875 0.1197371670.15393532 0.180719819 0.062086683

0.140955837 0.088276799 0.106789374 0.063201026

0.155284873 0.072997931 0.126574488 0.071254207

0.032794763 0.193156448 0.048258033 0.088578293

0.072144268 0.064064733 0.151568681 0.126407036

0.074231998 0.130287801 0.14192525 0.102662048 0.091131872

0.106844555 0.069635627 0.102362551 0.13995282 0.119204089

0.023139467 0.0117364980.044590176 0.080266832 0.12303928

0.127686454 0.108680864 0.014275252 0.064436508

0.084003314 0.097062833 0.1120732460.062354219 0.136079146

0.073642097 0.087643369 0.084170423 0.0711756990.029740288

0.09297835 0.010582165 0.064499854 0.11635468 0.068348729

0.075285065 0.050067518 0.158078701 0.035966667 0.10203799

0.045491213 0.120280784 0.04627736 0.123218951 0.107343594

0.03078906 0.084600106 0.1102372120.1130581110.117223921

0.041990219 0.14438657 0.133128633 0.1227789110.153374345

0.095565432 0.121008394 0.053377155 0.0611162550.121087487

0.139134614 0.0716118150.130189523 0.107854859 0.088440937

0.092169658 0.069907009 0.143777446 0.052763866

0.094444898 0.097288628 0.091465734 0.09985838 0.100595323

0.1137445890.12330442 0.1044111910.079075104 0.068874375

0.1029163110.146484358 0.089792075 0.0711494220.054590698

0.094292535 0.13070856 0.012660871 0.088041743 0.105992506

0.022778546	0.079484701	0.049721061	0.042159053
0.081042595	0.107349823	0.058126488	0.137159642
0.072529341	0.11182658	0.179246962	0.099767903	0.097606032
0.082913026	0.13216965	0.006556607	0.149420028	0.107220012
0.1167393280.169717556	0.092289231	0.063716026	0.038311004
0.058497906	0.142832397	0.044210701	0.083561892
0.035733765
KEGG_ENDOCYTOSIS 0.22786084	0.247876141	0.249694283
0.229163509	0.247558109	0.2189112660.2223211730.232521664
0.250064676	0.239094523	0.215767054	0.246554251
0.227019047	0.226429628	0.251689174	0.244188422
0.235553612	0.248294467	0.227694176	0.221887778
0.209143146	0.254527508	0.242853135	0.232741275
0.240284304	0.234591969	0.229916949	0.238659672
0.225953473	0.251934132	0.20522316	0.260853149	0.228215591
0.229965266	0.244939104	0.237816788	0.2211284040.224332753
0.232555994	0.199615526	0.244448476	0.234953472
0.232336618	0.227682601	0.247553278	0.236020546
0.235416931	0.242803191	0.247262438	0.221675441
0.252451897	0.239534089	0.237885264	0.231930939	0.23008789
0.241728601	0.258792273	0.256203799	0.226188842
0.231303933	0.242712441	0.261729238	0.237473235
0.259714562	0.260557453	0.234648935	0.246998525
0.244587375	0.244437281	0.231068642	0.248972188
0.2110395540.247739564	0.219819679	0.260403083	0.26407407
0.217962593	0.246853609	0.239739684	0.22245549	0.249606553
0.224072974	0.212813171	0.223057877	0.266365161	0.23346334
0.263620177	0.247913346	0.227581677	0.225031007
0.229924128	0.233183801	0.234177789	0.252723246
0.248538602	0.264909247	0.245145708	0.228137152
0.261519248	0.258026794	0.235253919	0.222015483
0.219199232	0.2511085720.220920102	0.258893423	0.234376761
0.236649915	0.23755135	0.256760648	0.234955677	0.232850968

0.218172796 0.237914825 0.229516724 0.251318412
0.230072643 0.228848087 0.243916722 0.24132887 0.240490189
0.223932715 0.2407765 0.240323901 0.2491116090.218796376
0.24826788 0.2322114570.230297038 0.25660207 0.252663926
0.25721674 0.227268042 0.255964437 0.241743277 0.234741225
0.245573224 0.247704438 0.243856837 0.258237123
0.239135142 0.254752961 0.238581526 0.23013299 0.231091814
0.255964818 0.244327997 0.239404766 0.236196871
0.239600886 0.254178698 0.224249898 0.227684591
0.232862795 0.250802125 0.275459313 0.251348049 0.25261171
0.234032173 0.262075585 0.250904915 0.232574624
0.232496488 0.255466222 0.23246266 0.247282186 0.222435115
0.234538747 0.254214564 0.230266534 0.252931934 0.24180494
0.226980165 0.229653494 0.23644678 0.256554658 0.240387138
0.237960941 0.250428402 0.24424606 0.256018489 0.217708545
0.245320781 0.232320506 0.249451098 0.218003406
0.215554886 0.25811479 0.217141085 0.234872701 0.23484754
0.252547032 0.261795456 0.254655676 0.252589521
0.241659716 0.24668426 0.248258371 0.252732772 0.250402623
0.269246775 0.231826701 0.249241886 0.240436377
0.263191487 0.21347931 0.246105657 0.238560536 0.236910291
0.251249445 0.242417905 0.244133199 0.248944952 0.23175518
0.243242182 0.247919238 0.238486276 0.26095002 0.256640938
0.230922905 0.255122403 0.24205584 0.24342238 0.227027217
0.274285532 0.215889216 0.236953427 0.233515898
0.206325562 0.258879408 0.220300874 0.251608691
0.2311136380.243418932 0.241093637 0.251786652 0.233450163
0.254295167 0.255130137 0.255686162 0.236123949
0.2411359450.215362094 0.229610495 0.250102915 0.244378475
0.2131175940.22929063 0.217177803 0.2224676110.240346998
0.265592742 0.261998161 0.233501378 0.209699431
0.251053094 0.222886964 0.239458612 0.228475507
0.2491189470.214634715 0.224882867 0.233098504 0.220199418

0.234555848 0.258073135 0.22820039 0.243983907 0.234139891
0.209851308 0.266761669 0.247296171 0.251939246
0.206592404 0.241573023 0.214304143 0.257886929
0.243093286 0.241333164 0.247160355 0.233617283
0.237260739 0.225458259 0.241966017 0.246764679
0.250988141 0.264309985 0.257483817 0.260930159
0.255258926 0.250697421 0.252402996 0.236972171
0.228557346 0.232801959 0.25074001 0.220619062 0.260446678
0.263705626 0.2311935330.228709592 0.246087554 0.250384129
0.253357624 0.253016565 0.258178454 0.25259326 0.238025456
0.245489966 0.248686621 0.259397262 0.251939269
0.224790204 0.236066238 0.2297811480.254245957 0.231311066
0.222256289 0.227387602 0.237783299 0.235965881
0.220803877 0.246580417 0.2340961110.241230595 0.226598196
0.24474483 0.221256915 0.241749328 0.243374456 0.245256446
0.2354511070.23536073 0.2458042 0.256636376 0.248960974
0.2329576 0.2521141140.228755816 0.228105505 0.248880192
0.222423894 0.237610524 0.224915868 0.264303731
0.242919733 0.23342132 0.251312661 0.2591991180.21427514
0.238310347 0.222730866

KEGG_ENDOMETRIAL_CANCER 0.183527778 0.233262871
0.214996249 0.19517021 0.200039553 0.222497175 0.230618071
0.216589251 0.224079247 0.226574127 0.190392521
0.198770276 0.232644157 0.251635595 0.224229263
0.207615687 0.21414505 0.2091101630.2113835650.229562829
0.242979738 0.225182267 0.207235599 0.217530775
0.242732501 0.243290941 0.20893214 0.241243308 0.240372202
0.2311437 0.194840736 0.218613017 0.221778054 0.242451876
0.244280657 0.203283648 0.217014284 0.193970262
0.200736829 0.189730745 0.231354498 0.210722574
0.193834651 0.1911810450.221258986 0.1990527 0.204016941
0.2184703110.2079112720.206226475 0.205507888 0.198588323
0.221406642 0.223058859 0.205200777 0.243404503

0.206432707 0.22882302 0.163691549 0.213867267 0.209671658

0.214092279 0.218220435 0.193123569 0.2111135250.207647687

0.17672369 0.205192106 0.212045129 0.2117495020.225670637

0.202581026 0.213955717 0.224239325 0.227196024

0.241588768 0.2111084070.223880814 0.214714193 0.224035798

0.202296281 0.190748537 0.212972468 0.247470971

0.207121474 0.190928744 0.2411953030.224272958 0.237105876

0.251793707 0.202964777 0.1876529110.19493789 0.188303579

0.239371947 0.220448452 0.204899633 0.217178332

0.204209704 0.208320702 0.2277751180.238315337 0.192414421

0.23476933 0.215406865 0.217996637 0.212107503 0.196596148

0.2148441180.216767663 0.190177948 0.214655727 0.214068295

0.222977491 0.202486065 0.220851391 0.217442625

0.186361488 0.227396582 0.233783774 0.220759152

0.2115982090.220830249 0.237589371 0.23478825 0.180258718

0.225449666 0.230957216 0.195222868 0.227202367

0.2234711770.216835898 0.228351749 0.217941867 0.230792081

0.251430857 0.243830924 0.235700669 0.243860202 0.23007598

0.219718299 0.256251445 0.209187879 0.247278424

0.213353414 0.2382511070.231256784 0.2115743260.230179015

0.229448241 0.222476761 0.233687071 0.242013795

0.213870013 0.229252165 0.236891641 0.228653941 0.24390877

0.225617652 0.248331569 0.216386418 0.244259275

0.236143384 0.232337475 0.210440224 0.206616823

0.221962841 0.24584026 0.218969572 0.249057223 0.243803458

0.2135011390.215907553 0.197329706 0.200629449 0.225499815

0.21329477 0.227284649 0.196320354 0.238918919 0.220964623

0.194170586 0.236431524 0.221921226 0.227417735

0.199085024 0.22160793 0.244473915 0.214436132 0.236202075

0.236032462 0.248800845 0.2164041190.230689357 0.224605454

0.242425745 0.209409024 0.240126977 0.257045971 0.23139741

0.233019502 0.212683302 0.225362404 0.225402363

0.236803986 0.227723975 0.2211470770.239561345 0.234858885

0.245000907 0.238631318 0.232403771 0.241456562

0.232097026 0.227901593 0.245815301 0.227375626

0.232221561 0.248136762 0.251816244 0.240337963

0.229562909 0.220654416 0.228802899 0.259534999

0.235495949 0.252193459 0.213589648 0.180791273

0.250103815 0.222142356 0.243947201 0.225823724

0.213058714 0.237759144 0.247766328 0.243993367

0.234887252 0.234451412 0.2247591140.206929138 0.212400697

0.201312645 0.200845762 0.223558003 0.245354436

0.175425893 0.231668396 0.178081565 0.230067066

0.225941393 0.236043962 0.217999836 0.182461005

0.209617146 0.2224116460.199873623 0.2284186110.196537393

0.236068107 0.215761868 0.229552443 0.183002593

0.229998708 0.18939231 0.228679525 0.186325502 0.204750992

0.222965318 0.235756965 0.217745778 0.203590681

0.259329393 0.184843761 0.196323676 0.221960672

0.204063327 0.199007098 0.218874293 0.2160015110.20715706

0.249655559 0.204797391 0.21126973 0.218580676 0.231139914

0.223843885 0.2402111430.229476531 0.245859445 0.23711753

0.256717916 0.191897482 0.232839864 0.202629483

0.219972446 0.230210058 0.237817838 0.23524484 0.229348189

0.200026176 0.2241465 0.250412404 0.21400037 0.232713294

0.238181945 0.250562617 0.231932541 0.228257698

0.253061326 0.243270873 0.226138905 0.222998032

0.206170982 0.244906359 0.244754599 0.227150617

0.192087775 0.21376085 0.23268422 0.21896959 0.218215285

0.236722412 0.228838266 0.213953331 0.206097375

0.225639248 0.198052068 0.232786706 0.226153221

0.269575998 0.228509402 0.226133823 0.226642632

0.241957443 0.235212035 0.20365349 0.21633714 0.232513536

0.208312424 0.237776016 0.231620193 0.227098258

0.222758459 0.242255825 0.216246947 0.263192863

0.230064845 0.242715472 0.241581488 0.218163884

0.216953475

KEGG_EPITHELIAL_CELL_SIGNALING_IN_HELICOBACTER_PYLORI_INFECTION 0.148667382 0.152029205 0.188931245 0.157292327 0.156554274 0.157737214 0.16338862 0.170781302 0.164260366 0.170296369 0.1403441 0.185546514 0.137393314 0.166080877 0.172270539 0.173960246 0.156493892 0.166055084 0.169467 0.167335213 0.13316707 0.2011350340.183903342 0.158929223 0.207076303 0.172466257 0.152315484 0.162185021 0.165481541 0.180666389 0.169198297 0.171832376 0.159231529 0.135775651 0.158459249 0.167421576 0.148890188 0.151671381 0.161946247 0.137223783 0.166015971 0.168544338 0.152589802 0.145922697 0.167856554 0.152477566 0.155154832 0.181577807 0.1876641190.166794623 0.177164829 0.1552691150.152065682 0.149031739 0.19678998 0.189533593 0.154668754 0.176459439 0.156739332 0.14623403 0.1452712110.180900546 0.171605695 0.187630564 0.163756107 0.141056807 0.1643214 0.191322067 0.174427568 0.17497472 0.180645604 0.152956976 0.168006333 0.159157961 0.160009638 0.207375844 0.167739494 0.181966963 0.18340002 0.178374396 0.157419445 0.140388816 0.162294626 0.152044312 0.160614545 0.185989159 0.176837974 0.162304816 0.151428661 0.168980897 0.161069 0.179682331 0.155813299 0.157349637 0.15915816 0.178477873 0.197344292 0.171646787 0.176743094 0.173330219 0.178183507 0.148463259 0.157190598 0.17296534 0.138268947 0.167215607 0.136169932 0.187812845 0.20584222 0.179693963 0.158621643 0.149755295 0.17913739 0.164379788 0.13680337 0.19728663 0.185668373 0.158003614 0.195139096 0.148538088 0.176859291 0.15239435 0.161078018 0.156237841 0.165358819 0.190756175 0.163524724 0.154470002 0.163467922 0.170596626 0.174819721 0.170151818 0.149659738 0.208475058 0.178703767 0.155855812 0.174031478 0.182583269 0.17286539 0.188240971 0.144483941 0.189013927

0.17537073 0.166308912 0.140795342 0.197620268 0.183145953
0.167616868 0.161326024 0.169853896 0.179682814 0.15663259
0.189991301 0.147272288 0.16716021 0.161833522 0.188915451
0.17932867 0.164362913 0.183064393 0.160916445 0.170386173
0.162545164 0.176142954 0.176717413 0.163471398
0.145192562 0.173193237 0.191024569 0.166033153
0.168717861 0.172518579 0.133265938 0.163417358
0.192654622 0.195747876 0.177744755 0.162598059
0.195541408 0.176303575 0.181466573 0.154368931
0.187425787 0.166378669 0.176156234 0.154332127
0.157143751 0.179597999 0.172951349 0.170979567
0.184210596 0.178595183 0.198619384 0.1837048110.178341446
0.16260195 0.178047124 0.176107218 0.18209858 0.18656698
0.192229192 0.13683849 0.162883679 0.15039696 0.179341307
0.139505299 0.142793056 0.1593819110.183496914 0.164940712
0.160200709 0.165541881 0.173993944 0.143856529
0.177426173 0.186387389 0.169478798 0.188482783
0.189653341 0.184733185 0.166760585 0.192571668
0.181826447 0.166808108 0.1963112590.158272969 0.17189274
0.164687226 0.170726166 0.1920114330.182901046 0.194294735
0.175963871 0.165015276 0.1882410110.17984214 0.146137551
0.188223157 0.17361319 0.178171848 0.177740914 0.193193425
0.154451821 0.160769738 0.193877625 0.178427704
0.169521508 0.155285832 0.169684351 0.181733175
0.175159978 0.191053726 0.20098863 0.162064913 0.160748365
0.156202296 0.174210783 0.168261299 0.170514133
0.176835231 0.148026388 0.156969438 0.171992456
0.1661180920.181669871 0.17869509 0.1660911580.179846047
0.157157159 0.156659938 0.166906009 0.160826696 0.16559909
0.163659719 0.171461496 0.158638355 0.175954238
0.172652712 0.147105381 0.1649323110.147063298 0.171063849
0.170289575 0.190475603 0.175069961 0.1879112320.190316548
0.189491338 0.195188163 0.1883048 0.18578166 0.181832648

0.168629647 0.159372954 0.218283281 0.18473698 0.158531489
0.197434403 0.193003091 0.168795817 0.164652134
0.169408653 0.194261033 0.1567811330.15890837 0.175492445
0.190457309 0.163850485 0.15674858 0.168877856 0.180672011
0.180522015 0.168677393 0.187603364 0.150188162
0.1832801170.173454502 0.181815749 0.180370044 0.165174594
0.174485225 0.172216808 0.135227921 0.174190591
0.189665989 0.160043638 0.168518079 0.171499903
0.168065337 0.193008583 0.168436191 0.178104295
0.184474545 0.1839290110.203879919 0.176749623 0.18713712
0.202587122 0.137880088 0.163176368 0.185661747
0.171835193 0.173868361 0.164772277 0.198673906
0.158905281 0.150313094 0.195324841 0.200086446
0.142602005 0.17470659 0.137154795
KEGG_ERBB_SIGNALING_PATHWAY 0.150714605 0.171984579
0.183314305 0.154172786 0.160171326 0.15697738 0.159937205
0.165669599 0.160967768 0.1879115390.1515211620.175760555
0.153875231 0.149186984 0.1771147360.165752825 0.149514758
0.150267139 0.162661201 0.179688361 0.159084133
0.173790759 0.150765194 0.141769501 0.169162157
0.172652738 0.144454459 0.171478357 0.153310843
0.179884208 0.141728863 0.170977701 0.143819644
0.152890026 0.173663533 0.156398222 0.152553092
0.147088041 0.13935878 0.147848099 0.162512658 0.172734351
0.144101779 0.139844402 0.173754552 0.152003054
0.153738009 0.146623944 0.146687388 0.141920834
0.160072802 0.155333035 0.162399129 0.169360037
0.139815445 0.173698569 0.157675587 0.163441315
0.136718774 0.1581361190.149812077 0.174282639 0.166450936
0.146367107 0.16203294 0.156615266 0.145952886 0.16009597
0.169435006 0.146035309 0.184270329 0.139723364
0.152815599 0.144010296 0.172166365 0.194445187
0.167901953 0.167436251 0.163045402 0.167432346

0.154538341	0.13460554	0.146756783	0.167642828	0.163033824
0.15209148	0.177398567	0.15717607	0.179192833	0.157364725
0.150709722	0.1550113570.154620156	0.146351519	0.182250537
0.1801186890.15801583	0.150510579	0.160360499	0.157823168
0.173606397	0.163396951	0.144694338	0.173538771
0.162917569	0.162715548	0.150024185	0.160750406
0.156823808	0.163482397	0.147542806	0.162237978
0.153327387	0.1632116970.152215469	0.165583546	0.167061221
0.136935154	0.176739867	0.170429936	0.17438244	0.155047608
0.177950702	0.179269654	0.184193943	0.11827895	0.181981041
0.16308719	0.164762641	0.188031333	0.179584967	0.154700501
0.157913395	0.171925296	0.186448387	0.172251496
0.187354746	0.188053538	0.176601252	0.179274893
0.165330583	0.19736461	0.166221229	0.167298167	0.157191455
0.197496479	0.178475632	0.157421525	0.160798445	0.18049845
0.178767004	0.169297827	0.19993288	0.136134517	0.163182548
0.16915718	0.167439143	0.176422913	0.187560377	0.190592097
0.1705637110.186853182	0.173640414	0.16132402	0.156244939
0.14814899	0.163966383	0.1713488110.161700333	0.164709139
0.182035603	0.16103332	0.158842545	0.1559114820.168305932
0.186332204	0.1625411360.163843887	0.151396488	0.186149172
0.170597435	0.162231887	0.162249817	0.159508974	0.19120389
0.155784706	0.147968755	0.190865046	0.165816738
0.174929892	0.176923694	0.187075136	0.20151448	0.194463788
0.170536022	0.173918967	0.160766365	0.188798646
0.2011222380.185942933	0.188463426	0.16569245	0.181214905
0.165306472	0.1732811820.1501119980.166975236	0.16544282
0.1864813110.159343907	0.194058748	0.180207313	0.190233063
0.169734047	0.171088105	0.184508698	0.1727471160.183721515
0.196031863	0.192279025	0.176171673	0.170012967	0.16695704
0.183559542	0.196018029	0.162771701	0.176331235
0.169219335	0.127004835	0.202356761	0.182831508	0.18497715
0.160489896	0.166807234	0.176681357	0.187192391	0.16835794

0.169910436 0.188196896 0.166392772 0.151556908
0.167610526 0.137768451 0.155610397 0.187409201
0.168671458 0.132463656 0.156478935 0.153577138
0.172094902 0.179841863 0.185386656 0.179075402
0.158840969 0.14734483 0.160923555 0.15476806 0.154041777
0.133067015 0.160427525 0.155013332 0.161288876
0.134098804 0.15903645 0.133054985 0.170524533 0.135885248
0.16158953 0.16299969 0.171463128 0.164616433 0.155477506
0.19088563 0.130873328 0.152246109 0.14618082 0.158173755
0.1550363 0.173121863 0.164788838 0.146309844 0.173487187
0.14769723 0.168651791 0.1697113980.171730808 0.180138182
0.188028198 0.171236407 0.178623745 0.183734003
0.197733879 0.152051083 0.167260099 0.166439571
0.173756537 0.150020566 0.1723289110.164260022 0.17325609
0.1471162410.1653311310.188930789 0.163760908 0.174054699
0.194842543 0.185949139 0.168890219 0.177203719
0.171544446 0.174576623 0.153258676 0.166902448
0.165530144 0.191490321 0.177779063 0.177546004 0.13614956
0.157687672 0.185425167 0.17435338 0.173154039 0.177116429
0.1876114650.143163991 0.155558663 0.165884147 0.154440947
0.1711714550.168758061 0.197897864 0.173155931 0.167186074
0.169898496 0.179781596 0.176671556 0.1511888570.168701715
0.153277647 0.167866364 0.191038775 0.171269025
0.176434021 0.157932894 0.1957411910.163255557 0.163947181
0.176368698 0.196505898 0.164770625 0.180382891
0.156432824

KEGG_ETHER_LIPID_METABOLISM 0.010696161 0.06939941
0.023679344 0.017633497 0.028647802 0.004621656
0.032403993 0.036068767 0.041049748 -0.007382557
0.006014246 0.025022446 0.00309506 0.022225352 0.014959872
-0.004823898 0.029517882 0.062527192 0.017786173
0.0116802610.003630791 0.042147162 0.03277846 0.016353098
0.03762302 0.023805364 -9.98E-05 0.033840029 0.052261617

0.04884957 0.016693649 0.030607689 0.00731221 0.100653907
0.003984682 0.04577277 0.01938089 -0.001332536 0.006208719
0.024626236 0.017825449 0.037716899 0.002498313
0.022733057 0.017998036 -0.008486291 0.036662246
0.031643502 0.020056036 0.019756627 0.088620775
0.002683314 0.010038967 0.040333534 0.033509916
0.010330437 0.004170931 0.024770845 0.013726019 0.02282665
0.018138963 0.001033087 0.034715677 0.051237488
0.008060264 0.037705484 0.040197446 0.045629335
0.002179313 0.023497743 0.0374787 0.021759559 0.010742132
-0.004902021 0.004037703 0.042419644 0.016664775
0.024999649 0.022456735 0.023700008 0.027306805
0.0135096110.010335096 0.020151655 0.025281676 -7.02E-05
0.025382089 0.055649221 0.023371581 0.048010633
0.009515613 0.02938506 -0.005536553 0.026296729 0.045238046
0.002395834 0.054629445 0.003817028 0.079971615
0.062482192 0.032949351 0.028213042 0.128647971
0.008142786 -0.01824345 0.0611463540.0261181250.011369485
0.048399485 0.036627515 0.045146586 0.009546224 -
0.025873332 0.031096519 -0.013483396 0.046480324 0.035933069
0.054862877 0.001958395 0.00594458 0.026942285 0.009245644
0.0361122380.0115696160.026650995 0.02149319 -0.001406952
0.034570867 0.019443037 0.040890458 0.025031413
0.001495751 0.026061292 0.005423462 0.0044311120.031652782
0.016885822 0.003155614 0.01352947 0.025300326 -0.013071936
0.018131455 0.048306224 0.020022527 0.031413721
0.018136473 0.016780648 0.028617882 0.019517831
0.018861203 0.036282213 0.047178886 0.047292299
0.029998731 0.049923147 0.042943823 0.054795134
0.059416564 0.022522095 0.035497013 0.036289256
0.026125831 0.037730357 0.017413065 0.015174586
0.023529152 0.022004439 0.023965631 0.007139759
0.032287582 0.053295729 0.027873104 0.029459878

0.024306755	0.00419678 0.034127573	0.036266695	0.054311292
0.015752968	0.0062925 0.019943654	0.067800302	-0.002203058
0.027177035	0.04244007 0.009920914	0.00616476 0.070612511
0.01541877 0.041308548	0.04711787 0.036402185	0.044658795
0.040945499	0.02469034 0.045279607	0.049174121	-0.003123874
0.033951177-0.000740013	0.032684881	0.007623853	0.042656723
0.031629885	0.020106815	0.042687697	0.016243206
0.0311842630.016273472	0.052727495	0.010332216	0.0140767
0.024391865	-0.003014906	0.023859132	0.056679425
0.0096116510.026228126	0.030940699	0.025818756	0.000205594
0.052071322	0.041449723	0.016665598	0.045370361
0.032058982	0.018486764	0.016340527	0.024922539
0.033771755	0.007740807	0.0429119430.026536742	0.02965143
0.050222377	0.017129593	0.041694774	0.035527929
0.026380322	0.015770341	0.038126382	0.042942003
0.0411480340.022919698	0.044879749	0.045504948	0.024445874
0.006402357	0.010697302	0.017630141	-0.012954638
0.016153691	0.018643918	0.032154152	0.032410942
0.044837649	0.047818793	0.051003425	0.0311492830.01043069
0.038366668	-0.015343119	-0.004144815	0.041334574
0.046912853	0.01015667 0.043662206	-0.01032145 -0.016599715
0.03088545 0.026938391	0.027448466	0.006656934	0.016027719
0.000170996	0.0391130180.040136078	0.053255335	-0.003094236
0.015101376	0.023398594	0.010977906	0.018543263
0.040158015	0.0113439290.019360771	0.027551539	0.029012651
0.052371381	0.044157815	0.013957123	0.064977886
0.014799437	0.0321011090.022929905	0.030381909	0.028041378
0.030780322	0.068964017	0.008884648	0.015460831
0.031232332	0.034734451	0.030341918	0.013690354
0.022934607	0.04399885 -0.010597884	0.052070356	0.058662869
0.038616853	0.030191547	-0.018201188	0.0311554020.018014073
0.144578652	0.0235136110.00866944 0.032159673	0.009030024	-
0.008816552	0.021792196	0.024373695	0.02897245 0.042616919	-

0.0134110320.040080732 0.038517241 0.034188235 0.028925947
0.042780629 0.014654223 0.032600979 0.036178166
0.035390192 0.0111422620.009324707 0.070772201 0.004146144
0.060506543 0.053624391 0.013069906 0.034332143
0.047071458 0.004604335 -0.013202882 -0.002605369
0.057857608 0.009430428 0.033558869 -0.033716123 -
0.002244413
KEGG_FATTY_ACID_METABOLISM 0.15069893 0.152109692
0.164021589 0.157470325 0.1928110780.13803161 0.156877472
0.136284487 0.177653913 0.121912043 0.164678156
0.156694241 0.136822479 0.175078437 0.130524149
0.178586538 0.123092523 0.176644831 0.12687084 0.134728365
0.1311375490.1411503410.176036678 0.180150639 0.13527627
0.170554028 0.146172889 0.179231513 0.148333103
0.162215099 0.158775406 0.154479819 0.144955496
0.132212979 0.153376731 0.179708281 0.126092154
0.168419539 0.149769345 0.150491832 0.163692651
0.152104108 0.163642003 0.187865878 0.150844391
0.146492928 0.149385191 0.1174005930.187696491 0.193368524
0.172699268 0.1661167180.168788618 0.153445129 0.166291841
0.148962433 0.166697326 0.178431943 0.163562561
0.1157086790.218624719 0.126473173 0.1610001150.161872414
0.164966091 0.170435769 0.1589931120.185266093 0.153246173
0.156506817 0.179696767 0.145589259 0.1311816350.133212299
0.161539143 0.140221843 0.1389954 0.127015672 0.14773821
0.178606896 0.203883379 0.144643782 0.145557787
0.1475920110.166067454 0.184814509 0.156034563 0.136176299
0.172647451 0.165988014 0.188178463 0.188751281
0.170548103 0.181910322 0.168946444 0.125026654
0.172336296 0.137848626 0.171473703 0.162648303
0.167182714 0.179962368 0.16716908 0.178296023 0.143387585
0.179373319 0.16739058 0.150692853 0.176120624 0.129111716
0.20274867 0.129782024 0.10175481 0.155196964 0.138241083

0.158100739 0.156308402 0.169188187 0.128584456

0.154607928 0.132225694 0.134587659 0.144752007

0.137200359 0.153855982 0.165949289 0.150021546

0.132232278 0.171260351 0.185982246 0.1535447110.13832918

0.154559401 0.17028519 0.15423621 0.174744043 0.14506034

0.126614558 0.122612768 0.180629723 0.131648298

0.165753996 0.160770788 0.1611810540.193562743 0.144690424

0.146930576 0.148803691 0.122138615 0.150094736

0.142862809 0.1930991190.197880421 0.152906584 0.174362995

0.136580229 0.11551704 0.197538262 0.188349409 0.162028571

0.1775411840.14719469 0.160144293 0.168001358 0.161740682

0.190103407 0.168089469 0.173276713 0.165617717

0.178822304 0.171288888 0.180250207 0.187043531

0.183433408 0.146334249 0.144277026 0.173580395

0.169595565 0.194159644 0.15622115 0.193962236 0.151688484

0.139534532 0.134517652 0.184624668 0.151517391

0.164088132 0.161402245 0.164363199 0.156010389 0.14856753

0.150527659 0.18165398 0.161934746 0.167703358 0.147997188

0.1298196110.151541734 0.152256639 0.17116036 0.173355304

0.139823351 0.1738611610.146285491 0.142685387 0.13351163

0.1182795280.124828979 0.134647455 0.173185596 0.12916455

0.144583353 0.139623622 0.149351357 0.181606549

0.154626246 0.138698525 0.167081595 0.169434107

0.138443572 0.145217567 0.162435314 0.167967603

0.134828384 0.147174648 0.1125056530.16008972 0.153300549

0.175954136 0.151735981 0.160249898 0.167855731

0.124460667 0.180571314 0.129900952 0.154320741

0.151693759 0.153761017 0.184181032 0.150307503

0.166431752 0.140300326 0.193377473 0.17126338 0.141946515

0.167170381 0.164673312 0.183021974 0.201044204 0.16119283

0.150814524 0.1530271120.138890383 0.191662483 0.170555675

0.139654989 0.17438628 0.174457548 0.164790302 0.181661946

0.192604589 0.15868375 0.1611779550.164275756 0.183060717

0.138373447 0.194245916 0.160247936 0.143744134 0.12121194
0.189545567 0.137535871 0.120077036 0.146859194 0.16892884
0.158093259 0.152205268 0.188792606 0.1141545790.175730502
0.189787955 0.173400365 0.180258256 0.155572324
0.153712004 0.150740968 0.157795126 0.179738709
0.157138424 0.1471120680.143189725 0.163238565 0.160081909
0.127455092 0.168860572 0.160468907 0.176787925
0.1551135850.175977606 0.1611709730.160135129 0.167478161
0.156864245 0.139340801 0.183401609 0.132429628 0.18023846
0.173632026 0.175502829 0.1456115370.169617066 0.145313308
0.123813374 0.143062514 0.127733682 0.198401915
0.160812593 0.181960169 0.168277977 0.1152349860.155706694
0.207934897 0.142004181 0.169694189 0.190576613
0.137305979 0.179922633 0.182318714 0.179789623
0.161078715 0.165757456 0.13783335 0.134585759 0.139512977
0.153441897 0.160427326 0.18412699 0.154899676 0.112956299
0.2279411660.148602591 0.125405755 0.129269022 0.128718856
0.137440582 0.139618776 0.163095782 0.164434005 0.1176351
0.136235792 0.160691493 0.15690433

KEGG_FC_EPSILON_RI_SIGNALING_PATHWAY 0.052191547
0.074997545 0.095084913 0.057572074 0.104161995
0.027773484 0.035468414 0.1185314350.047929689 0.097032484
0.03380046 0.109668272 0.012469656 0.032589914 0.108721471
0.077792843 0.027378531 0.063504 0.04202514 0.027746471
0.024937456 0.0849611170.090126992 0.033439855 0.14043487
0.09410577 0.050252962 0.065041641 0.051732755 0.089212944
0.035039377 0.08892242 0.031821074 0.03581317 0.093300183
0.081696274 0.022995968 0.048596452 0.083822506
0.004122507 0.068671891 0.067064049 0.084402189
0.0499011530.055071909 0.056098245 0.059733126 0.059044659
0.105164023 0.029494019 0.079401052 0.055294649
0.080261462 0.042631418 0.081204301 0.098667352
0.1001153290.073756209 0.062322434 0.034741257 0.053502247

0.062291758 0.080550034 0.109121042 0.038546032

0.035824627 0.048397093 0.082947444 0.105839093 0.06936595

0.07104314 0.038513597 0.053332722 0.025847569 0.061741671

0.133191337 0.055519626 0.107666855 0.060149621

0.076940549 0.107506445 0.050743755 0.037767044 0.06443522

0.085657818 0.091330867 0.127446012 0.079854463 0.05981367

0.060688316 0.064200585 0.072293756 0.071928252

0.0313055110.099019818 0.08381077 0.070260205 0.070009335

0.093241082 0.122720078 0.0681153 0.047761734 0.046939662

0.067733567 0.052033012 0.097872587 0.040521629

0.108207677 0.088794373 0.098085131 0.061067616

0.109412109 0.013906783 0.041093879 0.040551451 0.11509791

0.095622854 0.064933921 0.098360091 0.076397799

0.0661142330.055494326 0.07803996 0.096487584 0.094137773

0.0411805510.078599386 0.066913377 0.043793104 0.08720811

0.044290196 0.037656856 0.053731475 0.097816241

0.079901234 0.049748958 0.074177423 0.096360701

0.076107022 0.062419665 0.046967923 0.081366068

0.1118471350.050427666 0.074710887 0.083821799 0.112381517

0.062383004 0.0589511250.089871693 0.094007289 0.077106184

0.068166569 0.022047059 0.09375341 0.066739924 0.051567961

0.10996887 0.083483852 0.1177856790.078505706 0.074119631

0.0490252110.106173207 0.105170459 0.044264957 0.028892149

0.08303966 0.050252456 0.046659857 0.069331412 0.095036674

0.040519449 0.103204567 0.1197921840.1109120610.0978679

0.093598373 0.096244042 0.065125765 0.107051461 0.05293426

0.077208538 0.084279705 0.10658871 0.027240871 0.04248955

0.10786365 0.049338263 0.072325281 0.1150028270.101764294

0.131330268 0.1139714860.1110490530.062670467 0.133413813

0.090806318 0.1176756870.137925297 0.125166154 0.054299113

0.060588712 0.072307053 0.080801081 0.026619388

0.055452616 0.0741165150.077239781 0.065635727 0.089936248

0.06980172 0.089397282 0.0235334110.090741431 0.086955881

0.049915267 0.1110556980.11702193 0.092354793 0.064361664
0.122488645 0.049322552 0.085185126 0.089945853
0.034321834 0.07357146 0.057603636 0.02591824 0.073643477
0.089046201 0.104092992 0.077782155 0.065895626
0.1148653690.078467005 0.061685458 0.102184965 0.074723338
0.07778503 0.097093536 0.080480052 0.030976176 0.068725695
0.079058596 0.143777435 0.023473092 0.047784325
0.060950437 0.068690689 0.086682641 0.128858927
0.1155124490.068672354 0.072045892 0.052490389 0.081082896
0.067751619 0.068851561 0.0911763070.004086489 0.053414719
0.066176674 0.074064951 0.098205618 0.068660551
0.095469281 0.096304802 0.0308761130.056810472 0.09808451
0.103220107 0.081385733 0.095303922 0.058050827
0.007631985 0.086991955 0.091470335 0.0411068120.054220343
0.0511617770.071679589 0.041762867 0.078123513 0.072675559
0.057573664 0.088704107 0.121705363 0.149021618
0.132065993 0.097388131 0.096602429 0.059828145
0.031515768 0.102887806 0.1155371060.037794538 0.110918614
0.079799765 0.065078039 0.078641845 0.062606268
0.1186480970.045392124 0.056078567 0.086802508 0.081757055
0.0621138140.069255201 0.090105324 0.089284563 0.067704061
0.078362048 0.12214357 0.087150541 0.08763866 0.063177135
0.041470475 0.080956009 0.032993089 0.124426251
0.064714394 0.059594817 0.049722461 0.1133616350.085881856
0.080865102 0.093536138 0.089342655 0.126924199
0.092961617 0.100094397 0.1140909540.103471907 0.130945287
0.100142369 0.079277829 0.108750476 0.0472611080.066749428
0.126202386 0.029651596 0.061693438 0.04558165 0.121557154
0.075555533 0.058771515 0.096274552 0.078761878
0.044713362 0.043848347 0.022247131
KEGG_FC_GAMMA_R_MEDIATED_PHAGOCYTOSIS 0.176627851
0.197430737 0.201479551 0.169471036 0.184476336 0.15812712
0.169661858 0.18129223 0.186653197 0.194709456 0.145659032

0.218210455 0.150934495 0.16440536 0.2113106540.19110904
0.149798446 0.194279097 0.166077987 0.1511410620.153646869
0.243293138 0.192030954 0.166152339 0.220125849
0.212059608 0.175877498 0.213834201 0.191872624
0.216039353 0.136120436 0.198195169 0.183001223
0.160944005 0.171510593 0.160633366 0.140780935
0.1611890780.168510212 0.129071234 0.206908573 0.187570969
0.16630201 0.187663267 0.189873161 0.18462833 0.192545146
0.167905187 0.199444465 0.158621014 0.223968436
0.206174017 0.194945996 0.159422959 0.20735501 0.210705547
0.212345338 0.206599351 0.160627169 0.154566057
0.175921581 0.199173268 0.175602931 0.207950642
0.175533521 0.154869613 0.166276893 0.191610873
0.177353875 0.175610456 0.225772613 0.154823031
0.199894387 0.161082877 0.198825963 0.257309252
0.172910666 0.19179217 0.198974069 0.17582336 0.186287689
0.152429333 0.176908288 0.189880204 0.205049881
0.1791145220.210215177 0.208494741 0.186271019 0.195787417
0.15901698 0.19459596 0.184353178 0.167365639 0.194596511
0.2006211420.196966487 0.174285917 0.240042808 0.224367645
0.213864738 0.191471814 0.177625705 0.207838637
0.167899725 0.205007584 0.162190604 0.200875859
0.170908862 0.188700796 0.17627319 0.195108668 0.138575017
0.175555912 0.175179592 0.213150828 0.186995123
0.187215304 0.197637266 0.204360527 0.220077501
0.181840961 0.213872204 0.22254976 0.208693847 0.15932334
0.1761171240.167037949 0.172024709 0.233647209 0.196181343
0.172055585 0.173980051 0.210765684 0.207055808 0.17514244
0.219791285 0.198151816 0.192185266 0.21923721 0.18050177
0.2242113370.208584145 0.191728305 0.210476531 0.234438856
0.2115868530.16680016 0.182908792 0.188240163 0.224600919
0.204064749 0.227794321 0.160749201 0.23010025 0.206825723
0.21486537 0.206949714 0.20177707 0.248476469 0.205779555

0.194364204 0.187482279 0.199835715 0.196293364
0.1872401190.162073365 0.190025535 0.18494804 0.169998679
0.208014485 0.198630413 0.169300073 0.196437949
0.201766483 0.230675516 0.215935913 0.191323605
0.203419201 0.212306813 0.2290411150.170344326 0.193119455
0.19358888 0.214870869 0.178564268 0.192613227 0.239921052
0.180393892 0.188953393 0.225865358 0.231091684 0.21959064
0.242975409 0.210636847 0.20743429 0.22237191 0.22141964
0.237479443 0.219206266 0.238931728 0.175504889
0.202645238 0.194404146 0.220257018 0.170993491
0.194328289 0.186207309 0.223022574 0.213137541
0.213629051 0.185502999 0.217321402 0.179405909
0.2249611150.221578207 0.209295366 0.22938584 0.247590783
0.204378106 0.223694007 0.2216021160.182274225 0.185843798
0.208701703 0.165717937 0.225855659 0.185066557
0.141074328 0.232694165 0.187642453 0.246982997
0.1792901130.18696307 0.219361548 0.197866262 0.200443959
0.238834968 0.209294037 0.212634749 0.208426258
0.209376987 0.174165433 0.183082969 0.215460735
0.238240234 0.140850131 0.187101413 0.178494198
0.200996988 0.2181167160.232422086 0.220606627 0.179894075
0.174761308 0.185919443 0.175913427 0.205067446
0.181579707 0.197059563 0.139106638 0.183228254
0.168794124 0.204844278 0.205983862 0.217166266
0.207416802 0.203783535 0.165970467 0.187809927
0.236238049 0.22271974 0.2117827410.15424634 0.190181223
0.160165882 0.208677318 0.200066064 0.1848211380.208509802
0.181754186 0.203932145 0.181437689 0.197757757
0.213308086 0.1900925 0.233222281 0.247366518 0.241919338
0.227920847 0.2111394 0.24323461 0.178529906 0.164929207
0.200816716 0.227852322 0.1660041170.216340757 0.21084536
0.187960628 0.180402235 0.199668804 0.231373284 0.17999404
0.201731376 0.226524623 0.226893493 0.204087638

0.216189912	0.206589172	0.221859803	0.187280795
0.164443022	0.203124728	0.185048272	0.218135064
0.203258019	0.17277463 0.195781517	0.184279417	0.207398928
0.196633914	0.197605887	0.173632697	0.185542852
0.177047035	0.208703481	0.188203023	0.206531639
0.213133617	0.228578445	0.207724173	0.205086678
0.214053214	0.226076194	0.2115269530.194015832	0.21465359
0.1769811630.188304109	0.216635034	0.165026339	0.19022095
0.20747551 0.228756479	0.188158273	0.196324371	0.206862576
0.218904088	0.182644846	0.183781382	0.147937189
KEGG_FOCAL_ADHESION0.160588635	0.225389412	0.191423013
0.150061868	0.1724943 0.144475443	0.136820238	0.156173276
0.171220937	0.156562478	0.123272619	0.14064519 0.175605185
0.143870178	0.198333921	0.156881287	0.165650584
0.143334443	0.140180643	0.178625047	0.156880926
0.154948899	0.138810165	0.17569082 0.192789255	0.157220844
0.160055546	0.148704413	0.159010016	0.16669557 0.129603782
0.152080044	0.19170008 0.182333642	0.172216517	0.126827315
0.163803357	0.1182421750.135589503	0.103560383	0.198293719
0.151257084	0.144363804	0.143873285	0.1694112290.156587239
0.18885293 0.167878143	0.129946777	0.143969105	0.212112976
0.157709638	0.147956181	0.127916269	0.152961842
0.207881351	0.162738047	0.181227633	0.136824277
0.186618422	0.141453506	0.186861492	0.1587111370.17304963
0.156675757	0.131382166	0.1147082920.160532314	0.17741403
0.190452329	0.204315592	0.125954826	0.194108035
0.178560062	0.175639712	0.2110627810.175329229	0.187004681
0.205274491	0.187488098	0.149592906	0.120987341
0.123721416	0.162264369	0.137289233	0.144805604
0.173978374	0.18916508 0.181290684	0.191577015	0.126622655
0.149097413	0.164404781	0.137145122	0.19313053 0.16899832
0.157455758	0.150143645	0.151485922	0.139795397
0.178423153	0.162171412	0.15396618 0.181004077	0.146749002

0.2003881140.1130272370.179395438 0.157917068 0.170470356
0.120941394 0.183260659 0.143946313 0.182389274
0.159270486 0.200108891 0.153733644 0.147309739
0.172913041 0.1816932 0.140523882 0.176031538 0.172629596
0.207659569 0.190403021 0.1160630950.165424518 0.171670476
0.148005478 0.18074941 0.189135288 0.14199535 0.137814813
0.183530849 0.193462804 0.162268623 0.208804528
0.188444442 0.189053207 0.23355774 0.150070123 0.212977842
0.173224287 0.1711582610.156038775 0.219518381 0.194104996
0.13708969 0.15015355 0.149513447 0.175103936 0.150419034
0.163306053 0.137931687 0.20001239 0.181837824 0.219819134
0.176465762 0.173463039 0.1880658 0.154867882 0.161311678
0.151761368 0.188992816 0.197000951 0.1166479110.163881345
0.158430822 0.177240583 0.158782776 0.179707853
0.143441656 0.153140074 0.132744229 0.1811255170.190861027
0.165983244 0.18633723 0.132685663 0.1891186190.180862471
0.151202434 0.152274621 0.178682312 0.1641216110.17333678
0.17002497 0.234632025 0.164636905 0.174233559 0.206196743
0.198639937 0.199698588 0.183877224 0.188391237
0.221377568 0.159755556 0.199609714 0.215240985
0.178139282 0.223209719 0.156663392 0.208054404
0.160960851 0.173935834 0.142251818 0.171232915
0.191609725 0.20509356 0.149445161 0.201773915 0.155685944
0.192474206 0.184376407 0.196621001 0.225772493 0.1849707
0.213339178 0.242537575 0.171541635 0.209722678
0.181069444 0.187787786 0.167197391 0.218508685
0.1637891140.198133741 0.157936669 0.131951393 0.253147237
0.152219466 0.175388224 0.157004848 0.150490396
0.208937353 0.192120253 0.166008464 0.199089734 0.20620012
0.180465944 0.160325345 0.189683599 0.145060948
0.170379231 0.20838391 0.196913369 0.1199953460.124027386
0.132090142 0.163546393 0.19277901 0.201036675 0.187232363
0.1199954680.143931344 0.179073262 0.161062258 0.189376104

0.147598209	0.195130782	0.156592482	0.182960266
0.164229236	0.175822477	0.133282287	0.180094218
0.1187058530.156627224	0.183766948	0.164386357	0.170484339
0.142914338	0.220002734	0.128636789	0.1780521160.154482256
0.190513473	0.146626813	0.199181673	0.181512968
0.129620624	0.18886131 0.193153821	0.18673995 0.184536484
0.145233662	0.204801817	0.202981871	0.200976037	0.21641683
0.187612828	0.205888019	0.1362991170.153562856	0.189448665
0.20466278 0.164139596	0.206975574	0.17694931 0.164946073
0.156255809	0.162249556	0.21805532 0.146365504	0.169781642
0.172012221	0.188518702	0.157878891	0.182420184
0.188265778	0.200334161	0.185919693	0.160842374
0.158169892	0.172474461	0.23680821 0.177366814	0.153584567
0.145550914	0.183519401	0.191039822	0.1215611160.16166064
0.180402896	0.128497809	0.160406012	0.147702322
0.137714543	0.167275333	0.183641554	0.170739734
0.200849796	0.163006201	0.189687255	0.224600838
0.185420969	0.168191061	0.167496547	0.187779919
0.127490194	0.212976754	0.181866785	0.1891174770.226972139
0.1841828110.157195773	0.165781383	0.156780056	0.210151727
0.156138374	0.169137276	0.13240034
KEGG_FOLATE_BIOSYNTHESIS	0.132984284	0.140482096
0.167926488	0.26110734 0.212023499	0.164401484	0.204371668
0.202668803	0.224756109	0.20261363 0.3076366	0.173175453
0.252755016	0.219383807	0.220069157	0.160362201	0.2567291
0.153712634	0.214292577	0.166239878	0.1601157460.121870873
0.164396436	0.159618231	0.175377505	0.142784385
0.262824744	0.162535876	0.177633533	0.132893032
0.189864385	0.1176302750.1113877430.1844911240.322787303
0.2492014110.270678858	0.232657289	0.210604523	0.25910761
0.167825978	0.1183993220.251636569	0.219088128	0.166192376
0.149253134	0.165581591	0.294023022	0.1991117960.24993928
0.170903618	0.170761328	0.157940477	0.20320157 0.128263475

0.20451018 0.246873278 0.242412253 0.335566176 0.161206005
0.183554737 0.200553601 0.169615146 0.21952225 0.192169583
0.2261184180.220127635 0.242884695 0.208342725 0.19569407
0.168739923 0.228379452 0.150510549 0.156663786
0.138447598 0.146579233 0.134049466 0.171022647
0.1132941940.221323151 0.254356429 0.187229958 0.206491556
0.1661103530.156392558 0.222584872 0.165161806 0.18906033
0.225217486 0.138492133 0.329301555 0.161235049
0.190724625 0.125936745 0.122345632 0.242918039
0.239910587 0.243248014 0.206271092 0.320499125
0.131094469 0.1177742280.313957918 0.160779695 0.176813891
0.1907101160.238318398 0.274357149 0.261067165 0.192081421
0.2090711740.154909574 0.189199472 0.20280998 0.326768727
0.27984616 0.281440151 0.261993236 0.137657513 0.137139819
0.145961944 0.167386878 0.164318694 0.164609696
0.2279116660.161494817 0.300227703 0.20742668 0.229041632
0.121283363 0.192799494 0.176082245 0.329386349
0.181785036 0.16341922 0.179099083 0.1729696110.127528072
0.1774112840.170012005 0.173077864 0.129015273 0.250347382
0.180005578 0.242171337 0.156127421 0.150734326 0.23540126
0.2254332 0.2117082790.097867052 0.246308576 0.128055302
0.225120353 0.219451632 0.191061032 0.1157418230.193200913
0.198306706 0.1711159010.334978371 0.153014313 0.326292666
0.29243559 0.230842771 0.08929783 0.272246622 0.183355067
0.102180933 0.277678071 0.190138271 0.286728923
0.196253618 0.154333977 0.185687585 0.20905169 0.185583793
0.226886192 0.238635345 0.208147503 0.155524371
0.193261898 0.202445851 0.1426774110.1167917580.297733328
0.179377845 0.185078754 0.170722866 0.077133554
0.164336498 0.096484569 0.145069749 0.145558971
0.154829682 0.129309522 0.216891336 0.093486407
0.174995051 0.190192049 0.268526914 0.235046546 0.12443341
0.295851955 0.120477423 0.207247607 0.208163873

0.195341072 0.144027146 0.148169927 0.219941502
0.222309673 0.26517653 0.250184749 0.144365078 0.109476177
0.097482095 0.243599237 0.1683614 0.138869447 0.19778012
0.264403382 0.238723589 0.181393371 0.159205853 0.13389892
0.1744111140.148481665 0.228647489 0.174540688 0.154119621
0.130588614 0.150955236 0.239485007 0.1217521180.274446624
0.284701068 0.19891144 0.173357424 0.167702486 0.202177669
0.150964565 0.168293032 0.208463103 0.165917855
0.148574206 0.166440164 0.227820643 0.251664347
0.202314732 0.122103956 0.0603115890.128396887 0.303633321
0.238333869 0.161583522 0.214208589 0.213696456
0.274755787 0.161222654 0.210651714 0.166652786
0.242142869 0.14433464 0.191757909 0.198546454 0.210816542
0.204840548 0.181017715 0.169753233 0.217285557
0.215404619 0.096698262 0.259965555 0.2208296 0.13272126
0.198544516 0.199868443 0.185494802 0.099596402
0.213974346 0.120683405 0.125954763 0.240504912
0.296263422 0.15952048 0.167664446 0.157094466 0.16034357
0.179803678 0.1827629 0.073484352 0.292343389 0.312668841
0.215126012 0.259379026 0.272364677 0.17165007 0.11739917
0.188469587 0.220324919 0.163984894 0.11369034 0.254313827
0.1989521170.200265995 0.1164435130.294139919 0.29755212
0.107021731 0.227318266 0.169599507 0.204743479
0.203455677 0.129895158 0.058298543 0.237942062 0.21993095
0.221390778 0.100657317 0.194977377 0.210159389
0.156286372 0.248210278 0.247322718 0.083732109
0.175463023 0.244586104 0.199197583 0.1827115350.18129281
0.262966861 0.152958268 0.192184566 0.1165323480.177680428
0.189094529 0.151446513 0.154079524 0.256499703
0.165421784 0.177793889 0.20410935 0.085003839 0.150373739
0.278170401 0.274920835 0.273098383 0.191292424
0.133614854 0.247749956 0.291967208

KEGG_FRUCTOSE_AND_MANNOSE_METABOLISM 0.155473835

0.1633143 0.174816006 0.203261468 0.196870399 0.168807866

0.203908475 0.16427453 0.168313836 0.13366173 0.18164167

0.183606526 0.150947266 0.210967553 0.16670341 0.170837029

0.167692269 0.199350916 0.204400433 0.152368669 0.15637114

0.201775327 0.175243792 0.207732046 0.199340851

0.188801665 0.201560354 0.188921585 0.163031259

0.195137226 0.129923363 0.179295919 0.174130535

0.174482063 0.163325095 0.19922677 0.122790451 0.176552204

0.198565665 0.140609882 0.189660019 0.1958841160.174870034

0.191440946 0.195146901 0.181577457 0.179728976

0.1689112780.250288778 0.216823189 0.172862021 0.168665984

0.195319904 0.169341929 0.194992027 0.199518013

0.158222244 0.158064281 0.188495239 0.147224287

0.193238198 0.192390644 0.159046621 0.220952234 0.18321861

0.167835129 0.215042402 0.216926215 0.202257545

0.1712301140.196973447 0.142227345 0.154680475 0.178774399

0.177180813 0.179808083 0.159814073 0.192009431

0.178061423 0.188174876 0.188615197 0.173727395

0.153849278 0.162647607 0.159225438 0.19033349 0.157054421

0.127305974 0.160550062 0.186003375 0.2110921880.187824353

0.170594178 0.186172234 0.21319739 0.180258791 0.184121232

0.174730749 0.259070087 0.23217016 0.195102556 0.204877408

0.191932577 0.195104237 0.181836745 0.184251343

0.182041572 0.220369727 0.204229495 0.167252449

0.221086098 0.16105281 0.177918377 0.181605316 0.22214052

0.173640872 0.179418061 0.16007893 0.158280239 0.165676289

0.164012673 0.176560926 0.165742969 0.180822134 0.18087726

0.197555074 0.209001677 0.144337714 0.199185676

0.179299265 0.2112070060.182745531 0.180747233 0.195256639

0.180601082 0.198513192 0.174880786 0.16966005 0.159037459

0.181412253 0.189830199 0.177054437 0.208673218

0.155605256 0.188950055 0.184143504 0.1695937 0.161338118

0.176149624 0.167206555 0.156520565 0.16480869 0.175299798

0.178722979 0.220042901 0.18037316 0.188814974 0.216346406
0.180437906 0.193344295 0.172763164 0.18863594 0.215288311
0.212884528 0.196029339 0.178878376 0.159007719
0.207929207 0.19624719 0.209606147 0.225590017 0.170651373
0.163314317 0.202624549 0.202941206 0.2117514440.216778752
0.161263765 0.183305736 0.189774358 0.19106783 0.192797554
0.213893322 0.144406218 0.189497573 0.146978838
0.189273178 0.1431174880.163808081 0.179561685 0.179214315
0.17751279 0.210522969 0.198743007 0.188237854 0.165045142
0.208392213 0.192279862 0.175385856 0.20131789 0.183836907
0.171022984 0.193103926 0.181339347 0.168130492
0.190505746 0.160161274 0.19715367 0.184158861 0.17495298
0.177830402 0.176422385 0.17362055 0.161832487 0.211994088
0.198059643 0.195150715 0.197325309 0.182360481
0.197763077 0.169419451 0.205305518 0.2006221130.155040788
0.172327154 0.154608768 0.182270099 0.1791811850.183086301
0.166467741 0.221320065 0.187810785 0.208014296 0.16522972
0.187155077 0.2116600070.213088 0.190433513 0.203290418
0.181768501 0.209038965 0.175702419 0.162486171
0.178412339 0.185531725 0.173459302 0.187758976
0.245930585 0.160764717 0.184379176 0.176125619
0.159765052 0.206665887 0.198006885 0.173095357
0.155316336 0.177796152 0.182125759 0.191450647
0.186625715 0.182853644 0.1694505110.223979033 0.163571836
0.222586006 0.184589906 0.198826466 0.174161975
0.178585453 0.189721427 0.187409121 0.209513481
0.140689122 0.170186015 0.171366347 0.195859297
0.163667284 0.207069171 0.188214627 0.188348258
0.141096088 0.213940786 0.179061976 0.203001602
0.205761402 0.18666371 0.198446713 0.188386046 0.218093977
0.190303661 0.190663297 0.164683635 0.173258302
0.179874457 0.196541001 0.186849091 0.20809379 0.198250902
0.215952635 0.210069587 0.195266075 0.203436947

0.198650449 0.149684478 0.213081709 0.156899261
0.169325355 0.219643576 0.212350366 0.196173222
0.197442736 0.180417334 0.169613662 0.203603198
0.158385319 0.154894138 0.201410678 0.217208601
0.196839543 0.163523177 0.178868784 0.204922109
0.148770894 0.162791882 0.201043584 0.181062865
0.164738837 0.203518537 0.192997692 0.189548313
0.218493499 0.203793792 0.183206636 0.194948096
0.195173934 0.205829335 0.202465377 0.219713824
0.163331519 0.250944549 0.1917381110.184168087 0.197761998
0.148181459 0.185066723 0.189237972 0.200036865
0.159623331 0.175176126 0.151025063 0.181848862
0.188862021
KEGG_GALACTOSE_METABOLISM 0.1190028820.131551469
0.135412979 0.146338449 0.134926165 0.126235638
0.132234519 0.136372482 0.145907599 0.085273836
0.144018248 0.152842331 0.1209223110.150289005 0.147103846
0.1150402490.108309153 0.136268054 0.130347582 0.13638814
0.108743783 0.174747052 0.149908744 0.165997965
0.149993107 0.151406194 0.134810074 0.1391106330.147234641
0.145896309 0.1193596190.142231638 0.159916896 0.13156939
0.103443583 0.165769136 0.081617102 0.127227972
0.145089995 0.085206736 0.145334013 0.16294355 0.132350757
0.148032326 0.1554304 0.144950033 0.166207607 0.101161525
0.1840921130.168979045 0.160646825 0.120328238 0.138668316
0.138823021 0.142798955 0.14004983 0.134679399 0.129254879
0.143936193 0.129940778 0.13610218 0.152692402 0.122874143
0.1761144040.130580526 0.151654523 0.189333301 0.152331351
0.1462861140.1311189150.15607874 0.1151228510.12999892 0.075316509
0.136578183 0.15643402 0.121302017 0.145821033 0.117835932
0.142579521 0.160172462 0.1190182810.103290319 0.14711045
0.135184266 0.138000257 0.150674053 0.108459782
0.1152142080.1189437410.162052352 0.161309123 0.166606056

0.122947475 0.139176094 0.144673482 0.152800883
0.142725443 0.184508719 0.19117603 0.140588059 0.142543117
0.1141348840.136459526 0.1176524310.142855406 0.144967644
0.168800767 0.155101474 0.12967091 0.162794514 0.130255692
0.133713816 0.141646339 0.154599285 0.139929367 0.15365807
0.148006236 0.1174011780.133271299 0.135631751 0.130552109
0.153540603 0.141098556 0.15424346 0.160813696 0.143645453
0.105409632 0.134752494 0.143324072 0.155469034
0.125020003 0.142201703 0.152827225 0.130918045
0.143196751 0.13404699 0.131772151 0.141095941 0.132298825
0.143980159 0.139852854 0.16945671 0.108013649 0.127814405
0.152880299 0.146791578 0.125813766 0.120168602
0.148759617 0.1158461480.13495152 0.169758699 0.169495999
0.145305336 0.174080106 0.153825109 0.140443048
0.167629645 0.138061572 0.13244603 0.1211292840.169188255
0.156408024 0.149646758 0.1179691140.13829264 0.134458684
0.127209218 0.140470726 0.160135707 0.148787153
0.139353448 0.15181979 0.153327266 0.154655715 0.161202821
0.1124959950.16228382 0.145732482 0.146244524 0.122656412
0.146699576 0.135226366 0.155927362 0.125210362 0.15414821
0.13451372 0.107517554 0.1152571030.135064718 0.13365624
0.156165059 0.156144713 0.1149189040.162514602 0.168038766
0.148755924 0.149674085 0.159315537 0.157866645 0.15828941
0.144282849 0.1441193160.122336903 0.160528081 0.116903872
0.122969054 0.124233175 0.139984187 0.152158859
0.1156040470.131025392 0.13079852 0.152995164 0.138965497
0.155806363 0.166159003 0.153564194 0.146291849
0.147227665 0.16611338 0.149029323 0.129893342 0.135077739
0.102510561 0.135275645 0.139603691 0.134096358 0.1325609
0.17780763 0.152387886 0.152070428 0.129992085 0.142328065
0.183408977 0.143915287 0.15592964 0.158582754 0.158598921
0.125395863 0.139752435 0.121342555 0.125607823
0.145501665 0.1372121140.127714747 0.165062958 0.132588248

0.141210389 0.136634773 0.1186909790.155477336 0.159976261
0.141606066 0.149559426 0.147991204 0.135309067 0.13022941
0.127362457 0.146814196 0.13593165 0.165905288 0.132797897
0.168549332 0.148540219 0.152882759 0.147378922
0.131815052 0.1189007540.144610082 0.165497458 0.122357162
0.13370783 0.130136547 0.136729061 0.122574356 0.165908279
0.139335826 0.156849536 0.139122563 0.14566965 0.13531207
0.175616605 0.167770402 0.141434219 0.1511399710.161171976
0.15513941 0.132488287 0.13458021 0.134609475 0.117616564
0.153442673 0.149828308 0.130905351 0.158084929
0.157321595 0.155870782 0.143386283 0.1384221190.140082857
0.140623196 0.1169565460.146999376 0.13050073 0.135141049
0.169395478 0.151207175 0.134225157 0.154640212
0.127181628 0.1144318690.15823124 0.142617786 0.125552664
0.147233732 0.16090455 0.13556281 0.15080409 0.138629276
0.165524424 0.1511387050.125643185 0.163142165 0.151937897
0.13170154 0.143105416 0.150808762 0.143553926 0.165125488
0.162336648 0.147410233 0.156410278 0.160826399
0.1410421170.148636278 0.169435646 0.161940464 0.158798194
0.140627584 0.139625349 0.14616871 0.133661406 0.13499038
0.145309961 0.149621529 0.120072944 0.1425099 0.126305144
0.142583137 0.15897476

KEGG_GAP_JUNCTION 0.161320486 0.18318792 0.162739935
0.137163375 0.149430487 0.133262921 0.149374852
0.147915235 0.139610678 0.143955223 0.14231437 0.152745767
0.170418023 0.160574954 0.166306883 0.14553676 0.138812671
0.145148735 0.16091743 0.149407081 0.169915255 0.13719744
0.124008829 0.173039428 0.159189127 0.1442332 0.133365845
0.14952045 0.153591542 0.148128034 0.122971849 0.16329987
0.158867276 0.164749683 0.169838616 0.129177873
0.168982103 0.1180050470.129054787 0.132214879 0.161603934
0.155001384 0.151471056 0.148416324 0.157057199 0.15384713
0.170674592 0.137217966 0.127938445 0.153914408

0.180827976 0.1302668 0.151948519 0.146664938 0.140484182
0.175650628 0.145225451 0.153939042 0.142981249
0.170088812 0.134494585 0.163217421 0.150718522
0.159385268 0.174302547 0.144735052 0.126973279
0.158778462 0.155708875 0.148493237 0.178775008
0.145794436 0.177616056 0.14643014 0.152188362 0.182711834
0.154496689 0.156796933 0.167686782 0.172973867 0.13787401
0.136952254 0.149232134 0.150621061 0.137976749
0.1379314110.149865562 0.162344988 0.175591675 0.164091166
0.124823969 0.150583782 0.157960745 0.13999502 0.157098335
0.166384768 0.143361987 0.147286988 0.140032692
0.134121389 0.171947553 0.1613246 0.135178313 0.151056326
0.123183342 0.164979247 0.164065622 0.148016343
0.126199531 0.155094735 0.1417181120.155094175 0.147450049
0.146817274 0.159387671 0.162181907 0.151750178
0.140223534 0.157459578 0.161504825 0.131605593
0.167507221 0.14088309 0.1843265 0.159743984 0.10321667
0.14262589 0.151767061 0.145730132 0.147420174 0.152929489
0.140001721 0.1281100140.163841098 0.164264218 0.159328159
0.165840092 0.159577879 0.180670332 0.195786281
0.145580024 0.186143336 0.130584454 0.151543688
0.129021636 0.173449603 0.17989673 0.136395357 0.129410204
0.156648571 0.162321696 0.130161463 0.174085935
0.143324712 0.172401682 0.157512594 0.191280576
0.1550811790.17281688 0.150708216 0.147927669 0.146702114
0.159671968 0.152718857 0.156576923 0.13139879 0.190660812
0.147438876 0.157198457 0.154062778 0.171403328
0.140720182 0.133171776 0.1112223420.138781076 0.160435035
0.161614725 0.159587195 0.130213646 0.190463723
0.149600455 0.149627872 0.132134549 0.1699051110.160699804
0.147583464 0.17215763 0.182986142 0.153249268 0.154874069
0.17388273 0.167512513 0.18354144 0.157492673 0.163046138
0.186590326 0.144598699 0.16551729 0.175478141 0.149272287

0.181607912 0.1397688110.158050638 0.146301296 0.144225961
0.153768807 0.169847907 0.162853021 0.166796274 0.15838589
0.178056052 0.1511143660.163931094 0.1627892 0.16572431
0.16956167 0.175329092 0.170091478 0.208577334 0.161302186
0.180699741 0.154132287 0.153002439 0.159684438
0.1851131760.154519071 0.176101286 0.128933147 0.149130793
0.19555148 0.152870505 0.144991755 0.157349707 0.153034162
0.182487846 0.160445365 0.176596818 0.174838265
0.171468697 0.157468927 0.144995423 0.149088741 0.15605765
0.140918884 0.166912978 0.158466384 0.124062603 0.15340032
0.134276739 0.1491183680.165734679 0.158892806 0.171494556
0.130686451 0.147570845 0.175429436 0.142760946
0.171578577 0.123887908 0.157965131 0.151905048
0.154042908 0.135756044 0.151668882 0.136929651
0.1500114280.1122979290.16258922 0.16147603 0.145237736
0.158923288 0.137588107 0.198781273 0.1146455430.148326408
0.144552882 0.156771604 0.147445409 0.176501312
0.145686935 0.139575509 0.173247539 0.162168538
0.156931353 0.154176262 0.1370767 0.171327494 0.165080644
0.166095085 0.171323182 0.153829469 0.167857368
0.124794517 0.145797688 0.16296389 0.181774914 0.152078381
0.172286774 0.164590941 0.142888658 0.158912981
0.138957954 0.181239988 0.140853042 0.159101844
0.1591104360.171560017 0.1769142 0.153140996 0.169160904
0.175920954 0.157039698 0.158710735 0.147927801
0.169331013 0.202147065 0.146622862 0.147070766
0.149699596 0.166430738 0.167209387 0.133189036
0.148405478 0.164051594 0.125332434 0.127408656
0.140839614 0.131505288 0.146349579 0.159326076
0.185010264 0.155614632 0.146357139 0.15556556 0.174118273
0.169834779 0.157285587 0.170033663 0.17437983 0.134682452
0.181903203 0.162809359 0.17007295 0.198984996 0.150440097
0.138108319 0.17328547 0.15655703 0.172985465 0.168830461

0.14970973 0.158176329
KEGG_GLIOMA 0.135365175 0.1801156640.181953472 0.140795189
0.148086249 0.154287401 0.161849151 0.160049714
0.161545312 0.157650536 0.145605885 0.151905625
0.164710446 0.1443111170.177894756 0.1631133240.152646286
0.131612096 0.15375854 0.1777114420.180691346 0.168587553
0.146958029 0.168751761 0.198644296 0.152767298
0.140820082 0.167960537 0.182891907 0.172660139
0.141656638 0.175297475 0.177204014 0.160924959
0.159743538 0.131666292 0.166970204 0.108356251
0.138579955 0.154294658 0.1744211980.1447117 0.136055441
0.132538318 0.173529403 0.1682115060.168949164 0.15224672
0.158652814 0.157349742 0.175107936 0.147102161
0.171657682 0.159568346 0.149664704 0.185363089
0.154042198 0.17478029 0.125147431 0.161284208 0.146760279
0.178673027 0.16190784 0.170995528 0.161038038 0.136855235
0.1136068810.151026182 0.157316338 0.173615206 0.187383292
0.150004435 0.174375607 0.153400943 0.157858521
0.199689518 0.173330424 0.184274368 0.171407605
0.169512685 0.140715716 0.124067378 0.1571178 0.15998615
0.162354017 0.141778032 0.188968542 0.166875779
0.177179221 0.168928154 0.13085619 0.144673103 0.150565982
0.123181547 0.17392334 0.173854298 0.157610539 0.158132777
0.160804317 0.154846009 0.171965726 0.183404454
0.154913578 0.172991304 0.14752182 0.158023475 0.145392962
0.158425298 0.160746616 0.156590871 0.1186581210.163884961
0.147206674 0.16127259 0.155947531 0.176073081 0.161631946
0.12815183 0.171035882 0.185218477 0.165476247 0.163710883
0.165827368 0.201720366 0.183507072 0.13025656 0.162504567
0.168733819 0.138332859 0.168327215 0.1691285110.14308705
0.159500267 0.180648546 0.189764455 0.178853106
0.188705344 0.191845785 0.196649717 0.192391822 0.16161786
0.213027083 0.1553911080.168002033 0.161652477 0.198906187

0.18750602 0.153104983 0.17143905 0.158004563 0.170545982
0.152539999 0.1944139110.140788027 0.168671869 0.173454497
0.198360576 0.170296282 0.157817702 0.199453258
0.154388966 0.184429525 0.167439213 0.177539461
0.156354064 0.132331016 0.1792361110.172529539 0.150969468
0.159798617 0.187526069 0.153866918 0.133542699
0.139536769 0.167386649 0.189591703 0.160445166
0.192364357 0.142425492 0.197474753 0.181618185
0.155587474 0.169252738 0.14976842 0.165804995 0.149340824
0.16918365 0.182657601 0.142050926 0.164991021 0.173929502
0.18781254 0.176977748 0.182690478 0.18170781 0.203755605
0.148679235 0.18624808 0.19665235 0.186053996 0.201542829
0.153610926 0.170318191 0.159962285 0.178026481
0.162713124 0.164206858 0.166624197 0.186287632
0.158099979 0.195106907 0.15754166 0.18892602 0.199681306
0.189999741 0.189376455 0.172132127 0.19290813 0.227777962
0.193788482 0.198741347 0.181830655 0.157233743
0.1559011190.194793614 0.156773914 0.17616732 0.136858416
0.138095398 0.204108141 0.168716033 0.187129563 0.17044072
0.148603506 0.195249346 0.1848782110.1733172 0.181624503
0.199890071 0.178104026 0.161903581 0.15845563 0.137416599
0.148632959 0.1876455 0.183760065 0.10954833 0.14884911
0.152120451 0.150816744 0.189004726 0.184693313
0.169884247 0.136042391 0.147561023 0.164692993
0.143479552 0.162203272 0.142097127 0.182152188 0.15401922
0.164624025 0.139408187 0.158179605 0.152514946
0.171426926 0.13581811 0.16363534 0.137364159 0.155630626
0.160555631 0.165835183 0.197794814 0.122483701
0.158642791 0.151808854 0.175245002 0.152584404
0.184457617 0.170092553 0.141832193 0.197018324
0.156245537 0.182842635 0.174689684 0.149499849 0.17528506
0.197183143 0.196141387 0.200202273 0.183431976
0.196518368 0.126904268 0.165084419 0.157469894

0.191619529	0.172573868	0.188757983	0.180871787
0.168180973	0.139280174	0.15471451	0.200588396	0.151417373
0.178011091	0.166617171	0.210181667	0.163921269	0.176495845
0.163925258	0.18569851	0.170647945	0.161887825	0.151707892
0.17070747	0.191077029	0.175711821	0.145106732	0.148963067
0.188466982	0.182955614	0.144728126	0.16786127	0.180000459
0.1514058	0.150260314	0.168702731	0.149153416	0.171758428
0.175142192	0.212994566	0.182329057	0.175680566
0.166426512	0.202019687	0.187322882	0.171108752	0.17117945
0.138192828	0.147670759	0.190614553	0.183693961
0.169421618	0.201548162	0.192732348	0.174498539
0.184819827	0.17868464	0.207928056	0.144490148	0.151745855
0.143995858

KEGG_GLUTATHIONE_METABOLISM 0.251557595	0.227022731
0.222844981	0.246075614	0.266595704	0.24496159	0.237554948
0.227502901	0.272799113	0.233454003	0.268822587	0.231187807
0.22661875	0.228357676	0.235801063	0.270243218	0.211847558
0.265718896	0.248214838	0.237295919	0.279898576
0.259251908	0.288586751	0.257116355	0.247545012	0.238895008
0.250274225	0.24905272	0.239810647	0.239451475	0.242301801
0.252251119	0.214491805	0.260547926	0.227535517	0.241117022
0.262216875	0.256545669	0.275515487	0.228569652
0.203982809	0.270970274	0.250593766	0.275596632
0.241548476	0.234203351	0.241312438	0.222307988
0.267395526	0.258292811	0.249304758	0.246598204	0.242236279
0.272957393	0.268906693	0.226422478	0.229916098	0.26151155
0.263346606	0.221856975	0.311811773	0.233108786	0.239318627
0.25138059	0.242657053	0.235055213	0.291060193	0.285890562
0.222983766	0.251381374	0.239089656	0.247882173
0.242045031	0.22905301	0.243636793	0.232762375	0.214511468
0.236137283	0.266509753	0.240057466	0.27959105	0.237819406
0.23492856	0.228388194	0.24880068	0.233121609	0.219274582
0.25496021	0.251778577	0.238357432	0.242106325	0.251865519

0.269514088 0.247736294 0.25797764 0.250623851 0.252466414
0.21827885 0.252838229 0.243853722 0.256539111 0.198195031
0.296275928 0.23465663 0.235645371 0.284993716 0.269566304
0.259753485 0.226375009 0.217170552 0.273469852
0.226300064 0.206851499 0.231078332 0.22126469 0.240789248
0.266741233 0.25055848 0.215729538 0.240792107 0.241096326
0.234241284 0.239112039 0.210592829 0.23362085 0.283174968
0.242123903 0.220710514 0.251876265 0.269672078
0.211783388 0.234469805 0.25538369 0.213241098 0.219308767
0.282434352 0.215884156 0.21188534 0.209065674 0.234303204
0.233754737 0.198466335 0.236151468 0.206787046
0.247575382 0.224989939 0.228505076 0.24604231 0.195064223
0.237754237 0.261102224 0.235658983 0.23105688 0.267912434
0.22800649 0.230598927 0.228457 0.251169024 0.233615431
0.249013342 0.263012362 0.238099683 0.253020509
0.252899175 0.235960211 0.268849616 0.244975638 0.255764943
0.229313257 0.24684858 0.220785439 0.261787846 0.230164598
0.294799653 0.249169207 0.228651216 0.261080848
0.268352278 0.263945387 0.21143647 0.22756714 0.287591955
0.259444084 0.241340321 0.268509451 0.237884995
0.259964625 0.243835339 0.255476038 0.218916494
0.225209138 0.233323441 0.243012041 0.237886031
0.255495447 0.197254574 0.251729884 0.222955879
0.233165011 0.22313874 0.233497161 0.247826678 0.245185307
0.224774472 0.231156935 0.238409946 0.231547318 0.235047799
0.242207385 0.228286347 0.210921702 0.215706863
0.228034966 0.246265576 0.223298626 0.214528935
0.201347011 0.226007857 0.237842801 0.230648011 0.206531683
0.250342175 0.234139664 0.220956435 0.227539588
0.240873368 0.241622644 0.228382797 0.237264044 0.21840833
0.254899474 0.255953317 0.235612437 0.228353282
0.243016539 0.244830768 0.254114398 0.226313526 0.240409721
0.256719643 0.244566221 0.232563415 0.251548215

0.216886517 0.2293224 0.240557138 0.2810811780.259889914
0.266819404 0.235034661 0.219743903 0.223387022
0.231221222 0.240258733 0.253345765 0.237273632
0.231458736 0.233627408 0.24877096 0.242587708 0.262916209
0.2216898110.257171757 0.219218871 0.258759402 0.229641791
0.268138294 0.238520677 0.224741069 0.247686404
0.282372107 0.216999773 0.212625193 0.242874721
0.234433234 0.233932407 0.253502697 0.255480232
0.204615404 0.209649399 0.243552633 0.241690871
0.256379612 0.237309657 0.240209036 0.246058298
0.212595975 0.2401411490.248145425 0.276163537 0.246991161
0.214836874 0.256986425 0.240601041 0.243013792 0.26271691
0.235410026 0.231309616 0.28119453 0.228814221 0.261596226
0.253193671 0.226845158 0.237158827 0.258273836
0.204593518 0.260787929 0.241699958 0.2472197 0.220954156
0.245960507 0.243964897 0.234294507 0.204521657
0.208077273 0.245123958 0.249201926 0.253815973
0.234199215 0.216120542 0.260302298 0.308842571
0.237769052 0.270555951 0.253935583 0.215210084
0.2211655410.271498958 0.240746146 0.245605278 0.235931928
0.233449127 0.233421266 0.235416447 0.2271565110.219729908
0.2452444 0.245659717 0.223043262 0.282930803 0.252776418
0.233182077 0.216228993 0.214865947 0.226600261 0.24571022
0.222687137 0.240863851 0.228607844 0.233741375
0.241470708 0.23151584

KEGG_GLYCEROLIPID_METABOLISM0.151027826 0.14597477
0.120237571 0.1479004110.148289281 0.144248598 0.149687814
0.1145474 0.150069886 0.131863722 0.1501191590.138254924
0.1326616110.134984497 0.1194797610.130053892 0.126613765
0.148382773 0.135465232 0.126932151 0.158219418
0.149012563 0.13956783 0.143555949 0.155147887 0.128307531
0.127498786 0.135855509 0.13850382 0.156245175 0.135920126
0.134571566 0.165345851 0.136455292 0.135588804

0.138641078 0.096061044 0.145853524 0.133823781
0.131617919 0.138920229 0.1187408680.139509768 0.13685314
0.1193659280.13143736 0.147507202 0.147022313 0.138081484
0.142959317 0.161833915 0.103501444 0.125123665
0.154848558 0.138131724 0.132036912 0.1142612440.146938446
0.139388612 0.122382693 0.157551855 0.157669169
0.130893248 0.137709898 0.147878143 0.1150077750.143304535
0.149916337 0.1391158640.145354546 0.151086322 0.127868628
0.137998586 0.134386263 0.142012982 0.145791689
0.126268983 0.140555759 0.152913337 0.136942422
0.132133681 0.13410098 0.145273397 0.1142170580.119891607
0.150714964 0.140896838 0.126387524 0.138739877
0.139609441 0.1197927730.120683917 0.13603773 0.118442682
0.142871359 0.139908017 0.1146756770.140594587 0.149271095
0.146637551 0.1566941190.138069258 0.137165791 0.138458933
0.139566473 0.159797501 0.140272867 0.155902594
0.135718761 0.1411719010.145748749 0.153304383 0.12418345
0.132793633 0.1169547230.144120176 0.130689556 0.13362319
0.137820964 0.130681231 0.121623434 0.1155388490.127712476
0.146466976 0.127291222 0.136916416 0.146619375
0.120799051 0.133446228 0.126237574 0.140690607
0.145201066 0.132984629 0.150385395 0.129172417
0.1373897110.13110799 0.156701063 0.127534666 0.169100714
0.137308902 0.146970442 0.130880841 0.142076151
0.136333677 0.121754374 0.153405764 0.140833445
0.127474642 0.147594914 0.135083269 0.126070896
0.147026756 0.156235955 0.145768691 0.1176260470.148154683
0.153465088 0.156124689 0.142840607 0.153570546
0.150062254 0.1341105220.145600328 0.134205974 0.139507251
0.159877538 0.134671864 0.152418289 0.1609546110.124367235
0.13651383 0.139481583 0.15841475 0.1504531180.13371143
0.1468611220.1156020810.134922545 0.138181583 0.142668385
0.164445973 0.125825308 0.140163045 0.148531278

0.1192278720.123498752 0.124029012 0.157470757 0.136736635
0.158343897 0.14107245 0.124653691 0.12424318 0.142218417
0.14913177 0.128896439 0.128261547 0.135160653 0.135589132
0.155785943 0.158998056 0.124026667 0.1418113930.116973
0.15073286 0.124769874 0.138598304 0.140207244 0.151258725
0.140489935 0.11930407 0.12407611 0.136579052 0.141015366
0.142009395 0.1502805110.145941826 0.145596829 0.134259481
0.132141754 0.142757025 0.1524741 0.145182518 0.141601181
0.1194065590.149844336 0.144271323 0.104020198 0.146048399
0.14036599 0.141555214 0.143879744 0.107152818 0.154053418
0.130469769 0.159255202 0.136941846 0.139837655
0.140152585 0.157341507 0.148305705 0.144186783
0.142768203 0.138063123 0.1384018 0.125279356 0.156480953
0.124490952 0.11763571 0.141938362 0.123655143 0.144167296
0.148796823 0.140512386 0.138329436 0.1148141870.126001148
0.122688523 0.133991349 0.120039808 0.141038759
0.120699272 0.120662325 0.133196177 0.135083038
0.139939488 0.131895629 0.120453226 0.123380109
0.129334196 0.130374533 0.140442263 0.130472276
0.127073278 0.161571458 0.133217 0.132859727 0.13035097
0.134453897 0.1131593010.130038158 0.10567416 0.151460702
0.152284727 0.137259356 0.155744928 0.136514756
0.123355451 0.1371177490.139108514 0.149672756 0.152801765
0.139060467 0.143047895 0.153718557 0.145914792
0.141526714 0.1453311950.138460869 0.11846776 0.156444663
0.147622434 0.1316605 0.134800693 0.148877041 0.14737912
0.145555172 0.120225702 0.149597565 0.133367936
0.147539292 0.130648898 0.133540743 0.155182565
0.140619687 0.143149058 0.143634828 0.145197733
0.1100900160.142984132 0.125821834 0.13813121 0.173933471
0.146584262 0.144737902 0.131697628 0.149741563
0.125969402 0.136040716 0.1186191750.1400699 0.141474152
0.133830019 0.1448116350.157385382 0.148973029 0.144310634

0.146601034 0.164728055 0.157064961 0.13738252 0.137875237
0.136339627 0.142301846 0.126976332 0.12076253 0.138330632
0.124929394 0.128796106 0.132608178 0.125246783
KEGG_GLYCEROPHOSPHOLIPID_METABOLISM 0.171014672
0.180666599 0.1367911930.1591154430.174233432 0.164715523
0.16761703 0.1635145 0.1411470050.137253434 0.160890321
0.143283537 0.1549516 0.147621785 0.149472028 0.144878714
0.167058851 0.169135019 0.14393143 0.150856456 0.16185899
0.179252845 0.164942646 0.16703379 0.16796388 0.150130635
0.13870233 0.165449142 0.159234785 0.182333102 0.142072537
0.153176303 0.1511335240.185254919 0.141420028 0.16174522
0.134474891 0.155582871 0.133358795 0.157715163
0.153607828 0.155018806 0.162497895 0.148072082
0.142805321 0.1556724 0.158365277 0.176356569 0.149007381
0.150079946 0.169109758 0.130307655 0.142250534
0.145004343 0.15620249 0.149898069 0.145131246 0.154625338
0.147020168 0.152941272 0.155489025 0.137633354
0.155290933 0.163126538 0.155689027 0.153904876 0.17069453
0.15589936 0.156403743 0.137145803 0.165439041 0.149841656
0.157215356 0.154515484 0.163706074 0.171342234
0.154764446 0.165068034 0.157331839 0.162670472
0.152946431 0.142667526 0.154588616 0.148066636
0.159303087 0.154542352 0.151657886 0.141781052
0.1526411950.15279069 0.129440288 0.134552529 0.135055676
0.1273112830.167005276 0.155318323 0.155098713 0.14730868
0.16237005 0.160964553 0.150680814 0.157609577 0.165006678
0.145942315 0.142044947 0.165454627 0.160176344
0.166601281 0.147927174 0.174932419 0.164671928
0.164034515 0.1552711040.165305895 0.14221676 0.161593538
0.147818278 0.142927339 0.158613189 0.147604473
0.147660736 0.152629301 0.160620251 0.154849762
0.158786187 0.135744403 0.141558932 0.158004364
0.142561532 0.1514211810.170563772 0.145773825 0.14934572

0.13907523 0.158193855 0.169054648 0.149297429 0.175370622
0.164926482 0.16496653 0.152131111 0.1651162790.158215243
0.174423279 0.180601421 0.144138428 0.143886104
0.156992948 0.146176574 0.165645313 0.15649906 0.154726767
0.15419453 0.179658304 0.169186297 0.162351083 0.163707898
0.187466399 0.16219828 0.168826183 0.16192076 0.160903824
0.1711046590.153204451 0.145166743 0.143548528 0.159956422
0.163493195 0.164800954 0.159553634 0.177671338
0.159673732 0.159356729 0.169778104 0.148242962
0.147952958 0.177398483 0.15620542 0.146703971 0.162905939
0.15017607 0.190742779 0.163635956 0.154190904 0.170041168
0.14609817 0.14902032 0.162566453 0.172893017 0.161137353
0.171858404 0.167807685 0.151091308 0.1557311640.155395454
0.163420246 0.162097029 0.153815513 0.164932972
0.159151614 0.168647365 0.165412708 0.153964314
0.174924461 0.154144358 0.168537547 0.151573249
0.172180259 0.16510194 0.164022009 0.151431528 0.160737369
0.1619361170.15890548 0.156262669 0.168167427 0.170085128
0.152331624 0.156824916 0.1619115640.1511467790.166526264
0.16549954 0.1656417 0.167403357 0.17668109 0.153331799
0.162288933 0.1116820060.167902651 0.15840148 0.154640735
0.164582693 0.1350811550.171508439 0.138547255 0.155745945
0.157618325 0.154703755 0.133892797 0.152710694
0.151098589 0.142683038 0.15281509 0.150656276 0.184693062
0.132837774 0.147692834 0.147650724 0.134824305
0.144348252 0.15007964 0.173126365 0.144551648 0.150216464
0.157300896 0.158324142 0.148544063 0.148745412 0.14653853
0.155234163 0.149296714 0.127684497 0.163291928
0.150634245 0.167567476 0.153868502 0.153288257
0.160085522 0.157751013 0.1409011740.1672431110.161184504
0.131939134 0.141681427 0.16079465 0.1311812650.154798664
0.151801746 0.16765964 0.144552489 0.138666436 0.143296953
0.164773592 0.153172531 0.159899883 0.177185844

0.159228017 0.141505151 0.155099089 0.160144126
0.173438361 0.145618942 0.17497428 0.140484072 0.174527109
0.16698926 0.1647283110.161096285 0.154036424 0.15674913
0.146836397 0.158987094 0.1731157570.147273566 0.175278916
0.1616481180.1593705110.154838834 0.173404955 0.167679291
0.156777079 0.145919807 0.159822668 0.16471288 0.168734318
0.162286287 0.149350971 0.162228362 0.158476806
0.139489023 0.156251816 0.170804826 0.172392915
0.154204197 0.156750382 0.155100107 0.1652301110.173898111
0.155561947 0.166524227 0.1478582 0.151975178 0.144359222
0.178089436 0.176435039 0.152169925 0.173588623
0.177728882 0.166220659 0.1637083 0.167336522 0.179931454
0.1742871140.170461886 0.147256532 0.151546603 0.165149181
0.150717981 0.162419364 0.139316985 0.135728263
KEGG_GLYCINE_SERINE_AND_THREONINE_METABOLISM 0.036184498
0.096965061 0.022380029 0.1138999890.063731586 0.058275232
0.091828749 -0.004690234 0.069127746 0.0113199480.036077769
0.017184612 0.008838273 0.0611914760.033830413 0.101039377
0.03911038 0.068077323 0.0311068 0.073843679 0.072329956
0.051718526 0.076738557 0.057028538 0.03042917 0.034453067
0.06834537 0.087935803 0.034892308 0.017212885 0.058332903
0.084213157 0.069608972 0.07137389 0.073884393 0.047457518
0.031741203 0.068659056 0.043968037 0.04351243 0.049231137
0.045093004 0.031096602 0.0811815490.027283914 0.046754266
0.035182067 0.052815768 0.068588249 0.058784052
0.078175491 0.073415063 0.05387044 0.055040726 0.07931734
0.035463385 0.043236408 0.049901752 0.06954073 0.039348485
0.06052755 0.000233898 0.052164692 0.0648941130.077092636
0.063900071 0.064177182 0.054396531 0.026096906
0.052700839 0.052564695 0.053774338 0.035951443
0.024643808 0.028390616 0.015258328 0.0625511590.016763714
0.041222528 0.028598696 0.056651004 0.069081628
0.048059779 0.07003715 0.015512917 0.040108657 0.008281192

0.036729619 0.036694426 0.047393674 0.108262256
0.049688009 0.056765239 0.058148122 0.019726965
0.054128214 0.012412949 0.00457198 0.043876476 0.027806202
0.027705991 0.073506063 0.11949012 0.044803248 0.063663913
0.099895656 0.0973112520.037077793 0.039482195 0.084972303
0.076508274 0.053128389 0.045693491 0.013356374 0.03877122
0.052903303 0.04051779 0.040259659 0.0111957790.029454454
0.066729696 0.044498577 0.064538532 -0.000744689
0.0667817110.077127469 0.039318742 0.050102987 0.046750058
0.033337937 0.01615714 0.02543903 0.044734594 0.029379674
0.035348741 0.034829309 -0.011977116 0.009273866
0.044055253 0.054541823 0.0411307290.017999539 0.098038713
0.066934246 0.064379667 0.037628249 0.012229229
0.036716634 0.039993217 0.040376873 0.055386494
0.054149052 0.073808836 0.042661378 0.080461948
0.019906396 -0.02790501 0.030745309 0.090185344 0.074495053
0.037789662 0.070515061 0.0411469820.025075204 0.005549967
0.045162192 0.054261891 0.1144141090.052314061 0.055011412
0.036368055 0.061058283 0.097293557 0.084303428 0.03949658
0.046846277 0.038881392 0.0490024110.080654427 0.039197011
0.004250475 0.138740391 0.056838678 0.037636094
0.0566282110.036302584 0.049598427 0.065691089 0.074262804
0.067016918 0.094623864 0.012824682 0.043538124
0.048900853 0.029202899 0.066162176 0.002152377 0.06615285
0.013141838 0.060462972 0.032529135 0.059926685
0.033578082 0.06762408 0.043842307 0.069140638 -0.00680295
0.040967599 0.0484328110.074026923 -0.018255108 0.094254563
0.019902784 0.033738721 0.027802892 0.0544118470.033677907
0.038872041 0.044188598 0.065420291 0.026674033
0.026553972 0.034690313 0.060813319 -0.035299711
0.067103143 0.078978668 0.052836416 0.040239358
0.033536544 0.039148153 0.055917282 0.02537472 0.020846301
0.029046654 -0.000713051 0.090679228 0.064892769

0.058983308 0.050947922 0.029273234 0.020304598
0.056324731 0.01583879 0.03682657 0.0332045 0.050897015
0.092253175 0.049163996 0.016662097 0.048045788
0.012628494 -0.027552187 0.085675486 0.090000307
0.049769906 0.013380667 0.054309832 0.022438649
0.063457799 0.075129055 0.029475129 0.043868748
0.089216502 0.059680314 0.028939123 0.08462155 -0.020685938
0.061683364 0.034474154 0.070698091 0.079040204 0.01734871
0.047247391 0.021763989 0.105592443 0.036196673 0.04785981
0.040208014 -0.000607517 0.062285788 0.037232455
0.065250703 0.03407924 0.014735376 0.021417605 0.005461951
0.006813358 0.022448682 0.046001363 0.027687468
0.009903949 0.071048783 0.0975353110.077838648 0.040065268
0.048662678 0.035224109 0.049398341 0.059881377
0.060725488 0.08133814 0.036631828 0.034377209 0.075351364
-0.007021049 0.061747999 0.086933578 0.1146652630.070886687
-0.004833329 0.041879562 0.022827876 0.005505483
0.054672815 0.05887502 0.073819952 0.072813634 0.063493493
0.028487138 0.064874251 0.104299549 0.045192183
0.066525533 0.047328457 0.057491765 0.000158 0.08323713
0.066421425 0.020490941 0.100573759 0.063247072
0.025517872 0.016336712 0.050851715 0.0569571170.053943637
0.056731939 0.056165905 0.0809114220.06526393 0.027236993
0.018187153 0.026656131 0.033226148 0.028553052
0.084178455 0.070009413 0.034826847 0.06725939 0.016112986
0.076027716
KEGG_GLYCOLYSIS_GLUCONEOGENESIS 0.166699609 0.173995959
0.169982915 0.202798313 0.19953057 0.160383872 0.182930941
0.158885427 0.191641862 0.152039518 0.177291778
0.1834911990.155450044 0.200136615 0.184500206 0.19966142
0.159901321 0.180277134 0.177295518 0.157008245
0.147370722 0.182539973 0.196669372 0.1775110160.179283686
0.175149373 0.193220549 0.189162101 0.170223441

0.173091236 0.157513854 0.168192335 0.169124848
0.164532799 0.185166408 0.197757267 0.150740565
0.170021805 0.17524544 0.156456015 0.192106996 0.203795604
0.203343775 0.193996307 0.17985437 0.180476018 0.191947463
0.167871456 0.229338066 0.199985575 0.202986662
0.182232093 0.198496135 0.162658289 0.186668419
0.193962234 0.1811442730.189383249 0.188146389 0.146362488
0.208467006 0.175341681 0.189376583 0.187709133
0.173681446 0.170256215 0.2055115910.197873325 0.178713274
0.179575998 0.191092501 0.155083324 0.173564839
0.162155932 0.168787167 0.174320016 0.1661101380.172249313
0.181538716 0.190737847 0.209403426 0.175921889
0.153541233 0.16581909 0.179700897 0.180090327 0.169527082
0.1784231130.167615532 0.178170021 0.204832393 0.19284492
0.192040415 0.188775969 0.193473439 0.146864357
0.181359349 0.170813898 0.216238672 0.207085816 0.1928891
0.176315773 0.177359693 0.189692349 0.1776615110.174615102
0.184648784 0.181200283 0.180593435 0.1791155950.199042103
0.145644749 0.152269649 0.170647447 0.193573943
0.182771951 0.177125159 0.181642621 0.150179207
0.182907086 0.176148396 0.162993156 0.165851793
0.158823286 0.17160709 0.19888177 0.181632776 0.155496066
0.187883606 0.187380055 0.179924208 0.172655096 0.18360112
0.19023283 0.174628603 0.172140054 0.164578718 0.187058289
0.168552153 0.192845202 0.167704339 0.196376636
0.200571405 0.15654905 0.18095648 0.19043866 0.175020614
0.165344553 0.156753733 0.172877292 0.174277219
0.161341828 0.184499667 0.176400715 0.1911253340.174186439
0.15696934 0.219060593 0.194748632 0.199058009 0.19080862
0.1811180670.188891837 0.199637696 0.1836611220.198072809
0.173342683 0.201634577 0.179744109 0.190584704
0.202352381 0.201304485 0.18601455 0.188002652 0.19375339
0.1765051150.197314942 0.163846752 0.193290167 0.180867335

0.188928569 0.157759649 0.176427092 0.159844574
0.185002803 0.176017563 0.180010874 0.160229402
0.172064477 0.17438406 0.179275534 0.186551694 0.188341408
0.197782422 0.186489806 0.167084538 0.197419418
0.199176502 0.184297221 0.20566931 0.189080401 0.16397082
0.192528915 0.174182696 0.152217741 0.189445494
0.162639146 0.173254165 0.183532203 0.173467803
0.169551491 0.182978968 0.185359561 0.168820513
0.197796013 0.189766802 0.16806059 0.218841303 0.201038453
0.186061449 0.17342045 0.18911364 0.177123339 0.168102168
0.177890664 0.132829703 0.190317535 0.180727343
0.173367955 0.174734162 0.199338844 0.189493989
0.167909456 0.1805112390.176260775 0.195327163 0.207448504
0.1814775110.202210727 0.171728646 0.173754903 0.179631967
0.175603341 0.159499368 0.176524107 0.170778865
0.171408588 0.215867351 0.196047947 0.185456275
0.1852311380.159542064 0.17249108 0.197971368 0.194109369
0.15960547 0.184589293 0.182718155 0.188798574 0.182987904
0.1787162110.163502477 0.188319357 0.169592194 0.180905203
0.194101012 0.197463526 0.166178518 0.187010901
0.152737619 0.18612578 0.195788335 0.141548276 0.164612331
0.167609839 0.188953365 0.181244787 0.188215002
0.149166058 0.198521036 0.183062202 0.19227251 0.174320122
0.190269399 0.188093891 0.180238594 0.173495194
0.189854918 0.178615392 0.186367676 0.166507059
0.171077248 0.164131353 0.172414877 0.185417225
0.183723694 0.20369355 0.179185769 0.1923113950.179006619
0.175585221 0.173988712 0.18171586 0.175521871 0.201457629
0.168360856 0.173635517 0.204826975 0.19180977 0.185082518
0.190168406 0.174572725 0.15472803 0.169557364 0.145981421
0.159249643 0.194855318 0.200898841 0.179348692
0.150872206 0.169507762 0.20029432 0.174123553 0.165153923
0.190138537 0.166499696 0.174658292 0.178163154

0.189340557	0.171623386	0.214531986	0.180909302
0.156277497	0.179014606	0.175886527	0.176214813
0.1704779110.202206918	0.159843441	0.223499142	0.186694602
0.174136802	0.175971781	0.136933263	0.172032301
0.185983542	0.191692339	0.150057657	0.175594374
0.148216793	0.181700547	0.187747566
KEGG_GLYCOSAMINOGLYCAN_BIOSYNTHESIS_CHONDROITIN_SULFATE
0.07335378	0.1316581120.090465156	0.065301092	0.081774067
0.091995823	0.083734786	0.074399007	0.084365328
0.080821521	0.060722693	0.083318022	0.094654937
0.065750649	0.107948614	0.039559661	0.072051492
0.071958189	0.089513518	0.0810173	0.135877828	0.092343928
0.076248015	0.105356099	0.082778352	0.039376759
0.095222236	0.055352753	0.07757145	0.079545534	0.067579758
0.100224009	0.135542912	0.105722905	0.038592142
0.054792898	0.1182991730.041270543	0.080969861	0.059552878
0.1103684170.063742196	0.042384494	0.083801972	0.077324342
0.080566805	0.159650765	0.031556365	0.062512531
0.102883905	0.140252087	0.055514135	0.0211544110.057797253
0.075932549	0.122453029	0.040215309	0.083290766
0.049028973	0.093589807	0.048088094	0.105129824
0.088943671	0.127544414	0.090883865	0.074489077
0.071542733	0.080199494	0.092801931	0.108944938
0.1137399630.053334802	0.139135917	0.078848886	0.087905065
0.140700459	0.097135414	0.108282944	0.141879791
0.088809058	0.049772778	0.060188048	0.107388085
0.087232148	0.032830398	0.062106024	0.100926402
0.092502621	0.093096805	0.1138348840.07022703	0.079340581
0.087062678	0.023850202	0.08604274	0.101217255	0.0595977
0.104829403	0.140033683	0.102642399	0.125645198
0.124606916	0.0303711210.1160419510.066268172	0.122084211
0.089175601	0.1168764480.060304636	0.072287638	0.032474927
0.058903461	0.050251957	0.105555438	0.065956758

0.131094493 0.057665472 0.105788505 0.072038004

0.073098669 0.10474248 0.1129735760.101369396 0.124596602

0.077774894 0.056562938 0.06526051 0.074698542 0.057292389

0.0939110420.07224615 0.044667273 0.0451124750.100663048

0.101610615 0.0714417 0.1120249330.061356331 0.084204301

0.150756742 0.090553309 0.131276401 0.079364733

0.064724672 0.036790086 0.154616659 0.11394032 0.058692803

0.046038464 0.060668295 0.057953803 0.046717109

0.1123992420.036432561 0.134269584 0.105692004 0.173610326

0.069751569 0.045931458 0.096477286 0.041622635

0.083044622 0.065121917 0.095178704 0.129247676

0.052425396 0.121679222 0.043790332 0.079717245

0.097161818 0.080885459 0.058044141 0.065035374

0.047672404 0.089142188 0.1154562580.11073692 0.089070425

0.034926916 0.11056972 0.1172789820.107812871 0.078181854

0.097997499 0.04753899 0.104314571 0.091065968 0.126918582

0.080363653 0.086106082 0.104680815 0.091790043

0.096531498 0.095371279 0.09264027 0.1162390720.086160756

0.10328534 0.142122894 0.06725262 0.15518056 0.044625155

0.103985014 0.072079331 0.08884078 0.083823204 0.171179759

0.072658355 0.098654967 0.04989625 0.107466752 0.071037084

0.088582758 0.120542639 0.106457673 0.154531501

0.1132685410.13957577 0.200447246 0.064828965 0.136166054

0.094093263 0.093024362 0.1121996040.14529045 0.080419885

0.129253229 0.080708165 0.076609481 0.190076392

0.090050644 0.08609491 0.10482561 0.082771858 0.144364105

0.120576296 0.11193553 0.129601695 0.099226013 0.112023289

0.073153633 0.106219528 0.060568728 0.095326233

0.137126271 0.1113785290.055215079 0.1023831110.065997247

0.068584365 0.1122498430.097463847 0.089726353 0.059597324

0.0653129110.093259559 0.077020203 0.125245633 0.073420986

0.094070472 0.1168244830.108943122 0.088330165 0.06402014

0.09431621 0.101879073 0.052697569 0.081253637 0.092481268

0.077551922 0.096593713 0.056820448 0.154767393
0.043604824 0.093924754 0.095706314 0.124296195
0.097373673 0.144961982 0.12252818 0.048325722 0.127201135
0.101623443 0.109140473 0.132051059 0.069107576
0.099756874 0.108591641 0.133509871 0.155607072
0.117069931 0.157812029 0.05451234 0.072018587 0.098388428
0.151276886 0.119523777 0.152307145 0.075840405 0.069631868
0.033368014 0.100825782 0.142512305 0.055774833
0.099392476 0.051761973 0.106438527 0.102136192
0.090838014 0.129538897 0.127752879 0.084083602 0.09718432
0.069685974 0.09018719 0.202911995 0.078785058 0.075649451
0.0692314 0.105699882 0.130429501 0.059270246 0.089303551
0.112504091 0.059847471 0.041600505 0.053471883 0.051992624
0.061792062 0.125772865 0.093842088 0.085398781
0.075336633 0.125190142 0.152114983 0.114565229 0.098692135
0.113933572 0.084243763 0.03395207 0.153747544 0.136619234
0.113135963 0.177819991 0.052199935 0.079462239 0.072574782
0.099510738 0.125301768 0.038818678 0.067214202
0.050274819
KEGG_GLYCOSAMINOGLYCAN_BIOSYNTHESIS_HEPARAN_SULFATE -
0.003835479 0.042263207 0.00589694 -0.00163676 0.030423086
0.045658043 0.020214181 0.016552273 0.030995028
0.024141406 0.003477724 0.024696781 0.056833314
0.037481115 0.022349785 -0.006587544 0.000101345 0.02319465
0.044332585 0.004646244 0.07739985 0.002224262 0.017047047
0.070425585 0.02698193 -0.00806445 0.01574318 0.003979864
0.050231773 0.027014143 0.023221705 0.0465222 0.037198331
0.064963588 0.023510041 -0.002722187 0.065893372
0.003657452 0.007705487 0.020781137 0.047306992 0.008115952-
0.010184349 0.031536071 0.032951363 -2.61E-05 0.046062846 -
0.017198648 0.000921026 0.018687144 0.007738723 -0.016008236
-0.009927151 0.011691868 0.01082015 0.017483351 0.00324919
0.007966802 -0.00543023 0.021715795 -0.000465316 0.03855291

0.027134069 0.025103632 0.003243877 -0.013988402 -
0.00930588 0.022719256 0.025782458 0.018757587 0.047498459 -
0.001545408 0.032574661 0.023608225 0.040089762 0.035993076
0.021981556 0.038070481 0.04323481 0.008852303 -0.01455123
0.006051287 0.071337358 0.001096119-0.014896638 -0.003687776
0.010962428 0.024425354 0.021548367 0.051810084
0.004954402 0.029579807 0.013446562 -0.047638423
0.027720619 0.040107657 0.017710813 0.031613879
0.045468369 -0.011039598 0.040407682 0.080514894 -
0.020550596 0.042369468 0.030305748 0.056957662 -0.001263288
0.05305561 0.0116576830.037927213 0.03590548 0.029309255
0.022769809 0.042565941 0.018009934 0.044060626
0.007690157 0.01151135 0.022987029 0.000374934 -0.001918045
0.017889519 0.02540559 0.05268443 0.027623182 0.016751644
0.004547237 0.003607673 -0.004196773 0.0081101170.001038162
-0.009927233 0.003432709 0.023491738 0.03844286 0.05224638
0.020862624 0.010222777 0.01177641 0.063045969 0.020016415
0.051035583 0.023044914 0.043281366 0.028076684
0.0591138870.041440408 -0.003339543 -0.002359029 0.005066292
0.007869377 0.026843971 0.017813511-0.011829572 0.031335395
0.00717101 0.077004596 0.01205127 -0.001364895 0.007655297
0.029863544 0.016595612 0.02295523 0.027308523 0.041127393-
0.035805647 0.057165076 0.016612431 0.023970273 0.057903936
0.005283678 0.004146868 0.020755093 -0.000397924
0.010296587 0.036235805 0.027924149 0.015668359 -
0.007898419 0.02071961 0.028537589 0.020706048 0.012537962
0.024135201 0.007679051 0.008450431 0.049597605
0.042783223 0.022407672 0.041257145 0.042735606
0.004200197 0.027339727 -0.001280626 0.015291253
0.040321513 -0.006099848 0.018985489 0.064472717
0.012189573 0.047048072 0.009748945 0.028680339
0.024183729 0.00524359 0.005775209 0.080980743 0.005633898
0.034630485 0.001444355 0.012948807 0.0112046110.026170845

0.045037652 0.0421021160.064698832 0.039513442 0.047184773
0.088092821 0.026099668 0.057659622 0.038509871
0.032058057 0.057960394 0.059901698 0.031518074
0.059287275 -0.017071113 0.041024009 0.08396183 0.036680838
0.012364292 0.044954588 0.004433297 0.061480216
0.037783962 0.055202044 0.044657153 0.02832625 0.018506167
-0.00735918 0.0371851190.022512846 0.073082742 0.057693534
0.027002708 -0.011575716 0.055601764 0.0045911090.023315554
0.0274811250.0245206110.008368609 -0.001636277 0.015863607
0.030537321 0.023768814 0.025189977 0.0118792920.045916992
0.061722064 0.045451328 0.0465515110.016780068 0.010369241
0.027316573 0.028868691 0.03052416 0.034702218 0.050030384
0.01296879 0.00166009 0.074929388 -0.005130525 0.01915749
0.06336557 0.054037812 0.0057115850.079659173 0.032594474 -
0.003197012 0.042786979 0.021973488 0.035216222 0.063572191
0.010760031 0.031930633 0.012943126 0.027135721
0.071901648 0.040390788 0.06251238 0.006590206 0.017020275
0.037970972 0.04854213 0.052043795 0.033608899 -0.012906882
0.047025223 -0.012281452 0.037586831 0.049315631
0.013588145 0.005912985 0.006683122 0.010900138 0.0273382
0.024303643 0.039855234 0.04833778 0.023890132 0.035179027
0.0011272850.037462034 0.109394061 0.024693037 0.003963547
0.017784481 0.042426755 0.051818968 0.020095224 0.02286776
0.062049398 -0.000746838 0.014615841 0.014092701
0.033583523 0.016636128 0.0471146410.028937127 0.02386051
0.017624437 0.040671872 0.057383974 0.064233558
0.027475549 0.0223337 0.0121136240.015924997 0.051229952
0.051499544 0.027569905 0.066692144 0.01016821 0.029066852
0.036721279 0.02943843 0.025969427 0.0113109870.023279278
0.001441713
KEGG_GLYCOSAMINOGLYCAN_BIOSYNTHESIS_KERATAN_SULFATE
0.272534601 0.28536492 0.193943026 0.252262419 0.204753013
0.178668144 0.226006367 0.277358429 0.248255706

0.212307486 0.207330127 0.21603012 0.158280614 0.258435878

0.210497894 0.214036045 0.257707419 0.281356879

0.215519531 0.189622262 0.267838698 0.198498754

0.237716962 0.239695099 0.27702328 0.166224195 0.2347248

0.217836017 0.215862101 0.273371559 0.175374129 0.19407683

0.207159908 0.237478136 0.226603769 0.238061967

0.210142501 0.188860789 0.195834069 0.16930481 0.235290212

0.20844706 0.163399577 0.248050292 0.249066926 0.217700332

0.248674569 0.2450101 0.221387352 0.202344514 0.205540102

0.130889324 0.15175093 0.213107184 0.2107811140.215546783

0.183125397 0.221281314 0.255263901 0.20790173 0.217248786

0.239384528 0.23577459 0.263761272 0.230399744 0.190863386

0.2349921130.222623638 0.233914379 0.198987942 0.260626556

0.203793379 0.205829215 0.181823939 0.265914444

0.287960107 0.212449144 0.221206314 0.230370948

0.171792189 0.232542613 0.149707229 0.191433634

0.262171683 0.171985438 0.204131995 0.213037578

0.215827664 0.206745259 0.231465757 0.261560723

0.185320822 0.221643701 0.219944819 0.25129779 0.213237192

0.17819694 0.242560958 0.273436725 0.262856445 0.253650062

0.248710044 0.270247305 0.204470347 0.180603758

0.286504627 0.196493133 0.267807522 0.235946533

0.226421296 0.313882256 0.240285845 0.177847808

0.204148941 0.225217873 0.204694048 0.267079654

0.229222444 0.285683479 0.234708188 0.242609641

0.214875232 0.215429815 0.232643786 0.241727839

0.174982055 0.209422228 0.230684059 0.240954753

0.1969351180.239306678 0.279778942 0.246099233 0.26884196

0.189775957 0.217496599 0.235825391 0.260496121

0.202689418 0.232610659 0.217659649 0.246103431

0.223568562 0.237708763 0.191660101 0.215358281

0.294460921 0.201435167 0.254535188 0.271579428

0.212530314 0.187792243 0.191226351 0.222269893

0.3030374110.2489508 0.271074819 0.217439745 0.192582619
0.252258726 0.247633032 0.212224567 0.233533623
0.278847239 0.223475057 0.177060073 0.198142937 0.2610449
0.214427503 0.271834391 0.225423184 0.185592675
0.253017072 0.21781406 0.196565805 0.2711292940.254871703
0.217643187 0.264788752 0.221324877 0.252988593
0.184385166 0.266700076 0.22531326 0.230957235 0.221650104
0.208058812 0.1977114340.239185449 0.222852158 0.26753216
0.180173214 0.323142475 0.229728203 0.2911792930.220242433
0.237561843 0.195566746 0.21622173 0.248046049 0.286218924
0.231638941 0.208525347 0.2252374 0.247725743 0.237333137
0.192985596 0.209875475 0.2348411740.213204902 0.24801162
0.282720464 0.294990741 0.2156373110.249621444 0.259974933
0.247757723 0.191404183 0.22371446 0.255285839 0.215711014
0.23571599 0.258061081 0.205293254 0.24913438 0.220845009
0.20140016 0.199033514 0.17636285 0.257193188 0.237928167
0.169916391 0.1989042110.260678014 0.221096096 0.184916291
0.236747545 0.250699601 0.205082081 0.259102373
0.253459813 0.29030224 0.198414838 0.263773575 0.281842595
0.223825768 0.2467145 0.250960959 0.251615341 0.235936553
0.206463081 0.205020094 0.266373798 0.203499461
0.243029045 0.256538897 0.23988914 0.243612996 0.221319916
0.264173704 0.268737576 0.261210706 0.209465173
0.220868553 0.257601213 0.219640184 0.2600113690.212168303
0.209732945 0.208756445 0.287121808 0.197724976
0.214791445 0.199545906 0.247172408 0.171861317
0.263461719 0.285821259 0.187758808 0.251033829
0.228024479 0.1961122530.237857836 0.267744905 0.228637266
0.201495923 0.247522152 0.21938255 0.2551165210.23312051
0.186406001 0.248882365 0.200950579 0.213175034
0.296418143 0.231913021 0.2121467 0.269577489 0.207696478
0.190033985 0.260256639 0.307584629 0.254594686 0.22521966
0.213569571 0.206525429 0.215425425 0.279329999

0.303086918 0.24622816 0.269403773 0.276607077 0.216608019
0.194325664 0.218616724 0.25936014 0.239345869 0.203562329
0.250684034 0.251408325 0.232431519 0.2041148110.193418673
0.231833438 0.224807243 0.181989813 0.212070126
0.250989618 0.243252755 0.265526502 0.213277638
0.278847819 0.282923122 0.224316058 0.2393118370.227530211
0.3011541470.25509699 0.172442842 0.184710199 0.299443703
0.23468177 0.295385132 0.2650117160.227829686 0.208954065
0.227958085 0.22713155 0.212393942 0.238172961 0.171169994
0.182979083

KEGG_GLYCOSAMINOGLYCAN_DEGRADATION 0.130181608
0.172620078 0.133368058 0.127612889 0.167028977
0.132756459 0.125905838 0.1106454270.169631508 0.093599633
0.1107730040.1191888820.150626754 0.157240883 0.138222076
0.105606498 0.1011676360.162667186 0.168815488 0.127964587
0.094583684 0.160244928 0.159224287 0.1705056 0.130422438
0.159043657 0.106054489 0.1335611570.184194176 0.153105351
0.124613653 0.175975415 0.1130506730.195094869 0.107685836
0.142445548 0.1162431860.179238123 0.126531437 0.08246602
0.14161891 0.106468658 0.127495985 0.178597931 0.16834204
0.1153488720.143840253 0.13505188 0.123132786 0.139523788
0.178430069 0.138400333 0.157169595 0.127907368
0.145967454 0.158315598 0.120654848 0.151325064
0.148195759 0.160970086 0.171359267 0.149775214
0.077557251 0.184733942 0.214017187 0.086481919
0.145036025 0.165510638 0.159940031 0.127871775
0.161618493 0.105424105 0.13773998 0.089916927 0.15300246
0.170006019 0.13872717 0.157623272 0.152083752 0.105201018
0.124163136 0.11948904 0.13873732 0.153895139 0.156356426
0.129912004 0.162934781 0.099128655 0.101425122
0.162615426 0.1164756160.147382358 0.124183697 0.13386815
0.160026308 0.160880647 0.147177314 0.066898003
0.221383598 0.170485322 0.138692541 0.210563644

0.103857743 0.139648961 0.131913222 0.157775626

0.146905453 0.168623421 0.181500916 0.139022301

0.160630163 0.138964004 0.123500088 0.155051832

0.134796907 0.161948622 0.12495997 0.133505626 0.150196758

0.12981165 0.138429653 0.122618337 0.209499899 0.138905491

0.141086903 0.146037661 0.146368699 0.126973955

0.098731856 0.156058617 0.147393122 0.12906128 0.140784293

0.12851476 0.1172099280.179254139 0.156516612 0.165909189

0.136338662 0.146208525 0.12131202 0.163026015 0.171592218

0.14514163 0.125753152 0.132212148 0.159484032 0.115864153

0.134414522 0.1142201530.157267358 0.130222305 0.163249499

0.152580287 0.149429229 0.135626482 0.1103284630.146169935

0.166438385 0.123254309 0.1247306 0.152817892 0.151227087

0.151334627 0.154762234 0.14786182 0.10851099 0.128224863

0.134663325 0.147643026 0.177910848 0.144404778

0.1311844060.171745046 0.148256713 0.195167152 0.150762597

0.159002081 0.188945428 0.127388387 0.192179409

0.1126981290.143184712 0.16116065 0.138384538 0.08421808

0.171758778 0.163874405 0.123581686 0.145068135

0.168531303 0.14230125 0.155267545 0.156120068 0.183932063

0.158961684 0.146581274 0.137050841 0.163410086

0.148336356 0.170470601 0.157355266 0.123446318

0.1105680080.169146207 0.152042805 0.140976321 0.092316037

0.141451668 0.137267237 0.161451795 0.139333677

0.129731719 0.131469302 0.156753224 0.160194078

0.137785709 0.172733481 0.173485056 0.1148112330.150763828

0.159417975 0.14847805 0.13034589 0.141542918 0.096199207

0.164430091 0.162720385 0.074288126 0.177427513

0.174353182 0.174307108 0.127272073 0.144404366

0.161686522 0.180741052 0.156869903 0.190267831

0.151839872 0.178189375 0.171955369 0.159287686

0.1153544050.176845808 0.139858157 0.159498469 0.132904974

0.122867286 0.159789329 0.153302234 0.155405228 0.16951477

0.153159226 0.123273346 0.108303246 0.149685968
0.148937028 0.170649802 0.108046864 0.184443035
0.125758737 0.1522808110.126431874 0.17628147 0.16440605
0.150672503 0.172256165 0.180150262 0.1104778280.135961886
0.135069153 0.129040386 0.156335763 0.1031911570.120501195
0.172821887 0.165940672 0.156410256 0.174794065
0.132642379 0.125006821 0.131062567 0.14941816 0.185748833
0.186091584 0.191083633 0.175509955 0.195869671
0.192449144 0.155715103 0.16353164 0.166666321 0.105515669
0.123935657 0.157742813 0.149778229 0.125185281
0.146127853 0.158483852 0.144392221 0.163394605
0.150086078 0.160199991 0.157264105 0.1189193690.153921207
0.138473001 0.182620195 0.122337017 0.153868135
0.184477523 0.159993703 0.069048722 0.136041734
0.143877176 0.175444813 0.203628731 0.173644515 0.14436546
0.151973041 0.159604631 0.18753239 0.1117782320.165998357
0.183489756 0.175633957 0.19568425 0.148221782 0.175771189
0.130000505 0.168663979 0.134397546 0.152066942
0.178719458 0.182331212 0.129675464 0.151489448
0.157737342 0.173057247 0.120777359 0.15096947 0.113616693
0.158109804 0.160957273 0.1680211280.1055711260.14620625
0.128383003 0.128359708 0.121730258 0.1139782330.108085726

KEGG_GLYCOSPHINGOLIPID_BIOSYNTHESIS_GANGLIO_SERIES

0.100297986 0.16073992 0.089792558 0.158830272 0.14136745
0.08301832 0.130882503 0.160507342 0.136890082 0.095034178
0.0981182940.138959629 0.043610309 0.1136734860.088620284
0.129501382 0.1110037530.193365795 0.055351678 0.072884202
0.069638449 0.177855979 0.180936515 0.185804263 0.16805609
0.148755237 0.051853486 0.09075381 0.0625502110.211194365
0.158473452 0.155504332 0.087421018 0.1198651970.091894988
0.148194179 0.065339631 0.100641352 0.181706032
0.086530135 0.152005358 0.1011790390.108053426 0.180895548
0.158077791 0.107050073 0.076947324 0.126936489

0.190342217 0.101920085 0.085980314 0.127307926
0.103820169 0.088783217 0.127806736 0.104951751
0.135252543 0.128460757 0.166686399 0.167495179
0.150546741 0.232644984 0.101939367 0.192823052
0.160022491 0.1150220490.188292451 0.198821272 0.119681363
0.123228088 0.207726855 0.059256201 0.057297306
0.049893869 0.122609772 0.186409057 0.071679714
0.139150199 0.122585046 0.095586178 0.15454346 0.123707956
0.03359858 0.061338663 0.1108322010.10065358 0.181566098
0.1110827310.1150455230.077967524 0.1330011720.185725354
0.11645826 0.13150629 0.135027919 0.140283299 0.101541057
0.027171287 0.140906838 0.194703286 0.09321689 0.121784491
0.163636693 0.130916244 0.092934496 0.208160623
0.047603767 0.17788125 0.154060728 0.098928188 0.148614811
0.14438615 0.073598408 0.1176729290.083085809 0.153329807
0.1149379180.123138035 0.173756682 0.14025492 0.074722129
0.068435466 0.14858884 0.074922947 0.163483165 0.147913559
0.120782786 0.066781499 0.128740748 0.1351153630.147556403
0.149520962 0.109463017 0.144682626 0.082601041 0.06209685
0.122009663 0.159096805 0.0271107860.140186825 0.032743414
0.139063657 0.135176409 0.137749804 0.144427866
0.136130801 0.157221472 0.102432618 0.1123629640.122603439
0.124453597 0.022408209 0.148462469 0.076725427
0.193516044 0.135799687 0.103458918 0.093701868
0.146701325 0.163014312 0.164512574 0.074018234
0.098866385 0.123717915 0.171310002 0.124198699
0.0494511380.109616029 0.192850634 0.086302053 0.066022478
0.098166457 0.136628009 0.13199566 0.079024707 0.181660785
0.1133436370.0742114770.159673408 0.088255813 0.180393359
0.065474206 0.131221855 0.141344748 0.168205771
0.077479458 0.135777965 0.12997382 0.129202394 0.074015021
0.148185912 0.080770339 0.129226023 0.087953532
0.133767902 0.130960602 0.147708779 0.095076559 0.13158979

0.158498928 0.179044407 0.101295481 0.1140249680.085785144
0.1751174340.103293906 0.001970673 0.061415677 0.099603825
0.072209947 0.104200894 0.169044371 0.0611461150.08538345
0.123875588 0.093558469 0.089524399 0.082459921
0.120921295 0.1146209990.127623203 0.167590067 0.06590828
0.1154020540.157274467 0.047099002 0.175241855 0.089579001
0.150394954 0.137205103 0.15504226 0.180103372 0.058613267
0.166966391 0.134754586 0.1216381 0.105982589 0.162864116
0.19235639 0.172865426 0.127077354 0.180566492 0.103324805
0.205403094 0.153277822 0.126469283 0.154227434
0.101954199 0.169355335 0.145245156 0.148442062 0.16360956
0.175733102 0.096364763 0.135329474 0.139276668
0.129856452 0.158458365 0.103848269 0.157928784 0.1021955
0.176437305 0.173631966 0.101069663 0.159771097
0.136482667 0.209496794 0.18031372 0.1123898770.062339445
0.220493346 0.108042275 0.082746137 0.132540706
0.162158957 0.145531642 0.178131894 0.186552204
0.125630612 0.122959967 0.133384386 0.144084216
0.160381866 0.167873979 0.166900256 0.145892595
0.148569912 0.130812545 0.24066327 0.108739374 0.168409752
0.14486138 0.149036018 0.050917024 0.183508385 0.163843436
0.0664411040.159361312 0.06692305 0.099759051 0.076313142
0.173966766 0.178093365 0.081012382 0.10724489 0.097867962
0.1137104320.1123358470.1157298520.101943185 0.141261012
0.1459681 0.006946676 0.071670785 0.062008832 0.148143899
0.168524328 0.158304746 0.143059176 0.131582624
0.186855441 0.17663099 0.0735947110.094177414 0.189104063
0.092765575 0.1121057940.139066073 0.145980808 0.1829329
0.092006007 0.169948212 0.076370299 0.191845846
0.152926841 0.146789147 0.204235528 0.181928256
0.105632008 0.033094689 0.107978475 0.046868843
0.159825044 0.1163149890.1505981160.1188087620.077842014
0.121368525 0.07148999 0.04065297 0.058657121 0.031779042

KEGG_GLYCOSPHINGOLIPID_BIOSYNTHESIS_GLOBO_SERIES

0.102222394	0.1342511890.096939441	0.086649005	0.152482892
0.043002179	0.040076131	0.105713829	0.134571589
0.1211720290.0511549160.091897903	0.0229011730.054345195
0.076891049	0.070412676	0.061094752	0.151239944
0.122244323	0.029108136	0.017623647	0.140273364
0.1188438860.1102554590.101699177	0.044618087	0.088797683
0.088884136	0.046589429	0.1251184120.043947928	0.151672842
0.070592328	0.046809349	0.073628038	0.064894959
0.064689139	0.1100414070.095651545	0.075374224	0.116167189
0.093941932	0.041334185	0.136620066	0.126554169	0.14427704
0.1155893990.093896644	0.135375108	0.090217065	0.110672407
0.090262345	0.069713225	0.032022979	0.100676207
0.103347222	0.073369252	0.101662246	0.125607958
0.049523128	0.10789429	0.152081946	0.101588956	0.178816892
0.059992682	0.035690446	0.1107267310.141079996	0.143081867
0.087470798	0.153214843	-0.001391574	0.096138698	-
0.017084254	0.163850073	0.11810488	0.103314051	0.101272591
0.131817034	0.049328645	0.12503858	0.074463223	0.088195854
0.0995113860.066813435	0.021241063	0.109663133	0.105587293
0.097282586	0.047102445	0.152636758	0.1166842140.09861038
0.072935731	0.125941051	0.100475245	0.032480956
0.076151636	0.156017577	0.1197674950.128077506	0.106104529
0.1493740110.126227663	0.092060374	0.129747625	0.065309571
0.134962182	0.1197465840.063650469	0.155503244	0.096290612
0.0320304110.153598967	0.095748017	0.16160233	0.077563411
0.122003846	0.1173239740.095679806	0.061228131	0.072016066
0.122071227	0.075734126	0.108325886	0.100405965
0.067887232	0.05529842	0.132440855	0.102125289	0.088613031
0.101399787	0.143019197	0.1197855340.083418539	0.086850208
0.098010386	0.080492999	0.038221013	0.11352517	0.066500843
0.124487275	0.105285813	0.040725439	0.077406764
0.1126775380.1155154820.07609905	0.040383718	0.1128032

0.121551554 0.031813484 0.087937561 0.04645963 0.120099736
0.111305344 0.115128851 0.114412806 0.101225641 0.169977252
0.12687904 0.059951475 0.107869857 0.131405992 0.159119813
0.143906454 0.102135052 0.080175811 0.090424205 0.075645618
0.076910173 0.065302467 0.096958645 0.120715575
0.133893425 0.180130303 0.126824202 0.09330594 0.143722578
0.150064206 0.119480464 0.096044414 0.134894792 0.112500178
0.040286135 0.156538816 0.08531732 0.103966248 0.032183557
0.112337633 0.117311257 0.083527821 0.105630259 0.126707733
0.129230551 0.079821816 0.081150174 0.093816933 0.093682607
0.16199622 0.167048028 0.062641629 0.111268851 0.056929034
0.107716368 0.049862238 0.04507304 0.061316261 0.131458547
0.051828722 0.098024063 0.081745961 0.109497653
0.085731451 0.170657436 0.136339398 0.172526303
0.102521259 0.125645345 0.133070232 0.094142837
0.148908127 0.175777171 0.109759332 0.0711504 0.021868654
0.181784901 0.085728317 0.060368737 0.155127481
0.124225605 0.130265037 0.040737265 0.113418797 0.081646292
0.060801176 0.121528121 0.132104569 0.13790099 0.127427229
0.149762049 0.202332644 0.07518159 0.097691038 0.143783738
0.100469343 0.157734363 0.077461358 0.179193655
0.046513739 0.15092953 0.106396132 0.102978607 0.098498782
0.07734716 0.115804619 0.094345608 0.134239734 0.124610947
0.104297386 0.09296378 0.126599122 0.110321102 0.047948493
0.114575802 0.075813007 0.130829041 0.129484572 0.070821289
0.072339775 0.152862501 0.151710055 0.026334014
0.034970588 0.111113835 0.083145823 0.15423963 0.148486219
0.115538468 0.082384726 0.069144615 0.104000091 0.121305465
0.182774056 0.136697308 0.128814116 0.107582506 0.105755538
0.150276813 0.136178916 0.143408978 0.117534717 0.116989301
0.050849769 0.185966642 0.113935508 0.075647049 0.108168724
0.094762835 0.093680907 0.060239916 0.16342529 0.138023894
0.057645233 0.128066094 0.079680196 0.10655866 0.134973348

0.1339132 0.087895039 0.1171610480.14678014 0.068854511 0.122980258 0.024245078 0.125679806 0.157198178 0.12863438 0.1177114430.066793238 0.143109062 0.134869595 0.031349289 0.070348887 0.132730131 0.056200519 0.145629395 0.1198381140.06361843 0.099329109 0.083393163 0.10152197 0.100502419 0.1100324370.120312938 0.129735726 0.130919017 0.169203572 0.019796109 0.083294762 0.1654031140.073954079 0.104303795 0.087762705 0.083459825 0.102476853 0.095658255 0.1137808560.077305369 -0.017527302 0.070320849 0.010352505

KEGG_GLYCOSPHINGOLIPID_BIOSYNTHESIS_LACTO_AND_NEOLACTO_SERIES -0.015798941 -0.000557155 -0.006410914 -0.002368247 -0.007357247 -0.024479018 0.012951075 0.044932947 0.083863987 0.066828224 -0.001575909 0.001985148 -0.019631857 0.015336704 -0.017137362 0.013243857 0.000763873 0.015350253 0.035127205 0.010713323 -0.004478386 0.015060599 0.060864724 -0.016001678 0.012465627 -0.007764781 0.0391594 0.019612539 -0.068536168 0.061115404 0.006767208 0.018968684 -0.044701212 -0.037727069 0.019814996 0.012990169 0.009101844 -0.006047905 0.028586772 -0.016221565 0.023102026 -0.008379153 -0.000625237 0.035844992 0.069296649 0.040660407 -0.024088124 0.034432192 0.017432333 -0.031417293 -0.037587972 -0.013985577 0.035529833 0.005160528 -0.003381205 0.013385678 0.027523657 0.02179465 0.038336742 0.020770489 -0.016422746 0.064490992 0.004532538 0.011701094-0.023921383 -0.002108697 0.0100634110.021691466 0.060180176 0.012567747 0.061971359 -0.019582549 0.002545596 -0.011380507 0.020638899 -0.010134327 0.01493756 -0.01574466 0.002109918 -0.016307326 0.050615868 -0.017740773 -0.020324672 -0.038059913 0.01849631 -0.01516033 -0.022155458 0.01878329 -0.004402219 -0.028747725 0.014486912 -0.018567957 0.012137972 0.00252842 0.006224573 0.035616954 -0.026822579 0.005239752 0.001153987-0.015415517

0.062693134	-0.025987529	0.080570845	0.040382858	0.01871249
0.022407302	0.013288451	0.030835052	0.00563825	0.014805787
-0.000738426	0.024761737	0.002320532	0.0009785	0.026039618
0.022599911-0.007759083	-0.016724656	0.028736531	0.005475562
0.014457126	-0.008928811	0.040771042	0.003257435
0.002898916	0.021453649	0.0209285110.028629843	0.00756136
0.049226651	0.016232457	0.021217156	0.082897272
0.032456562	-0.010836184	0.005101274	0.015350917
0.052746506	-0.016147004	0.00178147	0.024978789	-0.01435134 -
0.028284564	-0.007329764	-0.002164125	0.004673304	0.043033445
0.015872326	0.050492593	-0.003469373	0.038776437
0.025252755	-0.034292108	-0.01421716	0.038086716	0.009396771
0.012177433	0.003405677	0.027758825	0.061347022
0.046556582	0.009338271	0.033182358	0.019281553
0.0116712470.029568486	0.054369365	0.010002785	0.016735534
0.04395724	-0.0131171	-0.004892304	0.036026132	0.015718914
0.036484108	0.025407349	0.017450234	0.031841575
0.028061505	0.017409587	0.017048947	-0.012150576	0.03377841
-0.005090085	-0.03852067	0.043491186-0.002949571	0.024259501
0.0115524890.047846225	0.042536499	-0.017241861	0.008485634
0.03225808	0.046942081	-0.032868889	0.028565087	-0.030954388
0.002107571	0.02323022	0.029248966	0.033310484	0.018111631-
0.002072109	0.049287606	0.013679802	0.037753014	0.013265783
0.010094746	-0.016861145	0.013416155	0.012134055
0.030718091	-0.007549654	0.010802573	0.002599253
0.008272661	-0.011064124	-0.000615252	-0.011432393	-
0.031094217	0.024219408	0.015051479	0.043738444	-0.0075913 -
0.000645626	0.004491667	-0.034074929	-0.039459433	0.058630614
0.04657522	-0.035071927	-0.021311735	0.001449828	-0.014895791
0.007078688	0.026943464	0.002171422	0.020846067
0.064249978	-0.051073691	0.043037997	-0.013532787
0.048322946	0.0686057	-0.002043805	0.05435803	-0.001816675
0.044559174	0.01360891	0.000573761	0.01204749	0.001711463

0.043917537 0.0040211830.032367726 0.016084087 0.01490012
0.030575032 0.026692178 0.007918781 0.04512695 0.006422064
0.006881747 -0.007653564 -0.008464148 0.017492231
0.039150739 -0.025976306 -0.010753388 0.013032523 0.03469074
-0.026635515 -0.000540878 0.007870103 0.00510652 0.037296585
0.031895192 0.0203511810.055199964 -0.000729147 0.03871994
0.055861392 0.033400694 -0.013715104 0.024656282 -
0.025766123 -0.005950931 0.01038487 0.0630396110.034641797 -
0.016176027 0.02743891 -0.04956566 0.031817178 -0.011351727
0.01788006 -0.033137261 -0.018163915 0.027419222 -0.01544186
0.072838731 0.020320963 0.052766098 0.025556302
0.019327568 -0.009754601 0.003920245 -0.012497099 -
0.016106934 -0.016368767 0.06733823 -0.010400232 0.018687818 -
0.013074476 0.034055709 0.021109633-0.007116534 0.038413982
0.016517931 0.0211315460.006691351 -0.018718991 0.030932858
0.033645564 0.023658243 0.052280235 0.0369862110.028294933
-0.000383917 0.0115750440.036596915 -0.010177444 0.019542931
0.0231221150.016163765 0.022092188 0.000213498 -0.007260579
0.05969884 0.072539338 0.012328166 0.034210456 -0.025109009
0.04020906 0.083133255 0.052904407 0.021390013 -0.021926116
0.01273522 0.076402873 -0.029294346
KEGG_GLYCOSYLPHOSPHATIDYLINOSITOL_GPI_ANCHOR_BIOSYNTHESI
S 0.124104092 0.11448883 0.127539122 0.169314795 0.131877241
0.196222165 0.136262238 0.147798905 0.098405241
0.108063475 0.15626002 0.132461314 0.135929196 0.138185394
0.1103653960.127368999 0.151763848 0.129897363 0.15762053
0.144634959 0.148857418 0.139583412 0.167168681
0.165230437 0.132150323 0.14900217 0.141596016 0.126287978
0.1169104630.159523488 0.197256584 0.173620676 0.15873097
0.149751538 0.120719528 0.121608548 0.137768452
0.146876146 0.173144866 0.16198731 0.075233007 0.112072798
0.147928533 0.152207679 0.153850853 0.125837854
0.124058244 0.130344355 0.12660154 0.129027293 0.109605152

0.136629486 0.164957865 0.145703099 0.1185492430.15098222
0.133676793 0.105166152 0.140854725 0.16281557 0.124192273
0.166213192 0.130516975 0.126652558 0.163849074
0.171667445 0.163874423 0.1177469370.153895566 0.17768327
0.1150816330.136786606 0.158212591 0.121242264 0.159145651
0.123084483 0.136967404 0.138302235 0.1195808240.109287202
0.136183337 0.168799284 0.1615131150.128436573 0.139861314
0.177700792 0.11275038 0.109656372 0.1191968560.145004138
0.1151880880.142926348 0.109756837 0.1127646470.125006714
0.164890606 0.180053141 0.1135228980.146924839 0.136062241
0.131962898 0.138680435 0.16041674 0.150385246 0.16879717
0.13362929 0.155665027 0.149096963 0.140563732 0.123004604
0.132576191 0.148536396 0.184652447 0.1114258860.124526074
0.128832726 0.133040484 0.140926075 0.140970443
0.139199042 0.156768625 0.131868172 0.164581236
0.109356686 0.134077879 0.161889696 0.104394322
0.134607843 0.173070301 0.133720451 0.136843742
0.130273173 0.121391742 0.150500526 0.1157044020.170820528
0.12228856 0.145045537 0.132786652 0.1142594050.13399948
0.1184735440.18066857 0.176265004 0.154084064 0.117189906
0.147126462 0.121465405 0.1186446950.1174193630.122513928
0.141207226 0.125409319 0.180979226 0.109934456
0.133050341 0.148524062 0.124022701 0.142317247
0.107062964 0.133085599 0.157842594 0.13776096 0.106890962
0.145716085 0.152231355 0.17648208 0.146502723 0.167399828
0.153923566 0.13672979 0.142128993 0.17232829 0.1755406
0.172009339 0.1465901160.138476559 0.092862615 0.153030278
0.1491929 0.157326616 0.15657842 0.149978617 0.131017968
0.181779907 0.153216974 0.1228859110.107923337 0.122676174
0.135025457 0.145914772 0.165258193 0.142349655
0.105365962 0.1142288080.140972816 0.10840252 0.147203551
0.142471604 0.1193491170.1155646080.158824376 0.139785415
0.149900585 0.125975666 0.166732864 0.133093471

0.122009483 0.146619391 0.1634451170.133449106 0.157292371
0.1134107510.136541262 0.14430738 0.1177687890.15185504
0.105786783 0.105890356 0.164898567 0.133317153
0.108073996 0.144213458 0.138713078 0.133260105
0.1601155350.147017349 0.127852244 0.149812332 0.100861103
0.186618601 0.159349636 0.148280087 0.1511322970.117490402
0.124399983 0.16865116 0.137424672 0.1395774110.14647405
0.1177726510.126437613 0.2117384770.187040161 0.114871528
0.140649529 0.179126551 0.169065609 0.155826705
0.161445439 0.132142654 0.144155062 0.15116836 0.147259815
0.140041766 0.125980999 0.161473556 0.13883138 0.146734382
0.1126255380.139323768 0.157550999 0.143658842 0.160312407
0.156890804 0.145432529 0.181936738 0.133909364
0.142204532 0.140086019 0.128904689 0.137094476
0.120203952 0.157559497 0.1172338220.134041643 0.129315123
0.146050286 0.187598473 0.12609255 0.136473101 0.122212547
0.1116355880.142031828 0.156865326 0.153252029 0.124965529
0.106904861 0.130304718 0.129377971 0.1271168220.145708407
0.168834415 0.166824487 0.151414899 0.136862223
0.173122836 0.138089073 0.163869266 0.191318899
0.135499643 0.1711225230.13478502 0.152306482 0.123017155
0.164496862 0.1155036420.135002822 0.108412476 0.152485118
0.1152724420.130736165 0.143757315 0.141866519 0.158066211
0.10936828 0.182879087 0.176634882 0.139884824 0.185879986
0.148260782 0.173442655 0.141737309 0.18693773 0.182571529
0.120552346 0.141715467 0.173008952 0.151629469
0.136797781 0.109190305 0.145874645 0.168966424
0.140347136 0.1160933310.164289193 0.146992879 0.171826534
0.139156288 0.187536203 0.101015775 0.139361002
0.163790805 0.167789061 0.135677086 0.132823591
0.089034872 0.147036568 0.1144238440.156102594 0.126661995
0.109712293

KEGG_GLYOXYLATE_AND_DICARBOXYLATE_METABOLISM

0.2112904810.217998666 0.196573889 0.269068197 0.251464147
0.236748784 0.232676248 0.213632448 0.222327979
0.186215457 0.2341411780.21976616 0.215014043 0.20304305
0.221690697 0.238162577 0.215136151 0.201447515
0.203484241 0.18515911 0.271980673 0.222738058 0.242908775
0.251999024 0.24696353 0.205067548 0.246032885 0.22160611
0.216129462 0.212698736 0.235798325 0.238228562
0.221629407 0.212924875 0.220255959 0.248042682
0.175492916 0.249192499 0.245930351 0.221684203
0.250876486 0.242849294 0.236620508 0.2519447110.263407735
0.215965335 0.200320242 0.232785421 0.2667488110.252807369
0.222028385 0.216451795 0.265289883 0.207510979 0.23264035
0.220435791 0.231274993 0.22627538 0.246377365 0.203358588
0.246755322 0.25729465 0.214885469 0.253845031 0.228860341
0.2917112760.244432973 0.232773073 0.255892821 0.255970766
0.216963203 0.24251542 0.214955597 0.190284935 0.221954345
0.220437754 0.238601963 0.235944123 0.191797361
0.247952266 0.247954421 0.22779254 0.226430913 0.241541856
0.218920818 0.235845334 0.226969176 0.186393849
0.227631502 0.246647651 0.246435935 0.245250473
0.258229827 0.238857548 0.220152741 0.213390983
0.241871402 0.222062733 0.246345852 0.243326898 0.22885775
0.201795399 0.239483173 0.241401355 0.261034656
0.235919192 0.256340127 0.252396129 0.231228735
0.229154265 0.26679507 0.241940214 0.258271749 0.186407898
0.228383057 0.243062982 0.237885994 0.226709988
0.230017834 0.2144161110.217874956 0.23188232 0.223702825
0.245974083 0.247185509 0.254164408 0.217334645
0.217161213 0.233703871 0.222403363 0.249905555
0.208462779 0.217608896 0.219348861 0.218603375
0.218303529 0.21694409 0.184524574 0.219197242 0.217170969
0.203486608 0.210917575 0.287608008 0.219788668
0.255250254 0.197291768 0.238197341 0.22869634 0.2259485

0.237996624 0.225746961 0.232978848 0.235390885

0.2117145320.2110080160.23432513 0.177650314 0.25242795

0.246934251 0.202148854 0.206948594 0.232847801

0.206961649 0.206436472 0.224598513 0.198537735

0.217420658 0.216833886 0.1978181 0.238289742 0.253306967

0.242195468 0.232491508 0.260174447 0.245865794

0.228097045 0.248572628 0.194946536 0.2784691180.235433054

0.21233624 0.238558614 0.225431096 0.232702894 0.231486266

0.23708101 0.245860875 0.203722072 0.20439574 0.188485428

0.205700826 0.239374944 0.210748186 0.226651064

0.2260501130.218140123 0.261513893 0.231513748 0.21612187

0.252847487 0.215386506 0.242901026 0.237300823

0.216699609 0.2117290860.240432308 0.178783342 0.209309297

0.213664767 0.237065957 0.195904678 0.200760878

0.209024258 0.21235327 0.234372698 0.214382066 0.206191365

0.235497419 0.180276689 0.217247248 0.174048544

0.224600152 0.220720088 0.241483109 0.216664173

0.207422789 0.243447721 0.175830309 0.23395674 0.193376049

0.226968616 0.201246847 0.245864593 0.225132016

0.214000088 0.240182074 0.214029371 0.2281596 0.207023153

0.222564722 0.244670747 0.21503535 0.24308956 0.235912259

0.195287613 0.218778084 0.226806396 0.26001398 0.262981397

0.238379873 0.227532518 0.177552297 0.203824682

0.259959045 0.270788978 0.209293818 0.218286543

0.230199305 0.242923259 0.236646158 0.251692232

0.234732979 0.247625251 0.232889978 0.228982679

0.198106688 0.261226381 0.215408556 0.224324254 0.24094458

0.2262114740.243069545 0.186613229 0.262728306 0.216296392

0.241403085 0.202179918 0.241606687 0.2230836 0.23636289

0.25782425 0.230269054 0.219230578 0.21477007 0.237812713

0.226954566 0.210515944 0.2095116530.243322104 0.21812484

0.2401193660.178818943 0.247714966 0.222942961 0.229647084

0.219732034 0.2669115360.247795362 0.202178036 0.231500871

0.223326714	0.247313814	0.236503767	0.233135521
0.204120618	0.186555952	0.216992809	0.250378046
0.230041464	0.205445246	0.231084614	0.22101811	0.213419149
0.250486643	0.22757585	0.20833012	0.226833078	0.26682353
0.236929442	0.182054767	0.231307732	0.22865603	0.225412491
0.217439085	0.245186593	0.235591736	0.196223882
0.258642665	0.224786599	0.242218451	0.215591848
0.219995412	0.238908131	0.253863534	0.195702435
0.232969979	0.230342544	0.24719966	0.194335576	0.297265555
0.193490579	0.198500966	0.234237785	0.200782191
0.2116321080.220774827	0.219000195	0.231479923	0.196717922
0.229408582	0.20776458	0.224834846
KEGG_GNRH_SIGNALING_PATHWAY 0.089027757	0.114353754
0.108975656	0.083849609	0.091490817	0.065559912	0.08358064
0.10063295	0.086486476	0.090626071	0.065510581	0.08649886
0.082495602	0.085156933	0.090252964	0.085285884
0.070707944	0.087558807	0.10210448	0.083177766	0.094649654
0.095606605	0.068082025	0.095067766	0.102802376
0.082324649	0.067736841	0.090055405	0.077288875
0.099049058	0.057988413	0.102681555	0.077756671
0.099636365	0.09858023	0.0791119240.082028996	0.081101376
0.066468079	0.072936675	0.095980885	0.1102245860.084080817
0.083000254	0.094951766	0.08035188	0.082754868	0.08989576
0.080936165	0.071829071	0.100725448	0.070604618
0.090024087	0.093928657	0.081825792	0.088144896
0.078665498	0.07567421	0.061763539	0.083636368	0.066344678
0.090185869	0.086106008	0.083361768	0.079623336
0.069952548	0.075157939	0.100551783	0.0899021190.084238574
0.09321261	0.065699729	0.084556341	0.076746662	0.088329249
0.10565138	0.099557453	0.091547749	0.096158436	0.082920191
0.080734966	0.065755733	0.097151574	0.076126224
0.069976884	0.078729997	0.085035556	0.08954821	0.094658535
0.094109352	0.053680458	0.064460525	0.080595771

0.070526235 0.087602628 0.094059804 0.082601489
0.067522841 0.101760974 0.085730991 0.102428773
0.0964019110.09055355 0.092396513 0.062055954 0.109360053
0.081024197 0.080481655 0.065401096 0.087678066
0.068228392 0.085931728 0.081207491 0.086802876
0.078455665 0.09449537 0.0847595 0.073066848 0.09062551
0.093567788 0.084226752 0.077868232 0.080698272
0.105481841 0.103928074 0.06121471 0.070761708 0.082465285
0.0801125740.098335418 0.087961837 0.083260627 0.082732309
0.085878579 0.086298965 0.095587484 0.100950794 0.09460417
0.096142287 0.10793601 0.068924391 0.1107354080.077511292
0.076447725 0.079824336 0.088407156 0.089753184
0.075768253 0.083599061 0.081060877 0.1178543150.075016384
0.1114277570.062526136 0.095910358 0.075201072 0.105630275
0.093571851 0.0843011390.104773023 0.088329898 0.09890155
0.078249666 0.084890207 0.078081741 0.090931413
0.099012451 0.080199485 0.087855739 0.08932445 0.103439119
0.079033536 0.085827939 0.067832121 0.083777678
0.101647963 0.097164241 0.101971604 0.076338949 0.11388364
0.091335802 0.104683057 0.068516869 0.086608404
0.088130516 0.076202109 0.0811555650.109546933 0.101147093
0.087370749 0.11221777 0.101490934 0.106698465 0.101988353
0.106417457 0.099754513 0.088462163 0.101505642
0.1101418110.100693521 0.1130311330.068887958 0.082182766
0.080829964 0.097050791 0.084374504 0.098159059
0.097067728 0.102463126 0.089666768 0.088081675
0.0835131160.090601315 0.076291488 0.094342287 0.106971197
0.101240652 0.093206719 0.106226332 0.106660926
0.087713865 0.079727689 0.088502672 0.091657584
0.1181696740.088339767 0.101777065 0.077232435 0.050510152
0.1138685410.090709829 0.108941336 0.091688241 0.07450963
0.092663458 0.104837146 0.100496714 0.09627022 0.10896198
0.094153207 0.0753321170.089948076 0.072577359 0.06600558

0.1114899240.0938611910.063657063 0.076042685 0.062217281
0.087875781 0.094256494 0.0989178 0.09525991 0.062725956
0.077668346 0.08309875 0.072947004 0.084201623 0.067938226
0.074224433 0.067108287 0.083345349 0.059357173
0.089896704 0.0821169090.090935987 0.066263505 0.080059229
0.085489389 0.085759788 0.096281631 0.086348218
0.1124618580.050049537 0.069542802 0.079174481 0.099013506
0.090837831 0.086255989 0.087422885 0.052555688
0.091289529 0.069250035 0.1147322020.0901188760.076642771
0.100333943 0.102863361 0.108585005 0.1031108660.099565662
0.109058218 0.071547961 0.091254866 0.080909482
0.089247701 0.079725749 0.094090924 0.1104327040.079617439
0.086300581 0.099228244 0.108394399 0.081977341
0.099801202 0.093237933 0.105393229 0.084844669
0.091393155 0.098918028 0.10794037 0.096631532 0.079784504
0.085159477 0.104430012 0.137074249 0.09198116 0.077875838
0.074159251 0.077790596 0.084842618 0.068365947
0.081278749 0.09311805 0.074231663 0.08608756 0.084712414
0.068352097 0.091095216 0.078580679 0.1166701540.084394783
0.0841959 0.089555296 0.10777748 0.101289183 0.080184215
0.091024254 0.126991262 0.089841543 0.1158127150.091786704
0.096915948 0.1097962110.097043954 0.079981447 0.08066295
0.107246472 0.100580584 0.1091170830.089336253 0.078849953
KEGG_GRAFT_VERSUS_HOST_DISEASE -0.047629929 0.010318808
0.068510327 -0.015159035 0.060225468 -0.051142749 -
0.0741105110.004395829 0.082766932 0.021797647 -0.094146113
0.10813531 -0.140282306 0.00177154 0.096225038 0.027366276
0.06212386 0.120166785 0.018105825 -0.008169205 -0.147313734
0.067585405 0.10514018 -0.050576394 0.097305515 0.078572824
0.055904169 0.056654708 0.027569442 0.039275511-0.013882491
0.107894792 -0.043500384 0.008068015 0.045231898
0.051381459 -0.042436821 0.070256848 0.113893175-0.106251494
0.0444113770.001456379 0.053514048 0.061594085 0.037128853

0.029036341 0.0318738 -0.001334653 0.1133543940.029746089
0.106486432 0.104370151 0.063382388 -0.108217426
0.101575342 0.0711822710.1144411480.1086119790.089481204 -
0.08247069 0.08592343 0.10231052 0.0118183660.10967139 0.127648247
0.018231963 0.079269003 0.08963996 0.066936962 0.06650433
0.055681563 -0.086169169 0.021302325 -0.007009843
0.032953376 0.109043921 -0.038386984 0.08014578 0.003293673
0.007850322 0.117759135-0.068315969 -0.039052753 0.011876533
0.130182496 0.016943699 0.146943641 0.080394104 -
0.014409917 0.058538496 0.056647862 0.108362297 0.034205533
0.108542661 0.050506468 0.055315595 0.091343449
0.018131723 0.14492997 0.126081832 0.02646592 -0.009989001 -
0.024102506 0.095466213 -0.048040309 0.109278393 -0.004517784
0.08115752 0.121816308 0.064154088 0.019570816 0.070469744
-0.145619904 -0.062864414 -0.024648256 0.077303076 0.08345586
0.062423939 0.105024073 0.0116878660.079044257 -0.047801055
0.085748488 0.028603278 0.036243147 0.06436028 0.07631151
0.038520043 -0.01980546 0.0743316110.034388377 0.080145148
0.024719876 0.1081194220.018161914 -0.031987657 0.118480576
0.069232045 -0.016182035 0.060914427 0.003808676
0.065817045 0.087638233 -0.016284031 0.062495796
0.089977098 0.093412163 0.053981218 0.024470048
0.050929355 0.1128026930.06148357 0.05216061 -0.065874248
0.071969891 0.143797545 0.0311065760.127023767 -0.031909304
0.127710196 0.044780448 -0.003374274 0.005656417
0.079737849 0.1100878030.108421819 -0.036435995 0.085822054
0.052631436 0.0200161 0.101064054 0.067109889 -0.01184304
0.043047341 0.133241977 0.101972687 0.063120878 0.07284581
0.100252088 -0.004213603 0.107311411-0.091497892 0.049552
0.080526148 0.082039364 -0.069409853 0.066878742
0.073417921 -0.027278617 0.03332546 0.066510294 0.11514513
0.059544682 0.099881625 0.083063628 0.018474233
0.1123330620.053868966 0.053203385 0.055481299 0.081189704

0.023326646 0.036751574 0.061271796 0.060431999 -
0.096982479 0.055315822 -0.009687494 0.046243906 0.086797254
0.0247117850.033946506 0.001445053 -0.054541473 0.065048714
0.065527206 -0.029845865 0.103666764 0.076321523
0.007473272 0.064680547 0.059872601 0.032183826 -
0.03479165 0.086321293 -0.069378253 0.052131392 0.093601228
0.042629052 0.037995092 0.096524479 0.076696876
0.035950232 0.070903629 0.074716222 0.103247805
0.029823465 0.086710913 0.040634198 0.096948095
0.086829251 0.092494418 0.058756937 0.067993898
0.0856565110.0811184890.055440498 -0.068028933 0.063073512
0.052721723 0.068314018 0.148613418 0.104496622
0.077596575 -0.046269724 0.072852298 0.046762598
0.093171952 0.031755775 0.042564286 -0.095721977
0.043473408 0.077164867 0.0584400110.103862756 0.066974389
0.102068787 0.14844063 0.029603463 -0.011102327 0.148106221
0.089647679 -0.024337273 0.075121285 0.035463351 -
0.050764746 0.094466049 0.138828659 -0.005675446 0.029418324
0.045973976 0.061809176 0.0119040770.0351011720.156699242
0.1120373670.082061697 0.14624713 0.152038523 0.07733567
0.072004318 0.092791252 0.09432548 -0.073731046 0.084207745
0.072337791 -0.050464379 0.109482993 0.105194085
0.000928024 -0.011661625 0.074362189 0.085419076
0.049623487 0.017140048 0.087329582 0.01250836 0.022051331
0.047478222 0.071078242 0.100683361 0.053049179 -0.0258717
-0.010783189 -0.071850563 0.038019962 0.024107427
0.087480802 0.052461764 0.006732634 0.084149343
0.095614461 0.046711846-0.017251895 0.131747382 0.019102767
0.095857212 0.065552969 0.099852891 0.098393682
0.070891456 0.065281434 0.1199281880.124773209 0.078908541
0.095095623 0.075497331 0.090036242 -0.095451463
0.0111443840.054719292 -0.116444724 0.017507966 -0.04404687
0.124905265 0.064128852 -0.004933869 0.0348154 0.031495811-

0.086112301-0.02696914-0.030515972
KEGG_HEDGEHOG_SIGNALING_PATHWAY 0.004160079 0.058645293 0.005558723 0.025263304 -0.017823713 -0.003894873 0.04025303 0.0112187810.003556578 0.025118625-0.006871409 -0.026693738 0.057210194 0.02330266 -0.007370814 -0.013687513 0.026303983 -0.01933186 0.017369407 0.049572855 0.082005799 0.014581664 -0.013638136 0.03260396 0.013855722 0.01340547 0.030448966 -0.002090337 0.042852746 -0.005827093 0.042561484 -0.000716621 0.062094299 0.075051512 0.042741655 -0.050799868 0.060958184 -0.018630796 -0.011585237 0.015431093 0.005281229 -0.044051521 -0.014704954 -0.033675873 0.013806406 0.00476692 0.061472506 0.016394987 -0.046911862 -0.015982821 -0.029182895 -0.038883157 -0.035746332 0.009866198 -0.044349173 0.0616708 -0.0280442 -0.01476912 4.65E-05 0.078499279 -0.064266698 0.0035116250.040517505 -0.027736793 0.007712698 0.00864888 -0.033073083 -0.043199624 0.026254378 -0.000454981 -0.019307844 0.036560698 0.030108642 0.040813804 0.013029594 -4.97E-05 0.051733509 0.011147879 0.042330851 0.032894834 -0.039423819 -0.005948764 0.006820275 -0.000966365 -0.049593597 -0.017250833 0.001465918 0.039947527 0.005191598 0.060097798 -0.008616275 -0.018614258 0.018974802 -0.031180091 -0.039385462 -0.007427548 -0.032278463 0.037878582 -0.058626249 -0.054220364 -0.020365837 0.010841209 -0.020927885 0.010389955 0.024133046 0.007449739 0.052320969 0.008427142 -0.043279887 0.051859015 -0.039206677 -0.030227796 0.010395149 0.026083777 0.007617528 0.032687534 -0.041802426 0.005646423 0.005524074 0.004369242 0.019821229 0.0699611210.030570837 0.033745091 -0.026233688 -0.031960021 -0.016684174 0.022704036 -0.027915571 -0.031382157 0.030430583 0.0114691840.009461352 -0.028652536 0.025743577 0.039004403 -0.004953733 0.015796239 0.066859534 0.006240673 0.059732468 0.059829297 -0.027712051

0.011858845-0.047317538 0.021045049 0.023668805 0.006025783
0.009705009 -0.016210776 -0.031874872 -0.038110604 -
0.010837689 0.031580429 0.012041033 -0.005278709 0.062421803
-0.015552489 -0.064293721 0.026459557 -0.031986207
0.022673159 -0.002470556 0.024351931 0.020519032 -
0.015432649 0.102530526 -0.013800922 0.01772019 0.048536487 -
0.045800865 0.019985963 0.01671445 -0.074543115 -0.010260592 -
0.002043541 0.003722408 0.008180017 -0.044959539 0.047872203
-0.008783009 -0.044451369 -0.015062303 0.022929582 -
0.062171402 0.041015153 0.045725613 0.026153932 0.02999601
0.017608639 0.050789723 0.001592074 -0.035356129 -
0.021362744 -0.018708702 0.046954633 -0.001886555 0.011298818
0.044085602 -0.050680995 0.034286375 -0.003829636 -
0.034412455 0.036929263 0.00548175 0.05129628 0.031572062
0.049733294 0.009707718 -0.001160989 0.006648104
0.007036201 0.041639374 0.069352519 0.016176501
0.055765246 0.027366229 0.023135589 0.068664142 0.01402748
0.033979354 0.0084811340.0621148130.025789221 0.068975373
0.058709005 0.022325255 0.009307637 0.003330812
0.066251879 0.008892132 0.00058209 0.020172099 -0.036535815
0.0536046 -0.004187836 0.044972429 0.01363143 0.019198273
0.019842992 -0.061734173 0.026001073 -0.004586789 -
0.007665718 0.012812454 -0.016505014 -0.039500191 0.008691685
-0.04572958 -0.002297911 0.011284924-0.028589734 -0.031051536 -
0.036370172 0.022791026 0.034590162 -0.026111802 -0.007604098
-0.011625789 -0.001883388 0.010087509 0.037903149 -
0.018425996 0.01087306 -0.045322494 0.043475538 -0.032323593 -
0.035415953 0.033348335 0.04314181 -0.040774819 -0.023711539
0.050338891 -0.016358003 -0.006103682 0.044942071
0.007308312 -0.044300605 0.060815097 -0.00028035 -0.038779585
0.021577162 -0.00126096 0.048728097 -0.000441678 -0.001977677
-0.010442289 -0.031478259 -0.011022516 0.031213895
0.026252534 0.0353429 -0.026393839 0.007914161 -0.029315269

0.035536258 0.056448675 0.008323377 -0.041105509 -
0.018382398 -0.017621438 -0.020372684 0.03165187 0.020886047
0.015105233 -0.010393405 -0.054930211 0.01179924 0.001631117
0.076061683 0.025886171 0.030947059 0.04468296 -0.011306841
0.048448349 0.046189313 -0.028269696 0.012909257
0.025616432 0.062768005 0.02161271 -0.062714199 0.039400548
0.057638478 -0.037346588 0.014498349 -0.044025623 -
0.030655592 -0.005372734 0.02032449 0.027755688 0.012647133
0.010058677 0.004381274 0.051272077 0.040349798 -
0.002489444 0.027399238 0.04825898 -0.057355836 0.080503758
0.104664148 0.002982918 0.075113063-0.007272149 -0.009006619
0.017979773 0.019074542 0.053252221 0.035088123 -
0.019875888 -0.003455958
KEGG_HEMATOPOIETIC_CELL_LINEAGE 0.029018122 0.047156535
0.105982439 0.0339358 0.11337792 -0.016538464 -0.023060503
0.100628379 0.042466894 0.070573642 -0.037173046
0.106877706 -0.059902632 0.002590579 0.126493437 0.05399219
0.004972029 0.04953008 0.035674099 -0.006527267 -0.092205593
0.082076486 0.085848569 0.012840342 0.123420938
0.086227694 0.081080944 0.048485351 0.038018353
0.069633885 0.011180653 0.073385853 0.02934186 0.002516517
0.061629934 0.076298166 -0.0090035 0.047197487 0.101571995
-0.060319606 0.075759666 0.026140682 0.08292221 0.063258359
0.042563621 0.049190484 0.057240429 0.021008493
0.106286871 0.028970077 0.141020008 0.096667359
0.084955323 -0.052035377 0.098000584 0.120310796
0.136765969 0.091173987 0.083712859 -0.031102327 0.042153559
0.0670757 0.043553599 0.141852678 0.055826005 0.003140689
0.057036799 0.125379053 0.085537818 0.060349778
0.077695197 -0.035506986 0.059037392 -0.028245631
0.062962865 0.160007139 0.004318638 0.103119087 0.042400404
0.033728051 0.102941932 0.003953953 -0.006058887
0.042177565 0.091719371 0.072381062 0.110117969 0.117699647

0.025957969 0.038251487 0.066910664 0.101042335

0.075171565 0.025388423 0.077266227 0.077404667

0.078587589 0.049432735 0.122981657 0.163378168

0.069401245 0.0118775370.015778645 0.059111153 0.006348093

0.083870371 0.000167933 0.1173800850.095886092 0.08569983

0.050172953 0.112849492-0.060301492 0.025058161 0.02107799

0.137246468 0.090475636 0.08009562 0.096157528 0.04451027

0.077473771 0.037526887 0.075092505 0.099382656 0.06821494

0.0411825310.060417328 0.037018766 0.007450405 0.072853275

0.043782143 0.043823046 0.049121377 0.127393099

0.052585917 -0.003382427 0.0835511980.05642712 0.038378521

0.084450149 0.027047505 0.088592238 0.1168383580.005056304

0.050634017 0.105094064 0.120342016 0.060512437

0.037067524 0.073591898 0.1166924530.072580316 0.032125631

-0.020289999 0.092028457 0.06134718 0.068900163 0.115055682

0.022941672 0.129421961 0.053377344 0.035819676

0.021023849 0.122835688 0.1244011270.071429969 -0.008676096

0.056168346 0.035289855 0.036647676 0.107337984 0.10792119

0.009532506 0.079134558 0.1149034080.135519192 0.073641785

0.08282016 0.123854091 0.055644759 0.126709201 -0.010180603

0.083136673 0.071975991 0.08936697 -0.004874523 0.045649206

0.129823206 0.01127033 0.062372084 0.1172192520.098626741

0.126014925 0.121495464 0.13457281 0.060276085 0.133323142

0.10505615 0.096717571 0.130793068 0.15048587 0.03868807

0.046293459 0.046395265 0.070809962 -0.047576167

0.044357876 0.034523933 0.058698701 0.044237377

0.087423718 0.044416166 0.078860417 0.010665469

0.095195385 0.088586 0.041415857 0.140268592 0.151026286

0.045914757 0.086091832 0.135147066 0.049127323

0.018574173 0.11534825 -0.042568474 0.059514217 0.068621903

0.022024982 0.087221927 0.082688937 0.081048197 0.05098504

0.075464213 0.100996775 0.074120646 0.023069848

0.127347813 0.061074578 0.077710436 0.120848494

0.126059405 0.044485218 0.059478322 0.07859682 0.12809556
0.041438284 -0.000171461 0.083846375 0.039892028
0.075331573 0.159580379 0.127061084 0.068436275 0.03130758
0.054865155 0.082741481 0.096548051 0.085740609
0.074149596 -0.040672266 0.0401177350.0960093110.06885703
0.139909255 0.072008822 0.104030144 0.1104342670.006569968
0.023368934 0.1123224550.09919829 0.044633672 0.067200203
0.067677052 -0.033440386 0.108127922 0.1128295430.021879579
0.060830132 0.065278042 0.069861606 0.052620887
0.067451962 0.089033474 0.060848001 0.100850928
0.146474646 0.195527871 0.137876487 0.095091676
0.092412916 0.023033784 -0.029663586 0.105535877
0.142491623 0.014284328 0.11785089 0.101639162 0.03627873
0.0532964 0.055286498 0.1181329540.02296625 0.043376887
0.095638224 0.041423105 0.074827672 0.0811345190.072216297
0.098147386 0.07105353 0.051785553 0.066874234 0.030151206
0.079451989 0.040913224 0.047073488 0.048804695 0.02418117
0.122683692 0.045679094 0.056264819 -0.002447602
0.144692282 0.06655807 0.101047166 0.082254473 0.067256553
0.129781219 0.079005839 0.099570778 0.098762921
0.1356801170.154567278 0.076156208 0.093595947 0.144665079
-0.002693171 0.01079652 0.133670329 -0.043509129 0.030759195
0.034196834 0.101380325 0.08619261 0.047538321 0.085184674
0.09238526 -0.042944825 0.02900782 0.000275009
KEGG_HISTIDINE_METABOLISM 0.003422528 0.042820549
0.057062343 0.094860477 0.091047036 0.015858589
0.052649524 0.012334593 0.127463955 0.061408739 0.04390126
0.052526197 0.026659142 0.084577885 0.019883782 0.10113884
0.048946663 0.121013915 0.086012045 0.033435795
0.059102468 0.102978234 0.079267386 0.007587269
0.031540081 0.083739514 0.060194821 0.07269984 0.0591639
0.02001017 0.047710968 0.007441657 0.0175811860.050742278
0.073812442 0.089770557 0.023097069 0.0720110220.063177524

0.032890649 0.062705952 0.081943459 0.078102379
0.1115392750.005057108 0.03888681 -0.006582663 0.091466043
0.071323199 0.0711063390.077623765 0.075266366 0.04394262
0.075409924 0.0928330110.051719469 0.081544814 0.063064311
0.079244694 0.005482078 0.093814533 0.024030787
0.068897643 0.057553862 0.066418355 0.019810827
0.095365982 0.094170273 0.099634083 0.033346354
0.014122555 0.03415234 0.017409396 0.061853867 0.011034478
0.032670121 0.054553518 0.019980727 0.0391101740.029904426
0.090348233 0.080207647 0.043686484 0.048482249
0.029777262 0.034213904 0.03925662 0.057285491 0.0354539
0.039323477 0.104797456 0.03456015 0.083035253 0.076268177
0.022148425 0.017385875 0.0118972020.046592258 0.069521601
0.048883098 0.063009721 0.052022827 0.096296734 0.05571715
0.054345304 0.0791115990.102990596 0.047604897 0.05881302
0.065082023 0.091095898 -0.010845071 0.00317581 0.062344559
0.031057829 0.042584685 0.021643812 0.090607819
0.019282132 0.025984361 0.015669718 0.018933549
0.039776001 0.01784968 -0.001910986 0.073472739 0.02106534
0.022486385 0.072465381 0.051894479 0.031664444
0.022604545 0.067582861 0.018841067 0.025228624 -
0.003105443 0.030486991 0.066443279 0.0203595110.065141818
0.0411058060.03531286 0.0438211150.059824349 0.053356551
0.020592157 0.013024939 0.035949274 0.017155094
0.066063653 0.075669933 0.036767374 0.005568214
0.074546897 0.055200699 0.008933605 -0.018906922 0.0659175
0.007697542 0.0621127230.07270899 0.0611596440.058607609
0.058899496 0.060937203 0.130701061 0.063839368
0.051924818 0.068072513 0.125204215 0.019289002
0.076338385 0.060799601 0.053433709 0.054643766
0.026159915 0.024997366 0.084355775 0.073970526
0.036844042 0.042536156 0.061916093 0.006465365
0.054698613 0.025099795 0.058824967 0.066098316

0.038970009 0.078699887 0.065763527 0.049217225
0.028080173 0.044807012 0.041352591 0.049264733
0.020049239 0.021490048 0.030345365 0.030899178 0.05126228
0.089686759 0.049132616 0.035502659 0.050140327
0.030545831 0.069209639 0.032931896 0.043247589
0.048607899 0.056065094 0.01749861 0.045182605 0.04122888
0.051303152 0.071977824 0.028318498 0.042189145
0.066295026 0.062529786 0.059375323 0.036907579
0.070908917 0.081769218 0.024241316 0.020744619 -
0.019854035 0.0607221120.053778219 -0.004364931 0.025666518
0.082058416 0.02759267 -0.005204387 0.038720668 0.004267324
0.021898771 0.0989118060.056476851 0.034084599 0.048814283
0.028441786 0.077381936 0.072650919 0.0162711220.047416863
0.029353004 0.107234378 0.107658019 0.083262375
0.021688148 0.032485517 0.012731605 -0.003028548
0.054498001 0.053017404 0.036791978 0.06767678 0.100209521
0.048770178 0.036827936 0.060063747 0.035772586 0.06351929
0.055412542 0.060140055 0.055095976 0.075436989
0.029239105 0.029038997 -6.30E-05 0.103775047 0.085134542
-0.034735131 0.050921661 0.024729013 0.090152695
0.077614772 0.0814114290.043934625 0.038292937 0.025217823
0.059397823 0.074482243 0.051323657 0.045494887
0.087166017 0.0028011760.010058285 0.005254437 0.048135177
0.071730785 0.0115853390.021914069 0.045772781 0.052631879
0.050702708 0.038530785 0.028557566 0.069127548
0.018185446 0.061388947 0.089756767 0.029576802
0.058913774 0.098264985 0.046780365 0.034387489
0.096036596 0.04071727 0.053793246 0.043243975 0.07261038
0.014982768 0.000918663 0.007390747 0.060634776
0.056894666 0.071365146 0.070341093 -0.009877756
0.0098296110.077081094 0.041632858 0.01616452 0.066443055
0.03296579 0.074382625 0.085187139 0.04206388 0.022122567
0.063657163 0.037838446 0.031482761 0.052832299

0.046282713	0.01694242	0.06147241	0.053008828	0.058076224
0.163295304	0.063345872	0.029301644	0.018672698	-
0.019095475	0.054194692	0.087614317	0.132964897	0.05481649
0.00545372	0.020737109	0.0997505110.07491461
KEGG_HOMOLOGOUS_RECOMBINATION0.1187542270.093211971
0.128718154	0.141444547	0.104089712	0.155403579
0.142673092	0.148284607	0.1508891	0.133387734	0.14967331
0.162938142	0.123584781	0.0941149680.139058705	0.169798255
0.127495281	0.0668831190.152038635	0.109575426	0.143176384
0.078019641	0.1128546880.149489989	0.153227678	0.128206442
0.137942795	0.133092638	0.136855333	0.087010807
0.169954562	0.156244337	0.161887667	0.1133081090.131653466
0.1711299670.152056613	0.122640865	0.170064476	0.178739964
0.1153430320.128500282	0.129434798	0.152446337	0.101326569
0.125976547	0.1144971360.099537035	0.1168302390.177519752
0.09782907	0.130459423	0.183856651	0.1109614550.1595216
0.09462566	0.16875622	0.1187543560.143820376	0.113582697
0.148570213	0.135508192	0.137839016	0.124022587	0.15974759
0.155153305	0.133309462	0.133997506	0.105773454
0.168962719	0.1195764160.179855741	0.1196375650.12300924
0.141605007	0.103846276	0.1158991810.16623651	0.088722031
0.129084924	0.139684624	0.188833684	0.188291604
0.126150615	0.139917239	0.167440596	0.129533228
0.094107467	0.140527705	0.1186564670.177685037	0.130104708
0.138378487	0.107555408	0.075457364	0.178753285
0.167232802	0.160330295	0.126808459	0.1311778570.135151118
0.149967169	0.149496161	0.09374168	0.176647637	0.101597356
0.136487426	0.155284528	0.143325332	0.1153736430.161851889
0.155896206	0.157658552	0.133692099	0.145812254
0.1110496620.155837805	0.102718262	0.133875223	0.132537105
0.128291953	0.165636135	0.095222126	0.124995867
0.170771269	0.13249973	0.1145710330.124974363	0.157274564
0.099593124	0.1199447740.099791638	0.1134546860.130249486

0.127100496 0.1106228830.096774772 0.07480219 0.132873217

0.09689466 0.1119440570.103128537 0.19798007 0.1269293

0.167635455 0.092345948 0.101391075 0.1312671130.085126268

0.153846661 0.1354597 0.149740071 0.157761779 0.101696397

0.09462026 0.126338553 0.081220033 0.131624382 0.162882445

0.070026328 0.140284516 0.124161481 0.120832225

0.097984361 0.11663481 0.092704745 0.1175741190.141094258

0.1147830370.142042926 0.132697942 0.154018705 0.109292235

0.15368789 0.164748853 0.11096363 0.111125841 0.124332609

0.166273005 0.107758062 0.093439446 0.162214333

0.151069607 0.1014869110.14297153 0.178121423 0.154212838

0.131053594 0.10805525 0.085477046 0.1144725810.13419184

0.1611817240.122196556 0.095664173 0.101814665 0.122819035

0.1168621360.1027431150.1337983110.085623017 0.124100005

0.094269612 0.154043628 0.1152895720.127905996 0.127623148

0.104088176 0.074925335 0.121304219 0.097077278

0.121871623 0.124465771 0.1170378610.122694284 0.064741828

0.102589774 0.1136844020.051243857 0.13063952 0.119451715

0.099841451 0.087996645 0.081223039 0.036867592

0.1194418160.1123089320.071493894 0.149946732 0.048849957

0.127674022 0.092924661 0.13683202 0.130459678 0.085150812

0.107959941 0.1105684790.135181366 0.093593233 0.110709078

0.161395461 0.074700121 0.160402807 0.133227956

0.078903259 0.140467941 0.1119231640.15722927 0.163327573

0.1174928010.128513064 0.082358057 0.086571714 0.136307192

0.139509003 0.094299714 0.102295937 0.1102510910.181124965

0.136791787 0.147050033 0.102180359 0.121209346

0.1116014360.170769736 0.080276489 0.14830504 0.128703548

0.088461735 0.1215662 0.129088064 0.109061417 0.11959344

0.153069304 0.136270975 0.131617849 0.1150368380.159309085

0.120077971 0.08970981 0.147657707 0.106438858 0.099122007

0.133252916 0.132270495 0.120731743 0.107049599

0.1112729650.11080313 0.07669361 0.078769866 0.082198282

0.129697084 0.1355581160.157947949 0.1150832480.155750417
0.104508278 0.1135961620.185478206 0.13082397 0.105024696
0.081003156 0.1151109050.079739656 0.098730787 0.102649587
0.171361427 0.148108198 0.064459882 0.056216271
0.087258178 0.160359264 0.1581194280.150806548 0.060374403
0.09518897 0.158337604 0.1135583670.1176287580.138596143
0.109950144 0.134756422 0.147170515 0.131388684
0.134930308 0.106434591 0.13466941 0.098859842 0.144030206
0.13152864 0.107524645 0.160940391 0.135686318 0.078298664
0.088853506 0.0794685110.122777502 0.126714219 0.149869737
0.057644701 0.091455514 0.089674334 0.127228706
0.1127425010.174334015 0.139130591 0.101078584 0.126163355
0.10395356 0.13660683 0.154014996
KEGG_HUNTINGTONS_DISEASE 0.325677582 0.316950727
0.345293729 0.344323214 0.344725538 0.335960376
0.347255392 0.338312618 0.334251516 0.321346626
0.343141217 0.33143631 0.309565053 0.323869379 0.327828919
0.334076414 0.336348792 0.320428929 0.334708507
0.338375131 0.313004332 0.319746084 0.343878327
0.342378378 0.313600238 0.335687606 0.323436707
0.333803647 0.301934446 0.320713808 0.349104126
0.316736723 0.3122721170.330829865 0.336331671 0.335566465
0.334022356 0.336566344 0.345326064 0.33970395 0.331315211
0.334460763 0.339957776 0.332279247 0.337623875
0.331700247 0.313516431 0.3307909110.335069989 0.337491646
0.3147110430.338708523 0.336462061 0.327847375 0.339296041
0.322425323 0.336809507 0.331453087 0.345159554
0.332527437 0.346517832 0.338075873 0.338652738
0.331563457 0.314229703 0.355637842 0.339650898
0.345936221 0.321601074 0.332636923 0.324844498
0.346253512 0.333232855 0.340704833 0.328342743
0.325489874 0.342438123 0.33654393 0.323764453 0.348077064
0.342956983 0.348939347 0.324053084 0.335296151

0.337217576 0.333217233 0.330687257 0.334310857
0.355423244 0.316618951 0.334146218 0.340181269
0.336830845 0.335586734 0.333015013 0.3370224 0.330786561
0.330363535 0.328198887 0.333022148 0.333459168
0.319381494 0.337762967 0.335981527 0.344926657
0.3337011420.31945251 0.326559218 0.336766203 0.319218875
0.355773761 0.331812518 0.335088058 0.328194975
0.323073713 0.323504755 0.341209231 0.3511255250.332644137
0.331545617 0.317315173 0.329682304 0.32234508 0.337891345
0.322680207 0.351784597 0.344374724 0.346358505 0.33768018
0.346408979 0.3474362 0.325885466 0.346961462 0.319071918
0.328186068 0.327828753 0.329596545 0.327267381
0.308092357 0.308019571 0.330834023 0.323139552
0.327854514 0.319021768 0.3391151840.314780226 0.324781754
0.344563299 0.302516595 0.334191354 0.3204110490.345526454
0.307965842 0.332200866 0.316829255 0.293406724
0.309573743 0.326182427 0.32129888 0.314066713 0.330923205
0.312137842 0.332689172 0.335597891 0.324547427
0.334697683 0.308946983 0.329922036 0.30573698 0.321381205
0.3312549 0.349653338 0.343294896 0.33411357 0.316309652
0.315048336 0.335505409 0.326543007 0.330578279
0.317645822 0.332218584 0.326120049 0.324196416
0.324947676 0.3233151160.331607262 0.331335187 0.322505438
0.331339251 0.331015275 0.29521479 0.304448689 0.326873268
0.318081038 0.3116153730.315256392 0.323283633 0.326346327
0.313990462 0.324464258 0.299946508 0.3419119890.33434176
0.328274075 0.338383915 0.338553823 0.325272312
0.341968823 0.318462417 0.318271038 0.3113074760.33032548
0.309515579 0.33519273 0.317614856 0.316580452 0.313023259
0.308743013 0.298806553 0.331869577 0.307822956
0.326818876 0.3241501190.329437471 0.309337264 0.325659642
0.3117039050.317434046 0.333930343 0.314307209 0.331084727
0.324629154 0.336722268 0.332224526 0.304964296

0.320744254 0.310260925 0.325922268 0.32832574 0.322103612
0.3361137590.325061216 0.322467889 0.335926389 0.318829913
0.321808866 0.342125343 0.333344778 0.34093622 0.33313237
0.332068786 0.31441995 0.3155042110.348931337 0.335071785
0.31850395 0.353701276 0.336532003 0.33829878 0.339645515
0.341486874 0.316871469 0.340339798 0.312757848
0.329083536 0.319972825 0.333139105 0.329028735
0.332095051 0.335160985 0.336143936 0.336207838
0.270384641 0.349276087 0.330874681 0.344501236
0.326934908 0.332441929 0.330404338 0.318560444
0.344952328 0.33727752 0.345707023 0.32289784 0.315867894
0.314183183 0.315553713 0.327050013 0.324105127 0.31183381
0.317529186 0.320429519 0.345146127 0.326958864
0.315364385 0.330655793 0.313523761 0.327266506
0.303058872 0.330870295 0.324335946 0.320927383
0.316172844 0.331454233 0.318322418 0.307039882
0.310427004 0.331547564 0.33068742 0.30591965 0.325328467
0.3274811470.328051742 0.316435597 0.294677736 0.334879149
0.314069436 0.340140286 0.331416991 0.326772394
0.319450594 0.3293784110.315439093 0.315784258 0.329549839
0.334589973 0.350535834 0.339651093 0.319728238
0.348009159 0.320337662 0.320836966 0.329912041
0.3210578110.30658533 0.326210144 0.347236606 0.325439356
0.288491684 0.341033651 0.301254667 0.31812422 0.332729123
0.313079942 0.293946679 0.316343172 0.329945004
0.314243752 0.309456653 0.3021078 0.321284221 0.322470458
KEGG_HYPERTROPHIC_CARDIOMYOPATHY_HCM 0.044833844
0.0571100120.059022681 0.0521811650.08627508 -0.016253402
0.002136419 0.062659923 0.014938562 0.080578457
0.0115936660.0409114360.0381112450.027752859 0.093390075
0.039444773 0.027546813 -0.01187624 0.003671083 0.026067591
-0.03578213 0.019427767 0.044698829 0.015228903 0.064987948
0.030140502 0.037695898 0.02757108 0.018351848 0.033110824

0.01627594 0.016357181 0.023246228 0.021700206 0.078955832
0.033094161 0.012868271 -0.000470031 0.047253572 -
0.038139996 0.0321762110.017796234 0.063706151 0.001219029
0.019540976 0.018050028 0.05836405 0.033327548 0.045637425
-0.007049071 0.072686742 0.021613206 0.035790817
0.007464887 0.026595488 0.08896183 0.024890285 0.022686849
0.043226915 0.0387111510.004693103 0.017893708 0.060461685
0.07939215 0.024102311-0.012095069 -0.00116269 0.054591671
0.094849865 0.0479010110.049501301 0.027734853 0.058362321
0.022170564 0.0281668110.066615171 0.029693855 0.07140477
0.067878131 0.059468051 0.047950325 0.020778172 -
0.009032733 0.034139565 0.038103453 0.055083785 0.043668731
0.019316345 0.032022934 0.0406877 0.050232053 0.045373453
0.060185098 -0.014324455 0.069508296 0.034971633
0.030914634 0.061048087 0.015562797 0.041707986
0.022555384 0.00334417 0.003161373 0.017742395 0.027275884
0.049062021 -0.010639899 0.088094377 0.054892021
0.082500293 -0.00729625 0.064885913 0.006713284 0.031889808
0.045028967 0.084466535 0.040631448 0.0411600970.045653325
0.024259491 -0.01775183 0.039427399 0.024286873 0.047465949
0.059682093 -0.01203597 0.059303524 0.041087709 0.016469229
0.013850897 0.03761773 0.022282518 0.0186228 0.067142746
0.062894451 0.019418046 0.048172817 0.065133432
0.045575456 0.046173588 0.031430959 0.059190536 0.05001527
0.006358719 0.006352866 0.076297773 0.093267847
0.018921462 0.009329338 0.066686101 0.020228492
0.031420714 -0.01362742 0.006230566 0.067517313 0.039075025
0.0291104980.042618163 0.053789007 0.040489276 0.033656272
0.046810513 0.012283999 0.078580694 0.107304884 -
0.002752834 0.014969729 0.030669381 0.0211176080.029131637
0.021007671 0.048851758 0.02632063 0.05112964 0.075649263
0.062524964 0.057945349 0.074835535 0.054814315
0.029518897 0.067134234 0.008188677 0.036375228

0.062259523	0.055439481	0.025100642	0.030927605
0.066376767	0.000242095	0.046613734	0.076597848
0.032599627	0.097462148	0.030966413	0.075088631
0.056845925	0.0759538	0.057504504	0.077346464	0.065718988
0.1143141780.0347711520.064261042	0.031267278	0.044047626	-
0.006720364	0.039031241	0.058676504	0.058208566	-0.003262565
0.058094172	0.04330098	0.063032449	0.029595492	0.060062891
0.067212976	0.032412384	0.0948089110.1176553320.031542218
0.046153293	0.06998687	0.033102541	0.053536134	0.078780593
0.008266192	0.030121835	0.02769413	0.026681075	0.079341761
0.055799491	0.038218867	0.023575889	0.034095091
0.101021705	0.051938512	0.006525298	0.0621108430.050504171
0.022036051	0.049763553	0.053928591	-0.002958222
0.062816375	0.058150715	0.077330024	0.021260776	0.00723181
0.025530835	0.027041478	0.056619402	0.076893225
0.084402873	0.003443372	0.040985139	0.013666824
0.072198324	0.048718139	0.039009371	0.094194332	-
0.004530859	0.026633685	0.050897692	0.020859058	0.045448047
0.023493949	0.02584438	0.0391129450.024289166	0.013018784
0.018243336	0.057221597	0.047732392	0.075243453
0.025090064	0.0137190110.047370179	0.031565374	0.044170869
0.039716366	0.047160149	0.0440001130.061875771	0.043242545
0.045721449	0.006099984	0.059748673	0.066824702
0.082310218	0.095384294	0.056852418	0.040822477	-
0.001971297	0.009076239	0.07745209	0.092719666	0.023407242
0.083152029	0.00263818	0.030326883	0.053365708	0.026588865
0.08806176	0.01636325	0.03026312	0.050275312	0.03292079	0.05927033
0.026546469	0.059598943	0.056323902	0.053500826
0.035667928	0.05869452	0.0811419140.0533411560.039238846
0.023654744	0.050620242	0.028128195	0.091213928	-
0.001643312	0.0243331160.032482505	0.054769593	0.043519024
0.033087995	0.061931329	0.026980365	0.078379704
0.006962154	0.077482492	0.058353479	0.05449961	0.121212076

0.0424401170.071517275 0.087067921 0.03999297 -0.019641529
0.1171952180.033928095 0.052205259 0.024788262 0.052175544
0.0603462 0.020083505 0.056644974 0.054487847 0.012373748
0.01873247 0.017635047

KEGG_INOSITOL_PHOSPHATE_METABOLISM 0.139254605 0.15020019
0.126812027 0.131442056 0.136466587 0.133632545
0.150078632 0.143799825 0.134523512 0.163461489
0.1153253230.1133288860.161713393 0.16147596 0.142554468
0.142488805 0.147248221 0.135774276 0.139098824
0.144393922 0.139124784 0.159728197 0.138800143
0.1194617780.156920837 0.143514512 0.136652588 0.161570783
0.151745758 0.148123081 0.0850600110.145299108 0.146042782
0.129369717 0.160203942 0.130435797 0.125277755
0.133336275 0.124348629 0.1001201190.153037457 0.13545017
0.128456742 0.120098629 0.136432092 0.146080228
0.1359011530.14772062 0.13419628 0.105710162 0.131347611
0.133386573 0.1487551150.1173789510.140603273 0.159290162
0.144699723 0.127047577 0.1168969150.128990776 0.132206004
0.135086608 0.133209491 0.1115247230.146329842 0.107846852
0.122776688 0.1144711730.145852727 0.136037047 0.15535448
0.120330146 0.150791455 0.148816631 0.165686194
0.154981797 0.1371398 0.132695568 0.134060782 0.139368403
0.126555624 0.1130248670.136475731 0.144219784 0.132445452
0.1109244690.1144083740.1100951080.122167662 0.156851918
0.121228087 0.103273124 0.1124845620.124285758 0.172591043
0.1177755290.146789247 0.160740871 0.147780674 0.141397302
0.131086077 0.202646328 0.13940331 0.1425531 0.138300949
0.1494117150.138805207 0.146659064 0.145003582 0.128758072
0.12884347 0.144441702 0.133888008 0.138214382 0.134318394
0.135180723 0.125614658 0.108220336 0.154804738
0.142156477 0.164424799 0.148913853 0.142539761
0.150372894 0.144910754 0.086276195 0.13377098 0.134900564
0.132286361 0.158186623 0.151776172 0.139971437

0.129921758 0.14708367 0.150328724 0.166392635 0.161228626
0.150181029 0.146040254 0.153518181 0.144099165
0.173726617 0.140685375 0.147232155 0.143969723
0.146850335 0.14403037 0.1125892220.142245671 0.157881765
0.164236763 0.140932508 0.160038512 0.121067212
0.155421609 0.158096447 0.131486287 0.172709576
0.148535785 0.175794125 0.142626999 0.168307721
0.143089387 0.154870168 0.124601969 0.145798694
0.162620916 0.141849305 0.154629502 0.170719757
0.174809621 0.142067615 0.146209372 0.134321047
0.138703374 0.157352001 0.15071816 0.148445295 0.14609606
0.159775923 0.160959354 0.146072097 0.1611071630.130619516
0.1456894 0.150945032 0.145507122 0.15251727 0.119078878
0.149869873 0.1641902110.179002103 0.164825598 0.162245401
0.156850645 0.133834893 0.151871398 0.153755283
0.1511637070.165557712 0.170708238 0.1241113950.144868576
0.153342545 0.151382677 0.134103403 0.159043768
0.155732743 0.18051988 0.152753566 0.14581432 0.163304049
0.159621035 0.144917109 0.164722338 0.169294998
0.161904766 0.1634111350.164803037 0.157535761 0.148372307
0.139769398 0.163432225 0.148182725 0.170339183
0.162052047 0.15707306 0.150729343 0.0962555110.177516694
0.137590163 0.163363603 0.13908666 0.146940731 0.148091082
0.156740396 0.170105634 0.156521912 0.160861859
0.139661084 0.139426318 0.142578948 0.139762328
0.135815206 0.150940728 0.164872704 0.09162716 0.151628677
0.102072806 0.139369474 0.155769829 0.166329914
0.164083279 0.100544607 0.12703083 0.121319691 0.123188614
0.123516227 0.107215657 0.14603196 0.1137313190.144965258
0.108336641 0.156229217 0.127577948 0.15363614 0.125831519
0.136816553 0.154505768 0.161242332 0.142386455
0.139017319 0.180730721 0.106591745 0.121440821
0.131289905 0.13853724 0.1103681990.162697321 0.161556062

0.102616123	0.142696554	0.108539191	0.137917601
0.142033809	0.159507907	0.154342467	0.166885892
0.146178091	0.158388327	0.161002813	0.173440507
0.1186033770.141383866	0.124779098	0.153550275	0.142768208
0.148377429	0.155425466	0.1329110170.15376298	0.159249991
0.1671123390.150016836	0.17572231	0.169803198	0.180634675
0.171326052	0.167681726	0.165230319	0.15998582	0.146370921
0.141330786	0.162784	0.141277507	0.177489784	0.165096968
0.1148945750.129182483	0.128666099	0.125138519	0.134733685
0.149991521	0.154393043	0.134238922	0.151544247
0.152307247	0.1112251490.149477928	0.14019662	0.174872402
0.130300017	0.1546842110.155071456	0.177160393	0.170840939
0.1189034290.163174636	0.165743167	0.153777042	0.150524672
0.15001437	0.166731767	0.157010706	0.165213195	0.151443927
0.143531397	0.168030216	0.166459067	0.174815794	0.16218772
0.147385789

KEGG_INSULIN_SIGNALING_PATHWAY	0.1911182260.196160393
0.184179503	0.196344527	0.189577646	0.184917644
0.180629296	0.196756768	0.188639844	0.202849146
0.178888532	0.195666692	0.18533437	0.183897079	0.192285122
0.1991001160.175140878	0.199709263	0.193551808	0.182493575
0.193903245	0.203551039	0.182881005	0.183277936
0.202414051	0.193017465	0.190062644	0.197622727
0.189378337	0.20690817	0.18204645	0.195128212	0.192904587
0.182201806	0.200945148	0.186154556	0.1631737	0.171893879
0.170789087	0.160192065	0.205048997	0.199177501
0.184445421	0.181958569	0.19275162	0.180671614	0.187637497
0.175731055	0.19959462	0.179630247	0.195485085	0.182221462
0.187173762	0.187964242	0.186143883	0.195178578
0.187045801	0.186172358	0.171370287	0.18188052	0.180773925
0.185788674	0.187576821	0.193402454	0.185593421
0.172782247	0.18008269	0.188551829	0.187912669	0.182576163
0.196771469	0.168163194	0.194813764	0.175890737

0.186373383 0.2052691160.1835811120.189531958 0.197380165
0.183633557 0.191403501 0.176135196 0.182358762
0.193089625 0.180886122 0.179418541 0.196516948
0.181863479 0.181491338 0.186918954 0.182347697
0.172394357 0.181380151 0.184529471 0.20970277 0.195784719
0.183508979 0.189254451 0.198733167 0.19605484 0.209317187
0.194702855 0.182292572 0.194615588 0.178002795
0.187774436 0.175277026 0.180558291 0.179816146
0.182464894 0.191357504 0.186226344 0.175844425
0.189216565 0.182805169 0.1846628110.182813994 0.178410791
0.186609718 0.205804164 0.192670286 0.183741793
0.187168812 0.200455454 0.210552776 0.172727323
0.189741281 0.176099515 0.200545342 0.199780065
0.189503173 0.186403131 0.176919498 0.201693545
0.197038918 0.197653212 0.195424666 0.203501028
0.192438431 0.220078633 0.179038536 0.216368231
0.197802796 0.196088147 0.192507886 0.191502934
0.196654822 0.182535751 0.198743696 0.192448037 0.19923484
0.188062865 0.201645955 0.166484699 0.19893279 0.1968565
0.186385656 0.201948574 0.200063327 0.207759872
0.214285812 0.204589268 0.185989243 0.188616397
0.184547321 0.181894274 0.19071709 0.199874632 0.193487275
0.188621846 0.204014065 0.184607946 0.193016725
0.187948359 0.19517608 0.204141212 0.197291417 0.190802616
0.193644732 0.205105882 0.202127047 0.204327712
0.196510743 0.176801749 0.196925678 0.172205947
0.178839976 0.197240371 0.192275851 0.190800306
0.208418262 0.203479633 0.210214713 0.202871529
0.210601399 0.198356283 0.199334243 0.205792575 0.2107401
0.221386518 0.215686714 0.191242607 0.199393213
0.195830706 0.199579641 0.181219499 0.1984361170.194744318
0.209055948 0.18994445 0.200068237 0.194140554 0.207690709
0.180332695 0.210525786 0.204865424 0.214419707

0.204897512 0.210600343 0.205799239 0.197586865
0.200809928 0.199108159 0.186507829 0.202765365
0.184067624 0.203717735 0.188540623 0.159803038 0.21259476
0.189465227 0.2118340430.198645957 0.183142788 0.193688536
0.205188062 0.204951438 0.195124625 0.209777574
0.190922712 0.181402582 0.186555014 0.181344476
0.177961586 0.198465972 0.194876126 0.168773933
0.187579649 0.178845651 0.1894118180.201535879 0.186510126
0.20187063 0.194597673 0.182715518 0.192787086 0.173778542
0.1811769650.180675826 0.19601454 0.196895067 0.185579411
0.171447551 0.183490156 0.200857214 0.194681862
0.186345456 0.186160484 0.191635368 0.173377722
0.191995898 0.20455342 0.193216392 0.164878139 0.175989602
0.189256853 0.187474876 0.195699269 0.198249276
0.197392493 0.179668877 0.193309091 0.181349177
0.200125953 0.194842908 0.201574246 0.197015809
0.206017121 0.200588238 0.207469629 0.208801026 0.20747075
0.175166571 0.177397038 0.185691954 0.193857066
0.198227419 0.200244775 0.203879977 0.202538952
0.182131291 0.193945365 0.202269542 0.188397694
0.220761759 0.20094844 0.205946423 0.188648109 0.197333603
0.195218664 0.202081705 0.19525324 0.18267522 0.193615528
0.1911286360.205089235 0.207761971 0.184738643 0.194422942
0.184003603 0.194154144 0.193726592 0.183550992
0.202425803 0.185036033 0.189494722 0.191282099
0.180635288 0.197126238 0.190682873 0.209424062
0.188355351 0.20099724 0.1898414110.2024011850.218432933
0.185393874 0.204447658 0.195682084 0.174968595
0.2084721130.193413183 0.192367476 0.202034982 0.202297093
0.185538458 0.18620097 0.205344846 0.206172695 0.186142636
0.186614749 0.190011473

KEGG_INTESTINAL_IMMUNE_NETWORK_FOR_IGA_PRODUCTION

0.103239529 0.21284137 0.3005797110.166487146 0.278186936

0.126326663 0.075799131 0.174107869 0.323933365

0.207738894 0.023752834 0.33451831 -0.071222311 0.219424399

0.318925024 0.2119745790.31091561 0.387602921 0.220963547

0.206658355 -0.05145915 0.314084551 0.337713233 0.116508691

0.316616838 0.313664241 0.283355259 0.297173944

0.257274971 0.23099542 0.169316671 0.342123061 0.138260196

0.213795201 0.256931445 0.246744458 0.083816758 0.30146556

0.334893775 0.012988647 0.25785827 0.198792681 0.241815131

0.303306721 0.213460458 0.2311639630.239675157 0.174618986

0.347094867 0.239703068 0.358829373 0.377043992

0.265187374 -0.018989622 0.362179407 0.300791649

0.365693194 0.357643337 0.325878785 0.035128748

0.308775105 0.32483868 0.182632279 0.330986144 0.380656658

0.208503564 0.273732472 0.327194124 0.271683871

0.299235232 0.227817606 0.028208595 0.226874418

0.188588069 0.227913347 0.35321142 0.137786412 0.306556535

0.20828745 0.194589283 0.352141387 0.074918287 0.126338451

0.210034513 0.359755826 0.171650431 0.367957513

0.333918341 0.1896099 0.301915714 0.250060259 0.351543149

0.236632546 0.342603901 0.268626368 0.232337079

0.348185894 0.180727073 0.364184746 0.356188568

0.2513591190.200023851 0.159314758 0.369889129 0.133656962

0.359831951 0.148442626 0.30356721 0.3559225 0.232126212

0.226095784 0.296451568 -0.040934215 0.0708809 0.168224036

0.298808046 0.309704434 0.300725103 0.333847471 0.21908856

0.299068694 0.084674076 0.341950625 0.234234639

0.220714566 0.291982323 0.281916708 0.237025208

0.156971331 0.297835524 0.27353573 0.296042512 0.234359848

0.337256199 0.229952496 0.144034514 0.344851957

0.290384406 0.163391661 0.293749012 0.202763687

0.313484791 0.321417556 0.179692342 0.286375952

0.347319038 0.31189782 0.264812021 0.235274819 0.251352895

0.393596505 0.302471466 0.299121557 0.062123142

0.308614085 0.357870626 0.261421091 0.359474969

0.133402006 0.387753622 0.248932423 0.179271519

0.189955054 0.295592023 0.35560103 0.376750416 0.117764739

0.333753841 0.259181581 0.200050644 0.34037293 0.28641974

0.185718248 0.230094251 0.369381055 0.349412766

0.282082994 0.276997362 0.320019222 0.1848911260.34411882

0.030407303 0.25285745 0.340630967 0.305495274 0.085181019

0.328739499 0.310956624 0.162453185 0.253543603

0.2801157860.375505451 0.226437107 0.346755402 0.320517062

0.214876929 0.350202792 0.274425259 0.277087103

0.263260585 0.31638385 0.215199973 0.260710373 0.282936187

0.287002885 0.025045267 0.2649711490.159340062 0.276022024

0.342083657 0.22951778 0.227570087 0.183753986 0.090105171

0.303414766 0.304999481 0.14909383 0.322019801 0.305288039

0.208086672 0.301799743 0.261053569 0.232915669

0.1180290980.310714318 0.096849185 0.283123444 0.355165353

0.254529616 0.264359479 0.356428223 0.330283336 0.24833626

0.283715435 0.293048966 0.305596545 0.247551062

0.308418452 0.26720393 0.324731859 0.294300347 0.350088419

0.319338469 0.2841113110.3357113780.303679979 0.29557585

0.058065628 0.307050684 0.285184918 0.308251897

0.404600262 0.323405428 0.3116013340.103528341 0.293344427

0.263228698 0.356687581 0.222594665 0.220782286

0.042629943 0.280388573 0.296773602 0.289707976

0.361451954 0.293240187 0.373244443 0.378651919

0.258538873 0.1811619040.4115031420.333424024 0.116029996

0.296077664 0.236356404 0.1386116360.328405819 0.370536897

0.180189369 0.237209682 0.258501955 0.285108353

0.213708103 0.252773862 0.330632732 0.398686803

0.289690923 0.376743047 0.417357017 0.305230422

0.308850393 0.355599986 0.334774576 0.057905284

0.298884883 0.295235417 0.091319637 0.34639396 0.358261073

0.200718688 0.142337871 0.3119362530.337772954 0.263680274

0.210652651 0.357754187 0.2119997370.221466705 0.288698726
0.267586414 0.354334255 0.256698067 0.125591634
0.135235483 0.034367659 0.249018071 0.215949072
0.329160159 0.282676742 0.200415268 0.309683597
0.356916563 0.263934393 0.140087848 0.382401409
0.193307947 0.326596357 0.289746502 0.372300828
0.337239466 0.330191439 0.290719574 0.36266615 0.401952282
0.297219285 0.3148261180.309503095 0.327916006 0.060499513
0.214500933 0.241710666 -0.007176529 0.249019319
0.143489871 0.369098063 0.273645286 0.174470338
0.235632264 0.236248441 0.068985714 0.1102861380.10820686
KEGG_JAK_STAT_SIGNALING_PATHWAY 0.000147434 0.012774034
0.022412159 -0.006867614 0.004287739 -0.033766611 -
0.017918096 0.020174665 0.007403197 0.013031222 -0.041644203
0.019394952 -0.008104486 0.001710959 0.042295276 -
0.007670009 -0.019669679 0.020038413 0.013058018 -0.001991584
-0.027607628 0.043977884 0.006164594 -0.036643885
0.031597262 0.026643672 0.0111879760.012866986 0.029270464
0.02523691 -0.033034701 0.013799288 0.009409037 -0.007414933
0.001534358 0.006625616 -0.020005782 0.003367007 -
0.009680145 -0.016935468 0.018767105 0.014806249 0.004356483
-0.012171773 0.025842018 -0.010471781 0.000891328 -
0.001503849 -0.00232451 -0.006568267 0.055709064 0.010290826
0.015250361 -0.009288697 0.028632471 0.027356891
0.033042561 0.025569794 0.005770508 -0.004979599 -
0.006791933 0.0119475070.004135396 0.015108557 6.26E-05 -
0.025022975 -0.0024673 0.018914388 0.018438135 -0.001751599
0.031267422 -0.022398851 0.017778447 -0.006214931
0.011117434 0.057668785 -0.003459943 0.019718109 0.004688061
-0.010778196 -0.001141375 -0.032138074 0.005784149
0.017680715 0.030970507 -0.006686341 0.047614324
0.040415892 -0.009515271 0.016738814 0.0114904280.021785126
0.00439291 0.003810056 0.020232936 0.008987192 0.002726805

-0.000121582 0.035612075 0.05025186 0.017700105 -0.002148628

-0.003307401 0.025134821 -0.028336926 0.012386302 -
0.0211595360.017493506 0.03330424 0.025054595 -0.023535855

0.011948298-0.035368536 0.018061788 -0.021851599 0.039715367

0.00123114 0.002826109 0.038971441 0.013933755 0.034143485

-0.023630507 0.018002836 0.038922647 0.021815712

0.000139796 0.02201422 0.001122442-0.011417837 0.022528914

0.012026127 0.034170654 0.010075134 0.048155756

0.026582842 -0.017668321 0.041299924 0.030366778

0.012609122 0.043858516 -0.013079941 0.0331901180.029928526

-0.002119602 0.000170162 0.04520558 0.027328286 0.003192088

0.010777557 0.02939559 0.04560533 0.0113697210.025762221 -
0.014930767 0.009747658 0.048301348 0.03877787 0.024293002

0.003973227 0.056560089 -0.004028828 -0.002257051 -
0.003759107 0.02698727 0.032804361 0.015526267 -0.023153799

0.0114047640.017263357 0.000244487 0.039829087 0.002439857

-0.015821306 0.002068442 0.042845642 0.049023973 -
0.009222753 0.013844858 0.033517262 0.023484343 0.030436214

-0.019833593 0.019127301 0.014963125 0.038847496 -
0.013203508 0.006043165 0.06096559 -0.01296015 0.033315799

0.037788684 0.046060082 0.024363682 0.0584091110.042835603

0.035669612 0.030610925 0.046791296 0.043523162

0.028692295 0.05909427 0.002243086 0.028849502 0.011289643

0.037539698 -0.011436606 0.0011864520.00779065 0.025958423

0.003188657 0.027622452 0.004059331 0.038096411-0.010082026

0.026172797 0.052212057 0.022408357 0.0511098450.060147152

0.01139971 0.033938491 0.040792862 0.035062843 0.010758066

0.040402419 -0.014232445 0.0112735050.031617039 -0.030726087

0.052097768 0.019333055 0.042636727 0.013401429

0.022189007 0.029456268 0.041438163 0.002709688

0.034945634 0.021703216 0.040134935 0.029372176

0.048090842 -0.018400816 0.0054011340.032374403 0.02386568

0.002081979 -0.036949236 -0.005091914 0.0301195730.00984328

0.054587219 0.054229945 -0.014603929 -0.023290693
0.025544482 0.001482788 0.016771062 0.00903047 0.009013124
-0.02419275 0.014764329 0.007977146 0.043536578 0.033972651
0.022753015 0.004714304 0.046276926 -0.01197176 0.001118193
0.026273887 0.015627349 0.035763885 -0.014147448
0.009344311-0.022753759 0.016376837 0.022881225 -0.018827134
0.024314175 -0.002606164 0.016413657 -0.002792586 0.02977905
0.0241116960.019214165 0.056744461 0.054400882 0.063176374
0.036082093 0.028720809 0.043958161 -0.013175249 -
0.000836261 0.017710919 0.030520026 -0.003573799 0.04014101
0.038789482 -0.016760805 0.003862095 0.017597016
0.037360952 -0.006398048 0.00952697 0.038145064 0.008304597
-0.005770842 0.022856436 0.03735563 0.036185917 0.01172505
0.007379549 -0.004617436 0.0080378110.036140058 0.010337551
-0.000218451 0.004444475 0.01869266 0.007212799 0.003867115
0.010433611-0.00737761 0.028457224 0.018743152 0.038685722 -
0.008715702 0.001539584 0.021516753 0.029275812 0.021287563
0.04218282 0.03824358 0.040574798 0.005315661 0.002529541
0.039458152 0.010051292 -0.035878994 0.058948445 -
0.0113694540.009019241 0.033398061 0.04319211 0.022265919
0.003983916 0.015792451 0.0483766 -0.000525067 0.012483252
-0.012652634
KEGG_LEISHMANIA_INFECTION 0.155585166 0.246612771
0.324863377 0.194390224 0.316679655 0.152095808
0.121518165 0.23334362 0.321881357 0.264529255 0.077004658
0.356467766 0.015908502 0.240240989 0.347628203
0.260697936 0.306790022 0.377270716 0.231056326 0.21113625
0.021225937 0.334062914 0.346312619 0.156026534
0.3611489310.335182321 0.305332182 0.3119906250.271558107
0.2684112890.209079104 0.347867914 0.173139356 0.225784365
0.286927936 0.273456818 0.129797596 0.282506416
0.355596699 0.059459032 0.271800438 0.220993819
0.2788991140.307831737 0.235188667 0.254620137 0.267701826

0.194667566 0.365830889 0.252383398 0.358466031

0.380851416 0.292970191 0.047965491 0.362545715

0.337053141 0.378738361 0.357825669 0.340718382

0.082650194 0.309910506 0.328851536 0.225283952

0.358261344 0.365175332 0.222473498 0.273804296

0.342162568 0.3117685390.3138011430.257675151 0.091331493

0.2539977110.19912339 0.254592917 0.388614229 0.181080825

0.3311647330.238056979 0.228129294 0.35553821 0.127912104

0.167282433 0.238107138 0.368221336 0.229126054

0.379990181 0.330897621 0.221700287 0.309397453

0.279524043 0.3611591940.275319055 0.334320925 0.28932608

0.264378683 0.340982378 0.227888682 0.365959967

0.376477901 0.269056726 0.219350344 0.189196622

0.362913698 0.174702698 0.3589323110.188726074 0.344357122

0.363241779 0.275912784 0.247072715 0.324424467

0.022633904 0.12055473 0.207080181 0.344304818 0.334733787

0.313161006 0.352691914 0.24321572 0.313249121 0.1520374

0.352813916 0.260643787 0.268577259 0.288362755

0.304652098 0.254950079 0.182740714 0.314544122

0.277994684 0.305197592 0.245543642 0.360560036

0.264764048 0.183867188 0.35177172 0.313161363 0.217524603

0.309803104 0.234605224 0.323235018 0.35332927 0.208746783

0.304258934 0.368857995 0.350834227 0.287600814

0.259241323 0.283460607 0.378306727 0.31716258 0.311258663

0.1150510320.340537916 0.3542114870.29269193 0.377608318

0.192996292 0.394234391 0.277097247 0.229733645

0.219915873 0.324997891 0.386054782 0.36301481 0.159539769

0.347313221 0.281747406 0.231257877 0.343829326

0.328500425 0.215602739 0.27457032 0.388878361 0.376116385

0.302852576 0.314005591 0.346403951 0.229760701

0.376001436 0.093845306 0.292798494 0.35577885 0.332241375

0.128692776 0.33336206 0.333155575 0.187019219 0.280735605

0.334876653 0.38389198 0.28652889 0.362169882 0.340574472

0.241858625 0.3811405770.3011694170.31774425 0.318054827
0.354915774 0.242083009 0.274716927 0.312906586
0.320739631 0.079415684 0.273618791 0.20422034 0.30673502
0.342675364 0.27529782 0.27009548 0.237734079 0.148184608
0.328419726 0.325042596 0.196060085 0.354136224
0.350634915 0.252816366 0.314151647 0.322727762
0.257942925 0.179084766 0.335360733 0.136780256
0.301963263 0.360886263 0.265109374 0.291941284
0.367738795 0.355836923 0.285551031 0.300450488
0.334693441 0.324999842 0.265682782 0.346720006
0.290833508 0.333032 0.32922624 0.360660415 0.315010609
0.308249968 0.340738251 0.358533745 0.29703263 0.114111036
0.321488352 0.295301736 0.333975918 0.416694934
0.350908801 0.328357815 0.176435693 0.309087014
0.295006154 0.361453106 0.258797448 0.2698119260.093531372
0.293665213 0.318593123 0.298039477 0.372718042
0.312754916 0.380939716 0.386367669 0.264484078
0.214792867 0.403274426 0.344839937 0.178224926
0.330863967 0.267478919 0.158960964 0.342372036
0.376896993 0.216495328 0.259289772 0.280214191 0.30489133
0.238549793 0.279727131 0.342887719 0.37768817 0.314441494
0.389567319 0.438876483 0.351407729 0.337713558 0.36108132
0.335687842 0.109003545 0.32550064 0.338447366 0.152686746
0.367433605 0.35832449 0.234557758 0.19316037 0.314765995
0.365776393 0.277267525 0.241555401 0.365980218
0.240238613 0.255732282 0.307804394 0.301374691
0.360918709 0.28277811 0.1989110330.209497524 0.11789787
0.276403534 0.24788153 0.32939589 0.301028678 0.224705338
0.352030516 0.347279452 0.280226868 0.184126824
0.398559144 0.23888549 0.32703677 0.323636375 0.371022093
0.370074969 0.337479182 0.331789612 0.37477524 0.404590401
0.346982383 0.346795482 0.3346794 0.354355204 0.11552339
0.23417975 0.307006597 0.071249639 0.267587963 0.19298408

0.387629158 0.299948779 0.213020753 0.269686006 0.27718915
0.1146724170.161272249 0.150045439
KEGG_LEUKOCYTE_TRANSENDOTHELIAL_MIGRATION0.160724631
0.187237923 0.1992311950.146689935 0.175777807 0.146321134
0.146384935 0.160783464 0.170612266 0.171378092
0.134058803 0.190257687 0.137978172 0.155198944
0.191684776 0.167883008 0.142851441 0.176935345
0.142005202 0.153645399 0.130426833 0.197893874
0.162210357 0.166928738 0.193038999 0.182263506
0.175280879 0.178337223 0.141423535 0.173212458
0.124694132 0.156041008 0.142316923 0.14548838 0.170568611
0.140899493 0.134987752 0.144819718 0.161650404
0.091781417 0.198921777 0.159695005 0.155067768
0.192669641 0.176924061 0.180405875 0.163948462
0.1611639950.184649085 0.150843902 0.225084959 0.167786796
0.160088278 0.129501486 0.200572476 0.194927791
0.181967157 0.199221758 0.154558393 0.147766009
0.162424602 0.189568498 0.165661699 0.205120022
0.153819862 0.13258297 0.144085709 0.196050079 0.177087974
0.174986166 0.1952011110.135097809 0.1824593110.151422654
0.175304293 0.225397103 0.15986129 0.181877662 0.179593687
0.1743901140.165473188 0.126988518 0.136459739 0.165849771
0.1656115490.164977891 0.167642453 0.204764746 0.178768852
0.182075235 0.157370092 0.192025587 0.175189443 0.15875968
0.187420676 0.179053242 0.169430086 0.143255365
0.196739665 0.191548373 0.184019836 0.128879784
0.1511049730.180779791 0.157160406 0.200214543 0.120858375
0.189424863 0.165832479 0.186027534 0.150492699
0.176898309 0.142089141 0.16546297 0.160160325 0.206305437
0.169688978 0.175281927 0.183187212 0.172125732
0.181704061 0.178409132 0.1775322 0.206271304 0.181043361
0.145374283 0.155870758 0.159495707 0.152429498
0.1911631380.198155772 0.165970627 0.16312913 0.188519725

0.194692067 0.141730396 0.20096231 0.173791031 0.177499966
0.208246965 0.176759861 0.214151448 0.17914297 0.152298394
0.159600251 0.216940262 0.19858335 0.162995653 0.161532128
0.163955785 0.205585384 0.152609405 0.166405652
0.133939716 0.19490287 0.192845261 0.205204695 0.193668521
0.179399816 0.217829667 0.183446596 0.172896481
0.152221449 0.204397991 0.198037978 0.147037028
0.145816258 0.177988349 0.164943829 0.156290314
0.188847813 0.181330068 0.141256377 0.154266383
0.174247157 0.203826776 0.179106633 0.176209998
0.182463286 0.195720465 0.20794346 0.15854049 0.162177623
0.172294057 0.183617804 0.153387276 0.179224438
0.222059425 0.166534637 0.167475422 0.189393231
0.199958075 0.199925397 0.212999547 0.2118246210.201166245
0.196471204 0.197871932 0.212062695 0.189463342
0.228933193 0.145990379 0.191012941 0.165464774
0.181984993 0.132801659 0.151605 0.180657604 0.178599172
0.1541811920.19587205 0.159187505 0.186655968 0.161357742
0.203412658 0.197618324 0.190304471 0.219572959
0.231392062 0.179505719 0.216608772 0.20587246 0.170272594
0.158822192 0.226561373 0.152926028 0.197657905 0.16948776
0.139769134 0.223789287 0.169050682 0.199319681
0.170431568 0.166224591 0.195458927 0.192554705
0.153460601 0.2113409770.202745228 0.200651873 0.181579771
0.204696575 0.146490426 0.164176298 0.194451896 0.20497094
0.148186465 0.130632372 0.158777874 0.170378748
0.1868113050.21811322 0.197793484 0.14169037 0.15384199
0.173365279 0.170394627 0.189829039 0.175714477
0.1871911250.148515438 0.172967245 0.1711707370.18919775
0.189067021 0.18765323 0.172662918 0.187065624 0.154969636
0.160806817 0.215713677 0.194713229 0.185575527
0.143742777 0.192046494 0.139490432 0.195556941
0.188709259 0.162580883 0.177305884 0.16570106 0.209872601

0.203172165 0.1761211950.190933253 0.170042435 0.207316881
0.210209435 0.232828986 0.217612502 0.1741158030.212670337
0.147127013 0.128158856 0.1825112960.209913877 0.155620135
0.201539712 0.18579464 0.177067217 0.152099021 0.167127868
0.212446959 0.163322579 0.169249258 0.1893114420.188235631
0.180870663 0.195796657 0.181654 0.210125532 0.187947992
0.150639251 0.166231094 0.153520284 0.187840929
0.174951395 0.178833122 0.172377276 0.172314865 0.1843463
0.163763565 0.178845683 0.152364182 0.1639533 0.174728158
0.190229028 0.172271551 0.18751862 0.206533173 0.209108611
0.199913496 0.169599893 0.207725027 0.220560191
0.180195394 0.190526094 0.188667744 0.149956171
0.123858644 0.185967967 0.13541225 0.17948581 0.162116615
0.199672396 0.184279562 0.180840064 0.181706861
0.202983125 0.142720158 0.156686049 0.111007977
KEGG_LIMONENE_AND_PINENE_DEGRADATION 0.292962659
0.27735384 0.293930799 0.317723745 0.334841349 0.32211542
0.346813591 0.248443671 0.3501100580.265381389 0.322528093
0.285486604 0.3001911980.350571346 0.281436888 0.317547342
0.295422514 0.303604195 0.26497717 0.344237663 0.317158849
0.283777177 0.307842103 0.320128933 0.294858735
0.310074661 0.295747768 0.314631823 0.279205148
0.315309295 0.284916724 0.298666168 0.294080714
0.315602004 0.297996041 0.326485828 0.296226999
0.295918607 0.299295105 0.259643582 0.304887959
0.3116543170.327223355 0.340564579 0.275487306 0.301193143
0.281777047 0.266235975 0.350314734 0.31271527 0.292730925
0.286077261 0.329297226 0.322384718 0.328223416
0.296277483 0.305614155 0.268129281 0.288334243
0.266745573 0.34638503 0.270272824 0.305262892 0.311627709
0.280675091 0.303364653 0.30083468 0.289538152 0.268591944
0.316647366 0.31788245 0.290383685 0.296306189 0.338486424
0.299996847 0.261296575 0.301064876 0.261836828

0.303908855 0.313279974 0.330992176 0.287852344
0.276916083 0.308992855 0.306460658 0.347643538
0.281593903 0.286826304 0.30247004 0.336284914 0.325263687
0.303804706 0.3192511380.363300793 0.315177308 0.234906485
0.294997274 0.283818561 0.298783308 0.29029914 0.285294061
0.379553479 0.286615063 0.342024232 0.320261993
0.296348636 0.340745087 0.293104076 0.339007926
0.299737924 0.330399607 0.278889679 0.255126965 0.27208171
0.3111476360.282835438 0.294008433 0.310200646 0.285953639
0.280339677 0.263064959 0.305883209 0.305675565
0.241984286 0.252518262 0.299786363 0.263025697
0.306336953 0.29870551 0.318167015 0.253026658 0.269665565
0.274401646 0.3211170470.275899691 0.261855302 0.294876806
0.312365512 0.281292217 0.323155835 0.281884099
0.302563238 0.320444269 0.337881692 0.331034279 0.31286691
0.285817274 0.258817258 0.2598114930.307962694 0.2822986
0.353776465 0.359655388 0.318461365 0.274270968
0.239878807 0.243230605 0.35996953 0.309431421 0.368529327
0.309431829 0.314605232 0.282492021 0.339305314
0.281949602 0.344734173 0.306709975 0.307255295
0.332318229 0.355790379 0.310514394 0.302284338
0.316674087 0.347622647 0.284031272 0.27596775 0.289113968
0.325238562 0.327521054 0.304143959 0.297606687
0.266978516 0.224444787 0.292471488 0.334460609
0.252641226 0.30720004 0.266291681 0.332459037 0.343215848
0.303505563 0.2975453110.315928895 0.307547961 0.338982497
0.267946339 0.268556475 0.266552012 0.289260428
0.281046128 0.293451704 0.253225568 0.323333462
0.269680275 0.222504409 0.274937977 0.279975654
0.303013556 0.3116428060.297718014 0.320195104 0.287420009
0.250775377 0.269735959 0.301533596 0.320528095
0.281258087 0.3091153190.310770551 0.306023039 0.279597457
0.272580765 0.305310896 0.299262246 0.316870822

0.271056569 0.316482085 0.325765523 0.283992236
0.303141877 0.312564474 0.2811644540.276095232 0.319682763
0.269568035 0.301317438 0.290364144 0.258624698
0.300077945 0.319871044 0.296416928 0.304312703
0.333307761 0.278048349 0.266500326 0.2813591150.291471774
0.296159037 0.29946901 0.337232248 0.294927294 0.33145012
0.2818117680.315396351 0.296935223 0.274624397 0.329025222
0.324200652 0.307453557 0.315148243 0.301604743
0.285522657 0.313816348 0.331386235 0.310043542
0.293733379 0.322593134 0.301788164 0.286886236
0.288049944 0.304691342 0.262670294 0.257502556
0.278487707 0.30803895 0.359863633 0.289201457 0.294965812
0.2932811360.318019807 0.281679649 0.330831469 0.313365727
0.244009327 0.295026782 0.292305577 0.295773898
0.304749778 0.294542757 0.287742218 0.291584313
0.321043931 0.276874615 0.267596007 0.306693557 0.29826117
0.324892819 0.302869548 0.338161671 0.271001395
0.347030952 0.290582941 0.313995882 0.289816125
0.299146954 0.290390908 0.323287356 0.300454745
0.299844121 0.298577221 0.303721732 0.292730806
0.259785491 0.30658544 0.326296214 0.329394428 0.346999845
0.302202684 0.32121315 0.278195969 0.282490343 0.307163655
0.298189526 0.327560133 0.302368915 0.275596893
0.310787987 0.28275417 0.316244504 0.283527214 0.298196922
0.292380376 0.244042837 0.291643752 0.27712642 0.302641712
0.304639866 0.263185702 0.231651449 0.365392975
0.280175669 0.271831425 0.294340121 0.209823403
0.292822894 0.287369225 0.336564466 0.287870753
0.242788325 0.290029738 0.30862662 0.314599316
KEGG_LINOLEIC_ACID_METABOLISM -0.233080823 -0.21097722 -
0.197616747 -0.221601293 -0.208513282 -0.246807527 -0.236671619
-0.231099595 -0.256840237 -0.254860499 -0.22899394 -0.239928273
-0.289800964 -0.210670392 -0.237155545 -0.253139064 -0.2404703

-0.176290341 -0.217578514 -0.232788791 -0.248207713 -
0.209858528 -0.224429935 -0.237203093 -0.209957889 -0.236791229
-0.247542513 -0.232432973 -0.217729112 -0.24166054 -0.269845716
-0.254243151 -0.248739538 -0.171111358 -0.255734577 -
0.18795818 -0.259856285 -0.197339798 -0.248379747 -0.21792793 -
0.217836125 -0.169018288 -0.235419857 -0.244679794 -0.249055002
-0.251616923 -0.245642238 -0.153074769 -0.224571195 -
0.226280498 -0.178486323 -0.23738387 -0.221022584 -0.190478249 -
0.21054057 -0.277070845 -0.226271475 -0.204820442 -0.261986193 -
0.241812592 -0.248533163 -0.275103257 -0.215187452 -0.237284628
-0.219781001 -0.226162941 -0.179236587 -0.207148802 -
0.229916358 -0.239519858 -0.24669482 -0.194665722 -0.273299057 -
0.211306813-0.228763397 -0.240617412 -0.205894385 -0.248029661 -
0.259264377 -0.234465659 -0.204008388 -0.261949343 -0.224703088
-0.258050236 -0.183269265 -0.2344437 -0.225203985 -0.191017736
-0.2324986 -0.216137786 -0.248847771 -0.241858019 -0.256414125
-0.234536334 -0.22762354 -0.263077369 -0.198001484 -0.243589478
-0.173336947 -0.206384144 -0.239458287 -0.238540192 -
0.077796403 -0.231049787 -0.263203097 -0.195353672 -0.217850305
-0.271006464 -0.216117791 -0.195677738 -0.23842848 -0.240003291
-0.287776718 -0.226014392 -0.228333979 -0.234989809 -
0.218745954 -0.219827432 -0.226129948 -0.2428634 -0.237968412 -
0.296511397-0.243241637 -0.227525887 -0.252902297 -0.201757409 -
0.208647108 -0.217809151 -0.248557823 -0.2147367 -0.218246671 -
0.226827712 -0.214698955 -0.257615177 -0.24490206 -0.190921364 -
0.258320994 -0.236627498 -0.270102829 -0.253906815 -0.235457926
-0.256016994 -0.213724383 -0.221998707 -0.202946866 -
0.24787372 -0.258461789 -0.230830059 -0.236671467 -0.254770261 -
0.178976856 -0.224770576 -0.219223678 -0.233906586 -0.256510653
-0.254999673 -0.253526814 -0.210063141 -0.253788323 -
0.182981903 -0.267822881 -0.221680213 -0.225688971 -0.224372104
-0.238884826 -0.199842834 -0.271080211 -0.223361103 -
0.250852642 -0.227185923 -0.26104817 -0.224085082 -0.226800716 -

0.228963567 -0.208775074 -0.219348098 -0.230394006 -0.181534199 -0.239221209 -0.264077549 -0.258535464 -0.160122484 -
0.254654256 -0.209191252 -0.205973847 -0.28455674 -0.246417779 -
0.187181493 -0.23922431 -0.218999424 -0.241946917 -0.230240656 -
0.242417324 -0.199980952 -0.223039097 -0.222365675 -0.198583248 -0.234804249 -0.223643499 -0.244604528 -0.249886218 -
0.226391147-0.245525994 -0.23403588 -0.219960498 -0.209811235 -
0.269880015 -0.22386165 -0.262425045 -0.195572335 -0.265698767 -
0.242015063 -0.240897347 -0.264188403 -0.236474556 -0.225846031 -0.275363999 -0.254121237 -0.271721553 -0.247984314 -
0.281452998 -0.237681502 -0.267261844 -0.256648508 -0.203303967 -0.250081402 -0.276383506 -0.236215354 -0.245314314 -
0.255660011-0.225843714 -0.245342101 -0.230956952 -0.233665057 -
0.23590544 -0.234895662 -0.211691721 -0.224691416 -0.223069907 -
0.222316739 -0.252915712 -0.225318392 -0.230712672 -0.220435219 -0.248530034 -0.220023858 -0.227273802 -0.217264523 -
0.246142855 -0.245512004 -0.261994546 -0.239385337 -0.238060269 -0.222774978 -0.241172744 -0.228031757 -0.215105251 -
0.198583947 -0.213910581 -0.260359218 -0.25623872 -0.26659964 -
0.259421025 -0.18680368 -0.240105362 -0.242295691 -0.224763103 -
0.240810644 -0.256047385 -0.209395341 -0.252999648 -0.245662302 -0.261413749 -0.221763403 -0.274637927 -0.240716407 -
0.212411858-0.226652894 -0.284461687 -0.265864618 -0.248693622 -
0.239969042 -0.238006254 -0.178928806 -0.241218823 -0.238798361 -0.244368209 -0.249772517 -0.219309989 -0.231911394 -
0.240657479 -0.235936702 -0.239768031 -0.237678926 -0.25062397 -
0.26341417 -0.249757755 -0.2479895 -0.221955523 -0.238516528 -
0.19498705 -0.264667226 -0.238789508 -0.228522362 -0.255481933 -
0.240218129 -0.253775745 -0.226515575 -0.228226363 -0.227651076 -0.252848889 -0.24664387 -0.261670877 -0.216797048 -0.253121702 -0.196156724 -0.261196802 -0.242533243 -0.247001292 -
0.244832404 -0.270326856 -0.21645435 -0.228865506 -0.257756903 -
0.204274359 -0.225090585 -0.218967633 -0.174595692 -0.216238605

-0.218041317 -0.213936094 -0.237066305 -0.224365753 -
0.20088645 -0.223513276 -0.282980856 -0.295423534 -0.231755242 -
0.191391756 -0.170694737 -0.241442469 -0.247111165 -0.228046895
-0.228469221 -0.241478097 -0.23804215 -0.231296354 -0.228809769
-0.248035731 -0.228060825 -0.2503302 -0.238951362
KEGG_LONG_TERM_DEPRESSION 0.03405051 0.06377441 0.052736757
0.019687507 0.050515039 0.035915719 0.046847791
0.061325804 0.016053543 0.044550918 0.033478751
0.039051855 0.031321848 0.059667694 0.038399103
0.032653244 0.056988031 0.048084396 0.056756288
0.058252033 0.044706621 0.044559968 0.029022275
0.041956304 0.077375966 0.029527826 0.033801943
0.025188572 0.053474636 0.047553972 0.00209287 0.05015313
0.036082135 0.083228335 0.044841854 0.040220712
0.0624404110.029022994 0.024493348 0.04168515 0.054951132
0.062372961 0.031734556 0.0318112890.056176737 0.024292916
0.053973928 0.045535399 0.028615466 0.040176396
0.0532112660.032880458 0.034041929 0.048899031 0.040033082
0.044690432 0.049555732 0.037042895 0.021002963
0.036566177 0.053152803 0.042612647 0.048563551
0.044997012 0.054039719 0.031592643 0.028474615
0.042209176 0.030918355 0.043522758 0.054776334
0.050955816 0.039080301 0.044286712 0.047878486 0.06371111
0.054183173 0.048329704 0.050442431 0.051988748
0.040323505 0.048353865 0.058807477 0.03542296 0.039142101
0.038746957 0.047774554 0.059077316 0.064522969
0.057389341 0.022085005 0.031622141 0.020610035
0.031982574 0.056742053 0.05898703 0.043267154 0.039892076
0.048871883 0.053459104 0.05407957 0.059682006 0.053478497
0.039336449 0.023046878 0.060097355 0.043803019
0.032303506 0.033882018 0.054655469 0.032088995
0.048306326 0.023909951 0.046530837 0.055680138
0.052790566 0.049096441 0.035090934 0.048557631

0.045101358	0.044462385	0.046648777	0.019542434	
0.061515031	0.044924718	0.024729556	0.026347282	0.04552237
0.067214025	0.049990064	0.041319171	0.034984677	
0.037552866	0.037822794	0.052226172	0.043885656	
0.035899642	0.040327589	0.042159104	0.05627704	0.026147134
0.046424927	0.035406095	0.055377413	0.04211327	0.054851893
0.048692127	0.034416516	0.037404145	0.047520703	
0.045534179	0.028278048	0.055405951	0.039097448	
0.051845367	0.051518959	0.054937918	0.056717931	
0.052432182	0.05087203	0.043747198	0.051217225	0.037294678
0.023584964	0.039417656	0.038300564	0.05148123	0.025478675
0.044561371	0.035860434	0.051384083	0.051466703	
0.034481546	0.027565171	0.027607283	0.055737342	
0.056086574	0.05271649	0.021317245	0.057095324	0.029991326
0.060573296	0.0115503180.052076603		0.061748872	0.032365801
0.051519284	0.060907684	0.0493683110.04544648		0.047787832
0.057272356	0.053490464	0.046029944	0.05645681	0.05885649
0.050288586	0.044122926	0.055031803	0.047721077	
0.051488809	0.029986891	0.033127424	0.045160106	
0.039089935	0.031527644	0.026826057	0.054033841	
0.052446873	0.043052057	0.055419198	0.041825209	
0.0481100290.035416563		0.03555128	0.058712483	0.069423688
0.056613671	0.066055916	0.05796866	0.048702227	0.042523822
0.038599184	0.05061546	0.057401078	0.058454641	0.073920703
0.020448614	0.028880051	0.065192777	0.039953787	
0.032209813	0.037381921	0.051288929	0.058883938	0.04001804
0.043389354	0.050679829	0.059607919	0.050157036	
0.030774554	0.051066014	0.042544549	0.032738009	
0.055960693	0.060109275	0.032937747	0.025200897	
0.016218921	0.038888829	0.03456193	0.043459879	0.039788109
0.048030823	0.040669416	0.047620136	0.035204388	
0.061846083	0.040360167	0.027587875	0.027442465	
0.027173199	0.01396711	0.045929009	0.029346455	0.045628275

0.02988372 0.042552271 0.037687655 0.045682974 0.046501884
0.036720796 0.075641275 0.0241160970.020939173 0.05498223
0.050080472 0.034336152 0.049508958 0.024648763
0.037608073 0.043131405 0.037648428 0.048200981 0.03509109
0.041448398 0.046108134 0.051031636 0.05445785 0.039391378
0.028891501 0.063184432 0.023371336 0.061732333
0.043468884 0.040389439 0.033986019 0.048334788
0.042122194 0.032535582 0.047074787 0.043697979
0.051015437 0.040840865 0.045869095 0.053988124
0.043731472 0.047219301 0.044906151 0.034906412
0.056859613 0.043597026 0.037081082 0.036677684
0.044912194 0.090479103 0.036159271 0.016895865
0.034654047 0.03399296 0.036226662 0.032638593 0.047115138
0.052072866 0.020937516 0.029477475 0.050930925
0.024107331 0.0451156250.05124225 0.048036404 0.03591545
0.033746871 0.039808731 0.05469569 0.0434119870.027785078
0.033306794 0.052921385 0.041515446 0.047590888
0.036093383 0.0619334 0.055610597 0.041987138 0.025127549
0.060554101 0.052068231 0.051901989 0.076072999
0.021222332 0.042844474
KEGG_LONG_TERM_POTENTIATION 0.1168232120.12870877 0.139536693
0.1155923980.123343797 0.103308768 0.145350641 0.133436006
0.1198937150.142994854 0.1187816540.128440248 0.132229733
0.14831131 0.122945625 0.126825992 0.108222475 0.137253554
0.15693469 0.135831285 0.1162945620.137360795 0.107674008
0.121921407 0.13880981 0.1160662480.1122968920.133047419
0.124315637 0.136203327 0.090496705 0.136293369
0.1137571570.12369105 0.134372305 0.1103792620.131149059
0.108209952 0.108366125 0.11505168 0.140975867 0.144303528
0.1149617160.122246219 0.147928539 0.126453949 0.116188531
0.1170695260.135186409 0.128868622 0.1174518720.121520414
0.125206214 0.122270743 0.1175515480.122310102 0.123087619
0.1123694110.091383508 0.12704815 0.1207118380.1300119340.1184816

0.132979316 0.1152586720.092198433 0.09779276 0.134943072
0.1150317540.120881987 0.144255097 0.134471495 0.125644524
0.133478816 0.108588805 0.147308855 0.132818354
0.128633267 0.132189735 0.13117693 0.122326622 0.107658002
0.132384746 0.1168309620.1197466440.100483002 0.121286931
0.124549785 0.152038738 0.126726789 0.1131814890.107768023
0.129355757 0.1168765 0.128961569 0.139654351 0.115052162
0.122800172 0.11787876 0.108307418 0.146867528 0.129725967
0.121214786 0.12977384 0.085231346 0.1137913650.123361887
0.103702485 0.109032493 0.12076632 0.126107184 0.114765189
0.100803304 0.1177045540.136866169 0.1291123240.137509985
0.1143029180.132493343 0.130034308 0.124365228 0.142529516
0.107236371 0.144805185 0.141588492 0.100580468
0.122041625 0.1126507910.123440952 0.145587871 0.127271052
0.1177606710.127689083 0.1254736110.134771419 0.13907396
0.137367344 0.127433187 0.133878095 0.136945818 0.12307859
0.153188252 0.106133556 0.125839224 0.1185123630.14151827
0.12786892 0.1163174840.1184351310.1162016810.14110161 0.125200277
0.140023524 0.1103878940.141594658 0.138226667 0.128788915
0.126526393 0.131348504 0.153546944 0.137215727 0.13742037
0.142390715 0.141512687 0.121644854 0.1188440650.142721172
0.131707435 0.124437971 0.128130541 0.133466357
0.1299311290.1134672920.0971109170.1134169810.148383858
0.1182427760.136933756 0.1041011470.161977784 0.125213458
0.1178340360.108407208 0.122955325 0.132972878 0.106831878
0.137536938 0.1411978620.121451908 0.131784796 0.134077339
0.139967159 0.156387639 0.136059951 0.138303191
0.139251213 0.121079628 0.141663402 0.135892083
0.135252637 0.147886258 0.1153755520.133059928 0.134023587
0.126791796 0.130893707 0.136509844 0.127892684
0.148067123 0.129604185 0.13214722 0.134469289 0.132719368
0.135887696 0.133466671 0.139844285 0.153852316
0.128545886 0.156892297 0.139227251 0.134495817

0.1130236590.131244228 0.1286664 0.156034952 0.123884777
0.158990824 0.1147879770.095823128 0.147723745 0.12029626
0.148139449 0.143672385 0.103851056 0.126496892
0.135598368 0.146164947 0.134256694 0.156607276
0.130863354 0.1115836280.133463349 0.1112864670.100630833
0.142433902 0.129165932 0.0986111050.134566237 0.112152912
0.1179709220.139919651 0.136978787 0.122080826 0.114675102
0.1165413350.1117534970.10414013 0.1175036280.11316063 0.121561586
0.10064654 0.1174745650.09943804 0.108684437 0.127496278
0.13222654 0.1145534080.121905467 0.1197742 0.109504812
0.134866589 0.14876763 0.16004066 0.098799554 0.109180928
0.127466903 0.140632182 0.120874319 0.132922128 0.12161339
0.120295875 0.142744633 0.128961625 0.127856599
0.122865524 0.124825948 0.131787816 0.137946065
0.141508782 0.129332124 0.131699027 0.147593531
0.1158822210.131475693 0.1169595980.130169056 0.119952211
0.132708707 0.139413487 0.125643682 0.132389974
0.125667984 0.144860972 0.1166922460.163883505 0.134927603
0.160833989 0.154252615 0.137938829 0.127934844
0.146333067 0.134145866 0.1164492580.1136265180.139659191
0.143194381 0.129757904 0.1125164390.133354815 0.117300587
0.1167926330.1173849670.1211372150.121798512 0.121497888
0.1111830990.14922522 0.107061498 0.120666324 0.120048124
0.167699287 0.1111542440.124888589 0.123133466 0.134388971
0.156712386 0.133934022 0.132005976 0.1416502110.123559477
0.133875258 0.123862793 0.135812578 0.143934212
0.1280117750.135804724 0.1527411540.136342662 0.129151925
0.143503062 0.1211407520.119979915
KEGG_LYSINE_DEGRADATION 0.255148783 0.261455495
0.247091088 0.26492406 0.26162921 0.24315177 0.278224934
0.248179069 0.233857459 0.244046138 0.261269898
0.265324945 0.268926923 0.266582046 0.258444991
0.273382595 0.230038867 0.245537649 0.232772018 0.23576428

0.299402157 0.233109887 0.237072157 0.271787388
0.267021027 0.268332753 0.260414544 0.258384995
0.272388944 0.263770558 0.255712136 0.259937488
0.279292929 0.234402052 0.260362691 0.254127107
0.244436875 0.255484037 0.243200562 0.238527529
0.274346017 0.23694859 0.243809392 0.243507898 0.20631431
0.264777064 0.245080233 0.242025974 0.2611109520.260779897
0.2001269110.226846964 0.252491259 0.266584198 0.249183718
0.259770831 0.249641575 0.250904645 0.24081879 0.250715028
0.286360122 0.234104425 0.255403175 0.229708179 0.26995116
0.267314098 0.260024092 0.248690127 0.247778743
0.254887228 0.273286312 0.253069307 0.243925706 0.258104
0.247899938 0.233145473 0.257787815 0.256793356
0.247544673 0.268282814 0.269022268 0.244841075
0.264767146 0.251915128 0.255817552 0.284959853
0.252812882 0.231846283 0.236458686 0.254896712
0.284345566 0.213965089 0.227922217 0.267645647
0.273218688 0.254957908 0.261541389 0.2540141190.25152993
0.234142855 0.25648423 0.279383262 0.2184400110.246280278
0.24881054 0.263467881 0.282423647 0.250085461 0.259264231
0.264320969 0.26285953 0.268029675 0.279673706 0.245397316
0.240600276 0.210684562 0.259989313 0.229654063
0.253239823 0.268988078 0.2553788 0.272141939 0.253645703
0.244660305 0.264638643 0.258940725 0.260584374
0.249065596 0.242826935 0.245876029 0.249346818
0.244028807 0.237745282 0.262499612 0.249678769
0.257218083 0.2400511070.273394568 0.2600211840.259323021
0.240197039 0.259015963 0.274929364 0.291658047
0.267630007 0.254912228 0.258389651 0.248178405
0.258907388 0.283561023 0.26592398 0.25853665 0.325187446
0.260734653 0.272942137 0.246677329 0.21859169 0.274153655
0.260495763 0.257083288 0.273420707 0.273455109 0.24029652
0.262075332 0.253456768 0.256236177 0.254917452 0.28734771

0.276531581 0.268853941 0.252292216 0.271796681

0.277462579 0.278399414 0.246774739 0.244272979

0.248060357 0.235368792 0.247616935 0.269252433

0.2538114370.253202358 0.252150278 0.262243539 0.284261548

0.233440397 0.266221449 0.231510396 0.297496254

0.250950162 0.270582974 0.272728625 0.2723117640.256505224

0.2476268110.263906577 0.262042792 0.246985562 0.255318036

0.265423192 0.254017441 0.2611541470.242973209 0.263442

0.237635025 0.256501926 0.254613793 0.257730505 0.25839954

0.27337232 0.27758482 0.264247582 0.234387187 0.251075509

0.257750754 0.263770807 0.24079114 0.235043181 0.234456424

0.259584675 0.2513117810.242553803 0.249553246 0.272733667

0.256088045 0.239831201 0.257921558 0.269952172

0.237590858 0.249050474 0.256986041 0.244361483

0.263730006 0.260285252 0.255403391 0.252301495

0.259509626 0.2451776110.264672828 0.26732244 0.240855631

0.225492615 0.266451084 0.231934968 0.232009107

0.261680941 0.194616884 0.2636088110.219610259 0.256649826

0.271996935 0.256789399 0.259201662 0.2411967290.262444122

0.265614342 0.227990733 0.217766468 0.229975422

0.262732644 0.273153249 0.259995041 0.222831789

0.271022797 0.217410543 0.250824707 0.234523601

0.237823526 0.267909751 0.262689898 0.248887297

0.247471822 0.297342944 0.227319984 0.254536228 0.28049766

0.209269291 0.22161154 0.2611156260.25311118 0.243577164

0.2424141170.2281164810.259352003 0.25992055 0.264516981

0.269636242 0.264977689 0.237932446 0.23217029 0.260438352

0.2652113480.2564971110.230013755 0.244971473 0.257882715

0.253623033 0.235183147 0.287653242 0.26070506 0.279205317

0.247834221 0.256832707 0.252519844 0.284860002

0.258238592 0.272741684 0.253498743 0.266293176

0.248368635 0.251351935 0.248790879 0.252480474

0.272686765 0.2890411460.240859288 0.255244609 0.244461792

0.24620903 0.23997427 0.258252595 0.236675676 0.2664876
0.268137863 0.225524748 0.286121374 0.222723097
0.216509913 0.286298982 0.249576281 0.27541948 0.251747142
0.245198488 0.248122155 0.246416144 0.273493071
0.235620942 0.260325403 0.241802059 0.276474472
0.231871979 0.261797827 0.241513455 0.255324925
0.254523924 0.257035709 0.260875817 0.241713769
0.238542885 0.260663674 0.268727871 0.288125622
KEGG_LYSOSOME 0.245257436 0.255502985 0.275428518
0.258822683 0.273482584 0.266546201 0.247316287
0.273227248 0.278672357 0.250732034 0.250908342
0.247588776 0.226870628 0.2490765110.261475185 0.243879804
0.234021934 0.302131435 0.2549711310.24157172 0.234540593
0.3017114750.267578877 0.287869587 0.270838561 0.267369673
0.257931666 0.245449023 0.271840459 0.284969125
0.254678415 0.27097846 0.2770611390.250822574 0.23662653
0.260559166 0.227472278 0.268454416 0.272512087 0.21689314
0.257412735 0.235461765 0.270335965 0.256722391
0.275245055 0.253782067 0.238295807 0.256909092
0.275995989 0.261600142 0.291741062 0.28112349 0.237159258
0.269468447 0.284263434 0.26412204 0.254532958 0.294556319
0.263922032 0.257520147 0.281053484 0.29765996 0.246076338
0.295344514 0.278925847 0.24922863 0.303884723 0.278915005
0.268295701 0.268605221 0.276656568 0.213462327
0.275120307 0.223347394 0.276083586 0.3112198260.248182718
0.274216533 0.274880581 0.247669628 0.269523321
0.248892004 0.259687879 0.245814827 0.2786051170.273303872
0.27376546 0.254627838 0.238892461 0.246735987 0.244709902
0.287628292 0.250337739 0.229170248 0.262464009
0.296037499 0.308725483 0.256358466 0.302957727
0.306255067 0.275015398 0.260636293 0.251656014
0.260280204 0.245209229 0.292863647 0.255256973
0.282893826 0.262591363 0.26916127 0.271317477 0.278691807

0.234462345 0.271709396 0.239979079 0.295005159
0.2673031150.28270362 0.279564087 0.258307334 0.29100049
0.253576407 0.295132979 0.252956151 0.262807407
0.2621149210.255216161 0.24899766 0.234754866 0.238666562
0.251457108 0.2744112570.228620237 0.286748179 0.246691927
0.281680504 0.279072377 0.280797076 0.263144625
0.266212064 0.253242645 0.270103338 0.282362377
0.249516784 0.261504871 0.277623172 0.277486089
0.278095717 0.2575163110.273508304 0.272084062 0.259812918
0.277434033 0.267888581 0.28043342 0.2755811780.271172668
0.271521898 0.266442601 0.278356286 0.275291914
0.270937667 0.268334435 0.272081933 0.26808482 0.274649714
0.274563221 0.272489162 0.2782411350.263296232 0.279984496
0.278131328 0.266764619 0.292983994 0.261956515
0.295625795 0.292606987 0.246816145 0.28398384 0.268107284
0.303990508 0.281447473 0.279696051 0.263833329
0.292480604 0.248493765 0.257342157 0.273735553
0.237917947 0.247800032 0.287364853 0.274447809
0.270920128 0.278038432 0.2598118020.279464186 0.279473732
0.280415855 0.284863755 0.284877028 0.293449615
0.2732050110.257369342 0.262779163 0.280987821 0.251360744
0.2472342 0.235767241 0.2724211870.282717296 0.273465302
0.275392346 0.264268693 0.258741666 0.287285247
0.2599114990.2570211490.278575132 0.285005298 0.264291881
0.266369409 0.268434316 0.272890785 0.263219373
0.285706165 0.241000976 0.260530096 0.283089399 0.24981169
0.273369209 0.273217954 0.299353303 0.24463397 0.298944376
0.295541409 0.292340945 0.265953959 0.297823024
0.284020444 0.295896091 0.27147138 0.279486961 0.269058135
0.283612198 0.267703756 0.295392744 0.244841823
0.241646873 0.277428007 0.269345374 0.272784704
0.3111976790.309464365 0.284592868 0.255022493 0.282404914
0.268490209 0.285153368 0.247565061 0.2684112850.248443072

0.250789547 0.264608243 0.268288355 0.290298796 0.27528603
0.271935053 0.293685235 0.263089125 0.246641737
0.288583196 0.2731129710.258733872 0.259271902 0.262862494
0.25051718 0.277546302 0.266803262 0.258420084 0.259400821
0.256072048 0.265292804 0.268593165 0.284593912 0.30226301
0.285044796 0.293043976 0.305236223 0.310365015
0.294178244 0.292983179 0.28754509 0.267193136 0.235357651
0.28291385 0.283156806 0.264718696 0.280098451 0.306445228
0.271638638 0.2671725 0.281297106 0.286896621 0.269488647
0.271768072 0.288437206 0.283681743 0.272813626
0.266854619 0.282721648 0.278068363 0.281364142
0.223223692 0.28882881 0.243989966 0.285162044 0.275086808
0.281486931 0.259468279 0.267825999 0.293935987
0.300836396 0.259636434 0.255423481 0.288029298
0.271514655 0.30136815 0.285781775 0.281496918 0.303820112
0.26625568 0.288689837 0.271335807 0.286056436 0.301241649
0.259392388 0.288098433 0.276989706 0.268982555
0.263128086 0.274124821 0.246369104 0.270798279
0.286596097 0.291209929 0.24753274 0.237349334 0.290891769
0.279237788 0.227575994 0.254648659 0.216862982
KEGG_MAPK_SIGNALING_PATHWAY 0.109410893 0.141409644
0.138019891 0.111111165 0.137589191 0.1123215890.111402713
0.135454851 0.1132826280.137100577 0.109397256 0.125065565
0.124649849 0.1184525960.147250314 0.11883327 0.118037533
0.104363982 0.101463407 0.128698772 0.096347064
0.130698607 0.1108264050.1105001410.126249286 0.104337509
0.1176195550.109444747 0.1153957940.1138860080.1167076410.11298621
0.1183423210.124055023 0.149639216 0.121364191 0.10694317
0.087736051 0.108494312 0.077408419 0.12261923 0.107256669
0.125171018 0.094271689 0.123686461 0.1143577360.125236225
0.1167033580.125100833 0.095190621 0.132405201 0.11340861
0.1132007420.1193513480.1106595040.133369276 0.115828726
0.1170258160.1170000370.122473371 0.108479645 0.11402597

0.127829707 0.143696092 0.11604126 0.098418367 0.099758943
0.12414547 0.13761852 0.111266112 0.122499502 0.10841992 0.12500637
0.1105445120.124625254 0.140709864 0.1108730020.132578382
0.134212424 0.1174793810.1172614540.109897144 0.10812326
0.1149737780.1174044080.1185921950.1248211620.135601935 0.12002674
0.1169581640.1151009010.10890781 0.122140213 0.108864276
0.130239873 0.121606481 0.1100771280.1175263550.122355029
0.130688935 0.1159355920.108156489 0.103636586 0.115770358
0.103679521 0.125263271 0.094776056 0.128493141
0.120502717 0.14257406 0.102947582 0.122719658 0.099503645
0.107987882 0.121746902 0.141322329 0.1192864350.105531451
0.124303448 0.120085141 0.1115176240.122336065 0.112181898
0.124996155 0.12194471 0.090762106 0.130965502 0.114695979
0.105238397 0.120092063 0.1193031640.106817686 0.102788186
0.132003696 0.135569042 0.11015529 0.126308833 0.131717576
0.1271134690.132430777 0.1197603530.141039938 0.120653367
0.103046099 0.1031521130.145431359 0.133559371 0.113660022
0.100859436 0.128854109 0.122404608 0.1167997940.113524462
0.107550134 0.132180801 0.129791691 0.12343125 0.131718907
0.1176582090.137310816 0.1230381180.1122252570.110271439
0.135554903 0.127496755 0.103096836 0.1183210010.11056534
0.1136886270.1129841820.1190056080.121022739 0.102647819
0.109667086 0.124146324 0.136144529 0.125296501
0.142032379 0.1131017990.131904486 0.123942269 0.091214755
0.12079873 0.132578407 0.129858601 0.0905728110.120480242
0.13885144 0.1108856350.13786985 0.140697352 0.122025853
0.148079021 0.120958273 0.139865217 0.144232465
0.128582741 0.123562987 0.140692076 0.136918699
0.158938694 0.109403886 0.127254051 0.120896332
0.123552313 0.094105019 0.11660052 0.133128499 0.120928041
0.105723378 0.132219693 0.11753671 0.13251459 0.118792851
0.1158654730.14130347 0.1119758410.155585449 0.157171053
0.120965507 0.125147944 0.130028617 0.1119740570.137747423

0.152655442 0.105494174 0.1106014630.1198479310.094206418
0.156744655 0.118421 0.133904713 0.124943837 0.110482592
0.146350893 0.123902384 0.105130136 0.130152601
0.137563304 0.11163454 0.11145275 0.127971279 0.088312445
0.108002201 0.126219794 0.137030103 0.092647782
0.096662404 0.100442555 0.1109030230.129082342 0.147543093
0.139552629 0.102639568 0.11505329 0.123451945 0.117356367
0.1172630330.1137284160.133523735 0.097045167 0.099125019
0.1101709180.105224941 0.1173267910.125682499 0.107251239
0.11083695 0.1122813510.093796419 0.11341931 0.123681034
0.13367393 0.1127096450.11600158 0.098383478 0.125677502
0.1107464260.1167344620.1176647440.1153391330.11863871 0.106090134
0.123382884 0.1180647930.120763475 0.128734882 0.135379223
0.143530292 0.137922026 0.120572735 0.128020449
0.093075155 0.097610327 0.1174851810.143268516 0.120488553
0.139696838 0.124143589 0.10682394 0.1172760280.10733058
0.138949709 0.105583391 0.1162910070.134627387 0.131102582
0.1134181390.1113597770.135250818 0.131946808 0.116934297
0.126408173 0.125769103 0.133699344 0.146688584
0.101207169 0.100634942 0.123550896 0.1163943150.123113726
0.0927676 0.108135766 0.125647464 0.1161766340.108812743
0.1249117440.109524766 0.1124332270.132450768 0.118286534
0.129881623 0.123883489 0.124005039 0.154742448
0.131545795 0.124178156 0.142238762 0.1120310440.099063731
0.152744938 0.1145464950.122039355 0.1219914110.137429111
0.1162775010.124596097 0.1326987110.134337905 0.096917039
0.099758377 0.102848552

KEGG_MATURITY_ONSET_DIABETES_OF_THE_YOUNG -0.268911303 -
0.209400311-0.198519958 -0.242874672 -0.231162266 -0.22679536 -
0.224685136 -0.228799424 -0.313406801 -0.225325808 -0.231347294
-0.25233946 -0.192757505 -0.274995194 -0.243045388 -0.232388423
-0.268523225 -0.229816452 -0.209639608 -0.281873105 -
0.070821392 -0.233852402 -0.263002018 -0.201978674 -0.254889343

-0.275993765 -0.218871798 -0.268435576 -0.223509021 -
0.29146156 -0.243988132 -0.219925788 -0.232487561 -0.272702515 -
0.215645326 -0.240899599 -0.211594965 -0.27792754 -0.226244383 -
0.259453236 -0.303554904 -0.258819982 -0.241229988 -0.301851395
-0.286723891 -0.161076408 -0.195467716 -0.212766165 -
0.27871977 -0.237551226 -0.269492873 -0.294426359 -0.251669106 -
0.265855518 -0.284744377 -0.213867681 -0.253815636 -0.293789192
-0.219857722 -0.209491593 -0.29523809 -0.253277265 -0.137069807
-0.259435685 -0.258632591 -0.196619649 -0.19416807 -0.265998663
-0.284721621 -0.280584601 -0.276322138 -0.232901166 -
0.260149093 -0.295560805 -0.224786961 -0.265845221 -0.205550551
-0.256554767 -0.266457124 -0.263247774 -0.268549982 -
0.192752566 -0.192156122 -0.218501183 -0.299891283 -0.263681276
-0.236876314 -0.247070892 -0.247525557 -0.203747333 -
0.162833065 -0.238977388 -0.22204153 -0.207190692 -0.195148423 -
0.253488886 -0.270160426 -0.211496719 -0.282273488 -0.210962726
-0.251193638 -0.153362974 -0.177721765 -0.292988873 -
0.166241267 -0.268101392 -0.231956637 -0.28689431 -0.266381975 -
0.228894664 -0.174218321 -0.225567713 -0.205109835 -0.256163607
-0.260148333 -0.279809123 -0.275756158 -0.255209551 -
0.270138382 -0.242446649 -0.248660467 -0.229215555 -0.274029643
-0.259422134 -0.311060639 -0.221340621 -0.227636839 -
0.285343362 -0.229421478 -0.28181035 -0.249212668 -0.254722961 -
0.224724745 -0.270648745 -0.251724781 -0.267795362 -0.279298769
-0.267879943 -0.279599314 -0.303221117 -0.182899343 -
0.29590263 -0.289605133 -0.261544487 -0.275417055 -0.274243517 -
0.240070803 -0.247717218 -0.265906748 -0.178460912 -0.284524567
-0.332119917 -0.23384666 -0.16587537 -0.272527096 -0.194094593 -
0.248195946 -0.315127244 -0.293818787 -0.247383797 -0.285344642
-0.279471901 -0.24078924 -0.289446275 -0.305549845 -0.221717045
-0.074978419 -0.290421526 -0.305359217 -0.253775584 -
0.260390249 -0.224372868 -0.272004459 -0.210313213 -0.29709843 -
0.272838873 -0.224896944 -0.272107197 -0.202969512 -0.27804153 -

0.277157324 0.075476573 -0.281587769 -0.300731115 -0.283765948 -0.130339306 -0.251663473 -0.29939654 -0.292007528 -0.203127879 -0.278246898 -0.301348076 -0.257352297 -0.266412901 -0.30102275 -0.260324557 -0.24632193 -0.230769437 -0.289343769 -0.225132568 -0.305847473 -0.251258521 -0.250504525 -0.223588132 -0.2530969 -0.204729036 -0.265920979 -0.170493834 -0.290231835 -0.262185513 -0.233083924 -0.246984271 -0.2516944 -0.162026792 -0.274136228 -0.228596659 -0.270460747 -0.23722509 -0.26729076 -0.254062362 -0.264041159 -0.188578287 -0.189839657 -0.237717896 -0.285479508 -0.2643669 -0.291896744 -0.279601149 -0.227079529 -0.304349891 -0.1981667 -0.297458879 -0.247943248 -0.25670336 -0.250425759 -0.252362774 -0.12605128 -0.238336482 -0.313440616 -0.291821036 -0.2733795 -0.246809667 -0.212342013 -0.279264573 -0.295650046 -0.244955407 -0.24617633 -0.18550041 -0.201498072 -0.194385314 -0.293287753 -0.282888794 -0.211287394 -0.206300465 -0.126824373 -0.234508895 -0.218434428 -0.222385867 -0.246469183 -0.300966984 -0.094703136 -0.259011648 -0.226355964 -0.255845551 -0.238819771 -0.228095024 -0.256384923 -0.247708627 -0.260674969 -0.204177197 -0.250310642 -0.201797383 -0.274957147 -0.241968538 -0.229421257 -0.265495464 -0.283313982 -0.251132483 -0.231990289 -0.227504008 -0.238911649 -0.275477574 -0.282407432 -0.278145169 -0.257846498 -0.297240544 -0.234317101 -0.322550548 -0.259100757 -0.255022243 -0.28109517 -0.336390422 -0.274024967 -0.249389651 -0.27845224 -0.230214856 -0.160401974 -0.279170087 -0.264145792 -0.310383058 -0.297790447 -0.193667586 -0.24870266 -0.223966382 -0.235320081 -0.280415667 -0.311945049 -0.225601649 -0.226394501 -0.208542172 -0.271938683 -0.271359116 -0.141653449 -0.15209707 -0.28820362 -0.274921809 -0.267072635 -0.31579561 -0.247770268 -0.280212755 -0.251316544 -0.270594032 -0.225186287 -0.273677963 -0.264810786 -0.290684607 -0.228960633 -0.195818054 -0.28428621 -0.246706261 -0.271102555 -0.253492388 -0.234884035 -0.298004921 -0.284145531 -0.269103845 -0.240992183 -0.232819469

-0.240334588 -0.092049195 -0.1805253 -0.23281428 -0.212204075 -
0.187391582 -0.246872777 -0.27036315 -0.246286831 -0.16609137 -
0.228769982 -0.251641897 -0.286503965 -0.255583898
KEGG_MELANOGENESIS 0.0911969590.1193656390.107691953
0.089647489 0.070691206 0.077630572 0.11958351 0.115589591
0.083699787 0.0950471150.083434083 0.08004788 0.121308775
0.124802203 0.097448588 0.068186252 0.10646499 0.083959409
0.124771481 0.1191046820.133423937 0.087892851 0.074884474
0.107432567 0.102339158 0.074915102 0.094902088
0.081368897 0.085338089 0.096945932 0.097707727
0.093043541 0.097218916 0.135372092 0.1190715860.073906565
0.124017606 0.066817455 0.084877323 0.089854562
0.106565023 0.075654754 0.078577854 0.075566224
0.107355885 0.086910102 0.122976931 0.096213322 0.07068246
0.0825114730.089585708 0.049779795 0.074545594 0.11145918
0.056428757 0.1114853960.05503969 0.07858023 0.065512959
0.121905956 0.056825268 0.10426356 0.1179300010.085408376
0.089994564 0.086797173 0.0603119150.080293916 0.092331552
0.099869334 0.088629404 0.107772435 0.094339639
0.100460994 0.072428081 0.107831371 0.1166087830.114272581
0.1155726270.106141454 0.068490908 0.070187238 0.1315433
0.097476871 0.047395943 0.076410523 0.100907054 0.11769991
0.121099809 0.126071457 0.07950525 0.076232627 0.104229089
0.0721167540.079542177 0.109454008 0.054913646 0.108592012
0.066287566 0.05866849 0.092343255 0.089878619 0.071585851
0.098649824 0.098493453 0.106302333 0.081742892 0.09640614
0.072210789 0.097481042 0.054290215 0.07121695 0.090046432
0.084386859 0.100268287 0.095982204 0.071488894
0.095310986 0.085630591 0.099190146 0.087761389
0.1011790080.100906255 0.1196522460.074582837 0.048510894
0.079105233 0.100261689 0.089013736 0.068903183
0.1137326750.103958275 0.091078351 0.076600328 0.09920701
0.1163140080.0851128790.09398793 0.123641618 0.102269108

0.087379898 0.141292092 0.046292876 0.103523663

0.052437799 0.106490814 0.102418973 0.086623624

0.087604525 0.083781067 0.084372424 0.064593764

0.092633133 0.094539861 0.10898849 0.08724456 0.130124797

0.079948287 0.064394466 0.107716957 0.0772118740.103254592

0.077674341 0.1126011870.108822187 0.072970304 0.148802831

0.081755692 0.096984337 0.1102170420.070284665 0.081877057

0.085632316 0.021438069 0.063023841 0.096016868

0.095070256 0.1163365390.056556396 0.121464015 0.075696094

0.093822532 0.057663185 0.1118808090.047929658 0.093480093

0.103679036 0.1142749670.109384463 0.087480182 0.117174303

0.074396993 0.076614551 0.06993461 0.108774065 0.107768737

0.081821902 0.083764681 0.1195277490.057779128 0.11140472

0.078698832 0.080618129 0.091388274 0.09651869 0.096043382

0.124890339 0.123083005 0.101874892 0.096764476

0.105041627 0.08432056 0.105227686 0.1169564830.080036143

0.1136109730.103278745 0.104897467 0.136616398 0.11700481

0.1178995040.08010439 0.104454834 0.109295213 0.143469267

0.12108523 0.1169982220.070426182 0.077320409 0.13158723

0.092295545 0.1131838340.123748081 0.06364155 0.132939638

0.106187573 0.1145871270.098591344 0.107458854 0.096775241

0.04946529 0.098940998 0.084041274 0.065673016 0.111778969

0.07099377 0.073463093 0.087132384 0.066271452 0.089818629

0.096049851 0.0635811290.06774351 0.045676874 0.093954187

0.10920591 0.0894545 0.09287572 0.085612517 0.082183067

0.089934016 0.108608246 0.0574953 0.085421685 0.059247963

0.097829366 0.036500638 0.065129487 0.108598271

0.103552051 0.079277626 0.085235737 0.121305022

0.076005917 0.085486105 0.096588265 0.1109059370.063245874

0.1100316 0.086721931 0.079866536 0.106282965 0.075912038

0.1153254560.094717968 0.064427047 0.084416239 0.073203762

0.102080823 0.106922292 0.101665942 0.122238397

0.076181336 0.098045965 0.073305069 0.1160946140.116898698

0.100525779 0.095019541 0.082953505 0.077762973
0.093862593 0.1154826760.0821121170.095523758 0.083937411
0.068048624 0.096386264 0.081425639 0.125360012
0.120316405 0.122452293 0.11143237 0.067473219 0.124443876
0.138100007 0.051017736 0.094135268 0.101641729
0.101735733 0.098486879 0.0388113760.101702423 0.11513497
0.071571048 0.0792111240.074606612 0.056528685 0.066014249
0.098385776 0.1196315770.0863114930.09941088 0.097791482
0.1154215620.1157259730.096357427 0.103535435 0.112636433
0.052493036 0.143777946 0.13064262 0.094289064 0.128373609
0.086176668 0.07822949 0.1013311350.100538347 0.103378777
0.130404596 0.077721804 0.086458354
KEGG_MELANOMA 0.054636508 0.106930841 0.094550533
0.062936534 0.068496756 0.101748896 0.080200472
0.059679715 0.071745722 0.079661067 0.055699945
0.061398528 0.09183367 0.073793188 0.091529016 0.078055654
0.1104602690.04581177 0.062862992 0.0968696 0.108057181
0.067668434 0.071760042 0.099580316 0.104595086
0.073142962 0.073088324 0.0681114010.09769035 0.074083652
0.094739261 0.070618889 0.1021140110.104945017 0.109849409
0.031853239 0.096623412 0.019808294 0.056558525
0.071097856 0.095310612 0.045517469 0.04990956 0.050246178
0.085228303 0.073254758 0.107860365 0.06308056 0.051475189
0.090199495 0.099073965 0.075216532 0.074247486
0.075057352 0.067637891 0.1139883070.071327405 0.093567495
0.056944625 0.1141127090.079666688 0.096866699 0.087645903
0.086720857 0.078042634 0.072952705 0.027686212
0.053287988 0.066606384 0.095853366 0.091701072
0.080182686 0.102094239 0.068148054 0.0968116 0.117717229
0.082609557 0.084382018 0.1157013250.098901463 0.051647674
0.052418775 0.091066041 0.083868988 0.075017056
0.062052921 0.099980865 0.103237086 0.0990307 0.111232282
0.05509673 0.077674015 0.078278022 0.051315543 0.082485847

0.089544368 0.073833527 0.078272491 0.069241269

0.074458569 0.080565308 0.094000663 0.076201751

0.091306976 0.075924493 0.072632188 0.053813907

0.075306231 0.080061399 0.07981099 0.04546737 0.089075761

0.069723258 0.08539983 0.073563933 0.102839937 0.077630731

0.05596686 0.089140793 0.088461349 0.0882796 0.075608428

0.076768773 0.1128740190.090102419 0.04021907 0.078965797

0.093149793 0.056630677 0.080345253 0.080151626

0.056510389 0.067628345 0.086384878 0.1161818080.076490466

0.101596196 0.099328698 0.1179875350.1127361990.075718231

0.127837081 0.078825697 0.08547969 0.0814581140.129873753

0.095435522 0.063974522 0.07050323 0.083744145 0.067733276

0.076577039 0.10434077 0.0875411580.099106862 0.096417793

0.128837478 0.085816622 0.069600604 0.1108759530.064450217

0.087577891 0.077584775 0.100949214 0.077039862

0.0271110190.121569596 0.077464035 0.08311338 0.08630895

0.09230371 0.072662646 0.053678053 0.055985224 0.063131639

0.09549248 0.080904027 0.102465385 0.0615267110.091141864

0.09083018 0.074492588 0.087632243 0.074205481 0.082620128

0.0886311090.094273742 0.107447196 0.049639691 0.113877835

0.103137783 0.090752055 0.095643356 0.086781886

0.086099438 0.12584389 0.057308221 0.096529444 0.104585149

0.090520009 0.104892594 0.064618332 0.1033728110.098488489

0.085787746 0.097741874 0.092302836 0.095633401

0.101964182 0.056188678 0.107735695 0.07700252 0.104545081

0.1151643550.101042915 0.1127125860.082956395 0.129293582

0.149639727 0.1094593 0.126458706 0.09406959 0.079962996

0.069407012 0.108472637 0.089678733 0.098086833

0.057206546 0.087482214 0.130265712 0.0651159060.073391753

0.096543683 0.0722521190.13087115 0.092907851 0.096591417

0.1135147420.098882978 0.086071342 0.080947894 0.084561903

0.078058612 0.076806226 0.086874178 0.1193513480.029154198

0.074193533 0.070035699 0.069853296 0.087164655

0.105397466 0.086490569 0.052238816 0.076305371
0.097590152 0.080444583 0.10578304 0.07855115 0.103506183
0.071521814 0.089018155 0.084806934 0.076662185 0.055247
0.091537812 0.045528459 0.076340121 0.070030666
0.081253982 0.078284905 0.059236176 0.137310656
0.060856854 0.095055819 0.09322472 0.090567222 0.062759561
0.09535101 0.08716892 0.06830044 0.1104771550.1010624 0.088900793
0.089781705 0.067264043 0.093536048 0.103466923
0.100906547 0.1102096840.082177752 0.1136596920.053533187
0.104326325 0.077468333 0.128727634 0.130837094
0.1146092350.076909741 0.088560002 0.044254477 0.065425895
0.1125546650.071562873 0.070685061 0.0766921 0.096795343
0.079841542 0.089478761 0.104513622 0.088330133
0.086479031 0.092088613 0.078669803 0.095955285
0.1189361490.086068362 0.074426508 0.073626476 0.111966166
0.086134471 0.068234366 0.083960452 0.104407499
0.045307286 0.053723173 0.07605591 0.056387217 0.076615186
0.099261373 0.094750535 0.102959241 0.0853814110.082460475
0.1151026450.095138165 0.085235173 0.094368553 0.046902024
0.059189158 0.090766438 0.1119031090.087146218 0.095667438
0.09736628 0.07289738 0.104684831 0.101304257 0.111095784
0.072235043 0.065695448 0.055353499
KEGG_METABOLISM_OF_XENOBIOTICS_BY_CYTOCHROME_P450 -
0.09635046 -0.108361468 -0.085888888 -0.101454837 -0.076580099 -
0.087561212 -0.08261223 -0.133475307 -0.060111728 -0.112322456 -
0.111877376-0.135681742 -0.134689509 -0.085801308 -0.120170937 -
0.056754234 -0.105724318 -0.066634083 -0.1088165 -0.085437919 -
0.053616262 -0.089602924 -0.035553441 -0.121060697 -0.092575564
-0.104580822 -0.080994581 -0.074974424 -0.083645576 -
0.100793944 -0.05652107 -0.143780766 -0.129872956 -0.070045837 -
0.097070729 -0.098435012 -0.088450953 -0.091711673 -0.083611913
-0.106130213 -0.117245366 -0.065257404 -0.0794594 -0.058644212
-0.105277011 -0.092610799 -0.125376992 -0.086839388 -

0.077070712 -0.070224985 -0.081847959 -0.093661956 -0.109279459
-0.070856472 -0.065829653 -0.112180907 -0.11025346 -0.05646137 -
0.095754448 -0.123797221 -0.051641738 -0.140521485 -0.104801109
-0.109274277 -0.124251664 -0.117031954 -0.074939127 -
0.060531496 -0.112390494 -0.103701256 -0.107752311 -0.089831596
-0.1059538 -0.060852151 -0.10675547 -0.097830049 -0.096124262 -
0.11993383 -0.080061383 -0.071453918 -0.072542045 -0.102164323 -
0.14083969 -0.112467334 -0.075192506 -0.126110804 -0.120888927 -
0.052092707 -0.090433773 -0.073580277 -0.124536667 -0.081961831
-0.068833286 -0.075699648 -0.110647604 -0.132369984 -
0.069510348 -0.134656131 -0.104182106 -0.103963894 -0.117570661
-0.156546584 -0.027861412 -0.097242388 -0.102648794 -
0.073606456 -0.061429079 -0.089755105 -0.111724848 -0.100478101
-0.081987287 -0.131385573 -0.135734707 -0.113489209 -
0.119322919-0.099233651 -0.085132333 -0.101360677 -0.152578364 -
0.124385218 -0.104142183 -0.150911674 -0.090563553 -0.140634458
-0.152033193 -0.066520032 -0.110159318 -0.105330018 -0.0940648
-0.076992695 -0.143056938 -0.095349947 -0.080138151 -
0.139198531 -0.115128928 -0.099511904 -0.136990909 -0.107913049
-0.122115315 -0.083087722 -0.112752004 -0.097845317 -
0.136059659 -0.099798089 -0.109421897 -0.120639231 -0.158808051
-0.065181263 -0.130781124 -0.125489521 -0.09684463 -0.109044545
-0.130038526 -0.072637009 -0.120895578 -0.151648709 -
0.142908649 -0.110299329 -0.123329172 -0.066009107 -0.111869981
-0.120411201 -0.071609169 -0.06702896 -0.109007263 -0.051002043
-0.11728513 -0.066116103 -0.107067585 -0.046933453 -0.14656843 -
0.094385563 -0.113872118 -0.085692434 -0.10681289 -0.13273402 -
0.091405847 -0.061446154 -0.062483118 -0.170888634 -0.127727369
-0.067285973 -0.112971328 -0.085079621 -0.07930494 -0.104898012
-0.090401758 -0.068939679 -0.088862277 -0.073462902 -
0.129651241 -0.096970658 -0.104350161 -0.110803012 -0.084289143
-0.121129512 -0.116965033 -0.117737362 -0.126274395 -
0.137092627 -0.102676964 -0.142264659 -0.123541017 -0.139448966

-0.107879925	-0.086364806	-0.116642257	-0.084230432	-
0.109366054	-0.079488225	-0.138722759	-0.116541396	-0.131096008
-0.085875879	-0.139096953	-0.120673788	-0.153528976	-
0.123881363	-0.090000933	-0.128333901	-0.133624864	-0.093329463
-0.107246832	-0.100293236	-0.105355101	-0.086514382	-
0.131611997-0.11041297-0.091804592	-0.093880493	-0.08639199-
0.111814813-0.109454542	-0.125940157	-0.09308796-0.108513109	-
0.091306035	-0.122408104	-0.125831102	-0.083472348	-0.107785609
-0.087240558	-0.09960548-0.118028032	-0.133670009	-0.098453039
-0.061939462	-0.105726097	-0.032864527	-0.117886815	-
0.127333623	-0.129191902	-0.118219009	-0.108748567	-0.089943709
-0.087992235	-0.087680794	-0.073399623	-0.090966094	-
0.103500694	-0.073744314	-0.09108605-0.066937345	-0.097448026	-
0.114989996-0.114433828	-0.096578811	-0.121690289	-0.099119771	-
0.083600619	-0.07780477-0.141637609	-0.153714825	-0.084622239	-
0.111417489-0.089729096	-0.081323702	-0.118906812	-0.163439799	-
0.120805686	-0.093246484	-0.088717967	-0.070075265	-0.11169452-
0.138443684	-0.141229691	-0.152213225	-0.13981081-0.122818223	-
0.100218501	-0.109236142	-0.09601003-0.109292386	-0.12264992-
0.123906165	-0.098199781	-0.135030528	-0.105277022	-0.091291549
-0.14713372-0.095508384	-0.117287913	-0.121875369	-0.100031372
-0.122018803	-0.12648805-0.09781484-0.099035527	-0.131550429	-
0.122454034	-0.09017657-0.094781886	-0.110746751	-0.127282307	-
0.153921109-0.073654172	-0.13569123-0.090760322	-0.128729837	-
0.120547105	-0.102805646	-0.040219233	-0.093268986	-0.084540425
-0.134863715	-0.142283566	-0.129417577	-0.058839033	-
0.101233375	-0.106734883	-0.100755269	-0.118797638	-0.136441284
-0.107842707	-0.124395034	-0.129203969	-0.12280421-0.124600158
-0.100062522	-0.006939286	-0.109482602	-0.104627062	-
0.110107339-0.16553365-0.120769868	-0.093395412	-0.076013119	-
0.084734749	-0.163588452	-0.060201378	-0.102930141	-0.107213399
KEGG_MISMATCH_REPAIR	0.274353346	0.201472091	0.240505111
0.2540811580.223602261	0.278953302	0.286289026	0.229543909

0.267488856 0.243904003 0.296416169 0.306496025 0.29452315
0.25779917 0.268967093 0.291769619 0.251976429 0.219732013
0.267235705 0.27772505 0.29285913 0.222794603 0.255135934
0.291277376 0.286056831 0.290068821 0.298078895
0.282486128 0.287459691 0.2116813470.320715531 0.289118626
0.299209754 0.259872894 0.276659661 0.255881284
0.2861141950.22273107 0.270064481 0.268159572 0.243158958
0.253174154 0.236939652 0.288155967 0.21470474 0.252191022
0.280199978 0.19173444 0.228645823 0.28493212 0.203717686
0.244870484 0.288479155 0.260923052 0.281204748 0.24854442
0.291041282 0.224958079 0.28187319 0.246269603 0.279479769
0.27073624 0.276071858 0.25091007 0.30433984 0.273534299
0.242954803 0.2711939390.229583277 0.297851248 0.281146383
0.295962127 0.233763836 0.264856321 0.259710713
0.223020768 0.233236705 0.271334948 0.2118904460.260811013
0.270588643 0.319464576 0.310184255 0.270612737
0.245492199 0.3116990720.2712117860.234404854 0.239989635
0.267605171 0.307352139 0.259387347 0.262804444
0.273098235 0.19290038 0.30640563 0.286202354 0.268144583
0.230523237 0.260677133 0.283645574 0.294929049
0.255353793 0.219708491 0.266097153 0.223044786
0.279678393 0.258092819 0.276284812 0.241623379
0.286703682 0.277107443 0.30515879 0.265145336 0.273030171
0.236958524 0.26898665 0.22010024 0.249891858 0.261921508
0.257368896 0.315968997 0.243431039 0.253472339
0.284513882 0.26379055 0.2656114780.240082712 0.273200979
0.231363336 0.269774053 0.238012305 0.26203335 0.268835487
0.277823765 0.275941823 0.2095161190.227332284 0.291166305
0.228770329 0.250922647 0.215143025 0.3211794140.285541015
0.290402404 0.230808066 0.233028139 0.233693894
0.215994856 0.300458566 0.2712711440.29796367 0.324545871
0.291055001 0.21312691 0.273744268 0.243827655 0.288266436
0.296556879 0.22035034 0.288257401 0.226592364 0.268527601

0.257847242 0.267266224 0.2429151160.24740126 0.316871935
0.238206188 0.287325653 0.269266764 0.2685705 0.242338791
0.27234424 0.295243319 0.243096246 0.248945969 0.25547448
0.278354413 0.244456682 0.200945907 0.300656655
0.266382689 0.226686346 0.284515946 0.282255933
0.293974949 0.240892701 0.286000187 0.240636012
0.249313522 0.286027985 0.260476406 0.236771379
0.2117235540.234408796 0.245658191 0.225965985 0.217182955
0.264413704 0.205733308 0.244208824 0.226184068
0.265678002 0.217400281 0.2808411960.290425973 0.237786988
0.203322808 0.254169052 0.2555011220.232521758 0.264839156
0.257435352 0.260945831 0.191300525 0.248221015
0.243493143 0.191836303 0.269244987 0.276270283
0.229828488 0.236467838 0.230819015 0.180340322
0.258843161 0.260868625 0.242036989 0.2926413110.181946917
0.276758271 0.235205655 0.300594354 0.26158682 0.237018706
0.228573638 0.275905027 0.2630571190.236533284 0.229659406
0.277903926 0.210643332 0.317555869 0.254691078
0.209068779 0.275567566 0.257892828 0.328914062
0.288732584 0.275819751 0.268301201 0.2119481430.234604351
0.266005431 0.302774097 0.222233971 0.245534777
0.226355733 0.323023658 0.296250078 0.314746376
0.243410103 0.263682454 0.276077413 0.30467929 0.218046429
0.280689517 0.268996356 0.205446891 0.253461002
0.247800484 0.253662271 0.267009927 0.284933989
0.275950447 0.270985534 0.217767742 0.3131121560.239313434
0.223310646 0.28819311 0.225528554 0.239786166 0.270753466
0.254258894 0.252431957 0.245487352 0.2581468 0.257205788
0.219519973 0.221451263 0.209785053 0.263872575
0.263701381 0.2778011980.255693371 0.2940299110.241328769
0.26997839 0.302564656 0.281590143 0.225548074 0.220044937
0.232920989 0.234598925 0.217398408 0.2686110060.302319192
0.271884154 0.203005325 0.207283913 0.21913912 0.283829968

0.300870276	0.297131428	0.195068426	0.227649554
0.289658663	0.252779421	0.27065806 0.276473831	0.242030435
0.298052103	0.299061926	0.26446887 0.26373866 0.24664916
0.257262498	0.257741765	0.27764656 0.284610655	0.262247232
0.296427396	0.252875628	0.23289375 0.256212861	0.233072041
0.248209191	0.241491034	0.270145612	0.1973763 0.269195269
0.235219712	0.283147382	0.252260073	0.301693033
0.287757306	0.236392584	0.250309321	0.259542598
0.267850938	0.2918608
KEGG_MTOR_SIGNALING_PATHWAY 0.159771578	0.171227001
0.156376692	0.151708821	0.159861978	0.168846959
0.159418695	0.178666559	0.159100789	0.182564178
0.138216513	0.170609412	0.167298105	0.1491311070.173920988
0.154641885	0.144620301	0.180671574	0.146910179
0.179881712	0.1709437110.189061832	0.162886905	0.17010078
0.177083166	0.170066227	0.154264791	0.166417637
0.1881669110.187839785	0.146344059	0.1671194180.164793232
0.155541662	0.159799242	0.155163825	0.136078572
0.140313539	0.133337404	0.147487933	0.1847793110.159175716
0.161857766	0.149646554	0.178174663	0.16432849 0.158074817
0.135098007	0.165053531	0.160646268	0.181981585
0.155635794	0.187103425	0.149708874	0.176778995
0.169501356	0.1548502 0.172938559	0.1343110770.161061418
0.17191663 0.176096925	0.15628978 0.166378134	0.167157294
0.1425114230.13809367 0.151904505	0.169495105	0.153642016
0.201612463	0.141362727	0.181008964	0.160364781
0.146979386	0.1991139920.161632962	0.176640347	0.167852744
0.177362956	0.152124712	0.140154209	0.165147053
0.170318604	0.16487394 0.152100563	0.19252676 0.146419467
0.164084008	0.164526715	0.145868666	0.161419631
0.151824364	0.155540372	0.179919108	0.173679626
0.157684487	0.150618496	0.170669757	0.165120969
0.188527206	0.180721751	0.163972169	0.17860874 0.156622611

0.174534773 0.149747189 0.1680114910.1515714 0.166081957

0.160067595 0.184352446 0.146183212 0.1695905110.166012546

0.160702549 0.1651177480.138581251 0.164565103 0.170199187

0.159036547 0.17625643 0.17492695 0.184104837 0.186847112

0.138062873 0.17808065 0.173426604 0.160287721 0.193760369

0.172152268 0.156559518 0.146885617 0.166479366

0.187932166 0.172228703 0.180755876 0.166856445

0.165244729 0.193202349 0.15761049 0.192481027 0.175121006

0.189306576 0.192376402 0.192342327 0.170386256 0.15172677

0.183027638 0.172181948 0.166919802 0.210557323

0.196094042 0.152537543 0.173847497 0.183248759

0.167903196 0.176910627 0.181501601 0.188218631

0.164737502 0.180147308 0.182531031 0.179694405

0.163810757 0.129440757 0.145305539 0.192662947

0.162459098 0.177862064 0.18793055 0.174857992 0.15547268

0.182619195 0.1711948510.171514852 0.171221349 0.161699322

0.157837801 0.187527227 0.176988773 0.162372109

0.173975619 0.151887322 0.206656182 0.12747288 0.169213385

0.194957979 0.162962722 0.186879088 0.182681841

0.179299237 0.19396351 0.19271257 0.183026062 0.186739708

0.168463861 0.187250345 0.207732598 0.194399487 0.18849676

0.172892799 0.200773938 0.171675787 0.172958851

0.176092657 0.1316295 0.1608935110.188126592 0.186609949

0.182263833 0.167157928 0.176220008 0.16396822 0.179120147

0.196343586 0.178209452 0.174172662 0.19509447 0.170998762

0.167370254 0.17164729 0.172144233 0.1761104290.178910136

0.160834812 0.17984599 0.168856789 0.130369756 0.211441899

0.163008337 0.194777277 0.169065395 0.162766646

0.1753061190.188509699 0.176663816 0.169781661 0.179238792

0.175598954 0.166678295 0.1678939110.156081564 0.161321731

0.179344916 0.195871888 0.134376759 0.1811562370.149727549

0.167463734 0.185836538 0.190385704 0.182568726

0.178276332 0.141534423 0.168716181 0.158590261

0.146137501 0.147008157 0.186163839 0.153037283
0.148280182 0.149056585 0.181201366 0.154148918 0.17272178
0.158195209 0.151676681 0.153455884 0.164014748
0.163791919 0.166763593 0.185450185 0.156940504 0.1691362
0.167001208 0.150724251 0.171278122 0.16854309 0.189631675
0.152293759 0.180155601 0.178316859 0.177824356
0.180092869 0.177495796 0.193970067 0.197854956
0.181224981 0.195956156 0.186890357 0.1911080890.1525026
0.160584533 0.171370746 0.17272243 0.18001089 0.172426708
0.182240524 0.193725959 0.176755169 0.156807356
0.190798724 0.149177953 0.1711830130.192906321 0.184540682
0.160136469 0.166934806 0.16853635 0.164695384 0.15656798
0.150160839 0.182077284 0.181882401 0.196063926
0.186682987 0.138709105 0.166638246 0.18851861 0.179319822
0.179994138 0.158647858 0.189417659 0.145515323
0.186662608 0.170804171 0.159768725 0.183910686
0.169575047 0.194560033 0.177790908 0.19396126 0.179177467
0.17957367 0.196652636 0.168486551 0.171440552 0.165330725
0.196296845 0.178521447 0.174162471 0.174219015
0.193107675 0.186949432 0.16690656 0.17890731 0.176527766
0.178993161 0.158279617 0.171703444 0.171332845
KEGG_N_GLYCAN_BIOSYNTHESIS 0.267582797 0.267461028
0.270285738 0.266638914 0.272174857 0.275323855
0.271407947 0.272340196 0.270888732 0.285478363
0.272909239 0.282802304 0.259337508 0.271216061
0.266133848 0.255375202 0.277150559 0.302722097
0.296038527 0.262575384 0.294178607 0.291070137
0.288635403 0.270684646 0.29404906 0.272017949 0.273739148
0.264406752 0.282478107 0.301582844 0.279867824
0.285425078 0.275979404 0.275073519 0.26335004 0.271834996
0.253426874 0.265420314 0.282774543 0.2753431130.284920537
0.269151789 0.2611545250.292594149 0.265399082 0.252912379
0.259005954 0.248793178 0.263612858 0.274275746

0.242852256 0.245138892 0.254704524 0.249106634

0.258880832 0.2678336 0.270354995 0.25895298 0.267063751

0.264336693 0.27404193 0.2996718 0.272469963 0.266591386

0.295859639 0.267493471 0.284679438 0.2742116370.274418402

0.30114079 0.3029117420.259776422 0.269689523 0.22787584

0.266032787 0.296216148 0.26398285 0.282649957 0.268928022

0.264801388 0.264892779 0.272874975 0.282172162

0.274056716 0.261972225 0.292124743 0.261401868

0.260986043 0.271013641 0.285648062 0.290315755

0.270650224 0.25603194 0.230067019 0.287312994 0.293951925

0.273306567 0.291248796 0.306085596 0.266563926

0.284167479 0.284659065 0.288180224 0.298255986

0.280949426 0.2892448 0.257871587 0.304544138 0.27587618

0.27712979 0.270440928 0.273445396 0.28092113 0.283039506

0.262522599 0.278654195 0.276125456 0.274332062

0.285605913 0.273516806 0.273220986 0.25167162 0.283240425

0.295188192 0.278363307 0.267172234 0.267333836

0.250963128 0.292043303 0.260098495 0.2811395610.259749369

0.275210078 0.275471792 0.275974692 0.292503122

0.272288317 0.281708666 0.2588111570.283523787 0.251724199

0.286180729 0.299048615 0.291534769 0.26912159 0.294996411

0.295819324 0.254898037 0.281366982 0.271270147

0.272380549 0.27849521 0.2923561150.285810395 0.284995443

0.295594737 0.3061188190.288654462 0.2871811640.278493666

0.299662446 0.260577305 0.277346014 0.274929803

0.3055044110.272674372 0.3010126 0.278070563 0.272757047

0.281519443 0.275791884 0.276280848 0.290243078

0.275297506 0.281777832 0.30924655 0.277588054 0.235232398

0.275808644 0.275538295 0.276225195 0.295556127

0.287144972 0.286785437 0.289314158 0.294374409

0.277974712 0.267960378 0.270496918 0.280375517

0.301971608 0.274278801 0.287431214 0.277122256

0.278871797 0.266238653 0.274071338 0.264160505

0.289825368 0.289236931 0.27828829 0.283893083 0.268726529

0.264966862 0.272803333 0.28265492 0.268324391 0.266532902

0.277729863 0.26577277 0.269219586 0.266816993 0.275798329

0.269214486 0.300529712 0.283368508 0.29699796 0.258850205

0.279594897 0.29335847 0.281331425 0.291950238 0.289984723

0.3001263 0.274289156 0.280297693 0.300667087 0.251576447

0.255415688 0.28919132 0.294420898 0.2674134 0.283699727

0.279916574 0.293101018 0.281396386 0.285956845

0.278323098 0.2777110230.295747326 0.271692773 0.290833314

0.295935246 0.2873116990.29957373 0.28257844 0.303411473

0.296022747 0.297567557 0.284526053 0.280890544

0.274133215 0.281487558 0.283328667 0.282001465 0.2805758

0.277144964 0.281864918 0.300977722 0.274326022

0.272689235 0.309744863 0.278275813 0.289650258

0.297569444 0.27573065 0.2896822110.283616229 0.265041455

0.284493207 0.291612524 0.270516584 0.288303849

0.276516846 0.286308987 0.277838187 0.282185635

0.290819381 0.302804562 0.284084831 0.253106676

0.288781805 0.272623595 0.305218395 0.303701396

0.286125065 0.280878483 0.263954425 0.297069229

0.292796131 0.2857463110.280373768 0.284009797 0.287397945

0.3054252110.292142083 0.299368351 0.281676404 0.269964513

0.30675062 0.2575411130.307757213 0.29771623 0.277426401

0.26658405 0.285334762 0.269393145 0.283624279 0.274960108

0.280078507 0.277519726 0.290158874 0.278557313

0.287590136 0.2840211720.265486617 0.278324538 0.284681396

0.289872618 0.273105455 0.292533218 0.31097554 0.268458076

0.297692266 0.299152533 0.2685118390.273501229 0.295078647

0.288733987 0.29507703 0.267192184 0.297130273 0.306077171

0.289737242 0.274462271 0.301436156 0.288324298

0.307055648 0.25826455 0.300476702 0.271028024 0.266986506

0.286450246 0.26474173 0.294250599 0.279717259 0.267128588

0.278232342 0.270486327 0.287710646 0.278563214 0.25550345

KEGG_NATURAL_KILLER_CELL_MEDIATED_CYTOTOXICITY -
0.12698733 -0.13232699 -0.078588438 -0.118629296 -0.101993582 -
0.154995626 -0.156727001 -0.093881899 -0.076535547 -0.095390565
-0.156977834 -0.041534929 -0.14599182 -0.138986613 -0.073122821
-0.109945573 -0.141982999 -0.086202959 -0.088933717 -
0.148926377 -0.172879633 -0.070663925 -0.068379636 -0.144812243
-0.041825592 -0.082642263 -0.101788487 -0.10331926 -0.116618989
-0.092081569 -0.158007529 -0.04908007 -0.139164954 -0.148261862
-0.099183567 -0.081289447 -0.117969395 -0.106791381 -
0.043372411 -0.144135893 -0.083879854 -0.100365709 -0.114728851 -
0.119607182 -0.060789885 -0.125698526 -0.124369034 -0.119698073 -
0.076783173 -0.103830737 -0.039246556 -0.091686994 -0.072026654
-0.142460372 -0.049519721 -0.110800642 -0.056144789 -
0.058089944 -0.109178378 -0.166955816 -0.07777038 -0.033981406 -
0.100761319 -0.057563875 -0.077436454 -0.137061023 -0.061609542
-0.065387563 -0.088005746 -0.105746821 -0.040289742 -
0.143141094 -0.112844811 -0.152675927 -0.079266664 -0.031925827
-0.128322976 -0.0903639 -0.127157502 -0.12696682 -0.052176001 -
0.127896499 -0.120959774 -0.089259292 -0.028167212 -0.083265918
-0.02267266 -0.087457167 -0.116869831 -0.118180352 -0.08448701 -
0.057286156 -0.107729663 -0.080709162 -0.083681094 -0.056613657
-0.08204447 -0.097689148 0.024137214 -0.025599462 -0.091528341
-0.11609531 -0.13510409 -0.087896922 -0.147604335 -0.07688344 -
0.113588837 -0.081018321 -0.048412513 -0.066301233 -0.105388172 -
0.093570462 -0.178184913 -0.123376753 -0.149167934 -0.073982215
-0.080573426 -0.087802356 -0.04671806 -0.100480579 -0.057017877
-0.118772396 -0.120320572 -0.092130106 -0.072710738 -
0.104234452 -0.099392764 -0.121571399 -0.11832406 -0.04087283 -
0.109030562 -0.089120599 -0.109572241 -0.029885648 -0.101919931
-0.142025472 -0.025636826 -0.091493648 -0.108077303 -
0.087562039 -0.125250443 -0.097799165 -0.039294671 -0.15125094 -
0.078040512 -0.081994465 -0.062503115 -0.07425695 -0.122696438 -
0.076049427 -0.055124021 -0.097576182 -0.084530809 -0.143778969

-0.10270446 -0.001350668 -0.094863264 -0.017599046 -0.10679308 -
0.044396174 -0.104089232 -0.118135367 -0.108802378 -0.066665985
-0.077956874 -0.067523222 -0.136423359 -0.095407689 -
0.095622547 -0.101243413 -0.039194173 -0.084819535 -0.143381554
-0.073677575 -0.035766296 -0.05733942 -0.07391699 -0.106128775 -
0.054784619 -0.10460467 -0.068698911 -0.144770886 -0.064015211 -
0.105929867 -0.053720406 -0.134568851 -0.125052527 -0.076548402
-0.134489911 -0.102518086 -0.095861183 -0.054545022 -
0.03694361 -0.04960961 -0.076026278 -0.117939682 -0.050824916 -
0.077613417 -0.081288973 -0.076467962 -0.079466398 -0.123310647
-0.096086205 -0.116982357 -0.094335125 -0.137511136 -
0.077283333 -0.123397425 -0.107134462 -0.098855132 -0.097903026
-0.124881948 -0.087752659 -0.13306436 -0.092548 -0.081761597 -
0.113317819-0.040474249 -0.076432183 -0.122500133 -0.090547472 -
0.071307099 -0.114846924 -0.121043342 -0.091226627 -0.159014747
-0.095803735 -0.111590984 -0.105590956 -0.091229832 -
0.080215484 -0.084718971 -0.112915503 -0.071179686 -0.098318404
-0.034517515 -0.111108253 -0.058942941 -0.102169888 -
0.040774386 -0.039238425 -0.066772923 -0.108502881 -0.084027182
-0.087352664 -0.0611457 -0.11371045 -0.101677821 -0.104446813 -
0.104276762 -0.104364756 0.001193154-0.035006972 -0.094270187 -
0.121437626 -0.09359517 -0.10393491 -0.076342129 -0.089458511 -
0.084569422 -0.148335896 -0.114851549 -0.081176432 -0.063289385
-0.058472405 -0.103004646 -0.078826297 -0.007345094 -
0.142539793 -0.105843117 -0.015695206 -0.074454421 -0.117939192
-0.109170959 -0.096019309 -0.173668223 -0.079074479 -
0.011370317-0.123551311 -0.102975968 -0.085036026 -0.084156397 -
0.114313612-0.087986202 -0.018751519 -0.085169239 -0.041682225
0.004442521 -0.018959564 -0.070351598 -0.072536944 -
0.093821771 -0.108273645 -0.129270956 -0.05220724 -0.078910457 -
0.12036931 -0.081629286 -0.070889642 -0.128469357 -0.100540321 -
0.090187809 -0.100173372 -0.126915945 -0.102391684 -0.096120437
-0.084163987 -0.090413197 -0.094958793 -0.06695677 -0.044817594

-0.087551336 -0.130134958 -0.098302295 -0.137170683 -0.108447881 -0.087961012 -0.079197667 -0.105130538 -0.135441955 -0.062358393 -0.07722583 -0.115645821 -0.12292288 -0.058198556 -0.109632573 -0.035618238 -0.09354323 -0.087552454 -0.066097324 -0.086261356 -0.094848759 -0.044696238 -0.05632411 -0.06810707 -0.094575608 -0.08936174 -0.057298016 -0.152249036 -0.124630463 -0.085627886 -0.150933602 -0.127579062 -0.132543648 -0.052415478 -0.076802437 -0.114871736 -0.094421172 -0.082982879 -0.156040103 -0.121113995 -0.138529788

KEGG_NEUROACTIVE_LIGAND_RECEPTOR_INTERACTION -0.20414915 -0.191014611 -0.202515105 -0.221999392 -0.205328657 -0.193233389 -0.232175228 -0.193271128 -0.207162389 -0.208408134 -0.220715386 -0.228182827 -0.191399287 -0.219685361 -0.198960394 -0.234349247 -0.207400179 -0.2236332 -0.207334112 -0.20476188 -0.229758981 -0.215566527 -0.213840556 -0.187206209 -0.207390299 -0.226146798 -0.219108956 -0.226476846 -0.209442766 -0.209988617 -0.228077352 -0.214970128 -0.221619225 -0.203508228 -0.204434552 -0.228192779 -0.217603684 -0.228854622 -0.209033044 -0.228451992 -0.210567897 -0.227689063 -0.213975006 -0.198241252 -0.209805064 -0.212592269 -0.180708683 -0.202209627 -0.223198996 -0.220564128 -0.17207167 -0.209265344 -0.235774536 -0.21617909 -0.208487376 -0.211797331 -0.209362427 -0.200314553 -0.226091288 -0.201422123 -0.183888096 -0.205922258 -0.213510664 -0.200379219 -0.22007013 -0.228118212 -0.216163252 -0.211507806 -0.231829378 -0.214468075 -0.206227969 -0.236364845 -0.203494687 -0.204022733 -0.211639823 -0.182949895 -0.22537324 -0.222554445 -0.204670271 -0.206214891 -0.215534219 -0.20678752 -0.209779065 -0.217322874 -0.209301795 -0.225409812 -0.229221867 -0.206600887 -0.206327594 -0.216515537 -0.218115132 -0.202897207 -0.203243133 -0.224422496 -0.219570044 -0.2034968 -0.221381533 -0.217687884 -0.203002276 -0.187613613 -0.200688008 -0.200631723 -0.216790334 -0.213658698 -0.20658362 -0.211736794 -0.238917863 -0.219986122 -0.241416865 -0.222240074 -

0.218548475 -0.204326059 -0.222345807 -0.230266055 -0.195683214 -0.20439667 -0.224093979 -0.202093013 -0.200286531 -0.212677464 -0.219164398 -0.212670224 -0.196419445 -0.206237391 -0.224660465 -0.218447871 -0.221900734 -0.21828218 -0.204486007 -0.223017189 -0.215620673 -0.223898379 -0.225082841 -0.195280951 -0.208484652 -0.242711722 -0.200736558 -0.217944691 -0.218151628 -0.186550095 -0.215862908 -0.191865426 -0.212938644 -0.21313286 -0.224769063 -0.19173907 -0.211438187 -0.217826665 -0.246337416 -0.203743 -0.215960132 -0.22779316 -0.199999562 -0.205779027 -0.176449417 -0.198219919 -0.178949307 -0.206342248 -0.218318098 -0.195015031 -0.190856316 -0.221807846 -0.233341744 -0.212863987 -0.206649668 -0.22919772 -0.216721455 -0.215623478 -0.222651326 -0.213153608 -0.202648439 -0.2209792 -0.205872424 -0.218569736 -0.203347256 -0.208637354 -0.207737264 -0.213968715 -0.205280806 -0.19821905 -0.196618849 -0.195541817 -0.229607732 -0.201555958 -0.225687956 -0.195691223 -0.197805692 -0.180767627 -0.2133446 -0.228749184 -0.189217823 -0.205204643 -0.198332153 -0.20691686 -0.19240907 -0.180262909 -0.194320273 -0.200089946 -0.207610639 -0.213054243 -0.181960033 -0.203477564 -0.205918667 -0.197737848 -0.199341675 -0.235952397 -0.186300413 -0.197801776 -0.193315733 -0.215439353 -0.202304793 -0.213876467 -0.20927146 -0.198581659 -0.208463858 -0.203272646 -0.204013968 -0.179077722 -0.17561707 -0.217396717 -0.199400326 -0.203776175 -0.214533506 -0.216609581 -0.209669948 -0.214021403 -0.191099846 -0.215123662 -0.210958318 -0.183402128 -0.215243999 -0.197843211 -0.207240953 -0.208725914 -0.179771074 -0.209892592 -0.208276761 -0.199321419 -0.215810876 -0.207796744 -0.210324721 -0.206864962 -0.218538276 -0.216374901 -0.208060984 -0.191085152 -0.22765596 -0.217980742 -0.20836066 -0.216131211 -0.212321591 -0.201406944 -0.222299089 -0.21202406 -0.213818361 -0.199823228 -0.206439533 -0.200236602 -0.215138281 -0.205272844 -0.216834621 -0.221253431 -0.189225257 -0.22155966 -0.188810279 -0.201024556 -0.188591455

-0.208224327 -0.205001931 -0.229397581 -0.198535498 -
0.182118651-0.218296096 -0.209361086 -0.213634368 -0.213593661 -
0.194124393 -0.202462363 -0.204168852 -0.210667105 -0.209348783
-0.208447896 -0.193144025 -0.220212277 -0.222163182 -
0.209433222 -0.197398171 -0.203304582 -0.177946099 -0.202964877
-0.215610991 -0.198981688 -0.230144487 -0.229056726 -
0.219504228 -0.194884465 -0.229986345 -0.187979652 -0.213668661
-0.218834598 -0.216198962 -0.208550302 -0.194016304 -
0.209257158 -0.221156206 -0.194437256 -0.196265463 -0.217597832
-0.204073733 -0.21441613 -0.207951795 -0.21702372 -0.204516691 -
0.226772823 -0.216913994 -0.178240732 -0.201297934 -0.211021033
-0.206355639 -0.203909862 -0.206091102 -0.211375496 -
0.197993503 -0.189949417 -0.213637647 -0.223814603 -0.20146757 -
0.232418064 -0.220840754 -0.186695559 -0.215439122 -0.209676104
-0.212173577 -0.218427598 -0.198102756 -0.19382705 -0.197463677
-0.186155923 -0.231060997 -0.238355966 -0.198497629 -
0.197317711-0.189501579 -0.175463057 -0.222915639 -0.231221731 -
0.209040348 -0.204735785 -0.211378659 -0.221841498 -0.239951189
-0.229612927
KEGG_NEUROTROPHIN_SIGNALING_PATHWAY 0.2056955 0.22060486
0.219175646 0.190430225 0.199166081 0.194903392
0.196869585 0.213705376 0.209057313 0.214129255
0.189595295 0.216809654 0.202978346 0.2011025290.2115818
0.2111776290.19610678 0.207600473 0.203521002 0.209841559
0.218856728 0.226570607 0.198231989 0.2054881110.229894121
0.207512635 0.205186727 0.2114633520.213000834 0.224450366
0.191478383 0.20048084 0.20659526 0.197954243 0.21813672
0.190577618 0.203363466 0.1761118340.194803672 0.187074973
0.220516177 0.212367409 0.193240464 0.190599393
0.213166426 0.201853288 0.208087402 0.184681652
0.212317434 0.190532085 0.215086765 0.194617337
0.209416289 0.202895745 0.198865095 0.221900468
0.2101169150.209382768 0.178996755 0.203880441 0.20041214

0.210070881 0.209973688 0.213132387 0.21533271 0.181618505
0.175209298 0.210532739 0.204916691 0.203241537
0.222480819 0.187945312 0.205869466 0.193206429
0.206300181 0.232697225 0.2110792440.215255776 0.217241068
0.205171514 0.200864442 0.187677376 0.209934563
0.205947691 0.207641621 0.203848237 0.215562879
0.2112146870.205173591 0.209996347 0.191853678 0.187952301
0.1951108020.199483527 0.223755553 0.209128524 0.199663602
0.202849818 0.214491991 0.210283624 0.2283968 0.214919601
0.1911743430.220098378 0.203829846 0.208182182 0.190395878
0.206705367 0.2101193170.206700321 0.186894166 0.215326383
0.194121761 0.207065788 0.199687287 0.216277054
0.200355901 0.176219516 0.209275001 0.212786224
0.2119321390.208707722 0.2050711070.2211438740.220796629
0.17927681 0.205904342 0.192628992 0.199143999 0.22092801
0.206423584 0.199521321 0.19228511 0.216276212 0.220352826
0.226157804 0.218932788 0.222599878 0.221313825
0.225995805 0.199818798 0.243281457 0.206318597
0.2058113260.205655249 0.22768271 0.221352805 0.199583963
0.206105086 0.21174344 0.217987295 0.209138171 0.232985451
0.18574556 0.215392456 0.222023701 0.225874351 0.22979968
0.220560197 0.240130285 0.216319781 0.229194668
0.205958186 0.219373771 0.205669279 0.201076846 0.2141949
0.220769537 0.203542023 0.193582057 0.217335054
0.209078157 0.188836152 0.197678955 0.22418438 0.226533533
0.210241923 0.216444321 0.189506457 0.219554133 0.21509525
0.197156351 0.2117134110.19901676 0.214018243 0.194237245
0.203867539 0.224014536 0.197940006 0.2196311170.219894658
0.228942043 0.233279657 0.225287348 0.223931724
0.222004212 0.209073276 0.217923934 0.237889536
0.234300389 0.237109545 0.1985941130.207265237 0.207802743
0.2198155 0.193487665 0.215262094 0.209439151 0.225132597
0.203584201 0.214968526 0.203232129 0.228971958

0.204562743	0.219218646	0.232896918	0.216880649
0.223437195	0.237462607	0.228006125	0.228208643
0.225638991	0.210267961	0.208848683	0.236217322
0.202260032	0.225742174	0.209109966	0.181207846	0.24109847
0.210324981	0.233095043	0.221061712	0.202121621	0.22471191
0.221769638	0.205945895	0.2211963560.228526476	0.208677036
0.202572096	0.215904483	0.197943742	0.184807234
0.218759151	0.228065395	0.172624544	0.206686001
0.194299525	0.204787776	0.227455791	0.226639027
0.221517689	0.201014278	0.199634636	0.209655522
0.190890365	0.197558617	0.192679102	0.212934536
0.192819185	0.208828028	0.184473853	0.205507999
0.209796064	0.2113980230.193622642	0.203624209	0.199953719
0.200622685	0.2117354030.221577928	0.234847143	0.182434168
0.2009673110.197525819	0.214938298	0.215365194	0.203074885
0.210678025	0.194798326	0.212553212	0.198645459
0.213972225	0.208052135	0.210283543	0.225020092
0.231343342	0.229970549	0.234165606	0.231386203
0.2359536110.199180472	0.206368849	0.201653742	0.223841113
0.2115511610.216654894	0.229260859	0.208347146	0.198883443
0.204497706	0.227815501	0.202773315	0.224165107
0.227506556	0.229080539	0.207120546	0.208969332	0.21879134
0.223800398	0.210892381	0.213613908	0.216563381	0.21248581
0.242171845	0.205763676	0.186743717	0.207346025
0.208672203	0.212576891	0.200624378	0.212313026
0.222895927	0.20265137 0.209665952	0.217120359	0.195244935
0.20567925 0.2115906750.241907963	0.212684295	0.216879001
0.208583081	0.237157966	0.23246962 0.207434417	0.214810219
0.2111921880.20168092 0.228904783	0.210406967	0.205278668
0.232844357	0.226328653	0.208639343	0.208029504
0.225892007	0.228748988	0.1974931120.205079868	0.204358036
KEGG_NICOTINATE_AND_NICOTINAMIDE_METABOLISM	0.065036937
0.093247842	0.09625571 0.07671522 0.091004536	0.049610138

0.034850401 0.086962144 0.132782641 0.081459889
0.005632946 0.106724528 0.020063384 0.039668628
0.1146988480.131614051 0.058483899 0.097022161 0.104543064
0.029348039 0.015087961 0.07319356 0.145199177 0.06080234
0.073074106 0.1131722580.090459159 0.123721863 0.095196133
0.1158887690.07428655 0.171951447 0.067944716 0.06187145
0.062565267 0.121619283 0.02794239 0.057676746 0.129736186
0.03530553 0.0674711420.148318877 0.07421552 0.11053702
0.120466985 0.051749029 0.069228141 0.027826221
0.134429079 0.12013177 0.148060646 0.090651926 0.133721702
0.003271006 0.10977398 0.134235127 0.078123757 0.121922239
0.107761533 0.02617625 0.122716668 0.1111219650.097695137
0.1271185690.102289415 0.083343446 0.092041644 0.110181771
0.097276414 0.091264887 0.1146733140.032301675 0.096744702
0.053688552 0.098370139 0.097581965 0.070717813 0.11999998
0.109034488 0.048039074 0.090149947 0.097164501
0.107830385 0.1112219420.130160786 0.069494945 0.121999169
0.083885327 0.124656853 0.081254474 0.10149005 0.174347645
0.1129988350.075567962 0.093443852 0.1168613040.093668158
0.046778247 0.1530119670.107419952 0.1193574260.13423751
0.096860075 0.152350471 0.084677812 0.103240752
0.088205286 0.104669581 0.097940759 0.089536647
0.075160631 0.073026443 0.050740133 0.1137896080.081346536
0.1115090360.1071105140.100134481 0.123735369 0.04044658
0.07956848 0.0299111990.084833871 0.086169664 0.133081587
0.09676478 0.056067445 0.0684911440.09747712 0.125095146
0.09132445 0.097342486 0.09067031 0.105039654 0.090449503
0.043506095 0.096445512 0.10469776 0.081462189 0.09089508
0.056335628 0.1178556620.124296698 0.086152402 0.099510697
0.101941746 0.100103924 0.076106582 0.04259979 0.050255466
0.097653177 0.09956419 0.1137572510.040997807 0.129526377
0.0973119920.0941134680.1125510730.1311631960.084565398
0.093785554 0.037408622 0.1163811420.078669142 0.136709688

0.081091738 0.045229266 0.123387107 0.107716188

0.135863457 0.089366717 0.097423467 0.080314134

0.1121548350.1153542320.11976709 0.102531753 0.096846845

0.128232431 0.079850264 0.1113917350.065428978 0.103685253

0.1095929110.1311696950.077983066 0.076351027 0.108684155

0.053513066 0.086081268 0.087037093 0.0668114770.115338009

0.1116909390.121045471 0.105734192 0.057129514 0.103121162

0.134817322 0.08304138 0.109862059 0.090679063 0.146884424

0.06721153 0.143886212 0.080178605 0.039607599 0.056999517

0.126554867 0.089168128 0.091996123 0.060990278

0.121291584 0.026802003 0.126687233 0.1156104970.070390422

0.121684843 0.095131675 0.062717826 0.092669052

0.085965738 0.089446723 0.075272307 0.081047055 0.03118728

0.103377972 0.052738921 0.043175775 0.100299097

0.133546537 0.095133784 0.08884858 0.108512894 0.100410973

0.1165725750.109696737 0.13055097 0.1107501210.100943974

0.1190945260.130364769 0.087567139 0.1561777110.135863332

0.1049271160.1412119590.098420384 0.143284264 0.086735412

0.128335597 0.100975625 0.101329201 0.098364124

0.103597293 0.079721836 0.106559904 0.140436418

0.129344916 0.1118740840.041630705 0.126428878 0.151453697

0.096549807 0.128068808 0.068779314 0.107380491

0.103491641 0.0622991160.077385299 0.147167651 0.087312586

0.074347002 0.106840233 0.138766477 0.057264182

0.1125380640.150895055 0.081637455 0.120369583 0.061154697

0.141592877 0.140002504 0.130005359 0.127517881 0.10896925

0.1138601680.1105839240.148086105 0.1164279860.106343947

0.096724351 0.076025631 0.076766137 0.135658183 0.09694665

0.079223498 0.1122039630.083536442 0.109710616 0.084977987

0.12423295 0.096966708 0.047172951 0.045971435 0.032029412

0.134076287 0.123817253 0.097298048 0.057383152

0.104571316 0.09093001 0.03622446 0.067950106 0.023283811

0.1189688740.1101252240.151056977 0.101255234 0.11957532

0.108959982 0.130097431 0.043606895 0.028748777
0.140357402 0.086807741 0.1112743810.153605285 0.096761107
0.104457046 0.075367855 0.093251632 0.08835463 0.145972189
0.086691204 0.0650969110.1153883130.1183723140.03365451
0.1117313590.130699863 0.003985978 0.1119075640.023017755
0.089465789 0.1127431320.164388192 0.046307098 0.089553457
0.035129404 0.1121614830.031420518
KEGG_NITROGEN_METABOLISM -0.062390224 -0.110406147 -
0.035997656 -0.068999199 -0.09139448 -0.053522677 0.023228303 -
0.018446754 -0.064524023 -0.062209404 -0.048756315 -0.081920023
-0.060389364 -0.000760385 -0.040363366 -0.043431213 -
0.025960162 -0.002620525 -0.012806678 -0.01452714 -0.050481498 -
0.049240474 -0.022949117 -0.054659397 -0.043207705 -0.00806436 -
0.051793938 -0.02868967 0.011967653-0.084149896 -0.043145907 -
0.054932785 -0.03383945 -0.031516585 -0.041048117 -0.008292354 -
0.063038847 -0.054166519 -0.050165068 -0.038974845 -0.02595554 -
0.007045767 -0.026021469 -0.010681753 -0.018006152 -0.056849137
-0.018640932 -0.019163821 -0.013854392 0.014770512 -
0.011557239-0.065296283 -0.057849527 -0.072880887 -0.028149786 -
0.028353441 -0.052160519 -0.060197719 -0.031989375 -0.061482321
0.016118338-0.069585492 -0.041299259 -0.03832962 0.028951032 -
0.054094258 -0.034668178 -0.06454522 -0.013279267 -0.029649708 -
0.0210011860.007250168 -0.068926864 -0.039716628 -0.05167843 -
0.054268082 -0.034864996 -0.060356277 -0.04137517 -0.055329128 -
0.017325884 -0.027747849 -0.049620794 -0.00114549 -0.071730347 -
0.023376943 -0.030031462 -0.112122187 -0.069964682 -0.050545389
-0.010199004 -0.041188783 -0.03137461 0.008546772 -0.013076038
-0.022700648 -0.049339658 -0.05490212 0.016843467 -0.033649786
-0.04362923 -0.014087601 -0.042275821 -0.082408386 -0.060161498
-0.102692992 -0.02825766 -0.001945067 0.022572121 -0.033537402
0.025198741 -0.08542447 -0.040612037 -0.044453246 -0.02438133
0.000376745 -0.04844246 -0.063015452 -0.084096323 -0.062382129
-0.095054834 0.036810726 -0.07437934 -0.070209332 -0.093936718

-0.067206312 -0.034971098 -0.044998191 -0.020515738 -
0.048703271 -0.010278807 -0.025206451 -0.074611557 -0.047571582
-0.03749023 -0.033207314 -0.065576578 -0.072873002 -0.065699249
-0.036297345 -0.053026591 -0.089651314 -0.009465037
0.013433224 -0.012105888 -0.05871371 -0.04868185 -0.072221753 -
0.066680049 0.011889694-0.05711065 -0.020491427 -0.035004315 -
0.011355196-0.012950501 0.018983223 -0.068971174 0.006784718 -
0.048804154 -0.090350664 -0.008317074 -0.076131472 0.001145725-
0.01769973 -0.048372143 -0.05847709 -0.041686454 0.022520031 -
0.070497853 0.006275854 -0.001796219 -0.088056627 -0.050141091
-0.02364555 -0.05252218 -0.062504051 -0.02243111 -0.06604589 -
0.015200367 -0.0682177 -0.081974474 -0.016798097 -0.000982687 -
0.066070822 -0.004724587 -0.076714766 0.00028647 -0.058369616 -
0.053340498 -0.002241444 -0.043353889 -0.067155728 0.004562463
-0.020278619 -0.072627241 -0.014481012 -0.036071255 -
0.054047915 -0.06689073 0.004549755 -0.064859639 -0.053104398 -
0.007279676 -0.045588915 -0.100813592 -0.034210104 -0.056397582
-0.066365737 -0.032113906 -0.025772503 -0.048812757 -
0.100717754 -0.053877504 -0.062374806 -0.080932704 -0.020992929
-0.043046285 -0.075273528 -0.066049081 -0.080796699 -
0.086031529 -0.000486262 -0.037306056 -0.054900419 -0.072611537
-0.063191047 -0.092259318 -0.061724561 -0.012178829 -0.0455603
-0.049180159 -0.059282226 -0.040879447 -0.019748083 -
0.046121043 -0.018946082 -0.035822247 -0.033827497 -0.042152516
-0.091414526 0.025303028 -0.014019324 -0.002672375 -
0.041670364 -0.042915234 -0.067609711 -0.018333807 -0.017968789
-0.045545797 -0.001511389 -0.062196477 -0.093375342 -
0.057825892 -0.01265649 -0.082184258 -0.040607644 -0.019014467 -
0.059796635 -0.025199978 -0.02581829 -0.03991731 -0.083362584 -
0.001359614 -0.061476508 0.006667278 -0.053757031 -0.046146177
-0.014385324 -0.045857474 -0.055245349 -0.04963428 -0.091119355
-0.03757378 -0.011464724 -0.026233769 0.017351647 -0.05958134 -
0.013581687 -0.076609224 -0.024711193 -0.015846248 -0.03289681 -

0.005657106 -0.048801602 -0.035394855 -0.02214676 -0.050579207 -
0.024650824 -0.043174254 -0.074402337 -0.065605167 -0.091856173
-0.051562022 -0.048955938 -0.019208157 -0.076385014 -
0.008038596 -0.034301467 -0.040611043 -0.068413052 0.022132817
-0.083604582 -0.0808821 -0.095070805 -0.065038789 -0.064196079
-0.049256312 -0.008172445 -0.047438237 -0.05323561 -0.070169406
-0.065564028 -0.069475412 -0.007019186 -0.053906792 -
0.05723929 -0.046424249 -0.024634885 -0.035920156 -0.030550958 -
0.07670829 -0.041918319 -0.065522286 -0.077286518 -0.012851778 -
0.033611274-0.049070744 -0.060418805 -0.039420399 -0.04537531 -
0.032964637 -0.027792861 -0.04704125 -0.05525643 -0.094307471 -
0.016711819-0.047415115 -0.005260798 -0.087335375 0.065961147-
0.041229359 -0.067160567 -0.031083821 -0.071459852 -0.047112053
-0.035538045 0.009808782 -0.077895638 -0.046908125 -
0.008012724 -0.02310346 0.008620811
KEGG_NOD_LIKE_RECEPTOR_SIGNALING_PATHWAY 0.057790967
0.088512962 0.170930009 0.076801708 0.182151651
0.014389074 0.041854583 0.182414765 0.077782797
0.145785457 0.016949692 0.173455684 -0.014466562
0.039990277 0.187980931 0.126255487 0.05868195 0.082199085
0.09752433 0.049340342 -0.019191021 0.126940406 0.155427232
0.020461819 0.194581838 0.137456346 0.109289268 0.08071707
0.059093388 0.1147117480.06118571 0.145269819 0.045227378
0.036641924 0.127050376 0.14845688 0.055995369 0.079298734
0.166574242 0.030703242 0.098696382 0.109419693 0.15277559
0.077187843 0.075946899 0.0823112870.098188466 0.088508501
0.187783601 0.083348933 0.145058779 0.125392624
0.149235492 -0.004612045 0.131316215 0.164784012
0.1639931130.130645874 0.16451861 0.0114476520.067940262
0.096084268 0.134307924 0.200789675 0.074728181
0.048563404 0.1011883380.154689436 0.178000319 0.115091913
0.098538766 0.054434927 0.084392493 0.025199916
0.098454742 0.189845583 0.086856954 0.179833987

0.096196064 0.1134422490.170702442 0.087375198 0.049956516
0.100839149 0.162259999 0.155710688 0.19162438 0.113168671
0.06748112 0.059851999 0.148719588 0.165121396 0.13236042
0.087730302 0.1427119250.1174866690.109522666 0.123556005
0.1611336410.19289486 0.092705594 0.0382671180.070947741
0.109927926 0.077562769 0.1164625170.057341488 0.196229023
0.19491788 0.176476647 0.086854414 0.1372041140.01588359
0.070749957 0.044537262 0.20675007 0.168476208 0.108677302
0.160124209 0.085446623 0.099751474 0.105401873
0.095979347 0.091028099 0.126053464 0.072100055
0.140918737 0.08682147 0.07409179 0.1140901540.093777055
0.088329514 0.097945783 0.158165164 0.127365108 0.03168164
0.13142499 0.12017056 0.103445223 0.105101205 0.08065905
0.107468258 0.1911479680.038152604 0.090559673 0.164159211
0.170875481 0.1113623210.052156184 0.149168613 0.132393596
0.122572341 0.06329913 0.042120167 0.1184085130.11150002
0.1131428690.188773648 0.097424269 0.138595515 0.093373014
0.09203839 0.067567177 0.161997854 0.196121044 0.100259412
0.022472712 0.1158274420.106706821 0.087672845 0.126980958
0.173288456 0.013144518 0.167186637 0.201414915
0.167795127 0.123736805 0.155015328 0.175491206
0.068500561 0.167100476 0.025973315 0.148265417
0.140276012 0.174597343 0.022473091 0.083552795 0.14700505
0.0238904 0.125761736 0.146687383 0.1242924 0.194111841
0.157268332 0.162175346 0.060960402 0.18855623 0.127446969
0.158533636 0.190047364 0.186082957 0.058696427
0.104247408 0.09961943 0.1211085380.001730535 0.082360127
0.092767181 0.091858345 0.057652981 0.1184899610.102218408
0.131322231 0.046358468 0.120296096 0.1166911760.060852587
0.204754408 0.188529902 0.107474261 0.083468967
0.192054232 0.095296553 0.099705527 0.129820277 0.01698412
0.087347465 0.090060581 0.073683951 0.11979386 0.157280739
0.10868627 0.135052955 0.1384118040.155437714 0.140997646

0.031818039 0.163066974 0.1091179380.100795544 0.15952358
0.166191057 0.044142784 0.1473111420.127612862 0.207741502
0.088400841 0.057862179 0.147636723 0.078774107
0.109237582 0.195665674 0.15431231 0.1120302690.07880931
0.086840926 0.162453927 0.126794885 0.1414911340.124661425
0.0011598890.093971866 0.161642887 0.080464842 0.147773526
0.109483701 0.137133664 0.148344752 0.022342895
0.080010877 0.134835353 0.13403156 0.0848651160.159887053
0.129970861 0.00576357 0.1443811670.184796126 0.04469491
0.083318678 0.1185251790.1113638510.09111351 0.119928019
0.120597633 0.1123594990.159623814 0.179596784 0.239628082
0.195608798 0.14653355 0.094531286 0.086430592 0.026074419
0.190737984 0.169436539 0.064963812 0.165468878 0.12600366
0.073295177 0.109138514 0.074754197 0.164134056
0.066405303 0.060339354 0.1178736580.076565871 0.11634478
0.092974996 0.123006788 0.1137410320.119447 0.132229976
0.15314627 0.069707329 0.085888314 0.088661231 0.087762982
0.104964875 0.073648413 0.181005844 0.124739759
0.077510388 0.036096627 0.208460942 0.1180654260.133282944
0.166420418 0.096221707 0.196560805 0.084335171
0.156910993 0.17925197 0.184171815 0.179666296 0.123609867
0.143835235 0.200497389 0.021403962 0.075170478
0.2002911060.033524784 0.069385836 0.0346831190.189821167
0.140148861 0.085300542 0.1178350050.1511660910.008600998
0.077458036 0.038348836

KEGG_NON_HOMOLOGOUS_END_JOINING 0.163489737 0.133896252
0.176656907 0.148822499 0.1832117540.176564044 0.201341413
0.172126968 0.188705856 0.173663384 0.194796928
0.182265765 0.182674835 0.195607914 0.203600467 0.17300356
0.192971461 0.1193959930.186717683 0.187943385 0.202491237
0.106535777 0.163159573 0.163580495 0.219593548
0.179826534 0.182055437 0.217121659 0.2146465110.148180554
0.21307097 0.172765484 0.203129028 0.172071712 0.185411534

0.147603512 0.177935267 0.182875034 0.156243047
0.206324274 0.150846572 0.157003793 0.17018492 0.166300997
0.173574852 0.177348242 0.155879947 0.180288648
0.161289984 0.160770766 0.141934692 0.174274504
0.186205863 0.184465158 0.186351814 0.1835193 0.207641679
0.180791951 0.2011258870.193804544 0.147729715 0.153694501
0.18462196 0.154086006 0.174065822 0.206285309 0.171584732
0.140786577 0.198472959 0.196961364 0.162716437
0.205423258 0.184790884 0.194849009 0.186741365
0.170324332 0.202241008 0.194101392 0.155520642
0.212193519 0.200296015 0.20911316 0.166643373 0.174997733
0.170862381 0.181062218 0.191551207 0.169572309
0.182271563 0.156804058 0.187746197 0.167879861
0.161729715 0.177196348 0.167370639 0.206874937
0.2007119310.2211286790.146107674 0.155635097 0.147578068
0.1987531190.188222572 0.168646576 0.196788044 0.155047106
0.201029534 0.169134527 0.191809715 0.193047303
0.185982583 0.180657782 0.200419545 0.198672487
0.157737059 0.169704351 0.195415552 0.174318842
0.210145006 0.177520055 0.165278574 0.179882703
0.147925867 0.159393643 0.20151114 0.178424902 0.179148017
0.1911397550.165405565 0.14223112 0.208914633 0.199485992
0.170844753 0.186686158 0.19668675 0.165680895 0.214125801
0.167674923 0.163145473 0.151456265 0.224534774
0.166477449 0.195074349 0.162706055 0.223555561
0.165761389 0.154403079 0.197035541 0.178594801 0.17273212
0.191584622 0.214939435 0.195462326 0.195740901
0.148227139 0.178707454 0.15498312 0.213701239 0.195025908
0.147966049 0.178309941 0.164782172 0.203680558
0.174601208 0.177194385 0.19630056 0.174361709 0.196910643
0.18902862 0.169181601 0.177655558 0.182720678 0.150109686
0.194408104 0.216272371 0.160104352 0.144806782
0.163654281 0.192516685 0.168587691 0.188646356 0.15803197

0.20936846 0.147499947 0.174498706 0.205273038 0.195990178

0.170321243 0.171690055 0.158403103 0.1527591150.19802947

0.167759993 0.199502106 0.156451334 0.169079009

0.190933348 0.209527937 0.155031297 0.158846021

0.197534766 0.179363085 0.167926476 0.198675158

0.161431362 0.152526433 0.21305932 0.174824677 0.164775095

0.167586257 0.191331044 0.191538395 0.181925941

0.167614171 0.192441248 0.17562315 0.175650123 0.186978007

0.142889132 0.188664385 0.1821811140.149407688 0.175394322

0.1878091160.144289364 0.187835214 0.160439927 0.180679273

0.204105873 0.125880938 0.189669439 0.154147092

0.200653583 0.181087817 0.146032731 0.1916291190.141100739

0.179810813 0.172928875 0.16726886 0.181842338 0.147082648

0.189260637 0.177862917 0.142229564 0.196913459

0.148675034 0.197736514 0.1639119050.185699808 0.17937054

0.160058547 0.12509812 0.161830052 0.171494241 0.165100832

0.161333952 0.149562554 0.1747277110.163678992 0.203747815

0.1806111690.149526169 0.155514023 0.157489179 0.160323855

0.153509772 0.206914386 0.190473061 0.214524954

0.171805088 0.176296544 0.2091129180.215296478 0.181689051

0.188405176 0.169078163 0.1602150110.162058591 0.173895718

0.152569561 0.148997006 0.160367972 0.202202171

0.214653467 0.185941092 0.1903934 0.178664675 0.165665412

0.155042851 0.167036752 0.179962245 0.201048819

0.220270157 0.175952994 0.17935724 0.152164298 0.176062849

0.177124177 0.195263703 0.185274374 0.19463342 0.166924652

0.175771553 0.190704782 0.152250086 0.13270359 0.203013777

0.212026092 0.128952636 0.166895059 0.176091218

0.201221623 0.214984995 0.222857698 0.1188649170.14276528

0.192371027 0.144195251 0.143141353 0.165022331 0.1562521

0.204472843 0.144622122 0.161326292 0.191991363

0.163293809 0.169258281 0.1996117290.175801898 0.201363831

0.165841305 0.212838484 0.195672151 0.18805744 0.165459122

0.159603794 0.156776205 0.15136955 0.220697398 0.142464018 0.21856575 0.177632821 0.176229171 0.185946426 0.230745711 0.200695651 0.17945829 0.165636958 0.214571801 0.22299796 0.204604903

KEGG_NON_SMALL_CELL_LUNG_CANCER 0.17235066 0.20701014 0.187554155 0.175930555 0.185281618 0.215349182 0.207628525 0.179782134 0.180203787 0.204838815 0.203685258 0.196302098 0.191929493 0.194722829 0.209431479 0.192900291 0.192154274 0.196474889 0.172917212 0.210022334 0.224051525 0.228263282 0.197809672 0.196872069 0.239499555 0.2110671340.193206076 0.203775232 0.240042305 0.2324211380.177045606 0.207148403 0.223460819 0.190827896 0.208425031 0.173605862 0.187833556 0.169357391 0.17207137 0.190198575 0.202865842 0.182988256 0.190515434 0.17344321 0.184240984 0.202982643 0.203895375 0.191037934 0.207327549 0.17691647 0.192218946 0.204773801 0.191456899 0.2115380290.197936481 0.20672156 0.200035321 0.202226837 0.1566114120.208805634 0.194210829 0.19915241 0.187309207 0.201997543 0.194526291 0.182682222 0.174627421 0.192859363 0.190492982 0.2003881120.215841692 0.195212862 0.193187542 0.192340669 0.198297095 0.231884424 0.178701374 0.1924673110.17761293 0.19399972 0.177122199 0.173920741 0.21278938 0.185407161 0.207634785 0.189779089 0.2239131160.203165784 0.190581987 0.201700851 0.194343922 0.178913883 0.166240496 0.174700866 0.212707497 0.189920252 0.195339147 0.181480552 0.203292002 0.206740456 0.201914862 0.23610291 0.180726758 0.202081938 0.185140158 0.2011513220.198607142 0.195119191 0.19296937 0.202090525 0.178821775 0.220946136 0.198853602 0.214737683 0.187712093 0.200998209 0.189479641 0.159142874 0.215486882 0.223092404 0.201688687 0.196005541 0.20900374 0.2138841 0.212560455 0.176955606 0.21304153 0.207203947 0.18144669 0.208795128 0.204316408

0.187605309 0.184325498 0.213223167 0.215613368

0.202335414 0.2119883210.230648516 0.22477361 0.205728939

0.19762764 0.216978754 0.190969144 0.224838249 0.217307624

0.216956533 0.2053241140.18297478 0.215532538 0.207789231

0.219370101 0.213997135 0.251490125 0.194604755

0.193901748 0.205919445 0.220819181 0.219862741

0.209298708 0.242181239 0.199404481 0.222401649

0.198008798 0.220105622 0.178151067 0.164937578

0.203629739 0.224043321 0.208293983 0.196522691

0.212600316 0.20044373 0.182590126 0.207094772 0.199744879

0.213457768 0.206142781 0.20602661 0.183668439 0.220352041

0.209829215 0.199410631 0.218989504 0.183874927

0.235727898 0.181330442 0.219108052 0.2125011460.20479751

0.208054232 0.219571547 0.235575604 0.218269841

0.215487174 0.1951841130.238451484 0.209274194 0.224559221

0.215310196 0.22893248 0.217625191 0.201578924 0.207119964

0.209556549 0.195863779 0.203536528 0.1876483110.20799909

0.213799493 0.222441618 0.217809601 0.196408009 0.20517415

0.221424328 0.210570263 0.209229508 0.208834176

0.214075184 0.230713852 0.226433196 0.224372927

0.2111471640.212070018 0.207533342 0.208644612 0.199597598

0.207451324 0.181325276 0.190567201 0.2068813110.213087487

0.2116512290.21535501 0.191483486 0.227390895 0.219524181

0.217328137 0.218604882 0.218513776 0.21637009 0.192214697

0.187091065 0.180274467 0.1811487170.196718153 0.229827958

0.154886592 0.193667253 0.171041366 0.196940267

0.209877885 0.2263112390.2110092640.18421215 0.16836475

0.204457587 0.168509564 0.1786110450.175786054 0.216183164

0.210296864 0.198609829 0.175002707 0.213556056 0.19245387

0.2117819440.180728769 0.201647289 0.185242032 0.198739902

0.204351658 0.203570025 0.222652003 0.184818242

0.192045069 0.20686765 0.189513314 0.18238966 0.190715078

0.2000737110.18978532 0.214085533 0.175818136 0.212596462

0.209973676 0.217161228 0.213461529 0.243449523
0.226648289 0.220423739 0.2201152160.226195872 0.19377055
0.191577918 0.194412817 0.214545804 0.1931156190.213912248
0.229132656 0.21276282 0.17588111 0.194153405 0.225987238
0.204475564 0.228530493 0.226501833 0.239938904
0.195620234 0.198417108 0.206700241 0.207316919
0.198748667 0.201324509 0.17988102 0.227591359 0.199556483
0.218777006 0.182494534 0.189632969 0.206803166
0.219306724 0.189147002 0.223153972 0.226028939
0.189647436 0.201356314 0.213197893 0.184759065
0.222283275 0.214042708 0.2300115370.212734219 0.217262351
0.199215464 0.2340114180.2243711210.199336325 0.211989563
0.208258069 0.208906769 0.206623086 0.220587635
0.195369386 0.2311413640.24612437 0.218594099 0.229842834
0.210510789 0.225484593 0.214366256 0.196029399
0.179651219

KEGG_NOTCH_SIGNALING_PATHWAY 0.2051149690.25142178
0.197083933 0.183940378 0.189007524 0.195197581
0.197547673 0.190142103 0.199156609 0.221685887
0.216971977 0.192734878 0.240162468 0.190673444
0.212566162 0.181608931 0.205272856 0.165529559
0.183214239 0.196438939 0.250984383 0.214127326
0.188937704 0.262526823 0.204153142 0.168171013
0.199527603 0.187959052 0.201803994 0.228583624
0.167844891 0.185457636 0.22773687 0.187735201 0.207282388
0.172803218 0.220878038 0.20300658 0.1804021110.182336773
0.227189518 0.189443008 0.194607843 0.17053565 0.172435748
0.20503013 0.220678696 0.207825463 0.173188759 0.150590082
0.220241643 0.178075931 0.196324401 0.206907301
0.195713191 0.2116331910.1941147330.194259354 0.163262678
0.22439256 0.1908752 0.183076416 0.173957097 0.190158942
0.232764067 0.201756403 0.162357251 0.175495913
0.1813491110.200598762 0.202585909 0.181357269 0.217838372

0.199531722 0.2113466850.231946362 0.205855061 0.210159027
0.214055056 0.206075309 0.16334712 0.186169001 0.192667964
0.1811098910.172450931 0.1906956 0.1923663 0.203992481
0.200602039 0.202397099 0.19021328 0.144378563 0.175812129
0.169078368 0.208398285 0.200864103 0.168449591 0.208879
0.179482298 0.175141786 0.194157351 0.184031565
0.182966124 0.212590938 0.221409443 0.234984162
0.2011410030.19995355 0.177143488 0.210952195 0.17301927
0.196350282 0.221960436 0.20064296 0.187247681 0.201825504
0.17782405 0.157031528 0.204096223 0.212425701 0.212816361
0.204970901 0.2071511020.223374775 0.2014915110.15933441
0.192781852 0.199637163 0.193733488 0.208212242
0.214899969 0.205753762 0.189462948 0.196312589 0.21005228
0.214882741 0.2126112350.195886872 0.24010407 0.252834032
0.2141123 0.233278423 0.175772864 0.209951256 0.20415255
0.2118960180.201841836 0.160459791 0.20921936 0.195346288
0.221451351 0.187290474 0.226475262 0.2078118360.218882503
0.233332602 0.233364063 0.176248275 0.195894124
0.228314326 0.210384109 0.217433904 0.165947995
0.207266387 0.196020201 0.1911080580.224459272 0.181347385
0.200247259 0.193943841 0.195865576 0.181673489
0.215470335 0.192593495 0.185983397 0.205933967
0.186956044 0.1941141820.172158162 0.2078110220.21696291
0.250810929 0.196141787 0.205295198 0.196240961 0.2284943
0.181267519 0.228835247 0.224647806 0.184185186 0.20769289
0.2259115060.2299005110.218915166 0.205170967 0.25323839
0.20034466 0.210237478 0.215329199 0.226443058 0.226028051
0.2112181910.206976607 0.212875053 0.206791701 0.200595124
0.213504107 0.225849868 0.21075328 0.198968567 0.216046656
0.21846998 0.214576103 0.23160021 0.183061051 0.216752513
0.21343191 0.217934634 0.229426243 0.2090411520.233985006
0.187245694 0.21374803 0.228383965 0.226372233 0.216519415
0.194648801 0.205012768 0.180001854 0.238673793 0.19131499

0.202838784 0.1842118790.184851508 0.20939526 0.213443891
0.210887813 0.210283685 0.197614461 0.21446808 0.172955338
0.174196433 0.202676395 0.17672116 0.195622317 0.224343177
0.145845966 0.178835916 0.132658721 0.200508783
0.199127356 0.207068982 0.195248975 0.1486011550.170341945
0.219635747 0.16484493 0.159908486 0.172423858 0.1984484
0.196549407 0.205551099 0.158873951 0.201601639
0.1599711410.222218859 0.183573918 0.176015535 0.221358734
0.215421827 0.171226019 0.213814215 0.241946186
0.175459489 0.197231577 0.197326458 0.2031137270.166998442
0.218098266 0.210672417 0.187381646 0.180642752
0.179018841 0.204829057 0.205355418 0.217823827
0.219804299 0.198303508 0.21783423 0.223898559 0.210447021
0.212174578 0.198728536 0.219236932 0.161857276
0.232443234 0.203038127 0.188168736 0.209446802
0.190783227 0.185587392 0.200494657 0.215455366
0.229198402 0.207341808 0.223831671 0.2150172110.167053063
0.2113709190.208008603 0.20264811 0.20090608 0.214017791
0.203925342 0.231563725 0.251872189 0.179258397
0.148629679 0.193949101 0.1978779 0.201876616 0.163097239
0.225403907 0.230268288 0.153454946 0.223936478
0.190465363 0.1441386 0.194577543 0.197081594 0.208564904
0.210959037 0.180376319 0.202449529 0.228015564
0.216037013 0.195573748 0.178899719 0.231580607
0.189896307 0.225402545 0.276101789 0.216959309
0.245736269 0.223959424 0.207172837 0.1811581240.21821607
0.235100581 0.2225118270.200891887 0.196675216
KEGG_NUCLEOTIDE_EXCISION_REPAIR 0.189592801 0.140104718
0.175025312 0.173779852 0.175784204 0.188216177
0.218702505 0.176773767 0.178962078 0.1810961160.205572385
0.195180009 0.198514216 0.188439596 0.195657303
0.2021111780.182265889 0.161339506 0.191343644 0.204891785
0.223633272 0.178357029 0.198162315 0.197872704

0.198272785 0.209180136 0.223693695 0.185692866 0.2100805
0.16482765 0.242478468 0.208047952 0.207214565 0.181410629
0.180033569 0.20441517 0.20373761 0.172738314 0.201015417
0.213467133 0.17181889 0.188225528 0.1904118730.188885654
0.171514957 0.194785055 0.187855051 0.15694697 0.187234056
0.206447173 0.146925374 0.20273372 0.20166055 0.189880491
0.210394501 0.202501663 0.21740083 0.180591858 0.209986603
0.1811109580.246187216 0.182928657 0.195893338 0.189782167
0.210180603 0.208555328 0.178681914 0.189100077
0.166120206 0.207708838 0.1937111340.234224328 0.170635786
0.207591705 0.190265757 0.155080834 0.190061761
0.203238798 0.17292033 0.183667399 0.183690858 0.20963417
0.228574596 0.198209802 0.187401479 0.219910578
0.188652845 0.166640582 0.175768076 0.19436446 0.214569337
0.201537004 0.19468527 0.198899198 0.165865152 0.207155084
0.206551935 0.19839012 0.181492675 0.182064454 0.18598039
0.221724477 0.18958461 0.169918592 0.182965803 0.154988094
0.206493983 0.191334062 0.199293742 0.17832767 0.22726743
0.2112538050.218971541 0.205704556 0.205590986 0.179204877
0.19970402 0.164227721 0.19281739 0.183977833 0.161355567
0.221993997 0.168516359 0.186663433 0.21485301 0.207478473
0.1872181170.179904379 0.200589853 0.174381423 0.178965269
0.169186801 0.195487738 0.178566094 0.203981558
0.209557717 0.167402988 0.161759079 0.17621956 0.163705686
0.189319563 0.160426615 0.2116278060.21100944 0.212885831
0.173932704 0.165085277 0.202166155 0.180619397
0.204960072 0.168699356 0.224769925 0.214897958
0.195520525 0.161220263 0.173657224 0.16715633 0.212929365
0.207646959 0.15703002 0.204042774 0.168008837 0.190924657
0.1916788110.196872595 0.193422967 0.185984321 0.216073279
0.167615743 0.174886866 0.204637329 0.206487883
0.192675868 0.207026389 0.2115542250.1730119470.187655104
0.197885787 0.191891439 0.1921211260.156903982 0.225967637

0.203007375 0.181042731 0.205660721 0.195262288

0.215447375 0.1611937890.199625506 0.19847023 0.186017032

0.201637597 0.195388789 0.16566958 0.161702907 0.169966754

0.183397605 0.165340048 0.143877765 0.193342728

0.152964906 0.172099537 0.15813511 0.190202612 0.160599209

0.191836258 0.197821828 0.185146276 0.154239926

0.181699767 0.190442336 0.178739219 0.176383374

0.188742709 0.179996885 0.170691519 0.187570395

0.166465099 0.133365821 0.186568292 0.179435402

0.169168886 0.174963649 0.190916304 0.15023661 0.182966336

0.183743483 0.1781128070.236362854 0.149144186 0.199454438

0.160069507 0.20862552 0.1948391120.1745861180.18680123

0.178859539 0.178274861 0.181822074 0.174051955

0.197586227 0.187604826 0.229872481 0.194564174

0.154037768 0.193072923 0.205731204 0.202107884

0.227764235 0.201275794 0.200772662 0.170150435

0.170437693 0.232933424 0.212243765 0.157390086

0.191864012 0.17865875 0.22392122 0.194793804 0.235763046

0.190398816 0.2127113470.192757893 0.2121139690.162899536

0.206640873 0.215473288 0.175324706 0.186286298

0.180331519 0.198874157 0.157718267 0.225065716

0.197819185 0.203444628 0.1611917110.2142511240.161847542

0.178904052 0.203848835 0.186040658 0.209082639

0.190848403 0.17977162 0.187298463 0.175552301 0.191456986

0.179105719 0.165819343 0.18768571 0.159654994 0.207612211

0.186375273 0.19764937 0.180380559 0.217018228 0.17071557

0.183245154 0.226649354 0.203370796 0.173978406

0.1599305110.17152562 0.171449485 0.167504942 0.202112246

0.205317969 0.191544822 0.156344732 0.170867184

0.168458773 0.221723029 0.225158965 0.177647548

0.166480195 0.170752634 0.208855338 0.181462203 0.18671269

0.205955235 0.214542332 0.19789736 0.19754225 0.196422864

0.196339803 0.19154248 0.205848048 0.18698959 0.196752323

0.187322923	0.192996535	0.205348881	0.179082933
0.164141328	0.199949396	0.194151294	0.180155669
0.197627157	0.22407413 0.147772606	0.183874991	0.162159036
0.201678554	0.1846118420.199061999	0.195444217	0.170164616
0.174688152	0.195103292	0.193629506	0.214502955
KEGG_O_GLYCAN_BIOSYNTHESIS	0.080850585	0.039767052
0.031064576	0.005150945	0.016253572	-0.010158995
0.056364047	0.050745215	0.03851079 0.023951035	0.023213112
0.02127038 0.023619266	0.099895296	0.023093287	0.022153954
0.027391653	0.087868605	0.071418654	0.067710344	-
0.032572186	0.038975892	0.036380959	-0.010026913	0.063967913
0.02702442 0.020644374	0.055731203	-0.030567774	0.086145523
-0.026531084	0.029043016	0.005593269	0.045464179
0.060037804	0.012697346	-0.012461179	-0.017754742
0.025549438	-0.005913303	0.035431863	0.066539122	-
0.010439071	0.069697674	0.003945916	0.001245126	0.005563672
0.00847834 -0.002601581	0.032212758	0.005387157	-0.019123246
-0.010254746	-0.014460518	0.035679644	0.036800562
0.025162854	0.017386424	0.044349308	-0.002203181	-
0.024439069	0.038913452	0.058210936	0.01191778 0.023739482
0.040037364	0.045160618	0.043515512	0.057838596	0.02823408
0.101309495	-0.006490728	0.025106167	0.05597076 0.076227634
0.063829499	0.0636711210.042301781	0.047049516	0.003933349
0.020268965	0.0203368 0.006220841	0.056838506	0.020993694
0.014296741	0.022937868	0.017513162	0.012374956	0.03521318
0.031931042	0.049632825	0.033076728	0.0287729 0.068501591
0.028965441	0.042929779	0.0563113440.02136385 -0.030755805
0.047483813	-0.020151701	0.073533545	0.072033772
0.019267568	0.042008176	0.03898685 0.023501262	0.037658983
0.013803148	0.040996204	-0.001913489	0.000196388
0.054899626	0.041575926	0.0317711820.0381154840.040676892
0.000964928	0.057846512	-0.012498225	0.080241341
0.027580292	0.0371129820.051056642	0.019271717	-0.00994232 -

0.012418722 0.077256531 0.007694605 0.029066893 0.077388536
0.065581742 0.045552739 0.036529931 0.036184984
0.042799278 0.062510476 0.030617863 0.064572911-0.006933233
0.05646709 -0.016378326 0.07124491 0.040396721 0.07820259
0.064736701 -0.000971561 0.055753162 0.036529953
0.033194274 -0.006222342 0.005266232 0.065918365
0.065738008 0.071701727 0.04955448 0.055715343 0.001596458
0.049329175 0.032931812 0.018616471 0.094510825
0.073264017 0.046052591 0.020281293 0.061345836
0.044218919 0.036042781 0.087656318 0.025783179 -
0.0014911440.049048681 0.0011432620.019267434 0.058844861
0.040266608 0.019272871 0.028981819 0.017828188
0.034839185 0.075769429 0.019668103 0.034542676 -
0.005199907 0.083408 0.029625173 0.026587009 0.019288449
0.1301110630.060947892 0.008759092 0.051475933 0.064446184
0.058622627 0.036384798 0.022485275 0.032280061
0.053154221 0.078153457 0.085664464 0.08078508 0.008377051
0.027258923 0.05583595 0.061215746 0.1153691780.012289699
0.041884519 0.014305783 0.044688006 0.075961423
0.064482694 0.024836479 0.0630111820.079206141 0.029066428
0.032416648 0.073466367 0.045001716 0.046577843
0.064213549 0.062994326 0.074289869 0.018320336
0.073932937 0.063504592 0.062403714 -0.028475849 0.07907307
0.063637201 0.003324461 0.016350056 0.04442822 0.046830554
0.028232036 0.046920613 0.05184469 0.044981205 0.051878247
0.0311163940.1181321740.008706377 0.027440078 0.089055423
0.0112176220.091393239 0.042231863 0.073719271 0.011360613
0.023777143 0.016018485 0.065740093 -0.00140771 0.055358393
0.048763572 0.044533624 0.085346394 0.044916201
0.040024064 0.022686614 0.071575455 0.040906133
0.016261333 -0.013987714 0.001667621 0.002230388 0.02752298
0.022677819 0.030150463 0.02746435 0.014569809 0.05830425
0.006280684 0.053555521 0.00748168 0.012295216 0.068669741

0.06313053 0.0403117230.014103988 0.028046352 0.025567099
0.048399418 0.052783197 -0.002814207 0.073992749 -
0.016444193 0.015917706 0.066553882 0.052918187 0.051296971
0.012968708 0.006690594 0.060080133 0.013993086
0.036577676 0.093592414 0.037191907 0.030850125
0.032032093 0.077529492 0.056290616 0.007502928
0.006826327 0.022414823 0.02496537 0.091677122 0.058904066
0.024573476 0.063536423 0.030826915 0.018399463
0.007745065 0.013272302 0.018615651 0.015201265
0.023249743 0.053127283 0.028395185 0.029057135
0.034002232 0.01565616 0.028289086 0.002710642 -0.039409851
0.025495546 0.058344678 0.021992652 0.047403367
0.018990389 0.13333063 0.042827782 0.05358158 0.035797691
0.007012203 0.04613791 0.070363672 0.006033269 0.055478136
0.074864104 0.04790976 0.097175844 -0.002016881 0.038279538
0.010921583 0.076424435 0.066130826 0.051494399
0.038860067 0.015180304 0.0065835
KEGG_OLFACTORY_TRANSDUCTION -0.444667163 -0.448748066 -
0.432320147 -0.450264501 -0.451838565 -0.43877482 -0.442201763 -
0.442306333 -0.443966961 -0.441604061 -0.441761444 -0.457131357
-0.446453496 -0.446985668 -0.446976416 -0.443638829 -
0.451106586-0.444946653 -0.440020265 -0.446150799 -0.43886652 -
0.450052825 -0.457763118 -0.455670296 -0.45135669 -0.451823493 -
0.449090689 -0.452234534 -0.438665498 -0.441446117 -0.450099697
-0.445601599 -0.448086078 -0.445602111 -0.453858431 -
0.452218109 -0.447679103 -0.445132642 -0.444558229 -0.441581331
-0.451837959 -0.442620146 -0.452125134 -0.452400917 -
0.449095166 -0.44396404 -0.452810638 -0.433445596 -0.450115074 -
0.441077822 -0.452043599 -0.446788149 -0.448252463 -0.427399564
-0.456756681 -0.44514095 -0.443043549 -0.450637588 -0.450666162
-0.431224108 -0.430627764 -0.450797663 -0.452925798 -
0.452814749 -0.449822554 -0.448697456 -0.451287808 -0.451673598
-0.442325142 -0.45024675 -0.447808972 -0.428687668 -0.457251692

-0.43480692 -0.45386887 -0.452425529 -0.441115913 -0.454202374 -
0.444881864 -0.442480453 -0.444407173 -0.447196911 -0.451760495
-0.460060792 -0.438122329 -0.442878004 -0.4413184 -0.451126661
-0.455462215 -0.452234937 -0.45423587 -0.455571357 -0.441972029
-0.445429664 -0.453058214 -0.453482157 -0.435555317 -
0.453044682 -0.448759214 -0.44077511 -0.446814809 -0.445020966 -
0.445486654 -0.451295227 -0.456525786 -0.452265258 -0.439944974
-0.459799449 -0.448867919 -0.449898273 -0.450577878 -
0.454464686 -0.450208731 -0.454702357 -0.451001536 -0.457835628
-0.453259043 -0.454156551 -0.446769083 -0.443087049 -
0.439054344 -0.459431072 -0.445201575 -0.448802511 -0.450624361
-0.451207494 -0.44227226 -0.441126716 -0.451168463 -0.448692988
-0.449900755 -0.448834159 -0.449927615 -0.451640199 -
0.457589608 -0.451783734 -0.442726397 -0.436191494 -0.450927184
-0.44664441 -0.446097022 -0.447935653 -0.444848576 -0.447728835
-0.451776626 -0.453238122 -0.45369607 -0.447150235 -0.440291602
-0.439263587 -0.448254529 -0.440264542 -0.449034638 -
0.450467997 -0.44679915 -0.448876132 -0.455705519 -0.448043944 -
0.444151717 -0.440628842 -0.447867755 -0.445510039 -0.441247641
-0.454694364 -0.460088897 -0.436242742 -0.455928511 -
0.447643848 -0.445408276 -0.457056809 -0.45342346 -0.453481531 -
0.443576434 -0.448467563 -0.448531573 -0.450873296 -0.449271355
-0.448571465 -0.449057318 -0.450636198 -0.444144093 -
0.453464149 -0.457239744 -0.441473839 -0.439864449 -0.455922339
-0.448231307 -0.449535061 -0.447325599 -0.447468427 -
0.447096873 -0.452144684 -0.449184815 -0.440413422 -0.442745996
-0.432589253 -0.446733396 -0.449310153 -0.451609963 -
0.454704689 -0.447747882 -0.441499278 -0.444410177 -0.44646607 -
0.436441605 -0.425047919 -0.45711585 -0.452524008 -0.452927349 -
0.438870406 -0.44034676 -0.440858238 -0.451518281 -0.450519636 -
0.452649115-0.448347035 -0.455347875 -0.457661213 -0.456607859 -
0.453792356 -0.450702094 -0.458044442 -0.454908513 -0.444058333
-0.438242953 -0.438511552 -0.446725869 -0.447325659 -

0.45122835 -0.454166965 -0.455079222 -0.446892942 -0.453455959 -
0.440694705 -0.444812455 -0.440384531 -0.448489747 -0.453752424
-0.443237367 -0.447837996 -0.453139743 -0.45472095 -0.454967588
-0.459550237 -0.448297117 -0.448815839 -0.452590894 -
0.455598387 -0.451971878 -0.449245324 -0.452305595 -0.453587431
-0.449142727 -0.445079325 -0.460950185 -0.453515997 -
0.453340431 -0.449402518 -0.448786229 -0.449043021 -0.454099044
-0.458283757 -0.45744688 -0.449406571 -0.44944883 -0.454031838 -
0.455509343 -0.441239207 -0.440720449 -0.446588289 -0.449267637
-0.453609943 -0.439939094 -0.43713057 -0.453694232 -0.439865921
-0.450774595 -0.450519458 -0.450137183 -0.449674591 -
0.443141571 -0.447718521 -0.447565028 -0.449422006 -0.449519293
-0.448686045 -0.444105562 -0.45277412 -0.442994758 -0.450104117
-0.454789099 -0.455140759 -0.452223672 -0.454805588 -
0.457653616 -0.452418053 -0.457118954 -0.450522194 -0.441377574
-0.447094463 -0.447697847 -0.443196494 -0.455515351 -
0.433747432 -0.451226354 -0.439604021 -0.44164124 -0.45324113 -
0.450299254 -0.443078848 -0.455027545 -0.44361613 -0.44965957 -
0.438867403 -0.439022308 -0.446808689 -0.446809189 -0.448709954
-0.452420554 -0.445548504 -0.449576458 -0.439269125 -
0.448390716 -0.445888184 -0.446467886 -0.445288714 -0.447930679
-0.456127031 -0.449572403 -0.452002625 -0.440061038 -
0.454952996 -0.450987397 -0.453522664 -0.451470434 -0.446999965
-0.451151284 -0.451316161 -0.434501081 -0.45398232 -0.442228745
-0.442137267 -0.438178381 -0.450843 -0.445039028 -0.451128153
-0.456326296 -0.457955599 -0.459690612 -0.435443129 -
0.442386469 -0.435607829
KEGG_ONE_CARBON_POOL_BY_FOLATE0.142819276 0.125950135
0.08823876 0.169035675 0.173348595 0.152957382 0.173317457
0.159722527 0.20034534 0.128166503 0.189949313 0.17223612
0.157656388 0.155480592 0.189785388 0.175264305
0.171635967 0.127907661 0.165763237 0.166625209
0.166677768 0.147876376 0.162655916 0.152682668

0.147690273 0.175516374 0.187875964 0.204980057

0.190550214 0.122279527 0.181820687 0.191780124

0.160884267 0.178523725 0.181545901 0.177812067

0.127788723 0.139552879 0.157859548 0.155982468

0.171272041 0.152292514 0.188076009 0.165383394

0.126814663 0.120167151 0.093022393 0.191877389

0.159331441 0.188532863 0.173235672 0.17610609 0.232632951

0.152807336 0.205127133 0.15769191 0.198460857 0.154957934

0.158426841 0.160863499 0.140085334 0.13246713 0.154848045

0.1164037640.236700824 0.208138737 0.178816877 0.163907462

0.16451453 0.16577274 0.136267875 0.18139014 0.145617166

0.159818529 0.143219929 0.146478564 0.154696391

0.193127242 0.133792432 0.155608836 0.16096474 0.220057871

0.156087625 0.1626231190.151036091 0.162897831 0.168667803

0.1168449830.155443368 0.155500937 0.186123983 0.151246191

0.15335949 0.158829016 0.135256949 0.166738772 0.172216937

0.150720701 0.135142559 0.146418634 0.1131736330.205202148

0.14587271 0.1266114320.171226748 0.136853999 0.169833469

0.175865441 0.184686623 0.20445247 0.198745387 0.121548078

0.175096922 0.157416958 0.124163805 0.136495772

0.186223666 0.131826363 0.137851686 0.159345675

0.191788534 0.179474588 0.153205684 0.162129313

0.186567492 0.165095578 0.157810528 0.164108284

0.162746929 0.143862815 0.196481043 0.139602924

0.153172305 0.133146089 0.169702979 0.172570407

0.157061961 0.135002241 0.135716966 0.134315767

0.153098121 0.137370689 0.209929832 0.155018778

0.189096068 0.1176878920.138744315 0.166932392 0.144828004

0.173979138 0.197415521 0.177356646 0.230635641

0.146930446 0.137951684 0.1611654250.12522547 0.171165518

0.200537708 0.099326857 0.162988886 0.190799901

0.191877996 0.149035403 0.143093467 0.148695635 0.15386789

0.208639564 0.154509846 0.177326015 0.189937578

0.170259102 0.165496506 0.165614879 0.19222722 0.146498317
0.134718851 0.12564409 0.165128337 0.132915841 0.108942486
0.140434555 0.172947487 0.174550265 0.157079529
0.153367359 0.169438254 0.140964803 0.183586389
0.145861952 0.164928767 0.18608346 0.155525538 0.152082113
0.100936657 0.149492961 0.148760156 0.161517708
0.179421758 0.189389348 0.13808682 0.17497321 0.11617724
0.179045663 0.1104992810.175161633 0.146056484 0.157832089
0.13681009 0.199952829 0.1274681180.163456643 0.139785512
0.132452637 0.13533777 0.156558521 0.140590013 0.160644224
0.130647381 0.176700145 0.167368897 0.15168446 0.146858796
0.218909805 0.096850157 0.147926338 0.208419726
0.141564745 0.168308046 0.1177257930.178046154 0.132139344
0.223354354 0.1112153620.103306942 0.181351432 0.16675889
0.175268143 0.12446337 0.141077357 0.181947801 0.130578764
0.192179237 0.1411735660.13333005 0.128952715 0.128719523
0.263139392 0.1811593430.176744521 0.155954698 0.126174853
0.1174861750.179349745 0.185609687 0.12438734 0.165557405
0.130032979 0.167849334 0.15542498 0.169971644 0.16067545
0.152744241 0.191773901 0.187883161 0.1176389980.162847919
0.15719089 0.146883376 0.163945973 0.174606572 0.131670229
0.186394553 0.181664623 0.151548399 0.217942741
0.133836981 0.132287709 0.190825102 0.126334662
0.166672251 0.128943909 0.1127827630.17612843 0.14178738
0.210364666 0.136689807 0.146350769 0.1651197340.152923496
0.139798571 0.135904965 0.166152288 0.242384774
0.183431692 0.162273492 0.202752668 0.167993447
0.145345448 0.20016678 0.165109837 0.148959632 0.165912581
0.163071588 0.146607174 0.163521481 0.140061933
0.210292639 0.175926207 0.156779312 0.156091909
0.135009273 0.150869624 0.188369453 0.2112178510.091201301
0.173971443 0.202185277 0.171314915 0.121882918 0.15260138
0.165779252 0.191443351 0.147610604 0.19221687 0.227192628

0.1143210760.176070922 0.15620031 0.1167962350.217108112 0.145794796 0.167122163 0.18458702 0.145834573 0.16259251 0.135019542 0.170675488 0.221209133 0.191234067 0.12380213 0.152754856 0.15974685 0.158164003 0.160128809 0.22188452 0.175237446 0.1137100090.148429661 0.151225852 0.17759057 0.213247131

KEGG_OOCYTE_MEIOSIS 0.197897782 0.185780758 0.185214752 0.19156599 0.185995263 0.170607105 0.200593212 0.194418817 0.187213206 0.203583976 0.205914 0.2113664820.209584563 0.173821542 0.181844503 0.208385588 0.1660522 0.167950198 0.205005901 0.185367168 0.204322006 0.174918731 0.17733071 0.1941127120.201220638 0.188526348 0.194689403 0.197335163 0.202908666 0.159029292 0.189423163 0.208071594 0.207529351 0.17640131 0.216100598 0.186718385 0.214193088 0.181655273 0.18965557 0.209121274 0.182074027 0.188105159 0.197391226 0.194363473 0.187643871 0.189888544 0.194079554 0.164463478 0.181016534 0.2114147830.177249041 0.186955841 0.218928125 0.183144734 0.20015029 0.186411592 0.192613421 0.18708199 0.18526036 0.180280563 0.186302627 0.209746535 0.210650633 0.186321469 0.196676642 0.191275833 0.17913116 0.208892716 0.189891749 0.203163106 0.209716519 0.206942356 0.189428608 0.166280903 0.19633264 0.177970826 0.179361715 0.196444415 0.166966132 0.187614057 0.195920834 0.176573904 0.208537001 0.196054442 0.188183284 0.200144121 0.193286417 0.1728501170.193562562 0.185235987 0.212584897 0.189976951 0.19810203 0.181436066 0.187164148 0.207070229 0.202572189 0.19676563 0.172525909 0.181665142 0.199513151 0.224933696 0.194321828 0.178699215 0.176275171 0.178065927 0.195826828 0.184903391 0.1875466110.176326284 0.205208652 0.212304394 0.206576162 0.180677536 0.209773586 0.180941232 0.204697792 0.181464255 0.186193602 0.213839983 0.194644017 0.215241873 0.174947825

0.207289095 0.225993936 0.184721626 0.19752303 0.194904762
0.191039727 0.184602126 0.1961066110.185969668 0.200807276
0.205627303 0.206905428 0.209212875 0.192899661
0.176892595 0.208928164 0.189328425 0.183859732 0.19750924
0.202505824 0.191923836 0.203028548 0.19652902 0.195437535
0.192621525 0.1834117550.207450441 0.207380348 0.182924079
0.21986266 0.17606799 0.159125793 0.2119028870.20005892
0.197278964 0.216585784 0.165859047 0.200840886
0.202203336 0.20480973 0.195614953 0.184995854 0.173864915
0.204470524 0.214022021 0.187280487 0.198927801
0.2112018460.219552499 0.185217453 0.185665492 0.192936983
0.188626734 0.19357522 0.192556617 0.201372105 0.218303004
0.176287845 0.207974301 0.1954776 0.16803016 0.193549249
0.206669812 0.196564712 0.183883652 0.19564889 0.149918708
0.197998424 0.205417207 0.200929383 0.185189843
0.173087285 0.180364302 0.185766828 0.195743091
0.181795821 0.212408692 0.195220693 0.186398179
0.185945452 0.188763966 0.1820921110.2116592760.180047043
0.179267722 0.19619063 0.196806734 0.19280999 0.177934353
0.206246275 0.188019724 0.1911269370.160501747 0.204755981
0.199272327 0.186437753 0.186313108 0.193488643
0.190595612 0.197519621 0.17720931 0.180495338 0.179160992
0.209942504 0.153513627 0.193605905 0.176375306
0.196558033 0.204929149 0.208068372 0.206397327
0.178990993 0.20343417 0.204989604 0.192502134 0.207838545
0.177804646 0.204846334 0.161460357 0.214741022
0.1727211780.1808502110.186604013 0.173356943 0.218492464
0.213003687 0.19977637 0.210199381 0.176779999 0.186767955
0.202859234 0.206733287 0.183194815 0.17820894 0.178446821
0.2028511510.188240319 0.196521761 0.167261964 0.182210219
0.175251928 0.214428499 0.17816048 0.192772738 0.196315131
0.171300236 0.175934285 0.18916933 0.19314323 0.206655433
0.18417635 0.197155849 0.178447679 0.175878501 0.198518955

0.190880589	0.173881707	0.204413255	0.197001791
0.187409291	0.206269392	0.196042206	0.188701017
0.199591924	0.197828665	0.193123294	0.187410638
0.182612345	0.170248733	0.203405868	0.190739918
0.209405497	0.19890537 0.220284739	0.166609651	0.212983456
0.202831874	0.216920629	0.17714126 0.189008519	0.180291405
0.232956242	0.199370194	0.199473325	0.216742886	0.20428799
0.178493848	0.201665515	0.1783251180.207204671	0.209526819
0.20526176 0.168988259	0.197008512	0.190439958	0.188919263
0.188715264	0.204051287	0.2008181140.198190985	0.201388129
0.192810615	0.177765889	0.18409709 0.185051905	0.182493413
0.187841464	0.22326888 0.197605523	0.210367451	0.197890051
0.190355226	0.190468585	0.1811161820.20047787 0.185704092
0.20566182 0.1811384940.1971134660.174731957	0.223411264
0.170356439	0.204494886	0.199525902	0.197356	0.19172753
0.167887401	0.203588971	0.197300693
KEGG_OTHER_GLYCAN_DEGRADATION 0.201653872	0.249565359
0.240444044	0.272327632	0.249286081	0.277292759
0.254649577	0.292621961	0.277154516	0.218512148
0.267735084	0.124285717	0.207553314	0.232355732
0.212170372	0.228781387	0.168857361	0.332022404
0.207928841	0.208915277	0.229812315	0.336213463
0.275786229	0.298955524	0.291553547	0.265464506
0.242356755	0.216289343	0.281753864	0.3031361170.273669111
0.27991008 0.287983598	0.188745336	0.19468682 0.232318178
0.215390928	0.259228365	0.245218951	0.1711664120.248533093
0.198634347	0.263894799	0.170047017	0.255548279	0.31625696
0.23954748 0.2241160080.283575072	0.28886384 0.254968741
0.299162291	0.153530326	0.267368161	0.254344847
0.247564789	0.208834851	0.295413376	0.269188692
0.287237281	0.302160902	0.294266254	0.235494292
0.285790682	0.26760693 0.262833756	0.326522081	0.299600329
0.27730606 0.231036553	0.287324416	0.1514809110.310006075

0.169367747 0.284218066 0.318409126 0.235825008
0.270475682 0.267507942 0.2099703 0.235230686 0.251415046
0.248885864 0.22162834 0.262643636 0.263097308 0.30392766
0.238763473 0.182807583 0.220033369 0.255359054
0.314178736 0.239848329 0.08634702 0.230350614 0.300516752
0.3251107540.253132848 0.304075607 0.2937992 0.236018973
0.26528826 0.286884153 0.237317339 0.237785976 0.294368647
0.268296296 0.30432348 0.239364729 0.26395973 0.275899493
0.300648551 0.188037603 0.282452379 0.193417902
0.306027226 0.264329351 0.273856209 0.219506379
0.240628627 0.32249253 0.24804992 0.321378958 0.203967141
0.230815355 0.239651955 0.244132723 0.20388727 0.217645355
0.127498388 0.130057464 0.272636047 0.168505603
0.279668586 0.2038118290.314025429 0.262591095 0.259435488
0.270729881 0.236936627 0.254978848 0.231246024
0.282372478 0.263764645 0.289320188 0.252560084
0.243901396 0.269873034 0.253291851 0.29631192 0.239715427
0.218757991 0.273799338 0.290736775 0.2789531190.292849434
0.238252169 0.202536977 0.267357907 0.276325532
0.272737306 0.294612315 0.298066237 0.280765346
0.249452965 0.290415756 0.300372739 0.295395415
0.324077404 0.225526405 0.266989505 0.274720881 0.29374639
0.310596335 0.226683806 0.273794812 0.303666693
0.217649448 0.289070543 0.257854005 0.312727478
0.299188753 0.305986142 0.262894145 0.325100644
0.260676726 0.303833401 0.238349228 0.21340838 0.256540245
0.2877841180.261583356 0.279505523 0.257760436 0.231842044
0.2717096110.22442887 0.28483117 0.2811712860.315359857
0.319758281 0.286253279 0.241844085 0.280870802
0.291412149 0.258880616 0.181959562 0.185015235
0.278900856 0.299399194 0.279513774 0.305834339
0.218394936 0.282066262 0.285618091 0.229631424
0.192739246 0.249826662 0.265763655 0.231885 0.194885529

0.187072883 0.278231742 0.271550961 0.2613511410.237074724
0.2294311060.30262446 0.275783238 0.258336496 0.275820477
0.309533727 0.175152078 0.3117196580.304816336 0.256890169
0.272044098 0.295301835 0.295637877 0.287545775
0.262667135 0.290763385 0.228877962 0.28517924 0.241493903
0.309507485 0.248424157 0.21989403 0.30068348 0.277477182
0.291737278 0.319157135 0.316053835 0.32014153 0.274850594
0.2926821150.263931085 0.25319618 0.235173656 0.269198444
0.273337913 0.247075647 0.264626551 0.226993795
0.286124609 0.303556125 0.277555421 0.307799845
0.254274572 0.217301075 0.277560785 0.273312613
0.256650949 0.2657496 0.264800082 0.26459005 0.239146281
0.262634331 0.260126161 0.235348382 0.235589361
0.245289391 0.257512659 0.285392882 0.309869481
0.304055254 0.316225069 0.29629353 0.313588626 0.304114725
0.277839741 0.310774624 0.273361461 0.201744315
0.283305361 0.265539564 0.247435987 0.248229913
0.330687394 0.270236303 0.246622007 0.277425239
0.287395068 0.229562099 0.290783708 0.313995054
0.318320618 0.280360121 0.254601297 0.294121517
0.261761917 0.287309329 0.187650025 0.279654121
0.238235819 0.263109887 0.247495753 0.2810011150.261245448
0.28812987 0.3065927 0.338772648 0.263594076 0.256802963
0.318079547 0.281023032 0.297947106 0.310380269
0.289792102 0.297771625 0.256371316 0.305840332
0.244299869 0.262805965 0.308832263 0.270452235
0.279402834 0.214677281 0.296336414 0.3111207830.287554797
0.231579844 0.270812182 0.257740952 0.28983798 0.230374732
0.2281176950.280532386 0.274888239 0.184903485 0.26206152
0.195517487

KEGG_OXIDATIVE_PHOSPHORYLATION 0.346215953 0.3294361
0.377444445 0.378493232 0.374207077 0.382732978
0.394378405 0.364638729 0.363247017 0.346190421

0.363772302 0.354596274 0.314324106 0.350333338

0.342225027 0.370201768 0.3691153690.340608609 0.359319948

0.365549659 0.324750044 0.3413768110.395345557 0.380412786

0.3436211730.3640811060.345508559 0.355445429 0.318135915

0.346363265 0.39731303 0.340725654 0.322739893 0.361085064

0.354478277 0.3806291 0.363489946 0.364374891 0.387650344

0.3667301120.353201809 0.351782726 0.366853808 0.373956727

0.378025549 0.366989433 0.335752516 0.379220155

0.389605517 0.381457673 0.337345983 0.382317297

0.365838384 0.350871394 0.390799549 0.332239562

0.361213844 0.365744235 0.386083158 0.364043672

0.394393858 0.379017632 0.358513481 0.374310767

0.346974562 0.381576929 0.383868736 0.382884149

0.347209407 0.368052432 0.345459175 0.3769425110.364504252

0.3801193410.354940458 0.346512287 0.37776926 0.366708305

0.346447972 0.375353533 0.376075596 0.375921339

0.341918675 0.358343403 0.365817775 0.374836012

0.356404523 0.35564122 0.381871366 0.337409815 0.367916188

0.385360618 0.371299816 0.369130456 0.341674279

0.361044016 0.3634113280.357719316 0.359383419 0.370956174

0.363128505 0.345279935 0.369143018 0.369841639

0.378595342 0.357207749 0.337583644 0.359064917

0.383929732 0.339741297 0.401261558 0.356424002

0.378875203 0.3511566150.358949861 0.354206275 0.377236036

0.388936051 0.369035016 0.34549506 0.339689859 0.3345755

0.347015922 0.354821286 0.3381587 0.41636535 0.372313578

0.379198925 0.372897268 0.376481399 0.372957867

0.354621314 0.364760054 0.3550211340.347258824 0.347584721

0.342530069 0.35337584 0.318344724 0.322760198 0.35091747

0.339581563 0.356555846 0.353205358 0.36802396 0.330834681

0.348464441 0.375393471 0.313365797 0.350164528

0.342689194 0.37482352 0.3230011490.354233345 0.343043409

0.296954493 0.319482644 0.345435174 0.349165264

0.336641802 0.354720321 0.328948332 0.358404187

0.358750441 0.346710772 0.353025426 0.321087466

0.364859805 0.334102214 0.345659081 0.344825088

0.375923921 0.37001986 0.371461825 0.351089883 0.339786451

0.359473274 0.3476141 0.376761325 0.336036735 0.370216982

0.3551159310.354234379 0.346857445 0.339620017 0.340991054

0.355281253 0.336425647 0.362158296 0.342593624

0.306681824 0.320375054 0.353283938 0.335412197

0.342019738 0.33004902 0.3442434110.347403195 0.31831261

0.331555293 0.310192254 0.356051718 0.362423317

0.349954908 0.370373653 0.359668726 0.33762502 0.355989937

0.345135534 0.338723168 0.325408801 0.3430118080.322299844

0.359517821 0.340260014 0.324319633 0.331789039

0.328841379 0.30456981 0.353208478 0.315196752 0.349647386

0.350856153 0.347060458 0.318441925 0.366209002

0.324474001 0.334934475 0.377976181 0.329196987

0.354410669 0.357320292 0.351945572 0.359915732

0.324535121 0.329035639 0.319389356 0.348785349

0.346250537 0.345193061 0.3698336110.352652553 0.349024489

0.385336431 0.347972435 0.331697648 0.382000755

0.356717026 0.382186552 0.36190268 0.362303472 0.328544377

0.339033126 0.395887738 0.366594688 0.332562014

0.3885036110.375857176 0.375046168 0.37856197 0.369432698

0.339059386 0.388349189 0.334918054 0.377650654

0.345237602 0.380683556 0.362683484 0.359635573

0.357483264 0.366986337 0.371945261 0.252854033

0.397295483 0.361291048 0.3869201170.36726521 0.363110338

0.350125076 0.338048804 0.389459144 0.375584713

0.394295253 0.342594643 0.342164314 0.330897268

0.329283849 0.343240626 0.353674015 0.324627193

0.345198698 0.336339568 0.375929855 0.337220456

0.356060278 0.351881768 0.337439416 0.357321399

0.320529832 0.353166882 0.3527986 0.34646111 0.331719313

0.343671583 0.328419019 0.314885135 0.339097146
0.358350019 0.347550192 0.319402951 0.346783241
0.363014234 0.346225374 0.334219038 0.296435518
0.353081244 0.33699942 0.385367196 0.36174603 0.35257006
0.352442976 0.365950793 0.3206117950.345066828 0.372669023
0.360871934 0.386790653 0.380569126 0.345233303
0.381341907 0.335214526 0.343766526 0.357879282 0.3441015
0.325696312 0.360743024 0.396197755 0.345637842
0.285563007 0.3656116890.309890286 0.335485745 0.357726283
0.325878363 0.29822253 0.331378533 0.352345316 0.322135493
0.312596331 0.304724282 0.33964853 0.328367621
KEGG_P53_SIGNALING_PATHWAY 0.197888986 0.213224371
0.232589752 0.194593138 0.203653804 0.178765747
0.2171195610.2113617620.225772503 0.203405354 0.215318404
0.229483928 0.187884194 0.188552386 0.228165066
0.228067275 0.199076705 0.214984462 0.233291931
0.220234275 0.182798664 0.200380239 0.214784162 0.2033477
0.208527971 0.225066902 0.216132596 0.226707535
0.210997937 0.193047566 0.249538709 0.226673643 0.20897283
0.201076223 0.218568129 0.219469733 0.242121329
0.1911988480.2115385440.22510801 0.209467295 0.206408577
0.221244631 0.206629286 0.2117817030.199500597 0.208581911
0.1691161470.19936591 0.24094626 0.228932413 0.237238105
0.234156485 0.198670326 0.227701057 0.224021977
0.206067256 0.236052798 0.223920825 0.204475086 0.2068325
0.237731568 0.221280521 0.22065964 0.186868436 0.191981402
0.197971388 0.21259811 0.226835527 0.24536693 0.230926262
0.209984461 0.21710993 0.18786161 0.226541757 0.221570781
0.238257446 0.225679521 0.257477526 0.239313764
0.2211335080.18911845 0.205429777 0.219305142 0.210322191
0.220508123 0.232769844 0.220021614 0.220534027
0.201688761 0.200485288 0.238991944 0.222786837
0.205103823 0.222666907 0.201605628 0.2311508550.205771635

0.2091143030.219875815 0.210438847 0.218962652 0.217373288
0.21167409 0.191028895 0.208355328 0.185939017 0.211968329
0.235499468 0.236707488 0.21172418 0.225452972 0.213652858
0.215571533 0.209598621 0.231252368 0.233983367
0.216517204 0.218824975 0.220269097 0.19540728 0.239677575
0.205004039 0.23803211 0.235812889 0.199635744 0.22662658
0.213479212 0.206332358 0.214292793 0.220140964
0.2116595960.24140951 0.234421577 0.238295924 0.19707764
0.237444229 0.219962644 0.230839201 0.22109653 0.207316618
0.240630521 0.213677049 0.209528004 0.230510441
0.230206631 0.239918842 0.214257363 0.219957953
0.218348824 0.240671295 0.208063174 0.239406008
0.185018687 0.18577242 0.236347959 0.250522095 0.244142886
0.225056609 0.204024322 0.200236795 0.209196039 0.252455
0.236142365 0.234161503 0.201684156 0.203178073
0.247978143 0.192325062 0.218249708 0.253228462
0.214713663 0.191551649 0.205710078 0.232194348
0.2237697110.218401737 0.243821325 0.229906686 0.216947491
0.206908544 0.201765431 0.237948038 0.208562597
0.219938352 0.2371907 0.236203614 0.230916813 0.210395671
0.212817569 0.226742423 0.224145936 0.230315807
0.224346291 0.219322862 0.2112279380.2114991570.226432326
0.206678187 0.236138252 0.226169221 0.217939557
0.219290145 0.210274657 0.189580767 0.218694095 0.20798436
0.214579328 0.214932527 0.190159538 0.224444324
0.203469472 0.228813644 0.222054497 0.213956659
0.219384816 0.200368504 0.237071944 0.232324584
0.194297263 0.231771673 0.239915858 0.221918678
0.200043426 0.223272003 0.188946258 0.223125279
0.189168203 0.232233826 0.223296009 0.228430576
0.194586127 0.21644937 0.2175069110.2114478430.23299426
0.2116487480.222544841 0.241876196 0.215121569 0.238004901
0.22087299 0.234742223 0.2283387110.205510678 0.232004119

0.213995101 0.239380877 0.25765621 0.234664454 0.218809185
0.221445442 0.212829277 0.204475703 0.219926257
0.202798387 0.226144948 0.219632691 0.238309216 0.23557651
0.199576421 0.217298364 0.234809643 0.199439438
0.218089771 0.204497371 0.219309052 0.208449813
0.189912372 0.202731032 0.239816738 0.189001019
0.210464012 0.22203683 0.2229019110.213556957 0.21431341
0.230060636 0.201679359 0.202467136 0.230019644 0.23865933
0.236817241 0.217074879 0.2119778360.212996931 0.232649349
0.249721318 0.246650123 0.243214962 0.210716759
0.188810733 0.19291643 0.200665179 0.234294099 0.227637044
0.19236722 0.210692939 0.212649436 0.204089863 0.194575758
0.192495206 0.222551097 0.195944913 0.214447317
0.202283998 0.227466776 0.239450561 0.220527395
0.183329818 0.213332384 0.202474566 0.216945476
0.226738518 0.178230184 0.177576485 0.226744704
0.210783965 0.217893306 0.2038211710.226780308 0.222715342
0.222398097 0.1955550110.21358915 0.180037304 0.213162061
0.237532136 0.193573087 0.230175832 0.258134704
0.237195692 0.242136913 0.222302472 0.210589266
0.220000803 0.225068544 0.2171106940.1822237 0.215755783
0.218704147 0.213677885 0.206073007 0.21992208 0.215755612
0.218993121 0.20440605 0.202515637 0.247075609 0.164842002
0.21463463 0.211683299

KEGG_PANCREATIC_CANCER0.182353942 0.2113105440.210019099
0.182764921 0.192966189 0.191710636 0.184274154
0.199655151 0.204632643 0.209501929 0.184607431
0.2208911140.210087875 0.191434061 0.224995014 0.204720839
0.195109195 0.194895291 0.192482402 0.204609326
0.188573988 0.225778926 0.199686817 0.197416771
0.232659704 0.222568599 0.198002742 0.215938529
0.233814893 0.223337554 0.167974524 0.21701291 0.210334212
0.181948025 0.212696381 0.168444034 0.194180794

0.159423181 0.192703109 0.178382087 0.2116993870.199941737
0.187285893 0.174876799 0.20541125 0.181238386 0.215076896
0.171543754 0.193995745 0.195718847 0.219183718
0.201968678 0.209662628 0.186301213 0.197482337
0.217962925 0.213690468 0.2117651270.158594733 0.203148477
0.195336307 0.196870522 0.216707527 0.204803007
0.202349298 0.195167835 0.166171357 0.189056529
0.200274497 0.205583567 0.233638344 0.189595434
0.206720896 0.18052987 0.203756689 0.247862382 0.197804233
0.213910809 0.203512526 0.205290122 0.196077148
0.185283493 0.1940991180.198315498 0.217668216 0.200562846
0.225953047 0.20526903 0.206544337 0.199328601 0.206775957
0.208285765 0.183532242 0.182734532 0.224452905 0.22015227
0.213149148 0.198368373 0.212851677 0.21179549 0.21504123
0.22852283 0.197248037 0.212971786 0.190621906 0.209464483
0.19827173 0.210648023 0.215447405 0.2093573110.17587435
0.230737104 0.184247664 0.213878157 0.195737449
0.224616332 0.209822535 0.17216359 0.219835601 0.218725637
0.215264966 0.2005211970.223703933 0.230043806 0.223649246
0.176166652 0.217545142 0.206201844 0.176661291
0.2186851130.207917219 0.184441863 0.189440208 0.220678572
0.232524194 0.207266331 0.222396039 0.218053437
0.232381687 0.229752738 0.189889295 0.232506624
0.212528813 0.209423866 0.210195974 0.248857295
0.228831904 0.187403389 0.2197519 0.2111270560.218898906
0.221405972 0.240456148 0.195297569 0.210480003
0.237176846 0.235776123 0.230929012 0.217376879
0.238188085 0.19826931 0.220283995 0.207055729 0.225656711
0.203427146 0.175655318 0.2096205 0.208853503 0.193533962
0.220963648 0.243067877 0.21442248 0.179047213 0.205780408
0.218288743 0.237429797 0.20571247 0.210020042 0.206278725
0.209540555 0.219776355 0.206207437 0.220565705
0.182564382 0.23452851 0.192896513 0.21305002 0.232639305

0.186986179 0.216842652 0.216768959 0.232166339
0.249353284 0.2250811950.209581869 0.225871695 0.210403829
0.226772249 0.224860097 0.232548968 0.240053923
0.202582698 0.23144385 0.2011849160.218662563 0.201049069
0.204322408 0.199056167 0.218439244 0.210700854
0.229277656 0.204751626 0.225180681 0.21453684 0.222518405
0.227696456 0.2177657110.245664804 0.249625588 0.22754632
0.239657035 0.232973915 0.210674358 0.192512669
0.220291553 0.187376522 0.223764431 0.185480579
0.181309537 0.241039382 0.223739529 0.224401299
0.218353522 0.210302723 0.231337121 0.229797531
0.222706438 0.2310841 0.230452041 0.215352312 0.214551063
0.200343935 0.199675604 0.202063039 0.214708991
0.251793353 0.16893573 0.200715841 0.198474594 0.209754938
0.22602826 0.231396499 0.222162509 0.194105666 0.176903366
0.2113860720.202846515 0.215593741 0.198795519 0.226733232
0.183946387 0.210185582 0.198839242 0.21878337 0.201151361
0.215877333 0.201700523 0.230408386 0.183807073
0.207996955 0.21630922 0.200906154 0.220249236 0.176460703
0.215843394 0.188055564 0.2111305660.222338716 0.200082649
0.208791444 0.200577637 0.230401338 0.2116735610.227013548
0.223691651 0.221037594 0.235165646 0.252278075 0.23704288
0.233022741 0.2267891 0.2245115150.188496567 0.200926767
0.225251377 0.229702973 0.214291577 0.21944657 0.229668846
0.226067584 0.1883843 0.193776052 0.237535683 0.210407787
0.219827121 0.235063219 0.243160614 0.2043555 0.222151235
0.217756469 0.217819452 0.203470945 0.206950693
0.2114095290.213624936 0.223012187 0.207086012 0.188784922
0.204930333 0.223579292 0.221742834 0.204639506
0.212768012 0.231310186 0.2025481110.198179103 0.218654319
0.200571472 0.210458585 0.225381456 0.230794541
0.226982834 0.2304351180.222308219 0.238075281 0.227924395
0.2087711260.222489428 0.175802345 0.197982924 0.217152025

0.229469221 0.2026011380.242546498 0.2407164 0.213660341
0.215000696 0.222644403 0.242519169 0.193738274
0.195573161 0.187338315
KEGG_PANTOTHENATE_AND_COA_BIOSYNTHESIS -0.002516553
0.046971894 0.0646117380.047749123 0.046444377 0.05004007
0.048316289 0.075083616 0.033069616 0.031650922 0.00584457
0.066253191 0.002352689 0.044883848 0.040535391
0.070074809 0.004263377 0.120632026 0.064513451
0.005334796 0.042526485 0.102409227 0.064371729
0.064758769 0.031592798 0.0611132610.063199534 0.063635682
0.028246886 0.062734021 0.000391087 0.090234946 0.04678095
0.048079412 0.035858047 0.127003959 0.007082401
0.057875038 0.056365916 0.044217236 0.073109898
0.1144519380.050201529 0.085025974 0.035233441 0.038370446
0.031517337 0.028169004 0.052446573 0.08965657 0.077266379
0.074149998 0.104316441 0.025332895 0.127229212
0.016313927 0.028524141 0.057905197 0.032812412
0.074508674 0.055069552 0.047953013 0.078458762
0.073439247 0.037496342 0.060173354 0.049344446
0.104558546 0.032764429 0.045702312 0.0358711620.023577207
0.034383173 0.040559142 0.034752876 0.066338918
0.006973685 0.080601669 0.045558343 -0.020472584
0.069246905 0.048354446 0.0681739 0.088283797 0.052011717
0.0488355110.084475992 0.045152858 0.055458893 0.106319172
0.03965069 0.052246208 0.045630834 0.008295861 0.091140961
0.105426188 0.071851905 0.066442613 0.087085517 0.0726006
0.058009253 0.064374797 0.1182167270.052429332 0.052139876
0.08401442 0.022530804 0.032174915 0.0911906560.019829774
0.073390951 0.016043814 0.025169712 0.056763734
0.0076115590.078043342 0.07307234 0.057433815 0.03546582
0.0341112940.065503136 0.057587134 0.045547373 0.068766873
0.032905789 0.073567613 0.017289348 0.012109707 0.01993676
0.062345701 0.078694629 0.033236481 0.02546581 0.032755697

0.035558834 0.09177789 0.025890762 0.029228486 0.037260886
0.046783334 0.008989754 0.088831667 0.07376305 0.046476447
0.051083765 0.046663409 0.052320057 0.068835483
0.005845942 0.037623038 0.1142757370.065871337 0.083526243
0.05898895 0.068824139 0.035069215 0.040257997 0.08879938
0.029930405 0.069767285 0.061492889 0.004882669 0.07091097
0.043634361 0.074320283 0.058879522 0.053469597 0.10308378
0.019367544 0.075988954 0.04549027 0.032135999 0.060031571
0.04590668 0.066625292 0.067935087 0.071424243 0.025113821
0.04039067 0.017827731 0.044309714 0.033146737 0.048311225
0.046120006 0.092070779 0.070769746 0.043481665
0.040773496 0.019884718 0.101800989 0.026329306
0.049396888 0.039169417 0.051597061 0.028733813 0.0447476
0.060475014 0.042838939 0.09303259 0.068872879 0.045408034
0.018464285 0.069351648 0.042074287 0.043483735
0.043743678 0.01753207 0.056082841 0.036960227 0.068245138
0.035349262 0.014022177 0.047343309 0.058004557
0.101937761 0.064488349 0.076312696 0.060269625
0.061350901 0.069798051 0.046254428 0.090596149
0.058621998 0.063474933 0.079099646 0.050803087
0.100570322 0.049095229 0.091476638 0.077125188
0.136772065 0.013842838 0.095296513 0.038682192
0.0434110250.042790791 0.083547334 0.07930589 0.085676615
0.062650551 0.025809679 0.062330773 0.052618383
0.047299982 0.06841639 0.045587805 0.070009941 0.041793652
0.074184053 0.061097582 0.042151089 0.065686999
0.043735016 0.04856128 0.036583327 0.037433299 0.065415081
0.0911235470.098540775 0.018568242 0.033535217 0.081567664
0.073018908 0.089212621 0.105386833 0.045134827
0.047174706 0.044291014 0.08554422 0.05929336 0.106817304
0.034566853 0.060102862 0.052915278 0.075431495
0.054349375 0.08350696 0.065584528 0.0711949840.06121404
0.054905276 0.120005942 0.0675451110.055337939 0.066721012

0.053317167 0.02709543 0.014526144 0.0921211160.082026144
0.071797799 0.097587818 0.071535932 0.051610988
0.041348984 0.081460093 0.030574923 0.068798303
0.055481041 0.101908036 0.041791447 0.074274638
0.069576247 0.015046876 0.050943254 0.023269437
0.032692137 0.029769593 0.06320511 0.079386923 0.026794274
0.02084584 0.069980931 0.03593361 0.054792623 0.066144992
0.033631268 0.075424349 0.053083864 0.027827421 0.06863123
0.075399831 0.032801457 0.0110293250.063184901 0.052876067
0.067624363 0.1186545650.046706391 0.080896993 0.0685704
0.02951228 0.048303366 0.089159174 0.035529237 0.057491993
0.067777727 0.035714122 0.053060495 0.1115207740.084783453
0.029687414 0.043623227 0.063538488 0.037772168
0.068798012 0.08364029 0.062532597 0.068086913 0.051079455
0.036642901 0.033569563
KEGG_PARKINSONS_DISEASE 0.371009403 0.36315767 0.392938246
0.39671584 0.405667909 0.392199423 0.39896234 0.384190802
0.387088737 0.371605473 0.389493312 0.377546319
0.338543257 0.357307207 0.37008108 0.388332336 0.382064474
0.369876847 0.3793388110.382158595 0.352515215 0.363280262
0.410300453 0.403760343 0.351413662 0.392267871
0.369023547 0.375102262 0.346973294 0.3652258 0.405906076
0.360641477 0.350406327 0.379066663 0.385167477
0.394686979 0.385487451 0.376502921 0.404405488
0.395284975 0.376886366 0.376078824 0.390655257
0.395187488 0.397058884 0.383260852 0.365839251
0.389239304 0.3967311680.40472101 0.366512883 0.402952304
0.394478288 0.377063968 0.39371412 0.352466103 0.389576106
0.387406748 0.4111687170.382962815 0.4116887480.396992893
0.385950288 0.3871160320.370190274 0.408161631 0.40515375
0.401539024 0.369149829 0.383659642 0.374471405
0.396368209 0.389599102 0.389165671 0.384108212
0.366823555 0.390269312 0.386638426 0.36198873 0.385099101

0.400455243 0.396226402 0.371675769 0.384722527

0.398715044 0.389924209 0.383729516 0.384308153

0.408689824 0.36078429 0.391485586 0.413218797 0.393545983

0.391048693 0.366101345 0.381816086 0.375673012

0.377800795 0.38334996 0.390702317 0.383979274 0.366772424

0.387059093 0.390256443 0.397958384 0.381587006

0.372170585 0.373561374 0.388683371 0.361387951 0.41607556

0.380925025 0.382936842 0.371967854 0.381331985 0.37452777

0.393918041 0.4139411260.377981212 0.379503692 0.368220197

0.368846885 0.372030125 0.38197071 0.362580598 0.414869356

0.39464225 0.399401292 0.389729579 0.408318014 0.392974452

0.368006558 0.389903188 0.361980124 0.368397741

0.3721211870.367295716 0.376757188 0.346308947 0.349467413

0.374866641 0.362957925 0.377474622 0.36351834 0.390199771

0.352591713 0.370566227 0.397910527 0.322058704

0.376074759 0.371025512 0.401766588 0.348216272

0.380040405 0.358808598 0.328001518 0.35810045 0.377596438

0.373043938 0.359436471 0.372703578 0.350524397

0.384853525 0.382244298 0.366301202 0.373617436

0.350022962 0.385169256 0.348145844 0.362213654

0.368414317 0.404036652 0.402454084 0.392803423

0.371306664 0.356228679 0.384359908 0.375884604

0.389433381 0.359876757 0.385434455 0.37410649 0.366417222

0.371792759 0.367102438 0.365410718 0.373188763 0.36587815

0.375938705 0.365921991 0.324801449 0.336398408 0.3770749

0.359222052 0.35006171 0.359049394 0.36136418 0.371700922

0.350563775 0.352836151 0.336336895 0.389882458

0.393227483 0.374998718 0.387755228 0.389442302 0.36307799

0.373895878 0.357675912 0.363949085 0.351374156

0.3641611580.345886543 0.387043023 0.36556479 0.344869812

0.348001067 0.349376376 0.332435246 0.370100631

0.343650577 0.372930003 0.365882633 0.364682041

0.342807128 0.3711488720.351032137 0.358378666 0.397267881

0.355315343	0.377917231	0.375028325	0.378887935
0.384582817	0.33916605 0.355925822	0.347528252	0.375116659
0.373967777	0.368164156	0.382276443	0.3711078430.37796988
0.405348133	0.362892293	0.353736287	0.39414654 0.387218046
0.405834925	0.3792113810.379405363	0.353805332	0.362022673
0.41585399 0.389178834	0.363586659	0.4119237990.396743666
0.393529191	0.392529978	0.388648762	0.357683472
0.4111262210.356416076	0.390486718	0.366208784	0.394051989
0.373883607	0.376933929	0.379767247	0.389357502
0.385412675	0.277517179	0.407449168	0.38861431 0.392814324
0.385096639	0.391859387	0.382228295	0.36144131 0.412643831
0.395207093	0.408813437	0.3693056 0.363307286	0.356448962
0.353905166	0.368891873	0.371797941	0.353193187
0.364727863	0.354730509	0.39080856 0.356595136	0.36221799
0.376576312	0.360546062	0.367532766	0.336902241
0.368734172	0.366836263	0.358524034	0.355386347
0.374784228	0.347875235	0.3381794 0.355293144	0.380382069
0.367181553	0.340926753	0.361340057	0.375694031
0.367957548	0.346717257	0.318870701	0.385303945
0.360528073	0.401704485	0.379232008	0.376396208
0.374336621	0.388645655	0.357492698	0.364973966
0.393338943	0.381631558	0.410331067	0.3985168110.364920103
0.398396505	0.365685766	0.361955972	0.375793753
0.367227091	0.34206981 0.379364005	0.410946771	0.367031436
0.3115268090.388342555	0.331612779	0.349088387	0.376997712
0.356260373	0.327759832	0.357174506	0.380419891
0.339089867	0.342718133	0.321755299	0.358413178
0.354157697

KEGG_PATHOGENIC_ESCHERICHIA_COLI_INFECTION	0.34151777
0.334514837	0.35655371 0.318698065	0.337156453	0.32212019
0.335831494	0.339147037	0.328186573	0.335631574	0.33304848
0.351509148	0.330013489	0.340467578	0.351018704
0.337027904	0.3335941 0.345791047	0.320053199	0.344882159

0.315304015 0.336316436 0.329079741 0.328001027

0.347304173 0.341465276 0.336154327 0.3445811840.339418671

0.346695824 0.321386304 0.336080404 0.330673861

0.332488251 0.336186252 0.315242037 0.351935224

0.316001733 0.332266075 0.3011565350.343159949 0.322959653

0.337972879 0.340231028 0.338693305 0.348836198

0.337609622 0.318785778 0.323204705 0.336730328

0.369372178 0.3301143350.335395757 0.333425921 0.338434526

0.353389815 0.344901316 0.34340726 0.332981263 0.347398174

0.324625686 0.337775197 0.3425110130.350047875 0.340748738

0.333020897 0.325337722 0.350359347 0.333817294

0.327573938 0.359132523 0.327038671 0.348245997

0.330963034 0.335777614 0.3748595 0.334051098 0.342628967

0.342630522 0.34919962 0.338713263 0.318527027 0.325195362

0.344503674 0.345266179 0.330398413 0.34219046 0.36795776

0.346460838 0.345425261 0.32546252 0.350312597 0.342701406

0.335165836 0.340202242 0.344397978 0.338654707

0.313384628 0.329910649 0.340757967 0.344153773

0.331821944 0.327007205 0.342700871 0.318615396 0.33364961

0.331683956 0.337103086 0.333229185 0.356389823

0.330123902 0.3429967110.326962226 0.326847356 0.340456211

0.34445697 0.3422471160.344057526 0.350732527 0.360169734

0.334854564 0.363492026 0.347555133 0.3521179680.339982827

0.307173271 0.331946722 0.340023232 0.332994695

0.3406115440.346377227 0.331479422 0.322144783 0.355144301

0.3507511930.339764327 0.350089874 0.35024294 0.357934388

0.350103047 0.344646769 0.359965797 0.326777873

0.3354111170.325667839 0.36008028 0.352689281 0.327152826

0.323404599 0.351685822 0.34880922 0.331058598 0.369738552

0.332048061 0.346190979 0.346482329 0.367522496

0.343735723 0.345691945 0.353545316 0.348714363

0.332449996 0.341467607 0.340702342 0.342520247

0.334531421 0.343492723 0.349502623 0.3359916 0.33092308

0.353749774 0.337111308 0.320442081 0.322461821 0.331386129
0.351758051 0.354427245 0.342517692 0.338399596
0.346930045 0.349131039 0.319884912 0.334209715
0.344139494 0.347549731 0.326966501 0.345296727
0.359384948 0.332553786 0.336052026 0.35385524 0.346221083
0.351650585 0.356643728 0.350269944 0.350237954 0.34204876
0.348309375 0.341306528 0.341875871 0.347906827
0.329521777 0.338432848 0.343747564 0.333325543
0.351170843 0.328055407 0.340659312 0.346587274 0.348949586
0.35631382 0.343279726 0.353490646 0.345910751 0.353080663
0.345878135 0.348330895 0.365507381 0.370080849
0.350102879 0.367145361 0.356466232 0.334856896
0.340411215 0.376419041 0.334240089 0.348354857 0.334169829
0.342457326 0.367377215 0.341379675 0.347709862
0.344931948 0.338993187 0.356794262 0.34388289 0.362783373
0.349647274 0.348090892 0.342978282 0.335735499
0.354166934 0.348165568 0.324210785 0.336249749
0.344611842 0.316706376 0.328550077 0.332437126 0.344184075
0.355848838 0.358691048 0.35809809 0.342988211 0.329060791
0.352293936 0.338200794 0.349448095 0.336798158
0.349192555 0.343130372 0.334462296 0.334381407
0.346240865 0.347816018 0.339393425 0.330828135
0.346251914 0.331075665 0.329389469 0.349750295
0.346054146 0.351569393 0.324501855 0.345803797
0.320053591 0.348405854 0.343288165 0.329924632
0.333634678 0.347819906 0.364038548 0.345499055
0.340023781 0.335795826 0.329960507 0.359332144
0.360553675 0.358814092 0.353415519 0.344508563
0.347250412 0.314682233 0.324937525 0.348888685
0.367326612 0.327071942 0.358054187 0.354957795
0.332169469 0.326094888 0.32686752 0.357496883 0.338168757
0.351035478 0.35452462 0.350477954 0.35314586 0.338181726
0.340747652 0.35433997 0.33426673 0.336994132 0.341548229

0.33752613 0.343126953 0.339292234 0.333502377 0.342343029
0.351540779 0.348466722 0.348706352 0.334152312
0.338645001 0.328929803 0.321330334 0.346727662
0.335808899 0.331867619 0.348873564 0.367789444
0.346614983 0.338646509 0.345394658 0.355310651
0.346658652 0.345527947 0.354291462 0.342475609
0.2981117780.351762985 0.337276203 0.341598554 0.34024734
0.342696395 0.326797279 0.349305648 0.337844626
0.351721254 0.338449523 0.3380118580.320066528

KEGG_PATHWAYS_IN_CANCER 0.158692368 0.183400168
0.171306412 0.150441457 0.150441538 0.147964384
0.1609678110.168707529 0.150402304 0.168700329 0.142072838
0.162745954 0.171692127 0.161592077 0.184436409
0.149226393 0.171340398 0.145665187 0.15921245 0.176032814
0.16519227 0.161369226 0.153783142 0.163258314 0.186589627
0.164319682 0.168012548 0.160475129 0.172345893
0.169845532 0.14886492 0.159848495 0.177568079 0.171331854
0.179739231 0.125173006 0.174465003 0.1194054650.145350711
0.141422023 0.181090736 0.1361167480.142853057 0.139607914
0.163553609 0.153762379 0.183478105 0.150908738
0.141603703 0.143859173 0.175782185 0.140130431
0.148487581 0.152258586 0.145555426 0.198817872
0.157835691 0.165325306 0.144957889 0.182193424
0.136569612 0.173156541 0.176498883 0.16497433 0.153169758
0.148668019 0.133405375 0.150976861 0.163189026 0.16616689
0.179560572 0.155002263 0.177249943 0.158852669 0.16977308
0.196045766 0.166332423 0.172240718 0.183256236
0.173135996 0.151950476 0.134172955 0.163080497
0.162443345 0.14204843 0.156213426 0.174332408 0.1879019
0.16762148 0.181375262 0.161353074 0.157035315 0.154767666
0.145782233 0.172136813 0.172931261 0.151085763
0.171207596 0.154696406 0.150696298 0.156086387
0.166066975 0.14728695 0.172412898 0.157209003 0.164973991

0.1456796110.173724856 0.160753274 0.178102036 0.130133388
0.168484227 0.158261099 0.164732836 0.161698552
0.185093504 0.156084997 0.142666848 0.171020616
0.176454106 0.165591266 0.161652062 0.169507388
0.192543732 0.157672737 0.123149925 0.161414591
0.168324276 0.149631358 0.158067238 0.173895983
0.164502785 0.152977889 0.17430396 0.184108734 0.1604393
0.175855794 0.177469221 0.194799252 0.186428316
0.154693718 0.194357232 0.152291977 0.179089123
0.1514561170.202175631 0.178668755 0.147247978 0.161354245
0.173056361 0.162456283 0.156561805 0.173830208
0.160605161 0.177100036 0.174295724 0.200639041
0.176947884 0.15722602 0.184293728 0.1521181660.167153612
0.155225595 0.190897731 0.1834266 0.136358 0.183779108
0.165714167 0.166586722 0.172123575 0.168426906
0.162771782 0.150740215 0.140641721 0.163909237
0.183425548 0.172593821 0.170465881 0.150138746
0.175890963 0.166688254 0.151749524 0.164076559
0.169626266 0.166782776 0.164008541 0.167735843
0.187109866 0.162123324 0.177949443 0.190964083
0.172300859 0.178672168 0.1711685990.172533883 0.18714379
0.168612244 0.171020242 0.190096953 0.171070374
0.195572942 0.15444439 0.179769472 0.165820761 0.164385049
0.1611064180.172177662 0.183356363 0.178343653 0.161632027
0.192207818 0.159372161 0.18535605 0.182276583 0.169089442
0.195982196 0.175850804 0.199927308 0.20815512 0.178037454
0.196585914 0.182298801 0.171753715 0.166945701
0.195563564 0.174128882 0.177783295 0.15645738 0.15438216
0.207417898 0.169068687 0.167294572 0.176361869
0.159394573 0.199433664 0.174601035 0.17330341 0.186831004
0.179165725 0.173263858 0.154946606 0.177843695
0.153892342 0.1577118210.180933263 0.193142256 0.129181838
0.154657316 0.14817976 0.169901879 0.17989664 0.177840838

0.170638192 0.136795598 0.160968743 0.181716607
0.167712827 0.16939816 0.1611066620.172560489 0.160126887
0.173536873 0.157125265 0.162129829 0.147226004
0.176316968 0.133809961 0.165480085 0.166479692
0.177890667 0.15654526 0.156165577 0.201041658 0.145495854
0.175914089 0.163971515 0.171522282 0.146765465
0.175124369 0.165087234 0.149541345 0.173863066
0.165044351 0.1811374720.177351015 0.162271495 0.187161774
0.180809103 0.188770185 0.190536798 0.17270539 0.18964985
0.144907857 0.168178572 0.170827716 0.199939584
0.186597873 0.18260479 0.164858006 0.165541694 0.15072005
0.157240233 0.195347857 0.16152389 0.16939869 0.181090783
0.165965385 0.169394929 0.174667141 0.191262818
0.182817431 0.170176863 0.184875533 0.16259601 0.189848665
0.180494599 0.1567023 0.15132683 0.166051344 0.175117035
0.177977987 0.137912712 0.179351825 0.183642263
0.136789335 0.16363214 0.151266489 0.146853495 0.16221833
0.182129169 0.181294979 0.1894911140.177484737 0.17540694
0.195166751 0.180707684 0.161646776 0.181014415
0.161959847 0.142651778 0.195536696 0.199830536
0.172649158 0.177979828 0.178488674 0.164783101 0.16569139
0.181427778 0.200379894 0.169923691 0.1611576470.145148994
KEGG_PENTOSE_AND_GLUCURONATE_INTERCONVERSIONS -
0.110763858-0.125099954 -0.055556259 -0.086147555 -0.114808 -
0.081196654-0.072897088 -0.104456146 -0.099280813 -0.134853668 -
0.080799293 -0.112728504 -0.123933693 -0.082049076 -0.120598083
-0.084240804 -0.111645618 -0.097015031 -0.133578233 -
0.088893874 -0.068479559 -0.118993904 -0.09828401 -0.12793577 -
0.135588749 -0.094370613 -0.074471583 -0.094564041 -0.081183529
-0.116414739 -0.024366205 -0.125954252 -0.144954701 -
0.104494729 -0.12799266 -0.088746807 -0.118112204 -0.125974917 -
0.099139096 -0.084306807 -0.128927439 -0.141893939 -0.108550275
-0.089922191 -0.11194419 -0.093011592 -0.114872342 -0.122646591

-0.126424212 -0.05931188 -0.11971917 -0.089277596 -0.119628402 -
0.094734864 -0.099997185 -0.099164841 -0.12686826 -0.087214165 -
0.08092242 -0.124101585 -0.103439942 -0.108659569 -0.093663909 -
0.097599414 -0.121487711 -0.047969226 -0.102968776 -0.0826511 -
0.094942154 -0.119513081 -0.112302893 -0.117809347 -0.096273186
-0.108331552 -0.107030966 -0.068123862 -0.130830334 -
0.108404541 -0.113860343 -0.09452393 -0.115882443 -0.072495078 -
0.146788975 -0.095927827 -0.092490245 -0.077742634 -0.087289386
-0.107291119 -0.096254391 -0.103200655 -0.089378885 -
0.045415671 -0.06776633 -0.095884339 -0.121106928 -0.117613629 -
0.051767851 -0.13876346 -0.105823171 -0.08526074 -0.116517786 -
0.138775152 -0.023639983 -0.124316473 -0.089290257 -0.115702205
-0.122061675 -0.096133757 -0.094875827 -0.12500665 -0.101599108
-0.112600682 -0.085552723 -0.117382326 -0.099492415 -
0.11020256 -0.079895886 -0.070776501 -0.137416613 -0.12330497 -
0.104837908 -0.144451226 -0.103399728 -0.109685434 -0.121964971
-0.109733316 -0.095131382 -0.066108611 -0.093145711 -
0.101882673 -0.120055827 -0.118608282 -0.085024113 -0.110490364
-0.103259285 -0.121304055 -0.093587198 -0.084673717 -
0.103227961 -0.117171305 -0.099481924 -0.095288222 -0.100111405
-0.096657844 -0.098043445 -0.128372511 -0.12418032 -0.01109972 -
0.099584994 -0.111258481 -0.128277141 -0.10573924 -0.130343174 -
0.080444009 -0.079026713 -0.144741259 -0.120968414 -0.122183645
-0.102424838 -0.119685479 -0.095731725 -0.102234883 -
0.054350929 -0.113971063 -0.102939378 -0.140285089 -0.084795278
-0.085964922 -0.085775271 -0.053610282 -0.063429164 -
0.064617412 -0.097377599 -0.094496484 -0.117517226 -0.101360129
-0.071241914 -0.136866961 -0.028295496 -0.116882164 -0.1183622
-0.0829861 -0.098062564 -0.101989956 -0.122203993 -0.110042267
-0.116848601 -0.104246747 -0.095607727 -0.113150539 -
0.109882612 -0.111240839 -0.095746285 -0.116037024 -0.12254017 -
0.104774982 -0.124556033 -0.14593633 -0.135573183 -0.137297534 -
0.107085708 -0.07702157 -0.125776442 -0.121157397 -0.092396235 -

0.042565254 -0.111994108 -0.097445602 -0.101796216 -0.092749929 -0.102919804 -0.118331448 -0.111254063 -0.070283929 -0.102603141 -0.116288164 -0.136900859 -0.125714696 -0.108408854 -0.081028847 -0.11230063 -0.092070783 -0.115002445 -0.111370798 -0.138178191 -0.08673848 -0.132128307 -0.06824764 -0.100490984 -0.112227727 -0.085312871 -0.081990192 -0.073293078 -0.095574032 -0.071489952 -0.102338287 -0.067325225 -0.106170346 -0.136015159 -0.101550591 -0.106922126 -0.102843192 -0.114607847 -0.099780166 -0.111154966 -0.10234093 -0.090468879 -0.116402771 -0.002444495 -0.094891485 -0.100905584 -0.121056314 -0.106156158 -0.079447898 -0.079238019 -0.116358756 -0.084168647 -0.069329462 -0.088760252 -0.106661605 -0.088892001 -0.109915033 -0.060098945 -0.085083841 -0.094210603 -0.103793569 -0.089250508 -0.098382488 -0.093953463 -0.071765899 -0.080002694 -0.109839694 -0.122562327 -0.050835278 -0.077153874 -0.103183045 -0.096152017 -0.113038994 -0.113956703 -0.111925488 -0.084294453 -0.115263217 -0.089023667 -0.117538927 -0.106342316 -0.161391363 -0.103119407 -0.101881391 -0.088139077 -0.099478359 -0.099155509 -0.084915349 -0.08710378 -0.08193118 -0.109848359 -0.107960849 -0.12393733 -0.089543366 -0.123113213 -0.102984824 -0.096226067 -0.110503406 -0.126453952 -0.107511979 -0.127256241 -0.113575732 -0.105446029 -0.102914189 -0.132826725 -0.109043896 -0.113183904 -0.111973839 -0.146352501 -0.03193125 -0.128365892 -0.12142646 -0.100483404 -0.085797726 -0.095501777 -0.084047765 -0.057849421 -0.057736636 -0.119759248 -0.083469307 -0.121857066 -0.108728378 -0.097355478 -0.065171332 -0.10351914 -0.090574182 -0.106436052 -0.09080557 -0.111258888 -0.098918871 -0.11918457 -0.090590198 -0.090601886 -0.09869662 -0.129377309 -0.027989101 -0.102382946 -0.119196751 -0.08014995 -0.137827969 -0.168329671 -0.132737709 -0.100614058 -0.048292129 -0.154569359 -0.089199464 -0.124808268 -0.09729898

KEGG_PENTOSE_PHOSPHATE_PATHWAY 0.252231361 0.254025145 0.2414711640.300265587 0.289464746 0.251621324 0.272580106

0.27152556 0.236402852 0.26790007 0.252772249 0.281504758
0.263767709 0.271916961 0.27686522 0.265289843 0.236599683
0.277356873 0.267964183 0.24797372 0.256395923 0.290049901
0.298773289 0.26125788 0.270030399 0.268663857 0.286313913
0.285034708 0.252740941 0.26482579 0.24921692 0.284024634
0.270571529 0.261408549 0.262244472 0.276339804
0.226487356 0.242378207 0.284789717 0.226375799
0.2561192680.2941347 0.279009938 0.2811886760.265065619
0.263672092 0.283124448 0.260341255 0.32049128 0.286869408
0.247568551 0.248346654 0.270169169 0.237415418
0.285565012 0.287282363 0.248156002 0.249665125 0.28420074
0.257489435 0.271937928 0.269320195 0.272914489
0.286608227 0.273898551 0.277951424 0.289675489
0.289104396 0.259488616 0.267616089 0.287326849 0.25851849
0.258784138 0.22927665 0.265001743 0.254872122 0.258278197
0.2769051170.258413685 0.272361478 0.300145054 0.26788545
0.250951385 0.2756116070.298174575 0.2785117910.26779532
0.259909755 0.279647831 0.261389557 0.302387098
0.267615305 0.2772615110.289871632 0.294272927 0.26746339
0.266667398 0.246865784 0.30834248 0.290969869 0.288288514
0.264430508 0.266786451 0.274157919 0.27745925 0.250677577
0.288398035 0.283764375 0.26883765 0.266273353 0.286506892
0.228317302 0.255873429 0.266257858 0.316886579
0.246769813 0.275398044 0.270304679 0.237236975 0.27872551
0.247577124 0.29953364 0.268923865 0.239721893 0.279712113
0.266041561 0.264106177 0.264531442 0.267440128
0.275260221 0.277053846 0.250715305 0.307955842
0.271745034 0.290491984 0.300962299 0.281023729
0.255679456 0.270623289 0.266975415 0.278632289
0.261594814 0.285344916 0.25494071 0.279884877 0.269084902
0.249737766 0.256098075 0.236307194 0.27372538 0.249861282
0.27268853 0.262164361 0.254410414 0.278520461 0.264737655
0.284175881 0.296885457 0.290344983 0.285176618 0.2685348

0.283226959 0.290288348 0.281003507 0.279919633
0.269561276 0.3061149130.286020028 0.276057506 0.279673146
0.312440387 0.289472292 0.279031246 0.273630138
0.273817852 0.291779049 0.280096109 0.28539966 0.260259526
0.273406328 0.289426307 0.251337984 0.3035011280.238579051
0.251698408 0.263494925 0.269739013 0.26325569 0.260583399
0.277527812 0.27434157 0.297567961 0.275501323 0.282576821
0.257278612 0.239050171 0.312640893 0.279517935
0.279336293 0.280537444 0.275992539 0.261525809
0.268248827 0.288637417 0.25865691 0.312059533 0.275107514
0.258367555 0.256777496 0.261635902 0.264259175
0.280986127 0.277531483 0.270242396 0.291856019
0.291460816 0.2762321110.277014075 0.295297928 0.271594192
0.25945147 0.309973589 0.253188348 0.268466473 0.268381987
0.237917333 0.278598316 0.26473149 0.252684562 0.259387479
0.302678164 0.2701133640.2970152110.274995223 0.274324455
0.293095634 0.326220475 0.274230868 0.280139039
0.263901465 0.257272223 0.26754401 0.274512736 0.259306113
0.278663423 0.25430395 0.264890677 0.2954753110.254233323
0.284969284 0.286760663 0.268512892 0.286044595
0.281613941 0.29353027 0.244596276 0.281748925 0.264247007
0.268047032 0.276257785 0.278120479 0.257826295
0.293520144 0.233237022 0.287557908 0.287595399
0.290067037 0.264549514 0.277984582 0.253083237
0.264390893 0.278255634 0.243523962 0.275772127
0.247633438 0.259189525 0.269247336 0.288305534
0.283516976 0.274687312 0.255161665 0.291208946
0.263697171 0.286134447 0.279894328 0.283547977
0.273743259 0.278881585 0.286825193 0.289306337 0.26194157
0.255638149 0.26926892 0.272659712 0.27537497 0.282267668
0.312418375 0.2711589740.293047265 0.299298865 0.271742407
0.266485564 0.284766037 0.275956906 0.319431592
0.271275957 0.275796386 0.292998338 0.291327346

0.272063432 0.298856501 0.267341801 0.24636623 0.287282165
0.237098824 0.2663541180.300685008 0.292679627 0.282115558
0.255164642 0.264207221 0.287230196 0.263424225
0.276770102 0.287914274 0.239181834 0.291616938
0.263217802 0.291810639 0.268500257 0.291593998
0.282596207 0.2698417 0.312169914 0.267553616 0.272050706
0.25815814 0.309615413 0.222483222 0.3110694110.280504794
0.264444893 0.266204844 0.263434743 0.260775557
0.265095974 0.306921872 0.25134265 0.254088478 0.22783254
0.263672338 0.2690318

KEGG_PEROXISOME 0.139480946 0.139055861 0.127033815
0.168926821 0.174742145 0.154221871 0.182683087
0.165254368 0.169427516 0.156582829 0.176650223
0.163520376 0.142325523 0.177235949 0.137601391
0.187947994 0.151540197 0.198894433 0.168214013
0.1375265110.145402372 0.17282327 0.200895957 0.175918823
0.150146437 0.175030912 0.161339387 0.158384561
0.160883226 0.1595014110.156458282 0.188710968 0.133188692
0.167455718 0.156408384 0.196796482 0.124464465
0.177218435 0.198560643 0.159368856 0.155752769
0.187652713 0.159263439 0.195919063 0.177983324 0.15098183
0.160853398 0.141412788 0.192459082 0.179532351
0.147716499 0.182430658 0.175976389 0.143142978
0.186865738 0.151791533 0.158034571 0.151794715
0.179222304 0.13210174 0.199156899 0.15162727 0.157365434
0.173731771 0.178814582 0.168769948 0.180500415
0.183472664 0.161331628 0.1621160920.169694684 0.147824579
0.142944682 0.146936172 0.1473762 0.145752307 0.157243011
0.168767614 0.163779717 0.15587905 0.181650879 0.181114481
0.155826533 0.1791175350.176513498 0.185359337 0.155860864
0.147466144 0.1633097 0.141769001 0.171269376 0.1683528
0.148074246 0.17596249 0.1649941140.14141612 0.156797376
0.124137539 0.182385719 0.176892613 0.18791103 0.155695879

0.173740015 0.191693533 0.176430426 0.177222728
0.169151869 0.176162343 0.172787759 0.14575607 0.168313268
0.12928975 0.139310171 0.152741861 0.158853406 0.134128346
0.171909048 0.176749621 0.139597733 0.150151734
0.148058586 0.151998575 0.160222219 0.122593771
0.166693734 0.193058728 0.1541175710.147227187 0.186012147
0.18997408 0.145416201 0.148263626 0.177732878 0.151523076
0.147960661 0.154228412 0.143466328 0.132623877
0.132293895 0.1451497 0.151237715 0.1164681830.16674104
0.174814337 0.168737521 0.152492866 0.135030249
0.168774128 0.132447004 0.120709224 0.151203137
0.159171497 0.156958826 0.170159751 0.171542966
0.139488346 0.133233685 0.166745364 0.1761171990.158503568
0.176884204 0.163830905 0.171307851 0.141923447
0.159779014 0.1886325110.14424133 0.1816556 0.182982851
0.15665184 0.173066557 0.161322339 0.18343859 0.176478529
0.149423186 0.174401308 0.16967518 0.150108299 0.161830077
0.17024674 0.155719583 0.19246939 0.192716605 0.166253814
0.169888724 0.134402394 0.15583265 0.135218364 0.170003597
0.170316706 0.143222559 0.1523511850.148043856 0.154541804
0.143573085 0.122179847 0.141954544 0.149068756
0.145057599 0.153609989 0.138555609 0.150690464
0.174349032 0.162233087 0.147570294 0.156060245
0.152781084 0.133586235 0.154586161 0.157247566
0.1162896380.177764684 0.148755541 0.125832878 0.169531097
0.145598718 0.165346629 0.135402432 0.126601499
0.166261035 0.127344904 0.176210478 0.159194484
0.159079188 0.13354077 0.148327975 0.172124101 0.153071013
0.1543116520.138094399 0.173126769 0.1811323480.141936409
0.186434776 0.131046462 0.156193138 0.189332032
0.155992549 0.166878806 0.175494241 0.153452948
0.178497989 0.186012042 0.162522044 0.181668929
0.146501596 0.200557176 0.184472328 0.173696789

0.1611818830.1772711320.160498217 0.145339465 0.197192714
0.145104044 0.1432491110.166940434 0.171064575 0.174236748
0.165230655 0.165727442 0.162454791 0.160695468
0.164956625 0.184219546 0.147807649 0.204191012
0.155494591 0.154657052 0.145902064 0.181987347
0.168543735 0.1173039940.188691788 0.170213585 0.17329175
0.165673533 0.194042321 0.154388177 0.149633974 0.18377361
0.178724374 0.168383191 0.172865919 0.173341855
0.176438913 0.130462339 0.155558334 0.159995362
0.143067944 0.173401493 0.141826602 0.190683359
0.158756963 0.175440936 0.143890514 0.153981497
0.159263258 0.166810387 0.178881071 0.157481209
0.179858796 0.147059482 0.13915122 0.159780701 0.139816354
0.183340722 0.1613121110.157429705 0.152007889 0.155595657
0.156045959 0.138614821 0.131659009 0.10673962 0.163615543
0.188774325 0.192371553 0.180496581 0.153372041
0.156253063 0.207554564 0.1211419850.1531511540.191026344
0.1488112140.168838006 0.191703728 0.185750751 0.150358633
0.156273451 0.154446927 0.155397015 0.166415433
0.146623886 0.179233612 0.180266649 0.177383125
0.141812969 0.230620258 0.147739314 0.1164323430.149842865
0.134843368 0.158843262 0.141924926 0.154797248
0.171471738 0.127802727 0.156604891 0.165210042
0.134521745

KEGG_PHENYLALANINE_METABOLISM 0.136304793 0.204105665
0.167896278 0.19604799 0.181886179 0.1132326790.137633743
0.128005517 0.209804379 0.17289547 0.1188364550.132672511
0.107513573 0.189556665 0.133724284 0.2094271190.181325495
0.200775409 0.176593196 0.13332185 0.141935694 0.19318191
0.184985518 0.096897674 0.162290301 0.167267172
0.174918028 0.206824266 0.129615127 0.151798627
0.088929541 0.141202128 0.109906144 0.176909665
0.173703602 0.201213061 0.1170105530.204159203 0.162422657

0.14013451 0.1674512110.20051045 0.166572694 0.220995833
0.1461156590.165535296 0.091859462 0.162147195 0.18905209
0.160330818 0.191399008 0.173803602 0.156531366
0.144072801 0.189619803 0.17167479 0.163589674 0.171311649
0.1769111520.107452188 0.198998566 0.141209701 0.179503121
0.1711482050.188476868 0.131754313 0.186917786 0.212461935
0.168965158 0.158431521 0.160653474 0.123304642
0.161600621 0.133952168 0.1331711870.163712182 0.198352788
0.164202764 0.163738225 0.152344855 0.178314543
0.160299365 0.136139294 0.154266082 0.147289542
0.142706975 0.101913186 0.173164647 0.156703366
0.149442841 0.200864655 0.129728066 0.17789377 0.178655116
0.138790978 0.099267796 0.1175305990.122576721 0.178957983
0.160248579 0.130201897 0.129014252 0.182207236
0.158274542 0.168339626 0.216201701 0.2394411960.176547045
0.15739246 0.21882131 0.16801821 0.1402611850.0986725110.122235071
0.162167385 0.185337707 0.145464012 0.151421208
0.155335466 0.15152433 0.156232831 0.142816906 0.184426721
0.141373223 0.140094306 0.156429751 0.147917559
0.144547675 0.196942595 0.163570421 0.164668579
0.154244722 0.192808149 0.128381384 0.14729614 0.139337145
0.095649375 0.184420747 0.141537782 0.223994576
0.159094557 0.177003642 0.179306938 0.163430181
0.162280318 0.143745088 0.144890653 0.159406077
0.151564026 0.195247657 0.168295458 0.140801622
0.087656528 0.120679856 0.183013897 0.158888932
0.082882021 0.18476939 0.150953701 0.201436375 0.173035859
0.135668128 0.152019414 0.1862259 0.1477545 0.232282226
0.142523918 0.1955411970.164935086 0.229523313 0.122657582
0.195861645 0.159004729 0.183406293 0.153263065
0.158037864 0.136626028 0.192833273 0.191843476
0.1479115230.134938304 0.122506837 0.147364562 0.147289181
0.156618212 0.160030898 0.204814444 0.20425274 0.186411565

0.157556482 0.156849198 0.162287263 0.162034583
0.173920468 0.180576149 0.133330374 0.151517446
0.145433342 0.154814582 0.178785728 0.194997926
0.167058437 0.157457048 0.166430809 0.170510348
0.153632024 0.1144983920.157214773 0.164310079 0.172259519
0.128909512 0.189603154 0.180887483 0.135342156
0.154591467 0.13626171 0.160321832 0.189079585 0.184481529
0.177724409 0.129573422 0.18372124 0.181606882 0.160626489
0.144815729 0.108073502 0.210094942 0.1381139650.065920998
0.199700958 0.171476541 0.162788057 0.1190616670.154908936
0.145404064 0.144079871 0.178544693 0.167862861 0.15451903
0.165128972 0.14606935 0.176378723 0.178420026 0.133004055
0.1406113250.144894087 0.196083168 0.194270562 0.159135286
0.1132055160.166855902 0.124913372 0.130510624 0.132063352
0.174005512 0.156730177 0.168402328 0.197204836
0.156193653 0.175429544 0.122872352 0.150593166
0.135719994 0.166137159 0.129088588 0.163472208
0.158592223 0.124981218 0.146596354 0.124714128
0.166842455 0.175155718 0.0832311960.142739712 0.139645198
0.156053654 0.180229563 0.140155295 0.138963353 0.15359014
0.157484219 0.170149715 0.1891130860.174269169 0.160936469
0.176752128 0.1112377240.1250113210.1132138860.163405811
0.156098933 0.135967522 0.149789568 0.120214488
0.168317153 0.172862963 0.12631517 0.125967977 0.165868785
0.141930353 0.174882589 0.187093471 0.17631221 0.159723168
0.223576569 0.146238655 0.160232487 0.186202872
0.180431644 0.144157184 0.156577058 0.177590318
0.147014658 0.099772048 0.099501945 0.167437689
0.162005949 0.152773241 0.162300532 0.099917993
0.1461274110.180608799 0.160378447 0.13016811 0.205406145
0.127412812 0.157829789 0.164376261 0.188259997
0.149094334 0.189934214 0.1588299110.149625135 0.173190382
0.167061996 0.157262897 0.146084274 0.164645613

0.1160794550.26108143 0.177389697 0.135767386 0.166122446
0.062342217 0.171512958 0.177903014 0.247093048
0.151752698 0.125519499 0.107389055 0.193937017
0.165628322
KEGG_PHOSPHATIDYLINOSITOL_SIGNALING_SYSTEM 0.095176824
0.1130233750.084856043 0.084262929 0.095895489 0.073603787
0.105941451 0.1144155730.086952482 0.1178235470.080307337
0.090492204 0.095817393 0.095896201 0.095726361
0.092294656 0.0846792110.091396964 0.095861337 0.088745504
0.085306434 0.121085082 0.093880678 0.082109713 0.11298597
0.101941495 0.074588908 0.1166422110.094608538 0.107559317
0.038987369 0.100965394 0.089599194 0.077810954
0.099686272 0.08654308 0.0701108070.091660274 0.072271209
0.066926618 0.1113210840.100609709 0.0811391 0.088601992
0.10018219 0.1145835710.07855815 0.0943101130.095127952
0.063285406 0.091393168 0.081806658 0.103256427
0.0811312540.093972147 0.0997853110.105430223 0.092683559
0.070699572 0.083689503 0.080491027 0.088147935
0.085901755 0.078167651 0.090328295 0.043224044
0.077039582 0.082260812 0.09673412 0.076439967 0.114399153
0.068281566 0.109039951 0.088136869 0.104893754
0.1132283640.102419961 0.093006103 0.102541373 0.085599105
0.092534714 0.05541116 0.101480745 0.086939698 0.084499929
0.067752248 0.088147785 0.075586212 0.085123837
0.1129209090.064658607 0.054653502 0.074656715 0.073924396
0.1176430830.085294421 0.085004567 0.105581518 0.108890162
0.095213407 0.098615282 0.130642951 0.086615439
0.099455509 0.085303736 0.10974755 0.088370089 0.099093103
0.085121395 0.093244679 0.085156877 0.095759924 0.0758961
0.088128509 0.079657564 0.085670446 0.082228291
0.058421013 0.105504612 0.109487995 0.1190201440.105516192
0.088657805 0.1107604960.1105885520.054657805 0.084764697
0.073359889 0.087153633 0.1133802050.104477412 0.08969957

0.101994707 0.105026038 0.101994825 0.1166308780.118374579
0.105258955 0.098212239 0.1118171720.096566851 0.133228482
0.090242582 0.1155704660.098659708 0.095502644 0.100032462
0.074275141 0.105230748 0.107726289 0.120592683 0.08430772
0.1175009610.066788936 0.10854062 0.1166796780.095554718
0.125344552 0.104747607 0.130059027 0.1128281150.116313122
0.11113585 0.102866218 0.076238103 0.107744886 0.120861408
0.094379968 0.102140949 0.104618932 0.1174078190.084223482
0.092889403 0.092020539 0.101504916 0.1170337440.100452641
0.103091513 0.097004004 0.12361973 0.120925632 0.10291996
0.097557252 0.092708694 0.09890637 0.08582429 0.102634303
0.1155518930.087839173 0.10030575 0.1101280480.134936146
0.1192761090.121278006 0.12263685 0.095424319 0.104298406
0.104802227 0.1179760860.120782864 0.12763339 0.087500524
0.090207323 0.102180163 0.1122649240.086820453 0.111888655
0.1009119450.125842124 0.1108751970.100097702 0.113643367
0.106217767 0.094423469 0.1132764880.121098812 0.123752156
0.109617142 0.127721463 0.1134733070.1044251110.089893918
0.102622797 0.106224128 0.120729746 0.095736439 0.11256465
0.091286397 0.032816915 0.133679303 0.096422941
0.126761268 0.099675748 0.083954948 0.097038307
0.1092210110.1197305340.1142672240.1185659750.094961264
0.088341226 0.095015646 0.0811130910.073363464 0.11706964
0.125913847 0.043616823 0.102988318 0.052928249
0.0879118160.1184674610.1174485820.1101679060.057250907
0.074505663 0.083967897 0.061985687 0.068614577 0.05748775
0.096137248 0.06520516 0.097669769 0.049178989 0.100705167
0.093210925 0.1066344 0.080699422 0.095421291 0.091723776
0.094871501 0.103871486 0.1195123030.134091663 0.042696855
0.072496488 0.081987362 0.101543187 0.075615754
0.106384082 0.120892876 0.056561002 0.102239732
0.055818084 0.1102809870.097845982 0.108607793 0.129009026
0.1184718170.109399897 0.1208553110.124106077 0.138178856

0.076232101 0.09463814 0.082571891 0.1101029820.088291231
0.102978797 0.120445464 0.091873943 0.103655671
0.1105223960.12434616 0.091221233 0.143596038 0.128139742
0.140844496 0.133727018 0.1107974450.121499791 0.125397226
0.098419445 0.082749243 0.1178612740.090917078 0.121939364
0.1155294410.071848746 0.09400323 0.07446218 0.082880158
0.089215219 0.103055537 0.1020401180.091667533 0.110976358
0.107758401 0.071907609 0.102299148 0.082856474
0.145265003 0.078815817 0.107596172 0.101945022
0.129261095 0.130203562 0.087266877 0.101837564
0.133783614 0.1148856170.1113697610.08934452 0.1120700880.11261358
0.124054133 0.098651497 0.1120990440.109867073 0.116446009
0.1187014110.100104125 0.092750684
KEGG_PORPHYRIN_AND_CHLOROPHYLL_METABOLISM 0.036404345
0.050061095 0.0624611930.0840741190.082565093 0.064857685
0.063787268 0.030276314 0.071689081 0.034413925
0.046097341 0.061803399 0.002457842 0.074369828
0.045700717 0.080375091 0.06704575 0.078678416 0.041507017
0.060244104 0.026748338 0.059579003 0.081797617
0.057344805 0.04744741 0.040185687 0.043689892 0.087165048
0.084532978 0.053767692 0.093158955 0.07448457 0.029177454
0.066062443 0.04908261 0.0801102260.007688704 0.065662273
0.0605118670.042603062 0.040696142 0.03646279 0.045376522
0.059308523 0.069617886 0.047059824 0.0392117160.074283244
0.069098568 0.073891415 0.050591075 0.071502355 0.07149246
0.049683876 0.074871276 0.06891853 0.053636541 0.075667349
0.078897608 0.023518885 0.077072275 0.056608742 0.02676956
0.0833976 0.051888506 0.0964431130.069782096 0.068059802
0.067895296 0.035444584 0.053366613 0.047250185
0.0411168280.049473602 0.074102352 0.076147645 0.051138078
0.059228262 0.052476974 0.058434578 0.064491401
0.065785906 0.0118437 0.059472234 0.06557135 0.058662237
0.0634418 0.053045152 0.070712455 0.056288715 0.074391758

0.103314645 0.048805825 0.082309433 0.056323648
0.034276519 0.095183636 0.017552576 0.070188409
0.092014076 0.054018334 0.054951737 0.08291219 0.069676252
0.071673642 0.06482945 0.045962313 0.063685514 0.056811264
0.049263584 0.086026753 0.037943987 0.052444707
0.037608288 0.048156571 0.053793138 0.0729891160.076024435
0.062069452 0.039843172 0.056060444 0.040580719
0.073065543 0.040130121 0.056478413 0.095447835
0.062726947 0.075212361 0.067076363 0.0626611450.048244776
0.057077147 0.076862351 0.054914788 0.040290876
0.014371743 0.054903613 0.055938847 0.037259742 0.03340223
0.054932672 0.05452669 0.0681183560.057270219 0.070794024
0.049583629 0.064109981 0.1128397520.053280779 0.048188712
0.058828637 0.086842084 0.042582071 0.069796571
0.080515715 0.025803436 0.041021859 0.066145184
0.062448105 0.05285736 0.051002427 0.04116312 0.091968268
0.058066805 0.051576149 0.068989771 0.030040363 0.08505831
0.05712942 0.082679435 0.071437742 0.078072728 0.072513333
0.084701097 0.052899588 0.068449887 0.064080358
0.030525946 0.098108541 0.0503117580.093133438 0.043281447
0.079053584 0.073408155 0.071785779 0.052918455
0.051786999 0.029672844 0.048478241 0.063142592
0.055524414 0.042924263 0.058543128 0.071221239
0.047930936 0.068873504 0.046908256 0.03899945 0.05916644
0.046737194 0.0511616740.033610366 0.046318584 0.05703924
0.053435543 0.09428861 0.024400768 0.031232089 0.06061934
0.082984185 0.038388843 0.064961559 0.039803547
0.075776082 0.073783913 0.052956629 0.034760432
0.053339697 0.043290928 0.066139342 0.045388506
0.063680106 0.062039312 0.052437501 0.023913832
0.055169692 0.056829376 0.070762251 0.052129549
0.035659747 0.063228909 0.070983291 0.074627192
0.067381718 0.059042498 0.074003651 0.075841527

0.066813664 0.0515511610.05723385 0.062905498 0.065367315
0.03668371 0.07875973 0.054778509 0.042145283 0.067032562
0.063041464 0.1104675050.062585518 0.066454829 0.079219339
0.059197657 0.082607199 0.070695126 0.061766933
0.076674185 0.079260827 0.070163589 0.072405234
0.045995791 0.066071446 0.075223466 0.079986286
0.054433109 0.062294103 0.108130841 0.063429873
0.067979418 0.07175076 0.085583367 0.075145981 0.010276337
0.079217443 0.061082394 0.057474433 0.064526939 0.06291885
0.061645379 0.049294403 0.066598698 0.058945433
0.069136741 0.043446509 0.080675763 0.062416596
0.037942992 0.075153766 0.066816824 0.06253562 0.042123746
0.081398366 0.076344197 0.058866009 0.063283021
0.059089596 0.0403987 0.070031297 0.056458521 0.049465267
0.055879719 0.077273377 0.049849852 0.067049271
0.060737165 0.051003297 0.052989722 0.069271847
0.054707085 0.055529164 0.067393936 0.053234281
0.016757808 0.0664001160.027434562 0.024453848 0.042852111
0.076478472 0.06990452 0.0826001130.065515508 0.102024444
0.0385511790.053064629 0.068104302 0.044555438 0.072799401
0.084762734 0.088444278 0.061583743 0.056004088
0.063710256 0.055779733 0.084385141 0.04487882 0.075673396
0.08015253 0.056711452-0.006608173 0.129717363 0.045784184
0.029414334 0.089161221 0.025484591 0.03874762 0.054388188
0.079054614 0.106281782 0.020183017 0.072003095
0.040140336 0.01923395

KEGG_PPAR_SIGNALING_PATHWAY 0.077035489 0.078836233
0.07605944 0.085268804 0.1190197950.065319885 0.053399942
0.068604229 0.07802729 0.050359335 0.070859033 0.100748509
0.041048554 0.076297509 0.084538512 0.050154308
0.051092407 0.1156578690.054728435 0.04066101 0.057124508
0.068629205 0.083019957 0.070701534 0.076153166
0.085805504 0.067779732 0.06808707 0.089596961 0.072573872

0.081708108 0.059021929 0.088245499 0.049734395
0.082232997 0.079399777 0.055853853 0.062083731 0.07381412
0.044433876 0.10945505 0.05044003 0.08773913 0.127158787
0.075863269 0.052298905 0.080934565 0.06766134 0.068802775
0.1166812530.130409879 0.067483592 0.046366591 0.064351571
0.081986295 0.088802173 0.090570981 0.122745175
0.091445053 0.061004996 0.124206497 0.0859673110.070565564
0.098810303 0.096902613 0.055655453 0.105283198
0.135046659 0.070607022 0.0871121260.107480242 0.078888845
0.079889075 0.059730607 0.0784411160.098223632 0.028877541
0.0774289 0.075648357 0.099545597 0.136015934 0.066302373
0.058934525 0.049202129 0.071801384 0.072860177
0.081984955 0.103542867 0.086183022 0.0850765110.089154326
0.1119904820.079307568 0.080145035 0.076992398 0.077950457
0.064216924 0.063383767 0.0958116240.1140036090.110901372
0.07656297 0.094143718 0.077853352 0.044126947 0.111956398
0.061315165 0.093452845 0.086277088 0.066458388
0.104900915 0.072179905 0.062458593 0.070800139 0.06102025
0.080554489 0.063258301 0.077762841 0.068690535
0.1127370210.047085647 0.060064687 0.067258334 0.096688211
0.087258378 0.064841779 0.074337902 0.055703537
0.095123437 0.066936132 0.085537537 0.095471714
0.063659253 0.099802254 0.070568769 0.055956531
0.066290654 0.079847093 0.049827938 0.155401466
0.047214443 0.098100837 0.077285476 0.074147148
0.082086579 0.079749138 0.079009806 0.062827572
0.050861935 0.074294283 0.074647979 0.06989864 0.097712586
0.060323373 0.1211850060.064229697 0.08677424 0.084484416
0.071504655 0.066688039 0.093423961 0.065404946
0.068348826 0.103964547 0.093916435 0.072232232
0.086488831 0.10434666 0.077151956 0.081873924 0.064445063
0.065902987 0.06567437 0.069787771 0.073543406 0.077183868
0.091904838 0.050677531 0.092703822 0.070651962 0.09886885

0.067175846 0.063359823 0.074220708 0.074365095
0.067279493 0.103248633 0.10689964 0.098826919 0.051801249
0.088280719 0.080229644 0.077154409 0.064076555
0.077942404 0.10223684 0.094797803 0.1164140950.068930116
0.085727127 0.122916141 0.075134405 0.080932301
0.076258108 0.066412972 0.062886498 0.03372564 0.069532042
0.078363431 0.090775998 0.069062698 0.055664481
0.053805595 0.062319466 0.08696728 0.0579651170.080964485
0.105095683 0.126103399 0.070928869 0.097980707
0.095779275 0.076279281 0.053161328 0.076659199
0.037531063 0.083431609 0.071586376 0.100070678
0.083700291 0.091322165 0.083557699 0.041508731
0.084871428 0.065446842 0.090901084 0.081572517
0.1150874010.1106580790.059916051 0.083145689 0.065600102
0.100120881 0.086665123 0.081893899 0.07835928 0.081291074
0.086167609 0.1232611790.088432573 0.081898438 0.060266464
0.058672639 0.085039424 0.092150605 0.076462944
0.093148569 0.1146351160.08835851 0.136794009 0.095982075
0.098570062 0.09288816 0.076991878 0.1117648440.074101122
0.1180932470.074048416 0.094886496 0.05439637 0.09470476
0.084140074 0.070024537 0.04783733 0.094576042 0.075011967
0.104512219 0.101274721 0.046702325 0.092472508 0.07699848
0.087555072 0.082173464 0.069436596 0.104556057
0.072156678 0.0813441120.078459518 0.12180112 0.08664851
0.064076905 0.076560222 0.086098628 0.034298662
0.102470598 0.1164918960.091339013 0.096887002 0.101648398
0.070240149 0.090495782 0.083245444 0.107759181
0.0490119630.101259198 0.060220828 0.098389135 0.094736457
0.0731667110.091951554 0.087953668 0.074565455 0.087190047
0.032594159 0.039276766 0.109687769 0.085489629
0.1141089820.064916423 0.027606569 0.091373723 0.078474868
0.076557427 0.075725307 0.097798403 0.053595997
0.082195484 0.107627972 0.086671669 0.1113255980.070623024

0.095648328 0.069056873 0.081237 0.084159873 0.075065552
0.076532938 0.065789754 0.058740564 0.100581267
0.092100393 0.060216659 0.070005765 0.068252823
0.065981852 0.076757655 0.058534862 0.051825915
0.071207058 0.060947971 0.059243217 0.064167836
KEGG_PRIMARY_BILE_ACID_BIOSYNTHESIS -0.069788795 -
0.074508147 -0.075393194 -0.094924277 -0.075885999 -0.102514248
-0.071565451 -0.065740231 -0.056432939 -0.084823296 -
0.115651304-0.124495835 -0.113237412 -0.068518446 -0.071556382 -
0.077679149 -0.099872848 -0.000295872 -0.092485224 -0.096696008
-0.135696863 -0.057570664 -0.057854309 -0.07736079 -0.083245332
-0.063120994 -0.089278958 -0.098428184 -0.109768293 -
0.073771724 -0.097116116 -0.089824976 -0.096329675 -0.078773343
-0.120028062 -0.060922961 -0.119739015 -0.111596978 -
0.054860026 -0.15589356 -0.079635186 -0.07591107 -0.092542505 -
0.018689915 -0.028305326 -0.120749588 -0.116589431 -0.074669608
-0.062413064 -0.052582441 -0.069915153 -0.088775411 -
0.087795753 -0.103128185 -0.061208057 -0.083110063 -0.087687453
-0.055980286 -0.057453332 -0.10265985 -0.079607559 -0.08149878 -
0.084362658 -0.043801164 -0.102154007 -0.091016962 -0.027450255
-0.035430503 -0.087971332 -0.098240128 -0.074238437 -
0.144959658 -0.103349837 -0.088836407 -0.131433134 -0.08194924 -
0.048117049-0.081360908 -0.060593335 -0.090300063 -0.066031479 -
0.07142188 -0.08949352 -0.055519984 -0.106513724 -0.082199461 -
0.074866766 -0.067052131 -0.05401423 -0.132767909 -0.106666632 -
0.058823847 -0.082879046 -0.049205631 -0.086036087 -0.06173116 -
0.082516364 -0.156284788 -0.041774646 -0.047042513 -0.056759643
-0.095200698 -0.099667763 -0.071492413 -0.081307822 -
0.074634254 -0.070968813 -0.04927595 -0.045597115 -0.100353278 -
0.07075819 -0.115595058 -0.141245529 -0.110230652 -0.107975549 -
0.096680969 -0.079445622 -0.076848228 -0.087055251 -0.1036537 -
0.091092808 -0.125782607 -0.076167989 -0.110619087 -0.1014477 -
0.064187543 -0.104451905 -0.110944288 -0.092031866 -0.05994373 -

0.085506945 -0.092380952 -0.097601804 -0.078904318 -0.10649708 -
0.109089607 -0.046609593 -0.081076528 -0.12387651 -0.054184693 -
0.142104058 -0.105806183 -0.073420694 -0.076149525 -0.115837724
-0.062817181 -0.108876028 -0.075290114 -0.131059573 -
0.125253456 -0.079433484 -0.094024276 -0.105273856 -0.062933355
-0.072774121 -0.102010494 -0.078215967 -0.087843516 -
0.086819723 -0.100349812 -0.085916907 -0.10322157 -0.071920795 -
0.092666111 -0.041981554 -0.066425936 -0.045522156 -0.054650423 -
0.077708521 -0.079224807 -0.107793484 -0.106144997 -0.101868181
-0.076180882 -0.073235978 -0.074031517 -0.066513355 -
0.071954876 -0.069466361 -0.092193666 -0.109349462 -0.102674179
-0.089159888 -0.057116095 -0.123026369 -0.18157415 -0.084646793
-0.066152564 -0.073283505 -0.059906472 -0.090497396 -
0.130866644 -0.071527487 -0.095747996 -0.066262154 -0.086567117
-0.088279843 -0.128168489 -0.100248171 -0.107521552 -
0.075098445 -0.106625518 -0.081537606 -0.129460694 -0.067292687
-0.10612399 -0.085382032 -0.099320858 -0.088115257 -0.073396567
-0.105247093 -0.097479384 -0.089211424 -0.131691536 -
0.072142216 -0.074281345 -0.082553911 -0.092740154 -0.036528315
-0.116921281 -0.110139646 -0.051570711 -0.079418683 -
0.067668536 -0.10027652 -0.11024579 -0.09990532 -0.097957758 -
0.085166661 -0.063304798 -0.054259676 -0.067286572 -0.142122081
-0.083814647 -0.103165402 -0.098519633 -0.114841004 -
0.069396043 -0.084630921 -0.057954632 -0.087243326 -0.034407665
-0.103519144 -0.074414198 -0.072340731 -0.081674273 -
0.05989652 -0.13241297 -0.082298622 -0.07798668 -0.107978731 -
0.101047803 -0.093788732 -0.10364369 -0.106407691 -0.076923033 -
0.040565467 -0.012978075 -0.087587209 -0.069221347 -0.11207824 -
0.063658899 -0.048128583 -0.084297973 -0.069937468 -0.137925313
-0.058967699 -0.100208382 -0.092773116 -0.083510493 -
0.076684852 -0.09418954 -0.132535579 -0.05744209 -0.109677526 -
0.058697838 -0.063134116 -0.046676799 -0.112994256 -0.090816412
-0.062479401 -0.064408503 -0.061411562 -0.09542611 -0.08372142 -

0.096822413	-0.089490405	-0.088169949	-0.063112376	-0.085173274
-0.070504458	-0.079714021	-0.081418417	-0.135499332	-
0.071022476	-0.108396196	-0.094548832	-0.074537591	-0.089428041
-0.099911582	-0.051121085	-0.07361556	-0.064073759	-0.123922615
-0.128224675	-0.112081509	-0.073513408	-0.09067067	-0.106699263
-0.10075091	-0.054943116	-0.066138629	-0.121545074	-0.12963218	-
0.139565861	-0.022697456	-0.071197685	-0.033772505	-0.08978371	-
0.096216045	-0.068436101	-0.041667892	-0.133552192	-0.107657728
-0.089931815	-0.084881003	-0.05690799	-0.051605433	-0.081717271
-0.069935955	-0.103874776	-0.07396278	-0.128257529	-0.073454462
-0.099310142	-0.106673921	-0.080282914	-0.086378574	-
0.124491633	-0.066253524	-0.083845839	-0.140177014	-0.088948249
-0.095076757	-0.104458853	-0.119345262	-0.081304915	-
0.120492193	-0.121802865	-0.067611705	-0.119829505	-0.143734325
KEGG_PRIMARY_IMMUNODEFICIENCY	0.002692194	0.021263416
0.132808484	0.07685858	0.103292306	-0.01322317	-0.025864819
0.06104514	0.133794787	0.083627889	0.007974073	0.176685385
-0.076507759	-0.029723026	0.140756779	0.06633878	0.021504331
0.070096589	0.086916741	-0.088439025	-0.108550671
0.1131947360.091114526-0.081440935	0.162640962	0.14738522
0.138120084	0.08327025	0.037797394	0.104559572	-0.086540873
0.142908693	0.008676591	0.004848339	0.086016865
0.109856783	0.102028149	0.11516076	0.150339551	0.061594073
0.092860465	0.051698322	0.105228127	0.105956324
0.042015419	0.019760821	0.024780451	0.027632268
0.127179776	0.103265716	0.247724395	0.1974118630.1332961
0.025901513	0.085651797	0.062575338	0.228213218
0.196394027	0.072447916	-0.104641093	0.045264323	0.15843013
0.085494226	0.144552693	0.169165932	0.087225901
0.145043365	0.1661186250.073376399	0.088990634	0.11856171	-
0.003897988	0.071075895	-0.043203168	0.154491812	0.197965271
-0.037605901	0.095326969	0.017932146	0.006291301
0.192957023	-0.027179741	0.108605912	0.04207234	0.218240725

0.121769877 0.175860433 0.188253058 -0.030799994
0.055555225 0.102325763 0.160625469 0.088391525
0.128749786 0.073394066 0.108140204 0.1935811390.066031707
0.214067732 0.281614664 0.087320401 0.043353417 0.0542967
0.111641786-0.084266101 0.153234697 0.080187566 0.108071126
0.150109142 0.1172961360.060142201 0.085566927 -0.092306737
0.029796591 -0.046021398 0.128835925 0.139814647
0.093231012 0.191638783 0.076791731 0.183378738
0.023571981 0.072206263 0.103262049 0.146676432
0.090165776 0.1190637670.102047644 0.030548223 0.098854987
0.0787478 0.1198570160.055375158 0.213677803 0.037764243 -
0.004421913 0.106567067 0.125636812 0.0875921130.105079753 -
0.053160088 0.095733023 0.156416678 -0.081692636 0.070873158
0.076450109 0.153908058 0.159349139 0.098879838
0.100438245 0.210320935 0.074268656 0.076280741 -
0.013501712 0.087603818 0.170073253 0.077053629 0.182044518
0.029680272 0.238370498 0.059481022 0.035332157
0.055060719 0.09204372 0.0866905110.151909201 0.004068486
0.054249126 0.137629526 -0.009502026 0.177072492
0.141244729 -0.036653263 0.150216529 0.198020525
0.245032553 0.056575536 0.034874636 0.195612498
0.062142974 0.16624562 -0.0911804 0.093957082 0.068839833
0.149245571 -0.010716702 0.001053657 0.132747735 -
0.042371549 -0.000175188 0.049075696 0.209894629 0.153581111
0.2112682360.156073219 0.016992742 0.185162692 0.180970405
0.077374532 0.1162648420.142392335 0.063724128 0.065182178
0.033339825 0.132899974 -0.005619836 0.067690147
0.019732013 0.039331538 0.090602868 0.042680303
0.036181073 0.072261562 0.031021209 0.100955184
0.046281453 0.048566904 0.204246695 0.13794047 0.040083027
0.156989058 0.092691829 0.07564193 -0.056031246 0.12343734 -
0.1173897560.1178768 0.04595067 0.083537627 0.063761735
[truncated: 1,035,594 more chars]
