# Supplementary figures and images for: Oxidative Stress Response Biomarkers of Ovarian Cancer Based on Single-Cell and Bulk RNA Sequencing
Source: Oxid Med Cell Longev. 2023 Jan 27;2023:1261039. doi: 10.1155/2023/1261039 (PMC9897923; doi:10.1155/2023/1261039)

TCGA Training cohort

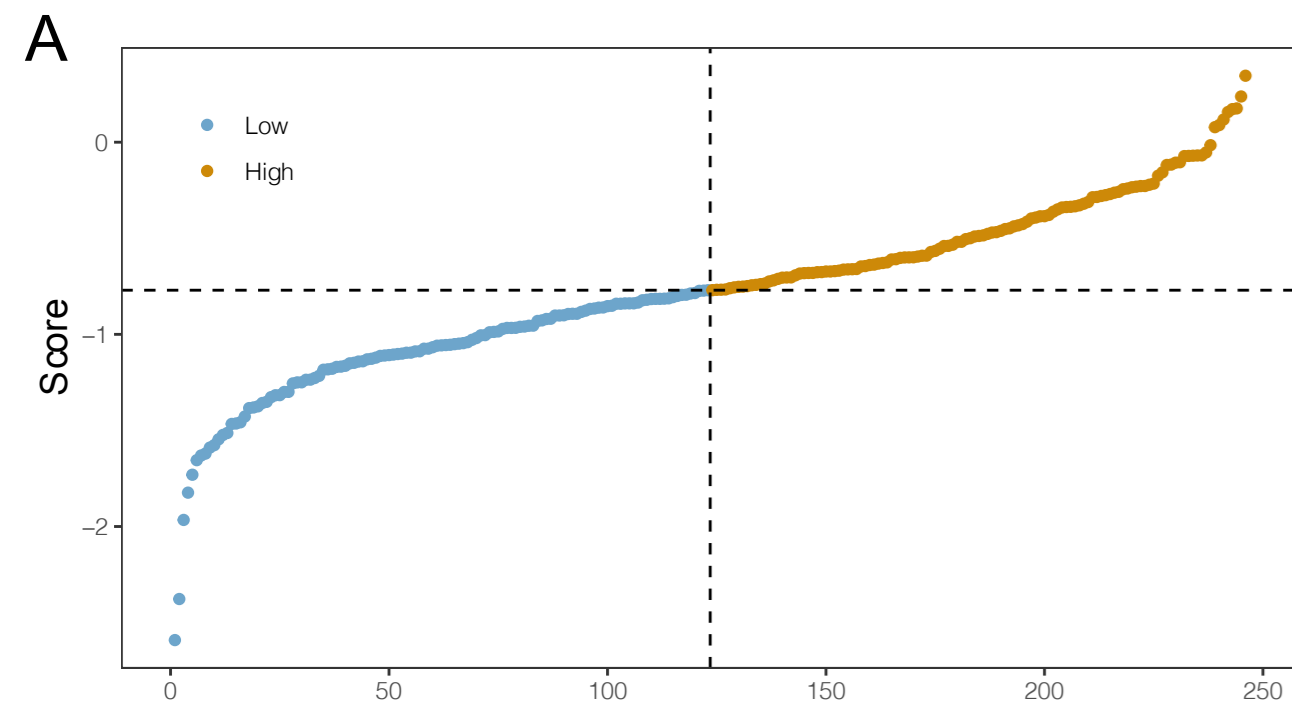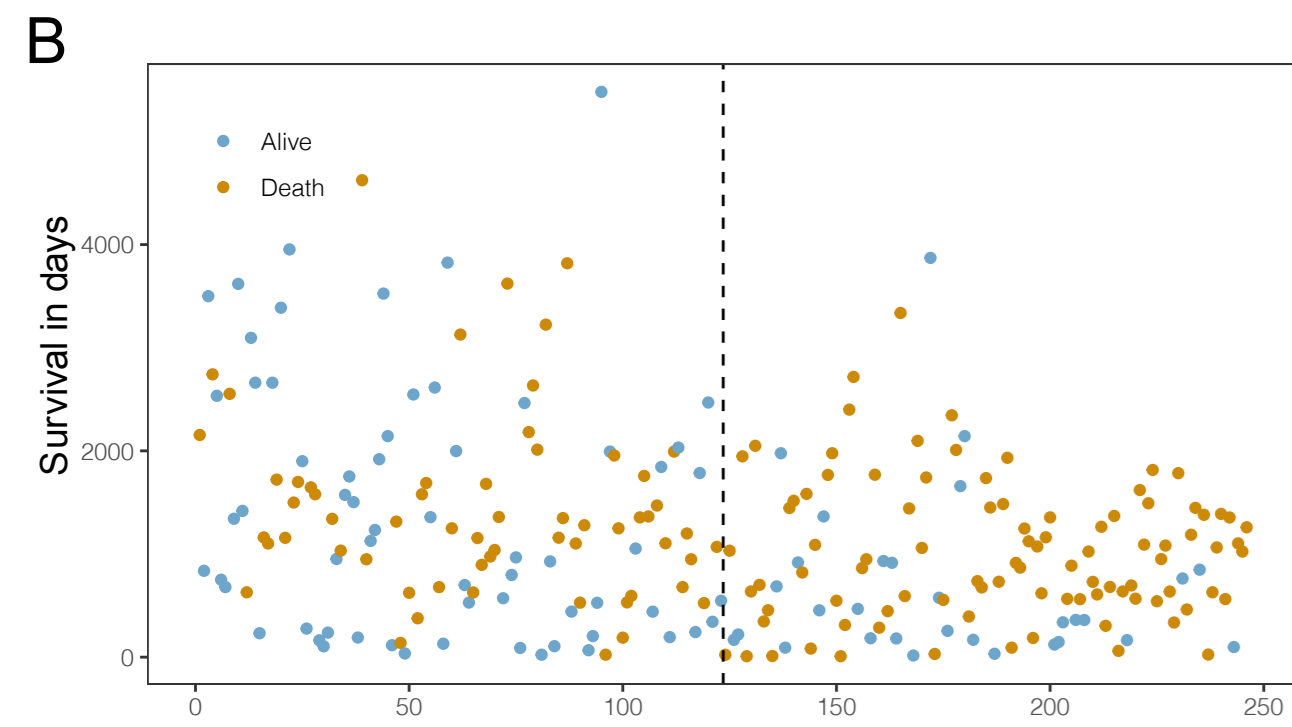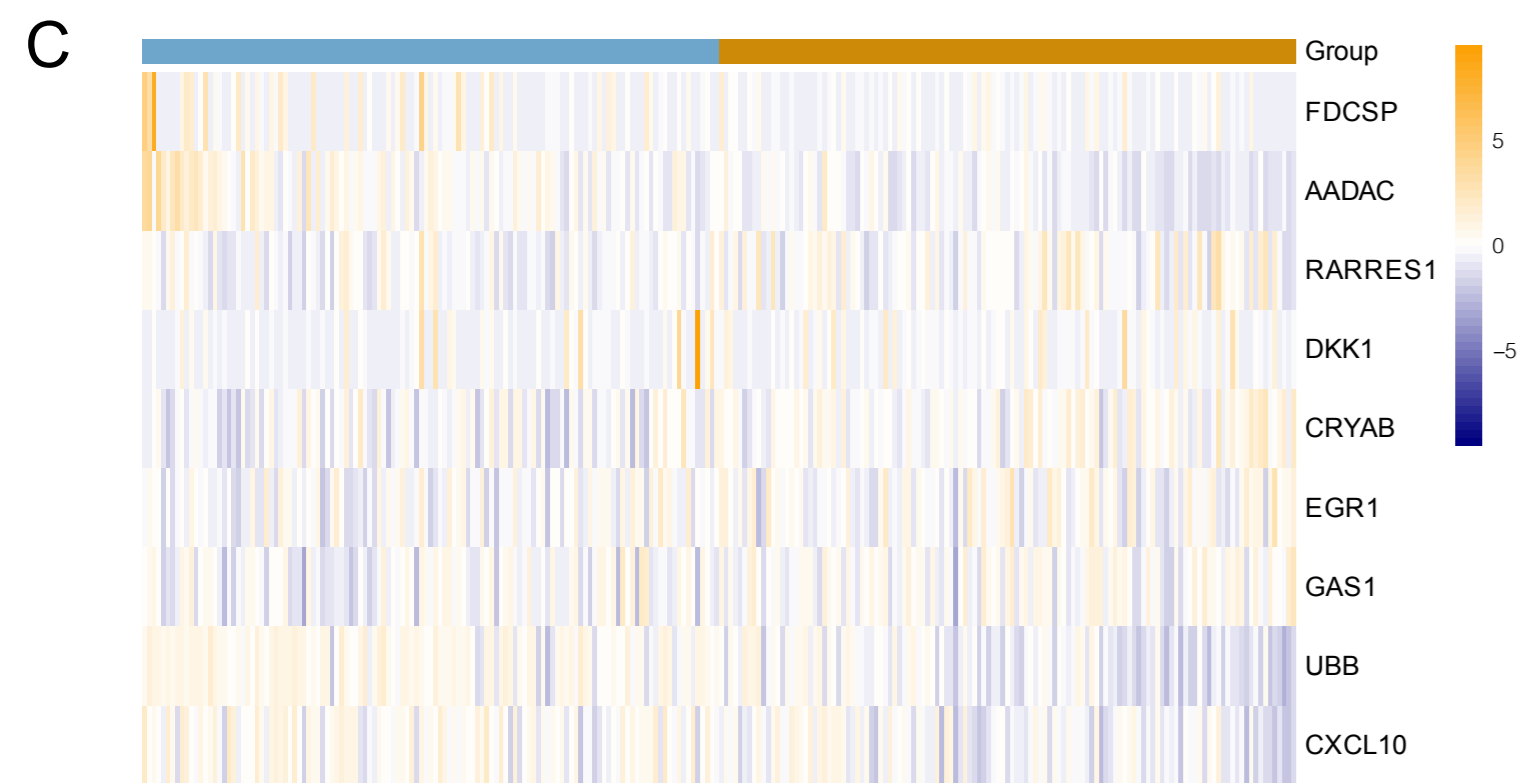

Entire TCGA cohort

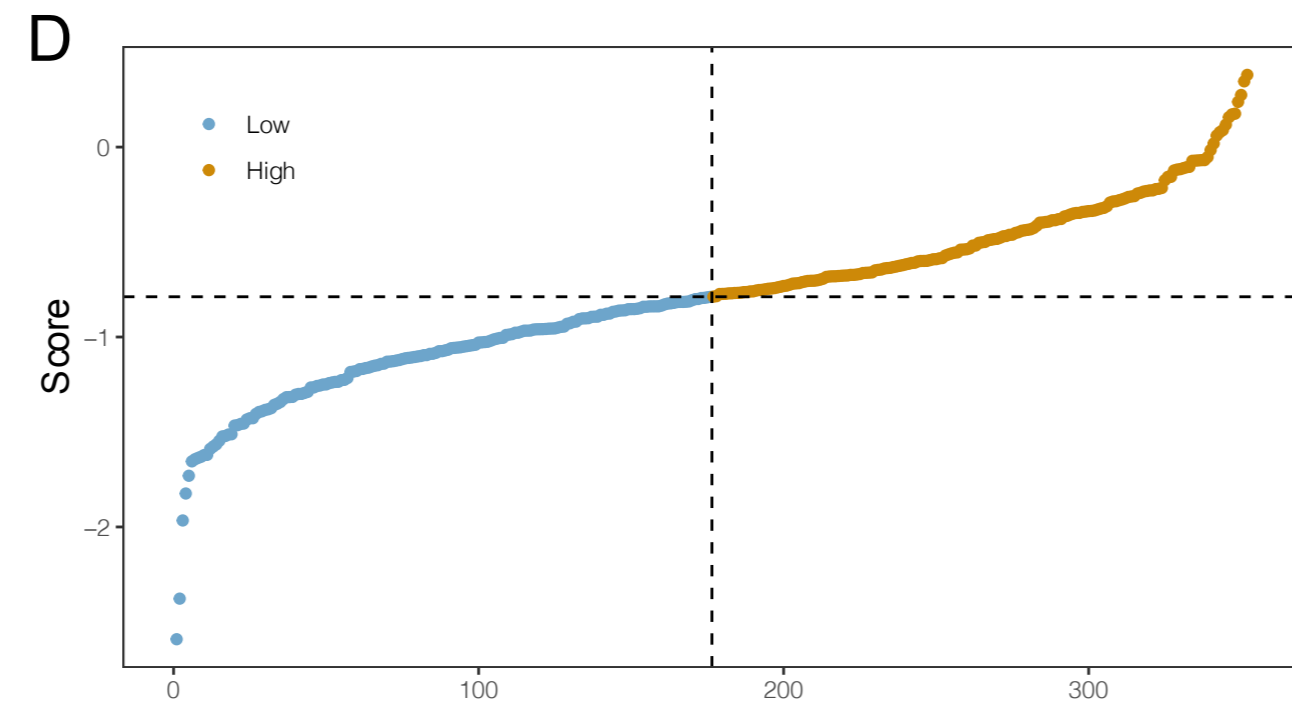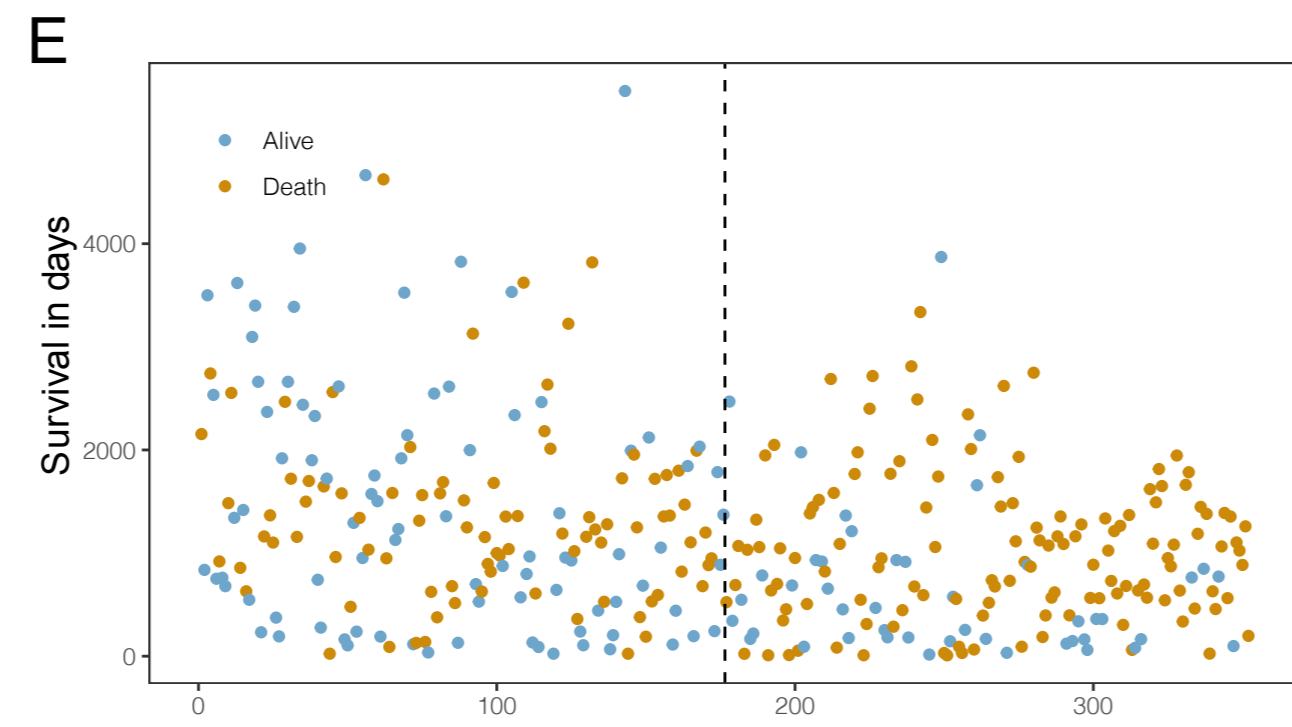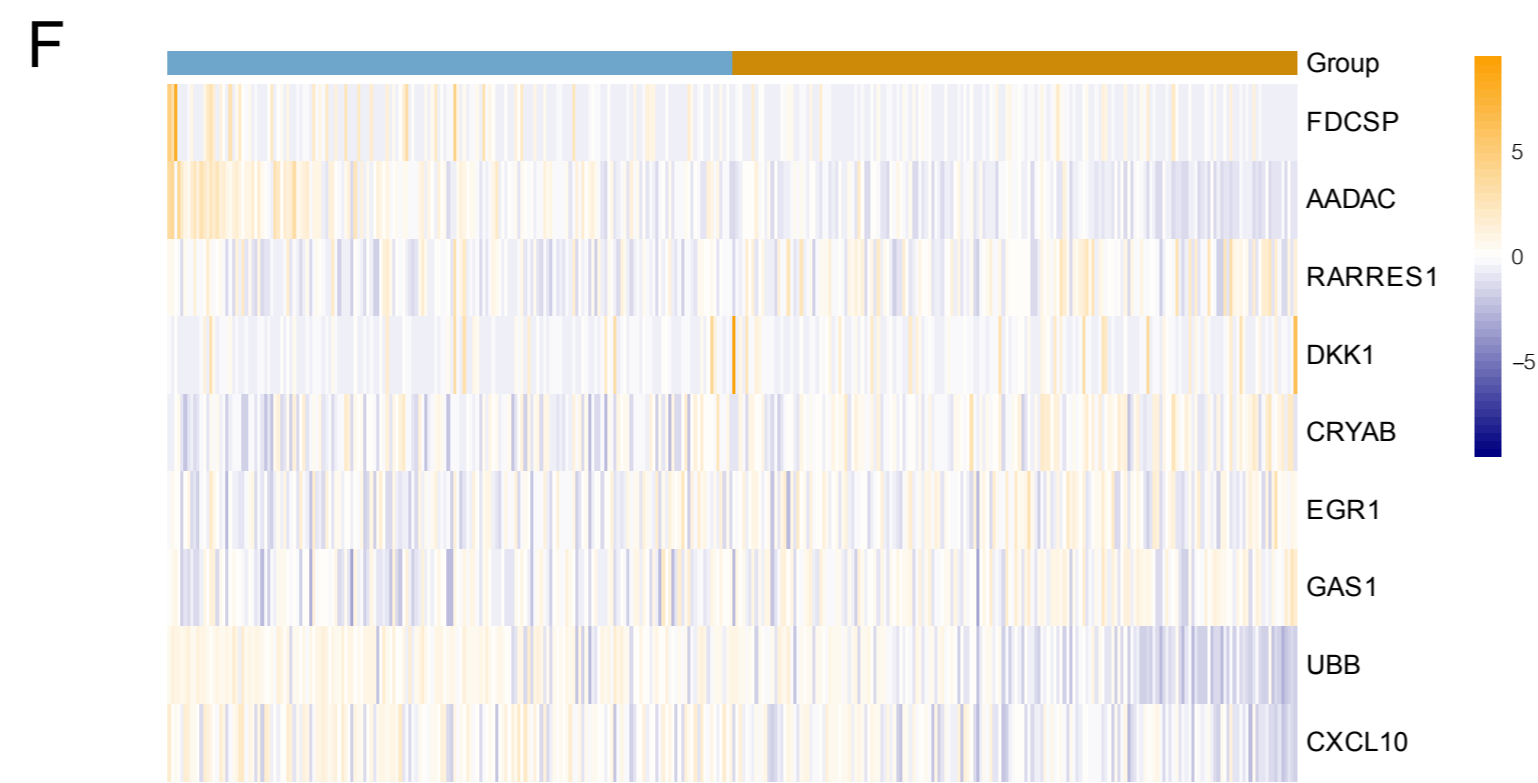

GSE17260 cohort

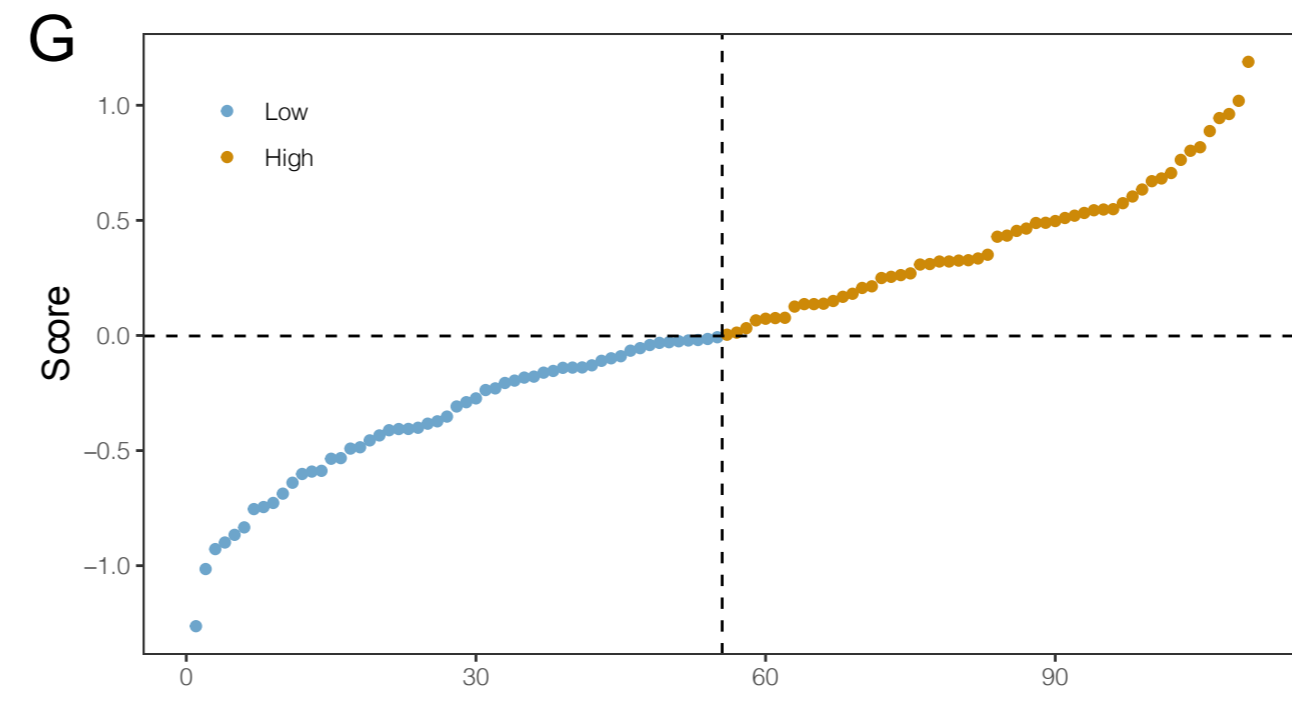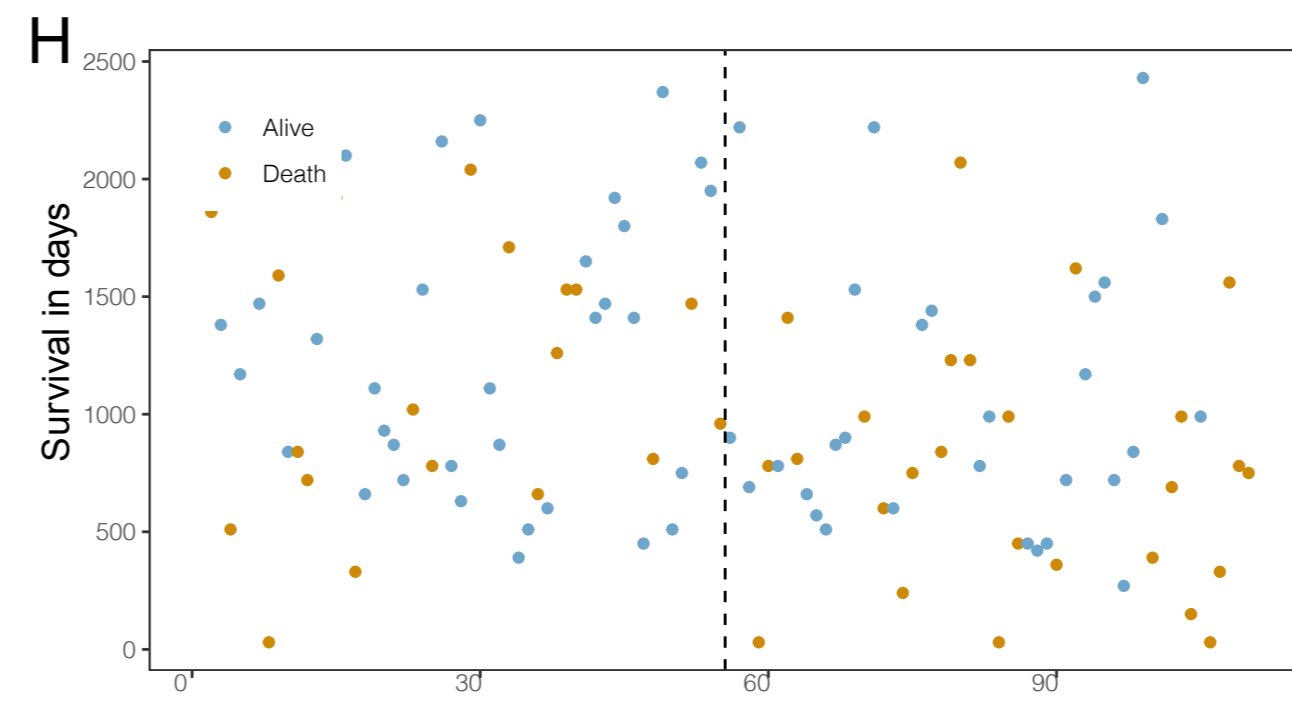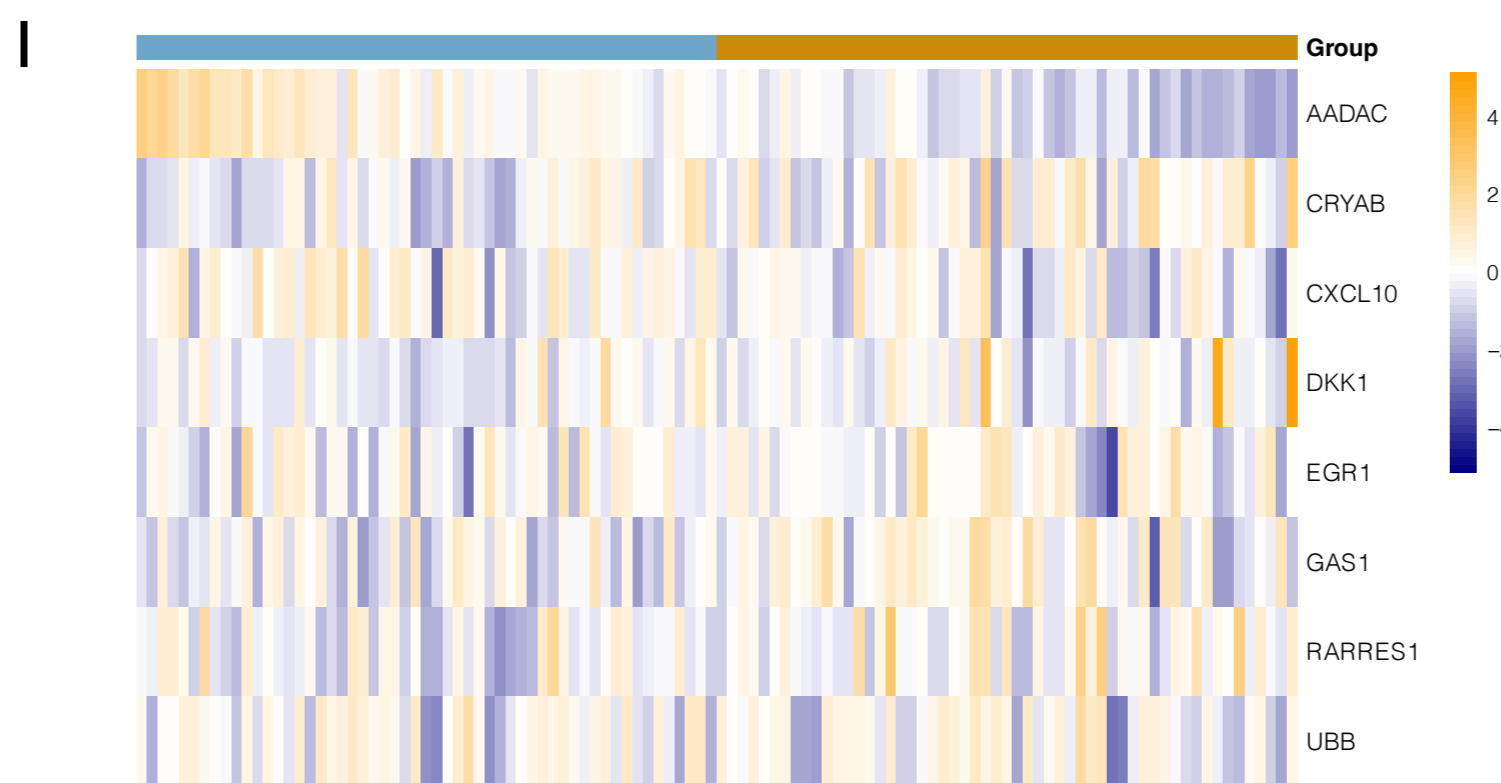

GSE26712 cohort

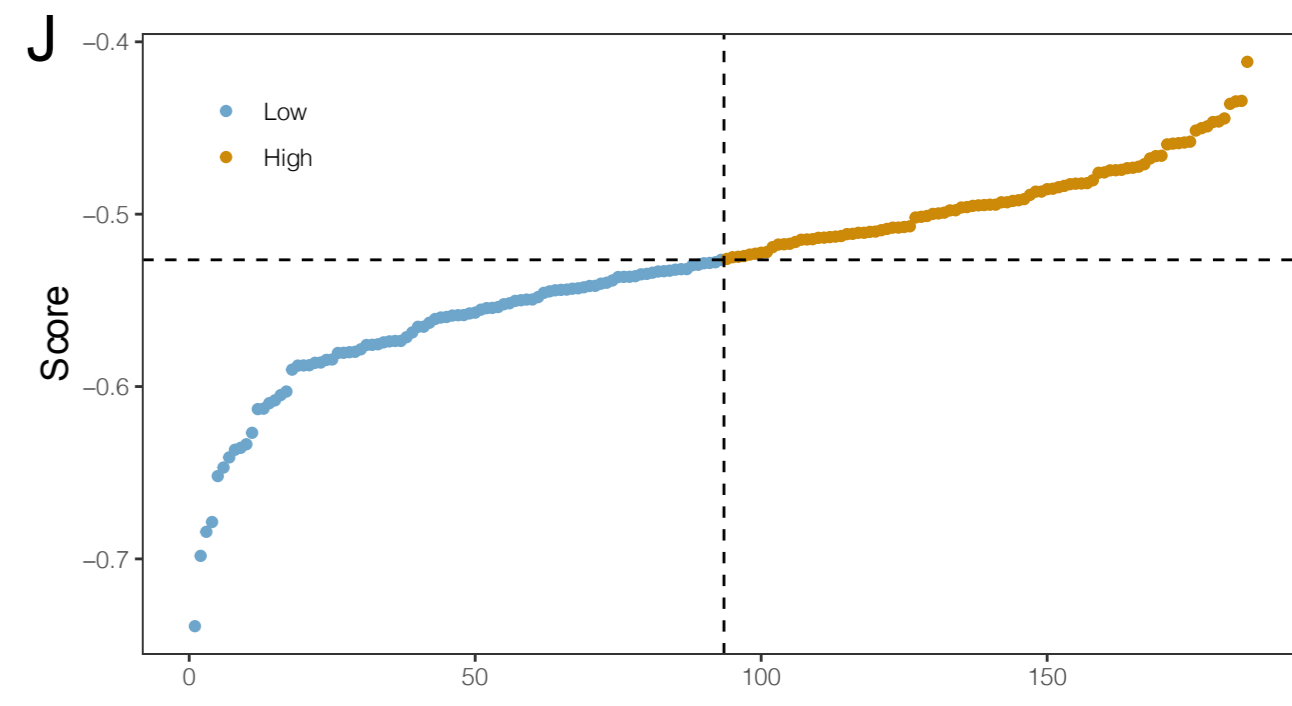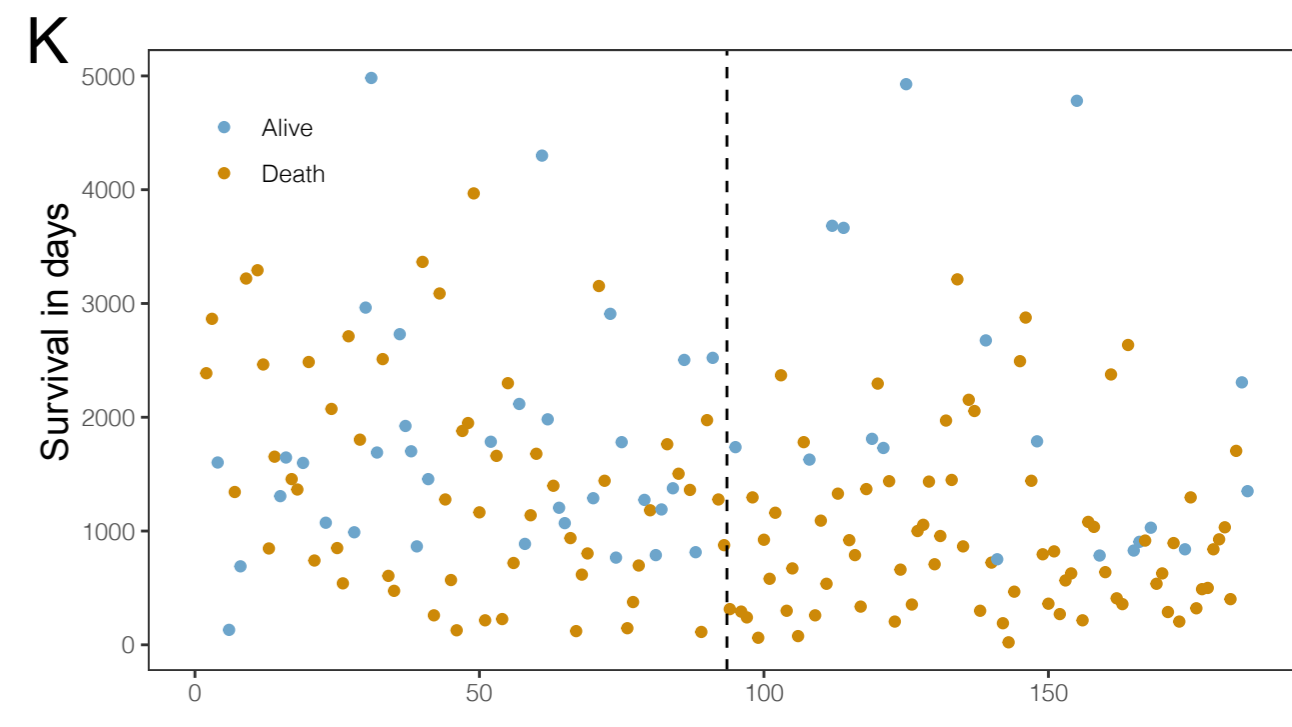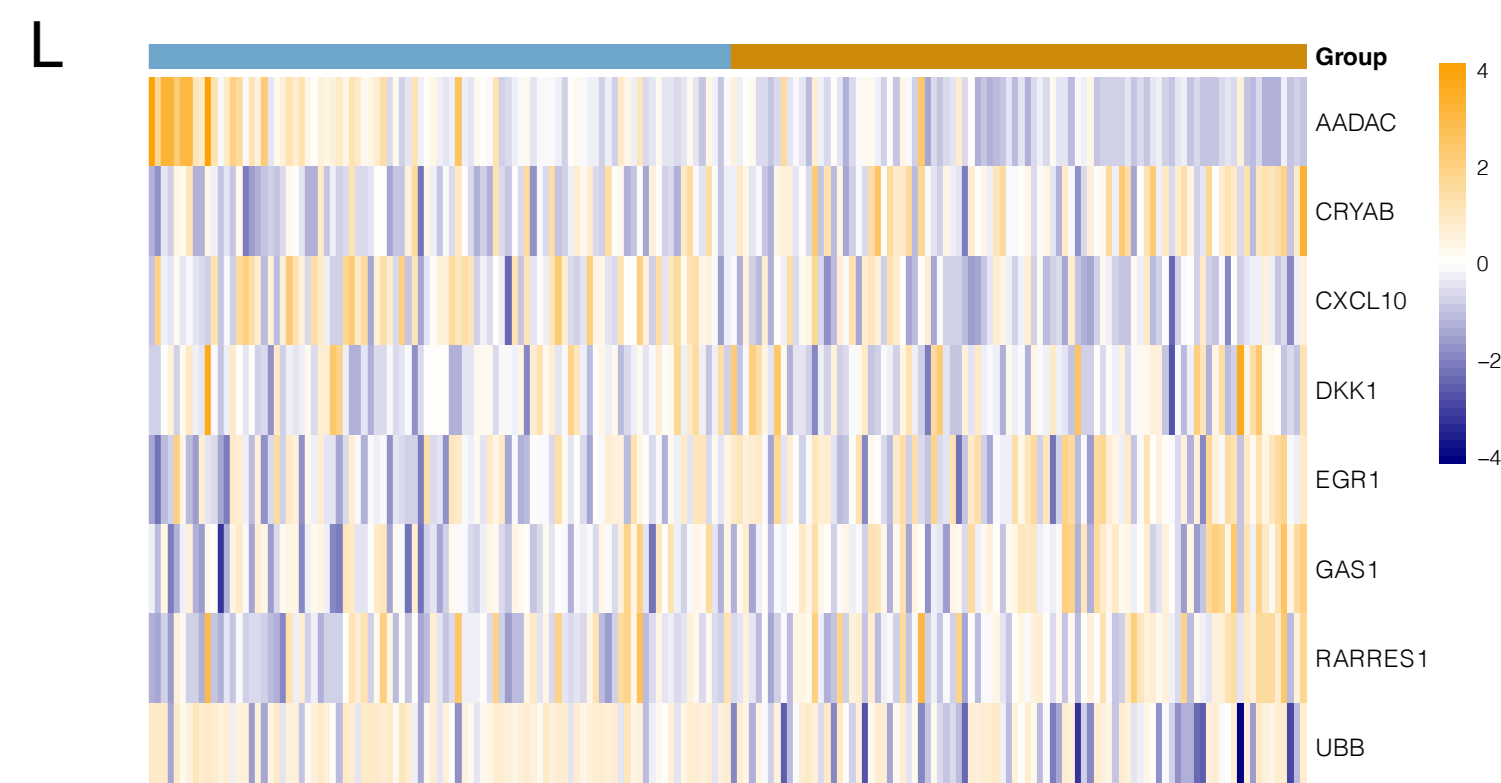

Supplement: Supplementary Materials — Supplement Figure 1: determining the functions of reactive subgroups of OS. (A) The bubble diagram shows the KEGG pathway enrichment analysis of marker genes of the active subgroup. The size of the dot represents the number of marker genes enriched, and the color represents the significant enrichment. (B–D) The bubble diagram shows the GO enrichment analysis of marker genes of active subgroup, biological process (BP), molecular function (MF), and cellular component (CC). (E) Broken line graph shows the gene enrichment score of GSEA. Supplement Figure 2: differential expression and functional enrichment analysis of bulk RNA-sequencing tumor vs. normal samples. (A) The expression heat map shows the intersection of DEGs and marker genes of active cell subgroup, red represents high expression, and blue represents low expression. (B) Broken line graph shows the gene enrichment score of GSEA. (C–F) The bubble diagram shows the GO and KEGG pathway enrichment analysis of DEGs. The size of the point represents the number of marker genes enriched, and the color represents the significant enrichment. Table S1: gene sets related to OS response in 17 ROS pathways. Table S2: 467 OS-related genes. Table S3: subgroup identification and annotation of preprocessed and integrated cells and genes. Table S4: violin plot analysis of the top two markers from each cluster. Table S5: 28 genes obtained from the intersection of marker genes with strong cell subgroup specificity and OS response-related gene sets. Table S6: 56 intersection genes as ROS markers obtained from the intersection of OS response factors and markers specific to cell subsets. Table S7: 2928 DEGs. Table S8: intersection of DEG and marker genes of active cell populations to obtain 151 differentially expressed marker genes. Table S9: the HALLMARK pathway enrichment analysis of DEG using GSEA. Table S10: identification of differentially expressed marker genes of an active cell population using univariate Cox regression anal [file 1261039.f1.zip › Supplement Fig3.pdf]
